# The role of socio-demographic factors and physical functioning in the intra- and interpersonal variability of older adults’ sedentary time: an observational two-country study

**DOI:** 10.1186/s12877-022-03186-1

**Published:** 2022-06-09

**Authors:** Sofie Compernolle, Ester Cerin, Anthony Barnett, Casper J. P. Zhang, Jelle Van Cauwenberg, Delfien Van Dyck

**Affiliations:** 1 Department of Movement and Sport Sciences, Faculty of Medicine and Health Sciences, Ghent University, Ghent, Belgium; 2 Research Foundation Flanders (FWO), Brussels, Belgium; 3 Mary MacKillop Institute for Health Research, Australian Catholic University, Melbourne, VIC Australia; 4 School of Public Health, The University of Hong Kong, Hong Kong, China; 5 Department of Public Health and Primary Care, Faculty of Medicine and Health Sciences, Ghent University, Ghent, Belgium

**Keywords:** Sitting time, elderly, diurnal patterns, epidemiology

## Abstract

**Background:**

Insight into the variability of older adults’ sedentary time is needed to inform future interventions. The aim of this study was to examine the intra- and interpersonal variability in sedentary time, and the moderating role of socio-demographics, physical functioning and geographical location in this variability.

**Methods:**

Cross-sectional data from 818 community-dwelling older adults (mean age: 74.8 years; 61.1%women) of the Active Lifestyle and the Environment in Chinese Seniors and Belgian Environmental Physical Activity Study in Seniors were used. An interview questionnaire was administered to collect socio-demographic information. The Short Physical Performance Battery was performed to evaluate physical functioning, and Actigraph GT3X( +) accelerometers were used to estimate sedentary time. Linear mixed models with random intercepts at the neighborhood, person and day levels examined the variability in sedentary time, and the moderating role of socio-demographics, physical functioning and geographical location within this variability.

**Results:**

Most of the variance in accelerometry-assessed sedentary time was due to intrapersonal variability across periods of the day (72.4%) followed by interpersonal variability within neighborhoods (25.6%). Those who were older, men, lived in Hong Kong, and experienced a lower level of physical functioning were more sedentary than their counterparts. Sedentary time increased throughout the day, with highest levels of sedentary time observed between 6:00 and 9:00 pm. The patterns of sedentary time across times of the day differed by gender, educational attainment, age, physical functioning and/or geographical location. No significant differences were detected between week and weekend day sedentary time.

**Conclusions:**

The oldest old, men, and those with functional limitations are important target groups for sedentary behavior interventions. As sedentary time was the highest in the evening future sedentary behavior intervention should pay particular attention to the evening hours. The variations in diurnal patterns of sedentary time between population subgroups suggest that personalized just-in-time adaptive interventions might be a promising strategy to reduce older adults’ sedentary time.

**Supplementary Information:**

The online version contains supplementary material available at 10.1186/s12877-022-03186-1.

## Background

Minimizing sedentary time (i.e. time while being awake, in a seated, reclining or lying posture, and with a low energy expenditure [1]) contributes to healthy aging [2, 3]. Older adults who accumulate more time sedentary are more likely to suffer from chronic diseases (e.g., type 2 diabetes, metabolic syndrome and cardiovascular disease), experience poor health-related quality of life, have worse physical and cognitive functioning, and die prematurely [4–9]. To mitigate the negative health effects of prolonged sedentary time, older adults should be supported to reduce it. Unfortunately, only a handful of interventions targeting older adults’ sedentary behavior are available [10], and most of the existing interventions yielded only small effects [11]. Consequently, efforts are urgently needed to increase the effectiveness of older adults’ sedentary behavior interventions. One such effort might be to gain a better understanding of the variability in sedentary time.

Sedentary behavior variability can be divided into interpersonal variability, reflecting variability between individuals, and intrapersonal variability, reflecting variability within individuals over time. Most existing research has focused on interpersonal variability of older adults’ sedentary behavior, often by examining socio-demographic differences. A systematic review, summarizing the available evidence regarding determinants of total sedentary time, reported that age and retirement were generally positively associated with older adults’ sedentary time, whereas educational level was usually inversely associated with older adults’ sedentary time [15]. Although this information is useful to tailor interventions to specific target groups, research into intrapersonal variability of older adults’ sedentary behavior is at least as important for the identification of the most appropriate time to deliver an intervention. Notwithstanding that information on patterns of older adults’ sedentary time is easily available when accelerometer data are collected, research on intrapersonal variability of older adults’ sedentary time is scarce. As far as we know, only a few studies have analyzed how older adults’ sedentary time is accumulated throughout the day [16–18]. The studies investigated diurnal variations in accelerometer-derived sedentary time in a US sample of older men (aged 71–91 years), a UK sample of older adults (aged 60 years and over), and a Belgian sample of older adults (aged 65 years and over; data of this study will also be used in this paper). All three studies confirmed that levels of sedentary behavior differ substantially over the course of the day, with highest levels in the evening. Consequently, interventions to reduce sedentary behavior among older adults might be most effective when targeting sedentary behavior in the evening, compared to the morning and the afternoon.

However, to make future sedentary behavior interventions as personalized as possible, it is also important to know if this intrapersonal within-day variability is similar across socio-demographic subgroups. By doing so, information can be obtained to offer the right support at the right moment to the right person. The few available studies examining moderators of older adults’ sedentary behavior patterns showed that the effect of age group on sedentary time differed throughout the day; the difference in sedentary time between age groups seems to be more pronounced in the morning and the afternoon compared to the evening, with the oldest older adults being more sedentary compared to the younger ones [16–18]. Additionally, retirement, educational, and marital status have been mentioned as potential moderators in the association of time of the day with sedentary time. However, previous study results regarding these moderating effects are inconsistent. In the study of Van Cauwenberg et al. [16], marital status was mentioned as a socio-demographic moderator, with widowed and non-married older adults being more sedentary compared to married older adults in the morning and in the afternoon, but showing no differences in the evening. In the study of Yerrakalva et al. [17], retirement and educational status were detected as socio-demographic moderators. No difference was found between retired and employed older adults in the morning, whereas retired older adults were more sedentary in the afternoon and evening. Furthermore, higher educated older adults were more sedentary than lower educated older adults in the morning, while in the afternoon and in the evening, it was the other way around. An important explanation for the inconsistencies regarding socio-demographic moderators might be the country-specificity of the findings. All previous findings were from single-country studies, conducted in Europe and North America, and thus limited by the study samples and contexts. The conclusions drawn from those single-country studies cannot be claimed to be universal nor generalizable across cultures and geographical regions. Hence, multi-country studies applying the same methodology are warranted to determine the generalizability of the findings.

Furthermore, individual factors other than socio-demographic attributes may also have an influence on the intrapersonal within-day variability of older adults’ sedentary behavior. Therefore, these other factors also deserve attention when examining moderators in the association between time of the day and accelerometer-assessed sedentary behavior. Based on physical activity evidence [18], it can be expected that health-related variables contribute to diurnal patterns in older adults’ sedentary behavior. An important health-related variable in older adults is physical functioning. Physical functioning is conceptualized as those physical abilities that allow functional independence and those related to movement [19–21]. Physical functioning can be evaluated across the three constructs of the International Classification of Functioning framework: body function and structure, activities, and participation [22]. Recent research has identified physical functioning as an important determinant of older adults’ sedentary behavior [23, 24]. In the study of Walker et al., a 0.3–0.4 points lower Short Physical Performance Battery score was related to 1.9 h more daily sedentary time [24]. However, at this point, it remains unclear if this relationship differs according to time of the day.

So, to tackle some of the shortcomings in the currently available studies and to advance the future development of sedentary behavior interventions for older adults, the aims of the present study were: 1) to estimate inter- and intrapersonal variability in older adults’ accelerometer-assessed sedentary time; 2) to examine the moderating role of socio-demographic factors and physical functioning within the intrapersonal variability; and 3) to examine whether these moderating effects vary by study site, that is, geographical location. In this study, we examined two samples from very different cultures, built environments and climatic zones – namely, Ghent (Flemish older adults living in a moderately dense medieval city with a marine West coast climate) and Hong Kong (Chinese older adults living in an ultra-dense modern metropolis with a humid subtropical climate) to test the potential generalizability of findings to ageing populations across the world. Similar findings across these two diverse study sites would support their potential generalizability to several other ageing populations across the world.

## Methods

### Study design

Data were obtained from two comparable cross-sectional studies in community-dwelling older adults (≥ 65 years). Data collection for the first study, i.e. the Active Lifestyle and the Environment in Chinese Seniors (ALECS; first wave), took place between May 2012 and December 2014 in Hong Kong [25]. Data collection for the second study, i.e. the Belgian Environmental Physical Activity Study in Seniors (BEPAS Seniors), occurred between October 2010 and September 2012 in Ghent [26]. The studies were conducted in accordance with the Helsinki Declaration and the underlying data protection regulation [27]. The studies received ethical approval from the Department of Health (Hong Kong Special Administrative Region, HKSAR) and the University of Hong Kong Research Ethics Committee for Non-Clinical Faculties (EA270211), and from Ghent University Hospital (B670201423000), respectively.

### Participants and procedure

Details on the two-stage sampling process and the recruitment strategy have been published elsewhere [25, 26, 28]. Briefly, 124 and 20 neighborhoods were selected respectively in Hong Kong (China) and Ghent (Belgium) based on walkability (i.e., a Geographic Information System (GIS)-based index of dwelling density, intersection density and land use mix), and neighborhood socio-economic status (estimated by the median annual household income) using stratified random sampling. Hong Kong participants were recruited in person in Elderly Health Centers of the Health Department (72%) and from elderly community centers close to the selected neighborhoods (28%). Inclusion criteria for Hong Kong participants were being able to understand and speak Cantonese, living in one of the selected neighborhoods for at least six months, being able to walk unassisted for at least 10 m and being cognitively intact (Mini-Mental State Examination Score > 22). A total of 1602 potential Hong Kong participants were approached, of which 909 (71% of those eligible) were enrolled in the study. Approximately forty-five percent of those enrolled (i.e., 416) were randomly selected to wear an accelerometer. Of these, 14 participants were excluded due to invalid accelerometer data, resulting in an analytical sample of 402 Hong Kong participants. In Ghent, stratified random sampling based on gender and age (< 75 years vs. ≥ 75 years) was applied by the public service of Ghent to select 1750 community-dwelling older adults. Selected older adults were sent a letter with study information, and the notification of a home visit by a trained interviewer during the next two weeks. After a maximum of three visit attempts, the trained interviewers found 1260 older Belgian adults at home, of which 633 agreed to participate (response rate: 50.2%). Of these, 125 (9.9%) did not meet the predefined inclusion criteria (i.e., being able to understand and speak Dutch, living independently and able to walk 200 m without severe physical restrictions) and were excluded. Of the 508 remaining Ghent participants, 416 provided valid accelerometer data and were included in the analysis. Thus, the final sample two-site sample consisted of 818 participants.

## Measures

### Socio-demographic factors and physical functioning

Age, gender, educational level (primary school versus higher), and marital status (married or cohabiting versus widowed or other) were assessed with an interview-administered questionnaire in both study sites. Physical functioning was evaluated using the Short Physical Performance Battery (SPPB) [29]. The SPPB is a validated measure of lower extremity physical functioning and consists of three tests (i.e., balance test, walking test, and sit-to-stand test). The balance test included three positions: side-by-side, semi-tandem and tandem. Participants were timed until they moved, or until ten seconds had elapsed. For, the walking test, participants were asked to walk three meters at their regular pace. The walking test was repeated two times, and the fastest attempt was reported. During the sit-to-stand test, participants were instructed to stand up from the chair, and then sit down as quickly as possible for five repetitions. Participants were asked to keep their arms folded across their chest. The time taken to complete the chair stands was recorded with a stopwatch. For each test, a categorical score ranging from 0 to 4 was created. A summary score for physical performance was obtained by summing the three categorical scores, resulting in a score ranging from 0 to 12 with higher scores denoting better physical performance [29]. All test procedures, including instructions, positioning and scoring, were conducted using a standardized protocol, as described by Guralnik and colleagues [29].

### Sedentary time

Sedentary time was assessed using the validated GT3X and GT3X + Actigraph accelerometers (Fort Walton Beach, FL, USA) [30, 31]. Participants were asked to wear the accelerometer above the right hip for seven consecutive days during waking hours, except during bathing and other water-based activities. Data were collected using 60-s epochs, and the low frequency extension filter was applied to all Actigraph data. Only data capturing the vertical axis were included for analysis. A valid day was defined as a day with at least 10 h of recorded activity, and periods with ≥ 90 min of consecutive zeros were categorized as non-wear time [32]. Only participants with at least one valid week day and one valid weekend day were included in the analysis. Minutes with less than 25 counts were categorized as sedentary time, according to the cut points developed by Aguilar-Farias and colleagues for older adults [33].

### Statistical analyses

Descriptive statistics were computed for the whole analytic sample and by study site. To estimate the between- and within-person variance of, and associations of socio-demographic factors and physical functioning with, accelerometer-assessed sedentary time, four-level linear mixed models (LMMs) were used. Random intercepts were included at the neighborhood, person, and day levels to account for the nested structure of the data (i.e. periods of the day [6am-9am; 9am-12 pm; 12 pm-3 pm; 3 pm-6 pm; 6 pm-9 pm] were nested within days, days were nested in individuals and individuals were nested in neighborhoods) and the respective variances were calculated. In addition, the error variance at the lowest level of variability (within-day across periods of the day), corresponding to the error variance given by single-level regression models, was estimated. The neighborhood-level intercept variance component was included because both study sites used a two-stage sampling strategy whereby participants were recruited from preselected administrative units (neighborhoods).

Between- and within-person variances in older adults’ accelerometer-assessed sedentary time throughout the day were estimated using a four-level unconditional random intercept LMM with accelerometer wear time as the only explanatory variable (M1). The percentage of variance attributable to a specific level of variability (neighborhood, person, day and within day) was calculated by dividing the variance components of the grouping variables by the total variance (i.e. sum of the variance components), and the likelihood ratio (LR) test was used to determine the statistical significance of three variance components as specified by Snijders and Bosker [34]. Socio-demographic factors (age, gender, educational attainment and marital status), physical functioning and study site were added as explanatory variables to the four-level LMM (M2) examining their associations with sedentary time and contribution to explaining between-person variance in sedentary time (obtained by dividing the difference between the neighborhood-level variances obtained in M1 and M2 by the neighborhood-level variance obtained in M1, then converting to a percent). Day of the week (weekday = 0; weekend day = 1) and time of the day (6:00 am – 8:59 am = 0; 9:00 am – 11:59 am = 1; 12:00 pm – 2:59 pm = 2; 3:00 pm – 5:59 pm = 3; 6:00 pm – 9:00 pm = 4) were added as explanatory variables in the third four-level LMM (M3) examining differences in sedentary time across days of the week and time of the day. Finally, a set of additional LMMs examined the moderating effects of socio-demographic factors, physical functioning and study site on the associations of day of the week and time of the day with sedentary time by adding two- and three-way interaction terms to M3. Significant interaction effects (*p* < 0.05) were probed and marginal means (and 95% CIs) of accelerometry-assessed sedentary time at meaningful values of explanatory variables and moderators were computed and plotted. All analyses were conducted in Stata 15.

## Results

### Sample characteristics, accelerometer wear time, and accelerometer-assessed sedentary time

Table 1 presents the descriptive statistics of the study sample. A total of 818 older adults were included with a mean age of 74.81 (± 6.19) years. The sample had a higher proportion of women (61.12%) and married or cohabiting persons (65.28%). The proportion of participants with an educational attainment above primary level was considerably higher in Belgian (74.76%) than Hong Kong older adults (46.77%). Physical functioning was generally high with a mean score of 10.2 (± 1.94) out of 12.

**Table 1:** Sample characteristics and accelerometer wear time

Characteristics	ALECS & BEPAS (*n* = 818)		ALECS (*n* = 402)	BEPAS (*n* = 416)
**Participant socio-demographics and physical function**				
Age (years; mean ± SD)	74.81 ± 6.19		75.55 ± 6.15	74.09 ± 6.15
Gender (% women)	61.12		68.91	53.61
Education (% above primary school)	61.00		46.77	74.76
Marital status (% married or cohabiting)	65.28		62.94	67.55
Physical functioning (SPPB score: range 1–12, mean ± SD)	10.02 ± 1.94		9.94 ± 1.95	10.10 ± 1.93
**Accelerometer-assessed wear time (mean ± SD)**				
Valid weekdays		5.16 ± 1.40	5.30 ± 1.55	5.01 ± 1.24
Valid weekend days		2.06 ± 0.68	2.06 ± 0.74	2.07 ± 0.62
Average wear time (h/day)		13.92 ± 1.46	13.53 ± 1.54	14.29 ± 1.26

*ALECS* Active Lifestyle and the Environment in Chinese Seniors study, *BEPAS* Belgian Environmental Physical Activity Study on Seniors study, *SD* standard deviation, *SPPB* Short Physical Performance Battery

Table 2 shows the average accelerometer-assessed sedentary time and the percentage of wear time spent sedentary by day of the week and time of the day. The percentage of wear time spent sedentary in the total sample was the lowest early in the morning (6:00 am – 8:59 am), and highest in the evening (6:00 pm – 9:00 pm), both on weekdays (42.7% and 57.7%, respectively) and on weekend days (42.1% and 58.1%, respectively).

**Table 2:** Average accelerometer-assessed sedentary time and percentage of wear time spent sedentary by day of the week and time of the day

Day of the week Time of the day	ALECS & BEPAS		ALECS		BEPAS	
	ST (min/h)	% of WT	ST (min/h)	% of WT	ST (min/h)	% of WT
*Weekday*						
6:00 am – 8:59 am	17.7 (13.0)	42.7	17.7 (12.3)	42.2	17.8 (13.9)	43.5
9:00 am – 11:59 am	24.4 (11.7)	42.2	26.9 (11.7)	46.5	21.7 (11.0)	37.8
12:00 pm – 2:59 pm	30.00 (11.4)	50.8	30.9 (11.5)	52.8	29.0 (11.2)	48.6
3:00 pm – 5:59 pm	28.8 (11.9)	49.2	29.4 (11.9)	51.2	28.2 (11.8)	47.2
6:00 pm – 9:00 pm	30.00 (13.75)	57.7	26.2 (14.8)	56.6	33.9 (11.3)	58.7
*Weekend day*						
6:00 am – 8:59 am	17.0 (12.4)	42.1	17.1 (11.9)	42.3	16.9 (13.1)	41.8
9:00 am – 11:59 am	24.6 (11.3)	43.0	26.2 (11.3)	45.9	22.8 (11.0)	39.8
12:00 pm – 2:59 pm	30.1 (11.7)	50.8	31.3 (11.9)	53.2	28.9 (11.2)	48.4
3:00 pm – 5:59 pm	29.4 (12.1)	50.3	30.2 (12.3)	52.7	28.6 (11.8)	48.0
6:00 pm – 9:00 pm	30.6 (13.1)	58.1	27.2 (14.1)	57.1	34.1 (10.9)	58.7

*ALECS* Active Lifestyle and the Environment in Chinese Seniors study, *BEPAS* Belgian Environmental Physical Activity Study on Seniors study, *ST* sedentary time, *WT* wear time

### Inter- and intrapersonal variability of older adults’ sedentary time and main effects of socio-demographic factors, physical functioning and study site

Tables 3, 4 and S1 summarize the findings from the LMMs. Most of the variance in accelerometry-assessed sedentary time was due to within-person differences across periods of the day (72.4%) followed by between-person differences within neighborhoods (25.6%) (Table 3; M1). Only 1.3% and 0.7% of the variance were due to between-neighborhood and within-person between-day differences, respectively. Age was positively, and physical functioning negatively, related to sedentary time (Table 3; M2). On average, women were 2.3 min (95% CI = -3.15 to -1.45) less sedentary per hour compared to men, and participants from Ghent (Belgium) were 1.6 min (95% CI = -2.54 to -0.67) less sedentary per hour than participants from Hong Kong. Socio-demographic characteristics, physical functioning and study site contributed to the explanation of nearly 55% of neighborhood-level variance and 16% of between-person level variance (Table 3; see random parts of M1 and M2). On average, participants tended to accumulate slightly more sedentary time on weekend days compared to weekdays (Table 3; M3). They were more sedentary in the afternoon than morning periods, most sedentary between 6:00 and 9:00 pm and least sedentary between 9:00 am and 11:59 am.

**Table 3:** Main-effect regression models of sedentary time in older adults

	**Models**					
**Correlates**	**M1**		**M2**		**M3**	
***Fixed part***	*b*	95% CI	*b*	95% CI	*b*	95% CI
Wear time (min/h)	0.53^***^	0.52, 0.54	0.53^***^	0.52, 0.54	0.52^***^	0.51, 0.53
Age (years)	-	-	0.28^***^	0.21, 0.34	0.28^***^	0.21, 0.34
Gender (ref: men)						
Women	-	-	-2.32^***^	-3.17, -1.47	-2.30^***^	-3.15, -1.45
Education (ref: up to primary school)						
Above primary school	-	-	0.75	-0.10, 1.61	0.73	-0.12, 1.59
Marital status (ref: no partner)						
Married or cohabiting	-	-	0.27	-0.59, 1.12	0.25	-0.60, 1.11
Physical function (SPPB score)	-	-	-0.43***	-0.65, -0.21	-0.43***	-0.65, -0.21
Study site (ref: ALECS [Hong Kong, China])						
BEPAS (Ghent, Belgium)	-	-	-1.57***	-2.50, -0.63	-1.61***	-2.54, -0.67
Day of week (ref: weekday)						
Weekend day	-	-	-	-	0.24	-0.02, 0.51
Period of the day (ref: 6:00 am – 8:59 am)						
9:00 am – 11:59 am	-	-	-	-	-1.59***	-1.97, -1.21
12:00 pm – 2:59 pm	-	-	-	-	3.15***	2.76, 3.53
3:00 pm – 5:59 pm	-	-	-	-	2.40***	2.02, 2.79
6:00 pm – 9:00 pm	-	-	-	-	6.90***	6.54, 7.27
***Random part***	Var. comp.(95% CI)	% total var	Var. comp.(95% CI)	% total var	Var. comp. (95% CI)	% total var
Neighborhood-level variance	1.66 (0.63, 4.36)	1.3%	0.75 (0.17, 3.24)	0.6%	0.77 (0.18, 3.25)	0.7%
Person-level variance	32.41 (29.04, 36.18)	25.6%	27.38 (24.50, 30.61)	22.7%	27.24 (24.37, 30.45)	24.3%
Day-level variance	0.91 (0.34, 1.43)	0.7%	0.91 (0.34, 2.43)	0.8%	3.17 (2.40, 4.18)	2.8%
Within-day-level variance	91.72 (90.00, 93.47)	72.4%	91.71 (90.00, 93.46)	76.0%	81.05 (79.53, 82.60)	72.2%
LR test	χ^2^(3) = 6452.28; *p* < .001		χ^2^(6) = 136.21; *p* < .001		χ^2^(5) = 2681.19; *p* < .001	
Type of LR test	Random effects		Fixed effects: M2 vs. M1		Fixed effects: M3 vs. M2	

*LR* likelihood ratio, *var* variance, *Var. comp* variance component, *ref* reference category, *ALECS* Active Lifestyle and the Environment in Chinese Seniors study, *BEPAS* Belgian Environmental Physical Activity Study on Seniors study, *SPPB* Short Physical Performance Battery, b, regression coefficient; *CI* confidence intervals; *** *p* < .001

**Table 4:** Interaction effects of time of day on accelerometer-assessed sedentary time

Interaction term	χ^2^(4)	*p*-value	Interaction term	χ^2^(4)	*p*-value
*Two-way interactions*			*Three-way interactions*		
Time of day by Age	16.70	.002	Time of day by Age by Study site	25.98	< .001
Time of day by Gender	17.99	.001	Time of day by Gender by Study site	36.61	< .001
Time of day by Education	44.64	< .001	Time of day by Education by Study site	31.64	< .001
Time of day by Marital status	4.74	.315	Time of day by Marital status by Study site	7.56	.109
Time of day by Physical function	13.28	.010	Time of day by Physical function by Study site	5.88	.208
Time of day by Study site	455.14	< .001			

All models with random intercepts at the neighborhood, person and day level, and adjusted for accelerometer wear time, age, gender, educational attainment, marital status, physical function, study site and day of the week; χ^2^, chi square; results from χ^2^(4) test because Time of day is a nominal variable with five categories

### Moderating effects of socio-demographic factors, physical functioning and study site in the intrapersonal variability of older adults’ sedentary time

The difference in sedentary time between weekdays and weekend days was similar across study sites, socio-demographic groups and participants with different levels of physical functioning (Table S1). However, the patterns of sedentary time across times of the day differed by gender, educational attainment, age, physical functioning levels and/or study site (see two -and three way interactions in Table 4).

**Fig. 1:**
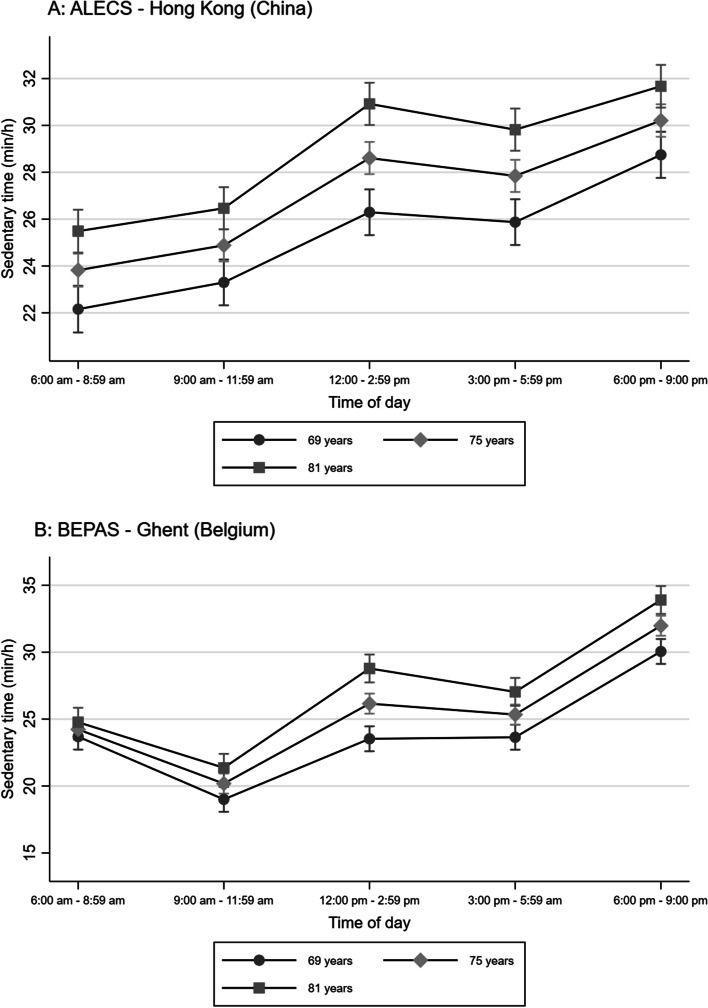
Marginal means of accelerometry-based sedentary time by time of day, age and study site

**Fig. 2:**
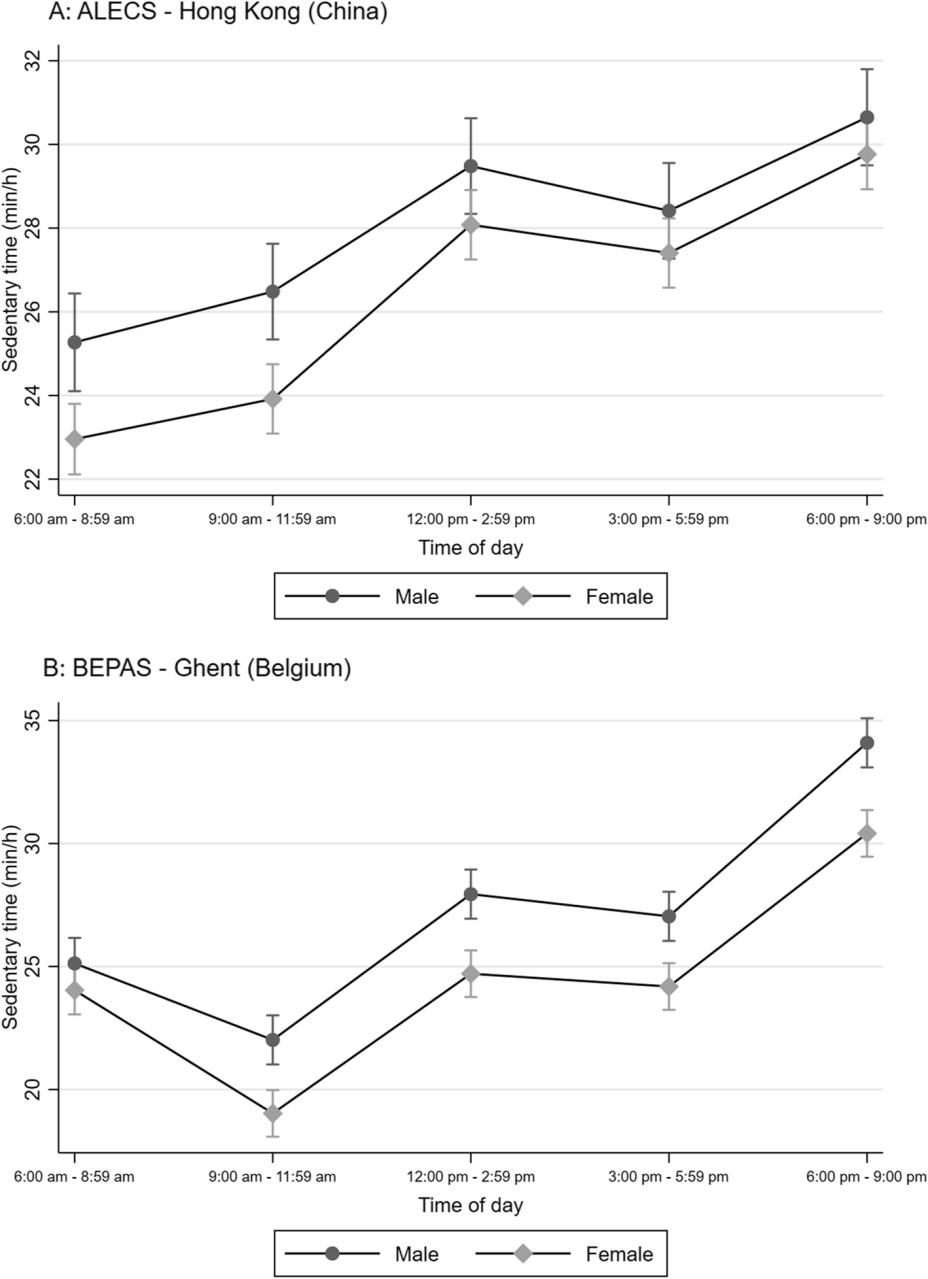
Marginal means of accelerometry-based sedentary time by time of day, gender and study site

**Fig. 3:**
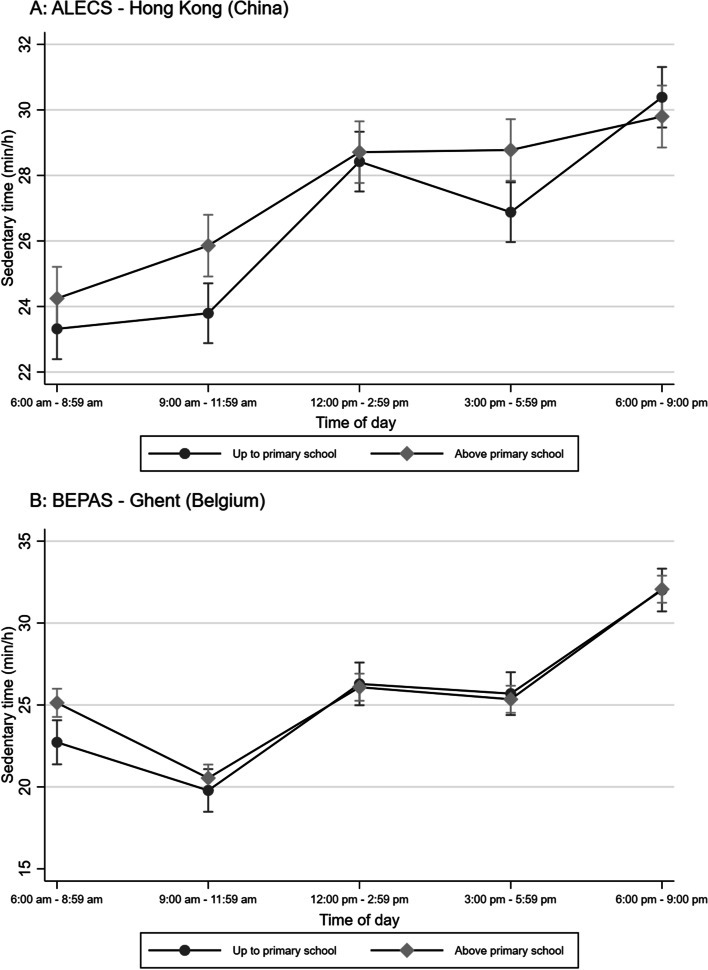
Marginal means of accelerometry-based sedentary time by time of day, education and study site

In Hong Kong, older adults tended to be the least sedentary early in the morning (6:00 am – 8:59 am), while in Ghent they were the least sedentary between 9:00 am and 11:59 am (Figs. 1–3). The difference in sedentary time between late afternoon (3:00 pm – 5:59 pm) and evening (6:00 pm – 9:00 pm) was considerably more pronounced in Ghent than in Hong Kong (Figs. 1–3), with older adults from Ghent increasing their sedentary time more than their Hong Kong counterparts in the evening.

In Hong Kong, the difference in sedentary time between age groups was similar across the various periods of the day, with older participants being more sedentary than younger participants (Fig. 1 – panel A). In Ghent, no significant difference in sedentary time was observed between age groups early in the morning (6:00 am – 8:59 am) (Fig. 1 – panel B). The difference between age groups was significant at other times of the day and the largest between noon and 2:59 pm, with older participants being more sedentary than their younger counterparts.

Hong Kong participants showed larger between-gender differences in sedentary time in the morning than afternoon and evening, with women being less sedentary than men and between-gender differences being the smallest and non-significant in the evening (Fig. 2 – panel A). Conversely, while Ghent men and women did not significantly differ in the amount of sedentary time they accrued early in the morning, they differed at other periods of the day whereby women were less sedentary than men (Fig. 2 – panel B). The largest between-gender differences were observed in the evening.

In Hong Kong, older adults with lower education attainment were less sedentary than those with higher education in the morning and late afternoon only (3:00 pm – 5:59 pm) (Fig. 3 – panel A), while in Ghent a significant difference in sedentary time between educational attainment groups was observed only between 6:00 am and 8:59 am, with the less educated being less sedentary (Fig. 3 – panel B).

**Fig. 4:**
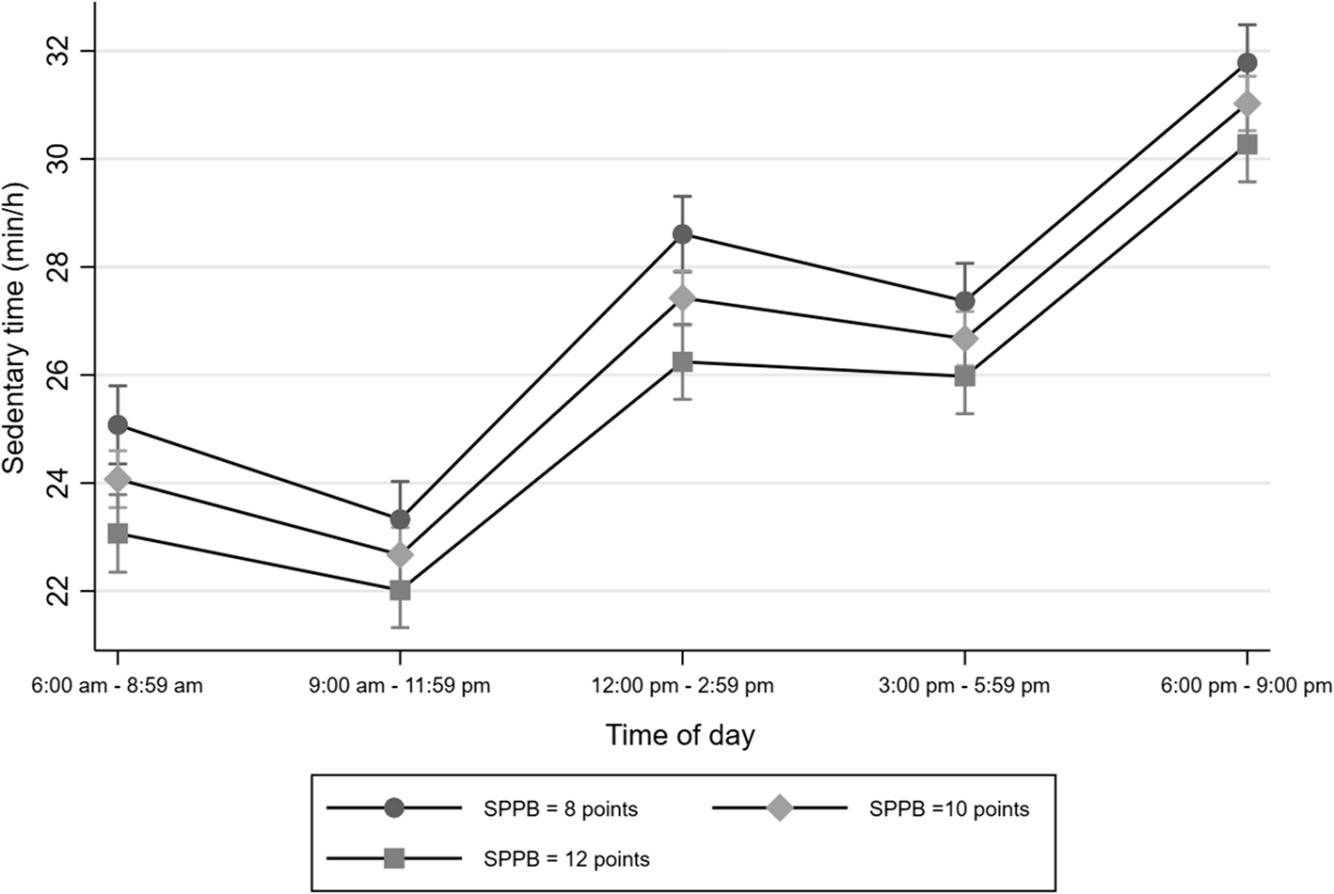
Marginal means of accelerometry-based sedentary time by time of day and physical function score

Finally, the difference in sedentary time between participants with varying levels of physical functioning was the greatest around noon to early afternoon (2:59 pm) followed by the early morning hours (Fig. 4). These patterns did not differ across study site.

## Discussion

This study investigated the variability of older adults’ sedentary time, and the role of socio-demographic factors, physical functioning, and study site in this variability. Almost three quarters of the variability in older adults’ sedentary time was attributed to within-person differences across the day. This finding is important, and points to the potential of JITAI’s providing support at the moment in which the person needs it most [12, 35, 36]. Similar to previous studies in older adults, sedentary time increased throughout the day, reaching its peak in the evening [17, 18]. Palmer et al. suggested that the evening peak may emerge as a way of managing their declining physical function [37]. Older adults indicated during semi-structured interviews that they sit more in the afternoon and/or evening to recover from, or relax after, non-sitting activities earlier in the day [37]. Although this evening peak was present across all investigated subgroups, the magnitude of the peak differed depending on socio-demographic factors and study site. For example, the results showed that the peak was less pronounced in Hong Kong older adults, compared to Belgian older adults, and among higher educated Hong Kong older adults compared to lower educated Hong Kong older adults. A potential explanation for the difference in evening peak might be the variation in television time, which is considered a typical evening sedentary activity. Previous research, conducted among older Japanese adults, already indicated that residents from dense urban areas are less likely to watch television, and more likely to go out in the evening [38].

Next to the differences in evening peak, variations also occurred throughout the rest of the day. For example, the current results show that Hong Kong older adults were least sedentary early in the morning (i.e., between 6 and 9 am), whereas Ghent older adults were least sedentary between 9 am and noon. This is not surprising, as recent research indicated that morning exercise, such as Tai Chi, and walking in the hills, is the second most popular activity of Hong Kong elderly [39, 40]. A detailed examination of morning sedentary time of Hong Kong older adults showed significant differences between Hong Kong men and women. Especially, Hong Kong older women spend little sedentary time in the morning. This was in line with previous results from a US study conducted by Bellettiere and colleagues, who also reported that women were significantly less sedentary in the morning compared to men [41]. It is, however, interesting that this difference was not observed in Ghent older adults. Furthermore, large differences were also noticeable in older adults’ sedentary time spent between 12 am and 3 pm depending on levels of physical functioning, and age group. This is in line with previous research [17, 18], and can possibly be explained by the often-occurring habit of older adults to take a nap after lunch. Research showed that the prevalence of post-lunch napping increased linearly with age and was inversely related to levels of physical functioning [42, 43]. Although the health effects of napping are not well established, a recent review of literature concluded that naps of short duration (e.g., 30 min) are related to better health, while naps of longer duration (e.g., > 90 min) have been linked to adverse cardiovascular and diabetes outcomes, declining cognitive function, and increased mortality [44].

About one quarter of the variability in older adults’ sedentary time was due to between-person differences within neighborhoods. Although this percentage is far less than the 72.4% reported for within-person differences across the day, it is clear that not all older adults are similarly sedentary. In line with previous research, our results indicate that those who were older, men and scored less well on physical functioning were more sedentary compared to their counterparts, suggesting that these subgroups should receive particular attention in future sedentary behavior interventions [15]. In contrast to previous studies, no significant difference in sedentary time was found between lower and higher educated older adults [15]. This is likely due to the fact that far more Hong Kong older adults were classified in the low education group compared to the Belgian older adults, and they tended to accumulate less sedentary time than their higher education counterparts at least in certain periods of the day. Previous research indicated that education was a consistent correlate of sedentary behavior, with an inverse association in European populations but not in studies from Asia, suggesting possible cultural and environmental differences contributing to the discrepant findings [15, 45]. Finally, study setting also contributed to the between-person differences in sedentary behavior; with older adults from Ghent being less sedentary than older adults from Hong Kong. This finding cannot be compared with previous results, as little research has been conducted on the prevalence of sedentary behavior in Asian populations [46], and self-report measures of sedentary behavior [45, 47–49] or different accelerometer cut-points for sedentary time [28, 50, 51] were used. A potential explanation for the difference in sedentary behavior between the study sites might be the difference in housing between Ghent and Hong Kong. A considerable number of Ghent older adults live in larger detached houses with many opportunities to engage in gardening and housework, whereas older Hong Kong residents typically live in small apartments [52] providing fewer opportunities for light-intensity household activities.

### Implications

The marked within-day variations in sedentary time are important for the development of future interventions aimed at the reduction of older adults’ sedentary time. As the majority of older adults’ sedentary time is spent in the evening, interventions focusing on that time period seems highly needed. An important caveat, however, is that, within this study, we did not investigate if older adults are also receptive towards sedentary behavior interventions in the evening. As explained above, high levels of evening sedentary time are often due to the fact that older adults need to recuperate from fatigue induced by morning or afternoon activities [37]. Moreover, older adults already indicated that they were not eager to interrupt/reduce their television time, which is a typical evening activity [53]. Indeed, a recent study, investigating breaks in older adults’ sedentary behavior immediately after receiving personalized haptic feedback, showed that older adults were most likely to interrupt their sedentary behavior in the afternoon [54]. To draw firm conclusions on the optimal time period to provide sedentary behavior support, future researchers are recommended to combine data collection on when older adults are susceptible to sedentary behavior, and when older adults are willing to receive, process and utilize support [13, 14]. Next to the potential of evening interventions, the current results also emphasize that total sedentary time, as well as diurnal patterns of sedentary time differ greatly depending on socio-demographic factors, physical functioning and geographical location. Hence, personalized JITAI’s, in which the delivery of support is tailored to an individual’s need over time, may be preferred over a one size fits all approach.

### Strengths and limitations

This study has several strengths. Firstly, no previous studies have examined the patterns of sedentary time in different countries. Including Asian studies in behavioral research is useful as Asian populations are quite different from Western populations in terms of social, cultural, physical and political environment and Asia is the continent where most of the global population resides. Secondly, this is the first study to examine if the variation in sedentary time throughout the day differs depending on physical functioning. Physical functioning is an important component of healthy aging, and deserves attention as a moderator in the daily patterns of sedentary behavior. Thirdly, the combination of examining intra -and interpersonal variability in older adults’ sedentary behavior is novel, and offers important information for the development of future JITAI’s. There are also a number of limitations. First, although hip-worn accelerometers were validated for the measurement of sedentary behavior [55], it should be acknowledged that hip-worn accelerometers are not able to distinguish sitting from standing still. As such, some periods of standing still, or very light-intensity physical activity may have been misclassified as sedentary behavior. Secondly, the response rate in Ghent was rather low (i.e., 45%) suggesting that sampling bias may have occurred. Indeed, the Ghent sample was highly educated compared to the general Flemish population, and mainly motivated and active older adults may have participated in the study, as participation was voluntary. Thirdly, efforts were made to include different geographical locations, however, the findings from these two cities may not be generalized to other (low-income) countries. Similarly, only older adults that were able to walk a couple of meters without assistance were considered to be eligible, and the current results may not be applicable for less mobile older adults. Finally, the data were collected ten years ago. Important events or changes, such as the Covid-19 pandemic, might have affected older adults’ lifestyles [56]. As such, the variability in sedentary behavior may differ for the current cohort of older adults. However, we are approaching the end of the Covid-9 pandemic and behavior patterns might revert to those of the pre-pandemic period in a relatively short time.

## Conclusions

This study provides useful insights into the variation of older adults’ sedentary time. The findings highlight that older adults’ sedentary time varied considerably over the course of the day, and between population subgroups. Older adults were most sedentary in the evening, which makes this the most appropriate period of the day to deliver behavioral support. Those who were oldest, were men, had lowest levels of physical functioning and lived in Hong Kong were the most sedentary population subgroups. Consequently, these subgroups should receive particular attention in future sedentary behavior interventions. Given the differences in daily patterns of sedentary time according to socio-demographics and physical functioning, tailored (just-in-time adaptive) interventions are recommended.

## Supplementary Information

**Additional file 1.** Accel long belgium.

**Additional file 2:** **Table S1.** Interaction effects of day of week on accelerometer-assessed sedentary time.

## Data Availability

Hong Kong data cannot be shared publicly because of ethics restrictions, as the participants consented to provide their data to the project investigators only. Data are available from the University of Hong Kong (contact via Ester Cerin; Ester.Cerin@acu.edu.au) for researchers who meet the criteria for access to confidential data upon approval of the Human Research Ethics Committee of the University of Hong Kong (hrec_data@hku.hk). Belgian data are publicly available. All relevant Belgian data are within the manuscript and its Supporting Information files.
