# Supplementary material for: The role of socio-demographic factors and physical functioning in the intra- and interpersonal variability of older adults’ sedentary time: an observational two-country study
Source: BMC Geriatr. 2022 Jun 9;22:495. doi: 10.1186/s12877-022-03186-1 (PMC9178546; doi:10.1186/s12877-022-03186-1)
Supplement: Supplementary file 2 — Additional file 2: Table S1. Interaction effects of day of week on accelerometer-assessed sedentary time. [file 12877_2022_3186_MOESM2_ESM.docx]

**Supplementary file**

**Table S1. Interaction effects of day of week on accelerometer-assessed sedentary time**

| **Interaction term** | ***b* or χ^2^(4)** | **95% CI or *p*-value** | **Interaction term** | ***b* or χ^2^(4)** | **95% CI or *p*-value** |
| --- | --- | --- | --- | --- | --- |
| *Two-way interactions* |  |  | *Three-way interactions* |  |  |
| Day of week by Age | -0.03 | -0.07, 0.01 | Day of week by Age by Study site | 0.03 | -0.05, 0.12 |
| Day of week by Sex | 0.13 | -0.41, 0.67 | Day of week by Sex by Study site | -0.03 | -1.13, 1.06 |
| Day of week by Education | -0.12 | -0.66, 0.41 | Day of week by Education by Study site | 0.12 | -1.02, 1.25 |
| Day of week by Marital status | 0.12 | -0.43, 0.67 | Day of week by Marital status by Study site | -0.25 | -1.36, 0.85 |
| Day of week by Physical function | 0.02 | -0.11, 0.16 | Day of week by Physical function by Study site | -0.002 | -0.28, 0.27 |
| Day of week by Time of day^a^ | 5.35 | 0.253 | Day of week by Time of day by Study site^a^ | 9.01 | 0.061 |
| Day of week by Study site | -0.20 | -0.73, 0.32 |  |  |  |

*Notes.* All models with random intercepts at the neighbourhood, person and day level, and adjusted for accelerometer wear time, age, sex, educational attainment, marital status, physical function and study site; *b*, regression coefficient; CI, confidence intervals; χ^2^, chi square; results from χ^2^(4) test because Time of day is a nominal variable with more than two categories.
