# Supplementary material for: The role of socio-demographic factors and physical functioning in the intra- and interpersonal variability of older adults’ sedentary time: an observational two-country study
Source: BMC Geriatr. 2022 Jun 9;22:495. doi: 10.1186/s12877-022-03186-1 (PMC9178546; doi:10.1186/s12877-022-03186-1)
Supplement: Supplementary file 1 — Additional file 1. Accel long belgium. [file 12877_2022_3186_MOESM1_ESM.pdf]

```
,"Person_ID","numdate","period","dayofweeknum","TypeDay","st","lpa","mvpa","wear","validp","S
1,1,2010-11-13,0,6,1,21,38,1,60,1,2,207,76,0,0,1,1,1,11,971.525907,1145.351723,0.830793247,4.32
2,1,2010-11-13,1,6,1,34,26,0,60,3,2,207,76,0,0,1,1,1,11,971.525907,1145.351723,0.830793247,4.32
3,1,2010-11-13,2,6,1,55,5,0,60,3,2,207,76,0,0,1,1,1,11,971.525907,1145.351723,0.830793247,4.32
4,1,2010-11-13,3,6,1,39,21,0,60,3,2,207,76,0,0,1,1,1,11,971.525907,1145.351723,0.830793247,4.32
5,1,2010-11-13,4,6,1,49,5,1,55,1,2,207,76,0,0,1,1,1,11,971.525907,1145.351723,0.830793247,4.32
6,1,2010-11-14,0,0,1,21,23.75,1.5,46.25,3,2,207,76,0,0,1,1,1,11,971.525907,1145.351723,0.830793
7,1,2010-11-14,1,0,1,14,41,5,60,3,2,207,76,0,0,1,1,1,11,971.525907,1145.351723,0.830793247,4.32
8,1,2010-11-14,2,0,1,45,14,1,60,3,2,207,76,0,0,1,1,1,11,971.525907,1145.351723,0.830793247,4.32
9,1,2010-11-14,3,0,1,43,17,0,60,3,2,207,76,0,0,1,1,1,11,971.525907,1145.351723,0.830793247,4.32
10,1,2010-11-14,4,0,1,54.6666679382324,5.33333349227905,0,60,3,2,207,76,0,0,1,1,1,11,971.525
11,1,2010-11-15,0,1,0,20.3333339691162,32.3333320617676,4.33333349227905,57,3,2,207,76,0,0
12,1,2010-11-15,1,1,0,8.25,29.25,22.5,60,3,2,207,76,0,0,1,1,1,11,971.525907,1145.351723,0.83079
13,1,2010-11-15,2,1,0,37,21.6666660308838,1.33333337306976,60,3,2,207,76,0,0,1,1,1,11,971.525
14,1,2010-11-15,3,1,0,37.3333320617676,20,2.66666674613953,60,3,2,207,76,0,0,1,1,1,11,971.525
15,1,2010-11-15,4,1,0,50,9.66666698455811,0.333333343267441,60,3,2,207,76,0,0,1,1,1,11,971.52
16,1,2010-11-16,0,2,0,15,38.5,0.5,54,2,2,207,76,0,0,1,1,1,11,971.525907,1145.351723,0.83079324
17,1,2010-11-16,1,2,0,17,36.75,6.25,60,3,2,207,76,0,0,1,1,1,11,971.525907,1145.351723,0.830793
18,1,2010-11-16,2,2,0,20.6666660308838,15,24.3333339691162,60,3,2,207,76,0,0,1,1,1,11,971.525
19,1,2010-11-16,3,2,0,28.6666660308838,16.6666660308838,14.6666669845581,60,3,2,207,76,0,0
20,1,2010-11-16,4,2,0,50,10,0,60,3,2,207,76,0,0,1,1,1,11,971.525907,1145.351723,0.830793247,4.
21,1,2010-11-17,0,3,0,8.66666698455811,30.6666660308838,7.33333349227905,46.6666679382
22,1,2010-11-17,1,3,0,6.66666650772095,25.6666660308838,27.6666660308838,60,3,2,207,76,0,0
23,1,2010-11-17,2,3,0,31,14.3333330154419,14.6666669845581,60,3,2,207,76,0,0,1,1,1,11,971.525
24,1,2010-11-17,3,3,0,35.6666679382324,23.3333339691162,1,60,3,2,207,76,0,0,1,1,1,11,971.525
25,1,2010-11-17,4,3,0,53.6666679382324,6.33333349227905,0,60,3,2,207,76,0,0,1,1,1,11,971.525
26,1,2010-11-18,0,4,0,27.3333339691162,20,1,48.3333320617676,3,2,207,76,0,0,1,1,1,11,971.525
27,1,2010-11-18,1,4,0,9.25,43.75,7,60,3,2,207,76,0,0,1,1,1,11,971.525907,1145.351723,0.8307932
28,1,2010-11-18,2,4,0,18,39.6666679382324,2.33333325386047,60,3,2,207,76,0,0,1,1,1,11,971.525
29,1,2010-11-18,3,4,0,21.3333339691162,27.6666660308838,11,60,3,2,207,76,0,0,1,1,1,11,971.525
30,1,2010-11-18,4,4,0,48.6666679382324,11.3333330154419,0,60,3,2,207,76,0,0,1,1,1,11,971.525
31,1,2010-11-19,0,5,0,16.5,22,15.5,54,2,2,207,76,0,0,1,1,1,11,971.525907,1145.351723,0.8307932
32,1,2010-11-19,1,5,0,15,39.6666679382324,5.33333349227905,60,3,2,207,76,0,0,1,1,1,11,971.525
33,1,2010-11-19,2,5,0,15.1999998092651,31.2000007629395,13.6000003814697,60,3,2,207,76,0,0
34,1,2010-11-19,3,5,0,30,21,9,60,3,2,207,76,0,0,1,1,1,11,971.525907,1145.351723,0.830793247,4.
35,1,2010-11-19,4,5,0,51.3333320617676,8.66666698455811,0,60,3,2,207,76,0,0,1,1,1,11,971.525
36,1,2010-11-20,0,6,1,28,14.3333330154419,0.666666686534882,43,3,2,207,76,0,0,1,1,1,11,971.52
37,1,2010-11-20,1,6,1,26.3333339691162,33.3333320617676,0.333333343267441,60,3,2,207,76,0,
38,1,2010-11-20,2,6,1,33.75,24.25,2,60,3,2,207,76,0,0,1,1,1,11,971.525907,1145.351723,0.830793
39,1,2010-11-20,3,6,1,24,31.75,4.25,60,3,2,207,76,0,0,1,1,1,11,971.525907,1145.351723,0.830793
40,1,2010-11-20,4,6,1,44,16,0,60,3,2,207,76,0,0,1,1,1,11,971.525907,1145.351723,0.830793247,4.
41,1,2010-11-21,3,0,1,45.5,14.5,0,60,2,2,207,76,0,0,1,1,1,11,971.525907,1145.351723,0.83079324
42,1,2010-11-21,4,0,1,36,5,1,42,1,2,207,76,0,0,1,1,1,11,971.525907,1145.351723,0.830793247,4.32
43,3,2010-11-13,1,6,1,9.5,44,3.5,57,3,2,207,74,0,0,0,1,1,8,859.58217,1004.655036,0.579601432,0,
44,3,2010-11-13,2,6,1,31.6666660308838,27.3333339691162,1,60,3,2,207,74,0,0,0,1,1,8,859.5821
45,3,2010-11-13,3,6,1,41.6666679382324,15.3333330154419,3,60,3,2,207,74,0,0,0,1,1,8,859.5821
46,3,2010-11-13,4,6,1,37,21.3333339691162,1.66666662693024,60,3,2,207,74,0,0,0,1,1,8,859.582
47,3,2010-11-14,1,0,1,16.5,42.75,0.75,60,3,2,207,74,0,0,0,1,1,8,859.58217,1004.655036,0.579601
48,3,2010-11-14,2,0,1,12.8000001907349,45.5999984741211,1.60000002384186,60,3,2,207,74,0,0
49,3,2010-11-14,3,0,1,12.3333330154419,44.6666679382324,3,60,3,2,207,74,0,0,0,1,1,8,859.5821
```

50,3,2010-11-14,4,0,1,46,14,0,60,3,2,207,74,0,0,0,1,1,8,859.58217,1004.655036,0.579601432,0,0.0
51,3,2010-11-15,2,1,0,29,30.75,0,59.75,3,2,207,74,0,0,0,1,1,8,859.58217,1004.655036,0.57960143
52,3,2010-11-15,3,1,0,34,26,0,60,3,2,207,74,0,0,0,1,1,8,859.58217,1004.655036,0.579601432,0,0.0
53,3,2010-11-15,4,1,0,39.25,20.75,0,60,3,2,207,74,0,0,0,1,1,8,859.58217,1004.655036,0.57960143
54,3,2010-11-16,0,2,0,2,17,0,19,1,2,207,74,0,0,0,1,1,8,859.58217,1004.655036,0.579601432,0,0.08
55,3,2010-11-16,1,2,0,30,29.3333339691162,0.666666686534882,60,3,2,207,74,0,0,0,1,1,8,859.58
56,3,2010-11-16,2,2,0,48.6666679382324,11.3333330154419,0,60,3,2,207,74,0,0,0,1,1,8,859.5821
57,3,2010-11-16,3,2,0,28.3333339691162,31.3333339691162,0.333333343267441,60,3,2,207,74,0,
58,3,2010-11-16,4,2,0,46.5999984741211,13.3999996185303,0,60,3,2,207,74,0,0,0,1,1,8,859.5821
59,3,2010-11-17,0,3,0,0,2,0,2,2,2,207,74,0,0,0,1,1,8,859.58217,1004.655036,0.579601432,0,0.0873
60,3,2010-11-17,1,3,0,4.5,29,0,33.5,2,2,207,74,0,0,0,1,1,8,859.58217,1004.655036,0.579601432,0,
61,3,2010-11-17,2,3,0,31.3333339691162,26.3333339691162,2.33333325386047,60,3,2,207,74,0,0
62,3,2010-11-17,3,3,0,24.6666660308838,32.3333320617676,3,60,3,2,207,74,0,0,0,1,1,8,859.5821
63,3,2010-11-17,4,3,0,34.3333320617676,25.6666660308838,0,60,3,2,207,74,0,0,0,1,1,8,859.5821
64,3,2010-11-18,1,4,0,7.66666650772095,51.3333320617676,1,60,3,2,207,74,0,0,0,1,1,8,859.5821
65,3,2010-11-18,2,4,0,16.6666660308838,42.6666679382324,0.666666686534882,60,3,2,207,74,0,
66,3,2010-11-18,3,4,0,14.6666669845581,42.3333320617676,3,60,3,2,207,74,0,0,0,1,1,8,859.5821
67,3,2010-11-18,4,4,0,31,18,0,49,2,2,207,74,0,0,0,1,1,8,859.58217,1004.655036,0.579601432,0,0.0
68,3,2010-11-21,0,0,1,34,23,3,60,1,2,207,74,0,0,0,1,1,8,859.58217,1004.655036,0.579601432,0,0.0
69,3,2010-11-21,1,0,1,13.5,40.75,2.75,57,3,2,207,74,0,0,0,1,1,8,859.58217,1004.655036,0.5796014
70,3,2010-11-21,2,0,1,19,40.6666679382324,0.333333343267441,60,3,2,207,74,0,0,0,1,1,8,859.58
71,3,2010-11-21,3,0,1,39.3333320617676,20,0.666666686534882,60,3,2,207,74,0,0,0,1,1,8,859.58
72,3,2010-11-21,4,0,1,30,24.5,0,54.5,2,2,207,74,0,0,0,1,1,8,859.58217,1004.655036,0.579601432,0
73,5,2010-11-13,1,6,1,16.3333339691162,42.6666679382324,1,60,3,2,207,85,1,1,0,1,1,9,942.38401
74,5,2010-11-13,2,6,1,17,43,0,60,3,2,207,85,1,1,0,1,1,9,942.3840143,1249.63125,0.892739755,6.9
75,5,2010-11-13,3,6,1,32,28,0,60,3,2,207,85,1,1,0,1,1,9,942.3840143,1249.63125,0.892739755,6.9
76,5,2010-11-13,4,6,1,46,14,0,60,3,2,207,85,1,1,0,1,1,9,942.3840143,1249.63125,0.892739755,6.9
77,5,2010-11-14,0,0,1,2,17,0,19,1,2,207,85,1,1,0,1,1,9,942.3840143,1249.63125,0.892739755,6.92
78,5,2010-11-14,1,0,1,29.5,29.5,1,60,2,2,207,85,1,1,0,1,1,9,942.3840143,1249.63125,0.892739755,
79,5,2010-11-14,2,0,1,34.6666679382324,25,0,59.6666679382324,3,2,207,85,1,1,0,1,1,9,942.38401
80,5,2010-11-14,3,0,1,32.3333320617676,27.6666660308838,0,60,3,2,207,85,1,1,0,1,1,9,942.38401
81,5,2010-11-14,4,0,1,42.8333320617676,17.1666660308838,0,60,3,2,207,85,1,1,0,1,1,9,942.38401
82,5,2010-11-15,0,1,0,0,1,0,1,1,2,207,85,1,1,0,1,1,9,942.3840143,1249.63125,0.892739755,6.9236
83,5,2010-11-15,1,1,0,13.3333330154419,29,0,42.3333320617676,3,2,207,85,1,1,0,1,1,9,942.38401
84,5,2010-11-15,2,1,0,43.5,16.5,0,60,3,2,207,85,1,1,0,1,1,9,942.3840143,1249.63125,0.892739755,
85,5,2010-11-15,3,1,0,28.3333339691162,31.3333339691162,0.333333343267441,60,3,2,207,85,1,
86,5,2010-11-15,4,1,0,44.3333320617676,15.6666669845581,0,60,3,2,207,85,1,1,0,1,1,9,942.38401
87,5,2010-11-16,0,2,0,0,3,0,3,1,2,207,85,1,1,0,1,1,9,942.3840143,1249.63125,0.892739755,6.9236
88,5,2010-11-16,1,2,0,7.66666650772095,51.3333320617676,1,60,3,2,207,85,1,1,0,1,1,9,942.38401
89,5,2010-11-16,2,2,0,23.2000007629395,35,1.79999995231628,60,3,2,207,85,1,1,0,1,1,9,942.384
90,5,2010-11-16,3,2,0,24.6666660308838,32.3333320617676,3,60,3,2,207,85,1,1,0,1,1,9,942.38401
91,5,2010-11-16,4,2,0,34.3333320617676,25.6666660308838,0,60,3,2,207,85,1,1,0,1,1,9,942.38401
92,5,2010-11-17,3,3,0,14.6666669845581,42.3333320617676,3,60,3,2,207,85,1,1,0,1,1,9,942.38401
93,5,2010-11-17,4,3,0,31,18,0,49,2,2,207,85,1,1,0,1,1,9,942.3840143,1249.63125,0.892739755,6.9
94,5,2010-11-18,0,4,0,34,23,3,60,1,2,207,85,1,1,0,1,1,9,942.3840143,1249.63125,0.892739755,6.9
95,5,2010-11-18,1,4,0,15.6666669845581,41,3.33333325386047,60,3,2,207,85,1,1,0,1,1,9,942.384
96,5,2010-11-18,2,4,0,25,34,1,60,1,2,207,85,1,1,0,1,1,9,942.3840143,1249.63125,0.892739755,6.9
97,5,2010-11-19,1,5,0,9,46.6666679382324,0.333333343267441,56,3,2,207,85,1,1,0,1,1,9,942.384
98,5,2010-11-19,2,5,0,25.3999996185303,34,0.600000023841858,60,3,2,207,85,1,1,0,1,1,9,942.38
99,5,2010-11-19,3,5,0,37.5999984741211,20.7999992370605,1.60000002384186,60,3,2,207,85,1,1

100,5,2010-11-19,4,5,0,30,24.5,0,54.5,2,2,207,85,1,1,0,1,1,9,942.3840143,1249.63125,0.89273975
101,5,2010-11-20,1,6,1,11.6666669845581,39.6666679382324,4.66666650772095,56,3,2,207,85,1,
102,5,2010-11-20,2,6,1,8.33333301544189,49,2.66666674613953,60,3,2,207,85,1,1,0,1,1,9,942.384
103,5,2010-11-20,3,6,1,23.6666660308838,32.3333320617676,4,60,3,2,207,85,1,1,0,1,1,9,942.384
104,5,2010-11-20,4,6,1,37,21.3333339691162,1.66666662693024,60,3,2,207,85,1,1,0,1,1,9,942.384
105,8,2010-11-13,0,6,1,35,22,0,57,1,2,207,71,0,1,0,1,1,11,881.3318984,1314.527243,0.687147966,
106,8,2010-11-13,1,6,1,25.6666660308838,30.6666660308838,3.66666674613953,60,3,2,207,71,0,
107,8,2010-11-13,2,6,1,28.3333339691162,31.3333339691162,0.333333343267441,60,3,2,207,71,
108,8,2010-11-13,3,6,1,41,17.6666660308838,1.33333337306976,60,3,2,207,71,0,1,0,1,1,11,881.33
109,8,2010-11-13,4,6,1,43.6666679382324,16.1666660308838,0.16666667163372,60,3,2,207,71,0,
110,8,2010-11-14,0,0,1,12,10.5,0.5,23,2,2,207,71,0,1,0,1,1,11,881.3318984,1314.527243,0.687147
111,8,2010-11-14,1,0,1,35,23.1666660308838,1.83333337306976,60,3,2,207,71,0,1,0,1,1,11,881.33
112,8,2010-11-14,2,0,1,21,38.25,0.75,60,3,2,207,71,0,1,0,1,1,11,881.3318984,1314.527243,0.68714
113,8,2010-11-14,3,0,1,29.6666660308838,30.3333339691162,0,60,3,2,207,71,0,1,0,1,1,11,881.331
114,8,2010-11-14,4,0,1,37.6666679382324,22.3333339691162,0,60,3,2,207,71,0,1,0,1,1,11,881.331
115,8,2010-11-15,0,1,0,16,41,1,58,1,2,207,71,0,1,0,1,1,11,881.3318984,1314.527243,0.687147966,
116,8,2010-11-15,1,1,0,28.3333339691162,30,1.66666662693024,60,3,2,207,71,0,1,0,1,1,11,881.33
117,8,2010-11-15,2,1,0,35,22.3999996185303,2.59999990463257,60,3,2,207,71,0,1,0,1,1,11,881.33
118,8,2010-11-15,3,1,0,34,17.3999996185303,0.600000023841858,52,3,2,207,71,0,1,0,1,1,11,881.3
119,8,2010-11-15,4,1,0,36,18.5,0.5,55,2,2,207,71,0,1,0,1,1,11,881.3318984,1314.527243,0.687147
120,8,2010-11-16,4,2,0,46.3333320617676,13.6666669845581,0,60,3,2,207,71,0,1,0,1,1,11,881.331
121,8,2010-11-17,0,3,0,41,17,2,60,1,2,207,71,0,1,0,1,1,11,881.3318984,1314.527243,0.687147966,
122,8,2010-11-17,1,3,0,21.3333339691162,37.3333320617676,1.33333337306976,60,3,2,207,71,0,
123,8,2010-11-17,2,3,0,36.3333320617676,21.6666660308838,2,60,3,2,207,71,0,1,0,1,1,11,881.331
124,8,2010-11-17,3,3,0,51,9,0,60,1,2,207,71,0,1,0,1,1,11,881.3318984,1314.527243,0.687147966,0
125,8,2010-11-18,0,4,0,15,36.5,1.5,53,2,2,207,71,0,1,0,1,1,11,881.3318984,1314.527243,0.687147
126,8,2010-11-18,1,4,0,16.6666660308838,43.3333320617676,0,60,3,2,207,71,0,1,0,1,1,11,881.331
127,8,2010-11-18,2,4,0,30.6666660308838,22.6666660308838,6.66666650772095,60,3,2,207,71,0,
128,8,2010-11-18,3,4,0,41.7999992370605,17.7999992370605,0.400000005960464,60,3,2,207,71,
129,8,2010-11-18,4,4,0,36.6666679382324,22.6666660308838,0.666666686534882,60,3,2,207,71,
130,8,2010-11-19,0,5,0,33,27,0,60,1,2,207,71,0,1,0,1,1,11,881.3318984,1314.527243,0.687147966,
131,8,2010-11-19,1,5,0,20.3333339691162,38.3333320617676,1.33333337306976,60,3,2,207,71,0,
132,8,2010-11-19,2,5,0,44.3333320617676,15.3333330154419,0.333333343267441,60,3,2,207,71,
133,8,2010-11-19,3,5,0,42,15.3333330154419,2.66666674613953,60,3,2,207,71,0,1,0,1,1,11,881.33
134,8,2010-11-19,4,5,0,38.7999992370605,21.2000007629395,0,60,3,2,207,71,0,1,0,1,1,11,881.331
135,8,2010-11-20,0,6,1,28,23,1,52,2,2,207,71,0,1,0,1,1,11,881.3318984,1314.527243,0.687147966,
136,8,2010-11-20,1,6,1,33.6666679382324,24.6666660308838,1.66666662693024,60,3,2,207,71,0,
137,8,2010-11-20,2,6,1,32,27.6666660308838,0.333333343267441,60,3,2,207,71,0,1,0,1,1,11,881.3
138,8,2010-11-20,3,6,1,39.6666679382324,20,0.333333343267441,60,3,2,207,71,0,1,0,1,1,11,881.3
139,8,2010-11-20,4,6,1,34,25,1,60,1,2,207,71,0,1,0,1,1,11,881.3318984,1314.527243,0.687147966,
140,10,2010-11-16,0,2,0,26,12.5,0,38.5,2,2,207,69,1,1,1,1,1,12,907.3593828,1234.444667,0.723627
141,10,2010-11-16,1,2,0,22.3333339691162,34,3.66666674613953,60,3,2,207,69,1,1,1,1,1,12,907.3
142,10,2010-11-16,2,2,0,21.6666660308838,35.6666679382324,2.66666674613953,60,3,2,207,69,
143,10,2010-11-16,3,2,0,11.3333330154419,46.3333320617676,2.33333325386047,60,3,2,207,69,
144,10,2010-11-16,4,2,0,27.3333339691162,32.6666679382324,0,60,3,2,207,69,1,1,1,1,1,12,907.35
145,10,2010-11-17,0,3,0,10.5,26.5,0.5,37.5,2,2,207,69,1,1,1,1,1,12,907.3593828,1234.444667,0.723
146,10,2010-11-17,1,3,0,19,37.5999984741211,3.40000009536743,60,3,2,207,69,1,1,1,1,1,12,907.3
147,10,2010-11-17,2,3,0,21,37.3333320617676,1.66666662693024,60,3,2,207,69,1,1,1,1,1,12,907.3
148,10,2010-11-17,3,3,0,16.3333339691162,40,3.66666674613953,60,3,2,207,69,1,1,1,1,1,12,907.3
149,10,2010-11-17,4,3,0,35.3333320617676,24.6666660308838,0,60,3,2,207,69,1,1,1,1,1,12,907.35

150,10,2010-11-18,0,4,0,8,13,0,21,1,2,207,69,1,1,1,1,1,12,907.3593828,1234.444667,0.723627363,
151,10,2010-11-18,1,4,0,15,41.25,3.75,60,3,2,207,69,1,1,1,1,1,12,907.3593828,1234.444667,0.723
152,10,2010-11-18,2,4,0,15,40.5999984741211,4.40000009536743,60,3,2,207,69,1,1,1,1,1,12,907.
153,10,2010-11-18,3,4,0,16.3333339691162,35.6666679382324,8,60,3,2,207,69,1,1,1,1,1,12,907.3
154,10,2010-11-18,4,4,0,22.3333339691162,37.6666679382324,0,60,3,2,207,69,1,1,1,1,1,12,907.3
155,10,2010-11-19,0,5,0,12.3333330154419,20.6666660308838,1.33333337306976,34.333332061
156,10,2010-11-19,1,5,0,32.3333320617676,26.1666660308838,1.5,60,3,2,207,69,1,1,1,1,1,12,907.
157,10,2010-11-19,2,5,0,23.1666660308838,35.3333320617676,1.5,60,3,2,207,69,1,1,1,1,1,12,907.
158,10,2010-11-19,3,5,0,30.5555553436279,26.6666660308838,2.77777767181396,60,3,2,207,69,
159,10,2010-11-19,4,5,0,32,27.8333339691162,0.16666667163372,60,3,2,207,69,1,1,1,1,1,12,907.
160,10,2010-11-20,0,6,1,9.66666698455811,16,0.333333343267441,26,3,2,207,69,1,1,1,1,1,12,907
161,10,2010-11-20,1,6,1,20.1666660308838,31,8.83333301544189,60,3,2,207,69,1,1,1,1,1,12,907.
162,10,2010-11-20,2,6,1,24.8571434020996,33,2.14285707473755,60,3,2,207,69,1,1,1,1,1,12,907.
163,10,2010-11-20,3,6,1,20.8333339691162,32.8333320617676,6.33333349227905,60,3,2,207,69,
164,10,2010-11-20,4,6,1,24.125,35.125,0.75,60,3,2,207,69,1,1,1,1,1,12,907.3593828,1234.444667,
165,10,2010-11-21,4,0,1,51,9,0,60,1,2,207,69,1,1,1,1,1,12,907.3593828,1234.444667,0.723627363,
166,11,2010-11-21,0,0,1,42,17,1,60,1,2,207,65,1,1,0,1,1,10,905.5895508,1438.075827,1.09908711
167,11,2010-11-21,1,0,1,20.1666660308838,36.6666679382324,1,57.8333320617676,3,2,207,65,1,
168,11,2010-11-21,2,0,1,15,44.6666679382324,0.333333343267441,60,3,2,207,65,1,1,0,1,1,10,905
169,11,2010-11-21,3,0,1,24,34.3333320617676,1.66666662693024,60,3,2,207,65,1,1,0,1,1,10,905.
170,11,2010-11-21,4,0,1,15,34,0.666666686534882,49.6666679382324,3,2,207,65,1,1,0,1,1,10,905
171,11,2010-11-22,0,1,0,38,0,0,38,1,2,207,65,1,1,0,1,1,10,905.5895508,1438.075827,1.099087112,
172,11,2010-11-22,1,1,0,18.6666660308838,39.3333320617676,2,60,3,2,207,65,1,1,0,1,1,10,905.5
173,11,2010-11-22,2,1,0,8.33333301544189,50.3333320617676,1.33333337306976,60,3,2,207,65,
174,11,2010-11-22,3,1,0,17.3999996185303,40.7999992370605,1.79999995231628,60,3,2,207,65,
175,11,2010-11-22,4,1,0,22.6666660308838,26,0.666666686534882,49.3333320617676,3,2,207,6
176,11,2010-11-23,1,2,0,7,45.75,0.25,53,3,2,207,65,1,1,0,1,1,10,905.5895508,1438.075827,1.0990
177,11,2010-11-23,2,2,0,13,35.6666679382324,11.3333330154419,60,3,2,207,65,1,1,0,1,1,10,905.
178,11,2010-11-23,3,2,0,11.5,44,4.5,60,3,2,207,65,1,1,0,1,1,10,905.5895508,1438.075827,1.09908
179,11,2010-11-23,4,2,0,9.83333301544189,45.5,1.33333337306976,56.6666679382324,3,2,207,6
180,11,2010-11-24,0,3,0,22,14,3,39,1,2,207,65,1,1,0,1,1,10,905.5895508,1438.075827,1.09908711
181,11,2010-11-24,1,3,0,5.25,51.75,3,60,3,2,207,65,1,1,0,1,1,10,905.5895508,1438.075827,1.0990
182,11,2010-11-24,2,3,0,9.60000038146973,48.2000007629395,2.20000004768372,60,3,2,207,65,
183,11,2010-11-24,3,3,0,3.33333325386047,43.6666679382324,13,60,3,2,207,65,1,1,0,1,1,10,905.
184,11,2010-11-24,4,3,0,6,47,1,54,3,2,207,65,1,1,0,1,1,10,905.5895508,1438.075827,1.099087112,
185,11,2010-11-25,1,4,0,11.5,47,0,58.5,2,2,207,65,1,1,0,1,1,10,905.5895508,1438.075827,1.09908
186,11,2010-11-25,2,4,0,24.75,34.25,1,60,3,2,207,65,1,1,0,1,1,10,905.5895508,1438.075827,1.099
187,11,2010-11-25,3,4,0,30.6666660308838,29.1666660308838,0.16666667163372,60,3,2,207,65,
188,11,2010-11-25,4,4,0,32,27.6666660308838,0.333333343267441,60,3,2,207,65,1,1,0,1,1,10,905
189,11,2010-11-26,1,5,0,9,1,0,10,1,2,207,65,1,1,0,1,1,10,905.5895508,1438.075827,1.099087112,0
190,11,2010-11-26,2,5,0,53.6666679382324,4,2.33333325386047,60,3,2,207,65,1,1,0,1,1,10,905.5
191,11,2010-11-26,3,5,0,44.3333320617676,11.6666669845581,4,60,3,2,207,65,1,1,0,1,1,10,905.5
192,11,2010-11-26,4,5,0,35.8333320617676,22.5,1.66666662693024,60,3,2,207,65,1,1,0,1,1,10,90
193,12,2010-11-13,0,6,1,5,29,1,35,1,2,207,78,1,0,1,1,1,11,900.4003243,1487.145876,0.914130501,
194,12,2010-11-13,1,6,1,8,22,30,60,3,2,207,78,1,0,1,1,1,11,900.4003243,1487.145876,0.91413050
195,12,2010-11-13,2,6,1,19.3333339691162,17.3333339691162,23.3333339691162,60,3,2,207,78,
196,12,2010-11-13,3,6,1,47,13,0,60,3,2,207,78,1,0,1,1,1,11,900.4003243,1487.145876,0.91413050
197,12,2010-11-13,4,6,1,31.3333339691162,27.6666660308838,1,60,3,2,207,78,1,0,1,1,1,11,900.4
198,12,2010-11-14,0,0,1,9.5,32,7.5,49,2,2,207,78,1,0,1,1,1,11,900.4003243,1487.145876,0.914130
199,12,2010-11-14,1,0,1,0,6,54,60,1,2,207,78,1,0,1,1,1,11,900.4003243,1487.145876,0.914130501,

200,12,2010-11-14,2,0,1,1,33.6666679382324,25.3333339691162,60,3,2,207,78,1,0,1,1,1,11,900.4
201,12,2010-11-14,3,0,1,25.6666660308838,27.3333339691162,7,60,3,2,207,78,1,0,1,1,1,11,900.4
202,12,2010-11-14,4,0,1,20,40,0,60,3,2,207,78,1,0,1,1,1,11,900.4003243,1487.145876,0.914130501
203,12,2010-11-15,0,1,0,19,23,1,43,1,2,207,78,1,0,1,1,1,11,900.4003243,1487.145876,0.914130501
204,12,2010-11-15,1,1,0,11.6666669845581,28.6666660308838,19.6666660308838,60,3,2,207,78,
205,12,2010-11-15,2,1,0,18.6666660308838,37.3333320617676,4,60,3,2,207,78,1,0,1,1,1,11,900.4
206,12,2010-11-15,3,1,0,16.3333339691162,38.3333320617676,5.33333349227905,60,3,2,207,78,
207,12,2010-11-15,4,1,0,25,33.6666679382324,1.33333337306976,60,3,2,207,78,1,0,1,1,1,11,900.
208,12,2010-11-16,0,2,0,13.5,16,1.5,31,2,2,207,78,1,0,1,1,1,11,900.4003243,1487.145876,0.91413
209,12,2010-11-16,1,2,0,8,42,10,60,3,2,207,78,1,0,1,1,1,11,900.4003243,1487.145876,0.914130501
210,12,2010-11-16,2,2,0,34.6666679382324,21.3333339691162,4,60,3,2,207,78,1,0,1,1,1,11,900.4
211,12,2010-11-16,3,2,0,52,6,2,60,3,2,207,78,1,0,1,1,1,11,900.4003243,1487.145876,0.914130501,
212,12,2010-11-16,4,2,0,41.3333320617676,16.3333339691162,2.33333325386047,60,3,2,207,78,
213,12,2010-11-19,0,5,0,9,42,4,55,1,2,207,78,1,0,1,1,1,11,900.4003243,1487.145876,0.914130501,
214,12,2010-11-19,1,5,0,34.5,18.5,7,60,2,2,207,78,1,0,1,1,1,11,900.4003243,1487.145876,0.91413
215,12,2010-11-19,2,5,0,22,31,7,60,3,2,207,78,1,0,1,1,1,11,900.4003243,1487.145876,0.914130501
216,12,2010-11-19,3,5,0,43,16.6666660308838,0.333333343267441,60,3,2,207,78,1,0,1,1,1,11,900
217,12,2010-11-19,4,5,0,6.33333349227905,53.6666679382324,0,60,3,2,207,78,1,0,1,1,1,11,900.4
218,12,2010-11-20,0,6,1,8,22,0,30,1,2,207,78,1,0,1,1,1,11,900.4003243,1487.145876,0.914130501,
219,12,2010-11-20,1,6,1,31.8333339691162,25.8333339691162,2.33333325386047,60,3,2,207,78,
220,12,2010-11-20,2,6,1,38.3333320617676,18.3333339691162,3.33333325386047,60,3,2,207,78,
221,12,2010-11-20,3,6,1,49.6666679382324,7.33333349227905,3,60,3,2,207,78,1,0,1,1,1,11,900.4
222,12,2010-11-20,4,6,1,10,47.3333320617676,2.66666674613953,60,3,2,207,78,1,0,1,1,1,11,900.
223,12,2010-11-21,2,0,1,31.3333339691162,25.3333339691162,3.33333325386047,60,3,2,207,78,
224,12,2010-11-21,3,0,1,25.6666660308838,31.6666660308838,2.66666674613953,60,3,2,207,78,
225,12,2010-11-21,4,0,1,40,20,0,60,3,2,207,78,1,0,1,1,1,11,900.4003243,1487.145876,0.914130501
226,12,2010-11-26,0,5,0,32,27,1,60,1,2,207,78,1,0,1,1,1,11,900.4003243,1487.145876,0.914130501
227,12,2010-11-26,1,5,0,6,40,14,60,1,2,207,78,1,0,1,1,1,11,900.4003243,1487.145876,0.914130501
228,14,2010-11-21,0,0,1,7,4,0,11,1,2,207,72,1,1,0,1,1,9,879.5047608,1090.12904,0.689521342,0,0.
229,14,2010-11-21,1,0,1,52.6666679382324,5.33333349227905,2,60,3,2,207,72,1,1,0,1,1,9,879.50
230,14,2010-11-25,0,4,0,46,13,1,60,1,2,207,72,1,1,0,1,1,9,879.5047608,1090.12904,0.689521342,0
231,14,2010-11-25,1,4,0,9,43,8,60,3,2,207,72,1,1,0,1,1,9,879.5047608,1090.12904,0.689521342,0,
232,14,2010-11-25,2,4,0,35,20,5,60,3,2,207,72,1,1,0,1,1,9,879.5047608,1090.12904,0.689521342,0
233,14,2010-11-25,3,4,0,33.3333320617676,20.3333339691162,6.33333349227905,60,3,2,207,72,
234,14,2010-11-25,4,4,0,19,13.5,1,33.5,2,2,207,72,1,1,0,1,1,9,879.5047608,1090.12904,0.6895213
235,14,2011-02-19,0,6,1,15,36,5,56,1,2,207,72,1,1,0,1,1,9,879.5047608,1090.12904,0.689521342,0
236,14,2011-02-19,1,6,1,16.3333339691162,42.6666679382324,1,60,3,2,207,72,1,1,0,1,1,9,879.50
237,14,2011-02-19,2,6,1,10.3999996185303,43,6.59999990463257,60,3,2,207,72,1,1,0,1,1,9,879.5
238,14,2011-02-19,3,6,1,21.75,37,1.25,60,3,2,207,72,1,1,0,1,1,9,879.5047608,1090.12904,0.68952
239,14,2011-02-19,4,6,1,26.6666660308838,33,0.333333343267441,60,3,2,207,72,1,1,0,1,1,9,879.
240,14,2011-02-20,0,0,1,26,26,5,57,1,2,207,72,1,1,0,1,1,9,879.5047608,1090.12904,0.689521342,0
241,14,2011-02-20,1,0,1,2,40.6666679382324,17.3333339691162,60,3,2,207,72,1,1,0,1,1,9,879.50
242,14,2011-02-20,2,0,1,31.6666660308838,23.6666660308838,4.66666650772095,60,3,2,207,72,
243,14,2011-02-20,3,0,1,19,39.5999984741211,1.39999997615814,60,3,2,207,72,1,1,0,1,1,9,879.5
244,14,2011-02-20,4,0,1,28.3999996185303,30.7999992370605,0.800000011920929,60,3,2,207,72
245,14,2011-02-21,0,1,0,11,32.6666679382324,4,47.6666679382324,3,2,207,72,1,1,0,1,1,9,879.50
246,14,2011-02-21,1,1,0,20.6666660308838,37.6666679382324,1.66666662693024,60,3,2,207,72,
247,14,2011-02-21,2,1,0,12.3333330154419,40.3333320617676,7.33333349227905,60,3,2,207,72,
248,14,2011-02-21,3,1,0,14,41.3333320617676,4.66666650772095,60,3,2,207,72,1,1,0,1,1,9,879.5
249,14,2011-02-21,4,1,0,39,20.25,0.75,60,3,2,207,72,1,1,0,1,1,9,879.5047608,1090.12904,0.68952

250,14,2011-02-22,0,2,0,21.5,24,0.5,46,2,2,207,72,1,1,0,1,1,9,879.5047608,1090.12904,0.6895213
251,14,2011-02-22,1,2,0,10.1999998092651,45.2000007629395,4.59999990463257,60,3,2,207,72,
252,14,2011-02-22,2,2,0,16.3333339691162,41.6666679382324,2,60,3,2,207,72,1,1,0,1,1,9,879.50
253,14,2011-02-22,3,2,0,24.6666660308838,34.3333320617676,1,60,3,2,207,72,1,1,0,1,1,9,879.50
254,14,2011-02-22,4,2,0,33,26,1,60,3,2,207,72,1,1,0,1,1,9,879.5047608,1090.12904,0.689521342,0
255,14,2011-02-23,0,3,0,28,21,0.5,49.5,2,2,207,72,1,1,0,1,1,9,879.5047608,1090.12904,0.6895213
256,14,2011-02-23,1,3,0,27.25,28.5,4.25,60,3,2,207,72,1,1,0,1,1,9,879.5047608,1090.12904,0.6895
257,14,2011-02-23,2,3,0,32,27.3999996185303,0.600000023841858,60,3,2,207,72,1,1,0,1,1,9,879.
258,14,2011-02-23,3,3,0,15.3333330154419,37.3333320617676,7.33333349227905,60,3,2,207,72,
259,14,2011-02-23,4,3,0,40.3333320617676,19.6666660308838,0,60,3,2,207,72,1,1,0,1,1,9,879.50
260,14,2011-02-24,2,4,0,18,42,0,60,1,2,207,72,1,1,0,1,1,9,879.5047608,1090.12904,0.689521342,0
261,14,2011-02-24,3,4,0,14.3333330154419,43,2.66666674613953,60,3,2,207,72,1,1,0,1,1,9,879.5
262,14,2011-02-24,4,4,0,1,16,1,18,1,2,207,72,1,1,0,1,1,9,879.5047608,1090.12904,0.689521342,0,
263,16,2010-11-20,1,6,1,11,40.6666679382324,0.666666686534882,52.3333320617676,3,2,207,70
264,16,2010-11-20,2,6,1,24.3333339691162,31.8333339691162,3.83333325386047,60,3,2,207,70,
265,16,2010-11-20,3,6,1,19.75,35.5,4.75,60,3,2,207,70,0,1,1,1,1,12,888.4749243,927.3234014,0.76
266,16,2010-11-20,4,6,1,41.3333320617676,18.3333339691162,0.333333343267441,60,3,2,207,70
267,16,2010-11-21,1,0,1,29.3333339691162,18.3333339691162,1,48.6666679382324,3,2,207,70,0,
268,16,2010-11-21,2,0,1,34,26,0,60,3,2,207,70,0,1,1,1,1,12,888.4749243,927.3234014,0.76007364
269,16,2010-11-21,3,0,1,40.2000007629395,18.6000003814697,1.20000004768372,60,3,2,207,70,
270,16,2010-11-21,4,0,1,35.7999992370605,24,0.200000002980232,60,3,2,207,70,0,1,1,1,1,12,888
271,16,2010-11-22,1,1,0,24.6666660308838,30,2,56.6666679382324,3,2,207,70,0,1,1,1,1,12,888.4
272,16,2010-11-22,2,1,0,46,14,0,60,3,2,207,70,0,1,1,1,1,12,888.4749243,927.3234014,0.76007364
273,16,2010-11-22,3,1,0,36,22.6666660308838,1.33333337306976,60,3,2,207,70,0,1,1,1,1,12,888.
274,16,2010-11-22,4,1,0,41.5,18.25,0.25,60,3,2,207,70,0,1,1,1,1,12,888.4749243,927.3234014,0.76
275,16,2010-11-23,2,2,0,34.6666679382324,24.6666660308838,0.666666686534882,60,3,2,207,70
276,16,2010-11-23,3,2,0,47.6666679382324,12,0.333333343267441,60,3,2,207,70,0,1,1,1,1,12,888
277,16,2010-11-23,4,2,0,37.6666679382324,22.3333339691162,0,60,3,2,207,70,0,1,1,1,1,12,888.4
278,16,2010-12-09,1,4,0,32.3333320617676,17.6666660308838,1,51,3,2,207,70,0,1,1,1,1,12,888.4
279,16,2010-12-09,2,4,0,42,15.6666669845581,2.33333325386047,60,3,2,207,70,0,1,1,1,1,12,888.
280,16,2010-12-09,3,4,0,50.3333320617676,9.33333301544189,0.333333343267441,60,3,2,207,70
281,16,2010-12-09,4,4,0,42.3333320617676,17.3333339691162,0.333333343267441,60,3,2,207,70
282,16,2011-02-24,1,4,0,4.66666650772095,29,14.6666669845581,48.3333320617676,3,2,207,70,
283,16,2011-02-24,2,4,0,20.3333339691162,32.6666679382324,7,60,3,2,207,70,0,1,1,1,1,12,888.4
284,16,2011-02-24,3,4,0,39,16.6666660308838,4.33333349227905,60,3,2,207,70,0,1,1,1,1,12,888.
285,16,2011-02-24,4,4,0,43.5,16.5,0,60,2,2,207,70,0,1,1,1,1,12,888.4749243,927.3234014,0.760073
286,16,2011-02-25,0,5,0,8,23.5,2,33.5,2,2,207,70,0,1,1,1,1,12,888.4749243,927.3234014,0.760073
287,16,2011-02-25,1,5,0,8.16666698455811,50.6666679382324,0.5,59.3333320617676,3,2,207,70,
288,16,2011-02-25,2,5,0,17,28.3333339691162,14.6666669845581,60,3,2,207,70,0,1,1,1,1,12,888.
289,16,2011-02-25,3,5,0,39.3333320617676,20,0.666666686534882,60,3,2,207,70,0,1,1,1,1,12,888
290,16,2011-02-25,4,5,0,36.75,21,2.25,60,3,2,207,70,0,1,1,1,1,12,888.4749243,927.3234014,0.760
291,17,2010-11-19,1,5,0,15.5,32.75,4.5,52.75,3,2,207,71,0,1,0,1,1,12,889.2661652,1098.183314,0.
292,17,2010-11-19,2,5,0,27,25.3999996185303,7.59999990463257,60,3,2,207,71,0,1,0,1,1,12,889.
293,17,2010-11-19,3,5,0,45.6666679382324,14.3333330154419,0,60,3,2,207,71,0,1,0,1,1,12,889.2
294,17,2010-11-19,4,5,0,34.3333320617676,25.3333339691162,0.333333343267441,60,3,2,207,71
295,17,2010-11-20,1,6,1,37.3333320617676,21.3333339691162,0.333333343267441,59,3,2,207,71
296,17,2010-11-20,2,6,1,33.5,21.5,5,60,3,2,207,71,0,1,0,1,1,12,889.2661652,1098.183314,0.727891
297,17,2010-11-20,3,6,1,36,23.3333339691162,0.666666686534882,60,3,2,207,71,0,1,0,1,1,12,889
298,17,2010-11-20,4,6,1,25.3333339691162,32.6666679382324,2,60,3,2,207,71,0,1,0,1,1,12,889.2
299,17,2010-11-21,0,0,1,30.5,23,1,54.5,2,2,207,71,0,1,0,1,1,12,889.2661652,1098.183314,0.727891

300,17,2010-11-21,1,0,1,37.3333320617676,20,2.66666674613953,60,3,2,207,71,0,1,0,1,1,12,889.
301,17,2010-11-21,2,0,1,31.3333339691162,28,0.666666686534882,60,3,2,207,71,0,1,0,1,1,12,889
302,17,2010-11-21,3,0,1,37.3333320617676,21.3333339691162,1.33333337306976,60,3,2,207,71,
303,17,2010-11-21,4,0,1,38.3333320617676,21.1666660308838,0.5,60,3,2,207,71,0,1,0,1,1,12,889.
304,17,2010-11-23,0,2,0,59,0,1,60,1,2,207,71,0,1,0,1,1,12,889.2661652,1098.183314,0.727891498,
305,17,2010-11-23,1,2,0,38.6666679382324,21.3333339691162,0,60,3,2,207,71,0,1,0,1,1,12,889.2
306,17,2010-11-23,2,2,0,28.6666660308838,25.3333339691162,6,60,3,2,207,71,0,1,0,1,1,12,889.2
307,17,2010-11-24,0,3,0,0,11,1,12,1,2,207,71,0,1,0,1,1,12,889.2661652,1098.183314,0.727891498,
308,17,2010-11-24,1,3,0,19.3333339691162,39.3333320617676,1.33333337306976,60,3,2,207,71,
309,17,2010-11-24,2,3,0,47.3333320617676,12.3333330154419,0.333333343267441,60,3,2,207,71
310,17,2010-11-24,3,3,0,35.1666679382324,17,7.83333349227905,60,3,2,207,71,0,1,0,1,1,12,889.
311,17,2010-11-24,4,3,0,34,24,2,60,3,2,207,71,0,1,0,1,1,12,889.2661652,1098.183314,0.727891498
312,17,2010-11-25,0,4,0,2,9.5,0,11.5,2,2,207,71,0,1,0,1,1,12,889.2661652,1098.183314,0.7278914
313,17,2010-11-25,1,4,0,41.6666679382324,18,0.333333343267441,60,3,2,207,71,0,1,0,1,1,12,889
314,17,2010-11-25,2,4,0,30,29.6666660308838,0.333333343267441,60,3,2,207,71,0,1,0,1,1,12,889
315,17,2010-11-25,3,4,0,40.3333320617676,18.6666660308838,1,60,3,2,207,71,0,1,0,1,1,12,889.2
316,17,2010-11-25,4,4,0,42.3333320617676,17.6666660308838,0,60,3,2,207,71,0,1,0,1,1,12,889.2
317,17,2010-11-26,1,5,0,23.5,25.5,1.25,50.25,3,2,207,71,0,1,0,1,1,12,889.2661652,1098.183314,0.
318,17,2010-11-26,2,5,0,33,27,0,60,3,2,207,71,0,1,0,1,1,12,889.2661652,1098.183314,0.727891498
319,17,2010-11-26,3,5,0,23,36.6666679382324,0.333333343267441,60,3,2,207,71,0,1,0,1,1,12,889
320,17,2010-11-26,4,5,0,41,18.3333339691162,0.666666686534882,60,3,2,207,71,0,1,0,1,1,12,889
321,20,2010-11-21,0,0,1,60,0,0,60,1,2,207,70,1,1,0,1,1,11,889.2661652,1098.183314,0.727891498,
322,20,2010-11-22,0,1,0,4,8,0,12,1,2,207,70,1,1,0,1,1,11,889.2661652,1098.183314,0.727891498,4
323,20,2010-11-22,1,1,0,11,38.8333320617676,10.1666669845581,60,3,2,207,70,1,1,0,1,1,11,889.
324,20,2010-11-22,2,1,0,12.3333330154419,47.3333320617676,0.333333343267441,60,3,2,207,70
325,20,2010-11-22,3,1,0,33.6666679382324,25,1.33333337306976,60,3,2,207,70,1,1,0,1,1,11,889.
326,20,2010-11-22,4,1,0,36,20.6666660308838,3.33333325386047,60,3,2,207,70,1,1,0,1,1,11,889.
327,20,2010-11-23,1,2,0,27.3333339691162,22.3333339691162,0.333333343267441,50,3,2,207,70
328,20,2010-11-23,2,2,0,21.3333339691162,33.1666679382324,5.5,60,3,2,207,70,1,1,0,1,1,11,889.
329,20,2010-11-23,3,2,0,26.3333339691162,32.6666679382324,1,60,3,2,207,70,1,1,0,1,1,11,889.2
330,20,2010-11-23,4,2,0,35.6666679382324,21,3.33333325386047,60,3,2,207,70,1,1,0,1,1,11,889.
331,20,2010-11-24,1,3,0,19,25,0.333333343267441,44.3333320617676,3,2,207,70,1,1,0,1,1,11,889
332,20,2010-11-24,2,3,0,50.6666679382324,9.33333301544189,0,60,3,2,207,70,1,1,0,1,1,11,889.2
333,20,2010-11-24,3,3,0,33,25,2,60,3,2,207,70,1,1,0,1,1,11,889.2661652,1098.183314,0.727891498
334,20,2010-11-24,4,3,0,32.5,25.5,2,60,3,2,207,70,1,1,0,1,1,11,889.2661652,1098.183314,0.727891
335,20,2010-11-25,1,4,0,12.3999996185303,20.7999992370605,4.80000019073486,38,3,2,207,70,
336,20,2010-11-25,2,4,0,29,27.3333339691162,3.66666674613953,60,3,2,207,70,1,1,0,1,1,11,889.
337,20,2010-11-25,3,4,0,32.6666679382324,25.3333339691162,2,60,3,2,207,70,1,1,0,1,1,11,889.2
338,20,2010-11-25,4,4,0,40.4000015258789,18.2000007629395,1.20000004768372,59.799999237
339,20,2010-11-26,1,5,0,20.6666660308838,38.6666679382324,0,59.3333320617676,3,2,207,70,1,
340,20,2010-11-26,2,5,0,17.8333339691162,37.5,4.66666650772095,60,3,2,207,70,1,1,0,1,1,11,889
341,20,2010-11-26,3,5,0,25.5,33.25,1.25,60,3,2,207,70,1,1,0,1,1,11,889.2661652,1098.183314,0.72
342,20,2010-11-26,4,5,0,31.3333339691162,27.6666660308838,1,60,3,2,207,70,1,1,0,1,1,11,889.2
343,20,2010-12-01,0,3,0,15,24.5,1.5,41,2,2,207,70,1,1,0,1,1,11,889.2661652,1098.183314,0.727891
344,20,2010-12-01,1,3,0,17.3333339691162,38.3333320617676,4.33333349227905,60,3,2,207,70,
345,20,2010-12-01,2,3,0,39.6666679382324,19.6666660308838,0.666666686534882,60,3,2,207,70
346,20,2010-12-01,3,3,0,39.2000007629395,20.6000003814697,0.200000002980232,60,3,2,207,70
347,20,2010-12-01,4,3,0,39.3333320617676,20.6666660308838,0,60,3,2,207,70,1,1,0,1,1,11,889.2
348,20,2010-12-02,1,4,0,36.3333320617676,3,1.33333337306976,40.6666679382324,3,2,207,70,1,
349,20,2010-12-02,2,4,0,45.3333320617676,8.66666698455811,6,60,3,2,207,70,1,1,0,1,1,11,889.2

350,20,2010-12-02,3,4,0,31,22.3333339691162,6.66666650772095,60,3,2,207,70,1,1,0,1,1,11,889.2
351,20,2010-12-02,4,4,0,42.5999984741211,15.8000001907349,1.39999997615814,59.799999237
352,21,2010-12-02,0,4,0,59,0,1,60,1,2,207,67,0,1,0,1,1,12,896.823243,929.6955618,0.770390858,3
353,21,2010-12-02,1,4,0,37,23,0,60,1,2,207,67,0,1,0,1,1,12,896.823243,929.6955618,0.770390858,
354,21,2010-12-03,0,5,0,0,11,1,12,1,2,207,67,0,1,0,1,1,12,896.823243,929.6955618,0.770390858,3
355,21,2010-12-03,1,5,0,27.3999996185303,31.7999992370605,0.800000011920929,60,3,2,207,67
356,21,2010-12-03,2,5,0,29.75,25.5,4.75,60,3,2,207,67,0,1,0,1,1,12,896.823243,929.6955618,0.770
357,21,2010-12-03,3,5,0,24.3333339691162,20,15.6666669845581,60,3,2,207,67,0,1,0,1,1,12,896.
358,21,2010-12-03,4,5,0,34,24,2,60,3,2,207,67,0,1,0,1,1,12,896.823243,929.6955618,0.770390858,
359,21,2010-12-04,0,6,1,4,10,0,14,1,2,207,67,0,1,0,1,1,12,896.823243,929.6955618,0.770390858,3
360,21,2010-12-04,1,6,1,41.6666679382324,18,0.333333343267441,60,3,2,207,67,0,1,0,1,1,12,896
361,21,2010-12-04,2,6,1,39.7999992370605,20,0.200000002980232,60,3,2,207,67,0,1,0,1,1,12,896
362,21,2010-12-04,3,6,1,41.5999984741211,17.7999992370605,0.600000023841858,60,3,2,207,67
363,21,2010-12-04,4,6,1,45,15,0,60,3,2,207,67,0,1,0,1,1,12,896.823243,929.6955618,0.770390858,
364,21,2010-12-05,0,0,1,0,9,0,9,1,2,207,67,0,1,0,1,1,12,896.823243,929.6955618,0.770390858,3.8
365,21,2010-12-05,1,0,1,30.3333339691162,29.3333339691162,0.333333343267441,60,3,2,207,67
366,21,2010-12-05,2,0,1,33,27,0,60,3,2,207,67,0,1,0,1,1,12,896.823243,929.6955618,0.770390858,
367,21,2010-12-05,3,0,1,30,29.75,0.25,60,3,2,207,67,0,1,0,1,1,12,896.823243,929.6955618,0.7703
368,21,2010-12-05,4,0,1,38.7999992370605,20.7999992370605,0.400000005960464,60,3,2,207,67
369,21,2010-12-06,1,1,0,13,29,4.40000009536743,46.4000015258789,3,2,207,67,0,1,0,1,1,12,896.
370,21,2010-12-06,2,1,0,44.6666679382324,15,0.333333343267441,60,3,2,207,67,0,1,0,1,1,12,896
371,21,2010-12-06,3,1,0,45.6666679382324,14.3333330154419,0,60,3,2,207,67,0,1,0,1,1,12,896.8
372,21,2010-12-06,4,1,0,37.75,22,0.25,60,3,2,207,67,0,1,0,1,1,12,896.823243,929.6955618,0.7703
373,21,2010-12-07,1,2,0,37.3333320617676,21.3333339691162,0.333333343267441,59,3,2,207,67
374,21,2010-12-07,2,2,0,22.5,28,9.5,60,3,2,207,67,0,1,0,1,1,12,896.823243,929.6955618,0.770390
375,21,2010-12-07,3,2,0,31.5,27.75,0.75,60,3,2,207,67,0,1,0,1,1,12,896.823243,929.6955618,0.770
376,21,2010-12-07,4,2,0,25.3333339691162,32.6666679382324,2,60,3,2,207,67,0,1,0,1,1,12,896.8
377,21,2010-12-08,0,3,0,30.5,23,1,54.5,2,2,207,67,0,1,0,1,1,12,896.823243,929.6955618,0.770390
378,21,2010-12-08,1,3,0,37.3333320617676,20,2.66666674613953,60,3,2,207,67,0,1,0,1,1,12,896.
379,21,2010-12-08,2,3,0,31.3333339691162,28,0.666666686534882,60,3,2,207,67,0,1,0,1,1,12,896
380,21,2010-12-08,3,3,0,40.4000015258789,18.6000003814697,1,60,3,2,207,67,0,1,0,1,1,12,896.8
381,21,2010-12-08,4,3,0,38.3333320617676,21.1666660308838,0.5,60,3,2,207,67,0,1,0,1,1,12,896.
382,22,2010-12-01,0,3,0,29.5,5.5,1,36,2,2,207,69,0,1,0,1,1,12,888.2367729,1073.063478,0.719105
383,22,2010-12-01,1,3,0,32.5999984741211,27.3999996185303,0,60,3,2,207,69,0,1,0,1,1,12,888.2
384,22,2010-12-01,2,3,0,28.6666660308838,25.3333339691162,6,60,3,2,207,69,0,1,0,1,1,12,888.2
385,22,2010-12-01,3,3,0,24.3333339691162,20,15.6666669845581,60,3,2,207,69,0,1,0,1,1,12,888.
386,22,2010-12-01,4,3,0,34,24,2,60,3,2,207,69,0,1,0,1,1,12,888.2367729,1073.063478,0.71910541
387,22,2010-12-02,0,4,0,4,10,0,14,1,2,207,69,0,1,0,1,1,12,888.2367729,1073.063478,0.719105418,
388,22,2010-12-02,1,4,0,34,24.75,1.25,60,3,2,207,69,0,1,0,1,1,12,888.2367729,1073.063478,0.7191
389,22,2010-12-02,2,4,0,41,18.6000003814697,0.400000005960464,60,3,2,207,69,0,1,0,1,1,12,888
390,22,2010-12-02,3,4,0,46,14,0,60,3,2,207,69,0,1,0,1,1,12,888.2367729,1073.063478,0.71910541
391,22,2010-12-02,4,4,0,45,15,0,60,3,2,207,69,0,1,0,1,1,12,888.2367729,1073.063478,0.71910541
392,22,2010-12-03,0,5,0,0,9,0,9,1,2,207,69,0,1,0,1,1,12,888.2367729,1073.063478,0.719105418,4.1
393,22,2010-12-03,1,5,0,30.3333339691162,29.3333339691162,0.333333343267441,60,3,2,207,69
394,22,2010-12-03,2,5,0,31.5,28.5,0,60,3,2,207,69,0,1,0,1,1,12,888.2367729,1073.063478,0.71910
395,22,2010-12-03,3,5,0,35.5,23.75,0.75,60,3,2,207,69,0,1,0,1,1,12,888.2367729,1073.063478,0.71
396,22,2010-12-03,4,5,0,39.6666679382324,20.3333339691162,0,60,3,2,207,69,0,1,0,1,1,12,888.2
397,22,2010-12-04,1,6,1,17.6666660308838,27.3333339691162,2,47,3,2,207,69,0,1,0,1,1,12,888.2
398,22,2010-12-04,2,6,1,44.6666679382324,15,0.333333343267441,60,3,2,207,69,0,1,0,1,1,12,888
399,22,2010-12-04,3,6,1,31.6666660308838,28,0.333333343267441,60,3,2,207,69,0,1,0,1,1,12,888

400,22,2010-12-04,4,6,1,41,18.3333339691162,0.666666686534882,60,3,2,207,69,0,1,0,1,1,12,888
401,22,2010-12-05,1,0,1,6,31.5,8,45.5,2,2,207,69,0,1,0,1,1,12,888.2367729,1073.063478,0.719105
402,22,2010-12-05,2,0,1,4.66666650772095,37.6666679382324,17.6666660308838,60,3,2,207,69,0
403,22,2010-12-05,3,0,1,37.75,21.5,0.75,60,3,2,207,69,0,1,0,1,1,12,888.2367729,1073.063478,0.71
404,22,2010-12-05,4,0,1,34.3333320617676,25.3333339691162,0.333333343267441,60,3,2,207,69
405,22,2010-12-06,1,1,0,37.3333320617676,21.3333339691162,0.333333343267441,59,3,2,207,69
406,22,2010-12-06,2,1,0,40.3333320617676,18.3333339691162,1.33333337306976,60,3,2,207,69,0
407,22,2010-12-06,3,1,0,38.75,21,0.25,60,3,2,207,69,0,1,0,1,1,12,888.2367729,1073.063478,0.7191
408,22,2010-12-06,4,1,0,33.2000007629395,25.3999996185303,1.39999997615814,60,3,2,207,69,0
409,22,2010-12-07,0,2,0,30.5,23,1,54.5,2,2,207,69,0,1,0,1,1,12,888.2367729,1073.063478,0.719105
410,22,2010-12-07,1,2,0,37.3333320617676,20,2.66666674613953,60,3,2,207,69,0,1,0,1,1,12,888.2
411,22,2010-12-07,2,2,0,31.3333339691162,28,0.666666686534882,60,3,2,207,69,0,1,0,1,1,12,888
412,22,2010-12-07,3,2,0,37.3333320617676,21.3333339691162,1.33333337306976,60,3,2,207,69,0
413,22,2010-12-07,4,2,0,35,24.5,0.5,60,3,2,207,69,0,1,0,1,1,12,888.2367729,1073.063478,0.719105
414,23,2010-12-01,0,3,0,0,9,0,9,1,2,207,76,0,1,0,1,1,9,879.5047608,1090.12904,0.689521342,0,0.0
415,23,2010-12-01,1,3,0,35,24.6000003814697,0.400000005960464,60,3,2,207,76,0,1,0,1,1,9,879.5
416,23,2010-12-01,2,3,0,30.5,29.25,0.25,60,3,2,207,76,0,1,0,1,1,9,879.5047608,1090.12904,0.6895
417,23,2010-12-01,3,3,0,40.3333320617676,18.6666660308838,1,60,3,2,207,76,0,1,0,1,1,9,879.504
418,23,2010-12-01,4,3,0,39.6666679382324,20.3333339691162,0,60,3,2,207,76,0,1,0,1,1,9,879.504
419,23,2010-12-02,1,4,0,17.6666660308838,27.3333339691162,2,47,3,2,207,76,0,1,0,1,1,9,879.504
420,23,2010-12-02,2,4,0,40.2000007629395,19.6000003814697,0.200000002980232,60,3,2,207,76
421,23,2010-12-02,3,4,0,30.6000003814697,29.2000007629395,0.200000002980232,60,3,2,207,76
422,23,2010-12-02,4,4,0,41,18.3333339691162,0.666666686534882,60,3,2,207,76,0,1,0,1,1,9,879.5
423,23,2010-12-03,1,5,0,17.3333339691162,26.3333339691162,5.66666650772095,49.333332061
424,23,2010-12-03,2,5,0,4.66666650772095,37.6666679382324,17.6666660308838,60,3,2,207,76,0
425,23,2010-12-03,3,5,0,35.75,23.5,0.75,60,3,2,207,76,0,1,0,1,1,9,879.5047608,1090.12904,0.6895
426,23,2010-12-03,4,5,0,29.8333339691162,29,1.16666662693024,60,3,2,207,76,0,1,0,1,1,9,879.50
427,23,2010-12-04,0,6,1,30.5,23,1,54.5,2,2,207,76,0,1,0,1,1,9,879.5047608,1090.12904,0.6895213
428,23,2010-12-04,1,6,1,36.7999992370605,21.6000003814697,1.60000002384186,60,3,2,207,76,0
429,23,2010-12-04,2,6,1,36.5,22.25,1.25,60,3,2,207,76,0,1,0,1,1,9,879.5047608,1090.12904,0.6895
430,23,2010-12-04,3,6,1,42,17.6666660308838,0.333333343267441,60,3,2,207,76,0,1,0,1,1,9,879.5
431,23,2010-12-04,4,6,1,43.6666679382324,16,0.333333343267441,60,3,2,207,76,0,1,0,1,1,9,879.5
432,23,2010-12-05,2,0,1,34.5,25,0.5,60,2,2,207,76,0,1,0,1,1,9,879.5047608,1090.12904,0.6895213
433,23,2010-12-05,3,0,1,37.3333320617676,21.3333339691162,1.33333337306976,60,3,2,207,76,0
434,23,2010-12-05,4,0,1,33,26.3333339691162,0.666666686534882,60,3,2,207,76,0,1,0,1,1,9,879.5
435,23,2010-12-07,0,2,0,59,0,1,60,1,2,207,76,0,1,0,1,1,9,879.5047608,1090.12904,0.689521342,0,0
436,23,2010-12-08,0,3,0,0,11,1,12,1,2,207,76,0,1,0,1,1,9,879.5047608,1090.12904,0.689521342,0,0
437,23,2010-12-08,1,3,0,32.5999984741211,27.3999996185303,0,60,3,2,207,76,0,1,0,1,1,9,879.504
438,23,2010-12-08,2,3,0,28.6666660308838,25.3333339691162,6,60,3,2,207,76,0,1,0,1,1,9,879.504
439,23,2010-12-08,3,3,0,24.3333339691162,20,15.6666669845581,60,3,2,207,76,0,1,0,1,1,9,879.50
440,23,2010-12-08,4,3,0,34,24,2,60,3,2,207,76,0,1,0,1,1,9,879.5047608,1090.12904,0.689521342,0
441,23,2010-12-09,0,4,0,4,10,0,14,1,2,207,76,0,1,0,1,1,9,879.5047608,1090.12904,0.689521342,0,0
442,23,2010-12-09,1,4,0,26,32,2,60,2,2,207,76,0,1,0,1,1,9,879.5047608,1090.12904,0.689521342,0
443,23,2010-12-09,2,4,0,47.3333320617676,12.3333330154419,0.333333343267441,60,3,2,207,76
444,23,2010-12-09,3,4,0,46,14,0,60,3,2,207,76,0,1,0,1,1,9,879.5047608,1090.12904,0.689521342,0
445,23,2010-12-09,4,4,0,45,15,0,60,3,2,207,76,0,1,0,1,1,9,879.5047608,1090.12904,0.689521342,0
446,28,2010-12-01,1,3,0,6,31.5,8,45.5,2,2,207,69,1,0,0,1,1,9,875.0711037,1066.556794,0.7153256
447,28,2010-12-01,2,3,0,4.66666650772095,37.6666679382324,17.6666660308838,60,3,2,207,69,1
448,28,2010-12-01,3,3,0,37.8333320617676,21.6666660308838,0.5,60,3,2,207,69,1,0,0,1,1,9,875.0
449,28,2010-12-01,4,3,0,31.2000007629395,27.6000003814697,1.20000004768372,60,3,2,207,69,1

450,28,2010-12-02,0,4,0,30.5,23,1,54.5,2,2,207,69,1,0,0,1,1,9,875.0711037,1066.556794,0.715325

451,28,2010-12-02,1,4,0,39,20,0.25,59.25,3,2,207,69,1,0,0,1,1,9,875.0711037,1066.556794,0.7153

452,28,2010-12-02,2,4,0,40.3333320617676,18.3333339691162,1.33333337306976,60,3,2,207,69,

453,28,2010-12-02,3,4,0,42,17.6666660308838,0.333333343267441,60,3,2,207,69,1,0,0,1,1,9,875.

454,28,2010-12-02,4,4,0,38.5,21,0.5,60,3,2,207,69,1,0,0,1,1,9,875.0711037,1066.556794,0.715325

455,28,2010-12-03,1,5,0,34,22,4,60,2,2,207,69,1,0,0,1,1,9,875.0711037,1066.556794,0.715325688,

456,28,2010-12-03,2,5,0,31.3333339691162,28,0.666666686534882,60,3,2,207,69,1,0,0,1,1,9,875.

457,28,2010-12-03,3,5,0,37.3333320617676,21.3333339691162,1.33333337306976,60,3,2,207,69,

458,28,2010-12-03,4,5,0,33,26.3333339691162,0.666666686534882,60,3,2,207,69,1,0,0,1,1,9,875.

459,28,2010-12-05,0,0,1,59,0,1,60,1,2,207,69,1,0,0,1,1,9,875.0711037,1066.556794,0.715325688,4

460,28,2010-12-05,1,0,1,38.6666679382324,21.3333339691162,0,60,3,2,207,69,1,0,0,1,1,9,875.07

461,28,2010-12-05,2,0,1,28.6666660308838,25.3333339691162,6,60,3,2,207,69,1,0,0,1,1,9,875.07

462,28,2010-12-05,3,0,1,1,13,46,60,1,2,207,69,1,0,0,1,1,9,875.0711037,1066.556794,0.715325688,

463,28,2010-12-06,0,1,0,0,11,1,12,1,2,207,69,1,0,0,1,1,9,875.0711037,1066.556794,0.715325688,4

464,28,2010-12-06,1,1,0,19.3333339691162,39.3333320617676,1.33333337306976,60,3,2,207,69,

465,28,2010-12-06,2,1,0,47.3333320617676,12.3333330154419,0.333333343267441,60,3,2,207,69

466,28,2010-12-06,3,1,0,42,17.7999992370605,0.200000002980232,60,3,2,207,69,1,0,0,1,1,9,875.

467,28,2010-12-06,4,1,0,34,24,2,60,3,2,207,69,1,0,0,1,1,9,875.0711037,1066.556794,0.715325688,

468,28,2010-12-07,0,2,0,4,10,0,14,1,2,207,69,1,0,0,1,1,9,875.0711037,1066.556794,0.715325688,4

469,28,2010-12-07,1,2,0,41.6666679382324,18,0.333333343267441,60,3,2,207,69,1,0,0,1,1,9,875.

470,28,2010-12-07,2,2,0,30,29.6666660308838,0.333333343267441,60,3,2,207,69,1,0,0,1,1,9,875.

471,28,2010-12-07,3,2,0,40.3333320617676,18.6666660308838,1,60,3,2,207,69,1,0,0,1,1,9,875.07

472,28,2010-12-07,4,2,0,42.3333320617676,17.6666660308838,0,60,3,2,207,69,1,0,0,1,1,9,875.07

473,28,2010-12-08,0,3,0,0,9,0,9,1,2,207,69,1,0,0,1,1,9,875.0711037,1066.556794,0.715325688,4.1

474,28,2010-12-08,1,3,0,24,28.3333339691162,1.16666662693024,53.5,3,2,207,69,1,0,0,1,1,9,875.

475,28,2010-12-08,2,3,0,38.8333320617676,21,0.16666667163372,60,3,2,207,69,1,0,0,1,1,9,875.0

476,28,2010-12-08,3,3,0,23,36.6666679382324,0.333333343267441,60,3,2,207,69,1,0,0,1,1,9,875.

477,28,2010-12-08,4,3,0,41,18.3333339691162,0.666666686534882,60,3,2,207,69,1,0,0,1,1,9,875.

478,32,2010-12-01,0,3,0,37,23,0,60,1,2,207,70,1,1,1,1,1,10,892.8488221,1129.61513,0.77418675,5

479,32,2010-12-01,1,3,0,37.3333320617676,20,2.66666674613953,60,3,2,207,70,1,1,1,1,1,10,892.

480,32,2010-12-01,2,3,0,31.3333339691162,28,0.666666686534882,60,3,2,207,70,1,1,1,1,1,10,892

481,32,2010-12-01,3,3,0,37.3333320617676,21.3333339691162,1.33333337306976,60,3,2,207,70,

482,32,2010-12-01,4,3,0,33,26.3333339691162,0.666666686534882,60,3,2,207,70,1,1,1,1,1,10,892

483,32,2010-12-03,0,5,0,59,0,1,60,1,2,207,70,1,1,1,1,1,10,892.8488221,1129.61513,0.77418675,5.

484,32,2010-12-03,1,5,0,38.6666679382324,21.3333339691162,0,60,3,2,207,70,1,1,1,1,1,10,892.8

485,32,2010-12-03,2,5,0,27,27,6,60,1,2,207,70,1,1,1,1,1,10,892.8488221,1129.61513,0.77418675,5

486,32,2010-12-04,0,6,1,0,11,1,12,1,2,207,70,1,1,1,1,1,10,892.8488221,1129.61513,0.77418675,5.

487,32,2010-12-04,1,6,1,19.3333339691162,39.3333320617676,1.33333337306976,60,3,2,207,70,

488,32,2010-12-04,2,6,1,40.2000007629395,17.2000007629395,2.59999990463257,60,3,2,207,70,

489,32,2010-12-04,3,6,1,32.5999984741211,18,9.39999961853027,60,3,2,207,70,1,1,1,1,1,10,892.

490,32,2010-12-04,4,6,1,34,24,2,60,3,2,207,70,1,1,1,1,1,10,892.8488221,1129.61513,0.77418675,5

491,32,2010-12-05,0,0,1,4,10,0,14,1,2,207,70,1,1,1,1,1,10,892.8488221,1129.61513,0.77418675,5.

492,32,2010-12-05,1,0,1,41.6666679382324,18,0.333333343267441,60,3,2,207,70,1,1,1,1,1,10,892

493,32,2010-12-05,2,0,1,30,29.6666660308838,0.333333343267441,60,3,2,207,70,1,1,1,1,1,10,892

494,32,2010-12-05,3,0,1,42.25,17,0.75,60,3,2,207,70,1,1,1,1,1,10,892.8488221,1129.61513,0.7741

495,32,2010-12-05,4,0,1,40.7999992370605,19.2000007629395,0,60,3,2,207,70,1,1,1,1,1,10,892.8

496,32,2010-12-06,0,1,0,0,9,0,9,1,2,207,70,1,1,1,1,1,10,892.8488221,1129.61513,0.77418675,5.15

497,32,2010-12-06,1,1,0,23.5,25.5,1.25,50.25,3,2,207,70,1,1,1,1,1,10,892.8488221,1129.61513,0.7

498,32,2010-12-06,2,1,0,33,27,0,60,3,2,207,70,1,1,1,1,1,10,892.8488221,1129.61513,0.77418675,5

499,32,2010-12-06,3,1,0,23,36.6666679382324,0.333333343267441,60,3,2,207,70,1,1,1,1,1,10,892

500,32,2010-12-06,4,1,0,43.25,16.25,0.5,60,3,2,207,70,1,1,1,1,1,10,892.8488221,1129.61513,0.774
501,32,2010-12-07,1,2,0,15.5,32.75,4.5,52.75,3,2,207,70,1,1,1,1,1,10,892.8488221,1129.61513,0.77
502,32,2010-12-07,2,2,0,24.6666660308838,26.3333339691162,9,60,3,2,207,70,1,1,1,1,1,10,892.84
503,32,2010-12-07,3,2,0,45.6666679382324,14.3333330154419,0,60,3,2,207,70,1,1,1,1,1,10,892.84
504,32,2010-12-07,4,2,0,34.3333320617676,25.3333339691162,0.333333343267441,60,3,2,207,70
505,32,2010-12-08,0,3,0,24,23,2,49,1,2,207,70,1,1,1,1,1,10,892.8488221,1129.61513,0.77418675,5
506,32,2010-12-08,1,3,0,37.3333320617676,21.3333339691162,0.333333343267441,59,3,2,207,70
507,32,2010-12-08,2,3,0,40.3333320617676,18.3333339691162,1.33333337306976,60,3,2,207,70,
508,32,2010-12-08,3,3,0,36,23.3333339691162,0.666666686534882,60,3,2,207,70,1,1,1,1,1,10,892
509,32,2010-12-08,4,3,0,34.5,24.3333339691162,1.16666662693024,60,3,2,207,70,1,1,1,1,1,10,892
510,33,2010-12-01,0,3,0,59,0,1,60,1,2,207,71,0,1,0,1,1,12,875.0284267,1084.406933,0.568658348,
511,33,2010-12-01,1,3,0,41.5,18.5,0,60,2,2,207,71,0,1,0,1,1,12,875.0284267,1084.406933,0.56865
512,33,2010-12-02,0,4,0,0,11,1,12,1,2,207,71,0,1,0,1,1,12,875.0284267,1084.406933,0.568658348,
513,33,2010-12-02,1,4,0,22.75,36.25,1,60,3,2,207,71,0,1,0,1,1,12,875.0284267,1084.406933,0.5686
514,33,2010-12-02,2,4,0,34,22.2000007629395,3.79999995231628,60,3,2,207,71,0,1,0,1,1,12,875.
515,33,2010-12-02,3,4,0,24.3333339691162,20,15.6666669845581,60,3,2,207,71,0,1,0,1,1,12,875.
516,33,2010-12-02,4,4,0,34,24,2,60,3,2,207,71,0,1,0,1,1,12,875.0284267,1084.406933,0.56865834
517,33,2010-12-03,0,5,0,4,10,0,14,1,2,207,71,0,1,0,1,1,12,875.0284267,1084.406933,0.568658348,
518,33,2010-12-03,1,5,0,41.6666679382324,18,0.333333343267441,60,3,2,207,71,0,1,0,1,1,12,875
519,33,2010-12-03,2,5,0,37,22.75,0.25,60,3,2,207,71,0,1,0,1,1,12,875.0284267,1084.406933,0.5686
520,33,2010-12-03,3,5,0,41.5999984741211,17.7999992370605,0.600000023841858,60,3,2,207,71
521,33,2010-12-03,4,5,0,45,15,0,60,3,2,207,71,0,1,0,1,1,12,875.0284267,1084.406933,0.56865834
522,33,2010-12-04,0,6,1,0,9,0,9,1,2,207,71,0,1,0,1,1,12,875.0284267,1084.406933,0.568658348,0,
523,33,2010-12-04,1,6,1,30.3333339691162,29.3333339691162,0.333333343267441,60,3,2,207,71
524,33,2010-12-04,2,6,1,33,27,0,60,3,2,207,71,0,1,0,1,1,12,875.0284267,1084.406933,0.56865834
525,33,2010-12-04,3,6,1,30,29.75,0.25,60,3,2,207,71,0,1,0,1,1,12,875.0284267,1084.406933,0.5686
526,33,2010-12-04,4,6,1,40.3333320617676,19.3333339691162,0.333333343267441,60,3,2,207,71
527,33,2010-12-05,1,0,1,13,29,4.40000009536743,46.4000015258789,3,2,207,71,0,1,0,1,1,12,875.
528,33,2010-12-05,2,0,1,27,25.3999996185303,7.59999990463257,60,3,2,207,71,0,1,0,1,1,12,875.
529,33,2010-12-05,3,0,1,45.6666679382324,14.3333330154419,0,60,3,2,207,71,0,1,0,1,1,12,875.0
530,33,2010-12-05,4,0,1,34.3333320617676,25.3333339691162,0.333333343267441,60,3,2,207,71
531,33,2010-12-06,1,1,0,37.3333320617676,21.3333339691162,0.333333343267441,59,3,2,207,71
532,33,2010-12-06,2,1,0,33.5,21.5,5,60,3,2,207,71,0,1,0,1,1,12,875.0284267,1084.406933,0.56865
533,33,2010-12-06,3,1,0,33.5999984741211,25.7999992370605,0.600000023841858,60,3,2,207,71
534,33,2010-12-06,4,1,0,25.3333339691162,32.6666679382324,2,60,3,2,207,71,0,1,0,1,1,12,875.0
535,33,2010-12-07,0,2,0,30.5,23,1,54.5,2,2,207,71,0,1,0,1,1,12,875.0284267,1084.406933,0.56865
536,33,2010-12-07,1,2,0,37.3333320617676,20,2.66666674613953,60,3,2,207,71,0,1,0,1,1,12,875.
537,33,2010-12-07,2,2,0,31.3333339691162,28,0.666666686534882,60,3,2,207,71,0,1,0,1,1,12,875
538,33,2010-12-07,3,2,0,40,18.75,1.25,60,3,2,207,71,0,1,0,1,1,12,875.0284267,1084.406933,0.5686
539,33,2010-12-07,4,2,0,41,18.5,0.5,60,3,2,207,71,0,1,0,1,1,12,875.0284267,1084.406933,0.56865
540,33,2010-12-08,4,3,0,33,26.5,0.5,60,2,2,207,71,0,1,0,1,1,12,875.0284267,1084.406933,0.56865
541,37,2010-12-01,0,3,0,4,10,0,14,1,2,207,78,0,0,0,1,1,10,892.7902768,1152.711043,0.735740717,
542,37,2010-12-01,1,3,0,41.6666679382324,18,0.333333343267441,60,3,2,207,78,0,0,0,1,1,10,892
543,37,2010-12-01,2,3,0,38.6666679382324,21,0.333333343267441,60,3,2,207,78,0,0,0,1,1,10,892
544,37,2010-12-01,3,3,0,46,14,0,60,3,2,207,78,0,0,0,1,1,10,892.7902768,1152.711043,0.73574071
545,37,2010-12-01,4,3,0,45,15,0,60,3,2,207,78,0,0,0,1,1,10,892.7902768,1152.711043,0.73574071
546,37,2010-12-02,0,4,0,0,9,0,9,1,2,207,78,0,0,0,1,1,10,892.7902768,1152.711043,0.735740717,5.
547,37,2010-12-02,1,4,0,30.3333339691162,29.3333339691162,0.333333343267441,60,3,2,207,78
548,37,2010-12-02,2,4,0,33,27,0,60,3,2,207,78,0,0,0,1,1,10,892.7902768,1152.711043,0.73574071
549,37,2010-12-02,3,4,0,31.6666660308838,27.6666660308838,0.666666686534882,60,3,2,207,78

550,37,2010-12-02,4,4,0,39.6666679382324,20.3333339691162,0,60,3,2,207,78,0,0,0,1,1,10,892.7
551,37,2010-12-03,1,5,0,14.5,26.5,2,43,3,2,207,78,0,0,0,1,1,10,892.7902768,1152.711043,0.73574
552,37,2010-12-03,2,5,0,44.6666679382324,15,0.333333343267441,60,3,2,207,78,0,0,0,1,1,10,892
553,37,2010-12-03,3,5,0,45.6666679382324,14.3333330154419,0,60,3,2,207,78,0,0,0,1,1,10,892.7
554,37,2010-12-03,4,5,0,37.6666679382324,21.8333339691162,0.5,60,3,2,207,78,0,0,0,1,1,10,892.
555,37,2010-12-04,1,6,1,29.75,25.75,3.75,59.25,3,2,207,78,0,0,0,1,1,10,892.7902768,1152.711043,
556,37,2010-12-04,2,6,1,22.5,28,9.5,60,3,2,207,78,0,0,0,1,1,10,892.7902768,1152.711043,0.73574
557,37,2010-12-04,3,6,1,31.5,27.75,0.75,60,3,2,207,78,0,0,0,1,1,10,892.7902768,1152.711043,0.73
558,37,2010-12-04,4,6,1,25.3333339691162,32.6666679382324,2,60,3,2,207,78,0,0,0,1,1,10,892.7
559,37,2010-12-05,0,0,1,30.5,23,1,54.5,2,2,207,78,0,0,0,1,1,10,892.7902768,1152.711043,0.73574
560,37,2010-12-05,1,0,1,37.3333320617676,20,2.66666674613953,60,3,2,207,78,0,0,0,1,1,10,892.
561,37,2010-12-05,2,0,1,31.3333339691162,28,0.666666686534882,60,3,2,207,78,0,0,0,1,1,10,892
562,37,2010-12-05,3,0,1,40.4000015258789,18.6000003814697,1,60,3,2,207,78,0,0,0,1,1,10,892.7
563,37,2010-12-05,4,0,1,43.6666679382324,16,0.333333343267441,60,3,2,207,78,0,0,0,1,1,10,892
564,37,2010-12-06,4,1,0,33,26.3333339691162,0.666666686534882,60,3,2,207,78,0,0,0,1,1,10,892
565,37,2010-12-08,0,3,0,29.5,5.5,1,36,2,2,207,78,0,0,0,1,1,10,892.7902768,1152.711043,0.735740
566,37,2010-12-08,1,3,0,29,30.3333339691162,0.666666686534882,60,3,2,207,78,0,0,0,1,1,10,892
567,37,2010-12-08,2,3,0,28.6666660308838,25.3333339691162,6,60,3,2,207,78,0,0,0,1,1,10,892.7
568,37,2010-12-08,3,3,0,24.3333339691162,20,15.6666669845581,60,3,2,207,78,0,0,0,1,1,10,892.
569,37,2010-12-08,4,3,0,34,24,2,60,3,2,207,78,0,0,0,1,1,10,892.7902768,1152.711043,0.73574071
570,38,2010-12-01,1,3,0,17.6666660308838,27.3333339691162,2,47,3,2,207,73,0,1,1,1,1,11,908.0
571,38,2010-12-01,2,3,0,44.6666679382324,15,0.333333343267441,60,3,2,207,73,0,1,1,1,1,11,908
572,38,2010-12-01,3,3,0,37,22.7999992370605,0.200000002980232,60,3,2,207,73,0,1,1,1,1,11,908
573,38,2010-12-01,4,3,0,37.4000015258789,22,0.600000023841858,60,3,2,207,73,0,1,1,1,1,11,908
574,38,2010-12-02,1,4,0,24.7999992370605,25.3999996185303,3.40000009536743,53.59999847
575,38,2010-12-02,2,4,0,17.5,29.25,13.25,60,3,2,207,73,0,1,1,1,1,11,908.0707363,599.2627876,1.0
576,38,2010-12-02,3,4,0,30,29,1,60,3,2,207,73,0,1,1,1,1,11,908.0707363,599.2627876,1.03574560
577,38,2010-12-02,4,4,0,28.75,29.75,1.5,60,3,2,207,73,0,1,1,1,1,11,908.0707363,599.2627876,1.03
578,38,2010-12-03,0,5,0,30.5,23,1,54.5,2,2,207,73,0,1,1,1,1,11,908.0707363,599.2627876,1.035745
579,38,2010-12-03,1,5,0,37.3333320617676,20,2.66666674613953,60,3,2,207,73,0,1,1,1,1,11,908.
580,38,2010-12-03,2,5,0,31.7999992370605,27,1.20000004768372,60,3,2,207,73,0,1,1,1,1,11,908.
581,38,2010-12-03,3,5,0,42,17.6666660308838,0.333333343267441,60,3,2,207,73,0,1,1,1,1,11,908
582,38,2010-12-03,4,5,0,43.6666679382324,16,0.333333343267441,60,3,2,207,73,0,1,1,1,1,11,908
583,38,2010-12-04,3,6,1,37.3333320617676,21.3333339691162,1.33333337306976,60,3,2,207,73,
584,38,2010-12-04,4,6,1,33,26.3333339691162,0.666666686534882,60,3,2,207,73,0,1,1,1,1,11,908
585,38,2010-12-06,0,1,0,59,0,1,60,1,2,207,73,0,1,1,1,1,11,908.0707363,599.2627876,1.035745602,
586,38,2010-12-06,1,1,0,38.6666679382324,21.3333339691162,0,60,3,2,207,73,0,1,1,1,1,11,908.0
587,38,2010-12-06,2,1,0,28.6666660308838,25.3333339691162,6,60,3,2,207,73,0,1,1,1,1,11,908.0
588,38,2010-12-06,3,1,0,24.3333339691162,20,15.6666669845581,60,3,2,207,73,0,1,1,1,1,11,908.
589,38,2010-12-06,4,1,0,35,24,1,60,2,2,207,73,0,1,1,1,1,11,908.0707363,599.2627876,1.03574560
590,38,2010-12-07,0,2,0,2,10.5,0.5,13,2,2,207,73,0,1,1,1,1,11,908.0707363,599.2627876,1.0357456
591,38,2010-12-07,1,2,0,24.75,34.25,1,60,3,2,207,73,0,1,1,1,1,11,908.0707363,599.2627876,1.035
592,38,2010-12-07,2,2,0,47.3333320617676,12.3333330154419,0.333333343267441,60,3,2,207,73
593,38,2010-12-07,3,2,0,46,14,0,60,3,2,207,73,0,1,1,1,1,11,908.0707363,599.2627876,1.03574560
594,38,2010-12-07,4,2,0,41.75,17.25,1,60,3,2,207,73,0,1,1,1,1,11,908.0707363,599.2627876,1.035
595,38,2010-12-08,0,3,0,0,9,0,9,1,2,207,73,0,1,1,1,1,11,908.0707363,599.2627876,1.035745602,4.
596,38,2010-12-08,1,3,0,35,24.6000003814697,0.400000005960464,60,3,2,207,73,0,1,1,1,1,11,908
597,38,2010-12-08,2,3,0,31.5,28.3333339691162,0.16666667163372,60,3,2,207,73,0,1,1,1,1,11,90
598,38,2010-12-08,3,3,0,35.5,23.75,0.75,60,3,2,207,73,0,1,1,1,1,11,908.0707363,599.2627876,1.03
599,38,2010-12-08,4,3,0,39.6666679382324,20.3333339691162,0,60,3,2,207,73,0,1,1,1,1,11,908.0

600,40,2010-12-01,0,3,0,30.5,23,1,54.5,2,2,207,71,1,0,0,1,1,9,875.0284267,1084.406933,0.5686583
601,40,2010-12-01,1,3,0,39,19,2,60,3,2,207,71,1,0,0,1,1,9,875.0284267,1084.406933,0.568658348,
602,40,2010-12-01,2,3,0,36.5,22.25,1.25,60,3,2,207,71,1,0,0,1,1,9,875.0284267,1084.406933,0.568
603,40,2010-12-01,3,3,0,42,17.6666660308838,0.333333343267441,60,3,2,207,71,1,0,0,1,1,9,875.0
604,40,2010-12-01,4,3,0,43.6666679382324,16,0.333333343267441,60,3,2,207,71,1,0,0,1,1,9,875.0
605,40,2010-12-02,2,4,0,34.5,25,0.5,60,2,2,207,71,1,0,0,1,1,9,875.0284267,1084.406933,0.5686583
606,40,2010-12-02,3,4,0,37.3333320617676,21.3333339691162,1.33333337306976,60,3,2,207,71,1
607,40,2010-12-02,4,4,0,33,26.3333339691162,0.666666686534882,60,3,2,207,71,1,0,0,1,1,9,875.0
608,40,2010-12-04,0,6,1,59,0,1,60,1,2,207,71,1,0,0,1,1,9,875.0284267,1084.406933,0.568658348,0
609,40,2010-12-04,1,6,1,38.6666679382324,21.3333339691162,0,60,3,2,207,71,1,0,0,1,1,9,875.028
610,40,2010-12-04,2,6,1,28.6666660308838,25.3333339691162,6,60,3,2,207,71,1,0,0,1,1,9,875.028
611,40,2010-12-04,3,6,1,24.3333339691162,20,15.6666669845581,60,3,2,207,71,1,0,0,1,1,9,875.02
612,40,2010-12-04,4,6,1,34,26,0,60,1,2,207,71,1,0,0,1,1,9,875.0284267,1084.406933,0.568658348,
613,40,2010-12-05,0,0,1,2,10.5,0.5,13,2,2,207,71,1,0,0,1,1,9,875.0284267,1084.406933,0.5686583
614,40,2010-12-05,1,0,1,19.3333339691162,39.3333320617676,1.33333337306976,60,3,2,207,71,1
615,40,2010-12-05,2,0,1,47.3333320617676,12.3333330154419,0.333333343267441,60,3,2,207,71
616,40,2010-12-05,3,0,1,46,14,0,60,3,2,207,71,1,0,0,1,1,9,875.0284267,1084.406933,0.568658348,
617,40,2010-12-05,4,0,1,40.5999984741211,18.2000007629395,1.20000004768372,60,3,2,207,71,1
618,40,2010-12-06,0,1,0,0,9,0,9,1,2,207,71,1,0,0,1,1,9,875.0284267,1084.406933,0.568658348,0,0.
619,40,2010-12-06,1,1,0,37,22.6000003814697,0.400000005960464,60,3,2,207,71,1,0,0,1,1,9,875.0
620,40,2010-12-06,2,1,0,30,29.6666660308838,0.333333343267441,60,3,2,207,71,1,0,0,1,1,9,875.0
621,40,2010-12-06,3,1,0,40.3333320617676,18.6666660308838,1,60,3,2,207,71,1,0,0,1,1,9,875.028
622,40,2010-12-06,4,1,0,39.6666679382324,20.3333339691162,0,60,3,2,207,71,1,0,0,1,1,9,875.028
623,40,2010-12-07,1,2,0,21,27.75,1.5,50.25,3,2,207,71,1,0,0,1,1,9,875.0284267,1084.406933,0.568
624,40,2010-12-07,2,2,0,38.8333320617676,21,0.16666667163372,60,3,2,207,71,1,0,0,1,1,9,875.02
625,40,2010-12-07,3,2,0,23,36.6666679382324,0.333333343267441,60,3,2,207,71,1,0,0,1,1,9,875.0
626,40,2010-12-07,4,2,0,41,18.3333339691162,0.666666686534882,60,3,2,207,71,1,0,0,1,1,9,875.0
627,40,2010-12-08,1,3,0,20,27.75,4.25,52,3,2,207,71,1,0,0,1,1,9,875.0284267,1084.406933,0.56865
628,40,2010-12-08,2,3,0,4.66666650772095,37.6666679382324,17.6666660308838,60,3,2,207,71,1
629,40,2010-12-08,3,3,0,37.8333320617676,21.6666660308838,0.5,60,3,2,207,71,1,0,0,1,1,9,875.0
630,40,2010-12-08,4,3,0,29.8333339691162,29,1.16666662693024,60,3,2,207,71,1,0,0,1,1,9,875.02
631,43,2010-12-02,0,4,0,59,0,1,60,1,2,207,73,1,1,0,1,1,9,892.7902768,1152.711043,0.735740717,5
632,43,2010-12-02,1,4,0,38.6666679382324,21.3333339691162,0,60,3,2,207,73,1,1,0,1,1,9,892.790
633,43,2010-12-02,2,4,0,28.6666660308838,25.3333339691162,6,60,3,2,207,73,1,1,0,1,1,9,892.790
634,43,2010-12-02,3,4,0,1,13,46,60,1,2,207,73,1,1,0,1,1,9,892.7902768,1152.711043,0.735740717,
635,43,2010-12-03,0,5,0,0,11,1,12,1,2,207,73,1,1,0,1,1,9,892.7902768,1152.711043,0.735740717,5
636,43,2010-12-03,1,5,0,19.3333339691162,39.3333320617676,1.33333337306976,60,3,2,207,73,1
637,43,2010-12-03,2,5,0,47.3333320617676,12.3333330154419,0.333333343267441,60,3,2,207,73
638,43,2010-12-03,3,5,0,42,17.7999992370605,0.200000002980232,60,3,2,207,73,1,1,0,1,1,9,892.7
639,43,2010-12-03,4,5,0,36,22.5,1.5,60,3,2,207,73,1,1,0,1,1,9,892.7902768,1152.711043,0.7357407
640,43,2010-12-04,0,6,1,2,9.5,0,11.5,2,2,207,73,1,1,0,1,1,9,892.7902768,1152.711043,0.735740717
641,43,2010-12-04,1,6,1,39,20.5,0.5,60,3,2,207,73,1,1,0,1,1,9,892.7902768,1152.711043,0.7357407
642,43,2010-12-04,2,6,1,30,29.6666660308838,0.333333343267441,60,3,2,207,73,1,1,0,1,1,9,892.7
643,43,2010-12-04,3,6,1,40.3333320617676,18.6666660308838,1,60,3,2,207,73,1,1,0,1,1,9,892.790
644,43,2010-12-04,4,6,1,42.4000015258789,17.6000003814697,0,60,3,2,207,73,1,1,0,1,1,9,892.790
645,43,2010-12-05,1,0,1,22.6000003814697,28.3999996185303,1.20000004768372,52.200000762
646,43,2010-12-05,2,0,1,38,22,0,60,3,2,207,73,1,1,0,1,1,9,892.7902768,1152.711043,0.735740717,
647,43,2010-12-05,3,0,1,23,36.6666679382324,0.333333343267441,60,3,2,207,73,1,1,0,1,1,9,892.7
648,43,2010-12-05,4,0,1,41,18.3333339691162,0.666666686534882,60,3,2,207,73,1,1,0,1,1,9,892.7
649,43,2010-12-06,1,1,0,6,31.5,8,45.5,2,2,207,73,1,1,0,1,1,9,892.7902768,1152.711043,0.7357407

650,43,2010-12-06,2,1,0,14.25,32.25,13.5,60,3,2,207,73,1,1,0,1,1,9,892.7902768,1152.711043,0.73
651,43,2010-12-06,3,1,0,37.8333320617676,21.6666660308838,0.5,60,3,2,207,73,1,1,0,1,1,9,892.7
652,43,2010-12-06,4,1,0,34.3333320617676,25.3333339691162,0.333333343267441,60,3,2,207,73
653,43,2010-12-07,0,2,0,24,23,2,49,1,2,207,73,1,1,0,1,1,9,892.7902768,1152.711043,0.735740717,
654,43,2010-12-07,1,2,0,37.3333320617676,21.3333339691162,0.333333343267441,59,3,2,207,73
655,43,2010-12-07,2,2,0,40.3333320617676,18.3333339691162,1.33333337306976,60,3,2,207,73,
656,43,2010-12-07,3,2,0,42,17.6666660308838,0.333333343267441,60,3,2,207,73,1,1,0,1,1,9,892.
657,43,2010-12-07,4,2,0,34.5,24.3333339691162,1.16666662693024,60,3,2,207,73,1,1,0,1,1,9,892.
658,43,2010-12-08,0,3,0,37,23,0,60,1,2,207,73,1,1,0,1,1,9,892.7902768,1152.711043,0.735740717,
659,43,2010-12-08,1,3,0,37.3333320617676,20,2.66666674613953,60,3,2,207,73,1,1,0,1,1,9,892.7
660,43,2010-12-08,2,3,0,31.3333339691162,28,0.666666686534882,60,3,2,207,73,1,1,0,1,1,9,892.
661,43,2010-12-08,3,3,0,37.3333320617676,21.3333339691162,1.33333337306976,60,3,2,207,73,
662,43,2010-12-08,4,3,0,33,26.3333339691162,0.666666686534882,60,3,2,207,73,1,1,0,1,1,9,892.
663,45,2010-12-01,0,3,0,0,11,1,12,1,2,207,68,0,1,0,1,1,10,891.2532305,915.9845891,0.861056559,
664,45,2010-12-01,1,3,0,19.3333339691162,39.3333320617676,1.33333337306976,60,3,2,207,68,
665,45,2010-12-01,2,3,0,44.5,12.25,3.25,60,3,2,207,68,0,1,0,1,1,10,891.2532305,915.9845891,0.86
666,45,2010-12-01,3,3,0,32.5999984741211,18,9.39999961853027,60,3,2,207,68,0,1,0,1,1,10,891.
667,45,2010-12-01,4,3,0,34,24,2,60,3,2,207,68,0,1,0,1,1,10,891.2532305,915.9845891,0.86105655
668,45,2010-12-02,0,4,0,4,10,0,14,1,2,207,68,0,1,0,1,1,10,891.2532305,915.9845891,0.861056559,
669,45,2010-12-02,1,4,0,41.6666679382324,18,0.333333343267441,60,3,2,207,68,0,1,0,1,1,10,891
670,45,2010-12-02,2,4,0,30,29.6666660308838,0.333333343267441,60,3,2,207,68,0,1,0,1,1,10,891
671,45,2010-12-02,3,4,0,42.25,17,0.75,60,3,2,207,68,0,1,0,1,1,10,891.2532305,915.9845891,0.861
672,45,2010-12-02,4,4,0,40.7999992370605,19.2000007629395,0,60,3,2,207,68,0,1,0,1,1,10,891.2
673,45,2010-12-03,0,5,0,0,9,0,9,1,2,207,68,0,1,0,1,1,10,891.2532305,915.9845891,0.861056559,4.
674,45,2010-12-03,1,5,0,22.2000007629395,28.7999992370605,1.20000004768372,52.200000762
675,45,2010-12-03,2,5,0,33,27,0,60,3,2,207,68,0,1,0,1,1,10,891.2532305,915.9845891,0.86105655
676,45,2010-12-03,3,5,0,23,36.6666679382324,0.333333343267441,60,3,2,207,68,0,1,0,1,1,10,891
677,45,2010-12-03,4,5,0,43.25,16.25,0.5,60,3,2,207,68,0,1,0,1,1,10,891.2532305,915.9845891,0.86
678,45,2010-12-04,1,6,1,15,29.6666660308838,5.66666650772095,50.3333320617676,3,2,207,68,
679,45,2010-12-04,2,6,1,24.6666660308838,26.3333339691162,9,60,3,2,207,68,0,1,0,1,1,10,891.2
680,45,2010-12-04,3,6,1,39.5999984741211,19.7999992370605,0.600000023841858,60,3,2,207,68
681,45,2010-12-04,4,6,1,34.3333320617676,25.3333339691162,0.333333343267441,60,3,2,207,68
682,45,2010-12-05,1,0,1,37.3333320617676,21.3333339691162,0.333333343267441,59,3,2,207,68
683,45,2010-12-05,2,0,1,40.3333320617676,18.3333339691162,1.33333337306976,60,3,2,207,68,
684,45,2010-12-05,3,0,1,38.75,21,0.25,60,3,2,207,68,0,1,0,1,1,10,891.2532305,915.9845891,0.861
685,45,2010-12-05,4,0,1,34.5,24.3333339691162,1.16666662693024,60,3,2,207,68,0,1,0,1,1,10,891
686,45,2010-12-06,0,1,0,30.5,23,1,54.5,2,2,207,68,0,1,0,1,1,10,891.2532305,915.9845891,0.86105
687,45,2010-12-06,1,1,0,37.3333320617676,20,2.66666674613953,60,3,2,207,68,0,1,0,1,1,10,891.
688,45,2010-12-06,2,1,0,31.3333339691162,28,0.666666686534882,60,3,2,207,68,0,1,0,1,1,10,891
689,45,2010-12-06,3,1,0,37.3333320617676,21.3333339691162,1.33333337306976,60,3,2,207,68,
690,45,2010-12-06,4,1,0,33,26.3333339691162,0.666666686534882,60,3,2,207,68,0,1,0,1,1,10,891
691,45,2010-12-08,0,3,0,59,0,1,60,1,2,207,68,0,1,0,1,1,10,891.2532305,915.9845891,0.861056559,
692,45,2010-12-08,1,3,0,38.6666679382324,21.3333339691162,0,60,3,2,207,68,0,1,0,1,1,10,891.2
693,45,2010-12-08,2,3,0,25,32,3,60,2,2,207,68,0,1,0,1,1,10,891.2532305,915.9845891,0.86105655
694,59,2010-12-01,0,3,0,6,30,4,40,1,2,207,87,1,1,1,0,1,11,900.7001648,1467.00565,0.972657636,0
695,59,2010-12-01,1,3,0,6.33333349227905,53,0.666666686534882,60,3,2,207,87,1,1,1,0,1,11,900
696,59,2010-12-01,2,3,0,51.3333320617676,8.33333301544189,0.333333343267441,60,3,2,207,87
697,59,2010-12-01,3,3,0,26.3333339691162,32.3333320617676,1.33333337306976,60,3,2,207,87,
698,59,2010-12-01,4,3,0,25.8333339691162,34.1666679382324,0,60,3,2,207,87,1,1,1,0,1,11,900.7
699,59,2010-12-02,0,4,0,3,20,0,23,3,2,207,87,1,1,1,0,1,11,900.7001648,1467.00565,0.972657636,0

700,59,2010-12-02,1,4,0,22,37.25,0.75,60,3,2,207,87,1,1,1,0,1,11,900.7001648,1467.00565,0.97265
701,59,2010-12-02,2,4,0,10.3333330154419,49,0.666666686534882,60,3,2,207,87,1,1,1,0,1,11,900
702,59,2010-12-02,3,4,0,31.3333339691162,27,1.66666662693024,60,3,2,207,87,1,1,1,0,1,11,900.7
703,59,2010-12-02,4,4,0,25.6666660308838,33.3333320617676,1,60,3,2,207,87,1,1,1,0,1,11,900.7
704,59,2010-12-03,0,5,0,13.3333330154419,35.6666679382324,0,49,3,2,207,87,1,1,1,0,1,11,900.7
705,59,2010-12-03,1,5,0,16.25,43,0.75,60,3,2,207,87,1,1,1,0,1,11,900.7001648,1467.00565,0.97265
706,59,2010-12-03,2,5,0,40,20,0,60,3,2,207,87,1,1,1,0,1,11,900.7001648,1467.00565,0.972657636,
707,59,2010-12-03,3,5,0,18.6666660308838,41.3333320617676,0,60,3,2,207,87,1,1,1,0,1,11,900.7
708,59,2010-12-03,4,5,0,41,19,0,60,3,2,207,87,1,1,1,0,1,11,900.7001648,1467.00565,0.972657636,
709,59,2010-12-04,1,6,1,13,47,0,60,1,2,207,87,1,1,1,0,1,11,900.7001648,1467.00565,0.972657636,
710,59,2010-12-04,2,6,1,46.6666679382324,13.3333330154419,0,60,3,2,207,87,1,1,1,0,1,11,900.7
711,59,2010-12-04,3,6,1,25,34.6666679382324,0.333333343267441,60,3,2,207,87,1,1,1,0,1,11,900
712,59,2010-12-04,4,6,1,17.6666660308838,42.3333320617676,0,60,3,2,207,87,1,1,1,0,1,11,900.7
713,59,2010-12-06,0,1,0,2,58,0,60,1,2,207,87,1,1,1,0,1,11,900.7001648,1467.00565,0.972657636,0
714,59,2010-12-07,0,2,0,0,6,1,7,1,2,207,87,1,1,1,0,1,11,900.7001648,1467.00565,0.972657636,0,0.
715,59,2010-12-07,1,2,0,11.5,47.6666679382324,0.833333313465118,60,3,2,207,87,1,1,1,0,1,11,9
716,59,2010-12-07,2,2,0,11,49,0,60,3,2,207,87,1,1,1,0,1,11,900.7001648,1467.00565,0.972657636,
717,59,2010-12-07,3,2,0,14.6666669845581,44.3333320617676,1,60,3,2,207,87,1,1,1,0,1,11,900.7
718,59,2010-12-07,4,2,0,48.6666679382324,11,0.333333343267441,60,3,2,207,87,1,1,1,0,1,11,900
719,59,2010-12-08,0,3,0,5,45,0,50,1,2,207,87,1,1,1,0,1,11,900.7001648,1467.00565,0.972657636,0
720,59,2010-12-08,1,3,0,22.6666660308838,35,2.33333325386047,60,3,2,207,87,1,1,1,0,1,11,900.7
721,59,2010-12-08,2,3,0,24,35,1,60,3,2,207,87,1,1,1,0,1,11,900.7001648,1467.00565,0.972657636,
722,59,2010-12-08,3,3,0,13.3333330154419,45.5,1.16666662693024,60,3,2,207,87,1,1,1,0,1,11,900
723,59,2010-12-08,4,3,0,41,19,0,60,3,2,207,87,1,1,1,0,1,11,900.7001648,1467.00565,0.972657636,
724,66,2010-12-01,0,3,0,3,16,1,20,1,2,207,76,0,1,0,1,1,10,887.3043179,1138.335613,0.792558912,
725,66,2010-12-01,1,3,0,5.33333349227905,44.6666679382324,10,60,3,2,207,76,0,1,0,1,1,10,887.3
726,66,2010-12-01,2,3,0,4.66666650772095,44,11.3333330154419,60,3,2,207,76,0,1,0,1,1,10,887.3
727,66,2010-12-01,3,3,0,25,32.7999992370605,2.20000004768372,60,3,2,207,76,0,1,0,1,1,10,887.3
728,66,2010-12-01,4,3,0,36.75,20.25,3,60,3,2,207,76,0,1,0,1,1,10,887.3043179,1138.335613,0.7925
729,66,2010-12-02,4,4,0,13.5,46.5,0,60,2,2,207,76,0,1,0,1,1,10,887.3043179,1138.335613,0.79255
730,66,2010-12-04,1,6,1,12.3333330154419,26.6666660308838,1.66666662693024,40.666667938
731,66,2010-12-04,2,6,1,40,19.3333339691162,0.666666686534882,60,3,2,207,76,0,1,0,1,1,10,887
732,66,2010-12-04,3,6,1,5,54,1,60,1,2,207,76,0,1,0,1,1,10,887.3043179,1138.335613,0.792558912,
733,66,2010-12-05,0,0,1,3.5,28.5,1,33,2,2,207,76,0,1,0,1,1,10,887.3043179,1138.335613,0.7925589
734,66,2010-12-05,1,0,1,15.6666669845581,41.6666679382324,2.66666674613953,60,3,2,207,76,
735,66,2010-12-05,2,0,1,24,36,0,60,3,2,207,76,0,1,0,1,1,10,887.3043179,1138.335613,0.792558912
736,66,2010-12-05,3,0,1,27.3999996185303,31,1.60000002384186,60,3,2,207,76,0,1,0,1,1,10,887.3
737,66,2010-12-05,4,0,1,22,37.3333320617676,0.666666686534882,60,3,2,207,76,0,1,0,1,1,10,887
738,66,2010-12-06,0,1,0,3,20,0,23,1,2,207,76,0,1,0,1,1,10,887.3043179,1138.335613,0.792558912,
739,66,2010-12-06,1,1,0,13.6666669845581,41.6666679382324,4.66666650772095,60,3,2,207,76,
740,66,2010-12-06,2,1,0,7,50.3333320617676,2.66666674613953,60,3,2,207,76,0,1,0,1,1,10,887.3
741,66,2010-12-06,3,1,0,32.3333320617676,27,0.666666686534882,60,3,2,207,76,0,1,0,1,1,10,887
742,66,2010-12-06,4,1,0,30.3999996185303,29.3999996185303,0.200000002980232,60,3,2,207,76
743,66,2010-12-07,0,2,0,23.5,19,0,42.5,2,2,207,76,0,1,0,1,1,10,887.3043179,1138.335613,0.79255
744,66,2010-12-07,1,2,0,6,44.25,9.75,60,3,2,207,76,0,1,0,1,1,10,887.3043179,1138.335613,0.79255
745,66,2010-12-07,2,2,0,12.6666669845581,45,2.33333325386047,60,3,2,207,76,0,1,0,1,1,10,887.3
746,66,2010-12-07,3,2,0,23,37,0,60,3,2,207,76,0,1,0,1,1,10,887.3043179,1138.335613,0.792558912
747,66,2010-12-07,4,2,0,26.25,33.25,0.5,60,3,2,207,76,0,1,0,1,1,10,887.3043179,1138.335613,0.79
748,66,2010-12-08,0,3,0,0,23,0,23,1,2,207,76,0,1,0,1,1,10,887.3043179,1138.335613,0.792558912,
749,66,2010-12-08,1,3,0,13.6000003814697,36.7999992370605,9.60000038146973,60,3,2,207,76,

750,66,2010-12-08,2,3,0,8.33333301544189,44.1666679382324,7.5,60,3,2,207,76,0,1,0,1,1,10,887.
751,66,2010-12-08,3,3,0,8.5,47.75,3.75,60,3,2,207,76,0,1,0,1,1,10,887.3043179,1138.335613,0.792
752,66,2010-12-08,4,3,0,33.3333320617676,26.3333339691162,0.333333343267441,60,3,2,207,76
753,70,2010-12-02,1,4,0,17.7999992370605,34.7999992370605,1.20000004768372,53.799999237(
754,70,2010-12-02,2,4,0,31.3333339691162,28.3333339691162,0.333333343267441,60,3,2,207,79
755,70,2010-12-02,3,4,0,27,33,0,60,3,2,207,79,1,1,0,1,1,11,1156.426087,924.9865309,1.875889181
756,70,2010-12-02,4,4,0,26.3333339691162,33.3333320617676,0.333333343267441,60,3,2,207,79
757,70,2010-12-03,0,5,0,8.5,22,0.5,31,2,2,207,79,1,1,0,1,1,11,1156.426087,924.9865309,1.8758891
758,70,2010-12-03,1,5,0,21.5,37.25,1.25,60,3,2,207,79,1,1,0,1,1,11,1156.426087,924.9865309,1.87
759,70,2010-12-03,2,5,0,26,33.25,0.75,60,3,2,207,79,1,1,0,1,1,11,1156.426087,924.9865309,1.875
760,70,2010-12-03,3,5,0,20,38.3333320617676,1.66666662693024,60,3,2,207,79,1,1,0,1,1,11,1156
761,70,2010-12-03,4,5,0,23.6666660308838,36,0.333333343267441,60,3,2,207,79,1,1,0,1,1,11,115
762,70,2010-12-04,0,6,1,0,1,0,1,1,2,207,79,1,1,0,1,1,11,1156.426087,924.9865309,1.875889181,3.3
763,70,2010-12-04,1,6,1,14.6666669845581,26,1.66666662693024,42.3333320617676,3,2,207,79,1
764,70,2010-12-04,2,6,1,28.2000007629395,31.6000003814697,0.200000002980232,60,3,2,207,79
765,70,2010-12-04,3,6,1,42.4000015258789,17.3999996185303,0.200000002980232,60,3,2,207,79
766,70,2010-12-04,4,6,1,28,21.6666660308838,0.333333343267441,50,3,2,207,79,1,1,0,1,1,11,115
767,70,2010-12-05,0,0,1,13,40.5,4.5,58,2,2,207,79,1,1,0,1,1,11,1156.426087,924.9865309,1.87588
768,70,2010-12-05,1,0,1,12.6666669845581,45,2.33333325386047,60,3,2,207,79,1,1,0,1,1,11,1156
769,70,2010-12-05,2,0,1,25.3333339691162,34,0.666666686534882,60,3,2,207,79,1,1,0,1,1,11,115
770,70,2010-12-05,3,0,1,28,32,0,60,3,2,207,79,1,1,0,1,1,11,1156.426087,924.9865309,1.875889181
771,70,2010-12-05,4,0,1,34.25,25.75,0,60,3,2,207,79,1,1,0,1,1,11,1156.426087,924.9865309,1.875
772,70,2010-12-06,0,1,0,0,2,1,3,1,2,207,79,1,1,0,1,1,11,1156.426087,924.9865309,1.875889181,3.3
773,70,2010-12-06,1,1,0,25.3333339691162,32,0.333333343267441,57.6666679382324,3,2,207,79
774,70,2010-12-06,2,1,0,31.6666660308838,28,0.333333343267441,60,3,2,207,79,1,1,0,1,1,11,115
775,70,2010-12-06,3,1,0,16.3333339691162,41.3333320617676,2.33333325386047,60,3,2,207,79,1
776,70,2010-12-06,4,1,0,43,16.7999992370605,0.200000002980232,60,3,2,207,79,1,1,0,1,1,11,115
777,70,2010-12-07,1,2,0,16.3333339691162,42,1.66666662693024,60,3,2,207,79,1,1,0,1,1,11,1156
778,70,2010-12-07,2,2,0,33,26.3333339691162,0.666666686534882,60,3,2,207,79,1,1,0,1,1,11,115
779,70,2010-12-07,3,2,0,16.3333339691162,41.6666679382324,2,60,3,2,207,79,1,1,0,1,1,11,1156.4
780,70,2010-12-07,4,2,0,29.3333339691162,29.6666660308838,1,60,3,2,207,79,1,1,0,1,1,11,1156.4
781,74,2010-12-01,1,3,0,7.59999990463257,38.2000007629395,4.40000009536743,50.200000762
782,74,2010-12-01,2,3,0,29.6666660308838,27.3333339691162,3,60,3,2,207,81,0,1,1,1,1,9,1188.07
783,74,2010-12-01,3,3,0,26,33.3333320617676,0.666666686534882,60,3,2,207,81,0,1,1,1,1,9,1188
784,74,2010-12-01,4,3,0,44.7999992370605,15,0.200000002980232,60,3,2,207,81,0,1,1,1,1,9,1188
785,74,2010-12-02,0,4,0,60,0,0,60,1,2,207,81,0,1,1,1,1,9,1188.075336,883.5809344,2.886997997,3
786,74,2010-12-02,1,4,0,19.6666660308838,39,1.33333337306976,60,3,2,207,81,0,1,1,1,1,9,1188.(
787,74,2010-12-02,2,4,0,32.6666679382324,26.5,0.833333313465118,60,3,2,207,81,0,1,1,1,1,9,118
788,74,2010-12-02,3,4,0,49,10,1,60,3,2,207,81,0,1,1,1,1,9,1188.075336,883.5809344,2.886997997,
789,74,2010-12-02,4,4,0,39,20,1,60,3,2,207,81,0,1,1,1,1,9,1188.075336,883.5809344,2.886997997,
790,74,2010-12-03,0,5,0,5,19,1,25,1,2,207,81,0,1,1,1,1,9,1188.075336,883.5809344,2.886997997,3
791,74,2010-12-03,1,5,0,15.3333330154419,42,2.66666674613953,60,3,2,207,81,0,1,1,1,1,9,1188.(
792,74,2010-12-03,2,5,0,30.6666660308838,28.6666660308838,0.666666686534882,60,3,2,207,81
793,74,2010-12-03,3,5,0,33.1666679382324,26.5,0.333333343267441,60,3,2,207,81,0,1,1,1,1,9,118
794,74,2010-12-03,4,5,0,45,15,0,60,3,2,207,81,0,1,1,1,1,9,1188.075336,883.5809344,2.886997997,
795,74,2010-12-04,0,6,1,3,38,5,46,1,2,207,81,0,1,1,1,1,9,1188.075336,883.5809344,2.886997997,3
796,74,2010-12-04,1,6,1,18,28.25,3.25,49.5,3,2,207,81,0,1,1,1,1,9,1188.075336,883.5809344,2.886
797,74,2010-12-04,2,6,1,13.6666669845581,44,2.33333325386047,60,3,2,207,81,0,1,1,1,1,9,1188.(
798,74,2010-12-04,3,6,1,31.3333339691162,28.3333339691162,0.333333343267441,60,3,2,207,81
799,74,2010-12-04,4,6,1,31.3333339691162,26.1666660308838,2.5,60,3,2,207,81,0,1,1,1,1,9,1188.

800,74,2010-12-05,1,0,1,4.5,51.5,4,60,2,2,207,81,0,1,1,1,1,9,1188.075336,883.5809344,2.8869979
801,74,2010-12-05,2,0,1,21.6666660308838,37.6666679382324,0.666666686534882,60,3,2,207,81
802,74,2010-12-05,3,0,1,32,27.6666660308838,0.333333343267441,60,3,2,207,81,0,1,1,1,1,9,1188
803,74,2010-12-05,4,0,1,23.6666660308838,36.3333320617676,0,60,3,2,207,81,0,1,1,1,1,9,1188.0
804,74,2010-12-07,1,2,0,41.3333320617676,5.66666650772095,4.33333349227905,51.333332061
805,74,2010-12-07,2,2,0,59,1,0,60,1,2,207,81,0,1,1,1,1,9,1188.075336,883.5809344,2.886997997,3
806,74,2010-12-08,0,3,0,5,23,1,29,1,2,207,81,0,1,1,1,1,9,1188.075336,883.5809344,2.886997997,3
807,74,2010-12-08,1,3,0,37.6666679382324,19.6666660308838,2.66666674613953,60,3,2,207,81,
808,74,2010-12-08,2,3,0,32,20.2000007629395,7.80000019073486,60,3,2,207,81,0,1,1,1,1,9,1188.
809,74,2010-12-08,3,3,0,39.5,17.5,3,60,3,2,207,81,0,1,1,1,1,9,1188.075336,883.5809344,2.886997
810,74,2010-12-08,4,3,0,24.25,20.25,0.75,45.25,3,2,207,81,0,1,1,1,1,9,1188.075336,883.5809344,2
811,77,2010-12-01,0,3,0,2,32,1,35,1,2,207,85,0,1,0,1,1,12,1142.255269,937.178978,0.91200097,3.
812,77,2010-12-01,1,3,0,5.66666650772095,36.3333320617676,18,60,3,2,207,85,0,1,0,1,1,12,1142
813,77,2010-12-01,2,3,0,28.3333339691162,30.8333339691162,0.833333313465118,60,3,2,207,85
814,77,2010-12-01,3,3,0,45,15,0,60,3,2,207,85,0,1,0,1,1,12,1142.255269,937.178978,0.91200097,3
815,77,2010-12-01,4,3,0,44,16,0,60,3,2,207,85,0,1,0,1,1,12,1142.255269,937.178978,0.91200097,3
816,77,2010-12-02,0,4,0,5,45,0,50,1,2,207,85,0,1,0,1,1,12,1142.255269,937.178978,0.91200097,3.
817,77,2010-12-02,1,4,0,10.6666669845581,40.6666679382324,8.66666698455811,60,3,2,207,85,
818,77,2010-12-02,2,4,0,35,24,1,60,3,2,207,85,0,1,0,1,1,12,1142.255269,937.178978,0.91200097,3
819,77,2010-12-02,3,4,0,13.3333330154419,45.6666679382324,1,60,3,2,207,85,0,1,0,1,1,12,1142.
820,77,2010-12-02,4,4,0,43,16,1,60,3,2,207,85,0,1,0,1,1,12,1142.255269,937.178978,0.91200097,3
821,77,2010-12-03,4,5,0,46.3333320617676,13.6666669845581,0,60,3,2,207,85,0,1,0,1,1,12,1142.
822,77,2010-12-05,0,0,1,34,26,0,60,1,2,207,85,0,1,0,1,1,12,1142.255269,937.178978,0.91200097,3
823,77,2010-12-05,1,0,1,15.3333330154419,42.3333320617676,2.33333325386047,60,3,2,207,85,
824,77,2010-12-05,2,0,1,55,4,1,60,1,2,207,85,0,1,0,1,1,12,1142.255269,937.178978,0.91200097,3.
825,77,2010-12-06,0,1,0,8,44,1,53,1,2,207,85,0,1,0,1,1,12,1142.255269,937.178978,0.91200097,3.
826,77,2010-12-06,1,1,0,6.33333349227905,41.3333320617676,12.3333330154419,60,3,2,207,85,
827,77,2010-12-06,2,1,0,36.5999984741211,19.2000007629395,4.19999980926514,60,3,2,207,85,
828,77,2010-12-06,3,1,0,10,38.3333320617676,11.6666669845581,60,3,2,207,85,0,1,0,1,1,12,1142
829,77,2010-12-06,4,1,0,49.3333320617676,10.6666669845581,0,60,3,2,207,85,0,1,0,1,1,12,1142.
830,77,2010-12-07,0,2,0,3.5,31.5,2,37,2,2,207,85,0,1,0,1,1,12,1142.255269,937.178978,0.9120009
831,77,2010-12-07,1,2,0,16.6666660308838,38,5.33333349227905,60,3,2,207,85,0,1,0,1,1,12,1142
832,77,2010-12-07,2,2,0,31.6666660308838,26.6666660308838,1.66666662693024,60,3,2,207,85,
833,77,2010-12-07,3,2,0,16.7999992370605,38.2000007629395,5,60,3,2,207,85,0,1,0,1,1,12,1142.
834,77,2010-12-07,4,2,0,49,9.66666698455811,1.33333337306976,60,3,2,207,85,0,1,0,1,1,12,1142
835,77,2010-12-08,0,3,0,10,35,0,45,1,2,207,85,0,1,0,1,1,12,1142.255269,937.178978,0.91200097,3
836,77,2010-12-08,1,3,0,23,26.3333339691162,9,58.3333320617676,3,2,207,85,0,1,0,1,1,12,1142.
837,77,2010-12-08,2,3,0,16,20.5,6,42.5,2,2,207,85,0,1,0,1,1,12,1142.255269,937.178978,0.912000
838,77,2010-12-08,3,3,0,19.5,37.5,3,60,3,2,207,85,0,1,0,1,1,12,1142.255269,937.178978,0.912000
839,77,2010-12-08,4,3,0,39.5,20.1666660308838,0.333333343267441,60,3,2,207,85,0,1,0,1,1,12,1
840,78,2010-12-03,0,5,0,27,31,2,60,1,2,207,69,1,1,0,1,1,11,948.8338653,855.9636594,0.68837276
841,78,2010-12-03,1,5,0,23.6666660308838,35,1.33333337306976,60,3,2,207,69,1,1,0,1,1,11,948.
842,78,2010-12-03,2,5,0,15.6666669845581,30.6666660308838,13.6666669845581,60,3,2,207,69,
843,78,2010-12-03,3,5,0,18.3333339691162,36,5.66666650772095,60,3,2,207,69,1,1,0,1,1,11,948.
844,78,2010-12-03,4,5,0,26,34,0,60,3,2,207,69,1,1,0,1,1,11,948.8338653,855.9636594,0.68837276
845,78,2010-12-04,0,6,1,19.75,26.5,0.25,46.5,3,2,207,69,1,1,0,1,1,11,948.8338653,855.9636594,0.
846,78,2010-12-04,1,6,1,23.75,35.75,0.5,60,3,2,207,69,1,1,0,1,1,11,948.8338653,855.9636594,0.68
847,78,2010-12-04,2,6,1,23.3333339691162,36,0.666666686534882,60,3,2,207,69,1,1,0,1,1,11,948
848,78,2010-12-04,3,6,1,23,36.3333320617676,0.666666686534882,60,3,2,207,69,1,1,0,1,1,11,948
849,78,2010-12-04,4,6,1,21.3333339691162,38,0.666666686534882,60,3,2,207,69,1,1,0,1,1,11,948

850,78,2010-12-05,0,0,1,17.5,26.5,1.5,45.5,2,2,207,69,1,1,0,1,1,11,948.8338653,855.9636594,0.688
851,78,2010-12-05,1,0,1,17.6000003814697,39.7999992370605,2.59999990463257,60,3,2,207,69,
852,78,2010-12-05,2,0,1,26,34,0,60,3,2,207,69,1,1,0,1,1,11,948.8338653,855.9636594,0.68837276
853,78,2010-12-05,3,0,1,24,34.3333320617676,1.66666662693024,60,3,2,207,69,1,1,0,1,1,11,948.
854,78,2010-12-05,4,0,1,22,37,1,60,3,2,207,69,1,1,0,1,1,11,948.8338653,855.9636594,0.68837276
855,78,2010-12-06,0,1,0,27,5.5,0.5,33,2,2,207,69,1,1,0,1,1,11,948.8338653,855.9636594,0.688372
856,78,2010-12-06,1,1,0,24.3333339691162,34.3333320617676,1.33333337306976,60,3,2,207,69,
857,78,2010-12-06,2,1,0,12.25,33.75,14,60,3,2,207,69,1,1,0,1,1,11,948.8338653,855.9636594,0.688
858,78,2010-12-06,3,1,0,18.3333339691162,35.3333320617676,6.33333349227905,60,3,2,207,69,
859,78,2010-12-06,4,1,0,20,35,5,60,3,2,207,69,1,1,0,1,1,11,948.8338653,855.9636594,0.68837276
860,78,2010-12-07,1,2,0,9.66666698455811,36,4,49.6666679382324,3,2,207,69,1,1,0,1,1,11,948.8
861,78,2010-12-07,2,2,0,29.3999996185303,29.6000003814697,1,60,3,2,207,69,1,1,0,1,1,11,948.8
862,78,2010-12-07,3,2,0,7.59999990463257,40.5999984741211,11.8000001907349,60,3,2,207,69,
863,78,2010-12-07,4,2,0,21.6666660308838,34.6666679382324,3.66666674613953,60,3,2,207,69,
864,78,2010-12-08,0,3,0,23.5,29.5,0.5,53.5,2,2,207,69,1,1,0,1,1,11,948.8338653,855.9636594,0.688
865,78,2010-12-08,1,3,0,12.6666669845581,39.3333320617676,8,60,3,2,207,69,1,1,0,1,1,11,948.8
866,78,2010-12-08,2,3,0,23.6666660308838,34.6666679382324,1.66666662693024,60,3,2,207,69,
867,78,2010-12-08,3,3,0,7.75,48.25,4,60,3,2,207,69,1,1,0,1,1,11,948.8338653,855.9636594,0.68837
868,78,2010-12-08,4,3,0,14.6666669845581,42.5,2.83333325386047,60,3,2,207,69,1,1,0,1,1,11,94
869,91,2010-12-08,0,3,0,27,31,2,60,1,2,208,80,1,1,0,1,1,8,1010.290313,1074.18078,6.013852546,0
870,91,2011-02-02,0,3,0,22.5,27.5,0.5,50.5,2,2,208,80,1,1,0,1,1,8,1010.290313,1074.18078,6.0138
871,91,2011-02-02,1,3,0,25.6000003814697,33.5999984741211,0.800000011920929,60,3,2,208,80
872,91,2011-02-02,2,3,0,15.6666669845581,30.6666660308838,13.6666669845581,60,3,2,208,80,
873,91,2011-02-02,3,3,0,18.3333339691162,36,5.66666650772095,60,3,2,208,80,1,1,0,1,1,8,1010.
874,91,2011-02-02,4,3,0,26,34,0,60,3,2,208,80,1,1,0,1,1,8,1010.290313,1074.18078,6.013852546,0
875,91,2011-02-03,0,4,0,17,25.5,0,42.5,2,2,208,80,1,1,0,1,1,8,1010.290313,1074.18078,6.0138525
876,91,2011-02-03,1,4,0,14.25,45,0.75,60,3,2,208,80,1,1,0,1,1,8,1010.290313,1074.18078,6.01385
877,91,2011-02-03,2,4,0,23.3333339691162,36,0.666666686534882,60,3,2,208,80,1,1,0,1,1,8,1010
878,91,2011-02-03,3,4,0,23,36.3333320617676,0.666666686534882,60,3,2,208,80,1,1,0,1,1,8,1010
879,91,2011-02-03,4,4,0,21.3333339691162,38,0.666666686534882,60,3,2,208,80,1,1,0,1,1,8,1010
880,91,2011-02-04,0,5,0,17.5,26.5,1.5,45.5,2,2,208,80,1,1,0,1,1,8,1010.290313,1074.18078,6.0138
881,91,2011-02-04,1,5,0,23,33,4,60,3,2,208,80,1,1,0,1,1,8,1010.290313,1074.18078,6.013852546,0
882,91,2011-02-04,2,5,0,24.5,35,0.5,60,3,2,208,80,1,1,0,1,1,8,1010.290313,1074.18078,6.0138525
883,91,2011-02-04,3,5,0,24,34.3333320617676,1.66666662693024,60,3,2,208,80,1,1,0,1,1,8,1010.
884,91,2011-02-04,4,5,0,22,37,1,60,3,2,208,80,1,1,0,1,1,8,1010.290313,1074.18078,6.013852546,0
885,91,2011-02-05,0,6,1,27,5.5,0.5,33,2,2,208,80,1,1,0,1,1,8,1010.290313,1074.18078,6.01385254
886,91,2011-02-05,1,6,1,24.3333339691162,34.3333320617676,1.33333337306976,60,3,2,208,80,
887,91,2011-02-05,2,6,1,21.3999996185303,27.6000003814697,11,60,3,2,208,80,1,1,0,1,1,8,1010.
888,91,2011-02-05,3,6,1,18.3333339691162,35.3333320617676,6.33333349227905,60,3,2,208,80,
889,91,2011-02-05,4,6,1,20,35,5,60,3,2,208,80,1,1,0,1,1,8,1010.290313,1074.18078,6.013852546,0
890,91,2011-02-06,1,0,1,9.66666698455811,36,4,49.6666679382324,3,2,208,80,1,1,0,1,1,8,1010.2
891,91,2011-02-06,2,0,1,23,35.6666679382324,1.33333337306976,60,3,2,208,80,1,1,0,1,1,8,1010.
892,91,2011-02-06,3,0,1,7.83333349227905,41.5,10.6666669845581,60,3,2,208,80,1,1,0,1,1,8,101
893,91,2011-02-06,4,0,1,18,39.5999984741211,2.40000009536743,60,3,2,208,80,1,1,0,1,1,8,1010.
894,91,2011-02-07,0,1,0,23.5,29.5,0.5,53.5,2,2,208,80,1,1,0,1,1,8,1010.290313,1074.18078,6.0138
895,91,2011-02-07,1,1,0,12.6666669845581,39.3333320617676,8,60,3,2,208,80,1,1,0,1,1,8,1010.2
896,91,2011-02-07,2,1,0,23.6666660308838,34.6666679382324,1.66666662693024,60,3,2,208,80,
897,91,2011-02-07,3,1,0,7.33333349227905,49,3.66666674613953,60,3,2,208,80,1,1,0,1,1,8,1010.
898,91,2011-02-07,4,1,0,15.75,40.25,4,60,3,2,208,80,1,1,0,1,1,8,1010.290313,1074.18078,6.01385
899,95,2010-12-29,2,3,0,28.5,30,1.5,60,2,2,208,67,1,1,0,1,1,11,869.5374285,1006.186545,3.74315

900,95,2010-12-29,3,3,0,29,28.6666660308838,2.33333325386047,60,3,2,208,67,1,1,0,1,1,11,869.5
901,95,2010-12-29,4,3,0,36.3333320617676,23.3333339691162,0.333333343267441,60,3,2,208,67
902,95,2011-01-01,1,6,1,23,28,7.66666650772095,58.6666679382324,3,2,208,67,1,1,0,1,1,11,869.5
903,95,2011-01-01,2,6,1,21,28.3999996185303,10.6000003814697,60,3,2,208,67,1,1,0,1,1,11,869.5
904,95,2011-01-01,3,6,1,16,35.4000015258789,8.60000038146973,60,3,2,208,67,1,1,0,1,1,11,869.5
905,95,2011-01-01,4,6,1,27.6666660308838,29,3.33333325386047,60,3,2,208,67,1,1,0,1,1,11,869.5
906,95,2011-01-02,0,0,1,1,15,2,18,1,2,208,67,1,1,0,1,1,11,869.5374285,1006.186545,3.743157906,
907,95,2011-01-02,1,0,1,39.6666679382324,17,3.33333325386047,60,3,2,208,67,1,1,0,1,1,11,869.5
908,95,2011-01-02,2,0,1,17.3333339691162,38,4.66666650772095,60,3,2,208,67,1,1,0,1,1,11,869.5
909,95,2011-01-02,3,0,1,23.75,28.25,8,60,3,2,208,67,1,1,0,1,1,11,869.5374285,1006.186545,3.743
910,95,2011-01-02,4,0,1,40.8333320617676,18.5,0.666666686534882,60,3,2,208,67,1,1,0,1,1,11,8
911,95,2011-01-03,0,1,0,14,16,0.5,30.5,2,2,208,67,1,1,0,1,1,11,869.5374285,1006.186545,3.74315
912,95,2011-01-03,1,1,0,34.4000015258789,23.7999992370605,1.79999995231628,60,3,2,208,67,
913,95,2011-01-03,2,1,0,26.3333339691162,31.6666660308838,2,60,3,2,208,67,1,1,0,1,1,11,869.5
914,95,2011-01-03,3,1,0,15,33,12,60,3,2,208,67,1,1,0,1,1,11,869.5374285,1006.186545,3.7431579
915,95,2011-01-03,4,1,0,32.6666679382324,23,4.33333349227905,60,3,2,208,67,1,1,0,1,1,11,869.5
916,95,2011-01-04,0,2,0,18.5,23.5,0,42,2,2,208,67,1,1,0,1,1,11,869.5374285,1006.186545,3.74315
917,95,2011-01-04,1,2,0,27.25,23.75,9,60,3,2,208,67,1,1,0,1,1,11,869.5374285,1006.186545,3.743
918,95,2011-01-04,2,2,0,29,30.5,0.5,60,3,2,208,67,1,1,0,1,1,11,869.5374285,1006.186545,3.74315
919,95,2011-01-04,3,2,0,19,33,8,60,3,2,208,67,1,1,0,1,1,11,869.5374285,1006.186545,3.74315790
920,95,2011-01-04,4,2,0,32.3333320617676,27.3333339691162,0.333333343267441,60,3,2,208,67
921,95,2011-02-07,0,1,0,53,6,1,60,1,2,208,67,1,1,0,1,1,11,869.5374285,1006.186545,3.743157906,
922,95,2011-02-07,1,1,0,39,18,3,60,1,2,208,67,1,1,0,1,1,11,869.5374285,1006.186545,3.74315790
923,95,2011-02-08,0,2,0,35,15.6666669845581,1.33333337306976,52,3,2,208,67,1,1,0,1,1,11,869.5
924,95,2011-02-08,1,2,0,22.7999992370605,33,4.19999980926514,60,3,2,208,67,1,1,0,1,1,11,869.5
925,95,2011-02-08,2,2,0,22.25,31,6.75,60,3,2,208,67,1,1,0,1,1,11,869.5374285,1006.186545,3.743
926,95,2011-02-08,3,2,0,18.3333339691162,28.3333339691162,13.3333330154419,60,3,2,208,67,
927,95,2011-02-08,4,2,0,19.6666660308838,39.6666679382324,0.666666686534882,60,3,2,208,67
928,102,2012-02-19,0,7,1,30,23,7,60,1,2,208,74,0,1,0,1,1,12,953.4619183,941.7441145,4.5628811
929,102,2012-02-19,1,7,1,47,7.66666650772095,5.33333349227905,60,3,2,208,74,0,1,0,1,1,12,953
930,102,2012-02-19,2,7,1,44.6666679382324,9,6.33333349227905,60,3,2,208,74,0,1,0,1,1,12,953.
931,102,2012-02-19,3,7,1,45,14,1,60,3,2,208,74,0,1,0,1,1,12,953.4619183,941.7441145,4.5628811
932,102,2012-02-19,4,7,1,53.3333320617676,6.66666650772095,0,60,3,2,208,74,0,1,0,1,1,12,953.
933,102,2012-02-20,0,1,0,11,8.66666698455811,4.33333349227905,24,3,2,208,74,0,1,0,1,1,12,953
934,102,2012-02-20,1,1,0,24.3333339691162,31.3333339691162,4.33333349227905,60,3,2,208,74
935,102,2012-02-20,2,1,0,43,16.3333339691162,0.666666686534882,60,3,2,208,74,0,1,0,1,1,12,95
936,102,2012-02-20,3,1,0,25,29.6666660308838,5.33333349227905,60,3,2,208,74,0,1,0,1,1,12,953
937,102,2012-02-20,4,1,0,51.6666679382324,7.33333349227905,1,60,3,2,208,74,0,1,0,1,1,12,953.
938,102,2012-02-21,0,2,0,9,12,1.33333337306976,22.3333339691162,3,2,208,74,0,1,0,1,1,12,953.
939,102,2012-02-21,1,2,0,6.66666650772095,35,18.3333339691162,60,3,2,208,74,0,1,0,1,1,12,953
940,102,2012-02-21,2,2,0,34.6666679382324,19,6.33333349227905,60,3,2,208,74,0,1,0,1,1,12,953
941,102,2012-02-21,3,2,0,0.666666686534882,28,31.3333339691162,60,3,2,208,74,0,1,0,1,1,12,95
942,102,2012-02-21,4,2,0,44.3333320617676,12.3333330154419,3.33333325386047,60,3,2,208,74
943,102,2012-02-22,0,3,0,7.66666650772095,14.6666669845581,4,26.3333339691162,3,2,208,74,
944,102,2012-02-22,1,3,0,35,24.3333339691162,0.666666686534882,60,3,2,208,74,0,1,0,1,1,12,95
945,102,2012-02-22,2,3,0,50.3333320617676,8.66666698455811,1,60,3,2,208,74,0,1,0,1,1,12,953.
946,102,2012-02-22,3,3,0,22,36,2,60,3,2,208,74,0,1,0,1,1,12,953.4619183,941.7441145,4.5628811
947,102,2012-02-22,4,3,0,51.6666679382324,7.33333349227905,1,60,3,2,208,74,0,1,0,1,1,12,953.
948,102,2012-02-23,0,4,0,3.33333325386047,22,13,38.3333320617676,3,2,208,74,0,1,0,1,1,12,953
949,102,2012-02-23,1,4,0,8,25.6666660308838,26.3333339691162,60,3,2,208,74,0,1,0,1,1,12,953.

950,102,2012-02-23,2,4,0,33,15,12,60,3,2,208,74,0,1,0,1,1,12,953.4619183,941.7441145,4.5628811
951,102,2012-02-23,3,4,0,11.3333330154419,31.6666660308838,17,60,3,2,208,74,0,1,0,1,1,12,953
952,102,2012-02-23,4,4,0,44.3333320617676,13,2.66666674613953,60,3,2,208,74,0,1,0,1,1,12,953
953,102,2012-02-24,0,5,0,4.66666650772095,16,2.33333325386047,23,3,2,208,74,0,1,0,1,1,12,953
954,102,2012-02-24,1,5,0,28.6666660308838,28.3333339691162,3,60,3,2,208,74,0,1,0,1,1,12,953.
955,102,2012-02-24,2,5,0,50.6666679382324,7.66666650772095,1.66666662693024,60,3,2,208,74
956,102,2012-02-24,3,5,0,50.6666679382324,8.33333301544189,1,60,3,2,208,74,0,1,0,1,1,12,953.
957,102,2012-02-24,4,5,0,47.6666679382324,10,2.33333325386047,60,3,2,208,74,0,1,0,1,1,12,953
958,102,2012-02-25,0,6,1,11,11,2,24,3,2,208,74,0,1,0,1,1,12,953.4619183,941.7441145,4.5628811
959,102,2012-02-25,1,6,1,14.6666669845581,36,9.33333301544189,60,3,2,208,74,0,1,0,1,1,12,953
960,102,2012-02-25,2,6,1,43.3333320617676,13,3.66666674613953,60,3,2,208,74,0,1,0,1,1,12,953
961,102,2012-02-25,3,6,1,33.6666679382324,18.6666660308838,7.66666650772095,60,3,2,208,74
962,102,2012-02-25,4,6,1,51.6666679382324,7.66666650772095,0.666666686534882,60,3,2,208,7
963,103,2012-02-19,0,7,1,55,2,3,60,1,2,208,70,1,1,1,1,1,12,1110.970266,1082.673038,5.29641283,
964,103,2012-02-19,1,7,1,11.3333330154419,41,7.66666650772095,60,3,2,208,70,1,1,1,1,1,12,111
965,103,2012-02-19,2,7,1,12.6666669845581,32.6666679382324,14.6666669845581,60,3,2,208,70
966,103,2012-02-19,3,7,1,22,28.3333339691162,9.66666698455811,60,3,2,208,70,1,1,1,1,1,12,111
967,103,2012-02-19,4,7,1,28.6666660308838,26,5.33333349227905,60,3,2,208,70,1,1,1,1,1,12,111
968,103,2012-02-20,0,1,0,8.33333301544189,12,4,24.3333339691162,3,2,208,70,1,1,1,1,1,12,1110
969,103,2012-02-20,1,1,0,28,31,1,60,3,2,208,70,1,1,1,1,1,12,1110.970266,1082.673038,5.2964128
970,103,2012-02-20,2,1,0,21,35.6666679382324,3.33333325386047,60,3,2,208,70,1,1,1,1,1,12,111
971,103,2012-02-20,3,1,0,17.6666660308838,40,2.33333325386047,60,3,2,208,70,1,1,1,1,1,12,111
972,103,2012-02-20,4,1,0,24.3333339691162,24.6666660308838,11,60,3,2,208,70,1,1,1,1,1,12,111
973,103,2012-02-21,0,2,0,1.33333337306976,9.66666698455811,5.66666650772095,16.66666603
974,103,2012-02-21,1,2,0,33,25.3333339691162,1.66666662693024,60,3,2,208,70,1,1,1,1,1,12,111
975,103,2012-02-21,2,2,0,16.3333339691162,38.3333320617676,5.33333349227905,60,3,2,208,70
976,103,2012-02-21,3,2,0,14.6666669845581,35.6666679382324,9.66666698455811,60,3,2,208,70
977,103,2012-02-21,4,2,0,28,27.6666660308838,4.33333349227905,60,3,2,208,70,1,1,1,1,1,12,111
978,103,2012-02-22,0,3,0,0,6.66666650772095,3.33333325386047,10,3,2,208,70,1,1,1,1,1,12,1110
979,103,2012-02-22,1,3,0,29,28.6666660308838,2.33333325386047,60,3,2,208,70,1,1,1,1,1,12,111
980,103,2012-02-22,2,3,0,25.3333339691162,26,8.66666698455811,60,3,2,208,70,1,1,1,1,1,12,111
981,103,2012-02-22,3,3,0,12,30,18,60,3,2,208,70,1,1,1,1,1,12,1110.970266,1082.673038,5.296412
982,103,2012-02-22,4,3,0,13.3333330154419,27.6666660308838,19,60,3,2,208,70,1,1,1,1,1,12,111
983,103,2012-02-23,0,4,0,2.66666674613953,10.3333330154419,3.33333325386047,16.3333339
984,103,2012-02-23,1,4,0,31.3333339691162,27,1.66666662693024,60,3,2,208,70,1,1,1,1,1,12,111
985,103,2012-02-23,2,4,0,38,17.6666660308838,4.33333349227905,60,3,2,208,70,1,1,1,1,1,12,111
986,103,2012-02-23,3,4,0,25.6666660308838,31,3.33333325386047,60,3,2,208,70,1,1,1,1,1,12,111
987,103,2012-02-23,4,4,0,3,21.3333339691162,24.3333339691162,48.6666679382324,3,2,208,70,
988,103,2012-02-24,0,5,0,8,11.3333330154419,11.6666669845581,31,3,2,208,70,1,1,1,1,1,12,1110
989,103,2012-02-24,1,5,0,30.6666660308838,25.3333339691162,4,60,3,2,208,70,1,1,1,1,1,12,1110
990,103,2012-02-24,2,5,0,22.3333339691162,33.3333320617676,4.33333349227905,60,3,2,208,70
991,103,2012-02-24,3,5,0,11.6666669845581,35,13.3333330154419,60,3,2,208,70,1,1,1,1,1,12,111
992,103,2012-02-24,4,5,0,1.33333337306976,10,10,21.3333339691162,3,2,208,70,1,1,1,1,1,12,111
993,105,2010-12-22,0,3,0,20.5,11,0,31.5,3,2,208,82,1,1,1,0,1,8,842.6951192,873.9591819,3.44596
994,105,2010-12-22,1,3,0,7,52,1,60,2,2,208,82,1,1,1,0,1,8,842.6951192,873.9591819,3.445969737,
995,105,2010-12-22,4,3,0,35.3333320617676,24.6666660308838,0,60,3,2,208,82,1,1,1,0,1,8,842.6
996,105,2010-12-23,1,4,0,14.6000003814697,45.2000007629395,0.200000002980232,60,3,2,208,8
997,105,2010-12-23,2,4,0,29.3999996185303,30.3999996185303,0.200000002980232,60,3,2,208,8
998,105,2010-12-24,2,5,0,17.2857151031494,42.2857131958008,0.428571432828903,60,3,2,208,8
999,105,2010-12-24,3,5,0,45,15,0,60,3,2,208,82,1,1,1,0,1,8,842.6951192,873.9591819,3.4459697

1000,105,2010-12-25,0,6,1,1,59,0,60,2,2,208,82,1,1,1,0,1,8,842.6951192,873.9591819,3.44596973
1001,105,2010-12-25,3,6,1,49,11,0,60,2,2,208,82,1,1,1,0,1,8,842.6951192,873.9591819,3.44596973
1002,105,2010-12-25,4,6,1,15.6666669845581,29.3333339691162,0,45,3,2,208,82,1,1,1,0,1,8,842.
1003,105,2010-12-26,0,0,1,1.39999997615814,58.5999984741211,0,60,3,2,208,82,1,1,1,0,1,8,842.
1004,105,2010-12-26,1,0,1,0.800000011920929,59.2000007629395,0,60,3,2,208,82,1,1,1,0,1,8,842
1005,105,2010-12-27,1,1,0,5.7142858505249,54.2857131958008,0,60,3,2,208,82,1,1,1,0,1,8,842.6
1006,105,2010-12-27,2,1,0,35.7142868041992,24.2857151031494,0,60,3,2,208,82,1,1,1,0,1,8,842.
1007,105,2010-12-28,0,2,0,5,4,0,9,2,2,208,82,1,1,1,0,1,8,842.6951192,873.9591819,3.445969737,4
1008,105,2010-12-28,2,2,0,45.2000007629395,14.8000001907349,0,60,3,2,208,82,1,1,1,0,1,8,842.
1009,105,2010-12-28,3,2,0,35.2000007629395,12.6400003433228,12.1599998474121,60,3,2,208,8
1010,105,2010-12-28,4,2,0,42.533332824707,10.5333337783813,0.266666680574417,53.3333320
1011,105,2010-12-29,0,3,0,27.8888893127441,14.222222328186,1.11111116409302,43.2222213
1012,105,2010-12-29,1,3,0,32.2222213745117,24,3.77777767181396,60,3,2,208,82,1,1,1,0,1,8,842
1013,105,2010-12-29,4,3,0,21.2000007629395,8.39999961853027,0,29.6000003814697,3,2,208,82
1014,105,2010-12-30,1,4,0,32.2222213745117,27.7777786254883,0,60,3,2,208,82,1,1,1,0,1,8,842.
1015,105,2010-12-30,2,4,0,35.461540222168,23.1538467407227,1.38461542129517,60,3,2,208,82
1016,105,2010-12-30,3,4,0,27,30,3,60,2,2,208,82,1,1,1,0,1,8,842.6951192,873.9591819,3.44596973
1017,105,2010-12-31,2,5,0,20.6000003814697,30.6000003814697,8.80000019073486,60,3,2,208,8
1018,105,2010-12-31,3,5,0,30.25,20.0625,9.6875,60,3,2,208,82,1,1,1,0,1,8,842.6951192,873.95918
1019,105,2010-12-31,4,5,0,35.3333320617676,24,0.666666686534882,60,3,2,208,82,1,1,1,0,1,8,84
1020,105,2011-01-01,0,6,1,23.529411315918,12.4117650985718,0.0588235296308994,36,3,2,208,
1021,105,2011-01-01,1,6,1,46,14,0,60,3,2,208,82,1,1,1,0,1,8,842.6951192,873.9591819,3.44596973
1022,105,2011-01-01,4,6,1,23.3999996185303,10,1.39999997615814,34.7999992370605,3,2,208,8
1023,105,2011-01-02,0,0,1,36,23,1,60,3,2,208,82,1,1,1,0,1,8,842.6951192,873.9591819,3.44596973
1024,105,2011-01-02,1,0,1,41.4736824035645,18.5263156890869,0,60,3,2,208,82,1,1,1,0,1,8,842.
1025,105,2011-01-02,2,0,1,29,17.3333339691162,13.6666669845581,60,3,2,208,82,1,1,1,0,1,8,842
1026,105,2011-01-03,1,1,0,33,27,0,60,2,2,208,82,1,1,1,0,1,8,842.6951192,873.9591819,3.44596973
1027,105,2011-01-03,2,1,0,34.1111106872559,17.5555553436279,8.33333301544189,60,3,2,208,8
1028,105,2011-01-03,3,1,0,54.4000015258789,5.59999990463257,0,60,3,2,208,82,1,1,1,0,1,8,842.
1029,105,2011-02-01,3,2,0,39,21,0,60,2,2,208,82,1,1,1,0,1,8,842.6951192,873.9591819,3.44596973
1030,105,2011-02-01,4,2,0,43.4000015258789,14.8000001907349,0.200000002980232,58.400001
1031,105,2011-03-16,0,3,0,28.6666660308838,7.33333349227905,0.666666686534882,36.666667
1032,105,2011-03-16,3,3,0,36,24,0,60,1,2,208,82,1,1,1,0,1,8,842.6951192,873.9591819,3.44596973
1033,105,2011-03-16,4,3,0,30,14.5,0.5,45,3,2,208,82,1,1,1,0,1,8,842.6951192,873.9591819,3.44596
1034,105,2011-03-17,0,4,0,58,2,0,60,2,2,208,82,1,1,1,0,1,8,842.6951192,873.9591819,3.44596973
1035,105,2011-03-17,1,4,0,48,12,0,60,3,2,208,82,1,1,1,0,1,8,842.6951192,873.9591819,3.44596973
1036,105,2011-03-17,2,4,0,53,7,0,60,3,2,208,82,1,1,1,0,1,8,842.6951192,873.9591819,3.44596973
1037,105,2011-03-18,2,5,0,9.44444465637207,19,31.5555553436279,60,3,2,208,82,1,1,1,0,1,8,842
1038,105,2011-03-18,3,5,0,33.4000015258789,11,15.6000003814697,60,3,2,208,82,1,1,1,0,1,8,842
1039,105,2011-03-19,3,6,1,49,11,0,60,2,2,208,82,1,1,1,0,1,8,842.6951192,873.9591819,3.44596973
1040,105,2011-03-19,4,6,1,35.6666679382324,23.3333339691162,1,60,3,2,208,82,1,1,1,0,1,8,842.
1041,107,2012-02-18,0,6,1,16,39,5,60,1,2,208,78,1,0,1,0,1,8,871.9416786,959.2939377,1.4618414
1042,107,2012-02-18,1,6,1,21,35.3333320617676,3.66666674613953,60,3,2,208,78,1,0,1,0,1,8,871
1043,107,2012-02-18,2,6,1,40.3333320617676,19.6666660308838,0,60,3,2,208,78,1,0,1,0,1,8,871.
1044,107,2012-02-18,3,6,1,23.6666660308838,34,2.33333325386047,60,3,2,208,78,1,0,1,0,1,8,871
1045,107,2012-02-18,4,6,1,40,20,0,60,3,2,208,78,1,0,1,0,1,8,871.9416786,959.2939377,1.4618414
1046,107,2012-02-19,0,7,1,2.33333325386047,23.6666660308838,1.33333337306976,27.333333
1047,107,2012-02-19,1,7,1,20,37.3333320617676,2.66666674613953,60,3,2,208,78,1,0,1,0,1,8,871
1048,107,2012-02-19,2,7,1,38.6666679382324,21.3333339691162,0,60,3,2,208,78,1,0,1,0,1,8,871.
1049,107,2012-02-19,3,7,1,7.33333349227905,42.6666679382324,10,60,3,2,208,78,1,0,1,0,1,8,871

1050,107,2012-02-19,4,7,1,38.6666679382324,19.3333339691162,2,60,3,2,208,78,1,0,1,0,1,8,871.9
1051,107,2012-02-20,0,1,0,19,27.3333339691162,0.333333343267441,46.6666679382324,3,2,208,
1052,107,2012-02-20,1,1,0,27.6666660308838,31.6666660308838,0.666666686534882,60,3,2,208,
1053,107,2012-02-20,2,1,0,39.6666679382324,13,7.33333349227905,60,3,2,208,78,1,0,1,0,1,8,871
1054,107,2012-02-20,3,1,0,31.6666660308838,28.3333339691162,0,60,3,2,208,78,1,0,1,0,1,8,871.9
1055,107,2012-02-20,4,1,0,40.3333320617676,19.3333339691162,0.333333343267441,60,3,2,208,
1056,107,2012-02-21,0,2,0,23.3333339691162,25.6666660308838,2.66666674613953,51.6666679
1057,107,2012-02-21,1,2,0,25.3333339691162,33.6666679382324,1,60,3,2,208,78,1,0,1,0,1,8,871.9
1058,107,2012-02-21,2,2,0,34.3333320617676,19,6.66666650772095,60,3,2,208,78,1,0,1,0,1,8,871
1059,107,2012-02-21,3,2,0,32.6666679382324,27,0.333333343267441,60,3,2,208,78,1,0,1,0,1,8,87
1060,107,2012-02-21,4,2,0,44.6666679382324,15,0.333333343267441,60,3,2,208,78,1,0,1,0,1,8,87
1061,107,2012-02-22,0,3,0,4.33333349227905,39.3333320617676,4.66666650772095,48.3333320
1062,107,2012-02-22,1,3,0,8.33333301544189,44.6666679382324,7,60,3,2,208,78,1,0,1,0,1,8,871.9
1063,107,2012-02-22,2,3,0,32,25.3333339691162,2.66666674613953,60,3,2,208,78,1,0,1,0,1,8,871
1064,107,2012-02-22,3,3,0,29.3333339691162,29,1.66666662693024,60,3,2,208,78,1,0,1,0,1,8,871
1065,107,2012-02-22,4,3,0,40.6666679382324,19,0.333333343267441,60,3,2,208,78,1,0,1,0,1,8,87
1066,107,2012-02-23,0,4,0,20.3333339691162,26,1.66666662693024,48,3,2,208,78,1,0,1,0,1,8,871
1067,107,2012-02-23,1,4,0,23.3333339691162,34,2.66666674613953,60,3,2,208,78,1,0,1,0,1,8,871
1068,107,2012-02-23,2,4,0,34.6666679382324,24.6666660308838,0.666666686534882,60,3,2,208,
1069,107,2012-02-23,3,4,0,33,26.3333339691162,0.666666686534882,60,3,2,208,78,1,0,1,0,1,8,87
1070,107,2012-02-23,4,4,0,47,12.6666669845581,0.333333343267441,60,3,2,208,78,1,0,1,0,1,8,87
1071,113,2011-03-16,0,3,0,7,14,0,21,1,2,208,74,0,1,0,1,1,9,368.6355938,331.6605803,4.6296273622
1072,113,2011-03-16,1,3,0,49,11,0,60,3,2,208,74,0,1,0,1,1,9,368.6355938,331.6605803,4.62962736
1073,113,2011-03-16,2,3,0,46.25,13.25,0.5,60,3,2,208,74,0,1,0,1,1,9,368.6355938,331.6605803,4.6
1074,113,2011-03-16,3,3,0,43,9.33333301544189,7.66666650772095,60,3,2,208,74,0,1,0,1,1,9,368
1075,113,2011-03-16,4,3,0,29.6666660308838,15.6666669845581,14.6666669845581,60,3,2,208,7
1076,113,2011-03-17,0,4,0,25,11,0,36,2,2,208,74,0,1,0,1,1,9,368.6355938,331.6605803,4.62962736
1077,113,2011-03-17,1,4,0,40.3333320617676,17,2.66666674613953,60,3,2,208,74,0,1,0,1,1,9,368
1078,113,2011-03-17,2,4,0,28.3999996185303,30,1.60000002384186,60,3,2,208,74,0,1,0,1,1,9,368
1079,113,2011-03-17,3,4,0,11.25,27.75,21,60,3,2,208,74,0,1,0,1,1,9,368.6355938,331.6605803,4.62
1080,113,2011-03-17,4,4,0,39.6666679382324,20.3333339691162,0,60,3,2,208,74,0,1,0,1,1,9,368.6
1081,113,2011-03-18,0,5,0,2,14,0,16,1,2,208,74,0,1,0,1,1,9,368.6355938,331.6605803,4.6296273622
1082,113,2011-03-18,1,5,0,23.6666660308838,35.3333320617676,1,60,3,2,208,74,0,1,0,1,1,9,368.6
1083,113,2011-03-18,2,5,0,28,28,4,60,3,2,208,74,0,1,0,1,1,9,368.6355938,331.6605803,4.62962736
1084,113,2011-03-18,3,5,0,12.1999998092651,44.2000007629395,3.59999990463257,60,3,2,208,7
1085,113,2011-03-18,4,5,0,38.1666679382324,21.8333339691162,0,60,3,2,208,74,0,1,0,1,1,9,368.6
1086,113,2011-03-19,0,6,1,47,13,0,60,1,2,208,74,0,1,0,1,1,9,368.6355938,331.6605803,4.62962736
1087,113,2011-03-19,1,6,1,37,23,0,60,1,2,208,74,0,1,0,1,1,9,368.6355938,331.6605803,4.62962736
1088,113,2011-03-20,0,0,1,11,31,11,53,1,2,208,74,0,1,0,1,1,9,368.6355938,331.6605803,4.6296273
1089,113,2011-03-20,1,0,1,34,25.3999996185303,0.600000023841858,60,3,2,208,74,0,1,0,1,1,9,36
1090,113,2011-03-20,2,0,1,34.25,25,0.75,60,3,2,208,74,0,1,0,1,1,9,368.6355938,331.6605803,4.629
1091,113,2011-03-20,3,0,1,42,17.3333339691162,0.666666686534882,60,3,2,208,74,0,1,0,1,1,9,36
1092,113,2011-03-20,4,0,1,43.6666679382324,15.6666669845581,0.666666686534882,60,3,2,208,
1093,113,2011-03-21,0,1,0,14,32,0,46,1,2,208,74,0,1,0,1,1,9,368.6355938,331.6605803,4.62962736
1094,113,2011-03-21,1,1,0,26.3333339691162,33.6666679382324,0,60,3,2,208,74,0,1,0,1,1,9,368.6
1095,113,2011-03-21,2,1,0,23,35.7999992370605,1.20000004768372,60,3,2,208,74,0,1,0,1,1,9,368
1096,113,2011-03-21,3,1,0,25.6666660308838,32,2.33333325386047,60,3,2,208,74,0,1,0,1,1,9,368
1097,113,2011-03-21,4,1,0,38.6666679382324,21.3333339691162,0,60,3,2,208,74,0,1,0,1,1,9,368.6
1098,113,2011-03-22,0,2,0,12,23,0.5,35.5,2,2,208,74,0,1,0,1,1,9,368.6355938,331.6605803,4.62962
1099,113,2011-03-22,1,2,0,25,31.2000007629395,3.79999995231628,60,3,2,208,74,0,1,0,1,1,9,368

1100,113,2011-03-22,2,2,0,26.3333339691162,31.3333339691162,2.33333325386047,60,3,2,208,7
1101,113,2011-03-22,3,2,0,27.3333339691162,27.6666660308838,5,60,3,2,208,74,0,1,0,1,1,9,368.6
1102,113,2011-03-22,4,2,0,40.5,19.3333339691162,0.16666667163372,60,3,2,208,74,0,1,0,1,1,9,36
1103,116,2012-03-21,0,3,0,18,38,4,60,1,2,208,68,0,1,0,1,1,10,351.0141299,378.9749549,1.696210
1104,116,2012-03-21,1,3,0,20,33.6666679382324,6.33333349227905,60,3,2,208,68,0,1,0,1,1,10,35
1105,116,2012-03-21,2,3,0,25,29,6,60,3,2,208,68,0,1,0,1,1,10,351.0141299,378.9749549,1.696210
1106,116,2012-03-21,3,3,0,8,44,8,60,3,2,208,68,0,1,0,1,1,10,351.0141299,378.9749549,1.6962104
1107,116,2012-03-21,4,3,0,33,24.3333339691162,2.66666674613953,60,3,2,208,68,0,1,0,1,1,10,35
1108,116,2012-03-22,0,4,0,4.66666650772095,0.333333343267441,0,5,3,2,208,68,0,1,0,1,1,10,351
1109,116,2012-03-22,1,4,0,44,15,1,60,3,2,208,68,0,1,0,1,1,10,351.0141299,378.9749549,1.696210
1110,116,2012-03-22,2,4,0,21.3333339691162,37,1.66666662693024,60,3,2,208,68,0,1,0,1,1,10,35
1111,116,2012-03-22,3,4,0,18.6666660308838,41.3333320617676,0,60,3,2,208,68,0,1,0,1,1,10,351
1112,116,2012-03-22,4,4,0,26.3333339691162,31,2.66666674613953,60,3,2,208,68,0,1,0,1,1,10,35
1113,116,2012-03-23,0,5,0,9,1,1,11,3,2,208,68,0,1,0,1,1,10,351.0141299,378.9749549,1.69621042
1114,116,2012-03-23,1,5,0,1.66666662693024,41.3333320617676,17,60,3,2,208,68,0,1,0,1,1,10,35
1115,116,2012-03-23,2,5,0,8.33333301544189,15.3333330154419,36.3333320617676,60,3,2,208,6
1116,116,2012-03-23,3,5,0,8.66666698455811,24.3333339691162,27,60,3,2,208,68,0,1,0,1,1,10,35
1117,116,2012-03-23,4,5,0,40.6666679382324,18.6666660308838,0.666666686534882,60,3,2,208,
1118,116,2012-03-24,0,6,1,13.3333330154419,0.666666686534882,0,14,3,2,208,68,0,1,0,1,1,10,35
1119,116,2012-03-24,1,6,1,35.3333320617676,19.6666660308838,5,60,3,2,208,68,0,1,0,1,1,10,351
1120,116,2012-03-24,2,6,1,19,31.6666660308838,9.33333301544189,60,3,2,208,68,0,1,0,1,1,10,35
1121,116,2012-03-24,3,6,1,8.33333301544189,42,9.66666698455811,60,3,2,208,68,0,1,0,1,1,10,35
1122,116,2012-03-24,4,6,1,39.6666679382324,19.3333339691162,1,60,3,2,208,68,0,1,0,1,1,10,351
1123,116,2012-03-25,0,7,1,19.6666660308838,28,2,49.6666679382324,3,2,208,68,0,1,0,1,1,10,351
1124,116,2012-03-25,1,7,1,6.66666650772095,50,3.33333325386047,60,3,2,208,68,0,1,0,1,1,10,35
1125,116,2012-03-25,2,7,1,12.3333330154419,45,2.66666674613953,60,3,2,208,68,0,1,0,1,1,10,35
1126,116,2012-03-25,3,7,1,11.5,40,2,53.5,2,2,208,68,0,1,0,1,1,10,351.0141299,378.9749549,1.696
1127,118,2011-03-18,0,5,0,43,16,1,60,1,2,208,76,1,1,0,1,1,8,1927.648785,1974.724251,8.2703490
1128,118,2011-03-19,0,6,1,4,11,2,17,1,2,208,76,1,1,0,1,1,8,1927.648785,1974.724251,8.27034900
1129,118,2011-03-19,1,6,1,17,29,14,60,3,2,208,76,1,1,0,1,1,8,1927.648785,1974.724251,8.270349
1130,118,2011-03-19,2,6,1,29.6666660308838,27,3.33333325386047,60,3,2,208,76,1,1,0,1,1,8,192
1131,118,2011-03-19,3,6,1,18.3333339691162,36.6666679382324,5,60,3,2,208,76,1,1,0,1,1,8,1927
1132,118,2011-03-19,4,6,1,31.6666660308838,28.3333339691162,0,60,3,2,208,76,1,1,0,1,1,8,1927
1133,118,2011-03-20,0,0,1,21,36,2,59,1,2,208,76,1,1,0,1,1,8,1927.648785,1974.724251,8.2703490
1134,118,2011-03-20,1,0,1,33.3333320617676,25.3333339691162,1.33333337306976,60,3,2,208,7
1135,118,2011-03-20,2,0,1,23,34.3333320617676,2.66666674613953,60,3,2,208,76,1,1,0,1,1,8,192
1136,118,2011-03-20,3,0,1,25.6666660308838,28.3333339691162,6,60,3,2,208,76,1,1,0,1,1,8,1927
1137,118,2011-03-20,4,0,1,39.6666679382324,20,0.333333343267441,60,3,2,208,76,1,1,0,1,1,8,19
1138,118,2011-03-21,1,1,0,9,27.6666660308838,16.6666660308838,53.3333320617676,3,2,208,76
1139,118,2011-03-21,2,1,0,33.6666679382324,24.3333339691162,2,60,3,2,208,76,1,1,0,1,1,8,1927
1140,118,2011-03-21,3,1,0,17.8333339691162,36.1666679382324,6,60,3,2,208,76,1,1,0,1,1,8,1927
1141,118,2011-03-21,4,1,0,33,26.6666660308838,0.333333343267441,60,3,2,208,76,1,1,0,1,1,8,19
1142,118,2011-03-22,0,2,0,4,23.6666660308838,2.66666674613953,30.3333339691162,3,2,208,76
1143,118,2011-03-22,1,2,0,28.1666660308838,22.6666660308838,9.16666698455811,60,3,2,208,7
1144,118,2011-03-22,2,2,0,30.25,25,4.75,60,3,2,208,76,1,1,0,1,1,8,1927.648785,1974.724251,8.270
1145,118,2011-03-22,3,2,0,22.6666660308838,30,7.33333349227905,60,3,2,208,76,1,1,0,1,1,8,192
1146,118,2011-03-22,4,2,0,33.6666679382324,26,0.333333343267441,60,3,2,208,76,1,1,0,1,1,8,19
1147,118,2012-03-01,0,4,0,34,24,2,60,1,2,208,76,1,1,0,1,1,8,1927.648785,1974.724251,8.2703490
1148,118,2012-03-01,1,4,0,33,25.6666660308838,1.33333337306976,60,3,2,208,76,1,1,0,1,1,8,192
1149,118,2012-03-01,2,4,0,23.7999992370605,32.2000007629395,4,60,3,2,208,76,1,1,0,1,1,8,1927

1150,118,2012-03-01,3,4,0,23,29.6666660308838,7.33333349227905,60,3,2,208,76,1,1,0,1,1,8,192
1151,118,2012-03-01,4,4,0,34.6666679382324,23.6666660308838,1.66666662693024,60,3,2,208,7
1152,118,2012-03-02,0,5,0,10,24,2,36,1,2,208,76,1,1,0,1,1,8,1927.648785,1974.724251,8.2703490
1153,118,2012-03-02,1,5,0,13.6666669845581,23.3333339691162,23,60,3,2,208,76,1,1,0,1,1,8,192
1154,118,2012-03-02,2,5,0,30.6666660308838,28.3333339691162,1,60,3,2,208,76,1,1,0,1,1,8,1927
1155,118,2012-03-02,3,5,0,27.3333339691162,29.3333339691162,3.33333325386047,60,3,2,208,7
1156,118,2012-03-02,4,5,0,35.75,23.25,1,60,3,2,208,76,1,1,0,1,1,8,1927.648785,1974.724251,8.270
1157,118,2012-03-03,4,6,1,50.5,9.5,0,60,2,2,208,76,1,1,0,1,1,8,1927.648785,1974.724251,8.270349
1158,122,2012-03-06,0,2,0,40,16,4,60,1,2,208,74,0,1,0,1,1,12,1671.466853,1124.491448,6.7694873
1159,122,2012-03-06,1,2,0,14.3333330154419,29,16.6666660308838,60,3,2,208,74,0,1,0,1,1,12,16
1160,122,2012-03-06,2,2,0,15.6666669845581,29,15.3333330154419,60,3,2,208,74,0,1,0,1,1,12,16
1161,122,2012-03-06,3,2,0,23.3333339691162,30.6666660308838,6,60,3,2,208,74,0,1,0,1,1,12,167
1162,122,2012-03-06,4,2,0,34.6666679382324,20,5.33333349227905,60,3,2,208,74,0,1,0,1,1,12,16
1163,122,2012-03-07,0,3,0,6.66666650772095,8,2.66666674613953,17.3333339691162,3,2,208,74
1164,122,2012-03-07,1,3,0,24.6666660308838,25.3333339691162,10,60,3,2,208,74,0,1,0,1,1,12,16
1165,122,2012-03-07,2,3,0,38.3333320617676,16.3333339691162,5.33333349227905,60,3,2,208,7
1166,122,2012-03-07,3,3,0,22.6666660308838,27,10.3333330154419,60,3,2,208,74,0,1,0,1,1,12,16
1167,122,2012-03-07,4,3,0,21.3333339691162,25.3333339691162,13.3333330154419,60,3,2,208,7
1168,122,2012-03-08,0,4,0,3.33333325386047,10.3333330154419,1,14.6666669845581,3,2,208,74
1169,122,2012-03-08,1,4,0,23.6666660308838,30.6666660308838,5.66666650772095,60,3,2,208,7
1170,122,2012-03-08,2,4,0,33,25.6666660308838,1.33333337306976,60,3,2,208,74,0,1,0,1,1,12,16
1171,122,2012-03-08,3,4,0,26.6666660308838,26.3333339691162,7,60,3,2,208,74,0,1,0,1,1,12,167
1172,122,2012-03-08,4,4,0,41,10.3333330154419,8.66666698455811,60,3,2,208,74,0,1,0,1,1,12,16
1173,122,2012-03-09,0,5,0,4,5,3.66666674613953,12.6666669845581,3,2,208,74,0,1,0,1,1,12,1671
1174,122,2012-03-09,1,5,0,10,32.6666679382324,17.3333339691162,60,3,2,208,74,0,1,0,1,1,12,16
1175,122,2012-03-09,2,5,0,24.6666660308838,24.3333339691162,11,60,3,2,208,74,0,1,0,1,1,12,16
1176,122,2012-03-09,3,5,0,24.3333339691162,18.6666660308838,17,60,3,2,208,74,0,1,0,1,1,12,16
1177,122,2012-03-09,4,5,0,4.66666650772095,42.6666679382324,12.6666669845581,60,3,2,208,7
1178,122,2012-03-10,0,6,1,1.33333337306976,4.66666650772095,3.66666674613953,9.6666669845
1179,122,2012-03-10,1,6,1,35.6666679382324,20,4.33333349227905,60,3,2,208,74,0,1,0,1,1,12,16
1180,122,2012-03-10,2,6,1,42.6666679382324,8.66666698455811,8.66666698455811,60,3,2,208,7
1181,122,2012-03-10,3,6,1,41.6666679382324,10.6666669845581,7.66666650772095,60,3,2,208,7
1182,122,2012-03-10,4,6,1,5.66666650772095,42.6666679382324,11.6666669845581,60,3,2,208,7
1183,122,2012-03-11,1,7,1,33.6666679382324,11,5,49.6666679382324,3,2,208,74,0,1,0,1,1,12,167
1184,122,2012-03-11,2,7,1,13.6666669845581,26.6666660308838,19.6666660308838,60,3,2,208,7
1185,122,2012-03-11,3,7,1,29.3333339691162,20.6666660308838,10,60,3,2,208,74,0,1,0,1,1,12,16
1186,122,2012-03-11,4,7,1,27.3333339691162,13.3333330154419,8.66666698455811,49.3333320
1187,122,2012-03-12,0,1,0,5.33333349227905,8.66666698455811,0.666666686534882,14.6666669
1188,122,2012-03-12,1,1,0,14,36.3333320617676,9.66666698455811,60,3,2,208,74,0,1,0,1,1,12,16
1189,122,2012-03-12,2,1,0,10,37.6666679382324,12.3333330154419,60,3,2,208,74,0,1,0,1,1,12,16
1190,122,2012-03-12,3,1,0,19.3333339691162,25.6666660308838,15,60,3,2,208,74,0,1,0,1,1,12,16
1191,122,2012-03-12,4,1,0,31,21.3333339691162,7.66666650772095,60,3,2,208,74,0,1,0,1,1,12,16
1192,125,2012-03-08,1,4,0,4.33333349227905,8,0.666666686534882,13,3,2,208,79,1,1,1,1,1,5,835
1193,125,2012-03-08,2,4,0,32,27.6666660308838,0.333333343267441,60,3,2,208,79,1,1,1,1,1,5,83
1194,125,2012-03-08,3,4,0,21,38,1,60,3,2,208,79,1,1,1,1,1,5,835.9987918,897.5603387,3.5347873
1195,125,2012-03-08,4,4,0,35.3333320617676,24.6666660308838,0,60,3,2,208,79,1,1,1,1,1,5,835.9
1196,125,2012-03-10,1,6,1,34.3333320617676,11,1,46.3333320617676,3,2,208,79,1,1,1,1,1,5,835.9
1197,125,2012-03-10,2,6,1,51,9,0,60,3,2,208,79,1,1,1,1,1,5,835.9987918,897.5603387,3.5347873
1198,125,2012-03-10,3,6,1,42.3333320617676,17.6666660308838,0,60,3,2,208,79,1,1,1,1,1,5,835.9
1199,125,2012-03-10,4,6,1,35,24.6666660308838,0.333333343267441,60,3,2,208,79,1,1,1,1,1,5,83

1200,125,2012-03-11,0,7,1,19,0,0.333333343267441,19.3333339691162,3,2,208,79,1,1,1,1,5,835
1201,125,2012-03-11,1,7,1,29.6666660308838,29.6666660308838,0.666666686534882,60,3,2,208,
1202,125,2012-03-11,2,7,1,31.3333339691162,28.3333339691162,0.333333343267441,60,3,2,208,
1203,125,2012-03-11,3,7,1,24,36,0,60,3,2,208,79,1,1,1,1,5,835.9987918,897.5603387,3.53478739
1204,125,2012-03-11,4,7,1,44.6666679382324,15,0.333333343267441,60,3,2,208,79,1,1,1,1,5,83
1205,125,2012-03-12,1,1,0,30.3333339691162,28,0.666666686534882,59,3,2,208,79,1,1,1,1,5,83
1206,125,2012-03-12,2,1,0,37,23,0,60,3,2,208,79,1,1,1,1,5,835.9987918,897.5603387,3.53478739
1207,125,2012-03-12,3,1,0,36,24,0,60,3,2,208,79,1,1,1,1,5,835.9987918,897.5603387,3.53478739
1208,125,2012-03-12,4,1,0,30.3333339691162,29.6666660308838,0,60,3,2,208,79,1,1,1,1,5,835.9
1209,125,2012-03-13,1,2,0,18,22.3333339691162,0.666666686534882,41,3,2,208,79,1,1,1,1,5,83
1210,125,2012-03-13,2,2,0,34.6666679382324,25.3333339691162,0,60,3,2,208,79,1,1,1,1,5,835.9
1211,125,2012-03-13,3,2,0,25,34,1,60,3,2,208,79,1,1,1,1,5,835.9987918,897.5603387,3.53478739
1212,125,2012-03-13,4,2,0,40.3333320617676,19.6666660308838,0,60,3,2,208,79,1,1,1,1,5,835.9
1213,125,2012-03-14,0,3,0,6.33333349227905,5,0.333333343267441,11.6666669845581,3,2,208,7
1214,125,2012-03-14,1,3,0,28,32,0,60,3,2,208,79,1,1,1,1,5,835.9987918,897.5603387,3.53478739
1215,125,2012-03-14,2,3,0,26.6666660308838,33.3333320617676,0,60,3,2,208,79,1,1,1,1,5,835.9
1216,125,2012-03-14,3,3,0,33,24,3,60,3,2,208,79,1,1,1,1,5,835.9987918,897.5603387,3.53478739
1217,125,2012-03-14,4,3,0,20.3333339691162,32,0.333333343267441,52.6666679382324,3,2,208,
1218,125,2012-03-15,1,4,0,12.3333330154419,35.3333320617676,0.666666686534882,48.3333320
1219,125,2012-03-15,2,4,0,25,35,0,60,3,2,208,79,1,1,1,1,5,835.9987918,897.5603387,3.53478739
1220,125,2012-03-15,3,4,0,34,22,4,60,3,2,208,79,1,1,1,1,5,835.9987918,897.5603387,3.53478739
1221,125,2012-03-15,4,4,0,49.3333320617676,10.6666669845581,0,60,3,2,208,79,1,1,1,1,5,835.9
1222,127,2011-02-17,0,4,0,11.6666669845581,38.6666679382324,2.33333325386047,52.6666679
1223,127,2011-02-17,1,4,0,13.3333330154419,44,2.66666674613953,60,3,2,208,72,1,1,1,1,1,9,850
1224,127,2011-02-17,2,4,0,13.8000001907349,44,2.20000004768372,60,3,2,208,72,1,1,1,1,1,9,850
1225,127,2011-02-17,3,4,0,9.33333301544189,44,6.66666650772095,60,3,2,208,72,1,1,1,1,1,9,850
1226,127,2011-02-17,4,4,0,18.7999992370605,35.4000015258789,2.20000004768372,56.4000015
1227,127,2012-03-03,0,6,1,26,33,1,60,1,2,208,72,1,1,1,1,1,9,850.1233058,827.6911942,2.48715746
1228,127,2012-03-03,1,6,1,5.33333349227905,52.3333320617676,2.33333325386047,60,3,2,208,7
1229,127,2012-03-03,2,6,1,28.6666660308838,31,0.333333343267441,60,3,2,208,72,1,1,1,1,1,9,85
1230,127,2012-03-03,3,6,1,19.6666660308838,36.6666679382324,3.66666674613953,60,3,2,208,7
1231,127,2012-03-03,4,6,1,31.6666660308838,27.3333339691162,1,60,3,2,208,72,1,1,1,1,1,9,850.1
1232,127,2012-03-04,0,0,1,14.3333330154419,38,2.66666674613953,55,3,2,208,72,1,1,1,1,1,9,850
1233,127,2012-03-04,1,0,1,5.66666650772095,49.6666679382324,4.66666650772095,60,3,2,208,7
1234,127,2012-03-04,2,0,1,16.3333339691162,39.3333320617676,4.33333349227905,60,3,2,208,7
1235,127,2012-03-04,3,0,1,8.66666698455811,49.3333320617676,2,60,3,2,208,72,1,1,1,1,1,9,850.1
1236,127,2012-03-04,4,0,1,20,37,3,60,3,2,208,72,1,1,1,1,1,9,850.1233058,827.6911942,2.48715746
1237,127,2012-03-05,0,1,0,7,43,0.666666686534882,50.6666679382324,3,2,208,72,1,1,1,1,1,9,850
1238,127,2012-03-05,1,1,0,6.33333349227905,50.6666679382324,3,60,3,2,208,72,1,1,1,1,1,9,850.1
1239,127,2012-03-05,2,1,0,13,45,2,60,3,2,208,72,1,1,1,1,1,9,850.1233058,827.6911942,2.48715746
1240,127,2012-03-05,3,1,0,6.33333349227905,43.3333320617676,10.3333330154419,60,3,2,208,7
1241,127,2012-03-05,4,1,0,8.66666698455811,38,2,48.6666679382324,3,2,208,72,1,1,1,1,1,9,850.1
1242,127,2012-03-06,0,2,0,7.66666650772095,43,2,52.6666679382324,3,2,208,72,1,1,1,1,1,9,850.1
1243,127,2012-03-06,1,2,0,18.3333339691162,39.3333320617676,2.33333325386047,60,3,2,208,7
1244,127,2012-03-06,2,2,0,18.3333339691162,41.3333320617676,0.333333343267441,60,3,2,208,
1245,127,2012-03-06,3,2,0,9.66666698455811,48.3333320617676,2,60,3,2,208,72,1,1,1,1,1,9,850.1
1246,127,2012-03-06,4,2,0,9.66666698455811,31.3333339691162,11.6666669845581,52.6666679
1247,127,2012-03-07,0,3,0,5,13.5,1.5,20,2,2,208,72,1,1,1,1,1,9,850.1233058,827.6911942,2.4871574
1248,127,2012-03-07,1,3,0,4.66666650772095,51.6666679382324,3.66666674613953,60,3,2,208,7
1249,127,2012-03-07,2,3,0,29.25,28.5,2.25,60,3,2,208,72,1,1,1,1,1,9,850.1233058,827.6911942,2.4

1250,127,2012-03-07,3,3,0,23.6666660308838,34.6666679382324,1.66666662693024,60,3,2,208,7
1251,127,2012-03-07,4,3,0,20,29.5,3.5,53,2,2,208,72,1,1,1,1,1,9,850.1233058,827.6911942,2.4871
1252,130,2011-02-17,0,4,0,20.3999996185303,32.7999992370605,3.79999995231628,57,3,2,208,6
1253,130,2011-02-17,1,4,0,15,38,7,60,3,2,208,66,0,1,0,1,1,9,1927.366351,1998.711604,7.765838
1254,130,2011-02-17,2,4,0,27.6666660308838,27.8333339691162,4.5,60,3,2,208,66,0,1,0,1,1,9,19
1255,130,2011-02-17,3,4,0,20.6666660308838,34.6666679382324,4.66666650772095,60,3,2,208,6
1256,130,2011-02-17,4,4,0,31.5,25.5,3,60,3,2,208,66,0,1,0,1,1,9,1927.366351,1998.711604,7.7658
1257,130,2011-02-18,0,5,0,28.7142848968506,20,1.42857146263123,50.1428565979004,3,2,208,6
1258,130,2011-02-18,1,5,0,15.5454549789429,37,7.45454549789429,60,3,2,208,66,0,1,0,1,1,9,192
1259,130,2011-02-18,2,5,0,18.9166660308838,36.4166679382324,4.66666650772095,60,3,2,208,6
1260,130,2011-02-18,3,5,0,9,34.5,16.5,60,3,2,208,66,0,1,0,1,1,9,1927.366351,1998.711604,7.7658
1261,130,2011-02-18,4,5,0,37.5999984741211,21.6000003814697,0.800000011920929,60,3,2,208,
1262,130,2011-02-19,0,6,1,14.8571424484253,29,4.57142877578735,48.4285697937012,3,2,208,6
1263,130,2011-02-19,1,6,1,19.8888893127441,34.1111106872559,6,60,3,2,208,66,0,1,0,1,1,9,1927
1264,130,2011-02-19,2,6,1,23.1111106872559,32.2222213745117,4.66666650772095,60,3,2,208,6
1265,130,2011-02-19,3,6,1,15.1111106872559,36.4444427490234,6.66666650772095,58.2222213
1266,130,2011-02-19,4,6,1,29.875,27.25,2.875,60,3,2,208,66,0,1,0,1,1,9,1927.366351,1998.711604
1267,131,2012-03-22,0,4,0,60,0,0,60,1,2,208,81,1,0,0,1,1,7,1780.078908,1508.454241,6.72758601
1268,131,2012-03-22,1,4,0,13.6666669845581,41.6666679382324,4.66666650772095,60,3,2,208,8
1269,131,2012-03-22,2,4,0,19.3333339691162,40.6666679382324,0,60,3,2,208,81,1,0,0,1,1,7,1780
1270,131,2012-03-22,3,4,0,27,32.6666679382324,0.333333343267441,60,3,2,208,81,1,0,0,1,1,7,17
1271,131,2012-03-22,4,4,0,27.6666660308838,28.3333339691162,4,60,3,2,208,81,1,0,0,1,1,7,1780
1272,131,2012-03-23,0,5,0,13.3333330154419,21,2,36.3333320617676,3,2,208,81,1,0,0,1,1,7,1780
1273,131,2012-03-23,1,5,0,14.6666669845581,38.3333320617676,7,60,3,2,208,81,1,0,0,1,1,7,1780
1274,131,2012-03-23,2,5,0,24,30.3333339691162,5.66666650772095,60,3,2,208,81,1,0,0,1,1,7,178
1275,131,2012-03-23,3,5,0,29,30,1,60,3,2,208,81,1,0,0,1,1,7,1780.078908,1508.454241,6.72758601
1276,131,2012-03-23,4,5,0,28.3333339691162,29.6666660308838,2,60,3,2,208,81,1,0,0,1,1,7,1780
1277,131,2012-03-24,0,6,1,0.333333343267441,3.66666674613953,2.33333325386047,6.3333334
1278,131,2012-03-24,1,6,1,32,26.3333339691162,1.66666662693024,60,3,2,208,81,1,0,0,1,1,7,178
1279,131,2012-03-24,2,6,1,16.3333339691162,35.3333320617676,8.33333301544189,60,3,2,208,8
1280,131,2012-03-24,3,6,1,21,36.3333320617676,2.66666674613953,60,3,2,208,81,1,0,0,1,1,7,178
1281,131,2012-03-24,4,6,1,23,16,2.33333325386047,41.3333320617676,3,2,208,81,1,0,0,1,1,7,178
1282,131,2012-03-25,0,7,1,27.6666660308838,10.3333330154419,1.66666662693024,39.6666679
1283,131,2012-03-25,1,7,1,20.3333339691162,36.6666679382324,3,60,3,2,208,81,1,0,0,1,1,7,1780
1284,131,2012-03-25,2,7,1,27.3333339691162,27.6666660308838,5,60,3,2,208,81,1,0,0,1,1,7,1780
1285,131,2012-03-25,3,7,1,27,29,4,60,3,2,208,81,1,0,0,1,1,7,1780.078908,1508.454241,6.72758601
1286,131,2012-03-25,4,7,1,11,10.3333330154419,1.33333337306976,22.6666660308838,3,2,208,8
1287,131,2012-03-27,0,2,0,2,17.3333339691162,0.333333343267441,19.6666660308838,3,2,208,8
1288,131,2012-03-27,1,2,0,26.3333339691162,33,0.666666686534882,60,3,2,208,81,1,0,0,1,1,7,17
1289,131,2012-03-27,2,2,0,12.3333330154419,43.3333320617676,4.33333349227905,60,3,2,208,8
1290,131,2012-03-27,3,2,0,21.3333339691162,33.3333320617676,5.33333349227905,60,3,2,208,8
1291,131,2012-03-27,4,2,0,10.3333330154419,6.33333349227905,1.33333337306976,18,3,2,208,8
1292,131,2012-03-28,0,3,0,22.3333339691162,35,2.66666674613953,60,3,2,208,81,1,0,0,1,1,7,178
1293,131,2012-03-28,1,3,0,17.3333339691162,36.3333320617676,6.33333349227905,60,3,2,208,8
1294,131,2012-03-28,2,3,0,27.3333339691162,29.3333339691162,3.33333325386047,60,3,2,208,8
1295,131,2012-03-28,3,3,0,25.6666660308838,32.3333320617676,2,60,3,2,208,81,1,0,0,1,1,7,1780
1296,131,2012-03-28,4,3,0,16,19.3333339691162,2,37.3333320617676,3,2,208,81,1,0,0,1,1,7,1780
1297,131,2012-03-29,0,4,0,36.3333320617676,22.3333339691162,1,59.6666679382324,3,2,208,81
1298,131,2012-03-29,1,4,0,21.6666660308838,35.6666679382324,2.66666674613953,60,3,2,208,8
1299,131,2012-03-29,2,4,0,17.6666660308838,38,4.33333349227905,60,3,2,208,81,1,0,0,1,1,7,178

1300,131,2012-03-29,3,4,0,16.3333339691162,38.6666679382324,5,60,3,2,208,81,1,0,0,1,1,7,1780
1301,131,2012-03-29,4,4,0,8.66666698455811,14,1.33333337306976,24,3,2,208,81,1,0,0,1,1,7,178
1302,133,2011-02-19,0,6,1,60,0,0,60,1,2,208,73,1,1,0,1,1,11,1751.96764,963.7080832,7.16907614
1303,133,2011-02-19,1,6,1,29.25,28.5,1,58.75,3,2,208,73,1,1,0,1,1,11,1751.96764,963.7080832,7.1
1304,133,2011-02-19,2,6,1,29.6666660308838,26,4.33333349227905,60,3,2,208,73,1,1,0,1,1,11,17
1305,133,2011-02-19,3,6,1,41,18,1,60,3,2,208,73,1,1,0,1,1,11,1751.96764,963.7080832,7.1690761
1306,133,2011-02-19,4,6,1,38.6666679382324,20.6666660308838,0.666666686534882,60,3,2,208,
1307,133,2011-02-20,0,0,1,1.5,14.5,0.25,16.25,3,2,208,73,1,1,0,1,1,11,1751.96764,963.7080832,7.1
1308,133,2011-02-20,1,0,1,27.1428565979004,31.6428565979004,1.21428573131561,60,3,2,208,7
1309,133,2011-02-20,2,0,1,26.2666664123535,30.1333332061768,3.59999990463257,60,3,2,208,7
1310,133,2011-02-20,3,0,1,23.1333332061768,33.4000015258789,3.46666669845581,60,3,2,208,7
1311,133,2011-02-20,4,0,1,37.0833320617676,22.4166660308838,0.5,60,3,2,208,73,1,1,0,1,1,11,17
1312,133,2011-02-21,0,1,0,14,36,0,50,1,2,208,73,1,1,0,1,1,11,1751.96764,963.7080832,7.1690761
1313,133,2011-02-21,1,1,0,36.6666679382324,23,0.333333343267441,60,3,2,208,73,1,1,0,1,1,11,1
1314,133,2011-02-21,2,1,0,18.3333339691162,41.6666679382324,0,60,3,2,208,73,1,1,0,1,1,11,175
1315,133,2011-02-21,3,1,0,18.6666660308838,41,0.333333343267441,60,3,2,208,73,1,1,0,1,1,11,1
1316,133,2011-02-21,4,1,0,31.5,28.3333339691162,0.16666667163372,60,3,2,208,73,1,1,0,1,1,11,
1317,136,2012-03-22,0,4,0,19,25,16,60,1,2,208,68,1,1,0,1,1,9,1736.306832,932.8440704,7.677956
1318,136,2012-03-22,1,4,0,17,30.6666660308838,12.3333330154419,60,3,2,208,68,1,1,0,1,1,9,173
1319,136,2012-03-22,2,4,0,13,42,5,60,3,2,208,68,1,1,0,1,1,9,1736.306832,932.8440704,7.6779568
1320,136,2012-03-22,3,4,0,31.6666660308838,25.6666660308838,2.66666674613953,60,3,2,208,6
1321,136,2012-03-22,4,4,0,46,13,1,60,3,2,208,68,1,1,0,1,1,9,1736.306832,932.8440704,7.6779568
1322,136,2012-03-23,0,5,0,11.6666669845581,9.66666698455811,2,23.3333339691162,3,2,208,68
1323,136,2012-03-23,1,5,0,2.66666674613953,37.3333320617676,20,60,3,2,208,68,1,1,0,1,1,9,173
1324,136,2012-03-23,2,5,0,35.3333320617676,20,4.66666650772095,60,3,2,208,68,1,1,0,1,1,9,173
1325,136,2012-03-23,3,5,0,18.6666660308838,21.3333339691162,20,60,3,2,208,68,1,1,0,1,1,9,173
1326,136,2012-03-23,4,5,0,50.3333320617676,9.33333301544189,0.333333343267441,60,3,2,208,
1327,136,2012-03-24,0,6,1,13,8.66666698455811,0.666666686534882,22.3333339691162,3,2,208,
1328,136,2012-03-24,1,6,1,13,44,3,60,3,2,208,68,1,1,0,1,1,9,1736.306832,932.8440704,7.6779568
1329,136,2012-03-24,2,6,1,26.3333339691162,28.6666660308838,5,60,3,2,208,68,1,1,0,1,1,9,1736
1330,136,2012-03-24,3,6,1,6.66666650772095,51.3333320617676,2,60,3,2,208,68,1,1,0,1,1,9,1736
1331,136,2012-03-24,4,6,1,39.6666679382324,19.3333339691162,1,60,3,2,208,68,1,1,0,1,1,9,1736
1332,136,2012-03-25,0,7,1,28.6666660308838,10.3333330154419,1.66666662693024,40.666667
1333,136,2012-03-25,1,7,1,30.6666660308838,28.3333339691162,1,60,3,2,208,68,1,1,0,1,1,9,1736
1334,136,2012-03-25,2,7,1,38.6666679382324,21.3333339691162,0,60,3,2,208,68,1,1,0,1,1,9,1736
1335,136,2012-03-25,3,7,1,16.3333339691162,18.6666660308838,25,60,3,2,208,68,1,1,0,1,1,9,173
1336,136,2012-03-25,4,7,1,49,10,1,60,3,2,208,68,1,1,0,1,1,9,1736.306832,932.8440704,7.6779568
1337,136,2012-03-26,0,1,0,18.6666660308838,14,8,40.6666679382324,3,2,208,68,1,1,0,1,1,9,1736
1338,136,2012-03-26,1,1,0,14.3333330154419,34.3333320617676,11.3333330154419,60,3,2,208,6
1339,136,2012-03-26,2,1,0,24.3333339691162,16.6666660308838,19,60,3,2,208,68,1,1,0,1,1,9,173
1340,136,2012-03-26,3,1,0,38,17.6666660308838,4.33333349227905,60,3,2,208,68,1,1,0,1,1,9,173
1341,136,2012-03-26,4,1,0,42.6666679382324,16,1.33333337306976,60,3,2,208,68,1,1,0,1,1,9,173
1342,136,2012-03-27,0,2,0,20,19.3333339691162,3,42.3333320617676,3,2,208,68,1,1,0,1,1,9,1736
1343,136,2012-03-27,1,2,0,11.6666669845581,38.6666679382324,9.66666698455811,60,3,2,208,6
1344,136,2012-03-27,2,2,0,16.3333339691162,26.6666660308838,17,60,3,2,208,68,1,1,0,1,1,9,173
1345,136,2012-03-27,3,2,0,18.3333339691162,25,16.6666660308838,60,3,2,208,68,1,1,0,1,1,9,173
1346,136,2012-03-27,4,2,0,46,14,0,60,3,2,208,68,1,1,0,1,1,9,1736.306832,932.8440704,7.6779568
1347,136,2012-03-28,0,3,0,20.3333339691162,20.3333339691162,1.33333337306976,42,3,2,208,6
1348,136,2012-03-28,1,3,0,23.6666660308838,30.3333339691162,6,60,3,2,208,68,1,1,0,1,1,9,1736
1349,136,2012-03-28,2,3,0,26.6666660308838,21.3333339691162,12,60,3,2,208,68,1,1,0,1,1,9,173

1350,136,2012-03-28,3,3,0,22,21.6666660308838,16.3333339691162,60,3,2,208,68,1,1,0,1,1,9,173
1351,136,2012-03-28,4,3,0,43.3333320617676,16.6666660308838,0,60,3,2,208,68,1,1,0,1,1,9,1736
1352,136,2012-03-29,0,4,0,20,16,4.33333349227905,40.3333320617676,3,2,208,68,1,1,0,1,1,9,173
1353,136,2012-03-29,1,4,0,26.3333339691162,29,4.66666650772095,60,3,2,208,68,1,1,0,1,1,9,173
1354,136,2012-03-29,2,4,0,13.6666669845581,20,26.3333339691162,60,3,2,208,68,1,1,0,1,1,9,173
1355,136,2012-03-29,3,4,0,41,17.6666660308838,1.33333337306976,60,3,2,208,68,1,1,0,1,1,9,173
1356,136,2012-03-29,4,4,0,46.3333320617676,13.6666669845581,0,60,3,2,208,68,1,1,0,1,1,9,1736
1357,140,2012-03-28,0,3,0,9,44,7,60,1,2,208,70,1,1,0,1,1,7,980.5752385,682.1134234,2.23263033
1358,140,2012-03-28,1,3,0,14.6666669845581,39,6.33333349227905,60,3,2,208,70,1,1,0,1,1,7,980
1359,140,2012-03-28,2,3,0,7,39,14,60,3,2,208,70,1,1,0,1,1,7,980.5752385,682.1134234,2.2326303
1360,140,2012-03-28,3,3,0,6.33333349227905,40,13.6666669845581,60,3,2,208,70,1,1,0,1,1,7,980
1361,140,2012-03-28,4,3,0,32,27.3333339691162,0.666666686534882,60,3,2,208,70,1,1,0,1,1,7,98
1362,140,2012-03-29,0,4,0,6.66666650772095,23,2.66666674613953,32.3333320617676,3,2,208,7
1363,140,2012-03-29,1,4,0,13,39.6666679382324,7.33333349227905,60,3,2,208,70,1,1,0,1,1,7,980
1364,140,2012-03-29,2,4,0,22,30.3333339691162,7.66666650772095,60,3,2,208,70,1,1,0,1,1,7,980
1365,140,2012-03-29,3,4,0,24.3333339691162,27.6666660308838,8,60,3,2,208,70,1,1,0,1,1,7,980.
1366,140,2012-03-29,4,4,0,37,21,2,60,3,2,208,70,1,1,0,1,1,7,980.5752385,682.1134234,2.2326303
1367,140,2012-03-30,0,5,0,5,23.3333339691162,7,35.3333320617676,3,2,208,70,1,1,0,1,1,7,980.5
1368,140,2012-03-30,1,5,0,13.6666669845581,34,12.3333330154419,60,3,2,208,70,1,1,0,1,1,7,980
1369,140,2012-03-30,2,5,0,18.6666660308838,28,13.3333330154419,60,3,2,208,70,1,1,0,1,1,7,980
1370,140,2012-03-30,3,5,0,13.3333330154419,37.3333320617676,9.33333301544189,60,3,2,208,7
1371,140,2012-03-30,4,5,0,44.3333320617676,15.3333330154419,0.333333343267441,60,3,2,208,
1372,140,2012-03-31,0,6,1,12,19.6666660308838,0.333333343267441,32,3,2,208,70,1,1,0,1,1,7,98
1373,140,2012-03-31,1,6,1,17,37.6666679382324,5.33333349227905,60,3,2,208,70,1,1,0,1,1,7,980
1374,140,2012-03-31,2,6,1,17,33.3333320617676,9.66666698455811,60,3,2,208,70,1,1,0,1,1,7,980
1375,140,2012-03-31,3,6,1,24,31.3333339691162,4.66666650772095,60,3,2,208,70,1,1,0,1,1,7,980
1376,140,2012-03-31,4,6,1,32.6666679382324,23.6666660308838,3.66666674613953,60,3,2,208,7
1377,140,2012-04-01,0,7,1,10,21,2,33,3,2,208,70,1,1,0,1,1,7,980.5752385,682.1134234,2.2326303
1378,140,2012-04-01,1,7,1,24.3333339691162,33.3333320617676,2.33333325386047,60,3,2,208,7
1379,140,2012-04-01,2,7,1,21,36.6666679382324,2.33333325386047,60,3,2,208,70,1,1,0,1,1,7,980
1380,140,2012-04-01,3,7,1,25.3333339691162,33.3333320617676,1.33333337306976,60,3,2,208,7
1381,140,2012-04-01,4,7,1,34.3333320617676,23.6666660308838,2,60,3,2,208,70,1,1,0,1,1,7,980.
1382,140,2012-04-02,0,1,0,8.33333301544189,21,4.66666650772095,34,3,2,208,70,1,1,0,1,1,7,980
1383,140,2012-04-02,1,1,0,15.3333330154419,37.6666679382324,7,60,3,2,208,70,1,1,0,1,1,7,980.
1384,140,2012-04-02,2,1,0,24.6666660308838,30.3333339691162,5,60,3,2,208,70,1,1,0,1,1,7,980.
1385,140,2012-04-02,3,1,0,10.6666669845581,43.3333320617676,6,60,3,2,208,70,1,1,0,1,1,7,980.
1386,140,2012-04-02,4,1,0,41,17.3333339691162,1.66666662693024,60,3,2,208,70,1,1,0,1,1,7,980
1387,140,2012-04-03,0,2,0,8.33333301544189,22.6666660308838,3.33333325386047,34.333332
1388,140,2012-04-03,1,2,0,9.33333301544189,45,5.66666650772095,60,3,2,208,70,1,1,0,1,1,7,980
1389,140,2012-04-03,2,2,0,21.3333339691162,31.6666660308838,7,60,3,2,208,70,1,1,0,1,1,7,980.
1390,140,2012-04-03,3,2,0,9.33333301544189,42.3333320617676,8.33333301544189,60,3,2,208,7
1391,140,2012-04-03,4,2,0,36,22.3333339691162,1.66666662693024,60,3,2,208,70,1,1,0,1,1,7,980
1392,141,2011-02-21,0,1,0,9.4285717010498,31.5714282989502,4.85714292526245,45.85714340
1393,141,2011-02-21,1,1,0,13,40,3.09999990463257,56.0999984741211,3,2,208,76,0,1,0,1,1,9,190
1394,141,2011-02-21,2,1,0,38.4444427490234,20.6666660308838,0.888888895511627,60,3,2,208,
1395,141,2011-02-21,3,1,0,28.5555553436279,29.2222213745117,2.22222232818604,60,3,2,208,7
1396,141,2011-02-21,4,1,0,33.3333320617676,26.4444446563721,0.222222223877907,60,3,2,208,
1397,141,2011-02-22,0,2,0,10.5,33.125,2.625,46.25,3,2,208,76,0,1,0,1,1,9,1907.586859,2188.86839
1398,141,2011-02-22,1,2,0,14.8333330154419,41.25,3.91666674613953,60,3,2,208,76,0,1,0,1,1,9,1
1399,141,2011-02-22,2,2,0,27.25,31.75,1,60,3,2,208,76,0,1,0,1,1,9,1907.586859,2188.868396,7.81

1400,141,2011-02-22,3,2,0,22.3333339691162,34.75,2.91666674613953,60,3,2,208,76,0,1,0,1,1,9,
1401,141,2011-02-22,4,2,0,29.1666660308838,29.4166660308838,1.41666662693024,60,3,2,208,7
1402,141,2011-02-23,2,3,0,29.6666660308838,29.3333339691162,1,60,3,2,208,76,0,1,0,1,1,9,1907
1403,141,2011-02-23,3,3,0,41.6666679382324,18,0.333333343267441,60,3,2,208,76,0,1,0,1,1,9,19
1404,141,2011-02-23,4,3,0,12,19,0,31,1,2,208,76,0,1,0,1,1,9,1907.586859,2188.868396,7.8156932
1405,142,2012-03-30,0,5,0,4,6.33333349227905,0.333333343267441,10.6666669845581,3,2,208,8
1406,142,2012-03-30,1,5,0,35.6666679382324,24.3333339691162,0,60,3,2,208,85,1,1,1,1,1,5,1891
1407,142,2012-03-30,2,5,0,39,20.3333339691162,0.666666686534882,60,3,2,208,85,1,1,1,1,1,5,18
1408,142,2012-03-30,3,5,0,22,37.3333320617676,0.666666686534882,60,3,2,208,85,1,1,1,1,1,5,18
1409,142,2012-03-30,4,5,0,51,7.66666650772095,1.33333337306976,60,3,2,208,85,1,1,1,1,1,5,189
1410,142,2012-03-31,0,6,1,17,2.33333325386047,0.666666686534882,20,3,2,208,85,1,1,1,1,1,5,18
1411,142,2012-03-31,1,6,1,29,25.6666660308838,5.33333349227905,60,3,2,208,85,1,1,1,1,1,5,189
1412,142,2012-03-31,2,6,1,28.3333339691162,27.3333339691162,4.33333349227905,60,3,2,208,8
1413,142,2012-03-31,3,6,1,29.6666660308838,29.6666660308838,0.666666686534882,60,3,2,208,
1414,142,2012-03-31,4,6,1,39.6666679382324,19,1,59.6666679382324,3,2,208,85,1,1,1,1,1,5,1891
1415,142,2012-04-01,1,7,1,46.6666679382324,9.33333301544189,0.666666686534882,56.666667
1416,142,2012-04-01,2,7,1,46,13.3333330154419,0.666666686534882,60,3,2,208,85,1,1,1,1,1,5,18
1417,142,2012-04-01,3,7,1,36.3333320617676,21.6666660308838,2,60,3,2,208,85,1,1,1,1,1,5,1891
1418,142,2012-04-01,4,7,1,31.3333339691162,27.6666660308838,1,60,3,2,208,85,1,1,1,1,1,5,1891
1419,142,2012-04-02,0,1,0,19.6666660308838,6.66666650772095,1,27.3333339691162,3,2,208,85
1420,142,2012-04-02,1,1,0,44,16,0,60,3,2,208,85,1,1,1,1,1,5,1891.832834,2156.541812,7.7054144
1421,142,2012-04-02,2,1,0,37.6666679382324,22,0.333333343267441,60,3,2,208,85,1,1,1,1,1,5,18
1422,142,2012-04-02,3,1,0,19,38.6666679382324,2.33333325386047,60,3,2,208,85,1,1,1,1,1,5,189
1423,142,2012-04-02,4,1,0,50.3333320617676,9.33333301544189,0.333333343267441,60,3,2,208,
1424,142,2012-04-03,0,2,0,0.333333343267441,0.333333343267441,0,0.666666686534882,3,2,20
1425,142,2012-04-03,1,2,0,9.66666698455811,15,1.33333337306976,26,3,2,208,85,1,1,1,1,1,5,189
1426,142,2012-04-03,2,2,0,40.6666679382324,18.6666660308838,0.666666686534882,60,3,2,208,
1427,142,2012-04-03,3,2,0,47.6666679382324,11.6666669845581,0.666666686534882,60,3,2,208,
1428,142,2012-04-03,4,2,0,41,17.6666660308838,1.33333337306976,60,3,2,208,85,1,1,1,1,1,5,189
1429,143,2012-03-21,0,3,0,32,27,1,60,1,2,208,69,1,1,0,1,1,9,1861.339337,2023.637127,7.5539055
1430,143,2012-03-21,1,3,0,17.3333339691162,38.3333320617676,4.33333349227905,60,3,2,208,6
1431,143,2012-03-21,2,3,0,23,33,4,60,3,2,208,69,1,1,0,1,1,9,1861.339337,2023.637127,7.5539055
1432,143,2012-03-21,3,3,0,21.6666660308838,32.6666679382324,5.66666650772095,60,3,2,208,6
1433,143,2012-03-21,4,3,0,35.6666679382324,24.3333339691162,0,60,3,2,208,69,1,1,0,1,1,9,1861
1434,143,2012-03-22,0,4,0,6.66666650772095,38.6666679382324,1.33333337306976,46.666667
1435,143,2012-03-22,1,4,0,19.6666660308838,37.6666679382324,2.66666674613953,60,3,2,208,6
1436,143,2012-03-22,2,4,0,36.6666679382324,23,0.333333343267441,60,3,2,208,69,1,1,0,1,1,9,18
1437,143,2012-03-22,3,4,0,20.3333339691162,36.3333320617676,3.33333325386047,60,3,2,208,6
1438,143,2012-03-22,4,4,0,39,20,1,60,3,2,208,69,1,1,0,1,1,9,1861.339337,2023.637127,7.5539055
1439,143,2012-03-23,0,5,0,11,13.6666669845581,1.33333337306976,26,3,2,208,69,1,1,0,1,1,9,186
1440,143,2012-03-23,1,5,0,9,48.6666679382324,2.33333325386047,60,3,2,208,69,1,1,0,1,1,9,1861
1441,143,2012-03-23,2,5,0,27,26.3333339691162,6.66666650772095,60,3,2,208,69,1,1,0,1,1,9,186
1442,143,2012-03-23,3,5,0,24.6666660308838,29,6.33333349227905,60,3,2,208,69,1,1,0,1,1,9,186
1443,143,2012-03-23,4,5,0,41.3333320617676,16,2.66666674613953,60,3,2,208,69,1,1,0,1,1,9,186
1444,143,2012-03-24,0,6,1,7.33333349227905,11,0.333333343267441,18.6666660308838,3,2,208,
1445,143,2012-03-24,1,6,1,16,43.3333320617676,0.666666686534882,60,3,2,208,69,1,1,0,1,1,9,18
1446,143,2012-03-24,2,6,1,37.6666679382324,20,2.33333325386047,60,3,2,208,69,1,1,0,1,1,9,186
1447,143,2012-03-24,3,6,1,21.3333339691162,36,2.66666674613953,60,3,2,208,69,1,1,0,1,1,9,186
1448,143,2012-03-24,4,6,1,34,25.6666660308838,0.333333343267441,60,3,2,208,69,1,1,0,1,1,9,18
1449,143,2012-03-25,0,7,1,14.6666669845581,14.6666669845581,0.333333343267441,29.666666

1450,143,2012-03-25,1,7,1,19.3333339691162,39.6666679382324,1,60,3,2,208,69,1,1,0,1,1,9,1861
1451,143,2012-03-25,2,7,1,26.3333339691162,31.3333339691162,2.33333325386047,60,3,2,208,6
1452,143,2012-03-25,3,7,1,39,20.6666660308838,0.333333343267441,60,3,2,208,69,1,1,0,1,1,9,18
1453,143,2012-03-25,4,7,1,48.6666679382324,10,1.33333337306976,60,3,2,208,69,1,1,0,1,1,9,186
1454,143,2012-03-26,0,1,0,5.33333349227905,12.6666669845581,1,19,3,2,208,69,1,1,0,1,1,9,1861
1455,143,2012-03-26,1,1,0,21.6666660308838,38,0.333333343267441,60,3,2,208,69,1,1,0,1,1,9,18
1456,143,2012-03-26,2,1,0,28,28,4,60,3,2,208,69,1,1,0,1,1,9,1861.339337,2023.637127,7.5539055
1457,143,2012-03-26,3,1,0,37.6666679382324,21.3333339691162,1,60,3,2,208,69,1,1,0,1,1,9,1861
1458,143,2012-03-26,4,1,0,20,21.6666660308838,1,42.6666679382324,3,2,208,69,1,1,0,1,1,9,1861
1459,143,2012-03-27,0,2,0,13,24.6666660308838,1,38.6666679382324,3,2,208,69,1,1,0,1,1,9,1861
1460,143,2012-03-27,1,2,0,20.3333339691162,38.6666679382324,1,60,3,2,208,69,1,1,0,1,1,9,1861
1461,143,2012-03-27,2,2,0,35.3333320617676,23.6666660308838,1,60,3,2,208,69,1,1,0,1,1,9,1861
1462,143,2012-03-27,3,2,0,29,16.6666660308838,1.33333337306976,47,3,2,208,69,1,1,0,1,1,9,186
1463,143,2012-03-27,4,2,0,16.3333339691162,1.66666662693024,0.333333343267441,18.333333
1464,144,2011-02-23,0,3,0,25.5555553436279,16.2222213745117,0.555555582046509,42.333332
1465,144,2011-02-23,1,3,0,17.9285717010498,36,6.07142877578735,60,3,2,208,68,0,1,0,1,1,9,169
1466,144,2011-02-23,2,3,0,43.6666679382324,13.6666669845581,2.66666674613953,60,3,2,208,6
1467,144,2011-02-23,3,3,0,29.8333339691162,26,4.16666650772095,60,3,2,208,68,0,1,0,1,1,9,169
1468,144,2011-02-23,4,3,0,38,14.3999996185303,1.10000002384186,53.5,3,2,208,68,0,1,0,1,1,9,16
1469,144,2011-02-24,0,4,0,31,15.8333330154419,1.16666662693024,48,3,2,208,68,0,1,0,1,1,9,169
1470,144,2011-02-24,1,4,0,20.8999996185303,33.9000015258789,5.19999980926514,60,3,2,208,6
1471,144,2011-02-24,2,4,0,41.75,14.5833330154419,3.66666674613953,60,3,2,208,68,0,1,0,1,1,9,
1472,144,2011-02-24,3,4,0,30.25,27.5,2.25,60,3,2,208,68,0,1,0,1,1,9,1692.707591,1190.550145,8.3
1473,144,2011-02-24,4,4,0,36.1666679382324,20.8333339691162,1.91666662693024,58.916667
1474,145,2012-03-22,0,4,0,6,53,1,60,1,2,208,74,1,1,0,1,1,8,1622.103134,1212.737065,9.8719604
1475,145,2012-03-22,1,4,0,11.3333330154419,37.6666679382324,11,60,3,2,208,74,1,1,0,1,1,8,162
1476,145,2012-03-22,2,4,0,40.6666679382324,17.6666660308838,1.66666662693024,60,3,2,208,7
1477,145,2012-03-22,3,4,0,6.66666650772095,30.3333339691162,23,60,3,2,208,74,1,1,0,1,1,8,162
1478,145,2012-03-22,4,4,0,37,21,2,60,3,2,208,74,1,1,0,1,1,8,1622.103134,1212.737065,9.8719604
1479,145,2012-03-23,0,5,0,10.3333330154419,23.6666660308838,2,36,3,2,208,74,1,1,0,1,1,8,1622
1480,145,2012-03-23,1,5,0,11.6666669845581,43.3333320617676,5,60,3,2,208,74,1,1,0,1,1,8,1622
1481,145,2012-03-23,2,5,0,34.6666679382324,21.3333339691162,4,60,3,2,208,74,1,1,0,1,1,8,1622
1482,145,2012-03-23,3,5,0,31.3333339691162,17,11.6666669845581,60,3,2,208,74,1,1,0,1,1,8,162
1483,145,2012-03-23,4,5,0,45.6666679382324,14,0.333333343267441,60,3,2,208,74,1,1,0,1,1,8,16
1484,145,2012-03-24,0,6,1,9,20.3333339691162,4.33333349227905,33.6666679382324,3,2,208,74
1485,145,2012-03-24,1,6,1,6,34,20,60,3,2,208,74,1,1,0,1,1,8,1622.103134,1212.737065,9.8719604
1486,145,2012-03-24,2,6,1,39,19,2,60,3,2,208,74,1,1,0,1,1,8,1622.103134,1212.737065,9.8719604
1487,145,2012-03-24,3,6,1,16,42,2,60,3,2,208,74,1,1,0,1,1,8,1622.103134,1212.737065,9.8719604
1488,145,2012-03-24,4,6,1,34,20.3333339691162,5.66666650772095,60,3,2,208,74,1,1,0,1,1,8,162
1489,145,2012-03-25,0,7,1,12.3333330154419,39.3333320617676,2.66666674613953,54.333332
1490,145,2012-03-25,1,7,1,24.6666660308838,33.6666679382324,1.66666662693024,60,3,2,208,7
1491,145,2012-03-25,2,7,1,12.6666669845581,40.3333320617676,7,60,3,2,208,74,1,1,0,1,1,8,1622
1492,145,2012-03-25,3,7,1,21.3333339691162,17.3333339691162,21.3333339691162,60,3,2,208,7
1493,145,2012-03-25,4,7,1,5,3,1,9,3,2,208,74,1,1,0,1,1,8,1622.103134,1212.737065,9.871960455,1
1494,145,2012-03-26,0,1,0,14.3333330154419,22.3333339691162,16.6666660308838,53.333332
1495,145,2012-03-26,1,1,0,9,20,31,60,3,2,208,74,1,1,0,1,1,8,1622.103134,1212.737065,9.8719604
1496,145,2012-03-26,2,1,0,29.6666660308838,18.6666660308838,11.6666669845581,60,3,2,208,7
1497,145,2012-03-26,3,1,0,21,23.6666660308838,15.3333330154419,60,3,2,208,74,1,1,0,1,1,8,162
1498,145,2012-03-26,4,1,0,5,2.66666674613953,0.666666686534882,8.33333301544189,3,2,208,7
1499,145,2012-03-27,0,2,0,20.3333339691162,17.3333339691162,11.6666669845581,49.333332

1500,145,2012-03-27,1,2,0,16.3333339691162,17,26.6666660308838,60,3,2,208,74,1,1,0,1,1,8,162
1501,145,2012-03-27,2,2,0,34.3333320617676,20.6666660308838,5,60,3,2,208,74,1,1,0,1,1,8,1622
1502,145,2012-03-27,3,2,0,16.6666660308838,18,25.3333339691162,60,3,2,208,74,1,1,0,1,1,8,162
1503,145,2012-03-27,4,2,0,7.33333349227905,5.66666650772095,1,14,3,2,208,74,1,1,0,1,1,8,1622
1504,145,2012-03-28,0,3,0,16.3333339691162,20.6666660308838,8.66666698455811,45.666679
1505,145,2012-03-28,1,3,0,15.3333330154419,17,27.6666660308838,60,3,2,208,74,1,1,0,1,1,8,162
1506,145,2012-03-28,2,3,0,26.6666660308838,24,9.33333301544189,60,3,2,208,74,1,1,0,1,1,8,162
1507,145,2012-03-28,3,3,0,30,17.3333339691162,12.6666669845581,60,3,2,208,74,1,1,0,1,1,8,162
1508,145,2012-03-28,4,3,0,1.66666662693024,3.33333325386047,1,6,3,2,208,74,1,1,0,1,1,8,1622.1
1509,146,2011-02-24,0,4,0,24,24.3333339691162,0.666666686534882,49,3,2,208,77,0,1,0,1,1,9,16
1510,146,2011-02-24,1,4,0,24.75,32.75,2.5,60,3,2,208,77,0,1,0,1,1,9,1629.955856,1212.392101,8.9
1511,146,2011-02-24,2,4,0,44.6666679382324,13.6666669845581,1.66666662693024,60,3,2,208,7
1512,146,2011-02-24,3,4,0,23.3333339691162,33,3.66666674613953,60,3,2,208,77,0,1,0,1,1,9,162
1513,146,2011-02-24,4,4,0,46.6666679382324,13,0.333333343267441,60,3,2,208,77,0,1,0,1,1,9,16
1514,146,2012-03-14,0,3,0,23,12.5,0.5,36,2,2,208,77,0,1,0,1,1,9,1629.955856,1212.392101,8.95129
1515,146,2012-03-14,1,3,0,20.7999992370605,32.4000015258789,6.80000019073486,60,3,2,208,7
1516,146,2012-03-14,2,3,0,50.3333320617676,9.66666698455811,0,60,3,2,208,77,0,1,0,1,1,9,1629
1517,146,2012-03-14,3,3,0,33,18.6666660308838,8.33333301544189,60,3,2,208,77,0,1,0,1,1,9,162
1518,146,2012-03-14,4,3,0,32.5,16,4,52.5,2,2,208,77,0,1,0,1,1,9,1629.955856,1212.392101,8.95129
1519,146,2012-03-15,0,4,0,19.5,13.5,1,34,2,2,208,77,0,1,0,1,1,9,1629.955856,1212.392101,8.95129
1520,146,2012-03-15,1,4,0,7.66666650772095,41,11.3333330154419,60,3,2,208,77,0,1,0,1,1,9,162
1521,146,2012-03-15,2,4,0,47.7999992370605,9.60000038146973,2.59999990463257,60,3,2,208,7
1522,146,2012-03-15,3,4,0,41.3333320617676,18.3333339691162,0.333333343267441,60,3,2,208,
1523,146,2012-03-15,4,4,0,34.5,23,0.5,58,2,2,208,77,0,1,0,1,1,9,1629.955856,1212.392101,8.95129
1524,146,2012-03-16,0,5,0,36.5,10.5,0,47,2,2,208,77,0,1,0,1,1,9,1629.955856,1212.392101,8.95129
1525,146,2012-03-16,1,5,0,21,35,4,60,3,2,208,77,0,1,0,1,1,9,1629.955856,1212.392101,8.9512939
1526,146,2012-03-16,2,5,0,29,24.75,6.25,60,3,2,208,77,0,1,0,1,1,9,1629.955856,1212.392101,8.951
1527,146,2012-03-16,3,5,0,21.6666660308838,34,4.33333349227905,60,3,2,208,77,0,1,0,1,1,9,162
1528,146,2012-03-16,4,5,0,35.3333320617676,9,0.333333343267441,44.6666679382324,3,2,208,7
1529,146,2012-03-17,0,6,1,25.5,20,1.5,47,2,2,208,77,0,1,0,1,1,9,1629.955856,1212.392101,8.95129
1530,146,2012-03-17,1,6,1,11.3333330154419,39.6666679382324,9,60,3,2,208,77,0,1,0,1,1,9,1629
1531,146,2012-03-17,2,6,1,32,19,9,60,3,2,208,77,0,1,0,1,1,9,1629.955856,1212.392101,8.9512939
1532,146,2012-03-17,3,6,1,23.75,33.75,2.5,60,3,2,208,77,0,1,0,1,1,9,1629.955856,1212.392101,8.9
1533,146,2012-03-17,4,6,1,34,21.6666660308838,0,55.6666679382324,3,2,208,77,0,1,0,1,1,9,1629
1534,146,2012-03-18,0,0,1,42.5,16,1.5,60,2,2,208,77,0,1,0,1,1,9,1629.955856,1212.392101,8.95129
1535,146,2012-03-18,1,0,1,31,26.6666660308838,2.33333325386047,60,3,2,208,77,0,1,0,1,1,9,162
1536,146,2012-03-18,2,0,1,52.3333320617676,6.66666650772095,1,60,3,2,208,77,0,1,0,1,1,9,1629
1537,146,2012-03-18,3,0,1,41.5,17.5,1,60,3,2,208,77,0,1,0,1,1,9,1629.955856,1212.392101,8.95129
1538,146,2012-03-18,4,0,1,43.3333320617676,16.3333339691162,0.333333343267441,60,3,2,208,
1539,146,2012-03-19,0,1,0,25,11.5,0.5,37,2,2,208,77,0,1,0,1,1,9,1629.955856,1212.392101,8.95129
1540,146,2012-03-19,1,1,0,14.6666669845581,41,4.33333349227905,60,3,2,208,77,0,1,0,1,1,9,162
1541,146,2012-03-19,2,1,0,44,15,1,60,3,2,208,77,0,1,0,1,1,9,1629.955856,1212.392101,8.9512939
1542,146,2012-03-19,3,1,0,25.5,31.25,3.25,60,3,2,208,77,0,1,0,1,1,9,1629.955856,1212.392101,8.9
1543,146,2012-03-19,4,1,0,23.6666660308838,30,6.33333349227905,60,3,2,208,77,0,1,0,1,1,9,162
1544,146,2012-03-20,4,2,0,43.6666679382324,15.3333330154419,1,60,3,2,208,77,0,1,0,1,1,9,1629
1545,148,2012-03-14,0,3,0,9.5,35.5,4.5,49.5,3,2,208,71,1,1,0,1,1,9,1480.891505,1147.10447,9.2518
1546,148,2012-03-14,1,3,0,14.3333330154419,45.3333320617676,0.333333343267441,60,3,2,208,
1547,148,2012-03-14,2,3,0,18.3333339691162,38.6666679382324,3,60,3,2,208,71,1,1,0,1,1,9,1480
1548,148,2012-03-14,3,3,0,25.6666660308838,26.3333339691162,8,60,3,2,208,71,1,1,0,1,1,9,1480
1549,148,2012-03-14,4,3,0,23.6666660308838,23.3333339691162,0.333333343267441,47.333332

1550,148,2012-03-15,0,4,0,9.33333301544189,39.3333320617676,1,49.6666679382324,3,2,208,71
1551,148,2012-03-15,1,4,0,13.5,45,1.5,60,3,2,208,71,1,1,0,1,1,9,1480.891505,1147.10447,9.251801
1552,148,2012-03-15,2,4,0,22,26.6666660308838,11.3333330154419,60,3,2,208,71,1,1,0,1,1,9,148
1553,148,2012-03-15,3,4,0,13.6666669845581,29.3333339691162,17,60,3,2,208,71,1,1,0,1,1,9,148
1554,148,2012-03-15,4,4,0,38,11.5,0,49.5,2,2,208,71,1,1,0,1,1,9,1480.891505,1147.10447,9.251801
1555,148,2012-03-16,0,5,0,9.33333301544189,36,1.66666662693024,47,3,2,208,71,1,1,0,1,1,9,148
1556,148,2012-03-16,1,5,0,11.3333330154419,41.3333320617676,7.33333349227905,60,3,2,208,7
1557,148,2012-03-16,2,5,0,19.6666660308838,39.6666679382324,0.666666686534882,60,3,2,208,
1558,148,2012-03-16,3,5,0,17.3333339691162,37.3333320617676,5.33333349227905,60,3,2,208,7
1559,148,2012-03-16,4,5,0,37,13,0,50,3,2,208,71,1,1,0,1,1,9,1480.891505,1147.10447,9.25180151
1560,148,2012-03-17,0,6,1,4.5,36.5,1.5,42.5,2,2,208,71,1,1,0,1,1,9,1480.891505,1147.10447,9.251
1561,148,2012-03-17,1,6,1,7.33333349227905,42,10.6666669845581,60,3,2,208,71,1,1,0,1,1,9,148
1562,148,2012-03-17,2,6,1,19.3333339691162,35.3333320617676,5.33333349227905,60,3,2,208,7
1563,148,2012-03-17,3,6,1,26.3333339691162,32.6666679382324,1,60,3,2,208,71,1,1,0,1,1,9,1480
1564,148,2012-03-17,4,6,1,28.6666660308838,19.6666660308838,0.333333343267441,48.666667
1565,148,2012-03-18,0,0,1,9.33333301544189,32.3333320617676,2,43.6666679382324,3,2,208,71
1566,148,2012-03-18,1,0,1,20,39.6666679382324,0.333333343267441,60,3,2,208,71,1,1,0,1,1,9,14
1567,148,2012-03-18,2,0,1,19.6666660308838,23.3333339691162,17,60,3,2,208,71,1,1,0,1,1,9,148
1568,148,2012-03-18,3,0,1,34,26,0,60,3,2,208,71,1,1,0,1,1,9,1480.891505,1147.10447,9.25180151
1569,148,2012-03-18,4,0,1,36.6666679382324,11,0.333333343267441,48,3,2,208,71,1,1,0,1,1,9,14
1570,148,2012-03-19,1,1,0,12,48,0,60,2,2,208,71,1,1,0,1,1,9,1480.891505,1147.10447,9.25180151
1571,148,2012-03-19,2,1,0,14.3333330154419,41.6666679382324,4,60,3,2,208,71,1,1,0,1,1,9,1480
1572,148,2012-03-19,3,1,0,12.3333330154419,23.6666660308838,24,60,3,2,208,71,1,1,0,1,1,9,148
1573,148,2012-03-19,4,1,0,34.3333320617676,25,0.666666686534882,60,3,2,208,71,1,1,0,1,1,9,14
1574,148,2012-03-20,0,2,0,6,47,1,54,2,2,208,71,1,1,0,1,1,9,1480.891505,1147.10447,9.251801512,
1575,148,2012-03-20,1,2,0,13.3333330154419,35.6666679382324,11,60,3,2,208,71,1,1,0,1,1,9,148
1576,148,2012-03-20,2,2,0,33,27,0,60,3,2,208,71,1,1,0,1,1,9,1480.891505,1147.10447,9.25180151
1577,148,2012-03-20,3,2,0,23.3333339691162,36.3333320617676,0.333333343267441,60,3,2,208,
1578,148,2012-03-20,4,2,0,51,9,0,60,2,2,208,71,1,1,0,1,1,9,1480.891505,1147.10447,9.251801512,
1579,148,2012-03-21,0,3,0,7,42.6666679382324,0.333333343267441,50,3,2,208,71,1,1,0,1,1,9,148
1580,148,2012-03-21,1,3,0,20,30.6666660308838,9.33333301544189,60,3,2,208,71,1,1,0,1,1,9,148
1581,148,2012-03-21,2,3,0,44,9.66666698455811,6.33333349227905,60,3,2,208,71,1,1,0,1,1,9,148
1582,148,2012-03-21,3,3,0,16,28.3333339691162,15.6666669845581,60,3,2,208,71,1,1,0,1,1,9,148
1583,148,2012-03-21,4,3,0,35,17.3333339691162,0.333333343267441,52.6666679382324,3,2,208,
1584,149,2012-03-19,1,1,0,6.33333349227905,37.6666679382324,0.333333343267441,44.333332
1585,149,2012-03-19,2,1,0,28,32,0,60,2,2,208,75,1,1,0,1,1,12,1689.442246,1177.284027,7.598358
1586,149,2012-03-20,0,2,0,15.5,41.5,0.5,57.5,2,2,208,75,1,1,0,1,1,12,1689.442246,1177.284027,7.
1587,149,2012-03-20,1,2,0,25,34.3333320617676,0.666666686534882,60,3,2,208,75,1,1,0,1,1,12,1
1588,149,2012-03-20,2,2,0,35.25,24.5,0.25,60,3,2,208,75,1,1,0,1,1,12,1689.442246,1177.284027,7.
1589,149,2012-03-20,3,2,0,35.6666679382324,24.3333339691162,0,60,3,2,208,75,1,1,0,1,1,12,168
1590,149,2012-03-20,4,2,0,40.3333320617676,19.3333339691162,0.333333343267441,60,3,2,208,
1591,149,2012-03-21,0,3,0,14.5,26.6666660308838,0.5,41.6666679382324,3,2,208,75,1,1,0,1,1,12,
1592,149,2012-03-21,1,3,0,31,22.3333339691162,6.66666650772095,60,3,2,208,75,1,1,0,1,1,12,16
1593,149,2012-03-21,2,3,0,28.8333339691162,31,0.16666667163372,60,3,2,208,75,1,1,0,1,1,12,16
1594,149,2012-03-21,3,3,0,43.3333320617676,16.5,0.16666667163372,60,3,2,208,75,1,1,0,1,1,12,
1595,149,2012-03-21,4,3,0,49.3333320617676,10.6666669845581,0,60,3,2,208,75,1,1,0,1,1,12,168
1596,149,2012-03-22,0,4,0,16,26.6666660308838,0.333333343267441,43,3,2,208,75,1,1,0,1,1,12,1
1597,149,2012-03-22,1,4,0,20,38.6666679382324,1.33333337306976,60,3,2,208,75,1,1,0,1,1,12,16
1598,149,2012-03-22,2,4,0,40.3333320617676,13,6.66666650772095,60,3,2,208,75,1,1,0,1,1,12,16
1599,149,2012-03-22,3,4,0,48,11.75,0.25,60,3,2,208,75,1,1,0,1,1,12,1689.442246,1177.284027,7.5

1600,149,2012-03-22,4,4,0,53,7,0,60,3,2,208,75,1,1,0,1,1,12,1689.442246,1177.284027,7.59835861
1601,149,2012-03-23,0,5,0,13.3333330154419,24.3333339691162,6.66666650772095,44.33333206
1602,149,2012-03-23,1,5,0,17,32.3333320617676,10.6666669845581,60,3,2,208,75,1,1,0,1,1,12,16
1603,149,2012-03-23,2,5,0,45.3333320617676,13.6666669845581,1,60,3,2,208,75,1,1,0,1,1,12,168
1604,149,2012-03-23,3,5,0,48,12,0,60,2,2,208,75,1,1,0,1,1,12,1689.442246,1177.284027,7.5983586
1605,149,2012-03-23,4,5,0,52,8,0,60,3,2,208,75,1,1,0,1,1,12,1689.442246,1177.284027,7.59835861
1606,149,2012-03-24,0,6,1,11,29,3.66666674613953,43.6666679382324,3,2,208,75,1,1,0,1,1,12,16
1607,149,2012-03-24,1,6,1,22.6666660308838,35.6666679382324,1.66666662693024,60,3,2,208,7
1608,149,2012-03-24,2,6,1,45.5,14.5,0,60,2,2,208,75,1,1,0,1,1,12,1689.442246,1177.284027,7.5983
1609,149,2012-03-24,3,6,1,53,7,0,60,3,2,208,75,1,1,0,1,1,12,1689.442246,1177.284027,7.59835861
1610,149,2012-03-24,4,6,1,52.6666679382324,7,0.333333343267441,60,3,2,208,75,1,1,0,1,1,12,16
1611,149,2012-03-25,0,0,1,16.6666660308838,25,1,42.6666679382324,3,2,208,75,1,1,0,1,1,12,168
1612,149,2012-03-25,1,0,1,23.3333339691162,35.6666679382324,1,60,3,2,208,75,1,1,0,1,1,12,168
1613,149,2012-03-25,2,0,1,57,3,0,60,1,2,208,75,1,1,0,1,1,12,1689.442246,1177.284027,7.59835861
1614,149,2012-03-25,3,0,1,52.3333320617676,7.33333349227905,0.333333343267441,60,3,2,208,
1615,149,2012-03-25,4,0,1,52,7.66666650772095,0.333333343267441,60,3,2,208,75,1,1,0,1,1,12,1
1616,149,2012-03-26,2,1,0,42.3333320617676,17,0.666666686534882,60,3,2,208,75,1,1,0,1,1,12,1
1617,149,2012-03-26,3,1,0,47.6666679382324,11.6666669845581,0.666666686534882,60,3,2,208,
1618,149,2012-03-26,4,1,0,52.3333320617676,7,0.666666686534882,60,3,2,208,75,1,1,0,1,1,12,16
1619,150,2012-03-22,0,4,0,45.5,12.5,1,59,2,2,208,78,0,1,0,1,1,9,1689.442246,1177.284027,7.59835
1620,150,2012-03-22,1,4,0,44.6666679382324,15.3333330154419,0,60,3,2,208,78,0,1,0,1,1,9,1689
1621,150,2012-03-22,2,4,0,24.3333339691162,35,0.666666686534882,60,3,2,208,78,0,1,0,1,1,9,16
1622,150,2012-03-22,3,4,0,38,20.5,1.5,60,3,2,208,78,0,1,0,1,1,9,1689.442246,1177.284027,7.59835
1623,150,2012-03-22,4,4,0,39.3333320617676,17.3333339691162,3.33333325386047,60,3,2,208,7
1624,150,2012-03-23,0,5,0,31,16.5,0.5,48,2,2,208,78,0,1,0,1,1,9,1689.442246,1177.284027,7.59835
1625,150,2012-03-23,1,5,0,42,18,0,60,3,2,208,78,0,1,0,1,1,9,1689.442246,1177.284027,7.59835861
1626,150,2012-03-23,2,5,0,27.6666660308838,28.3333339691162,4,60,3,2,208,78,0,1,0,1,1,9,1689
1627,150,2012-03-23,3,5,0,39.5,20.5,0,60,3,2,208,78,0,1,0,1,1,9,1689.442246,1177.284027,7.59835
1628,150,2012-03-23,4,5,0,34.25,25.25,0.5,60,3,2,208,78,0,1,0,1,1,9,1689.442246,1177.284027,7.5
1629,150,2012-03-24,0,6,1,10,23.5,3,36.5,2,2,208,78,0,1,0,1,1,9,1689.442246,1177.284027,7.59835
1630,150,2012-03-24,1,6,1,30,26,4,60,3,2,208,78,0,1,0,1,1,9,1689.442246,1177.284027,7.59835861
1631,150,2012-03-24,2,6,1,30.6666660308838,28,1.33333337306976,60,3,2,208,78,0,1,0,1,1,9,168
1632,150,2012-03-24,3,6,1,42.3333320617676,17.3333339691162,0.333333343267441,60,3,2,208,
1633,150,2012-03-24,4,6,1,41.5999984741211,18.3999996185303,0,60,3,2,208,78,0,1,0,1,1,9,1689
1634,150,2012-03-25,0,0,1,21.25,25.25,3.75,50.25,3,2,208,78,0,1,0,1,1,9,1689.442246,1177.284027
1635,150,2012-03-25,1,0,1,37.3333320617676,22.6666660308838,0,60,3,2,208,78,0,1,0,1,1,9,1689
1636,150,2012-03-25,2,0,1,26,34,0,60,3,2,208,78,0,1,0,1,1,9,1689.442246,1177.284027,7.59835861
1637,150,2012-03-25,3,0,1,41.3333320617676,18.6666660308838,0,60,3,2,208,78,0,1,0,1,1,9,1689
1638,150,2012-03-25,4,0,1,45,14.3333330154419,0.666666686534882,60,3,2,208,78,0,1,0,1,1,9,16
1639,150,2012-03-26,0,1,0,31,23.3333339691162,0.333333343267441,54.6666679382324,3,2,208,
1640,150,2012-03-26,1,1,0,27.4444446563721,29.2222213745117,3.33333325386047,60,3,2,208,7
1641,150,2012-03-26,2,1,0,39.3333320617676,19,1.66666662693024,60,3,2,208,78,0,1,0,1,1,9,168
1642,150,2012-03-26,3,1,0,38.3333320617676,21,0.666666686534882,60,3,2,208,78,0,1,0,1,1,9,16
1643,150,2012-03-26,4,1,0,45,15,0,60,3,2,208,78,0,1,0,1,1,9,1689.442246,1177.284027,7.59835861
1644,150,2012-03-27,0,2,0,15,22,2,39,1,2,208,78,0,1,0,1,1,9,1689.442246,1177.284027,7.59835861
1645,150,2012-03-27,1,2,0,46,13.6666669845581,0.333333343267441,60,3,2,208,78,0,1,0,1,1,9,16
1646,150,2012-03-27,2,2,0,33.8888893127441,25.8888893127441,0.222222223877907,60,3,2,208,
1647,150,2012-03-27,3,2,0,29.2857151031494,30.7142848968506,0,60,3,2,208,78,0,1,0,1,1,9,1689
1648,150,2012-03-27,4,2,0,41.6666679382324,18,0.333333343267441,60,3,2,208,78,0,1,0,1,1,9,16
1649,151,2012-03-17,1,6,1,10,33,4,47,2,2,208,69,0,1,1,1,1,9,1691.551281,1190.550145,8.4333326

1650,151,2012-03-17,2,6,1,5.33333349227905,44.3333320617676,10.3333330154419,60,3,2,208,6
1651,151,2012-03-17,3,6,1,34.4000015258789,24.2000007629395,1.39999997615814,60,3,2,208,6
1652,151,2012-03-17,4,6,1,36.6666679382324,22.6666660308838,0.666666686534882,60,3,2,208,
1653,151,2012-03-27,2,2,0,21.3333339691162,26.3333339691162,1.33333337306976,49,3,2,208,6
1654,151,2012-03-27,3,2,0,25,34.3333320617676,0.666666686534882,60,3,2,208,69,0,1,1,1,1,9,16
1655,151,2012-03-27,4,2,0,16,42,2,60,1,2,208,69,0,1,1,1,1,9,1691.551281,1190.550145,8.43333265
1656,151,2012-03-28,0,3,0,34,22,4,60,1,2,208,69,0,1,1,1,1,9,1691.551281,1190.550145,8.43333265
1657,151,2012-03-28,1,3,0,24,14.5,2.5,41,2,2,208,69,0,1,1,1,1,9,1691.551281,1190.550145,8.43333
1658,151,2012-03-28,2,3,0,20,23,1.33333337306976,44.3333320617676,3,2,208,69,0,1,1,1,1,9,169
1659,151,2012-03-28,3,3,0,17.6666660308838,14.6666669845581,27.6666660308838,60,3,2,208,6
1660,151,2012-03-28,4,3,0,32.5999984741211,26,1.39999997615814,60,3,2,208,69,0,1,1,1,1,9,169
1661,151,2012-03-29,0,4,0,19,16,1,36,1,2,208,69,0,1,1,1,1,9,1691.551281,1190.550145,8.43333265
1662,151,2012-03-29,1,4,0,30,22.1666660308838,7.83333349227905,60,3,2,208,69,0,1,1,1,1,9,169
1663,151,2012-03-29,2,4,0,26.5,26.5,2.33333325386047,55.3333320617676,3,2,208,69,0,1,1,1,1,9,
1664,151,2012-03-29,3,4,0,39,21,0,60,1,2,208,69,0,1,1,1,1,9,1691.551281,1190.550145,8.43333265
1665,151,2012-03-29,4,4,0,34.3333320617676,23.3333339691162,2,59.6666679382324,3,2,208,69
1666,151,2012-03-30,1,5,0,28.6666660308838,24.3333339691162,1,54,3,2,208,69,0,1,1,1,1,9,1691
1667,151,2012-03-30,2,5,0,31,14.6666669845581,1.33333337306976,47,3,2,208,69,0,1,1,1,1,9,169
1668,151,2012-03-30,3,5,0,13,18.3333339691162,11,42.3333320617676,3,2,208,69,0,1,1,1,1,9,169
1669,151,2012-03-30,4,5,0,38.6666679382324,18.6666660308838,2.66666674613953,60,3,2,208,6
1670,152,2012-03-27,0,2,0,37,23,0,60,1,2,208,71,0,1,0,1,1,7,996.2592061,724.3738923,2.6828399
1671,152,2012-03-27,1,2,0,36.6666679382324,22.6666660308838,0.666666686534882,60,3,2,208,
1672,152,2012-03-27,2,2,0,15.6666669845581,41,3.33333325386047,60,3,2,208,71,0,1,0,1,1,7,996
1673,152,2012-03-27,3,2,0,22.6666660308838,36.6666679382324,0.666666686534882,60,3,2,208,
1674,152,2012-03-27,4,2,0,44,15,1,60,3,2,208,71,0,1,0,1,1,7,996.2592061,724.3738923,2.6828399
1675,152,2012-03-28,0,3,0,5,6,0.333333343267441,11.3333330154419,3,2,208,71,0,1,0,1,1,7,996.2
1676,152,2012-03-28,1,3,0,36.6666679382324,21.6666660308838,1.66666662693024,60,3,2,208,7
1677,152,2012-03-28,2,3,0,27.6666660308838,31.6666660308838,0.666666686534882,60,3,2,208,
1678,152,2012-03-28,3,3,0,21.3333339691162,38.6666679382324,0,60,3,2,208,71,0,1,0,1,1,7,996.2
1679,152,2012-03-28,4,3,0,44.6666679382324,15.3333330154419,0,60,3,2,208,71,0,1,0,1,1,7,996.2
1680,152,2012-03-29,0,4,0,10,7,0.333333343267441,17.3333339691162,3,2,208,71,0,1,0,1,1,7,996
1681,152,2012-03-29,1,4,0,27.6666660308838,31.6666660308838,0.666666686534882,60,3,2,208,
1682,152,2012-03-29,2,4,0,25,32.3333320617676,2.66666674613953,60,3,2,208,71,0,1,0,1,1,7,996
1683,152,2012-03-29,3,4,0,28,31.6666660308838,0.333333343267441,60,3,2,208,71,0,1,0,1,1,7,99
1684,152,2012-03-29,4,4,0,39.6666679382324,20,0.333333343267441,60,3,2,208,71,0,1,0,1,1,7,99
1685,152,2012-03-30,0,5,0,16,8,0.333333343267441,24.3333339691162,3,2,208,71,0,1,0,1,1,7,996
1686,152,2012-03-30,1,5,0,25.3333339691162,32.3333320617676,2.33333325386047,60,3,2,208,7
1687,152,2012-03-30,2,5,0,40.6666679382324,18.6666660308838,0.666666686534882,60,3,2,208,
1688,152,2012-03-30,3,5,0,29.6666660308838,29,1.33333337306976,60,3,2,208,71,0,1,0,1,1,7,996
1689,152,2012-03-30,4,5,0,38.3333320617676,20,1.66666662693024,60,3,2,208,71,0,1,0,1,1,7,996
1690,152,2012-03-31,0,6,1,7.33333349227905,8.33333301544189,0.333333343267441,16,3,2,208,
1691,152,2012-03-31,1,6,1,25,32,3,60,3,2,208,71,0,1,0,1,1,7,996.2592061,724.3738923,2.6828399
1692,152,2012-03-31,2,6,1,35,25,0,60,3,2,208,71,0,1,0,1,1,7,996.2592061,724.3738923,2.6828399
1693,152,2012-03-31,3,6,1,8.33333301544189,51.3333320617676,0.333333343267441,60,3,2,208,
1694,152,2012-03-31,4,6,1,46.3333320617676,13.6666669845581,0,60,3,2,208,71,0,1,0,1,1,7,996.2
1695,152,2012-04-01,0,7,1,1,3.66666674613953,0,4.66666650772095,3,2,208,71,0,1,0,1,1,7,996.2
1696,152,2012-04-01,1,7,1,33,27,0,60,3,2,208,71,0,1,0,1,1,7,996.2592061,724.3738923,2.6828399
1697,152,2012-04-01,2,7,1,38.3333320617676,21.6666660308838,0,60,3,2,208,71,0,1,0,1,1,7,996.2
1698,152,2012-04-01,3,7,1,34.3333320617676,22.6666660308838,3,60,3,2,208,71,0,1,0,1,1,7,996.2
1699,152,2012-04-01,4,7,1,37.6666679382324,22.3333339691162,0,60,3,2,208,71,0,1,0,1,1,7,996.2

1700,152,2012-04-02,0,1,0,6.66666650772095,8,0,14.6666669845581,3,2,208,71,0,1,0,1,1,7,996.25
1701,152,2012-04-02,1,1,0,21,35.3333320617676,3.66666674613953,60,3,2,208,71,0,1,0,1,1,7,996
1702,152,2012-04-02,2,1,0,24,33,3,60,3,2,208,71,0,1,0,1,1,7,996.2592061,724.3738923,2.6828399
1703,152,2012-04-02,3,1,0,18.6666660308838,41.3333320617676,0,60,3,2,208,71,0,1,0,1,1,7,996.2
1704,152,2012-04-02,4,1,0,41,19,0,60,3,2,208,71,0,1,0,1,1,7,996.2592061,724.3738923,2.6828399
1705,152,2012-04-03,0,2,0,0,8,0,8,3,2,208,71,0,1,0,1,1,7,996.2592061,724.3738923,2.682839969,7
1706,152,2012-04-03,1,2,0,40.6666679382324,18.6666660308838,0.666666686534882,60,3,2,208,
1707,152,2012-04-03,2,2,0,29.6666660308838,30.3333339691162,0,60,3,2,208,71,0,1,0,1,1,7,996.2
1708,152,2012-04-03,3,2,0,41.6666679382324,18.3333339691162,0,60,3,2,208,71,0,1,0,1,1,7,996.2
1709,152,2012-04-03,4,2,0,50.3333320617676,9.66666698455811,0,60,3,2,208,71,0,1,0,1,1,7,996.2
1710,152,2012-04-04,0,3,0,11.3333330154419,6,0,17.3333339691162,3,2,208,71,0,1,0,1,1,7,996.25
1711,152,2012-04-04,1,3,0,38.6666679382324,20.3333339691162,1,60,3,2,208,71,0,1,0,1,1,7,996.2
1712,152,2012-04-04,2,3,0,44.3333320617676,15,0.666666686534882,60,3,2,208,71,0,1,0,1,1,7,99
1713,152,2012-04-04,3,3,0,40.3333320617676,19.6666660308838,0,60,3,2,208,71,0,1,0,1,1,7,996.2
1714,152,2012-04-04,4,3,0,42.3333320617676,17,0.666666686534882,60,3,2,208,71,0,1,0,1,1,7,99
1715,161,2012-03-28,0,3,0,60,0,0,60,1,2,208,76,1,1,0,1,1,11,1112.162054,1132.545046,3.2345398
1716,161,2012-03-28,1,3,0,43.6666679382324,16,0.333333343267441,60,3,2,208,76,1,1,0,1,1,11,1
1717,161,2012-03-28,2,3,0,26,31,3,60,3,2,208,76,1,1,0,1,1,11,1112.162054,1132.545046,3.234539
1718,161,2012-03-28,3,3,0,27,28.3333339691162,4.66666650772095,60,3,2,208,76,1,1,0,1,1,11,11
1719,161,2012-03-28,4,3,0,46.6666679382324,13,0.333333343267441,60,3,2,208,76,1,1,0,1,1,11,1
1720,161,2012-03-29,0,4,0,29.3333339691162,9.33333301544189,0,38.6666679382324,3,2,208,76
1721,161,2012-03-29,1,4,0,44,16,0,60,3,2,208,76,1,1,0,1,1,11,1112.162054,1132.545046,3.234539
1722,161,2012-03-29,2,4,0,26,32,2,60,3,2,208,76,1,1,0,1,1,11,1112.162054,1132.545046,3.234539
1723,161,2012-03-29,3,4,0,28.6666660308838,29,2.33333325386047,60,3,2,208,76,1,1,0,1,1,11,11
1724,161,2012-03-29,4,4,0,40.3333320617676,19,0.666666686534882,60,3,2,208,76,1,1,0,1,1,11,1
1725,161,2012-03-30,0,5,0,23,9,0.333333343267441,32.3333320617676,3,2,208,76,1,1,0,1,1,11,11
1726,161,2012-03-30,1,5,0,21.3333339691162,38,0.666666686534882,60,3,2,208,76,1,1,0,1,1,11,1
1727,161,2012-03-30,2,5,0,17.6666660308838,42,0.333333343267441,60,3,2,208,76,1,1,0,1,1,11,1
1728,161,2012-03-30,3,5,0,36,22.6666660308838,1.33333337306976,60,3,2,208,76,1,1,0,1,1,11,11
1729,161,2012-03-30,4,5,0,46,14,0,60,3,2,208,76,1,1,0,1,1,11,1112.162054,1132.545046,3.234539
1730,161,2012-03-31,0,6,1,41.3333320617676,12.3333330154419,0.333333343267441,54,3,2,208,
1731,161,2012-03-31,1,6,1,29.3333339691162,26,4.66666650772095,60,3,2,208,76,1,1,0,1,1,11,11
1732,161,2012-03-31,2,6,1,39,21,0,60,3,2,208,76,1,1,0,1,1,11,1112.162054,1132.545046,3.234539
1733,161,2012-03-31,3,6,1,44.3333320617676,15.6666669845581,0,60,3,2,208,76,1,1,0,1,1,11,111
1734,161,2012-03-31,4,6,1,48.6666679382324,11.3333330154419,0,60,3,2,208,76,1,1,0,1,1,11,111
1735,161,2012-04-01,0,7,1,23.3333339691162,10.3333330154419,0,33.6666679382324,3,2,208,76
1736,161,2012-04-01,1,7,1,34,23,3,60,3,2,208,76,1,1,0,1,1,11,1112.162054,1132.545046,3.234539
1737,161,2012-04-01,2,7,1,23,36.6666679382324,0.333333343267441,60,3,2,208,76,1,1,0,1,1,11,1
1738,161,2012-04-01,3,7,1,35.3333320617676,24,0.666666686534882,60,3,2,208,76,1,1,0,1,1,11,1
1739,161,2012-04-01,4,7,1,49.3333320617676,10.6666669845581,0,60,3,2,208,76,1,1,0,1,1,11,111
1740,161,2012-04-02,0,1,0,29.3333339691162,9.66666698455811,0,39,3,2,208,76,1,1,0,1,1,11,111
1741,161,2012-04-02,1,1,0,24.3333339691162,35,0.666666686534882,60,3,2,208,76,1,1,0,1,1,11,1
1742,161,2012-04-02,2,1,0,26,33.3333320617676,0.666666686534882,60,3,2,208,76,1,1,0,1,1,11,1
1743,161,2012-04-02,3,1,0,41.6666679382324,18.3333339691162,0,60,3,2,208,76,1,1,0,1,1,11,111
1744,161,2012-04-02,4,1,0,47.6666679382324,12.3333330154419,0,60,3,2,208,76,1,1,0,1,1,11,111
1745,161,2012-04-03,0,2,0,30.3333339691162,11,0,41.3333320617676,3,2,208,76,1,1,0,1,1,11,111
1746,161,2012-04-03,1,2,0,36.6666679382324,23,0.333333343267441,60,3,2,208,76,1,1,0,1,1,11,1
1747,161,2012-04-03,2,2,0,39,21,0,60,3,2,208,76,1,1,0,1,1,11,1112.162054,1132.545046,3.234539
1748,161,2012-04-03,3,2,0,24.6666660308838,34.6666679382324,0.666666686534882,60,3,2,208,
1749,161,2012-04-03,4,2,0,50.6666679382324,9,0.333333343267441,60,3,2,208,76,1,1,0,1,1,11,11

1750,161,2012-04-04,0,3,0,26,8.66666698455811,0.333333343267441,35,3,2,208,76,1,1,0,1,1,11,1
1751,161,2012-04-04,1,3,0,30.6666660308838,29,0.333333343267441,60,3,2,208,76,1,1,0,1,1,11,1
1752,161,2012-04-04,2,3,0,24,33,3,60,3,2,208,76,1,1,0,1,1,11,1112.162054,1132.545046,3.2345398
1753,161,2012-04-04,3,3,0,25,28.6666660308838,6.33333349227905,60,3,2,208,76,1,1,0,1,1,11,11
1754,161,2012-04-04,4,3,0,38.3333320617676,21,0.666666686534882,60,3,2,208,76,1,1,0,1,1,11,1
1755,189,2012-03-01,2,4,0,19,31,10,60,2,2,209,73,1,1,1,1,1,9,938.5354949,705.9742577,2.5465936
1756,189,2012-03-01,3,4,0,21,33.6666679382324,5.33333349227905,60,3,2,209,73,1,1,1,1,1,9,938
1757,189,2012-03-01,4,4,0,12,43,5,60,3,2,209,73,1,1,1,1,1,9,938.5354949,705.9742577,2.54659369
1758,189,2012-03-18,0,0,1,20,34,3,57,2,2,209,73,1,1,1,1,1,9,938.5354949,705.9742577,2.54659369
1759,189,2012-03-18,1,0,1,17.75,37.5,4.75,60,3,2,209,73,1,1,1,1,1,9,938.5354949,705.9742577,2.5
1760,189,2012-03-18,2,0,1,20,38.3333320617676,1.66666662693024,60,3,2,209,73,1,1,1,1,1,9,938
1761,189,2012-03-18,3,0,1,29.6666660308838,30,0.333333343267441,60,3,2,209,73,1,1,1,1,1,9,93
1762,189,2012-03-18,4,0,1,29,28.6666660308838,2.33333325386047,60,3,2,209,73,1,1,1,1,1,9,938
1763,189,2012-03-19,1,1,0,13.6000003814697,38.7999992370605,4.80000019073486,57.2000007
1764,189,2012-03-19,2,1,0,22,35.7999992370605,2.20000004768372,60,3,2,209,73,1,1,1,1,1,9,938
1765,189,2012-03-19,3,1,0,6.33333349227905,50.6666679382324,3,60,3,2,209,73,1,1,1,1,1,9,938.5
1766,189,2012-03-19,4,1,0,22.3333339691162,31.6666660308838,6,60,3,2,209,73,1,1,1,1,1,9,938.5
1767,189,2012-03-20,0,2,0,10,26,2,38,2,2,209,73,1,1,1,1,1,9,938.5354949,705.9742577,2.54659369
1768,189,2012-03-20,1,2,0,36.6666679382324,23.3333339691162,0,60,3,2,209,73,1,1,1,1,1,9,938.5
1769,189,2012-03-20,2,2,0,26.5,32.75,0.75,60,3,2,209,73,1,1,1,1,1,9,938.5354949,705.9742577,2.5
1770,189,2012-03-20,3,2,0,20.75,36.75,2.5,60,3,2,209,73,1,1,1,1,1,9,938.5354949,705.9742577,2.5
1771,189,2012-03-20,4,2,0,27.3333339691162,27,5.66666650772095,60,3,2,209,73,1,1,1,1,1,9,938
1772,189,2012-03-21,1,3,0,16.3333339691162,39,4.66666650772095,60,3,2,209,73,1,1,1,1,1,9,938
1773,189,2012-03-21,2,3,0,10,46,4,60,3,2,209,73,1,1,1,1,1,9,938.5354949,705.9742577,2.54659369
1774,189,2012-03-21,3,3,0,24.2000007629395,32.5999984741211,3.20000004768372,60,3,2,209,7
1775,189,2012-03-21,4,3,0,31.6000003814697,24.3999996185303,4,60,3,2,209,73,1,1,1,1,1,9,938.5
1776,189,2012-03-22,0,4,0,10,27.3333339691162,8.33333301544189,45.6666679382324,3,2,209,7
1777,189,2012-03-22,1,4,0,12.25,39.5,8.25,60,3,2,209,73,1,1,1,1,1,9,938.5354949,705.9742577,2.5
1778,189,2012-03-22,2,4,0,3.33333325386047,44.3333320617676,12.3333330154419,60,3,2,209,7
1779,189,2012-03-22,3,4,0,7,41,12,60,3,2,209,73,1,1,1,1,1,9,938.5354949,705.9742577,2.54659369
1780,189,2012-03-22,4,4,0,22.75,36,1.25,60,3,2,209,73,1,1,1,1,1,9,938.5354949,705.9742577,2.546
1781,189,2012-03-23,0,5,0,40,17,3,60,1,2,209,73,1,1,1,1,1,9,938.5354949,705.9742577,2.54659369
1782,189,2012-03-23,1,5,0,21.7999992370605,29.7999992370605,8.39999961853027,60,3,2,209,7
1783,189,2012-03-23,2,5,0,14.25,36,9.75,60,3,2,209,73,1,1,1,1,1,9,938.5354949,705.9742577,2.546
1784,189,2012-03-23,3,5,0,18.3333339691162,29,12.6666669845581,60,3,2,209,73,1,1,1,1,1,9,938
1785,189,2012-03-23,4,5,0,34,18,8,60,3,2,209,73,1,1,1,1,1,9,938.5354949,705.9742577,2.54659369
1786,195,2012-03-01,0,4,0,51,9,0,60,1,2,209,67,1,1,1,1,1,9,700.9786762,2605.764446,5.563852927
1787,195,2012-03-01,1,4,0,16.3333339691162,41,2.66666674613953,60,3,2,209,67,1,1,1,1,1,9,700
1788,195,2012-03-01,2,4,0,14,42.3333320617676,3.66666674613953,60,3,2,209,67,1,1,1,1,1,9,700
1789,195,2012-03-02,0,5,0,14,24,3,41,1,2,209,67,1,1,1,1,1,9,700.9786762,2605.764446,5.56385292
1790,195,2012-03-02,1,5,0,15,43.6666679382324,1.33333337306976,60,3,2,209,67,1,1,1,1,1,9,700
1791,195,2012-03-02,2,5,0,13.6666669845581,45,1.33333337306976,60,3,2,209,67,1,1,1,1,1,9,700
1792,195,2012-03-02,3,5,0,10.3333330154419,47.5,2.16666674613953,60,3,2,209,67,1,1,1,1,1,9,70
1793,195,2012-03-02,4,5,0,19,40.6666679382324,0.333333343267441,60,3,2,209,67,1,1,1,1,1,9,70
1794,195,2012-03-03,0,6,1,14,29,0,43,1,2,209,67,1,1,1,1,1,9,700.9786762,2605.764446,5.56385292
1795,195,2012-03-03,1,6,1,4.33333349227905,53.6666679382324,2,60,3,2,209,67,1,1,1,1,1,9,700.9
1796,195,2012-03-03,2,6,1,4.66666650772095,55,0.333333343267441,60,3,2,209,67,1,1,1,1,1,9,70
1797,195,2012-03-03,3,6,1,3.33333325386047,49.3333320617676,7.33333349227905,60,3,2,209,6
1798,195,2012-03-03,4,6,1,32.5,27,0.5,60,3,2,209,67,1,1,1,1,1,9,700.9786762,2605.764446,5.56385
1799,195,2012-03-04,0,0,1,15,34,6,55,1,2,209,67,1,1,1,1,1,9,700.9786762,2605.764446,5.56385292

1800,195,2012-03-04,1,0,1,16.5,30.75,6,53.25,3,2,209,67,1,1,1,1,1,9,700.9786762,2605.764446,5.5
1801,195,2012-03-04,2,0,1,5.25,46,8.75,60,3,2,209,67,1,1,1,1,1,9,700.9786762,2605.764446,5.5638
1802,195,2012-03-04,3,0,1,3.66666674613953,56,0.333333343267441,60,3,2,209,67,1,1,1,1,1,9,70
1803,195,2012-03-04,4,0,1,27.3999996185303,32.2000007629395,0.400000005960464,60,3,2,209,
1804,195,2012-03-05,0,1,0,0,3,0,3,1,2,209,67,1,1,1,1,1,9,700.9786762,2605.764446,5.563852927,7
1805,195,2012-03-05,1,1,0,17,39.3333320617676,3.66666674613953,60,3,2,209,67,1,1,1,1,1,9,700
1806,195,2012-03-05,2,1,0,15,43.4000015258789,1.60000002384186,60,3,2,209,67,1,1,1,1,1,9,700
1807,195,2012-03-05,3,1,0,21.5,29.25,9.25,60,3,2,209,67,1,1,1,1,1,9,700.9786762,2605.764446,5.5
1808,195,2012-03-05,4,1,0,11.3333330154419,42.6666679382324,6,60,3,2,209,67,1,1,1,1,1,9,700.
1809,195,2012-03-06,0,2,0,5.5,23.5,1.5,30.5,2,2,209,67,1,1,1,1,1,9,700.9786762,2605.764446,5.563
1810,195,2012-03-06,1,2,0,10.6666669845581,44.3333320617676,5,60,3,2,209,67,1,1,1,1,1,9,700.
1811,195,2012-03-06,2,2,0,5.33333349227905,27.6666660308838,27,60,3,2,209,67,1,1,1,1,1,9,700
1812,195,2012-03-06,3,2,0,7.40000009536743,37,15.6000003814697,60,3,2,209,67,1,1,1,1,1,9,700
1813,195,2012-03-06,4,2,0,37.6666679382324,21,1.33333337306976,60,3,2,209,67,1,1,1,1,1,9,700
1814,195,2012-03-07,0,3,0,13,21,0,34,1,2,209,67,1,1,1,1,1,9,700.9786762,2605.764446,5.56385292
1815,195,2012-03-07,1,3,0,7.66666650772095,41.3333320617676,11,60,3,2,209,67,1,1,1,1,1,9,700
1816,195,2012-03-07,2,3,0,17.6666660308838,40.6666679382324,1.66666662693024,60,3,2,209,6
1817,195,2012-03-07,3,3,0,21.3333339691162,33.3333320617676,5.33333349227905,60,3,2,209,6
1818,195,2012-03-07,4,3,0,30.8333339691162,27.5,1.66666662693024,60,3,2,209,67,1,1,1,1,1,9,70
1819,205,2012-03-08,0,4,0,5.66666650772095,40,0.333333343267441,46,3,2,209,69,1,1,0,1,1,9,85
1820,205,2012-03-08,1,4,0,16.3333339691162,42.6666679382324,1,60,3,2,209,69,1,1,0,1,1,9,854.
1821,205,2012-03-08,2,4,0,47.3333320617676,11.6666669845581,1,60,3,2,209,69,1,1,0,1,1,9,854.
1822,205,2012-03-08,3,4,0,24,35.3333320617676,0.666666686534882,60,3,2,209,69,1,1,0,1,1,9,85
1823,205,2012-03-08,4,4,0,56,4,0,60,1,2,209,69,1,1,0,1,1,9,854.420022,674.8695037,0,0,0,0,0,0,1.
1824,205,2012-03-24,0,6,1,12,30.5,1.5,44,2,2,209,69,1,1,0,1,1,9,854.420022,674.8695037,0,0,0,0,0
1825,205,2012-03-24,1,6,1,21.6666660308838,38.3333320617676,0,60,3,2,209,69,1,1,0,1,1,9,854.
1826,205,2012-03-24,2,6,1,18.3333339691162,31,10.6666669845581,60,3,2,209,69,1,1,0,1,1,9,854
1827,205,2012-03-24,3,6,1,23,32,5,60,3,2,209,69,1,1,0,1,1,9,854.420022,674.8695037,0,0,0,0,0,0,1
1828,205,2012-03-24,4,6,1,34.5999984741211,25.2000007629395,0.200000002980232,60,3,2,209,
1829,205,2012-03-25,0,0,1,16.3333339691162,43.3333320617676,0.333333343267441,60,3,2,209,
1830,205,2012-03-25,1,0,1,12.6666669845581,46.3333320617676,1,60,3,2,209,69,1,1,0,1,1,9,854.
1831,205,2012-03-25,2,0,1,17,37,6,60,3,2,209,69,1,1,0,1,1,9,854.420022,674.8695037,0,0,0,0,0,0,1
1832,205,2012-03-25,3,0,1,15,37,8,60,3,2,209,69,1,1,0,1,1,9,854.420022,674.8695037,0,0,0,0,0,0,1
1833,205,2012-03-25,4,0,1,6.5,14.5,1,22,2,2,209,69,1,1,0,1,1,9,854.420022,674.8695037,0,0,0,0,0,
1834,205,2012-03-26,0,1,0,26,13,0,39,1,2,209,69,1,1,0,1,1,9,854.420022,674.8695037,0,0,0,0,0,0,1
1835,205,2012-03-26,1,1,0,12,44,4,60,3,2,209,69,1,1,0,1,1,9,854.420022,674.8695037,0,0,0,0,0,0,1
1836,205,2012-03-26,2,1,0,34.3333320617676,25.6666660308838,0,60,3,2,209,69,1,1,0,1,1,9,854.
1837,205,2012-03-26,3,1,0,22,36.6666679382324,1.33333337306976,60,3,2,209,69,1,1,0,1,1,9,854
1838,205,2012-03-26,4,1,0,41.6666679382324,18.3333339691162,0,60,3,2,209,69,1,1,0,1,1,9,854.
1839,205,2012-03-27,0,2,0,9,47,4,60,2,2,209,69,1,1,0,1,1,9,854.420022,674.8695037,0,0,0,0,0,0,1.
1840,205,2012-03-27,1,2,0,27,33,0,60,3,2,209,69,1,1,0,1,1,9,854.420022,674.8695037,0,0,0,0,0,0,1
1841,205,2012-03-27,2,2,0,14.6666669845581,41.3333320617676,4,60,3,2,209,69,1,1,0,1,1,9,854.
1842,205,2012-03-27,3,2,0,23.3333339691162,34.3333320617676,2.33333325386047,60,3,2,209,6
1843,205,2012-03-27,4,2,0,39.6666679382324,20,0.333333343267441,60,3,2,209,69,1,1,0,1,1,9,85
1844,205,2012-03-28,0,3,0,15.3333330154419,41.6666679382324,1.33333337306976,58.333332
1845,205,2012-03-28,1,3,0,20.3333339691162,38,1.66666662693024,60,3,2,209,69,1,1,0,1,1,9,854
1846,205,2012-03-28,2,3,0,11.6666669845581,46.6666679382324,1.66666662693024,60,3,2,209,6
1847,205,2012-03-28,3,3,0,21.3333339691162,38.6666679382324,0,60,3,2,209,69,1,1,0,1,1,9,854.
1848,205,2012-03-28,4,3,0,44,16,0,60,2,2,209,69,1,1,0,1,1,9,854.420022,674.8695037,0,0,0,0,0,0,1
1849,205,2012-03-29,0,4,0,16,21,0.5,37.5,2,2,209,69,1,1,0,1,1,9,854.420022,674.8695037,0,0,0,0,0

1850,205,2012-03-29,1,4,0,27.6666660308838,32.3333320617676,0,60,3,2,209,69,1,1,0,1,1,9,854.4
1851,205,2012-03-29,2,4,0,25.6666660308838,34.3333320617676,0,60,3,2,209,69,1,1,0,1,1,9,854.4
1852,205,2012-03-29,3,4,0,17.6666660308838,41.3333320617676,1,60,3,2,209,69,1,1,0,1,1,9,854.4
1853,205,2012-03-29,4,4,0,30.5,29.25,0.25,60,3,2,209,69,1,1,0,1,1,9,854.420022,674.8695037,0,0,0
1854,205,2012-03-30,0,5,0,17,35.3333320617676,0,52.3333320617676,3,2,209,69,1,1,0,1,1,9,854.4
1855,205,2012-03-30,1,5,0,19.6666660308838,40.3333320617676,0,60,3,2,209,69,1,1,0,1,1,9,854.4
1856,205,2012-03-30,2,5,0,10.3333330154419,49.3333320617676,0.333333343267441,60,3,2,209,
1857,205,2012-03-30,3,5,0,11.6666669845581,46.6666679382324,1.66666662693024,60,3,2,209,6
1858,205,2012-03-30,4,5,0,23.6666660308838,36.3333320617676,0,60,3,2,209,69,1,1,0,1,1,9,854.4
1859,209,2012-03-24,0,6,1,25,33,2,60,1,2,209,77,0,1,0,1,1,9,1343.790738,1043.749955,0,0,0,0,0,0,
1860,209,2012-03-24,1,6,1,31,27.3333339691162,1.66666662693024,60,3,2,209,77,0,1,0,1,1,9,134
1861,209,2012-03-24,2,6,1,34.8333320617676,23.1666660308838,2,60,3,2,209,77,0,1,0,1,1,9,1343
1862,209,2012-03-24,3,6,1,9.66666698455811,34.6666679382324,15.6666669845581,60,3,2,209,7
1863,209,2012-03-24,4,6,1,44.6666679382324,11.3333330154419,0.333333343267441,56.3333320
1864,209,2012-03-25,0,0,1,11,38.5,8,57.5,2,2,209,77,0,1,0,1,1,9,1343.790738,1043.749955,0,0,0,0,
1865,209,2012-03-25,1,0,1,16.3333339691162,8,35.6666679382324,60,3,2,209,77,0,1,0,1,1,9,1343
1866,209,2012-03-25,2,0,1,9.66666698455811,11.6666669845581,38.6666679382324,60,3,2,209,7
1867,209,2012-03-25,3,0,1,8,51.3333320617676,0.666666686534882,60,3,2,209,77,0,1,0,1,1,9,134
1868,209,2012-03-25,4,0,1,28,30.6666660308838,1.33333337306976,60,3,2,209,77,0,1,0,1,1,9,134
1869,209,2012-03-26,0,1,0,10,34.5,9.5,54,2,2,209,77,0,1,0,1,1,9,1343.790738,1043.749955,0,0,0,0,
1870,209,2012-03-26,1,1,0,21,26,13,60,3,2,209,77,0,1,0,1,1,9,1343.790738,1043.749955,0,0,0,0,0,0
1871,209,2012-03-26,2,1,0,22.5,34.5,3,60,2,2,209,77,0,1,0,1,1,9,1343.790738,1043.749955,0,0,0,0,
1872,209,2012-03-26,3,1,0,31.3333339691162,20.3333339691162,8.33333301544189,60,3,2,209,7
1873,209,2012-03-26,4,1,0,49.6666679382324,10.3333330154419,0,60,3,2,209,77,0,1,0,1,1,9,1343
1874,209,2012-03-27,0,2,0,10,26,23,59,2,2,209,77,0,1,0,1,1,9,1343.790738,1043.749955,0,0,0,0,0,0
1875,209,2012-03-27,1,2,0,23,32.3333320617676,4.66666650772095,60,3,2,209,77,0,1,0,1,1,9,134
1876,209,2012-03-27,2,2,0,13,30,17,60,3,2,209,77,0,1,0,1,1,9,1343.790738,1043.749955,0,0,0,0,0,0
1877,209,2012-03-27,3,2,0,26.3333339691162,25.3333339691162,8.33333301544189,60,3,2,209,7
1878,209,2012-03-27,4,2,0,39,20.6666660308838,0.333333343267441,60,3,2,209,77,0,1,0,1,1,9,13
1879,209,2012-03-28,0,3,0,18.5,30,3.5,52,2,2,209,77,0,1,0,1,1,9,1343.790738,1043.749955,0,0,0,0,
1880,209,2012-03-28,1,3,0,16.3333339691162,33.3333320617676,10.3333330154419,60,3,2,209,7
1881,209,2012-03-28,2,3,0,33.3333320617676,25.6666660308838,1,60,3,2,209,77,0,1,0,1,1,9,1343
1882,209,2012-03-28,3,3,0,35.3333320617676,24.6666660308838,0,60,3,2,209,77,0,1,0,1,1,9,1343
1883,209,2012-03-28,4,3,0,47.3333320617676,12.6666669845581,0,60,3,2,209,77,0,1,0,1,1,9,1343
1884,209,2012-03-29,2,4,0,49,11,0,60,1,2,209,77,0,1,0,1,1,9,1343.790738,1043.749955,0,0,0,0,0,0,
1885,209,2012-03-29,3,4,0,36.6666679382324,19.6666660308838,3.66666674613953,60,3,2,209,7
1886,209,2012-03-29,4,4,0,33.3333320617676,22.6666660308838,4,60,3,2,209,77,0,1,0,1,1,9,1343
1887,209,2012-03-30,0,5,0,17,25.5,3,45.5,2,2,209,77,0,1,0,1,1,9,1343.790738,1043.749955,0,0,0,0,
1888,209,2012-03-31,0,6,1,17,26.5,8.5,52,2,2,209,77,0,1,0,1,1,9,1343.790738,1043.749955,0,0,0,0,
1889,209,2012-03-31,1,6,1,23.1666660308838,34.5,2.33333325386047,60,3,2,209,77,0,1,0,1,1,9,13
1890,209,2012-03-31,2,6,1,33.3333320617676,26.3333339691162,0.333333343267441,60,3,2,209,
1891,209,2012-03-31,3,6,1,41.3333320617676,18.6666660308838,0,60,3,2,209,77,0,1,0,1,1,9,1343
1892,209,2012-03-31,4,6,1,33.3333320617676,25.6666660308838,0,59,3,2,209,77,0,1,0,1,1,9,1343
1893,211,2012-03-27,0,2,0,23.3333339691162,18.3333339691162,4.66666650772095,46.3333320
1894,211,2012-03-27,1,2,0,6.33333349227905,37.6666679382324,16,60,3,2,209,68,0,1,0,1,1,9,918
1895,211,2012-03-27,2,2,0,18.5,34,7.5,60,2,2,209,68,0,1,0,1,1,9,918.0759661,1049.940098,4.13370
1896,211,2012-03-27,3,2,0,22,33,5,60,1,2,209,68,0,1,0,1,1,9,918.0759661,1049.940098,4.13370094
1897,211,2012-03-27,4,2,0,28.6666660308838,31.3333339691162,0,60,3,2,209,68,0,1,0,1,1,9,918.0
1898,211,2012-03-28,2,3,0,21,36,3,60,1,2,209,68,0,1,0,1,1,9,918.0759661,1049.940098,4.13370094
1899,211,2012-03-28,3,3,0,33.6666679382324,24.6666660308838,1.66666662693024,60,3,2,209,6

1900,211,2012-03-28,4,3,0,26,33.3333320617676,0.666666686534882,60,3,2,209,68,0,1,0,1,1,9,91
1901,211,2012-03-29,0,4,0,21.3333339691162,16.6666660308838,4.33333349227905,42.333332
1902,211,2012-03-29,1,4,0,31.3333339691162,28,0.666666686534882,60,3,2,209,68,0,1,0,1,1,9,91
1903,211,2012-03-29,2,4,0,33.5,26,0.5,60,2,2,209,68,0,1,0,1,1,9,918.0759661,1049.940098,4.1337
1904,211,2012-03-30,0,5,0,25.3333339691162,15.3333330154419,1.33333337306976,42,3,2,209,6
1905,211,2012-03-30,1,5,0,22.3333339691162,31.3333339691162,6.33333349227905,60,3,2,209,6
1906,211,2012-03-30,2,5,0,19.75,26.75,13.5,60,3,2,209,68,0,1,0,1,1,9,918.0759661,1049.940098,4.
1907,211,2012-03-30,3,5,0,7.5,28.75,23.75,60,3,2,209,68,0,1,0,1,1,9,918.0759661,1049.940098,4.1
1908,211,2012-03-30,4,5,0,31,26.3333339691162,2.66666674613953,60,3,2,209,68,0,1,0,1,1,9,918
1909,211,2012-03-31,0,6,1,5.66666650772095,24,20,49.6666679382324,3,2,209,68,0,1,0,1,1,9,918
1910,211,2012-03-31,1,6,1,17,25.6666660308838,17.3333339691162,60,3,2,209,68,0,1,0,1,1,9,918
1911,211,2012-03-31,2,6,1,29,30.6666660308838,0.333333343267441,60,3,2,209,68,0,1,0,1,1,9,91
1912,211,2012-03-31,3,6,1,11.3999996185303,36.4000015258789,12.1999998092651,60,3,2,209,6
1913,211,2012-03-31,4,6,1,29,30.6666660308838,0.333333343267441,60,3,2,209,68,0,1,0,1,1,9,91
1914,211,2012-04-01,0,0,1,21.6666660308838,15.3333330154419,4.33333349227905,41.333332
1915,211,2012-04-01,1,0,1,5.33333349227905,36.6666679382324,18,60,3,2,209,68,0,1,0,1,1,9,918
1916,211,2012-04-01,2,0,1,11,36.6666679382324,12.3333330154419,60,3,2,209,68,0,1,0,1,1,9,918
1917,211,2012-04-01,3,0,1,8.33333301544189,46,5.66666650772095,60,3,2,209,68,0,1,0,1,1,9,918
1918,211,2012-04-01,4,0,1,24.5,25.5,0.5,50.5,2,2,209,68,0,1,0,1,1,9,918.0759661,1049.940098,4.1
1919,211,2012-04-02,0,1,0,6,26.6666660308838,16.6666660308838,49.3333320617676,3,2,209,68
1920,211,2012-04-02,1,1,0,5,20.3333339691162,34.6666679382324,60,3,2,209,68,0,1,0,1,1,9,918.
1921,211,2012-04-02,2,1,0,24,32,4,60,3,2,209,68,0,1,0,1,1,9,918.0759661,1049.940098,4.1337009
1922,211,2012-04-02,3,1,0,21.6666660308838,34.6666679382324,3.66666674613953,60,3,2,209,6
1923,211,2012-04-02,4,1,0,26,33.6666679382324,0.333333343267441,60,3,2,209,68,0,1,0,1,1,9,91
1924,211,2012-04-03,0,2,0,26.6666660308838,19.3333339691162,0.333333343267441,46.333332
1925,211,2012-04-03,1,2,0,11,34,15,60,3,2,209,68,0,1,0,1,1,9,918.0759661,1049.940098,4.1337009
1926,211,2012-04-03,2,2,0,10,36.6666679382324,13.3333330154419,60,3,2,209,68,0,1,0,1,1,9,918
1927,211,2012-04-03,3,2,0,16,33,11,60,2,2,209,68,0,1,0,1,1,9,918.0759661,1049.940098,4.1337009
1928,211,2012-04-03,4,2,0,13,46.3333320617676,0.666666686534882,60,3,2,209,68,0,1,0,1,1,9,91
1929,224,2012-03-24,0,6,1,11,13,2,26,1,2,209,75,0,0,0,1,1,9,1000.548676,1138.897852,3.4380837
1930,224,2012-03-24,1,6,1,23,26.3333339691162,10.6666669845581,60,3,2,209,75,0,0,0,1,1,9,100
1931,224,2012-03-24,2,6,1,14.3333330154419,19.6666660308838,26,60,3,2,209,75,0,0,0,1,1,9,100
1932,224,2012-03-24,3,6,1,4.66666650772095,35.6666679382324,19.6666660308838,60,3,2,209,7
1933,224,2012-03-24,4,6,1,19.7999992370605,37.7999992370605,2.40000009536743,60,3,2,209,7
1934,224,2012-03-25,1,0,1,35,22.3333339691162,1,58.3333320617676,3,2,209,75,0,0,0,1,1,9,1000
1935,224,2012-03-25,2,0,1,11.6666669845581,42.3333320617676,6,60,3,2,209,75,0,0,0,1,1,9,1000
1936,224,2012-03-25,3,0,1,20.3333339691162,36.6666679382324,3,60,3,2,209,75,0,0,0,1,1,9,1000
1937,224,2012-03-25,4,0,1,1.5,44,14.5,60,3,2,209,75,0,0,0,1,1,9,1000.548676,1138.897852,3.4380
1938,224,2012-03-28,1,3,0,28,28.6666660308838,2,58.6666679382324,3,2,209,75,0,0,0,1,1,9,1000
1939,224,2012-03-28,2,3,0,15,37.3333320617676,7.66666650772095,60,3,2,209,75,0,0,0,1,1,9,100
1940,224,2012-03-28,3,3,0,19,36.5,4.5,60,2,2,209,75,0,0,0,1,1,9,1000.548676,1138.897852,3.4380
1941,224,2012-03-29,0,4,0,0,1,1,2,1,2,209,75,0,0,0,1,1,9,1000.548676,1138.897852,3.438083755,0
1942,224,2012-03-29,1,4,0,35,25,0,60,3,2,209,75,0,0,0,1,1,9,1000.548676,1138.897852,3.4380837
1943,224,2012-03-29,2,4,0,18,34.3333320617676,7.66666650772095,60,3,2,209,75,0,0,0,1,1,9,100
1944,224,2012-03-29,3,4,0,15.5,30.5,14,60,3,2,209,75,0,0,0,1,1,9,1000.548676,1138.897852,3.438
1945,224,2012-03-29,4,4,0,14.75,36.75,8.5,60,3,2,209,75,0,0,0,1,1,9,1000.548676,1138.897852,3.4
1946,224,2012-03-30,0,5,0,35,5,1,41,1,2,209,75,0,0,0,1,1,9,1000.548676,1138.897852,3.43808375
1947,224,2012-03-30,1,5,0,4,23,2,29,2,2,209,75,0,0,0,1,1,9,1000.548676,1138.897852,3.43808375
1948,224,2012-03-30,2,5,0,21.3333339691162,36.3333320617676,2.33333325386047,60,3,2,209,7
1949,224,2012-03-30,3,5,0,28.6666660308838,30.6666660308838,0.666666686534882,60,3,2,209,

1950,224,2012-03-30,4,5,0,8,44.4000015258789,7.59999990463257,60,3,2,209,75,0,0,0,1,1,9,1000
1951,224,2012-03-31,1,6,1,42.5,17.5,0,60,2,2,209,75,0,0,0,1,1,9,1000.548676,1138.897852,3.4380
1952,224,2012-03-31,2,6,1,14.6000003814697,35.2000007629395,5,54.7999992370605,3,2,209,75
1953,224,2012-03-31,3,6,1,18.8333339691162,38.6666679382324,2.5,60,3,2,209,75,0,0,0,1,1,9,10
1954,224,2012-03-31,4,6,1,2.66666674613953,42.6666679382324,14.6666669845581,60,3,2,209,7
1955,225,2012-03-25,0,0,1,18,42,0,60,1,2,209,73,0,0,1,1,1,9,899.3757817,1123.12525,4.02497611
1956,225,2012-03-25,1,0,1,5.5,54.5,0,60,2,2,209,73,0,0,1,1,1,9,899.3757817,1123.12525,4.0249761
1957,225,2012-03-26,0,1,0,57.5,1,0,58.5,2,2,209,73,0,0,1,1,1,9,899.3757817,1123.12525,4.0249761
1958,225,2012-03-26,1,1,0,11,44.75,4.25,60,3,2,209,73,0,0,1,1,1,9,899.3757817,1123.12525,4.024
1959,225,2012-03-26,2,1,0,29,30.7999992370605,0.200000002980232,60,3,2,209,73,0,0,1,1,1,9,89
1960,225,2012-03-26,3,1,0,21.6666660308838,35.6666679382324,2.66666674613953,60,3,2,209,7
1961,225,2012-03-26,4,1,0,29.6666660308838,30.3333339691162,0,60,3,2,209,73,0,0,1,1,1,9,899.
1962,225,2012-03-27,0,2,0,38,16,0.666666686534882,54.6666679382324,3,2,209,73,0,0,1,1,1,9,89
1963,225,2012-03-27,1,2,0,12.6666669845581,47,0.333333343267441,60,3,2,209,73,0,0,1,1,1,9,89
1964,225,2012-03-27,2,2,0,13.5,45.5,1,60,3,2,209,73,0,0,1,1,1,9,899.3757817,1123.12525,4.02497
1965,225,2012-03-27,3,2,0,16.25,43.5,0.25,60,3,2,209,73,0,0,1,1,1,9,899.3757817,1123.12525,4.02
1966,225,2012-03-27,4,2,0,25,33,2,60,3,2,209,73,0,0,1,1,1,9,899.3757817,1123.12525,4.02497611
1967,225,2012-03-28,0,3,0,16,19,1,36,1,2,209,73,0,0,1,1,1,9,899.3757817,1123.12525,4.02497611
1968,225,2012-03-28,1,3,0,15.6666669845581,42.6666679382324,1.66666662693024,60,3,2,209,7
1969,225,2012-03-28,2,3,0,11.3333330154419,46,2.66666674613953,60,3,2,209,73,0,0,1,1,1,9,899
1970,225,2012-03-28,3,3,0,26.2000007629395,32.5999984741211,1.20000004768372,60,3,2,209,7
1971,225,2012-03-28,4,3,0,22.6000003814697,36.5999984741211,0.800000011920929,60,3,2,209,
1972,225,2012-03-29,0,4,0,9,30,0,39,1,2,209,73,0,0,1,1,1,9,899.3757817,1123.12525,4.024976113,
1973,225,2012-03-29,1,4,0,16.6666660308838,39.1666679382324,2.5,58.3333320617676,3,2,209,
1974,225,2012-03-29,2,4,0,40.3333320617676,19.6666660308838,0,60,3,2,209,73,0,0,1,1,1,9,899.
1975,225,2012-03-29,3,4,0,22.3333339691162,35.6666679382324,2,60,3,2,209,73,0,0,1,1,1,9,899.
1976,225,2012-03-29,4,4,0,47.5,12.5,0,60,3,2,209,73,0,0,1,1,1,9,899.3757817,1123.12525,4.02497
1977,225,2012-03-30,0,5,0,13.5,25,0,38.5,2,2,209,73,0,0,1,1,1,9,899.3757817,1123.12525,4.02497
1978,225,2012-03-30,1,5,0,29.3333339691162,29,1.66666662693024,60,3,2,209,73,0,0,1,1,1,9,899
1979,225,2012-03-30,2,5,0,13.8333330154419,44.5,1.66666662693024,60,3,2,209,73,0,0,1,1,1,9,8
1980,225,2012-03-30,3,5,0,26,33,1,60,3,2,209,73,0,0,1,1,1,9,899.3757817,1123.12525,4.02497611
1981,225,2012-03-30,4,5,0,32.6666679382324,27,0.333333343267441,60,3,2,209,73,0,0,1,1,1,9,89
1982,225,2012-03-31,3,6,1,7,50,3,60,3,2,209,73,0,0,1,1,1,9,899.3757817,1123.12525,4.024976113,
1983,225,2012-03-31,4,6,1,40.6666679382324,19,0.333333343267441,60,3,2,209,73,0,0,1,1,1,9,89
1984,228,2012-03-24,0,6,1,19.5,15.5,2,37,2,2,209,68,1,1,0,1,1,9,908.46098,986.9408127,5.307884
1985,228,2012-03-24,1,6,1,32.75,27.25,0,60,3,2,209,68,1,1,0,1,1,9,908.46098,986.9408127,5.3078
1986,228,2012-03-24,2,6,1,26.6666660308838,32.6666679382324,0.666666686534882,60,3,2,209,
1987,228,2012-03-24,3,6,1,14.3333330154419,43.6666679382324,2,60,3,2,209,68,1,1,0,1,1,9,908.
1988,228,2012-03-24,4,6,1,37.75,22.25,0,60,3,2,209,68,1,1,0,1,1,9,908.46098,986.9408127,5.3078
1989,228,2012-03-25,1,0,1,19.6000003814697,37.2000007629395,2.59999990463257,59.400001
1990,228,2012-03-25,2,0,1,27.25,32.5,0.25,60,3,2,209,68,1,1,0,1,1,9,908.46098,986.9408127,5.307
1991,228,2012-03-25,3,0,1,27.3333339691162,28.6666660308838,4,60,3,2,209,68,1,1,0,1,1,9,908.
1992,228,2012-03-25,4,0,1,32,27,1,60,3,2,209,68,1,1,0,1,1,9,908.46098,986.9408127,5.307884048,
1993,228,2012-03-26,1,1,0,17.3333339691162,27.6666660308838,0.333333343267441,45.333332
1994,228,2012-03-26,2,1,0,15.6000003814697,42,2.40000009536743,60,3,2,209,68,1,1,0,1,1,9,908
1995,228,2012-03-26,3,1,0,21.3999996185303,25,13.6000003814697,60,3,2,209,68,1,1,0,1,1,9,908
1996,228,2012-03-26,4,1,0,33.3333320617676,26.3333339691162,0.333333343267441,60,3,2,209,
1997,228,2012-03-27,1,2,0,11,36.6666679382324,2.33333325386047,50,3,2,209,68,1,1,0,1,1,9,908
1998,228,2012-03-27,2,2,0,23,36.6666679382324,0.333333343267441,60,3,2,209,68,1,1,0,1,1,9,90
1999,228,2012-03-27,3,2,0,25.25,32.75,2,60,3,2,209,68,1,1,0,1,1,9,908.46098,986.9408127,5.3078

2000,228,2012-03-27,4,2,0,31.3999996185303,28.2000007629395,0.400000005960464,60,3,2,209,
2001,228,2012-03-28,0,3,0,27.5,13.5,0.5,41.5,2,2,209,68,1,1,0,1,1,9,908.46098,986.9408127,5.3078
2002,228,2012-03-28,1,3,0,32,28,0,60,3,2,209,68,1,1,0,1,1,9,908.46098,986.9408127,5.307884048,
2003,228,2012-03-28,2,3,0,29,30.3333339691162,0.666666686534882,60,3,2,209,68,1,1,0,1,1,9,90
2004,228,2012-03-28,3,3,0,12.6666669845581,46.3333320617676,1,60,3,2,209,68,1,1,0,1,1,9,908.4
2005,228,2012-03-28,4,3,0,14.75,22.5,18,55.25,3,2,209,68,1,1,0,1,1,9,908.46098,986.9408127,5.30
2006,228,2012-03-31,0,6,1,60,0,0,60,1,2,209,68,1,1,0,1,1,9,908.46098,986.9408127,5.307884048,0
2007,228,2012-03-31,1,6,1,8.5,5.5,1.5,15.5,2,2,209,68,1,1,0,1,1,9,908.46098,986.9408127,5.307884
2008,228,2012-03-31,2,6,1,37,22.6666660308838,0.333333343267441,60,3,2,209,68,1,1,0,1,1,9,90
2009,228,2012-03-31,3,6,1,20.3333339691162,39,0.666666686534882,60,3,2,209,68,1,1,0,1,1,9,90
2010,228,2012-03-31,4,6,1,15.5,42,2.5,60,2,2,209,68,1,1,0,1,1,9,908.46098,986.9408127,5.307884(
2011,230,2012-03-28,0,3,0,60,0,0,60,1,2,209,68,0,1,0,1,1,12,908.46098,986.9408127,5.307884048,
2012,230,2012-03-29,0,4,0,21,20,0,41,1,2,209,68,0,1,0,1,1,12,908.46098,986.9408127,5.30788404
2013,230,2012-03-29,1,4,0,32.5,25.5,2,60,3,2,209,68,0,1,0,1,1,12,908.46098,986.9408127,5.307884
2014,230,2012-03-29,2,4,0,25.3333339691162,31.6666660308838,3,60,3,2,209,68,0,1,0,1,1,12,908
2015,230,2012-03-29,3,4,0,10.3333330154419,45.6666679382324,4,60,3,2,209,68,0,1,0,1,1,12,908
2016,230,2012-03-29,4,4,0,22.3333339691162,31,6.66666650772095,60,3,2,209,68,0,1,0,1,1,12,90
2017,230,2012-03-30,0,5,0,28,13,1,42,1,2,209,68,0,1,0,1,1,12,908.46098,986.9408127,5.30788404
2018,230,2012-03-30,1,5,0,19.3333339691162,36.6666679382324,4,60,3,2,209,68,0,1,0,1,1,12,908
2019,230,2012-03-30,2,5,0,26.8333339691162,32.6666679382324,0.5,60,3,2,209,68,0,1,0,1,1,12,9(
2020,230,2012-03-30,3,5,0,18,35.3333320617676,6.66666650772095,60,3,2,209,68,0,1,0,1,1,12,90
2021,230,2012-03-30,4,5,0,27.3333339691162,30.3333339691162,2.33333325386047,60,3,2,209,6
2022,230,2012-03-31,1,6,1,13.5,23,12.5,49,2,2,209,68,0,1,0,1,1,12,908.46098,986.9408127,5.30788
2023,230,2012-03-31,2,6,1,13,44.6666679382324,2.33333325386047,60,3,2,209,68,0,1,0,1,1,12,90
2024,230,2012-03-31,3,6,1,34.6666679382324,17.1666660308838,8.16666698455811,60,3,2,209,6
2025,230,2012-03-31,4,6,1,28.7999992370605,30,1.20000004768372,60,3,2,209,68,0,1,0,1,1,12,90
2026,230,2012-04-01,1,0,1,33.5,19,1,53.5,2,2,209,68,0,1,0,1,1,12,908.46098,986.9408127,5.307884
2027,230,2012-04-01,2,0,1,29.6666660308838,30,0.333333343267441,60,3,2,209,68,0,1,0,1,1,12,9
2028,230,2012-04-01,3,0,1,22,35,3,60,3,2,209,68,0,1,0,1,1,12,908.46098,986.9408127,5.30788404
2029,230,2012-04-01,4,0,1,26.75,28.5,4.75,60,3,2,209,68,0,1,0,1,1,12,908.46098,986.9408127,5.30
2030,231,2012-03-20,1,2,0,22,17,1.66666662693024,40.6666679382324,3,2,209,81,0,1,0,1,1,12,93
2031,231,2012-03-20,2,2,0,18.3333339691162,41,0.666666686534882,60,3,2,209,81,0,1,0,1,1,12,9
2032,231,2012-03-20,3,2,0,36.8333320617676,21.6666660308838,1.5,60,3,2,209,81,0,1,0,1,1,12,93
2033,231,2012-03-20,4,2,0,43.25,16.5,0.25,60,3,2,209,81,0,1,0,1,1,12,930.7987446,991.1770082,4.
2034,231,2012-03-21,4,3,0,33,14,13,60,2,2,209,81,0,1,0,1,1,12,930.7987446,991.1770082,4.872394
2035,231,2012-03-27,1,2,0,9.66666698455811,33,15.6666669845581,58.3333320617676,3,2,209,8
2036,231,2012-03-27,2,2,0,44.5,14,1.5,60,3,2,209,81,0,1,0,1,1,12,930.7987446,991.1770082,4.8723
2037,231,2012-03-27,3,2,0,25,31,4,60,3,2,209,81,0,1,0,1,1,12,930.7987446,991.1770082,4.8723948
2038,231,2012-03-27,4,2,0,24,32.3333320617676,3.66666674613953,60,3,2,209,81,0,1,0,1,1,12,93
2039,231,2012-03-28,1,3,0,32,5.5,0.5,38,2,2,209,81,0,1,0,1,1,12,930.7987446,991.1770082,4.87239
2040,231,2012-03-28,2,3,0,26.6000003814697,27,6.40000009536743,60,3,2,209,81,0,1,0,1,1,12,93
2041,231,2012-03-28,3,3,0,33.1666679382324,24.5,2.33333325386047,60,3,2,209,81,0,1,0,1,1,12,9
2042,231,2012-03-28,4,3,0,45,11.5,3.5,60,3,2,209,81,0,1,0,1,1,12,930.7987446,991.1770082,4.8723
2043,231,2012-03-29,0,4,0,14,17,1,32,1,2,209,81,0,1,0,1,1,12,930.7987446,991.1770082,4.8723948
2044,231,2012-03-29,1,4,0,19.6666660308838,36,4.33333349227905,60,3,2,209,81,0,1,0,1,1,12,93
2045,231,2012-03-29,2,4,0,30.3333339691162,28.6666660308838,1,60,3,2,209,81,0,1,0,1,1,12,930
2046,231,2012-03-29,3,4,0,43.6666679382324,16.3333339691162,0,60,3,2,209,81,0,1,0,1,1,12,930
2047,231,2012-03-29,4,4,0,44.4000015258789,14.3999996185303,1.20000004768372,60,3,2,209,8
2048,231,2012-03-30,0,5,0,0,7,1,8,1,2,209,81,0,1,0,1,1,12,930.7987446,991.1770082,4.87239487,0
2049,231,2012-03-30,1,5,0,17,30,13,60,3,2,209,81,0,1,0,1,1,12,930.7987446,991.1770082,4.872394

2050,231,2012-03-30,2,5,0,16.3999996185303,33.2000007629395,4.19999980926514,53.79999923
2051,231,2012-03-30,3,5,0,36.3333320617676,22.3333339691162,1.33333337306976,60,3,2,209,8
2052,231,2012-03-30,4,5,0,40.6666679382324,16.6666660308838,2.66666674613953,60,3,2,209,8
2053,231,2012-04-01,0,0,1,35,25,0,60,1,2,209,81,0,1,0,1,1,12,930.7987446,991.1770082,4.8723948
2054,231,2012-04-01,1,0,1,31,27,2,60,1,2,209,81,0,1,0,1,1,12,930.7987446,991.1770082,4.8723948
2055,231,2012-04-02,0,1,0,0,5,1,6,1,2,209,81,0,1,0,1,1,12,930.7987446,991.1770082,4.87239487,0
2056,231,2012-04-02,1,1,0,23.6000003814697,30.6000003814697,5.80000019073486,60,3,2,209,8
2057,231,2012-04-02,2,1,0,36.3333320617676,23,0.666666686534882,60,3,2,209,81,0,1,0,1,1,12,9
2058,231,2012-04-02,3,1,0,46.3333320617676,13.6666669845581,0,60,3,2,209,81,0,1,0,1,1,12,930
2059,231,2012-04-02,4,1,0,51.6666679382324,8.33333301544189,0,60,3,2,209,81,0,1,0,1,1,12,930
2060,232,2012-04-11,0,3,0,52,8,0,60,1,2,209,66,1,1,0,1,1,12,926.2617828,991.1770082,5.1179637
2061,232,2012-04-11,1,3,0,33.6666679382324,25.6666660308838,0.666666686534882,60,3,2,209,
2062,232,2012-04-11,2,3,0,22.6666660308838,36.6666679382324,0.666666686534882,60,3,2,209,
2063,232,2012-04-11,3,3,0,38.6666679382324,21,0.333333343267441,60,3,2,209,66,1,1,0,1,1,12,9
2064,232,2012-04-11,4,3,0,32,27,1,60,3,2,209,66,1,1,0,1,1,12,926.2617828,991.1770082,5.1179637
2065,232,2012-04-12,0,4,0,8.33333301544189,11,2,21.3333339691162,3,2,209,66,1,1,0,1,1,12,926
2066,232,2012-04-12,1,4,0,10.6666669845581,42.6666679382324,6.66666650772095,60,3,2,209,6
2067,232,2012-04-12,2,4,0,28.6666660308838,25.6666660308838,5.66666650772095,60,3,2,209,6
2068,232,2012-04-12,3,4,0,9.66666698455811,44.6666679382324,5.66666650772095,60,3,2,209,6
2069,232,2012-04-12,4,4,0,27,32.3333320617676,0.666666686534882,60,3,2,209,66,1,1,0,1,1,12,9
2070,232,2012-04-13,0,5,0,13.6666669845581,6.66666650772095,0,20.3333339691162,3,2,209,66
2071,232,2012-04-13,1,5,0,26,34,0,60,3,2,209,66,1,1,0,1,1,12,926.2617828,991.1770082,5.1179637
2072,232,2012-04-13,2,5,0,16.6666660308838,40,3.33333325386047,60,3,2,209,66,1,1,0,1,1,12,92
2073,232,2012-04-13,3,5,0,14.3333330154419,26,19.6666660308838,60,3,2,209,66,1,1,0,1,1,12,92
2074,232,2012-04-13,4,5,0,19.6666660308838,39.6666679382324,0.666666686534882,60,3,2,209,
2075,232,2012-04-14,1,6,1,7.33333349227905,8,0.333333343267441,15.6666669845581,3,2,209,6
2076,232,2012-04-14,2,6,1,12.3333330154419,44.3333320617676,3.33333325386047,60,3,2,209,6
2077,232,2012-04-14,3,6,1,16,39.3333320617676,4.66666650772095,60,3,2,209,66,1,1,0,1,1,12,92
2078,232,2012-04-14,4,6,1,31,28,1,60,3,2,209,66,1,1,0,1,1,12,926.2617828,991.1770082,5.1179637
2079,232,2012-04-15,0,7,1,9.33333301544189,5.66666650772095,0.333333343267441,15.333333
2080,232,2012-04-15,1,7,1,16.3333339691162,29,14.6666669845581,60,3,2,209,66,1,1,0,1,1,12,92
2081,232,2012-04-15,2,7,1,40.6666679382324,12,7.33333349227905,60,3,2,209,66,1,1,0,1,1,12,92
2082,232,2012-04-15,3,7,1,40,16,4,60,3,2,209,66,1,1,0,1,1,12,926.2617828,991.1770082,5.1179637
2083,232,2012-04-15,4,7,1,36,22.6666660308838,1.33333337306976,60,3,2,209,66,1,1,0,1,1,12,92
2084,232,2012-04-16,0,1,0,8.33333301544189,4,0,12.3333330154419,3,2,209,66,1,1,0,1,1,12,926.2
2085,232,2012-04-16,1,1,0,21.3333339691162,37.3333320617676,1.33333337306976,60,3,2,209,6
2086,232,2012-04-16,2,1,0,18,35.6666679382324,6.33333349227905,60,3,2,209,66,1,1,0,1,1,12,92
2087,232,2012-04-16,3,1,0,13,39,8,60,3,2,209,66,1,1,0,1,1,12,926.2617828,991.1770082,5.1179637
2088,232,2012-04-16,4,1,0,35,25,0,60,3,2,209,66,1,1,0,1,1,12,926.2617828,991.1770082,5.1179637
2089,232,2012-04-17,0,2,0,9.66666698455811,10.6666669845581,0,20.3333339691162,3,2,209,66
2090,232,2012-04-17,1,2,0,15.3333330154419,43.3333320617676,1.33333337306976,60,3,2,209,6
2091,232,2012-04-17,2,2,0,17,34,9,60,3,2,209,66,1,1,0,1,1,12,926.2617828,991.1770082,5.1179637
2092,232,2012-04-17,3,2,0,29.6666660308838,25.3333339691162,5,60,3,2,209,66,1,1,0,1,1,12,926
2093,232,2012-04-17,4,2,0,38.3333320617676,21.3333339691162,0.333333343267441,60,3,2,209,
2094,232,2012-04-18,0,3,0,6.66666650772095,4.33333349227905,0.333333343267441,11.333333
2095,232,2012-04-18,1,3,0,25.3333339691162,30.3333339691162,4.33333349227905,60,3,2,209,6
2096,232,2012-04-18,2,3,0,8.66666698455811,50,1.33333337306976,60,3,2,209,66,1,1,0,1,1,12,92
2097,232,2012-04-18,3,3,0,10,46.3333320617676,3.66666674613953,60,3,2,209,66,1,1,0,1,1,12,92
2098,232,2012-04-18,4,3,0,35,23.3333339691162,1.66666662693024,60,3,2,209,66,1,1,0,1,1,12,92
2099,232,2012-04-19,0,4,0,6,8.66666698455811,0,14.6666669845581,3,2,209,66,1,1,0,1,1,12,926.2

2100,232,2012-04-19,1,4,0,16.3333339691162,37,6.66666650772095,60,3,2,209,66,1,1,0,1,1,12,92
2101,232,2012-04-19,2,4,0,26.6666660308838,22.6666660308838,10.6666669845581,60,3,2,209,6
2102,232,2012-04-19,3,4,0,50.3333320617676,9,0.666666686534882,60,3,2,209,66,1,1,0,1,1,12,92
2103,232,2012-04-19,4,4,0,46.6666679382324,10,3.33333325386047,60,3,2,209,66,1,1,0,1,1,12,92
2104,362,2012-02-21,1,2,0,13.3333330154419,19.6666660308838,1.33333337306976,34.3333320
2105,362,2012-02-21,2,2,0,26.6666660308838,33.3333320617676,0,60,3,2,210,74,1,1,0,1,1,9,2927
2106,362,2012-02-21,3,2,0,22.3333339691162,36.3333320617676,1.33333337306976,60,3,2,210,7
2107,362,2012-02-21,4,2,0,26.3333339691162,32.3333320617676,1.33333337306976,60,3,2,210,7
2108,362,2012-02-22,0,3,0,0,0.333333343267441,0.333333343267441,0.666666686534882,3,2,21
2109,362,2012-02-22,1,3,0,22.3333339691162,32.6666679382324,5,60,3,2,210,74,1,1,0,1,1,9,2927
2110,362,2012-02-22,2,3,0,51.3333320617676,7,1.66666662693024,60,3,2,210,74,1,1,0,1,1,9,2927
2111,362,2012-02-22,3,3,0,17.3333339691162,37,5.66666650772095,60,3,2,210,74,1,1,0,1,1,9,292
2112,362,2012-02-22,4,3,0,32.3333320617676,27.6666660308838,0,60,3,2,210,74,1,1,0,1,1,9,2927
2113,362,2012-02-23,0,4,0,8,11.6666669845581,1,20.6666660308838,3,2,210,74,1,1,0,1,1,9,2927.
2114,362,2012-02-23,1,4,0,19.6666660308838,38.6666679382324,1.66666662693024,60,3,2,210,7
2115,362,2012-02-23,2,4,0,33.3333320617676,24,2.66666674613953,60,3,2,210,74,1,1,0,1,1,9,292
2116,362,2012-02-23,3,4,0,24.3333339691162,25.3333339691162,10.3333330154419,60,3,2,210,7
2117,362,2012-02-23,4,4,0,23,33,4,60,3,2,210,74,1,1,0,1,1,9,2927.93979,3071.899213,3.789943871
2118,362,2012-02-24,0,5,0,28.6666660308838,2.66666674613953,0.333333343267441,31.66666
2119,362,2012-02-24,1,5,0,9,48.6666679382324,2.33333325386047,60,3,2,210,74,1,1,0,1,1,9,2927
2120,362,2012-02-24,2,5,0,26.6666660308838,33,0.333333343267441,60,3,2,210,74,1,1,0,1,1,9,29
2121,362,2012-02-24,3,5,0,16,43.6666679382324,0.333333343267441,60,3,2,210,74,1,1,0,1,1,9,29
2122,362,2012-02-24,4,5,0,35.3333320617676,24.3333339691162,0.333333343267441,60,3,2,210,
2123,362,2012-02-25,0,6,1,0.333333343267441,0,0,0.333333343267441,3,2,210,74,1,1,0,1,1,9,292
2124,362,2012-02-25,1,6,1,21.6666660308838,18.6666660308838,1,41.3333320617676,3,2,210,74
2125,362,2012-02-25,2,6,1,28,28,4,60,3,2,210,74,1,1,0,1,1,9,2927.93979,3071.899213,3.789943871
2126,362,2012-02-25,3,6,1,20,28.6666660308838,11.3333330154419,60,3,2,210,74,1,1,0,1,1,9,292
2127,362,2012-02-25,4,6,1,40.6666679382324,18.6666660308838,0.666666686534882,60,3,2,210,
2128,362,2012-02-26,0,7,1,9,5,0.666666686534882,14.6666669845581,3,2,210,74,1,1,0,1,1,9,2927
2129,362,2012-02-26,1,7,1,14.3333330154419,45,0.666666686534882,60,3,2,210,74,1,1,0,1,1,9,29
2130,362,2012-02-26,2,7,1,29.3333339691162,30.6666660308838,0,60,3,2,210,74,1,1,0,1,1,9,2927
2131,362,2012-02-26,3,7,1,13.3333330154419,33.3333320617676,13.3333330154419,60,3,2,210,7
2132,362,2012-02-26,4,7,1,36.6666679382324,23.3333339691162,0,60,3,2,210,74,1,1,0,1,1,9,2927
2133,362,2012-02-27,0,1,0,7.66666650772095,14.3333330154419,0,22,3,2,210,74,1,1,0,1,1,9,2927
2134,362,2012-02-27,1,1,0,18.3333339691162,38.3333320617676,3.33333325386047,60,3,2,210,7
2135,362,2012-02-27,2,1,0,28,29,3,60,3,2,210,74,1,1,0,1,1,9,2927.93979,3071.899213,3.789943871
2136,362,2012-02-27,3,1,0,18,33.3333320617676,8.66666698455811,60,3,2,210,74,1,1,0,1,1,9,292
2137,362,2012-02-27,4,1,0,38.3333320617676,21.6666660308838,0,60,3,2,210,74,1,1,0,1,1,9,2927
2138,362,2012-02-28,0,2,0,10,8.66666698455811,3.33333325386047,22,3,2,210,74,1,1,0,1,1,9,292
2139,362,2012-02-28,1,2,0,37,19.3333339691162,3.66666674613953,60,3,2,210,74,1,1,0,1,1,9,292
2140,362,2012-02-28,2,2,0,34.6666679382324,19,6.33333349227905,60,3,2,210,74,1,1,0,1,1,9,292
2141,362,2012-02-28,3,2,0,28.3333339691162,24.3333339691162,7.33333349227905,60,3,2,210,7
2142,362,2012-02-28,4,2,0,42,18,0,60,3,2,210,74,1,1,0,1,1,9,2927.93979,3071.899213,3.789943871
2143,363,2012-02-25,0,6,1,55,4,1,60,1,2,210,77,0,1,0,1,1,9,2798.385313,5615.175703,2.66571760
2144,363,2012-02-25,1,6,1,43,14,3,60,3,2,210,77,0,1,0,1,1,9,2798.385313,5615.175703,2.6657176
2145,363,2012-02-25,2,6,1,34.6666679382324,23.6666660308838,1.66666662693024,60,3,2,210,7
2146,363,2012-02-25,3,6,1,25.3333339691162,26,8.66666698455811,60,3,2,210,77,0,1,0,1,1,9,279
2147,363,2012-02-25,4,6,1,47,11.3333330154419,1.66666662693024,60,3,2,210,77,0,1,0,1,1,9,279
2148,363,2012-02-26,1,7,1,35.3333320617676,12,0.666666686534882,48,3,2,210,77,0,1,0,1,1,9,27
2149,363,2012-02-26,2,7,1,46.3333320617676,12,1.66666662693024,60,3,2,210,77,0,1,0,1,1,9,279

2150,363,2012-02-26,3,7,1,31.3333339691162,19.3333339691162,9.33333301544189,60,3,2,210,7
2151,363,2012-02-26,4,7,1,40.6666679382324,13.3333330154419,6,60,3,2,210,77,0,1,0,1,1,9,2798
2152,363,2012-02-27,0,1,0,1.33333337306976,8.66666698455811,4.33333349227905,14.3333330
2153,363,2012-02-27,1,1,0,26,29.3333339691162,4.66666650772095,60,3,2,210,77,0,1,0,1,1,9,279
2154,363,2012-02-27,2,1,0,32.6666679382324,25.3333339691162,2,60,3,2,210,77,0,1,0,1,1,9,2798
2155,363,2012-02-27,3,1,0,11,48.3333320617676,0.666666686534882,60,3,2,210,77,0,1,0,1,1,9,27
2156,363,2012-02-27,4,1,0,42,16.6666660308838,1.33333337306976,60,3,2,210,77,0,1,0,1,1,9,279
2157,363,2012-02-28,0,2,0,3.66666674613953,13,0.333333343267441,17,3,2,210,77,0,1,0,1,1,9,27
2158,363,2012-02-28,1,2,0,32.6666679382324,26.3333339691162,1,60,3,2,210,77,0,1,0,1,1,9,2798
2159,363,2012-02-28,2,2,0,24,33.3333320617676,2.66666674613953,60,3,2,210,77,0,1,0,1,1,9,279
2160,363,2012-02-28,3,2,0,29.3333339691162,25.6666660308838,5,60,3,2,210,77,0,1,0,1,1,9,2798
2161,363,2012-02-28,4,2,0,35.3333320617676,16.3333339691162,0.666666686534882,52.333332
2162,363,2012-02-29,0,3,0,8.33333301544189,10.6666669845581,1,20,3,2,210,77,0,1,0,1,1,9,2798
2163,363,2012-02-29,1,3,0,22.6666660308838,34.6666679382324,2.66666674613953,60,3,2,210,7
2164,363,2012-02-29,2,3,0,25,34,1,60,3,2,210,77,0,1,0,1,1,9,2798.385313,5615.175703,2.6657176
2165,363,2012-02-29,3,3,0,34.3333320617676,25,0.666666686534882,60,3,2,210,77,0,1,0,1,1,9,27
2166,363,2012-02-29,4,3,0,38,22,0,60,3,2,210,77,0,1,0,1,1,9,2798.385313,5615.175703,2.6657176
2167,363,2012-03-01,0,4,0,9.66666698455811,2.33333325386047,0.333333343267441,12.333333
2168,363,2012-03-01,1,4,0,34.6666679382324,24.6666660308838,0.666666686534882,60,3,2,210,
2169,363,2012-03-01,2,4,0,43.3333320617676,15.3333330154419,1.33333337306976,60,3,2,210,7
2170,363,2012-03-01,3,4,0,33,25,2,60,3,2,210,77,0,1,0,1,1,9,2798.385313,5615.175703,2.6657176
2171,363,2012-03-01,4,4,0,14.6666669845581,18.3333339691162,1.66666662693024,34.666667
2172,363,2012-03-02,1,5,0,9,27,0.666666686534882,36.6666679382324,3,2,210,77,0,1,0,1,1,9,279
2173,363,2012-03-02,2,5,0,33.6666679382324,25.3333339691162,1,60,3,2,210,77,0,1,0,1,1,9,2798
2174,363,2012-03-02,3,5,0,25.6666660308838,25.3333339691162,9,60,3,2,210,77,0,1,0,1,1,9,2798
2175,363,2012-03-02,4,5,0,40.6666679382324,19,0.333333343267441,60,3,2,210,77,0,1,0,1,1,9,27
2176,363,2012-03-03,0,6,1,18,0,0.333333343267441,18.3333339691162,3,2,210,77,0,1,0,1,1,9,279
2177,363,2012-03-03,1,6,1,26.3333339691162,26.6666660308838,7,60,3,2,210,77,0,1,0,1,1,9,2798
2178,363,2012-03-03,2,6,1,31.6666660308838,23.3333339691162,5,60,3,2,210,77,0,1,0,1,1,9,2798
2179,363,2012-03-03,3,6,1,34.6666679382324,25,0.333333343267441,60,3,2,210,77,0,1,0,1,1,9,27
2180,363,2012-03-03,4,6,1,22.3333339691162,36.6666679382324,1,60,3,2,210,77,0,1,0,1,1,9,2798
2181,364,2012-02-25,0,6,1,50,8,2,60,1,2,210,77,0,1,0,1,1,10,2798.385313,5615.175703,2.6657176
2182,364,2012-02-25,1,6,1,35.6666679382324,22.6666660308838,1.66666662693024,60,3,2,210,7
2183,364,2012-02-25,2,6,1,31.3333339691162,28.3333339691162,0.333333343267441,60,3,2,210,
2184,364,2012-02-25,3,6,1,33.6666679382324,17.3333339691162,9,60,3,2,210,77,0,1,0,1,1,10,279
2185,364,2012-02-25,4,6,1,39,13.6666669845581,0.333333343267441,53,3,2,210,77,0,1,0,1,1,10,2
2186,364,2012-02-26,0,7,1,1,5.33333349227905,0.333333343267441,6.66666650772095,3,2,210,7
2187,364,2012-02-26,1,7,1,11.3333330154419,40.6666679382324,8,60,3,2,210,77,0,1,0,1,1,10,279
2188,364,2012-02-26,2,7,1,37,22.3333339691162,0.666666686534882,60,3,2,210,77,0,1,0,1,1,10,2
2189,364,2012-02-26,3,7,1,34.3333320617676,14.3333330154419,11.3333330154419,60,3,2,210,7
2190,364,2012-02-26,4,7,1,42.6666679382324,11.3333330154419,6,60,3,2,210,77,0,1,0,1,1,10,279
2191,364,2012-02-27,0,1,0,0.333333343267441,0,0.666666686534882,1,3,2,210,77,0,1,0,1,1,10,27
2192,364,2012-02-27,1,1,0,11.6666669845581,23.6666660308838,2.66666674613953,38,3,2,210,7
2193,364,2012-02-27,2,1,0,34.3333320617676,25.6666660308838,0,60,3,2,210,77,0,1,0,1,1,10,279
2194,364,2012-02-27,3,1,0,13.3333330154419,44.3333320617676,2.33333325386047,60,3,2,210,7
2195,364,2012-02-27,4,1,0,42.6666679382324,17,0.333333343267441,60,3,2,210,77,0,1,0,1,1,10,2
2196,364,2012-02-28,0,2,0,6.33333349227905,10.6666669845581,0,17,3,2,210,77,0,1,0,1,1,10,279
2197,364,2012-02-28,1,2,0,21.6666660308838,35,3.33333325386047,60,3,2,210,77,0,1,0,1,1,10,27
2198,364,2012-02-28,2,2,0,29.6666660308838,30,0.333333343267441,60,3,2,210,77,0,1,0,1,1,10,2
2199,364,2012-02-28,3,2,0,10.6666669845581,38,11.3333330154419,60,3,2,210,77,0,1,0,1,1,10,27

2200,364,2012-02-28,4,2,0,31,28.6666660308838,0.333333343267441,60,3,2,210,77,0,1,0,1,1,10,2
2201,364,2012-02-29,0,3,0,12,13,1,26,3,2,210,77,0,1,0,1,1,10,2798.385313,5615.175703,2.665717
2202,364,2012-02-29,1,3,0,20,37.6666679382324,2.33333325386047,60,3,2,210,77,0,1,0,1,1,10,27
2203,364,2012-02-29,2,3,0,40.6666679382324,18.3333339691162,1,60,3,2,210,77,0,1,0,1,1,10,279
2204,364,2012-02-29,3,3,0,29.3333339691162,30,0.666666686534882,60,3,2,210,77,0,1,0,1,1,10,2
2205,364,2012-02-29,4,3,0,31.3333339691162,27,1.66666662693024,60,3,2,210,77,0,1,0,1,1,10,27
2206,365,2012-03-21,0,3,0,25.6666660308838,21.3333339691162,0.666666686534882,47.666667
2207,365,2012-03-21,1,3,0,18,42,0,60,3,2,210,68,0,1,0,1,1,9,1581.717696,1731.878306,2.3196142
2208,365,2012-03-21,2,3,0,30.6666660308838,29,0.333333343267441,60,3,2,210,68,0,1,0,1,1,9,15
2209,365,2012-03-21,3,3,0,12,44.6666679382324,3.33333325386047,60,3,2,210,68,0,1,0,1,1,9,158
2210,365,2012-03-21,4,3,0,40.3333320617676,19.3333339691162,0.333333343267441,60,3,2,210,
2211,365,2012-03-22,1,4,0,21.3333339691162,32.1666679382324,0.5,54,3,2,210,68,0,1,0,1,1,9,15
2212,365,2012-03-22,2,4,0,34,25.6000003814697,0.400000005960464,60,3,2,210,68,0,1,0,1,1,9,15
2213,365,2012-03-22,3,4,0,19,39.6666679382324,1.33333337306976,60,3,2,210,68,0,1,0,1,1,9,158
2214,365,2012-03-22,4,4,0,25,35,0,60,3,2,210,68,0,1,0,1,1,9,1581.717696,1731.878306,2.3196142
2215,365,2012-03-23,0,5,0,34.3333320617676,25.6666660308838,0,60,3,2,210,68,0,1,0,1,1,9,1581
2216,365,2012-03-23,1,5,0,32.6666679382324,27,0.333333343267441,60,3,2,210,68,0,1,0,1,1,9,15
2217,365,2012-03-23,2,5,0,26.75,33.25,0,60,3,2,210,68,0,1,0,1,1,9,1581.717696,1731.878306,2.31
2218,365,2012-03-23,3,5,0,21.3333339691162,37.1666679382324,1.5,60,3,2,210,68,0,1,0,1,1,9,15
2219,365,2012-03-23,4,5,0,16.3333339691162,43,0.666666686534882,60,3,2,210,68,0,1,0,1,1,9,15
2220,365,2012-03-24,0,6,1,8,24.5,0.5,33,2,2,210,68,0,1,0,1,1,9,1581.717696,1731.878306,2.319614
2221,365,2012-03-24,1,6,1,22.3333339691162,33.6666679382324,4,60,3,2,210,68,0,1,0,1,1,9,1581
2222,365,2012-03-24,2,6,1,20.6666660308838,33.6666679382324,5.66666650772095,60,3,2,210,6
2223,365,2012-03-24,3,6,1,45.3333320617676,14,0.666666686534882,60,3,2,210,68,0,1,0,1,1,9,15
2224,365,2012-03-24,4,6,1,29,30.3333339691162,0.666666686534882,60,3,2,210,68,0,1,0,1,1,9,15
2225,365,2012-03-25,0,0,1,16.6666660308838,26.3333339691162,0.333333343267441,43.333332
2226,365,2012-03-25,1,0,1,31,28.5,0.5,60,3,2,210,68,0,1,0,1,1,9,1581.717696,1731.878306,2.31961
2227,365,2012-03-25,2,0,1,25.6666660308838,34.3333320617676,0,60,3,2,210,68,0,1,0,1,1,9,1581
2228,365,2012-03-25,3,0,1,24.3333339691162,32,3.66666674613953,60,3,2,210,68,0,1,0,1,1,9,158
2229,365,2012-03-25,4,0,1,30,29.6666660308838,0.333333343267441,60,3,2,210,68,0,1,0,1,1,9,15
2230,365,2012-03-26,1,1,0,32,28,0,60,2,2,210,68,0,1,0,1,1,9,1581.717696,1731.878306,2.3196142
2231,365,2012-03-26,2,1,0,27.3333339691162,32.6666679382324,0,60,3,2,210,68,0,1,0,1,1,9,1581
2232,365,2012-03-26,3,1,0,31,28.6666660308838,0.333333343267441,60,3,2,210,68,0,1,0,1,1,9,15
2233,365,2012-03-26,4,1,0,23,36,1,60,3,2,210,68,0,1,0,1,1,9,1581.717696,1731.878306,2.3196142
2234,366,2012-04-07,1,6,1,21.3333339691162,12,3.33333325386047,36.6666679382324,3,2,210,6
2235,366,2012-04-07,2,6,1,39,15,6,60,3,2,210,68,0,1,0,1,1,12,2968.891659,3289.410553,3.816546
2236,366,2012-04-07,3,6,1,18,18.6666660308838,23.3333339691162,60,3,2,210,68,0,1,0,1,1,12,29
2237,366,2012-04-07,4,6,1,38.3333320617676,19,2.66666674613953,60,3,2,210,68,0,1,0,1,1,12,29
2238,366,2012-04-08,1,7,1,28.6666660308838,20.3333339691162,3,52,3,2,210,68,0,1,0,1,1,12,296
2239,366,2012-04-08,2,7,1,21,19.6666660308838,19.3333339691162,60,3,2,210,68,0,1,0,1,1,12,29
2240,366,2012-04-08,3,7,1,38,18,4,60,3,2,210,68,0,1,0,1,1,12,2968.891659,3289.410553,3.816546
2241,366,2012-04-08,4,7,1,35,22.3333339691162,2.66666674613953,60,3,2,210,68,0,1,0,1,1,12,29
2242,366,2012-04-09,1,1,0,5.33333349227905,25,18.6666660308838,49,3,2,210,68,0,1,0,1,1,12,29
2243,366,2012-04-09,2,1,0,22.6666660308838,25,12.3333330154419,60,3,2,210,68,0,1,0,1,1,12,29
2244,366,2012-04-09,3,1,0,34.3333320617676,21.6666660308838,4,60,3,2,210,68,0,1,0,1,1,12,296
2245,366,2012-04-09,4,1,0,36,21.3333339691162,2.66666674613953,60,3,2,210,68,0,1,0,1,1,12,29
2246,366,2012-04-10,0,2,0,9,19,1.33333337306976,29.3333339691162,3,2,210,68,0,1,0,1,1,12,296
2247,366,2012-04-10,1,2,0,2,16.3333339691162,41.6666679382324,60,3,2,210,68,0,1,0,1,1,12,296
2248,366,2012-04-10,2,2,0,3.33333325386047,13,43.6666679382324,60,3,2,210,68,0,1,0,1,1,12,29
2249,366,2012-04-10,3,2,0,38,16.6666660308838,5.33333349227905,60,3,2,210,68,0,1,0,1,1,12,29

2250,366,2012-04-10,4,2,0,41,18.6666660308838,0.333333343267441,60,3,2,210,68,0,1,0,1,1,12,2
2251,366,2012-04-11,0,3,0,12,12,2.33333325386047,26.3333339691162,3,2,210,68,0,1,0,1,1,12,29
2252,366,2012-04-11,1,3,0,28.6666660308838,26.6666660308838,4.66666650772095,60,3,2,210,6
2253,366,2012-04-11,2,3,0,25.6666660308838,25.6666660308838,8.66666698455811,60,3,2,210,6
2254,366,2012-04-11,3,3,0,17.3333339691162,19.3333339691162,23.3333339691162,60,3,2,210,6
2255,366,2012-04-11,4,3,0,25.6666660308838,30.3333339691162,4,60,3,2,210,68,0,1,0,1,1,12,296
2256,366,2012-04-12,0,4,0,0,10.3333330154419,2,12.3333330154419,3,2,210,68,0,1,0,1,1,12,2968
2257,366,2012-04-12,1,4,0,30.6666660308838,19.3333339691162,10,60,3,2,210,68,0,1,0,1,1,12,29
2258,366,2012-04-12,2,4,0,33.6666679382324,25.6666660308838,0.666666686534882,60,3,2,210,
2259,366,2012-04-12,3,4,0,45.6666679382324,14.3333330154419,0,60,3,2,210,68,0,1,0,1,1,12,296
2260,366,2012-04-12,4,4,0,29.3333339691162,29,1.66666662693024,60,3,2,210,68,0,1,0,1,1,12,29
2261,366,2012-04-13,0,5,0,10,7.66666650772095,1,18.6666660308838,3,2,210,68,0,1,0,1,1,12,296
2262,366,2012-04-13,1,5,0,18.6666660308838,25.3333339691162,16,60,3,2,210,68,0,1,0,1,1,12,29
2263,366,2012-04-13,2,5,0,11.3333330154419,17,31.6666660308838,60,3,2,210,68,0,1,0,1,1,12,29
2264,366,2012-04-13,3,5,0,24.3333339691162,24.6666660308838,11,60,3,2,210,68,0,1,0,1,1,12,29
2265,366,2012-04-13,4,5,0,29.3333339691162,20.3333339691162,10.3333330154419,60,3,2,210,6
2266,393,2012-03-26,0,1,0,18,40,2,60,1,2,210,67,0,1,0,1,1,12,1800.527392,1604.306334,4.499072
2267,393,2012-03-26,1,1,0,20.6666660308838,23.3333339691162,16,60,3,2,210,67,0,1,0,1,1,12,18
2268,393,2012-03-26,2,1,0,18,36.6666679382324,5.33333349227905,60,3,2,210,67,0,1,0,1,1,12,18
2269,393,2012-03-26,3,1,0,29,29.5,1.5,60,2,2,210,67,0,1,0,1,1,12,1800.527392,1604.306334,4.499
2270,393,2012-03-27,0,2,0,5,11,0,16,1,2,210,67,0,1,0,1,1,12,1800.527392,1604.306334,4.4990727
2271,393,2012-03-27,1,2,0,24.3333339691162,32,3.66666674613953,60,3,2,210,67,0,1,0,1,1,12,18
2272,393,2012-03-27,2,2,0,16.3333339691162,33.6666679382324,10,60,3,2,210,67,0,1,0,1,1,12,18
2273,393,2012-03-27,3,2,0,23.25,36.5,0.25,60,3,2,210,67,0,1,0,1,1,12,1800.527392,1604.306334,4.
2274,393,2012-03-27,4,2,0,35,25,0,60,3,2,210,67,0,1,0,1,1,12,1800.527392,1604.306334,4.499072
2275,393,2012-04-03,0,2,0,15.6666669845581,13,5.66666650772095,34.3333320617676,3,2,210,6
2276,393,2012-04-03,1,2,0,29,31,0,60,3,2,210,67,0,1,0,1,1,12,1800.527392,1604.306334,4.499072
2277,393,2012-04-03,2,2,0,41.6666679382324,18.3333339691162,0,60,3,2,210,67,0,1,0,1,1,12,180
2278,393,2012-04-03,3,2,0,38,22,0,60,3,2,210,67,0,1,0,1,1,12,1800.527392,1604.306334,4.499072
2279,393,2012-04-03,4,2,0,36.25,23.75,0,60,3,2,210,67,0,1,0,1,1,12,1800.527392,1604.306334,4.4
2280,393,2012-04-04,0,3,0,15,27,1,43,1,2,210,67,0,1,0,1,1,12,1800.527392,1604.306334,4.499072
2281,393,2012-04-04,1,3,0,24.6666660308838,34.8333320617676,0.5,60,3,2,210,67,0,1,0,1,1,12,1
2282,393,2012-04-04,2,3,0,41,18.6666660308838,0.333333343267441,60,3,2,210,67,0,1,0,1,1,12,1
2283,393,2012-04-04,3,3,0,40.6666679382324,18.6666660308838,0.666666686534882,60,3,2,210,
2284,393,2012-04-04,4,3,0,44.6666679382324,15,0.333333343267441,60,3,2,210,67,0,1,0,1,1,12,1
2285,393,2012-04-05,0,4,0,11,11,0,22,1,2,210,67,0,1,0,1,1,12,1800.527392,1604.306334,4.499072
2286,393,2012-04-05,1,4,0,26,31.6666660308838,2.33333325386047,60,3,2,210,67,0,1,0,1,1,12,18
2287,393,2012-04-05,2,4,0,22.1666660308838,26.8333339691162,11,60,3,2,210,67,0,1,0,1,1,12,18
2288,393,2012-04-05,3,4,0,13,37.3333320617676,9.66666698455811,60,3,2,210,67,0,1,0,1,1,12,18
2289,393,2012-04-05,4,4,0,38.3333320617676,21.3333339691162,0.333333343267441,60,3,2,210,
2290,393,2012-04-06,1,5,0,24.6666660308838,28,0,52.6666679382324,3,2,210,67,0,1,0,1,1,12,180
2291,393,2012-04-06,2,5,0,24.6666660308838,28.6666660308838,6.66666650772095,60,3,2,210,6
2292,393,2012-04-06,3,5,0,28.5,18,13.5,60,3,2,210,67,0,1,0,1,1,12,1800.527392,1604.306334,4.49
2293,393,2012-04-06,4,5,0,36.5,23.5,0,60,3,2,210,67,0,1,0,1,1,12,1800.527392,1604.306334,4.499
2294,393,2012-04-07,4,6,1,38.5,21,0.5,60,2,2,210,67,0,1,0,1,1,12,1800.527392,1604.306334,4.499
2295,401,2012-04-01,0,0,1,17,20.2000007629395,1.79999995231628,39,3,2,210,77,0,1,0,1,1,12,29
2296,401,2012-04-01,1,0,1,9,37.3333320617676,13.6666669845581,60,3,2,210,77,0,1,0,1,1,12,290
2297,401,2012-04-01,2,0,1,27,25,8,60,3,2,210,77,0,1,0,1,1,12,2900.66293,4548.083033,2.6585890
2298,401,2012-04-01,3,0,1,20.6666660308838,26.6666660308838,12.6666669845581,60,3,2,210,7
2299,401,2012-04-01,4,0,1,28.6666660308838,22.3333339691162,0.666666686534882,51.666667

2300,401,2012-04-02,0,1,0,23,29,8,60,1,2,210,77,0,1,0,1,1,12,2900.66293,4548.083033,2.65858906
2301,401,2012-04-02,1,1,0,23.3333339691162,35,1.66666662693024,60,3,2,210,77,0,1,0,1,1,12,29
2302,401,2012-04-02,2,1,0,40,20,0,60,3,2,210,77,0,1,0,1,1,12,2900.66293,4548.083033,2.65858906
2303,401,2012-04-02,3,1,0,22,34.6666679382324,3.33333325386047,60,3,2,210,77,0,1,0,1,1,12,29
2304,401,2012-04-02,4,1,0,29.3333339691162,29.3333339691162,1.33333337306976,60,3,2,210,7
2305,401,2012-04-07,0,6,1,30,26,4,60,1,2,210,77,0,1,0,1,1,12,2900.66293,4548.083033,2.65858906
2306,401,2012-04-07,1,6,1,14,43,3,60,3,2,210,77,0,1,0,1,1,12,2900.66293,4548.083033,2.65858906
2307,401,2012-04-07,2,6,1,23,31.3333339691162,5.66666650772095,60,3,2,210,77,0,1,0,1,1,12,29
2308,401,2012-04-07,3,6,1,25.3333339691162,31.6666660308838,3,60,3,2,210,77,0,1,0,1,1,12,290
2309,401,2012-04-07,4,6,1,24,36,0,60,1,2,210,77,0,1,0,1,1,12,2900.66293,4548.083033,2.65858906
2310,401,2012-04-08,0,0,1,13,31.5,0,44.5,2,2,210,77,0,1,0,1,1,12,2900.66293,4548.083033,2.65858
2311,401,2012-04-08,1,0,1,27.5,31.1666660308838,1.33333337306976,60,3,2,210,77,0,1,0,1,1,12,2
2312,401,2012-04-08,2,0,1,35.5,24.25,0.25,60,3,2,210,77,0,1,0,1,1,12,2900.66293,4548.083033,2.6
2313,401,2012-04-08,3,0,1,10.6666669845581,38.3333320617676,11,60,3,2,210,77,0,1,0,1,1,12,29
2314,401,2012-04-08,4,0,1,34,26,0,60,3,2,210,77,0,1,0,1,1,12,2900.66293,4548.083033,2.65858906
2315,401,2012-04-09,0,1,0,9,30,10,49,1,2,210,77,0,1,0,1,1,12,2900.66293,4548.083033,2.65858906
2316,401,2012-04-09,1,1,0,36,23.6666660308838,0.333333343267441,60,3,2,210,77,0,1,0,1,1,12,2
2317,401,2012-04-09,2,1,0,22.7999992370605,33,4.19999980926514,60,3,2,210,77,0,1,0,1,1,12,29
2318,401,2012-04-09,3,1,0,31.2000007629395,23,5.80000019073486,60,3,2,210,77,0,1,0,1,1,12,29
2319,401,2012-04-09,4,1,0,37.6666679382324,18.6666660308838,3.66666674613953,60,3,2,210,7
2320,401,2012-04-10,0,2,0,17,21,1,39,1,2,210,77,0,1,0,1,1,12,2900.66293,4548.083033,2.65858906
2321,401,2012-04-10,1,2,0,28.3333339691162,19.3333339691162,12.3333330154419,60,3,2,210,7
2322,401,2012-04-10,2,2,0,20.3333339691162,23.3333339691162,16.3333339691162,60,3,2,210,7
2323,401,2012-04-10,3,2,0,40,18.5,1.5,60,3,2,210,77,0,1,0,1,1,12,2900.66293,4548.083033,2.65858
2324,401,2012-04-10,4,2,0,23,27.5,9.5,60,3,2,210,77,0,1,0,1,1,12,2900.66293,4548.083033,2.65858
2325,404,2012-03-27,0,2,0,14.5,34.75,0.5,49.75,3,2,210,86,1,1,1,1,1,8,2795.864645,4992.769149,2
2326,404,2012-03-27,1,2,0,27.1428565979004,31.2857151031494,1.57142853736877,60,3,2,210,8
2327,404,2012-03-27,2,2,0,15.6666669845581,39.5,4.83333349227905,60,3,2,210,86,1,1,1,1,1,8,27
2328,404,2012-03-27,3,2,0,43.1666679382324,16.8333339691162,0,60,3,2,210,86,1,1,1,1,1,8,2795
2329,404,2012-03-27,4,2,0,37.3333320617676,15.6666669845581,0.333333343267441,53.333332
2330,404,2012-03-28,0,3,0,10,37,0,47,1,2,210,86,1,1,1,1,1,8,2795.864645,4992.769149,2.49175737
2331,404,2012-03-28,1,3,0,17.75,37.5,4.75,60,3,2,210,86,1,1,1,1,1,8,2795.864645,4992.769149,2.4
2332,404,2012-03-28,2,3,0,16.7999992370605,39.4000015258789,3.79999995231628,60,3,2,210,8
2333,404,2012-03-28,3,3,0,33,23.6666660308838,3.33333325386047,60,3,2,210,86,1,1,1,1,1,8,279
2334,404,2012-03-28,4,3,0,28.6666660308838,31.3333339691162,0,60,3,2,210,86,1,1,1,1,1,8,2795
2335,404,2012-03-29,0,4,0,22,14,0.5,36.5,2,2,210,86,1,1,1,1,1,8,2795.864645,4992.769149,2.49175
2336,404,2012-03-29,1,4,0,35.3333320617676,24.6666660308838,0,60,3,2,210,86,1,1,1,1,1,8,2795
2337,404,2012-03-29,2,4,0,17.75,36.25,6,60,3,2,210,86,1,1,1,1,1,8,2795.864645,4992.769149,2.491
2338,404,2012-03-29,3,4,0,32.75,27.25,0,60,3,2,210,86,1,1,1,1,1,8,2795.864645,4992.769149,2.491
2339,404,2012-03-29,4,4,0,32,28,0,60,3,2,210,86,1,1,1,1,1,8,2795.864645,4992.769149,2.49175737
2340,404,2012-03-30,0,5,0,14.5,18,2.5,35,2,2,210,86,1,1,1,1,1,8,2795.864645,4992.769149,2.49175
2341,404,2012-03-30,1,5,0,16.3333339691162,28,15.6666669845581,60,3,2,210,86,1,1,1,1,1,8,279
2342,404,2012-03-30,2,5,0,30,28.3333339691162,1.66666662693024,60,3,2,210,86,1,1,1,1,1,8,279
2343,404,2012-03-30,3,5,0,35.25,22.75,2,60,3,2,210,86,1,1,1,1,1,8,2795.864645,4992.769149,2.491
2344,404,2012-03-30,4,5,0,24.6666660308838,33.6666679382324,1.66666662693024,60,3,2,210,8
2345,404,2012-03-31,0,6,1,21.5,21.5,0,43,2,2,210,86,1,1,1,1,1,8,2795.864645,4992.769149,2.49175
2346,404,2012-03-31,1,6,1,26.6666660308838,29.3333339691162,4,60,3,2,210,86,1,1,1,1,1,8,2795
2347,404,2012-03-31,2,6,1,28.3333339691162,31.6666660308838,0,60,3,2,210,86,1,1,1,1,1,8,2795
2348,404,2012-03-31,3,6,1,36,23.25,0.75,60,3,2,210,86,1,1,1,1,1,8,2795.864645,4992.769149,2.491
2349,404,2012-03-31,4,6,1,38.3333320617676,20,1.66666662693024,60,3,2,210,86,1,1,1,1,1,8,279

2350,404,2012-04-01,0,0,1,20.5,17.5,0.5,38.5,2,2,210,86,1,1,1,1,1,8,2795.864645,4992.769149,2.49
2351,404,2012-04-01,1,0,1,28.3333339691162,28.6666660308838,3,60,3,2,210,86,1,1,1,1,1,8,2795
2352,404,2012-04-01,2,0,1,11.3333330154419,42.6666679382324,6,60,3,2,210,86,1,1,1,1,1,8,2795
2353,404,2012-04-01,3,0,1,25.3333339691162,32.6666679382324,2,60,3,2,210,86,1,1,1,1,1,8,2795
2354,404,2012-04-01,4,0,1,26.3333339691162,26.6666660308838,3,56,3,2,210,86,1,1,1,1,1,8,2795
2355,404,2012-04-02,0,1,0,23.3333339691162,23,0.333333343267441,46.6666679382324,3,2,210,
2356,404,2012-04-02,1,1,0,15.3333330154419,43.3333320617676,1.33333337306976,60,3,2,210,8
2357,404,2012-04-02,2,1,0,23.6666660308838,35.6666679382324,0.666666686534882,60,3,2,210,
2358,404,2012-04-02,3,1,0,22.6666660308838,36.6666679382324,0.666666686534882,60,3,2,210,
2359,404,2012-04-02,4,1,0,36.6666679382324,23,0.333333343267441,60,3,2,210,86,1,1,1,1,1,8,27
2360,404,2012-04-03,0,2,0,10,33,1,44,1,2,210,86,1,1,1,1,1,8,2795.864645,4992.769149,2.49175737
2361,404,2012-04-03,1,2,0,21.25,38,0.75,60,3,2,210,86,1,1,1,1,1,8,2795.864645,4992.769149,2.491
2362,404,2012-04-03,2,2,0,4,56,0,60,3,2,210,86,1,1,1,1,1,8,2795.864645,4992.769149,2.491757375
2363,404,2012-04-03,3,2,0,28,30.6666660308838,1.33333337306976,60,3,2,210,86,1,1,1,1,1,8,279
2364,404,2012-04-03,4,2,0,30.6666660308838,28.3333339691162,1,60,3,2,210,86,1,1,1,1,1,8,2795
2365,405,2012-03-28,0,3,0,34,9,0,43,2,2,210,76,0,1,0,1,1,9,2743.034449,4670.450585,2.438846143
2366,405,2012-03-28,1,3,0,12.5,39.5,8,60,3,2,210,76,0,1,0,1,1,9,2743.034449,4670.450585,2.43884
2367,405,2012-03-28,2,3,0,25.3333339691162,31,3.66666674613953,60,3,2,210,76,0,1,0,1,1,9,274
2368,405,2012-03-28,3,3,0,39,17.6666660308838,3.33333325386047,60,3,2,210,76,0,1,0,1,1,9,274
2369,405,2012-03-28,4,3,0,39.3333320617676,20.6666660308838,0,60,3,2,210,76,0,1,0,1,1,9,2743
2370,405,2012-03-29,0,4,0,11,28,2,41,1,2,210,76,0,1,0,1,1,9,2743.034449,4670.450585,2.43884614
2371,405,2012-03-29,1,4,0,6.80000019073486,31.6000003814697,21.6000003814697,60,3,2,210,7
2372,405,2012-03-29,2,4,0,19.3333339691162,18.6666660308838,22,60,3,2,210,76,0,1,0,1,1,9,274
2373,405,2012-03-29,3,4,0,13.3333330154419,42,4.66666650772095,60,3,2,210,76,0,1,0,1,1,9,274
2374,405,2012-03-29,4,4,0,29.6666660308838,29.6666660308838,0.666666686534882,60,3,2,210,
2375,405,2012-03-30,0,5,0,4,8,6,18,1,2,210,76,0,1,0,1,1,9,2743.034449,4670.450585,2.438846143,
2376,405,2012-03-30,1,5,0,18.6666660308838,40,1.33333337306976,60,3,2,210,76,0,1,0,1,1,9,274
2377,405,2012-03-30,2,5,0,32.7999992370605,25.7999992370605,1.39999997615814,60,3,2,210,7
2378,405,2012-03-30,3,5,0,19.3333339691162,38.6666679382324,2,60,3,2,210,76,0,1,0,1,1,9,2743
2379,405,2012-03-30,4,5,0,34.6666679382324,25.3333339691162,0,60,3,2,210,76,0,1,0,1,1,9,2743
2380,405,2012-03-31,1,6,1,20.6666660308838,27.3333339691162,1.33333337306976,49.3333320
2381,405,2012-03-31,2,6,1,35.5,23.75,0.75,60,3,2,210,76,0,1,0,1,1,9,2743.034449,4670.450585,2.4
2382,405,2012-03-31,3,6,1,28.2000007629395,31.3999996185303,0.400000005960464,60,3,2,210,
2383,405,2012-03-31,4,6,1,39,21,0,60,3,2,210,76,0,1,0,1,1,9,2743.034449,4670.450585,2.43884614
2384,405,2012-04-01,0,0,1,9,28,2.5,39.5,2,2,210,76,0,1,0,1,1,9,2743.034449,4670.450585,2.438846
2385,405,2012-04-01,1,0,1,16.6666660308838,39,4.33333349227905,60,3,2,210,76,0,1,0,1,1,9,274
2386,405,2012-04-01,2,0,1,38.3333320617676,19.6666660308838,2,60,3,2,210,76,0,1,0,1,1,9,2743
2387,405,2012-04-01,3,0,1,27.25,31,1.75,60,3,2,210,76,0,1,0,1,1,9,2743.034449,4670.450585,2.438
2388,405,2012-04-01,4,0,1,29,28.3333339691162,2.66666674613953,60,3,2,210,76,0,1,0,1,1,9,274
2389,405,2012-04-02,0,1,0,8.66666698455811,26.6666660308838,12,47.3333320617676,3,2,210,7
2390,405,2012-04-02,1,1,0,14.6666669845581,41.3333320617676,4,60,3,2,210,76,0,1,0,1,1,9,2743
2391,405,2012-04-02,2,1,0,19.6666660308838,35.3333320617676,5,60,3,2,210,76,0,1,0,1,1,9,2743
2392,405,2012-04-02,3,1,0,15,34,11,60,3,2,210,76,0,1,0,1,1,9,2743.034449,4670.450585,2.4388461
2393,405,2012-04-02,4,1,0,24.3333339691162,31,4.66666650772095,60,3,2,210,76,0,1,0,1,1,9,274
2394,411,2012-03-17,0,6,1,30,29,1,60,1,2,210,68,1,1,0,1,1,12,2743.034449,4670.450585,2.4388461
2395,411,2012-03-17,1,6,1,25,34.3333320617676,0.666666686534882,60,3,2,210,68,1,1,0,1,1,12,2
2396,411,2012-03-17,2,6,1,20.6666660308838,39.3333320617676,0,60,3,2,210,68,1,1,0,1,1,12,274
2397,411,2012-03-17,3,6,1,22.6666660308838,36.3333320617676,1,60,3,2,210,68,1,1,0,1,1,12,274
2398,411,2012-03-17,4,6,1,38,21,1,60,3,2,210,68,1,1,0,1,1,12,2743.034449,4670.450585,2.4388461
2399,411,2012-03-18,0,7,1,14.6666669845581,4.66666650772095,0,19.3333339691162,3,2,210,68

2400,411,2012-03-18,1,7,1,17.6666660308838,32.6666679382324,9.66666698455811,60,3,2,210,6
2401,411,2012-03-18,2,7,1,25,32,3,60,3,2,210,68,1,1,0,1,1,12,2743.034449,4670.450585,2.4388461
2402,411,2012-03-18,3,7,1,20.6666660308838,37,2.33333325386047,60,3,2,210,68,1,1,0,1,1,12,27
2403,411,2012-03-18,4,7,1,36.3333320617676,23,0.666666686534882,60,3,2,210,68,1,1,0,1,1,12,2
2404,411,2012-03-19,1,1,0,16.3333339691162,37.6666679382324,5,59,3,2,210,68,1,1,0,1,1,12,274
2405,411,2012-03-19,2,1,0,14.6666669845581,21.6666660308838,23.6666660308838,60,3,2,210,6
2406,411,2012-03-19,3,1,0,18.6666660308838,37.3333320617676,4,60,3,2,210,68,1,1,0,1,1,12,274
2407,411,2012-03-19,4,1,0,40,19.3333339691162,0.666666686534882,60,3,2,210,68,1,1,0,1,1,12,2
2408,411,2012-03-20,0,2,0,3.33333325386047,7.66666650772095,2.33333325386047,13.3333330
2409,411,2012-03-20,1,2,0,3.33333325386047,23.3333339691162,33.3333320617676,60,3,2,210,6
2410,411,2012-03-20,2,2,0,5,36,19,60,3,2,210,68,1,1,0,1,1,12,2743.034449,4670.450585,2.4388461
2411,411,2012-03-20,3,2,0,17.3333339691162,42,0.666666686534882,60,3,2,210,68,1,1,0,1,1,12,2
2412,411,2012-03-20,4,2,0,30.6666660308838,29.3333339691162,0,60,3,2,210,68,1,1,0,1,1,12,274
2413,411,2012-03-21,0,3,0,2.66666674613953,9.66666698455811,0,12.3333330154419,3,2,210,68
2414,411,2012-03-21,1,3,0,26,34,0,60,3,2,210,68,1,1,0,1,1,12,2743.034449,4670.450585,2.4388461
2415,411,2012-03-21,2,3,0,23,36,1,60,3,2,210,68,1,1,0,1,1,12,2743.034449,4670.450585,2.4388461
2416,411,2012-03-21,3,3,0,12.3333330154419,46,1.66666662693024,60,3,2,210,68,1,1,0,1,1,12,27
2417,411,2012-03-21,4,3,0,31.6666660308838,28.3333339691162,0,60,3,2,210,68,1,1,0,1,1,12,274
2418,411,2012-03-22,0,4,0,2.33333325386047,6.33333349227905,0.333333343267441,9,3,2,210,6
2419,411,2012-03-22,1,4,0,27.6666660308838,27.3333339691162,5,60,3,2,210,68,1,1,0,1,1,12,274
2420,411,2012-03-22,2,4,0,37,12.6666669845581,10.3333330154419,60,3,2,210,68,1,1,0,1,1,12,27
2421,411,2012-03-22,3,4,0,45,10.6666669845581,4.33333349227905,60,3,2,210,68,1,1,0,1,1,12,27
2422,411,2012-03-22,4,4,0,33.6666679382324,22.3333339691162,4,60,3,2,210,68,1,1,0,1,1,12,274
2423,411,2012-03-23,0,5,0,0,8,0.333333343267441,8.33333301544189,3,2,210,68,1,1,0,1,1,12,274
2424,411,2012-03-23,1,5,0,3,55.6666679382324,1.33333337306976,60,3,2,210,68,1,1,0,1,1,12,274
2425,411,2012-03-23,2,5,0,22,30.6666660308838,7.33333349227905,60,3,2,210,68,1,1,0,1,1,12,27
2426,411,2012-03-23,3,5,0,13.3333330154419,43,3.66666674613953,60,3,2,210,68,1,1,0,1,1,12,27
2427,411,2012-03-23,4,5,0,38.3333320617676,16.3333339691162,5.33333349227905,60,3,2,210,6
2428,413,2012-03-28,0,3,0,16,23.6666660308838,0.666666686534882,40.3333320617676,3,2,210,
2429,413,2012-03-28,1,3,0,33,26.6666660308838,0.333333343267441,60,3,2,210,73,0,1,0,1,1,12,2
2430,413,2012-03-28,2,3,0,40.5,17.5,2,60,3,2,210,73,0,1,0,1,1,12,2660.644625,4420.10785,2.35747
2431,413,2012-03-28,3,3,0,39.3333320617676,20.6666660308838,0,60,3,2,210,73,0,1,0,1,1,12,266
2432,413,2012-03-28,4,3,0,48.3333320617676,11.6666669845581,0,60,3,2,210,73,0,1,0,1,1,12,266
2433,413,2012-03-29,0,4,0,24,8,2,34,1,2,210,73,0,1,0,1,1,12,2660.644625,4420.10785,2.357478342
2434,413,2012-03-29,1,4,0,42,15.6666669845581,2.33333325386047,60,3,2,210,73,0,1,0,1,1,12,26
2435,413,2012-03-29,2,4,0,38.2000007629395,21.7999992370605,0,60,3,2,210,73,0,1,0,1,1,12,266
2436,413,2012-03-29,3,4,0,33.7999992370605,25.3999996185303,0.800000011920929,60,3,2,210,
2437,413,2012-03-29,4,4,0,24.6666660308838,34,1.33333337306976,60,3,2,210,73,0,1,0,1,1,12,26
2438,413,2012-03-30,0,5,0,21,21,2,44,1,2,210,73,0,1,0,1,1,12,2660.644625,4420.10785,2.35747834
2439,413,2012-03-30,1,5,0,46,13,1,60,3,2,210,73,0,1,0,1,1,12,2660.644625,4420.10785,2.35747834
2440,413,2012-03-30,2,5,0,46.3333320617676,12.3333330154419,1.33333337306976,60,3,2,210,7
2441,413,2012-03-30,3,5,0,54.75,5.25,0,60,3,2,210,73,0,1,0,1,1,12,2660.644625,4420.10785,2.3574
2442,413,2012-03-30,4,5,0,46.3333320617676,13,0.666666686534882,60,3,2,210,73,0,1,0,1,1,12,2
2443,413,2012-03-31,0,6,1,28.25,14.75,1.5,44.5,3,2,210,73,0,1,0,1,1,12,2660.644625,4420.10785,2
2444,413,2012-03-31,1,6,1,41.5,17,1.5,60,3,2,210,73,0,1,0,1,1,12,2660.644625,4420.10785,2.35747
2445,413,2012-03-31,2,6,1,42,17.3333339691162,0.666666686534882,60,3,2,210,73,0,1,0,1,1,12,2
2446,413,2012-03-31,3,6,1,47.6666679382324,12,0.333333343267441,60,3,2,210,73,0,1,0,1,1,12,2
2447,413,2012-03-31,4,6,1,49.6666679382324,10.3333330154419,0,60,3,2,210,73,0,1,0,1,1,12,266
2448,413,2012-04-01,0,0,1,34,12,1,47,1,2,210,73,0,1,0,1,1,12,2660.644625,4420.10785,2.35747834
2449,413,2012-04-01,1,0,1,34,25,1,60,3,2,210,73,0,1,0,1,1,12,2660.644625,4420.10785,2.35747834

2450,413,2012-04-01,2,0,1,46.1666679382324,13.6666669845581,0.16666667163372,60,3,2,210,7
2451,413,2012-04-01,3,0,1,42.5999984741211,16.3999996185303,1,60,3,2,210,73,0,1,0,1,1,12,266
2452,413,2012-04-01,4,0,1,37.6666679382324,22,0.333333343267441,60,3,2,210,73,0,1,0,1,1,12,2
2453,413,2012-04-02,0,1,0,29,16.6666660308838,2.66666674613953,48.3333320617676,3,2,210,7
2454,413,2012-04-02,1,1,0,44.4000015258789,14.3999996185303,1.20000004768372,60,3,2,210,7
2455,413,2012-04-02,2,1,0,49.6666679382324,10.3333330154419,0,60,3,2,210,73,0,1,0,1,1,12,266
2456,413,2012-04-02,3,1,0,47,12.25,0.75,60,3,2,210,73,0,1,0,1,1,12,2660.644625,4420.10785,2.357
2457,413,2012-04-02,4,1,0,49.8333320617676,10.1666669845581,0,60,3,2,210,73,0,1,0,1,1,12,266
2458,413,2012-04-03,0,2,0,25.5,15,2,42.5,2,2,210,73,0,1,0,1,1,12,2660.644625,4420.10785,2.35747
2459,413,2012-04-03,1,2,0,41.7999992370605,16.2999992370605,1.89999997615814,60,3,2,210,7
2460,413,2012-04-03,2,2,0,36.8888893127441,16.3333339691162,6.77777767181396,60,3,2,210,7
2461,413,2012-04-03,3,2,0,43.875,13,3.125,60,3,2,210,73,0,1,0,1,1,12,2660.644625,4420.10785,2.3
2462,413,2012-04-03,4,2,0,48.3333320617676,11.1666669845581,0.5,60,3,2,210,73,0,1,0,1,1,12,26
2463,413,2012-04-04,3,3,0,60,0,0,60,1,2,210,73,0,1,0,1,1,12,2660.644625,4420.10785,2.35747834
2464,413,2012-04-04,4,3,0,50.6666679382324,9.33333301544189,0,60,3,2,210,73,0,1,0,1,1,12,266
2465,414,2012-03-29,0,4,0,13,25,3,41,1,2,210,77,0,1,0,1,1,11,2660.644625,4420.10785,2.3574783
2466,414,2012-03-29,1,4,0,14,43,3,60,1,2,210,77,0,1,0,1,1,11,2660.644625,4420.10785,2.3574783
2467,414,2012-03-29,2,4,0,43.6666679382324,15.3333330154419,1,60,3,2,210,77,0,1,0,1,1,11,266
2468,414,2012-03-29,3,4,0,44.6666679382324,15.3333330154419,0,60,3,2,210,77,0,1,0,1,1,11,266
2469,414,2012-03-29,4,4,0,34,26,0,60,3,2,210,77,0,1,0,1,1,11,2660.644625,4420.10785,2.3574783
2470,414,2012-03-30,0,5,0,2,42,5,49,1,2,210,77,0,1,0,1,1,11,2660.644625,4420.10785,2.35747834
2471,414,2012-03-30,1,5,0,20.6000003814697,36.5999984741211,2.79999995231628,60,3,2,210,7
2472,414,2012-03-30,2,5,0,33.3333320617676,20.5,6.16666650772095,60,3,2,210,77,0,1,0,1,1,11,2
2473,414,2012-03-30,3,5,0,24.1666660308838,34,1.83333337306976,60,3,2,210,77,0,1,0,1,1,11,26
2474,414,2012-03-30,4,5,0,35,25,0,60,3,2,210,77,0,1,0,1,1,11,2660.644625,4420.10785,2.3574783
2475,414,2012-03-31,0,6,1,0.5,26.5,1,28,2,2,210,77,0,1,0,1,1,11,2660.644625,4420.10785,2.357478
2476,414,2012-03-31,1,6,1,24.3333339691162,33.5,2.16666674613953,60,3,2,210,77,0,1,0,1,1,11,2
2477,414,2012-03-31,2,6,1,36.5999984741211,22,1.39999997615814,60,3,2,210,77,0,1,0,1,1,11,26
2478,414,2012-03-31,3,6,1,24,35,1,60,3,2,210,77,0,1,0,1,1,11,2660.644625,4420.10785,2.3574783
2479,414,2012-03-31,4,6,1,36.7999992370605,21,2.20000004768372,60,3,2,210,77,0,1,0,1,1,11,26
2480,414,2012-04-04,0,3,0,22,25,1,48,2,2,210,77,0,1,0,1,1,11,2660.644625,4420.10785,2.3574783
2481,414,2012-04-04,1,3,0,17.3333339691162,39.5,3.16666674613953,60,3,2,210,77,0,1,0,1,1,11,2
2482,414,2012-04-04,2,3,0,30.25,23.25,6.5,60,3,2,210,77,0,1,0,1,1,11,2660.644625,4420.10785,2.3
2483,414,2012-04-04,3,3,0,27.8333339691162,29.1666660308838,3,60,3,2,210,77,0,1,0,1,1,11,266
2484,414,2012-04-04,4,3,0,39.75,16.25,0.5,56.5,3,2,210,77,0,1,0,1,1,11,2660.644625,4420.10785,2
2485,416,2012-04-04,0,3,0,37,22,1,60,1,2,210,77,1,1,1,1,1,8,2743.034449,4670.450585,2.4388461
2486,416,2012-04-04,1,3,0,23.6666660308838,34,2.33333325386047,60,3,2,210,77,1,1,1,1,1,8,274
2487,416,2012-04-04,2,3,0,34.6666679382324,22,3.33333325386047,60,3,2,210,77,1,1,1,1,1,8,274
2488,416,2012-04-04,3,3,0,18.5,38.5,3,60,2,2,210,77,1,1,1,1,1,8,2743.034449,4670.450585,2.43884
2489,416,2012-04-05,0,4,0,10,26.5,2,38.5,2,2,210,77,1,1,1,1,1,8,2743.034449,4670.450585,2.43884
2490,416,2012-04-05,1,4,0,11.75,44.5,3.75,60,3,2,210,77,1,1,1,1,1,8,2743.034449,4670.450585,2.4
2491,416,2012-04-05,2,4,0,43.6666679382324,15.3333330154419,1,60,3,2,210,77,1,1,1,1,1,8,2743
2492,416,2012-04-05,3,4,0,36.25,22,1.75,60,3,2,210,77,1,1,1,1,1,8,2743.034449,4670.450585,2.438
2493,416,2012-04-05,4,4,0,35.8333320617676,23.8333339691162,0.333333343267441,60,3,2,210,
2494,416,2012-04-06,0,5,0,2,42,5,49,1,2,210,77,1,1,1,1,1,8,2743.034449,4670.450585,2.43884614
2495,416,2012-04-06,1,5,0,20.6000003814697,36.5999984741211,2.79999995231628,60,3,2,210,7
2496,416,2012-04-06,2,5,0,33.3333320617676,20.5,6.16666650772095,60,3,2,210,77,1,1,1,1,1,8,27
2497,416,2012-04-06,3,5,0,27,30.3333339691162,2.66666674613953,60,3,2,210,77,1,1,1,1,1,8,274
2498,416,2012-04-06,4,5,0,36,24,0,60,3,2,210,77,1,1,1,1,1,8,2743.034449,4670.450585,2.4388461
2499,416,2012-04-07,0,6,1,0,35,1,36,1,2,210,77,1,1,1,1,1,8,2743.034449,4670.450585,2.43884614

2500,416,2012-04-07,1,6,1,26,30.3333339691162,3.66666674613953,60,3,2,210,77,1,1,1,1,1,8,274
2501,416,2012-04-07,2,6,1,32.6666679382324,26.6666660308838,0.666666686534882,60,3,2,210,
2502,416,2012-04-07,3,6,1,22,36.75,1.25,60,3,2,210,77,1,1,1,1,1,8,2743.034449,4670.450585,2.438
2503,416,2012-04-07,4,6,1,35.3333320617676,24.6666660308838,0,60,3,2,210,77,1,1,1,1,1,8,2743
2504,416,2012-04-08,0,0,1,1,18,1,20,1,2,210,77,1,1,1,1,1,8,2743.034449,4670.450585,2.43884614
2505,416,2012-04-08,1,0,1,22.6666660308838,36.6666679382324,0.666666686534882,60,3,2,210,
2506,416,2012-04-08,2,0,1,34,19,7,60,3,2,210,77,1,1,1,1,1,8,2743.034449,4670.450585,2.43884614
2507,416,2012-04-08,3,0,1,31,28,1,60,3,2,210,77,1,1,1,1,1,8,2743.034449,4670.450585,2.43884614
2508,416,2012-04-08,4,0,1,36.6666679382324,19.6666660308838,3.66666674613953,60,3,2,210,7
2509,416,2012-04-09,3,1,0,43,15,2,60,1,2,210,77,1,1,1,1,1,8,2743.034449,4670.450585,2.43884614
2510,416,2012-04-09,4,1,0,46,0,0,46,1,2,210,77,1,1,1,1,1,8,2743.034449,4670.450585,2.43884614
2511,418,2012-02-25,0,6,1,40,18,2,60,1,2,210,77,0,1,0,1,1,8,2694.68103,4687.400677,2.42377622
2512,418,2012-02-25,1,6,1,29.6666660308838,25.3333339691162,5,60,3,2,210,77,0,1,0,1,1,8,2694
2513,418,2012-02-25,2,6,1,42,16.3333339691162,1.66666662693024,60,3,2,210,77,0,1,0,1,1,8,269
2514,418,2012-02-25,3,6,1,32.3333320617676,23.3333339691162,4.33333349227905,60,3,2,210,7
2515,418,2012-02-25,4,6,1,40.3333320617676,18,1.33333337306976,59.6666679382324,3,2,210,7
2516,418,2012-02-26,0,7,1,0,0.333333343267441,0,0.333333343267441,3,2,210,77,0,1,0,1,1,8,269
2517,418,2012-02-26,1,7,1,22.3333339691162,32.6666679382324,5,60,3,2,210,77,0,1,0,1,1,8,2694
2518,418,2012-02-26,2,7,1,14.3333330154419,41.6666679382324,4,60,3,2,210,77,0,1,0,1,1,8,2694
2519,418,2012-02-26,3,7,1,22,37.6666679382324,0.333333343267441,60,3,2,210,77,0,1,0,1,1,8,26
2520,418,2012-02-26,4,7,1,35.3333320617676,23.3333339691162,1.33333337306976,60,3,2,210,7
2521,418,2012-02-27,1,1,0,14.3333330154419,12.6666669845581,0.333333343267441,27.333333
2522,418,2012-02-27,2,1,0,34.3333320617676,25,0.666666686534882,60,3,2,210,77,0,1,0,1,1,8,26
2523,418,2012-02-27,3,1,0,41.6666679382324,17.3333339691162,1,60,3,2,210,77,0,1,0,1,1,8,2694
2524,418,2012-02-27,4,1,0,49,9,0.333333343267441,58.3333320617676,3,2,210,77,0,1,0,1,1,8,269
2525,418,2012-02-28,1,2,0,16.3333339691162,39.6666679382324,3.66666674613953,59.666667
2526,418,2012-02-28,2,2,0,24.6666660308838,35.3333320617676,0,60,3,2,210,77,0,1,0,1,1,8,2694
2527,418,2012-02-28,3,2,0,36.6666679382324,23.3333339691162,0,60,3,2,210,77,0,1,0,1,1,8,2694
2528,418,2012-02-28,4,2,0,21.3333339691162,25.6666660308838,1.66666662693024,48.666667
2529,418,2012-02-29,1,3,0,13,26.3333339691162,5.66666650772095,45,3,2,210,77,0,1,0,1,1,8,269
2530,418,2012-02-29,2,3,0,30.6666660308838,26.6666660308838,2.66666674613953,60,3,2,210,7
2531,418,2012-02-29,3,3,0,31.3333339691162,25,3.66666674613953,60,3,2,210,77,0,1,0,1,1,8,269
2532,418,2012-02-29,4,3,0,28,18.6666660308838,1.66666662693024,48.3333320617676,3,2,210,7
2533,418,2012-03-01,1,4,0,46.3333320617676,10.3333330154419,0.333333343267441,57,3,2,210,
2534,418,2012-03-01,2,4,0,25.3333339691162,33.6666679382324,1,60,3,2,210,77,0,1,0,1,1,8,2694
2535,418,2012-03-01,3,4,0,38.6666679382324,21,0.333333343267441,60,3,2,210,77,0,1,0,1,1,8,26
2536,418,2012-03-01,4,4,0,41,18.3333339691162,0.666666686534882,60,3,2,210,77,0,1,0,1,1,8,26
2537,418,2012-03-02,0,5,0,14.6666669845581,10.3333330154419,0.333333343267441,25.333333
2538,418,2012-03-02,1,5,0,25.3333339691162,12.3333330154419,22.3333339691162,60,3,2,210,7
2539,418,2012-03-02,2,5,0,36.3333320617676,23,0.666666686534882,60,3,2,210,77,0,1,0,1,1,8,26
2540,418,2012-03-02,3,5,0,34,24.6666660308838,1.33333337306976,60,3,2,210,77,0,1,0,1,1,8,269
2541,418,2012-03-02,4,5,0,36.3333320617676,20.6666660308838,0.333333343267441,57.333332
2542,418,2012-03-03,1,6,1,12,17.3333339691162,5,34.3333320617676,3,2,210,77,0,1,0,1,1,8,2694
2543,418,2012-03-03,2,6,1,30,29,1,60,3,2,210,77,0,1,0,1,1,8,2694.68103,4687.400677,2.42377622
2544,418,2012-03-03,3,6,1,46,14,0,60,3,2,210,77,0,1,0,1,1,8,2694.68103,4687.400677,2.42377622
2545,418,2012-03-03,4,6,1,42.6666679382324,17,0.333333343267441,60,3,2,210,77,0,1,0,1,1,8,26
2546,418,2012-03-04,1,7,1,25.3333339691162,16,0.666666686534882,42,3,2,210,77,0,1,0,1,1,8,26
2547,418,2012-03-04,2,7,1,40.3333320617676,18,1.66666662693024,60,3,2,210,77,0,1,0,1,1,8,269
2548,418,2012-03-04,3,7,1,42.6666679382324,15,2.33333325386047,60,3,2,210,77,0,1,0,1,1,8,269
2549,418,2012-03-04,4,7,1,38.3333320617676,21.6666660308838,0,60,3,2,210,77,0,1,0,1,1,8,2694

2550,422,2012-04-04,0,3,0,9,45.5,5.5,60,2,2,210,70,1,1,0,1,1,8,936.7231122,992.443142,5.150399
2551,422,2012-04-04,1,3,0,8.33333301544189,48.3333320617676,3.33333325386047,60,3,2,210,7
2552,422,2012-04-04,2,3,0,18,38.6666679382324,3.33333325386047,60,3,2,210,70,1,1,0,1,1,8,936
2553,422,2012-04-04,3,3,0,12,45.3333320617676,2.66666674613953,60,3,2,210,70,1,1,0,1,1,8,936
2554,422,2012-04-04,4,3,0,27.5,20.5,4.5,52.5,2,2,210,70,1,1,0,1,1,8,936.7231122,992.443142,5.15
2555,422,2012-04-05,0,4,0,14,43.25,2.75,60,3,2,210,70,1,1,0,1,1,8,936.7231122,992.443142,5.150
2556,422,2012-04-05,1,4,0,10.3333330154419,44.3333320617676,5.33333349227905,60,3,2,210,7
2557,422,2012-04-05,2,4,0,10.3333330154419,46.3333320617676,3.33333325386047,60,3,2,210,7
2558,422,2012-04-05,3,4,0,14,39.3333320617676,6.66666650772095,60,3,2,210,70,1,1,0,1,1,8,936
2559,422,2012-04-05,4,4,0,36.6666679382324,16.6666660308838,1.33333337306976,54.666667
2560,422,2012-04-06,0,5,0,16,43,1,60,3,2,210,70,1,1,0,1,1,8,936.7231122,992.443142,5.15039946
2561,422,2012-04-06,1,5,0,12.6666669845581,43.6666679382324,3.66666674613953,60,3,2,210,7
2562,422,2012-04-06,2,5,0,22,30.3333339691162,7.66666650772095,60,3,2,210,70,1,1,0,1,1,8,936
2563,422,2012-04-06,3,5,0,13.3333330154419,39.6666679382324,7,60,3,2,210,70,1,1,0,1,1,8,936.
2564,422,2012-04-06,4,5,0,20,28,1.5,49.5,2,2,210,70,1,1,0,1,1,8,936.7231122,992.443142,5.15039
2565,422,2012-04-07,0,6,1,8,49,3,60,3,2,210,70,1,1,0,1,1,8,936.7231122,992.443142,5.150399464,
2566,422,2012-04-07,1,6,1,20.6666660308838,38.6666679382324,0.666666686534882,60,3,2,210,
2567,422,2012-04-07,2,6,1,21.6666660308838,37,1.33333337306976,60,3,2,210,70,1,1,0,1,1,8,936
2568,422,2012-04-07,3,6,1,34,24.3333339691162,1.66666662693024,60,3,2,210,70,1,1,0,1,1,8,936
2569,422,2012-04-07,4,6,1,43.5,15.5,0,59,2,2,210,70,1,1,0,1,1,8,936.7231122,992.443142,5.15039
2570,422,2012-04-08,0,0,1,12,46,2,60,1,2,210,70,1,1,0,1,1,8,936.7231122,992.443142,5.15039946
2571,422,2012-04-08,1,0,1,20.6666660308838,39.3333320617676,0,60,3,2,210,70,1,1,0,1,1,8,936.
2572,422,2012-04-08,2,0,1,15,41,4,60,3,2,210,70,1,1,0,1,1,8,936.7231122,992.443142,5.15039946
2573,422,2012-04-08,3,0,1,19.6666660308838,34,6.33333349227905,60,3,2,210,70,1,1,0,1,1,8,936
2574,422,2012-04-08,4,0,1,28,23,0,51,1,2,210,70,1,1,0,1,1,8,936.7231122,992.443142,5.15039946
2575,422,2012-04-09,0,1,0,4,48,8,60,1,2,210,70,1,1,0,1,1,8,936.7231122,992.443142,5.150399464,
2576,422,2012-04-09,1,1,0,8.66666698455811,47.3333320617676,4,60,3,2,210,70,1,1,0,1,1,8,936.
2577,422,2012-04-09,2,1,0,12.6666669845581,46.6666679382324,0.666666686534882,60,3,2,210,
2578,422,2012-04-09,3,1,0,11.6666669845581,41.3333320617676,7,60,3,2,210,70,1,1,0,1,1,8,936.
2579,422,2012-04-09,4,1,0,24.3333339691162,18,1,43.3333320617676,3,2,210,70,1,1,0,1,1,8,936.
2580,422,2012-04-10,0,2,0,11,46,3,60,3,2,210,70,1,1,0,1,1,8,936.7231122,992.443142,5.15039946
2581,422,2012-04-10,1,2,0,11,45.3333320617676,3.66666674613953,60,3,2,210,70,1,1,0,1,1,8,936
2582,422,2012-04-10,2,2,0,18,40.3333320617676,1.66666662693024,60,3,2,210,70,1,1,0,1,1,8,936
2583,422,2012-04-10,3,2,0,11.6666669845581,43.3333320617676,5,60,3,2,210,70,1,1,0,1,1,8,936.
2584,422,2012-04-10,4,2,0,25.5,27.5,0.5,53.5,2,2,210,70,1,1,0,1,1,8,936.7231122,992.443142,5.15
2585,423,2012-04-04,0,3,0,44.5,14,0,58.5,2,2,210,75,1,1,1,1,1,9,978.1989598,1005.05473,4.531778
2586,423,2012-04-04,1,3,0,29.6666660308838,27.6666660308838,2.66666674613953,60,3,2,210,7
2587,423,2012-04-04,2,3,0,49.3333320617676,10.3333330154419,0.333333343267441,60,3,2,210,
2588,423,2012-04-04,3,3,0,26,33.3333320617676,0.666666686534882,60,3,2,210,75,1,1,1,1,1,9,97
2589,423,2012-04-04,4,3,0,40.3333320617676,19.6666660308838,0,60,3,2,210,75,1,1,1,1,1,9,978.
2590,423,2012-04-06,0,5,0,36,16.3333339691162,0.333333343267441,52.6666679382324,3,2,210,
2591,423,2012-04-06,1,5,0,28.25,31,0.75,60,3,2,210,75,1,1,1,1,1,9,978.1989598,1005.05473,4.531
2592,423,2012-04-06,2,5,0,7.33333349227905,31.6666660308838,21,60,3,2,210,75,1,1,1,1,1,9,978
2593,423,2012-04-06,3,5,0,34,19.6666660308838,6.33333349227905,60,3,2,210,75,1,1,1,1,1,9,978
2594,423,2012-04-06,4,5,0,46,14,0,60,3,2,210,75,1,1,1,1,1,9,978.1989598,1005.05473,4.53177860
2595,423,2012-04-08,0,0,1,22,21.3333339691162,1.33333337306976,44.6666679382324,3,2,210,7
2596,423,2012-04-08,1,0,1,16.3333339691162,38.8333320617676,4.83333349227905,60,3,2,210,7
2597,423,2012-04-08,2,0,1,25.5,33.5,1,60,3,2,210,75,1,1,1,1,1,9,978.1989598,1005.05473,4.531778
2598,423,2012-04-08,3,0,1,35,24.6666660308838,0.333333343267441,60,3,2,210,75,1,1,1,1,1,9,97
2599,423,2012-04-08,4,0,1,38.3333320617676,21.3333339691162,0.333333343267441,60,3,2,210,

2600,423,2012-04-09,0,1,0,23,17.5,1,41.5,3,2,210,75,1,1,1,1,1,9,978.1989598,1005.05473,4.53177
2601,423,2012-04-09,1,1,0,23.3333339691162,34.6666679382324,2,60,3,2,210,75,1,1,1,1,1,9,978.
2602,423,2012-04-09,2,1,0,24.2000007629395,25,10.8000001907349,60,3,2,210,75,1,1,1,1,1,9,978
2603,423,2012-04-09,3,1,0,34.3333320617676,23.3333339691162,2.33333325386047,60,3,2,210,7
2604,423,2012-04-09,4,1,0,47,13,0,60,3,2,210,75,1,1,1,1,1,9,978.1989598,1005.05473,4.53177860
2605,423,2012-04-10,0,2,0,35,20,0.666666686534882,55.6666679382324,3,2,210,75,1,1,1,1,1,9,97
2606,423,2012-04-10,1,2,0,16.7999992370605,31.3999996185303,11.8000001907349,60,3,2,210,7
2607,423,2012-04-10,2,2,0,26.1666660308838,32.3333320617676,1.5,60,3,2,210,75,1,1,1,1,1,9,97
2608,423,2012-04-10,3,2,0,29.6666660308838,27.8333339691162,2.5,60,3,2,210,75,1,1,1,1,1,9,97
2609,423,2012-04-10,4,2,0,33.5,25.1666660308838,1.33333337306976,60,3,2,210,75,1,1,1,1,1,9,97
2610,427,2012-04-07,1,6,1,11,8.33333301544189,0.333333343267441,19.6666660308838,3,2,210,
2611,427,2012-04-07,2,6,1,15,41.6666679382324,3.33333325386047,60,3,2,210,67,0,1,0,1,1,11,95
2612,427,2012-04-07,3,6,1,17.6666660308838,40,2.33333325386047,60,3,2,210,67,0,1,0,1,1,11,95
2613,427,2012-04-07,4,6,1,34,25.3333339691162,0.666666686534882,60,3,2,210,67,0,1,0,1,1,11,9
2614,427,2012-04-08,2,7,1,30,15.3333330154419,1,46.3333320617676,3,2,210,67,0,1,0,1,1,11,957
2615,427,2012-04-08,3,7,1,44.6666679382324,15.3333330154419,0,60,3,2,210,67,0,1,0,1,1,11,957
2616,427,2012-04-08,4,7,1,37.6666679382324,22.3333339691162,0,60,3,2,210,67,0,1,0,1,1,11,957
2617,427,2012-04-09,1,1,0,19,8.66666698455811,0.333333343267441,28,3,2,210,67,0,1,0,1,1,11,9
2618,427,2012-04-09,2,1,0,14.6666669845581,44,1.33333337306976,60,3,2,210,67,0,1,0,1,1,11,95
2619,427,2012-04-09,3,1,0,24.6666660308838,33.3333320617676,2,60,3,2,210,67,0,1,0,1,1,11,957
2620,427,2012-04-09,4,1,0,37.3333320617676,22.3333339691162,0.333333343267441,60,3,2,210,
2621,427,2012-04-10,1,2,0,7.33333349227905,10,0,17.3333339691162,3,2,210,67,0,1,0,1,1,11,957
2622,427,2012-04-10,2,2,0,32.6666679382324,27.3333339691162,0,60,3,2,210,67,0,1,0,1,1,11,957
2623,427,2012-04-10,3,2,0,15.3333330154419,41,3.66666674613953,60,3,2,210,67,0,1,0,1,1,11,95
2624,427,2012-04-10,4,2,0,32.3333320617676,25.6666660308838,2,60,3,2,210,67,0,1,0,1,1,11,957
2625,427,2012-04-11,1,3,0,15,9.66666698455811,0.333333343267441,25,3,2,210,67,0,1,0,1,1,11,9
2626,427,2012-04-11,2,3,0,44.6666679382324,14,1.33333337306976,60,3,2,210,67,0,1,0,1,1,11,95
2627,427,2012-04-11,3,3,0,33,26.3333339691162,0.666666686534882,60,3,2,210,67,0,1,0,1,1,11,9
2628,427,2012-04-11,4,3,0,35.3333320617676,24,0.666666686534882,60,3,2,210,67,0,1,0,1,1,11,9
2629,427,2012-04-12,1,4,0,13.6666669845581,9.66666698455811,0.333333343267441,23.666666
2630,427,2012-04-12,2,4,0,34.6666679382324,24,1.33333337306976,60,3,2,210,67,0,1,0,1,1,11,95
2631,427,2012-04-12,3,4,0,11.6666669845581,39.3333320617676,9,60,3,2,210,67,0,1,0,1,1,11,957
2632,427,2012-04-12,4,4,0,29.3333339691162,26.6666660308838,4,60,3,2,210,67,0,1,0,1,1,11,957
2633,427,2012-04-13,1,5,0,11.6666669845581,8,0,19.6666660308838,3,2,210,67,0,1,0,1,1,11,957.
2634,427,2012-04-13,2,5,0,46.6666679382324,12.3333330154419,1,60,3,2,210,67,0,1,0,1,1,11,957
2635,427,2012-04-13,3,5,0,14.6666669845581,34.3333320617676,11,60,3,2,210,67,0,1,0,1,1,11,95
2636,427,2012-04-13,4,5,0,20,30.6666660308838,9.33333301544189,60,3,2,210,67,0,1,0,1,1,11,95
2637,428,2012-04-04,1,3,0,3.5,30,3.5,37,2,2,210,68,1,1,0,1,1,9,982.8623022,826.5964981,3.05917
2638,428,2012-04-04,2,3,0,13.8333330154419,40,6.16666650772095,60,3,2,210,68,1,1,0,1,1,9,982
2639,428,2012-04-04,3,3,0,19.6000003814697,31.7999992370605,8.60000038146973,60,3,2,210,6
2640,428,2012-04-04,4,3,0,33,22.6666660308838,4.33333349227905,60,3,2,210,68,1,1,0,1,1,9,982
2641,428,2012-04-05,1,4,0,7.40000009536743,33,11.1999998092651,51.5999984741211,3,2,210,6
2642,428,2012-04-05,2,4,0,13.3333330154419,34.6666679382324,12,60,3,2,210,68,1,1,0,1,1,9,982
2643,428,2012-04-05,3,4,0,23.5,28.75,7.75,60,3,2,210,68,1,1,0,1,1,9,982.8623022,826.5964981,3.0
2644,428,2012-04-05,4,4,0,24.5,33.8333320617676,1.66666662693024,60,3,2,210,68,1,1,0,1,1,9,9
2645,428,2012-04-06,1,5,0,8.5,17,8,33.5,2,2,210,68,1,1,0,1,1,9,982.8623022,826.5964981,3.05917
2646,428,2012-04-06,2,5,0,27.5,28,4.5,60,3,2,210,68,1,1,0,1,1,9,982.8623022,826.5964981,3.0591
2647,428,2012-04-06,3,5,0,29.8333339691162,25.5,4.66666650772095,60,3,2,210,68,1,1,0,1,1,9,9
2648,428,2012-04-06,4,5,0,18,24,1,43,3,2,210,68,1,1,0,1,1,9,982.8623022,826.5964981,3.0591712
2649,428,2012-04-07,0,6,1,4.5,22.5,4,31,2,2,210,68,1,1,0,1,1,9,982.8623022,826.5964981,3.05917

2650,428,2012-04-07,1,6,1,2.66666674613953,50.6666679382324,6.66666650772095,60,3,2,210,6
2651,428,2012-04-07,2,6,1,13.6666669845581,39,7.33333349227905,60,3,2,210,68,1,1,0,1,1,9,982
2652,428,2012-04-07,3,6,1,8,50.3333320617676,1.66666662693024,60,3,2,210,68,1,1,0,1,1,9,982.
2653,428,2012-04-07,4,6,1,26.6000003814697,29.3999996185303,4,60,3,2,210,68,1,1,0,1,1,9,982.
2654,428,2012-04-08,4,0,1,59,1,0,60,1,2,210,68,1,1,0,1,1,9,982.8623022,826.5964981,3.05917122
2655,428,2012-04-10,1,2,0,22,20,2.66666674613953,44.6666679382324,3,2,210,68,1,1,0,1,1,9,982
2656,429,2012-03-16,3,5,0,7,48,5,60,1,2,210,69,1,1,1,1,1,12,942.3434571,1028.103305,2.8288924
2657,429,2012-03-16,4,5,0,36.6666679382324,19.3333339691162,4,60,3,2,210,69,1,1,1,1,1,12,942
2658,429,2012-04-08,1,0,1,14.6000003814697,24,3,41.5999984741211,3,2,210,69,1,1,1,1,1,12,942
2659,429,2012-04-08,2,0,1,13.8333330154419,40,6.16666650772095,60,3,2,210,69,1,1,1,1,1,12,94
2660,429,2012-04-08,3,0,1,19.25,33,7.75,60,3,2,210,69,1,1,1,1,1,12,942.3434571,1028.103305,2.8
2661,429,2012-04-08,4,0,1,33,22.6666660308838,4.33333349227905,60,3,2,210,69,1,1,1,1,1,12,94
2662,429,2012-04-09,1,1,0,8.25,30,11.25,49.5,3,2,210,69,1,1,1,1,1,12,942.3434571,1028.103305,2.
2663,429,2012-04-09,2,1,0,13.3333330154419,34.6666679382324,12,60,3,2,210,69,1,1,1,1,1,12,94
2664,429,2012-04-09,3,1,0,23,28.3999996185303,8.60000038146973,60,3,2,210,69,1,1,1,1,1,12,94
2665,429,2012-04-09,4,1,0,24.5,33.8333320617676,1.66666662693024,60,3,2,210,69,1,1,1,1,1,12,
2666,429,2012-04-10,1,2,0,7,26.3333339691162,9,42.3333320617676,3,2,210,69,1,1,1,1,1,12,942.
2667,429,2012-04-10,2,2,0,22.3999996185303,32.4000015258789,5.19999980926514,60,3,2,210,6
2668,429,2012-04-10,3,2,0,36,24,0,60,3,2,210,69,1,1,1,1,1,12,942.3434571,1028.103305,2.828892
2669,429,2012-04-10,4,2,0,18,24,1,43,3,2,210,69,1,1,1,1,1,12,942.3434571,1028.103305,2.828892
2670,429,2012-04-11,0,3,0,4.5,22.5,4,31,2,2,210,69,1,1,1,1,1,12,942.3434571,1028.103305,2.8288
2671,429,2012-04-11,1,3,0,2.66666674613953,50.6666679382324,6.66666650772095,60,3,2,210,6
2672,429,2012-04-11,2,3,0,23.5,30.75,5.75,60,3,2,210,69,1,1,1,1,1,12,942.3434571,1028.103305,2.
2673,429,2012-04-11,3,3,0,17.6000003814697,36.7999992370605,5.59999990463257,60,3,2,210,6
2674,429,2012-04-11,4,3,0,27.3333339691162,30,2.66666674613953,60,3,2,210,69,1,1,1,1,1,12,94
2675,439,2012-03-16,0,5,0,11,34,15,60,1,2,210,72,1,1,0,1,1,12,952.8407981,946.0953896,3.06364
2676,439,2012-03-16,1,5,0,13.3333330154419,26.6666660308838,20,60,3,2,210,72,1,1,0,1,1,12,95
2677,439,2012-03-16,2,5,0,13.6666669845581,41.3333320617676,5,60,3,2,210,72,1,1,0,1,1,12,952
2678,439,2012-03-16,3,5,0,18.3333339691162,39,2.66666674613953,60,3,2,210,72,1,1,0,1,1,12,95
2679,439,2012-03-16,4,5,0,17,41,2,60,2,2,210,72,1,1,0,1,1,12,952.8407981,946.0953896,3.063640
2680,439,2012-03-17,0,6,1,20.5,20.25,7,47.75,3,2,210,72,1,1,0,1,1,12,952.8407981,946.0953896,3.
2681,439,2012-03-17,1,6,1,11,40.5,8.5,60,3,2,210,72,1,1,0,1,1,12,952.8407981,946.0953896,3.063
2682,439,2012-03-17,2,6,1,10.6666669845581,34,15.3333330154419,60,3,2,210,72,1,1,0,1,1,12,95
2683,439,2012-03-17,3,6,1,7.33333349227905,31.6666660308838,21,60,3,2,210,72,1,1,0,1,1,12,95
2684,439,2012-03-17,4,6,1,44.25,14.75,1,60,3,2,210,72,1,1,0,1,1,12,952.8407981,946.0953896,3.0
2685,439,2012-03-18,0,0,1,5,49,5,59,1,2,210,72,1,1,0,1,1,12,952.8407981,946.0953896,3.0636402
2686,439,2012-03-18,1,0,1,15.3999996185303,43.2000007629395,1.39999997615814,60,3,2,210,7
2687,439,2012-03-18,2,0,1,13.8333330154419,43.3333320617676,2.83333325386047,60,3,2,210,7
2688,439,2012-03-18,3,0,1,12.5,29,18.5,60,3,2,210,72,1,1,0,1,1,12,952.8407981,946.0953896,3.063
2689,439,2012-03-18,4,0,1,1,5,2,8,1,2,210,72,1,1,0,1,1,12,952.8407981,946.0953896,3.063640233,
2690,439,2012-03-19,0,1,0,1.5,20.5,6.5,28.5,2,2,210,72,1,1,0,1,1,12,952.8407981,946.0953896,3.0
2691,439,2012-03-19,1,1,0,10.1666669845581,35.3333320617676,14.5,60,3,2,210,72,1,1,0,1,1,12,
2692,439,2012-03-19,2,1,0,13,44.25,2.75,60,3,2,210,72,1,1,0,1,1,12,952.8407981,946.0953896,3.0
2693,439,2012-03-19,3,1,0,3.79999995231628,34.2000007629395,22,60,3,2,210,72,1,1,0,1,1,12,95
2694,439,2012-03-19,4,1,0,27.1666660308838,31.3333339691162,1.5,60,3,2,210,72,1,1,0,1,1,12,9
2695,439,2012-03-20,0,2,0,30.6666660308838,13,6,49.6666679382324,3,2,210,72,1,1,0,1,1,12,952
2696,439,2012-03-20,1,2,0,9,40.6666679382324,10.3333330154419,60,3,2,210,72,1,1,0,1,1,12,952
2697,439,2012-03-20,2,2,0,25,22.5,12.5,60,3,2,210,72,1,1,0,1,1,12,952.8407981,946.0953896,3.063
2698,439,2012-03-20,3,2,0,2,24.3333339691162,33.6666679382324,60,3,2,210,72,1,1,0,1,1,12,952
2699,439,2012-03-20,4,2,0,47.3333320617676,12.6666669845581,0,60,3,2,210,72,1,1,0,1,1,12,952

2700,439,2012-03-21,2,3,0,3,24,33,60,1,2,210,72,1,1,0,1,1,12,952.8407981,946.0953896,3.063640
2701,439,2012-03-21,3,3,0,27.6666660308838,27,5.33333349227905,60,3,2,210,72,1,1,0,1,1,12,95
2702,439,2012-03-21,4,3,0,46.3333320617676,13,0.666666686534882,60,3,2,210,72,1,1,0,1,1,12,9
2703,453,2012-02-23,0,4,0,8,40.5,0,48.5,2,2,210,89,0,1,0,1,1,10,925.3891482,1300.175058,4.5488
2704,453,2012-02-23,1,4,0,15.3333330154419,44,0.666666686534882,60,3,2,210,89,0,1,0,1,1,10,9
2705,453,2012-02-23,2,4,0,32.3333320617676,27.3333339691162,0.333333343267441,60,3,2,210,
2706,453,2012-02-23,3,4,0,21.3333339691162,37.3333320617676,1.33333337306976,60,3,2,210,8
2707,453,2012-02-23,4,4,0,31.6666660308838,28.3333339691162,0,60,3,2,210,89,0,1,0,1,1,10,925
2708,453,2012-02-24,0,5,0,16.6666660308838,36,1.33333337306976,54,3,2,210,89,0,1,0,1,1,10,92
2709,453,2012-02-24,1,5,0,20.6666660308838,39.3333320617676,0,60,3,2,210,89,0,1,0,1,1,10,925
2710,453,2012-02-24,2,5,0,32.3333320617676,27.3333339691162,0.333333343267441,60,3,2,210,
2711,453,2012-02-24,3,5,0,24.3333339691162,34.3333320617676,1.33333337306976,60,3,2,210,8
2712,453,2012-02-24,4,5,0,32.6666679382324,27.3333339691162,0,60,3,2,210,89,0,1,0,1,1,10,925
2713,453,2012-02-25,0,6,1,18,32.6666679382324,0.666666686534882,51.3333320617676,3,2,210,
2714,453,2012-02-25,1,6,1,24.3333339691162,34.6666679382324,1,60,3,2,210,89,0,1,0,1,1,10,925
2715,453,2012-02-25,2,6,1,31.6666660308838,28,0.333333343267441,60,3,2,210,89,0,1,0,1,1,10,9
2716,453,2012-02-25,3,6,1,38.6666679382324,21.3333339691162,0,60,3,2,210,89,0,1,0,1,1,10,925
2717,453,2012-02-25,4,6,1,19,40.6666679382324,0.333333343267441,60,3,2,210,89,0,1,0,1,1,10,9
2718,453,2012-02-26,0,0,1,7.5,32,1,40.5,2,2,210,89,0,1,0,1,1,10,925.3891482,1300.175058,4.5488
2719,453,2012-02-26,1,0,1,20.1666660308838,39.8333320617676,0,60,3,2,210,89,0,1,0,1,1,10,925
2720,453,2012-02-26,2,0,1,34.25,25.75,0,60,3,2,210,89,0,1,0,1,1,10,925.3891482,1300.175058,4.5
2721,453,2012-02-26,3,0,1,28,32,0,60,3,2,210,89,0,1,0,1,1,10,925.3891482,1300.175058,4.548863
2722,453,2012-02-26,4,0,1,32.3333320617676,27.3333339691162,0.333333343267441,60,3,2,210,
2723,453,2012-02-27,0,1,0,10.5,33.5,1.5,45.5,2,2,210,89,0,1,0,1,1,10,925.3891482,1300.175058,4.
2724,453,2012-02-27,1,1,0,20,39.6666679382324,0.333333343267441,60,3,2,210,89,0,1,0,1,1,10,9
2725,453,2012-02-27,2,1,0,40.75,19,0.25,60,3,2,210,89,0,1,0,1,1,10,925.3891482,1300.175058,4.5
2726,453,2012-02-27,3,1,0,33.3333320617676,26.6666660308838,0,60,3,2,210,89,0,1,0,1,1,10,925
2727,453,2012-02-27,4,1,0,32,28,0,60,3,2,210,89,0,1,0,1,1,10,925.3891482,1300.175058,4.548863
2728,453,2012-02-28,0,2,0,6,40.5,0.5,47,2,2,210,89,0,1,0,1,1,10,925.3891482,1300.175058,4.5488
2729,453,2012-02-28,1,2,0,25.6666660308838,33.6666679382324,0.666666686534882,60,3,2,210,
2730,453,2012-02-28,2,2,0,43.25,16.5,0.25,60,3,2,210,89,0,1,0,1,1,10,925.3891482,1300.175058,4.
2731,453,2012-02-28,3,2,0,34.3333320617676,25,0.666666686534882,60,3,2,210,89,0,1,0,1,1,10,9
2732,453,2012-02-28,4,2,0,32,28,0,60,3,2,210,89,0,1,0,1,1,10,925.3891482,1300.175058,4.548863
2733,453,2012-02-29,0,3,0,14,33,2,49,2,2,210,89,0,1,0,1,1,10,925.3891482,1300.175058,4.548863
2734,453,2012-02-29,1,3,0,20,39.3333320617676,0.666666686534882,60,3,2,210,89,0,1,0,1,1,10,9
2735,453,2012-02-29,2,3,0,45.6666679382324,14.3333330154419,0,60,3,2,210,89,0,1,0,1,1,10,925
2736,453,2012-02-29,3,3,0,34.6666679382324,25.3333339691162,0,60,3,2,210,89,0,1,0,1,1,10,925
2737,453,2012-02-29,4,3,0,30.3333339691162,29.3333339691162,0.333333343267441,60,3,2,210,
2738,453,2012-03-16,4,5,0,22,38,0,60,1,2,210,89,0,1,0,1,1,10,925.3891482,1300.175058,4.548863
2739,453,2012-03-21,0,3,0,15.25,37.5,1,53.75,3,2,210,89,0,1,0,1,1,10,925.3891482,1300.175058,4.
2740,453,2012-03-21,1,3,0,20.3333339691162,39.6666679382324,0,60,3,2,210,89,0,1,0,1,1,10,925
2741,453,2012-03-21,2,3,0,34,25.5,0.5,60,3,2,210,89,0,1,0,1,1,10,925.3891482,1300.175058,4.548
2742,453,2012-03-21,3,3,0,32.3333320617676,27,0.666666686534882,60,3,2,210,89,0,1,0,1,1,10,9
2743,453,2012-03-21,4,3,0,35.3333320617676,24,0.666666686534882,60,3,2,210,89,0,1,0,1,1,10,9
2744,453,2012-03-22,0,4,0,8.80000019073486,32.5999984741211,0.400000005960464,41.799999
2745,453,2012-03-22,1,4,0,19.3333339691162,39.5,1.16666662693024,60,3,2,210,89,0,1,0,1,1,10,
2746,453,2012-03-22,2,4,0,37.1666679382324,22.8333339691162,0,60,3,2,210,89,0,1,0,1,1,10,925
2747,453,2012-03-22,3,4,0,32.1428565979004,27.5714282989502,0.28571429848671,60,3,2,210,8
2748,453,2012-03-22,4,4,0,27,33,0,60,3,2,210,89,0,1,0,1,1,10,925.3891482,1300.175058,4.548863
2749,453,2012-03-23,0,5,0,7,28.5,0,35.5,2,2,210,89,0,1,0,1,1,10,925.3891482,1300.175058,4.5488

2750,453,2012-03-23,1,5,0,27.3333339691162,31.6666660308838,1,60,3,2,210,89,0,1,0,1,1,10,925
2751,453,2012-03-23,2,5,0,43,17,0,60,3,2,210,89,0,1,0,1,1,10,925.3891482,1300.175058,4.5488636
2752,453,2012-03-23,3,5,0,40.3333320617676,19.6666660308838,0,60,3,2,210,89,0,1,0,1,1,10,925
2753,453,2012-03-23,4,5,0,34.3333320617676,25.6666660308838,0,60,3,2,210,89,0,1,0,1,1,10,925
2754,453,2012-03-24,0,6,1,10.5,39,0.5,50,2,2,210,89,0,1,0,1,1,10,925.3891482,1300.175058,4.5488
2755,453,2012-03-24,1,6,1,16.6666660308838,43.3333320617676,0,60,3,2,210,89,0,1,0,1,1,10,925
2756,453,2012-03-24,2,6,1,37.6666679382324,22.3333339691162,0,60,3,2,210,89,0,1,0,1,1,10,925
2757,453,2012-03-24,3,6,1,43.25,16.75,0,60,3,2,210,89,0,1,0,1,1,10,925.3891482,1300.175058,4.54
2758,453,2012-03-24,4,6,1,33.3333320617676,26.6666660308838,0,60,3,2,210,89,0,1,0,1,1,10,925
2759,453,2012-03-25,0,0,1,10,32,2,44,2,2,210,89,0,1,0,1,1,10,925.3891482,1300.175058,4.5488636
2760,453,2012-03-25,1,0,1,21.3333339691162,37.3333320617676,1.33333337306976,60,3,2,210,8
2761,453,2012-03-25,2,0,1,37.3333320617676,22.6666660308838,0,60,3,2,210,89,0,1,0,1,1,10,925
2762,453,2012-03-25,3,0,1,36,23.6666660308838,0.333333343267441,60,3,2,210,89,0,1,0,1,1,10,9
2763,453,2012-03-25,4,0,1,26,33.6666679382324,0.333333343267441,60,3,2,210,89,0,1,0,1,1,10,9
2764,453,2012-03-26,0,1,0,11.5,38,0.5,50,2,2,210,89,0,1,0,1,1,10,925.3891482,1300.175058,4.5488
2765,453,2012-03-26,1,1,0,19,41,0,60,3,2,210,89,0,1,0,1,1,10,925.3891482,1300.175058,4.5488636
2766,453,2012-03-26,2,1,0,36,24,0,60,3,2,210,89,0,1,0,1,1,10,925.3891482,1300.175058,4.5488636
2767,453,2012-03-26,3,1,0,36,24,0,60,3,2,210,89,0,1,0,1,1,10,925.3891482,1300.175058,4.5488636
2768,453,2012-03-26,4,1,0,30.6666660308838,29.3333339691162,0,60,3,2,210,89,0,1,0,1,1,10,925
2769,453,2012-03-27,0,2,0,16,32,0.5,48.5,2,2,210,89,0,1,0,1,1,10,925.3891482,1300.175058,4.5488
2770,453,2012-03-27,1,2,0,22.6666660308838,36.3333320617676,1,60,3,2,210,89,0,1,0,1,1,10,925
2771,453,2012-03-27,2,2,0,41,19,0,60,3,2,210,89,0,1,0,1,1,10,925.3891482,1300.175058,4.5488636
2772,453,2012-03-27,3,2,0,31,29,0,60,3,2,210,89,0,1,0,1,1,10,925.3891482,1300.175058,4.5488636
2773,453,2012-03-27,4,2,0,36.2000007629395,23.7999992370605,0,60,3,2,210,89,0,1,0,1,1,10,925
2774,458,2012-03-16,0,5,0,26,9,1,36,2,2,212,77,1,0,0,1,1,10,1607.130336,1787.467877,4.10697485
2775,458,2012-03-16,1,5,0,14,43.3333320617676,2.66666674613953,60,3,2,212,77,1,0,0,1,1,10,16
2776,458,2012-03-16,2,5,0,17.6666660308838,42,0.333333343267441,60,3,2,212,77,1,0,0,1,1,10,1
2777,458,2012-03-16,3,5,0,10,49,1,60,3,2,212,77,1,0,0,1,1,10,1607.130336,1787.467877,4.1069748
2778,458,2012-03-16,4,5,0,14,38,2,54,3,2,212,77,1,0,0,1,1,10,1607.130336,1787.467877,4.1069748
2779,458,2012-03-17,0,6,1,15.3333330154419,9,0.333333343267441,24.6666660308838,3,2,212,7
2780,458,2012-03-17,1,6,1,17.6666660308838,39,3.33333325386047,60,3,2,212,77,1,0,0,1,1,10,16
2781,458,2012-03-17,2,6,1,28.3333339691162,31,0.666666686534882,60,3,2,212,77,1,0,0,1,1,10,1
2782,458,2012-03-17,3,6,1,5,51.3333320617676,3.66666674613953,60,3,2,212,77,1,0,0,1,1,10,160
2783,458,2012-03-17,4,6,1,22.3333339691162,36,1.66666662693024,60,3,2,212,77,1,0,0,1,1,10,16
2784,458,2012-03-18,0,0,1,11,23.5,3,37.5,2,2,212,77,1,0,0,1,1,10,1607.130336,1787.467877,4.1069
2785,458,2012-03-18,1,0,1,25.75,30,2.75,58.5,3,2,212,77,1,0,0,1,1,10,1607.130336,1787.467877,4.
2786,458,2012-03-18,2,0,1,32,27.3333339691162,0.666666686534882,60,3,2,212,77,1,0,0,1,1,10,1
2787,458,2012-03-18,3,0,1,16.3333339691162,43.6666679382324,0,60,3,2,212,77,1,0,0,1,1,10,160
2788,458,2012-03-18,4,0,1,33.3333320617676,26.6666660308838,0,60,3,2,212,77,1,0,0,1,1,10,160
2789,458,2012-03-19,0,1,0,27,18,1.5,46.5,2,2,212,77,1,0,0,1,1,10,1607.130336,1787.467877,4.1069
2790,458,2012-03-19,1,1,0,15,41.75,3.25,60,3,2,212,77,1,0,0,1,1,10,1607.130336,1787.467877,4.10
2791,458,2012-03-19,2,1,0,21,30.6666660308838,8.33333301544189,60,3,2,212,77,1,0,0,1,1,10,16
2792,458,2012-03-19,3,1,0,9.33333301544189,17.3333339691162,33.3333320617676,60,3,2,212,7
2793,458,2012-03-19,4,1,0,10.6666669845581,35,14.3333330154419,60,3,2,212,77,1,0,0,1,1,10,16
2794,458,2012-03-20,0,2,0,25,9,0.5,34.5,2,2,212,77,1,0,0,1,1,10,1607.130336,1787.467877,4.10697
2795,458,2012-03-20,1,2,0,16.25,39.25,4.5,60,3,2,212,77,1,0,0,1,1,10,1607.130336,1787.467877,4.
2796,458,2012-03-20,2,2,0,17.25,39.5,3.25,60,3,2,212,77,1,0,0,1,1,10,1607.130336,1787.467877,4.
2797,458,2012-03-20,3,2,0,18,34.3333320617676,7.66666650772095,60,3,2,212,77,1,0,0,1,1,10,16
2798,458,2012-03-20,4,2,0,17,25.3333339691162,0.666666686534882,43,3,2,212,77,1,0,0,1,1,10,1
2799,458,2012-03-21,0,3,0,17,38,5,60,2,2,212,77,1,0,0,1,1,10,1607.130336,1787.467877,4.1069748

2800,458,2012-03-21,1,3,0,2.66666674613953,52,5.33333349227905,60,3,2,212,77,1,0,0,1,1,10,16
2801,458,2012-03-21,2,3,0,10.1999998092651,39,10.8000001907349,60,3,2,212,77,1,0,0,1,1,10,16
2802,458,2012-03-21,3,3,0,3.5,44.25,12.25,60,3,2,212,77,1,0,0,1,1,10,1607.130336,1787.467877,4.
2803,458,2012-03-21,4,3,0,10,40.6666679382324,9.33333301544189,60,3,2,212,77,1,0,0,1,1,10,16
2804,458,2012-03-22,0,4,0,19,41,0,60,1,2,212,77,1,0,0,1,1,10,1607.130336,1787.467877,4.106974
2805,458,2012-03-22,1,4,0,25.6666660308838,33.3333320617676,1,60,3,2,212,77,1,0,0,1,1,10,160
2806,458,2012-03-22,2,4,0,27.6666660308838,27.3333339691162,5,60,3,2,212,77,1,0,0,1,1,10,160
2807,458,2012-03-22,3,4,0,10.8000001907349,40.2000007629395,9,60,3,2,212,77,1,0,0,1,1,10,160
2808,458,2012-03-22,4,4,0,7.75,35.25,5.75,48.75,3,2,212,77,1,0,0,1,1,10,1607.130336,1787.46787
2809,458,2012-03-23,4,5,0,31.5,27.5,1,60,2,2,212,77,1,0,0,1,1,10,1607.130336,1787.467877,4.106
2810,460,2012-03-20,0,2,0,25.25,20.5,0.25,46,3,2,212,79,0,0,1,0,1,12,1625.561504,1874.953605,4.
2811,460,2012-03-20,1,2,0,39.6666679382324,18.6666660308838,1.6666662693024,60,3,2,212,7
2812,460,2012-03-20,2,2,0,32,19,9,60,3,2,212,79,0,0,1,0,1,12,1625.561504,1874.953605,4.009862
2813,460,2012-03-20,3,2,0,39,20.3333339691162,0.666666686534882,60,3,2,212,79,0,0,1,0,1,12,1
2814,460,2012-03-20,4,2,0,52.6666679382324,7.33333349227905,0,60,3,2,212,79,0,0,1,0,1,12,162
2815,460,2012-03-21,0,3,0,38.4000015258789,21.3999996185303,0.200000002980232,60,3,2,212,
2816,460,2012-03-21,1,3,0,30.3333339691162,29,0.666666686534882,60,3,2,212,79,0,0,1,0,1,12,1
2817,460,2012-03-21,2,3,0,37.3333320617676,22.3333339691162,0.333333343267441,60,3,2,212,
2818,460,2012-03-21,3,3,0,23.3333339691162,26.3333339691162,10.3333330154419,60,3,2,212,7
2819,460,2012-03-21,4,3,0,53.3333320617676,6.66666650772095,0,60,3,2,212,79,0,0,1,0,1,12,162
2820,460,2012-03-22,0,4,0,22.6666660308838,37.3333320617676,0,60,3,2,212,79,0,0,1,0,1,12,162
2821,460,2012-03-22,1,4,0,37,22.6000003814697,0.400000005960464,60,3,2,212,79,0,0,1,0,1,12,1
2822,460,2012-03-22,2,4,0,31,26.6666660308838,2.33333325386047,60,3,2,212,79,0,0,1,0,1,12,16
2823,460,2012-03-22,3,4,0,46.6666679382324,13.3333330154419,0,60,3,2,212,79,0,0,1,0,1,12,162
2824,460,2012-03-22,4,4,0,47,7.66666650772095,0,54.6666679382324,3,2,212,79,0,0,1,0,1,12,162
2825,460,2012-03-23,0,5,0,27.5,29.5,0,57,3,2,212,79,0,0,1,0,1,12,1625.561504,1874.953605,4.009
2826,460,2012-03-23,1,5,0,37.8571434020996,21.4285717010498,0.714285731315613,60,3,2,212,
2827,460,2012-03-23,2,5,0,34.75,21.625,3.625,60,3,2,212,79,0,0,1,0,1,12,1625.561504,1874.95360
2828,460,2012-03-23,3,5,0,37.5,21,1.5,60,3,2,212,79,0,0,1,0,1,12,1625.561504,1874.953605,4.009
2829,460,2012-03-23,4,5,0,44.5999984741211,15.3999996185303,0,60,3,2,212,79,0,0,1,0,1,12,162
2830,460,2012-03-24,0,6,1,24.3333339691162,17.3333339691162,0.333333343267441,42,3,2,212,
2831,460,2012-03-24,1,6,1,36,24,0,60,3,2,212,79,0,0,1,0,1,12,1625.561504,1874.953605,4.009862
2832,460,2012-03-24,2,6,1,39.75,20,0.25,60,3,2,212,79,0,0,1,0,1,12,1625.561504,1874.953605,4.00
2833,460,2012-03-24,3,6,1,35.1666679382324,24,0.833333313465118,60,3,2,212,79,0,0,1,0,1,12,1
2834,460,2012-03-24,4,6,1,43.8333320617676,13.3333330154419,0.333333343267441,57.5,3,2,21
2835,460,2012-03-25,0,0,1,30.5,26,0.5,57,2,2,212,79,0,0,1,0,1,12,1625.561504,1874.953605,4.009
2836,460,2012-03-25,1,0,1,32,21.3333339691162,6.66666650772095,60,3,2,212,79,0,0,1,0,1,12,16
2837,460,2012-03-25,2,0,1,40.6666679382324,19.3333339691162,0,60,3,2,212,79,0,0,1,0,1,12,162
2838,460,2012-03-25,3,0,1,40.3333320617676,18.3333339691162,1.33333337306976,60,3,2,212,7
2839,460,2012-03-25,4,0,1,47.25,12.75,0,60,3,2,212,79,0,0,1,0,1,12,1625.561504,1874.953605,4.00
2840,462,2012-03-01,0,4,0,5,26,0,31,2,2,212,67,1,0,0,1,1,9,1548.69824,1731.923397,2.063125839,
2841,462,2012-03-01,1,4,0,24.6666660308838,31.6666660308838,3.66666674613953,60,3,2,212,6
2842,462,2012-03-01,2,4,0,35.5,23.25,1.25,60,3,2,212,67,1,0,0,1,1,9,1548.69824,1731.923397,2.06
2843,462,2012-03-01,3,4,0,48,12,0,60,3,2,212,67,1,0,0,1,1,9,1548.69824,1731.923397,2.06312583
2844,462,2012-03-01,4,4,0,28,30.6666660308838,1.33333337306976,60,3,2,212,67,1,0,0,1,1,9,154
2845,462,2012-03-02,0,5,0,24,32,2,58,2,2,212,67,1,0,0,1,1,9,1548.69824,1731.923397,2.06312583
2846,462,2012-03-02,1,5,0,42,17,1,60,3,2,212,67,1,0,0,1,1,9,1548.69824,1731.923397,2.06312583
2847,462,2012-03-02,2,5,0,49.3333320617676,10.3333330154419,0.333333343267441,60,3,2,212,
2848,462,2012-03-02,3,5,0,41.2000007629395,15.3999996185303,3.40000009536743,60,3,2,212,6
2849,462,2012-03-02,4,5,0,47.3333320617676,12.6666669845581,0,60,3,2,212,67,1,0,0,1,1,9,1548

2850,462,2012-03-03,4,6,1,29.6666660308838,30,0.333333343267441,60,3,2,212,67,1,0,0,1,1,9,15
2851,462,2012-03-24,0,6,1,21,37,2,60,1,2,212,67,1,0,0,1,1,9,1548.69824,1731.923397,2.063125839
2852,462,2012-03-24,1,6,1,34.6666679382324,25.3333339691162,0,60,3,2,212,67,1,0,0,1,1,9,1548
2853,462,2012-03-24,2,6,1,45.6666679382324,13.6666669845581,0.666666686534882,60,3,2,212,
2854,462,2012-03-24,3,6,1,43,13,4,60,1,2,212,67,1,0,0,1,1,9,1548.69824,1731.923397,2.063125839
2855,462,2012-03-25,0,0,1,3,1,1,5,1,2,212,67,1,0,0,1,1,9,1548.69824,1731.923397,2.063125839,5.
2856,462,2012-03-25,1,0,1,12.6666669845581,47.3333320617676,0,60,3,2,212,67,1,0,0,1,1,9,1548
2857,462,2012-03-25,2,0,1,8.5,24.5,0,33,2,2,212,67,1,0,0,1,1,9,1548.69824,1731.923397,2.0631258
2858,462,2012-03-25,3,0,1,40.4000015258789,18.7999992370605,0.800000011920929,60,3,2,212,
2859,462,2012-03-25,4,0,1,41.5,17.6666660308838,0.833333313465118,60,3,2,212,67,1,0,0,1,1,9,1
2860,462,2012-03-26,0,1,0,10,11,0,21,1,2,212,67,1,0,0,1,1,9,1548.69824,1731.923397,2.063125839
2861,462,2012-03-26,1,1,0,19,40.2000007629395,0.400000005960464,59.5999984741211,3,2,212,
2862,462,2012-03-26,2,1,0,33.7999992370605,25.6000003814697,0.600000023841858,60,3,2,212,
2863,462,2012-03-26,3,1,0,28,30.6666660308838,1.33333337306976,60,3,2,212,67,1,0,0,1,1,9,154
2864,462,2012-03-26,4,1,0,49.3333320617676,9,1.66666662693024,60,3,2,212,67,1,0,0,1,1,9,1548
2865,464,2012-03-03,0,6,1,10.6666669845581,32.3333320617676,0.333333343267441,43.333332
2866,464,2012-03-03,1,6,1,17,32.3333320617676,10.6666669845581,60,3,2,212,75,0,0,0,1,1,10,16
2867,464,2012-03-03,2,6,1,25.6666660308838,26,8.33333301544189,60,3,2,212,75,0,0,0,1,1,10,16
2868,464,2012-03-03,3,6,1,27,31.3333339691162,1.66666662693024,60,3,2,212,75,0,0,0,1,1,10,16
2869,464,2012-03-03,4,6,1,38,22,0,60,3,2,212,75,0,0,0,1,1,10,1607.130336,1787.467877,4.1069748
2870,464,2012-03-04,0,0,1,21.25,23.25,3.25,47.75,3,2,212,75,0,0,0,1,1,10,1607.130336,1787.4678
2871,464,2012-03-04,1,0,1,17.3333339691162,40,2.66666674613953,60,3,2,212,75,0,0,0,1,1,10,16
2872,464,2012-03-04,2,0,1,36,24,0,60,3,2,212,75,0,0,0,1,1,10,1607.130336,1787.467877,4.1069748
2873,464,2012-03-04,3,0,1,16,41.6666679382324,2.33333325386047,60,3,2,212,75,0,0,0,1,1,10,16
2874,464,2012-03-04,4,0,1,33.3333320617676,25,1.66666662693024,60,3,2,212,75,0,0,0,1,1,10,16
2875,464,2012-03-05,0,1,0,6.5,46.5,5.5,58.5,2,2,212,75,0,0,0,1,1,10,1607.130336,1787.467877,4.10
2876,464,2012-03-05,1,1,0,25.75,31.5,2.75,60,3,2,212,75,0,0,0,1,1,10,1607.130336,1787.467877,4.
2877,464,2012-03-05,2,1,0,32.3333320617676,27.3333339691162,0.333333343267441,60,3,2,212,
2878,464,2012-03-05,3,1,0,12,47.3333320617676,0.666666686534882,60,3,2,212,75,0,0,0,1,1,10,1
2879,464,2012-03-05,4,1,0,42,17.6666660308838,0.333333343267441,60,3,2,212,75,0,0,0,1,1,10,1
2880,464,2012-03-06,0,2,0,4,43,1,48,2,2,212,75,0,0,0,1,1,10,1607.130336,1787.467877,4.10697485
2881,464,2012-03-06,1,2,0,12.6000003814697,42.2000007629395,5.19999980926514,60,3,2,212,7
2882,464,2012-03-06,2,2,0,40.2000007629395,17.3999996185303,2.40000009536743,60,3,2,212,7
2883,464,2012-03-06,3,2,0,9.33333301544189,28.6666660308838,22,60,3,2,212,75,0,0,0,1,1,10,16
2884,464,2012-03-06,4,2,0,36.6666679382324,18.3333339691162,5,60,3,2,212,75,0,0,0,1,1,10,160
2885,464,2012-03-07,0,3,0,5.5,41,4.5,51,2,2,212,75,0,0,0,1,1,10,1607.130336,1787.467877,4.10697
2886,464,2012-03-07,1,3,0,5.66666650772095,51.6666679382324,2.66666674613953,60,3,2,212,7
2887,464,2012-03-07,2,3,0,29.25,29.5,1.25,60,3,2,212,75,0,0,0,1,1,10,1607.130336,1787.467877,4.
2888,464,2012-03-07,3,3,0,24.25,35.25,0.5,60,3,2,212,75,0,0,0,1,1,10,1607.130336,1787.467877,4.
2889,464,2012-03-07,4,3,0,28.6666660308838,31.3333339691162,0,60,3,2,212,75,0,0,0,1,1,10,160
2890,464,2012-03-08,0,4,0,8,32,0.333333343267441,40.3333320617676,3,2,212,75,0,0,0,1,1,10,16
2891,464,2012-03-08,1,4,0,8.66666698455811,46,5.33333349227905,60,3,2,212,75,0,0,0,1,1,10,16
2892,464,2012-03-08,2,4,0,28,31.3333339691162,0.666666686534882,60,3,2,212,75,0,0,0,1,1,10,1
2893,464,2012-03-08,3,4,0,13.5,44.25,2.25,60,3,2,212,75,0,0,0,1,1,10,1607.130336,1787.467877,4.
2894,464,2012-03-08,4,4,0,25.6666660308838,31.3333339691162,3,60,3,2,212,75,0,0,0,1,1,10,160
2895,464,2012-03-09,0,5,0,0,54,6,60,1,2,212,75,0,0,0,1,1,10,1607.130336,1787.467877,4.10697485
2896,464,2012-03-09,1,5,0,6.33333349227905,51.3333320617676,2.33333325386047,60,3,2,212,7
2897,464,2012-03-09,2,5,0,38.6666679382324,19.6666660308838,1.66666662693024,60,3,2,212,7
2898,464,2012-03-09,3,5,0,13.75,45.25,1,60,3,2,212,75,0,0,0,1,1,10,1607.130336,1787.467877,4.10
2899,464,2012-03-09,4,5,0,30,30,0,60,3,2,212,75,0,0,0,1,1,10,1607.130336,1787.467877,4.1069748

2900,464,2012-03-10,4,6,1,54,6,0,60,1,2,212,75,0,0,0,1,1,10,1607.130336,1787.467877,4.10697485
2901,470,2012-03-10,0,6,1,10,27,1,38,2,2,212,66,1,1,0,1,1,11,1548.69824,1731.923397,2.06312583
2902,470,2012-03-10,1,6,1,28.75,31,0.25,60,3,2,212,66,1,1,0,1,1,11,1548.69824,1731.923397,2.063
2903,470,2012-03-10,2,6,1,28.6666660308838,28.3333339691162,3,60,3,2,212,66,1,1,0,1,1,11,154
2904,470,2012-03-10,3,6,1,32.3333320617676,27,0.666666686534882,60,3,2,212,66,1,1,0,1,1,11,1
2905,470,2012-03-10,4,6,1,23,33,4,60,3,2,212,66,1,1,0,1,1,11,1548.69824,1731.923397,2.06312583
2906,470,2012-03-11,0,0,1,12,43,0,55,1,2,212,66,1,1,0,1,1,11,1548.69824,1731.923397,2.06312583
2907,470,2012-03-11,1,0,1,23.7999992370605,36,0.200000002980232,60,3,2,212,66,1,1,0,1,1,11,1
2908,470,2012-03-11,2,0,1,31.25,28,0.75,60,3,2,212,66,1,1,0,1,1,11,1548.69824,1731.923397,2.063
2909,470,2012-03-11,3,0,1,25.6666660308838,22.3333339691162,12,60,3,2,212,66,1,1,0,1,1,11,15
2910,470,2012-03-11,4,0,1,38,20,2,60,3,2,212,66,1,1,0,1,1,11,1548.69824,1731.923397,2.06312583
2911,470,2012-03-17,0,6,1,26,22,3,51,2,2,212,66,1,1,0,1,1,11,1548.69824,1731.923397,2.06312583
2912,470,2012-03-17,1,6,1,26,32.3333320617676,1.66666662693024,60,3,2,212,66,1,1,0,1,1,11,15
2913,470,2012-03-17,2,6,1,22,37.5,0.5,60,3,2,212,66,1,1,0,1,1,11,1548.69824,1731.923397,2.06312
2914,470,2012-03-17,3,6,1,24,35,1,60,3,2,212,66,1,1,0,1,1,11,1548.69824,1731.923397,2.06312583
2915,470,2012-03-17,4,6,1,30.6666660308838,27.3333339691162,2,60,3,2,212,66,1,1,0,1,1,11,154
2916,470,2012-03-18,0,0,1,9.5,31,2,42.5,2,2,212,66,1,1,0,1,1,11,1548.69824,1731.923397,2.063125
2917,470,2012-03-18,1,0,1,21.3333339691162,38.3333320617676,0.333333343267441,60,3,2,212,
2918,470,2012-03-18,2,0,1,15.5,42.75,1.75,60,3,2,212,66,1,1,0,1,1,11,1548.69824,1731.923397,2.0
2919,470,2012-03-18,3,0,1,28.5,31.5,0,60,3,2,212,66,1,1,0,1,1,11,1548.69824,1731.923397,2.06312
2920,470,2012-03-18,4,0,1,15,42.6666679382324,2.33333325386047,60,3,2,212,66,1,1,0,1,1,11,15
2921,470,2012-03-19,0,1,0,29,27.5,2,58.5,2,2,212,66,1,1,0,1,1,11,1548.69824,1731.923397,2.06312
2922,470,2012-03-19,1,1,0,26.3333339691162,31.6666660308838,2,60,3,2,212,66,1,1,0,1,1,11,154
2923,470,2012-03-19,2,1,0,40.3333320617676,19.3333339691162,0.333333343267441,60,3,2,212,
2924,470,2012-03-19,3,1,0,26.2000007629395,30.7999992370605,3,60,3,2,212,66,1,1,0,1,1,11,154
2925,470,2012-03-19,4,1,0,25.25,34.75,0,60,3,2,212,66,1,1,0,1,1,11,1548.69824,1731.923397,2.063
2926,470,2012-03-20,1,2,0,11.6666669845581,43.6666679382324,0.333333343267441,55.6666679
2927,470,2012-03-20,2,2,0,11.6666669845581,48.3333320617676,0,60,3,2,212,66,1,1,0,1,1,11,154
2928,470,2012-03-20,3,2,0,14,45.3333320617676,0.666666686534882,60,3,2,212,66,1,1,0,1,1,11,1
2929,470,2012-03-20,4,2,0,19.6000003814697,40.2000007629395,0.200000002980232,60,3,2,212,
2930,479,2012-03-17,0,6,1,26,22,3,51,2,2,212,66,1,0,0,1,1,12,1652.358901,1940.685086,4.3777094
2931,479,2012-03-17,1,6,1,27.25,31.5,1.25,60,3,2,212,66,1,0,0,1,1,12,1652.358901,1940.685086,4.
2932,479,2012-03-17,2,6,1,27.6666660308838,32.3333320617676,0,60,3,2,212,66,1,0,0,1,1,12,165
2933,479,2012-03-17,3,6,1,24,35,1,60,3,2,212,66,1,0,0,1,1,12,1652.358901,1940.685086,4.3777094
2934,479,2012-03-17,4,6,1,30.6666660308838,27.3333339691162,2,60,3,2,212,66,1,0,0,1,1,12,165
2935,479,2012-03-18,0,0,1,9.5,31,2,42.5,2,2,212,66,1,0,0,1,1,12,1652.358901,1940.685086,4.37770
2936,479,2012-03-18,1,0,1,21.3333339691162,38.3333320617676,0.333333343267441,60,3,2,212,
2937,479,2012-03-18,2,0,1,18.7999992370605,40,1.20000004768372,60,3,2,212,66,1,0,0,1,1,12,16
2938,479,2012-03-18,3,0,1,35,25,0,60,3,2,212,66,1,0,0,1,1,12,1652.358901,1940.685086,4.3777094
2939,479,2012-03-18,4,0,1,15,42.6666679382324,2.33333325386047,60,3,2,212,66,1,0,0,1,1,12,16
2940,479,2012-03-19,0,1,0,29,27.5,2,58.5,2,2,212,66,1,0,0,1,1,12,1652.358901,1940.685086,4.3777
2941,479,2012-03-19,1,1,0,26.3333339691162,31.6666660308838,2,60,3,2,212,66,1,0,0,1,1,12,165
2942,479,2012-03-19,2,1,0,31.5,27.5,1,60,3,2,212,66,1,0,0,1,1,12,1652.358901,1940.685086,4.3777
2943,479,2012-03-19,3,1,0,24.7999992370605,34.5999984741211,0.600000023841858,60,3,2,212,
2944,479,2012-03-19,4,1,0,26.3333339691162,33.6666679382324,0,60,3,2,212,66,1,0,0,1,1,12,165
2945,479,2012-03-20,0,2,0,19,40,1,60,1,2,212,66,1,0,0,1,1,12,1652.358901,1940.685086,4.3777094
2946,479,2012-03-20,1,2,0,18.75,37.75,0.25,56.75,3,2,212,66,1,0,0,1,1,12,1652.358901,1940.68508
2947,479,2012-03-20,2,2,0,11.6666669845581,48.3333320617676,0,60,3,2,212,66,1,0,0,1,1,12,165
2948,479,2012-03-20,3,2,0,14.5,42,3.5,60,3,2,212,66,1,0,0,1,1,12,1652.358901,1940.685086,4.3777
2949,479,2012-03-20,4,2,0,20,39.8333320617676,0.16666667163372,60,3,2,212,66,1,0,0,1,1,12,16

2950,479,2012-03-21,0,3,0,1,14,1,16,1,2,212,66,1,0,0,1,1,12,1652.358901,1940.685086,4.3777094
2951,479,2012-03-21,1,3,0,22.3999996185303,37.4000015258789,0.200000002980232,60,3,2,212,
2952,479,2012-03-21,2,3,0,28.6666660308838,28.3333339691162,3,60,3,2,212,66,1,0,0,1,1,12,165
2953,479,2012-03-21,3,3,0,32.3333320617676,27,0.666666686534882,60,3,2,212,66,1,0,0,1,1,12,1
2954,479,2012-03-21,4,3,0,23,33,4,60,3,2,212,66,1,0,0,1,1,12,1652.358901,1940.685086,4.377709
2955,479,2012-03-22,0,4,0,12,43,0,55,1,2,212,66,1,0,0,1,1,12,1652.358901,1940.685086,4.377709
2956,479,2012-03-22,1,4,0,25.5,34,0.5,60,2,2,212,66,1,0,0,1,1,12,1652.358901,1940.685086,4.377
2957,479,2012-03-22,2,4,0,31,28,1,60,3,2,212,66,1,0,0,1,1,12,1652.358901,1940.685086,4.377709
2958,479,2012-03-22,3,4,0,25.6666660308838,22.3333339691162,12,60,3,2,212,66,1,0,0,1,1,12,16
2959,479,2012-03-22,4,4,0,38,20,2,60,3,2,212,66,1,0,0,1,1,12,1652.358901,1940.685086,4.377709
2960,484,2012-03-20,0,2,0,36,6,0,42,1,2,212,72,0,1,0,1,1,12,1644.295588,2001.848272,4.7262474
2961,484,2012-03-20,1,2,0,27.3333339691162,32.3333320617676,0.333333343267441,60,3,2,212,
2962,484,2012-03-20,2,2,0,27.6666660308838,32.3333320617676,0,60,3,2,212,72,0,1,0,1,1,12,164
2963,484,2012-03-20,3,2,0,24,35,1,60,3,2,212,72,0,1,0,1,1,12,1644.295588,2001.848272,4.726247
2964,484,2012-03-20,4,2,0,30.6666660308838,27.3333339691162,2,60,3,2,212,72,0,1,0,1,1,12,164
2965,484,2012-03-21,0,3,0,11,30.7999992370605,2.40000009536743,44.2000007629395,3,2,212,7
2966,484,2012-03-21,1,3,0,27.375,31.875,0.75,60,3,2,212,72,0,1,0,1,1,12,1644.295588,2001.84827
2967,484,2012-03-21,2,3,0,22.1666660308838,35.5,2.33333325386047,60,3,2,212,72,0,1,0,1,1,12,
2968,484,2012-03-21,3,3,0,33.6666679382324,26,0.333333343267441,60,3,2,212,72,0,1,0,1,1,12,1
2969,484,2012-03-21,4,3,0,19,37.8333320617676,3.16666674613953,60,3,2,212,72,0,1,0,1,1,12,16
2970,484,2012-03-22,0,4,0,23.3333339691162,32.6666679382324,1.33333337306976,57.333332
2971,484,2012-03-22,1,4,0,22,37,1,60,3,2,212,72,0,1,0,1,1,12,1644.295588,2001.848272,4.726247
2972,484,2012-03-22,2,4,0,24.1666660308838,34.6666679382324,1.16666662693024,60,3,2,212,7
2973,484,2012-03-22,3,4,0,23.3333339691162,30.6666660308838,6,60,3,2,212,72,0,1,0,1,1,12,164
2974,484,2012-03-22,4,4,0,32.1666679382324,26.8333339691162,1,60,3,2,212,72,0,1,0,1,1,12,164
2975,484,2012-03-23,1,5,0,11.6666669845581,43.6666679382324,0.333333343267441,55.666667
2976,484,2012-03-23,2,5,0,26,33.8333320617676,0.16666667163372,60,3,2,212,72,0,1,0,1,1,12,16
2977,484,2012-03-23,3,5,0,17.6000003814697,39.2000007629395,3.20000004768372,60,3,2,212,7
2978,484,2012-03-23,4,5,0,19.6666660308838,40.3333320617676,0,60,3,2,212,72,0,1,0,1,1,12,164
2979,484,2012-03-24,3,6,1,31,28,1,60,1,2,212,72,0,1,0,1,1,12,1644.295588,2001.848272,4.726247
2980,484,2012-03-24,4,6,1,20.3333339691162,39.3333320617676,0.333333343267441,60,3,2,212,
2981,485,2012-03-17,0,6,1,9.5,31,2,42.5,2,2,212,74,0,0,0,1,1,12,1633.984958,1934.234114,4.16084
2982,485,2012-03-17,1,6,1,27.5,32,0.5,60,3,2,212,74,0,0,0,1,1,12,1633.984958,1934.234114,4.160
2983,485,2012-03-17,2,6,1,15.6666669845581,42.6666679382324,1.66666662693024,60,3,2,212,7
2984,485,2012-03-17,3,6,1,35,25,0,60,3,2,212,74,0,0,0,1,1,12,1633.984958,1934.234114,4.160849
2985,485,2012-03-17,4,6,1,15,42.6666679382324,2.33333325386047,60,3,2,212,74,0,0,0,1,1,12,16
2986,485,2012-03-18,0,0,1,29,27.5,2,58.5,2,2,212,74,0,0,0,1,1,12,1633.984958,1934.234114,4.160
2987,485,2012-03-18,1,0,1,26.3333339691162,31.6666660308838,2,60,3,2,212,74,0,0,0,1,1,12,163
2988,485,2012-03-18,2,0,1,28.8333339691162,30.3333339691162,0.833333313465118,60,3,2,212,
2989,485,2012-03-18,3,0,1,25.25,34.5,0.25,60,3,2,212,74,0,0,0,1,1,12,1633.984958,1934.234114,4.
2990,485,2012-03-18,4,0,1,26.3333339691162,33.6666679382324,0,60,3,2,212,74,0,0,0,1,1,12,163
2991,485,2012-03-19,1,1,0,11.6666669845581,43.6666679382324,0.333333343267441,55.666667
2992,485,2012-03-19,2,1,0,11.6666669845581,48.3333320617676,0,60,3,2,212,74,0,0,0,1,1,12,163
2993,485,2012-03-19,3,1,0,16.2000007629395,40.5999984741211,3.20000004768372,60,3,2,212,7
2994,485,2012-03-19,4,1,0,20,39.8333320617676,0.16666667163372,60,3,2,212,74,0,0,0,1,1,12,16
2995,485,2012-03-24,0,6,1,19,40,1,60,1,2,212,74,0,0,0,1,1,12,1633.984958,1934.234114,4.160849
2996,485,2012-03-24,1,6,1,28.6666660308838,31,0.333333343267441,60,3,2,212,74,0,0,0,1,1,12,1
2997,485,2012-03-24,2,6,1,28.6666660308838,28.3333339691162,3,60,3,2,212,74,0,0,0,1,1,12,163
2998,485,2012-03-24,3,6,1,32.3333320617676,27,0.666666686534882,60,3,2,212,74,0,0,0,1,1,12,1
2999,485,2012-03-24,4,6,1,15.5,38.5,6,60,2,2,212,74,0,0,0,1,1,12,1633.984958,1934.234114,4.160

3000,485,2012-03-25,0,0,1,6.5,28.5,0.5,35.5,2,2,212,74,0,0,0,1,1,12,1633.984958,1934.234114,4.16
3001,485,2012-03-25,1,0,1,22,38,0,60,3,2,212,74,0,0,0,1,1,12,1633.984958,1934.234114,4.160849
3002,485,2012-03-25,2,0,1,31,28,1,60,3,2,212,74,0,0,0,1,1,12,1633.984958,1934.234114,4.160849
3003,485,2012-03-25,3,0,1,25.6666660308838,22.3333339691162,12,60,3,2,212,74,0,0,0,1,1,12,16
3004,485,2012-03-25,4,0,1,38,20.5,1.5,60,3,2,212,74,0,0,0,1,1,12,1633.984958,1934.234114,4.160
3005,485,2012-03-26,0,1,0,26,22,3,51,2,2,212,74,0,0,0,1,1,12,1633.984958,1934.234114,4.160849
3006,485,2012-03-26,1,1,0,22.7999992370605,36.2000007629395,1,60,3,2,212,74,0,0,0,1,1,12,163
3007,485,2012-03-26,2,1,0,27.6666660308838,32.3333320617676,0,60,3,2,212,74,0,0,0,1,1,12,163
3008,485,2012-03-26,3,1,0,24,35,1,60,3,2,212,74,0,0,0,1,1,12,1633.984958,1934.234114,4.160849
3009,485,2012-03-26,4,1,0,30.6666660308838,27.3333339691162,2,60,3,2,212,74,0,0,0,1,1,12,163
3010,488,2012-03-16,1,5,0,16.3333339691162,34,6,56.3333320617676,3,2,212,69,1,0,0,1,1,11,165
3011,488,2012-03-16,2,5,0,40.6666679382324,19,0.333333343267441,60,3,2,212,69,1,0,0,1,1,11,1
3012,488,2012-03-16,3,5,0,19.3333339691162,40.3333320617676,0.333333343267441,60,3,2,212,
3013,488,2012-03-16,4,5,0,32.7999992370605,25.7999992370605,0.200000002980232,58.799999
3014,488,2012-03-20,0,2,0,32,28,0,60,1,2,212,69,1,0,0,1,1,11,1650.995833,2054.845439,4.321171
3015,488,2012-03-20,1,2,0,10.3333330154419,43,1.83333337306976,55.1666679382324,3,2,212,6
3016,488,2012-03-20,2,2,0,20.5,38.75,0.75,60,3,2,212,69,1,0,0,1,1,11,1650.995833,2054.845439,4.
3017,488,2012-03-20,3,2,0,23.3333339691162,36.3333320617676,0.333333343267441,60,3,2,212,
3018,488,2012-03-20,4,2,0,39.6666679382324,20,0.333333343267441,60,3,2,212,69,1,0,0,1,1,11,1
3019,488,2012-03-21,1,3,0,8.33333301544189,50.3333320617676,1.33333337306976,60,3,2,212,6
3020,488,2012-03-21,2,3,0,34.7999992370605,25,0.200000002980232,60,3,2,212,69,1,0,0,1,1,11,1
3021,488,2012-03-21,3,3,0,18,39.3333320617676,2.66666674613953,60,3,2,212,69,1,0,0,1,1,11,16
3022,488,2012-03-21,4,3,0,32.5,27.5,0,60,3,2,212,69,1,0,0,1,1,11,1650.995833,2054.845439,4.321
3023,488,2012-03-22,1,4,0,17.8333339691162,34.6666679382324,3.16666674613953,55.666667
3024,488,2012-03-22,2,4,0,33.25,26.75,0,60,3,2,212,69,1,0,0,1,1,11,1650.995833,2054.845439,4.3
3025,488,2012-03-22,3,4,0,21.3333339691162,37.3333320617676,1.33333337306976,60,3,2,212,6
3026,488,2012-03-22,4,4,0,27.2000007629395,27.6000003814697,0.800000011920929,55.599998
3027,488,2012-03-23,1,5,0,15.6666669845581,40.3333320617676,1,57,3,2,212,69,1,0,0,1,1,11,165
3028,488,2012-03-23,2,5,0,27.3999996185303,28.3999996185303,4.19999980926514,60,3,2,212,6
3029,488,2012-03-23,3,5,0,18.1666660308838,39.8333320617676,2,60,3,2,212,69,1,0,0,1,1,11,165
3030,488,2012-03-23,4,5,0,28,32,0,60,3,2,212,69,1,0,0,1,1,11,1650.995833,2054.845439,4.321171
3031,490,2012-03-16,1,5,0,11.6666669845581,43.6666679382324,0.333333343267441,55.666667
3032,490,2012-03-16,2,5,0,20.6000003814697,39.2000007629395,0.200000002980232,60,3,2,212,
3033,490,2012-03-16,3,5,0,25.6666660308838,29.3333339691162,5,60,3,2,212,68,1,1,0,1,1,12,164
3034,490,2012-03-16,4,5,0,19.6666660308838,40.3333320617676,0,60,3,2,212,68,1,1,0,1,1,12,164
3035,490,2012-03-17,0,6,1,10,27,1,38,2,2,212,68,1,1,0,1,1,12,1644.295588,2001.848272,4.726247
3036,490,2012-03-17,1,6,1,28.75,31,0.25,60,3,2,212,68,1,1,0,1,1,12,1644.295588,2001.848272,4.72
3037,490,2012-03-17,2,6,1,28.6666660308838,28.3333339691162,3,60,3,2,212,68,1,1,0,1,1,12,164
3038,490,2012-03-17,3,6,1,23.1666660308838,36.1666679382324,0.666666686534882,60,3,2,212,
3039,490,2012-03-17,4,6,1,21.6666660308838,36.1666679382324,2.16666674613953,60,3,2,212,6
3040,490,2012-03-18,0,0,1,12,43,0,55,1,2,212,68,1,1,0,1,1,12,1644.295588,2001.848272,4.726247
3041,490,2012-03-18,1,0,1,20,39.6666679382324,0.333333343267441,60,3,2,212,68,1,1,0,1,1,12,1
3042,490,2012-03-18,2,0,1,31,28,1,60,3,2,212,68,1,1,0,1,1,12,1644.295588,2001.848272,4.726247
3043,490,2012-03-18,3,0,1,25.6666660308838,22.3333339691162,12,60,3,2,212,68,1,1,0,1,1,12,16
3044,490,2012-03-18,4,0,1,38,20,2,60,3,2,212,68,1,1,0,1,1,12,1644.295588,2001.848272,4.726247
3045,490,2012-03-19,0,1,0,26,22,3,51,2,2,212,68,1,1,0,1,1,12,1644.295588,2001.848272,4.726247
3046,490,2012-03-19,1,1,0,21,37.6666679382324,1.33333337306976,60,3,2,212,68,1,1,0,1,1,12,16
3047,490,2012-03-19,2,1,0,27.6666660308838,32.3333320617676,0,60,3,2,212,68,1,1,0,1,1,12,164
3048,490,2012-03-19,3,1,0,24,35,1,60,3,2,212,68,1,1,0,1,1,12,1644.295588,2001.848272,4.726247
3049,490,2012-03-19,4,1,0,30.6666660308838,27.3333339691162,2,60,3,2,212,68,1,1,0,1,1,12,164

3050,490,2012-03-21,0,3,0,9.5,31,2,42.5,2,2,212,68,1,1,0,1,1,12,1644.295588,2001.848272,4.72624
3051,490,2012-03-21,1,3,0,33.3333320617676,26.3333339691162,0.333333343267441,60,3,2,212,
3052,490,2012-03-21,2,3,0,15.6666669845581,42.6666679382324,1.66666662693024,60,3,2,212,6
3053,490,2012-03-21,3,3,0,35,25,0,60,3,2,212,68,1,1,0,1,1,12,1644.295588,2001.848272,4.7262474
3054,490,2012-03-21,4,3,0,15,42.6666679382324,2.33333325386047,60,3,2,212,68,1,1,0,1,1,12,16
3055,490,2012-03-22,0,4,0,29,27.5,2,58.5,2,2,212,68,1,1,0,1,1,12,1644.295588,2001.848272,4.7262
3056,490,2012-03-22,1,4,0,23.3999996185303,35.2000007629395,1.39999997615814,60,3,2,212,6
3057,490,2012-03-22,2,4,0,26.25,32.75,1,60,3,2,212,68,1,1,0,1,1,12,1644.295588,2001.848272,4.72
3058,490,2012-03-22,3,4,0,21,39,0,60,3,2,212,68,1,1,0,1,1,12,1644.295588,2001.848272,4.7262474
3059,490,2012-03-22,4,4,0,26.3333339691162,33.6666679382324,0,60,3,2,212,68,1,1,0,1,1,12,164
3060,491,2012-03-17,0,6,1,19,40,1,60,1,2,212,71,0,0,0,1,1,12,1650.995833,2054.845439,4.3211717
3061,491,2012-03-17,1,6,1,28.6666660308838,31,0.333333343267441,60,3,2,212,71,0,0,0,1,1,12,1
3062,491,2012-03-17,2,6,1,28.6666660308838,28.3333339691162,3,60,3,2,212,71,0,0,0,1,1,12,165
3063,491,2012-03-17,3,6,1,37,22,1,60,2,2,212,71,0,0,0,1,1,12,1650.995833,2054.845439,4.3211717
3064,491,2012-03-18,0,0,1,1,14,1,16,1,2,212,71,0,0,0,1,1,12,1650.995833,2054.845439,4.32117177
3065,491,2012-03-18,1,0,1,22,38,0,60,3,2,212,71,0,0,0,1,1,12,1650.995833,2054.845439,4.3211717
3066,491,2012-03-18,2,0,1,31,28,1,60,3,2,212,71,0,0,0,1,1,12,1650.995833,2054.845439,4.3211717
3067,491,2012-03-18,3,0,1,21.3333339691162,28.6666660308838,10,60,3,2,212,71,0,0,0,1,1,12,16
3068,491,2012-03-18,4,0,1,23,33,4,60,3,2,212,71,0,0,0,1,1,12,1650.995833,2054.845439,4.3211717
3069,491,2012-03-19,0,1,0,12,43,0,55,1,2,212,71,0,0,0,1,1,12,1650.995833,2054.845439,4.3211717
3070,491,2012-03-19,1,1,0,27.3333339691162,32.3333320617676,0.333333343267441,60,3,2,212,
3071,491,2012-03-19,2,1,0,27.6666660308838,32.3333320617676,0,60,3,2,212,71,0,0,0,1,1,12,165
3072,491,2012-03-19,3,1,0,27,30.75,2.25,60,3,2,212,71,0,0,0,1,1,12,1650.995833,2054.845439,4.32
3073,491,2012-03-19,4,1,0,38,20,2,60,3,2,212,71,0,0,0,1,1,12,1650.995833,2054.845439,4.3211717
3074,491,2012-03-21,0,3,0,27.5,24.75,2.5,54.75,3,2,212,71,0,0,0,1,1,12,1650.995833,2054.845439,
3075,491,2012-03-21,1,3,0,21.375,35.5,1.5,58.375,3,2,212,71,0,0,0,1,1,12,1650.995833,2054.8454
3076,491,2012-03-21,2,3,0,28,31,1,60,3,2,212,71,0,0,0,1,1,12,1650.995833,2054.845439,4.3211717
3077,491,2012-03-21,3,3,0,30.3333339691162,27.1666660308838,2.5,60,3,2,212,71,0,0,0,1,1,12,16
3078,491,2012-03-21,4,3,0,25.1666660308838,33.8333320617676,1,60,3,2,212,71,0,0,0,1,1,12,165
3079,491,2012-03-22,0,4,0,9.5,31,2,42.5,2,2,212,71,0,0,0,1,1,12,1650.995833,2054.845439,4.32117
3080,491,2012-03-22,1,4,0,21.25,38.5,0.25,60,3,2,212,71,0,0,0,1,1,12,1650.995833,2054.845439,4.
3081,491,2012-03-22,2,4,0,14.5,44.8333320617676,0.666666686534882,60,3,2,212,71,0,0,0,1,1,12
3082,491,2012-03-22,3,4,0,17.5,42.1666679382324,0.333333343267441,60,3,2,212,71,0,0,0,1,1,12
3083,491,2012-03-22,4,4,0,20.5555553436279,38.5555572509766,0.888888895511627,60,3,2,212,
3084,493,2012-03-22,0,4,0,44,15,1,60,1,2,212,69,0,0,0,1,1,12,1652.358901,1940.685086,4.3777094
3085,493,2012-03-22,1,4,0,14.6666669845581,41,4.33333349227905,60,3,2,212,69,0,0,0,1,1,12,16
3086,493,2012-03-22,2,4,0,15,40.5,4.5,60,2,2,212,69,0,0,0,1,1,12,1652.358901,1940.685086,4.3777
3087,493,2012-03-23,0,5,0,5,10,0,15,1,2,212,69,0,0,0,1,1,12,1652.358901,1940.685086,4.37770946
3088,493,2012-03-23,1,5,0,15,44,1,60,3,2,212,69,0,0,0,1,1,12,1652.358901,1940.685086,4.3777094
3089,493,2012-03-23,2,5,0,8.5,46.5,5,60,2,2,212,69,0,0,0,1,1,12,1652.358901,1940.685086,4.37770
3090,493,2012-03-23,3,5,0,12,45,3,60,3,2,212,69,0,0,0,1,1,12,1652.358901,1940.685086,4.3777094
3091,493,2012-03-23,4,5,0,29.6666660308838,30,0.333333343267441,60,3,2,212,69,0,0,0,1,1,12,1
3092,493,2012-03-24,0,6,1,19,32,1,52,1,2,212,69,0,0,0,1,1,12,1652.358901,1940.685086,4.3777094
3093,493,2012-03-24,1,6,1,6,19,35,60,3,2,212,69,0,0,0,1,1,12,1652.358901,1940.685086,4.3777094
3094,493,2012-03-24,2,6,1,17.2000007629395,37.5999984741211,5.19999980926514,60,3,2,212,6
3095,493,2012-03-24,3,6,1,12.3333330154419,42.6666679382324,5,60,3,2,212,69,0,0,0,1,1,12,165
3096,493,2012-03-24,4,6,1,32.6666679382324,27,0.333333343267441,60,3,2,212,69,0,0,0,1,1,12,1
3097,493,2012-03-25,0,0,1,9,26,23,58,2,2,212,69,0,0,0,1,1,12,1652.358901,1940.685086,4.3777094
3098,493,2012-03-25,1,0,1,5.66666650772095,33.6666679382324,20.6666660308838,60,3,2,212,6
3099,493,2012-03-25,2,0,1,31.3333339691162,23.3333339691162,5.33333349227905,60,3,2,212,6

3100,493,2012-03-25,3,0,1,16.75,35.25,8,60,3,2,212,69,0,0,0,1,1,12,1652.358901,1940.685086,4.37
3101,493,2012-03-25,4,0,1,30.6666660308838,27.3333339691162,2,60,3,2,212,69,0,0,0,1,1,12,165
3102,493,2012-03-26,0,1,0,1,16,1,18,1,2,212,69,0,0,0,1,1,12,1652.358901,1940.685086,4.3777094
3103,493,2012-03-26,1,1,0,9.66666698455811,40.3333320617676,10,60,3,2,212,69,0,0,0,1,1,12,16
3104,493,2012-03-26,2,1,0,0.666666686534882,43.3333320617676,16,60,3,2,212,69,0,0,0,1,1,12,1
3105,493,2012-03-26,3,1,0,18.75,38.5,2.75,60,3,2,212,69,0,0,0,1,1,12,1652.358901,1940.685086,4.
3106,493,2012-03-26,4,1,0,21,38.6666679382324,0.333333343267441,60,3,2,212,69,0,0,0,1,1,12,1
3107,493,2012-03-27,0,2,0,2,30,1.5,33.5,2,2,212,69,0,0,0,1,1,12,1652.358901,1940.685086,4.3777
3108,493,2012-03-27,1,2,0,8,45,7,60,3,2,212,69,0,0,0,1,1,12,1652.358901,1940.685086,4.3777094
3109,493,2012-03-27,2,2,0,42,16,2,60,3,2,212,69,0,0,0,1,1,12,1652.358901,1940.685086,4.377709
3110,493,2012-03-27,3,2,0,12,38.75,9.25,60,3,2,212,69,0,0,0,1,1,12,1652.358901,1940.685086,4.37
3111,493,2012-03-27,4,2,0,31.7999992370605,28,0.200000002980232,60,3,2,212,69,0,0,0,1,1,12,1
3112,493,2012-04-03,0,2,0,8.5,22.5,2,33,2,2,212,69,0,0,0,1,1,12,1652.358901,1940.685086,4.3777
3113,493,2012-04-03,1,2,0,2.66666674613953,51,6.33333349227905,60,3,2,212,69,0,0,0,1,1,12,16
3114,493,2012-04-03,2,2,0,12.3333330154419,44.3333320617676,3.33333325386047,60,3,2,212,6
3115,493,2012-04-03,3,2,0,11.3333330154419,47.3333320617676,1.33333337306976,60,3,2,212,6
3116,493,2012-04-03,4,2,0,32.5,26.25,1.25,60,3,2,212,69,0,0,0,1,1,12,1652.358901,1940.685086,4.
3117,496,2012-04-04,0,3,0,13,43.6666679382324,2.66666674613953,59.3333320617676,3,2,212,7
3118,496,2012-04-04,1,3,0,31,27.5,1.5,60,3,2,212,76,0,0,0,1,1,12,1633.984958,1934.234114,4.160
3119,496,2012-04-04,2,3,0,45.3333320617676,14.3333330154419,0.333333343267441,60,3,2,212,
3120,496,2012-04-04,3,3,0,27.6666660308838,32.3333320617676,0,60,3,2,212,76,0,0,0,1,1,12,163
3121,496,2012-04-04,4,3,0,33.3333320617676,26,0.666666686534882,60,3,2,212,76,0,0,0,1,1,12,1
3122,496,2012-04-05,0,4,0,12,41,0.5,53.5,2,2,212,76,0,0,0,1,1,12,1633.984958,1934.234114,4.160
3123,496,2012-04-05,1,4,0,22.3999996185303,36.7999992370605,0.800000011920929,60,3,2,212,
3124,496,2012-04-05,2,4,0,40.6666679382324,15.3333330154419,4,60,3,2,212,76,0,0,0,1,1,12,163
3125,496,2012-04-05,3,4,0,36.3333320617676,23,0.666666686534882,60,3,2,212,76,0,0,0,1,1,12,1
3126,496,2012-04-05,4,4,0,32.3333320617676,27.6666660308838,0,60,3,2,212,76,0,0,0,1,1,12,163
3127,496,2012-04-06,0,5,0,24.5,25,1,50.5,2,2,212,76,0,0,0,1,1,12,1633.984958,1934.234114,4.160
3128,496,2012-04-06,1,5,0,21,37.6666679382324,1.33333337306976,60,3,2,212,76,0,0,0,1,1,12,16
3129,496,2012-04-06,2,5,0,39,20.8333339691162,0.16666667163372,60,3,2,212,76,0,0,0,1,1,12,16
3130,496,2012-04-06,3,5,0,36.5,23.5,0,60,3,2,212,76,0,0,0,1,1,12,1633.984958,1934.234114,4.160
3131,496,2012-04-06,4,5,0,33,26.6666660308838,0.333333343267441,60,3,2,212,76,0,0,0,1,1,12,1
3132,496,2012-04-07,0,6,1,35,23,0.5,58.5,2,2,212,76,0,0,0,1,1,12,1633.984958,1934.234114,4.160
3133,496,2012-04-07,1,6,1,34.6666679382324,22.6666660308838,2.66666674613953,60,3,2,212,7
3134,496,2012-04-07,2,6,1,36,22.3333339691162,1.66666662693024,60,3,2,212,76,0,0,0,1,1,12,16
3135,496,2012-04-07,3,6,1,42.2000007629395,17.7999992370605,0,60,3,2,212,76,0,0,0,1,1,12,163
3136,496,2012-04-07,4,6,1,39.7999992370605,20.2000007629395,0,60,3,2,212,76,0,0,0,1,1,12,163
3137,496,2012-04-08,0,0,1,19.6666660308838,20.6666660308838,0,40.3333320617676,3,2,212,76
3138,496,2012-04-08,1,0,1,36.6666679382324,21,2.33333325386047,60,3,2,212,76,0,0,0,1,1,12,16
3139,496,2012-04-08,2,0,1,35.3333320617676,24.3333339691162,0.333333343267441,60,3,2,212,
3140,496,2012-04-08,3,0,1,35,24.6666660308838,0.333333343267441,60,3,2,212,76,0,0,0,1,1,12,1
3141,496,2012-04-08,4,0,1,36.5,23.25,0.25,60,3,2,212,76,0,0,0,1,1,12,1633.984958,1934.234114,4.
3142,496,2012-04-09,0,1,0,24.25,27,0.75,52,3,2,212,76,0,0,0,1,1,12,1633.984958,1934.234114,4.16
3143,496,2012-04-09,1,1,0,30.6666660308838,29,0.333333343267441,60,3,2,212,76,0,0,0,1,1,12,1
3144,496,2012-04-09,2,1,0,53.6666679382324,6.33333349227905,0,60,3,2,212,76,0,0,0,1,1,12,163
3145,496,2012-04-09,3,1,0,42.6666679382324,17.3333339691162,0,60,3,2,212,76,0,0,0,1,1,12,163
3146,496,2012-04-09,4,1,0,34.3333320617676,25.6666660308838,0,60,3,2,212,76,0,0,0,1,1,12,163
3147,496,2012-04-10,1,2,0,36.3333320617676,22.3333339691162,1.33333337306976,60,3,2,212,7
3148,496,2012-04-10,2,2,0,45.3333320617676,14.6666669845581,0,60,3,2,212,76,0,0,0,1,1,12,163
3149,496,2012-04-10,3,2,0,34.6666679382324,24.6666660308838,0.666666686534882,60,3,2,212,

3150,496,2012-04-10,4,2,0,34.6666679382324,22.6666660308838,2.66666674613953,60,3,2,212,7
3151,503,2012-04-10,0,2,0,16,44,0,60,1,2,212,71,0,1,0,1,1,12,2363.404579,1219.513101,9.649747
3152,503,2012-04-10,1,2,0,34.6666679382324,25,0.333333343267441,60,3,2,212,71,0,1,0,1,1,12,2
3153,503,2012-04-11,0,3,0,11.5,43.5,4,59,2,2,212,71,0,1,0,1,1,12,2363.404579,1219.513101,9.6497
3154,503,2012-04-11,1,3,0,25,32.3333320617676,2.66666674613953,60,3,2,212,71,0,1,0,1,1,12,23
3155,503,2012-04-11,2,3,0,45.5999984741211,13,1.39999997615814,60,3,2,212,71,0,1,0,1,1,12,23
3156,503,2012-04-11,3,3,0,27.6666660308838,32.3333320617676,0,60,3,2,212,71,0,1,0,1,1,12,236
3157,503,2012-04-11,4,3,0,33.3333320617676,26,0.666666686534882,60,3,2,212,71,0,1,0,1,1,12,2
3158,503,2012-04-12,0,4,0,12,41,0.5,53.5,2,2,212,71,0,1,0,1,1,12,2363.404579,1219.513101,9.6497
3159,503,2012-04-12,1,4,0,19,40.6666679382324,0.333333343267441,60,3,2,212,71,0,1,0,1,1,12,2
3160,503,2012-04-12,2,4,0,34,24.5,1.5,60,3,2,212,71,0,1,0,1,1,12,2363.404579,1219.513101,9.6497
3161,503,2012-04-12,3,4,0,38.75,20.75,0.5,60,3,2,212,71,0,1,0,1,1,12,2363.404579,1219.513101,9.
3162,503,2012-04-12,4,4,0,32.3333320617676,27.6666660308838,0,60,3,2,212,71,0,1,0,1,1,12,236
3163,503,2012-04-13,0,5,0,24.5,25,1,50.5,2,2,212,71,0,1,0,1,1,12,2363.404579,1219.513101,9.6497
3164,503,2012-04-13,1,5,0,21,37.6666679382324,1.33333337306976,60,3,2,212,71,0,1,0,1,1,12,23
3165,503,2012-04-13,2,5,0,42.6666679382324,17,0.333333343267441,60,3,2,212,71,0,1,0,1,1,12,2
3166,503,2012-04-13,3,5,0,38.4000015258789,21.6000003814697,0,60,3,2,212,71,0,1,0,1,1,12,236
3167,503,2012-04-13,4,5,0,34.5,25.25,0.25,60,3,2,212,71,0,1,0,1,1,12,2363.404579,1219.513101,9.
3168,503,2012-04-14,0,6,1,35,23,0.5,58.5,2,2,212,71,0,1,0,1,1,12,2363.404579,1219.513101,9.6497
3169,503,2012-04-14,1,6,1,34.6666679382324,22.6666660308838,2.66666674613953,60,3,2,212,7
3170,503,2012-04-14,2,6,1,36,22.3333339691162,1.66666662693024,60,3,2,212,71,0,1,0,1,1,12,23
3171,503,2012-04-14,3,6,1,39.6666679382324,20.3333339691162,0,60,3,2,212,71,0,1,0,1,1,12,236
3172,503,2012-04-14,4,6,1,39.4000015258789,20.6000003814697,0,60,3,2,212,71,0,1,0,1,1,12,236
3173,503,2012-04-15,0,0,1,22.5,22.75,0,45.25,3,2,212,71,0,1,0,1,1,12,2363.404579,1219.513101,9.
3174,503,2012-04-15,1,0,1,36.6666679382324,21,2.33333325386047,60,3,2,212,71,0,1,0,1,1,12,23
3175,503,2012-04-15,2,0,1,35.3333320617676,24.3333339691162,0.333333343267441,60,3,2,212,
3176,503,2012-04-15,3,0,1,35,24.6666660308838,0.333333343267441,60,3,2,212,71,0,1,0,1,1,12,2
3177,503,2012-04-15,4,0,1,36.3333320617676,23.3333339691162,0.333333343267441,60,3,2,212,
3178,503,2012-04-16,0,1,0,22,26.3333339691162,1,49.3333320617676,3,2,212,71,0,1,0,1,1,12,236
3179,503,2012-04-16,1,1,0,31.5,28.25,0.25,60,3,2,212,71,0,1,0,1,1,12,2363.404579,1219.513101,9.
3180,503,2012-04-16,2,1,0,53.6666679382324,6.33333349227905,0,60,3,2,212,71,0,1,0,1,1,12,236
3181,503,2012-04-16,3,1,0,42.6666679382324,17.3333339691162,0,60,3,2,212,71,0,1,0,1,1,12,236
3182,503,2012-04-16,4,1,0,34.3333320617676,25.6666660308838,0,60,3,2,212,71,0,1,0,1,1,12,236
3183,503,2012-04-17,1,2,0,37.5,20.5,2,60,2,2,212,71,0,1,0,1,1,12,2363.404579,1219.513101,9.6497
3184,503,2012-04-17,2,2,0,45.3333320617676,14.6666669845581,0,60,3,2,212,71,0,1,0,1,1,12,236
3185,503,2012-04-17,3,2,0,34.6666679382324,24.6666660308838,0.666666686534882,60,3,2,212,
3186,503,2012-04-17,4,2,0,34.6666679382324,22.6666660308838,2.66666674613953,60,3,2,212,7
3187,509,2011-04-01,0,5,0,24.5,25,1,50.5,2,2,212,78,1,0,1,1,1,10,1763.879042,1585.449327,5.842
3188,509,2011-04-01,1,5,0,21,37.6666679382324,1.33333337306976,60,3,2,212,78,1,0,1,1,1,10,17
3189,509,2011-04-01,2,5,0,42.6666679382324,17,0.333333343267441,60,3,2,212,78,1,0,1,1,1,10,1
3190,509,2011-04-01,3,5,0,37,23,0,60,3,2,212,78,1,0,1,1,1,10,1763.879042,1585.449327,5.842911
3191,509,2011-04-01,4,5,0,33,26.6666660308838,0.333333343267441,60,3,2,212,78,1,0,1,1,1,10,1
3192,509,2011-04-02,0,6,1,35,23,0.5,58.5,2,2,212,78,1,0,1,1,1,10,1763.879042,1585.449327,5.842
3193,509,2011-04-02,1,6,1,34.6666679382324,22.6666660308838,2.66666674613953,60,3,2,212,7
3194,509,2011-04-02,2,6,1,36,22.3333339691162,1.66666662693024,60,3,2,212,78,1,0,1,1,1,10,17
3195,509,2011-04-02,3,6,1,39.6666679382324,20.3333339691162,0,60,3,2,212,78,1,0,1,1,1,10,176
3196,509,2011-04-02,4,6,1,39.3333320617676,20.6666660308838,0,60,3,2,212,78,1,0,1,1,1,10,176
3197,509,2011-04-03,0,0,1,19.6666660308838,20.6666660308838,0,40.3333320617676,3,2,212,78
3198,509,2011-04-03,1,0,1,36.6666679382324,21,2.33333325386047,60,3,2,212,78,1,0,1,1,1,10,17
3199,509,2011-04-03,2,0,1,35.3333320617676,24.3333339691162,0.333333343267441,60,3,2,212,

3200,509,2011-04-03,3,0,1,35,24.6666660308838,0.333333343267441,60,3,2,212,78,1,0,1,1,1,10,1
3201,509,2011-04-03,4,0,1,36.3333320617676,23.3333339691162,0.333333343267441,60,3,2,212,
3202,509,2011-04-04,0,1,0,25.3333339691162,23.6666660308838,0.333333343267441,49.333332
3203,509,2011-04-04,1,1,0,30.6666660308838,29,0.333333343267441,60,3,2,212,78,1,0,1,1,1,10,1
3204,509,2011-04-04,2,1,0,53.6666679382324,6.33333349227905,0,60,3,2,212,78,1,0,1,1,1,10,176
3205,509,2011-04-04,3,1,0,42.6666679382324,17.3333339691162,0,60,3,2,212,78,1,0,1,1,1,10,176
3206,509,2011-04-04,4,1,0,34.3333320617676,25.6666660308838,0,60,3,2,212,78,1,0,1,1,1,10,176
3207,509,2011-04-05,0,2,0,21,37,2,60,1,2,212,78,1,0,1,1,1,10,1763.879042,1585.449327,5.842911
3208,509,2011-04-05,1,2,0,36.3333320617676,22.3333339691162,1.33333337306976,60,3,2,212,7
3209,509,2011-04-05,2,2,0,45.3333320617676,14.6666669845581,0,60,3,2,212,78,1,0,1,1,1,10,176
3210,509,2011-04-05,3,2,0,34.6666679382324,24.6666660308838,0.666666686534882,60,3,2,212,
3211,509,2011-04-05,4,2,0,34.6666679382324,22.6666660308838,2.66666674613953,60,3,2,212,7
3212,509,2012-04-17,0,2,0,16,44,0,60,1,2,212,78,1,0,1,1,1,10,1763.879042,1585.449327,5.842911
3213,509,2012-04-17,1,2,0,34.6666679382324,25,0.333333343267441,60,3,2,212,78,1,0,1,1,1,10,1
3214,509,2012-04-17,2,2,0,39,20,1,60,1,2,212,78,1,0,1,1,1,10,1763.879042,1585.449327,5.842911
3215,509,2012-04-18,0,3,0,11.5,43.5,4,59,2,2,212,78,1,0,1,1,1,10,1763.879042,1585.449327,5.842
3216,509,2012-04-18,1,3,0,25,32.3333320617676,2.66666674613953,60,3,2,212,78,1,0,1,1,1,10,17
3217,509,2012-04-18,2,3,0,43.7999992370605,13.8000001907349,2.40000009536743,60,3,2,212,7
3218,509,2012-04-18,3,3,0,27.6666660308838,32.3333320617676,0,60,3,2,212,78,1,0,1,1,1,10,176
3219,509,2012-04-18,4,3,0,33.3333320617676,26,0.666666686534882,60,3,2,212,78,1,0,1,1,1,10,1
3220,509,2012-04-19,0,4,0,12,41,0.5,53.5,2,2,212,78,1,0,1,1,1,10,1763.879042,1585.449327,5.842
3221,509,2012-04-19,1,4,0,19,40.6666679382324,0.333333343267441,60,3,2,212,78,1,0,1,1,1,10,1
3222,509,2012-04-19,2,4,0,35.3333320617676,24.6666660308838,0,60,3,2,212,78,1,0,1,1,1,10,176
3223,509,2012-04-19,3,4,0,39.7999992370605,19.7999992370605,0.400000005960464,60,3,2,212,
3224,509,2012-04-19,4,4,0,32.3333320617676,27.6666660308838,0,60,3,2,212,78,1,0,1,1,1,10,176
3225,510,2011-03-24,0,4,0,35,23,0.5,58.5,2,2,212,72,1,1,0,1,1,10,1625.079858,1580.795093,4.240
3226,510,2011-03-24,1,4,0,34.6666679382324,22.6666660308838,2.66666674613953,60,3,2,212,7
3227,510,2011-03-24,2,4,0,36,22.3333339691162,1.66666662693024,60,3,2,212,72,1,1,0,1,1,10,16
3228,510,2011-03-24,3,4,0,46,14,0,60,2,2,212,72,1,1,0,1,1,10,1625.079858,1580.795093,4.240562
3229,510,2011-03-24,4,4,0,39.6666679382324,20.3333339691162,0,60,3,2,212,72,1,1,0,1,1,10,162
3230,510,2011-03-25,0,5,0,21,24,0,45,2,2,212,72,1,1,0,1,1,10,1625.079858,1580.795093,4.240562
3231,510,2011-03-25,1,5,0,36.6666679382324,21,2.33333325386047,60,3,2,212,72,1,1,0,1,1,10,16
3232,510,2011-03-25,2,5,0,35.3333320617676,24.3333339691162,0.333333343267441,60,3,2,212,
3233,510,2011-03-25,3,5,0,37.3333320617676,22.5,0.16666667163372,60,3,2,212,72,1,1,0,1,1,10,
3234,510,2011-03-25,4,5,0,39,21,0,60,3,2,212,72,1,1,0,1,1,10,1625.079858,1580.795093,4.240562
3235,510,2011-03-26,0,6,1,24,21.5,0,45.5,2,2,212,72,1,1,0,1,1,10,1625.079858,1580.795093,4.240
3236,510,2011-03-26,1,6,1,30.6666660308838,29,0.333333343267441,60,3,2,212,72,1,1,0,1,1,10,1
3237,510,2011-03-26,2,6,1,53.6666679382324,6.33333349227905,0,60,3,2,212,72,1,1,0,1,1,10,162
3238,510,2011-03-26,3,6,1,42.6666679382324,17.3333339691162,0,60,3,2,212,72,1,1,0,1,1,10,162
3239,510,2011-03-26,4,6,1,35.7999992370605,24,0.200000002980232,60,3,2,212,72,1,1,0,1,1,10,1
3240,510,2011-03-27,0,0,1,22,26.3333339691162,1,49.3333320617676,3,2,212,72,1,1,0,1,1,10,162
3241,510,2011-03-27,1,0,1,36.3333320617676,22.3333339691162,1.33333337306976,60,3,2,212,7
3242,510,2011-03-27,2,0,1,45.3333320617676,14.6666669845581,0,60,3,2,212,72,1,1,0,1,1,10,162
3243,510,2011-03-27,3,0,1,34.6666679382324,24.6666660308838,0.666666686534882,60,3,2,212,
3244,510,2011-03-27,4,0,1,34.25,23.75,2,60,3,2,212,72,1,1,0,1,1,10,1625.079858,1580.795093,4.2
3245,510,2011-03-31,0,4,0,24.5,25,1,50.5,2,2,212,72,1,1,0,1,1,10,1625.079858,1580.795093,4.240
3246,510,2011-03-31,1,4,0,21,37.6666679382324,1.33333337306976,60,3,2,212,72,1,1,0,1,1,10,16
3247,510,2011-03-31,2,4,0,42,17.75,0.25,60,3,2,212,72,1,1,0,1,1,10,1625.079858,1580.795093,4.2
3248,510,2011-03-31,3,4,0,36.5,23.5,0,60,3,2,212,72,1,1,0,1,1,10,1625.079858,1580.795093,4.240
3249,510,2011-03-31,4,4,0,33,26.6666660308838,0.333333343267441,60,3,2,212,72,1,1,0,1,1,10,1

3250,510,2011-04-05,0,2,0,16,44,0,60,1,2,212,72,1,1,0,1,1,10,1625.079858,1580.795093,4.2405625
3251,510,2011-04-05,1,2,0,24,35,1,60,1,2,212,72,1,1,0,1,1,10,1625.079858,1580.795093,4.2405625
3252,510,2011-04-06,0,3,0,11.5,43.5,4,59,2,2,212,72,1,1,0,1,1,10,1625.079858,1580.795093,4.2405
3253,510,2011-04-06,1,3,0,31,27.3999996185303,1.60000002384186,60,3,2,212,72,1,1,0,1,1,10,16
3254,510,2011-04-06,2,3,0,46.25,13.5,0.25,60,3,2,212,72,1,1,0,1,1,10,1625.079858,1580.795093,4.
3255,510,2011-04-06,3,3,0,27.6666660308838,32.3333320617676,0,60,3,2,212,72,1,1,0,1,1,10,162
3256,510,2011-04-06,4,3,0,33.3333320617676,26,0.666666686534882,60,3,2,212,72,1,1,0,1,1,10,1
3257,510,2011-04-07,0,4,0,12,41,0.5,53.5,2,2,212,72,1,1,0,1,1,10,1625.079858,1580.795093,4.2405
3258,510,2011-04-07,1,4,0,19,40.6666679382324,0.333333343267441,60,3,2,212,72,1,1,0,1,1,10,1
3259,510,2011-04-07,2,4,0,34.75,22.25,3,60,3,2,212,72,1,1,0,1,1,10,1625.079858,1580.795093,4.24
3260,510,2011-04-07,3,4,0,36.3333320617676,23,0.666666686534882,60,3,2,212,72,1,1,0,1,1,10,1
3261,510,2011-04-07,4,4,0,32.3333320617676,27.6666660308838,0,60,3,2,212,72,1,1,0,1,1,10,162
3262,511,2011-03-17,0,4,0,21,24,0,45,2,2,212,74,0,1,0,1,1,12,1625.079858,1580.795093,4.2405625
3263,511,2011-03-17,1,4,0,36.6666679382324,21,2.33333325386047,60,3,2,212,74,0,1,0,1,1,12,16
3264,511,2011-03-17,2,4,0,35.3333320617676,24.3333339691162,0.333333343267441,60,3,2,212,
3265,511,2011-03-17,3,4,0,35,24.6666660308838,0.333333343267441,60,3,2,212,74,0,1,0,1,1,12,1
3266,511,2011-03-17,4,4,0,38.7999992370605,21,0.200000002980232,60,3,2,212,74,0,1,0,1,1,12,1
3267,511,2011-03-18,0,5,0,24,21.5,0,45.5,2,2,212,74,0,1,0,1,1,12,1625.079858,1580.795093,4.2405
3268,511,2011-03-18,1,5,0,30.6666660308838,29,0.333333343267441,60,3,2,212,74,0,1,0,1,1,12,1
3269,511,2011-03-18,2,5,0,53.6666679382324,6.33333349227905,0,60,3,2,212,74,0,1,0,1,1,12,162
3270,511,2011-03-18,3,5,0,42.6666679382324,17.3333339691162,0,60,3,2,212,74,0,1,0,1,1,12,162
3271,511,2011-03-18,4,5,0,33.75,26.25,0,60,3,2,212,74,0,1,0,1,1,12,1625.079858,1580.795093,4.24
3272,511,2011-03-19,0,6,1,22,26.3333339691162,1,49.3333320617676,3,2,212,74,0,1,0,1,1,12,162
3273,511,2011-03-19,1,6,1,36.3333320617676,22.3333339691162,1.33333337306976,60,3,2,212,7
3274,511,2011-03-19,2,6,1,45.3333320617676,14.6666669845581,0,60,3,2,212,74,0,1,0,1,1,12,162
3275,511,2011-03-19,3,6,1,34.6666679382324,24.6666660308838,0.666666686534882,60,3,2,212,
3276,511,2011-03-19,4,6,1,34.6666679382324,22.6666660308838,2.66666674613953,60,3,2,212,7
3277,511,2011-03-27,0,0,1,16,44,0,60,1,2,212,74,0,1,0,1,1,12,1625.079858,1580.795093,4.2405625
3278,511,2011-03-28,0,1,0,11.5,43.5,4,59,2,2,212,74,0,1,0,1,1,12,1625.079858,1580.795093,4.2405
3279,511,2011-03-28,1,1,0,28.2000007629395,30,1.79999995231628,60,3,2,212,74,0,1,0,1,1,12,16
3280,511,2011-03-28,2,1,0,45.3333320617676,14.3333330154419,0.333333343267441,60,3,2,212,
3281,511,2011-03-28,3,1,0,27.6666660308838,32.3333320617676,0,60,3,2,212,74,0,1,0,1,1,12,162
3282,511,2011-03-28,4,1,0,33.3333320617676,26,0.666666686534882,60,3,2,212,74,0,1,0,1,1,12,1
3283,511,2011-03-29,0,2,0,12,41,0.5,53.5,2,2,212,74,0,1,0,1,1,12,1625.079858,1580.795093,4.2405
3284,511,2011-03-29,1,2,0,23.75,36,0.25,60,3,2,212,74,0,1,0,1,1,12,1625.079858,1580.795093,4.24
3285,511,2011-03-29,2,2,0,35,22,3,60,3,2,212,74,0,1,0,1,1,12,1625.079858,1580.795093,4.2405625
3286,511,2011-03-29,3,2,0,36.3333320617676,23,0.666666686534882,60,3,2,212,74,0,1,0,1,1,12,1
3287,511,2011-03-29,4,2,0,32.3333320617676,27.6666660308838,0,60,3,2,212,74,0,1,0,1,1,12,162
3288,511,2011-03-30,0,3,0,24.5,25,1,50.5,2,2,212,74,0,1,0,1,1,12,1625.079858,1580.795093,4.2405
3289,511,2011-03-30,1,3,0,21,37.6666679382324,1.33333337306976,60,3,2,212,74,0,1,0,1,1,12,16
3290,511,2011-03-30,2,3,0,43.2000007629395,16.6000003814697,0.200000002980232,60,3,2,212,
3291,511,2011-03-30,3,3,0,36.5,23.5,0,60,3,2,212,74,0,1,0,1,1,12,1625.079858,1580.795093,4.2405
3292,511,2011-03-30,4,3,0,33,26.6666660308838,0.333333343267441,60,3,2,212,74,0,1,0,1,1,12,1
3293,511,2011-03-31,0,4,0,35,23,0.5,58.5,2,2,212,74,0,1,0,1,1,12,1625.079858,1580.795093,4.2405
3294,511,2011-03-31,1,4,0,34.6666679382324,22.6666660308838,2.66666674613953,60,3,2,212,7
3295,511,2011-03-31,2,4,0,36,22.3333339691162,1.66666662693024,60,3,2,212,74,0,1,0,1,1,12,16
3296,511,2011-03-31,3,4,0,42.2000007629395,17.7999992370605,0,60,3,2,212,74,0,1,0,1,1,12,162
3297,511,2011-03-31,4,4,0,39.6666679382324,20.3333339691162,0,60,3,2,212,74,0,1,0,1,1,12,162
3298,515,2011-03-20,0,0,1,16,42.5,0.5,59,2,2,212,80,0,1,0,1,1,12,1559.30899,1705.458039,0.81823
3299,515,2011-03-20,1,0,1,34.6666679382324,25,0.333333343267441,60,3,2,212,80,0,1,0,1,1,12,1

3300,515,2011-03-20,2,0,1,45.3333320617676,14.3333330154419,0.333333343267441,60,3,2,212,
3301,515,2011-03-20,3,0,1,27.6666660308838,32.3333320617676,0,60,3,2,212,80,0,1,0,1,1,12,155
3302,515,2011-03-20,4,0,1,33.3333320617676,26,0.666666686534882,60,3,2,212,80,0,1,0,1,1,12,1
3303,515,2011-03-21,0,1,0,10.3333330154419,42.6666679382324,2.66666674613953,55.6666679
3304,515,2011-03-21,1,1,0,20.5,37.25,2.25,60,3,2,212,80,0,1,0,1,1,12,1559.30899,1705.458039,0.8
3305,515,2011-03-21,2,1,0,40.6666679382324,15.3333330154419,4,60,3,2,212,80,0,1,0,1,1,12,155
3306,515,2011-03-21,3,1,0,36.3333320617676,23,0.666666686534882,60,3,2,212,80,0,1,0,1,1,12,1
3307,515,2011-03-21,4,1,0,32.3333320617676,27.6666660308838,0,60,3,2,212,80,0,1,0,1,1,12,155
3308,515,2011-03-22,0,2,0,24.5,25,1,50.5,2,2,212,80,0,1,0,1,1,12,1559.30899,1705.458039,0.81823
3309,515,2011-03-22,1,2,0,23.75,35.25,1,60,3,2,212,80,0,1,0,1,1,12,1559.30899,1705.458039,0.818
3310,515,2011-03-22,2,2,0,35.3333320617676,24.6666660308838,0,60,3,2,212,80,0,1,0,1,1,12,155
3311,515,2011-03-22,3,2,0,43.3333320617676,16.6666660308838,0,60,3,2,212,80,0,1,0,1,1,12,155
3312,515,2011-03-22,4,2,0,33,26.6666660308838,0.333333343267441,60,3,2,212,80,0,1,0,1,1,12,1
3313,515,2011-04-01,0,5,0,35,23,0.5,58.5,2,2,212,80,0,1,0,1,1,12,1559.30899,1705.458039,0.81823
3314,515,2011-04-01,1,5,0,30.5,27.5,2,60,3,2,212,80,0,1,0,1,1,12,1559.30899,1705.458039,0.81823
3315,515,2011-04-01,2,5,0,40.7999992370605,18,1.20000004768372,60,3,2,212,80,0,1,0,1,1,12,15
3316,515,2011-04-01,3,5,0,36,24,0,60,3,2,212,80,0,1,0,1,1,12,1559.30899,1705.458039,0.81823067
3317,515,2011-04-01,4,5,0,39.6666679382324,20.3333339691162,0,60,3,2,212,80,0,1,0,1,1,12,155
3318,515,2011-04-02,0,6,1,21,24,0,45,2,2,212,80,0,1,0,1,1,12,1559.30899,1705.458039,0.81823067
3319,515,2011-04-02,1,6,1,36.6666679382324,21,2.33333325386047,60,3,2,212,80,0,1,0,1,1,12,15
3320,515,2011-04-02,2,6,1,34.5,25.25,0.25,60,3,2,212,80,0,1,0,1,1,12,1559.30899,1705.458039,0.8
3321,515,2011-04-02,3,6,1,33.25,26.75,0,60,3,2,212,80,0,1,0,1,1,12,1559.30899,1705.458039,0.818
3322,515,2011-04-02,4,6,1,39,21,0,60,3,2,212,80,0,1,0,1,1,12,1559.30899,1705.458039,0.81823067
3323,515,2011-04-03,0,0,1,24,21.5,0,45.5,2,2,212,80,0,1,0,1,1,12,1559.30899,1705.458039,0.81823
3324,515,2011-04-03,1,0,1,30.6666660308838,29,0.333333343267441,60,3,2,212,80,0,1,0,1,1,12,1
3325,515,2011-04-03,2,0,1,53.6666679382324,6.33333349227905,0,60,3,2,212,80,0,1,0,1,1,12,155
3326,515,2011-04-03,3,0,1,43.7999992370605,16,0.200000002980232,60,3,2,212,80,0,1,0,1,1,12,1
3327,515,2011-04-03,4,0,1,36.3333320617676,23.3333339691162,0.333333343267441,60,3,2,212,
3328,515,2011-04-04,0,1,0,22,26.3333339691162,1,49.3333320617676,3,2,212,80,0,1,0,1,1,12,155
3329,515,2011-04-04,1,1,0,36.3333320617676,22.3333339691162,1.33333337306976,60,3,2,212,8
3330,515,2011-04-04,2,1,0,45.3333320617676,14.6666669845581,0,60,3,2,212,80,0,1,0,1,1,12,155
3331,515,2011-04-04,3,1,0,34.6666679382324,24.6666660308838,0.666666686534882,60,3,2,212,
3332,515,2011-04-04,4,1,0,32,26,2,60,3,2,212,80,0,1,0,1,1,12,1559.30899,1705.458039,0.81823067
3333,515,2011-04-05,4,2,0,39.5,20.5,0,60,2,2,212,80,0,1,0,1,1,12,1559.30899,1705.458039,0.81823
3334,521,2011-03-17,0,4,0,21,24,0,45,2,2,212,74,0,1,0,1,1,12,1731.418007,2273.983634,9.263898(
3335,521,2011-03-17,1,4,0,36.6666679382324,21,2.33333325386047,60,3,2,212,74,0,1,0,1,1,12,17
3336,521,2011-03-17,2,4,0,37.5999984741211,22.2000007629395,0.200000002980232,60,3,2,212,
3337,521,2011-03-17,3,4,0,39.6666679382324,20.3333339691162,0,60,3,2,212,74,0,1,0,1,1,12,173
3338,521,2011-03-17,4,4,0,39,21,0,60,3,2,212,74,0,1,0,1,1,12,1731.418007,2273.983634,9.263898(
3339,521,2011-03-18,0,5,0,24,21.5,0,45.5,2,2,212,74,0,1,0,1,1,12,1731.418007,2273.983634,9.263
3340,521,2011-03-18,1,5,0,30.6666660308838,29,0.333333343267441,60,3,2,212,74,0,1,0,1,1,12,1
3341,521,2011-03-18,2,5,0,53.6666679382324,6.33333349227905,0,60,3,2,212,74,0,1,0,1,1,12,173
3342,521,2011-03-18,3,5,0,38.8333320617676,21,0.16666667163372,60,3,2,212,74,0,1,0,1,1,12,17
3343,521,2011-03-18,4,5,0,36.3333320617676,23.3333339691162,0.333333343267441,60,3,2,212,
3344,521,2011-03-19,0,6,1,22,26.3333339691162,1,49.3333320617676,3,2,212,74,0,1,0,1,1,12,173
3345,521,2011-03-19,1,6,1,36.3333320617676,22.3333339691162,1.33333337306976,60,3,2,212,7
3346,521,2011-03-19,2,6,1,45.3333320617676,14.6666669845581,0,60,3,2,212,74,0,1,0,1,1,12,173
3347,521,2011-03-19,3,6,1,34.6666679382324,24.6666660308838,0.666666686534882,60,3,2,212,
3348,521,2011-03-19,4,6,1,33.5999984741211,24.7999992370605,1.60000002384186,60,3,2,212,7
3349,521,2011-03-20,4,0,1,39,21,0,60,1,2,212,74,0,1,0,1,1,12,1731.418007,2273.983634,9.263898(

3350,521,2011-03-31,0,4,0,35,23,0.5,58.5,2,2,212,74,0,1,0,1,1,12,1731.418007,2273.983634,9.2638
3351,521,2011-03-31,1,4,0,34.6666679382324,22.6666660308838,2.66666674613953,60,3,2,212,7
3352,521,2011-03-31,2,4,0,38.5,20,1.5,60,3,2,212,74,0,1,0,1,1,12,1731.418007,2273.983634,9.2638
3353,521,2011-03-31,3,4,0,36,24,0,60,3,2,212,74,0,1,0,1,1,12,1731.418007,2273.983634,9.263898
3354,521,2011-03-31,4,4,0,39.6666679382324,20.3333339691162,0,60,3,2,212,74,0,1,0,1,1,12,173
3355,521,2011-04-05,0,2,0,16,44,0,60,1,2,212,74,0,1,0,1,1,12,1731.418007,2273.983634,9.263898
3356,521,2011-04-05,1,2,0,34.6666679382324,25,0.333333343267441,60,3,2,212,74,0,1,0,1,1,12,1
3357,521,2011-04-05,2,2,0,45.3333320617676,14.3333330154419,0.333333343267441,60,3,2,212,
3358,521,2011-04-05,3,2,0,27.6666660308838,32.3333320617676,0,60,3,2,212,74,0,1,0,1,1,12,173
3359,521,2011-04-05,4,2,0,33.3333320617676,26,0.666666686534882,60,3,2,212,74,0,1,0,1,1,12,1
3360,521,2011-04-06,0,3,0,11.75,42.25,2.25,56.25,3,2,212,74,0,1,0,1,1,12,1731.418007,2273.9836
3361,521,2011-04-06,1,3,0,25,32.3333320617676,2.66666674613953,60,3,2,212,74,0,1,0,1,1,12,17
3362,521,2011-04-06,2,3,0,40.6666679382324,15.3333330154419,4,60,3,2,212,74,0,1,0,1,1,12,173
3363,521,2011-04-06,3,3,0,36.3333320617676,23,0.666666686534882,60,3,2,212,74,0,1,0,1,1,12,1
3364,521,2011-04-06,4,3,0,32.3333320617676,27.6666660308838,0,60,3,2,212,74,0,1,0,1,1,12,173
3365,521,2011-04-07,0,4,0,24.5,25,1,50.5,2,2,212,74,0,1,0,1,1,12,1731.418007,2273.983634,9.2638
3366,521,2011-04-07,1,4,0,20,39.1666679382324,0.833333313465118,60,3,2,212,74,0,1,0,1,1,12,1
3367,521,2011-04-07,2,4,0,35.3333320617676,24.6666660308838,0,60,3,2,212,74,0,1,0,1,1,12,173
3368,521,2011-04-07,3,4,0,43.3333320617676,16.6666660308838,0,60,3,2,212,74,0,1,0,1,1,12,173
3369,521,2011-04-07,4,4,0,33,26.6666660308838,0.333333343267441,60,3,2,212,74,0,1,0,1,1,12,1
3370,524,2011-03-17,0,4,0,19.6666660308838,20.6666660308838,0,40.3333320617676,3,2,212,69
3371,524,2011-03-17,1,4,0,36.6666679382324,21,2.33333325386047,60,3,2,212,69,1,0,0,1,1,12,17
3372,524,2011-03-17,2,4,0,35.3333320617676,24.3333339691162,0.333333343267441,60,3,2,212,
3373,524,2011-03-17,3,4,0,35,24.6666660308838,0.333333343267441,60,3,2,212,69,1,0,0,1,1,12,1
3374,524,2011-03-17,4,4,0,36.5,23.25,0.25,60,3,2,212,69,1,0,0,1,1,12,1731.418007,2273.983634,9.
3375,524,2011-03-18,0,5,0,24.25,27,0.75,52,3,2,212,69,1,0,0,1,1,12,1731.418007,2273.983634,9.2
3376,524,2011-03-18,1,5,0,31.5,28.25,0.25,60,3,2,212,69,1,0,0,1,1,12,1731.418007,2273.983634,9.
3377,524,2011-03-18,2,5,0,53.6666679382324,6.33333349227905,0,60,3,2,212,69,1,0,0,1,1,12,173
3378,524,2011-03-18,3,5,0,42.6666679382324,17.3333339691162,0,60,3,2,212,69,1,0,0,1,1,12,173
3379,524,2011-03-18,4,5,0,34.3333320617676,25.6666660308838,0,60,3,2,212,69,1,0,0,1,1,12,173
3380,524,2011-03-19,1,6,1,37.5,20.5,2,60,2,2,212,69,1,0,0,1,1,12,1731.418007,2273.983634,9.2638
3381,524,2011-03-19,2,6,1,45.3333320617676,14.6666669845581,0,60,3,2,212,69,1,0,0,1,1,12,173
3382,524,2011-03-19,3,6,1,34.6666679382324,24.6666660308838,0.666666686534882,60,3,2,212,
3383,524,2011-03-19,4,6,1,34.6666679382324,22.6666660308838,2.66666674613953,60,3,2,212,6
3384,524,2011-03-20,0,0,1,13,43.6666679382324,2.66666674613953,59.3333320617676,3,2,212,6
3385,524,2011-03-20,1,0,1,28.2000007629395,30,1.79999995231628,60,3,2,212,69,1,0,0,1,1,12,17
3386,524,2011-03-20,2,0,1,45.3333320617676,14.3333330154419,0.333333343267441,60,3,2,212,
3387,524,2011-03-20,3,0,1,27.6666660308838,32.3333320617676,0,60,3,2,212,69,1,0,0,1,1,12,173
3388,524,2011-03-20,4,0,1,33.3333320617676,26,0.666666686534882,60,3,2,212,69,1,0,0,1,1,12,1
3389,524,2011-03-21,0,1,0,12,41,0.5,53.5,2,2,212,69,1,0,0,1,1,12,1731.418007,2273.983634,9.2638
3390,524,2011-03-21,1,1,0,23.75,36,0.25,60,3,2,212,69,1,0,0,1,1,12,1731.418007,2273.983634,9.2
3391,524,2011-03-21,2,1,0,38,20,2,60,3,2,212,69,1,0,0,1,1,12,1731.418007,2273.983634,9.263898
3392,524,2011-03-21,3,1,0,36.3333320617676,23,0.666666686534882,60,3,2,212,69,1,0,0,1,1,12,1
3393,524,2011-03-21,4,1,0,32.3333320617676,27.6666660308838,0,60,3,2,212,69,1,0,0,1,1,12,173
3394,524,2011-03-22,0,2,0,24.5,25,1,50.5,2,2,212,69,1,0,0,1,1,12,1731.418007,2273.983634,9.2638
3395,524,2011-03-22,1,2,0,21,37.6666679382324,1.33333337306976,60,3,2,212,69,1,0,0,1,1,12,17
3396,524,2011-03-22,2,2,0,42.6666679382324,17,0.333333343267441,60,3,2,212,69,1,0,0,1,1,12,1
3397,524,2011-03-22,3,2,0,39.6666679382324,20.3333339691162,0,60,3,2,212,69,1,0,0,1,1,12,173
3398,524,2011-03-22,4,2,0,33,26.6666660308838,0.333333343267441,60,3,2,212,69,1,0,0,1,1,12,1
3399,524,2011-03-23,0,3,0,35,23,0.5,58.5,2,2,212,69,1,0,0,1,1,12,1731.418007,2273.983634,9.2638

3400,524,2011-03-23,1,3,0,34.6666679382324,22.6666660308838,2.66666674613953,60,3,2,212,6
3401,524,2011-03-23,2,3,0,36,22.3333339691162,1.66666662693024,60,3,2,212,69,1,0,0,1,1,12,17
3402,524,2011-03-23,3,3,0,39.6666679382324,20.3333339691162,0,60,3,2,212,69,1,0,0,1,1,12,173
3403,524,2011-03-23,4,3,0,39.7999992370605,20.2000007629395,0,60,3,2,212,69,1,0,0,1,1,12,173
3404,536,2011-03-17,0,4,0,24,21.5,0,45.5,2,2,212,77,1,0,1,0,1,12,1778.425352,1840.084206,7.4937
3405,536,2011-03-17,1,4,0,30.6666660308838,29,0.333333343267441,60,3,2,212,77,1,0,1,0,1,12,1
3406,536,2011-03-17,2,4,0,47.5999984741211,12.1999998092651,0.200000002980232,60,3,2,212,
3407,536,2011-03-17,3,4,0,35,24.6666660308838,0.333333343267441,60,3,2,212,77,1,0,1,0,1,12,1
3408,536,2011-03-17,4,4,0,36.3333320617676,23.3333339691162,0.333333343267441,60,3,2,212,
3409,536,2011-03-18,0,5,0,22,26.3333339691162,1,49.3333320617676,3,2,212,77,1,0,1,0,1,12,177
3410,536,2011-03-18,1,5,0,36.3333320617676,22.3333339691162,1.33333337306976,60,3,2,212,7
3411,536,2011-03-18,2,5,0,45.3333320617676,14.6666669845581,0,60,3,2,212,77,1,0,1,0,1,12,177
3412,536,2011-03-18,3,5,0,38.4000015258789,21.6000003814697,0,60,3,2,212,77,1,0,1,0,1,12,177
3413,536,2011-03-18,4,5,0,34.3333320617676,25.6666660308838,0,60,3,2,212,77,1,0,1,0,1,12,177
3414,536,2011-03-19,0,6,1,16,44,0,60,1,2,212,77,1,0,1,0,1,12,1778.425352,1840.084206,7.4937325
3415,536,2011-03-19,1,6,1,34.6666679382324,25,0.333333343267441,60,3,2,212,77,1,0,1,0,1,12,1
3416,536,2011-03-19,2,6,1,43.5,16,0.5,60,2,2,212,77,1,0,1,0,1,12,1778.425352,1840.084206,7.4937
3417,536,2011-03-19,3,6,1,40,18,2,60,1,2,212,77,1,0,1,0,1,12,1778.425352,1840.084206,7.4937325
3418,536,2011-03-19,4,6,1,34.6666679382324,22.6666660308838,2.66666674613953,60,3,2,212,7
3419,536,2011-03-20,0,0,1,11.5,43.5,4,59,2,2,212,77,1,0,1,0,1,12,1778.425352,1840.084206,7.4937
3420,536,2011-03-20,1,0,1,25,32.3333320617676,2.66666674613953,60,3,2,212,77,1,0,1,0,1,12,17
3421,536,2011-03-20,2,0,1,42.75,14.25,3,60,3,2,212,77,1,0,1,0,1,12,1778.425352,1840.084206,7.49
3422,536,2011-03-20,3,0,1,32,27.6666660308838,0.333333343267441,60,3,2,212,77,1,0,1,0,1,12,1
3423,536,2011-03-20,4,0,1,33.3333320617676,26,0.666666686534882,60,3,2,212,77,1,0,1,0,1,12,1
3424,536,2011-03-21,0,1,0,14.3333330154419,34.3333320617676,0.666666686534882,49.333332
3425,536,2011-03-21,1,1,0,19,40.6666679382324,0.333333343267441,60,3,2,212,77,1,0,1,0,1,12,1
3426,536,2011-03-21,2,1,0,35.3333320617676,24.6666660308838,0,60,3,2,212,77,1,0,1,0,1,12,177
3427,536,2011-03-21,3,1,0,43.3333320617676,16.6666660308838,0,60,3,2,212,77,1,0,1,0,1,12,177
3428,536,2011-03-21,4,1,0,32.6666679382324,27.1666660308838,0.16666667163372,60,3,2,212,7
3429,536,2011-03-22,0,2,0,33.3333320617676,25,0.666666686534882,59,3,2,212,77,1,0,1,0,1,12,1
3430,536,2011-03-22,1,2,0,24,33.5999984741211,2.40000009536743,60,3,2,212,77,1,0,1,0,1,12,17
3431,536,2011-03-22,2,2,0,42.6666679382324,17,0.333333343267441,60,3,2,212,77,1,0,1,0,1,12,1
3432,536,2011-03-22,3,2,0,36,24,0,60,3,2,212,77,1,0,1,0,1,12,1778.425352,1840.084206,7.4937325
3433,536,2011-03-22,4,2,0,39.6666679382324,20.3333339691162,0,60,3,2,212,77,1,0,1,0,1,12,177
3434,536,2011-03-23,0,3,0,21,24,0,45,2,2,212,77,1,0,1,0,1,12,1778.425352,1840.084206,7.4937325
3435,536,2011-03-23,1,3,0,39.25,19,1.75,60,3,2,212,77,1,0,1,0,1,12,1778.425352,1840.084206,7.49
3436,536,2011-03-23,2,3,0,34.25,24.5,1.25,60,3,2,212,77,1,0,1,0,1,12,1778.425352,1840.084206,7.
3437,536,2011-03-23,3,3,0,39.6666679382324,20.3333339691162,0,60,3,2,212,77,1,0,1,0,1,12,177
3438,536,2011-03-23,4,3,0,39,21,0,60,3,2,212,77,1,0,1,0,1,12,1778.425352,1840.084206,7.4937325
3439,537,2011-03-17,0,4,0,21.6666660308838,18,0,39.6666679382324,3,2,212,69,0,0,0,1,1,12,179
3440,537,2011-03-17,1,4,0,30.6666660308838,29,0.333333343267441,60,3,2,212,69,0,0,0,1,1,12,1
3441,537,2011-03-17,2,4,0,53.6666679382324,6.33333349227905,0,60,3,2,212,69,0,0,0,1,1,12,179
3442,537,2011-03-17,3,4,0,42.6666679382324,17.3333339691162,0,60,3,2,212,69,0,0,0,1,1,12,179
3443,537,2011-03-17,4,4,0,33.75,26.25,0,60,3,2,212,69,0,0,0,1,1,12,1799.997799,1810.372266,6.06
3444,537,2011-03-18,0,5,0,24.5,34,1.5,60,2,2,212,69,0,0,0,1,1,12,1799.997799,1810.372266,6.0648
3445,537,2011-03-18,1,5,0,36.3333320617676,22.3333339691162,1.33333337306976,60,3,2,212,6
3446,537,2011-03-18,2,5,0,45.3333320617676,14.6666669845581,0,60,3,2,212,69,0,0,0,1,1,12,179
3447,537,2011-03-18,3,5,0,34.6666679382324,24.6666660308838,0.666666686534882,60,3,2,212,
3448,537,2011-03-18,4,5,0,34.6666679382324,22.6666660308838,2.66666674613953,60,3,2,212,6
3449,537,2011-03-19,0,6,1,16,44,0,60,1,2,212,69,0,0,0,1,1,12,1799.997799,1810.372266,6.064885

3450,537,2011-03-19,1,6,1,34.6666679382324,25,0.333333343267441,60,3,2,212,69,0,0,0,1,1,12,1
3451,537,2011-03-19,2,6,1,45.3333320617676,14.3333330154419,0.333333343267441,60,3,2,212,
3452,537,2011-03-19,3,6,1,27.6666660308838,32.3333320617676,0,60,3,2,212,69,0,0,0,1,1,12,179
3453,537,2011-03-19,4,6,1,33.3333320617676,26,0.666666686534882,60,3,2,212,69,0,0,0,1,1,12,1
3454,537,2011-03-20,0,0,1,11.75,42.25,2.25,56.25,3,2,212,69,0,0,0,1,1,12,1799.997799,1810.3722
3455,537,2011-03-20,1,0,1,22.7999992370605,35.4000015258789,1.79999995231628,60,3,2,212,6
3456,537,2011-03-20,2,0,1,40.6666679382324,15.3333330154419,4,60,3,2,212,69,0,0,0,1,1,12,179
3457,537,2011-03-20,3,0,1,36.3333320617676,23,0.666666686534882,60,3,2,212,69,0,0,0,1,1,12,1
3458,537,2011-03-20,4,0,1,32.3333320617676,27.6666660308838,0,60,3,2,212,69,0,0,0,1,1,12,179
3459,537,2011-03-21,0,1,0,24.5,25,1,50.5,2,2,212,69,0,0,0,1,1,12,1799.997799,1810.372266,6.064
3460,537,2011-03-21,1,1,0,20.25,38.75,1,60,3,2,212,69,0,0,0,1,1,12,1799.997799,1810.372266,6.0
3461,537,2011-03-21,2,1,0,39,20.8333339691162,0.16666667163372,60,3,2,212,69,0,0,0,1,1,12,17
3462,537,2011-03-21,3,1,0,43.3333320617676,16.6666660308838,0,60,3,2,212,69,0,0,0,1,1,12,179
3463,537,2011-03-21,4,1,0,33,26.6666660308838,0.333333343267441,60,3,2,212,69,0,0,0,1,1,12,1
3464,537,2011-03-22,0,2,0,35,23,0.5,58.5,2,2,212,69,0,0,0,1,1,12,1799.997799,1810.372266,6.064
3465,537,2011-03-22,1,2,0,34.6666679382324,22.6666660308838,2.66666674613953,60,3,2,212,6
3466,537,2011-03-22,2,2,0,36,22.3333339691162,1.66666662693024,60,3,2,212,69,0,0,0,1,1,12,17
3467,537,2011-03-22,3,2,0,37.8333320617676,22.1666660308838,0,60,3,2,212,69,0,0,0,1,1,12,179
3468,537,2011-03-22,4,2,0,39.6666679382324,20.3333339691162,0,60,3,2,212,69,0,0,0,1,1,12,179
3469,537,2011-03-23,0,3,0,21,24,0,45,2,2,212,69,0,0,0,1,1,12,1799.997799,1810.372266,6.064885
3470,537,2011-03-23,1,3,0,36.6666679382324,21,2.33333325386047,60,3,2,212,69,0,0,0,1,1,12,17
3471,537,2011-03-23,2,3,0,35.3333320617676,24.3333339691162,0.333333343267441,60,3,2,212,
3472,537,2011-03-23,3,3,0,35,24.6666660308838,0.333333343267441,60,3,2,212,69,0,0,0,1,1,12,1
3473,537,2011-03-23,4,3,0,38.7999992370605,21,0.200000002980232,60,3,2,212,69,0,0,0,1,1,12,1
3474,543,2011-03-18,0,5,0,16,44,0,60,1,2,212,78,1,0,1,1,1,9,1595.387563,1587.706318,1.3917359
3475,543,2011-03-18,1,5,0,33.5,26,0.5,60,2,2,212,78,1,0,1,1,1,9,1595.387563,1587.706318,1.3917
3476,543,2011-03-19,0,6,1,11.5,43.5,4,59,2,2,212,78,1,0,1,1,1,9,1595.387563,1587.706318,1.3917
3477,543,2011-03-19,1,6,1,28,30,2,60,3,2,212,78,1,0,1,1,1,9,1595.387563,1587.706318,1.3917359
3478,543,2011-03-19,2,6,1,45.5999984741211,13,1.39999997615814,60,3,2,212,78,1,0,1,1,1,9,159
3479,543,2011-03-19,3,6,1,27.6666660308838,32.3333320617676,0,60,3,2,212,78,1,0,1,1,1,9,1595
3480,543,2011-03-19,4,6,1,33.3333320617676,26,0.666666686534882,60,3,2,212,78,1,0,1,1,1,9,15
3481,543,2011-03-20,0,0,1,12,41,0.5,53.5,2,2,212,78,1,0,1,1,1,9,1595.387563,1587.706318,1.3917
3482,543,2011-03-20,1,0,1,19,40.6666679382324,0.333333343267441,60,3,2,212,78,1,0,1,1,1,9,15
3483,543,2011-03-20,2,0,1,34,24.5,1.5,60,3,2,212,78,1,0,1,1,1,9,1595.387563,1587.706318,1.3917
3484,543,2011-03-20,3,0,1,39.8333320617676,19.8333339691162,0.333333343267441,60,3,2,212,
3485,543,2011-03-20,4,0,1,32.3333320617676,27.6666660308838,0,60,3,2,212,78,1,0,1,1,1,9,1595
3486,543,2011-03-21,0,1,0,25,26.6666660308838,1,52.6666679382324,3,2,212,78,1,0,1,1,1,9,1595
3487,543,2011-03-21,1,1,0,21,37.6666679382324,1.33333337306976,60,3,2,212,78,1,0,1,1,1,9,159
3488,543,2011-03-21,2,1,0,42.6666679382324,17,0.333333343267441,60,3,2,212,78,1,0,1,1,1,9,15
3489,543,2011-03-21,3,1,0,36,24,0,60,3,2,212,78,1,0,1,1,1,9,1595.387563,1587.706318,1.3917359
3490,543,2011-03-21,4,1,0,36.3333320617676,23.5,0.16666667163372,60,3,2,212,78,1,0,1,1,1,9,15
3491,543,2011-03-22,0,2,0,28.6666660308838,21.3333339691162,0,50,3,2,212,78,1,0,1,1,1,9,1595
3492,543,2011-03-22,1,2,0,34.2000007629395,22.7999992370605,3,60,3,2,212,78,1,0,1,1,1,9,1595
3493,543,2011-03-22,2,2,0,36,22.3333339691162,1.66666662693024,60,3,2,212,78,1,0,1,1,1,9,159
3494,543,2011-03-22,3,2,0,39.6666679382324,20.3333339691162,0,60,3,2,212,78,1,0,1,1,1,9,1595
3495,543,2011-03-22,4,2,0,39,21,0,60,3,2,212,78,1,0,1,1,1,9,1595.387563,1587.706318,1.3917359
3496,543,2011-03-23,0,3,0,24,21.5,0,45.5,2,2,212,78,1,0,1,1,1,9,1595.387563,1587.706318,1.3917
3497,543,2011-03-23,1,3,0,33.75,26,0.25,60,3,2,212,78,1,0,1,1,1,9,1595.387563,1587.706318,1.391
3498,543,2011-03-23,2,3,0,39.25,20.5,0.25,60,3,2,212,78,1,0,1,1,1,9,1595.387563,1587.706318,1.3
3499,543,2011-03-23,3,3,0,35,24.6666660308838,0.333333343267441,60,3,2,212,78,1,0,1,1,1,9,15

3500,543,2011-03-23,4,3,0,36.3333320617676,23.3333339691162,0.333333343267441,60,3,2,212,
3501,543,2011-04-14,0,4,0,22,26.3333339691162,1,49.3333320617676,3,2,212,78,1,0,1,1,1,9,1595
3502,543,2011-04-14,1,4,0,36.3333320617676,22.3333339691162,1.33333337306976,60,3,2,212,7
3503,543,2011-04-14,2,4,0,48.6666679382324,11.3333330154419,0,60,3,2,212,78,1,0,1,1,1,9,1595
3504,543,2011-04-14,3,4,0,42.6666679382324,17.3333339691162,0,60,3,2,212,78,1,0,1,1,1,9,1595
3505,543,2011-04-14,4,4,0,34.3333320617676,25.6666660308838,0,60,3,2,212,78,1,0,1,1,1,9,1595
3506,543,2011-04-15,2,5,0,50,10,0,60,2,2,212,78,1,0,1,1,1,9,1595.387563,1587.706318,1.39173593
3507,543,2011-04-15,3,5,0,34.6666679382324,24.6666660308838,0.666666686534882,60,3,2,212,
3508,543,2011-04-15,4,5,0,34.6666679382324,22.6666660308838,2.66666674613953,60,3,2,212,7
3509,660,2011-03-17,0,4,0,22,29,1,52,2,2,206,66,0,1,0,1,1,9,11624.47064,9150.85946,10.13704895
3510,660,2011-03-17,1,4,0,25.7999992370605,32.7999992370605,1.39999997615814,60,3,2,206,6
3511,660,2011-03-17,2,4,0,26.3333339691162,30.3333339691162,3.33333325386047,60,3,2,206,6
3512,660,2011-03-17,3,4,0,33,27,0,60,3,2,206,66,0,1,0,1,1,9,11624.47064,9150.85946,10.13704895
3513,660,2011-03-17,4,4,0,24,33,3,60,3,2,206,66,0,1,0,1,1,9,11624.47064,9150.85946,10.13704895
3514,660,2011-03-18,0,5,0,21,25.5,0,46.5,2,2,206,66,0,1,0,1,1,9,11624.47064,9150.85946,10.13704
3515,660,2011-03-18,1,5,0,6,51.3333320617676,2.66666674613953,60,3,2,206,66,0,1,0,1,1,9,1162
3516,660,2011-03-18,2,5,0,31.1666660308838,25.1666660308838,3.66666674613953,60,3,2,206,6
3517,660,2011-03-18,3,5,0,8,39.3333320617676,12.6666669845581,60,3,2,206,66,0,1,0,1,1,9,1162
3518,660,2011-03-18,4,5,0,12,42.6666679382324,5.33333349227905,60,3,2,206,66,0,1,0,1,1,9,116
3519,660,2011-03-19,3,6,1,21,34.3333320617676,4.66666650772095,60,3,2,206,66,0,1,0,1,1,9,116
3520,660,2011-03-19,4,6,1,31.6666660308838,27,1.33333337306976,60,3,2,206,66,0,1,0,1,1,9,116
3521,660,2011-04-15,0,5,0,20,40,0,60,1,2,206,66,0,1,0,1,1,9,11624.47064,9150.85946,10.13704895
3522,660,2011-04-15,1,5,0,16.3333339691162,39,4.66666650772095,60,3,2,206,66,0,1,0,1,1,9,116
3523,660,2011-04-15,2,5,0,41.5,17,1.5,60,2,2,206,66,0,1,0,1,1,9,11624.47064,9150.85946,10.13704
3524,660,2011-04-16,0,6,1,9.5,39,1,49.5,2,2,206,66,0,1,0,1,1,9,11624.47064,9150.85946,10.137048
3525,660,2011-04-16,1,6,1,11.3333330154419,48.6666679382324,0,60,3,2,206,66,0,1,0,1,1,9,1162
3526,660,2011-04-16,2,6,1,6,54,0,60,1,2,206,66,0,1,0,1,1,9,11624.47064,9150.85946,10.13704895,
3527,660,2011-04-16,3,6,1,5,53.3333320617676,1.66666662693024,60,3,2,206,66,0,1,0,1,1,9,1162
3528,660,2011-04-16,4,6,1,24.6666660308838,34.3333320617676,1,60,3,2,206,66,0,1,0,1,1,9,1162
3529,660,2011-04-17,0,0,1,21.3333339691162,34,4.66666650772095,60,3,2,206,66,0,1,0,1,1,9,116
3530,660,2011-04-17,1,0,1,15.5,38,6.5,60,2,2,206,66,0,1,0,1,1,9,11624.47064,9150.85946,10.13704
3531,660,2011-04-17,2,0,1,33.6666679382324,25.6666660308838,0.666666686534882,60,3,2,206,
3532,660,2011-04-17,3,0,1,9.33333301544189,44.3333320617676,6.33333349227905,60,3,2,206,6
3533,660,2011-04-17,4,0,1,19.6666660308838,31.6666660308838,8.66666698455811,60,3,2,206,6
3534,660,2011-04-18,1,1,0,28,30.25,1.75,60,3,2,206,66,0,1,0,1,1,9,11624.47064,9150.85946,10.137
3535,660,2011-04-18,2,1,0,26.3999996185303,28.7999992370605,4.80000019073486,60,3,2,206,6
3536,660,2011-04-18,3,1,0,20,39.3333320617676,0.666666686534882,60,3,2,206,66,0,1,0,1,1,9,11
3537,660,2011-04-18,4,1,0,44,12.3333330154419,3.66666674613953,60,3,2,206,66,0,1,0,1,1,9,116
3538,660,2011-04-19,0,2,0,10.5,32.5,0.5,43.5,2,2,206,66,0,1,0,1,1,9,11624.47064,9150.85946,10.13
3539,660,2011-04-19,1,2,0,18.3333339691162,41.3333320617676,0.333333343267441,60,3,2,206,
3540,660,2011-04-19,2,2,0,0,49,11,60,1,2,206,66,0,1,0,1,1,9,11624.47064,9150.85946,10.13704895
3541,660,2011-04-19,3,2,0,4.33333349227905,54,1.66666662693024,60,3,2,206,66,0,1,0,1,1,9,116
3542,660,2011-04-19,4,2,0,12,43.6666679382324,4.33333349227905,60,3,2,206,66,0,1,0,1,1,9,116
3543,660,2011-04-20,0,3,0,5,28,8,41,2,2,206,66,0,1,0,1,1,9,11624.47064,9150.85946,10.13704895,
3544,660,2011-04-20,1,3,0,28,32,0,60,1,2,206,66,0,1,0,1,1,9,11624.47064,9150.85946,10.13704895
3545,660,2011-04-20,2,3,0,35.3333320617676,24,0.666666686534882,60,3,2,206,66,0,1,0,1,1,9,11
3546,660,2011-04-20,3,3,0,20.3333339691162,34.3333320617676,5.33333349227905,60,3,2,206,6
3547,660,2011-04-20,4,3,0,14.6666669845581,42.3333320617676,3,60,3,2,206,66,0,1,0,1,1,9,1162
3548,664,2011-03-17,0,4,0,33,27,0,60,1,2,206,88,1,1,0,0,1,9,11448.26986,8704.323754,10.0560743
3549,664,2011-03-17,1,4,0,13.3333330154419,32.6666679382324,14,60,3,2,206,88,1,1,0,0,1,9,114

3550,664,2011-03-17,2,4,0,36.3333320617676,23.3333339691162,0.333333343267441,60,3,2,206,
3551,664,2011-03-17,3,4,0,21.25,37.75,1,60,3,2,206,88,1,1,0,0,1,9,11448.26986,8704.323754,10.05
3552,664,2011-03-17,4,4,0,45.4000015258789,14.6000003814697,0,60,3,2,206,88,1,1,0,0,1,9,1144
3553,664,2011-03-18,4,5,0,10,5,0,15,1,2,206,88,1,1,0,0,1,9,11448.26986,8704.323754,10.0560743
3554,664,2011-03-19,0,6,1,13,29.3333339691162,0,42.3333320617676,3,2,206,88,1,1,0,0,1,9,1144
3555,664,2011-03-19,1,6,1,13.6666669845581,42,4.33333349227905,60,3,2,206,88,1,1,0,0,1,9,114
3556,664,2011-03-19,2,6,1,27,33,0,60,3,2,206,88,1,1,0,0,1,9,11448.26986,8704.323754,10.056074
3557,664,2011-03-19,3,6,1,26,34,0,60,2,2,206,88,1,1,0,0,1,9,11448.26986,8704.323754,10.056074
3558,664,2011-03-20,0,0,1,19.5,29,0.5,49,2,2,206,88,1,1,0,0,1,9,11448.26986,8704.323754,10.056
3559,664,2011-03-20,1,0,1,16.3333339691162,43.6666679382324,0,60,3,2,206,88,1,1,0,0,1,9,1144
3560,664,2011-03-20,2,0,1,39.3333320617676,20.6666660308838,0,60,3,2,206,88,1,1,0,0,1,9,1144
3561,664,2011-03-20,3,0,1,29.5,30.5,0,60,3,2,206,88,1,1,0,0,1,9,11448.26986,8704.323754,10.056
3562,664,2011-03-20,4,0,1,38.5,16.1666660308838,0.16666667163372,54.8333320617676,3,2,206
3563,664,2011-03-21,0,1,0,12.5,23.75,0.5,36.75,3,2,206,88,1,1,0,0,1,9,11448.26986,8704.323754,1
3564,664,2011-03-21,1,1,0,15.8000001907349,36.4000015258789,7.80000019073486,60,3,2,206,8
3565,664,2011-03-21,2,1,0,37,23,0,60,3,2,206,88,1,1,0,0,1,9,11448.26986,8704.323754,10.056074
3566,664,2011-03-21,3,1,0,35,25,0,60,3,2,206,88,1,1,0,0,1,9,11448.26986,8704.323754,10.056074
3567,664,2011-03-21,4,1,0,44.6666679382324,15.3333330154419,0,60,3,2,206,88,1,1,0,0,1,9,1144
3568,664,2011-03-22,0,2,0,25.5,32,0,57.5,2,2,206,88,1,1,0,0,1,9,11448.26986,8704.323754,10.056
3569,664,2011-03-22,1,2,0,20,32.75,7.25,60,3,2,206,88,1,1,0,0,1,9,11448.26986,8704.323754,10.05
3570,664,2011-03-22,2,2,0,38.4000015258789,20.3999996185303,1.20000004768372,60,3,2,206,8
3571,664,2011-03-22,3,2,0,29,27.6666660308838,3.33333325386047,60,3,2,206,88,1,1,0,0,1,9,114
3572,664,2011-03-22,4,2,0,42.3333320617676,11,0.666666686534882,54,3,2,206,88,1,1,0,0,1,9,11
3573,664,2011-03-23,0,3,0,21,27,7,55,1,2,206,88,1,1,0,0,1,9,11448.26986,8704.323754,10.056074
3574,664,2011-03-23,1,3,0,25,29.3333339691162,5.66666650772095,60,3,2,206,88,1,1,0,0,1,9,114
3575,664,2011-03-23,2,3,0,32,27.5,0.5,60,3,2,206,88,1,1,0,0,1,9,11448.26986,8704.323754,10.056
3576,664,2011-03-23,3,3,0,31.6000003814697,28,0.400000005960464,60,3,2,206,88,1,1,0,0,1,9,11
3577,664,2011-03-23,4,3,0,39.3333320617676,14,0,53.3333320617676,3,2,206,88,1,1,0,0,1,9,1144
3578,666,2011-03-18,0,5,0,57,2,1,60,1,2,206,84,1,0,1,0,1,9,11448.26986,8704.323754,10.0560743
3579,666,2011-03-18,1,5,0,20,36.8333320617676,0.5,57.3333320617676,3,2,206,84,1,0,1,0,1,9,114
3580,666,2011-03-18,2,5,0,32.6666679382324,27.3333339691162,0,60,3,2,206,84,1,0,1,0,1,9,1144
3581,666,2011-03-18,3,5,0,13.6666669845581,46,0.333333343267441,60,3,2,206,84,1,0,1,0,1,9,11
3582,666,2011-03-18,4,5,0,20,24,0.666666686534882,44.6666679382324,3,2,206,84,1,0,1,0,1,9,11
3583,666,2011-03-19,1,6,1,8.33333301544189,35.6666679382324,1,45,3,2,206,84,1,0,1,0,1,9,1144
3584,666,2011-03-19,2,6,1,28.6666660308838,31,0.333333343267441,60,3,2,206,84,1,0,1,0,1,9,11
3585,666,2011-03-19,3,6,1,22.2000007629395,37,0.800000011920929,60,3,2,206,84,1,0,1,0,1,9,11
3586,666,2011-03-19,4,6,1,34.3333320617676,14.6666669845581,0.333333343267441,49.333332
3587,666,2011-03-20,1,0,1,7.40000009536743,35.2000007629395,0.600000023841858,43.200000
3588,666,2011-03-20,2,0,1,21.3333339691162,38.3333320617676,0.333333343267441,60,3,2,206,
3589,666,2011-03-20,3,0,1,11,48.25,0.75,60,3,2,206,84,1,0,1,0,1,9,11448.26986,8704.323754,10.05
3590,666,2011-03-20,4,0,1,21,26.1666660308838,0.333333343267441,47.5,3,2,206,84,1,0,1,0,1,9,1
3591,666,2011-03-21,1,1,0,21,37,0.25,58.25,3,2,206,84,1,0,1,0,1,9,11448.26986,8704.323754,10.05
3592,666,2011-03-21,2,1,0,20.6666660308838,38.5,0.833333313465118,60,3,2,206,84,1,0,1,0,1,9,1
3593,666,2011-03-21,3,1,0,21.3999996185303,38.5999984741211,0,60,3,2,206,84,1,0,1,0,1,9,1144
3594,666,2011-03-21,4,1,0,34.6666679382324,25.3333339691162,0,60,3,2,206,84,1,0,1,0,1,9,1144
3595,666,2011-03-22,0,2,0,0,5,1,6,1,2,206,84,1,0,1,0,1,9,11448.26986,8704.323754,10.05607435,1
3596,666,2011-03-22,1,2,0,9,51,0,60,3,2,206,84,1,0,1,0,1,9,11448.26986,8704.323754,10.0560743
3597,666,2011-03-22,2,2,0,11.3333330154419,48.6666679382324,0,60,3,2,206,84,1,0,1,0,1,9,1144
3598,666,2011-03-22,3,2,0,13.25,46.75,0,60,3,2,206,84,1,0,1,0,1,9,11448.26986,8704.323754,10.05
3599,666,2011-03-22,4,2,0,43.8333320617676,16.1666660308838,0,60,3,2,206,84,1,0,1,0,1,9,1144

3600,666,2011-03-23,0,3,0,9,12,1,22,1,2,206,84,1,0,1,0,1,9,11448.26986,8704.323754,10.0560743
3601,666,2011-03-23,1,3,0,15,45,0,60,3,2,206,84,1,0,1,0,1,9,11448.26986,8704.323754,10.056074
3602,666,2011-03-23,2,3,0,18.3333339691162,41.6666679382324,0,60,3,2,206,84,1,0,1,0,1,9,1144
3603,666,2011-03-23,3,3,0,19.3333339691162,40.6666679382324,0,60,3,2,206,84,1,0,1,0,1,9,1144
3604,666,2011-03-23,4,3,0,18.3333339691162,22.3333339691162,0.333333343267441,41,3,2,206,
3605,667,2011-03-23,1,3,0,13,27.6666660308838,1,41.6666679382324,3,2,206,83,1,1,0,1,1,8,1040
3606,667,2011-04-14,1,4,0,28,14.6666669845581,0,42.6666679382324,3,2,206,83,1,1,0,1,1,8,1040
3607,667,2011-04-14,2,4,0,30.3333339691162,27.8333339691162,1.83333337306976,60,3,2,206,8
3608,667,2011-04-14,3,4,0,34,26,0,60,3,2,206,83,1,1,0,1,1,8,10408.71557,8725.475982,8.7852425
3609,667,2011-04-14,4,4,0,43.6666679382324,16.3333339691162,0,60,3,2,206,83,1,1,0,1,1,8,1040
3610,667,2011-04-15,1,5,0,31,23.5,0,54.5,2,2,206,83,1,1,0,1,1,8,10408.71557,8725.475982,8.7852
3611,667,2011-04-15,2,5,0,35.3333320617676,24.6666660308838,0,60,3,2,206,83,1,1,0,1,1,8,1040
3612,667,2011-04-15,3,5,0,27.3333339691162,32.5,0.16666667163372,60,3,2,206,83,1,1,0,1,1,8,10
3613,667,2011-04-15,4,5,0,41,19,0,60,3,2,206,83,1,1,0,1,1,8,10408.71557,8725.475982,8.7852425
3614,667,2011-04-16,1,6,1,23.75,17.25,0.25,41.25,3,2,206,83,1,1,0,1,1,8,10408.71557,8725.47598
3615,667,2011-04-16,2,6,1,34.3333320617676,25.6666660308838,0,60,3,2,206,83,1,1,0,1,1,8,1040
3616,667,2011-04-16,3,6,1,21.6666660308838,38.3333320617676,0,60,3,2,206,83,1,1,0,1,1,8,1040
3617,667,2011-04-16,4,6,1,35.3333320617676,24.6666660308838,0,60,3,2,206,83,1,1,0,1,1,8,1040
3618,667,2011-04-17,1,0,1,42,13.6666669845581,0.333333343267441,56,3,2,206,83,1,1,0,1,1,8,10
3619,667,2011-04-17,2,0,1,27.5,32.5,0,60,3,2,206,83,1,1,0,1,1,8,10408.71557,8725.475982,8.7852
3620,667,2011-04-17,3,0,1,17.3999996185303,42.5999984741211,0,60,3,2,206,83,1,1,0,1,1,8,1040
3621,667,2011-04-17,4,0,1,29.6666660308838,30.3333339691162,0,60,3,2,206,83,1,1,0,1,1,8,1040
3622,667,2011-04-18,3,1,0,28,32,0,60,1,2,206,83,1,1,0,1,1,8,10408.71557,8725.475982,8.7852425
3623,667,2011-04-18,4,1,0,31.6666660308838,28.3333339691162,0,60,3,2,206,83,1,1,0,1,1,8,1040
3624,670,2011-04-18,0,1,0,43,12,5,60,1,2,206,73,0,0,0,0,1,11,11448.26986,8704.323754,10.05607
3625,670,2011-04-18,1,1,0,22.3333339691162,37,0.666666686534882,60,3,2,206,73,0,0,0,0,1,11,1
3626,670,2011-04-18,2,1,0,31.3333339691162,25,3.66666674613953,60,3,2,206,73,0,0,0,0,1,11,11
3627,670,2011-04-18,3,1,0,49.6666679382324,10.3333330154419,0,60,3,2,206,73,0,0,0,0,1,11,114
3628,670,2011-04-19,0,2,0,35.5,6,0,41.5,2,2,206,73,0,0,0,0,1,11,11448.26986,8704.323754,10.056
3629,670,2011-04-19,1,2,0,14.3333330154419,41.6666679382324,4,60,3,2,206,73,0,0,0,0,1,11,114
3630,670,2011-04-19,2,2,0,9.33333301544189,45,5.66666650772095,60,3,2,206,73,0,0,0,0,1,11,11
3631,670,2011-04-19,3,2,0,21.3333339691162,32,6.66666650772095,60,3,2,206,73,0,0,0,0,1,11,11
3632,670,2011-04-19,4,2,0,55.5,4.5,0,60,3,2,206,73,0,0,0,0,1,11,11448.26986,8704.323754,10.056
3633,670,2011-04-20,0,3,0,25,6,0.5,31.5,2,2,206,73,0,0,0,0,1,11,11448.26986,8704.323754,10.056
3634,670,2011-04-20,1,3,0,38.3333320617676,19.6666660308838,2,60,3,2,206,73,0,0,0,0,1,11,114
3635,670,2011-04-20,2,3,0,29,31,0,60,3,2,206,73,0,0,0,0,1,11,11448.26986,8704.323754,10.05607
3636,670,2011-04-20,3,3,0,29.3333339691162,30.6666660308838,0,60,3,2,206,73,0,0,0,0,1,11,114
3637,670,2011-04-20,4,3,0,55.75,4,0.25,60,3,2,206,73,0,0,0,0,1,11,11448.26986,8704.323754,10.0
3638,670,2011-04-22,0,5,0,1,30,1,32,1,2,206,73,0,0,0,0,1,11,11448.26986,8704.323754,10.056074
3639,670,2011-04-22,1,5,0,28.6000003814697,25.2000007629395,5,58.7999992370605,3,2,206,73
3640,670,2011-04-22,2,5,0,47.6666679382324,12.3333330154419,0,60,3,2,206,73,0,0,0,0,1,11,114
3641,670,2011-04-22,3,5,0,40.6666679382324,17.3333339691162,2,60,3,2,206,73,0,0,0,0,1,11,114
3642,670,2011-04-22,4,5,0,51.5,7.75,0.75,60,3,2,206,73,0,0,0,0,1,11,11448.26986,8704.323754,10.
3643,670,2011-04-23,0,6,1,21,21.5,3.5,46,2,2,206,73,0,0,0,0,1,11,11448.26986,8704.323754,10.056
3644,670,2011-04-23,1,6,1,19.6666660308838,24.3333339691162,0.333333343267441,44.333332
3645,670,2011-04-23,2,6,1,35.5,18.1666660308838,6.33333349227905,60,3,2,206,73,0,0,0,0,1,11,
3646,670,2011-04-23,3,6,1,9,36.3333320617676,14.6666669845581,60,3,2,206,73,0,0,0,0,1,11,114
3647,670,2011-04-23,4,6,1,53,7,0,60,3,2,206,73,0,0,0,0,1,11,11448.26986,8704.323754,10.056074
3648,670,2011-04-24,0,0,1,0,9,8,17,1,2,206,73,0,0,0,0,1,11,11448.26986,8704.323754,10.0560743
3649,670,2011-04-24,1,0,1,40.3333320617676,19.3333339691162,0.333333343267441,60,3,2,206,

3650,670,2011-04-24,2,0,1,33.6666679382324,26,0.333333343267441,60,3,2,206,73,0,0,0,0,1,11,1
3651,670,2011-04-24,3,0,1,41.8333320617676,18,0.16666667163372,60,3,2,206,73,0,0,0,0,1,11,11
3652,670,2011-04-24,4,0,1,55.6666679382324,4.33333349227905,0,60,3,2,206,73,0,0,0,0,1,11,114
3653,670,2011-04-25,0,1,0,1,18,10,29,1,2,206,73,0,0,0,0,1,11,11448.26986,8704.323754,10.056074
3654,670,2011-04-25,1,1,0,41,17,2,60,3,2,206,73,0,0,0,0,1,11,11448.26986,8704.323754,10.056074
3655,670,2011-04-25,2,1,0,45.6666679382324,14.3333330154419,0,60,3,2,206,73,0,0,0,0,1,11,114
3656,670,2011-04-25,3,1,0,48,12,0,60,3,2,206,73,0,0,0,0,1,11,11448.26986,8704.323754,10.056074
3657,670,2011-04-25,4,1,0,51,2.16666674613953,0,53.1666679382324,3,2,206,73,0,0,0,0,1,11,114
3658,671,2011-04-25,0,1,0,60,0,0,60,1,2,206,73,1,1,1,0,1,10,11744.4059,9715.281987,9.560421271
3659,671,2011-04-26,1,2,0,32,22.6666660308838,3.33333325386047,58,3,2,206,73,1,1,1,0,1,10,11
3660,671,2011-04-26,2,2,0,45,14.3999996185303,0.600000023841858,60,3,2,206,73,1,1,1,0,1,10,1
3661,671,2011-04-26,3,2,0,36.6666679382324,21.3333339691162,2,60,3,2,206,73,1,1,1,0,1,10,117
3662,671,2011-04-26,4,2,0,41,18.6666660308838,0.333333343267441,60,3,2,206,73,1,1,1,0,1,10,1
3663,671,2011-04-27,0,3,0,3,12,1,16,1,2,206,73,1,1,1,0,1,10,11744.4059,9715.281987,9.560421271
3664,671,2011-04-27,1,3,0,27,32.6666679382324,0.333333343267441,60,3,2,206,73,1,1,1,0,1,10,1
3665,671,2011-04-27,2,3,0,24.2857151031494,24.7142848968506,4,53,3,2,206,73,1,1,1,0,1,10,117
3666,671,2011-04-27,3,3,0,32.6666679382324,26.6666660308838,0.666666686534882,60,3,2,206,
3667,671,2011-04-27,4,3,0,31,19.6000003814697,2.59999990463257,53.2000007629395,3,2,206,7
3668,671,2011-04-28,0,4,0,14,7,1,22,1,2,206,73,1,1,1,0,1,10,11744.4059,9715.281987,9.560421271
3669,671,2011-04-28,1,4,0,18,42,0,60,3,2,206,73,1,1,1,0,1,10,11744.4059,9715.281987,9.56042127
3670,671,2011-04-28,2,4,0,34.6666679382324,24.3333339691162,1,60,3,2,206,73,1,1,1,0,1,10,117
3671,671,2011-04-28,3,4,0,36.6666679382324,18.3333339691162,5,60,3,2,206,73,1,1,1,0,1,10,117
3672,671,2011-04-28,4,4,0,24.25,23.25,0.5,48,3,2,206,73,1,1,1,0,1,10,11744.4059,9715.281987,9.5
3673,671,2011-04-29,4,5,0,35,25,0,60,1,2,206,73,1,1,1,0,1,10,11744.4059,9715.281987,9.56042127
3674,673,2011-04-22,0,5,0,7.5,40.5,0.5,48.5,2,2,206,90,1,0,1,0,1,8,11130.38103,8849.234909,8.385
3675,673,2011-04-22,1,5,0,15.75,43.5,0.75,60,3,2,206,90,1,0,1,0,1,8,11130.38103,8849.234909,8.3
3676,673,2011-04-22,2,5,0,33,27,0,60,3,2,206,90,1,0,1,0,1,8,11130.38103,8849.234909,8.38527512
3677,673,2011-04-22,3,5,0,30.6666660308838,29.3333339691162,0,60,3,2,206,90,1,0,1,0,1,8,1113
3678,673,2011-04-22,4,5,0,30.3333339691162,22.3333339691162,0,52.6666679382324,3,2,206,90
3679,673,2011-04-23,0,6,1,13,32.5,0,45.5,2,2,206,90,1,0,1,0,1,8,11130.38103,8849.234909,8.38527
3680,673,2011-04-23,1,6,1,24.3333339691162,35,0.666666686534882,60,3,2,206,90,1,0,1,0,1,8,11
3681,673,2011-04-23,2,6,1,41,18.7999992370605,0.200000002980232,60,3,2,206,90,1,0,1,0,1,8,11
3682,673,2011-04-23,3,6,1,32.2000007629395,27.7999992370605,0,60,3,2,206,90,1,0,1,0,1,8,1113
3683,673,2011-04-23,4,6,1,29,30.5,0,59.5,2,2,206,90,1,0,1,0,1,8,11130.38103,8849.234909,8.38527
3684,673,2011-04-24,0,0,1,9,29,9,47,1,2,206,90,1,0,1,0,1,8,11130.38103,8849.234909,8.385275121
3685,673,2011-04-24,1,0,1,21.3333339691162,34,4.66666650772095,60,3,2,206,90,1,0,1,0,1,8,111
3686,673,2011-04-24,2,0,1,27.3333339691162,32.3333320617676,0.333333343267441,60,3,2,206,
3687,673,2011-04-24,3,0,1,27.25,32.5,0.25,60,3,2,206,90,1,0,1,0,1,8,11130.38103,8849.234909,8.3
3688,673,2011-04-24,4,0,1,28.3999996185303,24.6000003814697,0,53,3,2,206,90,1,0,1,0,1,8,1113
3689,673,2011-04-25,0,1,0,21.75,26.5,0.25,48.5,3,2,206,90,1,0,1,0,1,8,11130.38103,8849.234909,8
3690,673,2011-04-25,1,1,0,26.3333339691162,33.6666679382324,0,60,3,2,206,90,1,0,1,0,1,8,1113
3691,673,2011-04-25,2,1,0,40,20,0,60,3,2,206,90,1,0,1,0,1,8,11130.38103,8849.234909,8.38527512
3692,673,2011-04-25,3,1,0,42.3333320617676,17.6666660308838,0,60,3,2,206,90,1,0,1,0,1,8,1113
3693,673,2011-04-25,4,1,0,33.5,17,0,50.5,3,2,206,90,1,0,1,0,1,8,11130.38103,8849.234909,8.38527
3694,673,2011-04-26,1,2,0,26.3333339691162,33.6666679382324,0,60,3,2,206,90,1,0,1,0,1,8,1113
3695,673,2011-04-26,2,2,0,40.6666679382324,19.3333339691162,0,60,3,2,206,90,1,0,1,0,1,8,1113
3696,673,2011-04-26,3,2,0,24.6666660308838,35.3333320617676,0,60,3,2,206,90,1,0,1,0,1,8,1113
3697,673,2011-04-26,4,2,0,36.6666679382324,16.6666660308838,0.333333343267441,53.6666679
3698,673,2011-04-29,0,5,0,19.3333339691162,34.6666679382324,2.33333325386047,56.3333320
3699,673,2011-04-29,1,5,0,15.1999998092651,43,1.79999995231628,60,3,2,206,90,1,0,1,0,1,8,111

3700,673,2011-04-29,2,5,0,40.6666679382324,19.3333339691162,0,60,3,2,206,90,1,0,1,0,1,8,1113
3701,673,2011-04-29,3,5,0,26,34,0,60,3,2,206,90,1,0,1,0,1,8,11130.38103,8849.234909,8.3852751
3702,673,2011-04-29,4,5,0,34,10.6666669845581,0,44.6666679382324,3,2,206,90,1,0,1,0,1,8,1113
3703,674,2011-04-22,0,5,0,9.5,37.5,8,55,2,2,206,66,0,1,0,1,1,12,11356.30923,9081.389159,9.2948
3704,674,2011-04-22,1,5,0,11,32.3333320617676,16.6666660308838,60,3,2,206,66,0,1,0,1,1,12,11
3705,674,2011-04-22,2,5,0,11,34.3333320617676,14.6666669845581,60,3,2,206,66,0,1,0,1,1,12,11
3706,674,2011-04-22,3,5,0,16,35.7999992370605,8.19999980926514,60,3,2,206,66,0,1,0,1,1,12,11
3707,674,2011-04-22,4,5,0,27.6666660308838,28,4.33333349227905,60,3,2,206,66,0,1,0,1,1,12,11
3708,674,2011-04-23,0,6,1,6,33,2.5,41.5,2,2,206,66,0,1,0,1,1,12,11356.30923,9081.389159,9.2948
3709,674,2011-04-23,1,6,1,7.66666650772095,39.6666679382324,12.6666669845581,60,3,2,206,6
3710,674,2011-04-23,2,6,1,12.6666669845581,40.6666679382324,6.66666650772095,60,3,2,206,6
3711,674,2011-04-23,3,6,1,15.25,37,7.75,60,3,2,206,66,0,1,0,1,1,12,11356.30923,9081.389159,9.2
3712,674,2011-04-23,4,6,1,33,24.7999992370605,2.20000004768372,60,3,2,206,66,0,1,0,1,1,12,11
3713,674,2011-04-24,4,0,1,35,21,4,60,1,2,206,66,0,1,0,1,1,12,11356.30923,9081.389159,9.294888
3714,674,2011-04-26,0,2,0,22,32,6,60,1,2,206,66,0,1,0,1,1,12,11356.30923,9081.389159,9.294888
3715,674,2011-04-26,1,2,0,8,45.6666679382324,6.33333349227905,60,3,2,206,66,0,1,0,1,1,12,113
3716,674,2011-04-26,2,2,0,27,30,3,60,1,2,206,66,0,1,0,1,1,12,11356.30923,9081.389159,9.294888
3717,674,2011-04-27,0,3,0,9.5,34,1.5,45,2,2,206,66,0,1,0,1,1,12,11356.30923,9081.389159,9.2948
3718,674,2011-04-27,1,3,0,5.33333349227905,46,8.66666698455811,60,3,2,206,66,0,1,0,1,1,12,11
3719,674,2011-04-27,2,3,0,18.6000003814697,31.6000003814697,9.80000019073486,60,3,2,206,6
3720,674,2011-04-27,3,3,0,16.5,34.6666679382324,8.83333301544189,60,3,2,206,66,0,1,0,1,1,12,1
3721,674,2011-04-27,4,3,0,21.3333339691162,31.5,7.16666650772095,60,3,2,206,66,0,1,0,1,1,12,1
3722,674,2011-04-28,0,4,0,7.33333349227905,27.6666660308838,3.66666674613953,38.666667
3723,674,2011-04-28,1,4,0,9.80000019073486,39.5999984741211,10.6000003814697,60,3,2,206,6
3724,674,2011-04-28,2,4,0,18.3333339691162,35.6666679382324,6,60,3,2,206,66,0,1,0,1,1,12,113
3725,674,2011-04-28,3,4,0,19,34.3333320617676,6.66666650772095,60,3,2,206,66,0,1,0,1,1,12,11
3726,674,2011-04-28,4,4,0,11,41.6666679382324,7.33333349227905,60,3,2,206,66,0,1,0,1,1,12,11
3727,674,2011-04-29,0,5,0,11,28.5,4,43.5,2,2,206,66,0,1,0,1,1,12,11356.30923,9081.389159,9.294
3728,674,2011-04-29,1,5,0,16.75,35.75,7.5,60,3,2,206,66,0,1,0,1,1,12,11356.30923,9081.389159,9.
3729,674,2011-04-29,2,5,0,21.3333339691162,32.1666679382324,6.5,60,3,2,206,66,0,1,0,1,1,12,11
3730,674,2011-04-29,3,5,0,31.3333339691162,21,7.66666650772095,60,3,2,206,66,0,1,0,1,1,12,11
3731,674,2011-04-29,4,5,0,27.6666660308838,28,4.33333349227905,60,3,2,206,66,0,1,0,1,1,12,11
3732,675,2011-04-24,0,0,1,0,1,0,1,1,2,206,65,1,1,0,0,1,9,11573.04335,9045.961934,10.18516122,1
3733,675,2011-04-24,1,0,1,21.3999996185303,31,4.40000009536743,56.7999992370605,3,2,206,6
3734,675,2011-04-24,2,0,1,34.6666679382324,22.6666660308838,2.66666674613953,60,3,2,206,6
3735,675,2011-04-24,3,0,1,43.6666679382324,16,0.333333343267441,60,3,2,206,65,1,1,0,0,1,9,11
3736,675,2011-04-24,4,0,1,29,29.6666660308838,1.33333337306976,60,3,2,206,65,1,1,0,0,1,9,115
3737,675,2011-04-25,0,1,0,2,5,2,9,1,2,206,65,1,1,0,0,1,9,11573.04335,9045.961934,10.18516122,1
3738,675,2011-04-25,1,1,0,23.25,32.5,4.25,60,3,2,206,65,1,1,0,0,1,9,11573.04335,9045.961934,10.
3739,675,2011-04-25,2,1,0,25.1666660308838,31.6666660308838,3.16666674613953,60,3,2,206,6
3740,675,2011-04-25,3,1,0,36,21.6666660308838,2.33333325386047,60,3,2,206,65,1,1,0,0,1,9,115
3741,675,2011-04-25,4,1,0,31.3333339691162,26.6666660308838,2,60,3,2,206,65,1,1,0,0,1,9,1157
3742,675,2011-04-26,0,2,0,42,18,0,60,1,2,206,65,1,1,0,0,1,9,11573.04335,9045.961934,10.185161
3743,675,2011-04-26,1,2,0,35.3333320617676,19,5.66666650772095,60,3,2,206,65,1,1,0,0,1,9,115
3744,675,2011-04-26,2,2,0,32.3333320617676,27.3333339691162,0.333333343267441,60,3,2,206,
3745,675,2011-04-26,3,2,0,30.5,25.1666660308838,4.33333349227905,60,3,2,206,65,1,1,0,0,1,9,11
3746,675,2011-04-26,4,2,0,31,24.75,4.25,60,3,2,206,65,1,1,0,0,1,9,11573.04335,9045.961934,10.1
3747,675,2011-04-27,0,3,0,13,30.25,3.75,47,3,2,206,65,1,1,0,0,1,9,11573.04335,9045.961934,10.1
3748,675,2011-04-27,1,3,0,30.5,26.5,3,60,3,2,206,65,1,1,0,0,1,9,11573.04335,9045.961934,10.1851
3749,675,2011-04-27,2,3,0,30.5,27.5,2,60,3,2,206,65,1,1,0,0,1,9,11573.04335,9045.961934,10.1851

3750,675,2011-04-27,3,3,0,22.3333339691162,32,5.66666650772095,60,3,2,206,65,1,1,0,0,1,9,115
3751,675,2011-04-27,4,3,0,33,19,8,60,3,2,206,65,1,1,0,0,1,9,11573.04335,9045.961934,10.1851612
3752,675,2011-04-28,0,4,0,7,29,3,39,1,2,206,65,1,1,0,0,1,9,11573.04335,9045.961934,10.18516122
3753,675,2011-04-28,1,4,0,30,25.6666660308838,4.33333349227905,60,3,2,206,65,1,1,0,0,1,9,115
3754,675,2011-04-28,2,4,0,34.4000015258789,22.7999992370605,2.79999995231628,60,3,2,206,6
3755,675,2011-04-28,3,4,0,31.3999996185303,22.3999996185303,6.19999980926514,60,3,2,206,6
3756,675,2011-04-28,4,4,0,21.3333339691162,31,7.66666650772095,60,3,2,206,65,1,1,0,0,1,9,115
3757,675,2011-04-29,0,5,0,0,3,1,4,1,2,206,65,1,1,0,0,1,9,11573.04335,9045.961934,10.18516122,1
3758,675,2011-04-29,1,5,0,20.6666660308838,36,3.33333325386047,60,3,2,206,65,1,1,0,0,1,9,115
3759,675,2011-04-29,2,5,0,31,23,6,60,3,2,206,65,1,1,0,0,1,9,11573.04335,9045.961934,10.1851612
3760,675,2011-04-29,3,5,0,34.25,17,8.75,60,3,2,206,65,1,1,0,0,1,9,11573.04335,9045.961934,10.1
3761,675,2011-04-29,4,5,0,32,24.8333339691162,3.16666674613953,60,3,2,206,65,1,1,0,0,1,9,115
3762,680,2011-04-22,0,5,0,7,44,1,52,1,2,206,79,1,1,0,0,1,8,9966.128766,7792.199074,10.8596907
3763,680,2011-04-22,1,5,0,24.5,33.6666679382324,1.83333337306976,60,3,2,206,79,1,1,0,0,1,8,9
3764,680,2011-04-22,2,5,0,19.3333339691162,40.6666679382324,0,60,3,2,206,79,1,1,0,0,1,8,9966
3765,680,2011-04-22,3,5,0,18.6666660308838,41.3333320617676,0,60,3,2,206,79,1,1,0,0,1,8,9966
3766,680,2011-04-22,4,5,0,26.3333339691162,33.6666679382324,0,60,3,2,206,79,1,1,0,0,1,8,9966
3767,680,2011-04-23,0,6,1,1,19,2,22,1,2,206,79,1,1,0,0,1,8,9966.128766,7792.199074,10.8596907
3768,680,2011-04-23,1,6,1,24.6666660308838,33.3333320617676,2,60,3,2,206,79,1,1,0,0,1,8,9966
3769,680,2011-04-23,2,6,1,36.3333320617676,23.1666660308838,0.5,60,3,2,206,79,1,1,0,0,1,8,996
3770,680,2011-04-23,3,6,1,31.25,28,0.75,60,3,2,206,79,1,1,0,0,1,8,9966.128766,7792.199074,10.8
3771,680,2011-04-23,4,6,1,33,27,0,60,3,2,206,79,1,1,0,0,1,8,9966.128766,7792.199074,10.859690
3772,680,2011-04-24,0,0,1,40,0,0,40,1,2,206,79,1,1,0,0,1,8,9966.128766,7792.199074,10.8596907
3773,680,2011-04-24,1,0,1,26.6666660308838,32.3333320617676,1,60,3,2,206,79,1,1,0,0,1,8,9966
3774,680,2011-04-24,2,0,1,22.3333339691162,37.6666679382324,0,60,3,2,206,79,1,1,0,0,1,8,9966
3775,680,2011-04-24,3,0,1,27,33,0,60,3,2,206,79,1,1,0,0,1,8,9966.128766,7792.199074,10.859690
3776,680,2011-04-24,4,0,1,29.25,30.5,0.25,60,3,2,206,79,1,1,0,0,1,8,9966.128766,7792.199074,10.
3777,680,2011-04-25,0,1,0,49,1,0,50,1,2,206,79,1,1,0,0,1,8,9966.128766,7792.199074,10.8596907
3778,680,2011-04-25,1,1,0,9.80000019073486,41.7999992370605,0.200000002980232,51.799999
3779,680,2011-04-25,2,1,0,12,47,1,60,3,2,206,79,1,1,0,0,1,8,9966.128766,7792.199074,10.859690
3780,680,2011-04-25,3,1,0,30.3333339691162,28.3333339691162,1.33333337306976,60,3,2,206,7
3781,680,2011-04-25,4,1,0,23.3999996185303,26.3999996185303,0.400000005960464,50.200000
3782,680,2011-04-26,1,2,0,19.6666660308838,37,0.333333343267441,57,3,2,206,79,1,1,0,0,1,8,99
3783,680,2011-04-26,2,2,0,17.3333339691162,42.5,0.16666667163372,60,3,2,206,79,1,1,0,0,1,8,9
3784,680,2011-04-26,3,2,0,23.6000003814697,36.2000007629395,0.200000002980232,60,3,2,206,
3785,680,2011-04-26,4,2,0,48.6666679382324,11.3333330154419,0,60,3,2,206,79,1,1,0,0,1,8,9966
3786,680,2011-04-27,3,3,0,31,29,0,60,1,2,206,79,1,1,0,0,1,8,9966.128766,7792.199074,10.859690
3787,680,2011-04-27,4,3,0,24,35.6666679382324,0.333333343267441,60,3,2,206,79,1,1,0,0,1,8,99
3788,680,2011-04-29,0,5,0,26,32,2,60,1,2,206,79,1,1,0,0,1,8,9966.128766,7792.199074,10.859690
3789,686,2011-04-22,0,5,0,6,32,0,38,1,2,206,70,1,1,1,0,1,10,9872.508308,7702.139997,10.705306
3790,686,2011-04-22,1,5,0,38,21.6666660308838,0.333333343267441,60,3,2,206,70,1,1,1,0,1,10,9
3791,686,2011-04-22,2,5,0,22,35.3333320617676,2.66666674613953,60,3,2,206,70,1,1,1,0,1,10,98
3792,686,2011-04-22,3,5,0,35.2000007629395,24.3999996185303,0.400000005960464,60,3,2,206,
3793,686,2011-04-22,4,5,0,38.75,21,0.25,60,3,2,206,70,1,1,1,0,1,10,9872.508308,7702.139997,10.
3794,686,2011-04-23,0,6,1,10.3333330154419,20,0.666666686534882,31,3,2,206,70,1,1,1,0,1,10,9
3795,686,2011-04-23,1,6,1,5.66666650772095,49.6666679382324,4.66666650772095,60,3,2,206,7
3796,686,2011-04-23,2,6,1,40.6666679382324,18,1.33333337306976,60,3,2,206,70,1,1,1,0,1,10,98
3797,686,2011-04-23,3,6,1,28.6666660308838,27,4.33333349227905,60,3,2,206,70,1,1,1,0,1,10,98
3798,686,2011-04-23,4,6,1,46.4000015258789,13.3999996185303,0.200000002980232,60,3,2,206,
3799,686,2011-04-24,0,0,1,40.5,10.75,0.25,51.5,3,2,206,70,1,1,1,0,1,10,9872.508308,7702.139997,

3800,686,2011-04-24,1,0,1,39.6666679382324,20.3333339691162,0,60,3,2,206,70,1,1,1,0,1,10,987
3801,686,2011-04-24,2,0,1,37.3333320617676,20.3333339691162,2.33333325386047,60,3,2,206,7
3802,686,2011-04-24,3,0,1,20.3333339691162,38.3333320617676,1.33333337306976,60,3,2,206,7
3803,686,2011-04-24,4,0,1,41.3333320617676,18.6666660308838,0,60,3,2,206,70,1,1,1,0,1,10,987
3804,686,2011-04-25,0,1,0,38.3333320617676,20,1,59.3333320617676,3,2,206,70,1,1,1,0,1,10,987
3805,686,2011-04-25,1,1,0,33.4000015258789,25.7999992370605,0.800000011920929,60,3,2,206,
3806,686,2011-04-25,2,1,0,43.3333320617676,16.3333339691162,0.333333343267441,60,3,2,206,
3807,686,2011-04-25,3,1,0,41.6666679382324,17.3333339691162,1,60,3,2,206,70,1,1,1,0,1,10,987
3808,686,2011-04-25,4,1,0,33,26.6666660308838,0.333333343267441,60,3,2,206,70,1,1,1,0,1,10,9
3809,686,2011-04-26,0,2,0,20,24.5,0,44.5,2,2,206,70,1,1,1,0,1,10,9872.508308,7702.139997,10.705
3810,686,2011-04-26,1,2,0,37.75,19.75,2.5,60,3,2,206,70,1,1,1,0,1,10,9872.508308,7702.139997,10
3811,686,2011-04-26,2,2,0,31.3999996185303,27,1.60000002384186,60,3,2,206,70,1,1,1,0,1,10,98
3812,686,2011-04-26,3,2,0,27.6666660308838,31.3333339691162,1,60,3,2,206,70,1,1,1,0,1,10,987
3813,686,2011-04-26,4,2,0,47,13,0,60,3,2,206,70,1,1,1,0,1,10,9872.508308,7702.139997,10.70530
3814,686,2011-04-27,0,3,0,21.1666660308838,27,2.66666674613953,50.8333320617676,3,2,206,7
3815,686,2011-04-27,1,3,0,31.125,22.5,6.375,60,3,2,206,70,1,1,1,0,1,10,9872.508308,7702.139997
3816,686,2011-04-27,2,3,0,37.7142868041992,21.2857151031494,1,60,3,2,206,70,1,1,1,0,1,10,987
3817,686,2011-04-27,3,3,0,28.7777786254883,26.7777786254883,4.44444465637207,60,3,2,206,7
3818,686,2011-04-27,4,3,0,33.3333320617676,15.5,1.16666662693024,50,3,2,206,70,1,1,1,0,1,10,
3819,686,2011-04-28,0,4,0,21,35.6666679382324,3.33333325386047,60,3,2,206,70,1,1,1,0,1,10,98
3820,686,2011-04-28,1,4,0,15,40,5,60,3,2,206,70,1,1,1,0,1,10,9872.508308,7702.139997,10.70530
3821,686,2011-04-28,2,4,0,40,20,0,60,3,2,206,70,1,1,1,0,1,10,9872.508308,7702.139997,10.70530
3822,686,2011-04-28,3,4,0,19.6666660308838,21.3333339691162,19,60,3,2,206,70,1,1,1,0,1,10,98
3823,686,2011-04-28,4,4,0,46,14,0,60,3,2,206,70,1,1,1,0,1,10,9872.508308,7702.139997,10.70530
3824,686,2011-04-29,0,5,0,31,17.6666660308838,0.333333343267441,49,3,2,206,70,1,1,1,0,1,10,9
3825,686,2011-04-29,1,5,0,26.6666660308838,23.6666660308838,9.66666698455811,60,3,2,206,7
3826,686,2011-04-29,2,5,0,30,23.2000007629395,6.80000019073486,60,3,2,206,70,1,1,1,0,1,10,98
3827,686,2011-04-29,3,5,0,35,24.5,0.5,60,3,2,206,70,1,1,1,0,1,10,9872.508308,7702.139997,10.705
3828,686,2011-04-29,4,5,0,42,17.3333339691162,0.666666686534882,60,3,2,206,70,1,1,1,0,1,10,9
3829,688,2011-04-22,0,5,0,8,23.5,19.5,51,2,2,206,65,0,1,0,1,1,12,9444.103282,5540.207145,11.81
3830,688,2011-04-22,1,5,0,34,13.6666669845581,12.3333330154419,60,3,2,206,65,0,1,0,1,1,12,94
3831,688,2011-04-22,2,5,0,32.3333320617676,27,0.666666686534882,60,3,2,206,65,0,1,0,1,1,12,9
3832,688,2011-04-22,3,5,0,16,43,1,60,3,2,206,65,0,1,0,1,1,12,9444.103282,5540.207145,11.81245
3833,688,2011-04-22,4,5,0,28,31.3999996185303,0.600000023841858,60,3,2,206,65,0,1,0,1,1,12,9
3834,688,2011-04-23,0,6,1,20.75,21.5,1.25,43.5,3,2,206,65,0,1,0,1,1,12,9444.103282,5540.207145,
3835,688,2011-04-23,1,6,1,32.25,24,3.75,60,3,2,206,65,0,1,0,1,1,12,9444.103282,5540.207145,11.
3836,688,2011-04-23,2,6,1,14.6666669845581,30,15.3333330154419,60,3,2,206,65,0,1,0,1,1,12,94
3837,688,2011-04-23,3,6,1,25.6666660308838,31.6666660308838,2.66666674613953,60,3,2,206,6
3838,688,2011-04-23,4,6,1,25.3333339691162,32.3333320617676,2.33333325386047,60,3,2,206,6
3839,688,2011-04-24,0,0,1,21,13,7.5,41.5,2,2,206,65,0,1,0,1,1,12,9444.103282,5540.207145,11.81
3840,688,2011-04-24,1,0,1,25.6000003814697,15.1999998092651,19.2000007629395,60,3,2,206,6
3841,688,2011-04-24,2,0,1,26,31.3333339691162,2.66666674613953,60,3,2,206,65,0,1,0,1,1,12,94
3842,688,2011-04-24,3,0,1,30,25.3333339691162,4.66666650772095,60,3,2,206,65,0,1,0,1,1,12,94
3843,688,2011-04-24,4,0,1,33.3333320617676,22,4.66666650772095,60,3,2,206,65,0,1,0,1,1,12,94
3844,688,2011-04-25,0,1,0,27,18,2.5,47.5,2,2,206,65,0,1,0,1,1,12,9444.103282,5540.207145,11.81
3845,688,2011-04-25,1,1,0,12.3333330154419,39,8.66666698455811,60,3,2,206,65,0,1,0,1,1,12,94
3846,688,2011-04-25,2,1,0,21.1666660308838,23.6666660308838,15.1666669845581,60,3,2,206,6
3847,688,2011-04-25,3,1,0,29.3333339691162,15.3333330154419,15.3333330154419,60,3,2,206,6
3848,688,2011-04-25,4,1,0,37.6666679382324,21.6666660308838,0.666666686534882,60,3,2,206,
3849,688,2011-04-26,0,2,0,28,30.5,0,58.5,2,2,206,65,0,1,0,1,1,12,9444.103282,5540.207145,11.81

3850,688,2011-04-26,1,2,0,4.33333349227905,23.6666660308838,32,60,3,2,206,65,0,1,0,1,1,12,94
3851,688,2011-04-26,2,2,0,16,26.6666660308838,17.3333339691162,60,3,2,206,65,0,1,0,1,1,12,94
3852,688,2011-04-26,3,2,0,12.6666669845581,31.3333339691162,16,60,3,2,206,65,0,1,0,1,1,12,94
3853,688,2011-04-26,4,2,0,21,36.6666679382324,2.33333325386047,60,3,2,206,65,0,1,0,1,1,12,94
3854,688,2011-04-27,0,3,0,11,15,19.6666660308838,45.6666679382324,3,2,206,65,0,1,0,1,1,12,94
3855,688,2011-04-27,1,3,0,23.3333339691162,28.6666660308838,8,60,3,2,206,65,0,1,0,1,1,12,944
3856,688,2011-04-27,2,3,0,23,25.3333339691162,11.6666669845581,60,3,2,206,65,0,1,0,1,1,12,94
3857,688,2011-04-27,3,3,0,15.6666669845581,36,8.33333301544189,60,3,2,206,65,0,1,0,1,1,12,94
3858,688,2011-04-27,4,3,0,28.1666660308838,29.6666660308838,2.16666674613953,60,3,2,206,6
3859,688,2011-04-28,0,4,0,29,19,1.5,49.5,2,2,206,65,0,1,0,1,1,12,9444.103282,5540.207145,11.812
3860,688,2011-04-28,1,4,0,10.6666669845581,42.6666679382324,6.66666650772095,60,3,2,206,6
3861,688,2011-04-28,2,4,0,29,29.5,1.5,60,3,2,206,65,0,1,0,1,1,12,9444.103282,5540.207145,11.812
3862,688,2011-04-28,3,4,0,34.6666679382324,24.3333339691162,1,60,3,2,206,65,0,1,0,1,1,12,944
3863,688,2011-04-28,4,4,0,30,29.6666660308838,0.333333343267441,60,3,2,206,65,0,1,0,1,1,12,9
3864,688,2011-04-29,0,5,0,36,12,0,48,2,2,206,65,0,1,0,1,1,12,9444.103282,5540.207145,11.81245
3865,688,2011-04-29,1,5,0,30.6666660308838,28.6666660308838,0.666666686534882,60,3,2,206,
3866,688,2011-04-29,2,5,0,37.6666679382324,20.6666660308838,1.66666662693024,60,3,2,206,6
3867,688,2011-04-29,3,5,0,33.6666679382324,19,7.33333349227905,60,3,2,206,65,0,1,0,1,1,12,94
3868,688,2011-04-29,4,5,0,32.75,26,1.25,60,3,2,206,65,0,1,0,1,1,12,9444.103282,5540.207145,11.
3869,689,2011-04-22,1,5,0,20,29.1666660308838,2.5,51.6666679382324,3,2,206,71,1,1,0,1,1,11,9
3870,689,2011-04-22,2,5,0,10.5,28.25,21.25,60,3,2,206,71,1,1,0,1,1,11,9523.924014,5736.686029,
3871,689,2011-04-22,3,5,0,12,20.6666660308838,27.3333339691162,60,3,2,206,71,1,1,0,1,1,11,95
3872,689,2011-04-22,4,5,0,44.3333320617676,15.6666669845581,0,60,3,2,206,71,1,1,0,1,1,11,952
3873,689,2011-04-23,0,6,1,18,20,3.5,41.5,2,2,206,71,1,1,0,1,1,11,9523.924014,5736.686029,12.33
3874,689,2011-04-23,1,6,1,5,43.6666679382324,11.3333330154419,60,3,2,206,71,1,1,0,1,1,11,952
3875,689,2011-04-23,2,6,1,13,24,23,60,3,2,206,71,1,1,0,1,1,11,9523.924014,5736.686029,12.3338
3876,689,2011-04-23,3,6,1,11.25,37.5,11.25,60,3,2,206,71,1,1,0,1,1,11,9523.924014,5736.686029,
3877,689,2011-04-23,4,6,1,39.6666679382324,20,0.333333343267441,60,3,2,206,71,1,1,0,1,1,11,9
3878,689,2011-04-24,1,0,1,5.33333349227905,37.3333320617676,5.66666650772095,48.333332
3879,689,2011-04-24,2,0,1,15.6666669845581,30.3333339691162,14,60,3,2,206,71,1,1,0,1,1,11,95
3880,689,2011-04-24,3,0,1,14.8000001907349,35.5999984741211,9.60000038146973,60,3,2,206,7
3881,689,2011-04-24,4,0,1,31.3999996185303,27.3999996185303,1.20000004768372,60,3,2,206,7
3882,689,2011-04-25,4,1,0,42,18,0,60,1,2,206,71,1,1,0,1,1,11,9523.924014,5736.686029,12.33389
3883,689,2011-04-27,0,3,0,60,0,0,60,1,2,206,71,1,1,0,1,1,11,9523.924014,5736.686029,12.333897
3884,689,2011-04-27,1,3,0,24,14,22,60,3,2,206,71,1,1,0,1,1,11,9523.924014,5736.686029,12.3338
3885,689,2011-04-27,2,3,0,17,22,21,60,1,2,206,71,1,1,0,1,1,11,9523.924014,5736.686029,12.3338
3886,689,2011-04-28,1,4,0,16.6666660308838,34.6666679382324,1.66666662693024,53,3,2,206,7
3887,689,2011-04-28,2,4,0,29,25.7999992370605,5.19999980926514,60,3,2,206,71,1,1,0,1,1,11,95
3888,689,2011-04-28,3,4,0,23.1666660308838,31,5.83333349227905,60,3,2,206,71,1,1,0,1,1,11,95
3889,689,2011-04-28,4,4,0,31.6666660308838,23.3333339691162,5,60,3,2,206,71,1,1,0,1,1,11,952
3890,689,2011-04-29,0,5,0,8.33333301544189,25.6666660308838,13,47,3,2,206,71,1,1,0,1,1,11,95
3891,689,2011-04-29,1,5,0,4,32.6666679382324,23.3333339691162,60,3,2,206,71,1,1,0,1,1,11,952
3892,689,2011-04-29,2,5,0,1.33333337306976,15.6666669845581,43,60,3,2,206,71,1,1,0,1,1,11,95
3893,689,2011-04-29,3,5,0,7.33333349227905,32.6666679382324,20,60,3,2,206,71,1,1,0,1,1,11,95
3894,689,2011-04-29,4,5,0,35.6666679382324,24,0.333333343267441,60,3,2,206,71,1,1,0,1,1,11,9
3895,691,2011-04-25,0,1,0,18,0,0,18,1,2,206,80,1,1,1,0,1,4,11308.42662,8628.066712,10.2442484,
3896,691,2011-04-25,1,1,0,20,4.66666650772095,0,24.6666660308838,3,2,206,80,1,1,1,0,1,4,1130
3897,691,2011-04-25,2,1,0,22.6666660308838,33.3333320617676,0.333333343267441,56.33333
3898,691,2011-04-25,3,1,0,27,33,0,60,3,2,206,80,1,1,1,0,1,4,11308.42662,8628.066712,10.244248
3899,691,2011-04-25,4,1,0,36.3333320617676,23.6666660308838,0,60,3,2,206,80,1,1,1,0,1,4,1130

3900,691,2011-04-26,0,2,0,31,4,0,35,1,2,206,80,1,1,1,0,1,4,11308.42662,8628.066712,10.2442484,
3901,691,2011-04-26,1,2,0,34.3333320617676,25.6666660308838,0,60,3,2,206,80,1,1,1,0,1,4,1130
3902,691,2011-04-26,2,2,0,23.3333339691162,36,0.666666686534882,60,3,2,206,80,1,1,1,0,1,4,11
3903,691,2011-04-26,3,2,0,33,27,0,60,3,2,206,80,1,1,1,0,1,4,11308.42662,8628.066712,10.244248
3904,691,2011-04-26,4,2,0,37.5,22.5,0,60,3,2,206,80,1,1,1,0,1,4,11308.42662,8628.066712,10.244
3905,691,2011-04-27,1,3,0,11.6666669845581,23.3333339691162,0.333333343267441,35.333332
3906,691,2011-04-27,2,3,0,30.8333339691162,29.1666660308838,0,60,3,2,206,80,1,1,1,0,1,4,1130
3907,691,2011-04-27,3,3,0,24.5,35.3333320617676,0.16666667163372,60,3,2,206,80,1,1,1,0,1,4,1
3908,691,2011-04-27,4,3,0,25.8571434020996,34.1428565979004,0,60,3,2,206,80,1,1,1,0,1,4,1130
3909,691,2011-04-28,0,4,0,31,2,0,33,1,2,206,80,1,1,1,0,1,4,11308.42662,8628.066712,10.2442484,
3910,691,2011-04-28,1,4,0,46,13.3333330154419,0.666666686534882,60,3,2,206,80,1,1,1,0,1,4,11
3911,691,2011-04-28,2,4,0,40.6666679382324,19.3333339691162,0,60,3,2,206,80,1,1,1,0,1,4,1130
3912,691,2011-04-28,3,4,0,31.3333339691162,28.6666660308838,0,60,3,2,206,80,1,1,1,0,1,4,1130
3913,691,2011-04-28,4,4,0,20,28,0,48,3,2,206,80,1,1,1,0,1,4,11308.42662,8628.066712,10.244248
3914,693,2011-04-22,0,5,0,26,14,0.5,40.5,2,2,206,79,0,1,0,1,1,12,11315.10319,8466.706382,8.946
3915,693,2011-04-22,1,5,0,52.3333320617676,7.66666650772095,0,60,3,2,206,79,0,1,0,1,1,12,113
3916,693,2011-04-22,2,5,0,19,40,1,60,3,2,206,79,0,1,0,1,1,12,11315.10319,8466.706382,8.946095
3917,693,2011-04-22,3,5,0,38.5999984741211,14.3999996185303,7,60,3,2,206,79,0,1,0,1,1,12,113
3918,693,2011-04-22,4,5,0,52,8,0,60,3,2,206,79,0,1,0,1,1,12,11315.10319,8466.706382,8.9460952
3919,693,2011-04-23,0,6,1,17,17,1,35,1,2,206,79,0,1,0,1,1,12,11315.10319,8466.706382,8.946095
3920,693,2011-04-23,1,6,1,47.6666679382324,12.3333330154419,0,60,3,2,206,79,0,1,0,1,1,12,113
3921,693,2011-04-23,2,6,1,46.6666679382324,13.3333330154419,0,60,3,2,206,79,0,1,0,1,1,12,113
3922,693,2011-04-23,3,6,1,15,17.5,2.75,35.25,3,2,206,79,0,1,0,1,1,12,11315.10319,8466.706382,8.
3923,693,2011-04-23,4,6,1,35.5,13.5,11,60,3,2,206,79,0,1,0,1,1,12,11315.10319,8466.706382,8.94
3924,693,2011-04-24,0,0,1,22.25,23,4.25,49.5,3,2,206,79,0,1,0,1,1,12,11315.10319,8466.706382,8.
3925,693,2011-04-24,1,0,1,39.6666679382324,20.3333339691162,0,60,3,2,206,79,0,1,0,1,1,12,113
3926,693,2011-04-24,2,0,1,38,22,0,60,3,2,206,79,0,1,0,1,1,12,11315.10319,8466.706382,8.946095
3927,693,2011-04-24,3,0,1,43,16.3333339691162,0.666666686534882,60,3,2,206,79,0,1,0,1,1,12,1
3928,693,2011-04-24,4,0,1,50.3333320617676,9.66666698455811,0,60,3,2,206,79,0,1,0,1,1,12,113
3929,693,2011-04-25,0,1,0,34,20,1.33333337306976,55.3333320617676,3,2,206,79,0,1,0,1,1,12,11
3930,693,2011-04-25,1,1,0,36.3333320617676,19,4.66666650772095,60,3,2,206,79,0,1,0,1,1,12,11
3931,693,2011-04-25,2,1,0,44.3333320617676,6.33333349227905,0,50.6666679382324,3,2,206,79
3932,693,2011-04-25,3,1,0,30,11,10,51,2,2,206,79,0,1,0,1,1,12,11315.10319,8466.706382,8.94609
3933,693,2011-04-25,4,1,0,50.3333320617676,9.33333301544189,0.333333343267441,60,3,2,206,
3934,693,2011-04-26,2,2,0,44.6666679382324,15.3333330154419,0,60,3,2,206,79,0,1,0,1,1,12,113
3935,693,2011-04-26,3,2,0,46.3333320617676,13.6666669845581,0,60,3,2,206,79,0,1,0,1,1,12,113
3936,693,2011-04-26,4,2,0,34,25.6666660308838,0.333333343267441,60,3,2,206,79,0,1,0,1,1,12,1
3937,693,2011-04-28,0,4,0,31,29,0,60,1,2,206,79,0,1,0,1,1,12,11315.10319,8466.706382,8.946095
3938,693,2011-04-28,1,4,0,23.6666660308838,20.3333339691162,16,60,3,2,206,79,0,1,0,1,1,12,11
3939,693,2011-04-29,0,5,0,27,23.6666660308838,0,50.6666679382324,3,2,206,79,0,1,0,1,1,12,113
3940,693,2011-04-29,1,5,0,33.3333320617676,25.6666660308838,1,60,3,2,206,79,0,1,0,1,1,12,113
3941,693,2011-04-29,2,5,0,28.6666660308838,24.5,6.83333349227905,60,3,2,206,79,0,1,0,1,1,12,
3942,693,2011-04-29,3,5,0,44.3333320617676,11.3333330154419,4.33333349227905,60,3,2,206,7
3943,693,2011-04-29,4,5,0,43.3333320617676,16.3333339691162,0.333333343267441,60,3,2,206,
3944,696,2011-04-22,1,5,0,20.3333339691162,36.6666679382324,0,57,3,2,206,66,1,1,1,1,1,12,102
3945,696,2011-04-22,2,5,0,24.7999992370605,28.6000003814697,1,54.4000015258789,3,2,206,66
3946,696,2011-04-22,3,5,0,22.2000007629395,26.2000007629395,5,53.4000015258789,3,2,206,66
3947,696,2011-04-22,4,5,0,34.3333320617676,25.3333339691162,0.333333343267441,60,3,2,206,
3948,696,2011-04-23,0,6,1,0,8,0,8,1,2,206,66,1,1,1,1,1,12,10250.3735,5224.914772,12.24689595,4
3949,696,2011-04-23,1,6,1,14,32.75,5,51.75,3,2,206,66,1,1,1,1,1,12,10250.3735,5224.914772,12.2

3950,696,2011-04-23,2,6,1,18,38,4,60,3,2,206,66,1,1,1,1,1,12,10250.3735,5224.914772,12.2468959
3951,696,2011-04-23,3,6,1,23,36.3333320617676,0.666666686534882,60,3,2,206,66,1,1,1,1,1,12,1
3952,696,2011-04-23,4,6,1,26.6666660308838,31.5,0.16666667163372,58.3333320617676,3,2,206
3953,696,2011-04-24,1,0,1,25.25,29,0.25,54.5,3,2,206,66,1,1,1,1,1,12,10250.3735,5224.914772,12.
3954,696,2011-04-24,2,0,1,25.6666660308838,25,9.33333301544189,60,3,2,206,66,1,1,1,1,1,12,10
3955,696,2011-04-24,3,0,1,42.25,17.25,0.5,60,3,2,206,66,1,1,1,1,1,12,10250.3735,5224.914772,12.
3956,696,2011-04-24,4,0,1,14.5,41.5,3,59,2,2,206,66,1,1,1,1,1,12,10250.3735,5224.914772,12.2468
3957,696,2011-04-25,0,1,0,0,5,0,5,1,2,206,66,1,1,1,1,1,12,10250.3735,5224.914772,12.24689595,4
3958,696,2011-04-25,1,1,0,22.6666660308838,36.3333320617676,1,60,3,2,206,66,1,1,1,1,1,12,102
3959,696,2011-04-25,2,1,0,9.66666698455811,49.3333320617676,1,60,3,2,206,66,1,1,1,1,1,12,102
3960,696,2011-04-25,3,1,0,22,37.2000007629395,0.800000011920929,60,3,2,206,66,1,1,1,1,1,12,1
3961,696,2011-04-25,4,1,0,35,22,3,60,3,2,206,66,1,1,1,1,1,12,10250.3735,5224.914772,12.2468959
3962,696,2011-04-26,0,2,0,3,50,1,54,1,2,206,66,1,1,1,1,1,12,10250.3735,5224.914772,12.24689595
3963,696,2011-04-26,1,2,0,4.66666650772095,46.3333320617676,9,60,3,2,206,66,1,1,1,1,1,12,102
3964,696,2011-04-26,2,2,0,13.3333330154419,39.6666679382324,7,60,3,2,206,66,1,1,1,1,1,12,102
3965,696,2011-04-26,3,2,0,12,42,6,60,1,2,206,66,1,1,1,1,1,12,10250.3735,5224.914772,12.2468959
3966,696,2011-04-26,4,2,0,57,3,0,60,1,2,206,66,1,1,1,1,1,12,10250.3735,5224.914772,12.24689595
3967,696,2011-04-27,1,3,0,17.6666660308838,27.5,3.66666674613953,48.8333320617676,3,2,206
3968,696,2011-04-27,2,3,0,18.2000007629395,28,3.59999990463257,49.7999992370605,3,2,206,6
3969,696,2011-04-27,3,3,0,22.6000003814697,26.2000007629395,11.1999998092651,60,3,2,206,6
3970,696,2011-04-27,4,3,0,35.1666679382324,23,0.833333313465118,59,3,2,206,66,1,1,1,1,1,12,1
3971,696,2011-04-28,1,4,0,20,36,0.333333343267441,56.3333320617676,3,2,206,66,1,1,1,1,1,12,1
3972,696,2011-04-28,2,4,0,22,31,7,60,3,2,206,66,1,1,1,1,1,12,10250.3735,5224.914772,12.2468959
3973,696,2011-04-28,3,4,0,26.3333339691162,29,4.66666650772095,60,3,2,206,66,1,1,1,1,1,12,10
3974,696,2011-04-28,4,4,0,36,23.7999992370605,0.200000002980232,60,3,2,206,66,1,1,1,1,1,12,1
3975,696,2011-04-29,0,5,0,31.5,21,1,53.5,2,2,206,66,1,1,1,1,1,12,10250.3735,5224.914772,12.2468
3976,696,2011-04-29,1,5,0,14.1666669845581,43.6666679382324,2.16666674613953,60,3,2,206,6
3977,696,2011-04-29,2,5,0,17.25,39.5,3.25,60,3,2,206,66,1,1,1,1,1,12,10250.3735,5224.914772,12.
3978,696,2011-04-29,3,5,0,3,52.6666679382324,4.33333349227905,60,3,2,206,66,1,1,1,1,1,12,102
3979,696,2011-04-29,4,5,0,4.33333349227905,37,7,48.3333320617676,3,2,206,66,1,1,1,1,1,12,102
3980,703,2011-04-22,0,5,0,54,6,0,60,2,2,206,68,1,1,0,1,1,12,10396.67341,9785.089064,6.5435043
3981,703,2011-04-22,1,5,0,9,42.3333320617676,3.33333325386047,54.6666679382324,3,2,206,68
3982,703,2011-04-22,2,5,0,16.6666660308838,39.6666679382324,3.66666674613953,60,3,2,206,6
3983,703,2011-04-22,3,5,0,19,39.6666679382324,1.33333337306976,60,3,2,206,68,1,1,0,1,1,12,10
3984,703,2011-04-22,4,5,0,28.6000003814697,30.7999992370605,0.600000023841858,60,3,2,206,
3985,703,2011-04-23,0,6,1,31,28,1,60,1,2,206,68,1,1,0,1,1,12,10396.67341,9785.089064,6.5435043
3986,703,2011-04-23,1,6,1,25.3333339691162,32,2.66666674613953,60,3,2,206,68,1,1,0,1,1,12,10
3987,703,2011-04-23,2,6,1,14,43.3333320617676,2.66666674613953,60,3,2,206,68,1,1,0,1,1,12,10
3988,703,2011-04-23,3,6,1,31,25.6666660308838,3.33333325386047,60,3,2,206,68,1,1,0,1,1,12,10
3989,703,2011-04-23,4,6,1,30.3333339691162,29.6666660308838,0,60,3,2,206,68,1,1,0,1,1,12,103
3990,703,2011-04-26,0,2,0,50,8,2,60,1,2,206,68,1,1,0,1,1,12,10396.67341,9785.089064,6.5435043
3991,703,2011-04-26,1,2,0,14.1666669845581,42.3333320617676,2.33333325386047,58.8333320
3992,703,2011-04-26,2,2,0,12.3333330154419,44.3333320617676,3.33333325386047,60,3,2,206,6
3993,703,2011-04-26,3,2,0,34.6666679382324,24.6666660308838,0.666666686534882,60,3,2,206,
3994,703,2011-04-26,4,2,0,26,32.6666679382324,1.33333337306976,60,3,2,206,68,1,1,0,1,1,12,10
3995,703,2011-04-27,0,3,0,16,25,2,43,1,2,206,68,1,1,0,1,1,12,10396.67341,9785.089064,6.5435043
3996,703,2011-04-27,1,3,0,25.6666660308838,32.6666679382324,1.66666662693024,60,3,2,206,6
3997,703,2011-04-27,2,3,0,14.6666669845581,43.3333320617676,2,60,3,2,206,68,1,1,0,1,1,12,103
3998,703,2011-04-27,3,3,0,27.5,31.6666660308838,0.833333313465118,60,3,2,206,68,1,1,0,1,1,12
3999,703,2011-04-27,4,3,0,28.5,30.3333339691162,1.16666662693024,60,3,2,206,68,1,1,0,1,1,12,1

4000,703,2011-04-28,1,4,0,23.6666660308838,26.1666660308838,7,56.8333320617676,3,2,206,68
4001,703,2011-04-28,2,4,0,18.6000003814697,32.7999992370605,8.60000038146973,60,3,2,206,6
4002,703,2011-04-28,3,4,0,35,23.3333339691162,1.66666662693024,60,3,2,206,68,1,1,0,1,1,12,10
4003,703,2011-04-28,4,4,0,31.6666660308838,26.6666660308838,1.66666662693024,60,3,2,206,6
4004,703,2011-04-29,0,5,0,1,11,1,13,1,2,206,68,1,1,0,1,1,12,10396.67341,9785.089064,6.5435043
4005,703,2011-04-29,1,5,0,24,34,2,60,3,2,206,68,1,1,0,1,1,12,10396.67341,9785.089064,6.5435043
4006,703,2011-04-29,2,5,0,23.5,30.25,6.25,60,3,2,206,68,1,1,0,1,1,12,10396.67341,9785.089064,6.
4007,703,2011-04-29,3,5,0,25.1666660308838,24,10.8333330154419,60,3,2,206,68,1,1,0,1,1,12,10
4008,703,2011-04-29,4,5,0,13.75,42.5,3.75,60,3,2,206,68,1,1,0,1,1,12,10396.67341,9785.089064,6.
4009,705,2011-04-23,0,6,1,22,21,4,47,2,2,206,74,1,1,0,1,1,6,10965.62003,12899.68011,7.4323169
4010,705,2011-04-23,1,6,1,17.5,40,2.5,60,2,2,206,74,1,1,0,1,1,6,10965.62003,12899.68011,7.43231
4011,705,2011-04-24,0,0,1,16,26,0,42,1,2,206,74,1,1,0,1,1,6,10965.62003,12899.68011,7.4323169
4012,705,2011-04-24,1,0,1,22.25,37.75,0,60,3,2,206,74,1,1,0,1,1,6,10965.62003,12899.68011,7.432
4013,705,2011-04-24,2,0,1,32.2000007629395,27.7999992370605,0,60,3,2,206,74,1,1,0,1,1,6,1096
4014,705,2011-04-24,3,0,1,16.3333339691162,43.6666679382324,0,60,3,2,206,74,1,1,0,1,1,6,1096
4015,705,2011-04-24,4,0,1,29,31,0,60,3,2,206,74,1,1,0,1,1,6,10965.62003,12899.68011,7.4323169
4016,705,2011-04-25,0,1,0,11,29.3333339691162,1.66666662693024,42,3,2,206,74,1,1,0,1,1,6,109
4017,705,2011-04-25,1,1,0,14.6666669845581,43.6666679382324,1.66666662693024,60,3,2,206,7
4018,705,2011-04-25,2,1,0,37.5,22.25,0.25,60,3,2,206,74,1,1,0,1,1,6,10965.62003,12899.68011,7.4
4019,705,2011-04-25,3,1,0,27.75,32.25,0,60,3,2,206,74,1,1,0,1,1,6,10965.62003,12899.68011,7.432
4020,705,2011-04-25,4,1,0,22.6666660308838,37,0.333333343267441,60,3,2,206,74,1,1,0,1,1,6,10
4021,705,2011-04-26,0,2,0,27.6666660308838,13,0,40.6666679382324,3,2,206,74,1,1,0,1,1,6,1096
4022,705,2011-04-26,1,2,0,30.6666660308838,28.3333339691162,1,60,3,2,206,74,1,1,0,1,1,6,1096
4023,705,2011-04-26,2,2,0,41.6666679382324,17.3333339691162,1,60,3,2,206,74,1,1,0,1,1,6,1096
4024,705,2011-04-26,3,2,0,26.6000003814697,33.4000015258789,0,60,3,2,206,74,1,1,0,1,1,6,1096
4025,705,2011-04-26,4,2,0,21.6666660308838,35,0.666666686534882,57.3333320617676,3,2,206,
4026,705,2011-04-27,0,3,0,13,40,0.5,53.5,3,2,206,74,1,1,0,1,1,6,10965.62003,12899.68011,7.43231
4027,705,2011-04-27,1,3,0,21.1666660308838,38.1666679382324,0.666666686534882,60,3,2,206,
4028,705,2011-04-27,2,3,0,33.25,26.75,0,60,3,2,206,74,1,1,0,1,1,6,10965.62003,12899.68011,7.432
4029,705,2011-04-27,3,3,0,43,17,0,60,3,2,206,74,1,1,0,1,1,6,10965.62003,12899.68011,7.4323169
4030,705,2011-04-27,4,3,0,38.7999992370605,21,0.200000002980232,60,3,2,206,74,1,1,0,1,1,6,10
4031,705,2011-04-28,0,4,0,18,30.5,1,49.5,2,2,206,74,1,1,0,1,1,6,10965.62003,12899.68011,7.43231
4032,705,2011-04-28,1,4,0,24.3333339691162,35.6666679382324,0,60,3,2,206,74,1,1,0,1,1,6,1096
4033,705,2011-04-28,2,4,0,30.3999996185303,29.3999996185303,0.200000002980232,60,3,2,206,
4034,705,2011-04-28,3,4,0,40.5,19.25,0.25,60,3,2,206,74,1,1,0,1,1,6,10965.62003,12899.68011,7.4
4035,705,2011-04-28,4,4,0,34.3333320617676,25.6666660308838,0,60,3,2,206,74,1,1,0,1,1,6,1096
4036,705,2011-04-29,3,5,0,25.5,34,0.5,60,2,2,206,74,1,1,0,1,1,6,10965.62003,12899.68011,7.43231
4037,705,2011-04-29,4,5,0,42,18,0,60,3,2,206,74,1,1,0,1,1,6,10965.62003,12899.68011,7.4323169
4038,706,2011-04-22,1,5,0,18.6666660308838,26,1.33333337306976,46,3,2,206,73,1,1,1,1,1,12,10
4039,706,2011-04-22,2,5,0,16.1666660308838,27.6666660308838,16.1666660308838,60,3,2,206,7
4040,706,2011-04-22,3,5,0,13.25,34,12.75,60,3,2,206,73,1,1,1,1,1,12,10931.26621,8228.046922,10
4041,706,2011-04-22,4,5,0,34.6666679382324,24.3333339691162,1,60,3,2,206,73,1,1,1,1,1,12,109
4042,706,2011-04-23,0,6,1,60,0,0,60,1,2,206,73,1,1,1,1,1,12,10931.26621,8228.046922,10.253738
4043,706,2011-04-23,1,6,1,30,21,1,52,2,2,206,73,1,1,1,1,1,12,10931.26621,8228.046922,10.253738
4044,706,2011-04-23,2,6,1,19.6666660308838,38.3333320617676,2,60,3,2,206,73,1,1,1,1,1,12,109
4045,706,2011-04-23,3,6,1,31,25,4,60,3,2,206,73,1,1,1,1,1,12,10931.26621,8228.046922,10.253738
4046,706,2011-04-23,4,6,1,19,36.6666679382324,4.33333349227905,60,3,2,206,73,1,1,1,1,1,12,10
4047,706,2011-04-24,0,0,1,12,39,1,52,1,2,206,73,1,1,1,1,1,12,10931.26621,8228.046922,10.253738
4048,706,2011-04-24,1,0,1,20.1666660308838,32.3333320617676,7.5,60,3,2,206,73,1,1,1,1,1,12,10
4049,706,2011-04-24,2,0,1,44,15.6666669845581,0.333333343267441,60,3,2,206,73,1,1,1,1,1,12,1

4050,706,2011-04-24,3,0,1,17,33.3333320617676,9.66666698455811,60,3,2,206,73,1,1,1,1,1,12,10
4051,706,2011-04-24,4,0,1,19.3333339691162,37.6666679382324,3,60,3,2,206,73,1,1,1,1,1,12,109
4052,706,2011-04-25,1,1,0,12.6666669845581,42,0.666666686534882,55.3333320617676,3,2,206,
4053,706,2011-04-25,2,1,0,10.5,41.8333320617676,7.66666650772095,60,3,2,206,73,1,1,1,1,1,12,1
4054,706,2011-04-25,3,1,0,17.7999992370605,38,4.19999980926514,60,3,2,206,73,1,1,1,1,1,12,10
4055,706,2011-04-25,4,1,0,22.6666660308838,36.6666679382324,0.666666686534882,60,3,2,206,
4056,706,2011-04-26,1,2,0,7.5,24.5,0.5,32.5,2,2,206,73,1,1,1,1,1,12,10931.26621,8228.046922,10.2
4057,706,2011-04-26,2,2,0,14.6666669845581,44.6666679382324,0.666666686534882,60,3,2,206,
4058,706,2011-04-26,3,2,0,33.5,23.5,3,60,3,2,206,73,1,1,1,1,1,12,10931.26621,8228.046922,10.253
4059,706,2011-04-26,4,2,0,15.1666669845581,43.8333320617676,1,60,3,2,206,73,1,1,1,1,1,12,109
4060,706,2011-04-29,1,5,0,9.25,27.5,0.5,37.25,3,2,206,73,1,1,1,1,1,12,10931.26621,8228.046922,1
4061,706,2011-04-29,2,5,0,42.6666679382324,17.3333339691162,0,60,3,2,206,73,1,1,1,1,1,12,109
4062,706,2011-04-29,3,5,0,30.6666660308838,28.3333339691162,1,60,3,2,206,73,1,1,1,1,1,12,109
4063,706,2011-04-29,4,5,0,34.3333320617676,24.3333339691162,1.33333337306976,60,3,2,206,7
4064,708,2010-10-01,0,5,0,40,2,1,43,1,2,206,76,0,1,0,0,1,9,11208.03366,5832.334896,11.7524529,
4065,708,2010-10-01,1,5,0,26.25,21.5,0,47.75,3,2,206,76,0,1,0,0,1,9,11208.03366,5832.334896,11.
4066,708,2010-10-01,2,5,0,45,15,0,60,3,2,206,76,0,1,0,0,1,9,11208.03366,5832.334896,11.752452
4067,708,2010-10-01,3,5,0,39,21,0,60,3,2,206,76,0,1,0,0,1,9,11208.03366,5832.334896,11.752452
4068,708,2010-10-01,4,5,0,50,10,0,60,3,2,206,76,0,1,0,0,1,9,11208.03366,5832.334896,11.752452
4069,708,2010-10-02,0,6,1,42.3333320617676,17.6666660308838,0,60,3,2,206,76,0,1,0,0,1,9,1120
4070,708,2010-10-02,1,6,1,15,36.75,8.25,60,3,2,206,76,0,1,0,0,1,9,11208.03366,5832.334896,11.75
4071,708,2010-10-02,2,6,1,30,26.6666660308838,3.33333325386047,60,3,2,206,76,0,1,0,0,1,9,112
4072,708,2010-10-02,3,6,1,48.3333320617676,11.6666669845581,0,60,3,2,206,76,0,1,0,0,1,9,1120
4073,708,2010-10-02,4,6,1,50.3333320617676,8,1.66666662693024,60,3,2,206,76,0,1,0,0,1,9,1120
4074,708,2010-10-03,1,0,1,11.5,30.75,3.75,46,3,2,206,76,0,1,0,0,1,9,11208.03366,5832.334896,11.
4075,708,2010-10-03,2,0,1,38.3333320617676,19.1666660308838,2.5,60,3,2,206,76,0,1,0,0,1,9,112
4076,708,2010-10-03,3,0,1,38.5,21.5,0,60,3,2,206,76,0,1,0,0,1,9,11208.03366,5832.334896,11.7524
4077,708,2010-10-03,4,0,1,53.5,6.25,0.25,60,3,2,206,76,0,1,0,0,1,9,11208.03366,5832.334896,11.7
4078,708,2010-10-04,4,1,0,51,9,0,60,2,2,206,76,0,1,0,0,1,9,11208.03366,5832.334896,11.7524529,
4079,708,2011-04-26,0,2,0,30,20,10,60,1,2,206,76,0,1,0,0,1,9,11208.03366,5832.334896,11.752452
4080,708,2011-04-26,1,2,0,14,29,17,60,1,2,206,76,0,1,0,0,1,9,11208.03366,5832.334896,11.752452
4081,708,2011-04-27,0,3,0,16,23,1,40,1,2,206,76,0,1,0,0,1,9,11208.03366,5832.334896,11.752452
4082,708,2011-04-27,1,3,0,21.6000003814697,34.7999992370605,3.59999990463257,60,3,2,206,7
4083,708,2011-04-27,2,3,0,34.1666679382324,25.6666660308838,0.16666667163372,60,3,2,206,7
4084,708,2011-04-27,3,3,0,38.1666679382324,21.1666660308838,0.666666686534882,60,3,2,206,
4085,708,2011-04-27,4,3,0,55,5,0,60,3,2,206,76,0,1,0,0,1,9,11208.03366,5832.334896,11.7524529,
4086,708,2011-04-28,1,4,0,17.6666660308838,24,5.5,47.1666679382324,3,2,206,76,0,1,0,0,1,9,112
4087,708,2011-04-28,2,4,0,43.3333320617676,16.6666660308838,0,60,3,2,206,76,0,1,0,0,1,9,1120
4088,708,2011-04-28,3,4,0,36.3333320617676,22.6666660308838,1,60,3,2,206,76,0,1,0,0,1,9,1120
4089,708,2011-04-28,4,4,0,48.7999992370605,11.1999998092651,0,60,3,2,206,76,0,1,0,0,1,9,1120
4090,708,2011-04-29,1,5,0,23.5,18,3,44.5,2,2,206,76,0,1,0,0,1,9,11208.03366,5832.334896,11.7524
4091,708,2011-04-29,2,5,0,33.8333320617676,22.8333339691162,3.33333325386047,60,3,2,206,7
4092,708,2011-04-29,3,5,0,35.3333320617676,19.3333339691162,5.33333349227905,60,3,2,206,7
4093,708,2011-04-29,4,5,0,46.25,13,0.75,60,3,2,206,76,0,1,0,0,1,9,11208.03366,5832.334896,11.75
4094,710,2010-10-01,3,5,0,21,39,0,60,1,2,206,70,0,1,0,1,1,12,10975.77993,7092.243956,10.880867
4095,710,2010-10-01,4,5,0,40,15.6666669845581,4.33333349227905,60,3,2,206,70,0,1,0,1,1,12,10
4096,710,2010-10-04,0,1,0,23,8,0,31,1,2,206,70,0,1,0,1,1,12,10975.77993,7092.243956,10.880867
4097,710,2010-10-04,1,1,0,16.6000003814697,16.7999992370605,15.3999996185303,48.7999992
4098,710,2010-10-04,2,1,0,32,27.3333339691162,0.666666686534882,60,3,2,206,70,0,1,0,1,1,12,1
4099,710,2010-10-04,3,1,0,25,29.3333339691162,5.66666650772095,60,3,2,206,70,0,1,0,1,1,12,10

4100,710,2010-10-04,4,1,0,40,17,3,60,3,2,206,70,0,1,0,1,1,12,10975.77993,7092.243956,10.880867
4101,710,2010-10-05,0,2,0,1,0,0,1,1,2,206,70,0,1,0,1,1,12,10975.77993,7092.243956,10.88086781,
4102,710,2010-10-05,1,2,0,28,24.75,7.25,60,3,2,206,70,0,1,0,1,1,12,10975.77993,7092.243956,10.8
4103,710,2010-10-05,2,2,0,21.1666660308838,37.1666679382324,1.66666662693024,60,3,2,206,7
4104,710,2010-10-05,3,2,0,41.6666679382324,18,0.333333343267441,60,3,2,206,70,0,1,0,1,1,12,1
4105,710,2010-10-05,4,2,0,36,21.6666660308838,2.33333325386047,60,3,2,206,70,0,1,0,1,1,12,10
4106,710,2010-10-06,0,3,0,2,11,1,14,1,2,206,70,0,1,0,1,1,12,10975.77993,7092.243956,10.880867
4107,710,2010-10-06,1,3,0,29,28.3333339691162,2.66666674613953,60,3,2,206,70,0,1,0,1,1,12,10
4108,710,2010-10-06,2,3,0,41,17.3333339691162,1.66666662693024,60,3,2,206,70,0,1,0,1,1,12,10
4109,710,2010-10-06,3,3,0,23.3333339691162,16.5,20.1666660308838,60,3,2,206,70,0,1,0,1,1,12,1
4110,710,2010-10-06,4,3,0,41.5,18.5,0,60,3,2,206,70,0,1,0,1,1,12,10975.77993,7092.243956,10.880
4111,710,2010-10-07,0,4,0,0,10,1,11,1,2,206,70,0,1,0,1,1,12,10975.77993,7092.243956,10.880867
4112,710,2010-10-07,1,4,0,27.1666660308838,14.1666669845581,16.1666660308838,57.5,3,2,206
4113,710,2010-10-07,2,4,0,28,30.3333339691162,1.66666662693024,60,3,2,206,70,0,1,0,1,1,12,10
4114,710,2010-10-07,3,4,0,18,40.6666679382324,1.33333337306976,60,3,2,206,70,0,1,0,1,1,12,10
4115,710,2010-10-07,4,4,0,40.3333320617676,19.6666660308838,0,60,3,2,206,70,0,1,0,1,1,12,109
4116,710,2010-10-08,1,5,0,38.3333320617676,18.3333339691162,0,56.6666679382324,3,2,206,70
4117,710,2010-10-08,2,5,0,33.5,26.5,0,60,3,2,206,70,0,1,0,1,1,12,10975.77993,7092.243956,10.880
4118,710,2010-10-08,3,5,0,20.3999996185303,19.6000003814697,20,60,3,2,206,70,0,1,0,1,1,12,10
4119,710,2010-10-08,4,5,0,44.3333320617676,15.3333330154419,0.333333343267441,60,3,2,206,
4120,712,2010-09-30,0,4,0,14.3333330154419,38.3333320617676,6.66666650772095,59.333332
4121,712,2010-09-30,1,4,0,23.3333339691162,32.6666679382324,4,60,3,2,206,76,0,1,0,1,1,10,109
4122,712,2010-09-30,2,4,0,39.3333320617676,20.6666660308838,0,60,3,2,206,76,0,1,0,1,1,10,109
4123,712,2010-09-30,3,4,0,29,31,0,60,1,2,206,76,0,1,0,1,1,10,10901.90647,8355.935604,10.229358
4124,712,2010-09-30,4,4,0,36,16.6666660308838,0,52.6666679382324,3,2,206,76,0,1,0,1,1,10,109
4125,712,2010-10-01,0,5,0,9,47,4,60,1,2,206,76,0,1,0,1,1,10,10901.90647,8355.935604,10.2293589
4126,712,2010-10-01,1,5,0,10.6666669845581,43.6666679382324,5.66666650772095,60,3,2,206,7
4127,712,2010-10-01,2,5,0,36,23.6666660308838,0.333333343267441,60,3,2,206,76,0,1,0,1,1,10,1
4128,712,2010-10-01,3,5,0,20.6666660308838,33.6666679382324,5.66666650772095,60,3,2,206,7
4129,712,2010-10-01,4,5,0,21,39,0,60,1,2,206,76,0,1,0,1,1,10,10901.90647,8355.935604,10.229358
4130,712,2010-10-02,0,6,1,11,44.6666679382324,4.33333349227905,60,3,2,206,76,0,1,0,1,1,10,10
4131,712,2010-10-02,1,6,1,27.6666660308838,26.3333339691162,6,60,3,2,206,76,0,1,0,1,1,10,109
4132,712,2010-10-02,2,6,1,38.6666679382324,20.6666660308838,0.666666686534882,60,3,2,206,
4133,712,2010-10-02,3,6,1,36.3333320617676,20,3.66666674613953,60,3,2,206,76,0,1,0,1,1,10,10
4134,712,2010-10-02,4,6,1,47.5,12,0.25,59.75,3,2,206,76,0,1,0,1,1,10,10901.90647,8355.935604,10
4135,712,2010-10-03,0,0,1,17.6666660308838,40.6666679382324,1.66666662693024,60,3,2,206,7
4136,712,2010-10-03,1,0,1,23.6666660308838,32,4.33333349227905,60,3,2,206,76,0,1,0,1,1,10,10
4137,712,2010-10-03,2,0,1,36.3333320617676,19.6666660308838,4,60,3,2,206,76,0,1,0,1,1,10,109
4138,712,2010-10-03,3,0,1,26,32.6666679382324,1.33333337306976,60,3,2,206,76,0,1,0,1,1,10,10
4139,712,2010-10-03,4,0,1,30.3333339691162,17.6666660308838,0,48,3,2,206,76,0,1,0,1,1,10,109
4140,712,2010-10-04,0,1,0,12,36.3333320617676,2.33333325386047,50.6666679382324,3,2,206,7
4141,712,2010-10-04,1,1,0,12.6666669845581,45.6666679382324,1.66666662693024,60,3,2,206,7
4142,712,2010-10-04,2,1,0,30,27.3333339691162,2.66666674613953,60,3,2,206,76,0,1,0,1,1,10,10
4143,712,2010-10-04,3,1,0,23.6666660308838,36.3333320617676,0,60,3,2,206,76,0,1,0,1,1,10,109
4144,712,2010-10-04,4,1,0,29.5,13,0,42.5,2,2,206,76,0,1,0,1,1,10,10901.90647,8355.935604,10.229
4145,712,2010-10-05,0,2,0,9.33333301544189,48.6666679382324,2,60,3,2,206,76,0,1,0,1,1,10,109
4146,712,2010-10-05,1,2,0,16.6666660308838,39.3333320617676,4,60,3,2,206,76,0,1,0,1,1,10,109
4147,712,2010-10-05,2,2,0,32,28,0,60,3,2,206,76,0,1,0,1,1,10,10901.90647,8355.935604,10.229358
4148,712,2010-10-05,3,2,0,16.6666660308838,42.6666679382324,0.666666686534882,60,3,2,206,
4149,712,2010-10-05,4,2,0,19,11,0.5,30.5,2,2,206,76,0,1,0,1,1,10,10901.90647,8355.935604,10.229

4150,712,2010-10-06,0,3,0,13.3333330154419,45,0.666666686534882,59,3,2,206,76,0,1,0,1,1,10,1
4151,712,2010-10-06,1,3,0,14.3333330154419,42.6666679382324,3,60,3,2,206,76,0,1,0,1,1,10,109
4152,712,2010-10-06,2,3,0,41,19,0,60,3,2,206,76,0,1,0,1,1,10,10901.90647,8355.935604,10.22935
4153,712,2010-10-06,3,3,0,17.5,42,0.5,60,2,2,206,76,0,1,0,1,1,10,10901.90647,8355.935604,10.22
4154,712,2010-10-06,4,3,0,40.3333320617676,19,0.666666686534882,60,3,2,206,76,0,1,0,1,1,10,1
4155,712,2010-10-07,3,4,0,18.3333339691162,41.3333320617676,0.333333343267441,60,3,2,206,
4156,712,2010-10-07,4,4,0,40,20,0,60,3,2,206,76,0,1,0,1,1,10,10901.90647,8355.935604,10.22935
4157,714,2010-10-07,0,4,0,2,15,13,30,1,2,206,69,1,1,1,1,1,12,10711.84153,8490.900718,10.19124
4158,714,2010-10-07,1,4,0,9.66666698455811,38.6666679382324,11.6666669845581,60,3,2,206,6
4159,714,2010-10-07,2,4,0,35.6666679382324,24.3333339691162,0,60,3,2,206,69,1,1,1,1,1,12,107
4160,714,2010-10-08,0,5,0,2,44,14,60,1,2,206,69,1,1,1,1,1,12,10711.84153,8490.900718,10.19124
4161,714,2010-10-08,1,5,0,18.3333339691162,31.3333339691162,10.3333330154419,60,3,2,206,6
4162,714,2010-10-08,2,5,0,28.5,28,3.5,60,2,2,206,69,1,1,1,1,1,12,10711.84153,8490.900718,10.19
4163,714,2010-10-08,3,5,0,18.6666660308838,35,6.33333349227905,60,3,2,206,69,1,1,1,1,1,12,10
4164,714,2010-10-08,4,5,0,25,31.6666660308838,3.33333325386047,60,3,2,206,69,1,1,1,1,1,12,10
4165,714,2010-10-09,0,6,1,5.5,28.5,1.5,35.5,2,2,206,69,1,1,1,1,1,12,10711.84153,8490.900718,10.
4166,714,2010-10-09,1,6,1,8,35.3333320617676,16.6666660308838,60,3,2,206,69,1,1,1,1,1,12,107
4167,714,2010-10-09,2,6,1,1,49,10,60,1,2,206,69,1,1,1,1,1,12,10711.84153,8490.900718,10.19124
4168,714,2010-10-09,3,6,1,33.6666679382324,24,2.33333325386047,60,3,2,206,69,1,1,1,1,1,12,10
4169,714,2010-10-09,4,6,1,43,17,0,60,3,2,206,69,1,1,1,1,1,12,10711.84153,8490.900718,10.19124
4170,714,2010-10-10,0,0,1,26.5,11,1,38.5,2,2,206,69,1,1,1,1,1,12,10711.84153,8490.900718,10.19
4171,714,2010-10-10,1,0,1,2,44,14,60,1,2,206,69,1,1,1,1,1,12,10711.84153,8490.900718,10.19124
4172,714,2010-10-10,2,0,1,50,8.66666698455811,1.33333337306976,60,3,2,206,69,1,1,1,1,1,12,10
4173,714,2010-10-10,3,0,1,11,32.3333320617676,16.6666660308838,60,3,2,206,69,1,1,1,1,1,12,10
4174,714,2010-10-10,4,0,1,16.3333339691162,38.6666679382324,5,60,3,2,206,69,1,1,1,1,1,12,107
4175,714,2010-10-11,0,1,0,4,24,9,37,1,2,206,69,1,1,1,1,1,12,10711.84153,8490.900718,10.191245
4176,714,2010-10-11,1,1,0,1.25,40.25,18.5,60,3,2,206,69,1,1,1,1,1,12,10711.84153,8490.900718,1
4177,714,2010-10-11,2,1,0,19.6666660308838,35.3333320617676,5,60,3,2,206,69,1,1,1,1,1,12,107
4178,714,2010-10-11,3,1,0,10,46.3333320617676,3.66666674613953,60,3,2,206,69,1,1,1,1,1,12,10
4179,714,2010-10-11,4,1,0,12.6666669845581,35,5.66666650772095,53.3333320617676,3,2,206,6
4180,714,2010-10-12,0,2,0,1.5,31.5,9.5,42.5,2,2,206,69,1,1,1,1,1,12,10711.84153,8490.900718,10.
4181,714,2010-10-12,1,2,0,4.66666650772095,47,8.33333301544189,60,3,2,206,69,1,1,1,1,1,12,10
4182,714,2010-10-12,2,2,0,11,45.6666679382324,3.33333325386047,60,3,2,206,69,1,1,1,1,1,12,10
4183,714,2010-10-12,3,2,0,15,44,1,60,3,2,206,69,1,1,1,1,1,12,10711.84153,8490.900718,10.19124
4184,714,2010-10-12,4,2,0,13,32.5,3,48.5,2,2,206,69,1,1,1,1,1,12,10711.84153,8490.900718,10.19
4185,714,2010-10-13,0,3,0,5.5,30.5,0.5,36.5,2,2,206,69,1,1,1,1,1,12,10711.84153,8490.900718,10.
4186,714,2010-10-13,1,3,0,11.25,41.25,7.5,60,3,2,206,69,1,1,1,1,1,12,10711.84153,8490.900718,1
4187,714,2010-10-13,2,3,0,9.33333301544189,15.3333330154419,35.3333320617676,60,3,2,206,6
4188,714,2010-10-13,3,3,0,16.6666660308838,37.3333320617676,6,60,3,2,206,69,1,1,1,1,1,12,107
4189,714,2010-10-13,4,3,0,5,41.5,1.5,48,2,2,206,69,1,1,1,1,1,12,10711.84153,8490.900718,10.1912
4190,714,2010-10-14,2,4,0,15.6666669845581,42.3333320617676,2,60,3,2,206,69,1,1,1,1,1,12,107
4191,714,2010-10-14,3,4,0,8.33333301544189,49,2.66666674613953,60,3,2,206,69,1,1,1,1,1,12,10
4192,714,2010-10-14,4,4,0,11.3333330154419,45.6666679382324,3,60,3,2,206,69,1,1,1,1,1,12,107
4193,719,2010-10-07,0,4,0,12.3333330154419,26.6666660308838,2,41,3,2,206,66,0,1,1,1,1,12,110
4194,719,2010-10-07,1,4,0,38,19.3333339691162,2.66666674613953,60,3,2,206,66,0,1,1,1,1,12,11
4195,719,2010-10-07,2,4,0,25.25,30.25,4.5,60,3,2,206,66,0,1,1,1,1,12,11047.69049,8090.101009,1
4196,719,2010-10-07,3,4,0,12.3333330154419,44,3.66666674613953,60,3,2,206,66,0,1,1,1,1,12,11
4197,719,2010-10-07,4,4,0,18,36.6666679382324,5.33333349227905,60,3,2,206,66,0,1,1,1,1,12,11
4198,719,2010-10-08,0,5,0,16.3333339691162,23.3333339691162,1.66666662693024,41.333332
4199,719,2010-10-08,1,5,0,25.6666660308838,31.6666660308838,2.66666674613953,60,3,2,206,6

4200,719,2010-10-08,2,5,0,25.6666660308838,29,5.33333349227905,60,3,2,206,66,0,1,1,1,1,12,11
4201,719,2010-10-08,3,5,0,16.25,37.75,6,60,3,2,206,66,0,1,1,1,1,12,11047.69049,8090.101009,10.0
4202,719,2010-10-08,4,5,0,23,28.6666660308838,8.33333301544189,60,3,2,206,66,0,1,1,1,1,12,11
4203,719,2010-10-09,0,6,1,14.6666669845581,24.6666660308838,1.66666662693024,41,3,2,206,6
4204,719,2010-10-09,1,6,1,23.3333339691162,30.6666660308838,6,60,3,2,206,66,0,1,1,1,1,12,110
4205,719,2010-10-09,2,6,1,33,26.3333339691162,0.666666686534882,60,3,2,206,66,0,1,1,1,1,12,1
4206,719,2010-10-09,3,6,1,20.75,32.25,7,60,3,2,206,66,0,1,1,1,1,12,11047.69049,8090.101009,10.0
4207,719,2010-10-09,4,6,1,31,27,2,60,3,2,206,66,0,1,1,1,1,12,11047.69049,8090.101009,10.003467
4208,719,2010-10-10,0,0,1,35.5,23,0.5,59,2,2,206,66,0,1,1,1,1,12,11047.69049,8090.101009,10.003
4209,719,2010-10-10,1,0,1,21,28.6666660308838,10.3333330154419,60,3,2,206,66,0,1,1,1,1,12,11
4210,719,2010-10-10,2,0,1,38.6666679382324,19.3333339691162,2,60,3,2,206,66,0,1,1,1,1,12,110
4211,719,2010-10-10,3,0,1,17,26.25,16.75,60,3,2,206,66,0,1,1,1,1,12,11047.69049,8090.101009,10
4212,719,2010-10-10,4,0,1,17,41,2,60,3,2,206,66,0,1,1,1,1,12,11047.69049,8090.101009,10.003467
4213,719,2010-10-11,0,1,0,1,11,32,44,1,2,206,66,0,1,1,1,1,12,11047.69049,8090.101009,10.003467
4214,719,2010-10-11,1,1,0,14.3333330154419,27.3333339691162,18.3333339691162,60,3,2,206,6
4215,719,2010-10-11,2,1,0,27.6666660308838,31.6666660308838,0.666666686534882,60,3,2,206,
4216,719,2010-10-11,3,1,0,32,21.3333339691162,6.66666650772095,60,3,2,206,66,0,1,1,1,1,12,11
4217,719,2010-10-11,4,1,0,26.8333339691162,31.8333339691162,1.33333337306976,60,3,2,206,6
4218,719,2010-10-12,0,2,0,8.66666698455811,25.3333339691162,3.33333325386047,37.3333320
4219,719,2010-10-12,1,2,0,28.6666660308838,30.6666660308838,0.666666686534882,60,3,2,206,
4220,719,2010-10-12,2,2,0,30.3333339691162,28.6666660308838,1,60,3,2,206,66,0,1,1,1,1,12,110
4221,719,2010-10-12,3,2,0,27.6666660308838,30.6666660308838,1.66666662693024,60,3,2,206,6
4222,719,2010-10-12,4,2,0,22.3333339691162,33.3333320617676,4.33333349227905,60,3,2,206,6
4223,719,2010-10-13,0,3,0,11.5,43.5,5,60,2,2,206,66,0,1,1,1,1,12,11047.69049,8090.101009,10.003
4224,719,2010-10-13,1,3,0,19.6666660308838,33.3333320617676,7,60,3,2,206,66,0,1,1,1,1,12,110
4225,719,2010-10-13,2,3,0,31.3333339691162,25.6666660308838,3,60,3,2,206,66,0,1,1,1,1,12,110
4226,719,2010-10-13,3,3,0,23.3333339691162,30.6666660308838,6,60,3,2,206,66,0,1,1,1,1,12,110
4227,719,2010-10-13,4,3,0,29.3333339691162,27,3.66666674613953,60,3,2,206,66,0,1,1,1,1,12,11
4228,719,2010-10-14,0,4,0,11,29.6666660308838,3.66666674613953,44.3333320617676,3,2,206,6
4229,719,2010-10-14,1,4,0,13,46,1,60,1,2,206,66,0,1,1,1,1,12,11047.69049,8090.101009,10.003467
4230,719,2010-10-22,0,5,0,10,46,4,60,1,2,206,66,0,1,1,1,1,12,11047.69049,8090.101009,10.003467
4231,719,2010-10-22,1,5,0,32,26.2000007629395,1.79999995231628,60,3,2,206,66,0,1,1,1,1,12,11
4232,719,2010-10-22,2,5,0,34.2000007629395,24,1.79999995231628,60,3,2,206,66,0,1,1,1,1,12,11
4233,719,2010-10-22,3,5,0,33.6666679382324,8.66666698455811,0.666666686534882,43,3,2,206,
4234,719,2010-10-24,0,0,1,15,39.5,4.5,59,2,2,206,66,0,1,1,1,1,12,11047.69049,8090.101009,10.003
4235,719,2010-10-24,1,0,1,11.3333330154419,39.6666679382324,9,60,3,2,206,66,0,1,1,1,1,12,110
4236,719,2010-10-24,2,0,1,9.66666698455811,46.3333320617676,4,60,3,2,206,66,0,1,1,1,1,12,110
4237,719,2010-10-24,3,0,1,12,42.6666679382324,5.33333349227905,60,3,2,206,66,0,1,1,1,1,12,11
4238,719,2010-10-24,4,0,1,37,22,1,60,3,2,206,66,0,1,1,1,1,12,11047.69049,8090.101009,10.003467
4239,719,2010-10-25,0,1,0,15,32,1,48,1,2,206,66,0,1,1,1,1,12,11047.69049,8090.101009,10.003467
4240,719,2010-10-25,1,1,0,10.3333330154419,40,9.66666698455811,60,3,2,206,66,0,1,1,1,1,12,11
4241,719,2010-10-25,2,1,0,5,46,9,60,2,2,206,66,0,1,1,1,1,12,11047.69049,8090.101009,10.0034670
4242,719,2010-10-25,3,1,0,9.66666698455811,43.6666679382324,6.66666650772095,60,3,2,206,6
4243,719,2010-10-25,4,1,0,5.33333349227905,52,2.66666674613953,60,3,2,206,66,0,1,1,1,1,12,11
4244,719,2010-10-26,1,2,0,26.5,32,0.5,59,2,2,206,66,0,1,1,1,1,12,11047.69049,8090.101009,10.003
4245,719,2010-10-26,2,2,0,41.5999984741211,17.7999992370605,0.600000023841858,60,3,2,206,
4246,719,2010-10-26,3,2,0,13.5,39,7.5,60,3,2,206,66,0,1,1,1,1,12,11047.69049,8090.101009,10.003
4247,719,2010-10-26,4,2,0,21,35.3333320617676,3.66666674613953,60,3,2,206,66,0,1,1,1,1,12,11
4248,719,2010-10-27,0,3,0,10,44,4,58,2,2,206,66,0,1,1,1,1,12,11047.69049,8090.101009,10.003467
4249,719,2010-10-27,1,3,0,40.3333320617676,19.6666660308838,0,60,3,2,206,66,0,1,1,1,1,12,110

4250,719,2010-10-27,2,3,0,26.3333339691162,30,3.66666674613953,60,3,2,206,66,0,1,1,1,1,12,11
4251,719,2010-10-27,3,3,0,41.5,18,0.5,60,2,2,206,66,0,1,1,1,1,12,11047.69049,8090.101009,10.003
4252,719,2010-10-27,4,3,0,31.3333339691162,26.6666660308838,2,60,3,2,206,66,0,1,1,1,1,12,110
4253,719,2010-10-28,0,4,0,23.5,33.5,1.5,58.5,2,2,206,66,0,1,1,1,1,12,11047.69049,8090.101009,10
4254,719,2010-10-28,1,4,0,18,37.3333320617676,4.66666650772095,60,3,2,206,66,0,1,1,1,1,12,11
4255,719,2010-10-28,2,4,0,30,26.5,3.5,60,2,2,206,66,0,1,1,1,1,12,11047.69049,8090.101009,10.003
4256,719,2010-10-28,3,4,0,21.3333339691162,36.3333320617676,2.33333325386047,60,3,2,206,6
4257,719,2010-10-28,4,4,0,15.6666669845581,38.3333320617676,6,60,3,2,206,66,0,1,1,1,1,12,110
4258,720,2010-10-07,0,4,0,6,34,4.5,44.5,2,2,206,68,0,1,0,1,1,12,10932.06855,6902.306226,10.967
4259,720,2010-10-07,1,4,0,18,39.3333320617676,2.66666674613953,60,3,2,206,68,0,1,0,1,1,12,10
4260,720,2010-10-07,2,4,0,24.3333339691162,35.6666679382324,0,60,3,2,206,68,0,1,0,1,1,12,109
4261,720,2010-10-07,3,4,0,10.3333330154419,47.3333320617676,2.33333325386047,60,3,2,206,6
4262,720,2010-10-07,4,4,0,32.3333320617676,25.6666660308838,2,60,3,2,206,68,0,1,0,1,1,12,109
4263,720,2010-10-08,1,5,0,18.6666660308838,29.6666660308838,11.6666669845581,60,3,2,206,6
4264,720,2010-10-08,2,5,0,11.6666669845581,30.6666660308838,17.6666660308838,60,3,2,206,6
4265,720,2010-10-08,3,5,0,18.6666660308838,21.6666660308838,19.6666660308838,60,3,2,206,6
4266,720,2010-10-08,4,5,0,13.5,36,10.5,60,2,2,206,68,0,1,0,1,1,12,10932.06855,6902.306226,10.96
4267,720,2010-10-09,0,6,1,10.6666669845581,39,6.33333349227905,56,3,2,206,68,0,1,0,1,1,12,10
4268,720,2010-10-09,1,6,1,14.6666669845581,25,20.3333339691162,60,3,2,206,68,0,1,0,1,1,12,10
4269,720,2010-10-09,2,6,1,21,28.6666660308838,10.3333330154419,60,3,2,206,68,0,1,0,1,1,12,10
4270,720,2010-10-09,3,6,1,19,25,16,60,1,2,206,68,0,1,0,1,1,12,10932.06855,6902.306226,10.96795
4271,720,2010-10-09,4,6,1,15,39,6,60,1,2,206,68,0,1,0,1,1,12,10932.06855,6902.306226,10.967959
4272,720,2010-10-11,0,1,0,19,32,0,51,1,2,206,68,0,1,0,1,1,12,10932.06855,6902.306226,10.967959
4273,720,2010-10-11,1,1,0,32.6666679382324,25,2.33333325386047,60,3,2,206,68,0,1,0,1,1,12,10
4274,720,2010-10-11,2,1,0,29.3333339691162,28.6666660308838,2,60,3,2,206,68,0,1,0,1,1,12,109
4275,720,2010-10-11,3,1,0,18,29.3333339691162,12.6666669845581,60,3,2,206,68,0,1,0,1,1,12,10
4276,720,2010-10-11,4,1,0,20.3333339691162,38.6666679382324,1,60,3,2,206,68,0,1,0,1,1,12,109
4277,720,2010-10-13,0,3,0,13.6666669845581,34,2,49.6666679382324,3,2,206,68,0,1,0,1,1,12,109
4278,720,2010-10-13,1,3,0,11.3333330154419,44.3333320617676,4.33333349227905,60,3,2,206,6
4279,720,2010-10-13,2,3,0,17.3333339691162,41.6666679382324,1,60,3,2,206,68,0,1,0,1,1,12,109
4280,720,2010-10-13,3,3,0,9.5,29,21.5,60,2,2,206,68,0,1,0,1,1,12,10932.06855,6902.306226,10.967
4281,720,2010-10-13,4,3,0,42,17.6666660308838,0.333333343267441,60,3,2,206,68,0,1,0,1,1,12,1
4282,720,2010-10-14,0,4,0,13,36.3333320617676,2.33333325386047,51.6666679382324,3,2,206,6
4283,720,2010-10-14,1,4,0,4,53,3,60,3,2,206,68,0,1,0,1,1,12,10932.06855,6902.306226,10.9679597
4284,720,2010-10-14,2,4,0,26.6666660308838,32,1.33333337306976,60,3,2,206,68,0,1,0,1,1,12,10
4285,720,2010-10-14,3,4,0,22.3333339691162,23.6666660308838,14,60,3,2,206,68,0,1,0,1,1,12,10
4286,720,2010-10-14,4,4,0,31,22,7,60,3,2,206,68,0,1,0,1,1,12,10932.06855,6902.306226,10.967959
4287,720,2010-10-20,0,3,0,8.33333301544189,47.3333320617676,2.33333325386047,58,3,2,206,6
4288,720,2010-10-20,1,3,0,7.66666650772095,46.6666679382324,5.66666650772095,60,3,2,206,6
4289,720,2010-10-20,2,3,0,21,37,2,60,1,2,206,68,0,1,0,1,1,12,10932.06855,6902.306226,10.967959
4290,720,2010-10-20,3,3,0,15.6666669845581,35,9.33333301544189,60,3,2,206,68,0,1,0,1,1,12,10
4291,720,2010-10-20,4,3,0,27.6666660308838,30.3333339691162,2,60,3,2,206,68,0,1,0,1,1,12,109
4292,720,2010-10-21,2,4,0,18.5,7.5,34,60,2,2,206,68,0,1,0,1,1,12,10932.06855,6902.306226,10.967
4293,720,2010-10-21,3,4,0,29,17,14,60,3,2,206,68,0,1,0,1,1,12,10932.06855,6902.306226,10.96795
4294,720,2010-10-21,4,4,0,28.3333339691162,29,2.66666674613953,60,3,2,206,68,0,1,0,1,1,12,10
4295,721,2010-10-14,0,4,0,19.6666660308838,22.3333339691162,0.333333343267441,42.333332
4296,721,2010-10-14,1,4,0,16,33.25,10.75,60,3,2,206,73,1,1,0,1,1,11,11052.36828,6452.864273,11
4297,721,2010-10-14,2,4,0,15.25,41,3.75,60,3,2,206,73,1,1,0,1,1,11,11052.36828,6452.864273,11.0
4298,721,2010-10-14,3,4,0,10.3333330154419,43,6.66666650772095,60,3,2,206,73,1,1,0,1,1,11,11
4299,721,2010-10-14,4,4,0,14,24.5,0,38.5,2,2,206,73,1,1,0,1,1,11,11052.36828,6452.864273,11.067

4300,721,2010-10-15,2,5,0,49,11,0,60,2,2,206,73,1,1,0,1,1,11,11052.36828,6452.864273,11.06717
4301,721,2010-10-15,3,5,0,32,24.3333339691162,3.66666674613953,60,3,2,206,73,1,1,0,1,1,11,11
4302,721,2010-10-15,4,5,0,0,7,21,28,1,2,206,73,1,1,0,1,1,11,11052.36828,6452.864273,11.067173
4303,721,2010-10-21,0,4,0,28,28.5,1.5,58,2,2,206,73,1,1,0,1,1,11,11052.36828,6452.864273,11.067
4304,721,2010-10-21,1,4,0,10.3333330154419,41.3333320617676,8.33333301544189,60,3,2,206,7
4305,721,2010-10-21,2,4,0,4,36,20,60,1,2,206,73,1,1,0,1,1,11,11052.36828,6452.864273,11.06717
4306,721,2010-10-22,0,5,0,23,33,0,56,2,2,206,73,1,1,0,1,1,11,11052.36828,6452.864273,11.06717
4307,721,2010-10-22,1,5,0,23,32.3333320617676,4.66666650772095,60,3,2,206,73,1,1,0,1,1,11,11
4308,721,2010-10-22,2,5,0,47,13,0,60,2,2,206,73,1,1,0,1,1,11,11052.36828,6452.864273,11.06717
4309,721,2010-10-22,3,5,0,35.6666679382324,23.3333339691162,1,60,3,2,206,73,1,1,0,1,1,11,110
4310,721,2010-10-22,4,5,0,37.3333320617676,22.6666660308838,0,60,3,2,206,73,1,1,0,1,1,11,110
4311,721,2010-10-23,0,6,1,21.3333339691162,30,0.666666686534882,52,3,2,206,73,1,1,0,1,1,11,1
4312,721,2010-10-23,1,6,1,14,43,3,60,3,2,206,73,1,1,0,1,1,11,11052.36828,6452.864273,11.06717
4313,721,2010-10-23,2,6,1,19.25,37.25,3.5,60,3,2,206,73,1,1,0,1,1,11,11052.36828,6452.864273,11
4314,721,2010-10-23,3,6,1,18.6666660308838,29.6666660308838,11.6666669845581,60,3,2,206,7
4315,721,2010-10-23,4,6,1,49,9,1,59,1,2,206,73,1,1,0,1,1,11,11052.36828,6452.864273,11.067173
4316,721,2010-10-24,0,0,1,14.6666669845581,45.3333320617676,0,60,3,2,206,73,1,1,0,1,1,11,110
4317,721,2010-10-24,1,0,1,10,46.5,3.5,60,2,2,206,73,1,1,0,1,1,11,11052.36828,6452.864273,11.067
4318,721,2010-10-24,2,0,1,54,6,0,60,2,2,206,73,1,1,0,1,1,11,11052.36828,6452.864273,11.067173
4319,721,2010-10-24,3,0,1,22.6666660308838,37.3333320617676,0,60,3,2,206,73,1,1,0,1,1,11,110
4320,721,2010-10-24,4,0,1,33.3333320617676,26.6666660308838,0,60,3,2,206,73,1,1,0,1,1,11,110
4321,721,2010-10-25,0,1,0,29.5,20.5,0.5,50.5,2,2,206,73,1,1,0,1,1,11,11052.36828,6452.864273,11
4322,721,2010-10-25,1,1,0,15,43,2,60,3,2,206,73,1,1,0,1,1,11,11052.36828,6452.864273,11.06717
4323,721,2010-10-25,2,1,0,37,19.3333339691162,3.66666674613953,60,3,2,206,73,1,1,0,1,1,11,11
4324,721,2010-10-25,3,1,0,8.33333301544189,47.3333320617676,4.33333349227905,60,3,2,206,7
4325,721,2010-10-25,4,1,0,12.6666669845581,38,0.666666686534882,51.3333320617676,3,2,206,
4326,721,2010-10-26,0,2,0,10.5,28.5,1,40,2,2,206,73,1,1,0,1,1,11,11052.36828,6452.864273,11.067
4327,721,2010-10-26,1,2,0,11.3333330154419,45,3.66666674613953,60,3,2,206,73,1,1,0,1,1,11,11
4328,721,2010-10-26,2,2,0,28,31.6666660308838,0.333333343267441,60,3,2,206,73,1,1,0,1,1,11,1
4329,721,2010-10-26,3,2,0,43.6666679382324,16.3333339691162,0,60,3,2,206,73,1,1,0,1,1,11,110
4330,721,2010-10-26,4,2,0,36.6666679382324,23.3333339691162,0,60,3,2,206,73,1,1,0,1,1,11,110
4331,722,2010-10-15,0,5,0,6,14,0,20,1,2,206,67,0,1,1,1,1,12,11021.59726,7908.312506,10.137847
4332,722,2010-10-15,1,5,0,31,27.6666660308838,1.33333337306976,60,3,2,206,67,0,1,1,1,1,12,11
4333,722,2010-10-15,2,5,0,28.6666660308838,31.3333339691162,0,60,3,2,206,67,0,1,1,1,1,12,110
4334,722,2010-10-15,3,5,0,30.3333339691162,28,1.66666662693024,60,3,2,206,67,0,1,1,1,1,12,11
4335,722,2010-10-15,4,5,0,42,18,0,60,1,2,206,67,0,1,1,1,1,12,11021.59726,7908.312506,10.137847
4336,722,2010-10-16,0,6,1,12.5,7,3.5,23,2,2,206,67,0,1,1,1,1,12,11021.59726,7908.312506,10.1378
4337,722,2010-10-16,1,6,1,31.25,22.25,6.5,60,3,2,206,67,0,1,1,1,1,12,11021.59726,7908.312506,10
4338,722,2010-10-16,2,6,1,32,27.6666660308838,0.333333343267441,60,3,2,206,67,0,1,1,1,1,12,1
4339,722,2010-10-16,3,6,1,32,26.6666660308838,1.33333337306976,60,3,2,206,67,0,1,1,1,1,12,11
4340,722,2010-10-16,4,6,1,33.7999992370605,26,0.200000002980232,60,3,2,206,67,0,1,1,1,1,12,1
4341,722,2010-10-17,0,0,1,28,17,15,60,1,2,206,67,0,1,1,1,1,12,11021.59726,7908.312506,10.1378
4342,722,2010-10-17,1,0,1,36.4000015258789,22.3999996185303,1.20000004768372,60,3,2,206,6
4343,722,2010-10-17,2,0,1,35.5999984741211,24.3999996185303,0,60,3,2,206,67,0,1,1,1,1,12,110
4344,722,2010-10-17,3,0,1,32.6666679382324,25.6666660308838,1.66666662693024,60,3,2,206,6
4345,722,2010-10-17,4,0,1,28.6666660308838,28.6666660308838,2.66666674613953,60,3,2,206,6
4346,722,2010-10-18,0,1,0,38.5,9.5,7.5,55.5,2,2,206,67,0,1,1,1,1,12,11021.59726,7908.312506,10.
4347,722,2010-10-18,1,1,0,37.3333320617676,19.6666660308838,3,60,3,2,206,67,0,1,1,1,1,12,110
4348,722,2010-10-18,2,1,0,37.75,21.75,0.5,60,3,2,206,67,0,1,1,1,1,12,11021.59726,7908.312506,10
4349,722,2010-10-18,3,1,0,40,19.2000007629395,0.800000011920929,60,3,2,206,67,0,1,1,1,1,12,1

4350,722,2010-10-18,4,1,0,43,17,0,60,3,2,206,67,0,1,1,1,1,12,11021.59726,7908.312506,10.13784
4351,722,2010-10-19,0,2,0,11,10.5,2.5,24,2,2,206,67,0,1,1,1,1,12,11021.59726,7908.312506,10.13
4352,722,2010-10-19,1,2,0,44.6666679382324,10.6666669845581,4.66666650772095,60,3,2,206,6
4353,722,2010-10-19,2,2,0,31,28.3333339691162,0.666666686534882,60,3,2,206,67,0,1,1,1,1,12,1
4354,722,2010-10-19,3,2,0,33,25.25,1.75,60,3,2,206,67,0,1,1,1,1,12,11021.59726,7908.312506,10.1
4355,722,2010-10-19,4,2,0,44.8333320617676,14.5,0.666666686534882,60,3,2,206,67,0,1,1,1,1,12
4356,722,2010-10-20,1,3,0,36.6666679382324,21.6666660308838,1.66666662693024,60,3,2,206,6
4357,722,2010-10-20,2,3,0,41,18.6666660308838,0.333333343267441,60,3,2,206,67,0,1,1,1,1,12,1
4358,722,2010-10-20,3,3,0,24.6666660308838,34.6666679382324,0.666666686534882,60,3,2,206,
4359,722,2010-10-20,4,3,0,25.3333339691162,34.6666679382324,0,60,3,2,206,67,0,1,1,1,1,12,110
4360,724,2010-10-20,1,3,0,12,38,4,54,2,2,206,66,0,1,1,1,1,11,11237.29409,8012.061644,8.961519
4361,724,2010-10-20,2,3,0,20.6666660308838,39,0.333333343267441,60,3,2,206,66,0,1,1,1,1,11,1
4362,724,2010-10-20,3,3,0,25,23,12,60,1,2,206,66,0,1,1,1,1,11,11237.29409,8012.061644,8.96151
4363,724,2010-10-27,1,3,0,9,18,0,27,1,2,206,66,0,1,1,1,1,11,11237.29409,8012.061644,8.9615197
4364,724,2010-10-27,2,3,0,14,44.6666679382324,1.33333337306976,60,3,2,206,66,0,1,1,1,1,11,11
4365,724,2010-10-27,3,3,0,17.6000003814697,27.2000007629395,15.1999998092651,60,3,2,206,6
4366,724,2010-10-27,4,3,0,34.8333320617676,20.8333339691162,4.33333349227905,60,3,2,206,6
4367,724,2010-11-01,1,1,0,5.5,21,5,31.5,2,2,206,66,0,1,1,1,1,11,11237.29409,8012.061644,8.9615
4368,724,2010-11-01,2,1,0,12.6666669845581,41.3333320617676,6,60,3,2,206,66,0,1,1,1,1,11,112
4369,724,2010-11-01,3,1,0,25.2000007629395,26,8.80000019073486,60,3,2,206,66,0,1,1,1,1,11,11
4370,724,2010-11-01,4,1,0,31.6000003814697,27,1.39999997615814,60,3,2,206,66,0,1,1,1,1,11,11
4371,724,2010-11-02,0,2,0,12.5,18,3,33.5,2,2,206,66,0,1,1,1,1,11,11237.29409,8012.061644,8.961
4372,724,2010-11-02,1,2,0,23.3999996185303,27.3999996185303,9.19999980926514,60,3,2,206,6
4373,724,2010-11-02,2,2,0,32.3333320617676,23.6666660308838,4,60,3,2,206,66,0,1,1,1,1,11,112
4374,724,2010-11-02,3,2,0,18.3333339691162,33,8.66666698455811,60,3,2,206,66,0,1,1,1,1,11,11
4375,724,2010-11-02,4,2,0,32.5,22.5,5,60,3,2,206,66,0,1,1,1,1,11,11237.29409,8012.061644,8.961
4376,724,2010-11-03,1,3,0,25.5,26.5,5,57,3,2,206,66,0,1,1,1,1,11,11237.29409,8012.061644,8.961
4377,724,2010-11-03,2,3,0,31,27.5,1.5,60,3,2,206,66,0,1,1,1,1,11,11237.29409,8012.061644,8.961
4378,724,2010-11-03,3,3,0,23.5,34,2.5,60,3,2,206,66,0,1,1,1,1,11,11237.29409,8012.061644,8.961
4379,724,2010-11-03,4,3,0,34.6666679382324,25,0.333333343267441,60,3,2,206,66,0,1,1,1,1,11,1
4380,736,2010-10-14,4,4,0,45.5,8,0.5,54,2,2,206,69,1,1,1,1,1,12,11113.2294,8146.685575,10.9385
4381,736,2010-10-27,0,3,0,9,29,3,41,1,2,206,69,1,1,1,1,1,12,11113.2294,8146.685575,10.9385100
4382,736,2010-10-28,1,4,0,24,25.8333339691162,1.33333337306976,51.1666679382324,3,2,206,6
4383,736,2010-10-28,2,4,0,29,29.6666660308838,1.33333337306976,60,3,2,206,69,1,1,1,1,1,12,11
4384,736,2010-10-28,3,4,0,27.6666660308838,29.3333339691162,3,60,3,2,206,69,1,1,1,1,1,12,111
4385,736,2010-10-28,4,4,0,37,17,0.5,54.5,2,2,206,69,1,1,1,1,1,12,11113.2294,8146.685575,10.938
4386,736,2010-10-29,0,5,0,51,8,1,60,1,2,206,69,1,1,1,1,1,12,11113.2294,8146.685575,10.9385100
4387,736,2010-10-29,1,5,0,13,16.5,2,31.5,2,2,206,69,1,1,1,1,1,12,11113.2294,8146.685575,10.938
4388,736,2010-10-29,2,5,0,17.6666660308838,34.6666679382324,7.66666650772095,60,3,2,206,6
4389,736,2010-10-29,3,5,0,23,36.1666679382324,0.833333313465118,60,3,2,206,69,1,1,1,1,1,12,1
4390,736,2010-10-29,4,5,0,32.2000007629395,26.7999992370605,0,59,3,2,206,69,1,1,1,1,1,12,111
4391,736,2010-10-30,1,6,1,19.3333339691162,30.1666660308838,5.33333349227905,54.833332
4392,736,2010-10-30,2,6,1,18.8333339691162,35.6666679382324,5.5,60,3,2,206,69,1,1,1,1,1,12,1
4393,736,2010-10-30,3,6,1,0,3,4,7,1,2,206,69,1,1,1,1,1,12,11113.2294,8146.685575,10.93851009,1
4394,736,2010-10-30,4,6,1,32.6666679382324,16.3333339691162,0.333333343267441,49.333332
4395,736,2010-10-31,1,0,1,14.6666669845581,32,4.33333349227905,51,3,2,206,69,1,1,1,1,1,12,11
4396,736,2010-10-31,2,0,1,11,42.3333320617676,6.66666650772095,60,3,2,206,69,1,1,1,1,1,12,11
4397,736,2010-10-31,3,0,1,24.5,33,2.5,60,3,2,206,69,1,1,1,1,1,12,11113.2294,8146.685575,10.938
4398,736,2010-10-31,4,0,1,45.5,13.5,1,60,3,2,206,69,1,1,1,1,1,12,11113.2294,8146.685575,10.938
4399,739,2010-10-14,0,4,0,4,0.5,0,4.5,2,2,206,74,0,1,0,1,1,11,9447.379463,7203.209823,10.05212

4400,739,2010-10-14,1,4,0,43.3333320617676,15.3333330154419,1.33333337306976,60,3,2,206,7
4401,739,2010-10-14,2,4,0,21.3333339691162,33.6666679382324,5,60,3,2,206,74,0,1,0,1,1,11,944
4402,739,2010-10-14,3,4,0,39.3333320617676,19.3333339691162,1.33333337306976,60,3,2,206,7
4403,739,2010-10-14,4,4,0,32.6666679382324,26.3333339691162,1,60,3,2,206,74,0,1,0,1,1,11,944
4404,739,2010-10-15,1,5,0,28.8333339691162,16.1666660308838,1.83333337306976,46.833332
4405,739,2010-10-15,2,5,0,34.25,23.75,2,60,3,2,206,74,0,1,0,1,1,11,9447.379463,7203.209823,10.
4406,739,2010-10-15,3,5,0,26,32,2,60,3,2,206,74,0,1,0,1,1,11,9447.379463,7203.209823,10.05212
4407,739,2010-10-15,4,5,0,38.6666679382324,19,2.33333325386047,60,3,2,206,74,0,1,0,1,1,11,94
4408,739,2010-10-16,1,6,1,40.5,10.5,1.5,52.5,2,2,206,74,0,1,0,1,1,11,9447.379463,7203.209823,10
4409,739,2010-10-16,2,6,1,29.7999992370605,25.6000003814697,4.59999990463257,60,3,2,206,7
4410,739,2010-10-16,3,6,1,30,27.3999996185303,2.59999990463257,60,3,2,206,74,0,1,0,1,1,11,94
4411,739,2010-10-16,4,6,1,34.6666679382324,24,1.33333337306976,60,3,2,206,74,0,1,0,1,1,11,94
4412,739,2010-10-17,1,0,1,16,10,14,40,1,2,206,74,0,1,0,1,1,11,9447.379463,7203.209823,10.0521
4413,739,2010-10-17,2,0,1,24.3333339691162,29,6.66666650772095,60,3,2,206,74,0,1,0,1,1,11,94
4414,739,2010-10-17,3,0,1,34,24.75,1.25,60,3,2,206,74,0,1,0,1,1,11,9447.379463,7203.209823,10.
4415,739,2010-10-17,4,0,1,41.2000007629395,17.6000003814697,1.20000004768372,60,3,2,206,7
4416,739,2010-10-18,0,1,0,10.5,10.5,4,25,2,2,206,74,0,1,0,1,1,11,9447.379463,7203.209823,10.05
4417,739,2010-10-18,1,1,0,29.6666660308838,26,4.33333349227905,60,3,2,206,74,0,1,0,1,1,11,94
4418,739,2010-10-18,2,1,0,33.6666679382324,22.6666660308838,3.66666674613953,60,3,2,206,7
4419,739,2010-10-18,3,1,0,15,39.6666679382324,5.33333349227905,60,3,2,206,74,0,1,0,1,1,11,94
4420,739,2010-10-18,4,1,0,29.25,29,1.75,60,3,2,206,74,0,1,0,1,1,11,9447.379463,7203.209823,10.
4421,739,2010-10-19,0,2,0,56,3,1,60,1,2,206,74,0,1,0,1,1,11,9447.379463,7203.209823,10.052120
4422,739,2010-10-19,1,2,0,41.1666679382324,16.6666660308838,2.16666674613953,60,3,2,206,7
4423,739,2010-10-19,2,2,0,25,30.3333339691162,4.66666650772095,60,3,2,206,74,0,1,0,1,1,11,94
4424,739,2010-10-19,3,2,0,25.6666660308838,30.6666660308838,3.66666674613953,60,3,2,206,7
4425,739,2010-10-19,4,2,0,25.6666660308838,30.6666660308838,3.66666674613953,60,3,2,206,7
4426,739,2010-10-20,2,3,0,33.3333320617676,23.6666660308838,3,60,3,2,206,74,0,1,0,1,1,11,944
4427,739,2010-10-20,3,3,0,27.3333339691162,27.3333339691162,5.33333349227905,60,3,2,206,7
4428,739,2010-10-20,4,3,0,32,25,3,60,3,2,206,74,0,1,0,1,1,11,9447.379463,7203.209823,10.05212
4429,750,2010-10-16,0,6,1,22,9.5,0.5,32,2,2,216,71,0,1,0,1,1,10,10471.981,8368.769594,4.917849
4430,750,2010-10-16,1,6,1,24.6666660308838,32.3333320617676,3,60,3,2,216,71,0,1,0,1,1,10,104
4431,750,2010-10-16,2,6,1,35.2000007629395,24.2000007629395,0.600000023841858,60,3,2,216,
4432,750,2010-10-16,3,6,1,18.3333339691162,34.6666679382324,7,60,3,2,216,71,0,1,0,1,1,10,104
4433,750,2010-10-16,4,6,1,27.3333339691162,27.6666660308838,5,60,3,2,216,71,0,1,0,1,1,10,104
4434,750,2010-10-17,1,0,1,31.6666660308838,23.6666660308838,0.666666686534882,56,3,2,216,
4435,750,2010-10-17,2,0,1,36.75,23,0.25,60,3,2,216,71,0,1,0,1,1,10,10471.981,8368.769594,4.917
4436,750,2010-10-17,3,0,1,28,29,3,60,3,2,216,71,0,1,0,1,1,10,10471.981,8368.769594,4.9178496,1
4437,750,2010-10-17,4,0,1,40.4000015258789,18.6000003814697,1,60,3,2,216,71,0,1,0,1,1,10,104
4438,750,2010-10-18,0,1,0,4,5,0,9,1,2,216,71,0,1,0,1,1,10,10471.981,8368.769594,4.9178496,12.0
4439,750,2010-10-18,1,1,0,29.1666660308838,27.1666660308838,1.83333337306976,58.1666679
4440,750,2010-10-18,2,1,0,44.4000015258789,15.3999996185303,0.200000002980232,60,3,2,216,
4441,750,2010-10-18,3,1,0,28,31.3333339691162,0.666666686534882,60,3,2,216,71,0,1,0,1,1,10,1
4442,750,2010-10-18,4,1,0,39,20,1,60,3,2,216,71,0,1,0,1,1,10,10471.981,8368.769594,4.9178496,1
4443,750,2010-10-19,1,2,0,33.3333320617676,17.6666660308838,0.666666686534882,51.666667
4444,750,2010-10-19,2,2,0,48.75,11,0.25,60,3,2,216,71,0,1,0,1,1,10,10471.981,8368.769594,4.917
4445,750,2010-10-19,3,2,0,26.6666660308838,31.1666660308838,2.16666674613953,60,3,2,216,7
4446,750,2010-10-19,4,2,0,33.4000015258789,25.6000003814697,1,60,3,2,216,71,0,1,0,1,1,10,104
4447,750,2010-10-20,0,3,0,37.5,14.5,4,56,2,2,216,71,0,1,0,1,1,10,10471.981,8368.769594,4.91784
4448,750,2010-10-20,1,3,0,7.66666650772095,34.6666679382324,17.6666660308838,60,3,2,216,7
4449,750,2010-10-20,2,3,0,35,22.3333339691162,2.66666674613953,60,3,2,216,71,0,1,0,1,1,10,10

4450,750,2010-10-20,3,3,0,31,28.6666660308838,0.333333343267441,60,3,2,216,71,0,1,0,1,1,10,1
4451,750,2010-10-20,4,3,0,34.5,25.5,0,60,3,2,216,71,0,1,0,1,1,10,10471.981,8368.769594,4.91784
4452,751,2010-10-20,1,3,0,21,11.5,0.5,33,2,2,216,71,1,1,0,1,1,12,10255.35641,7919.353612,6.062
4453,751,2010-10-21,0,4,0,22,19,0,41,1,2,216,71,1,1,0,1,1,12,10255.35641,7919.353612,6.062603
4454,751,2010-10-21,1,4,0,21.25,34.25,4.5,60,3,2,216,71,1,1,0,1,1,12,10255.35641,7919.353612,6.
4455,751,2010-10-21,2,4,0,8,47,5,60,3,2,216,71,1,1,0,1,1,12,10255.35641,7919.353612,6.0626038
4456,751,2010-10-21,3,4,0,14.25,39,6.75,60,3,2,216,71,1,1,0,1,1,12,10255.35641,7919.353612,6.06
4457,751,2010-10-21,4,4,0,32.6666679382324,26.3333339691162,1,60,3,2,216,71,1,1,0,1,1,12,102
4458,751,2010-10-22,0,5,0,15,16,1,32,1,2,216,71,1,1,0,1,1,12,10255.35641,7919.353612,6.062603
4459,751,2010-10-22,1,5,0,19.3333339691162,40.3333320617676,0.333333343267441,60,3,2,216,
4460,751,2010-10-22,2,5,0,9.33333301544189,50.3333320617676,0.333333343267441,60,3,2,216,
4461,751,2010-10-22,3,5,0,5.19999980926514,44.5999984741211,10.1999998092651,60,3,2,216,7
4462,751,2010-10-22,4,5,0,6.16666650772095,49.5,4.33333349227905,60,3,2,216,71,1,1,0,1,1,12,1
4463,751,2010-10-23,0,6,1,0,3,1,4,1,2,216,71,1,1,0,1,1,12,10255.35641,7919.353612,6.062603857,
4464,751,2010-10-23,2,6,1,17.3999996185303,31.2000007629395,0,48.5999984741211,3,2,216,71
4465,751,2010-10-23,3,6,1,23.6666660308838,35.3333320617676,1,60,3,2,216,71,1,1,0,1,1,12,102
4466,751,2010-10-23,4,6,1,11,48.3333320617676,0.666666686534882,60,3,2,216,71,1,1,0,1,1,12,1
4467,751,2010-10-24,0,0,1,34,26,0,60,1,2,216,71,1,1,0,1,1,12,10255.35641,7919.353612,6.062603
4468,751,2010-10-24,1,0,1,3.66666674613953,52.6666679382324,3.66666674613953,60,3,2,216,7
4469,751,2010-10-24,2,0,1,5,35,10,50,3,2,216,71,1,1,0,1,1,12,10255.35641,7919.353612,6.062603
4470,751,2010-10-24,3,0,1,5.33333349227905,42.1666679382324,12.5,60,3,2,216,71,1,1,0,1,1,12,1
4471,751,2010-10-24,4,0,1,24.75,34.75,0.5,60,3,2,216,71,1,1,0,1,1,12,10255.35641,7919.353612,6.
4472,751,2010-10-25,4,1,0,13,43.5,3.5,60,2,2,216,71,1,1,0,1,1,12,10255.35641,7919.353612,6.062
4473,753,2012-07-11,0,3,0,60,0,0,60,1,2,216,69,0,1,0,1,1,12,10125.09005,8010.835743,6.5584732
4474,753,2012-07-11,1,3,0,18.3333339691162,40.6666679382324,1,60,3,2,216,69,0,1,0,1,1,12,101
4475,753,2012-07-11,2,3,0,23.3333339691162,32,4.66666650772095,60,3,2,216,69,0,1,0,1,1,12,10
4476,753,2012-07-11,3,3,0,14,39,7,60,3,2,216,69,0,1,0,1,1,12,10125.09005,8010.835743,6.558473
4477,753,2012-07-11,4,3,0,30.6666660308838,25.6666660308838,3.66666674613953,60,3,2,216,6
4478,753,2012-07-12,0,4,0,0,0.333333343267441,0,0.333333343267441,3,2,216,69,0,1,0,1,1,12,10
4479,753,2012-07-12,1,4,0,24.6666660308838,21,3,48.6666679382324,3,2,216,69,0,1,0,1,1,12,101
4480,753,2012-07-12,2,4,0,30,29.3333339691162,0.666666686534882,60,3,2,216,69,0,1,0,1,1,12,1
4481,753,2012-07-12,3,4,0,41.6666679382324,16.3333339691162,2,60,3,2,216,69,0,1,0,1,1,12,101
4482,753,2012-07-12,4,4,0,22,35,3,60,3,2,216,69,0,1,0,1,1,12,10125.09005,8010.835743,6.558473
4483,753,2012-07-13,0,5,0,0,0.333333343267441,0,0.333333343267441,3,2,216,69,0,1,0,1,1,12,10
4484,753,2012-07-13,1,5,0,14.3333330154419,37.3333320617676,8,59.6666679382324,3,2,216,69
4485,753,2012-07-13,2,5,0,32,25.6666660308838,2.33333325386047,60,3,2,216,69,0,1,0,1,1,12,10
4486,753,2012-07-13,3,5,0,31,26.6666660308838,2.33333325386047,60,3,2,216,69,0,1,0,1,1,12,10
4487,753,2012-07-13,4,5,0,19.3333339691162,36,4.66666650772095,60,3,2,216,69,0,1,0,1,1,12,10
4488,753,2012-07-14,1,6,1,21.6666660308838,11,2.66666674613953,35.3333320617676,3,2,216,6
4489,753,2012-07-14,2,6,1,19.3333339691162,30.6666660308838,10,60,3,2,216,69,0,1,0,1,1,12,10
4490,753,2012-07-14,3,6,1,50.3333320617676,9.33333301544189,0.333333343267441,60,3,2,216,
4491,753,2012-07-14,4,6,1,37.3333320617676,18.3333339691162,4.33333349227905,60,3,2,216,6
4492,753,2012-07-16,1,1,0,34,14.6666669845581,1.66666662693024,50.3333320617676,3,2,216,6
4493,753,2012-07-16,2,1,0,45,11.3333330154419,3.66666674613953,60,3,2,216,69,0,1,0,1,1,12,10
4494,753,2012-07-16,3,1,0,46.3333320617676,13.3333330154419,0.333333343267441,60,3,2,216,
4495,753,2012-07-16,4,1,0,45.3333320617676,12.3333330154419,2.33333325386047,60,3,2,216,6
4496,753,2012-07-17,1,2,0,11.3333330154419,8,2.33333325386047,21.6666660308838,3,2,216,69
4497,753,2012-07-17,2,2,0,46.3333320617676,13,0.666666686534882,60,3,2,216,69,0,1,0,1,1,12,1
4498,753,2012-07-17,3,2,0,43,15,2,60,3,2,216,69,0,1,0,1,1,12,10125.09005,8010.835743,6.558473
4499,753,2012-07-17,4,2,0,40.3333320617676,18.6666660308838,1,60,3,2,216,69,0,1,0,1,1,12,101

4500,753,2012-07-18,1,3,0,12,8,2,22,3,2,216,69,0,1,0,1,1,12,10125.09005,8010.835743,6.55847327
4501,753,2012-07-18,2,3,0,29.3333339691162,26.3333339691162,4.33333349227905,60,3,2,216,6
4502,753,2012-07-18,3,3,0,49,9,2,60,3,2,216,69,0,1,0,1,1,12,10125.09005,8010.835743,6.55847327
4503,753,2012-07-18,4,3,0,45.6666679382324,13.3333330154419,1,60,3,2,216,69,0,1,0,1,1,12,101
4504,753,2012-07-22,1,7,1,23,6.33333349227905,2.33333325386047,31.6666660308838,3,2,216,6
4505,753,2012-07-22,2,7,1,19.3333339691162,36.6666679382324,4,60,3,2,216,69,0,1,0,1,1,12,101
4506,753,2012-07-22,3,7,1,39.6666679382324,20,0.333333343267441,60,3,2,216,69,0,1,0,1,1,12,1
4507,753,2012-07-22,4,7,1,43.3333320617676,15,1.66666662693024,60,3,2,216,69,0,1,0,1,1,12,10
4508,753,2012-07-26,0,4,0,8,8,5,21,3,2,216,69,0,1,0,1,1,12,10125.09005,8010.835743,6.558473276
4509,753,2012-07-26,1,4,0,31,18.3333339691162,10.6666669845581,60,3,2,216,69,0,1,0,1,1,12,10
4510,753,2012-07-26,2,4,0,42,9.33333301544189,8.66666698455811,60,3,2,216,69,0,1,0,1,1,12,10
4511,753,2012-07-26,3,4,0,32.6666679382324,20.6666660308838,6.66666650772095,60,3,2,216,6
4512,753,2012-07-26,4,4,0,7,3.33333325386047,3.33333325386047,13.6666669845581,3,2,216,69
4513,753,2012-07-30,0,1,0,17,2.33333325386047,5.66666650772095,25,3,2,216,69,0,1,0,1,1,12,10
4514,753,2012-07-30,1,1,0,39,15,6,60,3,2,216,69,0,1,0,1,1,12,10125.09005,8010.835743,6.5584732
4515,753,2012-07-30,2,1,0,40.3333320617676,14.3333330154419,5.33333349227905,60,3,2,216,6
4516,753,2012-07-30,3,1,0,38,8.66666698455811,3,49.6666679382324,3,2,216,69,0,1,0,1,1,12,101
4517,753,2012-07-30,4,1,0,4.66666650772095,0.666666686534882,0,5.33333349227905,3,2,216,6
4518,755,2012-07-13,0,5,0,22,38,0,60,1,2,216,78,1,1,0,1,1,11,10383.68808,8008.09681,4.69310631
4519,755,2012-07-13,1,5,0,22.6666660308838,30.3333339691162,7,60,3,2,216,78,1,1,0,1,1,11,103
4520,755,2012-07-13,2,5,0,27,28.3333339691162,4.66666650772095,60,3,2,216,78,1,1,0,1,1,11,10
4521,755,2012-07-13,3,5,0,42,9.66666698455811,8.33333301544189,60,3,2,216,78,1,1,0,1,1,11,10
4522,755,2012-07-13,4,5,0,29,13.6666669845581,8,50.6666679382324,3,2,216,78,1,1,0,1,1,11,103
4523,755,2012-07-14,0,6,1,0,0.333333343267441,0.333333343267441,0.666666686534882,3,2,216
4524,755,2012-07-14,1,6,1,22.6666660308838,34,3.33333325386047,60,3,2,216,78,1,1,0,1,1,11,10
4525,755,2012-07-14,2,6,1,14.6666669845581,45,0.333333343267441,60,3,2,216,78,1,1,0,1,1,11,1
4526,755,2012-07-14,3,6,1,16.6666660308838,37,6.33333349227905,60,3,2,216,78,1,1,0,1,1,11,10
4527,755,2012-07-14,4,6,1,22.3333339691162,21,9.66666698455811,53,3,2,216,78,1,1,0,1,1,11,10
4528,755,2012-07-15,0,7,1,9.33333301544189,15.3333330154419,1.33333337306976,26,3,2,216,7
4529,755,2012-07-15,1,7,1,19,38.3333320617676,2.66666674613953,60,3,2,216,78,1,1,0,1,1,11,10
4530,755,2012-07-15,2,7,1,34.6666679382324,21,4.33333349227905,60,3,2,216,78,1,1,0,1,1,11,10
4531,755,2012-07-15,3,7,1,25.6666660308838,32,2.33333325386047,60,3,2,216,78,1,1,0,1,1,11,10
4532,755,2012-07-15,4,7,1,40.3333320617676,19,0.666666686534882,60,3,2,216,78,1,1,0,1,1,11,1
4533,755,2012-07-16,0,1,0,3.33333325386047,8.33333301544189,8.66666698455811,20.3333339
4534,755,2012-07-16,1,1,0,4.33333349227905,40.6666679382324,15,60,3,2,216,78,1,1,0,1,1,11,10
4535,755,2012-07-16,2,1,0,27.3333339691162,25,7.66666650772095,60,3,2,216,78,1,1,0,1,1,11,10
4536,755,2012-07-16,3,1,0,39,13,8,60,3,2,216,78,1,1,0,1,1,11,10383.68808,8008.09681,4.69310631
4537,755,2012-07-16,4,1,0,49.6666679382324,6.66666650772095,3.66666674613953,60,3,2,216,7
4538,755,2012-07-17,0,2,0,4.33333349227905,3.33333325386047,0,7.66666650772095,3,2,216,78
4539,755,2012-07-17,1,2,0,30.6666660308838,27.6666660308838,1.66666662693024,60,3,2,216,7
4540,755,2012-07-17,2,2,0,30.3333339691162,22.3333339691162,7.33333349227905,60,3,2,216,7
4541,755,2012-07-17,3,2,0,31.6666660308838,20.3333339691162,8,60,3,2,216,78,1,1,0,1,1,11,103
4542,755,2012-07-17,4,2,0,38,16,3,57,3,2,216,78,1,1,0,1,1,11,10383.68808,8008.09681,4.69310631
4543,755,2012-07-18,0,3,0,14.3333330154419,5,0.333333343267441,19.6666660308838,3,2,216,7
4544,755,2012-07-18,1,3,0,22.3333339691162,37.3333320617676,0.333333343267441,60,3,2,216,
4545,755,2012-07-18,2,3,0,38.6666679382324,19.6666660308838,1.66666662693024,60,3,2,216,7
4546,755,2012-07-18,3,3,0,28.6666660308838,28.3333339691162,3,60,3,2,216,78,1,1,0,1,1,11,103
4547,755,2012-07-18,4,3,0,43.6666679382324,15.6666669845581,0.666666686534882,60,3,2,216,
4548,755,2012-07-19,0,4,0,3.33333325386047,2.66666674613953,0,6,3,2,216,78,1,1,0,1,1,11,1038
4549,755,2012-07-19,1,4,0,31.6666660308838,27.3333339691162,1,60,3,2,216,78,1,1,0,1,1,11,103

4550,755,2012-07-19,2,4,0,40,20,0,60,3,2,216,78,1,1,0,1,1,11,10383.68808,8008.09681,4.69310631
4551,755,2012-07-19,3,4,0,16.3333339691162,40.3333320617676,3.33333325386047,60,3,2,216,7
4552,755,2012-07-19,4,4,0,15.3333330154419,11.6666669845581,0.666666686534882,27.666666
4553,755,2012-07-20,0,5,0,3.33333325386047,0,0,3.33333325386047,3,2,216,78,1,1,0,1,1,11,1038
4554,755,2012-07-20,1,5,0,30,28.6666660308838,1.33333337306976,60,3,2,216,78,1,1,0,1,1,11,10
4555,755,2012-07-20,2,5,0,42,17.3333339691162,0.666666686534882,60,3,2,216,78,1,1,0,1,1,11,1
4556,755,2012-07-20,3,5,0,38,10.6666669845581,11.3333330154419,60,3,2,216,78,1,1,0,1,1,11,10
4557,755,2012-07-20,4,5,0,27,15,7,49,3,2,216,78,1,1,0,1,1,11,10383.68808,8008.09681,4.69310631
4558,755,2012-07-21,1,6,1,12,41,5.66666650772095,58.6666679382324,3,2,216,78,1,1,0,1,1,11,10
4559,755,2012-07-21,2,6,1,47,13,0,60,3,2,216,78,1,1,0,1,1,11,10383.68808,8008.09681,4.69310631
4560,755,2012-07-21,3,6,1,36.6666679382324,15.6666669845581,7.66666650772095,60,3,2,216,7
4561,755,2012-07-21,4,6,1,39.6666679382324,13.6666669845581,6.66666650772095,60,3,2,216,7
4562,763,2010-10-25,1,1,0,7,29,4.66666650772095,40.6666679382324,3,2,216,69,0,1,0,1,1,11,697
4563,763,2010-10-25,2,1,0,35.6666679382324,22.3333339691162,2,60,3,2,216,69,0,1,0,1,1,11,697
4564,763,2010-10-25,3,1,0,16,26.3333339691162,17.6666660308838,60,3,2,216,69,0,1,0,1,1,11,69
4565,763,2010-10-25,4,1,0,44.5,14,1.5,60,2,2,216,69,0,1,0,1,1,11,6973.318114,5769.177813,5.458
4566,763,2010-10-26,1,2,0,7,44,4,55,3,2,216,69,0,1,0,1,1,11,6973.318114,5769.177813,5.4583183
4567,763,2010-10-26,2,2,0,9,48,3,60,3,2,216,69,0,1,0,1,1,11,6973.318114,5769.177813,5.4583183
4568,763,2010-10-26,3,2,0,23,28,9,60,3,2,216,69,0,1,0,1,1,11,6973.318114,5769.177813,5.458318
4569,763,2010-10-26,4,2,0,35,11.75,0.75,47.5,3,2,216,69,0,1,0,1,1,11,6973.318114,5769.177813,5.
4570,763,2010-11-09,0,2,0,0,0,1,1,1,2,216,69,0,1,0,1,1,11,6973.318114,5769.177813,5.458318323,
4571,763,2010-11-09,1,2,0,21.5,32.6666679382324,2.5,56.6666679382324,3,2,216,69,0,1,0,1,1,11,
4572,763,2010-11-09,2,2,0,16.6666660308838,43,0.333333343267441,60,3,2,216,69,0,1,0,1,1,11,6
4573,763,2010-11-09,3,2,0,24,34.6666679382324,1.33333337306976,60,3,2,216,69,0,1,0,1,1,11,69
4574,763,2010-11-09,4,2,0,25.6666660308838,29,5.33333349227905,60,3,2,216,69,0,1,0,1,1,11,69
4575,763,2010-11-10,0,3,0,15,12,8,35,1,2,216,69,0,1,0,1,1,11,6973.318114,5769.177813,5.458318
4576,763,2010-11-10,1,3,0,4.33333349227905,36,19.6666660308838,60,3,2,216,69,0,1,0,1,1,11,69
4577,763,2010-11-10,2,3,0,16.6666660308838,35.5,7.83333349227905,60,3,2,216,69,0,1,0,1,1,11,
4578,763,2010-11-10,3,3,0,13.25,31.75,15,60,3,2,216,69,0,1,0,1,1,11,6973.318114,5769.177813,5.
4579,763,2010-11-10,4,3,0,29.3333339691162,24.6666660308838,6,60,3,2,216,69,0,1,0,1,1,11,697
4580,763,2010-11-11,0,4,0,0,2,0.5,2.5,2,2,216,69,0,1,0,1,1,11,6973.318114,5769.177813,5.458318
4581,763,2010-11-11,1,4,0,14.6666669845581,39.6666679382324,5.66666650772095,60,3,2,216,6
4582,763,2010-11-11,2,4,0,29.6666660308838,30.3333339691162,0,60,3,2,216,69,0,1,0,1,1,11,697
4583,763,2010-11-11,3,4,0,26.7999992370605,23,10.1999998092651,60,3,2,216,69,0,1,0,1,1,11,69
4584,763,2010-11-11,4,4,0,33.6666679382324,22.3333339691162,4,60,3,2,216,69,0,1,0,1,1,11,697
4585,763,2010-11-12,0,5,0,7,4,7,18,1,2,216,69,0,1,0,1,1,11,6973.318114,5769.177813,5.45831832
4586,763,2010-11-12,1,5,0,21.6666660308838,31,7.33333349227905,60,3,2,216,69,0,1,0,1,1,11,69
4587,763,2010-11-12,2,5,0,31,28.6666660308838,0.333333343267441,60,3,2,216,69,0,1,0,1,1,11,6
4588,763,2010-11-12,3,5,0,25.3333339691162,31.6666660308838,3,60,3,2,216,69,0,1,0,1,1,11,697
4589,763,2010-11-12,4,5,0,34.8333320617676,22.8333339691162,2.33333325386047,60,3,2,216,6
4590,765,2010-11-12,1,5,0,3,7,4,14,1,2,216,69,1,1,0,1,1,12,6973.318114,5769.177813,5.45831832
4591,765,2010-11-13,1,6,1,9,32.5999984741211,15.3999996185303,57,3,2,216,69,1,1,0,1,1,12,697
4592,765,2010-11-13,2,6,1,19,34.4000015258789,6.59999990463257,60,3,2,216,69,1,1,0,1,1,12,69
4593,765,2010-11-13,3,6,1,9.66666698455811,31.6666660308838,18.6666660308838,60,3,2,216,6
4594,765,2010-11-13,4,6,1,37,9.33333301544189,0.666666686534882,47,3,2,216,69,1,1,0,1,1,12,6
4595,765,2010-11-14,1,0,1,9.66666698455811,44,1.66666662693024,55.3333320617676,3,2,216,6
4596,765,2010-11-14,2,0,1,12.75,45.75,1.5,60,3,2,216,69,1,1,0,1,1,12,6973.318114,5769.177813,5.
4597,765,2010-11-14,3,0,1,22,36,2,60,3,2,216,69,1,1,0,1,1,12,6973.318114,5769.177813,5.458318
4598,765,2010-11-14,4,0,1,35.75,19.75,2.5,58,3,2,216,69,1,1,0,1,1,12,6973.318114,5769.177813,5.
4599,765,2010-11-15,0,1,0,0,18,2,20,1,2,216,69,1,1,0,1,1,12,6973.318114,5769.177813,5.4583183

4600,765,2010-11-15,1,1,0,12.75,41.25,3.75,57.75,3,2,216,69,1,1,0,1,1,12,6973.318114,5769.17781
4601,765,2010-11-15,2,1,0,16.6666660308838,40,3.33333325386047,60,3,2,216,69,1,1,0,1,1,12,69
4602,765,2010-11-15,3,1,0,13,37,10,60,3,2,216,69,1,1,0,1,1,12,6973.318114,5769.177813,5.458318
4603,765,2010-11-15,4,1,0,25.6000003814697,32.5999984741211,1.79999995231628,60,3,2,216,6
4604,765,2010-11-16,1,2,0,26,28.7999992370605,4.80000019073486,59.5999984741211,3,2,216,6
4605,765,2010-11-16,2,2,0,32.8333320617676,26.3333339691162,0.833333313465118,60,3,2,216,
4606,765,2010-11-16,3,2,0,31.6666660308838,22.6666660308838,5.66666650772095,60,3,2,216,6
4607,765,2010-11-16,4,2,0,28,30.3333339691162,1.66666662693024,60,3,2,216,69,1,1,0,1,1,12,69
4608,765,2010-11-17,0,3,0,2,14,2,18,1,2,216,69,1,1,0,1,1,12,6973.318114,5769.177813,5.4583183
4609,765,2010-11-17,1,3,0,11.6666669845581,45,3.33333325386047,60,3,2,216,69,1,1,0,1,1,12,69
4610,765,2010-11-17,2,3,0,15.3333330154419,44,0.666666686534882,60,3,2,216,69,1,1,0,1,1,12,6
4611,765,2010-11-17,3,3,0,19.3333339691162,38,2.66666674613953,60,3,2,216,69,1,1,0,1,1,12,69
4612,765,2010-11-17,4,3,0,36,23.6666660308838,0.333333343267441,60,3,2,216,69,1,1,0,1,1,12,6
4613,765,2010-11-18,4,4,0,26,34,0,60,3,2,216,69,1,1,0,1,1,12,6973.318114,5769.177813,5.458318
4614,769,2012-08-04,1,6,1,47,0.666666686534882,1.33333337306976,49,3,2,216,70,0,1,0,1,1,12,8
4615,769,2012-08-04,2,6,1,0.333333343267441,24,35.6666679382324,60,3,2,216,70,0,1,0,1,1,12,8
4616,769,2012-08-04,3,6,1,3.33333325386047,26.3333339691162,30.3333339691162,60,3,2,216,7
4617,769,2012-08-04,4,6,1,36.3333320617676,18.6666660308838,5,60,3,2,216,70,0,1,0,1,1,12,898
4618,769,2012-08-05,1,7,1,16.6666660308838,17.3333339691162,2.66666674613953,36.666679
4619,769,2012-08-05,2,7,1,23,31.6666660308838,5.33333349227905,60,3,2,216,70,0,1,0,1,1,12,89
4620,769,2012-08-05,3,7,1,20.3333339691162,33.6666679382324,6,60,3,2,216,70,0,1,0,1,1,12,898
4621,769,2012-08-05,4,7,1,22.6666660308838,32.6666679382324,4.66666650772095,60,3,2,216,7
4622,769,2012-08-06,1,1,0,17.3333339691162,9.33333301544189,4,30.6666660308838,3,2,216,70
4623,769,2012-08-06,2,1,0,21.3333339691162,33.6666679382324,5,60,3,2,216,70,0,1,0,1,1,12,898
4624,769,2012-08-06,3,1,0,41.3333320617676,17.6666660308838,1,60,3,2,216,70,0,1,0,1,1,12,898
4625,769,2012-08-06,4,1,0,21.3333339691162,28.3333339691162,10.3333330154419,60,3,2,216,7
4626,769,2012-08-07,0,2,0,0.666666686534882,5.66666650772095,1,7.33333349227905,3,2,216,7
4627,769,2012-08-07,1,2,0,27.3333339691162,31.3333339691162,1.33333337306976,60,3,2,216,7
4628,769,2012-08-07,2,2,0,23.3333339691162,25.6666660308838,11,60,3,2,216,70,0,1,0,1,1,12,89
4629,769,2012-08-07,3,2,0,18.3333339691162,26.3333339691162,15.3333330154419,60,3,2,216,7
4630,769,2012-08-07,4,2,0,39.6666679382324,17,3.33333325386047,60,3,2,216,70,0,1,0,1,1,12,89
4631,769,2012-08-08,1,3,0,1.33333337306976,7.66666650772095,2,11,3,2,216,70,0,1,0,1,1,12,898
4632,769,2012-08-08,2,3,0,19.3333339691162,38,2.66666674613953,60,3,2,216,70,0,1,0,1,1,12,89
4633,769,2012-08-08,3,3,0,17,39.3333320617676,3.66666674613953,60,3,2,216,70,0,1,0,1,1,12,89
4634,769,2012-08-08,4,3,0,26,32,2,60,3,2,216,70,0,1,0,1,1,12,8981.848715,6792.212536,5.541889
4635,769,2012-08-09,0,4,0,30.3333339691162,29,0.666666686534882,60,3,2,216,70,0,1,0,1,1,12,8
4636,769,2012-08-09,1,4,0,24.6666660308838,32,3.33333325386047,60,3,2,216,70,0,1,0,1,1,12,89
4637,769,2012-08-09,2,4,0,28.6666660308838,23.6666660308838,7.66666650772095,60,3,2,216,7
4638,769,2012-08-09,3,4,0,32,25.3333339691162,2.66666674613953,60,3,2,216,70,0,1,0,1,1,12,89
4639,769,2012-08-09,4,4,0,24,30.6666660308838,5.33333349227905,60,3,2,216,70,0,1,0,1,1,12,89
4640,769,2012-08-10,1,5,0,23.6666660308838,17,2,42.6666679382324,3,2,216,70,0,1,0,1,1,12,898
4641,769,2012-08-10,2,5,0,38.6666679382324,19.6666660308838,1.66666662693024,60,3,2,216,7
4642,769,2012-08-10,3,5,0,24.6666660308838,25.3333339691162,10,60,3,2,216,70,0,1,0,1,1,12,89
4643,769,2012-08-10,4,5,0,22.6666660308838,20,17.3333339691162,60,3,2,216,70,0,1,0,1,1,12,89
4644,775,2010-10-16,0,6,1,9,48,2,59,1,2,216,74,0,1,0,1,1,12,10401.12111,12174.39974,6.7147296
4645,775,2010-10-16,1,6,1,26.6000003814697,31.2000007629395,2.20000004768372,60,3,2,216,7
4646,775,2010-10-16,2,6,1,26.5,33.5,0,60,3,2,216,74,0,1,0,1,1,12,10401.12111,12174.39974,6.714
4647,775,2010-10-16,3,6,1,34.3333320617676,23.3333339691162,2.33333325386047,60,3,2,216,7
4648,775,2010-10-16,4,6,1,20.3333339691162,36,3.66666674613953,60,3,2,216,74,0,1,0,1,1,12,10
4649,775,2010-10-17,1,0,1,9,26.3333339691162,8.66666698455811,44,3,2,216,74,0,1,0,1,1,12,104

4650,775,2010-10-17,2,0,1,27.7999992370605,30.7999992370605,1.39999997615814,60,3,2,216,7
4651,775,2010-10-17,3,0,1,21.3333339691162,37,1.66666662693024,60,3,2,216,74,0,1,0,1,1,12,10
4652,775,2010-10-17,4,0,1,35.6666679382324,22.3333339691162,2,60,3,2,216,74,0,1,0,1,1,12,104
4653,775,2010-10-18,0,1,0,0,10,8,18,1,2,216,74,0,1,0,1,1,12,10401.12111,12174.39974,6.7147296
4654,775,2010-10-18,1,1,0,18,38,4,60,3,2,216,74,0,1,0,1,1,12,10401.12111,12174.39974,6.714729
4655,775,2010-10-18,2,1,0,24,35,1,60,3,2,216,74,0,1,0,1,1,12,10401.12111,12174.39974,6.714729
4656,775,2010-10-18,3,1,0,30.3333339691162,28.5,1.16666662693024,60,3,2,216,74,0,1,0,1,1,12,1
4657,775,2010-10-18,4,1,0,36.75,21.5,1.75,60,3,2,216,74,0,1,0,1,1,12,10401.12111,12174.39974,6.
4658,775,2010-10-19,1,2,0,21,34.5,1.25,56.75,3,2,216,74,0,1,0,1,1,12,10401.12111,12174.39974,6.
4659,775,2010-10-19,2,2,0,30,29.6666660308838,0.333333343267441,60,3,2,216,74,0,1,0,1,1,12,1
4660,775,2010-10-19,3,2,0,29,19.6666660308838,11.3333330154419,60,3,2,216,74,0,1,0,1,1,12,10
4661,775,2010-10-19,4,2,0,36.4000015258789,22.3999996185303,1.20000004768372,60,3,2,216,7
4662,775,2010-10-20,1,3,0,15.5,41.5,3,60,2,2,216,74,0,1,0,1,1,12,10401.12111,12174.39974,6.7147
4663,775,2010-10-20,2,3,0,31,27.6666660308838,1.33333337306976,60,3,2,216,74,0,1,0,1,1,12,10
4664,775,2010-10-20,3,3,0,24,35,1,60,3,2,216,74,0,1,0,1,1,12,10401.12111,12174.39974,6.714729
4665,775,2010-10-20,4,3,0,37.6666679382324,22,0.333333343267441,60,3,2,216,74,0,1,0,1,1,12,1
4666,775,2010-11-18,0,4,0,48,7,5,60,1,2,216,74,0,1,0,1,1,12,10401.12111,12174.39974,6.7147296
4667,775,2010-11-18,1,4,0,8.25,39.5,0.5,48.25,3,2,216,74,0,1,0,1,1,12,10401.12111,12174.39974,6
4668,775,2010-11-18,2,4,0,20.6666660308838,38.3333320617676,1,60,3,2,216,74,0,1,0,1,1,12,104
4669,775,2010-11-18,3,4,0,18.3333339691162,37.3333320617676,4.33333349227905,60,3,2,216,7
4670,775,2010-11-18,4,4,0,20.3333339691162,39.6666679382324,0,60,3,2,216,74,0,1,0,1,1,12,104
4671,776,2010-10-20,0,3,0,54,6,0,60,1,2,216,73,1,1,0,1,1,12,10401.12111,12174.39974,6.7147296
4672,776,2010-10-20,1,3,0,15.5,37.5,7,60,2,2,216,73,1,1,0,1,1,12,10401.12111,12174.39974,6.7147
4673,776,2010-10-21,1,4,0,17.8571434020996,26,6.42857122421265,50.2857131958008,3,2,216,7
4674,776,2010-10-21,2,4,0,37.4444427490234,21.4444446563721,1.11111116409302,60,3,2,216,7
4675,776,2010-10-21,3,4,0,38.3333320617676,18.8333339691162,2.83333325386047,60,3,2,216,7
4676,776,2010-10-21,4,4,0,42.3333320617676,17.3333339691162,0.333333343267441,60,3,2,216,
4677,776,2010-10-22,0,5,0,6,28,2.5,36.5,2,2,216,73,1,1,0,1,1,12,10401.12111,12174.39974,6.71472
4678,776,2010-10-22,1,5,0,24.1666660308838,34,0.666666686534882,58.8333320617676,3,2,216,
4679,776,2010-10-22,2,5,0,32.6666679382324,25,2.33333325386047,60,3,2,216,73,1,1,0,1,1,12,10
4680,776,2010-10-22,3,5,0,33.3333320617676,24.4444446563721,2.22222232818604,60,3,2,216,7
4681,776,2010-10-22,4,5,0,41.5,17.3333339691162,1.16666662693024,60,3,2,216,73,1,1,0,1,1,12,1
4682,776,2010-10-23,1,6,1,26,29.5,1.25,56.75,3,2,216,73,1,1,0,1,1,12,10401.12111,12174.39974,6.
4683,776,2010-10-23,2,6,1,41.3333320617676,18.3333339691162,0.333333343267441,60,3,2,216,
4684,776,2010-10-23,3,6,1,33.6666679382324,15,11.3333330154419,60,3,2,216,73,1,1,0,1,1,12,10
4685,776,2010-10-23,4,6,1,36.3333320617676,21.5,1.33333337306976,59.1666679382324,3,2,216
4686,776,2010-10-24,1,0,1,11.5,46.5,2,60,2,2,216,73,1,1,0,1,1,12,10401.12111,12174.39974,6.7147
4687,776,2010-10-24,2,0,1,23.6666660308838,34.3333320617676,2,60,3,2,216,73,1,1,0,1,1,12,104
4688,776,2010-10-24,3,0,1,27.3333339691162,32,0.666666686534882,60,3,2,216,73,1,1,0,1,1,12,1
4689,776,2010-10-24,4,0,1,37.3333320617676,22.6666660308838,0,60,3,2,216,73,1,1,0,1,1,12,104
4690,785,2010-10-20,0,3,0,14,43,0.5,57.5,2,2,216,70,1,1,0,1,1,11,7243.419952,4348.015304,6.2253
4691,785,2010-10-20,1,3,0,20.6000003814697,36.7999992370605,2.59999990463257,60,3,2,216,7
4692,785,2010-10-20,2,3,0,36,22,2,60,3,2,216,70,1,1,0,1,1,11,7243.419952,4348.015304,6.225363
4693,785,2010-10-20,3,3,0,34.3333320617676,24.6666660308838,1,60,3,2,216,70,1,1,0,1,1,11,724
4694,785,2010-10-20,4,3,0,45,15,0,60,3,2,216,70,1,1,0,1,1,11,7243.419952,4348.015304,6.225363
4695,785,2010-10-21,1,4,0,5,25.5,16.25,46.75,3,2,216,70,1,1,0,1,1,11,7243.419952,4348.015304,6.
4696,785,2010-10-21,2,4,0,22.8333339691162,30.6666660308838,6.5,60,3,2,216,70,1,1,0,1,1,11,72
4697,785,2010-10-21,3,4,0,16,35,9,60,3,2,216,70,1,1,0,1,1,11,7243.419952,4348.015304,6.225363
4698,785,2010-10-21,4,4,0,33,26.3333339691162,0.666666686534882,60,3,2,216,70,1,1,0,1,1,11,7
4699,785,2010-10-22,0,5,0,8,20,0,28,1,2,216,70,1,1,0,1,1,11,7243.419952,4348.015304,6.2253636

4700,785,2010-10-22,1,5,0,7.33333349227905,43,9.66666698455811,60,3,2,216,70,1,1,0,1,1,11,72
4701,785,2010-10-22,2,5,0,13.6666669845581,46,0.333333343267441,60,3,2,216,70,1,1,0,1,1,11,7
4702,785,2010-10-22,3,5,0,19.6000003814697,34.5999984741211,5.80000019073486,60,3,2,216,7
4703,785,2010-10-22,4,5,0,33.7999992370605,22.3999996185303,3.79999995231628,60,3,2,216,7
4704,785,2010-10-23,1,6,1,35.6666679382324,14.6666669845581,2,52.3333320617676,3,2,216,70
4705,785,2010-10-23,2,6,1,28,29.6666660308838,2.33333325386047,60,3,2,216,70,1,1,0,1,1,11,72
4706,785,2010-10-23,3,6,1,22,35,3,60,3,2,216,70,1,1,0,1,1,11,7243.419952,4348.015304,6.225363
4707,785,2010-10-23,4,6,1,30.5,21.25,0.75,52.5,3,2,216,70,1,1,0,1,1,11,7243.419952,4348.015304,
4708,785,2010-10-24,0,0,1,42,18,0,60,1,2,216,70,1,1,0,1,1,11,7243.419952,4348.015304,6.225363
4709,785,2010-10-24,1,0,1,22,37,1,60,1,2,216,70,1,1,0,1,1,11,7243.419952,4348.015304,6.225363
4710,785,2010-10-24,2,0,1,14,31.6666660308838,14.3333330154419,60,3,2,216,70,1,1,0,1,1,11,72
4711,785,2010-10-24,3,0,1,43.6666679382324,12.3333330154419,4,60,3,2,216,70,1,1,0,1,1,11,724
4712,785,2010-10-24,4,0,1,26,9.66666698455811,4.66666650772095,40.3333320617676,3,2,216,7
4713,785,2010-10-25,0,1,0,18,15,1,34,1,2,216,70,1,1,0,1,1,11,7243.419952,4348.015304,6.225363
4714,785,2010-10-25,1,1,0,19.5,35.5,5,60,2,2,216,70,1,1,0,1,1,11,7243.419952,4348.015304,6.225
4715,785,2010-10-25,2,1,0,39.6666679382324,19.6666660308838,0.666666686534882,60,3,2,216,
4716,785,2010-10-25,3,1,0,15,42.3333320617676,2.66666674613953,60,3,2,216,70,1,1,0,1,1,11,72
4717,785,2010-10-25,4,1,0,48.6666679382324,11.3333330154419,0,60,3,2,216,70,1,1,0,1,1,11,724
4718,785,2010-10-28,1,4,0,15.3333330154419,44.6666679382324,0,60,3,2,216,70,1,1,0,1,1,11,724
4719,785,2010-10-28,2,4,0,17,40.6666679382324,2.33333325386047,60,3,2,216,70,1,1,0,1,1,11,72
4720,785,2010-10-28,3,4,0,34.3333320617676,22.6666660308838,3,60,3,2,216,70,1,1,0,1,1,11,724
4721,785,2010-10-28,4,4,0,33.6666679382324,23.6666660308838,2.66666674613953,60,3,2,216,7
4722,791,2010-10-24,0,0,1,25,22,0,47,1,2,216,72,0,0,0,1,1,12,7129.02886,5483.54693,5.83578322
4723,791,2010-10-24,1,0,1,32,27,1,60,3,2,216,72,0,0,0,1,1,12,7129.02886,5483.54693,5.83578322
4724,791,2010-10-24,2,0,1,35,23,2,60,3,2,216,72,0,0,0,1,1,12,7129.02886,5483.54693,5.83578322
4725,791,2010-10-24,3,0,1,44,16,0,60,1,2,216,72,0,0,0,1,1,12,7129.02886,5483.54693,5.83578322
4726,791,2010-10-25,0,1,0,29,21,1,51,1,2,216,72,0,0,0,1,1,12,7129.02886,5483.54693,5.83578322
4727,791,2010-10-25,1,1,0,22.6666660308838,30.3333339691162,7,60,3,2,216,72,0,0,0,1,1,12,712
4728,791,2010-10-25,2,1,0,22.3333339691162,30.3333339691162,7.33333349227905,60,3,2,216,7
4729,791,2010-10-25,3,1,0,23.7999992370605,32.5999984741211,3.59999990463257,60,3,2,216,7
4730,791,2010-10-25,4,1,0,40.5,19.5,0,60,3,2,216,72,0,0,0,1,1,12,7129.02886,5483.54693,5.83578
4731,791,2010-10-26,0,2,0,41,14,0,55,1,2,216,72,0,0,0,1,1,12,7129.02886,5483.54693,5.83578322
4732,791,2010-10-26,1,2,0,17.75,15.5,0.75,34,3,2,216,72,0,0,0,1,1,12,7129.02886,5483.54693,5.83
4733,791,2010-10-26,2,2,0,22.3999996185303,23.7999992370605,3.59999990463257,49.7999992
4734,791,2010-10-26,3,2,0,41.3333320617676,17.6666660308838,1,60,3,2,216,72,0,0,0,1,1,12,712
4735,791,2010-10-26,4,2,0,39.2000007629395,19.6000003814697,1.20000004768372,60,3,2,216,7
4736,791,2010-12-01,1,3,0,20.5,7,0.5,28,2,2,216,72,0,0,0,1,1,12,7129.02886,5483.54693,5.835783
4737,791,2010-12-01,2,3,0,22.5,34.25,3.25,60,3,2,216,72,0,0,0,1,1,12,7129.02886,5483.54693,5.83
4738,791,2010-12-01,3,3,0,38.8333320617676,19.8333339691162,1.33333337306976,60,3,2,216,7
4739,791,2010-12-01,4,3,0,38.25,18.25,3.5,60,3,2,216,72,0,0,0,1,1,12,7129.02886,5483.54693,5.83
4740,791,2010-12-02,0,4,0,31,13,0,44,1,2,216,72,0,0,0,1,1,12,7129.02886,5483.54693,5.83578322
4741,791,2010-12-02,1,4,0,35.75,23.5,0.5,59.75,3,2,216,72,0,0,0,1,1,12,7129.02886,5483.54693,5.
4742,791,2010-12-02,2,4,0,25,31,4,60,3,2,216,72,0,0,0,1,1,12,7129.02886,5483.54693,5.83578322
4743,791,2010-12-02,3,4,0,18.6666660308838,35.3333320617676,6,60,3,2,216,72,0,0,0,1,1,12,712
4744,791,2010-12-02,4,4,0,38.5999984741211,20.7999992370605,0.600000023841858,60,3,2,216,
4745,791,2010-12-03,1,5,0,47.5,9.5,3,60,2,2,216,72,0,0,0,1,1,12,7129.02886,5483.54693,5.835783
4746,791,2010-12-03,2,5,0,46.6666679382324,12.6666669845581,0.666666686534882,60,3,2,216,
4747,791,2010-12-03,3,5,0,41.6666679382324,17.6666660308838,0.666666686534882,60,3,2,216,
4748,791,2010-12-03,4,5,0,28.5,14.5,6.5,49.5,2,2,216,72,0,0,0,1,1,12,7129.02886,5483.54693,5.83
4749,792,2010-11-25,0,4,0,0,3,1,4,1,2,216,68,1,0,0,1,1,12,7129.02886,5483.54693,5.835783225,8.

4750,792,2010-11-25,1,4,0,26,30,4,60,3,2,216,68,1,0,0,1,1,12,7129.02886,5483.54693,5.835783225
4751,792,2010-11-25,2,4,0,12.3333330154419,45,2.66666674613953,60,3,2,216,68,1,0,0,1,1,12,71
4752,792,2010-11-25,3,4,0,14.3333330154419,41.3333320617676,4.33333349227905,60,3,2,216,6
4753,792,2010-11-25,4,4,0,33.6666679382324,25.3333339691162,1,60,3,2,216,68,1,0,0,1,1,12,712
4754,792,2010-11-26,0,5,0,5,8,1,14,1,2,216,68,1,0,0,1,1,12,7129.02886,5483.54693,5.835783225,8
4755,792,2010-11-26,1,5,0,45.5,14.5,0,60,2,2,216,68,1,0,0,1,1,12,7129.02886,5483.54693,5.835783
4756,792,2010-11-26,2,5,0,20,34.6666679382324,5.33333349227905,60,3,2,216,68,1,0,0,1,1,12,71
4757,792,2010-11-26,3,5,0,28,26.6666660308838,5.33333349227905,60,3,2,216,68,1,0,0,1,1,12,71
4758,792,2010-11-26,4,5,0,15.6666669845581,24.3333339691162,20,60,3,2,216,68,1,0,0,1,1,12,71
4759,792,2010-11-27,0,6,1,17.5,14.5,0,32,2,2,216,68,1,0,0,1,1,12,7129.02886,5483.54693,5.835783
4760,792,2010-11-27,1,6,1,24.7999992370605,32,3.20000004768372,60,3,2,216,68,1,0,0,1,1,12,71
4761,792,2010-11-27,2,6,1,19.6666660308838,33.3333320617676,7,60,3,2,216,68,1,0,0,1,1,12,712
4762,792,2010-11-27,3,6,1,17.3333339691162,34.3333320617676,8.33333301544189,60,3,2,216,6
4763,792,2010-11-27,4,6,1,27,29.6666660308838,3.33333325386047,60,3,2,216,68,1,0,0,1,1,12,71
4764,792,2010-11-28,0,0,1,0,1,0,1,1,2,216,68,1,0,0,1,1,12,7129.02886,5483.54693,5.835783225,8.1
4765,792,2010-11-28,1,0,1,39.6666679382324,17.3333339691162,3,60,3,2,216,68,1,0,0,1,1,12,712
4766,792,2010-11-28,2,0,1,8,34,18,60,3,2,216,68,1,0,0,1,1,12,7129.02886,5483.54693,5.835783225
4767,792,2010-11-28,3,0,1,23,29.6666660308838,7.33333349227905,60,3,2,216,68,1,0,0,1,1,12,71
4768,792,2010-11-28,4,0,1,32.6666679382324,24.6666660308838,2.66666674613953,60,3,2,216,6
4769,792,2010-11-29,2,1,0,45.3333320617676,13.6666669845581,1,60,3,2,216,68,1,0,0,1,1,12,712
4770,792,2010-11-29,3,1,0,42,15.6666669845581,2.33333325386047,60,3,2,216,68,1,0,0,1,1,12,71
4771,792,2010-11-29,4,1,0,26.5,13.5,8.5,48.5,2,2,216,68,1,0,0,1,1,12,7129.02886,5483.54693,5.835
4772,792,2010-12-03,0,5,0,23.5,10.5,0,34,2,2,216,68,1,0,0,1,1,12,7129.02886,5483.54693,5.835783
4773,792,2010-12-03,1,5,0,9.5,46,4.5,60,2,2,216,68,1,0,0,1,1,12,7129.02886,5483.54693,5.8357832
4774,792,2010-12-04,0,6,1,14,29.5,1.5,45,2,2,216,68,1,0,0,1,1,12,7129.02886,5483.54693,5.835783
4775,792,2010-12-04,1,6,1,22,35,3,60,2,2,216,68,1,0,0,1,1,12,7129.02886,5483.54693,5.835783225
4776,792,2010-12-04,2,6,1,16,42.6666679382324,1.33333337306976,60,3,2,216,68,1,0,0,1,1,12,71
4777,792,2010-12-04,3,6,1,42.3333320617676,17.3333339691162,0.333333343267441,60,3,2,216,
4778,792,2010-12-04,4,6,1,20,39,1,60,3,2,216,68,1,0,0,1,1,12,7129.02886,5483.54693,5.835783225
4779,792,2010-12-05,0,0,1,29,20,0,49,1,2,216,68,1,0,0,1,1,12,7129.02886,5483.54693,5.835783225
4780,792,2010-12-05,1,0,1,51.5,8,0.5,60,2,2,216,68,1,0,0,1,1,12,7129.02886,5483.54693,5.8357832
4781,792,2010-12-05,2,0,1,34.3333320617676,23.3333339691162,2.33333325386047,60,3,2,216,6
4782,792,2010-12-05,3,0,1,24,30,6,60,3,2,216,68,1,0,0,1,1,12,7129.02886,5483.54693,5.835783225
4783,792,2010-12-05,4,0,1,30.6666660308838,28.3333339691162,1,60,3,2,216,68,1,0,0,1,1,12,712
4784,793,2012-07-31,0,2,0,42,18,0,60,1,2,216,71,1,1,0,1,1,12,7091.496661,4023.456929,5.4282939
4785,793,2012-07-31,1,2,0,42.6666679382324,17.3333339691162,0,60,3,2,216,71,1,1,0,1,1,12,709
4786,793,2012-07-31,2,2,0,32.3333320617676,27.3333339691162,0.333333343267441,60,3,2,216,
4787,793,2012-07-31,3,2,0,20.3333339691162,25,14.6666669845581,60,3,2,216,71,1,1,0,1,1,12,70
4788,793,2012-07-31,4,2,0,25.3333339691162,33,1.66666662693024,60,3,2,216,71,1,1,0,1,1,12,70
4789,793,2012-08-01,0,3,0,7.66666650772095,9.66666698455811,1,18.3333339691162,3,2,216,71
4790,793,2012-08-01,1,3,0,15.3333330154419,42,2.66666674613953,60,3,2,216,71,1,1,0,1,1,12,70
4791,793,2012-08-01,2,3,0,36.6666679382324,22.6666660308838,0.666666686534882,60,3,2,216,
4792,793,2012-08-01,3,3,0,14.3333330154419,41,4.66666650772095,60,3,2,216,71,1,1,0,1,1,12,70
4793,793,2012-08-01,4,3,0,28.3333339691162,30.6666660308838,1,60,3,2,216,71,1,1,0,1,1,12,709
4794,793,2012-08-02,0,4,0,9.33333301544189,3.66666674613953,0.333333343267441,13.3333330
4795,793,2012-08-02,1,4,0,30,20,10,60,3,2,216,71,1,1,0,1,1,12,7091.496661,4023.456929,5.428293
4796,793,2012-08-02,2,4,0,40.3333320617676,14,5.66666650772095,60,3,2,216,71,1,1,0,1,1,12,70
4797,793,2012-08-02,3,4,0,31.3333339691162,27.3333339691162,1.33333337306976,60,3,2,216,7
4798,793,2012-08-02,4,4,0,20.3333339691162,39,0.666666686534882,60,3,2,216,71,1,1,0,1,1,12,7
4799,793,2012-08-03,0,5,0,10.3333330154419,22.3333339691162,1.33333337306976,34,3,2,216,7

4800,793,2012-08-03,1,5,0,32.6666679382324,22,5.33333349227905,60,3,2,216,71,1,1,0,1,1,12,70
4801,793,2012-08-03,2,5,0,36.6666679382324,23,0.333333343267441,60,3,2,216,71,1,1,0,1,1,12,7
4802,793,2012-08-03,3,5,0,45,13,2,60,3,2,216,71,1,1,0,1,1,12,7091.496661,4023.456929,5.428293
4803,793,2012-08-03,4,5,0,37,18.3333339691162,4.66666650772095,60,3,2,216,71,1,1,0,1,1,12,70
4804,793,2012-08-04,0,6,1,21,10.3333330154419,0.666666686534882,32,3,2,216,71,1,1,0,1,1,12,7
4805,793,2012-08-04,1,6,1,28,30.6666660308838,1.33333337306976,60,3,2,216,71,1,1,0,1,1,12,70
4806,793,2012-08-04,2,6,1,22.6666660308838,35.6666679382324,1.66666662693024,60,3,2,216,7
4807,793,2012-08-04,3,6,1,25.6666660308838,30.3333339691162,4,60,3,2,216,71,1,1,0,1,1,12,709
4808,793,2012-08-04,4,6,1,12,26,22,60,3,2,216,71,1,1,0,1,1,12,7091.496661,4023.456929,5.42829
4809,793,2012-08-05,0,7,1,7,18,0,25,3,2,216,71,1,1,0,1,1,12,7091.496661,4023.456929,5.4282939
4810,793,2012-08-05,1,7,1,4.66666650772095,42.6666679382324,12.6666669845581,60,3,2,216,7
4811,793,2012-08-05,2,7,1,13.3333330154419,45.6666679382324,1,60,3,2,216,71,1,1,0,1,1,12,709
4812,793,2012-08-05,3,7,1,35,24.3333339691162,0.666666686534882,60,3,2,216,71,1,1,0,1,1,12,7
4813,793,2012-08-05,4,7,1,11.3333330154419,48,0.666666686534882,60,3,2,216,71,1,1,0,1,1,12,7
4814,793,2012-08-06,0,1,0,1.33333337306976,6.66666650772095,0.333333343267441,8.3333330
4815,793,2012-08-06,1,1,0,8.33333301544189,50.3333320617676,1.33333337306976,60,3,2,216,7
4816,793,2012-08-06,2,1,0,45,12.3333330154419,2.66666674613953,60,3,2,216,71,1,1,0,1,1,12,70
4817,793,2012-08-06,3,1,0,13.3333330154419,31.3333339691162,15.3333330154419,60,3,2,216,7
4818,793,2012-08-06,4,1,0,18.6666660308838,39,2.33333325386047,60,3,2,216,71,1,1,0,1,1,12,70
4819,795,2010-11-23,0,2,0,20,27,0,47,1,2,216,69,0,1,0,1,1,11,7243.419952,4348.015304,6.225363
4820,795,2010-11-23,1,2,0,14,43.6666679382324,2.33333325386047,60,3,2,216,69,0,1,0,1,1,11,72
4821,795,2010-11-23,2,2,0,34.25,24,1.75,60,3,2,216,69,0,1,0,1,1,11,7243.419952,4348.015304,6.2
4822,795,2010-11-23,3,2,0,41.2000007629395,18.3999996185303,0.400000005960464,60,3,2,216,
4823,795,2010-11-23,4,2,0,39.3333320617676,16.6666660308838,4,60,3,2,216,69,0,1,0,1,1,11,724
4824,795,2010-11-24,0,3,0,19,16.5,0.5,36,2,2,216,69,0,1,0,1,1,11,7243.419952,4348.015304,6.225
4825,795,2010-11-24,1,3,0,26,33.3333320617676,0.666666686534882,60,3,2,216,69,0,1,0,1,1,11,7
4826,795,2010-11-24,2,3,0,26,31.6666660308838,2.33333325386047,60,3,2,216,69,0,1,0,1,1,11,72
4827,795,2010-11-24,3,3,0,19.5,38.75,1.75,60,3,2,216,69,0,1,0,1,1,11,7243.419952,4348.015304,6.
4828,795,2010-11-24,4,3,0,53,7,0,60,3,2,216,69,0,1,0,1,1,11,7243.419952,4348.015304,6.2253636
4829,795,2010-11-25,0,4,0,39,15,0,54,2,2,216,69,0,1,0,1,1,11,7243.419952,4348.015304,6.225363
4830,795,2010-11-25,1,4,0,12,36.3333320617676,11.6666669845581,60,3,2,216,69,0,1,0,1,1,11,72
4831,795,2010-11-25,2,4,0,14,41,5,60,3,2,216,69,0,1,0,1,1,11,7243.419952,4348.015304,6.225363
4832,795,2010-11-25,3,4,0,12.6666669845581,40,7.33333349227905,60,3,2,216,69,0,1,0,1,1,11,72
4833,795,2010-11-25,4,4,0,38.75,20.25,1,60,3,2,216,69,0,1,0,1,1,11,7243.419952,4348.015304,6.2
4834,795,2010-11-26,0,5,0,34,9.25,0.5,43.75,3,2,216,69,0,1,0,1,1,11,7243.419952,4348.015304,6.2
4835,795,2010-11-26,1,5,0,39.6666679382324,17.3333339691162,3,60,3,2,216,69,0,1,0,1,1,11,724
4836,795,2010-11-26,2,5,0,20,37.6666679382324,2.33333325386047,60,3,2,216,69,0,1,0,1,1,11,72
4837,795,2010-11-26,3,5,0,16.6666660308838,42.3333320617676,1,60,3,2,216,69,0,1,0,1,1,11,724
4838,795,2010-11-26,4,5,0,32.6666679382324,19,1.33333337306976,53,3,2,216,69,0,1,0,1,1,11,72
4839,795,2010-11-27,1,6,1,28.8333339691162,23,3.5,55.3333320617676,3,2,216,69,0,1,0,1,1,11,7
4840,795,2010-11-27,2,6,1,23.25,33,3.75,60,3,2,216,69,0,1,0,1,1,11,7243.419952,4348.015304,6.2
4841,795,2010-11-27,3,6,1,22.6666660308838,33.3333320617676,4,60,3,2,216,69,0,1,0,1,1,11,724
4842,795,2010-11-27,4,6,1,46.6666679382324,13,0.333333343267441,60,3,2,216,69,0,1,0,1,1,11,7
4843,795,2010-11-28,2,0,1,4,32,24,60,2,2,216,69,0,1,0,1,1,11,7243.419952,4348.015304,6.225363
4844,795,2010-11-28,3,0,1,43.3333320617676,14,2.66666674613953,60,3,2,216,69,0,1,0,1,1,11,72
4845,795,2010-11-28,4,0,1,25.3333339691162,11.6666669845581,3.66666674613953,40.666667
4846,795,2010-11-29,0,1,0,33,27,0,60,1,2,216,69,0,1,0,1,1,11,7243.419952,4348.015304,6.225363
4847,795,2010-11-29,1,1,0,16,44,0,60,3,2,216,69,0,1,0,1,1,11,7243.419952,4348.015304,6.225363
4848,795,2010-11-30,0,2,0,6,31,0,37,1,2,216,69,0,1,0,1,1,11,7243.419952,4348.015304,6.2253636
4849,795,2010-11-30,1,2,0,13.6666669845581,37.6666679382324,8.66666698455811,60,3,2,216,6

4850,795,2010-11-30,2,2,0,40.5999984741211,19.3999996185303,0,60,3,2,216,69,0,1,0,1,1,11,724
4851,795,2010-11-30,3,2,0,16,43.6666679382324,0.333333343267441,60,3,2,216,69,0,1,0,1,1,11,7
4852,795,2010-11-30,4,2,0,51.3333320617676,8.66666698455811,0,60,3,2,216,69,0,1,0,1,1,11,724
4853,804,2010-11-19,1,5,0,15,23.3999996185303,2.59999990463257,41,3,2,216,73,1,1,1,1,1,12,96
4854,804,2010-11-19,2,5,0,26.3333339691162,31.3333339691162,2.33333325386047,60,3,2,216,7
4855,804,2010-11-19,3,5,0,15.6666669845581,43.6666679382324,0.666666686534882,60,3,2,216,
4856,804,2010-11-19,4,5,0,16.1666660308838,37.8333320617676,6,60,3,2,216,73,1,1,1,1,1,12,968
4857,804,2010-11-20,2,6,1,10,39,11,60,3,2,216,73,1,1,1,1,1,12,9684.239553,5642.713735,4.768430
4858,804,2010-11-20,3,6,1,21.3333339691162,23.3333339691162,15.3333330154419,60,3,2,216,7
4859,804,2010-11-20,4,6,1,36.6666679382324,22.6666660308838,0.666666686534882,60,3,2,216,
4860,804,2010-11-28,1,0,1,17,22.6666660308838,17,56.6666679382324,3,2,216,73,1,1,1,1,1,12,96
4861,804,2010-11-28,2,0,1,40,20,0,60,3,2,216,73,1,1,1,1,1,12,9684.239553,5642.713735,4.7684301
4862,804,2010-11-28,3,0,1,36,19.5,4.5,60,2,2,216,73,1,1,1,1,1,12,9684.239553,5642.713735,4.768
4863,804,2010-11-29,0,1,0,5,9,1,15,1,2,216,73,1,1,1,1,1,12,9684.239553,5642.713735,4.76843014
4864,804,2010-11-29,1,1,0,32.6666679382324,26.3333339691162,1,60,3,2,216,73,1,1,1,1,1,12,968
4865,804,2010-11-29,2,1,0,18.6666660308838,39.3333320617676,2,60,3,2,216,73,1,1,1,1,1,12,968
4866,804,2010-11-29,3,1,0,16.25,32.75,11,60,3,2,216,73,1,1,1,1,1,12,9684.239553,5642.713735,4.
4867,804,2010-11-29,4,1,0,21.7999992370605,28.6000003814697,9.60000038146973,60,3,2,216,7
4868,804,2010-11-30,0,2,0,29,0,0,29,1,2,216,73,1,1,1,1,1,12,9684.239553,5642.713735,4.76843014
4869,804,2010-11-30,1,2,0,11.3333330154419,41.6666679382324,7,60,3,2,216,73,1,1,1,1,1,12,968
4870,804,2010-11-30,2,2,0,17.5,35.3333320617676,7.16666650772095,60,3,2,216,73,1,1,1,1,1,12,
4871,804,2010-11-30,3,2,0,18,31,11,60,3,2,216,73,1,1,1,1,1,12,9684.239553,5642.713735,4.768430
4872,804,2010-11-30,4,2,0,24,26.5,9.5,60,3,2,216,73,1,1,1,1,1,12,9684.239553,5642.713735,4.768
4873,815,2012-07-27,1,5,0,22,21.6666660308838,0,43.6666679382324,3,2,216,75,1,1,0,1,1,7,9332
4874,815,2012-07-27,2,5,0,33.6666679382324,25,1.33333337306976,60,3,2,216,75,1,1,0,1,1,7,933
4875,815,2012-07-27,3,5,0,46.3333320617676,13.6666669845581,0,60,3,2,216,75,1,1,0,1,1,7,9332
4876,815,2012-07-27,4,5,0,28.3333339691162,31.3333339691162,0.333333343267441,60,3,2,216,
4877,815,2012-07-28,0,6,1,10.6666669845581,2,0,12.6666669845581,3,2,216,75,1,1,0,1,1,7,9332.
4878,815,2012-07-28,1,6,1,25.3333339691162,34.3333320617676,0.333333343267441,60,3,2,216,
4879,815,2012-07-28,2,6,1,37.3333320617676,20.3333339691162,2.33333325386047,60,3,2,216,7
4880,815,2012-07-28,3,6,1,37.3333320617676,18,4.66666650772095,60,3,2,216,75,1,1,0,1,1,7,933
4881,815,2012-07-28,4,6,1,43.6666679382324,16.3333339691162,0,60,3,2,216,75,1,1,0,1,1,7,9332
4882,815,2012-07-29,0,7,1,4.33333349227905,3.33333325386047,0,7.66666650772095,3,2,216,75
4883,815,2012-07-29,1,7,1,30.6666660308838,28.3333339691162,1,60,3,2,216,75,1,1,0,1,1,7,9332
4884,815,2012-07-29,2,7,1,42.3333320617676,17,0.666666686534882,60,3,2,216,75,1,1,0,1,1,7,93
4885,815,2012-07-29,3,7,1,49.3333320617676,8,2.66666674613953,60,3,2,216,75,1,1,0,1,1,7,9332
4886,815,2012-07-29,4,7,1,38.3333320617676,20,1.66666662693024,60,3,2,216,75,1,1,0,1,1,7,933
4887,815,2012-07-30,0,1,0,3,2.33333325386047,0,5.33333349227905,3,2,216,75,1,1,0,1,1,7,9332.
4888,815,2012-07-30,1,1,0,38,21.3333339691162,0.666666686534882,60,3,2,216,75,1,1,0,1,1,7,93
4889,815,2012-07-30,2,1,0,44.6666679382324,15,0.333333343267441,60,3,2,216,75,1,1,0,1,1,7,93
4890,815,2012-07-30,3,1,0,37.3333320617676,18,4.66666650772095,60,3,2,216,75,1,1,0,1,1,7,933
4891,815,2012-07-30,4,1,0,50.6666679382324,9.33333301544189,0,60,3,2,216,75,1,1,0,1,1,7,9332
4892,815,2012-07-31,1,2,0,19,26.3333339691162,0,45.3333320617676,3,2,216,75,1,1,0,1,1,7,9332
4893,815,2012-07-31,2,2,0,53.6666679382324,6.33333349227905,0,60,3,2,216,75,1,1,0,1,1,7,9332
4894,815,2012-07-31,3,2,0,49,11,0,60,3,2,216,75,1,1,0,1,1,7,9332.168761,7176.567982,6.7799735
4895,815,2012-07-31,4,2,0,43.3333320617676,16.6666660308838,0,60,3,2,216,75,1,1,0,1,1,7,9332
4896,815,2012-08-01,1,3,0,12.3333330154419,26.3333339691162,0,38.6666679382324,3,2,216,75
4897,815,2012-08-01,2,3,0,32.6666679382324,27.3333339691162,0,60,3,2,216,75,1,1,0,1,1,7,9332
4898,815,2012-08-01,3,3,0,40.6666679382324,17.6666660308838,1.66666662693024,60,3,2,216,7
4899,815,2012-08-01,4,3,0,47.3333320617676,12.6666669845581,0,60,3,2,216,75,1,1,0,1,1,7,9332

4900,815,2012-08-02,1,4,0,11.6666669845581,20.3333339691162,0.333333343267441,32.333332[illegible]
4901,815,2012-08-02,2,4,0,48.6666679382324,11.3333330154419,0,60,3,2,216,75,1,1,0,1,1,7,9332
4902,815,2012-08-02,3,4,0,34.3333320617676,25.3333339691162,0.333333343267441,60,3,2,216,
4903,815,2012-08-02,4,4,0,45.3333320617676,14.6666669845581,0,60,3,2,216,75,1,1,0,1,1,7,9332
4904,818,2010-11-01,0,1,0,19,31.5,1.5,52,2,2,216,68,1,0,1,1,1,11,9332.168761,7176.567982,6.779[illegible]
4905,818,2010-11-01,1,1,0,18.6666660308838,21.6666660308838,19.6666660308838,60,3,2,216,6
4906,818,2010-11-01,2,1,0,6.5,34.5,19,60,3,2,216,68,1,0,1,1,1,11,9332.168761,7176.567982,6.779[illegible]
4907,818,2010-11-01,3,1,0,20,15,25,60,3,2,216,68,1,0,1,1,1,11,9332.168761,7176.567982,6.779973
4908,818,2010-11-01,4,1,0,17.6666660308838,41.6666679382324,0.666666686534882,60,3,2,216,
4909,818,2010-11-02,0,2,0,13.5,29.5,1.5,44.5,2,2,216,68,1,0,1,1,1,11,9332.168761,7176.567982,6.[illegible]
4910,818,2010-11-02,1,2,0,11.3333330154419,15,33.6666679382324,60,3,2,216,68,1,0,1,1,1,11,93
4911,818,2010-11-02,2,2,0,7.25,14.5,38.25,60,3,2,216,68,1,0,1,1,1,11,9332.168761,7176.567982,6.
4912,818,2010-11-02,3,2,0,26.2000007629395,22,11.8000001907349,60,3,2,216,68,1,0,1,1,1,11,93
4913,818,2010-11-02,4,2,0,28.3333339691162,30,1.66666662693024,60,3,2,216,68,1,0,1,1,1,11,93
4914,818,2010-11-03,0,3,0,35,17,0.5,52.5,2,2,216,68,1,0,1,1,1,11,9332.168761,7176.567982,6.779[illegible]
4915,818,2010-11-03,1,3,0,17,42.3333320617676,0.666666686534882,60,3,2,216,68,1,0,1,1,1,11,9
4916,818,2010-11-03,2,3,0,23,32.6666679382324,4.33333349227905,60,3,2,216,68,1,0,1,1,1,11,93
4917,818,2010-11-03,3,3,0,20,39.25,0.75,60,3,2,216,68,1,0,1,1,1,11,9332.168761,7176.567982,6.7[illegible]
4918,818,2010-11-03,4,3,0,32.6666679382324,27.3333339691162,0,60,3,2,216,68,1,0,1,1,1,11,933
4919,818,2010-11-04,1,4,0,15.6666669845581,42,0.333333343267441,58,3,2,216,68,1,0,1,1,1,11,9
4920,818,2010-11-04,2,4,0,14.6666669845581,43.6666679382324,1.66666662693024,60,3,2,216,6
4921,818,2010-11-04,3,4,0,12.3333330154419,37,10.6666669845581,60,3,2,216,68,1,0,1,1,1,11,93
4922,818,2010-11-04,4,4,0,8.39999961853027,42.4000015258789,6.19999980926514,57,3,2,216,6
4923,818,2010-11-20,0,6,1,37,23,0,60,1,2,216,68,1,0,1,1,1,11,9332.168761,7176.567982,6.779973[illegible]
4924,818,2010-11-20,1,6,1,7.33333349227905,35.6666679382324,17,60,3,2,216,68,1,0,1,1,1,11,93
4925,818,2010-11-20,2,6,1,5.5,49.5,5,60,2,2,216,68,1,0,1,1,1,11,9332.168761,7176.567982,6.7799[illegible]
4926,818,2010-11-21,1,0,1,14,38.6666679382324,0,52.6666679382324,3,2,216,68,1,0,1,1,1,11,933
4927,818,2010-11-21,2,0,1,22.25,32.5,5.25,60,3,2,216,68,1,0,1,1,1,11,9332.168761,7176.567982,6.
4928,818,2010-11-21,3,0,1,15.25,30,14.75,60,3,2,216,68,1,0,1,1,1,11,9332.168761,7176.567982,6.[illegible]
4929,818,2010-11-21,4,0,1,8.66666698455811,49.6666679382324,1.66666662693024,60,3,2,216,6
4930,818,2010-11-22,0,1,0,6,14,2,22,1,2,216,68,1,0,1,1,1,11,9332.168761,7176.567982,6.7799735[illegible]
4931,818,2010-11-22,1,1,0,24,35.3333320617676,0.666666686534882,60,3,2,216,68,1,0,1,1,1,11,9
4932,818,2010-11-22,2,1,0,26,34,0,60,3,2,216,68,1,0,1,1,1,11,9332.168761,7176.567982,6.779973[illegible]
4933,818,2010-11-22,3,1,0,13.1999998092651,42.2000007629395,4.59999990463257,60,3,2,216,6
4934,818,2010-11-22,4,1,0,27,32.5,0.5,60,3,2,216,68,1,0,1,1,1,11,9332.168761,7176.567982,6.779[illegible]
4935,818,2010-11-23,1,2,0,9,41.75,3,53.75,3,2,216,68,1,0,1,1,1,11,9332.168761,7176.567982,6.779[illegible]
4936,818,2010-11-23,2,2,0,16.6666660308838,43.3333320617676,0,60,3,2,216,68,1,0,1,1,1,11,933
4937,818,2010-11-23,3,2,0,19.6666660308838,37.6666679382324,2.66666674613953,60,3,2,216,6
4938,818,2010-11-23,4,2,0,14,46,0,60,3,2,216,68,1,0,1,1,1,11,9332.168761,7176.567982,6.779973[illegible]
4939,818,2010-11-24,1,3,0,10.8000001907349,42.4000015258789,4,57.2000007629395,3,2,216,68
4940,818,2010-11-24,2,3,0,22,37.5,0.5,60,3,2,216,68,1,0,1,1,1,11,9332.168761,7176.567982,6.779[illegible]
4941,818,2010-11-24,3,3,0,17,41.3333320617676,1.66666662693024,60,3,2,216,68,1,0,1,1,1,11,93
4942,818,2010-11-24,4,3,0,15.6666669845581,39,5.33333349227905,60,3,2,216,68,1,0,1,1,1,11,93
4943,818,2010-11-25,0,4,0,10.6666669845581,29.6666660308838,0.333333343267441,40.666667[illegible]
4944,818,2010-11-25,1,4,0,27.6666660308838,31.3333339691162,1,60,3,2,216,68,1,0,1,1,1,11,933
4945,818,2010-11-25,2,4,0,22,36.3333320617676,1.66666662693024,60,3,2,216,68,1,0,1,1,1,11,93
4946,818,2010-11-25,3,4,0,11.3333330154419,45.6666679382324,3,60,3,2,216,68,1,0,1,1,1,11,933
4947,818,2010-11-25,4,4,0,28,30.6666660308838,1.33333337306976,60,3,2,216,68,1,0,1,1,1,11,93
4948,823,2010-10-28,1,4,0,30.1666660308838,29.3333339691162,0.16666667163372,59.666667[illegible]
4949,823,2010-10-28,2,4,0,28.6666660308838,31,0.333333343267441,60,3,2,216,87,1,1,1,1,1,7,91

4950,823,2010-10-28,3,4,0,24.3333339691162,35.6666679382324,0,60,3,2,216,87,1,1,1,1,1,7,9139
4951,823,2010-10-28,4,4,0,30.6666660308838,29,0.333333343267441,60,3,2,216,87,1,1,1,1,1,7,91
4952,823,2010-10-29,0,5,0,54,6,0,60,1,2,216,87,1,1,1,1,1,7,9139.1861,6612.240396,6.253846161,8
4953,823,2010-10-29,1,5,0,14,45.3333320617676,0.666666686534882,60,3,2,216,87,1,1,1,1,1,7,91
4954,823,2010-10-29,2,5,0,32,27.8333339691162,0.16666667163372,60,3,2,216,87,1,1,1,1,1,7,913
4955,823,2010-10-29,3,5,0,21,38.6666679382324,0.333333343267441,60,3,2,216,87,1,1,1,1,1,7,91
4956,823,2010-10-29,4,5,0,19.3333339691162,40.6666679382324,0,60,3,2,216,87,1,1,1,1,1,7,9139
4957,823,2010-10-30,0,6,1,0,3,0,3,1,2,216,87,1,1,1,1,1,7,9139.1861,6612.240396,6.253846161,8.61
4958,823,2010-10-30,1,6,1,21,39,0,60,3,2,216,87,1,1,1,1,1,7,9139.1861,6612.240396,6.253846161,
4959,823,2010-10-30,2,6,1,33,27,0,60,3,2,216,87,1,1,1,1,1,7,9139.1861,6612.240396,6.253846161,
4960,823,2010-10-30,3,6,1,18.6000003814697,41.4000015258789,0,60,3,2,216,87,1,1,1,1,1,7,9139
4961,823,2010-10-30,4,6,1,18.6666660308838,41,0.333333343267441,60,3,2,216,87,1,1,1,1,1,7,91
4962,823,2010-10-31,0,0,1,3,4,0,7,1,2,216,87,1,1,1,1,1,7,9139.1861,6612.240396,6.253846161,8.61
4963,823,2010-10-31,1,0,1,22.6666660308838,37.3333320617676,0,60,3,2,216,87,1,1,1,1,1,7,9139
4964,823,2010-10-31,2,0,1,42.6666679382324,17.3333339691162,0,60,3,2,216,87,1,1,1,1,1,7,9139
4965,823,2010-10-31,3,0,1,22,37.75,0.25,60,3,2,216,87,1,1,1,1,1,7,9139.1861,6612.240396,6.2538
4966,823,2010-10-31,4,0,1,33,26.6666660308838,0.333333343267441,60,3,2,216,87,1,1,1,1,1,7,91
4967,823,2010-11-04,0,4,0,3,6,0,9,1,2,216,87,1,1,1,1,1,7,9139.1861,6612.240396,6.253846161,8.61
4968,823,2010-11-09,0,2,0,0,1,0,1,1,2,216,87,1,1,1,1,1,7,9139.1861,6612.240396,6.253846161,8.61
4969,823,2010-11-09,1,2,0,35.6666679382324,24,0.333333343267441,60,3,2,216,87,1,1,1,1,1,7,91
4970,823,2010-11-09,2,2,0,23.6666660308838,36.3333320617676,0,60,3,2,216,87,1,1,1,1,1,7,9139
4971,823,2010-11-09,3,2,0,18.3333339691162,41.6666679382324,0,60,3,2,216,87,1,1,1,1,1,7,9139
4972,823,2010-11-09,4,2,0,27.5,32.1666679382324,0.333333343267441,60,3,2,216,87,1,1,1,1,1,7,
4973,823,2010-11-10,1,3,0,16,37.6666679382324,0.333333343267441,54,3,2,216,87,1,1,1,1,1,7,91
4974,823,2010-11-10,2,3,0,27.3333339691162,32.6666679382324,0,60,3,2,216,87,1,1,1,1,1,7,9139
4975,823,2010-11-10,3,3,0,13.6666669845581,46,0.333333343267441,60,3,2,216,87,1,1,1,1,1,7,91
4976,823,2010-11-10,4,3,0,25.6666660308838,34.3333320617676,0,60,3,2,216,87,1,1,1,1,1,7,9139
4977,836,2010-11-10,0,3,0,32,24,4,60,1,2,216,69,0,1,0,1,1,11,9694.005866,6308.025976,6.563582
4978,836,2010-11-10,1,3,0,40,16.3333339691162,3.66666674613953,60,3,2,216,69,0,1,0,1,1,11,96
4979,836,2010-11-11,0,4,0,20,16,2.5,38.5,2,2,216,69,0,1,0,1,1,11,9694.005866,6308.025976,6.563
4980,836,2010-11-11,1,4,0,4,22,34,60,3,2,216,69,0,1,0,1,1,11,9694.005866,6308.025976,6.563582
4981,836,2010-11-11,2,4,0,32.6666679382324,23,4.33333349227905,60,3,2,216,69,0,1,0,1,1,11,96
4982,836,2010-11-11,3,4,0,13.6666669845581,40,6.33333349227905,60,3,2,216,69,0,1,0,1,1,11,96
4983,836,2010-11-11,4,4,0,15.3333330154419,35.3333320617676,9.33333301544189,60,3,2,216,6
4984,836,2010-11-12,1,5,0,28.3333339691162,19.6666660308838,1.66666662693024,49.666667
4985,836,2010-11-12,2,5,0,30.5,20.8333339691162,8.66666698455811,60,3,2,216,69,0,1,0,1,1,11,
4986,836,2010-11-12,3,5,0,46.6666679382324,11.6666669845581,1.66666662693024,60,3,2,216,6
4987,836,2010-11-12,4,5,0,27.6666660308838,25.3333339691162,7,60,3,2,216,69,0,1,0,1,1,11,969
4988,836,2010-11-13,0,6,1,31.6666660308838,11.6666669845581,2,45.3333320617676,3,2,216,69
4989,836,2010-11-13,1,6,1,28,31.6666660308838,0.333333343267441,60,3,2,216,69,0,1,0,1,1,11,9
4990,836,2010-11-13,2,6,1,38,19.3333339691162,2.66666674613953,60,3,2,216,69,0,1,0,1,1,11,96
4991,836,2010-11-13,3,6,1,20.6666660308838,32.3333320617676,7,60,3,2,216,69,0,1,0,1,1,11,969
4992,836,2010-11-13,4,6,1,8.66666698455811,33,18.3333339691162,60,3,2,216,69,0,1,0,1,1,11,96
4993,836,2010-11-14,1,0,1,23,32,3.33333325386047,58.3333320617676,3,2,216,69,0,1,0,1,1,11,96
4994,836,2010-11-14,2,0,1,27,17,1,45,1,2,216,69,0,1,0,1,1,11,9694.005866,6308.025976,6.563582
4995,836,2010-11-14,3,0,1,17.5,29,6.33333349227905,52.8333320617676,3,2,216,69,0,1,0,1,1,11,
4996,836,2010-11-14,4,0,1,17,34.5999984741211,8.39999961853027,60,3,2,216,69,0,1,0,1,1,11,96
4997,836,2010-11-15,0,1,0,4.5,17.5,2,24,2,2,216,69,0,1,0,1,1,11,9694.005866,6308.025976,6.56358
4998,836,2010-11-15,1,1,0,9,48,3,60,3,2,216,69,0,1,0,1,1,11,9694.005866,6308.025976,6.5635825
4999,836,2010-11-15,2,1,0,26.3333339691162,31,2.66666674613953,60,3,2,216,69,0,1,0,1,1,11,96

5000,836,2010-11-15,3,1,0,41.3333320617676,18,0.666666686534882,60,3,2,216,69,0,1,0,1,1,11,9
5001,836,2010-11-15,4,1,0,15,34,11,60,3,2,216,69,0,1,0,1,1,11,9694.005866,6308.025976,6.56358
5002,836,2010-11-16,1,2,0,40,19,1,60,3,2,216,69,0,1,0,1,1,11,9694.005866,6308.025976,6.5635825
5003,836,2010-11-16,2,2,0,20.3333339691162,32,7.66666650772095,60,3,2,216,69,0,1,0,1,1,11,96
5004,836,2010-11-16,3,2,0,8,42,10,60,3,2,216,69,0,1,0,1,1,11,9694.005866,6308.025976,6.5635825
5005,836,2010-11-16,4,2,0,29,11,9.5,49.5,2,2,216,69,0,1,0,1,1,11,9694.005866,6308.025976,6.563
5006,837,2010-11-16,0,2,0,50,10,0,60,1,2,216,69,1,1,0,0,1,10,9731.316406,6591.464379,6.948255
5007,837,2010-11-16,1,2,0,20,33.3333320617676,6.66666650772095,60,3,2,216,69,1,1,0,0,1,10,97
5008,837,2010-11-16,2,2,0,15.6666669845581,41.6666679382324,2.66666674613953,60,3,2,216,6
5009,837,2010-11-16,3,2,0,25,33.3333320617676,1.66666662693024,60,3,2,216,69,1,1,0,0,1,10,97
5010,837,2010-11-16,4,2,0,5,54,1,60,1,2,216,69,1,1,0,0,1,10,9731.316406,6591.464379,6.9482559
5011,837,2010-11-17,0,3,0,16.5,29,0,45.5,2,2,216,69,1,1,0,0,1,10,9731.316406,6591.464379,6.948
5012,837,2010-11-17,1,3,0,17.6666660308838,39,3.33333325386047,60,3,2,216,69,1,1,0,0,1,10,97
5013,837,2010-11-17,2,3,0,13.6666669845581,37,9.33333301544189,60,3,2,216,69,1,1,0,0,1,10,97
5014,837,2010-11-17,3,3,0,12.6666669845581,41,6.33333349227905,60,3,2,216,69,1,1,0,0,1,10,97
5015,837,2010-11-17,4,3,0,37,21.3333339691162,1.66666662693024,60,3,2,216,69,1,1,0,0,1,10,97
5016,837,2010-11-18,1,4,0,24.25,25,0.25,49.5,3,2,216,69,1,1,0,0,1,10,9731.316406,6591.464379,6.
5017,837,2010-11-18,2,4,0,32.6666679382324,25,2.33333325386047,60,3,2,216,69,1,1,0,0,1,10,97
5018,837,2010-11-18,3,4,0,13.3333330154419,43,3.66666674613953,60,3,2,216,69,1,1,0,0,1,10,97
5019,837,2010-11-18,4,4,0,29.7999992370605,30,0.200000002980232,60,3,2,216,69,1,1,0,0,1,10,9
5020,837,2010-11-19,0,5,0,13,13,0,26,1,2,216,69,1,1,0,0,1,10,9731.316406,6591.464379,6.948255
5021,837,2010-11-19,1,5,0,30.5,29.25,0.25,60,3,2,216,69,1,1,0,0,1,10,9731.316406,6591.464379,6.
5022,837,2010-11-19,2,5,0,27.6666660308838,30.6666660308838,1.66666662693024,60,3,2,216,6
5023,837,2010-11-19,3,5,0,44.6666679382324,15.3333330154419,0,60,3,2,216,69,1,1,0,0,1,10,973
5024,837,2010-11-19,4,5,0,43.6666679382324,16.3333339691162,0,60,3,2,216,69,1,1,0,0,1,10,973
5025,837,2010-11-20,0,6,1,19,16.5,0.5,36,2,2,216,69,1,1,0,0,1,10,9731.316406,6591.464379,6.948
5026,837,2010-11-20,1,6,1,12.25,45.25,2.5,60,3,2,216,69,1,1,0,0,1,10,9731.316406,6591.464379,6.
5027,837,2010-11-20,2,6,1,21.75,36.75,1.5,60,3,2,216,69,1,1,0,0,1,10,9731.316406,6591.464379,6.
5028,837,2010-11-20,3,6,1,49.6666679382324,10.3333330154419,0,60,3,2,216,69,1,1,0,0,1,10,973
5029,837,2010-11-20,4,6,1,33.6666679382324,25.6666660308838,0.666666686534882,60,3,2,216,
5030,837,2010-11-21,0,0,1,22.5,15,0,37.5,2,2,216,69,1,1,0,0,1,10,9731.316406,6591.464379,6.948
5031,837,2010-11-21,1,0,1,35.6666679382324,24,0.333333343267441,60,3,2,216,69,1,1,0,0,1,10,9
5032,837,2010-11-21,2,0,1,20.5,34.75,4.75,60,3,2,216,69,1,1,0,0,1,10,9731.316406,6591.464379,6.
5033,837,2010-11-21,3,0,1,7.66666650772095,47.6666679382324,4.66666650772095,60,3,2,216,6
5034,837,2010-11-21,4,0,1,28.6666660308838,30.6666660308838,0.666666686534882,60,3,2,216,
5035,837,2010-11-22,2,1,0,45,14,1,60,1,2,216,69,1,1,0,0,1,10,9731.316406,6591.464379,6.948255
5036,837,2010-11-22,3,1,0,16.6666660308838,37.3333320617676,6,60,3,2,216,69,1,1,0,0,1,10,973
5037,837,2010-11-22,4,1,0,29.6666660308838,29,1.33333337306976,60,3,2,216,69,1,1,0,0,1,10,97
5038,842,2010-11-06,0,6,1,1,3,1,5,1,2,216,69,0,1,0,0,1,12,9731.316406,6591.464379,6.948255905,
5039,842,2010-11-06,1,6,1,28,27.3333339691162,4.66666650772095,60,3,2,216,69,0,1,0,0,1,12,97
5040,842,2010-11-06,2,6,1,31,24.6666660308838,4.33333349227905,60,3,2,216,69,0,1,0,0,1,12,97
5041,842,2010-11-06,3,6,1,37,15.3333330154419,7.66666650772095,60,3,2,216,69,0,1,0,0,1,12,97
5042,842,2010-11-06,4,6,1,45.3333320617676,13.6666669845581,1,60,3,2,216,69,0,1,0,0,1,12,973
5043,842,2010-11-07,0,0,1,19,26,0.333333343267441,45.3333320617676,3,2,216,69,0,1,0,0,1,12,9
5044,842,2010-11-07,1,0,1,30.6666660308838,28,1.33333337306976,60,3,2,216,69,0,1,0,0,1,12,97
5045,842,2010-11-07,2,0,1,24,32.6666679382324,3.33333325386047,60,3,2,216,69,0,1,0,0,1,12,97
5046,842,2010-11-07,3,0,1,36,20,4,60,3,2,216,69,0,1,0,0,1,12,9731.316406,6591.464379,6.948255
5047,842,2010-11-07,4,0,1,29.75,29.25,1,60,3,2,216,69,0,1,0,0,1,12,9731.316406,6591.464379,6.9
5048,842,2010-11-08,0,1,0,14.5,21.5,8,44,2,2,216,69,0,1,0,0,1,12,9731.316406,6591.464379,6.948
5049,842,2010-11-08,1,1,0,12.8000001907349,35.5999984741211,11.6000003814697,60,3,2,216,6

5050,842,2010-11-08,2,1,0,24.6666660308838,31,4.33333349227905,60,3,2,216,69,0,1,0,0,1,12,97
5051,842,2010-11-08,3,1,0,6,49,5,60,3,2,216,69,0,1,0,0,1,12,9731.316406,6591.464379,6.9482559
5052,842,2010-11-08,4,1,0,26.6666660308838,30.3333339691162,3,60,3,2,216,69,0,1,0,0,1,12,973
5053,842,2010-11-09,1,2,0,18,42,0,60,1,2,216,69,0,1,0,0,1,12,9731.316406,6591.464379,6.948255
5054,842,2010-11-09,2,2,0,14,37.3333320617676,8.66666698455811,60,3,2,216,69,0,1,0,0,1,12,97
5055,842,2010-11-09,3,2,0,31.6666660308838,24.3333339691162,4,60,3,2,216,69,0,1,0,0,1,12,973
5056,842,2010-11-09,4,2,0,35,24.6666660308838,0.333333343267441,60,3,2,216,69,0,1,0,0,1,12,9
5057,842,2010-11-22,0,1,0,39,20,1,60,1,2,216,69,0,1,0,0,1,12,9731.316406,6591.464379,6.948255
5058,842,2010-11-22,1,1,0,12.6666669845581,41,6.33333349227905,60,3,2,216,69,0,1,0,0,1,12,97
5059,842,2010-11-22,2,1,0,19,37.3333320617676,3.66666674613953,60,3,2,216,69,0,1,0,0,1,12,97
5060,842,2010-11-22,3,1,0,39,20,1,60,1,2,216,69,0,1,0,0,1,12,9731.316406,6591.464379,6.948255
5061,842,2010-11-23,0,2,0,25,33.5,1.5,60,2,2,216,69,0,1,0,0,1,12,9731.316406,6591.464379,6.948
5062,842,2010-11-23,1,2,0,16.6666660308838,36,7.33333349227905,60,3,2,216,69,0,1,0,0,1,12,97
5063,842,2010-11-23,2,2,0,23,31.6666660308838,5.33333349227905,60,3,2,216,69,0,1,0,0,1,12,97
5064,842,2010-11-23,3,2,0,19.3999996185303,31.6000003814697,9,60,3,2,216,69,0,1,0,0,1,12,973
5065,842,2010-11-23,4,2,0,36.3333320617676,23.3333339691162,0.333333343267441,60,3,2,216,
5066,842,2010-11-24,0,3,0,11,26,7,44,1,2,216,69,0,1,0,0,1,12,9731.316406,6591.464379,6.948255
5067,842,2010-11-24,1,3,0,24.3333339691162,33,2.66666674613953,60,3,2,216,69,0,1,0,0,1,12,97
5068,842,2010-11-24,2,3,0,22.3333339691162,29,8.66666698455811,60,3,2,216,69,0,1,0,0,1,12,97
5069,842,2010-11-24,3,3,0,15,38,7,60,3,2,216,69,0,1,0,0,1,12,9731.316406,6591.464379,6.948255
5070,842,2010-11-24,4,3,0,38.2000007629395,21.3999996185303,0.400000005960464,60,3,2,216,
5071,843,2010-11-09,0,2,0,24.5,32,1,57.5,2,2,216,81,0,1,0,1,1,11,9646.36136,6458.018467,6.5281
5072,843,2010-11-09,1,2,0,20.6666660308838,37.6666679382324,1.66666662693024,60,3,2,216,8
5073,843,2010-11-10,0,3,0,18.6666660308838,24,0.333333343267441,43,3,2,216,81,0,1,0,1,1,11,9
5074,843,2010-11-10,1,3,0,22.3333339691162,36,1.66666662693024,60,3,2,216,81,0,1,0,1,1,11,96
5075,843,2010-11-10,2,3,0,25.1666660308838,33.3333320617676,1.5,60,3,2,216,81,0,1,0,1,1,11,9
5076,843,2010-11-10,3,3,0,4.33333349227905,52.3333320617676,3.33333325386047,60,3,2,216,8
5077,843,2010-11-10,4,3,0,17.6666660308838,42,0.333333343267441,60,3,2,216,81,0,1,0,1,1,11,9
5078,843,2010-11-11,0,4,0,8.5,27.5,0.5,36.5,2,2,216,81,0,1,0,1,1,11,9646.36136,6458.018467,6.52
5079,843,2010-11-11,1,4,0,10.3333330154419,48.6666679382324,1,60,3,2,216,81,0,1,0,1,1,11,964
5080,843,2010-11-11,2,4,0,28,30.6666660308838,1.33333337306976,60,3,2,216,81,0,1,0,1,1,11,96
5081,843,2010-11-11,3,4,0,11.5,45.6666679382324,2.83333325386047,60,3,2,216,81,0,1,0,1,1,11,
5082,843,2010-11-11,4,4,0,22.25,37.5,0.25,60,3,2,216,81,0,1,0,1,1,11,9646.36136,6458.018467,6.5
5083,843,2010-11-12,0,5,0,10.3333330154419,31.6666660308838,0,42,3,2,216,81,0,1,0,1,1,11,964
5084,843,2010-11-12,1,5,0,25.75,32.75,1.5,60,3,2,216,81,0,1,0,1,1,11,9646.36136,6458.018467,6.5
5085,843,2010-11-12,2,5,0,20.3333339691162,38,1.66666662693024,60,3,2,216,81,0,1,0,1,1,11,96
5086,843,2010-11-12,3,5,0,2,56.6666679382324,1.33333337306976,60,3,2,216,81,0,1,0,1,1,11,964
5087,843,2010-11-12,4,5,0,17.3999996185303,42.5999984741211,0,60,3,2,216,81,0,1,0,1,1,11,964
5088,843,2010-11-25,0,4,0,8,50,2,60,1,2,216,81,0,1,0,1,1,11,9646.36136,6458.018467,6.52812254
5089,843,2010-11-25,1,4,0,13.8000001907349,41.7999992370605,4.40000009536743,60,3,2,216,8
5090,843,2010-11-25,2,4,0,29,30.6666660308838,0.333333343267441,60,3,2,216,81,0,1,0,1,1,11,9
5091,843,2010-11-25,3,4,0,10.3333330154419,48.6666679382324,1,60,3,2,216,81,0,1,0,1,1,11,964
5092,843,2010-11-25,4,4,0,13.6666669845581,46.3333320617676,0,60,3,2,216,81,0,1,0,1,1,11,964
5093,843,2010-11-26,1,5,0,0,55,5,60,1,2,216,81,0,1,0,1,1,11,9646.36136,6458.018467,6.52812254
5094,843,2010-11-26,2,5,0,2,49,9,60,3,2,216,81,0,1,0,1,1,11,9646.36136,6458.018467,6.52812254
5095,843,2010-11-26,3,5,0,22.3333339691162,33,4.66666650772095,60,3,2,216,81,0,1,0,1,1,11,96
5096,843,2010-11-26,4,5,0,24.3333339691162,34.3333320617676,1.33333337306976,60,3,2,216,8
5097,843,2010-12-01,0,3,0,19.6666660308838,26.3333339691162,0.333333343267441,46.333332
5098,843,2010-12-01,1,3,0,15,44.5,0.5,60,2,2,216,81,0,1,0,1,1,11,9646.36136,6458.018467,6.5281
5099,843,2010-12-01,2,3,0,23.6666660308838,36.3333320617676,0,60,3,2,216,81,0,1,0,1,1,11,964

5100,843,2010-12-01,3,3,0,12.3333330154419,47,0.666666686534882,60,3,2,216,81,0,1,0,1,1,11,9
5101,843,2010-12-01,4,3,0,30.6666660308838,29.3333339691162,0,60,3,2,216,81,0,1,0,1,1,11,964
5102,844,2012-08-14,0,2,0,58,1,1,60,1,2,216,69,1,1,0,1,1,11,9694.005866,6308.025976,6.5635825
5103,844,2012-08-14,1,2,0,40,19,1,60,3,2,216,69,1,1,0,1,1,11,9694.005866,6308.025976,6.5635825
5104,844,2012-08-14,2,2,0,32,27.3333339691162,0.666666686534882,60,3,2,216,69,1,1,0,1,1,11,9
5105,844,2012-08-14,3,2,0,10.6666669845581,44.6666679382324,4.66666650772095,60,3,2,216,6
5106,844,2012-08-14,4,2,0,8,42.6666679382324,9.33333301544189,60,3,2,216,69,1,1,0,1,1,11,969
5107,844,2012-08-15,1,3,0,4.66666650772095,22,4,30.6666660308838,3,2,216,69,1,1,0,1,1,11,969
5108,844,2012-08-15,2,3,0,20,38.6666679382324,1.33333337306976,60,3,2,216,69,1,1,0,1,1,11,96
5109,844,2012-08-15,3,3,0,39.6666679382324,20,0.333333343267441,60,3,2,216,69,1,1,0,1,1,11,9
5110,844,2012-08-15,4,3,0,18.6666660308838,41.3333320617676,0,60,3,2,216,69,1,1,0,1,1,11,969
5111,844,2012-08-16,1,4,0,3.33333325386047,14,0.666666686534882,18,3,2,216,69,1,1,0,1,1,11,9
5112,844,2012-08-16,2,4,0,52.6666679382324,7.33333349227905,0,60,3,2,216,69,1,1,0,1,1,11,969
5113,844,2012-08-16,3,4,0,33,26.3333339691162,0.666666686534882,60,3,2,216,69,1,1,0,1,1,11,9
5114,844,2012-08-16,4,4,0,12.3333330154419,45.6666679382324,2,60,3,2,216,69,1,1,0,1,1,11,969
5115,844,2012-08-17,1,5,0,21.6666660308838,22.6666660308838,0.333333343267441,44.666667
5116,844,2012-08-17,2,5,0,31.6666660308838,28.3333339691162,0,60,3,2,216,69,1,1,0,1,1,11,969
5117,844,2012-08-17,3,5,0,14.3333330154419,44,1.66666662693024,60,3,2,216,69,1,1,0,1,1,11,96
5118,844,2012-08-17,4,5,0,20.3333339691162,37,2.66666674613953,60,3,2,216,69,1,1,0,1,1,11,96
5119,844,2012-08-18,1,6,1,3,9,0.666666686534882,12.6666669845581,3,2,216,69,1,1,0,1,1,11,969
5120,844,2012-08-18,2,6,1,8.33333301544189,34,17.6666660308838,60,3,2,216,69,1,1,0,1,1,11,96
5121,844,2012-08-18,3,6,1,20.3333339691162,34.3333320617676,5.33333349227905,60,3,2,216,6
5122,844,2012-08-18,4,6,1,22.3333339691162,35.6666679382324,2,60,3,2,216,69,1,1,0,1,1,11,969
5123,844,2012-08-19,1,7,1,18,22.6666660308838,9,49.6666679382324,3,2,216,69,1,1,0,1,1,11,969
5124,844,2012-08-19,2,7,1,15.3333330154419,42.3333320617676,2.33333325386047,60,3,2,216,6
5125,844,2012-08-19,3,7,1,8.66666698455811,16.6666660308838,0.333333343267441,25.666666
5126,844,2012-08-19,4,7,1,8,5,6,19,3,2,216,69,1,1,0,1,1,11,9694.005866,6308.025976,6.56358257
5127,844,2012-08-20,1,1,0,18,11.6666669845581,0.333333343267441,30,3,2,216,69,1,1,0,1,1,11,9
5128,844,2012-08-20,2,1,0,16.6666660308838,43.3333320617676,0,60,3,2,216,69,1,1,0,1,1,11,969
5129,844,2012-08-20,3,1,0,8.33333301544189,44.6666679382324,7,60,3,2,216,69,1,1,0,1,1,11,969
5130,844,2012-08-20,4,1,0,18.6666660308838,7,7.33333349227905,33,3,2,216,69,1,1,0,1,1,11,969
5131,846,2010-11-26,1,5,0,19.5,38,0.5,58,2,2,216,73,1,1,1,1,1,11,9094.195764,5760.148353,5.3609
5132,846,2010-11-26,2,5,0,27,32.6666679382324,0.333333343267441,60,3,2,216,73,1,1,1,1,1,11,9
5133,846,2010-11-26,3,5,0,32,28,0,60,2,2,216,73,1,1,1,1,1,11,9094.195764,5760.148353,5.360974
5134,846,2010-11-27,0,6,1,39,20,1,60,1,2,216,73,1,1,1,1,1,11,9094.195764,5760.148353,5.360974
5135,846,2010-11-27,1,6,1,44,16,0,60,3,2,216,73,1,1,1,1,1,11,9094.195764,5760.148353,5.360974
5136,846,2010-11-27,2,6,1,20.3333339691162,37.6666679382324,2,60,3,2,216,73,1,1,1,1,1,11,909
5137,846,2010-11-27,3,6,1,18.75,16.25,0,35,3,2,216,73,1,1,1,1,1,11,9094.195764,5760.148353,5.36
5138,846,2010-11-27,4,6,1,42.2000007629395,17.3999996185303,0.400000005960464,60,3,2,216,
5139,846,2010-11-28,0,0,1,9,15,0,24,1,2,216,73,1,1,1,1,1,11,9094.195764,5760.148353,5.3609740
5140,846,2010-11-28,1,0,1,15,13.5,0,28.5,2,2,216,73,1,1,1,1,1,11,9094.195764,5760.148353,5.3609
5141,846,2010-11-28,2,0,1,29.3333339691162,30.6666660308838,0,60,3,2,216,73,1,1,1,1,1,11,909
5142,846,2010-11-28,3,0,1,21,8.33333301544189,0,29.3333339691162,3,2,216,73,1,1,1,1,1,11,909
5143,846,2010-11-28,4,0,1,33.3333320617676,26.3333339691162,0.333333343267441,60,3,2,216,
5144,846,2010-11-29,0,1,0,22,20.5,0.5,43,2,2,216,73,1,1,1,1,1,11,9094.195764,5760.148353,5.3609
5145,846,2010-11-29,1,1,0,11.6666669845581,15.6666669845581,0.333333343267441,27.666666
5146,846,2010-11-29,2,1,0,29.3333339691162,26.3333339691162,0,55.6666679382324,3,2,216,73
5147,846,2010-11-29,3,1,0,25,15,0,40,1,2,216,73,1,1,1,1,1,11,9094.195764,5760.148353,5.360974
5148,846,2010-11-29,4,1,0,34.5,25,0.5,60,3,2,216,73,1,1,1,1,1,11,9094.195764,5760.148353,5.3609
5149,846,2010-11-30,1,2,0,11.25,31.75,1.5,44.5,3,2,216,73,1,1,1,1,1,11,9094.195764,5760.148353,

5150,846,2010-11-30,2,2,0,24.7999992370605,29.7999992370605,1,55.5999984741211,3,2,216,73
5151,846,2010-11-30,3,2,0,31.3333339691162,9.66666698455811,6.66666650772095,47.6666679
5152,846,2010-11-30,4,2,0,19,21,0.333333343267441,40.3333320617676,3,2,216,73,1,1,1,1,1,11,9
5153,846,2010-12-01,2,3,0,7,6,0,13,1,2,216,73,1,1,1,1,1,11,9094.195764,5760.148353,5.36097407
5154,846,2010-12-01,3,3,0,18.5,29.5,0,48,2,2,216,73,1,1,1,1,1,11,9094.195764,5760.148353,5.360
5155,846,2010-12-01,4,3,0,29.6666660308838,28.3333339691162,2,60,3,2,216,73,1,1,1,1,1,11,909
5156,848,2012-08-11,1,6,1,12.3333330154419,27.3333339691162,5.66666650772095,45.333332
5157,848,2012-08-11,2,6,1,21.3333339691162,32,6.66666650772095,60,3,2,216,67,1,1,0,1,1,12,10
5158,848,2012-08-11,3,6,1,20.3333339691162,36,3.66666674613953,60,3,2,216,67,1,1,0,1,1,12,10
5159,848,2012-08-11,4,6,1,15,37.6666679382324,7.33333349227905,60,3,2,216,67,1,1,0,1,1,12,10
5160,848,2012-08-12,1,7,1,8,41.6666679382324,7.33333349227905,57,3,2,216,67,1,1,0,1,1,12,102
5161,848,2012-08-12,2,7,1,20,34,6,60,3,2,216,67,1,1,0,1,1,12,10241.16811,9088.747035,6.276338
5162,848,2012-08-12,3,7,1,26.6666660308838,28.6666660308838,4.66666650772095,60,3,2,216,6
5163,848,2012-08-12,4,7,1,19,34.3333320617676,6.66666650772095,60,3,2,216,67,1,1,0,1,1,12,10
5164,848,2012-08-13,1,1,0,6.66666650772095,26,8.66666698455811,41.3333320617676,3,2,216,6
5165,848,2012-08-13,2,1,0,10.3333330154419,41.6666679382324,8,60,3,2,216,67,1,1,0,1,1,12,102
5166,848,2012-08-13,3,1,0,28.3333339691162,29.6666660308838,2,60,3,2,216,67,1,1,0,1,1,12,102
5167,848,2012-08-13,4,1,0,17,37.6666679382324,5.33333349227905,60,3,2,216,67,1,1,0,1,1,12,10
5168,848,2012-08-14,1,2,0,10,30,9.33333301544189,49.3333320617676,3,2,216,67,1,1,0,1,1,12,10
5169,848,2012-08-14,2,2,0,12,41,7,60,3,2,216,67,1,1,0,1,1,12,10241.16811,9088.747035,6.276338
5170,848,2012-08-14,3,2,0,25.3333339691162,30.6666660308838,4,60,3,2,216,67,1,1,0,1,1,12,102
5171,848,2012-08-14,4,2,0,15,35,10,60,3,2,216,67,1,1,0,1,1,12,10241.16811,9088.747035,6.276338
5172,848,2012-08-15,1,3,0,4.66666650772095,35.3333320617676,14,54,3,2,216,67,1,1,0,1,1,12,10
5173,848,2012-08-15,2,3,0,3.33333325386047,41,15.6666669845581,60,3,2,216,67,1,1,0,1,1,12,10
5174,848,2012-08-15,3,3,0,15.3333330154419,33.3333320617676,11.3333330154419,60,3,2,216,6
5175,848,2012-08-15,4,3,0,26.3333339691162,21.6666660308838,12,60,3,2,216,67,1,1,0,1,1,12,10
5176,848,2012-08-16,1,4,0,8.33333301544189,38.3333320617676,6.33333349227905,53,3,2,216,6
5177,848,2012-08-16,2,4,0,14.3333330154419,41.3333320617676,4.33333349227905,60,3,2,216,6
5178,848,2012-08-16,3,4,0,16.6666660308838,39.6666679382324,3.66666674613953,60,3,2,216,6
5179,848,2012-08-16,4,4,0,21.3333339691162,33.3333320617676,5.33333349227905,60,3,2,216,6
5180,848,2012-08-17,0,5,0,0.666666686534882,9.66666698455811,1.33333337306976,11.666666
5181,848,2012-08-17,1,5,0,15,28,17,60,3,2,216,67,1,1,0,1,1,12,10241.16811,9088.747035,6.276338
5182,848,2012-08-17,2,5,0,26.6666660308838,25,8.33333301544189,60,3,2,216,67,1,1,0,1,1,12,10
5183,848,2012-08-17,3,5,0,29.3333339691162,27.3333339691162,3.33333325386047,60,3,2,216,6
5184,848,2012-08-17,4,5,0,24.6666660308838,31.6666660308838,3.66666674613953,60,3,2,216,6
5185,848,2012-08-18,1,6,1,13,39,4,56,3,2,216,67,1,1,0,1,1,12,10241.16811,9088.747035,6.276338
5186,848,2012-08-18,2,6,1,7.33333349227905,46.3333320617676,6.33333349227905,60,3,2,216,6
5187,848,2012-08-18,3,6,1,18.3333339691162,6.66666650772095,2.66666674613953,27.666666
5188,848,2012-08-18,4,6,1,35,11.6666669845581,1.33333337306976,48,3,2,216,67,1,1,0,1,1,12,10
5189,848,2012-08-19,0,7,1,0.333333343267441,1.66666662693024,1,3,3,2,216,67,1,1,0,1,1,12,102
5190,848,2012-08-19,1,7,1,15.6666669845581,40.3333320617676,4,60,3,2,216,67,1,1,0,1,1,12,102
5191,848,2012-08-19,2,7,1,19.3333339691162,40.3333320617676,0.333333343267441,60,3,2,216,
5192,848,2012-08-19,3,7,1,40.6666679382324,17.3333339691162,2,60,3,2,216,67,1,1,0,1,1,12,102
5193,848,2012-08-19,4,7,1,7.33333349227905,2.66666674613953,1.33333337306976,11.3333330
5194,855,2010-11-18,0,4,0,16,30,4,50,1,2,217,67,0,1,0,1,1,12,6628.818269,5923.880539,6.083232
5195,855,2010-11-18,1,4,0,18.6666660308838,27,14.3333330154419,60,3,2,217,67,0,1,0,1,1,12,66
5196,855,2010-11-18,2,4,0,20.5,30.75,8.75,60,3,2,217,67,0,1,0,1,1,12,6628.818269,5923.880539,6.
5197,855,2010-11-18,3,4,0,27.7999992370605,28.3999996185303,3.79999995231628,60,3,2,217,6
5198,855,2010-11-18,4,4,0,36,23.3333339691162,0.666666686534882,60,3,2,217,67,0,1,0,1,1,12,6
5199,855,2010-11-19,0,5,0,12,21,0.5,33.5,2,2,217,67,0,1,0,1,1,12,6628.818269,5923.880539,6.083

5200,855,2010-11-19,1,5,0,27.3333339691162,26.3333339691162,6.33333349227905,60,3,2,217,6
5201,855,2010-11-19,2,5,0,32.3333320617676,27.3333339691162,0.333333343267441,60,3,2,217,
5202,855,2010-11-19,3,5,0,40.5,19.25,0.25,60,3,2,217,67,0,1,0,1,1,12,6628.818269,5923.880539,6.
5203,855,2010-11-19,4,5,0,41.2000007629395,18.6000003814697,0.200000002980232,60,3,2,217,
5204,855,2010-11-20,0,6,1,14.5,33.5,1,49,2,2,217,67,0,1,0,1,1,12,6628.818269,5923.880539,6.0832
5205,855,2010-11-20,1,6,1,33.75,25,1.25,60,3,2,217,67,0,1,0,1,1,12,6628.818269,5923.880539,6.08
5206,855,2010-11-20,2,6,1,31.6666660308838,27.6666660308838,0.666666686534882,60,3,2,217,
5207,855,2010-11-20,3,6,1,28,29.3333339691162,2.66666674613953,60,3,2,217,67,0,1,0,1,1,12,66
5208,855,2010-11-20,4,6,1,33.75,24,2.25,60,3,2,217,67,0,1,0,1,1,12,6628.818269,5923.880539,6.08
5209,855,2010-11-21,0,0,1,23,20,0,43,2,2,217,67,0,1,0,1,1,12,6628.818269,5923.880539,6.083232
5210,855,2010-11-21,1,0,1,25,28.3999996185303,6.59999990463257,60,3,2,217,67,0,1,0,1,1,12,66
5211,855,2010-11-21,2,0,1,23,27.3333339691162,9.66666698455811,60,3,2,217,67,0,1,0,1,1,12,66
5212,855,2010-11-21,3,0,1,26.3333339691162,20.6666660308838,13,60,3,2,217,67,0,1,0,1,1,12,66
5213,855,2010-11-21,4,0,1,46.6666679382324,13.3333330154419,0,60,3,2,217,67,0,1,0,1,1,12,662
5214,855,2010-11-22,0,1,0,13.5,19.5,5.5,38.5,2,2,217,67,0,1,0,1,1,12,6628.818269,5923.880539,6.0
5215,855,2010-11-22,1,1,0,27,21,12,60,3,2,217,67,0,1,0,1,1,12,6628.818269,5923.880539,6.083232
5216,855,2010-11-22,2,1,0,30.25,22.75,0,53,3,2,217,67,0,1,0,1,1,12,6628.818269,5923.880539,6.08
5217,855,2010-11-22,3,1,0,33.5,19.25,0,52.75,3,2,217,67,0,1,0,1,1,12,6628.818269,5923.880539,6.
5218,855,2010-11-22,4,1,0,31.3333339691162,28,0.666666686534882,60,3,2,217,67,0,1,0,1,1,12,6
5219,855,2010-11-23,0,2,0,28,28,1,57,1,2,217,67,0,1,0,1,1,12,6628.818269,5923.880539,6.083232
5220,855,2010-11-23,1,2,0,23,36.6666679382324,0.333333343267441,60,3,2,217,67,0,1,0,1,1,12,6
5221,855,2010-11-23,2,2,0,29.6666660308838,30.3333339691162,0,60,3,2,217,67,0,1,0,1,1,12,662
5222,855,2010-11-23,3,2,0,33.3333320617676,26.3333339691162,0.333333343267441,60,3,2,217,
5223,855,2010-11-23,4,2,0,43.8333320617676,16,0.16666667163372,60,3,2,217,67,0,1,0,1,1,12,66
5224,855,2010-12-01,0,3,0,26,32,2,60,1,2,217,67,0,1,0,1,1,12,6628.818269,5923.880539,6.083232
5225,855,2010-12-01,1,3,0,15,28,17,60,3,2,217,67,0,1,0,1,1,12,6628.818269,5923.880539,6.083232
5226,855,2010-12-01,2,3,0,18,34.5,7.5,60,2,2,217,67,0,1,0,1,1,12,6628.818269,5923.880539,6.0832
5227,857,2010-11-24,0,3,0,27,14,0.333333343267441,41.3333320617676,3,2,217,76,1,1,0,1,1,11,6
5228,857,2010-11-24,1,3,0,16.2000007629395,43.5999984741211,0.200000002980232,60,3,2,217,
5229,857,2010-11-24,2,3,0,19.6666660308838,39.6666679382324,0.666666686534882,60,3,2,217,
5230,857,2010-11-24,3,3,0,15.6666669845581,43.6666679382324,0.666666686534882,60,3,2,217,
5231,857,2010-11-24,4,3,0,39.6666679382324,20.3333339691162,0,60,3,2,217,76,1,1,0,1,1,11,623
5232,857,2010-11-25,0,4,0,16.5,30,0.5,47,2,2,217,76,1,1,0,1,1,11,6238.843127,6192.89729,6.93728
5233,857,2010-11-25,1,4,0,10.5,49,0.5,60,3,2,217,76,1,1,0,1,1,11,6238.843127,6192.89729,6.93728
5234,857,2010-11-25,2,4,0,18.5,38.8333320617676,2.66666674613953,60,3,2,217,76,1,1,0,1,1,11,6
5235,857,2010-11-25,3,4,0,25.3333339691162,33.6666679382324,1,60,3,2,217,76,1,1,0,1,1,11,623
5236,857,2010-11-25,4,4,0,28,19,1,48,1,2,217,76,1,1,0,1,1,11,6238.843127,6192.89729,6.93728242
5237,857,2010-11-26,0,5,0,8,10,1,19,1,2,217,76,1,1,0,1,1,11,6238.843127,6192.89729,6.937282421
5238,857,2010-11-26,1,5,0,20.3333339691162,36.3333320617676,1,57.6666679382324,3,2,217,76
5239,857,2010-11-26,2,5,0,22.3333339691162,37.3333320617676,0.333333343267441,60,3,2,217,
5240,857,2010-11-26,3,5,0,25.3333339691162,34.5,0.16666667163372,60,3,2,217,76,1,1,0,1,1,11,6
5241,857,2010-11-26,4,5,0,28.75,29.5,0.25,58.5,3,2,217,76,1,1,0,1,1,11,6238.843127,6192.89729,6
5242,857,2010-11-27,0,6,1,11,46,3,60,1,2,217,76,1,1,0,1,1,11,6238.843127,6192.89729,6.93728242
5243,857,2010-11-27,1,6,1,28.6666660308838,28.6666660308838,0.333333343267441,57.666667
5244,857,2010-11-27,2,6,1,26.6000003814697,33.2000007629395,0.200000002980232,60,3,2,217,
5245,857,2010-11-27,3,6,1,29,30.3333339691162,0.666666686534882,60,3,2,217,76,1,1,0,1,1,11,6
5246,857,2010-11-27,4,6,1,40,14.5,0.5,55,2,2,217,76,1,1,0,1,1,11,6238.843127,6192.89729,6.93728
5247,857,2010-11-28,0,0,1,14.5,39,0.5,54,2,2,217,76,1,1,0,1,1,11,6238.843127,6192.89729,6.93728
5248,857,2010-11-28,1,0,1,17,43,0,60,3,2,217,76,1,1,0,1,1,11,6238.843127,6192.89729,6.93728242
5249,857,2010-11-28,2,0,1,26,33.75,0.25,60,3,2,217,76,1,1,0,1,1,11,6238.843127,6192.89729,6.937

5250,857,2010-11-28,3,0,1,22,38,0,60,3,2,217,76,1,1,0,1,1,11,6238.843127,6192.89729,6.9372824
5251,857,2010-11-28,4,0,1,24.6666660308838,18.3333339691162,0.333333343267441,43.333332
5252,857,2010-11-29,3,1,0,20,40,0,60,3,2,217,76,1,1,0,1,1,11,6238.843127,6192.89729,6.9372824
5253,857,2010-11-29,4,1,0,21.6666660308838,21.3333339691162,0,43,3,2,217,76,1,1,0,1,1,11,623
5254,858,2010-11-23,0,2,0,4,26.5,5,35.5,2,2,217,70,1,1,0,1,1,12,7070.489604,6321.696741,5.71431
5255,858,2010-11-23,1,2,0,7,43.5999984741211,9.39999961853027,60,3,2,217,70,1,1,0,1,1,12,707
5256,858,2010-11-23,2,2,0,43.3333320617676,16.6666660308838,0,60,3,2,217,70,1,1,0,1,1,12,707
5257,858,2010-11-23,3,2,0,14,46,0,60,3,2,217,70,1,1,0,1,1,12,7070.489604,6321.696741,5.714319
5258,858,2010-11-23,4,2,0,11.3333330154419,48,0.666666686534882,60,3,2,217,70,1,1,0,1,1,12,7
5259,858,2010-11-24,0,3,0,3,11,0,14,1,2,217,70,1,1,0,1,1,12,7070.489604,6321.696741,5.7143193
5260,858,2010-11-24,1,3,0,8.25,48.5,3.25,60,3,2,217,70,1,1,0,1,1,12,7070.489604,6321.696741,5.7
5261,858,2010-11-24,2,3,0,10,48.6666679382324,1.33333337306976,60,3,2,217,70,1,1,0,1,1,12,70
5262,858,2010-11-24,3,3,0,12.25,45.75,2,60,3,2,217,70,1,1,0,1,1,12,7070.489604,6321.696741,5.71
5263,858,2010-11-24,4,3,0,34.6666679382324,23,2.33333325386047,60,3,2,217,70,1,1,0,1,1,12,70
5264,858,2010-11-25,0,4,0,5,31.5,0.5,37,2,2,217,70,1,1,0,1,1,12,7070.489604,6321.696741,5.71431
5265,858,2010-11-25,1,4,0,19,40.6666679382324,0.333333343267441,60,3,2,217,70,1,1,0,1,1,12,7
5266,858,2010-11-25,2,4,0,24,33,3,60,3,2,217,70,1,1,0,1,1,12,7070.489604,6321.696741,5.714319
5267,858,2010-11-25,3,4,0,11,43.25,5.75,60,3,2,217,70,1,1,0,1,1,12,7070.489604,6321.696741,5.71
5268,858,2010-11-25,4,4,0,20,39.6666679382324,0.333333343267441,60,3,2,217,70,1,1,0,1,1,12,7
5269,858,2010-11-26,0,5,0,6,50,4,60,1,2,217,70,1,1,0,1,1,12,7070.489604,6321.696741,5.7143193
5270,858,2010-11-26,1,5,0,11.6666669845581,35.3333320617676,13,60,3,2,217,70,1,1,0,1,1,12,70
5271,858,2010-11-26,2,5,0,13.6666669845581,40.6666679382324,5.66666650772095,60,3,2,217,7
5272,858,2010-11-26,3,5,0,15.25,44.25,0.5,60,3,2,217,70,1,1,0,1,1,12,7070.489604,6321.696741,5.
5273,858,2010-11-26,4,5,0,31.1666660308838,28.8333339691162,0,60,3,2,217,70,1,1,0,1,1,12,707
5274,858,2010-11-29,0,1,0,6.5,30.5,0,37,2,2,217,70,1,1,0,1,1,12,7070.489604,6321.696741,5.71431
5275,858,2010-11-29,1,1,0,3,50.3333320617676,6.66666650772095,60,3,2,217,70,1,1,0,1,1,12,707
5276,858,2010-11-29,2,1,0,21,37.3333320617676,1.66666662693024,60,3,2,217,70,1,1,0,1,1,12,70
5277,858,2010-11-29,3,1,0,25.6666660308838,32.3333320617676,2,60,3,2,217,70,1,1,0,1,1,12,707
5278,858,2010-11-30,0,2,0,3,41,0,44,3,2,217,70,1,1,0,1,1,12,7070.489604,6321.696741,5.7143193
5279,858,2010-11-30,1,2,0,11,35.3333320617676,13.6666669845581,60,3,2,217,70,1,1,0,1,1,12,70
5280,858,2010-11-30,2,2,0,9,46.3333320617676,4.66666650772095,60,3,2,217,70,1,1,0,1,1,12,707
5281,858,2010-11-30,3,2,0,22,36.3333320617676,1.66666662693024,60,3,2,217,70,1,1,0,1,1,12,70
5282,858,2010-11-30,4,2,0,22.6666660308838,37.3333320617676,0,60,3,2,217,70,1,1,0,1,1,12,707
5283,859,2010-11-19,0,5,0,1,1,0,2,1,2,217,72,0,1,0,1,1,12,7070.489604,6321.696741,5.714319331,
5284,859,2010-11-19,1,5,0,31,26.3333339691162,2.66666674613953,60,3,2,217,72,0,1,0,1,1,12,70
5285,859,2010-11-19,2,5,0,18,40.3333320617676,1.66666662693024,60,3,2,217,72,0,1,0,1,1,12,70
5286,859,2010-11-19,3,5,0,34.6666679382324,24,1.33333337306976,60,3,2,217,72,0,1,0,1,1,12,70
5287,859,2010-11-19,4,5,0,26.6666660308838,28.6666660308838,4.66666650772095,60,3,2,217,7
5288,859,2010-11-20,1,6,1,12.3333330154419,33.6666679382324,12.3333330154419,58.333332
5289,859,2010-11-20,2,6,1,25.6666660308838,32.3333320617676,2,60,3,2,217,72,0,1,0,1,1,12,707
5290,859,2010-11-20,3,6,1,29.6666660308838,27.6666660308838,2.66666674613953,60,3,2,217,7
5291,859,2010-11-20,4,6,1,32.6666679382324,23.3333339691162,4,60,3,2,217,72,0,1,0,1,1,12,707
5292,859,2010-11-21,0,0,1,48.25,3,0.5,51.75,3,2,217,72,0,1,0,1,1,12,7070.489604,6321.696741,5.7
5293,859,2010-11-21,1,0,1,18.3999996185303,22.2000007629395,9.80000019073486,50.4000015
5294,859,2010-11-21,2,0,1,28.7999992370605,20.6000003814697,4,53.4000015258789,3,2,217,72
5295,859,2010-11-21,3,0,1,0,1,0,1,1,2,217,72,0,1,0,1,1,12,7070.489604,6321.696741,5.714319331,
5296,859,2010-11-21,4,0,1,29.5,1.5,0,31,2,2,217,72,0,1,0,1,1,12,7070.489604,6321.696741,5.71431
5297,859,2010-11-22,2,1,0,11,32,17,60,1,2,217,72,0,1,0,1,1,12,7070.489604,6321.696741,5.71431
5298,859,2010-11-22,3,1,0,31,27.3333339691162,1.66666662693024,60,3,2,217,72,0,1,0,1,1,12,70
5299,859,2010-11-22,4,1,0,40.3333320617676,19.6666660308838,0,60,3,2,217,72,0,1,0,1,1,12,707

5300,859,2010-11-28,0,0,1,2,29,10,41,1,2,217,72,0,1,0,1,1,12,7070.489604,6321.696741,5.7143193
5301,859,2010-11-28,1,0,1,16.6000003814697,24.6000003814697,12,53.2000007629395,3,2,217,7
5302,859,2010-11-28,2,0,1,21.6666660308838,38,0.333333343267441,60,3,2,217,72,0,1,0,1,1,12,7
5303,859,2010-11-28,3,0,1,37,22,1,60,3,2,217,72,0,1,0,1,1,12,7070.489604,6321.696741,5.7143193
5304,859,2010-11-28,4,0,1,28.3333339691162,30.6666660308838,1,60,3,2,217,72,0,1,0,1,1,12,707
5305,859,2010-11-29,0,1,0,16,34.5,1,51.5,2,2,217,72,0,1,0,1,1,12,7070.489604,6321.696741,5.714
5306,859,2010-11-29,1,1,0,16.75,41.75,1.5,60,3,2,217,72,0,1,0,1,1,12,7070.489604,6321.696741,5.
5307,859,2010-11-29,2,1,0,15.6000003814697,40.7999992370605,3.59999990463257,60,3,2,217,7
5308,859,2010-11-29,3,1,0,40.3333320617676,18.6666660308838,1,60,3,2,217,72,0,1,0,1,1,12,707
5309,859,2010-11-29,4,1,0,26,32,2,60,3,2,217,72,0,1,0,1,1,12,7070.489604,6321.696741,5.7143193
5310,859,2010-11-30,0,2,0,8,7,0,15,1,2,217,72,0,1,0,1,1,12,7070.489604,6321.696741,5.714319331
5311,859,2010-11-30,1,2,0,3,40,17,60,3,2,217,72,0,1,0,1,1,12,7070.489604,6321.696741,5.7143193
5312,859,2010-11-30,2,2,0,22.5,36,1.5,60,3,2,217,72,0,1,0,1,1,12,7070.489604,6321.696741,5.714
5313,859,2010-11-30,3,2,0,23.3333339691162,35.1666679382324,1.5,60,3,2,217,72,0,1,0,1,1,12,70
5314,859,2010-11-30,4,2,0,12.6666669845581,42,5.33333349227905,60,3,2,217,72,0,1,0,1,1,12,70
5315,860,2010-10-28,1,4,0,51,9,0,60,1,2,217,74,0,1,0,1,1,11,6318.759237,6244.984202,6.92421541
5316,860,2010-10-28,2,4,0,12,1,0,13,1,2,217,74,0,1,0,1,1,11,6318.759237,6244.984202,6.92421541
5317,860,2010-11-01,0,1,0,13,23.6666660308838,1.33333337306976,38,3,2,217,74,0,1,0,1,1,11,63
5318,860,2010-11-01,1,1,0,44.6666679382324,11.6666669845581,3.66666674613953,60,3,2,217,7
5319,860,2010-11-01,2,1,0,31,28.6666660308838,0.333333343267441,60,3,2,217,74,0,1,0,1,1,11,6
5320,860,2010-11-01,3,1,0,31.6666660308838,21.6666660308838,6.66666650772095,60,3,2,217,7
5321,860,2010-11-01,4,1,0,40.75,16.5,2.75,60,3,2,217,74,0,1,0,1,1,11,6318.759237,6244.984202,6.
5322,860,2010-11-02,0,2,0,20.3333339691162,37.6666679382324,2,60,3,2,217,74,0,1,0,1,1,11,631
5323,860,2010-11-02,1,2,0,17.25,33.5,9.25,60,3,2,217,74,0,1,0,1,1,11,6318.759237,6244.984202,6.
5324,860,2010-11-02,2,2,0,39.3333320617676,20.6666660308838,0,60,3,2,217,74,0,1,0,1,1,11,631
5325,860,2010-11-02,3,2,0,31.3333339691162,27.3333339691162,1.33333337306976,60,3,2,217,7
5326,860,2010-11-02,4,2,0,51.3333320617676,8.66666698455811,0,60,3,2,217,74,0,1,0,1,1,11,631
5327,860,2010-11-03,0,3,0,30.3333339691162,26.6666660308838,3,60,3,2,217,74,0,1,0,1,1,11,631
5328,860,2010-11-03,1,3,0,16.25,43.25,0.5,60,3,2,217,74,0,1,0,1,1,11,6318.759237,6244.984202,6.
5329,860,2010-11-03,2,3,0,18.3333339691162,33.3333320617676,8.33333301544189,60,3,2,217,7
5330,860,2010-11-03,3,3,0,42.6666679382324,15.3333330154419,2,60,3,2,217,74,0,1,0,1,1,11,631
5331,860,2010-11-03,4,3,0,48,12,0,60,3,2,217,74,0,1,0,1,1,11,6318.759237,6244.984202,6.9242154
5332,860,2010-11-22,0,1,0,38,21,1,60,1,2,217,74,0,1,0,1,1,11,6318.759237,6244.984202,6.9242154
5333,860,2010-11-22,1,1,0,30,18.6666660308838,11.3333330154419,60,3,2,217,74,0,1,0,1,1,11,63
5334,860,2010-11-22,2,1,0,33.6666679382324,23.3333339691162,3,60,3,2,217,74,0,1,0,1,1,11,631
5335,860,2010-11-22,3,1,0,35,21.3333339691162,3.66666674613953,60,3,2,217,74,0,1,0,1,1,11,63
5336,860,2010-11-23,0,2,0,29.5,7,3.5,40,2,2,217,74,0,1,0,1,1,11,6318.759237,6244.984202,6.92421
5337,860,2010-11-23,1,2,0,41.3333320617676,15,3.66666674613953,60,3,2,217,74,0,1,0,1,1,11,63
5338,860,2010-11-23,2,2,0,29,19,12,60,3,2,217,74,0,1,0,1,1,11,6318.759237,6244.984202,6.924215
5339,860,2010-11-23,3,2,0,50.3333320617676,9.66666698455811,0,60,3,2,217,74,0,1,0,1,1,11,631
5340,860,2010-11-23,4,2,0,48.5,10.6666669845581,0.833333313465118,60,3,2,217,74,0,1,0,1,1,11
5341,860,2010-11-24,0,3,0,11,9,0,20,1,2,217,74,0,1,0,1,1,11,6318.759237,6244.984202,6.92421541
5342,860,2010-11-24,1,3,0,37,16,7,60,3,2,217,74,0,1,0,1,1,11,6318.759237,6244.984202,6.9242154
5343,860,2010-11-24,2,3,0,40.3333320617676,19.6666660308838,0,60,3,2,217,74,0,1,0,1,1,11,631
5344,860,2010-11-24,3,3,0,39.6666679382324,18,2.33333325386047,60,3,2,217,74,0,1,0,1,1,11,63
5345,860,2010-11-24,4,3,0,48.6666679382324,11.3333330154419,0,60,3,2,217,74,0,1,0,1,1,11,631
5346,860,2010-11-25,0,4,0,36.5,0,0.5,37,2,2,217,74,0,1,0,1,1,11,6318.759237,6244.984202,6.92421
5347,860,2010-11-25,1,4,0,52.6666679382324,7.33333349227905,0,60,3,2,217,74,0,1,0,1,1,11,631
5348,860,2010-11-25,2,4,0,30.6666660308838,24,5.33333349227905,60,3,2,217,74,0,1,0,1,1,11,63
5349,860,2010-11-25,3,4,0,44,10,6,60,3,2,217,74,0,1,0,1,1,11,6318.759237,6244.984202,6.9242154

5350,860,2010-11-25,4,4,0,43.5,16.5,0,60,2,2,217,74,0,1,0,1,1,11,6318.759237,6244.984202,6.9242
5351,861,2010-10-28,0,4,0,15,27,0.5,42.5,2,2,217,76,0,1,0,1,1,11,6733.220527,5884.297864,6.3734
5352,861,2010-10-28,1,4,0,22,29,0.333333343267441,51.3333320617676,3,2,217,76,0,1,0,1,1,11,6
5353,861,2010-10-28,2,4,0,35.3333320617676,24.6666660308838,0,60,3,2,217,76,0,1,0,1,1,11,673
5354,861,2010-10-28,3,4,0,33.3333320617676,26.6666660308838,0,60,3,2,217,76,0,1,0,1,1,11,673
5355,861,2010-10-28,4,4,0,25.8333339691162,24.1666660308838,0.16666667163372,50.1666679
5356,861,2010-10-29,0,5,0,18,29,2,49,1,2,217,76,0,1,0,1,1,11,6733.220527,5884.297864,6.373420
5357,861,2010-10-29,1,5,0,28.7999992370605,31,0.200000002980232,60,3,2,217,76,0,1,0,1,1,11,6
5358,861,2010-10-29,2,5,0,31.8333339691162,28.1666660308838,0,60,3,2,217,76,0,1,0,1,1,11,673
5359,861,2010-10-29,3,5,0,30.5,5,0,35.5,2,2,217,76,0,1,0,1,1,11,6733.220527,5884.297864,6.37342
5360,861,2010-10-29,4,5,0,38.6666679382324,18.6666660308838,0,57.3333320617676,3,2,217,76
5361,861,2010-10-30,1,6,1,25,33,0.5,58.5,2,2,217,76,0,1,0,1,1,11,6733.220527,5884.297864,6.3734
5362,861,2010-10-30,2,6,1,33.3333320617676,26.6666660308838,0,60,3,2,217,76,0,1,0,1,1,11,673
5363,861,2010-10-30,3,6,1,41.3333320617676,18.6666660308838,0,60,3,2,217,76,0,1,0,1,1,11,673
5364,861,2010-10-30,4,6,1,38,22,0,60,3,2,217,76,0,1,0,1,1,11,6733.220527,5884.297864,6.373420
5365,861,2010-10-31,1,0,1,18,32.7999992370605,2,52.7999992370605,3,2,217,76,0,1,0,1,1,11,673
5366,861,2010-10-31,2,0,1,10,44.6666679382324,5.33333349227905,60,3,2,217,76,0,1,0,1,1,11,67
5367,861,2010-10-31,3,0,1,8,44,8,60,3,2,217,76,0,1,0,1,1,11,6733.220527,5884.297864,6.37342002
5368,861,2010-10-31,4,0,1,45,14.8000001907349,0.200000002980232,60,3,2,217,76,0,1,0,1,1,11,6
5369,861,2010-11-01,0,1,0,20,30,2,52,1,2,217,76,0,1,0,1,1,11,6733.220527,5884.297864,6.373420
5370,861,2010-11-01,1,1,0,20.75,37.25,2,60,3,2,217,76,0,1,0,1,1,11,6733.220527,5884.297864,6.37
5371,861,2010-11-01,2,1,0,25,28,7,60,3,2,217,76,0,1,0,1,1,11,6733.220527,5884.297864,6.373420
5372,861,2010-11-01,3,1,0,54,4,2,60,3,2,217,76,0,1,0,1,1,11,6733.220527,5884.297864,6.37342002
5373,861,2010-11-01,4,1,0,46,13.3333330154419,0.666666686534882,60,3,2,217,76,0,1,0,1,1,11,6
5374,861,2010-11-02,0,2,0,50,10,0,60,1,2,217,76,0,1,0,1,1,11,6733.220527,5884.297864,6.373420
5375,861,2010-11-02,1,2,0,30.3333339691162,29.6666660308838,0,60,3,2,217,76,0,1,0,1,1,11,673
5376,861,2010-11-02,2,2,0,31.6000003814697,22,0,53.5999984741211,3,2,217,76,0,1,0,1,1,11,673
5377,861,2010-11-02,3,2,0,46,13.8333330154419,0,59.8333320617676,3,2,217,76,0,1,0,1,1,11,673
5378,861,2010-11-02,4,2,0,33,11,2,46,1,2,217,76,0,1,0,1,1,11,6733.220527,5884.297864,6.373420
5379,863,2010-10-28,0,4,0,12,48,0,60,1,2,217,73,1,1,0,1,1,10,6715.377771,5969.745349,6.225711
5380,863,2010-10-28,1,4,0,8.33333301544189,51.6666679382324,0,60,3,2,217,73,1,1,0,1,1,10,671
5381,863,2010-10-28,2,4,0,24.3333339691162,35.6666679382324,0,60,3,2,217,73,1,1,0,1,1,10,671
5382,863,2010-10-28,3,4,0,38.3333320617676,21.3333339691162,0.333333343267441,60,3,2,217,
5383,863,2010-10-28,4,4,0,45,15,0,60,3,2,217,73,1,1,0,1,1,10,6715.377771,5969.745349,6.225711
5384,863,2010-10-29,0,5,0,11.75,21.75,0,33.5,3,2,217,73,1,1,0,1,1,10,6715.377771,5969.745349,6.
5385,863,2010-10-29,1,5,0,19.3333339691162,40.3333320617676,0.333333343267441,60,3,2,217,
5386,863,2010-10-29,2,5,0,31,29,0,60,3,2,217,73,1,1,0,1,1,10,6715.377771,5969.745349,6.225711
5387,863,2010-10-29,3,5,0,34.3333320617676,25.6666660308838,0,60,3,2,217,73,1,1,0,1,1,10,671
5388,863,2010-10-29,4,5,0,35.6666679382324,24.3333339691162,0,60,3,2,217,73,1,1,0,1,1,10,671
5389,863,2010-10-30,0,6,1,7.5,23.5,0,31,2,2,217,73,1,1,0,1,1,10,6715.377771,5969.745349,6.22571
5390,863,2010-10-30,1,6,1,10.6666669845581,49,0.333333343267441,60,3,2,217,73,1,1,0,1,1,10,6
5391,863,2010-10-30,2,6,1,18.3333339691162,41.6666679382324,0,60,3,2,217,73,1,1,0,1,1,10,671
5392,863,2010-10-30,3,6,1,33,27,0,60,3,2,217,73,1,1,0,1,1,10,6715.377771,5969.745349,6.225711
5393,863,2010-10-30,4,6,1,38.3333320617676,21.6666660308838,0,60,3,2,217,73,1,1,0,1,1,10,671
5394,863,2010-10-31,1,0,1,18.3333339691162,41.6666679382324,0,60,3,2,217,73,1,1,0,1,1,10,671
5395,863,2010-10-31,2,0,1,30,30,0,60,3,2,217,73,1,1,0,1,1,10,6715.377771,5969.745349,6.225711
5396,863,2010-10-31,3,0,1,27.6666660308838,32.3333320617676,0,60,3,2,217,73,1,1,0,1,1,10,671
5397,863,2010-10-31,4,0,1,43.3333320617676,16.6666660308838,0,60,3,2,217,73,1,1,0,1,1,10,671
5398,863,2010-11-26,0,5,0,10.5,26.5,0.5,37.5,2,2,217,73,1,1,0,1,1,10,6715.377771,5969.745349,6.
5399,863,2010-11-26,1,5,0,13.6666669845581,46.3333320617676,0,60,3,2,217,73,1,1,0,1,1,10,671

5400,863,2010-11-26,2,5,0,31,29,0,60,3,2,217,73,1,1,0,1,1,10,6715.377771,5969.745349,6.2257115
5401,863,2010-11-26,3,5,0,34.3333320617676,25.6666660308838,0,60,3,2,217,73,1,1,0,1,1,10,671
5402,863,2010-11-26,4,5,0,43,17,0,60,3,2,217,73,1,1,0,1,1,10,6715.377771,5969.745349,6.2257115
5403,863,2010-11-29,4,1,0,39,19.6666660308838,0.333333343267441,59,3,2,217,73,1,1,0,1,1,10,6
5404,863,2010-12-01,0,3,0,15,34,0,49,1,2,217,73,1,1,0,1,1,10,6715.377771,5969.745349,6.2257115
5405,863,2010-12-01,1,3,0,24.6666660308838,35.3333320617676,0,60,3,2,217,73,1,1,0,1,1,10,671
5406,863,2010-12-01,2,3,0,27.3333339691162,31.6666660308838,1,60,3,2,217,73,1,1,0,1,1,10,671
5407,863,2010-12-01,3,3,0,31,29,0,60,3,2,217,73,1,1,0,1,1,10,6715.377771,5969.745349,6.2257115
5408,863,2010-12-01,4,3,0,35.5,24.5,0,60,2,2,217,73,1,1,0,1,1,10,6715.377771,5969.745349,6.2257
5409,863,2010-12-02,0,4,0,8,46,0,54,1,2,217,73,1,1,0,1,1,10,6715.377771,5969.745349,6.22571152
5410,863,2010-12-02,1,4,0,12,43,5,60,3,2,217,73,1,1,0,1,1,10,6715.377771,5969.745349,6.2257115
5411,863,2010-12-02,2,4,0,18,42,0,60,3,2,217,73,1,1,0,1,1,10,6715.377771,5969.745349,6.2257115
5412,863,2010-12-02,3,4,0,22.6666660308838,37.3333320617676,0,60,3,2,217,73,1,1,0,1,1,10,671
5413,863,2010-12-02,4,4,0,44,16,0,60,1,2,217,73,1,1,0,1,1,10,6715.377771,5969.745349,6.2257115
5414,863,2010-12-04,0,6,1,17,34,0.5,51.5,2,2,217,73,1,1,0,1,1,10,6715.377771,5969.745349,6.2257
5415,863,2010-12-04,1,6,1,21.6666660308838,38.3333320617676,0,60,3,2,217,73,1,1,0,1,1,10,671
5416,863,2010-12-04,2,6,1,21.6666660308838,38,0.333333343267441,60,3,2,217,73,1,1,0,1,1,10,6
5417,863,2010-12-04,3,6,1,22.6666660308838,37.3333320617676,0,60,3,2,217,73,1,1,0,1,1,10,671
5418,863,2010-12-04,4,6,1,44.3333320617676,15.6666669845581,0,60,3,2,217,73,1,1,0,1,1,10,671
5419,864,2010-11-29,0,1,0,12,48,0,60,1,2,217,81,0,1,0,1,1,12,6690.192015,5928.880252,6.4670431
5420,864,2010-11-29,1,1,0,8.33333301544189,51.6666679382324,0,60,3,2,217,81,0,1,0,1,1,12,669
5421,864,2010-11-29,2,1,0,24.3333339691162,35.6666679382324,0,60,3,2,217,81,0,1,0,1,1,12,669
5422,864,2010-11-29,3,1,0,38.3333320617676,21.3333339691162,0.333333343267441,60,3,2,217,
5423,864,2010-11-29,4,1,0,44,16,0,60,1,2,217,81,0,1,0,1,1,12,6690.192015,5928.880252,6.4670431
5424,864,2010-11-30,0,2,0,12,23.5,0,35.5,2,2,217,81,0,1,0,1,1,12,6690.192015,5928.880252,6.4670
5425,864,2010-11-30,1,2,0,19.3333339691162,40.3333320617676,0.333333343267441,60,3,2,217,
5426,864,2010-11-30,2,2,0,31,29,0,60,3,2,217,81,0,1,0,1,1,12,6690.192015,5928.880252,6.4670431
5427,864,2010-11-30,3,2,0,42,18,0,60,2,2,217,81,0,1,0,1,1,12,6690.192015,5928.880252,6.4670431
5428,864,2010-11-30,4,2,0,45.5,14.5,0,60,2,2,217,81,0,1,0,1,1,12,6690.192015,5928.880252,6.4670
5429,864,2010-12-01,0,3,0,11.5,20,0,31.5,2,2,217,81,0,1,0,1,1,12,6690.192015,5928.880252,6.4670
5430,864,2010-12-01,1,3,0,10.6666669845581,49,0.333333343267441,60,3,2,217,81,0,1,0,1,1,12,6
5431,864,2010-12-01,2,3,0,18.3333339691162,41.6666679382324,0,60,3,2,217,81,0,1,0,1,1,12,669
5432,864,2010-12-01,3,3,0,29.5,30.5,0,60,3,2,217,81,0,1,0,1,1,12,6690.192015,5928.880252,6.4670
5433,864,2010-12-01,4,3,0,32.75,27.25,0,60,3,2,217,81,0,1,0,1,1,12,6690.192015,5928.880252,6.46
5434,864,2010-12-02,0,4,0,7.5,23.5,0,31,2,2,217,81,0,1,0,1,1,12,6690.192015,5928.880252,6.46704
5435,864,2010-12-02,1,4,0,18.3333339691162,41.6666679382324,0,60,3,2,217,81,0,1,0,1,1,12,669
5436,864,2010-12-02,2,4,0,30,30,0,60,3,2,217,81,0,1,0,1,1,12,6690.192015,5928.880252,6.4670431
5437,864,2010-12-02,3,4,0,27.6666660308838,32.3333320617676,0,60,3,2,217,81,0,1,0,1,1,12,669
5438,864,2010-12-02,4,4,0,44.2000007629395,15.8000001907349,0,60,3,2,217,81,0,1,0,1,1,12,669
5439,864,2010-12-03,0,5,0,11.5,40,0,51.5,2,2,217,81,0,1,0,1,1,12,6690.192015,5928.880252,6.4670
5440,864,2010-12-03,1,5,0,24,36,0,60,3,2,217,81,0,1,0,1,1,12,6690.192015,5928.880252,6.4670431
5441,864,2010-12-03,2,5,0,27.3333339691162,31.6666660308838,1,60,3,2,217,81,0,1,0,1,1,12,669
5442,864,2010-12-03,3,5,0,31,29,0,60,3,2,217,81,0,1,0,1,1,12,6690.192015,5928.880252,6.4670431
5443,864,2010-12-03,4,5,0,38.3333320617676,21.6666660308838,0,60,3,2,217,81,0,1,0,1,1,12,669
5444,864,2010-12-04,0,6,1,17,34,0.5,51.5,2,2,217,81,0,1,0,1,1,12,6690.192015,5928.880252,6.4670
5445,864,2010-12-04,1,6,1,12.3333330154419,42.6666679382324,5,60,3,2,217,81,0,1,0,1,1,12,669
5446,864,2010-12-04,2,6,1,18,42,0,60,3,2,217,81,0,1,0,1,1,12,6690.192015,5928.880252,6.4670431
5447,864,2010-12-04,3,6,1,22.6666660308838,37.3333320617676,0,60,3,2,217,81,0,1,0,1,1,12,669
5448,864,2010-12-04,4,6,1,44.3333320617676,15.6666669845581,0,60,3,2,217,81,0,1,0,1,1,12,669
5449,864,2010-12-05,0,0,1,10.5,26.5,0.5,37.5,2,2,217,81,0,1,0,1,1,12,6690.192015,5928.880252,6.4

5450,864,2010-12-05,1,0,1,21,39,0,60,2,2,217,81,0,1,0,1,1,12,6690.192015,5928.880252,6.4670431
5451,864,2010-12-05,2,0,1,21.6666660308838,38,0.333333343267441,60,3,2,217,81,0,1,0,1,1,12,6
5452,864,2010-12-05,3,0,1,22.6666660308838,37.3333320617676,0,60,3,2,217,81,0,1,0,1,1,12,669
5453,864,2010-12-05,4,0,1,43,17,0,60,3,2,217,81,0,1,0,1,1,12,6690.192015,5928.880252,6.4670431
5454,864,2010-12-06,1,1,0,13.6666669845581,46.3333320617676,0,60,3,2,217,81,0,1,0,1,1,12,669
5455,864,2010-12-06,2,1,0,31,29,0,60,3,2,217,81,0,1,0,1,1,12,6690.192015,5928.880252,6.4670431
5456,864,2010-12-06,3,1,0,34.3333320617676,25.6666660308838,0,60,3,2,217,81,0,1,0,1,1,12,669
5457,864,2010-12-06,4,1,0,39,19.6666660308838,0.333333343267441,59,3,2,217,81,0,1,0,1,1,12,6
5458,865,2010-11-30,0,2,0,13,33.5,0,46.5,2,2,217,73,0,1,0,1,1,11,6238.843127,6192.89729,6.93728
5459,865,2010-11-30,1,2,0,17.3333339691162,41.3333320617676,1.33333337306976,60,3,2,217,7
5460,865,2010-11-30,2,2,0,30.5,26,3.5,60,3,2,217,73,0,1,0,1,1,11,6238.843127,6192.89729,6.93728
5461,865,2010-11-30,3,2,0,35,23.3333339691162,1.66666662693024,60,3,2,217,73,0,1,0,1,1,11,62
5462,865,2010-11-30,4,2,0,23,23,0,46,1,2,217,73,0,1,0,1,1,11,6238.843127,6192.89729,6.93728242
5463,865,2010-12-06,0,1,0,51,8,1,60,1,2,217,73,0,1,0,1,1,11,6238.843127,6192.89729,6.937282421
5464,865,2010-12-06,1,1,0,31.5,26.5,2,60,2,2,217,73,0,1,0,1,1,11,6238.843127,6192.89729,6.93728
5465,865,2010-12-07,0,2,0,11.5,19.5,0.5,31.5,2,2,217,73,0,1,0,1,1,11,6238.843127,6192.89729,6.93
5466,865,2010-12-07,1,2,0,11.5,47.25,1.25,60,3,2,217,73,0,1,0,1,1,11,6238.843127,6192.89729,6.9
5467,865,2010-12-07,2,2,0,19.6666660308838,38.6666679382324,1.66666662693024,60,3,2,217,7
5468,865,2010-12-07,3,2,0,22,36.3333320617676,1.66666662693024,60,3,2,217,73,0,1,0,1,1,11,62
5469,865,2010-12-07,4,2,0,31,28.3333339691162,0.666666686534882,60,3,2,217,73,0,1,0,1,1,11,6
5470,865,2012-07-13,1,5,0,17.3333339691162,37.6666679382324,3.66666674613953,58.6666679
5471,865,2012-07-13,2,5,0,25.3333339691162,32.3333320617676,2.33333325386047,60,3,2,217,7
5472,865,2012-07-13,3,5,0,27.6666660308838,30.3333339691162,2,60,3,2,217,73,0,1,0,1,1,11,623
5473,865,2012-07-13,4,5,0,33.6666679382324,26.3333339691162,0,60,3,2,217,73,0,1,0,1,1,11,623
5474,865,2012-07-14,0,6,1,14.5,23.5,1,39,2,2,217,73,0,1,0,1,1,11,6238.843127,6192.89729,6.93728
5475,865,2012-07-14,1,6,1,34.3333320617676,25.6666660308838,0,60,3,2,217,73,0,1,0,1,1,11,623
5476,865,2012-07-14,2,6,1,22.6666660308838,35.3333320617676,2,60,3,2,217,73,0,1,0,1,1,11,623
5477,865,2012-07-14,3,6,1,29.3333339691162,30,0.666666686534882,60,3,2,217,73,0,1,0,1,1,11,6
5478,865,2012-07-14,4,6,1,29.3333339691162,30.5,0.16666667163372,60,3,2,217,73,0,1,0,1,1,11,6
5479,865,2012-07-15,0,0,1,6.5,23,0,29.5,2,2,217,73,0,1,0,1,1,11,6238.843127,6192.89729,6.937282
5480,865,2012-07-15,1,0,1,27,33,0,60,3,2,217,73,0,1,0,1,1,11,6238.843127,6192.89729,6.93728242
5481,865,2012-07-15,2,0,1,30.3333339691162,28.3333339691162,1.33333337306976,60,3,2,217,7
5482,865,2012-07-15,3,0,1,31.6666660308838,28.3333339691162,0,60,3,2,217,73,0,1,0,1,1,11,623
5483,865,2012-07-15,4,0,1,33.6666679382324,26.3333339691162,0,60,3,2,217,73,0,1,0,1,1,11,623
5484,865,2012-07-16,0,1,0,17,43,0,60,1,2,217,73,0,1,0,1,1,11,6238.843127,6192.89729,6.93728242
5485,865,2012-07-16,1,1,0,18,40,2,60,3,2,217,73,0,1,0,1,1,11,6238.843127,6192.89729,6.93728242
5486,865,2012-07-16,2,1,0,24.3333339691162,34.6666679382324,1,60,3,2,217,73,0,1,0,1,1,11,623
5487,865,2012-07-16,3,1,0,28,31.6666660308838,0.333333343267441,60,3,2,217,73,0,1,0,1,1,11,6
5488,865,2012-07-16,4,1,0,17.6666660308838,42,0.333333343267441,60,3,2,217,73,0,1,0,1,1,11,6
5489,873,2012-07-15,0,0,1,28,24.5,2,54.5,2,2,217,68,1,1,0,1,1,12,9298.075199,8995.118758,6.9603
5490,873,2012-07-15,1,0,1,15,45,0,60,3,2,217,68,1,1,0,1,1,12,9298.075199,8995.118758,6.9603350
5491,873,2012-07-15,2,0,1,28,28.3333339691162,3.66666674613953,60,3,2,217,68,1,1,0,1,1,12,92
5492,873,2012-07-15,3,0,1,32,27.6666660308838,0.333333343267441,60,3,2,217,68,1,1,0,1,1,12,9
5493,873,2012-07-15,4,0,1,44.3333320617676,15.6666669845581,0,60,3,2,217,68,1,1,0,1,1,12,929
5494,873,2012-07-16,0,1,0,32,24.3333339691162,0.666666686534882,57,3,2,217,68,1,1,0,1,1,12,9
5495,873,2012-07-16,1,1,0,16,37,7,60,3,2,217,68,1,1,0,1,1,12,9298.075199,8995.118758,6.9603350
5496,873,2012-07-16,2,1,0,13.75,26.25,20,60,3,2,217,68,1,1,0,1,1,12,9298.075199,8995.118758,6.9
5497,873,2012-07-16,3,1,0,13.3333330154419,43.3333320617676,3.33333325386047,60,3,2,217,6
5498,873,2012-07-16,4,1,0,45.3333320617676,14.6666669845581,0,60,3,2,217,68,1,1,0,1,1,12,929
5499,873,2012-07-17,0,2,0,26.75,24.75,3.5,55,3,2,217,68,1,1,0,1,1,12,9298.075199,8995.118758,6.

5500,873,2012-07-17,1,2,0,16.6666660308838,33.1666679382324,10.1666669845581,60,3,2,217,6
5501,873,2012-07-17,2,2,0,28,19.6666660308838,12.3333330154419,60,3,2,217,68,1,1,0,1,1,12,92
5502,873,2012-07-17,3,2,0,41.7142868041992,16.5714282989502,1.71428573131561,60,3,2,217,6
5503,873,2012-07-17,4,2,0,49.1666679382324,10.5,0.333333343267441,60,3,2,217,68,1,1,0,1,1,12
5504,873,2012-07-18,0,3,0,34,15,2,51,2,2,217,68,1,1,0,1,1,12,9298.075199,8995.118758,6.960335(
5505,873,2012-07-18,1,3,0,7.75,35.25,17,60,3,2,217,68,1,1,0,1,1,12,9298.075199,8995.118758,6.96
5506,873,2012-07-18,2,3,0,9.33333301544189,27.3333339691162,23.3333339691162,60,3,2,217,6
5507,873,2012-07-18,3,3,0,35.5999984741211,23.2000007629395,1.20000004768372,60,3,2,217,6
5508,873,2012-07-18,4,3,0,51.1666679382324,8.33333301544189,0.5,60,3,2,217,68,1,1,0,1,1,12,92
5509,873,2012-07-19,0,4,0,22,30.5,1,53.5,2,2,217,68,1,1,0,1,1,12,9298.075199,8995.118758,6.9603
5510,873,2012-07-19,1,4,0,17,21.3333339691162,21.6666660308838,60,3,2,217,68,1,1,0,1,1,12,92
5511,873,2012-07-19,2,4,0,21,25.7999992370605,13.1999998092651,60,3,2,217,68,1,1,0,1,1,12,92
5512,873,2012-07-19,3,4,0,13.3333330154419,33,13.6666669845581,60,3,2,217,68,1,1,0,1,1,12,92
5513,873,2012-07-19,4,4,0,34.6666679382324,25,0.333333343267441,60,3,2,217,68,1,1,0,1,1,12,9
5514,884,2012-07-14,0,6,1,9,27,0,36,1,2,217,75,1,1,0,1,1,9,9917.015989,9642.992356,7.29479593
5515,884,2012-07-14,1,6,1,32,28,0,60,3,2,217,75,1,1,0,1,1,9,9917.015989,9642.992356,7.29479593
5516,884,2012-07-14,2,6,1,20.3333339691162,39.6666679382324,0,60,3,2,217,75,1,1,0,1,1,9,9917
5517,884,2012-07-14,3,6,1,29,31,0,60,3,2,217,75,1,1,0,1,1,9,9917.015989,9642.992356,7.29479593
5518,884,2012-07-14,4,6,1,37,23,0,60,2,2,217,75,1,1,0,1,1,9,9917.015989,9642.992356,7.29479593
5519,884,2012-07-15,0,0,1,13,32,0,45,1,2,217,75,1,1,0,1,1,9,9917.015989,9642.992356,7.29479593
5520,884,2012-07-15,1,0,1,20.3333339691162,32.6666679382324,7,60,3,2,217,75,1,1,0,1,1,9,9917
5521,884,2012-07-15,2,0,1,28,31.6666660308838,0.333333343267441,60,3,2,217,75,1,1,0,1,1,9,99
5522,884,2012-07-15,3,0,1,22,37.5,0.5,60,2,2,217,75,1,1,0,1,1,9,9917.015989,9642.992356,7.2947
5523,884,2012-07-15,4,0,1,33.3333320617676,26.6666660308838,0,60,3,2,217,75,1,1,0,1,1,9,9917
5524,884,2012-07-16,3,1,0,17,43,0,60,1,2,217,75,1,1,0,1,1,9,9917.015989,9642.992356,7.29479593
5525,884,2012-07-16,4,1,0,28.3333339691162,31.6666660308838,0,60,3,2,217,75,1,1,0,1,1,9,9917
5526,884,2012-07-18,0,3,0,28,32,0,60,1,2,217,75,1,1,0,1,1,9,9917.015989,9642.992356,7.29479593
5527,884,2012-07-18,1,3,0,21.6666660308838,32,6.33333349227905,60,3,2,217,75,1,1,0,1,1,9,991
5528,884,2012-07-18,2,3,0,30,30,0,60,2,2,217,75,1,1,0,1,1,9,9917.015989,9642.992356,7.29479593
5529,884,2012-07-19,0,4,0,12,37,0,49,1,2,217,75,1,1,0,1,1,9,9917.015989,9642.992356,7.29479593
5530,884,2012-07-19,1,4,0,20.6666660308838,39.3333320617676,0,60,3,2,217,75,1,1,0,1,1,9,9917
5531,884,2012-07-19,2,4,0,22.6666660308838,35.3333320617676,2,60,3,2,217,75,1,1,0,1,1,9,9917
5532,884,2012-07-19,3,4,0,31.3333339691162,28.6666660308838,0,60,3,2,217,75,1,1,0,1,1,9,9917
5533,884,2012-07-19,4,4,0,41,19,0,60,3,2,217,75,1,1,0,1,1,9,9917.015989,9642.992356,7.29479593
5534,884,2012-07-20,0,5,0,12.5,20,0,32.5,2,2,217,75,1,1,0,1,1,9,9917.015989,9642.992356,7.2947
5535,884,2012-07-20,1,5,0,24.6666660308838,32,3.33333325386047,60,3,2,217,75,1,1,0,1,1,9,991
5536,884,2012-07-20,2,5,0,37.75,18.75,3.5,60,3,2,217,75,1,1,0,1,1,9,9917.015989,9642.992356,7.2
5537,884,2012-07-20,3,5,0,24,34.5,1.5,60,3,2,217,75,1,1,0,1,1,9,9917.015989,9642.992356,7.2947
5538,884,2012-07-20,4,5,0,29.5,29.5,1,60,3,2,217,75,1,1,0,1,1,9,9917.015989,9642.992356,7.2947
5539,884,2012-07-21,0,6,1,11.5,21.5,0,33,2,2,217,75,1,1,0,1,1,9,9917.015989,9642.992356,7.2947
5540,884,2012-07-21,1,6,1,15,39.3333320617676,5.66666650772095,60,3,2,217,75,1,1,0,1,1,9,991
5541,884,2012-07-21,2,6,1,33,26,1,60,3,2,217,75,1,1,0,1,1,9,9917.015989,9642.992356,7.29479593
5542,884,2012-07-21,3,6,1,23,35,2,60,3,2,217,75,1,1,0,1,1,9,9917.015989,9642.992356,7.29479593
5543,884,2012-07-21,4,6,1,22,38,0,60,1,2,217,75,1,1,0,1,1,9,9917.015989,9642.992356,7.29479593
5544,886,2012-07-14,0,6,1,14,41,1,56,1,2,217,84,0,1,0,1,1,12,9856.30886,10482.39374,7.4426159
5545,886,2012-07-14,1,6,1,24.5,35,0.5,60,2,2,217,84,0,1,0,1,1,12,9856.30886,10482.39374,7.44261
5546,886,2012-07-14,2,6,1,21,39,0,60,3,2,217,84,0,1,0,1,1,12,9856.30886,10482.39374,7.4426159
5547,886,2012-07-14,3,6,1,30.3333339691162,29.6666660308838,0,60,3,2,217,84,0,1,0,1,1,12,985
5548,886,2012-07-14,4,6,1,37.3333320617676,21.3333339691162,1.33333337306976,60,3,2,217,8
5549,886,2012-07-15,1,0,1,22,38,0,60,1,2,217,84,0,1,0,1,1,12,9856.30886,10482.39374,7.4426159

5550,886,2012-07-15,2,0,1,40,20,0,60,3,2,217,84,0,1,0,1,1,12,9856.30886,10482.39374,7.4426159[illegible]
5551,886,2012-07-15,3,0,1,42.6666679382324,17.3333339691162,0,60,3,2,217,84,0,1,0,1,1,12,985[illegible]
5552,886,2012-07-15,4,0,1,38,21.3333339691162,0.666666686534882,60,3,2,217,84,0,1,0,1,1,12,9[illegible]
5553,886,2012-07-16,0,1,0,60,0,0,60,1,2,217,84,0,1,0,1,1,12,9856.30886,10482.39374,7.44261595[illegible]
5554,886,2012-07-16,1,1,0,33,25.3333339691162,1.66666662693024,60,3,2,217,84,0,1,0,1,1,12,98[illegible]
5555,886,2012-07-16,2,1,0,26.3333339691162,33.6666679382324,0,60,3,2,217,84,0,1,0,1,1,12,985[illegible]
5556,886,2012-07-16,3,1,0,16.6666660308838,35.6666679382324,7.66666650772095,60,3,2,217,8[illegible]
5557,886,2012-07-17,0,2,0,31,16,0,47,1,2,217,84,0,1,0,1,1,12,9856.30886,10482.39374,7.4426159[illegible]
5558,886,2012-07-17,1,2,0,25,34.3333320617676,0.666666686534882,60,3,2,217,84,0,1,0,1,1,12,9[illegible]
5559,886,2012-07-17,2,2,0,25.3333339691162,34.6666679382324,0,60,3,2,217,84,0,1,0,1,1,12,985[illegible]
5560,886,2012-07-17,3,2,0,13,47,0,60,3,2,217,84,0,1,0,1,1,12,9856.30886,10482.39374,7.4426159[illegible]
5561,886,2012-07-17,4,2,0,30.6000003814697,27.6000003814697,1.79999995231628,60,3,2,217,8[illegible]
5562,886,2012-07-18,0,3,0,5,20,1.5,26.5,2,2,217,84,0,1,0,1,1,12,9856.30886,10482.39374,7.442615[illegible]
5563,886,2012-07-18,1,3,0,15,40,5,60,3,2,217,84,0,1,0,1,1,12,9856.30886,10482.39374,7.4426159[illegible]
5564,886,2012-07-18,2,3,0,7.33333349227905,48.3333320617676,4.33333349227905,60,3,2,217,8[illegible]
5565,886,2012-07-18,3,3,0,17.3333339691162,42,0.666666686534882,60,3,2,217,84,0,1,0,1,1,12,9[illegible]
5566,886,2012-07-18,4,3,0,28,32,0,60,3,2,217,84,0,1,0,1,1,12,9856.30886,10482.39374,7.4426159[illegible]
5567,886,2012-07-19,0,4,0,10.5,47,2.5,60,2,2,217,84,0,1,0,1,1,12,9856.30886,10482.39374,7.44261[illegible]
5568,886,2012-07-19,1,4,0,6.59999990463257,51.4000015258789,2,60,3,2,217,84,0,1,0,1,1,12,985[illegible]
5569,886,2012-07-19,2,4,0,25,35,0,60,3,2,217,84,0,1,0,1,1,12,9856.30886,10482.39374,7.4426159[illegible]
5570,886,2012-07-19,3,4,0,8,51.6666679382324,0.333333343267441,60,3,2,217,84,0,1,0,1,1,12,98[illegible]
5571,886,2012-07-19,4,4,0,30,28,2,60,3,2,217,84,0,1,0,1,1,12,9856.30886,10482.39374,7.4426159[illegible]
5572,886,2012-07-20,0,5,0,10,41.5,1.5,53,2,2,217,84,0,1,0,1,1,12,9856.30886,10482.39374,7.44261[illegible]
5573,886,2012-07-20,1,5,0,22,37.5,0.5,60,3,2,217,84,0,1,0,1,1,12,9856.30886,10482.39374,7.44261[illegible]
5574,886,2012-07-20,2,5,0,19.6666660308838,40,0.333333343267441,60,3,2,217,84,0,1,0,1,1,12,9[illegible]
5575,886,2012-07-20,3,5,0,8,51.6666679382324,0.333333343267441,60,3,2,217,84,0,1,0,1,1,12,98[illegible]
5576,886,2012-07-20,4,5,0,33.3333320617676,26.3333339691162,0.333333343267441,60,3,2,217,[illegible]
5577,887,2012-07-15,0,0,1,29,30,1,60,1,2,217,84,1,1,0,1,1,8,9856.30886,10482.39374,7.44261595[illegible]
5578,887,2012-07-15,1,0,1,14.5,39.5,6,60,2,2,217,84,1,1,0,1,1,8,9856.30886,10482.39374,7.442615[illegible]
5579,887,2012-07-16,0,1,0,15,31,1,47,1,2,217,84,1,1,0,1,1,8,9856.30886,10482.39374,7.44261595[illegible]
5580,887,2012-07-16,1,1,0,9.66666698455811,44.6666679382324,5.66666650772095,60,3,2,217,8[illegible]
5581,887,2012-07-16,2,1,0,26,33,1,60,3,2,217,84,1,1,0,1,1,8,9856.30886,10482.39374,7.44261595[illegible]
5582,887,2012-07-16,3,1,0,16.6666660308838,34.6666679382324,8.66666698455811,60,3,2,217,8[illegible]
5583,887,2012-07-16,4,1,0,32.3333320617676,26.6666660308838,1,60,3,2,217,84,1,1,0,1,1,8,9856[illegible]
5584,887,2012-07-17,0,2,0,0,10,1,11,1,2,217,84,1,1,0,1,1,8,9856.30886,10482.39374,7.442615954,
5585,887,2012-07-17,1,2,0,11,45,4,60,3,2,217,84,1,1,0,1,1,8,9856.30886,10482.39374,7.44261595[illegible]
5586,887,2012-07-17,2,2,0,13,43.5,3.5,60,3,2,217,84,1,1,0,1,1,8,9856.30886,10482.39374,7.442615[illegible]
5587,887,2012-07-17,3,2,0,12.3333330154419,44.6666679382324,3,60,3,2,217,84,1,1,0,1,1,8,9856[illegible]
5588,887,2012-07-17,4,2,0,19,39.3333320617676,1.66666662693024,60,3,2,217,84,1,1,0,1,1,8,985[illegible]
5589,887,2012-07-18,0,3,0,9.5,35.5,1,46,2,2,217,84,1,1,0,1,1,8,9856.30886,10482.39374,7.442615[illegible]
5590,887,2012-07-18,1,3,0,7.33333349227905,34.6666679382324,18,60,3,2,217,84,1,1,0,1,1,8,985[illegible]
5591,887,2012-07-18,2,3,0,15.3333330154419,41,3.66666674613953,60,3,2,217,84,1,1,0,1,1,8,985[illegible]
5592,887,2012-07-18,3,3,0,19.3333339691162,39.6666679382324,1,60,3,2,217,84,1,1,0,1,1,8,9856[illegible]
5593,887,2012-07-18,4,3,0,24.3333339691162,35.6666679382324,0,60,3,2,217,84,1,1,0,1,1,8,9856[illegible]
5594,887,2012-07-19,1,4,0,13,35,1.66666662693024,49.6666679382324,3,2,217,84,1,1,0,1,1,8,985[illegible]
5595,887,2012-07-19,2,4,0,33.7999992370605,24.3999996185303,1.79999995231628,60,3,2,217,8[illegible]
5596,887,2012-07-19,3,4,0,17,31,12,60,3,2,217,84,1,1,0,1,1,8,9856.30886,10482.39374,7.4426159[illegible]
5597,887,2012-07-19,4,4,0,25,31.6666660308838,3.33333325386047,60,3,2,217,84,1,1,0,1,1,8,985[illegible]
5598,887,2012-07-20,0,5,0,49,3,0,52,1,2,217,84,1,1,0,1,1,8,9856.30886,10482.39374,7.442615954,
5599,887,2012-07-20,1,5,0,30,28.6666660308838,1.33333337306976,60,3,2,217,84,1,1,0,1,1,8,985[illegible]

5600,887,2012-07-20,2,5,0,18.3333339691162,41,0.666666686534882,60,3,2,217,84,1,1,0,1,1,8,98
5601,887,2012-07-20,3,5,0,22.2000007629395,34.7999992370605,3,60,3,2,217,84,1,1,0,1,1,8,9856
5602,887,2012-07-20,4,5,0,42.3333320617676,17,0.666666686534882,60,3,2,217,84,1,1,0,1,1,8,98
5603,887,2012-07-25,0,3,0,8.5,25,1,34.5,2,2,217,84,1,1,0,1,1,8,9856.30886,10482.39374,7.442615
5604,887,2012-07-25,1,3,0,14.6666669845581,43.6666679382324,1.6666662693024,60,3,2,217,8
5605,887,2012-07-25,2,3,0,24.3333339691162,35.6666679382324,0,60,3,2,217,84,1,1,0,1,1,8,9856
5606,887,2012-07-25,3,3,0,31.5,26.75,1.75,60,3,2,217,84,1,1,0,1,1,8,9856.30886,10482.39374,7.44
5607,887,2012-07-25,4,3,0,22,35,3,60,3,2,217,84,1,1,0,1,1,8,9856.30886,10482.39374,7.442615954
5608,887,2012-07-26,4,4,0,28.5,31.5,0,60,2,2,217,84,1,1,0,1,1,8,9856.30886,10482.39374,7.442615
5609,889,2012-07-25,1,3,0,41,4,15,60,1,2,217,81,1,1,1,0,1,7,7962.436902,7499.037717,7.5864162
5610,889,2012-07-25,2,3,0,45.6666679382324,3,11.3333330154419,60,3,2,217,81,1,1,1,0,1,7,7962
5611,889,2012-07-25,3,3,0,50,6.33333349227905,3.66666674613953,60,3,2,217,81,1,1,1,0,1,7,796
5612,889,2012-07-25,4,3,0,35.5,1,0,36.5,2,2,217,81,1,1,1,0,1,7,7962.436902,7499.037717,7.586416
5613,889,2012-07-26,0,4,0,3,28,1,32,2,2,217,81,1,1,1,0,1,7,7962.436902,7499.037717,7.58641626
5614,889,2012-07-26,1,4,0,12.6666669845581,46.3333320617676,1,60,3,2,217,81,1,1,1,0,1,7,7962
5615,889,2012-07-26,2,4,0,33.3333320617676,26.3333339691162,0.333333343267441,60,3,2,217,
5616,889,2012-07-26,3,4,0,24,35.6666679382324,0.333333343267441,60,3,2,217,81,1,1,1,0,1,7,79
5617,889,2012-07-26,4,4,0,45,14.3333330154419,0.666666686534882,60,3,2,217,81,1,1,1,0,1,7,79
5618,889,2012-07-27,0,5,0,13,40.6666679382324,0.333333343267441,54,3,2,217,81,1,1,1,0,1,7,79
5619,889,2012-07-27,1,5,0,23.8333339691162,36.1666679382324,0,60,3,2,217,81,1,1,1,0,1,7,7962
5620,889,2012-07-27,2,5,0,42,17.6666660308838,0.333333343267441,60,3,2,217,81,1,1,1,0,1,7,79
5621,889,2012-07-27,3,5,0,34,23.3333339691162,2.66666674613953,60,3,2,217,81,1,1,1,0,1,7,796
5622,889,2012-07-27,4,5,0,29,29,2,60,3,2,217,81,1,1,1,0,1,7,7962.436902,7499.037717,7.5864162
5623,889,2012-07-28,0,6,1,13,43,1,57,2,2,217,81,1,1,1,0,1,7,7962.436902,7499.037717,7.5864162
5624,889,2012-07-28,1,6,1,19.3333339691162,40,0.666666686534882,60,3,2,217,81,1,1,1,0,1,7,79
5625,889,2012-07-28,2,6,1,35.8333320617676,24.1666660308838,0,60,3,2,217,81,1,1,1,0,1,7,7962
5626,889,2012-07-28,3,6,1,41.6666679382324,18.3333339691162,0,60,3,2,217,81,1,1,1,0,1,7,7962
5627,889,2012-07-28,4,6,1,28.6666660308838,31,0.333333343267441,60,3,2,217,81,1,1,1,0,1,7,79
5628,889,2012-07-29,0,0,1,1.5,39,0.5,41,2,2,217,81,1,1,1,0,1,7,7962.436902,7499.037717,7.586416
5629,889,2012-07-29,1,0,1,14,44,2,60,3,2,217,81,1,1,1,0,1,7,7962.436902,7499.037717,7.5864162
5630,889,2012-07-29,2,0,1,28.6666660308838,31,0.333333343267441,60,3,2,217,81,1,1,1,0,1,7,79
5631,889,2012-07-29,3,0,1,38.1666679382324,21.6666660308838,0.16666667163372,60,3,2,217,8
5632,889,2012-07-29,4,0,1,27,33,0,60,3,2,217,81,1,1,1,0,1,7,7962.436902,7499.037717,7.5864162
5633,889,2012-07-30,0,1,0,16.25,37,0.75,54,3,2,217,81,1,1,1,0,1,7,7962.436902,7499.037717,7.58
5634,889,2012-07-30,1,1,0,16.6666660308838,43.3333320617676,0,60,3,2,217,81,1,1,1,0,1,7,7962
5635,889,2012-07-30,2,1,0,39.6666679382324,20,0.333333343267441,60,3,2,217,81,1,1,1,0,1,7,79
5636,889,2012-07-30,3,1,0,21,39,0,60,3,2,217,81,1,1,1,0,1,7,7962.436902,7499.037717,7.5864162
5637,889,2012-07-30,4,1,0,35.4000015258789,24.6000003814697,0,60,3,2,217,81,1,1,1,0,1,7,7962
5638,889,2012-07-31,0,2,0,50,2.5,1,53.5,2,2,217,81,1,1,1,0,1,7,7962.436902,7499.037717,7.586416
5639,889,2012-07-31,1,2,0,30.6000003814697,25.7999992370605,3.59999990463257,60,3,2,217,8
5640,889,2012-07-31,2,2,0,40.3333320617676,19.6666660308838,0,60,3,2,217,81,1,1,1,0,1,7,7962
5641,889,2012-07-31,3,2,0,36,22.6666660308838,1.33333337306976,60,3,2,217,81,1,1,1,0,1,7,796
5642,889,2012-07-31,4,2,0,33,27,0,60,3,2,217,81,1,1,1,0,1,7,7962.436902,7499.037717,7.5864162
5643,895,2012-07-14,0,6,1,3,9,0,12,1,2,217,90,1,1,0,1,1,7,6800.099454,6688.081755,5.960078711,
5644,895,2012-07-14,1,6,1,29.25,22.5,0.75,52.5,3,2,217,90,1,1,0,1,1,7,6800.099454,6688.081755,5
5645,895,2012-07-14,2,6,1,27.3333339691162,32.3333320617676,0.333333343267441,60,3,2,217,
5646,895,2012-07-14,3,6,1,37.6666679382324,22.3333339691162,0,60,3,2,217,90,1,1,0,1,1,7,6800
5647,895,2012-07-14,4,6,1,30.3333339691162,29.6666660308838,0,60,3,2,217,90,1,1,0,1,1,7,6800
5648,895,2012-07-15,1,0,1,20,29.5,1.5,51,2,2,217,90,1,1,0,1,1,7,6800.099454,6688.081755,5.9600
5649,895,2012-07-15,2,0,1,29.3333339691162,30.6666660308838,0,60,3,2,217,90,1,1,0,1,1,7,6800

5650,895,2012-07-15,3,0,1,47.25,12.75,0,60,3,2,217,90,1,1,0,1,1,7,6800.099454,6688.081755,5.960
5651,895,2012-07-15,4,0,1,38.3333320617676,21.6666660308838,0,60,3,2,217,90,1,1,0,1,1,7,6800
5652,895,2012-07-16,0,1,0,31,3,0,34,1,2,217,90,1,1,0,1,1,7,6800.099454,6688.081755,5.96007871
5653,895,2012-07-16,1,1,0,30,29.6666660308838,0.333333343267441,60,3,2,217,90,1,1,0,1,1,7,68
5654,895,2012-07-16,2,1,0,25.3333339691162,34.6666679382324,0,60,3,2,217,90,1,1,0,1,1,7,6800
5655,895,2012-07-16,3,1,0,44.5999984741211,15.3999996185303,0,60,3,2,217,90,1,1,0,1,1,7,6800
5656,895,2012-07-16,4,1,0,29.6666660308838,30.3333339691162,0,60,3,2,217,90,1,1,0,1,1,7,6800
5657,895,2012-07-17,0,2,0,0,1,0,1,1,2,217,90,1,1,0,1,1,7,6800.099454,6688.081755,5.960078711,1
5658,895,2012-07-17,1,2,0,29.3333339691162,19.6666660308838,2,51,3,2,217,90,1,1,0,1,1,7,6800
5659,895,2012-07-17,2,2,0,26.6666660308838,33.3333320617676,0,60,3,2,217,90,1,1,0,1,1,7,6800
5660,895,2012-07-17,3,2,0,47.3333320617676,12.6666669845581,0,60,3,2,217,90,1,1,0,1,1,7,6800
5661,895,2012-07-17,4,2,0,42.5,16.5,1,60,3,2,217,90,1,1,0,1,1,7,6800.099454,6688.081755,5.9600
5662,895,2012-07-18,0,3,0,0,1,0,1,1,2,217,90,1,1,0,1,1,7,6800.099454,6688.081755,5.960078711,1
5663,895,2012-07-18,1,3,0,10.5,44.5,1,56,2,2,217,90,1,1,0,1,1,7,6800.099454,6688.081755,5.9600
5664,895,2012-07-18,2,3,0,18,42,0,60,3,2,217,90,1,1,0,1,1,7,6800.099454,6688.081755,5.9600787
5665,895,2012-07-18,3,3,0,44.6666679382324,15.3333330154419,0,60,3,2,217,90,1,1,0,1,1,7,6800
5666,895,2012-07-18,4,3,0,45,15,0,60,3,2,217,90,1,1,0,1,1,7,6800.099454,6688.081755,5.9600787
5667,895,2012-07-19,0,4,0,1,7,1,9,1,2,217,90,1,1,0,1,1,7,6800.099454,6688.081755,5.960078711,1
5668,895,2012-07-19,1,4,0,27.6666660308838,32.3333320617676,0,60,3,2,217,90,1,1,0,1,1,7,6800
5669,895,2012-07-19,2,4,0,20.75,39.25,0,60,3,2,217,90,1,1,0,1,1,7,6800.099454,6688.081755,5.960
5670,895,2012-07-19,3,4,0,31.6666660308838,25,3.33333325386047,60,3,2,217,90,1,1,0,1,1,7,680
5671,895,2012-07-19,4,4,0,48,12,0,60,3,2,217,90,1,1,0,1,1,7,6800.099454,6688.081755,5.9600787
5672,895,2012-07-20,1,5,0,2,4,1,7,1,2,217,90,1,1,0,1,1,7,6800.099454,6688.081755,5.960078711,1
5673,895,2012-07-20,2,5,0,29.5,30,0.5,60,3,2,217,90,1,1,0,1,1,7,6800.099454,6688.081755,5.9600
5674,895,2012-07-20,3,5,0,17.75,42.25,0,60,3,2,217,90,1,1,0,1,1,7,6800.099454,6688.081755,5.960
5675,895,2012-07-20,4,5,0,21.3333339691162,38.6666679382324,0,60,3,2,217,90,1,1,0,1,1,7,6800
5676,895,2012-07-25,0,3,0,44,11,0,55,1,2,217,90,1,1,0,1,1,7,6800.099454,6688.081755,5.9600787
5677,895,2012-07-25,1,3,0,28.75,16.5,0,45.25,3,2,217,90,1,1,0,1,1,7,6800.099454,6688.081755,5.9
5678,895,2012-07-25,2,3,0,28.5,26.3333339691162,0.333333343267441,55.1666679382324,3,2,21
5679,895,2012-07-25,3,3,0,26.3999996185303,33,0.600000023841858,60,3,2,217,90,1,1,0,1,1,7,68
5680,895,2012-07-25,4,3,0,30,30,0,60,3,2,217,90,1,1,0,1,1,7,6800.099454,6688.081755,5.9600787
5681,895,2012-07-26,1,4,0,28,20.6666660308838,0.333333343267441,49,3,2,217,90,1,1,0,1,1,7,68
5682,895,2012-07-26,2,4,0,19.6666660308838,40,0.333333343267441,60,3,2,217,90,1,1,0,1,1,7,68
5683,895,2012-07-26,3,4,0,34.6666679382324,25.3333339691162,0,60,3,2,217,90,1,1,0,1,1,7,6800
5684,895,2012-07-26,4,4,0,37.3333320617676,22.6666660308838,0,60,3,2,217,90,1,1,0,1,1,7,6800
5685,895,2012-07-27,0,5,0,2,7,1,10,1,2,217,90,1,1,0,1,1,7,6800.099454,6688.081755,5.960078711,
5686,895,2012-07-27,1,5,0,27.5,23.5,0.16666667163372,51.1666679382324,3,2,217,90,1,1,0,1,1,7,
5687,895,2012-07-27,2,5,0,29.3333339691162,30.6666660308838,0,60,3,2,217,90,1,1,0,1,1,7,6800
5688,895,2012-07-27,3,5,0,32.3333320617676,27.6666660308838,0,60,3,2,217,90,1,1,0,1,1,7,6800
5689,895,2012-07-27,4,5,0,45.6666679382324,14.3333330154419,0,60,3,2,217,90,1,1,0,1,1,7,6800
5690,895,2012-07-28,0,6,1,18,10,1,29,1,2,217,90,1,1,0,1,1,7,6800.099454,6688.081755,5.9600787
5691,895,2012-07-28,1,6,1,44.3333320617676,15,0.666666686534882,60,3,2,217,90,1,1,0,1,1,7,68
5692,895,2012-07-28,2,6,1,33.2000007629395,26.7999992370605,0,60,3,2,217,90,1,1,0,1,1,7,6800
5693,895,2012-07-28,3,6,1,37,23,0,60,3,2,217,90,1,1,0,1,1,7,6800.099454,6688.081755,5.9600787
5694,895,2012-07-28,4,6,1,39.6666679382324,20,0.333333343267441,60,3,2,217,90,1,1,0,1,1,7,68
5695,895,2012-07-29,0,0,1,21,13,0.5,34.5,2,2,217,90,1,1,0,1,1,7,6800.099454,6688.081755,5.9600
5696,895,2012-07-29,1,0,1,33,26,1,60,3,2,217,90,1,1,0,1,1,7,6800.099454,6688.081755,5.9600787
5697,895,2012-07-29,2,0,1,30,30,0,60,1,2,217,90,1,1,0,1,1,7,6800.099454,6688.081755,5.9600787
5698,895,2012-07-29,3,0,1,25.6666660308838,34.3333320617676,0,60,3,2,217,90,1,1,0,1,1,7,6800
5699,895,2012-07-29,4,0,1,31,29,0,60,3,2,217,90,1,1,0,1,1,7,6800.099454,6688.081755,5.9600787

5700,895,2012-07-30,0,1,0,22,32,1,55,1,2,217,90,1,1,0,1,1,7,6800.099454,6688.081755,5.96007871
5701,895,2012-07-30,1,1,0,31.6666660308838,28.3333339691162,0,60,3,2,217,90,1,1,0,1,1,7,6800
5702,895,2012-07-30,2,1,0,33.6666679382324,26.3333339691162,0,60,3,2,217,90,1,1,0,1,1,7,6800
5703,895,2012-07-30,3,1,0,35.6666679382324,24,0.333333343267441,60,3,2,217,90,1,1,0,1,1,7,68
5704,895,2012-07-30,4,1,0,44.3333320617676,15.6666669845581,0,60,3,2,217,90,1,1,0,1,1,7,6800
5705,895,2012-07-31,1,2,0,1,9,1,11,1,2,217,90,1,1,0,1,1,7,6800.099454,6688.081755,5.960078711,
5706,895,2012-07-31,2,2,0,27.3333339691162,31.3333339691162,1.33333337306976,60,3,2,217,9
5707,895,2012-07-31,3,2,0,45.5,14.1666669845581,0.333333343267441,60,3,2,217,90,1,1,0,1,1,7,
5708,895,2012-07-31,4,2,0,30.3333339691162,29.1666660308838,0.5,60,3,2,217,90,1,1,0,1,1,7,68
5709,895,2012-08-12,1,0,1,28,23.5,1,52.5,2,2,217,90,1,1,0,1,1,7,6800.099454,6688.081755,5.96007
5710,895,2012-08-12,2,0,1,39,15,0.5,54.5,2,2,217,90,1,1,0,1,1,7,6800.099454,6688.081755,5.96007
5711,895,2012-08-12,3,0,1,39,20.6666660308838,0.333333343267441,60,3,2,217,90,1,1,0,1,1,7,68
5712,895,2012-08-12,4,0,1,35.5,24.5,0,60,3,2,217,90,1,1,0,1,1,7,6800.099454,6688.081755,5.96007
5713,895,2012-08-13,1,1,0,7,53,0,60,1,2,217,90,1,1,0,1,1,7,6800.099454,6688.081755,5.96007871
5714,895,2012-08-13,2,1,0,24,36,0,60,3,2,217,90,1,1,0,1,1,7,6800.099454,6688.081755,5.96007871
5715,895,2012-08-13,3,1,0,35,25,0,60,3,2,217,90,1,1,0,1,1,7,6800.099454,6688.081755,5.96007871
5716,895,2012-08-13,4,1,0,36.3333320617676,23.6666660308838,0,60,3,2,217,90,1,1,0,1,1,7,6800
5717,907,2012-08-12,0,0,1,28,29.3333339691162,2.66666674613953,60,3,2,217,67,0,1,0,1,1,12,78
5718,907,2012-08-12,1,0,1,33,17.6000003814697,1.79999995231628,52.4000015258789,3,2,217,6
5719,907,2012-08-12,2,0,1,25,33.3333320617676,1.66666662693024,60,3,2,217,67,0,1,0,1,1,12,78
5720,907,2012-08-12,3,0,1,9,40.6666679382324,10.3333330154419,60,3,2,217,67,0,1,0,1,1,12,784
5721,907,2012-08-12,4,0,1,21.6666660308838,36,2.33333325386047,60,3,2,217,67,0,1,0,1,1,12,78
5722,907,2012-08-13,0,1,0,18,28,9,55,1,2,217,67,0,1,0,1,1,12,7843.104683,6803.830615,6.443889
5723,907,2012-08-13,1,1,0,45.5,10,0.666666686534882,56.1666679382324,3,2,217,67,0,1,0,1,1,12
5724,907,2012-08-13,2,1,0,46.2222213745117,12.5555553436279,1.22222220897675,60,3,2,217,6
5725,907,2012-08-13,3,1,0,47.75,11.5,0.75,60,3,2,217,67,0,1,0,1,1,12,7843.104683,6803.830615,6.
5726,907,2012-08-13,4,1,0,26.3333339691162,32,1.66666662693024,60,3,2,217,67,0,1,0,1,1,12,78
5727,907,2012-08-14,0,2,0,10,36,7,53,1,2,217,67,0,1,0,1,1,12,7843.104683,6803.830615,6.443889
5728,907,2012-08-14,1,2,0,43,9.16666698455811,1.5,53.6666679382324,3,2,217,67,0,1,0,1,1,12,7
5729,907,2012-08-14,2,2,0,41.6666679382324,17.1666660308838,1.16666662693024,60,3,2,217,6
5730,907,2012-08-14,3,2,0,44.2727279663086,14.0909090042114,1.63636362552643,60,3,2,217,6
5731,907,2012-08-14,4,2,0,18,40.6666679382324,1.33333337306976,60,3,2,217,67,0,1,0,1,1,12,78
5732,907,2012-08-15,0,3,0,21,7,0.5,28.5,2,2,217,67,0,1,0,1,1,12,7843.104683,6803.830615,6.4438
5733,907,2012-08-15,1,3,0,16.5,13.75,3.5,33.75,3,2,217,67,0,1,0,1,1,12,7843.104683,6803.830615,
5734,907,2012-08-15,2,3,0,22.1666660308838,30.5,5.66666650772095,58.3333320617676,3,2,217
5735,907,2012-08-15,3,3,0,47,12.5,0.5,60,3,2,217,67,0,1,0,1,1,12,7843.104683,6803.830615,6.443
5736,907,2012-08-15,4,3,0,29.6666660308838,25.75,4.58333349227905,60,3,2,217,67,0,1,0,1,1,12
5737,907,2012-08-16,0,4,0,18.3333339691162,3.66666674613953,0.666666686534882,22.666666
5738,907,2012-08-16,1,4,0,32,22.6000003814697,2.79999995231628,57.4000015258789,3,2,217,6
5739,907,2012-08-16,2,4,0,42.6666679382324,14.3333330154419,3,60,3,2,217,67,0,1,0,1,1,12,784
5740,907,2012-08-16,3,4,0,45.8333320617676,14,0.16666667163372,60,3,2,217,67,0,1,0,1,1,12,78
5741,907,2012-08-16,4,4,0,39.3333320617676,19.8333339691162,0.833333313465118,60,3,2,217,
5742,907,2012-08-17,0,5,0,36,16.3333339691162,2.66666674613953,55,3,2,217,67,0,1,0,1,1,12,78
5743,907,2012-08-17,1,5,0,21,35,4,60,3,2,217,67,0,1,0,1,1,12,7843.104683,6803.830615,6.443889
5744,907,2012-08-17,2,5,0,43.3333320617676,13.3333330154419,3.33333325386047,60,3,2,217,6
5745,907,2012-08-17,3,5,0,51.3333320617676,8.66666698455811,0,60,3,2,217,67,0,1,0,1,1,12,784
5746,907,2012-08-17,4,5,0,39,21,0,60,3,2,217,67,0,1,0,1,1,12,7843.104683,6803.830615,6.443889
5747,907,2012-08-21,1,2,0,29.5,25,5.5,60,2,2,217,67,0,1,0,1,1,12,7843.104683,6803.830615,6.443
5748,907,2012-08-21,2,2,0,42.6666679382324,9.66666698455811,7.66666650772095,60,3,2,217,6
5749,907,2012-08-21,3,2,0,38.3333320617676,21,0.666666686534882,60,3,2,217,67,0,1,0,1,1,12,7

5750,907,2012-08-21,4,2,0,43,17,0,60,3,2,217,67,0,1,0,1,1,12,7843.104683,6803.830615,6.4438898
5751,909,2012-07-14,1,6,1,15,30,3,48,3,2,217,89,0,1,1,1,1,10,7843.104683,6803.830615,6.4438898
5752,909,2012-07-14,2,6,1,16.6666660308838,43.3333320617676,0,60,3,2,217,89,0,1,1,1,1,10,784
5753,909,2012-07-14,3,6,1,26,33.3333320617676,0.666666686534882,60,3,2,217,89,0,1,1,1,1,10,7
5754,909,2012-07-14,4,6,1,36.4000015258789,22.6000003814697,1,60,3,2,217,89,0,1,1,1,1,10,784
5755,909,2012-07-15,1,0,1,33.5999984741211,18,0.200000002980232,51.7999992370605,3,2,217,
5756,909,2012-07-15,2,0,1,41.3333320617676,18.6666660308838,0,60,3,2,217,89,0,1,1,1,1,10,784
5757,909,2012-07-15,3,0,1,39.3333320617676,20.6666660308838,0,60,3,2,217,89,0,1,1,1,1,10,784
5758,909,2012-07-15,4,0,1,32,26.5,1.5,60,3,2,217,89,0,1,1,1,1,10,7843.104683,6803.830615,6.4438
5759,909,2012-07-16,1,1,0,27.25,19.25,1.75,48.25,3,2,217,89,0,1,1,1,1,10,7843.104683,6803.83061
5760,909,2012-07-16,2,1,0,43.25,16.75,0,60,3,2,217,89,0,1,1,1,1,10,7843.104683,6803.830615,6.44
5761,909,2012-07-16,3,1,0,37,23,0,60,3,2,217,89,0,1,1,1,1,10,7843.104683,6803.830615,6.4438898
5762,909,2012-07-16,4,1,0,36,24,0,60,3,2,217,89,0,1,1,1,1,10,7843.104683,6803.830615,6.4438898
5763,909,2012-07-17,0,2,0,1,4,1,6,1,2,217,89,0,1,1,1,1,10,7843.104683,6803.830615,6.443889833,
5764,909,2012-07-17,1,2,0,38,21.3333339691162,0.666666686534882,60,3,2,217,89,0,1,1,1,1,10,7
5765,909,2012-07-17,2,2,0,48.5999984741211,7.19999980926514,4.19999980926514,60,3,2,217,8
5766,909,2012-07-17,3,2,0,29.75,26.5,3.75,60,3,2,217,89,0,1,1,1,1,10,7843.104683,6803.830615,6.
5767,909,2012-07-17,4,2,0,26.6666660308838,32.6666679382324,0.666666686534882,60,3,2,217,
5768,909,2012-07-18,1,3,0,10,13,1,24,2,2,217,89,0,1,1,1,1,10,7843.104683,6803.830615,6.4438898
5769,909,2012-07-18,2,3,0,36.4000015258789,23.6000003814697,0,60,3,2,217,89,0,1,1,1,1,10,784
5770,909,2012-07-18,3,3,0,40.5999984741211,18.6000003814697,0.800000011920929,60,3,2,217,
5771,909,2012-07-18,4,3,0,36.5,12.5,1.25,50.25,3,2,217,89,0,1,1,1,1,10,7843.104683,6803.830615,
5772,909,2012-07-19,2,4,0,39,21,0,60,1,2,217,89,0,1,1,1,1,10,7843.104683,6803.830615,6.4438898
5773,909,2012-07-19,3,4,0,28.6666660308838,29.6666660308838,1.66666662693024,60,3,2,217,8
5774,909,2012-07-19,4,4,0,32.6666679382324,27.3333339691162,0,60,3,2,217,89,0,1,1,1,1,10,784
5775,909,2012-08-16,1,4,0,26.3333339691162,17.6666660308838,0.333333343267441,44.333332
5776,909,2012-08-16,2,4,0,39,21,0,60,2,2,217,89,0,1,1,1,1,10,7843.104683,6803.830615,6.4438898
5777,909,2012-08-17,1,5,0,23.6666660308838,25,0.333333343267441,49,3,2,217,89,0,1,1,1,1,10,7
5778,909,2012-08-17,2,5,0,24.75,35.25,0,60,3,2,217,89,0,1,1,1,1,10,7843.104683,6803.830615,6.44
5779,909,2012-08-17,3,5,0,33.5,26.3333339691162,0.16666667163372,60,3,2,217,89,0,1,1,1,1,10,7
5780,909,2012-08-17,4,5,0,45.5,14.5,0,60,3,2,217,89,0,1,1,1,1,10,7843.104683,6803.830615,6.4438
5781,909,2012-08-21,0,2,0,12,5,2,19,1,2,217,89,0,1,1,1,1,10,7843.104683,6803.830615,6.44388983
5782,909,2012-08-21,1,2,0,26.6666660308838,29,4.33333349227905,60,3,2,217,89,0,1,1,1,1,10,78
5783,909,2012-08-21,2,2,0,39.3333320617676,20.6666660308838,0,60,3,2,217,89,0,1,1,1,1,10,784
5784,909,2012-08-21,3,2,0,30,29,1,60,3,2,217,89,0,1,1,1,1,10,7843.104683,6803.830615,6.4438898
5785,909,2012-08-21,4,2,0,40.5,19.5,0,60,2,2,217,89,0,1,1,1,1,10,7843.104683,6803.830615,6.4438
5786,918,2012-07-19,0,4,0,2,34,2,38,1,2,217,76,1,1,1,1,1,11,7227.904603,6699.99811,5.785850325
5787,918,2012-07-19,1,4,0,2.66666674613953,55,2.33333325386047,60,3,2,217,76,1,1,1,1,1,11,72
5788,918,2012-07-19,2,4,0,15.3333330154419,38.3333320617676,6.33333349227905,60,3,2,217,7
5789,918,2012-07-20,0,5,0,1,17,4,22,1,2,217,76,1,1,1,1,1,11,7227.904603,6699.99811,5.785850325
5790,918,2012-07-20,1,5,0,7,44,9,60,3,2,217,76,1,1,1,1,1,11,7227.904603,6699.99811,5.785850325
5791,918,2012-07-20,2,5,0,17.6666660308838,39.6666679382324,2.66666674613953,60,3,2,217,7
5792,918,2012-07-20,3,5,0,12.6000003814697,42,5.40000009536743,60,3,2,217,76,1,1,1,1,1,11,72
5793,918,2012-07-20,4,5,0,21.6666660308838,34,4.33333349227905,60,3,2,217,76,1,1,1,1,1,11,72
5794,918,2012-07-25,0,3,0,4,41,2,47,1,2,217,76,1,1,1,1,1,11,7227.904603,6699.99811,5.785850325
5795,918,2012-07-25,1,3,0,13.3333330154419,43.3333320617676,3.33333325386047,60,3,2,217,7
5796,918,2012-07-25,2,3,0,11,43.3333320617676,5.66666650772095,60,3,2,217,76,1,1,1,1,1,11,72
5797,918,2012-07-25,3,3,0,34,24,2,60,2,2,217,76,1,1,1,1,1,11,7227.904603,6699.99811,5.78585032
5798,918,2012-07-25,4,3,0,20.6666660308838,23,3.33333325386047,47,3,2,217,76,1,1,1,1,1,11,72
5799,918,2012-08-01,1,3,0,5.66666650772095,40.3333320617676,2.66666674613953,48.6666679

5800,918,2012-08-01,2,3,0,13,44.6666679382324,2.33333325386047,60,3,2,217,76,1,1,1,1,1,11,72
5801,918,2012-08-01,3,3,0,23.3999996185303,35,1.60000002384186,60,3,2,217,76,1,1,1,1,1,11,72
5802,918,2012-08-01,4,3,0,5.66666650772095,53.6666679382324,0.666666686534882,60,3,2,217,
5803,918,2012-08-02,0,4,0,19,41,0,60,1,2,217,76,1,1,1,1,1,11,7227.904603,6699.99811,5.7858503
5804,918,2012-08-02,1,4,0,6.5,34.5,2.5,43.5,2,2,217,76,1,1,1,1,1,11,7227.904603,6699.99811,5.785
5805,918,2012-08-02,2,4,0,15,42,3,60,3,2,217,76,1,1,1,1,1,11,7227.904603,6699.99811,5.7858503
5806,918,2012-08-02,3,4,0,11.3333330154419,42.3333320617676,6.33333349227905,60,3,2,217,7
5807,918,2012-08-02,4,4,0,11.1666669845581,43.1666679382324,2.5,56.8333320617676,3,2,217,
5808,918,2012-08-03,1,5,0,10.8333330154419,40,6.33333349227905,57.1666679382324,3,2,217,7
5809,918,2012-08-03,2,5,0,15,38.3333320617676,6.66666650772095,60,3,2,217,76,1,1,1,1,1,11,72
5810,918,2012-08-03,3,5,0,7.33333349227905,43.3333320617676,9.33333301544189,60,3,2,217,7
5811,918,2012-08-03,4,5,0,21.6666660308838,35.3333320617676,3,60,3,2,217,76,1,1,1,1,1,11,722
5812,918,2012-08-04,0,6,1,8,43,4,55,1,2,217,76,1,1,1,1,1,11,7227.904603,6699.99811,5.78585032
5813,918,2012-08-04,1,6,1,21.3333339691162,35.3333320617676,3.33333325386047,60,3,2,217,7
5814,918,2012-08-04,2,6,1,17.3333339691162,38.6666679382324,4,60,3,2,217,76,1,1,1,1,1,11,722
5815,918,2012-08-04,3,6,1,7.66666650772095,47.6666679382324,4.66666650772095,60,3,2,217,7
5816,918,2012-08-04,4,6,1,16.3333339691162,34,9.66666698455811,60,3,2,217,76,1,1,1,1,1,11,72
5817,918,2012-08-05,2,0,1,7,48,5,60,3,2,217,76,1,1,1,1,1,11,7227.904603,6699.99811,5.78585032
5818,918,2012-08-05,3,0,1,22,37.3333320617676,0.666666686534882,60,3,2,217,76,1,1,1,1,1,11,7
5819,918,2012-08-05,4,0,1,28,31,1,60,3,2,217,76,1,1,1,1,1,11,7227.904603,6699.99811,5.7858503
5820,930,2012-07-31,0,2,0,1,17,4,22,1,2,217,79,1,1,0,1,1,8,7843.104683,6803.830615,6.44388983
5821,930,2012-07-31,1,2,0,7,44,9,60,3,2,217,79,1,1,0,1,1,8,7843.104683,6803.830615,6.44388983
5822,930,2012-07-31,2,2,0,16,38,6,60,3,2,217,79,1,1,0,1,1,8,7843.104683,6803.830615,6.4438898
5823,930,2012-07-31,3,2,0,13.6666669845581,37.3333320617676,9,60,3,2,217,79,1,1,0,1,1,8,7843
5824,930,2012-07-31,4,2,0,21.6666660308838,34,4.33333349227905,60,3,2,217,79,1,1,0,1,1,8,784
5825,930,2012-08-05,0,0,1,2,34,2,38,1,2,217,79,1,1,0,1,1,8,7843.104683,6803.830615,6.44388983
5826,930,2012-08-05,1,0,1,2.66666674613953,55,2.33333325386047,60,3,2,217,79,1,1,0,1,1,8,784
5827,930,2012-08-05,2,0,1,10,49,1,60,1,2,217,79,1,1,0,1,1,8,7843.104683,6803.830615,6.4438898
5828,930,2012-08-10,0,5,0,4,41,2,47,1,2,217,79,1,1,0,1,1,8,7843.104683,6803.830615,6.44388983
5829,930,2012-08-10,1,5,0,13.3333330154419,43.3333320617676,3.33333325386047,60,3,2,217,7
5830,930,2012-08-10,2,5,0,14.5,40.75,4.75,60,3,2,217,79,1,1,0,1,1,8,7843.104683,6803.830615,6.4
5831,930,2012-08-10,3,5,0,20.6666660308838,39.3333320617676,0,60,3,2,217,79,1,1,0,1,1,8,7843
5832,930,2012-08-10,4,5,0,20.6666660308838,23,3.33333325386047,47,3,2,217,79,1,1,0,1,1,8,784
5833,930,2012-08-12,1,0,1,5.66666650772095,40.3333320617676,2.66666674613953,48.6666679
5834,930,2012-08-12,2,0,1,13,44.6666679382324,2.33333325386047,60,3,2,217,79,1,1,0,1,1,8,784
5835,930,2012-08-12,3,0,1,28.5,30,1.5,60,3,2,217,79,1,1,0,1,1,8,7843.104683,6803.830615,6.4438
5836,930,2012-08-12,4,0,1,5.66666650772095,53.6666679382324,0.666666686534882,60,3,2,217,
5837,930,2012-08-13,1,1,0,6.5,34.5,2.5,43.5,2,2,217,79,1,1,0,1,1,8,7843.104683,6803.830615,6.443
5838,930,2012-08-13,2,1,0,15,42,3,60,3,2,217,79,1,1,0,1,1,8,7843.104683,6803.830615,6.4438898
5839,930,2012-08-13,3,1,0,13,42,5,60,3,2,217,79,1,1,0,1,1,8,7843.104683,6803.830615,6.4438898
5840,930,2012-08-13,4,1,0,15,43.25,1.75,60,3,2,217,79,1,1,0,1,1,8,7843.104683,6803.830615,6.443
5841,930,2012-08-14,0,2,0,19,41,0,60,1,2,217,79,1,1,0,1,1,8,7843.104683,6803.830615,6.4438898
5842,930,2012-08-14,1,2,0,8.33333301544189,44.3333320617676,7.33333349227905,60,3,2,217,7
5843,930,2012-08-14,2,2,0,15,38.3333320617676,6.66666650772095,60,3,2,217,79,1,1,0,1,1,8,784
5844,930,2012-08-14,3,2,0,7.33333349227905,43.3333320617676,9.33333301544189,60,3,2,217,7
5845,930,2012-08-14,4,2,0,14.3999996185303,38.4000015258789,3.40000009536743,56.2000007
5846,930,2012-08-15,0,3,0,8,43,4,55,1,2,217,79,1,1,0,1,1,8,7843.104683,6803.830615,6.44388983
5847,930,2012-08-15,1,3,0,13.3333330154419,35.6666679382324,5.33333349227905,54.333332
5848,930,2012-08-15,2,3,0,17.3333339691162,38.6666679382324,4,60,3,2,217,79,1,1,0,1,1,8,7843
5849,930,2012-08-15,3,3,0,7.66666650772095,47.6666679382324,4.66666650772095,60,3,2,217,7

5850,930,2012-08-15,4,3,0,16.3333339691162,34,9.66666698455811,60,3,2,217,79,1,1,0,1,1,8,784
5851,930,2012-08-16,1,4,0,21.3333339691162,35.3333320617676,3.33333325386047,60,3,2,217,7
5852,930,2012-08-16,2,4,0,7,48,5,60,3,2,217,79,1,1,0,1,1,8,7843.104683,6803.830615,6.4438898
5853,930,2012-08-16,3,4,0,22,37.3333320617676,0.666666686534882,60,3,2,217,79,1,1,0,1,1,8,78
5854,930,2012-08-16,4,4,0,28,31,1,60,3,2,217,79,1,1,0,1,1,8,7843.104683,6803.830615,6.4438898
5855,937,2012-07-27,4,5,0,17,39.6666679382324,3.33333325386047,60,3,2,217,76,1,1,1,1,1,9,739
5856,937,2012-08-01,0,3,0,35.6666679382324,21.3333339691162,0,57,3,2,217,76,1,1,1,1,1,9,7396
5857,937,2012-08-01,1,3,0,22,34.3333320617676,3.66666674613953,60,3,2,217,76,1,1,1,1,1,9,739
5858,937,2012-08-01,2,3,0,14.3333330154419,42.6666679382324,3,60,3,2,217,76,1,1,1,1,1,9,7396
5859,937,2012-08-01,3,3,0,10.3333330154419,36.6666679382324,0.333333343267441,47.333332
5860,937,2012-08-01,4,3,0,4.33333349227905,46,9.66666698455811,60,3,2,217,76,1,1,1,1,1,9,739
5861,937,2012-08-02,0,4,0,43,5,0,48,1,2,217,76,1,1,1,1,1,9,7396.303897,6782.338417,5.83536613
5862,937,2012-08-02,1,4,0,4.33333349227905,30.6666660308838,8.66666698455811,43.666667
5863,937,2012-08-02,2,4,0,9.33333301544189,45.6666679382324,5,60,3,2,217,76,1,1,1,1,1,9,7396
5864,937,2012-08-02,3,4,0,5.75,42.5,8.75,57,3,2,217,76,1,1,1,1,1,9,7396.303897,6782.338417,5.83
5865,937,2012-08-02,4,4,0,6.33333349227905,45.3333320617676,8.33333301544189,60,3,2,217,7
5866,937,2012-08-16,0,4,0,42,14.5,0.5,57,2,2,217,76,1,1,1,1,1,9,7396.303897,6782.338417,5.8353
5867,937,2012-08-17,0,5,0,25,19.3333339691162,0.666666686534882,45,3,2,217,76,1,1,1,1,1,9,73
5868,937,2012-08-17,1,5,0,21.7999992370605,31,7.19999980926514,60,3,2,217,76,1,1,1,1,1,9,739
5869,937,2012-08-17,2,5,0,4.66666650772095,39.3333320617676,16,60,3,2,217,76,1,1,1,1,1,9,739
5870,937,2012-08-17,3,5,0,12.3333330154419,36.6666679382324,11,60,3,2,217,76,1,1,1,1,1,9,739
5871,937,2012-08-17,4,5,0,30.3333339691162,22.6666660308838,7,60,3,2,217,76,1,1,1,1,1,9,7396
5872,937,2012-08-18,0,6,1,20,36.3333320617676,3.66666674613953,60,3,2,217,76,1,1,1,1,1,9,739
5873,937,2012-08-18,1,6,1,4.66666650772095,40.3333320617676,12,57,3,2,217,76,1,1,1,1,1,9,739
5874,937,2012-08-18,2,6,1,7.59999990463257,38.4000015258789,13.1999998092651,59.200000
5875,937,2012-08-18,3,6,1,8.33333301544189,38.6666679382324,13,60,3,2,217,76,1,1,1,1,1,9,739
5876,937,2012-08-18,4,6,1,17.6666660308838,37.6666679382324,4.66666650772095,60,3,2,217,7
5877,937,2012-08-19,0,0,1,5,51,4,60,1,2,217,76,1,1,1,1,1,9,7396.303897,6782.338417,5.83536613
5878,937,2012-08-19,1,0,1,18,26.3333339691162,15.6666669845581,60,3,2,217,76,1,1,1,1,1,9,739
5879,937,2012-08-19,2,0,1,6,39,15,60,3,2,217,76,1,1,1,1,1,9,7396.303897,6782.338417,5.8353661
5880,937,2012-08-19,3,0,1,12.5,40.3333320617676,7.16666650772095,60,3,2,217,76,1,1,1,1,1,9,73
5881,937,2012-08-19,4,0,1,3.33333325386047,39,17.6666660308838,60,3,2,217,76,1,1,1,1,1,9,739
5882,937,2012-08-20,0,1,0,18,27.3333339691162,7.66666650772095,53,3,2,217,76,1,1,1,1,1,9,739
5883,937,2012-08-20,1,1,0,9,36,15,60,3,2,217,76,1,1,1,1,1,9,7396.303897,6782.338417,5.8353661
5884,937,2012-08-20,2,1,0,7,18,6.5,31.5,2,2,217,76,1,1,1,1,1,9,7396.303897,6782.338417,5.83536
5885,937,2012-08-20,3,1,0,21.5,27,0.5,49,2,2,217,76,1,1,1,1,1,9,7396.303897,6782.338417,5.83536
5886,937,2012-08-20,4,1,0,23.3333339691162,32,4.66666650772095,60,3,2,217,76,1,1,1,1,1,9,739
5887,937,2012-08-21,0,2,0,22.6666660308838,29.3333339691162,4.33333349227905,56.333332
5888,937,2012-08-21,1,2,0,21,36.6666679382324,2.33333325386047,60,3,2,217,76,1,1,1,1,1,9,739
5889,937,2012-08-21,2,2,0,34.3333320617676,22,3.66666674613953,60,3,2,217,76,1,1,1,1,1,9,739
5890,937,2012-08-21,3,2,0,31.5,25.5,3,60,2,2,217,76,1,1,1,1,1,9,7396.303897,6782.338417,5.83536
5891,937,2012-08-21,4,2,0,28.3333339691162,31.3333339691162,0.333333343267441,60,3,2,217,
5892,949,2012-07-27,0,5,0,10,40,0.5,50.5,2,2,217,75,1,0,0,1,1,6,9090.788545,6986.262374,6.50706
5893,949,2012-07-27,1,5,0,1,56.6666679382324,2.33333325386047,60,3,2,217,75,1,0,0,1,1,6,9090
5894,949,2012-07-27,2,5,0,6.66666650772095,53,0.333333343267441,60,3,2,217,75,1,0,0,1,1,6,90
5895,949,2012-07-27,3,5,0,18.6666660308838,41.3333320617676,0,60,3,2,217,75,1,0,0,1,1,6,9090
5896,949,2012-07-27,4,5,0,9,49,2,60,1,2,217,75,1,0,0,1,1,6,9090.788545,6986.262374,6.50706489
5897,949,2012-07-28,0,6,1,18.5,19.5,0,38,2,2,217,75,1,0,0,1,1,6,9090.788545,6986.262374,6.50706
5898,949,2012-07-28,1,6,1,23,31,6,60,3,2,217,75,1,0,0,1,1,6,9090.788545,6986.262374,6.5070648
5899,949,2012-07-28,2,6,1,8.66666698455811,49.3333320617676,2,60,3,2,217,75,1,0,0,1,1,6,9090

5900,949,2012-07-28,3,6,1,31,29,0,60,3,2,217,75,1,0,0,1,1,6,9090.788545,6986.262374,6.50706489
5901,949,2012-07-28,4,6,1,12,46.75,1.25,60,3,2,217,75,1,0,0,1,1,6,9090.788545,6986.262374,6.507
5902,949,2012-07-29,0,0,1,8,29.5,0,37.5,2,2,217,75,1,0,0,1,1,6,9090.788545,6986.262374,6.507064
5903,949,2012-07-29,1,0,1,8.33333301544189,51.6666679382324,0,60,3,2,217,75,1,0,0,1,1,6,9090
5904,949,2012-07-29,2,0,1,18.3333339691162,41.6666679382324,0,60,3,2,217,75,1,0,0,1,1,6,9090
5905,949,2012-07-29,3,0,1,22,37.6666679382324,0.333333343267441,60,3,2,217,75,1,0,0,1,1,6,90
5906,949,2012-07-29,4,0,1,14.75,44,1.25,60,3,2,217,75,1,0,0,1,1,6,9090.788545,6986.262374,6.507
5907,949,2012-07-30,0,1,0,16.6666660308838,24,0.666666686534882,41.3333320617676,3,2,217,
5908,949,2012-07-30,1,1,0,25,34.75,0.25,60,3,2,217,75,1,0,0,1,1,6,9090.788545,6986.262374,6.507
5909,949,2012-07-30,2,1,0,22.6666660308838,37.3333320617676,0,60,3,2,217,75,1,0,0,1,1,6,9090
5910,949,2012-07-30,3,1,0,11.6666669845581,47.6666679382324,0.666666686534882,60,3,2,217,
5911,949,2012-07-30,4,1,0,24.6666660308838,35.3333320617676,0,60,3,2,217,75,1,0,0,1,1,6,9090
5912,949,2012-07-31,0,2,0,5,55,0,60,1,2,217,75,1,0,0,1,1,6,9090.788545,6986.262374,6.50706489
5913,949,2012-07-31,1,2,0,16.75,41.5,1.75,60,3,2,217,75,1,0,0,1,1,6,9090.788545,6986.262374,6.5
5914,949,2012-07-31,2,2,0,14.6666669845581,45.3333320617676,0,60,3,2,217,75,1,0,0,1,1,6,9090
5915,949,2012-07-31,3,2,0,15.6666669845581,39.3333320617676,5,60,3,2,217,75,1,0,0,1,1,6,9090
5916,949,2012-07-31,4,2,0,12.6666669845581,47.3333320617676,0,60,3,2,217,75,1,0,0,1,1,6,9090
5917,949,2012-08-14,0,2,0,18,23,0,41,2,2,217,75,1,0,0,1,1,6,9090.788545,6986.262374,6.50706489
5918,949,2012-08-14,1,2,0,14,46,0,60,3,2,217,75,1,0,0,1,1,6,9090.788545,6986.262374,6.50706489
5919,949,2012-08-14,2,2,0,22,38,0,60,3,2,217,75,1,0,0,1,1,6,9090.788545,6986.262374,6.50706489
5920,949,2012-08-14,3,2,0,17,42.3333320617676,0.666666686534882,60,3,2,217,75,1,0,0,1,1,6,90
5921,949,2012-08-14,4,2,0,20.6666660308838,39.3333320617676,0,60,3,2,217,75,1,0,0,1,1,6,9090
5922,949,2012-08-15,2,3,0,13.5,44,2.5,60,2,2,217,75,1,0,0,1,1,6,9090.788545,6986.262374,6.50706
5923,949,2012-08-15,3,3,0,5.66666650772095,52,2.33333325386047,60,3,2,217,75,1,0,0,1,1,6,909
5924,949,2012-08-15,4,3,0,37.3333320617676,22.3333339691162,0.333333343267441,60,3,2,217,
5925,955,2012-08-15,0,3,0,55,2,3,60,1,2,217,77,0,1,0,1,1,8,9922.740955,9707.536865,7.33224371
5926,955,2012-08-15,1,3,0,23,35.6666679382324,1.33333337306976,60,3,2,217,77,0,1,0,1,1,8,992
5927,955,2012-08-15,2,3,0,35.6666679382324,22.5,1.83333337306976,60,3,2,217,77,0,1,0,1,1,8,99
5928,955,2012-08-15,3,3,0,9,47.3333320617676,3.66666674613953,60,3,2,217,77,0,1,0,1,1,8,9922
5929,955,2012-08-15,4,3,0,33.6666679382324,22,4.33333349227905,60,3,2,217,77,0,1,0,1,1,8,992
5930,955,2012-08-16,0,4,0,19.6666660308838,17.6666660308838,2,39.3333320617676,3,2,217,77
5931,955,2012-08-16,1,4,0,21.2000007629395,33.2000007629395,5.59999990463257,60,3,2,217,7
5932,955,2012-08-16,2,4,0,35.6666679382324,23.6666660308838,0.666666686534882,60,3,2,217,
5933,955,2012-08-16,3,4,0,31.6666660308838,27.6666660308838,0.666666686534882,60,3,2,217,
5934,955,2012-08-16,4,4,0,30.3333339691162,29,0.666666686534882,60,3,2,217,77,0,1,0,1,1,8,99
5935,955,2012-08-17,0,5,0,0,8,4,12,1,2,217,77,0,1,0,1,1,8,9922.740955,9707.536865,7.332243713,
5936,955,2012-08-17,1,5,0,21.25,35,3.75,60,3,2,217,77,0,1,0,1,1,8,9922.740955,9707.536865,7.332
5937,955,2012-08-17,2,5,0,40.1666679382324,19.1666660308838,0.666666686534882,60,3,2,217,
5938,955,2012-08-17,3,5,0,25.3999996185303,30,4.59999990463257,60,3,2,217,77,0,1,0,1,1,8,992
5939,955,2012-08-17,4,5,0,21,36,3,60,3,2,217,77,0,1,0,1,1,8,9922.740955,9707.536865,7.33224371
5940,955,2012-08-18,0,6,1,10,12,1,23,1,2,217,77,0,1,0,1,1,8,9922.740955,9707.536865,7.33224371
5941,955,2012-08-18,1,6,1,21.6666660308838,37,1.33333337306976,60,3,2,217,77,0,1,0,1,1,8,992
5942,955,2012-08-18,2,6,1,41.3333320617676,18.6666660308838,0,60,3,2,217,77,0,1,0,1,1,8,9922
5943,955,2012-08-18,3,6,1,25,33.25,1.75,60,3,2,217,77,0,1,0,1,1,8,9922.740955,9707.536865,7.332
5944,955,2012-08-18,4,6,1,33,24.3999996185303,2.59999990463257,60,3,2,217,77,0,1,0,1,1,8,992
5945,955,2012-08-19,0,0,1,8.5,16.5,1.5,26.5,2,2,217,77,0,1,0,1,1,8,9922.740955,9707.536865,7.332
5946,955,2012-08-19,1,0,1,19.6000003814697,38.2000007629395,2.20000004768372,60,3,2,217,7
5947,955,2012-08-19,2,0,1,32.3333320617676,27.6666660308838,0,60,3,2,217,77,0,1,0,1,1,8,9922
5948,955,2012-08-19,3,0,1,33,26.6666660308838,0.333333343267441,60,3,2,217,77,0,1,0,1,1,8,99
5949,955,2012-08-19,4,0,1,29,28.75,2.25,60,3,2,217,77,0,1,0,1,1,8,9922.740955,9707.536865,7.332

5950,955,2012-08-20,0,1,0,16,20,0,36,1,2,217,77,0,1,0,1,1,8,9922.740955,9707.536865,7.3322437
5951,955,2012-08-20,1,1,0,10,46.75,3.25,60,3,2,217,77,0,1,0,1,1,8,9922.740955,9707.536865,7.332
5952,955,2012-08-20,2,1,0,21.3333339691162,38,0.666666686534882,60,3,2,217,77,0,1,0,1,1,8,99
5953,955,2012-08-20,3,1,0,13,46.6666679382324,0.333333343267441,60,3,2,217,77,0,1,0,1,1,8,99
5954,955,2012-08-20,4,1,0,24.6666660308838,35.3333320617676,0,60,3,2,217,77,0,1,0,1,1,8,9922
5955,1098,2012-08-01,0,3,0,28,32,0,60,1,2,218,90,1,0,1,0,1,4,4512.627589,4528.536465,10.14112
5956,1098,2012-08-01,1,3,0,33,26.3333339691162,0.666666686534882,60,3,2,218,90,1,0,1,0,1,4,4
5957,1098,2012-08-01,2,3,0,52.3333320617676,7,0.666666686534882,60,3,2,218,90,1,0,1,0,1,4,45
5958,1098,2012-08-01,3,3,0,43.6666679382324,16.3333339691162,0,60,3,2,218,90,1,0,1,0,1,4,451
5959,1098,2012-08-01,4,3,0,38.3333320617676,21.6666660308838,0,60,3,2,218,90,1,0,1,0,1,4,451
5960,1098,2012-08-02,0,4,0,16,23.3333339691162,1,40.3333320617676,3,2,218,90,1,0,1,0,1,4,451
5961,1098,2012-08-02,1,4,0,44.3333320617676,15.3333330154419,0.333333343267441,60,3,2,218
5962,1098,2012-08-02,2,4,0,42,18,0,60,3,2,218,90,1,0,1,0,1,4,4512.627589,4528.536465,10.14112
5963,1098,2012-08-02,3,4,0,36.6666679382324,16.3333339691162,7,60,3,2,218,90,1,0,1,0,1,4,451
5964,1098,2012-08-02,4,4,0,27,26.3333339691162,6.66666650772095,60,3,2,218,90,1,0,1,0,1,4,45
5965,1098,2012-08-03,0,5,0,1.33333337306976,3.33333325386047,0.333333343267441,5,3,2,218,
5966,1098,2012-08-03,1,5,0,34,25.6666660308838,0.333333343267441,60,3,2,218,90,1,0,1,0,1,4,4
5967,1098,2012-08-03,2,5,0,47.6666679382324,12.3333330154419,0,60,3,2,218,90,1,0,1,0,1,4,451
5968,1098,2012-08-03,3,5,0,37.6666679382324,20,2.33333325386047,60,3,2,218,90,1,0,1,0,1,4,45
5969,1098,2012-08-03,4,5,0,40,20,0,60,3,2,218,90,1,0,1,0,1,4,4512.627589,4528.536465,10.14112
5970,1098,2012-08-04,0,6,1,7,4.33333349227905,0.666666686534882,12,3,2,218,90,1,0,1,0,1,4,45
5971,1098,2012-08-04,1,6,1,33,26.3333339691162,0.666666686534882,60,3,2,218,90,1,0,1,0,1,4,4
5972,1098,2012-08-04,2,6,1,50,9.66666698455811,0.333333343267441,60,3,2,218,90,1,0,1,0,1,4,4
5973,1098,2012-08-04,3,6,1,51,9,0,60,3,2,218,90,1,0,1,0,1,4,4512.627589,4528.536465,10.141122
5974,1098,2012-08-04,4,6,1,30.6666660308838,28.6666660308838,0.666666686534882,60,3,2,218
5975,1098,2012-08-05,0,7,1,0,0.666666686534882,0.333333343267441,1,3,2,218,90,1,0,1,0,1,4,45
5976,1098,2012-08-05,1,7,1,37,23,0,60,3,2,218,90,1,0,1,0,1,4,4512.627589,4528.536465,10.14112
5977,1098,2012-08-05,2,7,1,50.6666679382324,9.33333301544189,0,60,3,2,218,90,1,0,1,0,1,4,451
5978,1098,2012-08-05,3,7,1,40.6666679382324,18,1.33333337306976,60,3,2,218,90,1,0,1,0,1,4,45
5979,1098,2012-08-05,4,7,1,36.3333320617676,22.6666660308838,1,60,3,2,218,90,1,0,1,0,1,4,451
5980,1098,2012-08-06,0,1,0,8.33333301544189,4.33333349227905,0.333333343267441,13,3,2,218
5981,1098,2012-08-06,1,1,0,38.3333320617676,21.6666660308838,0,60,3,2,218,90,1,0,1,0,1,4,451
5982,1098,2012-08-06,2,1,0,35.6666679382324,24,0.333333343267441,60,3,2,218,90,1,0,1,0,1,4,4
5983,1098,2012-08-06,3,1,0,35.3333320617676,24.6666660308838,0,60,3,2,218,90,1,0,1,0,1,4,451
5984,1098,2012-08-06,4,1,0,41.3333320617676,18,0.666666686534882,60,3,2,218,90,1,0,1,0,1,4,4
5985,1098,2012-08-07,0,2,0,4.66666650772095,12.3333330154419,0.333333343267441,17.333333
5986,1098,2012-08-07,1,2,0,29.3333339691162,30.6666660308838,0,60,3,2,218,90,1,0,1,0,1,4,451
5987,1098,2012-08-07,2,2,0,46.3333320617676,13.6666669845581,0,60,3,2,218,90,1,0,1,0,1,4,451
5988,1098,2012-08-07,3,2,0,37,20.3333339691162,2.66666674613953,60,3,2,218,90,1,0,1,0,1,4,45
5989,1098,2012-08-07,4,2,0,38.6666679382324,21.3333339691162,0,60,3,2,218,90,1,0,1,0,1,4,451
5990,1100,2012-08-15,0,3,0,16,31,13,60,1,2,218,69,0,1,0,1,1,12,4777.074849,4408.728467,15.3391
5991,1100,2012-08-15,1,3,0,15.6666669845581,37,7.33333349227905,60,3,2,218,69,0,1,0,1,1,12,4
5992,1100,2012-08-15,2,3,0,20.3333339691162,36.6666679382324,3,60,3,2,218,69,0,1,0,1,1,12,47
5993,1100,2012-08-16,0,4,0,15.5,16.5,1,33,2,2,218,69,0,1,0,1,1,12,4777.074849,4408.728467,15.33
5994,1100,2012-08-16,1,4,0,17.3333339691162,29,13.6666669845581,60,3,2,218,69,0,1,0,1,1,12,4
5995,1100,2012-08-16,2,4,0,28.3333339691162,30,1.66666662693024,60,3,2,218,69,0,1,0,1,1,12,4
5996,1100,2012-08-16,3,4,0,41.1666679382324,18.1666660308838,0.666666686534882,60,3,2,218
5997,1100,2012-08-16,4,4,0,31.3333339691162,26.6666660308838,2,60,3,2,218,69,0,1,0,1,1,12,47
5998,1100,2012-08-17,0,5,0,11.5,12.5,15,39,2,2,218,69,0,1,0,1,1,12,4777.074849,4408.728467,15.3
5999,1100,2012-08-17,1,5,0,24.6666660308838,26.3333339691162,9,60,3,2,218,69,0,1,0,1,1,12,47

6000,1100,2012-08-17,2,5,0,33.6666679382324,14.3333330154419,12,60,3,2,218,69,0,1,0,1,1,12,4
6001,1100,2012-08-17,3,5,0,39.3333320617676,10,10.6666669845581,60,3,2,218,69,0,1,0,1,1,12,4
6002,1100,2012-08-17,4,5,0,25,25,10,60,3,2,218,69,0,1,0,1,1,12,4777.074849,4408.728467,15.3391
6003,1100,2012-08-18,0,6,1,12,22,3,37,1,2,218,69,0,1,0,1,1,12,4777.074849,4408.728467,15.33913
6004,1100,2012-08-18,1,6,1,19,33.3333320617676,7.66666650772095,60,3,2,218,69,0,1,0,1,1,12,4
6005,1100,2012-08-18,2,6,1,24,33.3333320617676,2.66666674613953,60,3,2,218,69,0,1,0,1,1,12,4
6006,1100,2012-08-18,3,6,1,36.3333320617676,21,2.66666674613953,60,3,2,218,69,0,1,0,1,1,12,4
6007,1100,2012-08-18,4,6,1,24.3999996185303,31.2000007629395,4.40000009536743,60,3,2,218,
6008,1100,2012-08-19,0,0,1,12,11.5,1.5,25,2,2,218,69,0,1,0,1,1,12,4777.074849,4408.728467,15.33
6009,1100,2012-08-19,1,0,1,10,42.3333320617676,7.66666650772095,60,3,2,218,69,0,1,0,1,1,12,4
6010,1100,2012-08-19,2,0,1,35.6666679382324,23.6666660308838,0.666666686534882,60,3,2,218
6011,1100,2012-08-19,3,0,1,35.6666679382324,23,1.33333337306976,60,3,2,218,69,0,1,0,1,1,12,4
6012,1100,2012-08-19,4,0,1,40,17.5,2.5,60,3,2,218,69,0,1,0,1,1,12,4777.074849,4408.728467,15.33
6013,1100,2012-08-20,0,1,0,40,16,1,57,1,2,218,69,0,1,0,1,1,12,4777.074849,4408.728467,15.33913
6014,1100,2012-08-20,1,1,0,18.25,21,13.75,53,3,2,218,69,0,1,0,1,1,12,4777.074849,4408.728467,1
6015,1100,2012-08-20,2,1,0,31.7999992370605,16,2.20000004768372,50,3,2,218,69,0,1,0,1,1,12,4
6016,1100,2012-08-20,3,1,0,33.3333320617676,23.6666660308838,3,60,3,2,218,69,0,1,0,1,1,12,47
6017,1100,2012-08-20,4,1,0,31.3333339691162,27,1.66666662693024,60,3,2,218,69,0,1,0,1,1,12,4
6018,1100,2012-08-21,3,2,0,29.6666660308838,24.6666660308838,5.66666650772095,60,3,2,218,
6019,1100,2012-08-21,4,2,0,34,24,2,60,3,2,218,69,0,1,0,1,1,12,4777.074849,4408.728467,15.33913
6020,1102,2012-08-15,0,3,0,27,32,1,60,1,2,218,84,0,1,1,1,1,12,4542.304285,4594.983269,10.09726
6021,1102,2012-08-15,1,3,0,18,26.3333339691162,2,46.3333320617676,3,2,218,84,0,1,1,1,1,12,45
6022,1102,2012-08-15,2,3,0,40.6666679382324,19,0.333333343267441,60,3,2,218,84,0,1,1,1,1,12,
6023,1102,2012-08-15,3,3,0,18.3333339691162,34.3333320617676,7.33333349227905,60,3,2,218,
6024,1102,2012-08-15,4,3,0,36,23.6000003814697,0,59.5999984741211,3,2,218,84,0,1,1,1,1,12,45
6025,1102,2012-08-16,0,4,0,10.5,46.5,3,60,2,2,218,84,0,1,1,1,1,12,4542.304285,4594.983269,10.09
6026,1102,2012-08-16,1,4,0,12,37,9,58,3,2,218,84,0,1,1,1,1,12,4542.304285,4594.983269,10.09726
6027,1102,2012-08-16,2,4,0,29.6666660308838,30,0.333333343267441,60,3,2,218,84,0,1,1,1,1,12,
6028,1102,2012-08-16,3,4,0,23.3333339691162,30.6666660308838,6,60,3,2,218,84,0,1,1,1,1,12,45
6029,1102,2012-08-16,4,4,0,31.3333339691162,26.6666660308838,2,60,3,2,218,84,0,1,1,1,1,12,45
6030,1102,2012-08-17,0,5,0,20,7,1,28,1,2,218,84,0,1,1,1,1,12,4542.304285,4594.983269,10.097260
6031,1102,2012-08-17,1,5,0,13.5,31.5,8.25,53.25,3,2,218,84,0,1,1,1,1,12,4542.304285,4594.983269
6032,1102,2012-08-17,2,5,0,22.5,37.25,0.25,60,3,2,218,84,0,1,1,1,1,12,4542.304285,4594.983269,1
6033,1102,2012-08-17,3,5,0,32,27,1,60,3,2,218,84,0,1,1,1,1,12,4542.304285,4594.983269,10.09726
6034,1102,2012-08-17,4,5,0,39,19,2,60,3,2,218,84,0,1,1,1,1,12,4542.304285,4594.983269,10.09726
6035,1102,2012-08-18,1,6,1,25,21.3333339691162,0,46.3333320617676,3,2,218,84,0,1,1,1,1,12,45
6036,1102,2012-08-18,2,6,1,35.7999992370605,24.2000007629395,0,60,3,2,218,84,0,1,1,1,1,12,45
6037,1102,2012-08-18,3,6,1,27,32.2000007629395,0.800000011920929,60,3,2,218,84,0,1,1,1,1,12,
6038,1102,2012-08-18,4,6,1,41,19,0,60,3,2,218,84,0,1,1,1,1,12,4542.304285,4594.983269,10.09726
6039,1102,2012-08-19,0,0,1,12,16,10,38,2,2,218,84,0,1,1,1,1,12,4542.304285,4594.983269,10.0972
6040,1102,2012-08-19,1,0,1,22,32.6666679382324,5.33333349227905,60,3,2,218,84,0,1,1,1,1,12,4
6041,1102,2012-08-19,2,0,1,24.3333339691162,31,4.66666650772095,60,3,2,218,84,0,1,1,1,1,12,4
6042,1102,2012-08-19,3,0,1,27.25,31.75,1,60,3,2,218,84,0,1,1,1,1,12,4542.304285,4594.983269,10
6043,1102,2012-08-19,4,0,1,21.3333339691162,31.6666660308838,3.33333325386047,56.333332
6044,1102,2012-08-20,0,1,0,43.6666679382324,12,4.33333349227905,60,3,2,218,84,0,1,1,1,1,12,4
6045,1102,2012-08-20,1,1,0,26,28.6666660308838,5.33333349227905,60,3,2,218,84,0,1,1,1,1,12,4
6046,1102,2012-08-20,2,1,0,23.3333339691162,32.3333320617676,4.33333349227905,60,3,2,218,
6047,1102,2012-08-20,3,1,0,38,22,0,60,3,2,218,84,0,1,1,1,1,12,4542.304285,4594.983269,10.09726
6048,1102,2012-08-20,4,1,0,27.25,25.5,0.75,53.5,3,2,218,84,0,1,1,1,1,12,4542.304285,4594.983269
6049,1102,2012-08-21,0,2,0,34.5,23.5,2,60,3,2,218,84,0,1,1,1,1,12,4542.304285,4594.983269,10.09

6050,1102,2012-08-21,1,2,0,18.5,25.25,5.75,49.5,3,2,218,84,0,1,1,1,1,12,4542.304285,4594.983269
6051,1102,2012-08-21,2,2,0,23.3333339691162,32,4.66666650772095,60,3,2,218,84,0,1,1,1,1,12,4
6052,1102,2012-08-21,3,2,0,21.6000003814697,27.6000003814697,1.20000004768372,50.400001
6053,1102,2012-08-21,4,2,0,44.5,15.5,0,60,2,2,218,84,0,1,1,1,1,12,4542.304285,4594.983269,10.0
6054,1103,2012-08-01,0,3,0,4,36.5,11,51.5,2,2,218,78,1,1,0,0,1,12,4092.313692,3394.355042,9.18
6055,1103,2012-08-01,1,3,0,6.66666650772095,29.6666660308838,23.6666660308838,60,3,2,218,
6056,1103,2012-08-01,2,3,0,15,34.3333320617676,10.6666669845581,60,3,2,218,78,1,1,0,0,1,12,4
6057,1103,2012-08-01,3,3,0,12,46.2000007629395,1.79999995231628,60,3,2,218,78,1,1,0,0,1,12,4
6058,1103,2012-08-01,4,3,0,21,36,3,60,3,2,218,78,1,1,0,0,1,12,4092.313692,3394.355042,9.18900
6059,1103,2012-08-02,0,4,0,12,28,5.66666650772095,45.6666679382324,3,2,218,78,1,1,0,0,1,12,4
6060,1103,2012-08-02,1,4,0,6,32,22,60,3,2,218,78,1,1,0,0,1,12,4092.313692,3394.355042,9.18900
6061,1103,2012-08-02,2,4,0,14,41.6666679382324,4.33333349227905,60,3,2,218,78,1,1,0,0,1,12,4
6062,1103,2012-08-02,3,4,0,21.6666660308838,32.3333320617676,6,60,3,2,218,78,1,1,0,0,1,12,40
6063,1103,2012-08-02,4,4,0,29.6666660308838,28.3333339691162,2,60,3,2,218,78,1,1,0,0,1,12,40
6064,1103,2012-08-03,0,5,0,6.5,44,3.25,53.75,3,2,218,78,1,1,0,0,1,12,4092.313692,3394.355042,9.
6065,1103,2012-08-03,1,5,0,14.25,40,5.75,60,3,2,218,78,1,1,0,0,1,12,4092.313692,3394.355042,9.1
6066,1103,2012-08-03,2,5,0,32.3333320617676,26.3333339691162,1.33333337306976,60,3,2,218,
6067,1103,2012-08-03,3,5,0,13,37,10,60,3,2,218,78,1,1,0,0,1,12,4092.313692,3394.355042,9.1890
6068,1103,2012-08-03,4,5,0,32.3333320617676,26.6666660308838,1,60,3,2,218,78,1,1,0,0,1,12,40
6069,1103,2012-08-04,0,6,1,14,19.5,3.5,37,2,2,218,78,1,1,0,0,1,12,4092.313692,3394.355042,9.18
6070,1103,2012-08-04,1,6,1,6,39.25,14.75,60,3,2,218,78,1,1,0,0,1,12,4092.313692,3394.355042,9.1
6071,1103,2012-08-04,2,6,1,2.66666674613953,49.6666679382324,7.66666650772095,60,3,2,218,
6072,1103,2012-08-04,3,6,1,13,35,12,60,3,2,218,78,1,1,0,0,1,12,4092.313692,3394.355042,9.1890
6073,1103,2012-08-04,4,6,1,32.6666679382324,20,7.33333349227905,60,3,2,218,78,1,1,0,0,1,12,4
6074,1103,2012-08-05,0,0,1,14,35,2,51,2,2,218,78,1,1,0,0,1,12,4092.313692,3394.355042,9.18900
6075,1103,2012-08-05,1,0,1,12.75,44,3.25,60,3,2,218,78,1,1,0,0,1,12,4092.313692,3394.355042,9.1
6076,1103,2012-08-05,2,0,1,23,21.6666660308838,15.3333330154419,60,3,2,218,78,1,1,0,0,1,12,4
6077,1103,2012-08-05,3,0,1,42.3333320617676,9.66666698455811,8,60,3,2,218,78,1,1,0,0,1,12,40
6078,1103,2012-08-05,4,0,1,40.6666679382324,15.6666669845581,3.66666674613953,60,3,2,218,
6079,1103,2012-08-06,2,1,0,15,39,6,60,3,2,218,78,1,1,0,0,1,12,4092.313692,3394.355042,9.18900
6080,1103,2012-08-06,3,1,0,9,47.6666679382324,3.33333325386047,60,3,2,218,78,1,1,0,0,1,12,40
6081,1103,2012-08-06,4,1,0,14.6666669845581,45.3333320617676,0,60,3,2,218,78,1,1,0,0,1,12,40
6082,1103,2012-08-21,0,2,0,10,29,21,60,1,2,218,78,1,1,0,0,1,12,4092.313692,3394.355042,9.1890
6083,1103,2012-08-21,1,2,0,16.6666660308838,41,2.33333325386047,60,3,2,218,78,1,1,0,0,1,12,4
6084,1103,2012-08-21,2,2,0,24.3333339691162,35.3333320617676,0.333333343267441,60,3,2,21
6085,1103,2012-08-21,3,2,0,24,36,0,60,1,2,218,78,1,1,0,0,1,12,4092.313692,3394.355042,9.18900
6086,1105,2012-07-27,1,5,0,16.6000003814697,31.2000007629395,3.20000004768372,51,3,2,218,
6087,1105,2012-07-27,2,5,0,26.75,30,3.25,60,3,2,218,70,1,1,0,0,1,12,3278.02934,3573.773391,9.8
6088,1105,2012-07-27,3,5,0,33,20.3333339691162,6.66666650772095,60,3,2,218,70,1,1,0,0,1,12,3
6089,1105,2012-07-27,4,5,0,39.75,12.75,3.5,56,3,2,218,70,1,1,0,0,1,12,3278.02934,3573.773391,9.
6090,1105,2012-07-28,0,6,1,43,9,2,54,1,2,218,70,1,1,0,0,1,12,3278.02934,3573.773391,9.892468
6091,1105,2012-07-28,1,6,1,36.6666679382324,20.6666660308838,2.66666674613953,60,3,2,218,
6092,1105,2012-07-28,2,6,1,26.2000007629395,29.2000007629395,4.59999990463257,60,3,2,218,
6093,1105,2012-07-28,3,6,1,29.8333339691162,26.5,3.66666674613953,60,3,2,218,70,1,1,0,0,1,12
6094,1105,2012-07-28,4,6,1,40.75,19,0.25,60,3,2,218,70,1,1,0,0,1,12,3278.02934,3573.773391,9.8
6095,1105,2012-07-29,0,0,1,13.5,18,0.5,32,2,2,218,70,1,1,0,0,1,12,3278.02934,3573.773391,9.892
6096,1105,2012-07-29,1,0,1,25.5,29,2.33333325386047,56.8333320617676,3,2,218,70,1,1,0,0,1,12
6097,1105,2012-07-29,2,0,1,25.6666660308838,30,4.33333349227905,60,3,2,218,70,1,1,0,0,1,12,3
6098,1105,2012-07-29,3,0,1,21.3333339691162,38.6666679382324,0,60,3,2,218,70,1,1,0,0,1,12,32
6099,1105,2012-07-29,4,0,1,10,16.5,1,27.5,2,2,218,70,1,1,0,0,1,12,3278.02934,3573.773391,9.892

6100,1105,2012-07-30,2,1,0,36.3333320617676,22.3333339691162,1.33333337306976,60,3,2,218,
6101,1105,2012-07-30,3,1,0,50.3333320617676,9.66666698455811,0,60,3,2,218,70,1,1,0,0,1,12,32
6102,1105,2012-07-30,4,1,0,23.3333339691162,19.3333339691162,1,43.6666679382324,3,2,218,7
6103,1105,2012-07-31,1,2,0,17.3333339691162,23.3333339691162,13,53.6666679382324,3,2,218,
6104,1105,2012-07-31,2,2,0,34,25,1,60,3,2,218,70,1,1,0,0,1,12,3278.02934,3573.773391,9.892468
6105,1105,2012-07-31,3,2,0,22.5,30.1666660308838,7.33333349227905,60,3,2,218,70,1,1,0,0,1,12
6106,1105,2012-07-31,4,2,0,25,20,0,45,3,2,218,70,1,1,0,0,1,12,3278.02934,3573.773391,9.892468
6107,1105,2012-08-02,0,4,0,16.5,30,0.5,47,2,2,218,70,1,1,0,0,1,12,3278.02934,3573.773391,9.892
6108,1105,2012-08-02,1,4,0,16.3333339691162,23.3333339691162,20.3333339691162,60,3,2,218,
6109,1105,2012-08-02,2,4,0,36.6666679382324,19.6666660308838,3.66666674613953,60,3,2,218,
6110,1105,2012-08-02,3,4,0,26.6666660308838,27,6.33333349227905,60,3,2,218,70,1,1,0,0,1,12,3
6111,1105,2012-08-02,4,4,0,42.3333320617676,17.6666660308838,0,60,3,2,218,70,1,1,0,0,1,12,32
6112,1105,2012-08-06,0,1,0,12.5,25,1,38.5,2,2,218,70,1,1,0,0,1,12,3278.02934,3573.773391,9.892
6113,1105,2012-08-06,1,1,0,20,36.6666679382324,3.33333325386047,60,3,2,218,70,1,1,0,0,1,12,3
6114,1105,2012-08-06,2,1,0,28,24,8,60,3,2,218,70,1,1,0,0,1,12,3278.02934,3573.773391,9.892468
6115,1106,2010-12-03,0,5,0,9,7,0,16,1,2,218,83,1,0,1,0,1,5,4988.767506,5107.342735,10.513001
6116,1106,2010-12-03,1,5,0,41.3333320617676,18.3333339691162,0.333333343267441,60,3,2,21
6117,1106,2010-12-03,2,5,0,40,20,0,60,3,2,218,83,1,0,1,0,1,5,4988.767506,5107.342735,10.513001
6118,1106,2010-12-03,3,5,0,46.6666679382324,13.3333330154419,0,60,3,2,218,83,1,0,1,0,1,5,498
6119,1106,2010-12-03,4,5,0,37.7999992370605,8.60000038146973,0,46.4000015258789,3,2,218,8
6120,1106,2010-12-04,1,6,1,33.7999992370605,21.7999992370605,0,55.5999984741211,3,2,218,8
6121,1106,2010-12-04,2,6,1,42.7999992370605,17.2000007629395,0,60,3,2,218,83,1,0,1,0,1,5,498
6122,1106,2010-12-04,3,6,1,47,13,0,60,3,2,218,83,1,0,1,0,1,5,4988.767506,5107.342735,10.513001
6123,1106,2010-12-04,4,6,1,44.6666679382324,5.66666650772095,0,50.3333320617676,3,2,218,8
6124,1106,2010-12-05,1,0,1,28.5,24,0.5,53,2,2,218,83,1,0,1,0,1,5,4988.767506,5107.342735,10.51
6125,1106,2010-12-05,2,0,1,37.25,22.75,0,60,3,2,218,83,1,0,1,0,1,5,4988.767506,5107.342735,10.
6126,1106,2010-12-05,3,0,1,43.1666679382324,16.8333339691162,0,60,3,2,218,83,1,0,1,0,1,5,498
6127,1106,2010-12-05,4,0,1,33.25,15.75,0.25,49.25,3,2,218,83,1,0,1,0,1,5,4988.767506,5107.3427
6128,1106,2010-12-06,1,1,0,24.75,20,0.25,45,3,2,218,83,1,0,1,0,1,5,4988.767506,5107.342735,10.
6129,1106,2010-12-06,2,1,0,50.75,9.25,0,60,3,2,218,83,1,0,1,0,1,5,4988.767506,5107.342735,10.51
6130,1106,2010-12-06,3,1,0,35,25,0,60,3,2,218,83,1,0,1,0,1,5,4988.767506,5107.342735,10.513001
6131,1106,2010-12-06,4,1,0,51,9,0,60,3,2,218,83,1,0,1,0,1,5,4988.767506,5107.342735,10.513001
6132,1106,2010-12-07,1,2,0,28,12.3333330154419,0,40.3333320617676,3,2,218,83,1,0,1,0,1,5,498
6133,1106,2010-12-07,2,2,0,43.4000015258789,16.6000003814697,0,60,3,2,218,83,1,0,1,0,1,5,498
6134,1106,2010-12-07,3,2,0,47.4000015258789,12.6000003814697,0,60,3,2,218,83,1,0,1,0,1,5,498
6135,1106,2010-12-07,4,2,0,48.4000015258789,11.3999996185303,0.200000002980232,60,3,2,21
6136,1106,2010-12-08,1,3,0,22.25,22.25,0,44.5,3,2,218,83,1,0,1,0,1,5,4988.767506,5107.342735,1
6137,1106,2010-12-08,2,3,0,46,14,0,60,3,2,218,83,1,0,1,0,1,5,4988.767506,5107.342735,10.513001
6138,1106,2010-12-08,3,3,0,48.25,11.75,0,60,3,2,218,83,1,0,1,0,1,5,4988.767506,5107.342735,10.
6139,1106,2010-12-08,4,3,0,46,13,0,59,3,2,218,83,1,0,1,0,1,5,4988.767506,5107.342735,10.513001
6140,1106,2010-12-09,1,4,0,19.5,16.5,0.5,36.5,2,2,218,83,1,0,1,0,1,5,4988.767506,5107.342735,10
6141,1106,2010-12-09,2,4,0,45.4000015258789,14.6000003814697,0,60,3,2,218,83,1,0,1,0,1,5,498
6142,1106,2010-12-09,3,4,0,40.6666679382324,19.3333339691162,0,60,3,2,218,83,1,0,1,0,1,5,498
6143,1106,2010-12-09,4,4,0,44.3333320617676,15.6666669845581,0,60,3,2,218,83,1,0,1,0,1,5,498
6144,1106,2010-12-10,1,5,0,35.5,21.5,0.5,57.5,2,2,218,83,1,0,1,0,1,5,4988.767506,5107.342735,10
6145,1106,2010-12-10,2,5,0,44.6666679382324,15.3333330154419,0,60,3,2,218,83,1,0,1,0,1,5,498
6146,1106,2010-12-10,3,5,0,42.8333320617676,17.1666660308838,0,60,3,2,218,83,1,0,1,0,1,5,498
6147,1106,2010-12-10,4,5,0,48,12,0,60,3,2,218,83,1,0,1,0,1,5,4988.767506,5107.342735,10.513001
6148,1106,2012-07-30,0,1,0,60,0,0,60,1,2,218,83,1,0,1,0,1,5,4988.767506,5107.342735,10.513001
6149,1106,2012-07-30,1,1,0,41,18.6666660308838,0.333333343267441,60,3,2,218,83,1,0,1,0,1,5,4

6150,1106,2012-07-30,2,1,0,46.3333320617676,13.6666669845581,0,60,3,2,218,83,1,0,1,0,1,5,498
6151,1106,2012-07-30,3,1,0,50,9,1,60,1,2,218,83,1,0,1,0,1,5,4988.767506,5107.342735,10.513001
6152,1106,2012-07-31,0,2,0,15,16,0,31,1,2,218,83,1,0,1,0,1,5,4988.767506,5107.342735,10.513001
6153,1106,2012-07-31,1,2,0,41.3333320617676,18.6666660308838,0,60,3,2,218,83,1,0,1,0,1,5,498
6154,1106,2012-07-31,2,2,0,52.6666679382324,7.33333349227905,0,60,3,2,218,83,1,0,1,0,1,5,498
6155,1106,2012-07-31,3,2,0,46.4000015258789,13.3999996185303,0.200000002980232,60,3,2,21
6156,1106,2012-07-31,4,2,0,38.25,16.25,0.25,54.75,3,2,218,83,1,0,1,0,1,5,4988.767506,5107.3427
6157,1108,2010-12-01,0,3,0,23,24,13,60,1,2,218,72,0,0,0,1,1,9,4963.058463,5668.318631,10.2726
6158,1108,2010-12-01,1,3,0,6.66666650772095,50,3.33333325386047,60,3,2,218,72,0,0,0,1,1,9,49
6159,1108,2010-12-01,2,3,0,19.3333339691162,39,1.66666662693024,60,3,2,218,72,0,0,0,1,1,9,49
6160,1108,2010-12-01,3,3,0,32,27,1,60,3,2,218,72,0,0,0,1,1,9,4963.058463,5668.318631,10.27268
6161,1108,2010-12-01,4,3,0,43.6666679382324,16.3333339691162,0,60,3,2,218,72,0,0,0,1,1,9,496
6162,1108,2010-12-07,0,2,0,26.5,18,13.5,58,2,2,218,72,0,0,0,1,1,9,4963.058463,5668.318631,10.27
6163,1108,2010-12-07,1,2,0,9,49.6666679382324,1.33333337306976,60,3,2,218,72,0,0,0,1,1,9,496
6164,1108,2010-12-07,2,2,0,20.6666660308838,33,6.33333349227905,60,3,2,218,72,0,0,0,1,1,9,49
6165,1108,2010-12-07,3,2,0,10,46.3333320617676,3.66666674613953,60,3,2,218,72,0,0,0,1,1,9,49
6166,1108,2010-12-07,4,2,0,52,7,1,60,3,2,218,72,0,0,0,1,1,9,4963.058463,5668.318631,10.272685
6167,1108,2010-12-08,1,3,0,28.6666660308838,23.6666660308838,7.66666650772095,60,3,2,218,
6168,1108,2010-12-08,2,3,0,44,14,2,60,3,2,218,72,0,0,0,1,1,9,4963.058463,5668.318631,10.27268
6169,1108,2010-12-08,3,3,0,15,40,5,60,3,2,218,72,0,0,0,1,1,9,4963.058463,5668.318631,10.27268
6170,1108,2010-12-08,4,3,0,25.6666660308838,33,1.33333337306976,60,3,2,218,72,0,0,0,1,1,9,49
6171,1108,2010-12-09,0,4,0,1.5,26.5,11,39,2,2,218,72,0,0,0,1,1,9,4963.058463,5668.318631,10.27
6172,1108,2010-12-09,1,4,0,11.6666669845581,41.6666679382324,6.66666650772095,60,3,2,218,
6173,1108,2010-12-09,2,4,0,32.3333320617676,25.3333339691162,2.33333325386047,60,3,2,218,
6174,1108,2010-12-09,3,4,0,35,24.6666660308838,0.333333343267441,60,3,2,218,72,0,0,0,1,1,9,4
6175,1108,2010-12-09,4,4,0,39.3333320617676,20,0.666666686534882,60,3,2,218,72,0,0,0,1,1,9,4
6176,1108,2010-12-10,0,5,0,7,21.5,7.5,36,2,2,218,72,0,0,0,1,1,9,4963.058463,5668.318631,10.272
6177,1108,2010-12-10,1,5,0,22.3333339691162,36.6666679382324,1,60,3,2,218,72,0,0,0,1,1,9,496
6178,1108,2010-12-10,2,5,0,20,38.3333320617676,1.66666662693024,60,3,2,218,72,0,0,0,1,1,9,49
6179,1108,2010-12-10,3,5,0,47.3333320617676,9,3.66666674613953,60,3,2,218,72,0,0,0,1,1,9,496
6180,1108,2010-12-10,4,5,0,39.5,15.5,5,60,2,2,218,72,0,0,0,1,1,9,4963.058463,5668.318631,10.27
6181,1108,2010-12-11,0,6,1,14,24,12,50,1,2,218,72,0,0,0,1,1,9,4963.058463,5668.318631,10.2726
6182,1108,2010-12-11,1,6,1,12,45.3333320617676,2.66666674613953,60,3,2,218,72,0,0,0,1,1,9,49
6183,1108,2010-12-11,2,6,1,36.6666679382324,19.6666660308838,3.66666674613953,60,3,2,218,
6184,1108,2010-12-11,3,6,1,50,8.66666698455811,1.33333337306976,60,3,2,218,72,0,0,0,1,1,9,49
6185,1108,2010-12-11,4,6,1,46.3333320617676,13.3333330154419,0.333333343267441,60,3,2,21
6186,1108,2010-12-12,0,0,1,17,25,16,58,1,2,218,72,0,0,0,1,1,9,4963.058463,5668.318631,10.2726
6187,1108,2010-12-12,1,0,1,12.6666669845581,37.6666679382324,9.66666698455811,60,3,2,218,
6188,1108,2010-12-12,2,0,1,23.6666660308838,32,4.33333349227905,60,3,2,218,72,0,0,0,1,1,9,49
6189,1108,2010-12-12,3,0,1,38.6666679382324,18.3333339691162,3,60,3,2,218,72,0,0,0,1,1,9,496
6190,1108,2010-12-12,4,0,1,57.5,2.5,0,60,2,2,218,72,0,0,0,1,1,9,4963.058463,5668.318631,10.272
6191,1108,2010-12-13,0,1,0,7,13,7,27,2,2,218,72,0,0,0,1,1,9,4963.058463,5668.318631,10.272685
6192,1108,2010-12-13,1,1,0,18.6666660308838,38,3.33333325386047,60,3,2,218,72,0,0,0,1,1,9,49
6193,1108,2010-12-13,2,1,0,23.6666660308838,35.3333320617676,1,60,3,2,218,72,0,0,0,1,1,9,496
6194,1108,2010-12-13,3,1,0,22.3333339691162,34.6666679382324,3,60,3,2,218,72,0,0,0,1,1,9,496
6195,1108,2010-12-13,4,1,0,39,9,0.75,48.75,3,2,218,72,0,0,0,1,1,9,4963.058463,5668.318631,10.27
6196,1110,2010-11-30,0,2,0,14.5,30.5,1,46,2,2,218,80,1,1,0,0,1,12,4280.803481,3942.205668,9.10
6197,1110,2010-11-30,1,2,0,10,39.6666679382324,10.3333330154419,60,3,2,218,80,1,1,0,0,1,12,4
6198,1110,2010-11-30,2,2,0,29.6000003814697,28.3999996185303,2,60,3,2,218,80,1,1,0,0,1,12,42
6199,1110,2010-11-30,3,2,0,20.6666660308838,39.3333320617676,0,60,3,2,218,80,1,1,0,0,1,12,42

6200,1110,2010-11-30,4,2,0,46.3333320617676,13.6666669845581,0,60,3,2,218,80,1,1,0,0,1,12,42
6201,1110,2010-12-01,0,3,0,6,48,6,60,1,2,218,80,1,1,0,0,1,12,4280.803481,3942.205668,9.105738
6202,1110,2010-12-01,1,3,0,6,39,15,60,3,2,218,80,1,1,0,0,1,12,4280.803481,3942.205668,9.10573
6203,1110,2010-12-01,2,3,0,6,42,12,60,1,2,218,80,1,1,0,0,1,12,4280.803481,3942.205668,9.10573
6204,1110,2010-12-01,3,3,0,36.3333320617676,18.3333339691162,5.33333349227905,60,3,2,218,
6205,1110,2010-12-01,4,3,0,41,16.6666660308838,2.33333325386047,60,3,2,218,80,1,1,0,0,1,12,4
6206,1110,2010-12-02,0,4,0,6,33.5,7,46.5,2,2,218,80,1,1,0,0,1,12,4280.803481,3942.205668,9.1057
6207,1110,2010-12-02,1,4,0,2,54.6666679382324,3.33333325386047,60,3,2,218,80,1,1,0,0,1,12,42
6208,1110,2010-12-02,2,4,0,23.6000003814697,35.7999992370605,0.600000023841858,60,3,2,21
6209,1110,2010-12-02,3,4,0,28,23.6666660308838,8.33333301544189,60,3,2,218,80,1,1,0,0,1,12,4
6210,1110,2010-12-02,4,4,0,29.6666660308838,30,0.333333343267441,60,3,2,218,80,1,1,0,0,1,12,
6211,1110,2010-12-03,0,5,0,6.5,41.5,0.5,48.5,2,2,218,80,1,1,0,0,1,12,4280.803481,3942.205668,9.
6212,1110,2010-12-03,1,5,0,3,53,4,60,3,2,218,80,1,1,0,0,1,12,4280.803481,3942.205668,9.105738
6213,1110,2010-12-03,2,5,0,26.25,33.5,0.25,60,3,2,218,80,1,1,0,0,1,12,4280.803481,3942.205668,
6214,1110,2010-12-03,3,5,0,26.2000007629395,33.5999984741211,0.200000002980232,60,3,2,21
6215,1110,2010-12-03,4,5,0,29,30,1,60,3,2,218,80,1,1,0,0,1,12,4280.803481,3942.205668,9.10573
6216,1110,2010-12-04,0,6,1,2.66666674613953,41,5.66666650772095,49.3333320617676,3,2,218,
6217,1110,2010-12-04,1,6,1,14.6666669845581,42.3333320617676,3,60,3,2,218,80,1,1,0,0,1,12,42
6218,1110,2010-12-04,2,6,1,28.6666660308838,31.3333339691162,0,60,3,2,218,80,1,1,0,0,1,12,42
6219,1110,2010-12-04,3,6,1,18.5,36,5.5,60,3,2,218,80,1,1,0,0,1,12,4280.803481,3942.205668,9.10
6220,1110,2010-12-04,4,6,1,33.25,26.25,0.5,60,3,2,218,80,1,1,0,0,1,12,4280.803481,3942.205668,
6221,1110,2010-12-05,0,0,1,3,39,0.5,42.5,2,2,218,80,1,1,0,0,1,12,4280.803481,3942.205668,9.1057
6222,1110,2010-12-05,1,0,1,16,36.3333320617676,7.66666650772095,60,3,2,218,80,1,1,0,0,1,12,4
6223,1110,2010-12-05,2,0,1,29.6666660308838,27.3333339691162,3,60,3,2,218,80,1,1,0,0,1,12,42
6224,1110,2010-12-05,3,0,1,20.6666660308838,32.6666679382324,6.66666650772095,60,3,2,218,
6225,1110,2010-12-05,4,0,1,38.5999984741211,21.3999996185303,0,60,3,2,218,80,1,1,0,0,1,12,42
6226,1110,2010-12-06,0,1,0,8.5,48.5,0.5,57.5,2,2,218,80,1,1,0,0,1,12,4280.803481,3942.205668,9.
6227,1110,2010-12-06,1,1,0,11.5,45.6666679382324,2.83333325386047,60,3,2,218,80,1,1,0,0,1,12
6228,1110,2010-12-06,2,1,0,21.6666660308838,37,1.33333337306976,60,3,2,218,80,1,1,0,0,1,12,4
6229,1110,2010-12-06,3,1,0,23.6666660308838,19,17.3333339691162,60,3,2,218,80,1,1,0,0,1,12,4
6230,1110,2010-12-06,4,1,0,35.6666679382324,24.3333339691162,0,60,3,2,218,80,1,1,0,0,1,12,42
6231,1113,2012-08-01,0,3,0,10,43,7,60,1,2,218,77,1,1,1,0,1,11,5009.894787,6013.015951,9.86330
6232,1113,2012-08-01,1,3,0,6.66666650772095,46.6666679382324,6.66666650772095,60,3,2,218,
6233,1113,2012-08-01,2,3,0,13,38,9,60,3,2,218,77,1,1,1,0,1,11,5009.894787,6013.015951,9.86330
6234,1113,2012-08-01,3,3,0,39,21,0,60,3,2,218,77,1,1,1,0,1,11,5009.894787,6013.015951,9.86330
6235,1113,2012-08-01,4,3,0,28.3333339691162,28.6666660308838,3,60,3,2,218,77,1,1,1,0,1,11,50
6236,1113,2012-08-02,0,4,0,8.33333301544189,24.3333339691162,4,36.6666679382324,3,2,218,7
6237,1113,2012-08-02,1,4,0,4,44.6666679382324,11.3333330154419,60,3,2,218,77,1,1,1,0,1,11,50
6238,1113,2012-08-02,2,4,0,8.33333301544189,48,3.66666674613953,60,3,2,218,77,1,1,1,0,1,11,5
6239,1113,2012-08-02,3,4,0,30.3333339691162,28,1.66666662693024,60,3,2,218,77,1,1,1,0,1,11,5
6240,1113,2012-08-02,4,4,0,48,11.3333330154419,0.666666686534882,60,3,2,218,77,1,1,1,0,1,11,
6241,1113,2012-08-03,0,5,0,12,26,3.33333325386047,41.3333320617676,3,2,218,77,1,1,1,0,1,11,5
6242,1113,2012-08-03,1,5,0,18,41,1,60,3,2,218,77,1,1,1,0,1,11,5009.894787,6013.015951,9.86330
6243,1113,2012-08-03,2,5,0,32.6666679382324,23.6666660308838,3.66666674613953,60,3,2,218,
6244,1113,2012-08-03,3,5,0,33.6666679382324,11,15.3333330154419,60,3,2,218,77,1,1,1,0,1,11,5
6245,1113,2012-08-03,4,5,0,35,21,4,60,3,2,218,77,1,1,1,0,1,11,5009.894787,6013.015951,9.86330
6246,1113,2012-08-04,0,6,1,9,26.3333339691162,3,38.3333320617676,3,2,218,77,1,1,1,0,1,11,500
6247,1113,2012-08-04,1,6,1,14.6666669845581,33,12.3333330154419,60,3,2,218,77,1,1,1,0,1,11,5
6248,1113,2012-08-04,2,6,1,31.3333339691162,26.6666660308838,2,60,3,2,218,77,1,1,1,0,1,11,50
6249,1113,2012-08-04,3,6,1,24.3333339691162,30,5.66666650772095,60,3,2,218,77,1,1,1,0,1,11,5

6250,1113,2012-08-04,4,6,1,39.3333320617676,20.6666660308838,0,60,3,2,218,77,1,1,1,0,1,11,50
6251,1113,2012-08-05,0,7,1,13.3333330154419,14.6666669845581,1.33333337306976,29.333333
6252,1113,2012-08-05,1,7,1,10,46,4,60,3,2,218,77,1,1,1,0,1,11,5009.894787,6013.015951,9.863304
6253,1113,2012-08-05,2,7,1,36.3333320617676,23,0.666666686534882,60,3,2,218,77,1,1,1,0,1,11,
6254,1113,2012-08-05,3,7,1,43.6666679382324,16,0.333333343267441,60,3,2,218,77,1,1,1,0,1,11,
6255,1113,2012-08-05,4,7,1,34.3333320617676,25.3333339691162,0.333333343267441,60,3,2,218
6256,1113,2012-08-06,0,1,0,15,21.3333339691162,1.66666662693024,38,3,2,218,77,1,1,1,0,1,11,5
6257,1113,2012-08-06,1,1,0,25.3333339691162,33.6666679382324,1,60,3,2,218,77,1,1,1,0,1,11,50
6258,1113,2012-08-06,2,1,0,30.3333339691162,26.6666660308838,3,60,3,2,218,77,1,1,1,0,1,11,50
6259,1113,2012-08-06,3,1,0,37,22,1,60,3,2,218,77,1,1,1,0,1,11,5009.894787,6013.015951,9.863304
6260,1113,2012-08-06,4,1,0,47.3333320617676,12.6666669845581,0,60,3,2,218,77,1,1,1,0,1,11,50
6261,1113,2012-08-07,0,2,0,4.33333349227905,26,4.33333349227905,34.6666679382324,3,2,218,
6262,1113,2012-08-07,1,2,0,5,40.6666679382324,14.3333330154419,60,3,2,218,77,1,1,1,0,1,11,50
6263,1113,2012-08-07,2,2,0,25.6666660308838,32.6666679382324,1.66666662693024,60,3,2,218,
6264,1113,2012-08-07,3,2,0,21,36.3333320617676,2.66666674613953,60,3,2,218,77,1,1,1,0,1,11,5
6265,1113,2012-08-07,4,2,0,37,20.3333339691162,2.66666674613953,60,3,2,218,77,1,1,1,0,1,11,5
6266,1115,2010-12-01,0,3,0,35,24,1,60,1,2,218,79,1,1,1,0,1,9,3901.101314,2851.382529,7.780744
6267,1115,2010-12-01,1,3,0,37.3333320617676,22.6666660308838,0,60,3,2,218,79,1,1,1,0,1,9,390
6268,1115,2010-12-01,2,3,0,24.6666660308838,33,2.33333325386047,60,3,2,218,79,1,1,1,0,1,9,39
6269,1115,2010-12-01,3,3,0,38,20,2,60,3,2,218,79,1,1,1,0,1,9,3901.101314,2851.382529,7.780744
6270,1115,2010-12-02,0,4,0,21,23.6666660308838,0.666666686534882,45.3333320617676,3,2,218
6271,1115,2010-12-02,1,4,0,35,24.3333339691162,0.666666686534882,60,3,2,218,79,1,1,1,0,1,9,3
6272,1115,2010-12-02,2,4,0,37.6666679382324,21.6666660308838,0.666666686534882,60,3,2,218
6273,1115,2010-12-02,3,4,0,40.6666679382324,18,1.33333337306976,60,3,2,218,79,1,1,1,0,1,9,39
6274,1115,2010-12-02,4,4,0,32.5,26.6666660308838,0.833333313465118,60,3,2,218,79,1,1,1,0,1,9
6275,1115,2010-12-03,0,5,0,20.7999992370605,29.3999996185303,0.800000011920929,51,3,2,218
6276,1115,2010-12-03,1,5,0,30.2000007629395,28.6000003814697,1.20000004768372,60,3,2,218,
6277,1115,2010-12-03,2,5,0,24,34,2,60,3,2,218,79,1,1,1,0,1,9,3901.101314,2851.382529,7.780744
6278,1115,2010-12-03,3,5,0,32.3333320617676,26,1.66666662693024,60,3,2,218,79,1,1,1,0,1,9,39
6279,1115,2010-12-03,4,5,0,28.5,2,1.5,32,2,2,218,79,1,1,1,0,1,9,3901.101314,2851.382529,7.7807
6280,1115,2010-12-04,0,6,1,30,12.3333330154419,0.333333343267441,42.6666679382324,3,2,218
6281,1115,2010-12-04,1,6,1,28.25,31.5,0.25,60,3,2,218,79,1,1,1,0,1,9,3901.101314,2851.382529,7.
6282,1115,2010-12-04,2,6,1,36.4000015258789,23.3999996185303,0.200000002980232,60,3,2,218
6283,1115,2010-12-04,3,6,1,35.3333320617676,24.6666660308838,0,60,3,2,218,79,1,1,1,0,1,9,390
6284,1115,2010-12-04,4,6,1,33,26.6666660308838,0.333333343267441,60,3,2,218,79,1,1,1,0,1,9,3
6285,1115,2010-12-05,0,0,1,6,17,1,24,1,2,218,79,1,1,1,0,1,9,3901.101314,2851.382529,7.7807449
6286,1115,2010-12-05,1,0,1,34.6666679382324,23.3333339691162,2,60,3,2,218,79,1,1,1,0,1,9,390
6287,1115,2010-12-05,2,0,1,44.5,15.5,0,60,3,2,218,79,1,1,1,0,1,9,3901.101314,2851.382529,7.7807
6288,1115,2010-12-05,3,0,1,41.8333320617676,18,0.16666667163372,60,3,2,218,79,1,1,1,0,1,9,39
6289,1115,2010-12-05,4,0,1,45,15,0,60,3,2,218,79,1,1,1,0,1,9,3901.101314,2851.382529,7.780744
6290,1115,2010-12-06,0,1,0,25.6666660308838,22,0.333333343267441,48,3,2,218,79,1,1,1,0,1,9,3
6291,1115,2010-12-06,1,1,0,36,21,3,60,3,2,218,79,1,1,1,0,1,9,3901.101314,2851.382529,7.780744
6292,1115,2010-12-06,2,1,0,34.3333320617676,25.6666660308838,0,60,3,2,218,79,1,1,1,0,1,9,390
6293,1115,2010-12-06,3,1,0,42,18,0,60,3,2,218,79,1,1,1,0,1,9,3901.101314,2851.382529,7.780744
6294,1115,2010-12-06,4,1,0,33.5,13.25,0.25,47,3,2,218,79,1,1,1,0,1,9,3901.101314,2851.382529,7.
6295,1119,2012-08-02,0,4,0,13,46,1,60,1,2,218,77,0,0,0,1,1,7,3855.192467,3124.816474,7.044439
6296,1119,2012-08-02,1,4,0,21.3333339691162,35,3.66666674613953,60,3,2,218,77,0,0,0,1,1,7,38
6297,1119,2012-08-02,2,4,0,31.6666660308838,28,0.333333343267441,60,3,2,218,77,0,0,0,1,1,7,3
6298,1119,2012-08-02,3,4,0,29,27.6666660308838,3.33333325386047,60,3,2,218,77,0,0,0,1,1,7,38
6299,1119,2012-08-02,4,4,0,38,19,3,60,3,2,218,77,0,0,0,1,1,7,3855.192467,3124.816474,7.044439

6300,1119,2012-08-03,0,5,0,0.666666686534882,0.333333343267441,0,1,3,2,218,77,0,0,0,1,1,7,38
6301,1119,2012-08-03,1,5,0,25.6666660308838,10.3333330154419,1.33333337306976,37.333332
6302,1119,2012-08-03,2,5,0,29,27,4,60,3,2,218,77,0,0,0,1,1,7,3855.192467,3124.816474,7.0444396
6303,1119,2012-08-03,3,5,0,38,22,0,60,3,2,218,77,0,0,0,1,1,7,3855.192467,3124.816474,7.0444396
6304,1119,2012-08-03,4,5,0,45.3333320617676,14.6666669845581,0,60,3,2,218,77,0,0,0,1,1,7,385
6305,1119,2012-08-04,0,6,1,22.6666660308838,36.3333320617676,1,60,3,2,218,77,0,0,0,1,1,7,385
6306,1119,2012-08-04,1,6,1,19,37.6666679382324,3.33333325386047,60,3,2,218,77,0,0,0,1,1,7,38
6307,1119,2012-08-04,2,6,1,36,23.3333339691162,0.666666686534882,60,3,2,218,77,0,0,0,1,1,7,3
6308,1119,2012-08-04,3,6,1,38,22,0,60,3,2,218,77,0,0,0,1,1,7,3855.192467,3124.816474,7.0444396
6309,1119,2012-08-04,4,6,1,43.3333320617676,16,0.666666686534882,60,3,2,218,77,0,0,0,1,1,7,3
6310,1119,2012-08-05,0,7,1,28,20.3333339691162,0.333333343267441,48.6666679382324,3,2,218
6311,1119,2012-08-05,1,7,1,38.3333320617676,21.6666660308838,0,60,3,2,218,77,0,0,0,1,1,7,385
6312,1119,2012-08-05,2,7,1,44.3333320617676,15.6666669845581,0,60,3,2,218,77,0,0,0,1,1,7,385
6313,1119,2012-08-05,3,7,1,38.3333320617676,21,0.666666686534882,60,3,2,218,77,0,0,0,1,1,7,3
6314,1119,2012-08-05,4,7,1,39.3333320617676,11.6666669845581,0.666666686534882,51.666667
6315,1119,2012-08-06,0,1,0,30,16,0,46,3,2,218,77,0,0,0,1,1,7,3855.192467,3124.816474,7.0444396
6316,1119,2012-08-06,1,1,0,28.6666660308838,27.6666660308838,3.66666674613953,60,3,2,218,
6317,1119,2012-08-06,2,1,0,39.6666679382324,16.6666660308838,3.66666674613953,60,3,2,218,
6318,1119,2012-08-06,3,1,0,38.3333320617676,21,0.666666686534882,60,3,2,218,77,0,0,0,1,1,7,3
6319,1119,2012-08-06,4,1,0,46,9,0.666666686534882,55.6666679382324,3,2,218,77,0,0,0,1,1,7,38
6320,1119,2012-08-07,0,2,0,31.6666660308838,18.6666660308838,0,50.3333320617676,3,2,218,7
6321,1119,2012-08-07,1,2,0,41.3333320617676,18.6666660308838,0,60,3,2,218,77,0,0,0,1,1,7,385
6322,1119,2012-08-07,2,2,0,36.3333320617676,23.6666660308838,0,60,3,2,218,77,0,0,0,1,1,7,385
6323,1119,2012-08-07,3,2,0,35.6666679382324,23.3333339691162,1,60,3,2,218,77,0,0,0,1,1,7,385
6324,1119,2012-08-07,4,2,0,45.3333320617676,14.6666669845581,0,60,3,2,218,77,0,0,0,1,1,7,385
6325,1119,2012-08-08,0,3,0,22,33.6666679382324,0,55.6666679382324,3,2,218,77,0,0,0,1,1,7,385
6326,1119,2012-08-08,1,3,0,21.6666660308838,38.3333320617676,0,60,3,2,218,77,0,0,0,1,1,7,385
6327,1119,2012-08-08,2,3,0,34,25.6666660308838,0.333333343267441,60,3,2,218,77,0,0,0,1,1,7,3
6328,1119,2012-08-08,3,3,0,46,14,0,60,3,2,218,77,0,0,0,1,1,7,3855.192467,3124.816474,7.0444396
6329,1119,2012-08-08,4,3,0,46,14,0,60,3,2,218,77,0,0,0,1,1,7,3855.192467,3124.816474,7.0444396
6330,1119,2012-08-09,0,4,0,26,20.3333339691162,0,46.3333320617676,3,2,218,77,0,0,0,1,1,7,385
6331,1119,2012-08-09,1,4,0,27,32.6666679382324,0.333333343267441,60,3,2,218,77,0,0,0,1,1,7,3
6332,1119,2012-08-09,2,4,0,22.6666660308838,5,2.33333325386047,30,3,2,218,77,0,0,0,1,1,7,385
6333,1119,2012-08-09,3,4,0,36.3333320617676,20.6666660308838,3,60,3,2,218,77,0,0,0,1,1,7,385
6334,1119,2012-08-09,4,4,0,20.3333339691162,3,3.33333325386047,26.6666660308838,3,2,218,7
6335,1147,2010-11-30,1,2,0,10.3333330154419,39.3333320617676,1.66666662693024,51.333332
6336,1147,2010-11-30,2,2,0,27.5,31.75,0.75,60,3,2,218,67,0,1,0,1,1,11,4331.19471,4305.44311,9.3
6337,1147,2010-11-30,3,2,0,23.8333339691162,34.5,1.66666662693024,60,3,2,218,67,0,1,0,1,1,11
6338,1147,2010-11-30,4,2,0,40,19.6666660308838,0.333333343267441,60,3,2,218,67,0,1,0,1,1,11,
6339,1147,2010-12-02,1,4,0,16.75,32.5,1.25,50.5,3,2,218,67,0,1,0,1,1,11,4331.19471,4305.44311,9
6340,1147,2010-12-02,2,4,0,51.3333320617676,8.33333301544189,0.333333343267441,60,3,2,218
6341,1147,2010-12-02,3,4,0,46.3333320617676,13.6666669845581,0,60,3,2,218,67,0,1,0,1,1,11,43
6342,1147,2010-12-02,4,4,0,36.1666679382324,23.1666660308838,0.666666686534882,60,3,2,218
6343,1147,2010-12-03,0,5,0,38,19,1,58,1,2,218,67,0,1,0,1,1,11,4331.19471,4305.44311,9.3647847
6344,1147,2010-12-03,1,5,0,6.40000009536743,48.5999984741211,5,60,3,2,218,67,0,1,0,1,1,11,43
6345,1147,2010-12-03,2,5,0,16.8333339691162,37.6666679382324,5.5,60,3,2,218,67,0,1,0,1,1,11,4
6346,1147,2010-12-03,3,5,0,35.6666679382324,21.6666660308838,2.66666674613953,60,3,2,218,
6347,1147,2010-12-03,4,5,0,22.6666660308838,35,2.33333325386047,60,3,2,218,67,0,1,0,1,1,11,4
6348,1147,2010-12-04,1,6,1,21,12.5,0,33.5,2,2,218,67,0,1,0,1,1,11,4331.19471,4305.44311,9.36478
6349,1147,2010-12-04,2,6,1,32,27.3333339691162,0.666666686534882,60,3,2,218,67,0,1,0,1,1,11,

6350,1147,2010-12-04,3,6,1,38.1666679382324,19.5,2.33333325386047,60,3,2,218,67,0,1,0,1,1,11
6351,1147,2010-12-04,4,6,1,39.5999984741211,20,0.400000005960464,60,3,2,218,67,0,1,0,1,1,11,
6352,1147,2010-12-05,4,0,1,40,20,0,60,1,2,218,67,0,1,0,1,1,11,4331.19471,4305.44311,9.3647847
6353,1147,2010-12-06,0,1,0,60,0,0,60,1,2,218,67,0,1,0,1,1,11,4331.19471,4305.44311,9.36478476
6354,1147,2010-12-06,1,1,0,40,19,1,60,1,2,218,67,0,1,0,1,1,11,4331.19471,4305.44311,9.3647847
6355,1147,2010-12-07,0,2,0,7,8,0,15,1,2,218,67,0,1,0,1,1,11,4331.19471,4305.44311,9.364784769,
6356,1147,2010-12-07,1,2,0,17.6000003814697,28.6000003814697,13.8000001907349,60,3,2,218,
6357,1147,2010-12-07,2,2,0,28.2000007629395,29.7999992370605,2,60,3,2,218,67,0,1,0,1,1,11,43
6358,1147,2010-12-07,3,2,0,25,24.3333339691162,10.6666669845581,60,3,2,218,67,0,1,0,1,1,11,4
6359,1147,2010-12-07,4,2,0,41,18.3333339691162,0.666666686534882,60,3,2,218,67,0,1,0,1,1,11,
6360,1156,2010-12-01,1,3,0,40,14.3333330154419,0.666666686534882,55,3,2,218,82,1,0,1,1,1,10,
6361,1156,2010-12-01,2,3,0,39.3333320617676,20.6666660308838,0,60,3,2,218,82,1,0,1,1,1,10,45
6362,1156,2010-12-01,3,3,0,29.6666660308838,28.6666660308838,1.66666662693024,60,3,2,218,
6363,1156,2010-12-01,4,3,0,36,23.6000003814697,0.400000005960464,60,3,2,218,82,1,0,1,1,1,10,
6364,1156,2010-12-02,0,4,0,25,17.5,0.5,43,2,2,218,82,1,0,1,1,1,10,4577.866569,4611.536619,11.01
6365,1156,2010-12-02,1,4,0,35.3333320617676,22,2.66666674613953,60,3,2,218,82,1,0,1,1,1,10,4
6366,1156,2010-12-02,2,4,0,38.6666679382324,19.3333339691162,2,60,3,2,218,82,1,0,1,1,1,10,45
6367,1156,2010-12-02,3,4,0,37.6666679382324,21,1.33333337306976,60,3,2,218,82,1,0,1,1,1,10,4
6368,1156,2010-12-02,4,4,0,30.6666660308838,8.33333301544189,0.333333343267441,39.33333
6369,1156,2010-12-05,0,0,1,26,15,0,41,3,2,218,82,1,0,1,1,1,10,4577.866569,4611.536619,11.0151
6370,1156,2010-12-05,1,0,1,49,10.6666669845581,0.333333343267441,60,3,2,218,82,1,0,1,1,1,10,
6371,1156,2010-12-05,2,0,1,32.6666679382324,27.3333339691162,0,60,3,2,218,82,1,0,1,1,1,10,45
6372,1156,2010-12-05,3,0,1,31.3333339691162,28.3333339691162,0.333333343267441,60,3,2,21
6373,1156,2010-12-05,4,0,1,26.3333339691162,33.3333320617676,0.333333343267441,60,3,2,21
6374,1156,2010-12-06,0,1,0,4,38,12,54,1,2,218,82,1,0,1,1,1,10,4577.866569,4611.536619,11.0151
6375,1156,2010-12-06,1,1,0,28.5,26.3333339691162,5.16666650772095,60,3,2,218,82,1,0,1,1,1,10
6376,1156,2010-12-06,2,1,0,38.6666679382324,21.3333339691162,0,60,3,2,218,82,1,0,1,1,1,10,45
6377,1156,2010-12-06,3,1,0,47.3333320617676,12.3333330154419,0.333333343267441,60,3,2,21
6378,1156,2010-12-06,4,1,0,47.6666679382324,12,0.333333343267441,60,3,2,218,82,1,0,1,1,1,10,
6379,1156,2010-12-07,0,2,0,22,16,6,44,3,2,218,82,1,0,1,1,1,10,4577.866569,4611.536619,11.0151
6380,1156,2010-12-07,1,2,0,43.5,16.5,0,60,2,2,218,82,1,0,1,1,1,10,4577.866569,4611.536619,11.01
6381,1156,2010-12-07,2,2,0,35,24.3333339691162,0.666666686534882,60,3,2,218,82,1,0,1,1,1,10,
6382,1156,2010-12-07,3,2,0,34.6666679382324,21.3333339691162,4,60,3,2,218,82,1,0,1,1,1,10,45
6383,1156,2010-12-07,4,2,0,27,32.6666679382324,0.333333343267441,60,3,2,218,82,1,0,1,1,1,10,
6384,1156,2010-12-08,0,3,0,23.5,19,1,43.5,2,2,218,82,1,0,1,1,1,10,4577.866569,4611.536619,11.01
6385,1156,2010-12-08,1,3,0,9,50.75,0.25,60,3,2,218,82,1,0,1,1,1,10,4577.866569,4611.536619,11.
6386,1156,2010-12-08,2,3,0,35.5,24.5,0,60,3,2,218,82,1,0,1,1,1,10,4577.866569,4611.536619,11.01
6387,1156,2010-12-08,3,3,0,17.2000007629395,41.2000007629395,1.60000002384186,60,3,2,218,
6388,1156,2010-12-08,4,3,0,25.3333339691162,14.6666669845581,1,41,3,2,218,82,1,0,1,1,1,10,45
6389,1156,2010-12-09,1,4,0,21.3333339691162,32.3333320617676,0.666666686534882,54.33333
6390,1156,2010-12-09,2,4,0,25.6666660308838,34.3333320617676,0,60,3,2,218,82,1,0,1,1,1,10,45
6391,1156,2010-12-09,3,4,0,32.75,26,1.25,60,3,2,218,82,1,0,1,1,1,10,4577.866569,4611.536619,11
6392,1156,2010-12-09,4,4,0,36,23.3333339691162,0.666666686534882,60,3,2,218,82,1,0,1,1,1,10,
6393,1190,2012-06-15,0,5,0,45,9,6,60,1,2,203,67,1,1,0,1,0,8,16797.86577,13188.21368,14.325264
6394,1190,2012-06-15,1,5,0,39.3333320617676,11.3333330154419,9.33333301544189,60,3,2,203,
6395,1190,2012-06-15,2,5,0,26.6666660308838,17,16.3333339691162,60,3,2,203,67,1,1,0,1,0,8,16
6396,1190,2012-06-15,3,5,0,19.3333339691162,33.6666679382324,7,60,3,2,203,67,1,1,0,1,0,8,167
6397,1190,2012-06-15,4,5,0,29,29,2,60,3,2,203,67,1,1,0,1,0,8,16797.86577,13188.21368,14.32526
6398,1190,2012-06-16,0,6,1,5,4.66666650772095,4.33333349227905,14,3,2,203,67,1,1,0,1,0,8,167
6399,1190,2012-06-16,1,6,1,25.3333339691162,31.3333339691162,3.33333325386047,60,3,2,203,

6400,1190,2012-06-16,2,6,1,18.6666660308838,34.6666679382324,6.66666650772095,60,3,2,203,
6401,1190,2012-06-16,3,6,1,19.3333339691162,37.6666679382324,3,60,3,2,203,67,1,1,0,1,0,8,167
6402,1190,2012-06-16,4,6,1,25.6666660308838,29.3333339691162,5,60,3,2,203,67,1,1,0,1,0,8,167
6403,1190,2012-06-17,0,7,1,6,6,3,15,3,2,203,67,1,1,0,1,0,8,16797.86577,13188.21368,14.3252646[illegible]
6404,1190,2012-06-17,1,7,1,22,35.3333320617676,2.66666674613953,60,3,2,203,67,1,1,0,1,0,8,16
6405,1190,2012-06-17,2,7,1,12.3333330154419,33,14.6666669845581,60,3,2,203,67,1,1,0,1,0,8,16
6406,1190,2012-06-17,3,7,1,34.3333320617676,23.3333339691162,2.33333325386047,60,3,2,203,
6407,1190,2012-06-17,4,7,1,37,21.6666660308838,1.33333337306976,60,3,2,203,67,1,1,0,1,0,8,16
6408,1190,2012-06-18,0,1,0,4.33333349227905,18,1.33333337306976,23.6666660308838,3,2,203,
6409,1190,2012-06-18,1,1,0,2.33333325386047,14.3333330154419,4,20.6666660308838,3,2,203,6
6410,1190,2012-06-18,2,1,0,8.33333301544189,35.6666679382324,2.66666674613953,46.666667[illegible]
6411,1190,2012-06-18,3,1,0,17.6666660308838,36,6.33333349227905,60,3,2,203,67,1,1,0,1,0,8,16
6412,1190,2012-06-18,4,1,0,24.3333339691162,31,4.66666650772095,60,3,2,203,67,1,1,0,1,0,8,16
6413,1190,2012-06-19,0,2,0,5,17.3333339691162,6.33333349227905,28.6666660308838,3,2,203,6
6414,1190,2012-06-19,1,2,0,13,39.6666679382324,7.33333349227905,60,3,2,203,67,1,1,0,1,0,8,16
6415,1190,2012-06-19,2,2,0,21.3333339691162,18.3333339691162,20.3333339691162,60,3,2,203,
6416,1190,2012-06-19,3,2,0,24.3333339691162,25.3333339691162,10.3333330154419,60,3,2,203,
6417,1190,2012-06-19,4,2,0,34.6666679382324,22.3333339691162,3,60,3,2,203,67,1,1,0,1,0,8,167
6418,1190,2012-06-20,0,3,0,4.33333349227905,4,4.66666650772095,13,3,2,203,67,1,1,0,1,0,8,167
6419,1190,2012-06-20,1,3,0,18.3333339691162,36.6666679382324,5,60,3,2,203,67,1,1,0,1,0,8,167
6420,1190,2012-06-20,2,3,0,22.3333339691162,30.3333339691162,7.33333349227905,60,3,2,203,
6421,1190,2012-06-20,3,3,0,19.3333339691162,36.3333320617676,4.33333349227905,60,3,2,203,
6422,1190,2012-06-20,4,3,0,12.3333330154419,40.6666679382324,7,60,3,2,203,67,1,1,0,1,0,8,167
6423,1190,2012-06-21,0,4,0,14.6666669845581,11.3333330154419,2.66666674613953,28.666666[illegible]
6424,1190,2012-06-21,1,4,0,36.3333320617676,23,0.666666686534882,60,3,2,203,67,1,1,0,1,0,8,1
6425,1190,2012-06-21,2,4,0,42,18,0,60,3,2,203,67,1,1,0,1,0,8,16797.86577,13188.21368,14.325264[illegible]
6426,1190,2012-06-21,3,4,0,37.6666679382324,22.3333339691162,0,60,3,2,203,67,1,1,0,1,0,8,167
6427,1190,2012-06-21,4,4,0,21.6666660308838,36,2.33333325386047,60,3,2,203,67,1,1,0,1,0,8,16
6428,1192,2012-06-16,0,6,1,25,33,2,60,1,2,203,67,1,1,0,1,0,9,16575.50861,12563.64133,15.722111[illegible]
6429,1192,2012-06-16,1,6,1,7.66666650772095,40.3333320617676,12,60,3,2,203,67,1,1,0,1,0,9,16
6430,1192,2012-06-16,2,6,1,28.6666660308838,23.6666660308838,7.66666650772095,60,3,2,203,
6431,1192,2012-06-16,3,6,1,30.6666660308838,22.3333339691162,7,60,3,2,203,67,1,1,0,1,0,9,165
6432,1192,2012-06-16,4,6,1,40.6666679382324,19,0.333333343267441,60,3,2,203,67,1,1,0,1,0,9,1
6433,1192,2012-06-17,0,7,1,2.33333325386047,9.33333301544189,0,11.6666669845581,3,2,203,6
6434,1192,2012-06-17,1,7,1,23.6666660308838,36,0.333333343267441,60,3,2,203,67,1,1,0,1,0,9,1
6435,1192,2012-06-17,2,7,1,28.3333339691162,30.3333339691162,1.33333337306976,60,3,2,203,
6436,1192,2012-06-17,3,7,1,24,26,10,60,3,2,203,67,1,1,0,1,0,9,16575.50861,12563.64133,15.7221[illegible]
6437,1192,2012-06-17,4,7,1,41.3333320617676,18.6666660308838,0,60,3,2,203,67,1,1,0,1,0,9,165
6438,1192,2012-06-18,0,1,0,5.33333349227905,16,6.33333349227905,27.6666660308838,3,2,203,
6439,1192,2012-06-18,1,1,0,22.6666660308838,28.3333339691162,9,60,3,2,203,67,1,1,0,1,0,9,165
6440,1192,2012-06-18,2,1,0,28,23.6666660308838,8.33333301544189,60,3,2,203,67,1,1,0,1,0,9,16
6441,1192,2012-06-18,3,1,0,44.3333320617676,15.6666669845581,0,60,3,2,203,67,1,1,0,1,0,9,165
6442,1192,2012-06-18,4,1,0,43.3333320617676,16.3333339691162,0.333333343267441,60,3,2,20[illegible]
6443,1192,2012-06-19,0,2,0,5.66666650772095,20.6666660308838,2.33333325386047,28.666666[illegible]
6444,1192,2012-06-19,1,2,0,26.3333339691162,29.3333339691162,4.33333349227905,60,3,2,203,
6445,1192,2012-06-19,2,2,0,22.3333339691162,35,2.66666674613953,60,3,2,203,67,1,1,0,1,0,9,16
6446,1192,2012-06-19,3,2,0,25.3333339691162,33.6666679382324,1,60,3,2,203,67,1,1,0,1,0,9,165
6447,1192,2012-06-19,4,2,0,35,19,6,60,3,2,203,67,1,1,0,1,0,9,16575.50861,12563.64133,15.722111[illegible]
6448,1192,2012-06-20,0,3,0,3.66666674613953,7.66666650772095,0.333333343267441,11.666666[illegible]
6449,1192,2012-06-20,1,3,0,8.66666698455811,36.3333320617676,15,60,3,2,203,67,1,1,0,1,0,9,16

6450,1192,2012-06-20,2,3,0,32,27.6666660308838,0.333333343267441,60,3,2,203,67,1,1,0,1,0,9,1
6451,1192,2012-06-20,3,3,0,39.6666679382324,20,0.333333343267441,60,3,2,203,67,1,1,0,1,0,9,1
6452,1192,2012-06-20,4,3,0,24.3333339691162,31,4.66666650772095,60,3,2,203,67,1,1,0,1,0,9,16
6453,1192,2012-06-21,0,4,0,4.33333349227905,14.3333330154419,3.33333325386047,22,3,2,203,
6454,1192,2012-06-21,1,4,0,22,31.3333339691162,6.66666650772095,60,3,2,203,67,1,1,0,1,0,9,16
6455,1192,2012-06-21,2,4,0,25.3333339691162,33,1.66666662693024,60,3,2,203,67,1,1,0,1,0,9,16
6456,1192,2012-06-21,3,4,0,34.6666679382324,25,0.333333343267441,60,3,2,203,67,1,1,0,1,0,9,1
6457,1192,2012-06-21,4,4,0,36.3333320617676,21.3333339691162,2.33333325386047,60,3,2,203,
6458,1192,2012-06-22,0,5,0,0.666666686534882,1.66666662693024,0,2.33333325386047,3,2,203,
6459,1192,2012-06-22,1,5,0,18.6666660308838,30,11.3333330154419,60,3,2,203,67,1,1,0,1,0,9,16
6460,1192,2012-06-22,2,5,0,47.3333320617676,7.33333349227905,5.33333349227905,60,3,2,203,
6461,1192,2012-06-22,3,5,0,18.3333339691162,30.3333339691162,11.3333330154419,60,3,2,203,
6462,1192,2012-06-22,4,5,0,33.3333320617676,26,0.666666686534882,60,3,2,203,67,1,1,0,1,0,9,1
6463,1195,2012-04-05,0,4,0,60,0,0,60,1,2,203,74,1,1,1,0,0,12,16375.70692,14655.37573,13.18420
6464,1195,2012-04-05,1,4,0,17.3333339691162,33,9.66666698455811,60,3,2,203,74,1,1,1,0,0,12,1
6465,1195,2012-04-05,2,4,0,34,23,3,60,3,2,203,74,1,1,1,0,0,12,16375.70692,14655.37573,13.1842
6466,1195,2012-04-05,3,4,0,25.3333339691162,33.3333320617676,1.33333337306976,60,3,2,203,
6467,1195,2012-04-05,4,4,0,40,20,0,60,3,2,203,74,1,1,1,0,0,12,16375.70692,14655.37573,13.1842
6468,1195,2012-04-06,1,5,0,15.3333330154419,38.6666679382324,3,57,3,2,203,74,1,1,1,0,0,12,16
6469,1195,2012-04-06,2,5,0,37.3333320617676,19.3333339691162,3.33333325386047,60,3,2,203,
6470,1195,2012-04-06,3,5,0,39.3333320617676,19.6666660308838,1,60,3,2,203,74,1,1,1,0,0,12,16
6471,1195,2012-04-06,4,5,0,37.6666679382324,19.3333339691162,3,60,3,2,203,74,1,1,1,0,0,12,16
6472,1195,2012-04-07,0,6,1,0.333333343267441,2.33333325386047,0.333333343267441,3,3,2,20
6473,1195,2012-04-07,1,6,1,43.6666679382324,16.3333339691162,0,60,3,2,203,74,1,1,1,0,0,12,16
6474,1195,2012-04-07,2,6,1,27,15.6666669845581,17.3333339691162,60,3,2,203,74,1,1,1,0,0,12,1
6475,1195,2012-04-07,3,6,1,18.6666660308838,38,3.33333325386047,60,3,2,203,74,1,1,1,0,0,12,1
6476,1195,2012-04-07,4,6,1,21.3333339691162,29,9.66666698455811,60,3,2,203,74,1,1,1,0,0,12,1
6477,1195,2012-04-08,1,7,1,15.6666669845581,20,9.33333301544189,45,3,2,203,74,1,1,1,0,0,12,1
6478,1195,2012-04-08,2,7,1,49.3333320617676,10,0.666666686534882,60,3,2,203,74,1,1,1,0,0,12,
6479,1195,2012-04-08,3,7,1,42,17,1,60,3,2,203,74,1,1,1,0,0,12,16375.70692,14655.37573,13.1842
6480,1195,2012-04-08,4,7,1,46,14,0,60,3,2,203,74,1,1,1,0,0,12,16375.70692,14655.37573,13.1842
6481,1195,2012-04-09,1,1,0,3.33333325386047,12,1,16.3333339691162,3,2,203,74,1,1,1,0,0,12,16
6482,1195,2012-04-09,2,1,0,32.3333320617676,26.6666660308838,1,60,3,2,203,74,1,1,1,0,0,12,16
6483,1195,2012-04-09,3,1,0,28,23,9,60,3,2,203,74,1,1,1,0,0,12,16375.70692,14655.37573,13.1842
6484,1195,2012-04-09,4,1,0,39.3333320617676,16.3333339691162,4.33333349227905,60,3,2,203,
6485,1195,2012-04-10,1,2,0,22.3333339691162,25.6666660308838,4.33333349227905,52.333332
6486,1195,2012-04-10,2,2,0,49.3333320617676,10.3333330154419,0.333333343267441,60,3,2,20
6487,1195,2012-04-10,3,2,0,30,27.6666660308838,2.33333325386047,60,3,2,203,74,1,1,1,0,0,12,1
6488,1195,2012-04-10,4,2,0,38.6666679382324,16.6666660308838,4.66666650772095,60,3,2,203,
6489,1222,2010-11-30,0,2,0,23,29,1,53,1,2,203,83,0,1,0,1,0,9,16832.62164,15040.94259,13.96310
6490,1222,2010-11-30,1,2,0,19,38,3,60,3,2,203,83,0,1,0,1,0,9,16832.62164,15040.94259,13.96310
6491,1222,2010-11-30,2,2,0,19,38,3,60,3,2,203,83,0,1,0,1,0,9,16832.62164,15040.94259,13.96310
6492,1222,2010-11-30,3,2,0,9,37.6666679382324,13.3333330154419,60,3,2,203,83,0,1,0,1,0,9,168
6493,1222,2010-11-30,4,2,0,26.3333339691162,33.6666679382324,0,60,3,2,203,83,0,1,0,1,0,9,168
6494,1222,2010-12-02,0,4,0,60,0,0,60,1,2,203,83,0,1,0,1,0,9,16832.62164,15040.94259,13.963100
6495,1222,2010-12-02,1,4,0,19,33,8,60,1,2,203,83,0,1,0,1,0,9,16832.62164,15040.94259,13.96310
6496,1222,2010-12-03,0,5,0,35,11,0,46,1,2,203,83,0,1,0,1,0,9,16832.62164,15040.94259,13.96310
6497,1222,2010-12-03,1,5,0,13.6000003814697,35,11.3999996185303,60,3,2,203,83,0,1,0,1,0,9,16
6498,1222,2010-12-03,2,5,0,39,21,0,60,3,2,203,83,0,1,0,1,0,9,16832.62164,15040.94259,13.96310
6499,1222,2010-12-03,3,5,0,41.6666679382324,17.6666660308838,0.666666686534882,60,3,2,20

6500,1222,2010-12-03,4,5,0,39.6666679382324,20.3333339691162,0,60,3,2,203,83,0,1,0,1,0,9,168
6501,1222,2010-12-04,0,6,1,16,36,0,52,1,2,203,83,0,1,0,1,0,9,16832.62164,15040.94259,13.96310
6502,1222,2010-12-04,1,6,1,9.33333301544189,43,7.66666650772095,60,3,2,203,83,0,1,0,1,0,9,16
6503,1222,2010-12-04,2,6,1,20.25,34.25,5.5,60,3,2,203,83,0,1,0,1,0,9,16832.62164,15040.94259,1
6504,1222,2010-12-04,3,6,1,30,28,2,60,3,2,203,83,0,1,0,1,0,9,16832.62164,15040.94259,13.96310
6505,1222,2010-12-04,4,6,1,36,23.6666660308838,0.333333343267441,60,3,2,203,83,0,1,0,1,0,9,1
6506,1222,2010-12-06,0,1,0,12,32,13,57,1,2,203,83,0,1,0,1,0,9,16832.62164,15040.94259,13.9631
6507,1222,2010-12-06,1,1,0,9,35.3333320617676,15.6666669845581,60,3,2,203,83,0,1,0,1,0,9,168
6508,1222,2010-12-06,2,1,0,21,28,11,60,3,2,203,83,0,1,0,1,0,9,16832.62164,15040.94259,13.9631
6509,1222,2010-12-06,3,1,0,22.6666660308838,36.1666679382324,1.1666662693024,60,3,2,203,
6510,1222,2010-12-06,4,1,0,32.6666679382324,27.3333339691162,0,60,3,2,203,83,0,1,0,1,0,9,168
6511,1222,2010-12-07,0,2,0,40.6666679382324,18.6666660308838,0.666666686534882,60,3,2,20
6512,1222,2010-12-07,1,2,0,27.6666660308838,28,4.33333349227905,60,3,2,203,83,0,1,0,1,0,9,16
6513,1222,2010-12-07,2,2,0,27.6666660308838,28.3333339691162,4,60,3,2,203,83,0,1,0,1,0,9,168
6514,1222,2010-12-07,3,2,0,41.3333320617676,18.6666660308838,0,60,3,2,203,83,0,1,0,1,0,9,168
6515,1222,2010-12-07,4,2,0,44,15.6666669845581,0.333333343267441,60,3,2,203,83,0,1,0,1,0,9,1
6516,1222,2010-12-08,0,3,0,16,14,0.5,30.5,2,2,203,83,0,1,0,1,0,9,16832.62164,15040.94259,13.963
6517,1222,2010-12-08,1,3,0,22.6666660308838,36.6666679382324,0.666666686534882,60,3,2,20
6518,1222,2010-12-08,2,3,0,13.6666669845581,46,0.333333343267441,60,3,2,203,83,0,1,0,1,0,9,1
6519,1222,2010-12-08,3,3,0,6.5,53,0.5,60,2,2,203,83,0,1,0,1,0,9,16832.62164,15040.94259,13.9631
6520,1222,2010-12-08,4,3,0,41,18.3333339691162,0.666666686534882,60,3,2,203,83,0,1,0,1,0,9,1
6521,1222,2010-12-14,0,2,0,12.5,18.5,0,31,2,2,203,83,0,1,0,1,0,9,16832.62164,15040.94259,13.963
6522,1222,2010-12-14,1,2,0,8.33333301544189,45.6666679382324,6,60,3,2,203,83,0,1,0,1,0,9,168
6523,1222,2010-12-14,2,2,0,7.66666650772095,52,0.333333343267441,60,3,2,203,83,0,1,0,1,0,9,1
6524,1222,2010-12-14,3,2,0,15.6666669845581,44.3333320617676,0,60,3,2,203,83,0,1,0,1,0,9,168
6525,1222,2010-12-14,4,2,0,34,25,1,60,3,2,203,83,0,1,0,1,0,9,16832.62164,15040.94259,13.96310
6526,1222,2010-12-15,3,3,0,11,47,2,60,1,2,203,83,0,1,0,1,0,9,16832.62164,15040.94259,13.96310
6527,1222,2010-12-15,4,3,0,40.3333320617676,17.6666660308838,2,60,3,2,203,83,0,1,0,1,0,9,168
6528,1223,2012-06-16,0,6,1,54,6,0,60,1,2,203,67,0,1,1,1,0,11,16858.17065,18090.34459,12.40154
6529,1223,2012-06-16,1,6,1,23,33.6666679382324,3.33333325386047,60,3,2,203,67,0,1,1,1,0,11,1
6530,1223,2012-06-16,2,6,1,30.3333339691162,29.6666660308838,0,60,3,2,203,67,0,1,1,1,0,11,16
6531,1223,2012-06-16,3,6,1,39,20,1,60,3,2,203,67,0,1,1,1,0,11,16858.17065,18090.34459,12.4015
6532,1223,2012-06-16,4,6,1,38,21,1,60,3,2,203,67,0,1,1,1,0,11,16858.17065,18090.34459,12.4015
6533,1223,2012-06-17,1,7,1,17,34.3333320617676,4.66666650772095,56,3,2,203,67,0,1,1,1,0,11,1
6534,1223,2012-06-17,2,7,1,34,25.6666660308838,0.333333343267441,60,3,2,203,67,0,1,1,1,0,11,
6535,1223,2012-06-17,3,7,1,25,34.3333320617676,0.666666686534882,60,3,2,203,67,0,1,1,1,0,11,
6536,1223,2012-06-17,4,7,1,47.3333320617676,11.6666669845581,1,60,3,2,203,67,0,1,1,1,0,11,16
6537,1223,2012-06-18,1,1,0,10,19.6666660308838,21.6666660308838,51.3333320617676,3,2,203,
6538,1223,2012-06-18,2,1,0,35.3333320617676,23.6666660308838,1,60,3,2,203,67,0,1,1,1,0,11,16
6539,1223,2012-06-18,3,1,0,15.6666669845581,38.3333320617676,6,60,3,2,203,67,0,1,1,1,0,11,16
6540,1223,2012-06-18,4,1,0,36.3333320617676,23.6666660308838,0,60,3,2,203,67,0,1,1,1,0,11,16
6541,1223,2012-06-19,0,2,0,32.6666679382324,4,0.666666686534882,37.3333320617676,3,2,203,
6542,1223,2012-06-19,1,2,0,24.6666660308838,25.6666660308838,9.66666698455811,60,3,2,203,
6543,1223,2012-06-19,2,2,0,46.3333320617676,12.3333330154419,1.33333337306976,60,3,2,203,
6544,1223,2012-06-19,3,2,0,31,27.6666660308838,1.33333337306976,60,3,2,203,67,0,1,1,1,0,11,1
6545,1223,2012-06-19,4,2,0,45.6666679382324,13.6666669845581,0.666666686534882,60,3,2,20
6546,1223,2012-06-20,1,3,0,10,32.6666679382324,8.66666698455811,51.3333320617676,3,2,203,
6547,1223,2012-06-20,2,3,0,35.6666679382324,23.6666660308838,0.666666686534882,60,3,2,20
6548,1223,2012-06-20,3,3,0,15.3333330154419,42.6666679382324,2,60,3,2,203,67,0,1,1,1,0,11,16
6549,1223,2012-06-20,4,3,0,31,28,1,60,3,2,203,67,0,1,1,1,0,11,16858.17065,18090.34459,12.4015

6550,1223,2012-06-21,1,4,0,19.3333339691162,30.6666660308838,7.66666650772095,57.666667
6551,1223,2012-06-21,2,4,0,41.3333320617676,18.6666660308838,0,60,3,2,203,67,0,1,1,1,0,11,16
6552,1223,2012-06-21,3,4,0,49.3333320617676,10.6666669845581,0,60,3,2,203,67,0,1,1,1,0,11,16
6553,1223,2012-06-21,4,4,0,43,17,0,60,3,2,203,67,0,1,1,1,0,11,16858.17065,18090.34459,12.4015
6554,1223,2012-06-22,0,5,0,0.666666686534882,3,0,3.66666674613953,3,2,203,67,0,1,1,1,0,11,16
6555,1223,2012-06-22,1,5,0,21.6666660308838,31,7.33333349227905,60,3,2,203,67,0,1,1,1,0,11,1
6556,1223,2012-06-22,2,5,0,37.3333320617676,22.6666660308838,0,60,3,2,203,67,0,1,1,1,0,11,16
6557,1223,2012-06-22,3,5,0,36.6666679382324,21.3333339691162,2,60,3,2,203,67,0,1,1,1,0,11,16
6558,1223,2012-06-22,4,5,0,46.6666679382324,13,0.333333343267441,60,3,2,203,67,0,1,1,1,0,11,
6559,1225,2012-06-29,0,5,0,49.3333320617676,9,1.33333337306976,59.6666679382324,3,2,203,7
6560,1225,2012-06-29,1,5,0,20,40,0,60,3,2,203,75,1,0,1,0,0,11,16793.12444,14125.37203,11.7014
6561,1225,2012-06-29,2,5,0,30.3333339691162,29.6666660308838,0,60,3,2,203,75,1,0,1,0,0,11,16
6562,1225,2012-06-29,3,5,0,32.6666679382324,27.3333339691162,0,60,3,2,203,75,1,0,1,0,0,11,16
6563,1225,2012-06-29,4,5,0,35.3333320617676,24.3333339691162,0.333333343267441,60,3,2,20
6564,1225,2012-06-30,0,6,1,8.33333301544189,2.33333325386047,0.333333343267441,11,3,2,20
6565,1225,2012-06-30,1,6,1,17.6666660308838,42,0.333333343267441,60,3,2,203,75,1,0,1,0,0,11,
6566,1225,2012-06-30,2,6,1,23.6666660308838,35.3333320617676,1,60,3,2,203,75,1,0,1,0,0,11,16
6567,1225,2012-06-30,3,6,1,29.6666660308838,30.3333339691162,0,60,3,2,203,75,1,0,1,0,0,11,16
6568,1225,2012-06-30,4,6,1,27.6666660308838,32.3333320617676,0,60,3,2,203,75,1,0,1,0,0,11,16
6569,1225,2012-07-02,0,1,0,7,2.33333325386047,0,9.33333301544189,3,2,203,75,1,0,1,0,0,11,167
6570,1225,2012-07-02,1,1,0,15.3333330154419,44.6666679382324,0,60,3,2,203,75,1,0,1,0,0,11,16
6571,1225,2012-07-02,2,1,0,34,25.6666660308838,0.333333343267441,60,3,2,203,75,1,0,1,0,0,11,
6572,1225,2012-07-02,3,1,0,34.3333320617676,25.6666660308838,0,60,3,2,203,75,1,0,1,0,0,11,16
6573,1225,2012-07-02,4,1,0,28.6666660308838,31.3333339691162,0,60,3,2,203,75,1,0,1,0,0,11,16
6574,1225,2012-07-04,0,3,0,22.3333339691162,36.3333320617676,0.666666686534882,59.33333
6575,1225,2012-07-04,1,3,0,22.6666660308838,37.3333320617676,0,60,3,2,203,75,1,0,1,0,0,11,16
6576,1225,2012-07-04,2,3,0,24.6666660308838,35.3333320617676,0,60,3,2,203,75,1,0,1,0,0,11,16
6577,1225,2012-07-04,3,3,0,20,39.6666679382324,0.333333343267441,60,3,2,203,75,1,0,1,0,0,11,
6578,1225,2012-07-04,4,3,0,18,41.6666679382324,0.333333343267441,60,3,2,203,75,1,0,1,0,0,11,
6579,1225,2012-07-05,0,4,0,31.6666660308838,10.3333330154419,0,42,3,2,203,75,1,0,1,0,0,11,16
6580,1225,2012-07-05,1,4,0,25.6666660308838,33,1.33333337306976,60,3,2,203,75,1,0,1,0,0,11,1
6581,1225,2012-07-05,2,4,0,52,4.33333349227905,0.333333343267441,56.6666679382324,3,2,20
6582,1225,2012-07-05,3,4,0,0,1.33333337306976,0,1.33333337306976,3,2,203,75,1,0,1,0,0,11,167
6583,1225,2012-07-05,4,4,0,20,38,2,60,3,2,203,75,1,0,1,0,0,11,16793.12444,14125.37203,11.7014
6584,1225,2012-07-06,0,5,0,13.6666669845581,0,0,13.6666669845581,3,2,203,75,1,0,1,0,0,11,167
6585,1225,2012-07-06,1,5,0,4,0.666666686534882,0,4.66666650772095,3,2,203,75,1,0,1,0,0,11,16
6586,1225,2012-07-06,2,5,0,43,17,0,60,3,2,203,75,1,0,1,0,0,11,16793.12444,14125.37203,11.7014
6587,1225,2012-07-06,3,5,0,51.3333320617676,8.66666698455811,0,60,3,2,203,75,1,0,1,0,0,11,16
6588,1225,2012-07-06,4,5,0,42,17.3333339691162,0.666666686534882,60,3,2,203,75,1,0,1,0,0,11,
6589,1225,2012-07-08,0,7,1,2.66666674613953,0.666666686534882,0.666666686534882,4,3,2,20
6590,1225,2012-07-08,1,7,1,53.3333320617676,6,0.666666686534882,60,3,2,203,75,1,0,1,0,0,11,1
6591,1225,2012-07-08,2,7,1,36.3333320617676,23.3333339691162,0.333333343267441,60,3,2,20
6592,1225,2012-07-08,3,7,1,34.6666679382324,25.3333339691162,0,60,3,2,203,75,1,0,1,0,0,11,16
6593,1225,2012-07-08,4,7,1,14,29.3333339691162,0.666666686534882,44,3,2,203,75,1,0,1,0,0,11,
6594,1225,2012-07-09,0,1,0,30,25.6666660308838,0.666666686534882,56.3333320617676,3,2,20
6595,1225,2012-07-09,1,1,0,29,31,0,60,3,2,203,75,1,0,1,0,0,11,16793.12444,14125.37203,11.7014
6596,1225,2012-07-09,2,1,0,28.3333339691162,31,0.666666686534882,60,3,2,203,75,1,0,1,0,0,11,
6597,1225,2012-07-09,3,1,0,41,19,0,60,3,2,203,75,1,0,1,0,0,11,16793.12444,14125.37203,11.7014
6598,1225,2012-07-09,4,1,0,32,26.3333339691162,1.66666662693024,60,3,2,203,75,1,0,1,0,0,11,1
6599,1225,2012-07-10,0,2,0,26.3333339691162,5.33333349227905,0.666666686534882,32.33333

6600,1225,2012-07-10,1,2,0,29.6666660308838,28.6666660308838,1.66666662693024,60,3,2,203,
6601,1225,2012-07-10,2,2,0,38.6666679382324,21.3333339691162,0,60,3,2,203,75,1,0,1,0,0,11,16
6602,1225,2012-07-10,3,2,0,35,24.3333339691162,0.666666686534882,60,3,2,203,75,1,0,1,0,0,11,
6603,1225,2012-07-10,4,2,0,27.3333339691162,19.3333339691162,0.333333343267441,47,3,2,20
6604,1225,2012-07-11,0,3,0,36.3333320617676,23.3333339691162,0.333333343267441,60,3,2,20
6605,1225,2012-07-11,1,3,0,40.6666679382324,19.3333339691162,0,60,3,2,203,75,1,0,1,0,0,11,16
6606,1225,2012-07-11,2,3,0,34,25.6666660308838,0.333333343267441,60,3,2,203,75,1,0,1,0,0,11,
6607,1225,2012-07-11,3,3,0,36,24,0,60,3,2,203,75,1,0,1,0,0,11,16793.12444,14125.37203,11.7014
6608,1225,2012-07-11,4,3,0,26,34,0,60,3,2,203,75,1,0,1,0,0,11,16793.12444,14125.37203,11.7014
6609,1228,2012-06-20,0,3,0,60,0,0,60,1,2,203,65,0,1,1,1,0,12,17042.39645,14407.10233,11.740291
6610,1228,2012-06-20,1,3,0,27,31,2,60,3,2,203,65,0,1,1,1,0,12,17042.39645,14407.10233,11.7402
6611,1228,2012-06-20,2,3,0,40.3333320617676,19.6666660308838,0,60,3,2,203,65,0,1,1,1,0,12,17
6612,1228,2012-06-20,3,3,0,38.3333320617676,19.3333339691162,2.33333325386047,60,3,2,203,
6613,1228,2012-06-20,4,3,0,33,27,0,60,3,2,203,65,0,1,1,1,0,12,17042.39645,14407.10233,11.7402
6614,1228,2012-06-21,0,4,0,0,1,0.333333343267441,1.33333337306976,3,2,203,65,0,1,1,1,0,12,17
6615,1228,2012-06-21,1,4,0,26,34,0,60,3,2,203,65,0,1,1,1,0,12,17042.39645,14407.10233,11.7402
6616,1228,2012-06-21,2,4,0,28.3333339691162,31.6666660308838,0,60,3,2,203,65,0,1,1,1,0,12,17
6617,1228,2012-06-21,3,4,0,43.3333320617676,16.6666660308838,0,60,3,2,203,65,0,1,1,1,0,12,17
6618,1228,2012-06-21,4,4,0,32.3333320617676,25.6666660308838,2,60,3,2,203,65,0,1,1,1,0,12,17
6619,1228,2012-06-22,0,5,0,4,7,0.333333343267441,11.3333330154419,3,2,203,65,0,1,1,1,0,12,17
6620,1228,2012-06-22,1,5,0,26.6666660308838,33.3333320617676,0,60,3,2,203,65,0,1,1,1,0,12,17
6621,1228,2012-06-22,2,5,0,35.3333320617676,24.6666660308838,0,60,3,2,203,65,0,1,1,1,0,12,17
6622,1228,2012-06-22,3,5,0,38.3333320617676,20,1.66666662693024,60,3,2,203,65,0,1,1,1,0,12,1
6623,1228,2012-06-22,4,5,0,41.3333320617676,18.6666660308838,0,60,3,2,203,65,0,1,1,1,0,12,17
6624,1228,2012-06-23,1,6,1,16.6666660308838,31.6666660308838,1.33333337306976,49.666667
6625,1228,2012-06-23,2,6,1,38.6666679382324,21,0.333333343267441,60,3,2,203,65,0,1,1,1,0,12,
6626,1228,2012-06-23,3,6,1,39,20.3333339691162,0.666666686534882,60,3,2,203,65,0,1,1,1,0,12,
6627,1228,2012-06-23,4,6,1,37.3333320617676,22.3333339691162,0.333333343267441,60,3,2,20
6628,1228,2012-06-24,1,7,1,27.3333339691162,22.6666660308838,0,50,3,2,203,65,0,1,1,1,0,12,17
6629,1228,2012-06-24,2,7,1,40,20,0,60,3,2,203,65,0,1,1,1,0,12,17042.39645,14407.10233,11.7402
6630,1228,2012-06-24,3,7,1,35.6666679382324,23.6666660308838,0.666666686534882,60,3,2,20
6631,1228,2012-06-24,4,7,1,26.6666660308838,18.6666660308838,0.666666686534882,46,3,2,20
6632,1228,2012-06-25,1,1,0,16,26,0,42,3,2,203,65,0,1,1,1,0,12,17042.39645,14407.10233,11.7402
6633,1228,2012-06-25,2,1,0,30.6666660308838,28.3333339691162,1,60,3,2,203,65,0,1,1,1,0,12,17
6634,1228,2012-06-25,3,1,0,40.6666679382324,19.3333339691162,0,60,3,2,203,65,0,1,1,1,0,12,17
6635,1228,2012-06-25,4,1,0,37.3333320617676,17.6666660308838,2.66666674613953,57.666667
6636,1228,2012-06-26,1,2,0,12.3333330154419,35.6666679382324,1,49,3,2,203,65,0,1,1,1,0,12,17
6637,1228,2012-06-26,2,2,0,32,28,0,60,3,2,203,65,0,1,1,1,0,12,17042.39645,14407.10233,11.7402
6638,1228,2012-06-26,3,2,0,28.6666660308838,30.6666660308838,0.666666686534882,60,3,2,20
6639,1228,2012-06-26,4,2,0,37,22.6666660308838,0.333333343267441,60,3,2,203,65,0,1,1,1,0,12,
6640,1229,2010-12-15,1,3,0,6,29,6,41,3,2,203,77,1,1,0,1,0,11,16792.78982,13837.30984,14.78997
6641,1229,2010-12-15,2,3,0,19.3333339691162,32.6666679382324,8,60,3,2,203,77,1,1,0,1,0,11,16
6642,1229,2010-12-15,3,3,0,22.6666660308838,34,3.33333325386047,60,3,2,203,77,1,1,0,1,0,11,1
6643,1229,2010-12-15,4,3,0,22,38,0,60,2,2,203,77,1,1,0,1,0,11,16792.78982,13837.30984,14.7899
6644,1229,2010-12-16,0,4,0,13,19.5,2,34.5,2,2,203,77,1,1,0,1,0,11,16792.78982,13837.30984,14.7
6645,1229,2010-12-16,1,4,0,9.66666698455811,40.6666679382324,9.66666698455811,60,3,2,203,
6646,1229,2010-12-16,2,4,0,6.66666650772095,33.6666679382324,19.6666660308838,60,3,2,203,
6647,1229,2010-12-16,3,4,0,17.6666660308838,36,6.33333349227905,60,3,2,203,77,1,1,0,1,0,11,1
6648,1229,2010-12-16,4,4,0,39.75,19.5,0.75,60,3,2,203,77,1,1,0,1,0,11,16792.78982,13837.30984,1
6649,1229,2010-12-17,0,5,0,25,24.3333339691162,1,50.3333320617676,3,2,203,77,1,1,0,1,0,11,16

6650,1229,2010-12-17,1,5,0,24.75,33.5,1.75,60,3,2,203,77,1,1,0,1,0,11,16792.78982,13837.30984,
6651,1229,2010-12-17,2,5,0,18,41.6666679382324,0.333333343267441,60,3,2,203,77,1,1,0,1,0,11,
6652,1229,2010-12-17,3,5,0,23.6666660308838,33.3333320617676,3,60,3,2,203,77,1,1,0,1,0,11,16
6653,1229,2010-12-17,4,5,0,34.6666679382324,24.6666660308838,0.666666686534882,60,3,2,20
6654,1229,2010-12-18,0,6,1,1,8,3,12,1,2,203,77,1,1,0,1,0,11,16792.78982,13837.30984,14.789976
6655,1229,2010-12-18,1,6,1,18,31.7999992370605,10.1999998092651,60,3,2,203,77,1,1,0,1,0,11,
6656,1229,2010-12-18,2,6,1,22,34.6666679382324,3.33333325386047,60,3,2,203,77,1,1,0,1,0,11,
6657,1229,2010-12-18,3,6,1,13.6666669845581,38,8.33333301544189,60,3,2,203,77,1,1,0,1,0,11,
6658,1229,2010-12-18,4,6,1,27.6666660308838,28.3333339691162,4,60,3,2,203,77,1,1,0,1,0,11,16
6659,1229,2010-12-19,1,0,1,19,31,0.666666686534882,50.6666679382324,3,2,203,77,1,1,0,1,0,11,
6660,1229,2010-12-19,2,0,1,22.5,34.6666679382324,2.83333325386047,60,3,2,203,77,1,1,0,1,0,11
6661,1229,2010-12-19,3,0,1,23.6666660308838,27,9.33333301544189,60,3,2,203,77,1,1,0,1,0,11,
6662,1229,2010-12-19,4,0,1,29,28,3,60,3,2,203,77,1,1,0,1,0,11,16792.78982,13837.30984,14.7899
6663,1229,2010-12-20,1,1,0,17,29,7.66666650772095,53.6666679382324,3,2,203,77,1,1,0,1,0,11,
6664,1229,2010-12-20,2,1,0,20,37.6666679382324,2.33333325386047,60,3,2,203,77,1,1,0,1,0,11,
6665,1229,2010-12-20,3,1,0,30,27,3,60,3,2,203,77,1,1,0,1,0,11,16792.78982,13837.30984,14.7899
6666,1229,2010-12-20,4,1,0,31,27,2,60,3,2,203,77,1,1,0,1,0,11,16792.78982,13837.30984,14.7899
6667,1229,2010-12-21,4,2,0,32.6666679382324,25.6666660308838,1.66666662693024,60,3,2,203,
6668,1230,2012-06-26,0,2,0,16,34,10,60,1,2,203,77,1,1,1,0,0,7,16726.97049,13921.3779,12.932877
6669,1230,2012-06-26,1,2,0,21.6666660308838,36.6666679382324,1.66666662693024,60,3,2,203,
6670,1230,2012-06-26,2,2,0,41,17,2,60,3,2,203,77,1,1,1,0,0,7,16726.97049,13921.3779,12.932877
6671,1230,2012-06-26,3,2,0,31,27.3333339691162,1.66666662693024,60,3,2,203,77,1,1,1,0,0,7,16
6672,1230,2012-06-26,4,2,0,25.3333339691162,30,4.66666650772095,60,3,2,203,77,1,1,1,0,0,7,16
6673,1230,2012-06-27,0,3,0,4.33333349227905,14.3333330154419,0,18.6666660308838,3,2,203,7
6674,1230,2012-06-27,1,3,0,28.6666660308838,21.3333339691162,10,60,3,2,203,77,1,1,1,0,0,7,16
6675,1230,2012-06-27,2,3,0,33,24.6666660308838,2.33333325386047,60,3,2,203,77,1,1,1,0,0,7,16
6676,1230,2012-06-27,3,3,0,29,27.6666660308838,3.33333325386047,60,3,2,203,77,1,1,1,0,0,7,16
6677,1230,2012-06-27,4,3,0,36.3333320617676,22,1.66666662693024,60,3,2,203,77,1,1,1,0,0,7,16
6678,1230,2012-06-28,0,4,0,7.66666650772095,16.6666660308838,0.333333343267441,24.66666
6679,1230,2012-06-28,1,4,0,17.3333339691162,36,6.66666650772095,60,3,2,203,77,1,1,1,0,0,7,16
6680,1230,2012-06-28,2,4,0,33.6666679382324,23.6666660308838,2.66666674613953,60,3,2,203,
6681,1230,2012-06-28,3,4,0,45.3333320617676,12,2.66666674613953,60,3,2,203,77,1,1,1,0,0,7,16
6682,1230,2012-06-28,4,4,0,27.6666660308838,31.3333339691162,1,60,3,2,203,77,1,1,1,0,0,7,167
6683,1230,2012-06-29,0,5,0,3.33333325386047,12.6666669845581,1.66666662693024,17.666666
6684,1230,2012-06-29,1,5,0,13.3333330154419,42.6666679382324,4,60,3,2,203,77,1,1,1,0,0,7,167
6685,1230,2012-06-29,2,5,0,26,28.3333339691162,5.66666650772095,60,3,2,203,77,1,1,1,0,0,7,16
6686,1230,2012-06-29,3,5,0,29.3333339691162,24,6.66666650772095,60,3,2,203,77,1,1,1,0,0,7,16
6687,1230,2012-06-29,4,5,0,43.3333320617676,15.6666669845581,1,60,3,2,203,77,1,1,1,0,0,7,167
6688,1230,2012-06-30,0,6,1,3.66666674613953,3.66666674613953,0.666666686534882,8,3,2,203,
6689,1230,2012-06-30,1,6,1,17.6666660308838,34.6666679382324,7.66666650772095,60,3,2,203,
6690,1230,2012-06-30,2,6,1,16.3333339691162,35.6666679382324,8,60,3,2,203,77,1,1,1,0,0,7,167
6691,1230,2012-06-30,3,6,1,29.3333339691162,30.3333339691162,0.333333343267441,60,3,2,20
6692,1230,2012-06-30,4,6,1,34.6666679382324,18.3333339691162,0.333333343267441,53.33333
6693,1230,2012-07-01,0,7,1,24,15,0.333333343267441,39.3333320617676,3,2,203,77,1,1,1,0,0,7,1
6694,1230,2012-07-01,1,7,1,36.6666679382324,20.6666660308838,2.66666674613953,60,3,2,203,
6695,1230,2012-07-01,2,7,1,30.6666660308838,19.6666660308838,9.66666698455811,60,3,2,203,
6696,1230,2012-07-01,3,7,1,26.6666660308838,27.3333339691162,6,60,3,2,203,77,1,1,1,0,0,7,167
6697,1230,2012-07-01,4,7,1,34,16.6666660308838,0.666666686534882,51.3333320617676,3,2,20
6698,1230,2012-07-02,0,1,0,0.666666686534882,4.66666650772095,0.333333343267441,5.66666
6699,1230,2012-07-02,1,1,0,16,40.6666679382324,3.33333325386047,60,3,2,203,77,1,1,1,0,0,7,16

6700,1230,2012-07-02,2,1,0,33,21.3333339691162,5.66666650772095,60,3,2,203,77,1,1,1,0,0,7,16
6701,1230,2012-07-02,3,1,0,35.3333320617676,18.6666660308838,6,60,3,2,203,77,1,1,1,0,0,7,167
6702,1230,2012-07-02,4,1,0,35.3333320617676,13.6666669845581,0.666666686534882,49.66666
6703,1231,2010-12-14,0,2,0,26.6666660308838,18,0,44.6666679382324,3,2,203,78,0,1,0,1,0,7,167
6704,1231,2010-12-14,1,2,0,38.6666679382324,21.3333339691162,0,60,3,2,203,78,0,1,0,1,0,7,167
6705,1231,2010-12-14,2,2,0,23.3333339691162,36.6666679382324,0,60,3,2,203,78,0,1,0,1,0,7,167
6706,1231,2010-12-14,3,2,0,17,43,0,60,2,2,203,78,0,1,0,1,0,7,16793.12444,14125.37203,11.701476
6707,1231,2010-12-14,4,2,0,39,21,0,60,3,2,203,78,0,1,0,1,0,7,16793.12444,14125.37203,11.701476
6708,1231,2010-12-15,0,3,0,40,20,0,60,1,2,203,78,0,1,0,1,0,7,16793.12444,14125.37203,11.701476
6709,1231,2010-12-15,1,3,0,10,49.6666679382324,0.333333343267441,60,3,2,203,78,0,1,0,1,0,7,1
6710,1231,2010-12-15,2,3,0,45,15,0,60,3,2,203,78,0,1,0,1,0,7,16793.12444,14125.37203,11.701476
6711,1231,2010-12-15,3,3,0,38.5,21.5,0,60,3,2,203,78,0,1,0,1,0,7,16793.12444,14125.37203,11.701
6712,1231,2010-12-15,4,3,0,32.75,27.25,0,60,3,2,203,78,0,1,0,1,0,7,16793.12444,14125.37203,11.7
6713,1231,2010-12-16,0,4,0,16,40,0.333333343267441,56.3333320617676,3,2,203,78,0,1,0,1,0,7,1
6714,1231,2010-12-16,1,4,0,18.3333339691162,41.3333320617676,0.333333343267441,60,3,2,203
6715,1231,2010-12-16,2,4,0,31,27.6666660308838,1.33333337306976,60,3,2,203,78,0,1,0,1,0,7,16
6716,1231,2010-12-16,3,4,0,28,30.6666660308838,1.33333337306976,60,3,2,203,78,0,1,0,1,0,7,16
6717,1231,2010-12-16,4,4,0,36.6666679382324,23.3333339691162,0,60,3,2,203,78,0,1,0,1,0,7,167
6718,1231,2010-12-17,0,5,0,12,26.5,0,38.5,2,2,203,78,0,1,0,1,0,7,16793.12444,14125.37203,11.701
6719,1231,2010-12-17,1,5,0,17.6666660308838,41.6666679382324,0.666666686534882,60,3,2,203
6720,1231,2010-12-17,2,5,0,39.3333320617676,20.6666660308838,0,60,3,2,203,78,0,1,0,1,0,7,167
6721,1231,2010-12-17,3,5,0,32.6666679382324,26.3333339691162,1,60,3,2,203,78,0,1,0,1,0,7,167
6722,1231,2010-12-17,4,5,0,33.5999984741211,26.3999996185303,0,60,3,2,203,78,0,1,0,1,0,7,167
6723,1231,2010-12-18,0,6,1,27.6666660308838,32.3333320617676,0,60,3,2,203,78,0,1,0,1,0,7,167
6724,1231,2010-12-18,1,6,1,13.6666669845581,46.3333320617676,0,60,3,2,203,78,0,1,0,1,0,7,167
6725,1231,2010-12-18,2,6,1,36.6666679382324,23.3333339691162,0,60,3,2,203,78,0,1,0,1,0,7,167
6726,1231,2010-12-18,3,6,1,22,38,0,60,3,2,203,78,0,1,0,1,0,7,16793.12444,14125.37203,11.701476
6727,1231,2010-12-18,4,6,1,38,22,0,60,3,2,203,78,0,1,0,1,0,7,16793.12444,14125.37203,11.701476
6728,1231,2010-12-19,0,0,1,15,42.5,1,58.5,2,2,203,78,0,1,0,1,0,7,16793.12444,14125.37203,11.701
6729,1231,2010-12-19,1,0,1,21.6666660308838,38.3333320617676,0,60,3,2,203,78,0,1,0,1,0,7,167
6730,1231,2010-12-19,2,0,1,43.3333320617676,16.6666660308838,0,60,3,2,203,78,0,1,0,1,0,7,167
6731,1231,2010-12-19,3,0,1,46,14,0,60,3,2,203,78,0,1,0,1,0,7,16793.12444,14125.37203,11.701476
6732,1231,2010-12-19,4,0,1,39.3333320617676,20.6666660308838,0,60,3,2,203,78,0,1,0,1,0,7,167
6733,1231,2010-12-21,0,2,0,27,32,0,59,3,2,203,78,0,1,0,1,0,7,16793.12444,14125.37203,11.701476
6734,1231,2010-12-21,1,2,0,33,27,0,60,3,2,203,78,0,1,0,1,0,7,16793.12444,14125.37203,11.701476
6735,1231,2010-12-21,2,2,0,47,13,0,60,3,2,203,78,0,1,0,1,0,7,16793.12444,14125.37203,11.701476
6736,1231,2010-12-21,3,2,0,49.3333320617676,10.6666669845581,0,60,3,2,203,78,0,1,0,1,0,7,167
6737,1233,2012-06-28,0,4,0,9,33,18,60,1,2,203,69,1,1,0,1,0,8,16790.51585,13868.94857,14.719129
6738,1233,2012-06-28,1,4,0,19.3333339691162,25,15.6666669845581,60,3,2,203,69,1,1,0,1,0,8,16
6739,1233,2012-06-28,2,4,0,36.6666679382324,16.6666660308838,6.66666650772095,60,3,2,203,
6740,1233,2012-06-28,3,4,0,18.3333339691162,21.6666660308838,20,60,3,2,203,69,1,1,0,1,0,8,16
6741,1233,2012-06-28,4,4,0,23.6666660308838,28.6666660308838,7.66666650772095,60,3,2,203,
6742,1233,2012-06-29,0,5,0,1,17,12,30,3,2,203,69,1,1,0,1,0,8,16790.51585,13868.94857,14.719129
6743,1233,2012-06-29,1,5,0,7.66666650772095,27.3333339691162,25,60,3,2,203,69,1,1,0,1,0,8,16
6744,1233,2012-06-29,2,5,0,6,30,24,60,3,2,203,69,1,1,0,1,0,8,16790.51585,13868.94857,14.719129
6745,1233,2012-06-29,3,5,0,12,22.3333339691162,25.6666660308838,60,3,2,203,69,1,1,0,1,0,8,16
6746,1233,2012-06-29,4,5,0,20.3333339691162,19.6666660308838,20,60,3,2,203,69,1,1,0,1,0,8,16
6747,1233,2012-06-30,0,6,1,1.33333337306976,15.6666669845581,5.33333349227905,22.3333339
6748,1233,2012-06-30,1,6,1,17.3333339691162,31,11.6666669845581,60,3,2,203,69,1,1,0,1,0,8,16
6749,1233,2012-06-30,2,6,1,23.6666660308838,19.3333339691162,17,60,3,2,203,69,1,1,0,1,0,8,16

6750,1233,2012-06-30,3,6,1,26.3333339691162,19,14.6666669845581,60,3,2,203,69,1,1,0,1,0,8,16
6751,1233,2012-06-30,4,6,1,27.3333339691162,20.6666660308838,12,60,3,2,203,69,1,1,0,1,0,8,16
6752,1233,2012-07-01,0,7,1,4,11.3333330154419,5.33333349227905,20.6666660308838,3,2,203,6
6753,1233,2012-07-01,1,7,1,6.33333349227905,30.6666660308838,23,60,3,2,203,69,1,1,0,1,0,8,16
6754,1233,2012-07-01,2,7,1,25.3333339691162,23,11.6666669845581,60,3,2,203,69,1,1,0,1,0,8,16
6755,1233,2012-07-01,3,7,1,24.3333339691162,13,22.6666660308838,60,3,2,203,69,1,1,0,1,0,8,16
6756,1233,2012-07-01,4,7,1,25.3333339691162,19,15.6666669845581,60,3,2,203,69,1,1,0,1,0,8,16
6757,1233,2012-07-02,0,1,0,4.33333349227905,25.6666660308838,11.6666669845581,41.666667
6758,1233,2012-07-02,1,1,0,8.33333301544189,34.6666679382324,17,60,3,2,203,69,1,1,0,1,0,8,16
6759,1233,2012-07-02,2,1,0,23,28.6666660308838,8.33333301544189,60,3,2,203,69,1,1,0,1,0,8,16
6760,1233,2012-07-02,3,1,0,33.3333320617676,6.66666650772095,20,60,3,2,203,69,1,1,0,1,0,8,16
6761,1233,2012-07-02,4,1,0,17.3333339691162,30.3333339691162,12.3333330154419,60,3,2,203,
6762,1233,2012-07-03,0,2,0,8,19.3333339691162,23.3333339691162,50.6666679382324,3,2,203,6
6763,1233,2012-07-03,1,2,0,25.3333339691162,22.3333339691162,12.3333330154419,60,3,2,203,
6764,1233,2012-07-03,2,2,0,11,21.3333339691162,27.6666660308838,60,3,2,203,69,1,1,0,1,0,8,16
6765,1233,2012-07-03,3,2,0,22,17.6666660308838,20.3333339691162,60,3,2,203,69,1,1,0,1,0,8,16
6766,1233,2012-07-03,4,2,0,21.6666660308838,33.6666679382324,4.66666650772095,60,3,2,203,
6767,1233,2012-07-04,0,3,0,11,9.66666698455811,15.3333330154419,36,3,2,203,69,1,1,0,1,0,8,16
6768,1233,2012-07-04,1,3,0,21,22.6666660308838,16.3333339691162,60,3,2,203,69,1,1,0,1,0,8,16
6769,1233,2012-07-04,2,3,0,24,15.3333330154419,20.6666660308838,60,3,2,203,69,1,1,0,1,0,8,16
6770,1233,2012-07-04,3,3,0,18,18.3333339691162,23.6666660308838,60,3,2,203,69,1,1,0,1,0,8,16
6771,1233,2012-07-04,4,3,0,19.6666660308838,23,17.3333339691162,60,3,2,203,69,1,1,0,1,0,8,16
6772,1234,2010-12-07,0,2,0,14,39.5,1.5,55,3,2,203,77,1,1,0,1,0,11,16933.18454,13588.13628,12.42
6773,1234,2010-12-07,1,2,0,13,38.6666679382324,8.33333301544189,60,3,2,203,77,1,1,0,1,0,11,1
6774,1234,2010-12-07,2,2,0,27.6666660308838,31.3333339691162,1,60,3,2,203,77,1,1,0,1,0,11,16
6775,1234,2010-12-07,3,2,0,18.6666660308838,25.6666660308838,15.6666669845581,60,3,2,203,
6776,1234,2010-12-07,4,2,0,43.3333320617676,16,0.666666686534882,60,3,2,203,77,1,1,0,1,0,11,
6777,1234,2010-12-08,1,3,0,44.6666679382324,11,4.33333349227905,60,3,2,203,77,1,1,0,1,0,11,1
6778,1234,2010-12-08,2,3,0,30.6666660308838,27,2.33333325386047,60,3,2,203,77,1,1,0,1,0,11,1
6779,1234,2010-12-08,3,3,0,24,31.3333339691162,4.66666650772095,60,3,2,203,77,1,1,0,1,0,11,1
6780,1234,2010-12-08,4,3,0,51,9,0,60,3,2,203,77,1,1,0,1,0,11,16933.18454,13588.13628,12.425756
6781,1234,2010-12-09,0,4,0,12,30.25,8,50.25,3,2,203,77,1,1,0,1,0,11,16933.18454,13588.13628,12
6782,1234,2010-12-09,1,4,0,28.5,23.75,7.75,60,3,2,203,77,1,1,0,1,0,11,16933.18454,13588.13628,1
6783,1234,2010-12-09,2,4,0,16.6666660308838,39,4.33333349227905,60,3,2,203,77,1,1,0,1,0,11,1
6784,1234,2010-12-09,3,4,0,33,20.3333339691162,6.66666650772095,60,3,2,203,77,1,1,0,1,0,11,1
6785,1234,2010-12-09,4,4,0,45.3333320617676,14,0.666666686534882,60,3,2,203,77,1,1,0,1,0,11,
6786,1234,2010-12-10,0,5,0,13,32.3333320617676,4.66666650772095,50,3,2,203,77,1,1,0,1,0,11,1
6787,1234,2010-12-10,1,5,0,11.3333330154419,36.3333320617676,12.3333330154419,60,3,2,203,
6788,1234,2010-12-10,2,5,0,47,12,1,60,3,2,203,77,1,1,0,1,0,11,16933.18454,13588.13628,12.42575
6789,1234,2010-12-10,3,5,0,25,29.3333339691162,5.66666650772095,60,3,2,203,77,1,1,0,1,0,11,1
6790,1234,2010-12-10,4,5,0,45.6666679382324,13.6666669845581,0.666666686534882,60,3,2,203
6791,1234,2010-12-11,1,6,1,17.5,34.5,8,60,2,2,203,77,1,1,0,1,0,11,16933.18454,13588.13628,12.42
6792,1234,2010-12-11,2,6,1,18.6666660308838,34.6666679382324,6.66666650772095,60,3,2,203,
6793,1234,2010-12-11,3,6,1,25,33.3333320617676,1.66666662693024,60,3,2,203,77,1,1,0,1,0,11,1
6794,1234,2010-12-11,4,6,1,35.6666679382324,20.3333339691162,4,60,3,2,203,77,1,1,0,1,0,11,16
6795,1234,2010-12-19,0,0,1,2,22,0,24,1,2,203,77,1,1,0,1,0,11,16933.18454,13588.13628,12.425756
6796,1234,2010-12-20,0,1,0,20.6666660308838,29,2,51.6666679382324,3,2,203,77,1,1,0,1,0,11,16
6797,1234,2010-12-20,1,1,0,12.3333330154419,34.3333320617676,13.3333330154419,60,3,2,203,
6798,1234,2010-12-20,2,1,0,42.3333320617676,14,3.66666674613953,60,3,2,203,77,1,1,0,1,0,11,1
6799,1234,2010-12-20,3,1,0,42.3333320617676,14.6666669845581,3,60,3,2,203,77,1,1,0,1,0,11,16

6800,1234,2010-12-20,4,1,0,34.3333320617676,24,1.66666662693024,60,3,2,203,77,1,1,0,1,0,11,1
6801,1234,2010-12-21,0,2,0,5.33333349227905,38,1.66666662693024,45,3,2,203,77,1,1,0,1,0,11,1
6802,1234,2010-12-21,1,2,0,13,43.3333320617676,3.66666674613953,60,3,2,203,77,1,1,0,1,0,11,1
6803,1234,2010-12-21,2,2,0,26.6666660308838,21.6666660308838,11.6666669845581,60,3,2,203,
6804,1234,2010-12-21,3,2,0,24.6666660308838,20.3333339691162,15,60,3,2,203,77,1,1,0,1,0,11,1
6805,1234,2010-12-21,4,2,0,42,17,1,60,3,2,203,77,1,1,0,1,0,11,16933.18454,13588.13628,12.4257
6806,1235,2012-06-15,0,5,0,26,29,5,60,1,2,203,67,1,1,1,0,0,8,16708.00427,13786.18024,13.25324
6807,1235,2012-06-15,1,5,0,30,24.6666660308838,5.33333349227905,60,3,2,203,67,1,1,1,0,0,8,16
6808,1235,2012-06-15,2,5,0,12.3333330154419,44,3.66666674613953,60,3,2,203,67,1,1,1,0,0,8,16
6809,1235,2012-06-15,3,5,0,9.66666698455811,47.3333320617676,3,60,3,2,203,67,1,1,1,0,0,8,167
6810,1235,2012-06-15,4,5,0,41,18.3333339691162,0.666666686534882,60,3,2,203,67,1,1,1,0,0,8,1
6811,1235,2012-06-16,0,6,1,19.3333339691162,29.3333339691162,1,49.6666679382324,3,2,203,6
6812,1235,2012-06-16,1,6,1,28.3333339691162,24,7.66666650772095,60,3,2,203,67,1,1,1,0,0,8,16
6813,1235,2012-06-16,2,6,1,35.6666679382324,21.6666660308838,2.66666674613953,60,3,2,203,
6814,1235,2012-06-16,3,6,1,28,26.3333339691162,5.66666650772095,60,3,2,203,67,1,1,1,0,0,8,16
6815,1235,2012-06-16,4,6,1,28,20.6666660308838,0,48.6666679382324,3,2,203,67,1,1,1,0,0,8,167
6816,1235,2012-06-17,0,7,1,33,12.6666669845581,0.333333343267441,46,3,2,203,67,1,1,1,0,0,8,1
6817,1235,2012-06-17,1,7,1,43,16.3333339691162,0.666666686534882,60,3,2,203,67,1,1,1,0,0,8,1
6818,1235,2012-06-17,2,7,1,38,21.6666660308838,0.333333343267441,60,3,2,203,67,1,1,1,0,0,8,1
6819,1235,2012-06-17,3,7,1,26.3333339691162,28,5.66666650772095,60,3,2,203,67,1,1,1,0,0,8,16
6820,1235,2012-06-17,4,7,1,47.3333320617676,12.6666669845581,0,60,3,2,203,67,1,1,1,0,0,8,167
6821,1235,2012-06-18,0,1,0,22,27.3333339691162,2.33333325386047,51.6666679382324,3,2,203,
6822,1235,2012-06-18,1,1,0,25,24,11,60,3,2,203,67,1,1,1,0,0,8,16708.00427,13786.18024,13.2532
6823,1235,2012-06-18,2,1,0,48,12,0,60,3,2,203,67,1,1,1,0,0,8,16708.00427,13786.18024,13.25324
6824,1235,2012-06-18,3,1,0,49,11,0,60,3,2,203,67,1,1,1,0,0,8,16708.00427,13786.18024,13.25324
6825,1235,2012-06-18,4,1,0,53.3333320617676,6.66666650772095,0,60,3,2,203,67,1,1,1,0,0,8,167
6826,1235,2012-06-19,0,2,0,21.6666660308838,20.3333339691162,2.66666674613953,44.666667
6827,1235,2012-06-19,1,2,0,17.3333339691162,38,4.66666650772095,60,3,2,203,67,1,1,1,0,0,8,16
6828,1235,2012-06-19,2,2,0,42.3333320617676,17.6666660308838,0,60,3,2,203,67,1,1,1,0,0,8,167
6829,1235,2012-06-19,3,2,0,42,18,0,60,3,2,203,67,1,1,1,0,0,8,16708.00427,13786.18024,13.25324
6830,1235,2012-06-19,4,2,0,54.3333320617676,5.66666650772095,0,60,3,2,203,67,1,1,1,0,0,8,167
6831,1235,2012-06-20,0,3,0,27,18.6666660308838,1.66666662693024,47.3333320617676,3,2,203,
6832,1235,2012-06-20,1,3,0,37.6666679382324,18,4.33333349227905,60,3,2,203,67,1,1,1,0,0,8,16
6833,1235,2012-06-20,2,3,0,20.3333339691162,38,1.66666662693024,60,3,2,203,67,1,1,1,0,0,8,16
6834,1235,2012-06-20,3,3,0,13.3333330154419,40.3333320617676,6.33333349227905,60,3,2,203,
6835,1235,2012-06-20,4,3,0,43.6666679382324,15,1.33333337306976,60,3,2,203,67,1,1,1,0,0,8,16
6836,1235,2012-06-21,0,4,0,27.6666660308838,24,1.66666662693024,53.3333320617676,3,2,203,
6837,1235,2012-06-21,1,4,0,38.6666679382324,17.6666660308838,3.66666674613953,60,3,2,203,
6838,1235,2012-06-21,2,4,0,38.3333320617676,20,1.66666662693024,60,3,2,203,67,1,1,1,0,0,8,16
6839,1235,2012-06-21,3,4,0,39.6666679382324,15.3333330154419,5,60,3,2,203,67,1,1,1,0,0,8,167
6840,1235,2012-06-21,4,4,0,54.3333320617676,5.66666650772095,0,60,3,2,203,67,1,1,1,0,0,8,167
6841,1239,2012-06-19,0,2,0,18,39,3,60,1,2,203,69,0,1,0,0,0,8,17006.89949,15203.92973,14.45335
6842,1239,2012-06-19,1,2,0,21.6666660308838,29.6666660308838,8.66666698455811,60,3,2,203,
6843,1239,2012-06-19,2,2,0,21,24.6666660308838,14.3333330154419,60,3,2,203,69,0,1,0,0,0,8,17
6844,1239,2012-06-19,3,2,0,26,29.3333339691162,4.66666650772095,60,3,2,203,69,0,1,0,0,0,8,17
6845,1239,2012-06-19,4,2,0,44,16,0,60,3,2,203,69,0,1,0,0,0,8,17006.89949,15203.92973,14.45335
6846,1239,2012-06-20,0,3,0,19.6666660308838,14.6666669845581,0.333333343267441,34.66666
6847,1239,2012-06-20,1,3,0,12.6666669845581,36,11.3333330154419,60,3,2,203,69,0,1,0,0,0,8,17
6848,1239,2012-06-20,2,3,0,30,23.3333339691162,6.66666650772095,60,3,2,203,69,0,1,0,0,0,8,17
6849,1239,2012-06-20,3,3,0,22.6666660308838,24.6666660308838,12.6666669845581,60,3,2,203,

6850,1239,2012-06-20,4,3,0,44.3333320617676,15.6666669845581,0,60,3,2,203,69,0,1,0,0,0,8,170
6851,1239,2012-06-21,0,4,0,15,12,0.666666686534882,27.6666660308838,3,2,203,69,0,1,0,0,0,8,1
6852,1239,2012-06-21,1,4,0,21.6666660308838,35,3.33333325386047,60,3,2,203,69,0,1,0,0,0,8,17
6853,1239,2012-06-21,2,4,0,25,32.6666679382324,2.33333325386047,60,3,2,203,69,0,1,0,0,0,8,17
6854,1239,2012-06-21,3,4,0,26.3333339691162,21,12.6666669845581,60,3,2,203,69,0,1,0,0,0,8,17
6855,1239,2012-06-21,4,4,0,47,13,0,60,3,2,203,69,0,1,0,0,0,8,17006.89949,15203.92973,14.453358
6856,1239,2012-06-22,0,5,0,6.66666650772095,25,10.3333330154419,42,3,2,203,69,0,1,0,0,0,8,17
6857,1239,2012-06-22,1,5,0,35.3333320617676,23,1.66666662693024,60,3,2,203,69,0,1,0,0,0,8,17
6858,1239,2012-06-22,2,5,0,31.3333339691162,23,5.66666650772095,60,3,2,203,69,0,1,0,0,0,8,17
6859,1239,2012-06-22,3,5,0,40,18.3333339691162,1.66666662693024,60,3,2,203,69,0,1,0,0,0,8,17
6860,1239,2012-06-22,4,5,0,51.6666679382324,8.33333301544189,0,60,3,2,203,69,0,1,0,0,0,8,170
6861,1239,2012-06-23,0,6,1,5.66666650772095,15,29.3333339691162,50,3,2,203,69,0,1,0,0,0,8,17
6862,1239,2012-06-23,1,6,1,12.3333330154419,33.6666679382324,14,60,3,2,203,69,0,1,0,0,0,8,17
6863,1239,2012-06-23,2,6,1,13.3333330154419,27.6666660308838,19,60,3,2,203,69,0,1,0,0,0,8,17
6864,1239,2012-06-23,3,6,1,23.3333339691162,20.6666660308838,16,60,3,2,203,69,0,1,0,0,0,8,17
6865,1239,2012-06-23,4,6,1,47,13,0,60,3,2,203,69,0,1,0,0,0,8,17006.89949,15203.92973,14.453358
6866,1239,2012-06-24,0,7,1,9.66666698455811,25.6666660308838,2.66666674613953,38,3,2,203,
6867,1239,2012-06-24,1,7,1,34.3333320617676,15,10.6666669845581,60,3,2,203,69,0,1,0,0,0,8,17
6868,1239,2012-06-24,2,7,1,27,22,11,60,3,2,203,69,0,1,0,0,0,8,17006.89949,15203.92973,14.45335
6869,1239,2012-06-24,3,7,1,37,18,5,60,3,2,203,69,0,1,0,0,0,8,17006.89949,15203.92973,14.453358
6870,1239,2012-06-24,4,7,1,35.3333320617676,24.3333339691162,0.333333343267441,60,3,2,203
6871,1239,2012-06-25,0,1,0,1.66666662693024,31.6666660308838,2.33333325386047,35.6666679
6872,1239,2012-06-25,1,1,0,18,39,3,60,3,2,203,69,0,1,0,0,0,8,17006.89949,15203.92973,14.453358
6873,1239,2012-06-25,2,1,0,31.6666660308838,14,14.3333330154419,60,3,2,203,69,0,1,0,0,0,8,17
6874,1239,2012-06-25,3,1,0,24,29.6666660308838,6.33333349227905,60,3,2,203,69,0,1,0,0,0,8,17
6875,1239,2012-06-25,4,1,0,49,11,0,60,3,2,203,69,0,1,0,0,0,8,17006.89949,15203.92973,14.453358
6876,1240,2012-06-29,0,5,0,58,2,0,60,1,2,203,70,0,1,1,0,0,12,16715.68645,16565.66933,12.805433
6877,1240,2012-06-29,1,5,0,32,15,13,60,3,2,203,70,0,1,1,0,0,12,16715.68645,16565.66933,12.8054
6878,1240,2012-06-29,2,5,0,18.6666660308838,31.6666660308838,9.66666698455811,60,3,2,203,
6879,1240,2012-06-29,3,5,0,31.6666660308838,21.6666660308838,6.66666650772095,60,3,2,203,
6880,1240,2012-06-29,4,5,0,32.6666679382324,27.3333339691162,0,60,3,2,203,70,0,1,1,0,0,12,16
6881,1240,2012-06-30,0,6,1,0.666666686534882,28.3333339691162,6.33333349227905,35.333332
6882,1240,2012-06-30,1,6,1,16,32,12,60,3,2,203,70,0,1,1,0,0,12,16715.68645,16565.66933,12.8054
6883,1240,2012-06-30,2,6,1,25.6666660308838,29.6666660308838,4.66666650772095,60,3,2,203,
6884,1240,2012-06-30,3,6,1,16,28.6666660308838,15.3333330154419,60,3,2,203,70,0,1,1,0,0,12,1
6885,1240,2012-06-30,4,6,1,12,37.6666679382324,10.3333330154419,60,3,2,203,70,0,1,1,0,0,12,1
6886,1240,2012-07-01,0,7,1,6.33333349227905,15,1.33333337306976,22.6666660308838,3,2,203,
6887,1240,2012-07-01,1,7,1,12.3333330154419,28.6666660308838,19,60,3,2,203,70,0,1,1,0,0,12,1
6888,1240,2012-07-01,2,7,1,21.6666660308838,27.3333339691162,11,60,3,2,203,70,0,1,1,0,0,12,1
6889,1240,2012-07-01,3,7,1,9,41.6666679382324,9.33333301544189,60,3,2,203,70,0,1,1,0,0,12,16
6890,1240,2012-07-01,4,7,1,4.66666650772095,41.3333320617676,14,60,3,2,203,70,0,1,1,0,0,12,1
6891,1240,2012-07-02,0,1,0,3.66666674613953,15.6666669845581,9,28.3333339691162,3,2,203,7
6892,1240,2012-07-02,1,1,0,5,33,22,60,3,2,203,70,0,1,1,0,0,12,16715.68645,16565.66933,12.80543
6893,1240,2012-07-02,2,1,0,4.66666650772095,37,18.3333339691162,60,3,2,203,70,0,1,1,0,0,12,1
6894,1240,2012-07-02,3,1,0,12.3333330154419,39.6666679382324,8,60,3,2,203,70,0,1,1,0,0,12,16
6895,1240,2012-07-02,4,1,0,23.3333339691162,25,11.6666669845581,60,3,2,203,70,0,1,1,0,0,12,1
6896,1240,2012-07-03,0,2,0,8.66666698455811,21.6666660308838,7.66666650772095,38,3,2,203,
6897,1240,2012-07-03,1,2,0,10,39.3333320617676,10.6666669845581,60,3,2,203,70,0,1,1,0,0,12,1
6898,1240,2012-07-03,2,2,0,17.3333339691162,34.6666679382324,8,60,3,2,203,70,0,1,1,0,0,12,16
6899,1240,2012-07-03,3,2,0,12.3333330154419,31.6666660308838,16,60,3,2,203,70,0,1,1,0,0,12,1

6900,1240,2012-07-03,4,2,0,24,33,3,60,3,2,203,70,0,1,1,0,0,12,16715.68645,16565.66933,12.80543
6901,1240,2012-07-04,0,3,0,10.6666669845581,17,6.33333349227905,34,3,2,203,70,0,1,1,0,0,12,1
6902,1240,2012-07-04,1,3,0,6.33333349227905,33.6666679382324,20,60,3,2,203,70,0,1,1,0,0,12,1
6903,1240,2012-07-04,2,3,0,16,34.6666679382324,9.33333301544189,60,3,2,203,70,0,1,1,0,0,12,1
6904,1240,2012-07-04,3,3,0,34,24,2,60,3,2,203,70,0,1,1,0,0,12,16715.68645,16565.66933,12.80543
6905,1240,2012-07-04,4,3,0,33.3333320617676,25,1.66666662693024,60,3,2,203,70,0,1,1,0,0,12,1
6906,1240,2012-07-05,0,4,0,13,22,5.33333349227905,40.3333320617676,3,2,203,70,0,1,1,0,0,12,1
6907,1240,2012-07-05,1,4,0,6.66666650772095,40.3333320617676,13,60,3,2,203,70,0,1,1,0,0,12,1
6908,1240,2012-07-05,2,4,0,23,30,7,60,3,2,203,70,0,1,1,0,0,12,16715.68645,16565.66933,12.80543
6909,1240,2012-07-05,3,4,0,19,33,8,60,3,2,203,70,0,1,1,0,0,12,16715.68645,16565.66933,12.80543
6910,1240,2012-07-05,4,4,0,11.3333330154419,45,3.66666674613953,60,3,2,203,70,0,1,1,0,0,12,1
6911,1241,2010-12-09,0,4,0,30,15,15,60,1,2,203,74,1,1,0,1,0,10,16714.78116,12797.5841,17.32279
6912,1241,2010-12-09,1,4,0,18.3333339691162,36,5.66666650772095,60,3,2,203,74,1,1,0,1,0,10,1
6913,1241,2010-12-09,2,4,0,35,24,1,60,3,2,203,74,1,1,0,1,0,10,16714.78116,12797.5841,17.322793
6914,1241,2010-12-09,3,4,0,43.6666679382324,15.3333330154419,1,60,3,2,203,74,1,1,0,1,0,10,16
6915,1241,2010-12-09,4,4,0,39.5,20.25,0.25,60,3,2,203,74,1,1,0,1,0,10,16714.78116,12797.5841,17
6916,1241,2010-12-10,0,5,0,6,18,7,31,2,2,203,74,1,1,0,1,0,10,16714.78116,12797.5841,17.3227939
6917,1241,2010-12-10,1,5,0,23.6666660308838,34,2.33333325386047,60,3,2,203,74,1,1,0,1,0,10,1
6918,1241,2010-12-10,2,5,0,32.6666679382324,27.3333339691162,0,60,3,2,203,74,1,1,0,1,0,10,16
6919,1241,2010-12-10,3,5,0,21.3333339691162,38,0.666666686534882,60,3,2,203,74,1,1,0,1,0,10,
6920,1241,2010-12-10,4,5,0,30.5,29.25,0.25,60,3,2,203,74,1,1,0,1,0,10,16714.78116,12797.5841,17
6921,1241,2010-12-11,0,6,1,17.5,20.75,1.25,39.5,3,2,203,74,1,1,0,1,0,10,16714.78116,12797.5841,
6922,1241,2010-12-11,1,6,1,14.3333330154419,39.8333320617676,5.83333349227905,60,3,2,203,
6923,1241,2010-12-11,2,6,1,20.25,36,3.75,60,3,2,203,74,1,1,0,1,0,10,16714.78116,12797.5841,17.3
6924,1241,2010-12-11,3,6,1,12,39,9,60,3,2,203,74,1,1,0,1,0,10,16714.78116,12797.5841,17.322793
6925,1241,2010-12-11,4,6,1,27.25,32.25,0.5,60,3,2,203,74,1,1,0,1,0,10,16714.78116,12797.5841,17
6926,1241,2010-12-12,0,0,1,4,24,17,45,1,2,203,74,1,1,0,1,0,10,16714.78116,12797.5841,17.322793
6927,1241,2010-12-12,1,0,1,27.1666660308838,26.8333339691162,6,60,3,2,203,74,1,1,0,1,0,10,16
6928,1241,2010-12-12,2,0,1,31.375,28.25,0.375,60,3,2,203,74,1,1,0,1,0,10,16714.78116,12797.584
6929,1241,2010-12-12,3,0,1,29.3333339691162,25.1666660308838,5.5,60,3,2,203,74,1,1,0,1,0,10,1
6930,1241,2010-12-12,4,0,1,34.8333320617676,23,0,57.8333320617676,3,2,203,74,1,1,0,1,0,10,16
6931,1241,2010-12-13,0,1,0,28,11,0,39,1,2,203,74,1,1,0,1,0,10,16714.78116,12797.5841,17.322793
6932,1241,2010-12-13,1,1,0,24.3333339691162,31,4.66666650772095,60,3,2,203,74,1,1,0,1,0,10,1
6933,1241,2010-12-13,2,1,0,22,30.6666660308838,7.33333349227905,60,3,2,203,74,1,1,0,1,0,10,1
6934,1241,2010-12-13,3,1,0,26.3333339691162,28.3333339691162,5.33333349227905,60,3,2,203,
6935,1241,2010-12-13,4,1,0,37,23,0,60,3,2,203,74,1,1,0,1,0,10,16714.78116,12797.5841,17.322793
6936,1242,2010-12-12,0,0,1,22,37,1,60,1,2,203,67,1,1,0,1,0,8,16727.90661,14989.50623,14.182294
6937,1242,2010-12-12,1,0,1,33.6666679382324,25.3333339691162,1,60,3,2,203,67,1,1,0,1,0,8,167
6938,1242,2010-12-13,0,1,0,10,4,1,15,1,2,203,67,1,1,0,1,0,8,16727.90661,14989.50623,14.1822946
6939,1242,2010-12-13,1,1,0,32,28,0,60,3,2,203,67,1,1,0,1,0,8,16727.90661,14989.50623,14.182294
6940,1242,2010-12-13,2,1,0,38.7999992370605,20.6000003814697,0.600000023841858,60,3,2,203
6941,1242,2010-12-13,3,1,0,43,16.6666660308838,0.333333343267441,60,3,2,203,67,1,1,0,1,0,8,1
6942,1242,2010-12-13,4,1,0,22,37.3333320617676,0.666666686534882,60,3,2,203,67,1,1,0,1,0,8,1
6943,1242,2010-12-14,1,2,0,14.6666669845581,31,0.666666686534882,46.3333320617676,3,2,203
6944,1242,2010-12-14,2,2,0,36.25,23.5,0.25,60,3,2,203,67,1,1,0,1,0,8,16727.90661,14989.50623,14
6945,1242,2010-12-14,3,2,0,26.8333339691162,32.5,0.666666686534882,60,3,2,203,67,1,1,0,1,0,8
6946,1242,2010-12-14,4,2,0,39.6666679382324,20,0.333333343267441,60,3,2,203,67,1,1,0,1,0,8,1
6947,1242,2010-12-15,0,3,0,33,6,1,40,1,2,203,67,1,1,0,1,0,8,16727.90661,14989.50623,14.1822946
6948,1242,2010-12-15,1,3,0,20.3333339691162,39,0.666666686534882,60,3,2,203,67,1,1,0,1,0,8,1
6949,1242,2010-12-15,2,3,0,43,17,0,60,3,2,203,67,1,1,0,1,0,8,16727.90661,14989.50623,14.182294

6950,1242,2010-12-15,3,3,0,15.6666669845581,44.3333320617676,0,60,3,2,203,67,1,1,0,1,0,8,167
6951,1242,2010-12-15,4,3,0,36.6666679382324,22.8333339691162,0.5,60,3,2,203,67,1,1,0,1,0,8,16
6952,1242,2011-07-09,0,6,1,17,15,1,33,2,2,203,67,1,1,0,1,0,8,16727.90661,14989.50623,14.18229
6953,1242,2011-07-09,1,6,1,23,37,0,60,3,2,203,67,1,1,0,1,0,8,16727.90661,14989.50623,14.18229
6954,1242,2011-07-09,2,6,1,20.6666660308838,37.6666679382324,1.6666662693024,60,3,2,203,
6955,1242,2011-07-09,3,6,1,37,22.3333339691162,0.666666686534882,60,3,2,203,67,1,1,0,1,0,8,1
6956,1242,2011-07-09,4,6,1,10.3333330154419,48.3333320617676,1.33333337306976,60,3,2,203,
6957,1242,2011-07-10,0,0,1,20,24,1,45,1,2,203,67,1,1,0,1,0,8,16727.90661,14989.50623,14.18229
6958,1242,2011-07-10,1,0,1,24,36,0,60,3,2,203,67,1,1,0,1,0,8,16727.90661,14989.50623,14.18229
6959,1242,2011-07-10,2,0,1,33,27,0,60,3,2,203,67,1,1,0,1,0,8,16727.90661,14989.50623,14.18229
6960,1242,2011-07-10,3,0,1,35.6666679382324,22.6666660308838,1.6666662693024,60,3,2,203,
6961,1242,2011-07-10,4,0,1,32.6666679382324,26,1.33333337306976,60,3,2,203,67,1,1,0,1,0,8,16
6962,1242,2011-07-11,2,1,0,33,22.5,4.5,60,2,2,203,67,1,1,0,1,0,8,16727.90661,14989.50623,14.18
6963,1242,2011-07-11,3,1,0,40.6666679382324,19.3333339691162,0,60,3,2,203,67,1,1,0,1,0,8,167
6964,1242,2011-07-11,4,1,0,37.3333320617676,21,1.6666662693024,60,3,2,203,67,1,1,0,1,0,8,16
6965,1244,2012-06-30,1,6,1,14.3333330154419,0.666666686534882,0.666666686534882,15.6666
6966,1244,2012-06-30,2,6,1,44,13.6666669845581,2.33333325386047,60,3,2,203,71,0,1,0,1,0,12,1
6967,1244,2012-06-30,3,6,1,41.6666679382324,18,0.333333343267441,60,3,2,203,71,0,1,0,1,0,12,
6968,1244,2012-06-30,4,6,1,22.6666660308838,35,2.33333325386047,60,3,2,203,71,0,1,0,1,0,12,1
6969,1244,2012-07-01,1,7,1,7.66666650772095,9.33333301544189,4,21,3,2,203,71,0,1,0,1,0,12,16
6970,1244,2012-07-01,2,7,1,43.3333320617676,15,1.6666662693024,60,3,2,203,71,0,1,0,1,0,12,1
6971,1244,2012-07-01,3,7,1,50,9.66666698455811,0.333333343267441,60,3,2,203,71,0,1,0,1,0,12,
6972,1244,2012-07-01,4,7,1,46,13.3333330154419,0.666666686534882,60,3,2,203,71,0,1,0,1,0,12,
6973,1244,2012-07-02,0,1,0,10.3333330154419,16,6,32.3333320617676,3,2,203,71,0,1,0,1,0,12,16
6974,1244,2012-07-02,1,1,0,42,12.3333330154419,5.66666650772095,60,3,2,203,71,0,1,0,1,0,12,1
6975,1244,2012-07-02,2,1,0,34,24.3333339691162,1.6666662693024,60,3,2,203,71,0,1,0,1,0,12,1
6976,1244,2012-07-02,3,1,0,44.3333320617676,15.3333330154419,0.333333343267441,60,3,2,20
6977,1244,2012-07-02,4,1,0,41.3333320617676,17.6666660308838,1,60,3,2,203,71,0,1,0,1,0,12,16
6978,1244,2012-07-04,1,3,0,2,17.3333339691162,1.6666662693024,21,3,2,203,71,0,1,0,1,0,12,16
6979,1244,2012-07-04,2,3,0,3.66666674613953,51.6666679382324,4.66666650772095,60,3,2,203,
6980,1244,2012-07-04,3,3,0,20.6666660308838,37,2.33333325386047,60,3,2,203,71,0,1,0,1,0,12,1
6981,1244,2012-07-04,4,3,0,20.3333339691162,38.3333320617676,1.33333337306976,60,3,2,203,
6982,1244,2012-07-05,1,4,0,20,20,3,43,3,2,203,71,0,1,0,1,0,12,16932.5167,13307.38451,13.12429
6983,1244,2012-07-05,2,4,0,23,31.3333339691162,5.66666650772095,60,3,2,203,71,0,1,0,1,0,12,1
6984,1244,2012-07-05,3,4,0,46,14,0,60,3,2,203,71,0,1,0,1,0,12,16932.5167,13307.38451,13.12429
6985,1244,2012-07-05,4,4,0,43,16.6666660308838,0.333333343267441,60,3,2,203,71,0,1,0,1,0,12,
6986,1244,2012-07-06,1,5,0,13.6666669845581,25,0.666666686534882,39.3333320617676,3,2,20
6987,1244,2012-07-06,2,5,0,25.6666660308838,31.6666660308838,2.66666674613953,60,3,2,203,
6988,1244,2012-07-06,3,5,0,16.3333339691162,42,1.6666662693024,60,3,2,203,71,0,1,0,1,0,12,1
6989,1244,2012-07-06,4,5,0,15,44.6666679382324,0.333333343267441,60,3,2,203,71,0,1,0,1,0,12,
6990,1244,2012-07-07,1,6,1,8,35,0.666666686534882,43.6666679382324,3,2,203,71,0,1,0,1,0,12,1
6991,1244,2012-07-07,2,6,1,25.6666660308838,32,2.33333325386047,60,3,2,203,71,0,1,0,1,0,12,1
6992,1244,2012-07-07,3,6,1,27,31.6666660308838,1.33333337306976,60,3,2,203,71,0,1,0,1,0,12,1
6993,1244,2012-07-07,4,6,1,28.3333339691162,29.6666660308838,2,60,3,2,203,71,0,1,0,1,0,12,16
6994,1244,2012-07-08,2,7,1,44.3333320617676,12.6666669845581,0.333333343267441,57.33333
6995,1244,2012-07-08,3,7,1,52.6666679382324,7,0.333333343267441,60,3,2,203,71,0,1,0,1,0,12,1
6996,1244,2012-07-08,4,7,1,46.3333320617676,13,0.666666686534882,60,3,2,203,71,0,1,0,1,0,12,
6997,1244,2012-07-09,1,1,0,21,22.3333339691162,0.333333343267441,43.6666679382324,3,2,20
6998,1244,2012-07-09,2,1,0,28,32,0,60,3,2,203,71,0,1,0,1,0,12,16932.5167,13307.38451,13.12429
6999,1244,2012-07-09,3,1,0,23.6666660308838,35.6666679382324,0.666666686534882,60,3,2,20

7000,1244,2012-07-09,4,1,0,26,34,0,60,3,2,203,71,0,1,0,1,0,12,16932.5167,13307.38451,13.124298
7001,1244,2012-07-10,1,2,0,18,18.6666660308838,1.33333337306976,38,3,2,203,71,0,1,0,1,0,12,1
7002,1244,2012-07-10,2,2,0,21.6666660308838,29.6666660308838,8.66666698455811,60,3,2,203,
7003,1244,2012-07-10,3,2,0,28.3333339691162,31.6666660308838,0,60,3,2,203,71,0,1,0,1,0,12,16
7004,1244,2012-07-10,4,2,0,44.3333320617676,15.3333330154419,0.333333343267441,60,3,2,203
7005,1244,2012-07-11,1,3,0,15.3333330154419,22.6666660308838,1,39,3,2,203,71,0,1,0,1,0,12,16
7006,1244,2012-07-11,2,3,0,40,20,0,60,3,2,203,71,0,1,0,1,0,12,16932.5167,13307.38451,13.124298
7007,1244,2012-07-11,3,3,0,37.6666679382324,22,0.333333343267441,60,3,2,203,71,0,1,0,1,0,12,
7008,1244,2012-07-11,4,3,0,27.3333339691162,31.6666660308838,1,60,3,2,203,71,0,1,0,1,0,12,16
7009,1245,2012-06-21,0,4,0,56,4,0,60,1,2,203,65,0,1,1,0,0,11,16389.04523,13864.28693,13.608500
7010,1245,2012-06-21,1,4,0,54,6,0,60,3,2,203,65,0,1,1,0,0,11,16389.04523,13864.28693,13.608500
7011,1245,2012-06-21,2,4,0,46,8.66666698455811,5.33333349227905,60,3,2,203,65,0,1,1,0,0,11,1
7012,1245,2012-06-21,3,4,0,55.3333320617676,4.66666650772095,0,60,3,2,203,65,0,1,1,0,0,11,16
7013,1245,2012-06-21,4,4,0,52.3333320617676,7.66666650772095,0,60,3,2,203,65,0,1,1,0,0,11,16
7014,1245,2012-06-22,0,5,0,0,0.333333343267441,0,0.333333343267441,3,2,203,65,0,1,1,0,0,11,1
7015,1245,2012-06-22,1,5,0,30.6666660308838,11.6666669845581,17.6666660308838,60,3,2,203,
7016,1245,2012-06-22,2,5,0,57.3333320617676,2.33333325386047,0.333333343267441,60,3,2,203
7017,1245,2012-06-22,3,5,0,56,4,0,60,3,2,203,65,0,1,1,0,0,11,16389.04523,13864.28693,13.608500
7018,1245,2012-06-22,4,5,0,53,6.66666650772095,0.333333343267441,60,3,2,203,65,0,1,1,0,0,11,
7019,1245,2012-06-23,0,6,1,0,0.333333343267441,0,0.333333343267441,3,2,203,65,0,1,1,0,0,11,1
7020,1245,2012-06-23,1,6,1,55.6666679382324,4.33333349227905,0,60,3,2,203,65,0,1,1,0,0,11,16
7021,1245,2012-06-23,2,6,1,52,7.66666650772095,0.333333343267441,60,3,2,203,65,0,1,1,0,0,11,
7022,1245,2012-06-23,3,6,1,57,3,0,60,3,2,203,65,0,1,1,0,0,11,16389.04523,13864.28693,13.608500
7023,1245,2012-06-23,4,6,1,57,3,0,60,3,2,203,65,0,1,1,0,0,11,16389.04523,13864.28693,13.608500
7024,1245,2012-06-24,1,7,1,46.3333320617676,4.66666650772095,0.333333343267441,51.333332
7025,1245,2012-06-24,2,7,1,32.6666679382324,4,0,36.6666679382324,3,2,203,65,0,1,1,0,0,11,163
7026,1245,2012-06-24,3,7,1,43,3,0,46,3,2,203,65,0,1,1,0,0,11,16389.04523,13864.28693,13.608500
7027,1245,2012-06-24,4,7,1,54.3333320617676,5.66666650772095,0,60,3,2,203,65,0,1,1,0,0,11,16
7028,1245,2012-06-25,1,1,0,47.6666679382324,4.66666650772095,0.666666686534882,53,3,2,203
7029,1245,2012-06-25,2,1,0,25,6.66666650772095,0,31.6666660308838,3,2,203,65,0,1,1,0,0,11,16
7030,1245,2012-06-25,3,1,0,53.6666679382324,2.33333325386047,0,56,3,2,203,65,0,1,1,0,0,11,16
7031,1245,2012-06-25,4,1,0,56,4,0,60,3,2,203,65,0,1,1,0,0,11,16389.04523,13864.28693,13.608500
7032,1245,2012-06-26,0,2,0,14.6666669845581,1,0.333333343267441,16,3,2,203,65,0,1,1,0,0,11,1
7033,1245,2012-06-26,1,2,0,56.6666679382324,3.33333325386047,0,60,3,2,203,65,0,1,1,0,0,11,16
7034,1245,2012-06-26,2,2,0,52.6666679382324,7,0.333333343267441,60,3,2,203,65,0,1,1,0,0,11,1
7035,1245,2012-06-26,3,2,0,24,3,0,27,3,2,203,65,0,1,1,0,0,11,16389.04523,13864.28693,13.608500
7036,1245,2012-06-26,4,2,0,55,3.66666674613953,1,59.6666679382324,3,2,203,65,0,1,1,0,0,11,16
7037,1245,2012-06-27,0,3,0,17,1.66666662693024,0,18.6666660308838,3,2,203,65,0,1,1,0,0,11,16
7038,1245,2012-06-27,1,3,0,56.3333320617676,3.66666674613953,0,60,3,2,203,65,0,1,1,0,0,11,16
7039,1245,2012-06-27,2,3,0,54.3333320617676,5.66666650772095,0,60,3,2,203,65,0,1,1,0,0,11,16
7040,1245,2012-06-27,3,3,0,57.6666679382324,2.33333325386047,0,60,3,2,203,65,0,1,1,0,0,11,16
7041,1245,2012-06-27,4,3,0,52.3333320617676,7.66666650772095,0,60,3,2,203,65,0,1,1,0,0,11,16
7042,1258,2012-06-12,0,2,0,50,7,3,60,1,2,203,84,0,1,0,1,0,9,16714.78116,12797.5841,17.32279391
7043,1258,2012-06-12,1,2,0,19.6666660308838,38.6666679382324,1.66666662693024,60,3,2,203,
7044,1258,2012-06-12,2,2,0,31.6666660308838,27.6666660308838,0.666666686534882,60,3,2,203
7045,1258,2012-06-12,3,2,0,31.6666660308838,27.6666660308838,0.666666686534882,60,3,2,203
7046,1258,2012-06-12,4,2,0,48.3333320617676,11.6666669845581,0,60,3,2,203,84,0,1,0,1,0,9,167
7047,1258,2012-06-13,0,3,0,0,1,0,1,3,2,203,84,0,1,0,1,0,9,16714.78116,12797.5841,17.32279391,1
7048,1258,2012-06-13,1,3,0,49.3333320617676,10,0.666666686534882,60,3,2,203,84,0,1,0,1,0,9,1
7049,1258,2012-06-13,2,3,0,42.3333320617676,17,0.666666686534882,60,3,2,203,84,0,1,0,1,0,9,1

7050,1258,2012-06-13,3,3,0,29,30.6666660308838,0.333333343267441,60,3,2,203,84,0,1,0,1,0,9,1
7051,1258,2012-06-13,4,3,0,47.3333320617676,12.6666669845581,0,60,3,2,203,84,0,1,0,1,0,9,167
7052,1258,2012-06-14,1,4,0,21.6666660308838,28,0.666666686534882,50.3333320617676,3,2,203
7053,1258,2012-06-14,2,4,0,33.6666679382324,21.6666660308838,4.66666650772095,60,3,2,203,
7054,1258,2012-06-14,3,4,0,13.3333330154419,13,33.6666679382324,60,3,2,203,84,0,1,0,1,0,9,16
7055,1258,2012-06-14,4,4,0,48,12,0,60,3,2,203,84,0,1,0,1,0,9,16714.78116,12797.5841,17.3227939
7056,1258,2012-06-15,0,5,0,0,4.66666650772095,5.33333349227905,10,3,2,203,84,0,1,0,1,0,9,167
7057,1258,2012-06-15,1,5,0,28.6666660308838,25,6.33333349227905,60,3,2,203,84,0,1,0,1,0,9,16
7058,1258,2012-06-15,2,5,0,36.6666679382324,23,0.333333343267441,60,3,2,203,84,0,1,0,1,0,9,1
7059,1258,2012-06-15,3,5,0,49.3333320617676,10,0.666666686534882,60,3,2,203,84,0,1,0,1,0,9,1
7060,1258,2012-06-15,4,5,0,50.6666679382324,9.33333301544189,0,60,3,2,203,84,0,1,0,1,0,9,167
7061,1258,2012-06-16,0,6,1,7,3.33333325386047,0,10.3333330154419,3,2,203,84,0,1,0,1,0,9,1671
7062,1258,2012-06-16,1,6,1,28.3333339691162,17.3333339691162,14.3333330154419,60,3,2,203,
7063,1258,2012-06-16,2,6,1,37,23,0,60,3,2,203,84,0,1,0,1,0,9,16714.78116,12797.5841,17.3227939
7064,1258,2012-06-16,3,6,1,31.6666660308838,27.6666660308838,0.666666686534882,60,3,2,203
7065,1258,2012-06-16,4,6,1,30,30,0,60,3,2,203,84,0,1,0,1,0,9,16714.78116,12797.5841,17.3227939
7066,1258,2012-06-17,0,7,1,2.66666674613953,17.3333339691162,0.333333343267441,20.333333
7067,1258,2012-06-17,1,7,1,22,33.6666679382324,4.33333349227905,60,3,2,203,84,0,1,0,1,0,9,16
7068,1258,2012-06-17,2,7,1,44,10.6666669845581,5.33333349227905,60,3,2,203,84,0,1,0,1,0,9,16
7069,1258,2012-06-17,3,7,1,18.6666660308838,34.6666679382324,6.66666650772095,60,3,2,203,
7070,1258,2012-06-17,4,7,1,36.3333320617676,23.3333339691162,0.333333343267441,60,3,2,203
7071,1258,2012-06-18,0,1,0,27.3333339691162,12.3333330154419,0.333333343267441,40,3,2,203
7072,1258,2012-06-18,1,1,0,40,19.6666660308838,0.333333343267441,60,3,2,203,84,0,1,0,1,0,9,1
7073,1258,2012-06-18,2,1,0,39.3333320617676,19.6666660308838,1,60,3,2,203,84,0,1,0,1,0,9,167
7074,1258,2012-06-18,3,1,0,35,24,1,60,3,2,203,84,0,1,0,1,0,9,16714.78116,12797.5841,17.3227939
7075,1258,2012-06-18,4,1,0,34.6666679382324,24.6666660308838,0.666666686534882,60,3,2,203
7076,1260,2012-06-14,0,4,0,8,38,14,60,1,2,203,67,0,1,0,1,0,9,16575.50861,12563.64133,15.722118
7077,1260,2012-06-14,1,4,0,15.6666669845581,30.3333339691162,14,60,3,2,203,67,0,1,0,1,0,9,16
7078,1260,2012-06-14,2,4,0,22,22.3333339691162,15.6666669845581,60,3,2,203,67,0,1,0,1,0,9,16
7079,1260,2012-06-14,3,4,0,36,19,5,60,3,2,203,67,0,1,0,1,0,9,16575.50861,12563.64133,15.722118
7080,1260,2012-06-14,4,4,0,43,15.3333330154419,1.66666662693024,60,3,2,203,67,0,1,0,1,0,9,16
7081,1260,2012-06-15,0,5,0,3.33333325386047,6.33333349227905,0,9.66666698455811,3,2,203,6
7082,1260,2012-06-15,1,5,0,19.6666660308838,36.3333320617676,4,60,3,2,203,67,0,1,0,1,0,9,165
7083,1260,2012-06-15,2,5,0,39.6666679382324,17.6666660308838,2.66666674613953,60,3,2,203,
7084,1260,2012-06-15,3,5,0,47.6666679382324,10,2.33333325386047,60,3,2,203,67,0,1,0,1,0,9,16
7085,1260,2012-06-15,4,5,0,45,14,1,60,3,2,203,67,0,1,0,1,0,9,16575.50861,12563.64133,15.722118
7086,1260,2012-06-16,0,6,1,12.6666669845581,12.3333330154419,0.333333343267441,25.333333
7087,1260,2012-06-16,1,6,1,13.3333330154419,22.3333339691162,24.3333339691162,60,3,2,203,
7088,1260,2012-06-16,2,6,1,23.6666660308838,24,12.3333330154419,60,3,2,203,67,0,1,0,1,0,9,16
7089,1260,2012-06-16,3,6,1,29.3333339691162,22.3333339691162,8.33333301544189,60,3,2,203,
7090,1260,2012-06-16,4,6,1,41.6666679382324,15.6666669845581,2.66666674613953,60,3,2,203,
7091,1260,2012-06-17,0,7,1,5.33333349227905,8,0,13.3333330154419,3,2,203,67,0,1,0,1,0,9,1657
7092,1260,2012-06-17,1,7,1,33,25.3333339691162,1.66666662693024,60,3,2,203,67,0,1,0,1,0,9,16
7093,1260,2012-06-17,2,7,1,37,20.6666660308838,2.33333325386047,60,3,2,203,67,0,1,0,1,0,9,16
7094,1260,2012-06-17,3,7,1,43.3333320617676,9.33333301544189,7.33333349227905,60,3,2,203,
7095,1260,2012-06-17,4,7,1,51.6666679382324,8.33333301544189,0,60,3,2,203,67,0,1,0,1,0,9,165
7096,1260,2012-06-18,0,1,0,9,16,3.33333325386047,28.3333339691162,3,2,203,67,0,1,0,1,0,9,165
7097,1260,2012-06-18,1,1,0,29.3333339691162,14,16.6666660308838,60,3,2,203,67,0,1,0,1,0,9,16
7098,1260,2012-06-18,2,1,0,30.6666660308838,26.6666660308838,2.66666674613953,60,3,2,203,
7099,1260,2012-06-18,3,1,0,36,15,9,60,3,2,203,67,0,1,0,1,0,9,16575.50861,12563.64133,15.722118

7100,1260,2012-06-18,4,1,0,35.3333320617676,24,0.666666686534882,60,3,2,203,67,0,1,0,1,0,9,1
7101,1260,2012-06-19,0,2,0,7.66666650772095,17,5,29.6666660308838,3,2,203,67,0,1,0,1,0,9,165
7102,1260,2012-06-19,1,2,0,14.6666669845581,34.3333320617676,11,60,3,2,203,67,0,1,0,1,0,9,16
7103,1260,2012-06-19,2,2,0,22.6666660308838,33,4.33333349227905,60,3,2,203,67,0,1,0,1,0,9,16
7104,1260,2012-06-19,3,2,0,41,17,2,60,3,2,203,67,0,1,0,1,0,9,16575.50861,12563.64133,15.722118
7105,1260,2012-06-19,4,2,0,39,13,8,60,3,2,203,67,0,1,0,1,0,9,16575.50861,12563.64133,15.722118
7106,1260,2012-06-20,0,3,0,6.33333349227905,5.66666650772095,0,12,3,2,203,67,0,1,0,1,0,9,165
7107,1260,2012-06-20,1,3,0,21.3333339691162,25.3333339691162,13.3333330154419,60,3,2,203,
7108,1260,2012-06-20,2,3,0,25.6666660308838,31.3333339691162,3,60,3,2,203,67,0,1,0,1,0,9,165
7109,1260,2012-06-20,3,3,0,41,13,6,60,3,2,203,67,0,1,0,1,0,9,16575.50861,12563.64133,15.722118
7110,1260,2012-06-20,4,3,0,25,24,11,60,3,2,203,67,0,1,0,1,0,9,16575.50861,12563.64133,15.72211
7111,1261,2012-06-20,0,3,0,14,42,4,60,1,2,203,73,1,1,0,1,0,11,16707.62546,13160.44526,16.4818
7112,1261,2012-06-20,1,3,0,14.3333330154419,41.3333320617676,4.33333349227905,60,3,2,203,
7113,1261,2012-06-20,2,3,0,26.3333339691162,22.3333339691162,11.3333330154419,60,3,2,203,
7114,1261,2012-06-20,3,3,0,21,27.6666660308838,11.3333330154419,60,3,2,203,73,1,1,0,1,0,11,1
7115,1261,2012-06-20,4,3,0,37.6666679382324,19,3.33333325386047,60,3,2,203,73,1,1,0,1,0,11,1
7116,1261,2012-06-21,0,4,0,5,30,9,44,3,2,203,73,1,1,0,1,0,11,16707.62546,13160.44526,16.481801
7117,1261,2012-06-21,1,4,0,22.3333339691162,32.3333320617676,5.33333349227905,60,3,2,203,
7118,1261,2012-06-21,2,4,0,15,38,7,60,3,2,203,73,1,1,0,1,0,11,16707.62546,13160.44526,16.4818
7119,1261,2012-06-21,3,4,0,12,42,6,60,3,2,203,73,1,1,0,1,0,11,16707.62546,13160.44526,16.4818
7120,1261,2012-06-21,4,4,0,28.3333339691162,29.3333339691162,2.33333325386047,60,3,2,203,
7121,1261,2012-06-22,0,5,0,1.66666662693024,16,10.6666669845581,28.3333339691162,3,2,203,
7122,1261,2012-06-22,1,5,0,3.33333325386047,46.3333320617676,10.3333330154419,60,3,2,203,
7123,1261,2012-06-22,2,5,0,31.6666660308838,24,4.33333349227905,60,3,2,203,73,1,1,0,1,0,11,1
7124,1261,2012-06-22,3,5,0,31.6666660308838,22.6666660308838,5.66666650772095,60,3,2,203,
7125,1261,2012-06-22,4,5,0,34.3333320617676,22.3333339691162,3.33333325386047,60,3,2,203,
7126,1261,2012-06-23,0,6,1,2.66666674613953,14.6666669845581,1,18.3333339691162,3,2,203,7
7127,1261,2012-06-23,1,6,1,8,36,16,60,3,2,203,73,1,1,0,1,0,11,16707.62546,13160.44526,16.4818
7128,1261,2012-06-23,2,6,1,27,28.3333339691162,4.66666650772095,60,3,2,203,73,1,1,0,1,0,11,1
7129,1261,2012-06-23,3,6,1,21.3333339691162,25.6666660308838,13,60,3,2,203,73,1,1,0,1,0,11,1
7130,1261,2012-06-23,4,6,1,40.3333320617676,14.6666669845581,5,60,3,2,203,73,1,1,0,1,0,11,16
7131,1261,2012-06-24,0,7,1,4,12.3333330154419,1,17.3333339691162,3,2,203,73,1,1,0,1,0,11,167
7132,1261,2012-06-24,1,7,1,22,33,5,60,3,2,203,73,1,1,0,1,0,11,16707.62546,13160.44526,16.4818
7133,1261,2012-06-24,2,7,1,27.3333339691162,28.3333339691162,4.33333349227905,60,3,2,203,
7134,1261,2012-06-24,3,7,1,35.6666679382324,19,5.33333349227905,60,3,2,203,73,1,1,0,1,0,11,1
7135,1261,2012-06-24,4,7,1,33.6666679382324,21,5.33333349227905,60,3,2,203,73,1,1,0,1,0,11,1
7136,1261,2012-06-25,0,1,0,7.33333349227905,22.3333339691162,10.6666669845581,40.333332
7137,1261,2012-06-25,1,1,0,23.6666660308838,33.3333320617676,3,60,3,2,203,73,1,1,0,1,0,11,16
7138,1261,2012-06-25,2,1,0,9.66666698455811,45.3333320617676,5,60,3,2,203,73,1,1,0,1,0,11,16
7139,1261,2012-06-25,3,1,0,20,33.6666679382324,6.33333349227905,60,3,2,203,73,1,1,0,1,0,11,1
7140,1261,2012-06-25,4,1,0,38,20.3333339691162,1.66666662693024,60,3,2,203,73,1,1,0,1,0,11,1
7141,1261,2012-06-26,0,2,0,15.6666669845581,20.3333339691162,6,42,3,2,203,73,1,1,0,1,0,11,16
7142,1261,2012-06-26,1,2,0,24,31,5,60,3,2,203,73,1,1,0,1,0,11,16707.62546,13160.44526,16.4818
7143,1261,2012-06-26,2,2,0,33,20,7,60,3,2,203,73,1,1,0,1,0,11,16707.62546,13160.44526,16.4818
7144,1261,2012-06-26,3,2,0,30.3333339691162,25.3333339691162,4.33333349227905,60,3,2,203,
7145,1261,2012-06-26,4,2,0,39.3333320617676,18.3333339691162,2.33333325386047,60,3,2,203,
7146,1265,2012-06-12,0,2,0,28,23,9,60,1,2,203,79,0,1,0,1,0,9,16727.90661,14989.50623,14.18229
7147,1265,2012-06-12,1,2,0,47.6666679382324,10,2.33333325386047,60,3,2,203,79,0,1,0,1,0,9,16
7148,1265,2012-06-12,2,2,0,34.6666679382324,20.6666660308838,4.66666650772095,60,3,2,203,
7149,1265,2012-06-12,3,2,0,29.6666660308838,21.6666660308838,8.66666698455811,60,3,2,203,

7150,1265,2012-06-12,4,2,0,28,25,7,60,3,2,203,79,0,1,0,1,0,9,16727.90661,14989.50623,14.182294
7151,1265,2012-06-13,0,3,0,11.6666669845581,23.6666660308838,5.66666650772095,41,3,2,203,
7152,1265,2012-06-13,1,3,0,29.3333339691162,25.3333339691162,5.33333349227905,60,3,2,203,
7153,1265,2012-06-13,2,3,0,33,21.3333339691162,5.66666650772095,60,3,2,203,79,0,1,0,1,0,9,16
7154,1265,2012-06-13,3,3,0,36,21.6666660308838,2.33333325386047,60,3,2,203,79,0,1,0,1,0,9,16
7155,1265,2012-06-13,4,3,0,32,21.6666660308838,6.33333349227905,60,3,2,203,79,0,1,0,1,0,9,16
7156,1265,2012-06-14,0,4,0,14,21,7.33333349227905,42.3333320617676,3,2,203,79,0,1,0,1,0,9,16
7157,1265,2012-06-14,1,4,0,19,31.6666660308838,9.33333301544189,60,3,2,203,79,0,1,0,1,0,9,16
7158,1265,2012-06-14,2,4,0,18,32,10,60,3,2,203,79,0,1,0,1,0,9,16727.90661,14989.50623,14.18229
7159,1265,2012-06-14,3,4,0,31,20,9,60,3,2,203,79,0,1,0,1,0,9,16727.90661,14989.50623,14.182294
7160,1265,2012-06-14,4,4,0,40,14.3333330154419,5.66666650772095,60,3,2,203,79,0,1,0,1,0,9,16
7161,1265,2012-06-15,0,5,0,7.33333349227905,10.6666669845581,3.33333325386047,21.333333
7162,1265,2012-06-15,1,5,0,28.6666660308838,22,9.33333301544189,60,3,2,203,79,0,1,0,1,0,9,16
7163,1265,2012-06-15,2,5,0,31.3333339691162,19.6666660308838,9,60,3,2,203,79,0,1,0,1,0,9,167
7164,1265,2012-06-15,3,5,0,14.3333330154419,41.3333320617676,4.33333349227905,60,3,2,203,
7165,1265,2012-06-15,4,5,0,37.6666679382324,19.6666660308838,2.66666674613953,60,3,2,203,
7166,1265,2012-06-16,0,6,1,2,3.33333325386047,1.33333337306976,6.66666650772095,3,2,203,7
7167,1265,2012-06-16,1,6,1,36.3333320617676,19.3333339691162,4.33333349227905,60,3,2,203,
7168,1265,2012-06-16,2,6,1,34.3333320617676,20.3333339691162,5.33333349227905,60,3,2,203,
7169,1265,2012-06-16,3,6,1,39,19.6666660308838,1.33333337306976,60,3,2,203,79,0,1,0,1,0,9,16
7170,1265,2012-06-16,4,6,1,33.3333320617676,24.3333339691162,2.33333325386047,60,3,2,203,
7171,1265,2012-06-17,0,7,1,5.33333349227905,8,1,14.3333330154419,3,2,203,79,0,1,0,1,0,9,1672
7172,1265,2012-06-17,1,7,1,35,19,6,60,3,2,203,79,0,1,0,1,0,9,16727.90661,14989.50623,14.182294
7173,1265,2012-06-17,2,7,1,38.6666679382324,20,1.33333337306976,60,3,2,203,79,0,1,0,1,0,9,16
7174,1265,2012-06-17,3,7,1,44,12.6666669845581,3.33333325386047,60,3,2,203,79,0,1,0,1,0,9,16
7175,1265,2012-06-17,4,7,1,44,12.3333330154419,3.66666674613953,60,3,2,203,79,0,1,0,1,0,9,16
7176,1265,2012-06-18,0,1,0,18,18.6666660308838,6.33333349227905,43,3,2,203,79,0,1,0,1,0,9,16
7177,1265,2012-06-18,1,1,0,27.3333339691162,22.3333339691162,10.3333330154419,60,3,2,203,
7178,1265,2012-06-18,2,1,0,26.6666660308838,28.3333339691162,5,60,3,2,203,79,0,1,0,1,0,9,167
7179,1265,2012-06-18,3,1,0,26,31.6666660308838,2.33333325386047,60,3,2,203,79,0,1,0,1,0,9,16
7180,1265,2012-06-18,4,1,0,32.6666679382324,22.6666660308838,4.66666650772095,60,3,2,203,
7181,1267,2011-07-11,0,1,0,60,0,0,60,1,2,203,82,1,1,0,1,0,8,16832.62164,15040.94259,13.963100
7182,1267,2011-07-11,1,1,0,29.6666660308838,24,6.33333349227905,60,3,2,203,82,1,1,0,1,0,8,16
7183,1267,2011-07-11,2,1,0,41.6666679382324,17,1.33333337306976,60,3,2,203,82,1,1,0,1,0,8,16
7184,1267,2011-07-11,3,1,0,59,1,0,60,1,2,203,82,1,1,0,1,0,8,16832.62164,15040.94259,13.963100
7185,1267,2011-07-12,0,2,0,28,26,1,55,1,2,203,82,1,1,0,1,0,8,16832.62164,15040.94259,13.96310
7186,1267,2011-07-12,1,2,0,7,37.6666679382324,15.3333330154419,60,3,2,203,82,1,1,0,1,0,8,168
7187,1267,2011-07-12,2,2,0,28,31.6666660308838,0.333333343267441,60,3,2,203,82,1,1,0,1,0,8,1
7188,1267,2011-07-12,3,2,0,22.6000003814697,35,2.40000009536743,60,3,2,203,82,1,1,0,1,0,8,16
7189,1267,2011-07-12,4,2,0,30,29.75,0.25,60,3,2,203,82,1,1,0,1,0,8,16832.62164,15040.94259,13.9
7190,1267,2011-07-13,0,3,0,18,35,2,55,1,2,203,82,1,1,0,1,0,8,16832.62164,15040.94259,13.96310
7191,1267,2011-07-13,1,3,0,13.6666669845581,41,5.33333349227905,60,3,2,203,82,1,1,0,1,0,8,16
7192,1267,2011-07-13,2,3,0,17.6666660308838,38.3333320617676,4,60,3,2,203,82,1,1,0,1,0,8,168
7193,1267,2011-07-13,3,3,0,18.6666660308838,40.3333320617676,1,60,3,2,203,82,1,1,0,1,0,8,168
7194,1267,2011-07-13,4,3,0,33.4000015258789,26.6000003814697,0,60,3,2,203,82,1,1,0,1,0,8,168
7195,1267,2011-07-14,0,4,0,16.5,13.75,1,31.25,3,2,203,82,1,1,0,1,0,8,16832.62164,15040.94259,13
7196,1267,2011-07-14,1,4,0,3.33333325386047,50.3333320617676,6.33333349227905,60,3,2,203,
7197,1267,2011-07-14,2,4,0,27.3333339691162,30,2.66666674613953,60,3,2,203,82,1,1,0,1,0,8,16
7198,1267,2011-07-14,3,4,0,13.6666669845581,40.3333320617676,6,60,3,2,203,82,1,1,0,1,0,8,168
7199,1267,2011-07-14,4,4,0,41.6666679382324,17.6666660308838,0.666666686534882,60,3,2,20

7200,1267,2011-07-16,0,6,1,41,12,4,57,1,2,203,82,1,1,0,1,0,8,16832.62164,15040.94259,13.96310(
7201,1267,2011-07-16,1,6,1,22.7999992370605,32.2000007629395,5,60,3,2,203,82,1,1,0,1,0,8,168
7202,1267,2011-07-16,2,6,1,31.3333339691162,24.6666660308838,4,60,3,2,203,82,1,1,0,1,0,8,168
7203,1267,2011-07-16,3,6,1,42.6666679382324,17.3333339691162,0,60,3,2,203,82,1,1,0,1,0,8,168
7204,1267,2011-07-16,4,6,1,34.6666679382324,22.6666660308838,2.66666674613953,60,3,2,203,
7205,1267,2011-07-17,0,0,1,26,27,6,59,1,2,203,82,1,1,0,1,0,8,16832.62164,15040.94259,13.96310(
7206,1267,2011-07-17,1,0,1,4.25,47.5,8.25,60,3,2,203,82,1,1,0,1,0,8,16832.62164,15040.94259,13.
7207,1267,2011-07-17,2,0,1,14.8000001907349,40,5.19999980926514,60,3,2,203,82,1,1,0,1,0,8,16
7208,1267,2011-07-17,3,0,1,10,41,9,60,3,2,203,82,1,1,0,1,0,8,16832.62164,15040.94259,13.96310(
7209,1267,2011-07-17,4,0,1,41.3333320617676,15.3333330154419,3.33333325386047,60,3,2,203,
7210,1267,2011-07-18,0,1,0,26,32,2,60,1,2,203,82,1,1,0,1,0,8,16832.62164,15040.94259,13.96310(
7211,1267,2011-07-18,1,1,0,6.33333349227905,51.6666679382324,2,60,3,2,203,82,1,1,0,1,0,8,168
7212,1267,2011-07-18,2,1,0,26.5,30.25,3.25,60,3,2,203,82,1,1,0,1,0,8,16832.62164,15040.94259,13
7213,1267,2011-07-18,3,1,0,31,24.75,4.25,60,3,2,203,82,1,1,0,1,0,8,16832.62164,15040.94259,13.9
7214,1267,2011-07-18,4,1,0,36,22.3333339691162,1.66666662693024,60,3,2,203,82,1,1,0,1,0,8,16
7215,1267,2011-07-19,3,2,0,33,26.5,0.5,60,2,2,203,82,1,1,0,1,0,8,16832.62164,15040.94259,13.963
7216,1267,2011-07-19,4,2,0,37.6666679382324,21.3333339691162,1,60,3,2,203,82,1,1,0,1,0,8,168
7217,1288,2011-07-16,0,6,1,15.3333330154419,37.6666679382324,7,60,3,2,203,82,1,1,0,1,0,8,167
7218,1288,2011-07-16,1,6,1,23.6666660308838,35.6666679382324,0.666666686534882,60,3,2,203
7219,1288,2011-07-16,2,6,1,39.3333320617676,19.3333339691162,1.33333337306976,60,3,2,203,
7220,1288,2011-07-16,3,6,1,12.6666669845581,31.3333339691162,16,60,3,2,203,82,1,1,0,1,0,8,16
7221,1288,2011-07-16,4,6,1,14.6666669845581,26.6666660308838,18.6666660308838,60,3,2,203,
7222,1288,2011-07-17,0,0,1,2,36,7.33333349227905,45.3333320617676,3,2,203,82,1,1,0,1,0,8,167
7223,1288,2011-07-17,1,0,1,8.33333301544189,47.6666679382324,4,60,3,2,203,82,1,1,0,1,0,8,167
7224,1288,2011-07-17,2,0,1,37,21.3333339691162,1.66666662693024,60,3,2,203,82,1,1,0,1,0,8,16
7225,1288,2011-07-17,3,0,1,21,31,8,60,3,2,203,82,1,1,0,1,0,8,16754.48221,14258.17485,13.181388
7226,1288,2011-07-17,4,0,1,31.5,22.75,5.75,60,3,2,203,82,1,1,0,1,0,8,16754.48221,14258.17485,13
7227,1288,2011-07-18,0,1,0,14,40,6,60,3,2,203,82,1,1,0,1,0,8,16754.48221,14258.17485,13.181388
7228,1288,2011-07-18,1,1,0,4.33333349227905,40.3333320617676,15.3333330154419,60,3,2,203,
7229,1288,2011-07-18,2,1,0,27,27.3333339691162,5.66666650772095,60,3,2,203,82,1,1,0,1,0,8,16
7230,1288,2011-07-18,3,1,0,25,32,3,60,3,2,203,82,1,1,0,1,0,8,16754.48221,14258.17485,13.181388
7231,1288,2011-07-18,4,1,0,24.6666660308838,25.3333339691162,10,60,3,2,203,82,1,1,0,1,0,8,16
7232,1288,2011-07-19,0,2,0,3,39,18,60,1,2,203,82,1,1,0,1,0,8,16754.48221,14258.17485,13.181388
7233,1288,2011-07-19,1,2,0,6.33333349227905,45.6666679382324,8,60,3,2,203,82,1,1,0,1,0,8,167
7234,1288,2011-07-19,2,2,0,28,27.6666660308838,4.33333349227905,60,3,2,203,82,1,1,0,1,0,8,16
7235,1288,2011-07-19,3,2,0,14,39,7,60,3,2,203,82,1,1,0,1,0,8,16754.48221,14258.17485,13.181388
7236,1288,2011-07-19,4,2,0,8,45,7,60,1,2,203,82,1,1,0,1,0,8,16754.48221,14258.17485,13.1813885
7237,1288,2011-07-20,0,3,0,11.6666669845581,42,6.33333349227905,60,3,2,203,82,1,1,0,1,0,8,16
7238,1288,2011-07-20,1,3,0,6.33333349227905,49,4.66666650772095,60,3,2,203,82,1,1,0,1,0,8,16
7239,1288,2011-07-20,2,3,0,46,11.6666669845581,2.33333325386047,60,3,2,203,82,1,1,0,1,0,8,16
7240,1288,2011-07-20,3,3,0,13.3333330154419,38.6666679382324,8,60,3,2,203,82,1,1,0,1,0,8,167
7241,1288,2011-07-20,4,3,0,21.6666660308838,32.3333320617676,6,60,3,2,203,82,1,1,0,1,0,8,167
7242,1288,2011-07-21,0,4,0,1.66666662693024,30,9.66666698455811,41.3333320617676,3,2,203,
7243,1288,2011-07-21,1,4,0,14.6666669845581,40,5.33333349227905,60,3,2,203,82,1,1,0,1,0,8,16
7244,1288,2011-07-21,2,4,0,36,19.3333339691162,4.66666650772095,60,3,2,203,82,1,1,0,1,0,8,16
7245,1288,2011-07-21,3,4,0,14,42.3333320617676,3.66666674613953,60,3,2,203,82,1,1,0,1,0,8,16
7246,1288,2011-07-21,4,4,0,15.75,35.25,9,60,3,2,203,82,1,1,0,1,0,8,16754.48221,14258.17485,13.1
7247,1288,2011-07-22,0,5,0,2.66666674613953,32.6666679382324,6,41.3333320617676,3,2,203,8
7248,1288,2011-07-22,1,5,0,7.66666650772095,40.3333320617676,12,60,3,2,203,82,1,1,0,1,0,8,16
7249,1288,2011-07-22,2,5,0,44,15,1,60,3,2,203,82,1,1,0,1,0,8,16754.48221,14258.17485,13.181388

7250,1288,2011-07-22,3,5,0,9.33333301544189,42.6666679382324,8,60,3,2,203,82,1,1,0,1,0,8,167
7251,1288,2011-07-22,4,5,0,26,28.3333339691162,5.66666650772095,60,3,2,203,82,1,1,0,1,0,8,16
7252,1290,2012-06-14,0,4,0,26,27,7,60,1,2,203,67,1,1,1,1,0,8,16565.44508,14213.85733,12.52508
7253,1290,2012-06-14,1,4,0,23.6666660308838,27.6666660308838,8.66666698455811,60,3,2,203,
7254,1290,2012-06-14,2,4,0,25.3333339691162,26.6666660308838,8,60,3,2,203,67,1,1,1,1,0,8,165
7255,1290,2012-06-14,3,4,0,29.6666660308838,21.6666660308838,8.66666698455811,60,3,2,203,
7256,1290,2012-06-14,4,4,0,32.3333320617676,23.3333339691162,4.33333349227905,60,3,2,203,
7257,1290,2012-06-15,0,5,0,25.3333339691162,4,2.66666674613953,32,3,2,203,67,1,1,1,1,0,8,165
7258,1290,2012-06-15,1,5,0,27,28,5,60,3,2,203,67,1,1,1,1,0,8,16565.44508,14213.85733,12.52508
7259,1290,2012-06-15,2,5,0,28.6666660308838,27.3333339691162,4,60,3,2,203,67,1,1,1,1,0,8,165
7260,1290,2012-06-15,3,5,0,32.3333320617676,22.3333339691162,5.33333349227905,60,3,2,203,
7261,1290,2012-06-15,4,5,0,28,30.3333339691162,1.66666662693024,60,3,2,203,67,1,1,1,1,0,8,16
7262,1290,2012-06-16,0,6,1,8.33333301544189,17,2.66666674613953,28,3,2,203,67,1,1,1,1,0,8,16
7263,1290,2012-06-16,1,6,1,31.3333339691162,27,1.66666662693024,60,3,2,203,67,1,1,1,1,0,8,16
7264,1290,2012-06-16,2,6,1,40.6666679382324,15.3333330154419,4,60,3,2,203,67,1,1,1,1,0,8,165
7265,1290,2012-06-16,3,6,1,44,14.6666669845581,1.33333337306976,60,3,2,203,67,1,1,1,1,0,8,16
7266,1290,2012-06-16,4,6,1,47.3333320617676,12.6666669845581,0,60,3,2,203,67,1,1,1,1,0,8,165
7267,1290,2012-06-17,1,7,1,10.6666669845581,9.33333301544189,0.666666686534882,20.66666
7268,1290,2012-06-17,2,7,1,28,31,1,60,3,2,203,67,1,1,1,1,0,8,16565.44508,14213.85733,12.52508
7269,1290,2012-06-17,3,7,1,26.6666660308838,33,0.333333343267441,60,3,2,203,67,1,1,1,1,0,8,1
7270,1290,2012-06-17,4,7,1,16.6666660308838,43,0.333333343267441,60,3,2,203,67,1,1,1,1,0,8,1
7271,1290,2012-06-18,0,1,0,20.6666660308838,22.3333339691162,2.33333325386047,45.333332
7272,1290,2012-06-18,1,1,0,12.3333330154419,40.3333320617676,7.33333349227905,60,3,2,203,
7273,1290,2012-06-18,2,1,0,14.6666669845581,38.3333320617676,7,60,3,2,203,67,1,1,1,1,0,8,165
7274,1290,2012-06-18,3,1,0,19.3333339691162,35.6666679382324,5,60,3,2,203,67,1,1,1,1,0,8,165
7275,1290,2012-06-18,4,1,0,24.6666660308838,29.3333339691162,6,60,3,2,203,67,1,1,1,1,0,8,165
7276,1290,2012-06-19,0,2,0,19,14.3333330154419,2.66666674613953,36,3,2,203,67,1,1,1,1,0,8,16
7277,1290,2012-06-19,1,2,0,30.6666660308838,27.3333339691162,2,60,3,2,203,67,1,1,1,1,0,8,165
7278,1290,2012-06-19,2,2,0,31,16.6666660308838,12.3333330154419,60,3,2,203,67,1,1,1,1,0,8,16
7279,1290,2012-06-19,3,2,0,31,27,2,60,3,2,203,67,1,1,1,1,0,8,16565.44508,14213.85733,12.52508
7280,1290,2012-06-19,4,2,0,20.3333339691162,38.3333320617676,1.33333337306976,60,3,2,203,
7281,1290,2012-06-20,0,3,0,9.33333301544189,11.3333330154419,2,22.6666660308838,3,2,203,6
7282,1290,2012-06-20,1,3,0,33,18.6666660308838,8.33333301544189,60,3,2,203,67,1,1,1,1,0,8,16
7283,1290,2012-06-20,2,3,0,26.6666660308838,26.3333339691162,7,60,3,2,203,67,1,1,1,1,0,8,165
7284,1290,2012-06-20,3,3,0,40,13,7,60,3,2,203,67,1,1,1,1,0,8,16565.44508,14213.85733,12.52508
7285,1290,2012-06-20,4,3,0,28,30.3333339691162,1.66666662693024,60,3,2,203,67,1,1,1,1,0,8,16
7286,1293,2011-07-08,1,5,0,23.6666660308838,29.3333339691162,6,59,3,2,205,76,1,1,1,1,0,8,164
7287,1293,2011-07-08,2,5,0,34,26,0,60,3,2,205,76,1,1,1,1,0,8,16431.22913,14709.99232,10.41849
7288,1293,2011-07-08,3,5,0,34.8333320617676,25,0.16666667163372,60,3,2,205,76,1,1,1,1,0,8,16
7289,1293,2011-07-08,4,5,0,34.6666679382324,14.3333330154419,0,49,3,2,205,76,1,1,1,1,0,8,164
7290,1293,2011-07-09,0,6,1,10.5,20,12,42.5,2,2,205,76,1,1,1,1,0,8,16431.22913,14709.99232,10.41
7291,1293,2011-07-09,1,6,1,36.3333320617676,16,7.66666650772095,60,3,2,205,76,1,1,1,1,0,8,16
7292,1293,2011-07-09,2,6,1,53.6666679382324,6.33333349227905,0,60,3,2,205,76,1,1,1,1,0,8,164
7293,1293,2011-07-09,3,6,1,39,18,3,60,3,2,205,76,1,1,1,1,0,8,16431.22913,14709.99232,10.41849
7294,1293,2011-07-09,4,6,1,45.5999984741211,14,0.400000005960464,60,3,2,205,76,1,1,1,1,0,8,1
7295,1293,2011-07-10,4,0,1,29,8,0,37,1,2,205,76,1,1,1,1,0,8,16431.22913,14709.99232,10.418492
7296,1293,2011-07-19,0,2,0,26,19,0.333333343267441,45.3333320617676,3,2,205,76,1,1,1,1,0,8,1
7297,1293,2011-07-19,1,2,0,34,23,3,60,3,2,205,76,1,1,1,1,0,8,16431.22913,14709.99232,10.41849
7298,1293,2011-07-19,2,2,0,32.3333320617676,20,7.66666650772095,60,3,2,205,76,1,1,1,1,0,8,16
7299,1293,2011-07-19,3,2,0,40.3333320617676,14,5.66666650772095,60,3,2,205,76,1,1,1,1,0,8,16

7300,1293,2011-07-19,4,2,0,46.3333320617676,12,0.333333343267441,58.6666679382324,3,2,205
7301,1293,2011-07-20,0,3,0,19,19.5,1,39.5,2,2,205,76,1,1,1,1,0,8,16431.22913,14709.99232,10.418
7302,1293,2011-07-20,1,3,0,30.3333339691162,29.3333339691162,0.333333343267441,60,3,2,205
7303,1293,2011-07-20,2,3,0,51.6666679382324,8,0.333333343267441,60,3,2,205,76,1,1,1,1,0,8,16
7304,1293,2011-07-20,3,3,0,22.6666660308838,29.3333339691162,8,60,3,2,205,76,1,1,1,1,0,8,164
7305,1293,2011-07-20,4,3,0,39,14.3333330154419,0.333333343267441,53.6666679382324,3,2,205
7306,1293,2011-07-21,0,4,0,20,14,1.5,35.5,2,2,205,76,1,1,1,1,0,8,16431.22913,14709.99232,10.418
7307,1293,2011-07-21,1,4,0,31.5,25.5,3,60,3,2,205,76,1,1,1,1,0,8,16431.22913,14709.99232,10.418
7308,1293,2011-07-21,2,4,0,45.3333320617676,14.3333330154419,0.333333343267441,60,3,2,205
7309,1293,2011-07-21,3,4,0,33.6666679382324,23.6666660308838,2.66666674613953,60,3,2,205,
7310,1293,2011-07-21,4,4,0,35,24.3333339691162,0.666666686534882,60,3,2,205,76,1,1,1,1,0,8,1
7311,1293,2011-07-22,1,5,0,19.5,27.75,1.75,49,3,2,205,76,1,1,1,1,0,8,16431.22913,14709.99232,10
7312,1293,2011-07-22,2,5,0,33.6666679382324,23.1666660308838,3.16666674613953,60,3,2,205,
7313,1293,2011-07-22,3,5,0,41.3333320617676,17.6666660308838,1,60,3,2,205,76,1,1,1,1,0,8,164
7314,1293,2011-07-22,4,5,0,37.3333320617676,20,2.66666674613953,60,3,2,205,76,1,1,1,1,0,8,16
7315,1295,2011-07-10,0,0,1,23.5,21,3,47.5,2,2,205,85,0,1,1,0,0,9,16480.55589,14992.63173,10.382
7316,1295,2011-07-10,1,0,1,27.75,27.75,4.5,60,3,2,205,85,0,1,1,0,0,9,16480.55589,14992.63173,10
7317,1295,2011-07-10,2,0,1,34.3333320617676,25.6666660308838,0,60,3,2,205,85,0,1,1,0,0,9,164
7318,1295,2011-07-10,3,0,1,27.6666660308838,26.3333339691162,6,60,3,2,205,85,0,1,1,0,0,9,164
7319,1295,2011-07-10,4,0,1,36.6666679382324,16,1,53.6666679382324,3,2,205,85,0,1,1,0,0,9,164
7320,1295,2011-07-11,0,1,0,14,11,1,26,1,2,205,85,0,1,1,0,0,9,16480.55589,14992.63173,10.382064
7321,1295,2011-07-11,1,1,0,35.4000015258789,21.3999996185303,3.20000004768372,60,3,2,205,
7322,1295,2011-07-11,2,1,0,36.8333320617676,20.8333339691162,2.33333325386047,60,3,2,205,
7323,1295,2011-07-11,3,1,0,17.3333339691162,36.3333320617676,6.33333349227905,60,3,2,205,
7324,1295,2011-07-11,4,1,0,10.5,31.5,4.5,46.5,2,2,205,85,0,1,1,0,0,9,16480.55589,14992.63173,10
7325,1295,2011-07-12,0,2,0,10,10,1,21,1,2,205,85,0,1,1,0,0,9,16480.55589,14992.63173,10.382064
7326,1295,2011-07-12,1,2,0,31.6666660308838,24.3333339691162,4,60,3,2,205,85,0,1,1,0,0,9,164
7327,1295,2011-07-12,2,2,0,31.3333339691162,26.6666660308838,2,60,3,2,205,85,0,1,1,0,0,9,164
7328,1295,2011-07-12,3,2,0,26.7999992370605,25,8.19999980926514,60,3,2,205,85,0,1,1,0,0,9,16
7329,1295,2011-07-12,4,2,0,13.3333330154419,27.3333339691162,5.66666650772095,46.333332
7330,1295,2011-07-13,0,3,0,23,22,1,46,1,2,205,85,0,1,1,0,0,9,16480.55589,14992.63173,10.382064
7331,1295,2011-07-13,1,3,0,29,28,3,60,3,2,205,85,0,1,1,0,0,9,16480.55589,14992.63173,10.382064
7332,1295,2011-07-13,2,3,0,31.3333339691162,26,2.66666674613953,60,3,2,205,85,0,1,1,0,0,9,16
7333,1295,2011-07-13,3,3,0,34,19.25,6.75,60,3,2,205,85,0,1,1,0,0,9,16480.55589,14992.63173,10.3
7334,1295,2011-07-13,4,3,0,20.5,22.5,5.75,48.75,3,2,205,85,0,1,1,0,0,9,16480.55589,14992.63173,
7335,1295,2011-07-14,0,4,0,13.5,16,4,33.5,2,2,205,85,0,1,1,0,0,9,16480.55589,14992.63173,10.382
7336,1295,2011-07-14,1,4,0,38.6666679382324,21,0.333333343267441,60,3,2,205,85,0,1,1,0,0,9,1
7337,1295,2011-07-14,2,4,0,36.3333320617676,23.6666660308838,0,60,3,2,205,85,0,1,1,0,0,9,164
7338,1295,2011-07-14,3,4,0,32.6666679382324,27.3333339691162,0,60,3,2,205,85,0,1,1,0,0,9,164
7339,1295,2011-07-14,4,4,0,20.6666660308838,23.6666660308838,0,44.3333320617676,3,2,205,8
7340,1296,2011-07-14,0,4,0,57,3,0,60,1,2,205,72,1,1,1,0,0,5,16671.47638,17680.59529,13.1523690
7341,1296,2011-07-14,1,4,0,3,47,10,60,1,2,205,72,1,1,1,0,0,5,16671.47638,17680.59529,13.152369
7342,1296,2011-07-15,1,5,0,11,42.5999984741211,5.40000009536743,59,3,2,205,72,1,1,1,0,0,5,16
7343,1296,2011-07-15,2,5,0,4.66666650772095,52.3333320617676,3,60,3,2,205,72,1,1,1,0,0,5,166
7344,1296,2011-07-15,3,5,0,13,45.3333320617676,1.66666662693024,60,3,2,205,72,1,1,1,0,0,5,16
7345,1296,2011-07-15,4,5,0,34.3333320617676,25.6666660308838,0,60,3,2,205,72,1,1,1,0,0,5,166
7346,1296,2011-08-11,1,4,0,5.66666650772095,20.3333339691162,20.6666660308838,46.6666679
7347,1296,2011-08-11,2,4,0,29.5,27.25,3.25,60,3,2,205,72,1,1,1,0,0,5,16671.47638,17680.59529,13
7348,1296,2011-08-11,3,4,0,41.3333320617676,16.6666660308838,2,60,3,2,205,72,1,1,1,0,0,5,166
7349,1296,2011-08-11,4,4,0,27.3333339691162,32.6666679382324,0,60,3,2,205,72,1,1,1,0,0,5,166

7350,1296,2011-08-12,0,5,0,0,1,0,1,1,2,205,72,1,1,1,0,0,5,16671.47638,17680.59529,13.15236901,
7351,1296,2011-08-12,1,5,0,21.3333339691162,33,5.66666650772095,60,3,2,205,72,1,1,1,0,0,5,16
7352,1296,2011-08-12,2,5,0,23.3999996185303,28.6000003814697,8,60,3,2,205,72,1,1,1,0,0,5,166
7353,1296,2011-08-12,3,5,0,16.25,39.5,4.25,60,3,2,205,72,1,1,1,0,0,5,16671.47638,17680.59529,13
7354,1296,2011-08-12,4,5,0,18,24.6666660308838,2,44.6666679382324,3,2,205,72,1,1,1,0,0,5,166
7355,1296,2011-08-13,4,6,1,21,37,2,60,3,2,205,72,1,1,1,0,0,5,16671.47638,17680.59529,13.152369
7356,1296,2011-09-12,0,1,0,2,8,0,10,1,2,205,72,1,1,1,0,0,5,16671.47638,17680.59529,13.15236901
7357,1296,2011-09-12,1,1,0,17,36.3333320617676,6.66666650772095,60,3,2,205,72,1,1,1,0,0,5,16
7358,1296,2011-09-12,2,1,0,12.6666669845581,47.3333320617676,0,60,3,2,205,72,1,1,1,0,0,5,166
7359,1296,2011-09-12,3,1,0,28.2000007629395,30.7999992370605,1,60,3,2,205,72,1,1,1,0,0,5,166
7360,1296,2011-09-12,4,1,0,25,30,1.66666662693024,56.6666679382324,3,2,205,72,1,1,1,0,0,5,16
7361,1307,2011-11-04,0,5,0,56,2,2,60,1,2,205,75,1,1,1,1,0,11,17224.0234,21459.55025,11.3946492
7362,1307,2011-11-04,1,5,0,17.3333339691162,40.3333320617676,2.33333325386047,60,3,2,205,
7363,1307,2011-11-04,2,5,0,23.6666660308838,33,3.33333325386047,60,3,2,205,75,1,1,1,1,0,11,1
7364,1307,2011-11-04,3,5,0,22.6666660308838,31.6666660308838,5.66666650772095,60,3,2,205,
7365,1307,2011-11-04,4,5,0,40.3333320617676,18.6666660308838,1,60,3,2,205,75,1,1,1,1,0,11,17
7366,1307,2011-11-05,0,6,1,6.66666650772095,7.66666650772095,0.333333343267441,14.666666
7367,1307,2011-11-05,1,6,1,9.66666698455811,40.6666679382324,9.66666698455811,60,3,2,205,
7368,1307,2011-11-05,2,6,1,30.3333339691162,27.3333339691162,2.33333325386047,60,3,2,205,
7369,1307,2011-11-05,3,6,1,20.6666660308838,30.6666660308838,8.66666698455811,60,3,2,205,
7370,1307,2011-11-05,4,6,1,29.3333339691162,29.6666660308838,1,60,3,2,205,75,1,1,1,1,0,11,17
7371,1307,2011-11-06,1,7,1,30.6666660308838,27,0.666666686534882,58.3333320617676,3,2,205
7372,1307,2011-11-06,2,7,1,17.3333339691162,37.6666679382324,5,60,3,2,205,75,1,1,1,1,0,11,17
7373,1307,2011-11-06,3,7,1,49,11,0,60,3,2,205,75,1,1,1,1,0,11,17224.0234,21459.55025,11.394649
7374,1307,2011-11-06,4,7,1,32.3333320617676,25.6666660308838,2,60,3,2,205,75,1,1,1,1,0,11,17
7375,1307,2011-11-07,0,1,0,16,3.33333325386047,0.333333343267441,19.6666660308838,3,2,205
7376,1307,2011-11-07,1,1,0,24.3333339691162,35.3333320617676,0.333333343267441,60,3,2,205
7377,1307,2011-11-07,2,1,0,26.3333339691162,23,10.6666669845581,60,3,2,205,75,1,1,1,1,0,11,1
7378,1307,2011-11-07,3,1,0,17.6666660308838,13,29.3333339691162,60,3,2,205,75,1,1,1,1,0,11,1
7379,1307,2011-11-07,4,1,0,41.6666679382324,18.3333339691162,0,60,3,2,205,75,1,1,1,1,0,11,17
7380,1307,2011-11-08,0,2,0,16.3333339691162,10.3333330154419,1.33333337306976,28,3,2,205,
7381,1307,2011-11-08,1,2,0,15,37.3333320617676,7.66666650772095,60,3,2,205,75,1,1,1,1,0,11,1
7382,1307,2011-11-08,2,2,0,9.33333301544189,43,7.66666650772095,60,3,2,205,75,1,1,1,1,0,11,1
7383,1307,2011-11-08,3,2,0,34,21.6666660308838,4.33333349227905,60,3,2,205,75,1,1,1,1,0,11,1
7384,1307,2011-11-08,4,2,0,38,21.3333339691162,0.666666686534882,60,3,2,205,75,1,1,1,1,0,11,
7385,1307,2011-11-09,0,3,0,15.6666669845581,6,0.333333343267441,22,3,2,205,75,1,1,1,1,0,11,1
7386,1307,2011-11-09,1,3,0,17,31,12,60,3,2,205,75,1,1,1,1,0,11,17224.0234,21459.55025,11.39464
7387,1307,2011-11-09,2,3,0,25,34.3333320617676,0.666666686534882,60,3,2,205,75,1,1,1,1,0,11,
7388,1307,2011-11-09,3,3,0,38,14,8,60,3,2,205,75,1,1,1,1,0,11,17224.0234,21459.55025,11.394649
7389,1307,2011-11-09,4,3,0,34.3333320617676,24.6666660308838,1,60,3,2,205,75,1,1,1,1,0,11,17
7390,1309,2011-10-27,0,4,0,39,21,0,60,1,2,205,82,0,1,0,1,0,8,16579.17176,18264.6455,12.6922984
7391,1309,2011-10-27,1,4,0,13,44.3333320617676,2.66666674613953,60,3,2,205,82,0,1,0,1,0,8,16
7392,1309,2011-10-27,2,4,0,34,24,2,60,3,2,205,82,0,1,0,1,0,8,16579.17176,18264.6455,12.6922984
7393,1309,2011-10-27,3,4,0,30,28.3333339691162,1.66666662693024,60,3,2,205,82,0,1,0,1,0,8,16
7394,1309,2011-10-27,4,4,0,41.3333320617676,18.3333339691162,0.333333343267441,60,3,2,205
7395,1309,2011-10-28,1,5,0,13,26,2,41,3,2,205,82,0,1,0,1,0,8,16579.17176,18264.6455,12.6922984
7396,1309,2011-10-28,2,5,0,48.3333320617676,11.6666669845581,0,60,3,2,205,82,0,1,0,1,0,8,165
7397,1309,2011-10-28,3,5,0,40.3333320617676,19.6666660308838,0,60,3,2,205,82,0,1,0,1,0,8,165
7398,1309,2011-10-28,4,5,0,45,15,0,60,3,2,205,82,0,1,0,1,0,8,16579.17176,18264.6455,12.6922984
7399,1309,2011-10-29,1,6,1,18.6666660308838,17.3333339691162,5,41,3,2,205,82,0,1,0,1,0,8,165

7400,1309,2011-10-29,2,6,1,51,9,0,60,3,2,205,82,0,1,0,1,0,8,16579.17176,18264.6455,12.69229849
7401,1309,2011-10-29,3,6,1,41.6666679382324,18.3333339691162,0,60,3,2,205,82,0,1,0,1,0,8,165
7402,1309,2011-10-29,4,6,1,45.3333320617676,14.6666669845581,0,60,3,2,205,82,0,1,0,1,0,8,165
7403,1309,2011-10-30,0,7,1,5,9,0.666666686534882,14.6666669845581,3,2,205,82,0,1,0,1,0,8,165
7404,1309,2011-10-30,1,7,1,24.6666660308838,34,1.33333337306976,60,3,2,205,82,0,1,0,1,0,8,16
7405,1309,2011-10-30,2,7,1,51,9,0,60,3,2,205,82,0,1,0,1,0,8,16579.17176,18264.6455,12.69229849
7406,1309,2011-10-30,3,7,1,51.3333320617676,8.66666698455811,0,60,3,2,205,82,0,1,0,1,0,8,165
7407,1309,2011-10-30,4,7,1,49,11,0,60,3,2,205,82,0,1,0,1,0,8,16579.17176,18264.6455,12.6922984
7408,1309,2011-10-31,0,1,0,0,4,0.333333343267441,4.33333349227905,3,2,205,82,0,1,0,1,0,8,165
7409,1309,2011-10-31,1,1,0,23.6666660308838,32.3333320617676,4,60,3,2,205,82,0,1,0,1,0,8,165
7410,1309,2011-10-31,2,1,0,47.6666679382324,12.3333330154419,0,60,3,2,205,82,0,1,0,1,0,8,165
7411,1309,2011-10-31,3,1,0,29.3333339691162,26.6666660308838,4,60,3,2,205,82,0,1,0,1,0,8,165
7412,1309,2011-10-31,4,1,0,49.6666679382324,10.3333330154419,0,60,3,2,205,82,0,1,0,1,0,8,165
7413,1309,2011-11-01,1,2,0,36.3333320617676,11.6666669845581,0.333333343267441,48.333332
7414,1309,2011-11-01,2,2,0,45,15,0,60,3,2,205,82,0,1,0,1,0,8,16579.17176,18264.6455,12.6922984
7415,1309,2011-11-01,3,2,0,44.3333320617676,15.6666669845581,0,60,3,2,205,82,0,1,0,1,0,8,165
7416,1309,2011-11-01,4,2,0,46.3333320617676,13.6666669845581,0,60,3,2,205,82,0,1,0,1,0,8,165
7417,1309,2011-11-02,1,3,0,23,25.3333339691162,5.33333349227905,53.6666679382324,3,2,205,
7418,1309,2011-11-02,2,3,0,35.3333320617676,18.6666660308838,6,60,3,2,205,82,0,1,0,1,0,8,165
7419,1309,2011-11-02,3,3,0,29,28.3333339691162,2.66666674613953,60,3,2,205,82,0,1,0,1,0,8,16
7420,1309,2011-11-02,4,3,0,48.3333320617676,11.6666669845581,0,60,3,2,205,82,0,1,0,1,0,8,165
7421,1311,2011-10-27,0,4,0,46,14,0,60,1,2,205,69,1,0,0,1,0,9,17007.07681,19995.97223,10.978589
7422,1311,2011-10-27,1,4,0,41.6666679382324,13.6666669845581,4.66666650772095,60,3,2,205,
7423,1311,2011-10-27,2,4,0,32.3333320617676,21,6.66666650772095,60,3,2,205,69,1,0,0,1,0,9,17
7424,1311,2011-10-27,3,4,0,42,18,0,60,3,2,205,69,1,0,0,1,0,9,17007.07681,19995.97223,10.978589
7425,1311,2011-10-27,4,4,0,48,12,0,60,3,2,205,69,1,0,0,1,0,9,17007.07681,19995.97223,10.978589
7426,1311,2011-10-28,0,5,0,0.666666686534882,4,0,4.66666650772095,3,2,205,69,1,0,0,1,0,9,170
7427,1311,2011-10-28,1,5,0,27.3333339691162,24.6666660308838,8,60,3,2,205,69,1,0,0,1,0,9,170
7428,1311,2011-10-28,2,5,0,36,19,5,60,3,2,205,69,1,0,0,1,0,9,17007.07681,19995.97223,10.978589
7429,1311,2011-10-28,3,5,0,39,15,6,60,3,2,205,69,1,0,0,1,0,9,17007.07681,19995.97223,10.978589
7430,1311,2011-10-28,4,5,0,52.3333320617676,7,0.666666686534882,60,3,2,205,69,1,0,0,1,0,9,17
7431,1311,2011-10-29,0,6,1,3.33333325386047,4.66666650772095,0.333333343267441,8.3333330
7432,1311,2011-10-29,1,6,1,18,41,1,60,3,2,205,69,1,0,0,1,0,9,17007.07681,19995.97223,10.978589
7433,1311,2011-10-29,2,6,1,34,24.6666660308838,1.33333337306976,60,3,2,205,69,1,0,0,1,0,9,17
7434,1311,2011-10-29,3,6,1,34.6666679382324,24.6666660308838,0.666666686534882,60,3,2,205
7435,1311,2011-10-29,4,6,1,39,20,1,60,3,2,205,69,1,0,0,1,0,9,17007.07681,19995.97223,10.978589
7436,1311,2011-10-30,1,7,1,17.6666660308838,24.3333339691162,4,46,3,2,205,69,1,0,0,1,0,9,170
7437,1311,2011-10-30,2,7,1,50.6666679382324,9.33333301544189,0,60,3,2,205,69,1,0,0,1,0,9,170
7438,1311,2011-10-30,3,7,1,47.6666679382324,11,1.33333337306976,60,3,2,205,69,1,0,0,1,0,9,17
7439,1311,2011-10-30,4,7,1,48,11.3333330154419,0.666666686534882,60,3,2,205,69,1,0,0,1,0,9,1
7440,1311,2011-10-31,0,1,0,12.3333330154419,13,0.333333343267441,25.6666660308838,3,2,205
7441,1311,2011-10-31,1,1,0,31.3333339691162,24,4.66666650772095,60,3,2,205,69,1,0,0,1,0,9,17
7442,1311,2011-10-31,2,1,0,54.6666679382324,3,2.33333325386047,60,3,2,205,69,1,0,0,1,0,9,170
7443,1311,2011-10-31,3,1,0,40,15.6666669845581,4.33333349227905,60,3,2,205,69,1,0,0,1,0,9,17
7444,1311,2011-10-31,4,1,0,42.3333320617676,17.3333339691162,0.333333343267441,60,3,2,205
7445,1311,2011-11-01,1,2,0,17.6666660308838,19,2.33333325386047,39,3,2,205,69,1,0,0,1,0,9,17
7446,1311,2011-11-01,2,2,0,18.3333339691162,38,3.66666674613953,60,3,2,205,69,1,0,0,1,0,9,17
7447,1311,2011-11-01,3,2,0,58.6666679382324,1.33333337306976,0,60,3,2,205,69,1,0,0,1,0,9,170
7448,1311,2011-11-01,4,2,0,49,11,0,60,3,2,205,69,1,0,0,1,0,9,17007.07681,19995.97223,10.978589
7449,1311,2011-11-02,0,3,0,11.3333330154419,14,0.666666686534882,26,3,2,205,69,1,0,0,1,0,9,1

7450,1311,2011-11-02,1,3,0,18.3333339691162,30.6666660308838,11,60,3,2,205,69,1,0,0,1,0,9,17
7451,1311,2011-11-02,2,3,0,40.3333320617676,11.6666669845581,8,60,3,2,205,69,1,0,0,1,0,9,170
7452,1311,2011-11-02,3,3,0,39.3333320617676,17.6666660308838,3,60,3,2,205,69,1,0,0,1,0,9,170
7453,1311,2011-11-02,4,3,0,48.6666679382324,11.3333330154419,0,60,3,2,205,69,1,0,0,1,0,9,170
7454,1312,2011-10-28,0,5,0,15,26,19,60,1,2,205,68,1,1,0,1,0,10,16906.60888,18676.31681,11.9652
7455,1312,2011-10-28,1,5,0,15.3333330154419,41.3333320617676,3.33333325386047,60,3,2,205,
7456,1312,2011-10-28,2,5,0,25,28.6666660308838,6.33333349227905,60,3,2,205,68,1,1,0,1,0,10,1
7457,1312,2011-10-28,3,5,0,25.6666660308838,18.6666660308838,15.6666669845581,60,3,2,205,
7458,1312,2011-10-28,4,5,0,33,26,1,60,3,2,205,68,1,1,0,1,0,10,16906.60888,18676.31681,11.96521
7459,1312,2011-10-29,0,6,1,5.33333349227905,8,5.33333349227905,18.6666660308838,3,2,205,6
7460,1312,2011-10-29,1,6,1,23.6666660308838,31,5.33333349227905,60,3,2,205,68,1,1,0,1,0,10,1
7461,1312,2011-10-29,2,6,1,21.3333339691162,35.3333320617676,3.33333325386047,60,3,2,205,
7462,1312,2011-10-29,3,6,1,28,18.3333339691162,13.6666669845581,60,3,2,205,68,1,1,0,1,0,10,1
7463,1312,2011-10-29,4,6,1,44,10,6,60,3,2,205,68,1,1,0,1,0,10,16906.60888,18676.31681,11.96521
7464,1312,2011-10-30,0,7,1,0,2.33333325386047,0,2.33333325386047,3,2,205,68,1,1,0,1,0,10,169
7465,1312,2011-10-30,1,7,1,25.3333339691162,25,9.66666698455811,60,3,2,205,68,1,1,0,1,0,10,1
7466,1312,2011-10-30,2,7,1,36.6666679382324,23.3333339691162,0,60,3,2,205,68,1,1,0,1,0,10,16
7467,1312,2011-10-30,3,7,1,25.3333339691162,15.3333330154419,19.3333339691162,60,3,2,205,
7468,1312,2011-10-30,4,7,1,34,13.6666669845581,12.3333330154419,60,3,2,205,68,1,1,0,1,0,10,1
7469,1312,2011-10-31,0,1,0,6,15.3333330154419,2,23.3333339691162,3,2,205,68,1,1,0,1,0,10,169
7470,1312,2011-10-31,1,1,0,9.33333301544189,40,10.6666669845581,60,3,2,205,68,1,1,0,1,0,10,1
7471,1312,2011-10-31,2,1,0,29.6666660308838,30.3333339691162,0,60,3,2,205,68,1,1,0,1,0,10,16
7472,1312,2011-10-31,3,1,0,25,21,14,60,3,2,205,68,1,1,0,1,0,10,16906.60888,18676.31681,11.9652
7473,1312,2011-10-31,4,1,0,33.3333320617676,24,2.66666674613953,60,3,2,205,68,1,1,0,1,0,10,1
7474,1312,2011-11-01,0,2,0,2,5.33333349227905,0,7.33333349227905,3,2,205,68,1,1,0,1,0,10,169
7475,1312,2011-11-01,1,2,0,23.6666660308838,27.3333339691162,9,60,3,2,205,68,1,1,0,1,0,10,16
7476,1312,2011-11-01,2,2,0,29.3333339691162,30,0.666666686534882,60,3,2,205,68,1,1,0,1,0,10,
7477,1312,2011-11-01,3,2,0,31.6666660308838,20.6666660308838,7.66666650772095,60,3,2,205,
7478,1312,2011-11-01,4,2,0,42,12,6,60,3,2,205,68,1,1,0,1,0,10,16906.60888,18676.31681,11.96521
7479,1312,2011-11-02,0,3,0,6.66666650772095,8,7.33333349227905,22,3,2,205,68,1,1,0,1,0,10,16
7480,1312,2011-11-02,1,3,0,23,31.3333339691162,5.66666650772095,60,3,2,205,68,1,1,0,1,0,10,1
7481,1312,2011-11-02,2,3,0,22.6666660308838,37.3333320617676,0,60,3,2,205,68,1,1,0,1,0,10,16
7482,1312,2011-11-02,3,3,0,33.6666679382324,15,11.3333330154419,60,3,2,205,68,1,1,0,1,0,10,1
7483,1312,2011-11-02,4,3,0,30,29.6666660308838,0.333333343267441,60,3,2,205,68,1,1,0,1,0,10,
7484,1312,2011-11-03,0,4,0,7.66666650772095,14,0,21.6666660308838,3,2,205,68,1,1,0,1,0,10,16
7485,1312,2011-11-03,1,4,0,18.6666660308838,31.6666660308838,9.66666698455811,60,3,2,205,
7486,1312,2011-11-03,2,4,0,31.6666660308838,28.3333339691162,0,60,3,2,205,68,1,1,0,1,0,10,16
7487,1312,2011-11-03,3,4,0,33,27,0,60,3,2,205,68,1,1,0,1,0,10,16906.60888,18676.31681,11.96521
7488,1312,2011-11-03,4,4,0,28,32,0,60,3,2,205,68,1,1,0,1,0,10,16906.60888,18676.31681,11.96521
7489,1313,2011-11-04,0,5,0,19,32,9,60,1,2,205,70,1,1,1,0,0,12,16789.15885,21002.09294,9.795398
7490,1313,2011-11-04,1,5,0,8,48.6666679382324,3.33333325386047,60,3,2,205,70,1,1,1,0,0,12,16
7491,1313,2011-11-04,2,5,0,27.3333339691162,30.6666660308838,2,60,3,2,205,70,1,1,1,0,0,12,16
7492,1313,2011-11-04,3,5,0,32,16.3333339691162,11.6666669845581,60,3,2,205,70,1,1,1,0,0,12,1
7493,1313,2011-11-04,4,5,0,25,32.6666679382324,2,59.6666679382324,3,2,205,70,1,1,1,0,0,12,16
7494,1313,2011-11-06,0,7,1,0,1,0.666666686534882,1.66666662693024,3,2,205,70,1,1,1,0,0,12,16
7495,1313,2011-11-06,1,7,1,21.6666660308838,34.6666679382324,3.66666674613953,60,3,2,205,
7496,1313,2011-11-06,2,7,1,16,40,4,60,3,2,205,70,1,1,1,0,0,12,16789.15885,21002.09294,9.795398
7497,1313,2011-11-06,3,7,1,41.3333320617676,18.6666660308838,0,60,3,2,205,70,1,1,1,0,0,12,16
7498,1313,2011-11-06,4,7,1,28,16,2.66666674613953,46.6666679382324,3,2,205,70,1,1,1,0,0,12,1
7499,1313,2011-11-07,0,1,0,2,7.33333349227905,0.333333343267441,9.66666698455811,3,2,205,

7500,1313,2011-11-07,1,1,0,5.66666650772095,53.6666679382324,0.666666686534882,60,3,2,205
7501,1313,2011-11-07,2,1,0,17,41,2,60,3,2,205,70,1,1,1,0,0,12,16789.15885,21002.09294,9.795398
7502,1313,2011-11-07,3,1,0,13.6666669845581,19.3333339691162,27,60,3,2,205,70,1,1,1,0,0,12,1
7503,1313,2011-11-07,4,1,0,10.3333330154419,19.3333339691162,2.66666674613953,32.333332
7504,1313,2011-11-08,0,2,0,0.333333343267441,3.66666674613953,0.666666686534882,4.66666
7505,1313,2011-11-08,1,2,0,17.6666660308838,39.6666679382324,2.66666674613953,60,3,2,205,
7506,1313,2011-11-08,2,2,0,14.6666669845581,43.3333320617676,2,60,3,2,205,70,1,1,1,0,0,12,16
7507,1313,2011-11-08,3,2,0,12,32.6666679382324,15.3333330154419,60,3,2,205,70,1,1,1,0,0,12,1
7508,1313,2011-11-08,4,2,0,7,9.33333301544189,1.66666662693024,18,3,2,205,70,1,1,1,0,0,12,16
7509,1313,2011-11-09,0,3,0,0.333333343267441,5,0.666666686534882,6,3,2,205,70,1,1,1,0,0,12,1
7510,1313,2011-11-09,1,3,0,7.33333349227905,40.3333320617676,12.3333330154419,60,3,2,205,
7511,1313,2011-11-09,2,3,0,23,35.3333320617676,1.66666662693024,60,3,2,205,70,1,1,1,0,0,12,1
7512,1313,2011-11-09,3,3,0,6.66666650772095,33.6666679382324,19.6666660308838,60,3,2,205,
7513,1313,2011-11-09,4,3,0,30,29,1,60,3,2,205,70,1,1,1,0,0,12,16789.15885,21002.09294,9.795398
7514,1321,2011-11-04,0,5,0,27,30,3,60,1,2,205,68,1,1,1,1,0,12,16570.15182,18226.32443,12.83928
7515,1321,2011-11-04,1,5,0,12.6666669845581,37.3333320617676,10,60,3,2,205,68,1,1,1,1,0,12,1
7516,1321,2011-11-04,2,5,0,13.6666669845581,34.3333320617676,12,60,3,2,205,68,1,1,1,1,0,12,1
7517,1321,2011-11-04,3,5,0,5,23,29.3333339691162,57.3333320617676,3,2,205,68,1,1,1,1,0,12,16
7518,1321,2011-11-04,4,5,0,10.3333330154419,0,0,10.3333330154419,3,2,205,68,1,1,1,1,0,12,165
7519,1321,2011-11-05,0,6,1,2,3.33333325386047,0.666666686534882,6,3,2,205,68,1,1,1,1,0,12,16
7520,1321,2011-11-05,1,6,1,38,17.6666660308838,4.33333349227905,60,3,2,205,68,1,1,1,1,0,12,1
7521,1321,2011-11-05,2,6,1,17.6666660308838,18.6666660308838,23.6666660308838,60,3,2,205,
7522,1321,2011-11-05,3,6,1,9.33333301544189,28,22.6666660308838,60,3,2,205,68,1,1,1,1,0,12,1
7523,1321,2011-11-05,4,6,1,23.6666660308838,24,0,47.6666679382324,3,2,205,68,1,1,1,1,0,12,16
7524,1321,2011-11-06,0,7,1,8.66666698455811,16.3333339691162,3,28,3,2,205,68,1,1,1,1,0,12,16
7525,1321,2011-11-06,1,7,1,7.66666650772095,25.3333339691162,27,60,3,2,205,68,1,1,1,1,0,12,1
7526,1321,2011-11-06,2,7,1,22.3333339691162,7.33333349227905,30.3333339691162,60,3,2,205,
7527,1321,2011-11-06,3,7,1,20.3333339691162,17.6666660308838,22,60,3,2,205,68,1,1,1,1,0,12,1
7528,1321,2011-11-06,4,7,1,46.6666679382324,13.3333330154419,0,60,3,2,205,68,1,1,1,1,0,12,16
7529,1321,2011-11-08,0,2,0,8.66666698455811,20.3333339691162,2,31,3,2,205,68,1,1,1,1,0,12,16
7530,1321,2011-11-08,1,2,0,27.6666660308838,18,14.3333330154419,60,3,2,205,68,1,1,1,1,0,12,1
7531,1321,2011-11-08,2,2,0,24,34,2,60,3,2,205,68,1,1,1,1,0,12,16570.15182,18226.32443,12.83928
7532,1321,2011-11-08,3,2,0,32.6666679382324,26.3333339691162,1,60,3,2,205,68,1,1,1,1,0,12,16
7533,1321,2011-11-08,4,2,0,40.6666679382324,17,2.33333325386047,60,3,2,205,68,1,1,1,1,0,12,1
7534,1321,2011-11-09,0,3,0,9.33333301544189,9,3,21.3333339691162,3,2,205,68,1,1,1,1,0,12,165
7535,1321,2011-11-09,1,3,0,17.3333339691162,36,6.66666650772095,60,3,2,205,68,1,1,1,1,0,12,1
7536,1321,2011-11-09,2,3,0,26.6666660308838,29,4.33333349227905,60,3,2,205,68,1,1,1,1,0,12,1
7537,1321,2011-11-09,3,3,0,11.6666669845581,25,23.3333339691162,60,3,2,205,68,1,1,1,1,0,12,1
7538,1321,2011-11-09,4,3,0,15.3333330154419,10,0,25.3333339691162,3,2,205,68,1,1,1,1,0,12,16
7539,1321,2011-11-10,1,4,0,31.6666660308838,18.6666660308838,3,53.3333320617676,3,2,205,6
7540,1321,2011-11-10,2,4,0,5.66666650772095,28.3333339691162,26,60,3,2,205,68,1,1,1,1,0,12,1
7541,1321,2011-11-10,3,4,0,20.3333339691162,23,16.6666660308838,60,3,2,205,68,1,1,1,1,0,12,1
7542,1321,2011-11-10,4,4,0,7,29.3333339691162,1.66666662693024,38,3,2,205,68,1,1,1,1,0,12,16
7543,1323,2011-11-04,0,5,0,28,31,1,60,1,2,205,84,1,1,1,1,0,12,16906.60888,18676.31681,11.96521
7544,1323,2011-11-04,1,5,0,13,39.3333320617676,7.66666650772095,60,3,2,205,84,1,1,1,1,0,12,1
7545,1323,2011-11-04,2,5,0,25,32,3,60,3,2,205,84,1,1,1,1,0,12,16906.60888,18676.31681,11.96521
7546,1323,2011-11-04,3,5,0,22.6666660308838,34,3.33333325386047,60,3,2,205,84,1,1,1,1,0,12,1
7547,1323,2011-11-04,4,5,0,29.3333339691162,28.6666660308838,2,60,3,2,205,84,1,1,1,1,0,12,16
7548,1323,2011-11-05,0,6,1,23.6666660308838,35.3333320617676,1,60,3,2,205,84,1,1,1,1,0,12,16
7549,1323,2011-11-05,1,6,1,10.3333330154419,47,2.66666674613953,60,3,2,205,84,1,1,1,1,0,12,1

7550,1323,2011-11-05,2,6,1,27.6666660308838,30.3333339691162,2,60,3,2,205,84,1,1,1,1,0,12,16
7551,1323,2011-11-05,3,6,1,19.3333339691162,11,29.6666660308838,60,3,2,205,84,1,1,1,1,0,12,1
7552,1323,2011-11-05,4,6,1,19.6666660308838,35.3333320617676,5,60,3,2,205,84,1,1,1,1,0,12,16
7553,1323,2011-11-06,0,7,1,13.3333330154419,17,1.33333337306976,31.6666660308838,3,2,205,
7554,1323,2011-11-06,1,7,1,33,20.6666660308838,6.33333349227905,60,3,2,205,84,1,1,1,1,0,12,1
7555,1323,2011-11-06,2,7,1,24.3333339691162,26.3333339691162,9.33333301544189,60,3,2,205,
7556,1323,2011-11-06,3,7,1,15,35,10,60,3,2,205,84,1,1,1,1,0,12,16906.60888,18676.31681,11.965
7557,1323,2011-11-06,4,7,1,29.3333339691162,27.6666660308838,3,60,3,2,205,84,1,1,1,1,0,12,16
7558,1323,2011-11-07,0,1,0,10.3333330154419,17.6666660308838,0.666666686534882,28.66666
7559,1323,2011-11-07,1,1,0,19.6666660308838,37.6666679382324,2.66666674613953,60,3,2,205,
7560,1323,2011-11-07,2,1,0,29,26.3333339691162,4.66666650772095,60,3,2,205,84,1,1,1,1,0,12,1
7561,1323,2011-11-07,3,1,0,35.3333320617676,18,6.66666650772095,60,3,2,205,84,1,1,1,1,0,12,1
7562,1323,2011-11-07,4,1,0,31,28.3333339691162,0.666666686534882,60,3,2,205,84,1,1,1,1,0,12,
7563,1323,2011-11-08,0,2,0,10,24,2.33333325386047,36.3333320617676,3,2,205,84,1,1,1,1,0,12,1
7564,1323,2011-11-08,1,2,0,21.3333339691162,35.6666679382324,3,60,3,2,205,84,1,1,1,1,0,12,16
7565,1323,2011-11-08,2,2,0,23.6666660308838,31.3333339691162,5,60,3,2,205,84,1,1,1,1,0,12,16
7566,1323,2011-11-08,3,2,0,35.6666679382324,21.3333339691162,3,60,3,2,205,84,1,1,1,1,0,12,16
7567,1323,2011-11-08,4,2,0,31.6666660308838,26.3333339691162,2,60,3,2,205,84,1,1,1,1,0,12,16
7568,1323,2011-11-09,0,3,0,12,25.6666660308838,2.33333325386047,40,3,2,205,84,1,1,1,1,0,12,1
7569,1323,2011-11-09,1,3,0,8,42,10,60,3,2,205,84,1,1,1,1,0,12,16906.60888,18676.31681,11.9652
7570,1323,2011-11-09,2,3,0,21.3333339691162,27.6666660308838,11,60,3,2,205,84,1,1,1,1,0,12,1
7571,1323,2011-11-09,3,3,0,38.6666679382324,15.6666669845581,5.66666650772095,60,3,2,205,
7572,1323,2011-11-09,4,3,0,28.6666660308838,30,1.33333337306976,60,3,2,205,84,1,1,1,1,0,12,1
7573,1323,2011-11-10,0,4,0,10,27,2.66666674613953,39.6666679382324,3,2,205,84,1,1,1,1,0,12,1
7574,1323,2011-11-10,1,4,0,10.3333330154419,37.3333320617676,12.3333330154419,60,3,2,205,
7575,1323,2011-11-10,2,4,0,12.3333330154419,45.6666679382324,2,60,3,2,205,84,1,1,1,1,0,12,16
7576,1323,2011-11-10,3,4,0,26.3333339691162,29,4.66666650772095,60,3,2,205,84,1,1,1,1,0,12,1
7577,1323,2011-11-10,4,4,0,16.3333339691162,35.3333320617676,8.33333301544189,60,3,2,205,
7578,1325,2011-10-27,0,4,0,18,22,20,60,1,2,205,85,0,1,0,1,0,10,16789.15885,21002.09294,9.7953
7579,1325,2011-10-27,1,4,0,29.6666660308838,15.3333330154419,15,60,3,2,205,85,0,1,0,1,0,10,1
7580,1325,2011-10-27,2,4,0,36.3333320617676,22.3333339691162,1.33333337306976,60,3,2,205,
7581,1325,2011-10-27,3,4,0,45,14,1,60,3,2,205,85,0,1,0,1,0,10,16789.15885,21002.09294,9.79539
7582,1325,2011-10-27,4,4,0,42.6666679382324,17.3333339691162,0,60,3,2,205,85,0,1,0,1,0,10,16
7583,1325,2011-10-28,0,5,0,6,2.66666674613953,0,8.66666698455811,3,2,205,85,0,1,0,1,0,10,167
7584,1325,2011-10-28,1,5,0,27,16.3333339691162,16.6666660308838,60,3,2,205,85,0,1,0,1,0,10,1
7585,1325,2011-10-28,2,5,0,33,26.3333339691162,0.666666686534882,60,3,2,205,85,0,1,0,1,0,10,
7586,1325,2011-10-28,3,5,0,39.6666679382324,15.3333330154419,5,60,3,2,205,85,0,1,0,1,0,10,16
7587,1325,2011-10-28,4,5,0,43.3333320617676,10,6.66666650772095,60,3,2,205,85,0,1,0,1,0,10,1
7588,1325,2011-10-29,0,6,1,5,3.33333325386047,0,8.33333301544189,3,2,205,85,0,1,0,1,0,10,167
7589,1325,2011-10-29,1,6,1,26.6666660308838,14.6666669845581,18.6666660308838,60,3,2,205,
7590,1325,2011-10-29,2,6,1,50.6666679382324,9,0.333333343267441,60,3,2,205,85,0,1,0,1,0,10,1
7591,1325,2011-10-29,3,6,1,39.6666679382324,16,4.33333349227905,60,3,2,205,85,0,1,0,1,0,10,1
7592,1325,2011-10-29,4,6,1,41.6666679382324,13,5.33333349227905,60,3,2,205,85,0,1,0,1,0,10,1
7593,1325,2011-10-30,1,7,1,8.33333301544189,9.33333301544189,11.3333330154419,29,3,2,205,
7594,1325,2011-10-30,2,7,1,37.3333320617676,16.3333339691162,6.33333349227905,60,3,2,205,
7595,1325,2011-10-30,3,7,1,35,24,1,60,3,2,205,85,0,1,0,1,0,10,16789.15885,21002.09294,9.79539
7596,1325,2011-10-30,4,7,1,47.3333320617676,12.6666669845581,0,60,3,2,205,85,0,1,0,1,0,10,16
7597,1325,2011-10-31,1,1,0,21.6666660308838,26,1.66666662693024,49.3333320617676,3,2,205,
7598,1325,2011-10-31,2,1,0,37,23,0,60,3,2,205,85,0,1,0,1,0,10,16789.15885,21002.09294,9.79539
7599,1325,2011-10-31,3,1,0,30.3333339691162,13.6666669845581,16,60,3,2,205,85,0,1,0,1,0,10,1

7600,1325,2011-10-31,4,1,0,50,10,0,60,3,2,205,85,0,1,0,1,0,10,16789.15885,21002.09294,9.795398
7601,1325,2011-11-01,1,2,0,27.6666660308838,17,9.33333301544189,54,3,2,205,85,0,1,0,1,0,10,1
7602,1325,2011-11-01,2,2,0,42.6666679382324,13,4.33333349227905,60,3,2,205,85,0,1,0,1,0,10,1
7603,1325,2011-11-01,3,2,0,54.3333320617676,5.66666650772095,0,60,3,2,205,85,0,1,0,1,0,10,16
7604,1325,2011-11-01,4,2,0,44.3333320617676,15.6666669845581,0,60,3,2,205,85,0,1,0,1,0,10,16
7605,1325,2011-11-02,1,3,0,18,18.6666660308838,12.6666669845581,49.3333320617676,3,2,205,
7606,1325,2011-11-02,2,3,0,33.3333320617676,18.6666660308838,8,60,3,2,205,85,0,1,0,1,0,10,16
7607,1325,2011-11-02,3,3,0,31,28,1,60,3,2,205,85,0,1,0,1,0,10,16789.15885,21002.09294,9.795398
7608,1325,2011-11-02,4,3,0,41.3333320617676,18,0.666666686534882,60,3,2,205,85,0,1,0,1,0,10,
7609,1334,2011-11-24,0,4,0,39,20,1,60,1,2,205,80,1,1,1,1,0,4,17192.51095,21541.38367,11.423178
7610,1334,2011-11-24,1,4,0,38,22,0,60,3,2,205,80,1,1,1,1,0,4,17192.51095,21541.38367,11.423178
7611,1334,2011-11-24,2,4,0,42,17.6666660308838,0.333333343267441,60,3,2,205,80,1,1,1,1,0,4,1
7612,1334,2011-11-24,3,4,0,36.3333320617676,23.6666660308838,0,60,3,2,205,80,1,1,1,1,0,4,171
7613,1334,2011-11-24,4,4,0,53.6666679382324,6.33333349227905,0,60,3,2,205,80,1,1,1,1,0,4,171
7614,1334,2011-11-25,0,5,0,33.3333320617676,7,2,42.3333320617676,3,2,205,80,1,1,1,1,0,4,1719
7615,1334,2011-11-25,1,5,0,44.3333320617676,15.6666669845581,0,60,3,2,205,80,1,1,1,1,0,4,171
7616,1334,2011-11-25,2,5,0,41.3333320617676,18.6666660308838,0,60,3,2,205,80,1,1,1,1,0,4,171
7617,1334,2011-11-25,3,5,0,50,10,0,60,3,2,205,80,1,1,1,1,0,4,17192.51095,21541.38367,11.423178
7618,1334,2011-11-25,4,5,0,51.3333320617676,8.33333301544189,0.333333343267441,60,3,2,205
7619,1334,2011-11-26,0,6,1,26.3333339691162,5.33333349227905,1,32.6666679382324,3,2,205,8
7620,1334,2011-11-26,1,6,1,37.3333320617676,22.6666660308838,0,60,3,2,205,80,1,1,1,1,0,4,171
7621,1334,2011-11-26,2,6,1,48.6666679382324,11.3333330154419,0,60,3,2,205,80,1,1,1,1,0,4,171
7622,1334,2011-11-26,3,6,1,31.6666660308838,28.3333339691162,0,60,3,2,205,80,1,1,1,1,0,4,171
7623,1334,2011-11-26,4,6,1,38.6666679382324,21,0.333333343267441,60,3,2,205,80,1,1,1,1,0,4,1
7624,1334,2011-11-27,0,7,1,31.6666660308838,3,1,35.6666679382324,3,2,205,80,1,1,1,1,0,4,1719
7625,1334,2011-11-27,1,7,1,53,6.66666650772095,0.333333343267441,60,3,2,205,80,1,1,1,1,0,4,1
7626,1334,2011-11-27,2,7,1,51,9,0,60,3,2,205,80,1,1,1,1,0,4,17192.51095,21541.38367,11.4231788
7627,1334,2011-11-27,3,7,1,40,20,0,60,3,2,205,80,1,1,1,1,0,4,17192.51095,21541.38367,11.423178
7628,1334,2011-11-27,4,7,1,50,10,0,60,3,2,205,80,1,1,1,1,0,4,17192.51095,21541.38367,11.423178
7629,1334,2011-11-28,0,1,0,36,5.66666650772095,1,42.6666679382324,3,2,205,80,1,1,1,1,0,4,171
7630,1334,2011-11-28,1,1,0,52.6666679382324,7.33333349227905,0,60,3,2,205,80,1,1,1,1,0,4,171
7631,1334,2011-11-28,2,1,0,51,8.66666698455811,0.333333343267441,60,3,2,205,80,1,1,1,1,0,4,1
7632,1334,2011-11-28,3,1,0,37.3333320617676,22.3333339691162,0.333333343267441,60,3,2,205
7633,1334,2011-11-28,4,1,0,40,20,0,60,3,2,205,80,1,1,1,1,0,4,17192.51095,21541.38367,11.423178
7634,1334,2011-11-29,0,2,0,34,6.33333349227905,0,40.3333320617676,3,2,205,80,1,1,1,1,0,4,171
7635,1334,2011-11-29,1,2,0,47.3333320617676,12.6666669845581,0,60,3,2,205,80,1,1,1,1,0,4,171
7636,1334,2011-11-29,2,2,0,55.3333320617676,4.66666650772095,0,60,3,2,205,80,1,1,1,1,0,4,171
7637,1334,2011-11-29,3,2,0,45.6666679382324,14.3333330154419,0,60,3,2,205,80,1,1,1,1,0,4,171
7638,1334,2011-11-29,4,2,0,46,14,0,60,3,2,205,80,1,1,1,1,0,4,17192.51095,21541.38367,11.423178
7639,1334,2011-11-30,0,3,0,10,5.33333349227905,0.333333343267441,15.6666669845581,3,2,205
7640,1334,2011-11-30,1,3,0,25,2,0.333333343267441,27.3333339691162,3,2,205,80,1,1,1,1,0,4,17
7641,1334,2011-11-30,2,3,0,46.6666679382324,13,0.333333343267441,60,3,2,205,80,1,1,1,1,0,4,1
7642,1334,2011-11-30,3,3,0,39,21,0,60,3,2,205,80,1,1,1,1,0,4,17192.51095,21541.38367,11.423178
7643,1334,2011-11-30,4,3,0,48.6666679382324,11.3333330154419,0,60,3,2,205,80,1,1,1,1,0,4,171
7644,1334,2011-12-01,1,4,0,38.3333320617676,13.6666669845581,1,53,3,2,205,80,1,1,1,1,0,4,171
7645,1334,2011-12-01,2,4,0,40.3333320617676,7.33333349227905,12.3333330154419,60,3,2,205,
7646,1334,2011-12-01,3,4,0,28.6666660308838,11,20.3333339691162,60,3,2,205,80,1,1,1,1,0,4,17
7647,1334,2011-12-01,4,4,0,55.3333320617676,4.33333349227905,0.333333343267441,60,3,2,205
7648,1341,2011-08-13,0,6,1,25,22.5,8,55.5,2,2,205,67,1,1,0,1,0,12,15563.65478,15242.70854,12.50
7649,1341,2011-08-13,1,6,1,9.66666698455811,30,20.3333339691162,60,3,2,205,67,1,1,0,1,0,12,1

7650,1341,2011-08-13,2,6,1,15,25.3333339691162,19.6666660308838,60,3,2,205,67,1,1,0,1,0,12,1
7651,1341,2011-08-13,3,6,1,44.6666679382324,12.3333330154419,3,60,3,2,205,67,1,1,0,1,0,12,15
7652,1341,2011-08-13,4,6,1,53,6.66666650772095,0.333333343267441,60,3,2,205,67,1,1,0,1,0,12,
7653,1341,2011-08-14,0,0,1,3.5,35,7,45.5,2,2,205,67,1,1,0,1,0,12,15563.65478,15242.70854,12.505
7654,1341,2011-08-14,1,0,1,8.5,42.6666679382324,8.83333301544189,60,3,2,205,67,1,1,0,1,0,12,1
7655,1341,2011-08-14,2,0,1,35,23.6666660308838,1.33333337306976,60,3,2,205,67,1,1,0,1,0,12,1
7656,1341,2011-08-14,3,0,1,28.3333339691162,25.6666660308838,6,60,3,2,205,67,1,1,0,1,0,12,15
7657,1341,2011-08-14,4,0,1,19.6666660308838,35.3333320617676,5,60,3,2,205,67,1,1,0,1,0,12,15
7658,1341,2011-08-15,0,1,0,7,37,7,51,1,2,205,67,1,1,0,1,0,12,15563.65478,15242.70854,12.505716
7659,1341,2011-08-15,1,1,0,27,33,0,60,3,2,205,67,1,1,0,1,0,12,15563.65478,15242.70854,12.50571
7660,1341,2011-08-15,2,1,0,24.5,31.8333339691162,3.66666674613953,60,3,2,205,67,1,1,0,1,0,12
7661,1341,2011-08-15,3,1,0,18.6000003814697,37.2000007629395,4.19999980926514,60,3,2,205,
7662,1341,2011-08-15,4,1,0,37.6666679382324,22.3333339691162,0,60,3,2,205,67,1,1,0,1,0,12,15
7663,1341,2011-08-16,0,2,0,2.5,25.5,4,32,2,2,205,67,1,1,0,1,0,12,15563.65478,15242.70854,12.505
7664,1341,2011-08-16,1,2,0,8,44.6666679382324,7.33333349227905,60,3,2,205,67,1,1,0,1,0,12,15
7665,1341,2011-08-16,2,2,0,35,20.3333339691162,4.66666650772095,60,3,2,205,67,1,1,0,1,0,12,1
7666,1341,2011-08-16,3,2,0,41,16.25,2.75,60,3,2,205,67,1,1,0,1,0,12,15563.65478,15242.70854,12
7667,1341,2011-08-16,4,2,0,39.6666679382324,19.8333339691162,0.5,60,3,2,205,67,1,1,0,1,0,12,1
7668,1341,2011-08-17,0,3,0,5,37,1,43,1,2,205,67,1,1,0,1,0,12,15563.65478,15242.70854,12.505716
7669,1341,2011-08-17,1,3,0,11.3333330154419,42.6666679382324,6,60,3,2,205,67,1,1,0,1,0,12,15
7670,1341,2011-08-17,2,3,0,25.3333339691162,30,4.66666650772095,60,3,2,205,67,1,1,0,1,0,12,1
7671,1341,2011-08-17,3,3,0,45.3333320617676,14.3333330154419,0.333333343267441,60,3,2,205
7672,1341,2011-08-17,4,3,0,52.3333320617676,7.33333349227905,0.333333343267441,60,3,2,205
7673,1341,2011-08-18,1,4,0,9.33333301544189,36,14.6666669845581,60,3,2,205,67,1,1,0,1,0,12,1
7674,1341,2011-08-18,2,4,0,16.3333339691162,41,2.66666674613953,60,3,2,205,67,1,1,0,1,0,12,1
7675,1341,2011-08-18,3,4,0,28.6666660308838,29.3333339691162,2,60,3,2,205,67,1,1,0,1,0,12,15
7676,1341,2011-08-18,4,4,0,31.3333339691162,28.3333339691162,0.333333343267441,60,3,2,205
7677,1345,2011-08-18,0,4,0,28,23.5,1,52.5,2,2,205,67,0,1,1,1,0,12,15664.41972,14514.16229,12.03
7678,1345,2011-08-18,1,4,0,14.6666669845581,45,0.333333343267441,60,3,2,205,67,0,1,1,1,0,12,
7679,1345,2011-08-19,0,5,0,37,1,0,38,1,2,205,67,0,1,1,1,0,12,15664.41972,14514.16229,12.033112
7680,1345,2011-08-19,1,5,0,18.6666660308838,26.6666660308838,14.6666669845581,60,3,2,205,
7681,1345,2011-08-19,2,5,0,23.5,32.5,0.16666667163372,56.1666679382324,3,2,205,67,0,1,1,1,0,1
7682,1345,2011-08-19,3,5,0,19.3333339691162,39.3333320617676,1.33333337306976,60,3,2,205,
7683,1345,2011-08-19,4,5,0,32.3333320617676,27.6666660308838,0,60,3,2,205,67,0,1,1,1,0,12,15
7684,1345,2011-08-20,0,6,1,18,39,1,58,1,2,205,67,0,1,1,1,0,12,15664.41972,14514.16229,12.03311
7685,1345,2011-08-20,1,6,1,20.3333339691162,37.6666679382324,2,60,3,2,205,67,0,1,1,1,0,12,15
7686,1345,2011-08-20,2,6,1,19,36.3333320617676,4.66666650772095,60,3,2,205,67,0,1,1,1,0,12,1
7687,1345,2011-08-20,3,6,1,14.75,27.5,16.5,58.75,3,2,205,67,0,1,1,1,0,12,15664.41972,14514.1622
7688,1345,2011-08-20,4,6,1,33,19.3333339691162,7.66666650772095,60,3,2,205,67,0,1,1,1,0,12,1
7689,1345,2011-08-21,0,0,1,29,4.5,0.5,34,2,2,205,67,0,1,1,1,0,12,15664.41972,14514.16229,12.033
7690,1345,2011-08-21,1,0,1,18.3333339691162,38.6666679382324,3,60,3,2,205,67,0,1,1,1,0,12,15
7691,1345,2011-08-21,2,0,1,28.3333339691162,31.6666660308838,0,60,3,2,205,67,0,1,1,1,0,12,15
7692,1345,2011-08-21,3,0,1,15,44.3333320617676,0.666666686534882,60,3,2,205,67,0,1,1,1,0,12,
7693,1345,2011-08-21,4,0,1,39,19,2,60,3,2,205,67,0,1,1,1,0,12,15664.41972,14514.16229,12.03311
7694,1345,2011-08-22,0,1,0,31,1,0,32,1,2,205,67,0,1,1,1,0,12,15664.41972,14514.16229,12.033112
7695,1345,2011-08-22,1,1,0,20.6666660308838,37,2.33333325386047,60,3,2,205,67,0,1,1,1,0,12,1
7696,1345,2011-08-22,2,1,0,21.3333339691162,37.6666679382324,1,60,3,2,205,67,0,1,1,1,0,12,15
7697,1345,2011-08-22,3,1,0,15.3333330154419,31.6666660308838,13,60,3,2,205,67,0,1,1,1,0,12,1
7698,1345,2011-08-22,4,1,0,42.3333320617676,17.6666660308838,0,60,3,2,205,67,0,1,1,1,0,12,15
7699,1345,2011-08-23,2,2,0,24,33.3333320617676,2.66666674613953,60,3,2,205,67,0,1,1,1,0,12,1

7700,1345,2011-08-23,3,2,0,3.66666674613953,55.6666679382324,0.666666686534882,60,3,2,205
7701,1345,2011-08-23,4,2,0,43,17,0,60,3,2,205,67,0,1,1,1,0,12,15664.41972,14514.16229,12.03311
7702,1348,2011-08-23,0,2,0,8,51,1,60,1,2,205,67,1,1,0,1,0,11,16211.61748,14435.82793,10.745893
7703,1348,2011-08-23,1,2,0,22.3333339691162,35,2.66666674613953,60,3,2,205,67,1,1,0,1,0,11,1
7704,1348,2011-08-23,2,2,0,36.5,23,0.5,60,2,2,205,67,1,1,0,1,0,11,16211.61748,14435.82793,10.74
7705,1348,2011-08-24,1,3,0,4.66666650772095,35.6666679382324,3.33333325386047,43.6666679
7706,1348,2011-08-24,2,3,0,26.5,29,4.5,60,3,2,205,67,1,1,0,1,0,11,16211.61748,14435.82793,10.74
7707,1348,2011-08-24,3,3,0,15.3333330154419,31.8333339691162,12.8333330154419,60,3,2,205,
7708,1348,2011-08-24,4,3,0,10.3333330154419,44,5.66666650772095,60,3,2,205,67,1,1,0,1,0,11,1
7709,1348,2011-08-25,0,4,0,17,21,1,39,1,2,205,67,1,1,0,1,0,11,16211.61748,14435.82793,10.74589
7710,1348,2011-08-25,1,4,0,25.6000003814697,25.3999996185303,2.79999995231628,53.7999992
7711,1348,2011-08-25,2,4,0,24,25.3333339691162,10.6666669845581,60,3,2,205,67,1,1,0,1,0,11,1
7712,1348,2011-08-25,3,4,0,6.66666650772095,35.6666679382324,17.6666660308838,60,3,2,205,
7713,1348,2011-08-25,4,4,0,8.16666698455811,48,3.83333325386047,60,3,2,205,67,1,1,0,1,0,11,1
7714,1348,2011-08-26,0,5,0,28.3333339691162,31.3333339691162,0.333333343267441,60,3,2,205
7715,1348,2011-08-26,1,5,0,23,35.5,1.5,60,2,2,205,67,1,1,0,1,0,11,16211.61748,14435.82793,10.74
7716,1348,2011-08-26,2,5,0,14.6666669845581,36,9.33333301544189,60,3,2,205,67,1,1,0,1,0,11,1
7717,1348,2011-08-26,3,5,0,1.33333337306976,25,33.6666679382324,60,3,2,205,67,1,1,0,1,0,11,1
7718,1348,2011-08-26,4,5,0,1.33333337306976,50.6666679382324,8,60,3,2,205,67,1,1,0,1,0,11,16
7719,1348,2011-08-27,0,6,1,27.6666660308838,21.6666660308838,0,49.3333320617676,3,2,205,6
7720,1348,2011-08-27,1,6,1,24,30.75,5.25,60,3,2,205,67,1,1,0,1,0,11,16211.61748,14435.82793,10
7721,1348,2011-08-27,2,6,1,21.7999992370605,29.3999996185303,8.80000019073486,60,3,2,205,
7722,1348,2011-08-27,3,6,1,3.66666674613953,47.3333320617676,9,60,3,2,205,67,1,1,0,1,0,11,16
7723,1348,2011-08-27,4,6,1,7.33333349227905,48,4.66666650772095,60,3,2,205,67,1,1,0,1,0,11,1
7724,1348,2011-08-30,0,2,0,59,1,0,60,3,2,205,67,1,1,0,1,0,11,16211.61748,14435.82793,10.745893
7725,1348,2011-08-30,1,2,0,23,30.3333339691162,6.66666650772095,60,3,2,205,67,1,1,0,1,0,11,1
7726,1348,2011-08-30,2,2,0,33,20,7,60,1,2,205,67,1,1,0,1,0,11,16211.61748,14435.82793,10.74589
7727,1348,2011-08-30,3,2,0,17,40.3333320617676,2.66666674613953,60,3,2,205,67,1,1,0,1,0,11,1
7728,1348,2011-08-30,4,2,0,12,43.3333320617676,4.66666650772095,60,3,2,205,67,1,1,0,1,0,11,1
7729,1348,2011-08-31,2,3,0,18,27,15,60,3,2,205,67,1,1,0,1,0,11,16211.61748,14435.82793,10.7458
7730,1348,2011-08-31,3,3,0,10.6666669845581,41,8.33333301544189,60,3,2,205,67,1,1,0,1,0,11,1
7731,1348,2011-08-31,4,3,0,3.66666674613953,44.6666679382324,11.6666669845581,60,3,2,205,
7732,1353,2011-07-08,0,5,0,19,17,2,38,1,2,205,75,0,1,1,1,0,11,16379.73288,15318.15979,12.53706
7733,1353,2011-07-08,1,5,0,19.3333339691162,40.6666679382324,0,60,3,2,205,75,0,1,1,1,0,11,16
7734,1353,2011-07-08,2,5,0,32,26.6666660308838,1.33333337306976,60,3,2,205,75,0,1,1,1,0,11,1
7735,1353,2011-07-08,3,5,0,33.75,19.25,7,60,3,2,205,75,0,1,1,1,0,11,16379.73288,15318.15979,12
7736,1353,2011-07-08,4,5,0,33.6666679382324,26,0.333333343267441,60,3,2,205,75,0,1,1,1,0,11,
7737,1353,2011-07-09,0,6,1,23.5,21.5,0,45,2,2,205,75,0,1,1,1,0,11,16379.73288,15318.15979,12.53
7738,1353,2011-07-09,1,6,1,18.6666660308838,41,0.333333343267441,60,3,2,205,75,0,1,1,1,0,11,
7739,1353,2011-07-09,2,6,1,33,22.6666660308838,4.33333349227905,60,3,2,205,75,0,1,1,1,0,11,1
7740,1353,2011-07-09,3,6,1,16.5,39,4.5,60,3,2,205,75,0,1,1,1,0,11,16379.73288,15318.15979,12.53
7741,1353,2011-07-09,4,6,1,31.7999992370605,27.7999992370605,0.400000005960464,60,3,2,205
7742,1353,2011-07-10,0,0,1,25.6666660308838,25.3333339691162,0,51,3,2,205,75,0,1,1,1,0,11,16
7743,1353,2011-07-10,1,0,1,11.3333330154419,48.6666679382324,0,60,3,2,205,75,0,1,1,1,0,11,16
7744,1353,2011-07-10,2,0,1,24.3333339691162,35,0.666666686534882,60,3,2,205,75,0,1,1,1,0,11,
7745,1353,2011-07-10,3,0,1,13.6666669845581,43.3333320617676,3,60,3,2,205,75,0,1,1,1,0,11,16
7746,1353,2011-07-10,4,0,1,39.25,20.75,0,60,3,2,205,75,0,1,1,1,0,11,16379.73288,15318.15979,12
7747,1353,2011-07-11,0,1,0,34.3333320617676,23.6666660308838,0,58,3,2,205,75,0,1,1,1,0,11,16
7748,1353,2011-07-11,1,1,0,10,50,0,60,3,2,205,75,0,1,1,1,0,11,16379.73288,15318.15979,12.53706
7749,1353,2011-07-11,2,1,0,29.6666660308838,30.3333339691162,0,60,3,2,205,75,0,1,1,1,0,11,16

7750,1353,2011-07-11,3,1,0,31.6666660308838,27,1.33333337306976,60,3,2,205,75,0,1,1,1,0,11,1
7751,1353,2011-07-11,4,1,0,24,34,2,60,3,2,205,75,0,1,1,1,0,11,16379.73288,15318.15979,12.5370€
7752,1353,2011-07-12,0,2,0,27,23,0.5,50.5,2,2,205,75,0,1,1,1,0,11,16379.73288,15318.15979,12.53
7753,1353,2011-07-12,1,2,0,11.6666669845581,48.3333320617676,0,60,3,2,205,75,0,1,1,1,0,11,16
7754,1353,2011-07-12,2,2,0,30.5,29.5,0,60,3,2,205,75,0,1,1,1,0,11,16379.73288,15318.15979,12.53
7755,1353,2011-07-12,3,2,0,9.33333301544189,50.6666679382324,0,60,3,2,205,75,0,1,1,1,0,11,16
7756,1353,2011-07-12,4,2,0,28,32,0,60,3,2,205,75,0,1,1,1,0,11,16379.73288,15318.15979,12.5370€
7757,1353,2011-07-13,3,3,0,20.3333339691162,39.6666679382324,0,60,3,2,205,75,0,1,1,1,0,11,16
7758,1353,2011-07-13,4,3,0,19,40.3333320617676,0.666666686534882,60,3,2,205,75,0,1,1,1,0,11,
7759,1353,2011-08-31,0,3,0,46,14,0,60,1,2,205,75,0,1,1,1,0,11,16379.73288,15318.15979,12.5370€
7760,1353,2011-08-31,1,3,0,11.6666669845581,48.3333320617676,0,60,3,2,205,75,0,1,1,1,0,11,16
7761,1353,2011-08-31,2,3,0,30.6666660308838,29.3333339691162,0,60,3,2,205,75,0,1,1,1,0,11,16
7762,1353,2011-08-31,3,3,0,12,21,27,60,1,2,205,75,0,1,1,1,0,11,16379.73288,15318.15979,12.537(
7763,1371,2011-07-13,0,3,0,38,21,1,60,1,2,205,76,0,1,0,1,0,12,16072.20968,15137.33475,11.4614
7764,1371,2011-07-13,1,3,0,12,35,13,60,3,2,205,76,0,1,0,1,0,12,16072.20968,15137.33475,11.4614
7765,1371,2011-07-13,2,3,0,37.3333320617676,22,0.666666686534882,60,3,2,205,76,0,1,0,1,0,12,
7766,1371,2011-07-13,3,3,0,26.6666660308838,32.6666679382324,0.666666686534882,60,3,2,20!
7767,1371,2011-07-13,4,3,0,42,18,0,60,2,2,205,76,0,1,0,1,0,12,16072.20968,15137.33475,11.4614
7768,1371,2011-07-14,0,4,0,26.5,15.75,0.75,43,3,2,205,76,0,1,0,1,0,12,16072.20968,15137.33475,1
7769,1371,2011-07-14,1,4,0,4.66666650772095,51.6666679382324,3.66666674613953,60,3,2,205,
7770,1371,2011-07-14,2,4,0,40.3333320617676,17.6666660308838,2,60,3,2,205,76,0,1,0,1,0,12,16
7771,1371,2011-07-14,3,4,0,10.3333330154419,47.3333320617676,2.33333325386047,60,3,2,205,
7772,1371,2011-07-14,4,4,0,42.75,17.25,0,60,3,2,205,76,0,1,0,1,0,12,16072.20968,15137.33475,11
7773,1371,2011-07-15,0,5,0,20.6666660308838,23,1,44.6666679382324,3,2,205,76,0,1,0,1,0,12,16
7774,1371,2011-07-15,1,5,0,20.6666660308838,34.3333320617676,5,60,3,2,205,76,0,1,0,1,0,12,16
7775,1371,2011-07-15,2,5,0,45.6666679382324,12,2.33333325386047,60,3,2,205,76,0,1,0,1,0,12,1
7776,1371,2011-07-15,3,5,0,18.3333339691162,38.3333320617676,3.33333325386047,60,3,2,205,
7777,1371,2011-07-15,4,5,0,37,22.3333339691162,0.666666686534882,60,3,2,205,76,0,1,0,1,0,12,
7778,1371,2011-07-16,0,6,1,6,36.5,0.5,43,2,2,205,76,0,1,0,1,0,12,16072.20968,15137.33475,11.461
7779,1371,2011-07-16,1,6,1,13.3333330154419,46,0.666666686534882,60,3,2,205,76,0,1,0,1,0,12,
7780,1371,2011-07-16,2,6,1,31.2000007629395,27.6000003814697,1.20000004768372,60,3,2,205,
7781,1371,2011-07-16,3,6,1,30.3333339691162,26.3333339691162,3.33333325386047,60,3,2,205,
7782,1371,2011-07-16,4,6,1,48.3333320617676,11.6666669845581,0,60,3,2,205,76,0,1,0,1,0,12,16
7783,1371,2011-07-17,0,0,1,13.5,37.5,2,53,2,2,205,76,0,1,0,1,0,12,16072.20968,15137.33475,11.4€
7784,1371,2011-07-17,1,0,1,2.33333325386047,55,2.66666674613953,60,3,2,205,76,0,1,0,1,0,12,1
7785,1371,2011-07-17,2,0,1,30.5,28.25,1.25,60,3,2,205,76,0,1,0,1,0,12,16072.20968,15137.33475,1
7786,1371,2011-07-17,3,0,1,15.5,39.75,4.75,60,3,2,205,76,0,1,0,1,0,12,16072.20968,15137.33475,1
7787,1371,2011-07-17,4,0,1,49,10.3333330154419,0.666666686534882,60,3,2,205,76,0,1,0,1,0,12,
7788,1371,2011-07-18,0,1,0,6,19.5,12.5,38,2,2,205,76,0,1,0,1,0,12,16072.20968,15137.33475,11.4€
7789,1371,2011-07-18,1,1,0,18.6666660308838,34.6666679382324,6.66666650772095,60,3,2,205,
7790,1371,2011-07-18,2,1,0,33.3333320617676,15.6666669845581,2.33333325386047,51.333332(
7791,1371,2011-07-18,3,1,0,18.3333339691162,26,3,47.3333320617676,3,2,205,76,0,1,0,1,0,12,16
7792,1371,2011-07-18,4,1,0,40,20,0,60,3,2,205,76,0,1,0,1,0,12,16072.20968,15137.33475,11.4614
7793,1372,2011-07-18,0,1,0,57,2,1,60,1,2,205,65,1,1,0,1,0,11,16105.48561,15129.5483,11.568306
7794,1372,2011-07-18,1,1,0,27,28.5,4.5,60,2,2,205,65,1,1,0,1,0,11,16105.48561,15129.5483,11.568
7795,1372,2011-07-19,0,2,0,28,16.5,1.5,46,2,2,205,65,1,1,0,1,0,11,16105.48561,15129.5483,11.568
7796,1372,2011-07-19,1,2,0,32.5,22.25,5.25,60,3,2,205,65,1,1,0,1,0,11,16105.48561,15129.5483,11
7797,1372,2011-07-19,2,2,0,31.6000003814697,24.7999992370605,3.59999990463257,60,3,2,205,
7798,1372,2011-07-19,3,2,0,11.6666669845581,37.6666679382324,10.6666669845581,60,3,2,205,
7799,1372,2011-07-19,4,2,0,22.3333339691162,37,0.666666686534882,60,3,2,205,65,1,1,0,1,0,11,

7800,1372,2011-08-01,0,1,0,2,17,1,20,1,2,205,65,1,1,0,1,0,11,16105.48561,15129.5483,11.5683068
7801,1372,2011-08-01,1,1,0,35.6666679382324,23.6666660308838,0.666666686534882,60,3,2,205
7802,1372,2011-08-01,2,1,0,26.6666660308838,32.6666679382324,0.666666686534882,60,3,2,205
7803,1372,2011-08-01,3,1,0,17,38.6666679382324,4.33333349227905,60,3,2,205,65,1,1,0,1,0,11,1
7804,1372,2011-08-01,4,1,0,16,42.6666679382324,1.33333337306976,60,3,2,205,65,1,1,0,1,0,11,1
7805,1372,2011-08-02,0,2,0,0,2,0,2,1,2,205,65,1,1,0,1,0,11,16105.48561,15129.5483,11.5683068,2
7806,1372,2011-08-02,1,2,0,32,23.5,3,58.5,2,2,205,65,1,1,0,1,0,11,16105.48561,15129.5483,11.568
7807,1372,2011-08-02,2,2,0,23.5,28,8.5,60,3,2,205,65,1,1,0,1,0,11,16105.48561,15129.5483,11.568
7808,1372,2011-08-02,3,2,0,21,38,1,60,3,2,205,65,1,1,0,1,0,11,16105.48561,15129.5483,11.568306
7809,1372,2011-08-02,4,2,0,40.3333320617676,19.3333339691162,0.333333343267441,60,3,2,205
7810,1372,2011-08-04,0,4,0,22.5,12,0,34.5,2,2,205,65,1,1,0,1,0,11,16105.48561,15129.5483,11.568
7811,1372,2011-08-04,1,4,0,20.3333339691162,36,3.66666674613953,60,3,2,205,65,1,1,0,1,0,11,1
7812,1372,2011-08-04,2,4,0,36.6666679382324,22.6666660308838,0.666666686534882,60,3,2,205
7813,1372,2011-08-04,3,4,0,32,26.3333339691162,1.66666662693024,60,3,2,205,65,1,1,0,1,0,11,1
7814,1372,2011-08-04,4,4,0,13,42.3333320617676,4.66666650772095,60,3,2,205,65,1,1,0,1,0,11,1
7815,1372,2011-08-05,0,5,0,16.5,25,2,43.5,2,2,205,65,1,1,0,1,0,11,16105.48561,15129.5483,11.568
7816,1372,2011-08-05,1,5,0,18.6666660308838,36,5.33333349227905,60,3,2,205,65,1,1,0,1,0,11,1
7817,1372,2011-08-05,2,5,0,28,27.3333339691162,4.66666650772095,60,3,2,205,65,1,1,0,1,0,11,1
7818,1372,2011-08-05,3,5,0,13.6666669845581,29.6666660308838,16.6666660308838,60,3,2,205,
7819,1372,2011-08-05,4,5,0,24,36,0,60,3,2,205,65,1,1,0,1,0,11,16105.48561,15129.5483,11.568306
7820,1372,2011-08-06,0,6,1,21,16,2,39,1,2,205,65,1,1,0,1,0,11,16105.48561,15129.5483,11.568306
7821,1372,2011-08-06,1,6,1,39.3333320617676,20.6666660308838,0,60,3,2,205,65,1,1,0,1,0,11,16
7822,1372,2011-08-06,2,6,1,29,27.3333339691162,3.66666674613953,60,3,2,205,65,1,1,0,1,0,11,1
7823,1372,2011-08-06,3,6,1,15.5,38.75,5.75,60,3,2,205,65,1,1,0,1,0,11,16105.48561,15129.5483,11
7824,1372,2011-08-06,4,6,1,26.3333339691162,30.6666660308838,3,60,3,2,205,65,1,1,0,1,0,11,16
7825,1372,2011-08-07,3,0,1,29.5,23,7.5,60,2,2,205,65,1,1,0,1,0,11,16105.48561,15129.5483,11.568
7826,1372,2011-08-07,4,0,1,21.3333339691162,35,3.66666674613953,60,3,2,205,65,1,1,0,1,0,11,1
7827,1374,2011-11-26,2,6,1,14.3333330154419,9.33333301544189,0,23.6666660308838,3,2,205,6
7828,1374,2011-11-26,3,6,1,31,28.6666660308838,0.333333343267441,60,3,2,205,68,0,1,0,1,0,11,
7829,1374,2011-11-26,4,6,1,32.3333320617676,27.6666660308838,0,60,3,2,205,68,0,1,0,1,0,11,16
7830,1374,2011-11-27,2,7,1,21,18.3333339691162,2,41.3333320617676,3,2,205,68,0,1,0,1,0,11,16
7831,1374,2011-11-27,3,7,1,28,31,1,60,3,2,205,68,0,1,0,1,0,11,16073.21775,15388.50828,12.21612
7832,1374,2011-11-27,4,7,1,37.6666679382324,21.6666660308838,0.666666686534882,60,3,2,205
7833,1374,2011-11-28,0,1,0,0,0.333333343267441,0,0.333333343267441,3,2,205,68,0,1,0,1,0,11,1
7834,1374,2011-11-28,1,1,0,22.6666660308838,36,1.33333337306976,60,3,2,205,68,0,1,0,1,0,11,1
7835,1374,2011-11-28,2,1,0,41.6666679382324,15.3333330154419,3,60,3,2,205,68,0,1,0,1,0,11,16
7836,1374,2011-11-28,3,1,0,46.6666679382324,9.33333301544189,4,60,3,2,205,68,0,1,0,1,0,11,16
7837,1374,2011-11-28,4,1,0,38.6666679382324,21.3333339691162,0,60,3,2,205,68,0,1,0,1,0,11,16
7838,1374,2011-11-29,0,2,0,14.6666669845581,15,0.333333343267441,30,3,2,205,68,0,1,0,1,0,11,
7839,1374,2011-11-29,1,2,0,38.6666679382324,20.6666660308838,0.666666686534882,60,3,2,205
7840,1374,2011-11-29,2,2,0,23,35.6666679382324,1.33333337306976,60,3,2,205,68,0,1,0,1,0,11,1
7841,1374,2011-11-29,3,2,0,23.6666660308838,32,4.33333349227905,60,3,2,205,68,0,1,0,1,0,11,1
7842,1374,2011-11-29,4,2,0,41,17,2,60,3,2,205,68,0,1,0,1,0,11,16073.21775,15388.50828,12.21612
7843,1374,2011-12-01,1,4,0,2.33333325386047,7,6,15.3333330154419,3,2,205,68,0,1,0,1,0,11,160
7844,1374,2011-12-01,2,4,0,41,10.6666669845581,8.33333301544189,60,3,2,205,68,0,1,0,1,0,11,1
7845,1374,2011-12-01,3,4,0,28,12.3333330154419,19.6666660308838,60,3,2,205,68,0,1,0,1,0,11,1
7846,1374,2011-12-01,4,4,0,54.6666679382324,5,0.333333343267441,60,3,2,205,68,0,1,0,1,0,11,1
7847,1379,2011-11-10,0,4,0,24,20,16,60,1,2,205,68,0,1,0,0,0,12,15783.45732,14399.01356,11.9777
7848,1379,2011-11-10,1,4,0,16.3333339691162,38.3333320617676,5.33333349227905,60,3,2,205,
7849,1379,2011-11-10,2,4,0,12,31.3333339691162,16.6666660308838,60,3,2,205,68,0,1,0,0,0,12,1

7850,1379,2011-11-10,3,4,0,31.6666660308838,18.6666660308838,9.66666698455811,60,3,2,205,
7851,1379,2011-11-10,4,4,0,29.3333339691162,28.3333339691162,2.33333325386047,60,3,2,205,
7852,1379,2011-11-11,1,5,0,35.3333320617676,21.3333339691162,3,59.6666679382324,3,2,205,6
7853,1379,2011-11-11,2,5,0,36.3333320617676,20.6666660308838,3,60,3,2,205,68,0,1,0,0,0,12,15
7854,1379,2011-11-11,3,5,0,18,13,29,60,3,2,205,68,0,1,0,0,0,12,15783.45732,14399.01356,11.977
7855,1379,2011-11-11,4,5,0,36.3333320617676,22.6666660308838,1,60,3,2,205,68,0,1,0,0,0,12,15
7856,1379,2011-11-12,0,6,1,0,6.33333349227905,3,9.33333301544189,3,2,205,68,0,1,0,0,0,12,157
7857,1379,2011-11-12,1,6,1,31.3333339691162,19.6666660308838,9,60,3,2,205,68,0,1,0,0,0,12,15
7858,1379,2011-11-12,2,6,1,11.3333330154419,15.6666669845581,33,60,3,2,205,68,0,1,0,0,0,12,1
7859,1379,2011-11-12,3,6,1,4.66666650772095,15,40.3333320617676,60,3,2,205,68,0,1,0,0,0,12,1
7860,1379,2011-11-12,4,6,1,42.3333320617676,11.3333330154419,6.33333349227905,60,3,2,205,
7861,1379,2011-11-13,0,7,1,2.33333325386047,1.66666662693024,0.333333343267441,4.333333
7862,1379,2011-11-13,1,7,1,16,37,7,60,3,2,205,68,0,1,0,0,0,12,15783.45732,14399.01356,11.9777
7863,1379,2011-11-13,2,7,1,29,25,6,60,3,2,205,68,0,1,0,0,0,12,15783.45732,14399.01356,11.9777
7864,1379,2011-11-13,3,7,1,33.6666679382324,25.3333339691162,1,60,3,2,205,68,0,1,0,0,0,12,15
7865,1379,2011-11-13,4,7,1,27,31,2,60,3,2,205,68,0,1,0,0,0,12,15783.45732,14399.01356,11.9777
7866,1379,2011-11-14,0,1,0,1,4.33333349227905,0.333333343267441,5.66666650772095,3,2,205,
7867,1379,2011-11-14,1,1,0,19.3333339691162,34.6666679382324,6,60,3,2,205,68,0,1,0,0,0,12,15
7868,1379,2011-11-14,2,1,0,28.3333339691162,26.3333339691162,5.33333349227905,60,3,2,205,
7869,1379,2011-11-14,3,1,0,48.3333320617676,8.33333301544189,3.33333325386047,60,3,2,205,
7870,1379,2011-11-14,4,1,0,28.6666660308838,27.6666660308838,3.66666674613953,60,3,2,205,
7871,1379,2011-11-15,1,2,0,19,24.3333339691162,16,59.3333320617676,3,2,205,68,0,1,0,0,0,12,1
7872,1379,2011-11-15,2,2,0,27.6666660308838,18.3333339691162,14,60,3,2,205,68,0,1,0,0,0,12,1
7873,1379,2011-11-15,3,2,0,34.6666679382324,23.6666660308838,1.66666662693024,60,3,2,205,
7874,1379,2011-11-15,4,2,0,28.6666660308838,21.6666660308838,9.66666698455811,60,3,2,205,
7875,1379,2011-11-16,0,3,0,0.333333343267441,2.66666674613953,1,4,3,2,205,68,0,1,0,0,0,12,15
7876,1379,2011-11-16,1,3,0,8.33333301544189,30.3333339691162,21.3333339691162,60,3,2,205,
7877,1379,2011-11-16,2,3,0,20.3333339691162,25.3333339691162,14.3333330154419,60,3,2,205,
7878,1379,2011-11-16,3,3,0,12.6666669845581,37,10.3333330154419,60,3,2,205,68,0,1,0,0,0,12,1
7879,1379,2011-11-16,4,3,0,25.3333339691162,30.3333339691162,4.33333349227905,60,3,2,205,
7880,1383,2011-11-10,0,4,0,18,37,5,60,1,2,205,75,1,1,1,0,0,12,16188.31984,14448.61268,10.5295
7881,1383,2011-11-10,1,4,0,21.3333339691162,29.3333339691162,9.33333301544189,60,3,2,205,
7882,1383,2011-11-10,2,4,0,22.3333339691162,23,14.6666669845581,60,3,2,205,75,1,1,1,0,0,12,1
7883,1383,2011-11-10,3,4,0,13.3333330154419,25,21.6666660308838,60,3,2,205,75,1,1,1,0,0,12,1
7884,1383,2011-11-10,4,4,0,30.3333339691162,29.6666660308838,0,60,3,2,205,75,1,1,1,0,0,12,16
7885,1383,2011-11-11,0,5,0,2,10.6666669845581,0.333333343267441,13,3,2,205,75,1,1,1,0,0,12,1
7886,1383,2011-11-11,1,5,0,10.3333330154419,33.6666679382324,16,60,3,2,205,75,1,1,1,0,0,12,1
7887,1383,2011-11-11,2,5,0,20.3333339691162,38.6666679382324,1,60,3,2,205,75,1,1,1,0,0,12,16
7888,1383,2011-11-11,3,5,0,36,23.6666660308838,0.333333343267441,60,3,2,205,75,1,1,1,0,0,12,
7889,1383,2011-11-11,4,5,0,29.6666660308838,19.6666660308838,0,49.3333320617676,3,2,205,7
7890,1383,2011-11-12,0,6,1,0.333333343267441,0,0,0.333333343267441,3,2,205,75,1,1,1,0,0,12,1
7891,1383,2011-11-12,1,6,1,7,16.3333339691162,18.6666660308838,42,3,2,205,75,1,1,1,0,0,12,16
7892,1383,2011-11-12,2,6,1,21.6666660308838,32.6666679382324,5.66666650772095,60,3,2,205,
7893,1383,2011-11-12,3,6,1,10,42,8,60,3,2,205,75,1,1,1,0,0,12,16188.31984,14448.61268,10.5295
7894,1383,2011-11-12,4,6,1,46.3333320617676,13,0,59.3333320617676,3,2,205,75,1,1,1,0,0,12,16
7895,1383,2011-11-13,0,7,1,5,10,0,15,3,2,205,75,1,1,1,0,0,12,16188.31984,14448.61268,10.52955
7896,1383,2011-11-13,1,7,1,33.3333320617676,26.6666660308838,0,60,3,2,205,75,1,1,1,0,0,12,16
7897,1383,2011-11-13,2,7,1,34.3333320617676,25.6666660308838,0,60,3,2,205,75,1,1,1,0,0,12,16
7898,1383,2011-11-13,3,7,1,20.6666660308838,19,20.3333339691162,60,3,2,205,75,1,1,1,0,0,12,1
7899,1383,2011-11-13,4,7,1,41,15.6666669845581,0,56.6666679382324,3,2,205,75,1,1,1,0,0,12,16

7900,1383,2011-11-14,1,1,0,12.6666669845581,29,17.6666660308838,59.3333320617676,3,2,205,
7901,1383,2011-11-14,2,1,0,2.33333325386047,48,9.66666698455811,60,3,2,205,75,1,1,1,0,0,12,1
7902,1383,2011-11-14,3,1,0,26.3333339691162,17.6666660308838,16,60,3,2,205,75,1,1,1,0,0,12,1
7903,1383,2011-11-14,4,1,0,23.3333339691162,36.6666679382324,0,60,3,2,205,75,1,1,1,0,0,12,16
7904,1383,2011-11-15,0,2,0,3,15,0.333333343267441,18.3333339691162,3,2,205,75,1,1,1,0,0,12,1
7905,1383,2011-11-15,1,2,0,16,38.3333320617676,5.66666650772095,60,3,2,205,75,1,1,1,0,0,12,1
7906,1383,2011-11-15,2,2,0,24.6666660308838,30.6666660308838,4.66666650772095,60,3,2,205,
7907,1383,2011-11-15,3,2,0,28.3333339691162,26.6666660308838,5,60,3,2,205,75,1,1,1,0,0,12,16
7908,1383,2011-11-15,4,2,0,33.6666679382324,26.3333339691162,0,60,3,2,205,75,1,1,1,0,0,12,16
7909,1383,2011-11-16,0,3,0,1.33333337306976,5.66666650772095,0.333333343267441,7.333333
7910,1383,2011-11-16,1,3,0,3.66666674613953,40.6666679382324,15.6666669845581,60,3,2,205,
7911,1383,2011-11-16,2,3,0,4.66666650772095,44,11.3333330154419,60,3,2,205,75,1,1,1,0,0,12,1
7912,1383,2011-11-16,3,3,0,24,33.6666679382324,2.33333325386047,60,3,2,205,75,1,1,1,0,0,12,1
7913,1383,2011-11-16,4,3,0,22.3333339691162,37.6666679382324,0,60,3,2,205,75,1,1,1,0,0,12,16
7914,1395,2011-07-30,0,6,1,2,6,0,8,1,2,205,87,1,1,1,1,0,10,15990.01803,14461.79292,12.5110305
7915,1395,2011-07-30,1,6,1,47.3333320617676,9.33333301544189,3.33333325386047,60,3,2,205,
7916,1395,2011-07-30,2,6,1,30,24.3333339691162,5.66666650772095,60,3,2,205,87,1,1,1,1,0,10,1
7917,1395,2011-07-30,3,6,1,28.3333339691162,30.6666660308838,1,60,3,2,205,87,1,1,1,1,0,10,15
7918,1395,2011-07-30,4,6,1,37.5,11,0,48.5,2,2,205,87,1,1,1,1,0,10,15990.01803,14461.79292,12.51
7919,1395,2011-07-31,1,0,1,22.5,37.5,0,60,2,2,205,87,1,1,1,1,0,10,15990.01803,14461.79292,12.51
7920,1395,2011-07-31,2,0,1,35.3333320617676,22.6666660308838,2,60,3,2,205,87,1,1,1,1,0,10,15
7921,1395,2011-07-31,3,0,1,50.3333320617676,9.66666698455811,0,60,3,2,205,87,1,1,1,1,0,10,15
7922,1395,2011-07-31,4,0,1,49,9.5,0,58.5,2,2,205,87,1,1,1,1,0,10,15990.01803,14461.79292,12.511
7923,1395,2011-08-07,0,0,1,52,7,1,60,1,2,205,87,1,1,1,1,0,10,15990.01803,14461.79292,12.51103
7924,1395,2011-08-07,1,0,1,40.6666679382324,19.3333339691162,0,60,3,2,205,87,1,1,1,1,0,10,15
7925,1395,2011-08-07,2,0,1,23.6666660308838,35.6666679382324,0.666666686534882,60,3,2,20
7926,1395,2011-08-07,3,0,1,8,51,1,60,1,2,205,87,1,1,1,1,0,10,15990.01803,14461.79292,12.51103
7927,1395,2011-08-08,1,1,0,42,12,3.33333325386047,57.3333320617676,3,2,205,87,1,1,1,1,0,10,1
7928,1395,2011-08-08,2,1,0,24,32.6666679382324,3.33333325386047,60,3,2,205,87,1,1,1,1,0,10,1
7929,1395,2011-08-08,3,1,0,30,29.3999996185303,0.600000023841858,60,3,2,205,87,1,1,1,1,0,10,
7930,1395,2011-08-08,4,1,0,44,15,0,59,2,2,205,87,1,1,1,1,0,10,15990.01803,14461.79292,12.5110
7931,1395,2011-08-09,1,2,0,31.75,13.25,0.25,45.25,3,2,205,87,1,1,1,1,0,10,15990.01803,14461.79
7932,1395,2011-08-09,2,2,0,43,8,9,60,3,2,205,87,1,1,1,1,0,10,15990.01803,14461.79292,12.51103
7933,1395,2011-08-09,3,2,0,44.3333320617676,15.6666669845581,0,60,3,2,205,87,1,1,1,1,0,10,15
7934,1395,2011-08-09,4,2,0,48.25,6,0,54.25,3,2,205,87,1,1,1,1,0,10,15990.01803,14461.79292,12.
7935,1397,2011-11-25,0,5,0,10,19,31,60,1,2,205,87,0,1,1,0,0,12,16028.75751,14564.01996,12.028
7936,1397,2011-11-25,1,5,0,7,28.6666660308838,24.3333339691162,60,3,2,205,87,0,1,1,0,0,12,16
7937,1397,2011-11-25,2,5,0,35.3333320617676,15.3333330154419,9.33333301544189,60,3,2,205,
7938,1397,2011-11-25,3,5,0,33.6666679382324,25,1.33333337306976,60,3,2,205,87,0,1,1,0,0,12,1
7939,1397,2011-11-25,4,5,0,44.3333320617676,10.6666669845581,5,60,3,2,205,87,0,1,1,0,0,12,16
7940,1397,2011-11-26,0,6,1,2.33333325386047,6,5,13.3333330154419,3,2,205,87,0,1,1,0,0,12,160
7941,1397,2011-11-26,1,6,1,16.6666660308838,40.3333320617676,3,60,3,2,205,87,0,1,1,0,0,12,16
7942,1397,2011-11-26,2,6,1,39,20.6666660308838,0.333333343267441,60,3,2,205,87,0,1,1,0,0,12,
7943,1397,2011-11-26,3,6,1,33.3333320617676,15.3333330154419,11.3333330154419,60,3,2,205,
7944,1397,2011-11-26,4,6,1,38,21.3333339691162,0.666666686534882,60,3,2,205,87,0,1,1,0,0,12,
7945,1397,2011-11-27,0,7,1,1.66666662693024,6,8.33333301544189,16,3,2,205,87,0,1,1,0,0,12,16
7946,1397,2011-11-27,1,7,1,20.6666660308838,36,3.33333325386047,60,3,2,205,87,0,1,1,0,0,12,1
7947,1397,2011-11-27,2,7,1,28.6666660308838,18.6666660308838,12.6666669845581,60,3,2,205,
7948,1397,2011-11-27,3,7,1,28.6666660308838,18.6666660308838,12.6666669845581,60,3,2,205,
7949,1397,2011-11-27,4,7,1,44.3333320617676,13.3333330154419,2.33333325386047,60,3,2,205,

7950,1397,2011-11-28,0,1,0,1,8.66666698455811,7,16.6666660308838,3,2,205,87,0,1,1,0,0,12,160
7951,1397,2011-11-28,1,1,0,16.3333339691162,41,2.66666674613953,60,3,2,205,87,0,1,1,0,0,12,1
7952,1397,2011-11-28,2,1,0,21,31.6666660308838,7.33333349227905,60,3,2,205,87,0,1,1,0,0,12,1
7953,1397,2011-11-28,3,1,0,44,16,0,60,3,2,205,87,0,1,1,0,0,12,16028.75751,14564.01996,12.0286
7954,1397,2011-11-28,4,1,0,41.6666679382324,15.3333330154419,3,60,3,2,205,87,0,1,1,0,0,12,16
7955,1397,2011-11-29,0,2,0,2,10.3333330154419,6.66666650772095,19,3,2,205,87,0,1,1,0,0,12,16
7956,1397,2011-11-29,1,2,0,16.6666660308838,23,20.3333339691162,60,3,2,205,87,0,1,1,0,0,12,1
7957,1397,2011-11-29,2,2,0,15.3333330154419,21.3333339691162,23.3333339691162,60,3,2,205,
7958,1397,2011-11-29,3,2,0,24,30.6666660308838,5.33333349227905,60,3,2,205,87,0,1,1,0,0,12,1
7959,1397,2011-11-29,4,2,0,39,16.6666660308838,4.33333349227905,60,3,2,205,87,0,1,1,0,0,12,1
7960,1397,2011-11-30,0,3,0,3.66666674613953,6.66666650772095,9.66666698455811,20,3,2,205,
7961,1397,2011-11-30,1,3,0,18.3333339691162,27.6666660308838,14,60,3,2,205,87,0,1,1,0,0,12,1
7962,1397,2011-11-30,2,3,0,25.3333339691162,30,4.66666650772095,60,3,2,205,87,0,1,1,0,0,12,1
7963,1397,2011-11-30,3,3,0,34,13.6666669845581,12.3333330154419,60,3,2,205,87,0,1,1,0,0,12,1
7964,1397,2011-11-30,4,3,0,42.6666679382324,16,1.33333337306976,60,3,2,205,87,0,1,1,0,0,12,1
7965,1397,2011-12-01,0,4,0,1.33333337306976,9.66666698455811,3.66666674613953,14.666666
7966,1397,2011-12-01,1,4,0,12,33.3333320617676,14.6666669845581,60,3,2,205,87,0,1,1,0,0,12,1
7967,1397,2011-12-01,2,4,0,16.3333339691162,20.3333339691162,23.3333339691162,60,3,2,205,
7968,1397,2011-12-01,3,4,0,32,21,7,60,3,2,205,87,0,1,1,0,0,12,16028.75751,14564.01996,12.0286
7969,1397,2011-12-01,4,4,0,39.6666679382324,19.6666660308838,0.666666686534882,60,3,2,20
7970,1398,2011-11-24,0,4,0,39,21,0,60,1,2,205,70,0,0,0,1,0,11,16602.24814,20012.11008,11.3816
7971,1398,2011-11-24,1,4,0,15.6666669845581,31.3333339691162,13,60,3,2,205,70,0,0,0,1,0,11,1
7972,1398,2011-11-24,2,4,0,26,31.6666660308838,2.33333325386047,60,3,2,205,70,0,0,0,1,0,11,1
7973,1398,2011-11-24,3,4,0,42.3333320617676,15.3333330154419,2.33333325386047,60,3,2,205,
7974,1398,2011-11-24,4,4,0,29.6666660308838,28.6666660308838,1.66666662693024,60,3,2,205,
7975,1398,2011-11-25,1,5,0,13.3333330154419,27.6666660308838,12,53,3,2,205,70,0,0,0,1,0,11,1
7976,1398,2011-11-25,2,5,0,23.3333339691162,28.3333339691162,8.33333301544189,60,3,2,205,
7977,1398,2011-11-25,3,5,0,14.3333330154419,29.6666660308838,16,60,3,2,205,70,0,0,0,1,0,11,1
7978,1398,2011-11-25,4,5,0,25.3333339691162,32.3333320617676,2.33333325386047,60,3,2,205,
7979,1398,2011-11-26,1,6,1,14.3333330154419,24,11,49.3333320617676,3,2,205,70,0,0,0,1,0,11,1
7980,1398,2011-11-26,2,6,1,33.6666679382324,24.3333339691162,2,60,3,2,205,70,0,0,0,1,0,11,16
7981,1398,2011-11-26,3,6,1,37.3333320617676,18,4.66666650772095,60,3,2,205,70,0,0,0,1,0,11,1
7982,1398,2011-11-26,4,6,1,34.3333320617676,23.6666660308838,2,60,3,2,205,70,0,0,0,1,0,11,16
7983,1398,2011-11-27,0,7,1,0.333333343267441,4.33333349227905,0,4.66666650772095,3,2,205,
7984,1398,2011-11-27,1,7,1,26.3333339691162,25,8.66666698455811,60,3,2,205,70,0,0,0,1,0,11,1
7985,1398,2011-11-27,2,7,1,25.6666660308838,28.3333339691162,6,60,3,2,205,70,0,0,0,1,0,11,16
7986,1398,2011-11-27,3,7,1,45.3333320617676,7.66666650772095,7,60,3,2,205,70,0,0,0,1,0,11,16
7987,1398,2011-11-27,4,7,1,32.6666679382324,24.6666660308838,2.66666674613953,60,3,2,205,
7988,1398,2011-11-28,0,1,0,14,21.6666660308838,2.66666674613953,38.3333320617676,3,2,205,
7989,1398,2011-11-28,1,1,0,54.3333320617676,5.33333349227905,0.333333343267441,60,3,2,20
7990,1398,2011-11-28,2,1,0,38.6666679382324,18,3.33333325386047,60,3,2,205,70,0,0,0,1,0,11,1
7991,1398,2011-11-28,3,1,0,41,17.3333339691162,1.66666662693024,60,3,2,205,70,0,0,0,1,0,11,1
7992,1398,2011-11-28,4,1,0,25.3333339691162,34,0.666666686534882,60,3,2,205,70,0,0,0,1,0,11,
7993,1398,2011-11-29,0,2,0,13,11.6666669845581,2.33333325386047,27,3,2,205,70,0,0,0,1,0,11,1
7994,1398,2011-11-29,1,2,0,19,29,12,60,3,2,205,70,0,0,0,1,0,11,16602.24814,20012.11008,11.381
7995,1398,2011-11-29,2,2,0,34,24.6666660308838,1.33333337306976,60,3,2,205,70,0,0,0,1,0,11,1
7996,1398,2011-11-29,3,2,0,47.3333320617676,12.6666669845581,0,60,3,2,205,70,0,0,0,1,0,11,16
7997,1398,2011-11-29,4,2,0,18.6666660308838,32.3333320617676,9,60,3,2,205,70,0,0,0,1,0,11,16
7998,1398,2011-11-30,1,3,0,38.3333320617676,20.3333339691162,0.666666686534882,59.33333
7999,1398,2011-11-30,2,3,0,34.6666679382324,25,0.333333343267441,60,3,2,205,70,0,0,0,1,0,11,

8000,1398,2011-11-30,3,3,0,29,26.6666660308838,4.33333349227905,60,3,2,205,70,0,0,0,1,0,11,1
8001,1398,2011-11-30,4,3,0,23,35.6666679382324,1.33333337306976,60,3,2,205,70,0,0,0,1,0,11,1
8002,1398,2011-12-01,1,4,0,27,2.66666674613953,1.66666662693024,31.3333339691162,3,2,205,
8003,1398,2011-12-01,2,4,0,41.3333320617676,7.66666650772095,11,60,3,2,205,70,0,0,0,1,0,11,1
8004,1398,2011-12-01,3,4,0,27.6666660308838,12,20.3333339691162,60,3,2,205,70,0,0,0,1,0,11,1
8005,1398,2011-12-01,4,4,0,54.6666679382324,5,0.333333343267441,60,3,2,205,70,0,0,0,1,0,11,1
8006,1399,2011-11-24,0,4,0,22,35,3,60,1,2,205,69,0,1,0,1,0,12,16300.36756,14631.54202,12.22100
8007,1399,2011-11-24,1,4,0,10.6666669845581,40.6666679382324,8.66666698455811,60,3,2,205,
8008,1399,2011-11-24,2,4,0,13.3333330154419,36,10.6666669845581,60,3,2,205,69,0,1,0,1,0,12,1
8009,1399,2011-11-24,3,4,0,0.666666686534882,50.3333320617676,9,60,3,2,205,69,0,1,0,1,0,12,1
8010,1399,2011-11-24,4,4,0,30.6666660308838,25.6666660308838,3.66666674613953,60,3,2,205,
8011,1399,2011-11-25,0,5,0,30.3333339691162,25.3333339691162,4.33333349227905,60,3,2,205,
8012,1399,2011-11-25,1,5,0,19.3333339691162,35.6666679382324,5,60,3,2,205,69,0,1,0,1,0,12,16
8013,1399,2011-11-25,2,5,0,24.6666660308838,31.6666660308838,3.66666674613953,60,3,2,205,
8014,1399,2011-11-25,3,5,0,13,42.6666679382324,4.33333349227905,60,3,2,205,69,0,1,0,1,0,12,1
8015,1399,2011-11-25,4,5,0,26.6666660308838,29,4.33333349227905,60,3,2,205,69,0,1,0,1,0,12,1
8016,1399,2011-11-26,0,6,1,11.3333330154419,9.66666698455811,1.66666662693024,22.6666660
8017,1399,2011-11-26,1,6,1,4.33333349227905,42.3333320617676,13.3333330154419,60,3,2,205,
8018,1399,2011-11-26,2,6,1,25.6666660308838,30,4.33333349227905,60,3,2,205,69,0,1,0,1,0,12,1
8019,1399,2011-11-26,3,6,1,11,33.3333320617676,15.6666669845581,60,3,2,205,69,0,1,0,1,0,12,1
8020,1399,2011-11-26,4,6,1,19.6666660308838,38,2.33333325386047,60,3,2,205,69,0,1,0,1,0,12,1
8021,1399,2011-11-27,0,7,1,6,15.6666669845581,1,22.6666660308838,3,2,205,69,0,1,0,1,0,12,163
8022,1399,2011-11-27,1,7,1,15.6666669845581,34,10.3333330154419,60,3,2,205,69,0,1,0,1,0,12,1
8023,1399,2011-11-27,2,7,1,5.33333349227905,37.3333320617676,17.3333339691162,60,3,2,205,
8024,1399,2011-11-27,3,7,1,5.33333349227905,36.3333320617676,18.3333339691162,60,3,2,205,
8025,1399,2011-11-27,4,7,1,16,37,7,60,3,2,205,69,0,1,0,1,0,12,16300.36756,14631.54202,12.22100
8026,1399,2011-11-29,0,2,0,9.33333301544189,24.6666660308838,3.66666674613953,37.6666679
8027,1399,2011-11-29,1,2,0,8,23,29,60,3,2,205,69,0,1,0,1,0,12,16300.36756,14631.54202,12.22100
8028,1399,2011-11-29,2,2,0,15,39,6,60,3,2,205,69,0,1,0,1,0,12,16300.36756,14631.54202,12.22100
8029,1399,2011-11-29,3,2,0,2,45.3333320617676,12.6666669845581,60,3,2,205,69,0,1,0,1,0,12,16
8030,1399,2011-11-29,4,2,0,20.3333339691162,33,6.66666650772095,60,3,2,205,69,0,1,0,1,0,12,1
8031,1399,2011-11-30,0,3,0,15,16,7.33333349227905,38.3333320617676,3,2,205,69,0,1,0,1,0,12,1
8032,1399,2011-11-30,1,3,0,27.3333339691162,29.6666660308838,3,60,3,2,205,69,0,1,0,1,0,12,16
8033,1399,2011-11-30,2,3,0,22,21.3333339691162,16.6666660308838,60,3,2,205,69,0,1,0,1,0,12,1
8034,1399,2011-11-30,3,3,0,36.3333320617676,20.3333339691162,3.33333325386047,60,3,2,205,
8035,1399,2011-11-30,4,3,0,30.6666660308838,27.3333339691162,2,60,3,2,205,69,0,1,0,1,0,12,16
8036,1399,2011-12-01,0,4,0,33.3333320617676,21.3333339691162,5.33333349227905,60,3,2,205,
8037,1399,2011-12-01,1,4,0,34,18.3333339691162,7.66666650772095,60,3,2,205,69,0,1,0,1,0,12,1
8038,1399,2011-12-01,2,4,0,39.6666679382324,10,10.3333330154419,60,3,2,205,69,0,1,0,1,0,12,1
8039,1399,2011-12-01,3,4,0,28.3333339691162,11.6666669845581,20,60,3,2,205,69,0,1,0,1,0,12,1
8040,1399,2011-12-01,4,4,0,54.6666679382324,4.66666650772095,0.666666686534882,60,3,2,205
8041,1401,2011-07-29,0,5,0,33,13,0,46,1,2,220,72,1,1,1,1,0,9,18824.71365,22376.08185,11.876068
8042,1401,2011-07-29,1,5,0,20.75,39.25,0,60,3,2,220,72,1,1,1,1,0,9,18824.71365,22376.08185,11.8
8043,1401,2011-07-29,2,5,0,37.6666679382324,16.3333339691162,6,60,3,2,220,72,1,1,1,1,0,9,188
8044,1401,2011-07-29,3,5,0,44.6666679382324,15.3333330154419,0,60,3,2,220,72,1,1,1,1,0,9,188
8045,1401,2011-07-29,4,5,0,35.3333320617676,22.6666660308838,2,60,3,2,220,72,1,1,1,1,0,9,188
8046,1401,2011-07-30,2,6,1,41.3333320617676,18.6666660308838,0,60,3,2,220,72,1,1,1,1,0,9,188
8047,1401,2011-07-30,3,6,1,35.3333320617676,23,1.66666662693024,60,3,2,220,72,1,1,1,1,0,9,18
8048,1401,2011-07-30,4,6,1,47,12.6666669845581,0.333333343267441,60,3,2,220,72,1,1,1,1,0,9,1
8049,1401,2011-07-31,0,0,1,35,23,2,60,1,2,220,72,1,1,1,1,0,9,18824.71365,22376.08185,11.876068

8050,1401,2011-07-31,1,0,1,45,15,0,60,1,2,220,72,1,1,1,1,0,9,18824.71365,22376.08185,11.87606
8051,1401,2011-08-01,0,1,0,22,27,0,49,1,2,220,72,1,1,1,1,0,9,18824.71365,22376.08185,11.87606
8052,1401,2011-08-01,1,1,0,19.25,29.25,11.5,60,3,2,220,72,1,1,1,1,0,9,18824.71365,22376.08185,1
8053,1401,2011-08-01,2,1,0,33.3333320617676,26.3333339691162,0.333333343267441,60,3,2,22
8054,1401,2011-08-01,3,1,0,42.6666679382324,17.3333339691162,0,60,3,2,220,72,1,1,1,1,0,9,188
8055,1401,2011-08-01,4,1,0,43.3333320617676,16.6666660308838,0,60,3,2,220,72,1,1,1,1,0,9,188
8056,1401,2011-08-02,0,2,0,8,12,2,22,1,2,220,72,1,1,1,1,0,9,18824.71365,22376.08185,11.876068
8057,1401,2011-08-02,1,2,0,41.6666679382324,17,1.33333337306976,60,3,2,220,72,1,1,1,1,0,9,18
8058,1401,2011-08-02,2,2,0,21.6666660308838,34,4.33333349227905,60,3,2,220,72,1,1,1,1,0,9,18
8059,1401,2011-08-02,3,2,0,26.3333339691162,31,2.66666674613953,60,3,2,220,72,1,1,1,1,0,9,18
8060,1401,2011-08-02,4,2,0,44.3333320617676,13,2.66666674613953,60,3,2,220,72,1,1,1,1,0,9,18
8061,1401,2011-08-03,0,3,0,25,28,0,53,1,2,220,72,1,1,1,1,0,9,18824.71365,22376.08185,11.87606
8062,1401,2011-08-03,1,3,0,35,25,0,60,3,2,220,72,1,1,1,1,0,9,18824.71365,22376.08185,11.87606
8063,1401,2011-08-03,2,3,0,26.6666660308838,27.6666660308838,5.66666650772095,60,3,2,220,
8064,1401,2011-08-03,3,3,0,29,25.6666660308838,5.33333349227905,60,3,2,220,72,1,1,1,1,0,9,18
8065,1401,2011-08-03,4,3,0,45,14.6666669845581,0.333333343267441,60,3,2,220,72,1,1,1,1,0,9,1
8066,1401,2011-08-04,0,4,0,14,14,0,28,1,2,220,72,1,1,1,1,0,9,18824.71365,22376.08185,11.87606
8067,1401,2011-08-04,1,4,0,25.3333339691162,34.3333320617676,0.333333343267441,60,3,2,22
8068,1401,2011-08-04,2,4,0,41.75,17.5,0.75,60,3,2,220,72,1,1,1,1,0,9,18824.71365,22376.08185,1
8069,1401,2011-08-04,3,4,0,34.6666679382324,25,0.333333343267441,60,3,2,220,72,1,1,1,1,0,9,1
8070,1401,2011-08-04,4,4,0,53.6666679382324,6.33333349227905,0,60,3,2,220,72,1,1,1,1,0,9,188
8071,1401,2011-08-05,0,5,0,18,10,0,28,1,2,220,72,1,1,1,1,0,9,18824.71365,22376.08185,11.87606
8072,1401,2011-08-05,1,5,0,29,22,9,60,2,2,220,72,1,1,1,1,0,9,18824.71365,22376.08185,11.87606
8073,1401,2011-08-05,2,5,0,32,28,0,60,2,2,220,72,1,1,1,1,0,9,18824.71365,22376.08185,11.87606
8074,1401,2011-08-05,3,5,0,42,18,0,60,3,2,220,72,1,1,1,1,0,9,18824.71365,22376.08185,11.87606
8075,1401,2011-08-05,4,5,0,55.3333320617676,4.66666650772095,0,60,3,2,220,72,1,1,1,1,0,9,188
8076,1402,2011-07-29,2,5,0,28,18.5,13.5,60,2,2,220,70,0,1,0,1,0,12,19014.77955,22101.05705,11.
8077,1402,2011-07-29,3,5,0,18.6666660308838,25.3333339691162,16,60,3,2,220,70,0,1,0,1,0,12,1
8078,1402,2011-07-29,4,5,0,51.3333320617676,8.66666698455811,0,60,3,2,220,70,0,1,0,1,0,12,19
8079,1402,2011-07-30,0,6,1,3,44,13,60,1,2,220,70,0,1,0,1,0,12,19014.77955,22101.05705,11.0395
8080,1402,2011-07-30,1,6,1,35,23.3333339691162,1.66666662693024,60,3,2,220,70,0,1,0,1,0,12,1
8081,1402,2011-07-30,2,6,1,19.5,35.5,5,60,2,2,220,70,0,1,0,1,0,12,19014.77955,22101.05705,11.0
8082,1402,2011-07-31,0,0,1,19,10.5,13.5,43,2,2,220,70,0,1,0,1,0,12,19014.77955,22101.05705,11.
8083,1402,2011-07-31,1,0,1,8.66666698455811,42.3333320617676,9,60,3,2,220,70,0,1,0,1,0,12,19
8084,1402,2011-07-31,2,0,1,39.5,17.25,3.25,60,3,2,220,70,0,1,0,1,0,12,19014.77955,22101.05705,1
8085,1402,2011-07-31,3,0,1,17.5,34.75,7.75,60,3,2,220,70,0,1,0,1,0,12,19014.77955,22101.05705,1
8086,1402,2011-07-31,4,0,1,32,25.3333339691162,2.66666674613953,60,3,2,220,70,0,1,0,1,0,12,1
8087,1402,2011-08-01,0,1,0,19,14,14,47,2,2,220,70,0,1,0,1,0,12,19014.77955,22101.05705,11.039
8088,1402,2011-08-01,1,1,0,13.6666669845581,33.3333320617676,13,60,3,2,220,70,0,1,0,1,0,12,1
8089,1402,2011-08-01,2,1,0,27.6666660308838,31.6666660308838,0.666666686534882,60,3,2,22
8090,1402,2011-08-01,3,1,0,22.3333339691162,37,0.666666686534882,60,3,2,220,70,0,1,0,1,0,12,
8091,1402,2011-08-01,4,1,0,44.3333320617676,14,1.66666662693024,60,3,2,220,70,0,1,0,1,0,12,1
8092,1402,2011-08-02,0,2,0,1,10,15,26,1,2,220,70,0,1,0,1,0,12,19014.77955,22101.05705,11.0395
8093,1402,2011-08-02,1,2,0,21.3333339691162,32.6666679382324,6,60,3,2,220,70,0,1,0,1,0,12,19
8094,1402,2011-08-02,2,2,0,37.3333320617676,22.6666660308838,0,60,3,2,220,70,0,1,0,1,0,12,19
8095,1402,2011-08-02,3,2,0,10,46,4,60,3,2,220,70,0,1,0,1,0,12,19014.77955,22101.05705,11.0395
8096,1402,2011-08-02,4,2,0,37.3333320617676,17,1.33333337306976,55.6666679382324,3,2,220,
8097,1402,2011-08-03,0,3,0,29,23.5,4.5,57,2,2,220,70,0,1,0,1,0,12,19014.77955,22101.05705,11.0
8098,1402,2011-08-03,1,3,0,17,15.6666669845581,27.3333339691162,60,3,2,220,70,0,1,0,1,0,12,1
8099,1402,2011-08-03,2,3,0,38.6666679382324,21,0.333333343267441,60,3,2,220,70,0,1,0,1,0,12,

8100,1402,2011-08-03,3,3,0,21.3333339691162,33.6666679382324,5,60,3,2,220,70,0,1,0,1,0,12,19
8101,1402,2011-08-03,4,3,0,48,9.66666698455811,0.666666686534882,58.3333320617676,3,2,22
8102,1402,2011-08-04,0,4,0,22.6666660308838,32,5.33333349227905,60,3,2,220,70,0,1,0,1,0,12,1
8103,1402,2011-08-04,1,4,0,34.3333320617676,20.6666660308838,5,60,3,2,220,70,0,1,0,1,0,12,19
8104,1402,2011-08-04,2,4,0,29,18.3333339691162,12.6666669845581,60,3,2,220,70,0,1,0,1,0,12,1
8105,1402,2011-08-04,3,4,0,15.3333330154419,31,13.6666669845581,60,3,2,220,70,0,1,0,1,0,12,1
8106,1402,2011-08-04,4,4,0,44,14,0,58,2,2,220,70,0,1,0,1,0,12,19014.77955,22101.05705,11.0395
8107,1402,2011-08-05,0,5,0,30,11.5,9,50.5,2,2,220,70,0,1,0,1,0,12,19014.77955,22101.05705,11.0
8108,1402,2011-08-05,1,5,0,22,22.6666660308838,15.3333330154419,60,3,2,220,70,0,1,0,1,0,12,1
8109,1402,2011-08-05,2,5,0,48,12,0,60,1,2,220,70,0,1,0,1,0,12,19014.77955,22101.05705,11.0395
8110,1402,2011-08-05,3,5,0,23,31,6,60,2,2,220,70,0,1,0,1,0,12,19014.77955,22101.05705,11.0395
8111,1402,2011-08-05,4,5,0,23.3333339691162,34.6666679382324,0.666666686534882,58.66666
8112,1403,2011-07-27,0,3,0,35,23,2,60,1,2,220,65,0,1,0,1,0,12,19014.77955,22101.05705,11.0395
8113,1403,2011-07-27,1,3,0,12.3333330154419,13,34.6666679382324,60,3,2,220,65,0,1,0,1,0,12,1
8114,1403,2011-07-27,2,3,0,19,20.3333339691162,20.6666660308838,60,3,2,220,65,0,1,0,1,0,12,1
8115,1403,2011-07-27,3,3,0,34.6666679382324,24.3333339691162,1,60,3,2,220,65,0,1,0,1,0,12,19
8116,1403,2011-07-27,4,3,0,30,29.3333339691162,0.666666686534882,60,3,2,220,65,0,1,0,1,0,12,
8117,1403,2011-07-29,0,5,0,22,36,2,60,1,2,220,65,0,1,0,1,0,12,19014.77955,22101.05705,11.0395
8118,1403,2011-07-29,1,5,0,7,33.6666679382324,19.3333339691162,60,3,2,220,65,0,1,0,1,0,12,19
8119,1403,2011-07-29,2,5,0,21,30.5,8.5,60,2,2,220,65,0,1,0,1,0,12,19014.77955,22101.05705,11.0
8120,1403,2011-07-30,0,6,1,15.5,25,6.5,47,2,2,220,65,0,1,0,1,0,12,19014.77955,22101.05705,11.0
8121,1403,2011-07-30,1,6,1,11.3333330154419,34.3333320617676,14.3333330154419,60,3,2,220,
8122,1403,2011-07-30,2,6,1,17,24.75,18.25,60,3,2,220,65,0,1,0,1,0,12,19014.77955,22101.05705,1
8123,1403,2011-07-30,3,6,1,26.3333339691162,32.6666679382324,1,60,3,2,220,65,0,1,0,1,0,12,19
8124,1403,2011-07-30,4,6,1,26.6666660308838,33,0.333333343267441,60,3,2,220,65,0,1,0,1,0,12,
8125,1403,2011-07-31,0,0,1,18.5,22.5,9.5,50.5,2,2,220,65,0,1,0,1,0,12,19014.77955,22101.05705,1
8126,1403,2011-07-31,1,0,1,10,23,27,60,3,2,220,65,0,1,0,1,0,12,19014.77955,22101.05705,11.039
8127,1403,2011-07-31,2,0,1,17.3333339691162,26.3333339691162,16.3333339691162,60,3,2,220,
8128,1403,2011-07-31,3,0,1,23.3999996185303,28,8.60000038146973,60,3,2,220,65,0,1,0,1,0,12,1
8129,1403,2011-07-31,4,0,1,17.3333339691162,40.6666679382324,2,60,3,2,220,65,0,1,0,1,0,12,19
8130,1403,2011-08-01,0,1,0,18.5,17,10.5,46,2,2,220,65,0,1,0,1,0,12,19014.77955,22101.05705,11.
8131,1403,2011-08-01,1,1,0,18.3333339691162,30.3333339691162,11.3333330154419,60,3,2,220,
8132,1403,2011-08-01,2,1,0,28.3333339691162,23.6666660308838,8,60,3,2,220,65,0,1,0,1,0,12,19
8133,1403,2011-08-01,3,1,0,33.5,25.25,1.25,60,3,2,220,65,0,1,0,1,0,12,19014.77955,22101.05705,1
8134,1403,2011-08-01,4,1,0,31.75,18,10.25,60,3,2,220,65,0,1,0,1,0,12,19014.77955,22101.05705,1
8135,1403,2011-08-02,0,2,0,16.5,30,1,47.5,2,2,220,65,0,1,0,1,0,12,19014.77955,22101.05705,11.0
8136,1403,2011-08-02,1,2,0,27,31,2,60,3,2,220,65,0,1,0,1,0,12,19014.77955,22101.05705,11.0395
8137,1403,2011-08-02,2,2,0,26,31.3333339691162,2.66666674613953,60,3,2,220,65,0,1,0,1,0,12,1
8138,1403,2011-08-02,3,2,0,42,18,0,60,3,2,220,65,0,1,0,1,0,12,19014.77955,22101.05705,11.0395
8139,1403,2011-08-02,4,2,0,31.2000007629395,28.2000007629395,0.600000023841858,60,3,2,22
8140,1403,2011-08-03,0,3,0,20,16.3333339691162,6.66666650772095,43,3,2,220,65,0,1,0,1,0,12,1
8141,1403,2011-08-03,1,3,0,25.3333339691162,25.3333339691162,9.33333301544189,60,3,2,220,
8142,1403,2011-08-03,2,3,0,30.3333339691162,29.6666660308838,0,60,3,2,220,65,0,1,0,1,0,12,19
8143,1403,2011-08-03,3,3,0,24.6666660308838,34.3333320617676,1,60,3,2,220,65,0,1,0,1,0,12,19
8144,1403,2011-08-03,4,3,0,36.6666679382324,23,0.333333343267441,60,3,2,220,65,0,1,0,1,0,12,
8145,1404,2011-07-27,1,3,0,0,2,9,11,1,2,220,78,0,1,0,1,0,8,18371.52759,19538.94135,10.5566045
8146,1404,2011-07-27,2,3,0,21,31,8,60,1,2,220,78,0,1,0,1,0,8,18371.52759,19538.94135,10.55660
8147,1404,2011-07-28,1,4,0,37.3333320617676,19.6666660308838,2.33333325386047,59.333332
8148,1404,2011-07-28,2,4,0,30.6000003814697,25.3999996185303,4,60,3,2,220,78,0,1,0,1,0,8,183
8149,1404,2011-07-28,3,4,0,41.2000007629395,17,1.79999995231628,60,3,2,220,78,0,1,0,1,0,8,18

8150,1404,2011-07-28,4,4,0,55.6666679382324,4.33333349227905,0,60,3,2,220,78,0,1,0,1,0,8,183
8151,1404,2011-07-29,0,5,0,49,11,0,60,1,2,220,78,0,1,0,1,0,8,18371.52759,19538.94135,10.55660
8152,1404,2011-07-29,1,5,0,34.6666679382324,19.6666660308838,5.66666650772095,60,3,2,220,
8153,1404,2011-07-29,2,5,0,37,20.3333339691162,2.66666674613953,60,3,2,220,78,0,1,0,1,0,8,18
8154,1404,2011-07-29,3,5,0,28.25,28,3.75,60,3,2,220,78,0,1,0,1,0,8,18371.52759,19538.94135,10.5
8155,1404,2011-07-29,4,5,0,46.4000015258789,13.3999996185303,0.200000002980232,60,3,2,22
8156,1404,2011-07-30,1,6,1,24.5,24.5,5.25,54.25,3,2,220,78,0,1,0,1,0,8,18371.52759,19538.94135,
8157,1404,2011-07-30,2,6,1,28,23.1666660308838,8.83333301544189,60,3,2,220,78,0,1,0,1,0,8,18
8158,1404,2011-07-30,3,6,1,21.6666660308838,33,5.33333349227905,60,3,2,220,78,0,1,0,1,0,8,18
8159,1404,2011-07-30,4,6,1,41.5,18.5,0,60,3,2,220,78,0,1,0,1,0,8,18371.52759,19538.94135,10.55
8160,1404,2011-07-31,1,0,1,40,13.3333330154419,0.333333343267441,53.6666679382324,3,2,22
8161,1404,2011-07-31,2,0,1,33.6666679382324,22.6666660308838,3.66666674613953,60,3,2,220,
8162,1404,2011-07-31,3,0,1,41.2000007629395,14.3999996185303,4.40000009536743,60,3,2,220,
8163,1404,2011-07-31,4,0,1,44.6666679382324,15.3333330154419,0,60,3,2,220,78,0,1,0,1,0,8,183
8164,1404,2011-08-01,1,1,0,29,19.6666660308838,7.66666650772095,56.3333320617676,3,2,220,
8165,1404,2011-08-01,2,1,0,28.3333339691162,30,1.66666662693024,60,3,2,220,78,0,1,0,1,0,8,18
8166,1404,2011-08-01,3,1,0,11.5,47,1.5,60,3,2,220,78,0,1,0,1,0,8,18371.52759,19538.94135,10.55
8167,1404,2011-08-01,4,1,0,34.5,25.5,0,60,3,2,220,78,0,1,0,1,0,8,18371.52759,19538.94135,10.55
8168,1405,2011-07-29,0,5,0,2,38,5.5,45.5,2,2,220,78,1,0,1,1,0,8,19014.77955,22101.05705,11.039
8169,1405,2011-07-29,1,5,0,6.33333349227905,45.3333320617676,8.33333301544189,60,3,2,220,
8170,1405,2011-07-29,2,5,0,0.333333343267441,32.6666679382324,27,60,3,2,220,78,1,0,1,1,0,8,1
8171,1405,2011-07-29,3,5,0,5.66666650772095,24.6666660308838,29.6666660308838,60,3,2,220,
8172,1405,2011-07-29,4,5,0,29.5,29.75,0.75,60,3,2,220,78,1,0,1,1,0,8,19014.77955,22101.05705,1
8173,1405,2011-07-30,0,6,1,25.6666660308838,18.3333339691162,0.333333343267441,44.33333
8174,1405,2011-07-30,1,6,1,19,38.6666679382324,2.33333325386047,60,3,2,220,78,1,0,1,1,0,8,19
8175,1405,2011-07-30,2,6,1,12,44.6666679382324,3.33333325386047,60,3,2,220,78,1,0,1,1,0,8,19
8176,1405,2011-07-30,3,6,1,14.3333330154419,44.6666679382324,1,60,3,2,220,78,1,0,1,1,0,8,190
8177,1405,2011-07-30,4,6,1,37,17.75,0.75,55.5,3,2,220,78,1,0,1,1,0,8,19014.77955,22101.05705,1
8178,1405,2011-07-31,1,0,1,32.3333320617676,27.3333339691162,0.333333343267441,60,3,2,22
8179,1405,2011-07-31,2,0,1,10,48,2,60,3,2,220,78,1,0,1,1,0,8,19014.77955,22101.05705,11.03955
8180,1405,2011-07-31,3,0,1,18.3333339691162,41.3333320617676,0.333333343267441,60,3,2,22
8181,1405,2011-07-31,4,0,1,27.6666660308838,32,0.333333343267441,60,3,2,220,78,1,0,1,1,0,8,1
8182,1405,2011-08-01,0,1,0,2,6,0,8,1,2,220,78,1,0,1,1,0,8,19014.77955,22101.05705,11.03955857,
8183,1405,2011-08-02,0,2,0,18,39,0,57,1,2,220,78,1,0,1,1,0,8,19014.77955,22101.05705,11.03955
8184,1405,2011-08-02,1,2,0,18.6666660308838,36.5,4.83333349227905,60,3,2,220,78,1,0,1,1,0,8,1
8185,1405,2011-08-02,2,2,0,18.3333339691162,40,1.66666662693024,60,3,2,220,78,1,0,1,1,0,8,19
8186,1405,2011-08-02,3,2,0,11.3333330154419,33.3333320617676,15.3333330154419,60,3,2,220,
8187,1405,2011-08-02,4,2,0,34,25.3333339691162,0.666666686534882,60,3,2,220,78,1,0,1,1,0,8,1
8188,1405,2011-08-03,0,3,0,9.5,20.5,1,31,2,2,220,78,1,0,1,1,0,8,19014.77955,22101.05705,11.039
8189,1405,2011-08-03,1,3,0,8.33333301544189,42.3333320617676,9.33333301544189,60,3,2,220,
8190,1405,2011-08-03,2,3,0,16.6666660308838,41.6666679382324,1.66666662693024,60,3,2,220,
8191,1405,2011-08-03,3,3,0,14.6666669845581,38.3333320617676,7,60,3,2,220,78,1,0,1,1,0,8,190
8192,1405,2011-08-03,4,3,0,21,37.3333320617676,1.66666662693024,60,3,2,220,78,1,0,1,1,0,8,19
8193,1405,2011-08-04,1,4,0,31.6666660308838,25,3,59.6666679382324,3,2,220,78,1,0,1,1,0,8,190
8194,1405,2011-08-04,2,4,0,17.3333339691162,40.3333320617676,2.33333325386047,60,3,2,220,
8195,1405,2011-08-04,3,4,0,19.3333339691162,36.1666679382324,4.5,60,3,2,220,78,1,0,1,1,0,8,1
8196,1405,2011-08-04,4,4,0,21.5,35.25,3.25,60,3,2,220,78,1,0,1,1,0,8,19014.77955,22101.05705,1
8197,1407,2011-07-31,0,0,1,15,1,0,16,1,2,220,75,1,1,1,1,0,12,18824.71365,22376.08185,11.87606
8198,1407,2011-07-31,1,0,1,47,13,0,60,3,2,220,75,1,1,1,1,0,12,18824.71365,22376.08185,11.8760
8199,1407,2011-08-10,0,3,0,60,0,0,60,1,2,220,75,1,1,1,1,0,12,18824.71365,22376.08185,11.87606

8200,1407,2011-08-10,1,3,0,21,38.3333320617676,0.666666686534882,60,3,2,220,75,1,1,1,1,0,12,
8201,1407,2011-08-10,2,3,0,26.6666660308838,32.8333320617676,0.5,60,3,2,220,75,1,1,1,1,0,12,
8202,1407,2011-08-10,3,3,0,30.3333339691162,29.6666660308838,0,60,3,2,220,75,1,1,1,1,0,12,18
8203,1407,2011-08-10,4,3,0,42.6666679382324,17.3333339691162,0,60,3,2,220,75,1,1,1,1,0,12,18
8204,1407,2011-08-11,0,4,0,17,26.5,3.5,47,2,2,220,75,1,1,1,1,0,12,18824.71365,22376.08185,11.8
8205,1407,2011-08-11,1,4,0,36,21.3333339691162,2.66666674613953,60,3,2,220,75,1,1,1,1,0,12,
8206,1407,2011-08-11,2,4,0,44.6666679382324,14.6666669845581,0.666666686534882,60,3,2,22
8207,1407,2011-08-11,3,4,0,34.5,22.5,3,60,3,2,220,75,1,1,1,1,0,12,18824.71365,22376.08185,11.8
8208,1407,2011-08-11,4,4,0,39,21,0,60,3,2,220,75,1,1,1,1,0,12,18824.71365,22376.08185,11.8760
8209,1407,2011-08-12,0,5,0,37.5,12,0.5,50,2,2,220,75,1,1,1,1,0,12,18824.71365,22376.08185,11.8
8210,1407,2011-08-12,1,5,0,25.5,33,1.25,59.75,3,2,220,75,1,1,1,1,0,12,18824.71365,22376.08185,
8211,1407,2011-08-12,2,5,0,38.6666679382324,21,0.333333343267441,60,3,2,220,75,1,1,1,1,0,12,
8212,1407,2011-08-12,3,5,0,34.3333320617676,24.6666660308838,1,60,3,2,220,75,1,1,1,1,0,12,18
8213,1407,2011-08-12,4,5,0,31.5,28.1666660308838,0.16666667163372,59.8333320617676,3,2,22
8214,1407,2011-08-13,1,6,1,35.5,24,0.5,60,2,2,220,75,1,1,1,1,0,12,18824.71365,22376.08185,11.8
8215,1407,2011-08-13,2,6,1,13,45,2,60,3,2,220,75,1,1,1,1,0,12,18824.71365,22376.08185,11.8760
8216,1407,2011-08-13,3,6,1,24.6666660308838,33,2.33333325386047,60,3,2,220,75,1,1,1,1,0,12,
8217,1407,2011-08-13,4,6,1,36.6666679382324,23.3333339691162,0,60,3,2,220,75,1,1,1,1,0,12,18
8218,1408,2011-08-13,1,6,1,16.3333339691162,26.3333339691162,0.333333343267441,43,3,2,22
8219,1408,2011-08-13,2,6,1,38.3333320617676,21.6666660308838,0,60,3,2,220,80,1,1,1,0,0,9,190
8220,1408,2011-08-14,1,0,1,10.5,33,6.5,50,2,2,220,80,1,1,1,0,0,9,19014.77955,22101.05705,11.03
8221,1408,2011-08-14,2,0,1,36.3333320617676,23.3333339691162,0.333333343267441,60,3,2,22
8222,1408,2011-08-14,3,0,1,42.8333320617676,17,0.16666667163372,60,3,2,220,80,1,1,1,0,0,9,19
8223,1408,2011-08-14,4,0,1,44.3333320617676,15.6666669845581,0,60,3,2,220,80,1,1,1,0,0,9,190
8224,1408,2011-08-15,1,1,0,8.60000038146973,31.7999992370605,6.19999980926514,46.599998
8225,1408,2011-08-15,2,1,0,28.8333339691162,28.5,2.66666674613953,60,3,2,220,80,1,1,1,0,0,9,
8226,1408,2011-08-15,3,1,0,28.6666660308838,30.3333339691162,1,60,3,2,220,80,1,1,1,0,0,9,190
8227,1408,2011-08-15,4,1,0,45,15,0,60,3,2,220,80,1,1,1,0,0,9,19014.77955,22101.05705,11.039558
8228,1408,2011-08-16,0,2,0,60,0,0,60,1,2,220,80,1,1,1,0,0,9,19014.77955,22101.05705,11.0395585
8229,1408,2011-08-16,1,2,0,25,34.6666679382324,0.333333343267441,60,3,2,220,80,1,1,1,0,0,9,
8230,1408,2011-08-16,2,2,0,43,17,0,60,3,2,220,80,1,1,1,0,0,9,19014.77955,22101.05705,11.039558
8231,1408,2011-08-16,3,2,0,34.8333320617676,23.8333339691162,1.33333337306976,60,3,2,220,
8232,1408,2011-08-16,4,2,0,46.5,13.5,0,60,3,2,220,80,1,1,1,0,0,9,19014.77955,22101.05705,11.03
8233,1408,2011-08-17,0,3,0,25,30,1,56,1,2,220,80,1,1,1,0,0,9,19014.77955,22101.05705,11.039558
8234,1408,2011-08-17,1,3,0,27.6000003814697,19.7999992370605,1.79999995231628,49.200000
8235,1408,2011-08-17,2,3,0,43.3333320617676,16.3333339691162,0.333333343267441,60,3,2,22
8236,1408,2011-08-17,3,3,0,44,15.6666669845581,0.333333343267441,60,3,2,220,80,1,1,1,0,0,9,
8237,1408,2011-08-17,4,3,0,50,10,0,60,3,2,220,80,1,1,1,0,0,9,19014.77955,22101.05705,11.039558
8238,1408,2012-08-15,2,3,0,23.3333339691162,36.6666679382324,0,60,3,2,220,80,1,1,1,0,0,9,190
8239,1408,2012-08-15,3,3,0,21,37.6666679382324,1.33333337306976,60,3,2,220,80,1,1,1,0,0,9,19
8240,1408,2012-08-15,4,3,0,20,38.6666679382324,1.33333337306976,60,3,2,220,80,1,1,1,0,0,9,19
8241,1410,2012-08-15,0,3,0,53,7,0,60,1,2,220,67,1,1,1,0,0,11,19014.77955,22101.05705,11.039558
8242,1410,2012-08-15,1,3,0,17,35,8,60,3,2,220,67,1,1,1,0,0,11,19014.77955,22101.05705,11.03955
8243,1410,2012-08-15,2,3,0,6.66666650772095,43,10.3333330154419,60,3,2,220,67,1,1,1,0,0,11,
8244,1410,2012-08-16,0,4,0,19,13,0,32,1,2,220,67,1,1,1,0,0,11,19014.77955,22101.05705,11.03955
8245,1410,2012-08-16,1,4,0,36,21,3,60,3,2,220,67,1,1,1,0,0,11,19014.77955,22101.05705,11.03955
8246,1410,2012-08-16,2,4,0,19.3333339691162,28.6666660308838,12,60,3,2,220,67,1,1,1,0,0,11,
8247,1410,2012-08-16,3,4,0,9,38.6666679382324,12.3333330154419,60,3,2,220,67,1,1,1,0,0,11,19
8248,1410,2012-08-16,4,4,0,44.5,14.75,0.75,60,3,2,220,67,1,1,1,0,0,11,19014.77955,22101.05705,
8249,1410,2012-08-17,0,5,0,12,19,0,31,1,2,220,67,1,1,1,0,0,11,19014.77955,22101.05705,11.03955

8250,1410,2012-08-17,1,5,0,31.1666660308838,20.6666660308838,4.16666650772095,56,3,2,220,
8251,1410,2012-08-17,2,5,0,16,37,7,60,3,2,220,67,1,1,1,0,0,11,19014.77955,22101.05705,11.03955
8252,1410,2012-08-17,3,5,0,8.33333301544189,43,8.66666698455811,60,3,2,220,67,1,1,1,0,0,11,1
8253,1410,2012-08-17,4,5,0,22.5,34.75,0.5,57.75,3,2,220,67,1,1,1,0,0,11,19014.77955,22101.05705
8254,1410,2012-08-18,0,6,1,0,10,0,10,1,2,220,67,1,1,1,0,0,11,19014.77955,22101.05705,11.039558
8255,1410,2012-08-18,1,6,1,19,32,9,60,3,2,220,67,1,1,1,0,0,11,19014.77955,22101.05705,11.03955
8256,1410,2012-08-18,2,6,1,21.3333339691162,31.1666660308838,7.5,60,3,2,220,67,1,1,1,0,0,11,1
8257,1410,2012-08-18,3,6,1,39.5,17.75,2.75,60,3,2,220,67,1,1,1,0,0,11,19014.77955,22101.05705,1
8258,1410,2012-08-18,4,6,1,35.3333320617676,23,1.66666662693024,60,3,2,220,67,1,1,1,0,0,11,1
8259,1410,2012-08-19,0,0,1,14,14,1,29,1,2,220,67,1,1,1,0,0,11,19014.77955,22101.05705,11.03955
8260,1410,2012-08-19,1,0,1,21.6666660308838,26,12.3333330154419,60,3,2,220,67,1,1,1,0,0,11,1
8261,1410,2012-08-19,2,0,1,12.3333330154419,27,20.6666660308838,60,3,2,220,67,1,1,1,0,0,11,1
8262,1410,2012-08-19,3,0,1,30.3999996185303,21,8.60000038146973,60,3,2,220,67,1,1,1,0,0,11,1
8263,1410,2012-08-19,4,0,1,36.2000007629395,21,2.79999995231628,60,3,2,220,67,1,1,1,0,0,11,1
8264,1410,2012-08-20,0,1,0,0,2,0,2,1,2,220,67,1,1,1,0,0,11,19014.77955,22101.05705,11.0395585
8265,1410,2012-08-20,1,1,0,13.6666669845581,28,18.3333339691162,60,3,2,220,67,1,1,1,0,0,11,1
8266,1410,2012-08-20,2,1,0,19.3333339691162,35,5.66666650772095,60,3,2,220,67,1,1,1,0,0,11,1
8267,1410,2012-08-20,3,1,0,33,25.3333339691162,1.66666662693024,60,3,2,220,67,1,1,1,0,0,11,1
8268,1410,2012-08-20,4,1,0,32,27.6666660308838,0.333333343267441,60,3,2,220,67,1,1,1,0,0,11,
8269,1410,2012-08-21,4,2,0,33,26,1,60,1,2,220,67,1,1,1,0,0,11,19014.77955,22101.05705,11.03955
8270,1412,2012-08-21,0,2,0,12,16,1,29,1,2,220,88,1,1,1,0,0,11,18472.09613,23165.89682,10.24154
8271,1412,2012-08-21,1,2,0,24.125,29.125,0.625,53.875,3,2,220,88,1,1,1,0,0,11,18472.09613,2316
8272,1412,2012-08-21,2,2,0,31.4285717010498,27.5714282989502,1,60,3,2,220,88,1,1,1,0,0,11,18
8273,1412,2012-08-21,3,2,0,32.5,27.1666660308838,0.333333343267441,60,3,2,220,88,1,1,1,0,0,1
8274,1412,2012-08-21,4,2,0,48.1666679382324,11.5,0.333333343267441,60,3,2,220,88,1,1,1,0,0,1
8275,1412,2012-08-22,0,3,0,59,1,0,60,1,2,220,88,1,1,1,0,0,11,18472.09613,23165.89682,10.241547
8276,1412,2012-08-22,1,3,0,24.3333339691162,30,5.66666650772095,60,3,2,220,88,1,1,1,0,0,11,1
8277,1412,2012-08-22,2,3,0,42.7999992370605,13.1999998092651,4,60,3,2,220,88,1,1,1,0,0,11,18
8278,1412,2012-08-22,3,3,0,36,22.3333339691162,1.66666662693024,60,3,2,220,88,1,1,1,0,0,11,1
8279,1412,2012-08-22,4,3,0,54.3333320617676,5,0.666666686534882,60,3,2,220,88,1,1,1,0,0,11,1
8280,1412,2012-08-23,0,4,0,16,1,0,17,1,2,220,88,1,1,1,0,0,11,18472.09613,23165.89682,10.241547
8281,1412,2012-08-23,1,4,0,33.7999992370605,16.3999996185303,3.40000009536743,53.599998
8282,1412,2012-08-23,2,4,0,39,20.8333339691162,0.16666667163372,60,3,2,220,88,1,1,1,0,0,11,1
8283,1412,2012-08-23,3,4,0,24.1666660308838,31,4.83333349227905,60,3,2,220,88,1,1,1,0,0,11,1
8284,1412,2012-08-23,4,4,0,48.8333320617676,11,0.16666667163372,60,3,2,220,88,1,1,1,0,0,11,1
8285,1412,2012-08-24,3,5,0,40.6666679382324,15.6666669845581,3.66666674613953,60,3,2,220,
8286,1412,2012-08-24,4,5,0,36,22,2,60,3,2,220,88,1,1,1,0,0,11,18472.09613,23165.89682,10.24154
8287,1418,2012-08-24,0,5,0,20.5,15.5,0,36,2,2,220,75,1,1,1,1,0,8,18438.10389,20676.03065,11.145
8288,1418,2012-08-24,1,5,0,7,48.6666679382324,4.33333349227905,60,3,2,220,75,1,1,1,1,0,8,184
8289,1418,2012-08-24,2,5,0,30.3333339691162,26,3.66666674613953,60,3,2,220,75,1,1,1,1,0,8,18
8290,1418,2012-08-24,3,5,0,20,34.3333320617676,5.66666650772095,60,3,2,220,75,1,1,1,1,0,8,18
8291,1418,2012-08-24,4,5,0,39,20.6666660308838,0.333333343267441,60,3,2,220,75,1,1,1,1,0,8,1
8292,1418,2012-08-25,0,6,1,13.25,34.75,1.25,49.25,3,2,220,75,1,1,1,1,0,8,18438.10389,20676.0306
8293,1418,2012-08-25,1,6,1,30.3333339691162,28.3333339691162,1.33333337306976,60,3,2,220,
8294,1418,2012-08-25,2,6,1,36.6666679382324,22,1.33333337306976,60,3,2,220,75,1,1,1,1,0,8,18
8295,1418,2012-08-25,3,6,1,26.3333339691162,32,1.66666662693024,60,3,2,220,75,1,1,1,1,0,8,18
8296,1418,2012-08-25,4,6,1,40.3333320617676,19.3333339691162,0.333333343267441,60,3,2,220
8297,1418,2012-08-26,0,0,1,17,26.6666660308838,1.33333337306976,45,3,2,220,75,1,1,1,1,0,8,18
8298,1418,2012-08-26,1,0,1,14.3333330154419,45,0.666666686534882,60,3,2,220,75,1,1,1,1,0,8,1
8299,1418,2012-08-26,2,0,1,11.6666669845581,47.6666679382324,0.666666686534882,60,3,2,220

8300,1418,2012-08-26,3,0,1,33,24.3333339691162,2.66666674613953,60,3,2,220,75,1,1,1,1,0,8,18
8301,1418,2012-08-26,4,0,1,29,29.6666660308838,1.33333337306976,60,3,2,220,75,1,1,1,1,0,8,18
8302,1418,2012-08-27,0,1,0,27.3333339691162,21,1,49.3333320617676,3,2,220,75,1,1,1,1,0,8,184
8303,1418,2012-08-27,1,1,0,13,43.6666679382324,3.33333325386047,60,3,2,220,75,1,1,1,1,0,8,18
8304,1418,2012-08-27,2,1,0,29.3333339691162,24,6.66666650772095,60,3,2,220,75,1,1,1,1,0,8,18
8305,1418,2012-08-27,3,1,0,23.3333339691162,33.3333320617676,3.33333325386047,60,3,2,220,
8306,1418,2012-08-27,4,1,0,35,23.25,1.75,60,3,2,220,75,1,1,1,1,0,8,18438.10389,20676.03065,11.1
8307,1418,2012-08-28,0,2,0,14.5,34,5,53.5,2,2,220,75,1,1,1,1,0,8,18438.10389,20676.03065,11.145
8308,1418,2012-08-28,1,2,0,10,44,6,60,3,2,220,75,1,1,1,1,0,8,18438.10389,20676.03065,11.145641
8309,1418,2012-08-28,2,2,0,26.3333339691162,30.6666660308838,3,60,3,2,220,75,1,1,1,1,0,8,184
8310,1418,2012-08-28,3,2,0,18,38,4,60,3,2,220,75,1,1,1,1,0,8,18438.10389,20676.03065,11.145641
8311,1418,2012-08-28,4,2,0,33.5999984741211,25.2000007629395,1.20000004768372,60,3,2,220,
8312,1422,2012-08-21,0,2,0,11,30,4,45,1,2,220,81,0,1,1,0,0,9,18322.04021,21196.07101,11.156407
8313,1422,2012-08-21,1,2,0,37.3333320617676,21.6666660308838,1,60,3,2,220,81,0,1,1,0,0,9,183
8314,1422,2012-08-21,2,2,0,34.6666679382324,25.3333339691162,0,60,3,2,220,81,0,1,1,0,0,9,183
8315,1422,2012-08-21,3,2,0,34,26,0,60,3,2,220,81,0,1,1,0,0,9,18322.04021,21196.07101,11.156407
8316,1422,2012-08-21,4,2,0,42.3333320617676,17.6666660308838,0,60,3,2,220,81,0,1,1,0,0,9,183
8317,1422,2012-08-22,0,3,0,13,22,5,40,1,2,220,81,0,1,1,0,0,9,18322.04021,21196.07101,11.156407
8318,1422,2012-08-22,1,3,0,41.3333320617676,16.6666660308838,2,60,3,2,220,81,0,1,1,0,0,9,183
8319,1422,2012-08-22,2,3,0,46.6666679382324,13,0.333333343267441,60,3,2,220,81,0,1,1,0,0,9,1
8320,1422,2012-08-22,3,3,0,41.5,18.25,0.25,60,3,2,220,81,0,1,1,0,0,9,18322.04021,21196.07101,11
8321,1422,2012-08-22,4,3,0,39.7999992370605,20.2000007629395,0,60,3,2,220,81,0,1,1,0,0,9,183
8322,1422,2012-08-23,0,4,0,13,16,4,33,1,2,220,81,0,1,1,0,0,9,18322.04021,21196.07101,11.156407
8323,1422,2012-08-23,1,4,0,31.8333339691162,21.5,3.83333325386047,57.1666679382324,3,2,22
8324,1422,2012-08-23,2,4,0,39,21,0,60,3,2,220,81,0,1,1,0,0,9,18322.04021,21196.07101,11.156407
8325,1422,2012-08-23,3,4,0,33.3333320617676,26.3333339691162,0.333333343267441,60,3,2,22
8326,1422,2012-08-23,4,4,0,32.5,27.25,0.25,60,3,2,220,81,0,1,1,0,0,9,18322.04021,21196.07101,11
8327,1422,2012-08-24,0,5,0,10,17,1,28,1,2,220,81,0,1,1,0,0,9,18322.04021,21196.07101,11.156407
8328,1422,2012-08-24,1,5,0,37,21,2,60,3,2,220,81,0,1,1,0,0,9,18322.04021,21196.07101,11.156407
8329,1422,2012-08-24,2,5,0,39.3333320617676,20.5,0.16666667163372,60,3,2,220,81,0,1,1,0,0,9,1
8330,1422,2012-08-24,3,5,0,40.25,19.75,0,60,3,2,220,81,0,1,1,0,0,9,18322.04021,21196.07101,11.1
8331,1422,2012-08-24,4,5,0,38.3333320617676,21.3333339691162,0.333333343267441,60,3,2,22
8332,1422,2012-08-25,3,6,1,43.5,16.5,0,60,2,2,220,81,0,1,1,0,0,9,18322.04021,21196.07101,11.15
8333,1422,2012-08-25,4,6,1,38.3333320617676,21.6666660308838,0,60,3,2,220,81,0,1,1,0,0,9,183
8334,1422,2012-08-28,0,2,0,23,32,5,60,1,2,220,81,0,1,1,0,0,9,18322.04021,21196.07101,11.156407
8335,1422,2012-08-28,1,2,0,38.6666679382324,20.6666660308838,0.666666686534882,60,3,2,22
8336,1422,2012-08-28,2,2,0,34,21,5,60,3,2,220,81,0,1,1,0,0,9,18322.04021,21196.07101,11.156407
8337,1422,2012-08-28,3,2,0,13,38,9,60,1,2,220,81,0,1,1,0,0,9,18322.04021,21196.07101,11.156407
8338,1422,2012-08-29,0,3,0,12,16,1,29,1,2,220,81,0,1,1,0,0,9,18322.04021,21196.07101,11.156407
8339,1422,2012-08-29,1,3,0,24.6666660308838,32.6666679382324,2.66666674613953,60,3,2,220,
8340,1422,2012-08-29,2,3,0,46,14,0,60,3,2,220,81,0,1,1,0,0,9,18322.04021,21196.07101,11.156407
8341,1422,2012-08-29,3,3,0,32.5,27.5,0,60,2,2,220,81,0,1,1,0,0,9,18322.04021,21196.07101,11.15
8342,1422,2012-08-29,4,3,0,31.6666660308838,28.3333339691162,0,60,3,2,220,81,0,1,1,0,0,9,183
8343,1423,2012-08-20,0,1,0,0,2,0,2,1,2,220,71,1,1,1,0,0,10,18438.10389,20676.03065,11.14564197
8344,1423,2012-08-20,1,1,0,32.3333320617676,27,0.666666686534882,60,3,2,220,71,1,1,1,0,0,10,
8345,1423,2012-08-20,2,1,0,8.66666698455811,46,5.33333349227905,60,3,2,220,71,1,1,1,0,0,10,1
8346,1423,2012-08-20,3,1,0,27.3333339691162,27.3333339691162,5.33333349227905,60,3,2,220,
8347,1423,2012-08-20,4,1,0,34.6666679382324,25.3333339691162,0,60,3,2,220,71,1,1,1,0,0,10,18
8348,1423,2012-08-23,0,4,0,21,36,3,60,1,2,220,71,1,1,1,0,0,10,18438.10389,20676.03065,11.1456
8349,1423,2012-08-23,1,4,0,15.3333330154419,38.6666679382324,6,60,3,2,220,71,1,1,1,0,0,10,18

8350,1423,2012-08-23,2,4,0,12.3333330154419,47.6666679382324,0,60,3,2,220,71,1,1,1,0,0,10,18
8351,1423,2012-08-23,3,4,0,10,43.6666679382324,6.33333349227905,60,3,2,220,71,1,1,1,0,0,10,1
8352,1423,2012-08-23,4,4,0,29.3333339691162,30.6666660308838,0,60,3,2,220,71,1,1,1,0,0,10,18
8353,1423,2012-08-24,0,5,0,20.5,24.5,0.5,45.5,2,2,220,71,1,1,1,0,0,10,18438.10389,20676.03065,1
8354,1423,2012-08-24,1,5,0,12,37,9.5,58.5,2,2,220,71,1,1,1,0,0,10,18438.10389,20676.03065,11.14
8355,1423,2012-08-24,2,5,0,15.6666669845581,39,5.33333349227905,60,3,2,220,71,1,1,1,0,0,10,1
8356,1423,2012-08-24,3,5,0,23.3333339691162,32,4.66666650772095,60,3,2,220,71,1,1,1,0,0,10,1
8357,1423,2012-08-24,4,5,0,33.3333320617676,26.3333339691162,0.333333343267441,60,3,2,220
8358,1423,2012-08-25,0,6,1,25,31,4,60,2,2,220,71,1,1,1,0,0,10,18438.10389,20676.03065,11.14564
8359,1423,2012-08-25,1,6,1,18.3333339691162,37,4.66666650772095,60,3,2,220,71,1,1,1,0,0,10,1
8360,1423,2012-08-25,2,6,1,13.8333330154419,41.1666679382324,5,60,3,2,220,71,1,1,1,0,0,10,18
8361,1423,2012-08-25,3,6,1,21.1666660308838,35,3.83333325386047,60,3,2,220,71,1,1,1,0,0,10,1
8362,1423,2012-08-25,4,6,1,31.5,26,2.5,60,3,2,220,71,1,1,1,0,0,10,18438.10389,20676.03065,11.14
8363,1423,2012-08-26,0,0,1,19,24,0,43,2,2,220,71,1,1,1,0,0,10,18438.10389,20676.03065,11.14564
8364,1423,2012-08-26,1,0,1,12.6666669845581,40.3333320617676,7,60,3,2,220,71,1,1,1,0,0,10,18
8365,1423,2012-08-26,2,0,1,38,21,1,60,3,2,220,71,1,1,1,0,0,10,18438.10389,20676.03065,11.14564
8366,1423,2012-08-26,3,0,1,31.6666660308838,26.3333339691162,2,60,3,2,220,71,1,1,1,0,0,10,18
8367,1423,2012-08-26,4,0,1,42.5999984741211,15.6000003814697,1.79999995231628,60,3,2,220,
8368,1423,2012-08-27,0,1,0,23.25,26,0.5,49.75,3,2,220,71,1,1,1,0,0,10,18438.10389,20676.03065,1
8369,1423,2012-08-27,1,1,0,15,41.3333320617676,3.66666674613953,60,3,2,220,71,1,1,1,0,0,10,1
8370,1423,2012-08-27,2,1,0,10.6666669845581,38.6666679382324,10.6666669845581,60,3,2,220,
8371,1423,2012-08-27,3,1,0,19.6666660308838,29,11.3333330154419,60,3,2,220,71,1,1,1,0,0,10,1
8372,1423,2012-08-27,4,1,0,33,26.6666660308838,0.333333343267441,60,3,2,220,71,1,1,1,0,0,10,
8373,1430,2012-08-17,1,5,0,23.5,12.5,0,36,2,2,220,76,0,1,0,1,0,10,18517.61472,22288.67712,10.04
8374,1430,2012-08-17,2,5,0,37,23,0,60,3,2,220,76,0,1,0,1,0,10,18517.61472,22288.67712,10.04851
8375,1430,2012-08-17,3,5,0,41,19,0,60,3,2,220,76,0,1,0,1,0,10,18517.61472,22288.67712,10.04851
8376,1430,2012-08-17,4,5,0,41.5999984741211,17.7999992370605,0.600000023841858,60,3,2,220
8377,1430,2012-08-18,1,6,1,31.75,15.5,2,49.25,3,2,220,76,0,1,0,1,0,10,18517.61472,22288.67712,1
8378,1430,2012-08-18,2,6,1,49,11,0,60,3,2,220,76,0,1,0,1,0,10,18517.61472,22288.67712,10.04851
8379,1430,2012-08-18,3,6,1,27,32.6666679382324,0.333333343267441,60,3,2,220,76,0,1,0,1,0,10,
8380,1430,2012-08-18,4,6,1,45.6666679382324,14.3333330154419,0,60,3,2,220,76,0,1,0,1,0,10,18
8381,1430,2012-08-19,1,0,1,40,20,0,60,1,2,220,76,0,1,0,1,0,10,18517.61472,22288.67712,10.04851
8382,1430,2012-08-19,2,0,1,42.6666679382324,17.3333339691162,0,60,3,2,220,76,0,1,0,1,0,10,18
8383,1430,2012-08-19,3,0,1,44,15.3333330154419,0.666666686534882,60,3,2,220,76,0,1,0,1,0,10,
8384,1430,2012-08-19,4,0,1,39,21,0,60,3,2,220,76,0,1,0,1,0,10,18517.61472,22288.67712,10.04851
8385,1430,2012-08-26,0,0,1,60,0,0,60,1,2,220,76,0,1,0,1,0,10,18517.61472,22288.67712,10.048512
8386,1430,2012-08-26,1,0,1,46.3333320617676,12.3333330154419,1.33333337306976,60,3,2,220,
8387,1430,2012-08-26,2,0,1,44.3333320617676,15.3333330154419,0.333333343267441,60,3,2,220
8388,1430,2012-08-26,3,0,1,47.3333320617676,12.6666669845581,0,60,3,2,220,76,0,1,0,1,0,10,18
8389,1430,2012-08-26,4,0,1,38,21,1,60,2,2,220,76,0,1,0,1,0,10,18517.61472,22288.67712,10.04851
8390,1430,2012-08-27,0,1,0,0,13,1,14,1,2,220,76,0,1,0,1,0,10,18517.61472,22288.67712,10.048512
8391,1430,2012-08-27,1,1,0,21.6666660308838,31,7.33333349227905,60,3,2,220,76,0,1,0,1,0,10,1
8392,1430,2012-08-27,2,1,0,44.3333320617676,15.6666669845581,0,60,3,2,220,76,0,1,0,1,0,10,18
8393,1430,2012-08-27,3,1,0,30.6666660308838,29.3333339691162,0,60,3,2,220,76,0,1,0,1,0,10,18
8394,1430,2012-08-27,4,1,0,40.5,19,0.5,60,2,2,220,76,0,1,0,1,0,10,18517.61472,22288.67712,10.04
8395,1430,2012-08-30,1,4,0,33,11.3333330154419,1.66666662693024,46,3,2,220,76,0,1,0,1,0,10,1
8396,1430,2012-08-30,2,4,0,40.6666679382324,17.3333339691162,2,60,3,2,220,76,0,1,0,1,0,10,18
8397,1430,2012-08-30,3,4,0,48.6666679382324,9.66666698455811,1.66666662693024,60,3,2,220,
8398,1430,2012-08-30,4,4,0,35.3333320617676,24.3333339691162,0.333333343267441,60,3,2,220
8399,1430,2012-08-31,1,5,0,34.3333320617676,16.3333339691162,3.33333325386047,54,3,2,220,

8400,1430,2012-08-31,2,5,0,46.6666679382324,12,1.33333337306976,60,3,2,220,76,0,1,0,1,0,10,1
8401,1430,2012-08-31,3,5,0,53.6666679382324,5.33333349227905,1,60,3,2,220,76,0,1,0,1,0,10,18
8402,1430,2012-08-31,4,5,0,47.3333320617676,12,0.666666686534882,60,3,2,220,76,0,1,0,1,0,10,
8403,1433,2012-08-19,0,0,1,51,6,3,60,1,2,220,77,1,1,0,1,0,7,18498.08569,19605.36342,10.771194
8404,1433,2012-08-19,1,0,1,38,19.5,2.5,60,2,2,220,77,1,1,0,1,0,7,18498.08569,19605.36342,10.771
8405,1433,2012-08-20,0,1,0,13,6,0,19,1,2,220,77,1,1,0,1,0,7,18498.08569,19605.36342,10.771194
8406,1433,2012-08-20,1,1,0,32,23,5,60,1,2,220,77,1,1,0,1,0,7,18498.08569,19605.36342,10.771194
8407,1433,2012-08-20,2,1,0,38.6666679382324,16.6666660308838,4.66666650772095,60,3,2,220,
8408,1433,2012-08-20,3,1,0,40,16,4,60,3,2,220,77,1,1,0,1,0,7,18498.08569,19605.36342,10.771194
8409,1433,2012-08-20,4,1,0,43.3333320617676,16.6666660308838,0,60,3,2,220,77,1,1,0,1,0,7,184
8410,1433,2012-08-21,0,2,0,21,39,0,60,1,2,220,77,1,1,0,1,0,7,18498.08569,19605.36342,10.771194
8411,1433,2012-08-21,1,2,0,52,7.66666650772095,0.333333343267441,60,3,2,220,77,1,1,0,1,0,7,1
8412,1433,2012-08-21,2,2,0,47,13,0,60,3,2,220,77,1,1,0,1,0,7,18498.08569,19605.36342,10.771194
8413,1433,2012-08-21,3,2,0,42,18,0,60,3,2,220,77,1,1,0,1,0,7,18498.08569,19605.36342,10.771194
8414,1433,2012-08-21,4,2,0,43.5,16.5,0,60,2,2,220,77,1,1,0,1,0,7,18498.08569,19605.36342,10.771
8415,1433,2012-08-23,0,4,0,10,9,1,20,1,2,220,77,1,1,0,1,0,7,18498.08569,19605.36342,10.771194
8416,1433,2012-08-23,1,4,0,32,28,0,60,3,2,220,77,1,1,0,1,0,7,18498.08569,19605.36342,10.771194
8417,1433,2012-08-23,2,4,0,37.3333320617676,20,2.66666674613953,60,3,2,220,77,1,1,0,1,0,7,18
8418,1433,2012-08-23,3,4,0,46.6666679382324,13.3333330154419,0,60,3,2,220,77,1,1,0,1,0,7,184
8419,1433,2012-08-23,4,4,0,21.5,11.5,2,35,2,2,220,77,1,1,0,1,0,7,18498.08569,19605.36342,10.771
8420,1433,2012-08-24,0,5,0,21,26,1,48,1,2,220,77,1,1,0,1,0,7,18498.08569,19605.36342,10.771194
8421,1433,2012-08-24,1,5,0,45.6666679382324,14.3333330154419,0,60,3,2,220,77,1,1,0,1,0,7,184
8422,1433,2012-08-24,2,5,0,38.3333320617676,21.6666660308838,0,60,3,2,220,77,1,1,0,1,0,7,184
8423,1433,2012-08-24,3,5,0,37.6666679382324,20,2.33333325386047,60,3,2,220,77,1,1,0,1,0,7,18
8424,1433,2012-08-24,4,5,0,39.5,20.5,0,60,2,2,220,77,1,1,0,1,0,7,18498.08569,19605.36342,10.771
8425,1433,2012-08-25,0,6,1,27.5,27.5,0.5,55.5,2,2,220,77,1,1,0,1,0,7,18498.08569,19605.36342,10
8426,1433,2012-08-25,1,6,1,42,18,0,60,3,2,220,77,1,1,0,1,0,7,18498.08569,19605.36342,10.771194
8427,1433,2012-08-25,2,6,1,32.3333320617676,27,0.666666686534882,60,3,2,220,77,1,1,0,1,0,7,1
8428,1433,2012-08-25,3,6,1,47,13,0,60,1,2,220,77,1,1,0,1,0,7,18498.08569,19605.36342,10.771194
8429,1433,2012-08-25,4,6,1,50.6666679382324,9.33333301544189,0,60,3,2,220,77,1,1,0,1,0,7,184
8430,1433,2012-08-27,0,1,0,17,22,2,41,1,2,220,77,1,1,0,1,0,7,18498.08569,19605.36342,10.771194
8431,1433,2012-08-27,1,1,0,42.3333320617676,17.6666660308838,0,60,3,2,220,77,1,1,0,1,0,7,184
8432,1433,2012-08-27,2,1,0,36.3333320617676,21.6666660308838,2,60,3,2,220,77,1,1,0,1,0,7,184
8433,1433,2012-08-27,3,1,0,36,23.5,0.5,60,2,2,220,77,1,1,0,1,0,7,18498.08569,19605.36342,10.771
8434,1433,2012-08-27,4,1,0,42.3333320617676,17.6666660308838,0,60,3,2,220,77,1,1,0,1,0,7,184
8435,1433,2012-08-28,3,2,0,37,13.6666669845581,9.33333301544189,60,3,2,220,77,1,1,0,1,0,7,18
8436,1433,2012-08-28,4,2,0,21,39,0,60,3,2,220,77,1,1,0,1,0,7,18498.08569,19605.36342,10.771194
8437,1434,2012-08-02,0,4,0,30.3333339691162,23.6666660308838,6,60,3,2,220,72,0,1,0,1,0,12,18
8438,1434,2012-08-02,1,4,0,12.3333330154419,43.6666679382324,4,60,3,2,220,72,0,1,0,1,0,12,18
8439,1434,2012-08-02,2,4,0,23.6666660308838,32.6666679382324,3.66666674613953,60,3,2,220,
8440,1434,2012-08-02,3,4,0,16,40.3333320617676,3.66666674613953,60,3,2,220,72,0,1,0,1,0,12,1
8441,1434,2012-08-02,4,4,0,43.6666679382324,16.3333339691162,0,60,3,2,220,72,0,1,0,1,0,12,18
8442,1434,2012-08-03,0,5,0,14,32,1,47,1,2,220,72,0,1,0,1,0,12,18498.08569,19605.36342,10.77119
8443,1434,2012-08-03,1,5,0,9.66666698455811,47.8333320617676,2.5,60,3,2,220,72,0,1,0,1,0,12,1
8444,1434,2012-08-03,2,5,0,30.6666660308838,28.3333339691162,1,60,3,2,220,72,0,1,0,1,0,12,18
8445,1434,2012-08-03,3,5,0,18.3333339691162,36.3333320617676,5.33333349227905,60,3,2,220,
8446,1434,2012-08-03,4,5,0,41.6666679382324,18,0.333333343267441,60,3,2,220,72,0,1,0,1,0,12,
8447,1434,2012-08-04,0,6,1,13.5,24.5,6.5,44.5,2,2,220,72,0,1,0,1,0,12,18498.08569,19605.36342,1
8448,1434,2012-08-04,1,6,1,10,46.3333320617676,3.66666674613953,60,3,2,220,72,0,1,0,1,0,12,1
8449,1434,2012-08-04,2,6,1,14.1999998092651,40.7999992370605,5,60,3,2,220,72,0,1,0,1,0,12,18

8450,1434,2012-08-04,3,6,1,29.3333339691162,30.6666660308838,0,60,3,2,220,72,0,1,0,1,0,12,18
8451,1434,2012-08-04,4,6,1,16.3333339691162,41,2.66666674613953,60,3,2,220,72,0,1,0,1,0,12,1
8452,1434,2012-08-05,2,0,1,11,44,5,60,1,2,220,72,0,1,0,1,0,12,18498.08569,19605.36342,10.77119
8453,1434,2012-08-05,3,0,1,18.6666660308838,35.6666679382324,5.66666650772095,60,3,2,220,
8454,1434,2012-08-05,4,0,1,25,7,1,33,2,2,220,72,0,1,0,1,0,12,18498.08569,19605.36342,10.771194
8455,1434,2012-08-28,0,2,0,25,32,3,60,1,2,220,72,0,1,0,1,0,12,18498.08569,19605.36342,10.77119
8456,1434,2012-08-28,1,2,0,18.6666660308838,40,1.33333337306976,60,3,2,220,72,0,1,0,1,0,12,1
8457,1434,2012-08-28,2,2,0,19,37.5,3.5,60,2,2,220,72,0,1,0,1,0,12,18498.08569,19605.36342,10.77
8458,1434,2012-08-29,0,3,0,6.5,31.5,1.5,39.5,2,2,220,72,0,1,0,1,0,12,18498.08569,19605.36342,10
8459,1434,2012-08-29,1,3,0,12.6666669845581,46.3333320617676,1,60,3,2,220,72,0,1,0,1,0,12,18
8460,1434,2012-08-29,2,3,0,18,40,2,60,1,2,220,72,0,1,0,1,0,12,18498.08569,19605.36342,10.77119
8461,1434,2012-08-29,3,3,0,30.3333339691162,28.3333339691162,1.33333337306976,60,3,2,220,
8462,1434,2012-08-29,4,3,0,29,30.3333339691162,0.666666686534882,60,3,2,220,72,0,1,0,1,0,12,
8463,1434,2012-08-30,0,4,0,8,23.5,2,33.5,2,2,220,72,0,1,0,1,0,12,18498.08569,19605.36342,10.771
8464,1434,2012-08-30,1,4,0,12,44,4,60,1,2,220,72,0,1,0,1,0,12,18498.08569,19605.36342,10.77119
8465,1434,2012-08-30,2,4,0,34,25.6666660308838,0.333333343267441,60,3,2,220,72,0,1,0,1,0,12,
8466,1434,2012-08-30,3,4,0,18.6666660308838,41,0.333333343267441,60,3,2,220,72,0,1,0,1,0,12,
8467,1434,2012-08-30,4,4,0,43.3333320617676,16.6666660308838,0,60,3,2,220,72,0,1,0,1,0,12,18
8468,1434,2012-08-31,0,5,0,15,32,0,47,1,2,220,72,0,1,0,1,0,12,18498.08569,19605.36342,10.77119
8469,1434,2012-08-31,1,5,0,4.5,54.5,1,60,2,2,220,72,0,1,0,1,0,12,18498.08569,19605.36342,10.771
8470,1434,2012-08-31,2,5,0,25.6666660308838,31,3.33333325386047,60,3,2,220,72,0,1,0,1,0,12,1
8471,1434,2012-08-31,3,5,0,25,34.6666679382324,0.333333343267441,60,3,2,220,72,0,1,0,1,0,12,
8472,1434,2012-08-31,4,5,0,37,23,0,60,3,2,220,72,0,1,0,1,0,12,18498.08569,19605.36342,10.77119
8473,1444,2012-08-05,0,0,1,18,30,12,60,1,2,220,73,0,1,0,1,0,11,18470.14657,21687.77583,12.6121
8474,1444,2012-08-05,1,0,1,27,22.6666660308838,10.3333330154419,60,3,2,220,73,0,1,0,1,0,11,1
8475,1444,2012-08-05,2,0,1,24.3333339691162,28,7.66666650772095,60,3,2,220,73,0,1,0,1,0,11,1
8476,1444,2012-08-05,3,0,1,19.6666660308838,38.3333320617676,2,60,3,2,220,73,0,1,0,1,0,11,18
8477,1444,2012-08-05,4,0,1,49,11,0,60,1,2,220,73,0,1,0,1,0,11,18470.14657,21687.77583,12.61213
8478,1444,2012-08-06,1,1,0,22.6000003814697,26.2000007629395,7,55.7999992370605,3,2,220,7
8479,1444,2012-08-06,2,1,0,40.6666679382324,16.6666660308838,2.66666674613953,60,3,2,220,
8480,1444,2012-08-06,3,1,0,26.6666660308838,26,7.33333349227905,60,3,2,220,73,0,1,0,1,0,11,1
8481,1444,2012-08-06,4,1,0,46,14,0,60,3,2,220,73,0,1,0,1,0,11,18470.14657,21687.77583,12.61213
8482,1444,2012-08-07,1,2,0,9,31.5,8.75,49.25,3,2,220,73,0,1,0,1,0,11,18470.14657,21687.77583,12
8483,1444,2012-08-07,2,2,0,28.1666660308838,28.6666660308838,3.16666674613953,60,3,2,220,
8484,1444,2012-08-07,3,2,0,16.3333339691162,32.6666679382324,11,60,3,2,220,73,0,1,0,1,0,11,1
8485,1444,2012-08-07,4,2,0,41.6666679382324,18,0.333333343267441,60,3,2,220,73,0,1,0,1,0,11,
8486,1444,2012-08-08,0,3,0,32.5,0.5,0,33,2,2,220,73,0,1,0,1,0,11,18470.14657,21687.77583,12.612
8487,1444,2012-08-08,1,3,0,29,25.3333339691162,5.66666650772095,60,3,2,220,73,0,1,0,1,0,11,1
8488,1444,2012-08-08,2,3,0,42.5,15,2.5,60,2,2,220,73,0,1,0,1,0,11,18470.14657,21687.77583,12.61
8489,1444,2012-08-08,3,3,0,33.3333320617676,22.6666660308838,4,60,3,2,220,73,0,1,0,1,0,11,18
8490,1444,2012-08-08,4,3,0,43.6666679382324,16.3333339691162,0,60,3,2,220,73,0,1,0,1,0,11,18
8491,1444,2012-08-09,0,4,0,14.5,30.5,6.5,51.5,2,2,220,73,0,1,0,1,0,11,18470.14657,21687.77583,1
8492,1444,2012-08-09,1,4,0,26.3333339691162,25.6666660308838,8,60,3,2,220,73,0,1,0,1,0,11,18
8493,1444,2012-08-09,2,4,0,31.5,26.25,2.25,60,3,2,220,73,0,1,0,1,0,11,18470.14657,21687.77583,1
8494,1444,2012-08-09,3,4,0,42.25,17,0.75,60,3,2,220,73,0,1,0,1,0,11,18470.14657,21687.77583,12
8495,1444,2012-08-09,4,4,0,40,20,0,60,3,2,220,73,0,1,0,1,0,11,18470.14657,21687.77583,12.61213
8496,1444,2012-08-22,1,3,0,14.3333330154419,35.3333320617676,4.33333349227905,54,3,2,220,
8497,1444,2012-08-22,2,3,0,33.3333320617676,22,4.66666650772095,60,3,2,220,73,0,1,0,1,0,11,1
8498,1444,2012-08-22,3,3,0,38.5999984741211,21.2000007629395,0.200000002980232,60,3,2,220
8499,1444,2012-08-22,4,3,0,39,20,1,60,3,2,220,73,0,1,0,1,0,11,18470.14657,21687.77583,12.61213

8500,1444,2012-08-23,1,4,0,37.3333320617676,7,9.16666698455811,53.5,3,2,220,73,0,1,0,1,0,11,1
8501,1444,2012-08-23,2,4,0,23.6666660308838,29.6666660308838,6.66666650772095,60,3,2,220,
8502,1444,2012-08-23,3,4,0,23.6666660308838,29,7.33333349227905,60,3,2,220,73,0,1,0,1,0,11,1
8503,1444,2012-08-23,4,4,0,43.7999992370605,16.2000007629395,0,60,3,2,220,73,0,1,0,1,0,11,18
8504,1444,2012-08-24,2,5,0,42.3333320617676,7.33333349227905,10.3333330154419,60,3,2,220,
8505,1444,2012-08-24,3,5,0,35,16,9,60,3,2,220,73,0,1,0,1,0,11,18470.14657,21687.77583,12.61213
8506,1444,2012-08-24,4,5,0,56,3,1,60,3,2,220,73,0,1,0,1,0,11,18470.14657,21687.77583,12.612136
8507,1446,2012-08-22,0,3,0,0,1,0,1,1,2,220,92,0,1,0,1,0,9,18676.8879,20130.46913,11.12898118,1
8508,1446,2012-08-22,1,3,0,27.7999992370605,24,5.19999980926514,57,3,2,220,92,0,1,0,1,0,9,18
8509,1446,2012-08-22,2,3,0,42.6666679382324,17.3333339691162,0,60,3,2,220,92,0,1,0,1,0,9,186
8510,1446,2012-08-22,3,3,0,27,24,9,60,3,2,220,92,0,1,0,1,0,9,18676.8879,20130.46913,11.1289811
8511,1446,2012-08-22,4,3,0,37,23,0,60,3,2,220,92,0,1,0,1,0,9,18676.8879,20130.46913,11.1289811
8512,1446,2012-08-23,0,4,0,3,8,0,11,1,2,220,92,0,1,0,1,0,9,18676.8879,20130.46913,11.12898118,
8513,1446,2012-08-23,1,4,0,26.5,28.5,5,60,3,2,220,92,0,1,0,1,0,9,18676.8879,20130.46913,11.1289
8514,1446,2012-08-23,2,4,0,40.3333320617676,17.5,2.16666674613953,60,3,2,220,92,0,1,0,1,0,9,1
8515,1446,2012-08-23,3,4,0,38.6666679382324,18,3.33333325386047,60,3,2,220,92,0,1,0,1,0,9,18
8516,1446,2012-08-23,4,4,0,48,12,0,60,3,2,220,92,0,1,0,1,0,9,18676.8879,20130.46913,11.1289811
8517,1446,2012-08-24,0,5,0,46,13,1,60,1,2,220,92,0,1,0,1,0,9,18676.8879,20130.46913,11.1289811
8518,1446,2012-08-24,1,5,0,33,22,2.33333325386047,57.3333320617676,3,2,220,92,0,1,0,1,0,9,18
8519,1446,2012-08-24,2,5,0,37.1666679382324,22.8333339691162,0,60,3,2,220,92,0,1,0,1,0,9,186
8520,1446,2012-08-24,3,5,0,33.4285697937012,21.8571434020996,4.7142858505249,60,3,2,220,9
8521,1446,2012-08-24,4,5,0,44.3333320617676,15.6666669845581,0,60,3,2,220,92,0,1,0,1,0,9,186
8522,1446,2012-08-25,0,6,1,28,7.66666650772095,0.333333343267441,36,3,2,220,92,0,1,0,1,0,9,1
8523,1446,2012-08-25,1,6,1,26.7999992370605,23.3999996185303,3,53.2000007629395,3,2,220,9
8524,1446,2012-08-25,2,6,1,35.1666679382324,23.6666660308838,1.16666662693024,60,3,2,220,
8525,1446,2012-08-25,3,6,1,31.75,27.5,0.75,60,3,2,220,92,0,1,0,1,0,9,18676.8879,20130.46913,11.
8526,1446,2012-08-25,4,6,1,43.1818199157715,16.8181819915771,0,60,3,2,220,92,0,1,0,1,0,9,186
8527,1446,2012-08-26,0,0,1,19,5.5,0.5,25,2,2,220,92,0,1,0,1,0,9,18676.8879,20130.46913,11.1289
8528,1446,2012-08-26,1,0,1,36.625,19.625,3.75,60,3,2,220,92,0,1,0,1,0,9,18676.8879,20130.46913
8529,1446,2012-08-26,2,0,1,39.8333320617676,14.6666669845581,5.5,60,3,2,220,92,0,1,0,1,0,9,1
8530,1446,2012-08-26,3,0,1,32.3333320617676,20.5,7.16666650772095,60,3,2,220,92,0,1,0,1,0,9,1
8531,1446,2012-08-26,4,0,1,46.5714302062988,13,0.428571432828903,60,3,2,220,92,0,1,0,1,0,9,1
8532,1446,2012-08-27,1,1,0,23.5,30.5,4,58,3,2,220,92,0,1,0,1,0,9,18676.8879,20130.46913,11.1289
8533,1446,2012-08-27,2,1,0,37.3333320617676,20.6666660308838,2,60,3,2,220,92,0,1,0,1,0,9,186
8534,1446,2012-08-27,3,1,0,27,31,2,60,3,2,220,92,0,1,0,1,0,9,18676.8879,20130.46913,11.1289811
8535,1446,2012-08-27,4,1,0,41.3333320617676,18.6666660308838,0,60,3,2,220,92,0,1,0,1,0,9,186
8536,1446,2012-08-28,1,2,0,16.6666660308838,30.3333339691162,11.3333330154419,58.333332
8537,1446,2012-08-28,2,2,0,48.3333320617676,11.6666669845581,0,60,3,2,220,92,0,1,0,1,0,9,186
8538,1446,2012-08-28,3,2,0,27,32.5,0.5,60,3,2,220,92,0,1,0,1,0,9,18676.8879,20130.46913,11.1289
8539,1446,2012-08-28,4,2,0,39.7999992370605,20.2000007629395,0,60,3,2,220,92,0,1,0,1,0,9,186
8540,1446,2012-08-29,0,3,0,36.5,0.5,0.5,37.5,3,2,220,92,0,1,0,1,0,9,18676.8879,20130.46913,11.12
8541,1446,2012-08-29,1,3,0,35.3333320617676,20.3333339691162,4.33333349227905,60,3,2,220,
8542,1446,2012-08-29,2,3,0,45.3333320617676,14.3333330154419,0.333333343267441,60,3,2,220
8543,1446,2012-08-29,3,3,0,25.3333339691162,30.3333339691162,4.33333349227905,60,3,2,220,
8544,1446,2012-08-29,4,3,0,41,19,0,60,3,2,220,92,0,1,0,1,0,9,18676.8879,20130.46913,11.1289811
8545,1447,2012-08-09,0,4,0,13,43,1,57,1,2,220,79,1,1,1,1,0,9,18831.06727,21027.23344,11.289954
8546,1447,2012-08-09,1,4,0,14.6666669845581,39.6666679382324,5.66666650772095,60,3,2,220,
8547,1447,2012-08-09,2,4,0,19,38,3,60,3,2,220,79,1,1,1,1,0,9,18831.06727,21027.23344,11.289954
8548,1447,2012-08-09,3,4,0,19.6666660308838,37.6666679382324,2.66666674613953,60,3,2,220,
8549,1447,2012-08-09,4,4,0,26,33,1,60,3,2,220,79,1,1,1,1,0,9,18831.06727,21027.23344,11.289954

8550,1447,2012-08-10,0,5,0,19,34,1,54,2,2,220,79,1,1,1,1,0,9,18831.06727,21027.23344,11.289954
8551,1447,2012-08-10,1,5,0,18.2000007629395,32.7999992370605,9,60,3,2,220,79,1,1,1,1,0,9,188
8552,1447,2012-08-10,2,5,0,31.6666660308838,23,5.33333349227905,60,3,2,220,79,1,1,1,1,0,9,18
8553,1447,2012-08-10,3,5,0,13,40,7,60,3,2,220,79,1,1,1,1,0,9,18831.06727,21027.23344,11.289954
8554,1447,2012-08-10,4,5,0,26,32.6666679382324,1.33333337306976,60,3,2,220,79,1,1,1,1,0,9,18
8555,1447,2012-08-11,0,6,1,0,13,2,15,1,2,220,79,1,1,1,1,0,9,18831.06727,21027.23344,11.2899544
8556,1447,2012-08-11,1,6,1,25.25,32.5,2.25,60,3,2,220,79,1,1,1,1,0,9,18831.06727,21027.23344,11
8557,1447,2012-08-11,2,6,1,27.25,28,4.75,60,3,2,220,79,1,1,1,1,0,9,18831.06727,21027.23344,11.2
8558,1447,2012-08-11,3,6,1,24.6666660308838,35,0.333333343267441,60,3,2,220,79,1,1,1,1,0,9,1
8559,1447,2012-08-11,4,6,1,24.6666660308838,35,0.333333343267441,60,3,2,220,79,1,1,1,1,0,9,1
8560,1447,2012-08-12,2,0,1,24.5,35.5,0,60,2,2,220,79,1,1,1,1,0,9,18831.06727,21027.23344,11.28
8561,1447,2012-08-12,3,0,1,28.3333339691162,31.3333339691162,0.333333343267441,60,3,2,22
8562,1447,2012-08-12,4,0,1,28,22,2,52,1,2,220,79,1,1,1,1,0,9,18831.06727,21027.23344,11.289954
8563,1447,2012-08-26,0,0,1,17,42,1,60,1,2,220,79,1,1,1,1,0,9,18831.06727,21027.23344,11.289954
8564,1447,2012-08-26,1,0,1,13.3333330154419,39.3333320617676,7.33333349227905,60,3,2,220,
8565,1447,2012-08-26,2,0,1,23.5,35.5,1,60,2,2,220,79,1,1,1,1,0,9,18831.06727,21027.23344,11.28
8566,1447,2012-08-27,1,1,0,31,24,3.33333325386047,58.3333320617676,3,2,220,79,1,1,1,1,0,9,18
8567,1447,2012-08-27,2,1,0,27,28,5,60,3,2,220,79,1,1,1,1,0,9,18831.06727,21027.23344,11.289954
8568,1447,2012-08-27,3,1,0,35.6666679382324,23.6666660308838,0.666666686534882,60,3,2,22
8569,1447,2012-08-27,4,1,0,32,28,0,60,3,2,220,79,1,1,1,1,0,9,18831.06727,21027.23344,11.289954
8570,1447,2012-08-28,0,2,0,8.5,29,1,38.5,2,2,220,79,1,1,1,1,0,9,18831.06727,21027.23344,11.289
8571,1447,2012-08-28,1,2,0,5,44.6666679382324,10.3333330154419,60,3,2,220,79,1,1,1,1,0,9,188
8572,1447,2012-08-28,2,2,0,26.6666660308838,31.3333339691162,2,60,3,2,220,79,1,1,1,1,0,9,188
8573,1447,2012-08-28,3,2,0,27.1666660308838,24.6666660308838,8.16666698455811,60,3,2,220,
8574,1447,2012-08-28,4,2,0,35,24.6666660308838,0.333333343267441,60,3,2,220,79,1,1,1,1,0,9,1
8575,1451,2012-08-07,0,2,0,5.5,35.5,17.5,58.5,2,2,220,65,1,1,0,1,0,11,18614.81459,23081.53767,8
8576,1451,2012-08-07,1,2,0,25.6666660308838,31,3.33333325386047,60,3,2,220,65,1,1,0,1,0,11,1
8577,1451,2012-08-07,2,2,0,9.25,35,15.75,60,3,2,220,65,1,1,0,1,0,11,18614.81459,23081.53767,8.8
8578,1451,2012-08-07,3,2,0,19,34,7,60,3,2,220,65,1,1,0,1,0,11,18614.81459,23081.53767,8.811227
8579,1451,2012-08-07,4,2,0,26.3333339691162,28.3333339691162,5.33333349227905,60,3,2,220,
8580,1451,2012-08-08,0,3,0,24,18.5,7,49.5,2,2,220,65,1,1,0,1,0,11,18614.81459,23081.53767,8.81
8581,1451,2012-08-08,1,3,0,40,16.6666660308838,3.33333325386047,60,3,2,220,65,1,1,0,1,0,11,1
8582,1451,2012-08-08,2,3,0,23.7999992370605,19.2000007629395,17,60,3,2,220,65,1,1,0,1,0,11,1
8583,1451,2012-08-08,3,3,0,31,27.3333339691162,1.66666662693024,60,3,2,220,65,1,1,0,1,0,11,1
8584,1451,2012-08-08,4,3,0,27.3333339691162,26.6666660308838,6,60,3,2,220,65,1,1,0,1,0,11,18
8585,1451,2012-08-09,3,4,0,15.3333330154419,31,13.6666669845581,60,3,2,220,65,1,1,0,1,0,11,1
8586,1451,2012-08-09,4,4,0,37,21,2,60,3,2,220,65,1,1,0,1,0,11,18614.81459,23081.53767,8.811227
8587,1451,2012-08-12,0,0,1,10,46,4,60,1,2,220,65,1,1,0,1,0,11,18614.81459,23081.53767,8.811227
8588,1451,2012-08-12,1,0,1,7,39,14,60,3,2,220,65,1,1,0,1,0,11,18614.81459,23081.53767,8.811227
8589,1451,2012-08-12,2,0,1,19,33.6666679382324,7.33333349227905,60,3,2,220,65,1,1,0,1,0,11,1
8590,1451,2012-08-12,3,0,1,13,41.3333320617676,5.66666650772095,60,3,2,220,65,1,1,0,1,0,11,1
8591,1451,2012-08-13,0,1,0,10,18,1,29,2,2,220,65,1,1,0,1,0,11,18614.81459,23081.53767,8.811227
8592,1451,2012-08-13,1,1,0,11.6666669845581,34,14.3333330154419,60,3,2,220,65,1,1,0,1,0,11,1
8593,1451,2012-08-13,2,1,0,25,29,6,60,3,2,220,65,1,1,0,1,0,11,18614.81459,23081.53767,8.811227
8594,1451,2012-08-13,3,1,0,32.6666679382324,24,3.33333325386047,60,3,2,220,65,1,1,0,1,0,11,1
8595,1451,2012-08-13,4,1,0,21.5,33.1666679382324,5.33333349227905,60,3,2,220,65,1,1,0,1,0,11
8596,1451,2012-08-14,0,2,0,1,10,2,13,1,2,220,65,1,1,0,1,0,11,18614.81459,23081.53767,8.8112279
8597,1451,2012-08-14,1,2,0,13.1999998092651,32.4000015258789,14.3999996185303,60,3,2,220,
8598,1451,2012-08-14,2,2,0,17.6666660308838,32.3333320617676,10,60,3,2,220,65,1,1,0,1,0,11,1
8599,1451,2012-08-14,3,2,0,20.3333339691162,39.3333320617676,0.333333343267441,60,3,2,22

8600,1451,2012-08-14,4,2,0,30.3333339691162,22.6666660308838,7,60,3,2,220,65,1,1,0,1,0,11,18
8601,1451,2012-08-15,0,3,0,13,26,1,40,1,2,220,65,1,1,0,1,0,11,18614.81459,23081.53767,8.811227
8602,1451,2012-08-15,1,3,0,5.25,40,14.75,60,3,2,220,65,1,1,0,1,0,11,18614.81459,23081.53767,8.8
8603,1451,2012-08-15,2,3,0,13,43,4,60,3,2,220,65,1,1,0,1,0,11,18614.81459,23081.53767,8.811227
8604,1451,2012-08-15,3,3,0,26.3333339691162,31.3333339691162,2.33333325386047,60,3,2,220,
8605,1451,2012-08-15,4,3,0,20.6666660308838,30,9.33333301544189,60,3,2,220,65,1,1,0,1,0,11,1
8606,1467,2012-06-13,0,3,0,21.3333339691162,25.6666660308838,3,50,3,2,220,73,1,1,1,1,0,9,185
8607,1467,2012-06-13,1,3,0,14.6666669845581,41.3333320617676,4,60,3,2,220,73,1,1,1,1,0,9,185
8608,1467,2012-06-13,2,3,0,34,25,1,60,3,2,220,73,1,1,1,1,0,9,18554.83465,22089.8048,13.180219
8609,1467,2012-06-13,3,3,0,12.6666669845581,45.6666679382324,1.66666662693024,60,3,2,220,
8610,1467,2012-06-13,4,3,0,46.6666679382324,13,0.333333343267441,60,3,2,220,73,1,1,1,1,0,9,1
8611,1467,2012-06-14,3,4,0,15.3333330154419,28.6666660308838,16,60,3,2,220,73,1,1,1,1,0,9,18
8612,1467,2012-06-14,4,4,0,31,29,0,60,3,2,220,73,1,1,1,1,0,9,18554.83465,22089.8048,13.180219
8613,1467,2012-08-09,1,4,0,9,46.5,1,56.5,2,2,220,73,1,1,1,1,0,9,18554.83465,22089.8048,13.18021
8614,1467,2012-08-09,2,4,0,26.3333339691162,33.3333320617676,0.333333343267441,60,3,2,22
8615,1467,2012-08-09,3,4,0,24.3333339691162,23.3333339691162,12.3333330154419,60,3,2,220,
8616,1467,2012-08-09,4,4,0,29,31,0,60,1,2,220,73,1,1,1,1,0,9,18554.83465,22089.8048,13.180219
8617,1467,2012-08-10,0,5,0,2,39,1.5,42.5,2,2,220,73,1,1,1,1,0,9,18554.83465,22089.8048,13.18021
8618,1467,2012-08-10,1,5,0,13.3333330154419,46,0.666666686534882,60,3,2,220,73,1,1,1,1,0,9,1
8619,1467,2012-08-10,2,5,0,26.6666660308838,29.3333339691162,4,60,3,2,220,73,1,1,1,1,0,9,185
8620,1467,2012-08-10,3,5,0,16.6666660308838,39.6666679382324,3.66666674613953,60,3,2,220,
8621,1467,2012-08-10,4,5,0,28.75,31,0.25,60,3,2,220,73,1,1,1,1,0,9,18554.83465,22089.8048,13.18
8622,1467,2012-08-11,0,6,1,7,38.5,5.5,51,2,2,220,73,1,1,1,1,0,9,18554.83465,22089.8048,13.18021
8623,1467,2012-08-11,1,6,1,10.3333330154419,31.6666660308838,2.33333325386047,44.333332
8624,1467,2012-08-11,2,6,1,21.3333339691162,37,1.66666662693024,60,3,2,220,73,1,1,1,1,0,9,18
8625,1467,2012-08-11,3,6,1,7.33333349227905,45,7.66666650772095,60,3,2,220,73,1,1,1,1,0,9,18
8626,1467,2012-08-11,4,6,1,37,23,0,60,3,2,220,73,1,1,1,1,0,9,18554.83465,22089.8048,13.180219
8627,1467,2012-08-12,1,0,1,19,21.5,7.5,48,3,2,220,73,1,1,1,1,0,9,18554.83465,22089.8048,13.1802
8628,1467,2012-08-12,2,0,1,21.6666660308838,37,1.33333337306976,60,3,2,220,73,1,1,1,1,0,9,18
8629,1467,2012-08-12,3,0,1,17,36.3333320617676,6.66666650772095,60,3,2,220,73,1,1,1,1,0,9,18
8630,1467,2012-08-12,4,0,1,41,16.3333339691162,2.66666674613953,60,3,2,220,73,1,1,1,1,0,9,18
8631,1467,2012-08-13,0,1,0,1,45.5,7,53.5,2,2,220,73,1,1,1,1,0,9,18554.83465,22089.8048,13.18021
8632,1467,2012-08-13,1,1,0,25,33.6666679382324,1.33333337306976,60,3,2,220,73,1,1,1,1,0,9,18
8633,1467,2012-08-13,2,1,0,40.3333320617676,18.6666660308838,1,60,3,2,220,73,1,1,1,1,0,9,185
8634,1467,2012-08-13,3,1,0,17,32.6666679382324,10.3333330154419,60,3,2,220,73,1,1,1,1,0,9,18
8635,1467,2012-08-13,4,1,0,28.3333339691162,31.3333339691162,0.333333343267441,60,3,2,22
8636,1467,2012-08-14,0,2,0,1,0,0,1,1,2,220,73,1,1,1,1,0,9,18554.83465,22089.8048,13.18021942,1
8637,1467,2012-08-14,1,2,0,6.25,38.75,4.25,49.25,3,2,220,73,1,1,1,1,0,9,18554.83465,22089.8048,
8638,1467,2012-08-14,2,2,0,36,23.3999996185303,0.600000023841858,60,3,2,220,73,1,1,1,1,0,9,1
8639,1467,2012-08-14,3,2,0,22.3333339691162,22.6666660308838,15,60,3,2,220,73,1,1,1,1,0,9,18
8640,1467,2012-08-14,4,2,0,46,14,0,60,3,2,220,73,1,1,1,1,0,9,18554.83465,22089.8048,13.180219
8641,1471,2012-06-14,0,4,0,20.5,24,11,55.5,2,2,220,74,0,0,0,1,0,12,18234.55723,22331.15283,13.9
8642,1471,2012-06-14,1,4,0,10.6666669845581,25,24.3333339691162,60,3,2,220,74,0,0,0,1,0,12,1
8643,1471,2012-06-14,2,4,0,20.6666660308838,36.6666679382324,2.66666674613953,60,3,2,220,
8644,1471,2012-06-14,3,4,0,22,38,0,60,1,2,220,74,0,0,0,1,0,12,18234.55723,22331.15283,13.90834
8645,1471,2012-06-15,0,5,0,16,28,0.5,44.5,2,2,220,74,0,0,0,1,0,12,18234.55723,22331.15283,13.90
8646,1471,2012-06-15,1,5,0,27.6666660308838,32,0.333333343267441,60,3,2,220,74,0,0,0,1,0,12,
8647,1471,2012-06-15,2,5,0,19,40.6666679382324,0.333333343267441,60,3,2,220,74,0,0,0,1,0,12,
8648,1471,2012-06-15,3,5,0,15.5,41.5,3,60,3,2,220,74,0,0,0,1,0,12,18234.55723,22331.15283,13.90
8649,1471,2012-06-15,4,5,0,28.3333339691162,30.6666660308838,1,60,3,2,220,74,0,0,0,1,0,12,18

8650,1471,2012-06-16,0,6,1,8,51,0,59,1,2,220,74,0,0,0,1,0,12,18234.55723,22331.15283,13.90834
8651,1471,2012-06-16,1,6,1,7.33333349227905,44,8.66666698455811,60,3,2,220,74,0,0,0,1,0,12,1
8652,1471,2012-06-16,2,6,1,31.6666660308838,24.3333339691162,4,60,3,2,220,74,0,0,0,1,0,12,18
8653,1471,2012-06-16,3,6,1,21.25,38,0.75,60,3,2,220,74,0,0,0,1,0,12,18234.55723,22331.15283,13
8654,1471,2012-06-16,4,6,1,35.5999984741211,23.2000007629395,1.20000004768372,60,3,2,220,
8655,1471,2012-06-17,0,0,1,13.5,25.25,0.75,39.5,3,2,220,74,0,0,0,1,0,12,18234.55723,22331.1528
8656,1471,2012-06-17,1,0,1,14.5,40.6666679382324,4.83333349227905,60,3,2,220,74,0,0,0,1,0,12
8657,1471,2012-06-17,2,0,1,22.3999996185303,33.2000007629395,4.40000009536743,60,3,2,220,
8658,1471,2012-06-17,3,0,1,10.3333330154419,49.3333320617676,0.333333343267441,60,3,2,22
8659,1471,2012-06-17,4,0,1,25.5,32.5,2,60,3,2,220,74,0,0,0,1,0,12,18234.55723,22331.15283,13.9
8660,1471,2012-06-18,0,1,0,26.5,11,0,37.5,2,2,220,74,0,0,0,1,0,12,18234.55723,22331.15283,13.9
8661,1471,2012-06-18,1,1,0,27,32,1,60,3,2,220,74,0,0,0,1,0,12,18234.55723,22331.15283,13.9083
8662,1471,2012-06-18,2,1,0,30,29.25,0.75,60,3,2,220,74,0,0,0,1,0,12,18234.55723,22331.15283,13
8663,1471,2012-06-18,3,1,0,16,38.6666679382324,5.33333349227905,60,3,2,220,74,0,0,0,1,0,12,1
8664,1471,2012-06-18,4,1,0,34.3333320617676,25.6666660308838,0,60,3,2,220,74,0,0,0,1,0,12,18
8665,1471,2012-06-19,3,2,0,33.6666679382324,26.3333339691162,0,60,3,2,220,74,0,0,0,1,0,12,18
8666,1471,2012-06-19,4,2,0,41.3333320617676,18.6666660308838,0,60,3,2,220,74,0,0,0,1,0,12,18
8667,1472,2012-06-19,0,2,0,58,1,1,60,1,2,220,66,0,1,0,1,0,11,18676.28449,23135.48091,12.84645
8668,1472,2012-06-19,1,2,0,19.3333339691162,32,8.66666698455811,60,3,2,220,66,0,1,0,1,0,11,1
8669,1472,2012-06-19,2,2,0,35,21.3333339691162,3.66666674613953,60,3,2,220,66,0,1,0,1,0,11,1
8670,1472,2012-06-19,3,2,0,25.5,29.5,5,60,2,2,220,66,0,1,0,1,0,11,18676.28449,23135.48091,12.84
8671,1472,2012-06-20,1,3,0,26.75,17,0,43.75,3,2,220,66,0,1,0,1,0,11,18676.28449,23135.48091,12
8672,1472,2012-06-20,2,3,0,39,21,0,60,3,2,220,66,0,1,0,1,0,11,18676.28449,23135.48091,12.84645
8673,1472,2012-06-20,3,3,0,32.5,27.5,0,60,3,2,220,66,0,1,0,1,0,11,18676.28449,23135.48091,12.84
8674,1472,2012-06-20,4,3,0,36,24,0,60,3,2,220,66,0,1,0,1,0,11,18676.28449,23135.48091,12.84645
8675,1472,2012-06-26,0,2,0,7,9,1,17,1,2,220,66,0,1,0,1,0,11,18676.28449,23135.48091,12.846459
8676,1472,2012-06-26,1,2,0,31.3333339691162,28,0.666666686534882,60,3,2,220,66,0,1,0,1,0,11,
8677,1472,2012-06-26,2,2,0,23.3333339691162,36.6666679382324,0,60,3,2,220,66,0,1,0,1,0,11,18
8678,1472,2012-06-26,3,2,0,42,18,0,60,3,2,220,66,0,1,0,1,0,11,18676.28449,23135.48091,12.84645
8679,1472,2012-06-26,4,2,0,44,16,0,60,3,2,220,66,0,1,0,1,0,11,18676.28449,23135.48091,12.84645
8680,1472,2012-06-27,0,3,0,10,23,0,33,1,2,220,66,0,1,0,1,0,11,18676.28449,23135.48091,12.84645
8681,1472,2012-06-27,1,3,0,25,24.3999996185303,8.39999961853027,57.7999992370605,3,2,220,
8682,1472,2012-06-27,2,3,0,23,22,15,60,3,2,220,66,0,1,0,1,0,11,18676.28449,23135.48091,12.8464
8683,1472,2012-06-27,3,3,0,40,20,0,60,3,2,220,66,0,1,0,1,0,11,18676.28449,23135.48091,12.84645
8684,1472,2012-06-27,4,3,0,40.2000007629395,19.7999992370605,0,60,3,2,220,66,0,1,0,1,0,11,18
8685,1472,2012-06-28,1,4,0,16.75,28,8.25,53,3,2,220,66,0,1,0,1,0,11,18676.28449,23135.48091,12
8686,1472,2012-06-28,2,4,0,28,28.5,3.5,60,3,2,220,66,0,1,0,1,0,11,18676.28449,23135.48091,12.84
8687,1472,2012-06-28,3,4,0,40,17,3,60,3,2,220,66,0,1,0,1,0,11,18676.28449,23135.48091,12.84645
8688,1472,2012-06-28,4,4,0,44,16,0,60,3,2,220,66,0,1,0,1,0,11,18676.28449,23135.48091,12.84645
8689,1472,2012-06-29,1,5,0,34.3333320617676,22,0,56.3333320617676,3,2,220,66,0,1,0,1,0,11,18
8690,1472,2012-06-29,2,5,0,25,22.6666660308838,12.3333330154419,60,3,2,220,66,0,1,0,1,0,11,1
8691,1472,2012-06-29,3,5,0,29.5,29.3333339691162,1.16666662693024,60,3,2,220,66,0,1,0,1,0,11
8692,1472,2012-06-29,4,5,0,48.6666679382324,11.3333330154419,0,60,3,2,220,66,0,1,0,1,0,11,18
8693,1472,2012-06-30,0,6,1,8,10,0,18,1,2,220,66,0,1,0,1,0,11,18676.28449,23135.48091,12.846459
8694,1472,2012-06-30,1,6,1,33,23.6666660308838,3.33333325386047,60,3,2,220,66,0,1,0,1,0,11,1
8695,1472,2012-06-30,2,6,1,33,16.6666660308838,10.3333330154419,60,3,2,220,66,0,1,0,1,0,11,1
8696,1472,2012-06-30,3,6,1,29.6666660308838,25.3333339691162,5,60,3,2,220,66,0,1,0,1,0,11,18
8697,1472,2012-06-30,4,6,1,43.5,16.5,0,60,3,2,220,66,0,1,0,1,0,11,18676.28449,23135.48091,12.84
8698,1472,2012-07-01,0,0,1,10,17,0,27,1,2,220,66,0,1,0,1,0,11,18676.28449,23135.48091,12.84645
8699,1472,2012-07-01,1,0,1,31,25.625,2.125,58.75,3,2,220,66,0,1,0,1,0,11,18676.28449,23135.480

8700,1472,2012-07-01,2,0,1,21.3333339691162,31,7.66666650772095,60,3,2,220,66,0,1,0,1,0,11,1
8701,1472,2012-07-01,3,0,1,29.6666660308838,23.8333339691162,6.5,60,3,2,220,66,0,1,0,1,0,11,1
8702,1472,2012-07-01,4,0,1,42.5,17.5,0,60,3,2,220,66,0,1,0,1,0,11,18676.28449,23135.48091,12.84
8703,1472,2012-07-02,1,1,0,25,30.3333339691162,2.33333325386047,57.6666679382324,3,2,220,
8704,1472,2012-07-02,2,1,0,23.6666660308838,29,7.33333349227905,60,3,2,220,66,0,1,0,1,0,11,1
8705,1472,2012-07-02,3,1,0,29.75,26,4.25,60,3,2,220,66,0,1,0,1,0,11,18676.28449,23135.48091,12
8706,1472,2012-07-02,4,1,0,39,21,0,60,3,2,220,66,0,1,0,1,0,11,18676.28449,23135.48091,12.8464
8707,1489,2012-06-26,0,2,0,24,30,3,57,1,2,220,65,1,1,0,1,0,12,18735.57054,23248.74648,10.4175
8708,1489,2012-06-26,1,2,0,13.6666669845581,37.3333320617676,9,60,3,2,220,65,1,1,0,1,0,12,18
8709,1489,2012-06-26,2,2,0,39.6666679382324,19.3333339691162,1,60,3,2,220,65,1,1,0,1,0,12,18
8710,1489,2012-06-26,3,2,0,33.2000007629395,21.7999992370605,5,60,3,2,220,65,1,1,0,1,0,12,18
8711,1489,2012-06-26,4,2,0,35.75,20.5,3.75,60,3,2,220,65,1,1,0,1,0,12,18735.57054,23248.74648,1
8712,1489,2012-06-27,0,3,0,20.25,17.5,2.25,40,3,2,220,65,1,1,0,1,0,12,18735.57054,23248.74648,1
8713,1489,2012-06-27,1,3,0,36,24,0,60,3,2,220,65,1,1,0,1,0,12,18735.57054,23248.74648,10.4175
8714,1489,2012-06-27,2,3,0,35.3333320617676,23,1.66666662693024,60,3,2,220,65,1,1,0,1,0,12,1
8715,1489,2012-06-27,3,3,0,42.6666679382324,15.6666669845581,1.66666662693024,60,3,2,220,
8716,1489,2012-06-27,4,3,0,42.2000007629395,17.7999992370605,0,60,3,2,220,65,1,1,0,1,0,12,18
8717,1489,2012-06-28,0,4,0,3.5,28.5,11.5,43.5,2,2,220,65,1,1,0,1,0,12,18735.57054,23248.74648,1
8718,1489,2012-06-28,1,4,0,19.1666660308838,35.8333320617676,5,60,3,2,220,65,1,1,0,1,0,12,18
8719,1489,2012-06-28,2,4,0,43.6666679382324,15,1.33333337306976,60,3,2,220,65,1,1,0,1,0,12,1
8720,1489,2012-06-28,3,4,0,36.3333320617676,23.3333339691162,0.333333343267441,60,3,2,22
8721,1489,2012-06-28,4,4,0,31.3333339691162,28.6666660308838,0,60,3,2,220,65,1,1,0,1,0,12,18
8722,1489,2012-06-29,0,5,0,15,17.5,4,36.5,2,2,220,65,1,1,0,1,0,12,18735.57054,23248.74648,10.41
8723,1489,2012-06-29,1,5,0,31.3333339691162,28.6666660308838,0,60,3,2,220,65,1,1,0,1,0,12,18
8724,1489,2012-06-29,2,5,0,29.8333339691162,26.5,3.66666674613953,60,3,2,220,65,1,1,0,1,0,12
8725,1489,2012-06-29,3,5,0,25,26,9,60,3,2,220,65,1,1,0,1,0,12,18735.57054,23248.74648,10.4175
8726,1489,2012-06-29,4,5,0,45.3333320617676,14.6666669845581,0,60,3,2,220,65,1,1,0,1,0,12,18
8727,1489,2012-06-30,3,6,1,35,25,0,60,1,2,220,65,1,1,0,1,0,12,18735.57054,23248.74648,10.4175
8728,1489,2012-06-30,4,6,1,40.3333320617676,19.6666660308838,0,60,3,2,220,65,1,1,0,1,0,12,18
8729,1489,2012-07-01,0,0,1,14,41,5,60,1,2,220,65,1,1,0,1,0,12,18735.57054,23248.74648,10.4175
8730,1489,2012-07-01,1,0,1,29.6666660308838,30.3333339691162,0,60,3,2,220,65,1,1,0,1,0,12,18
8731,1489,2012-07-02,0,1,0,14,25.5,8.5,48,2,2,220,65,1,1,0,1,0,12,18735.57054,23248.74648,10.41
8732,1489,2012-07-02,1,1,0,19.3333339691162,32.3333320617676,8.33333301544189,60,3,2,220,
8733,1489,2012-07-02,2,1,0,28.3333339691162,27.3333339691162,4.33333349227905,60,3,2,220,
8734,1489,2012-07-02,3,1,0,21.75,31.75,6.5,60,3,2,220,65,1,1,0,1,0,12,18735.57054,23248.74648,1
8735,1489,2012-07-02,4,1,0,36.3333320617676,23.6666660308838,0,60,3,2,220,65,1,1,0,1,0,12,18
8736,1617,2012-06-30,0,6,1,10.5,24,0,34.5,2,2,215,79,1,0,1,0,0,7,17360.10716,16203.72517,11.197
8737,1617,2012-06-30,1,6,1,34.3333320617676,25.6666660308838,0,60,3,2,215,79,1,0,1,0,0,7,173
8738,1617,2012-06-30,2,6,1,50,10,0,60,3,2,215,79,1,0,1,0,0,7,17360.10716,16203.72517,11.19799
8739,1617,2012-06-30,3,6,1,36,23,1,60,3,2,215,79,1,0,1,0,0,7,17360.10716,16203.72517,11.19799
8740,1617,2012-06-30,4,6,1,14,27,1.5,42.5,2,2,215,79,1,0,1,0,0,7,17360.10716,16203.72517,11.197
8741,1617,2012-07-01,0,0,1,20.3333339691162,32.3333320617676,0,52.6666679382324,3,2,215,7
8742,1617,2012-07-01,1,0,1,17.75,42,0.25,60,3,2,215,79,1,0,1,0,0,7,17360.10716,16203.72517,11.1
8743,1617,2012-07-01,2,0,1,26,30.3333339691162,3.66666674613953,60,3,2,215,79,1,0,1,0,0,7,17
8744,1617,2012-07-01,3,0,1,23,34,3,60,3,2,215,79,1,0,1,0,0,7,17360.10716,16203.72517,11.19799
8745,1617,2012-07-01,4,0,1,24.5,19,0,43.5,2,2,215,79,1,0,1,0,0,7,17360.10716,16203.72517,11.197
8746,1617,2012-07-02,0,1,0,6,24.5,1.5,32,2,2,215,79,1,0,1,0,0,7,17360.10716,16203.72517,11.197
8747,1617,2012-07-02,1,1,0,14,43,3,60,3,2,215,79,1,0,1,0,0,7,17360.10716,16203.72517,11.19799
8748,1617,2012-07-02,2,1,0,41.2000007629395,17.7999992370605,1,60,3,2,215,79,1,0,1,0,0,7,173
8749,1617,2012-07-02,3,1,0,27.3333339691162,31.6666660308838,1,60,3,2,215,79,1,0,1,0,0,7,173

8750,1617,2012-07-02,4,1,0,28,18.5,0,46.5,2,2,215,79,1,0,1,0,0,7,17360.10716,16203.72517,11.197
8751,1617,2012-07-03,0,2,0,19,26,0,45,1,2,215,79,1,0,1,0,0,7,17360.10716,16203.72517,11.19799
8752,1617,2012-07-03,1,2,0,24,35.6666679382324,0.333333343267441,60,3,2,215,79,1,0,1,0,0,7,1
8753,1617,2012-07-03,2,2,0,41.75,16.25,2,60,3,2,215,79,1,0,1,0,0,7,17360.10716,16203.72517,11.
8754,1617,2012-07-03,3,2,0,34.3333320617676,25.1666660308838,0.5,60,3,2,215,79,1,0,1,0,0,7,17
8755,1617,2012-07-03,4,2,0,28,30,1,59,2,2,215,79,1,0,1,0,0,7,17360.10716,16203.72517,11.19799
8756,1617,2012-07-04,0,3,0,16.5,24.5,0.5,41.5,2,2,215,79,1,0,1,0,0,7,17360.10716,16203.72517,11
8757,1617,2012-07-04,1,3,0,38.3333320617676,21.6666660308838,0,60,3,2,215,79,1,0,1,0,0,7,173
8758,1617,2012-07-04,2,3,0,23.3333339691162,34,2.66666674613953,60,3,2,215,79,1,0,1,0,0,7,17
8759,1617,2012-07-04,3,3,0,18.3333339691162,40.6666679382324,1,60,3,2,215,79,1,0,1,0,0,7,173
8760,1617,2012-07-04,4,3,0,25.6666660308838,15,0,40.6666679382324,3,2,215,79,1,0,1,0,0,7,173
8761,1618,2012-06-14,0,4,0,58,1,1,60,1,2,215,65,1,0,1,0,0,7,17876.25883,16942.24658,10.541640
8762,1618,2012-06-14,1,4,0,16.3333339691162,42.6666679382324,1,60,3,2,215,65,1,0,1,0,0,7,178
8763,1618,2012-06-14,2,4,0,21,37.6666679382324,1.33333337306976,60,3,2,215,65,1,0,1,0,0,7,17
8764,1618,2012-06-14,3,4,0,27,32.3333320617676,0.666666686534882,60,3,2,215,65,1,0,1,0,0,7,1
8765,1618,2012-06-14,4,4,0,13.3333330154419,46,0.666666686534882,60,3,2,215,65,1,0,1,0,0,7,1
8766,1618,2012-06-15,1,5,0,2.33333325386047,12.6666669845581,0,15,3,2,215,65,1,0,1,0,0,7,178
8767,1618,2012-06-15,2,5,0,13.3333330154419,45,1.66666662693024,60,3,2,215,65,1,0,1,0,0,7,17
8768,1618,2012-06-15,3,5,0,16.6666660308838,40.3333320617676,3,60,3,2,215,65,1,0,1,0,0,7,178
8769,1618,2012-06-15,4,5,0,9.66666698455811,49.6666679382324,0.666666686534882,60,3,2,21
8770,1618,2012-06-16,1,6,1,8,33,4,45,3,2,215,65,1,0,1,0,0,7,17876.25883,16942.24658,10.541640
8771,1618,2012-06-16,2,6,1,16,43,1,60,3,2,215,65,1,0,1,0,0,7,17876.25883,16942.24658,10.54164
8772,1618,2012-06-16,3,6,1,7.66666650772095,52.3333320617676,0,60,3,2,215,65,1,0,1,0,0,7,178
8773,1618,2012-06-16,4,6,1,19,40.3333320617676,0.666666686534882,60,3,2,215,65,1,0,1,0,0,7,1
8774,1618,2012-06-17,1,7,1,12.3333330154419,28.6666660308838,0,41,3,2,215,65,1,0,1,0,0,7,178
8775,1618,2012-06-17,2,7,1,4.66666650772095,53,2.33333325386047,60,3,2,215,65,1,0,1,0,0,7,17
8776,1618,2012-06-17,3,7,1,17,40.6666679382324,2.33333325386047,60,3,2,215,65,1,0,1,0,0,7,17
8777,1618,2012-06-17,4,7,1,6.66666650772095,47.3333320617676,6,60,3,2,215,65,1,0,1,0,0,7,178
8778,1618,2012-06-20,0,3,0,6.66666650772095,14.3333330154419,0.333333343267441,21.333333
8779,1618,2012-06-20,1,3,0,33.6666679382324,26.3333339691162,0,60,3,2,215,65,1,0,1,0,0,7,178
8780,1618,2012-06-20,2,3,0,22.6666660308838,37,0.333333343267441,60,3,2,215,65,1,0,1,0,0,7,1
8781,1618,2012-06-20,3,3,0,10,12.3333330154419,0,22.3333339691162,3,2,215,65,1,0,1,0,0,7,178
8782,1618,2012-06-20,4,3,0,47,1.66666662693024,0.333333343267441,49,3,2,215,65,1,0,1,0,0,7,1
8783,1619,2012-06-06,1,3,0,17.3333339691162,20,4,41.3333320617676,3,2,215,81,1,0,1,0,0,9,173
8784,1619,2012-06-06,2,3,0,32.6666679382324,27,0.333333343267441,60,3,2,215,81,1,0,1,0,0,9,1
8785,1619,2012-06-06,3,3,0,40.3333320617676,19,0.666666686534882,60,3,2,215,81,1,0,1,0,0,9,1
8786,1619,2012-06-06,4,3,0,36.6666679382324,22.3333339691162,1,60,3,2,215,81,1,0,1,0,0,9,173
8787,1619,2012-06-07,0,4,0,11.3333330154419,33.6666679382324,2,47,3,2,215,81,1,0,1,0,0,9,173
8788,1619,2012-06-07,1,4,0,24,35,1,60,3,2,215,81,1,0,1,0,0,9,17345.30124,17921.39574,9.397299
8789,1619,2012-06-07,2,4,0,38.6666679382324,21,0.333333343267441,60,3,2,215,81,1,0,1,0,0,9,1
8790,1619,2012-06-07,3,4,0,43.3333320617676,16,0.666666686534882,60,3,2,215,81,1,0,1,0,0,9,1
8791,1619,2012-06-07,4,4,0,32.6666679382324,26,1.33333337306976,60,3,2,215,81,1,0,1,0,0,9,17
8792,1619,2012-06-08,0,5,0,18.3333339691162,23.6666660308838,1.33333337306976,43.333332
8793,1619,2012-06-08,1,5,0,16.6666660308838,36,7.33333349227905,60,3,2,215,81,1,0,1,0,0,9,17
8794,1619,2012-06-08,2,5,0,49,11,0,60,3,2,215,81,1,0,1,0,0,9,17345.30124,17921.39574,9.397299
8795,1619,2012-06-08,3,5,0,41.6666679382324,17.6666660308838,0.666666686534882,60,3,2,21
8796,1619,2012-06-08,4,5,0,35.6666679382324,24,0.333333343267441,60,3,2,215,81,1,0,1,0,0,9,1
8797,1619,2012-06-09,0,6,1,20.3333339691162,31.6666660308838,0.666666686534882,52.66666
8798,1619,2012-06-09,1,6,1,29.3333339691162,30,0.666666686534882,60,3,2,215,81,1,0,1,0,0,9,1
8799,1619,2012-06-09,2,6,1,31.3333339691162,24.3333339691162,4.33333349227905,60,3,2,215,

8800,1619,2012-06-09,3,6,1,40.3333320617676,19.6666660308838,0,60,3,2,215,81,1,0,1,0,0,9,173
8801,1619,2012-06-09,4,6,1,25,25,0.333333343267441,50.3333320617676,3,2,215,81,1,0,1,0,0,9,1
8802,1619,2012-06-10,0,7,1,20,19,1.66666662693024,40.6666679382324,3,2,215,81,1,0,1,0,0,9,17
8803,1619,2012-06-10,1,7,1,33.3333320617676,26.3333339691162,0.333333343267441,60,3,2,21
8804,1619,2012-06-10,2,7,1,38,21.6666660308838,0.333333343267441,60,3,2,215,81,1,0,1,0,0,9,1
8805,1619,2012-06-10,3,7,1,34.6666679382324,24.6666660308838,0.666666686534882,60,3,2,21
8806,1619,2012-06-10,4,7,1,28.3333339691162,31.6666660308838,0,60,3,2,215,81,1,0,1,0,0,9,173
8807,1619,2012-06-11,0,1,0,16.6666660308838,27,1.33333337306976,45,3,2,215,81,1,0,1,0,0,9,17
8808,1619,2012-06-11,1,1,0,29.6666660308838,30,0.333333343267441,60,3,2,215,81,1,0,1,0,0,9,1
8809,1619,2012-06-11,2,1,0,24,29.6666660308838,6.33333349227905,60,3,2,215,81,1,0,1,0,0,9,17
8810,1619,2012-06-11,3,1,0,43,17,0,60,3,2,215,81,1,0,1,0,0,9,17345.30124,17921.39574,9.397299
8811,1619,2012-06-11,4,1,0,34.6666679382324,25,0.333333343267441,60,3,2,215,81,1,0,1,0,0,9,1
8812,1619,2012-06-12,0,2,0,14.3333330154419,34,1.33333337306976,49.6666679382324,3,2,215,
8813,1619,2012-06-12,1,2,0,23,36.3333320617676,0.666666686534882,60,3,2,215,81,1,0,1,0,0,9,1
8814,1619,2012-06-12,2,2,0,40,20,0,60,3,2,215,81,1,0,1,0,0,9,17345.30124,17921.39574,9.397299
8815,1619,2012-06-12,3,2,0,38.3333320617676,21.6666660308838,0,60,3,2,215,81,1,0,1,0,0,9,173
8816,1619,2012-06-12,4,2,0,32,27.3333339691162,0.666666686534882,60,3,2,215,81,1,0,1,0,0,9,1
8817,1620,2012-06-12,0,2,0,17.5,33,1.5,52,2,2,215,68,1,0,0,1,0,12,15887.44106,15092.06233,11.0
8818,1620,2012-06-12,1,2,0,17.3333339691162,39.3333320617676,3.33333325386047,60,3,2,215,
8819,1620,2012-06-12,2,2,0,15.6666669845581,42.3333320617676,2,60,3,2,215,68,1,0,0,1,0,12,15
8820,1620,2012-06-12,3,2,0,17.5,41,1.5,60,2,2,215,68,1,0,0,1,0,12,15887.44106,15092.06233,11.0
8821,1620,2012-06-12,4,2,0,30.3333339691162,29.3333339691162,0.333333343267441,60,3,2,21
8822,1620,2012-06-13,0,3,0,3,24,10,37,1,2,215,68,1,0,0,1,0,12,15887.44106,15092.06233,11.0915
8823,1620,2012-06-13,1,3,0,3,48.6666679382324,8.33333301544189,60,3,2,215,68,1,0,0,1,0,12,15
8824,1620,2012-06-13,2,3,0,23.6666660308838,35.3333320617676,1,60,3,2,215,68,1,0,0,1,0,12,15
8825,1620,2012-06-13,3,3,0,25.2000007629395,32.7999992370605,2,60,3,2,215,68,1,0,0,1,0,12,15
8826,1620,2012-06-13,4,3,0,20.3333339691162,33.6666679382324,0,54,3,2,215,68,1,0,0,1,0,12,15
8827,1620,2012-06-14,0,4,0,10.5,39.5,1.5,51.5,2,2,215,68,1,0,0,1,0,12,15887.44106,15092.06233,1
8828,1620,2012-06-14,1,4,0,14,42,4,60,3,2,215,68,1,0,0,1,0,12,15887.44106,15092.06233,11.0915
8829,1620,2012-06-14,2,4,0,29.3333339691162,30.6666660308838,0,60,3,2,215,68,1,0,0,1,0,12,15
8830,1620,2012-06-14,3,4,0,35.25,24.75,0,60,3,2,215,68,1,0,0,1,0,12,15887.44106,15092.06233,11
8831,1620,2012-06-14,4,4,0,22.25,22,1.25,45.5,3,2,215,68,1,0,0,1,0,12,15887.44106,15092.06233,
8832,1620,2012-06-28,0,4,0,28.5,21.5,0,50,2,2,215,68,1,0,0,1,0,12,15887.44106,15092.06233,11.0
8833,1620,2012-06-28,1,4,0,32.6666679382324,24,3.33333325386047,60,3,2,215,68,1,0,0,1,0,12,1
8834,1620,2012-06-28,2,4,0,23,33.3333320617676,3.66666674613953,60,3,2,215,68,1,0,0,1,0,12,1
8835,1620,2012-06-28,3,4,0,5.66666650772095,16,38.3333320617676,60,3,2,215,68,1,0,0,1,0,12,1
8836,1620,2012-06-28,4,4,0,26.3333339691162,33.3333320617676,0,59.6666679382324,3,2,215,6
8837,1620,2012-06-29,0,5,0,16,36,0,52,2,2,215,68,1,0,0,1,0,12,15887.44106,15092.06233,11.0915
8838,1620,2012-06-29,1,5,0,19,38,3,60,3,2,215,68,1,0,0,1,0,12,15887.44106,15092.06233,11.0915
8839,1620,2012-06-29,2,5,0,24.5,30.5,5,60,3,2,215,68,1,0,0,1,0,12,15887.44106,15092.06233,11.0
8840,1620,2012-06-29,3,5,0,43,17,0,60,3,2,215,68,1,0,0,1,0,12,15887.44106,15092.06233,11.0915
8841,1620,2012-06-29,4,5,0,28.6666660308838,31.3333339691162,0,60,3,2,215,68,1,0,0,1,0,12,15
8842,1620,2012-06-30,0,6,1,15,39,0.5,54.5,2,2,215,68,1,0,0,1,0,12,15887.44106,15092.06233,11.0
8843,1620,2012-06-30,1,6,1,32.6666679382324,25.3333339691162,2,60,3,2,215,68,1,0,0,1,0,12,15
8844,1620,2012-06-30,2,6,1,12.8000001907349,41,6.19999980926514,60,3,2,215,68,1,0,0,1,0,12,1
8845,1620,2012-06-30,3,6,1,25.25,25,9.75,60,3,2,215,68,1,0,0,1,0,12,15887.44106,15092.06233,11
8846,1620,2012-06-30,4,6,1,19,39.6666679382324,0,58.6666679382324,3,2,215,68,1,0,0,1,0,12,15
8847,1620,2012-07-04,0,3,0,24,33,3,60,1,2,215,68,1,0,0,1,0,12,15887.44106,15092.06233,11.0915
8848,1620,2012-07-04,1,3,0,23,36,1,60,1,2,215,68,1,0,0,1,0,12,15887.44106,15092.06233,11.0915
8849,1621,2012-06-23,0,6,1,17,9,34,60,1,2,215,68,0,0,0,1,0,12,15887.44106,15092.06233,11.0915

8850,1621,2012-06-23,1,6,1,35,17.3333339691162,7.66666650772095,60,3,2,215,68,0,0,0,1,0,12,1
8851,1621,2012-06-23,2,6,1,31,23,6,60,3,2,215,68,0,0,0,1,0,12,15887.44106,15092.06233,11.0915
8852,1621,2012-06-23,3,6,1,5,16,39,60,3,2,215,68,0,0,0,1,0,12,15887.44106,15092.06233,11.0915
8853,1621,2012-06-23,4,6,1,39.6666679382324,17.6666660308838,2.33333325386047,59.666667
8854,1621,2012-06-24,0,7,1,28.3333339691162,15.6666669845581,1,45,3,2,215,68,0,0,0,1,0,12,15
8855,1621,2012-06-24,1,7,1,37,16.3333339691162,6.66666650772095,60,3,2,215,68,0,0,0,1,0,12,1
8856,1621,2012-06-24,2,7,1,36.6666679382324,17.3333339691162,6,60,3,2,215,68,0,0,0,1,0,12,15
8857,1621,2012-06-24,3,7,1,41,17.6666660308838,1.33333337306976,60,3,2,215,68,0,0,0,1,0,12,1
8858,1621,2012-06-24,4,7,1,46,13.6666669845581,0.333333343267441,60,3,2,215,68,0,0,0,1,0,12,
8859,1621,2012-06-25,0,1,0,14.3333330154419,28.6666660308838,2.33333325386047,45.333332
8860,1621,2012-06-25,1,1,0,37.3333320617676,21.3333339691162,1.33333337306976,60,3,2,215,
8861,1621,2012-06-25,2,1,0,38.6666679382324,19,2.33333325386047,60,3,2,215,68,0,0,0,1,0,12,1
8862,1621,2012-06-25,3,1,0,30.3333339691162,18.3333339691162,11.3333330154419,60,3,2,215,
8863,1621,2012-06-25,4,1,0,50.6666679382324,8.33333301544189,0.666666686534882,59.66666
8864,1621,2012-06-26,0,2,0,20.6666660308838,25.6666660308838,2,48.3333320617676,3,2,215,6
8865,1621,2012-06-26,1,2,0,19.6666660308838,34.6666679382324,5.66666650772095,60,3,2,215,
8866,1621,2012-06-26,2,2,0,9.66666698455811,24.6666660308838,25.6666660308838,60,3,2,215,
8867,1621,2012-06-26,3,2,0,19,36.6666679382324,4.33333349227905,60,3,2,215,68,0,0,0,1,0,12,1
8868,1621,2012-06-26,4,2,0,42,17.6666660308838,0.333333343267441,60,3,2,215,68,0,0,0,1,0,12,
8869,1621,2012-06-27,0,3,0,20,23.3333339691162,1.66666662693024,45,3,2,215,68,0,0,0,1,0,12,1
8870,1621,2012-06-27,1,3,0,34,24.3333339691162,1.66666662693024,60,3,2,215,68,0,0,0,1,0,12,1
8871,1621,2012-06-27,2,3,0,25.6666660308838,31,3.33333325386047,60,3,2,215,68,0,0,0,1,0,12,1
8872,1621,2012-06-27,3,3,0,22,36.3333320617676,1.66666662693024,60,3,2,215,68,0,0,0,1,0,12,1
8873,1621,2012-06-27,4,3,0,44.3333320617676,10,1,55.3333320617676,3,2,215,68,0,0,0,1,0,12,15
8874,1621,2012-06-28,0,4,0,10,29.3333339691162,4,43.3333320617676,3,2,215,68,0,0,0,1,0,12,15
8875,1621,2012-06-28,1,4,0,9.66666698455811,38,12.3333330154419,60,3,2,215,68,0,0,0,1,0,12,1
8876,1621,2012-06-28,2,4,0,29.3333339691162,29.3333339691162,1.33333337306976,60,3,2,215,
8877,1621,2012-06-28,3,4,0,25,32.6666679382324,2.33333325386047,60,3,2,215,68,0,0,0,1,0,12,1
8878,1621,2012-06-28,4,4,0,43,13.6666669845581,3.33333325386047,60,3,2,215,68,0,0,0,1,0,12,1
8879,1622,2012-06-12,0,2,0,12,46,2,60,3,2,215,72,0,0,0,1,0,12,16182.54506,15128.0199,12.210741
8880,1622,2012-06-12,1,2,0,24.3333339691162,31,4.66666650772095,60,3,2,215,72,0,0,0,1,0,12,1
8881,1622,2012-06-12,2,2,0,25.75,33.75,0.5,60,3,2,215,72,0,0,0,1,0,12,16182.54506,15128.0199,12
8882,1622,2012-06-12,3,2,0,7.75,50.5,1.75,60,3,2,215,72,0,0,0,1,0,12,16182.54506,15128.0199,12.
8883,1622,2012-06-12,4,2,0,14.5,30,2,46.5,2,2,215,72,0,0,0,1,0,12,16182.54506,15128.0199,12.21
8884,1622,2012-06-13,0,3,0,11,46.6666679382324,2.33333325386047,60,3,2,215,72,0,0,0,1,0,12,1
8885,1622,2012-06-13,1,3,0,23.3333339691162,31.6666660308838,5,60,3,2,215,72,0,0,0,1,0,12,16
8886,1622,2012-06-13,2,3,0,4.66666650772095,52,3.33333325386047,60,3,2,215,72,0,0,0,1,0,12,1
8887,1622,2012-06-13,3,3,0,8.5,48.5,3,60,3,2,215,72,0,0,0,1,0,12,16182.54506,15128.0199,12.210
8888,1622,2012-06-13,4,3,0,11,34.6666679382324,11,56.6666679382324,3,2,215,72,0,0,0,1,0,12,1
8889,1622,2012-06-14,3,4,0,4,53,3,60,1,2,215,72,0,0,0,1,0,12,16182.54506,15128.0199,12.210741
8890,1622,2012-06-14,4,4,0,0,18,0,18,1,2,215,72,0,0,0,1,0,12,16182.54506,15128.0199,12.210741
8891,1622,2012-06-15,0,5,0,8.5,6.5,0,15,2,2,215,72,0,0,0,1,0,12,16182.54506,15128.0199,12.2107
8892,1622,2012-06-15,1,5,0,25,22.25,4.5,51.75,3,2,215,72,0,0,0,1,0,12,16182.54506,15128.0199,12
8893,1622,2012-06-15,2,5,0,30.3333339691162,23.6666660308838,6,60,3,2,215,72,0,0,0,1,0,12,16
8894,1622,2012-06-15,3,5,0,43.6666679382324,16.3333339691162,0,60,3,2,215,72,0,0,0,1,0,12,16
8895,1622,2012-06-15,4,5,0,23.3333339691162,22.3333339691162,1.66666662693024,47.333332
8896,1622,2012-06-16,0,6,1,11.6666669845581,47,1.33333337306976,60,3,2,215,72,0,0,0,1,0,12,1
8897,1622,2012-06-16,1,6,1,25.25,33.25,1.5,60,3,2,215,72,0,0,0,1,0,12,16182.54506,15128.0199,12
8898,1622,2012-06-16,2,6,1,18.75,38.5,2.75,60,3,2,215,72,0,0,0,1,0,12,16182.54506,15128.0199,12
8899,1622,2012-06-16,3,6,1,48,12,0,60,3,2,215,72,0,0,0,1,0,12,16182.54506,15128.0199,12.210741

8900,1622,2012-06-16,4,6,1,23.6666660308838,21.3333339691162,3,48,3,2,215,72,0,0,0,1,0,12,16
8901,1622,2012-06-17,0,0,1,11.3333330154419,47,1.66666662693024,60,3,2,215,72,0,0,0,1,0,12,1
8902,1622,2012-06-17,1,0,1,17.6666660308838,34.3333320617676,8,60,3,2,215,72,0,0,0,1,0,12,16
8903,1622,2012-06-17,2,0,1,16,38,6,60,2,2,215,72,0,0,0,1,0,12,16182.54506,15128.0199,12.210741
8904,1622,2012-06-17,3,0,1,8,48.6666679382324,3.33333325386047,60,3,2,215,72,0,0,0,1,0,12,16
8905,1622,2012-06-17,4,0,1,7,37.3333320617676,3.66666674613953,48,3,2,215,72,0,0,0,1,0,12,16
8906,1622,2012-06-18,0,1,0,13,45,2,60,3,2,215,72,0,0,0,1,0,12,16182.54506,15128.0199,12.210741
8907,1622,2012-06-18,1,1,0,31,27.3333339691162,1.66666662693024,60,3,2,215,72,0,0,0,1,0,12,1
8908,1622,2012-06-18,2,1,0,24.6000003814697,33.7999992370605,1.60000002384186,60,3,2,215,
8909,1622,2012-06-18,3,1,0,16.6666660308838,42,1.33333337306976,60,3,2,215,72,0,0,0,1,0,12,1
8910,1622,2012-06-18,4,1,0,8.66666698455811,33.3333320617676,5,47,3,2,215,72,0,0,0,1,0,12,16
8911,1623,2012-06-23,0,6,1,26,34,0,60,1,2,215,81,0,1,0,0,0,12,17610.50476,15354.15598,11.32378
8912,1623,2012-06-23,1,6,1,11.6666669845581,45,3.33333325386047,60,3,2,215,81,0,1,0,0,0,12,1
8913,1623,2012-06-23,2,6,1,27.6666660308838,30.6666660308838,1.66666662693024,60,3,2,215,
8914,1623,2012-06-23,3,6,1,22,31.6666660308838,6.33333349227905,60,3,2,215,81,0,1,0,0,0,12,1
8915,1623,2012-06-23,4,6,1,31.6666660308838,27.3333339691162,1,60,3,2,215,81,0,1,0,0,0,12,17
8916,1623,2012-06-24,0,7,1,12.6666669845581,10.3333330154419,0.333333343267441,23.333333
8917,1623,2012-06-24,1,7,1,11,47.3333320617676,1.66666662693024,60,3,2,215,81,0,1,0,0,0,12,1
8918,1623,2012-06-24,2,7,1,38,20.6666660308838,1.33333337306976,60,3,2,215,81,0,1,0,0,0,12,1
8919,1623,2012-06-24,3,7,1,25,33,2,60,3,2,215,81,0,1,0,0,0,12,17610.50476,15354.15598,11.32378
8920,1623,2012-06-24,4,7,1,22.3333339691162,36,1.66666662693024,60,3,2,215,81,0,1,0,0,0,12,1
8921,1623,2012-06-25,0,1,0,18.6666660308838,36,5.33333349227905,60,3,2,215,81,0,1,0,0,0,12,1
8922,1623,2012-06-25,1,1,0,29,26.6666660308838,4.33333349227905,60,3,2,215,81,0,1,0,0,0,12,1
8923,1623,2012-06-25,2,1,0,20.3333339691162,38,1.66666662693024,60,3,2,215,81,0,1,0,0,0,12,1
8924,1623,2012-06-25,3,1,0,20,39.6666679382324,0.333333343267441,60,3,2,215,81,0,1,0,0,0,12,
8925,1623,2012-06-25,4,1,0,25.3333339691162,34,0.666666686534882,60,3,2,215,81,0,1,0,0,0,12,
8926,1623,2012-06-26,0,2,0,22,12,1,35,3,2,215,81,0,1,0,0,0,12,17610.50476,15354.15598,11.32378
8927,1623,2012-06-26,1,2,0,6,32,22,60,3,2,215,81,0,1,0,0,0,12,17610.50476,15354.15598,11.32378
8928,1623,2012-06-26,2,2,0,25.3333339691162,32,2.66666674613953,60,3,2,215,81,0,1,0,0,0,12,1
8929,1623,2012-06-26,3,2,0,29,29.3333339691162,1.66666662693024,60,3,2,215,81,0,1,0,0,0,12,1
8930,1623,2012-06-26,4,2,0,32.6666679382324,27,0.333333343267441,60,3,2,215,81,0,1,0,0,0,12,
8931,1623,2012-06-27,0,3,0,16.6666660308838,10,0.666666686534882,27.3333339691162,3,2,215
8932,1623,2012-06-27,1,3,0,17,36.3333320617676,6.66666650772095,60,3,2,215,81,0,1,0,0,0,12,1
8933,1623,2012-06-27,2,3,0,24,29.3333339691162,6.66666650772095,60,3,2,215,81,0,1,0,0,0,12,1
8934,1623,2012-06-27,3,3,0,30,22,8,60,3,2,215,81,0,1,0,0,0,12,17610.50476,15354.15598,11.32378
8935,1623,2012-06-27,4,3,0,29.6666660308838,29,1.33333337306976,60,3,2,215,81,0,1,0,0,0,12,1
8936,1623,2012-06-28,0,4,0,17,6.33333349227905,0.333333343267441,23.6666660308838,3,2,215
8937,1623,2012-06-28,1,4,0,22.6666660308838,35.6666679382324,1.66666662693024,60,3,2,215,
8938,1623,2012-06-28,2,4,0,27.6666660308838,31.3333339691162,1,60,3,2,215,81,0,1,0,0,0,12,17
8939,1623,2012-06-28,3,4,0,33.6666679382324,26,0.333333343267441,60,3,2,215,81,0,1,0,0,0,12,
8940,1623,2012-06-28,4,4,0,21.6666660308838,24.6666660308838,5.66666650772095,52,3,2,215,
8941,1623,2012-06-29,0,5,0,10.3333330154419,17,0.333333343267441,27.6666660308838,3,2,215
8942,1623,2012-06-29,1,5,0,25,32,3,60,3,2,215,81,0,1,0,0,0,12,17610.50476,15354.15598,11.32378
8943,1623,2012-06-29,2,5,0,36.3333320617676,22.6666660308838,1,60,3,2,215,81,0,1,0,0,0,12,17
8944,1623,2012-06-29,3,5,0,21.3333339691162,37,1.66666662693024,60,3,2,215,81,0,1,0,0,0,12,1
8945,1623,2012-06-29,4,5,0,23.6666660308838,35.6666679382324,0.666666686534882,60,3,2,215
8946,1624,2012-06-19,0,2,0,24,36,0,60,1,2,215,69,1,0,1,1,0,8,16781.55686,15178.89413,10.831633
8947,1624,2012-06-19,1,2,0,9,51,0,60,3,2,215,69,1,0,1,1,0,8,16781.55686,15178.89413,10.831633
8948,1624,2012-06-19,2,2,0,36.6666679382324,22.6666660308838,0.666666686534882,60,3,2,215
8949,1624,2012-06-19,3,2,0,47.3333320617676,12,0.666666686534882,60,3,2,215,69,1,0,1,1,0,8,1

8950,1624,2012-06-19,4,2,0,31.3333339691162,28.6666660308838,0,60,3,2,215,69,1,0,1,1,0,8,167
8951,1624,2012-06-20,0,3,0,1,0.333333343267441,0.666666686534882,2,3,2,215,69,1,0,1,1,0,8,16
8952,1624,2012-06-20,1,3,0,30,29,1,60,3,2,215,69,1,0,1,1,0,8,16781.55686,15178.89413,10.831633
8953,1624,2012-06-20,2,3,0,29,29.3333339691162,1.66666662693024,60,3,2,215,69,1,0,1,1,0,8,16
8954,1624,2012-06-20,3,3,0,9.33333301544189,49.3333320617676,1.33333337306976,60,3,2,215,
8955,1624,2012-06-20,4,3,0,32,28,0,60,3,2,215,69,1,0,1,1,0,8,16781.55686,15178.89413,10.831633
8956,1624,2012-06-21,0,4,0,13.6666669845581,15,1,29.6666660308838,3,2,215,69,1,0,1,1,0,8,167
8957,1624,2012-06-21,1,4,0,10.6666669845581,46.6666679382324,2.66666674613953,60,3,2,215,
8958,1624,2012-06-21,2,4,0,22,38,0,60,3,2,215,69,1,0,1,1,0,8,16781.55686,15178.89413,10.831633
8959,1624,2012-06-21,3,4,0,45.6666679382324,14.3333330154419,0,60,3,2,215,69,1,0,1,1,0,8,167
8960,1624,2012-06-21,4,4,0,44.6666679382324,15.3333330154419,0,60,3,2,215,69,1,0,1,1,0,8,167
8961,1624,2012-06-22,0,5,0,16,18,0.333333343267441,34.3333320617676,3,2,215,69,1,0,1,1,0,8,1
8962,1624,2012-06-22,1,5,0,13,43.6666679382324,3.33333325386047,60,3,2,215,69,1,0,1,1,0,8,16
8963,1624,2012-06-22,2,5,0,39.6666679382324,20.3333339691162,0,60,3,2,215,69,1,0,1,1,0,8,167
8964,1624,2012-06-22,3,5,0,39.3333320617676,20,0.666666686534882,60,3,2,215,69,1,0,1,1,0,8,1
8965,1624,2012-06-22,4,5,0,39.6666679382324,20.3333339691162,0,60,3,2,215,69,1,0,1,1,0,8,167
8966,1624,2012-06-23,0,6,1,2.66666674613953,12.6666669845581,0.666666686534882,16,3,2,215
8967,1624,2012-06-23,1,6,1,39.6666679382324,20,0.333333343267441,60,3,2,215,69,1,0,1,1,0,8,1
8968,1624,2012-06-23,2,6,1,29.3333339691162,27.6666660308838,3,60,3,2,215,69,1,0,1,1,0,8,167
8969,1624,2012-06-23,3,6,1,48,12,0,60,3,2,215,69,1,0,1,1,0,8,16781.55686,15178.89413,10.831633
8970,1624,2012-06-23,4,6,1,36,23.6666660308838,0.333333343267441,60,3,2,215,69,1,0,1,1,0,8,1
8971,1624,2012-06-24,0,7,1,0.666666686534882,4,0.333333343267441,5,3,2,215,69,1,0,1,1,0,8,16
8972,1624,2012-06-24,1,7,1,34,25.6666660308838,0.333333343267441,60,3,2,215,69,1,0,1,1,0,8,1
8973,1624,2012-06-24,2,7,1,47,13,0,60,3,2,215,69,1,0,1,1,0,8,16781.55686,15178.89413,10.831633
8974,1624,2012-06-24,3,7,1,50,10,0,60,3,2,215,69,1,0,1,1,0,8,16781.55686,15178.89413,10.831633
8975,1624,2012-06-24,4,7,1,45.3333320617676,14.6666669845581,0,60,3,2,215,69,1,0,1,1,0,8,167
8976,1624,2012-06-25,0,1,0,3.33333325386047,13.6666669845581,0.333333343267441,17.333333
8977,1624,2012-06-25,1,1,0,10.3333330154419,49,0.666666686534882,60,3,2,215,69,1,0,1,1,0,8,1
8978,1624,2012-06-25,2,1,0,36,23,1,60,3,2,215,69,1,0,1,1,0,8,16781.55686,15178.89413,10.831633
8979,1624,2012-06-25,3,1,0,14.6666669845581,43.6666679382324,1.66666662693024,60,3,2,215,
8980,1624,2012-06-25,4,1,0,39.6666679382324,20.3333339691162,0,60,3,2,215,69,1,0,1,1,0,8,167
8981,1624,2012-06-26,1,2,0,17.3333339691162,39,1,57.3333320617676,3,2,215,69,1,0,1,1,0,8,167
8982,1624,2012-06-26,2,2,0,23,36.3333320617676,0.666666686534882,60,3,2,215,69,1,0,1,1,0,8,1
8983,1624,2012-06-26,3,2,0,28.3333339691162,29,2.66666674613953,60,3,2,215,69,1,0,1,1,0,8,16
8984,1624,2012-06-26,4,2,0,58.3333320617676,1.66666662693024,0,60,3,2,215,69,1,0,1,1,0,8,167
8985,1625,2012-06-22,1,5,0,10.3333330154419,12.6666669845581,5.66666650772095,28.666666
8986,1625,2012-06-22,2,5,0,36,21.3333339691162,2.66666674613953,60,3,2,215,68,0,1,1,0,0,12,1
8987,1625,2012-06-22,3,5,0,23,28.6666660308838,8.33333301544189,60,3,2,215,68,0,1,1,0,0,12,1
8988,1625,2012-06-22,4,5,0,32,19,9,60,3,2,215,68,0,1,1,0,0,12,16236.32172,14999.09627,12.01208
8989,1625,2012-06-23,1,6,1,23.3333339691162,18,4.33333349227905,45.6666679382324,3,2,215,
8990,1625,2012-06-23,2,6,1,25.6666660308838,26.3333339691162,8,60,3,2,215,68,0,1,1,0,0,12,16
8991,1625,2012-06-23,3,6,1,28.6666660308838,29.3333339691162,2,60,3,2,215,68,0,1,1,0,0,12,16
8992,1625,2012-06-23,4,6,1,25,29.6666660308838,5.33333349227905,60,3,2,215,68,0,1,1,0,0,12,1
8993,1625,2012-06-24,1,7,1,11,12.6666669845581,5,28.6666660308838,3,2,215,68,0,1,1,0,0,12,16
8994,1625,2012-06-24,2,7,1,27.3333339691162,19.3333339691162,13.3333330154419,60,3,2,215,
8995,1625,2012-06-24,3,7,1,35.3333320617676,14.6666669845581,10,60,3,2,215,68,0,1,1,0,0,12,1
8996,1625,2012-06-24,4,7,1,7,53,0,60,3,2,215,68,0,1,1,0,0,12,16236.32172,14999.09627,12.012088
8997,1625,2012-06-25,0,1,0,5.66666650772095,9.66666698455811,1.33333337306976,16.666666
8998,1625,2012-06-25,1,1,0,18.6666660308838,32.6666679382324,8.66666698455811,60,3,2,215,
8999,1625,2012-06-25,2,1,0,27,26.6666660308838,6.33333349227905,60,3,2,215,68,0,1,1,0,0,12,1

9000,1625,2012-06-25,3,1,0,25.3333339691162,29.6666660308838,5,60,3,2,215,68,0,1,1,0,0,12,16
9001,1625,2012-06-25,4,1,0,13,44.3333320617676,2.66666674613953,60,3,2,215,68,0,1,1,0,0,12,1
9002,1625,2012-06-26,0,2,0,0.333333343267441,2.66666674613953,0.333333343267441,3.333333
9003,1625,2012-06-26,1,2,0,33.6666679382324,20,6.33333349227905,60,3,2,215,68,0,1,1,0,0,12,1
9004,1625,2012-06-26,2,2,0,25.6666660308838,23.3333339691162,11,60,3,2,215,68,0,1,1,0,0,12,1
9005,1625,2012-06-26,3,2,0,26,29.6666660308838,4.33333349227905,60,3,2,215,68,0,1,1,0,0,12,1
9006,1625,2012-06-26,4,2,0,33,23,4,60,3,2,215,68,0,1,1,0,0,12,16236.32172,14999.09627,12.01208
9007,1625,2012-06-27,0,3,0,7.33333349227905,7.33333349227905,1.33333337306976,16,3,2,215,
9008,1625,2012-06-27,1,3,0,36.3333320617676,17.6666660308838,6,60,3,2,215,68,0,1,1,0,0,12,16
9009,1625,2012-06-27,2,3,0,24,28.6666660308838,7.33333349227905,60,3,2,215,68,0,1,1,0,0,12,1
9010,1625,2012-06-27,3,3,0,33.3333320617676,20,6.66666650772095,60,3,2,215,68,0,1,1,0,0,12,1
9011,1625,2012-06-27,4,3,0,26,27.6666660308838,6.33333349227905,60,3,2,215,68,0,1,1,0,0,12,1
9012,1625,2012-06-28,0,4,0,6,14,0.666666686534882,20.6666660308838,3,2,215,68,0,1,1,0,0,12,1
9013,1625,2012-06-28,1,4,0,18.3333339691162,27.3333339691162,14.3333330154419,60,3,2,215,
9014,1625,2012-06-28,2,4,0,25,29.3333339691162,5.66666650772095,60,3,2,215,68,0,1,1,0,0,12,1
9015,1625,2012-06-28,3,4,0,20.3333339691162,17.6666660308838,22,60,3,2,215,68,0,1,1,0,0,12,1
9016,1625,2012-06-28,4,4,0,48,9,3,60,3,2,215,68,0,1,1,0,0,12,16236.32172,14999.09627,12.01208
9017,1626,2012-06-21,0,4,0,51,8,1,60,1,2,215,67,0,1,0,1,0,11,15780.9296,17854.54902,10.848075
9018,1626,2012-06-21,1,4,0,26,9.66666698455811,24.3333339691162,60,3,2,215,67,0,1,0,1,0,11,1
9019,1626,2012-06-21,2,4,0,17.6666660308838,38,4.33333349227905,60,3,2,215,67,0,1,0,1,0,11,1
9020,1626,2012-06-21,3,4,0,9.66666698455811,46,4.33333349227905,60,3,2,215,67,0,1,0,1,0,11,1
9021,1626,2012-06-21,4,4,0,50.6666679382324,9.33333301544189,0,60,3,2,215,67,0,1,0,1,0,11,15
9022,1626,2012-06-22,0,5,0,24,10.6666669845581,0.333333343267441,35,3,2,215,67,0,1,0,1,0,11,
9023,1626,2012-06-22,1,5,0,15.3333330154419,32.3333320617676,12.3333330154419,60,3,2,215,
9024,1626,2012-06-22,2,5,0,33,17.3333339691162,9.66666698455811,60,3,2,215,67,0,1,0,1,0,11,1
9025,1626,2012-06-22,3,5,0,27.3333339691162,22,10.6666669845581,60,3,2,215,67,0,1,0,1,0,11,1
9026,1626,2012-06-22,4,5,0,43.6666679382324,16,0.333333343267441,60,3,2,215,67,0,1,0,1,0,11,
9027,1626,2012-06-23,0,6,1,18.3333339691162,5.66666650772095,0.333333343267441,24.333333
9028,1626,2012-06-23,1,6,1,3,43,14,60,3,2,215,67,0,1,0,1,0,11,15780.9296,17854.54902,10.848075
9029,1626,2012-06-23,2,6,1,35.6666679382324,16,8.33333301544189,60,3,2,215,67,0,1,0,1,0,11,1
9030,1626,2012-06-23,3,6,1,44.3333320617676,15.3333330154419,0.333333343267441,60,3,2,215
9031,1626,2012-06-23,4,6,1,36,16.6666660308838,7.33333349227905,60,3,2,215,67,0,1,0,1,0,11,1
9032,1626,2012-06-24,0,7,1,15.3333330154419,18.6666660308838,1.33333337306976,35.333332
9033,1626,2012-06-24,1,7,1,36,23,1,60,3,2,215,67,0,1,0,1,0,11,15780.9296,17854.54902,10.848075
9034,1626,2012-06-24,2,7,1,34,18,8,60,3,2,215,67,0,1,0,1,0,11,15780.9296,17854.54902,10.848075
9035,1626,2012-06-24,3,7,1,45,14.6666669845581,0.333333343267441,60,3,2,215,67,0,1,0,1,0,11,
9036,1626,2012-06-24,4,7,1,48.3333320617676,11.3333330154419,0.333333343267441,60,3,2,215
9037,1626,2012-06-25,0,1,0,19.6666660308838,15,2.66666674613953,37.3333320617676,3,2,215,
9038,1626,2012-06-25,1,1,0,38,20.6666660308838,1.33333337306976,60,3,2,215,67,0,1,0,1,0,11,1
9039,1626,2012-06-25,2,1,0,43,15.6666669845581,1.33333337306976,60,3,2,215,67,0,1,0,1,0,11,1
9040,1626,2012-06-25,3,1,0,55.6666679382324,4.33333349227905,0,60,3,2,215,67,0,1,0,1,0,11,15
9041,1626,2012-06-25,4,1,0,45.3333320617676,14.6666669845581,0,60,3,2,215,67,0,1,0,1,0,11,15
9042,1626,2012-06-26,0,2,0,8.66666698455811,13.3333330154419,4,26,3,2,215,67,0,1,0,1,0,11,15
9043,1626,2012-06-26,1,2,0,24.3333339691162,27.6666660308838,8,60,3,2,215,67,0,1,0,1,0,11,15
9044,1626,2012-06-26,2,2,0,48.6666679382324,11.3333330154419,0,60,3,2,215,67,0,1,0,1,0,11,15
9045,1626,2012-06-26,3,2,0,46.6666679382324,13,0.333333343267441,60,3,2,215,67,0,1,0,1,0,11,
9046,1626,2012-06-26,4,2,0,50,9.66666698455811,0.333333343267441,60,3,2,215,67,0,1,0,1,0,11,
9047,1626,2012-06-27,0,3,0,22.3333339691162,4.66666650772095,1,28,3,2,215,67,0,1,0,1,0,11,15
9048,1626,2012-06-27,1,3,0,14.3333330154419,23.6666660308838,22,60,3,2,215,67,0,1,0,1,0,11,1
9049,1626,2012-06-27,2,3,0,45.6666679382324,12.3333330154419,2,60,3,2,215,67,0,1,0,1,0,11,15

9050,1626,2012-06-27,3,3,0,45.6666679382324,13.6666669845581,0.666666686534882,60,3,2,21
9051,1626,2012-06-27,4,3,0,47.3333320617676,9.33333301544189,3.33333325386047,60,3,2,215,
9052,1635,2012-06-06,1,3,0,13,12,1,26,3,2,215,72,0,1,1,1,0,11,17743.21627,16866.64374,11.8745
9053,1635,2012-06-06,2,3,0,38.3333320617676,21.3333339691162,0.333333343267441,60,3,2,21
9054,1635,2012-06-06,3,3,0,43.3333320617676,14.6666669845581,2,60,3,2,215,72,0,1,1,1,0,11,17
9055,1635,2012-06-06,4,3,0,25,31,4,60,3,2,215,72,0,1,1,1,0,11,17743.21627,16866.64374,11.8745
9056,1635,2012-06-07,1,4,0,13.3333330154419,15.3333330154419,15.6666669845581,44.333332
9057,1635,2012-06-07,2,4,0,39.3333320617676,19.6666660308838,1,60,3,2,215,72,0,1,1,1,0,11,17
9058,1635,2012-06-07,3,4,0,28,27.3333339691162,4.66666650772095,60,3,2,215,72,0,1,1,1,0,11,1
9059,1635,2012-06-07,4,4,0,35,22.3333339691162,2.66666674613953,60,3,2,215,72,0,1,1,1,0,11,1
9060,1635,2012-06-08,1,5,0,22,29.3333339691162,4.66666650772095,56,3,2,215,72,0,1,1,1,0,11,1
9061,1635,2012-06-08,2,5,0,21,28,11,60,3,2,215,72,0,1,1,1,0,11,17743.21627,16866.64374,11.874
9062,1635,2012-06-08,3,5,0,35,18.3333339691162,6.66666650772095,60,3,2,215,72,0,1,1,1,0,11,1
9063,1635,2012-06-08,4,5,0,22,34.3333320617676,3.66666674613953,60,3,2,215,72,0,1,1,1,0,11,1
9064,1635,2012-06-09,0,6,1,16.6666660308838,0,0,16.6666660308838,3,2,215,72,0,1,1,1,0,11,177
9065,1635,2012-06-09,1,6,1,20.3333339691162,22,8.33333301544189,50.6666679382324,3,2,215,
9066,1635,2012-06-09,2,6,1,26.3333339691162,28.6666660308838,5,60,3,2,215,72,0,1,1,1,0,11,17
9067,1635,2012-06-09,3,6,1,44.3333320617676,11.6666669845581,4,60,3,2,215,72,0,1,1,1,0,11,17
9068,1635,2012-06-09,4,6,1,30,29.6666660308838,0.333333343267441,60,3,2,215,72,0,1,1,1,0,11,
9069,1635,2012-06-10,0,7,1,0.333333343267441,0.333333343267441,1,1.66666662693024,3,2,21
9070,1635,2012-06-10,1,7,1,11.3333330154419,43,5.66666650772095,60,3,2,215,72,0,1,1,1,0,11,1
9071,1635,2012-06-10,2,7,1,24,28.6666660308838,7.33333349227905,60,3,2,215,72,0,1,1,1,0,11,1
9072,1635,2012-06-10,3,7,1,41.3333320617676,14.3333330154419,4.33333349227905,60,3,2,215,
9073,1635,2012-06-10,4,7,1,38.3333320617676,20.6666660308838,1,60,3,2,215,72,0,1,1,1,0,11,17
9074,1635,2012-06-11,0,1,0,6.66666650772095,11.3333330154419,2.33333325386047,20.333333
9075,1635,2012-06-11,1,1,0,22.6666660308838,30,7.33333349227905,60,3,2,215,72,0,1,1,1,0,11,1
9076,1635,2012-06-11,2,1,0,35.6666679382324,12.6666669845581,11.6666669845581,60,3,2,215,
9077,1635,2012-06-11,3,1,0,28,29,3,60,3,2,215,72,0,1,1,1,0,11,17743.21627,16866.64374,11.8745
9078,1635,2012-06-11,4,1,0,14.3333330154419,32.3333320617676,13.3333330154419,60,3,2,215,
9079,1635,2012-06-12,0,2,0,45,13.6666669845581,1.33333337306976,60,3,2,215,72,0,1,1,1,0,11,1
9080,1635,2012-06-12,1,2,0,38,18,4,60,3,2,215,72,0,1,1,1,0,11,17743.21627,16866.64374,11.8745
9081,1635,2012-06-12,2,2,0,15,8,6.66666650772095,29.6666660308838,3,2,215,72,0,1,1,1,0,11,17
9082,1635,2012-06-12,3,2,0,31.3333339691162,26,2.66666674613953,60,3,2,215,72,0,1,1,1,0,11,1
9083,1635,2012-06-12,4,2,0,33.6666679382324,22.6666660308838,3.66666674613953,60,3,2,215,
9084,1648,2012-06-07,0,4,0,46,10,4,60,1,2,215,76,0,0,1,0,0,5,17322.7285,15374.26336,11.602757
9085,1648,2012-06-07,1,4,0,51.3333320617676,8.66666698455811,0,60,3,2,215,76,0,0,1,0,0,5,173
9086,1648,2012-06-07,2,4,0,49,11,0,60,3,2,215,76,0,0,1,0,0,5,17322.7285,15374.26336,11.602757
9087,1648,2012-06-07,3,4,0,36.6666679382324,23.3333339691162,0,60,3,2,215,76,0,0,1,0,0,5,173
9088,1648,2012-06-07,4,4,0,30.6666660308838,9.33333301544189,0.333333343267441,40.33333
9089,1648,2012-06-08,0,5,0,25,22.6666660308838,1.66666662693024,49.3333320617676,3,2,215,
9090,1648,2012-06-08,1,5,0,42.3333320617676,17.6666660308838,0,60,3,2,215,76,0,0,1,0,0,5,173
9091,1648,2012-06-08,2,5,0,50.3333320617676,9.66666698455811,0,60,3,2,215,76,0,0,1,0,0,5,173
9092,1648,2012-06-08,3,5,0,39,21,0,60,3,2,215,76,0,0,1,0,0,5,17322.7285,15374.26336,11.602757
9093,1648,2012-06-08,4,5,0,3,20.3333339691162,0.333333343267441,23.6666660308838,3,2,215,
9094,1648,2012-06-09,1,6,1,22.6666660308838,13.6666669845581,0.333333343267441,36.66666
9095,1648,2012-06-09,2,6,1,51,9,0,60,3,2,215,76,0,0,1,0,0,5,17322.7285,15374.26336,11.60275781
9096,1648,2012-06-09,3,6,1,45.6666679382324,14.3333330154419,0,60,3,2,215,76,0,0,1,0,0,5,173
9097,1648,2012-06-09,4,6,1,41.6666679382324,18.3333339691162,0,60,3,2,215,76,0,0,1,0,0,5,173
9098,1648,2012-06-10,1,7,1,36,21.3333339691162,0.666666686534882,58,3,2,215,76,0,0,1,0,0,5,1
9099,1648,2012-06-10,2,7,1,45,15,0,60,3,2,215,76,0,0,1,0,0,5,17322.7285,15374.26336,11.602757

9100,1648,2012-06-10,3,7,1,50.3333320617676,9.66666698455811,0,60,3,2,215,76,0,0,1,0,0,5,173
9101,1648,2012-06-10,4,7,1,40,8.66666698455811,0.333333343267441,49,3,2,215,76,0,0,1,0,0,5,1
9102,1648,2012-06-11,0,1,0,21.3333339691162,21.3333339691162,0.666666686534882,43.333332
9103,1648,2012-06-11,1,1,0,36,24,0,60,3,2,215,76,0,0,1,0,0,5,17322.7285,15374.26336,11.6027578
9104,1648,2012-06-11,2,1,0,53,7,0,60,3,2,215,76,0,0,1,0,0,5,17322.7285,15374.26336,11.60275781
9105,1648,2012-06-11,3,1,0,49.6666679382324,10.3333330154419,0,60,3,2,215,76,0,0,1,0,0,5,173
9106,1648,2012-06-11,4,1,0,49,11,0,60,3,2,215,76,0,0,1,0,0,5,17322.7285,15374.26336,11.6027578
9107,1648,2012-06-12,0,2,0,6,5.66666650772095,0.666666686534882,12.3333330154419,3,2,215,
9108,1648,2012-06-12,1,2,0,50.3333320617676,9.66666698455811,0,60,3,2,215,76,0,0,1,0,0,5,173
9109,1648,2012-06-12,2,2,0,52.3333320617676,7.66666650772095,0,60,3,2,215,76,0,0,1,0,0,5,173
9110,1648,2012-06-12,3,2,0,48.6666679382324,11.3333330154419,0,60,3,2,215,76,0,0,1,0,0,5,173
9111,1648,2012-06-12,4,2,0,27.3333339691162,10.6666669845581,0.666666686534882,38.666667
9112,1648,2012-06-13,0,3,0,22,37.6666679382324,0.333333343267441,60,3,2,215,76,0,0,1,0,0,5,1
9113,1648,2012-06-13,1,3,0,39,21,0,60,3,2,215,76,0,0,1,0,0,5,17322.7285,15374.26336,11.6027578
9114,1648,2012-06-13,2,3,0,38.6666679382324,21.3333339691162,0,60,3,2,215,76,0,0,1,0,0,5,173
9115,1648,2012-06-13,3,3,0,28,32,0,60,3,2,215,76,0,0,1,0,0,5,17322.7285,15374.26336,11.6027578
9116,1648,2012-06-13,4,3,0,23.3333339691162,9.66666698455811,0.333333343267441,33.333332
9117,1663,2012-06-29,0,5,0,2,42,16,60,1,2,215,65,0,1,0,1,0,10,15839.36368,15181.51531,11.72934
9118,1663,2012-06-29,1,5,0,5.66666650772095,35.6666679382324,18.6666660308838,60,3,2,215,
9119,1663,2012-06-29,2,5,0,14.6666669845581,38.3333320617676,7,60,3,2,215,65,0,1,0,1,0,10,15
9120,1663,2012-06-29,3,5,0,20.3333339691162,35.3333320617676,4.33333349227905,60,3,2,215,
9121,1663,2012-06-29,4,5,0,11.6666669845581,13,3,27.6666660308838,3,2,215,65,0,1,0,1,0,10,15
9122,1663,2012-06-30,0,6,1,2.33333325386047,17.3333339691162,4.33333349227905,24,3,2,215,
9123,1663,2012-06-30,1,6,1,16,39,5,60,3,2,215,65,0,1,0,1,0,10,15839.36368,15181.51531,11.72934
9124,1663,2012-06-30,2,6,1,14,43.6666679382324,2.33333325386047,60,3,2,215,65,0,1,0,1,0,10,1
9125,1663,2012-06-30,3,6,1,14.3333330154419,41.3333320617676,4.33333349227905,60,3,2,215,
9126,1663,2012-06-30,4,6,1,17,19,3,39,3,2,215,65,0,1,0,1,0,10,15839.36368,15181.51531,11.72934
9127,1663,2012-07-01,1,7,1,9.33333301544189,20.3333339691162,5,34.6666679382324,3,2,215,6
9128,1663,2012-07-01,2,7,1,10.3333330154419,35.3333320617676,14.3333330154419,60,3,2,215,
9129,1663,2012-07-01,3,7,1,14,44.6666679382324,1.33333337306976,60,3,2,215,65,0,1,0,1,0,10,1
9130,1663,2012-07-01,4,7,1,8.33333301544189,20.3333339691162,2.33333325386047,31,3,2,215,
9131,1663,2012-07-02,0,1,0,4.66666650772095,27,5.33333349227905,37,3,2,215,65,0,1,0,1,0,10,1
9132,1663,2012-07-02,1,1,0,12.6666669845581,36.6666679382324,10.6666669845581,60,3,2,215,
9133,1663,2012-07-02,2,1,0,18.6666660308838,39.3333320617676,2,60,3,2,215,65,0,1,0,1,0,10,15
9134,1663,2012-07-02,3,1,0,5.33333349227905,46.6666679382324,8,60,3,2,215,65,0,1,0,1,0,10,15
9135,1663,2012-07-02,4,1,0,0,2,0.333333343267441,2.33333325386047,3,2,215,65,0,1,0,1,0,10,15
9136,1663,2012-07-03,0,2,0,3.33333325386047,20,4,27.3333339691162,3,2,215,65,0,1,0,1,0,10,15
9137,1663,2012-07-03,1,2,0,19,32,9,60,3,2,215,65,0,1,0,1,0,10,15839.36368,15181.51531,11.72934
9138,1663,2012-07-03,2,2,0,16,37.3333320617676,6.66666650772095,60,3,2,215,65,0,1,0,1,0,10,1
9139,1663,2012-07-03,3,2,0,4,52.3333320617676,3.66666674613953,60,3,2,215,65,0,1,0,1,0,10,15
9140,1663,2012-07-03,4,2,0,6,6,1,13,3,2,215,65,0,1,0,1,0,10,15839.36368,15181.51531,11.7293418
9141,1663,2012-07-04,0,3,0,4,20.3333339691162,1.66666662693024,26,3,2,215,65,0,1,0,1,0,10,15
9142,1663,2012-07-04,1,3,0,9,48,3,60,3,2,215,65,0,1,0,1,0,10,15839.36368,15181.51531,11.729341
9143,1663,2012-07-04,2,3,0,13.3333330154419,33,13.6666669845581,60,3,2,215,65,0,1,0,1,0,10,1
9144,1663,2012-07-04,3,3,0,32.3333320617676,25.6666660308838,2,60,3,2,215,65,0,1,0,1,0,10,15
9145,1663,2012-07-04,4,3,0,10.6666669845581,13.6666669845581,2,26.3333339691162,3,2,215,6
9146,1663,2012-07-05,0,4,0,0,8.66666698455811,2,10.6666669845581,3,2,215,65,0,1,0,1,0,10,158
9147,1663,2012-07-05,1,4,0,23,35.6666679382324,1.33333337306976,60,3,2,215,65,0,1,0,1,0,10,1
9148,1663,2012-07-05,2,4,0,40.6666679382324,19,0.333333343267441,60,3,2,215,65,0,1,0,1,0,10,
9149,1663,2012-07-05,3,4,0,28.6666660308838,30.3333339691162,1,60,3,2,215,65,0,1,0,1,0,10,15

9150,1663,2012-07-05,4,4,0,13.3333330154419,6,0.333333343267441,19.6666660308838,3,2,215,
9151,1663,2012-07-11,0,3,0,4.33333349227905,18,0.666666686534882,23,3,2,215,65,0,1,0,1,0,10,
9152,1663,2012-07-11,1,3,0,18.3333339691162,33.3333320617676,8.33333301544189,60,3,2,215,
9153,1663,2012-07-11,2,3,0,26,32.3333320617676,1.66666662693024,60,3,2,215,65,0,1,0,1,0,10,1
9154,1663,2012-07-11,3,3,0,13.6666669845581,44,2.33333325386047,60,3,2,215,65,0,1,0,1,0,10,1
9155,1663,2012-07-11,4,3,0,0.666666686534882,5.66666650772095,3,9.33333301544189,3,2,215,
9156,1665,2012-06-19,0,2,0,14,35,11,60,1,2,215,74,1,1,0,1,0,9,16127.5117,16239.43454,10.31320
9157,1665,2012-06-19,1,2,0,25.6666660308838,26.3333339691162,8,60,3,2,215,74,1,1,0,1,0,9,161
9158,1665,2012-06-19,2,2,0,30,22.6666660308838,7.33333349227905,60,3,2,215,74,1,1,0,1,0,9,16
9159,1665,2012-06-19,3,2,0,24.6666660308838,27,8.33333301544189,60,3,2,215,74,1,1,0,1,0,9,16
9160,1665,2012-06-19,4,2,0,29.6666660308838,22.6666660308838,7.66666650772095,60,3,2,215,
9161,1665,2012-06-20,0,3,0,0,2.66666674613953,0.666666686534882,3.33333325386047,3,2,215,
9162,1665,2012-06-20,1,3,0,26.6666660308838,30.3333339691162,3,60,3,2,215,74,1,1,0,1,0,9,161
9163,1665,2012-06-20,2,3,0,35.6666679382324,23,1.33333337306976,60,3,2,215,74,1,1,0,1,0,9,16
9164,1665,2012-06-20,3,3,0,8.33333301544189,50.6666679382324,1,60,3,2,215,74,1,1,0,1,0,9,161
9165,1665,2012-06-20,4,3,0,33.6666679382324,26.3333339691162,0,60,3,2,215,74,1,1,0,1,0,9,161
9166,1665,2012-06-21,0,4,0,3.33333325386047,10.3333330154419,1.66666662693024,15.333333
9167,1665,2012-06-21,1,4,0,7,50.6666679382324,2.33333325386047,60,3,2,215,74,1,1,0,1,0,9,161
9168,1665,2012-06-21,2,4,0,20,39.6666679382324,0.333333343267441,60,3,2,215,74,1,1,0,1,0,9,1
9169,1665,2012-06-21,3,4,0,11,46.3333320617676,2.66666674613953,60,3,2,215,74,1,1,0,1,0,9,16
9170,1665,2012-06-21,4,4,0,23.6666660308838,35,1.33333337306976,60,3,2,215,74,1,1,0,1,0,9,16
9171,1665,2012-06-22,0,5,0,0.333333343267441,3.66666674613953,0,4,3,2,215,74,1,1,0,1,0,9,161
9172,1665,2012-06-22,1,5,0,15.3333330154419,38.3333320617676,6.33333349227905,60,3,2,215,
9173,1665,2012-06-22,2,5,0,24,30,6,60,3,2,215,74,1,1,0,1,0,9,16127.5117,16239.43454,10.313202
9174,1665,2012-06-22,3,5,0,14.6666669845581,36.3333320617676,9,60,3,2,215,74,1,1,0,1,0,9,161
9175,1665,2012-06-22,4,5,0,32,26.3333339691162,1.66666662693024,60,3,2,215,74,1,1,0,1,0,9,16
9176,1665,2012-06-23,0,6,1,6,14.3333330154419,0.333333343267441,20.6666660308838,3,2,215,
9177,1665,2012-06-23,1,6,1,11.6666669845581,37,11.3333330154419,60,3,2,215,74,1,1,0,1,0,9,16
9178,1665,2012-06-23,2,6,1,30,29,1,60,3,2,215,74,1,1,0,1,0,9,16127.5117,16239.43454,10.313202
9179,1665,2012-06-23,3,6,1,10,41.6666679382324,8.33333301544189,60,3,2,215,74,1,1,0,1,0,9,16
9180,1665,2012-06-23,4,6,1,11,35,14,60,3,2,215,74,1,1,0,1,0,9,16127.5117,16239.43454,10.31320
9181,1665,2012-06-24,0,7,1,5.66666650772095,13,2,20.6666660308838,3,2,215,74,1,1,0,1,0,9,161
9182,1665,2012-06-24,1,7,1,25.3333339691162,28,6.66666650772095,60,3,2,215,74,1,1,0,1,0,9,16
9183,1665,2012-06-24,2,7,1,5.33333349227905,50.3333320617676,4.33333349227905,60,3,2,215,
9184,1665,2012-06-24,3,7,1,12.3333330154419,45.6666679382324,2,60,3,2,215,74,1,1,0,1,0,9,161
9185,1665,2012-06-24,4,7,1,11,45.6666679382324,3.33333325386047,60,3,2,215,74,1,1,0,1,0,9,16
9186,1665,2012-06-25,0,1,0,7.33333349227905,19,1,27.3333339691162,3,2,215,74,1,1,0,1,0,9,161
9187,1665,2012-06-25,1,1,0,10.6666669845581,41.3333320617676,8,60,3,2,215,74,1,1,0,1,0,9,161
9188,1665,2012-06-25,2,1,0,36.6666679382324,23.3333339691162,0,60,3,2,215,74,1,1,0,1,0,9,161
9189,1665,2012-06-25,3,1,0,21,37,2,60,3,2,215,74,1,1,0,1,0,9,16127.5117,16239.43454,10.313202
9190,1665,2012-06-25,4,1,0,36,23.3333339691162,0.666666686534882,60,3,2,215,74,1,1,0,1,0,9,1
9191,1666,2012-06-29,0,5,0,1,30,29,60,1,2,215,65,1,0,0,1,0,10,16465.88525,17016.9516,10.91437
9192,1666,2012-06-29,1,5,0,2.33333325386047,23.3333339691162,34.3333320617676,60,3,2,215,
9193,1666,2012-06-29,2,5,0,14,39.6666679382324,6.33333349227905,60,3,2,215,65,1,0,0,1,0,10,1
9194,1666,2012-06-29,3,5,0,10.3333330154419,30,19.6666660308838,60,3,2,215,65,1,0,0,1,0,10,1
9195,1666,2012-06-29,4,5,0,13.3333330154419,41.6666679382324,5,60,3,2,215,65,1,0,0,1,0,10,16
9196,1666,2012-06-30,0,6,1,7.33333349227905,34.3333320617676,7,48.6666679382324,3,2,215,6
9197,1666,2012-06-30,1,6,1,12.3333330154419,24.3333339691162,23.3333339691162,60,3,2,215,
9198,1666,2012-06-30,2,6,1,36.3333320617676,17.6666660308838,6,60,3,2,215,65,1,0,0,1,0,10,16
9199,1666,2012-06-30,3,6,1,16.6666660308838,38.6666679382324,4.66666650772095,60,3,2,215,

9200,1666,2012-06-30,4,6,1,24.6666660308838,33.6666679382324,1.66666662693024,60,3,2,215,
9201,1666,2012-07-01,1,7,1,7.66666650772095,27.6666660308838,10.3333330154419,45.6666679
9202,1666,2012-07-01,2,7,1,10.6666669845581,46,3.33333325386047,60,3,2,215,65,1,0,0,1,0,10,1
9203,1666,2012-07-01,3,7,1,13.6666669845581,44.6666679382324,1.66666662693024,60,3,2,215,
9204,1666,2012-07-01,4,7,1,3,50,5,58,3,2,215,65,1,0,0,1,0,10,16465.88525,17016.9516,10.9143748
9205,1666,2012-07-02,0,1,0,18.3333339691162,21.6666660308838,4.33333349227905,44.3333320
9206,1666,2012-07-02,1,1,0,13.6666669845581,31,15.3333330154419,60,3,2,215,65,1,0,0,1,0,10,1
9207,1666,2012-07-02,2,1,0,5.66666650772095,35.6666679382324,18.6666660308838,60,3,2,215,
9208,1666,2012-07-02,3,1,0,0,47.3333320617676,12.6666669845581,60,3,2,215,65,1,0,0,1,0,10,16
9209,1666,2012-07-02,4,1,0,4.66666650772095,7,2,13.6666669845581,3,2,215,65,1,0,0,1,0,10,164
9210,1666,2012-07-03,0,2,0,5.33333349227905,24,4.33333349227905,33.6666679382324,3,2,215,
9211,1666,2012-07-03,1,2,0,6.33333349227905,40.3333320617676,13.3333330154419,60,3,2,215,
9212,1666,2012-07-03,2,2,0,18.6666660308838,37.6666679382324,3.66666674613953,60,3,2,215,
9213,1666,2012-07-03,3,2,0,3.33333325386047,46,10.6666669845581,60,3,2,215,65,1,0,0,1,0,10,1
9214,1666,2012-07-03,4,2,0,4,5,1,10,3,2,215,65,1,0,0,1,0,10,16465.88525,17016.9516,10.91437488
9215,1666,2012-07-04,0,3,0,9.66666698455811,41,9.33333301544189,60,3,2,215,65,1,0,0,1,0,10,1
9216,1666,2012-07-04,1,3,0,4.66666650772095,42.3333320617676,13,60,3,2,215,65,1,0,0,1,0,10,1
9217,1666,2012-07-04,2,3,0,15.6666669845581,35,9.33333301544189,60,3,2,215,65,1,0,0,1,0,10,1
9218,1666,2012-07-04,3,3,0,14,37,8.66666698455811,59.6666679382324,3,2,215,65,1,0,0,1,0,10,1
9219,1690,2012-06-13,0,3,0,3,13,2,18,1,2,215,85,1,1,1,1,0,7,16665.03067,14189.76616,10.8795653
9220,1690,2012-06-13,1,3,0,31,27.2000007629395,1.79999995231628,60,3,2,215,85,1,1,1,1,0,7,16
9221,1690,2012-06-13,2,3,0,42.5,16.75,0.75,60,3,2,215,85,1,1,1,1,0,7,16665.03067,14189.76616,10
9222,1690,2012-06-13,3,3,0,34.6666679382324,24.6666660308838,0.666666686534882,60,3,2,215
9223,1690,2012-06-13,4,3,0,43,17,0,60,3,2,215,85,1,1,1,1,0,7,16665.03067,14189.76616,10.879565
9224,1690,2012-06-14,0,4,0,43,17,0,60,1,2,215,85,1,1,1,1,0,7,16665.03067,14189.76616,10.879565
9225,1690,2012-06-14,1,4,0,32,24.3333339691162,3.66666674613953,60,3,2,215,85,1,1,1,1,0,7,16
9226,1690,2012-06-14,2,4,0,32.2000007629395,25.2000007629395,2.59999990463257,60,3,2,215,
9227,1690,2012-06-14,3,4,0,38.8333320617676,19.6666660308838,1.5,60,3,2,215,85,1,1,1,1,0,7,16
9228,1690,2012-06-14,4,4,0,35.4000015258789,15,0.200000002980232,50.5999984741211,3,2,215
9229,1690,2012-06-15,0,5,0,12.3333330154419,17.3333339691162,0,29.6666660308838,3,2,215,8
9230,1690,2012-06-15,1,5,0,29.6666660308838,29,1.33333337306976,60,3,2,215,85,1,1,1,1,0,7,16
9231,1690,2012-06-15,2,5,0,49.6666679382324,10.3333330154419,0,60,3,2,215,85,1,1,1,1,0,7,166
9232,1690,2012-06-15,3,5,0,33.3333320617676,26.6666660308838,0,60,3,2,215,85,1,1,1,1,0,7,166
9233,1690,2012-06-15,4,5,0,41.6666679382324,17.3333339691162,1,60,3,2,215,85,1,1,1,1,0,7,166
9234,1690,2012-06-16,0,6,1,21.6666660308838,22,0.333333343267441,44,3,2,215,85,1,1,1,1,0,7,1
9235,1690,2012-06-16,1,6,1,23.3333339691162,35.6666679382324,1,60,3,2,215,85,1,1,1,1,0,7,166
9236,1690,2012-06-16,2,6,1,42.3333320617676,16,1.66666662693024,60,3,2,215,85,1,1,1,1,0,7,16
9237,1690,2012-06-16,3,6,1,44,16,0,60,3,2,215,85,1,1,1,1,0,7,16665.03067,14189.76616,10.879565
9238,1690,2012-06-16,4,6,1,44.3333320617676,15.6666669845581,0,60,3,2,215,85,1,1,1,1,0,7,166
9239,1690,2012-06-17,0,0,1,18,14,0.5,32.5,2,2,215,85,1,1,1,1,0,7,16665.03067,14189.76616,10.879
9240,1690,2012-06-17,1,0,1,21,34.6666679382324,4.33333349227905,60,3,2,215,85,1,1,1,1,0,7,16
9241,1690,2012-06-17,2,0,1,42,17,1,60,3,2,215,85,1,1,1,1,0,7,16665.03067,14189.76616,10.879565
9242,1690,2012-06-17,3,0,1,50.6666679382324,9.33333301544189,0,60,3,2,215,85,1,1,1,1,0,7,166
9243,1690,2012-06-17,4,0,1,47.3333320617676,12.6666669845581,0,60,3,2,215,85,1,1,1,1,0,7,166
9244,1690,2012-06-18,0,1,0,23,33,0.666666686534882,56.6666679382324,3,2,215,85,1,1,1,1,0,7,1
9245,1690,2012-06-18,1,1,0,33.25,23.75,3,60,3,2,215,85,1,1,1,1,0,7,16665.03067,14189.76616,10.8
9246,1690,2012-06-18,2,1,0,44.3333320617676,15,0.666666686534882,60,3,2,215,85,1,1,1,1,0,7,1
9247,1690,2012-06-18,3,1,0,51,8.66666698455811,0.333333343267441,60,3,2,215,85,1,1,1,1,0,7,1
9248,1690,2012-06-18,4,1,0,43,16.6666660308838,0.333333343267441,60,3,2,215,85,1,1,1,1,0,7,1
9249,1692,2012-06-14,0,4,0,42,17,1,60,1,2,215,77,0,1,0,1,0,9,16236.32172,14999.09627,12.012088

9250,1692,2012-06-14,1,4,0,21,27.3333339691162,11.6666669845581,60,3,2,215,77,0,1,0,1,0,9,16
9251,1692,2012-06-14,2,4,0,33,27,0,60,3,2,215,77,0,1,0,1,0,9,16236.32172,14999.09627,12.012088
9252,1692,2012-06-14,3,4,0,29,31,0,60,1,2,215,77,0,1,0,1,0,9,16236.32172,14999.09627,12.012088
9253,1692,2012-06-15,0,5,0,0,17,1,18,1,2,215,77,0,1,0,1,0,9,16236.32172,14999.09627,12.0120882
9254,1692,2012-06-15,1,5,0,26,31.3333339691162,2.66666674613953,60,3,2,215,77,0,1,0,1,0,9,16
9255,1692,2012-06-15,2,5,0,42.3333320617676,17.3333339691162,0.333333343267441,60,3,2,215
9256,1692,2012-06-15,3,5,0,30,26.7999992370605,3.20000004768372,60,3,2,215,77,0,1,0,1,0,9,16
9257,1692,2012-06-15,4,5,0,47,13,0,60,3,2,215,77,0,1,0,1,0,9,16236.32172,14999.09627,12.012088
9258,1692,2012-06-16,0,6,1,0,12,3,15,1,2,215,77,0,1,0,1,0,9,16236.32172,14999.09627,12.0120882
9259,1692,2012-06-16,1,6,1,37.3333320617676,21.3333339691162,1.33333337306976,60,3,2,215,
9260,1692,2012-06-16,2,6,1,27.6666660308838,31,1.33333337306976,60,3,2,215,77,0,1,0,1,0,9,16
9261,1692,2012-06-16,3,6,1,4.66666650772095,47.3333320617676,8,60,3,2,215,77,0,1,0,1,0,9,162
9262,1692,2012-06-16,4,6,1,48.5999984741211,11.3999996185303,0,60,3,2,215,77,0,1,0,1,0,9,162
9263,1692,2012-06-17,0,0,1,22.5,20,0,42.5,2,2,215,77,0,1,0,1,0,9,16236.32172,14999.09627,12.012
9264,1692,2012-06-17,1,0,1,27.6666660308838,15.3333330154419,17,60,3,2,215,77,0,1,0,1,0,9,16
9265,1692,2012-06-17,2,0,1,41.6666679382324,17,1.33333337306976,60,3,2,215,77,0,1,0,1,0,9,16
9266,1692,2012-06-17,3,0,1,37.3333320617676,20.3333339691162,2.33333325386047,60,3,2,215,
9267,1692,2012-06-17,4,0,1,33.25,23.5,3.25,60,3,2,215,77,0,1,0,1,0,9,16236.32172,14999.09627,12
9268,1692,2012-06-18,0,1,0,23,21,1,45,1,2,215,77,0,1,0,1,0,9,16236.32172,14999.09627,12.012088
9269,1692,2012-06-18,1,1,0,29.25,18.25,12.5,60,3,2,215,77,0,1,0,1,0,9,16236.32172,14999.09627,1
9270,1692,2012-06-18,2,1,0,46,14,0,60,3,2,215,77,0,1,0,1,0,9,16236.32172,14999.09627,12.012088
9271,1692,2012-06-18,3,1,0,42,18,0,60,3,2,215,77,0,1,0,1,0,9,16236.32172,14999.09627,12.012088
9272,1692,2012-06-18,4,1,0,47.6666679382324,12.3333330154419,0,60,3,2,215,77,0,1,0,1,0,9,162
9273,1692,2012-06-19,0,2,0,13,35,0,48,1,2,215,77,0,1,0,1,0,9,16236.32172,14999.09627,12.012088
9274,1692,2012-06-19,1,2,0,16.5,31.25,12.25,60,3,2,215,77,0,1,0,1,0,9,16236.32172,14999.09627,1
9275,1692,2012-06-19,2,2,0,23.3333339691162,34.6666679382324,2,60,3,2,215,77,0,1,0,1,0,9,162
9276,1692,2012-06-19,3,2,0,20.3333339691162,37.3333320617676,2.33333325386047,60,3,2,215,
9277,1692,2012-06-19,4,2,0,42.6666679382324,15,2.33333325386047,60,3,2,215,77,0,1,0,1,0,9,16
9278,1692,2012-06-20,1,3,0,2,54,4,60,1,2,215,77,0,1,0,1,0,9,16236.32172,14999.09627,12.0120882
9279,1692,2012-06-20,2,3,0,22.6666660308838,35,2.33333325386047,60,3,2,215,77,0,1,0,1,0,9,16
9280,1692,2012-06-20,3,3,0,47,12.6666669845581,0.333333343267441,60,3,2,215,77,0,1,0,1,0,9,1
9281,1692,2012-06-20,4,3,0,51,9,0,60,3,2,215,77,0,1,0,1,0,9,16236.32172,14999.09627,12.0120882
9282,1699,2012-06-14,0,4,0,10,25,0,35,1,2,215,67,0,0,0,1,0,11,15654.76472,14505.19543,11.29606
9283,1699,2012-06-14,1,4,0,37.6666679382324,20.6666660308838,1.66666662693024,60,3,2,215,
9284,1699,2012-06-14,2,4,0,29,29.75,1.25,60,3,2,215,67,0,0,0,1,0,11,15654.76472,14505.19543,11
9285,1699,2012-06-14,3,4,0,27.25,26.75,6,60,3,2,215,67,0,0,0,1,0,11,15654.76472,14505.19543,11
9286,1699,2012-06-14,4,4,0,52.6666679382324,7.33333349227905,0,60,3,2,215,67,0,0,0,1,0,11,15
9287,1699,2012-06-15,0,5,0,3,12,0,15,1,2,215,67,0,0,0,1,0,11,15654.76472,14505.19543,11.296061
9288,1699,2012-06-15,1,5,0,20,36.3333320617676,3.66666674613953,60,3,2,215,67,0,0,0,1,0,11,1
9289,1699,2012-06-15,2,5,0,26,33.3333320617676,0.666666686534882,60,3,2,215,67,0,0,0,1,0,11,
9290,1699,2012-06-15,3,5,0,35.5999984741211,23.7999992370605,0.600000023841858,60,3,2,215
9291,1699,2012-06-15,4,5,0,49.8333320617676,10.1666669845581,0,60,3,2,215,67,0,0,0,1,0,11,15
9292,1699,2012-06-16,0,6,1,14.75,40.25,1.25,56.25,3,2,215,67,0,0,0,1,0,11,15654.76472,14505.195
9293,1699,2012-06-16,1,6,1,12.25,41.75,6,60,3,2,215,67,0,0,0,1,0,11,15654.76472,14505.19543,11
9294,1699,2012-06-16,2,6,1,34.6666679382324,25.3333339691162,0,60,3,2,215,67,0,0,0,1,0,11,15
9295,1699,2012-06-16,3,6,1,36.6666679382324,23.3333339691162,0,60,3,2,215,67,0,0,0,1,0,11,15
9296,1699,2012-06-16,4,6,1,37.6666679382324,21.3333339691162,1,60,3,2,215,67,0,0,0,1,0,11,15
9297,1699,2012-06-17,0,0,1,8.5,26,3.5,38,2,2,215,67,0,0,0,1,0,11,15654.76472,14505.19543,11.296
9298,1699,2012-06-17,1,0,1,34,25.2000007629395,0.800000011920929,60,3,2,215,67,0,0,0,1,0,11,
9299,1699,2012-06-17,2,0,1,29,23.5,7.5,60,3,2,215,67,0,0,0,1,0,11,15654.76472,14505.19543,11.29

9300,1699,2012-06-17,3,0,1,30.3333339691162,21.3333339691162,8.33333301544189,60,3,2,215,
9301,1699,2012-06-17,4,0,1,48,12,0,60,3,2,215,67,0,0,0,1,0,11,15654.76472,14505.19543,11.2960
9302,1699,2012-06-18,0,1,0,11,27.5,3,41.5,2,2,215,67,0,0,0,1,0,11,15654.76472,14505.19543,11.2
9303,1699,2012-06-18,1,1,0,29,26,5,60,3,2,215,67,0,0,0,1,0,11,15654.76472,14505.19543,11.2960
9304,1699,2012-06-18,2,1,0,34.2000007629395,24.2000007629395,1.60000002384186,60,3,2,215,
9305,1699,2012-06-18,3,1,0,33.5999984741211,26,0.400000005960464,60,3,2,215,67,0,0,0,1,0,11,
9306,1699,2012-06-18,4,1,0,49.6666679382324,10,0.333333343267441,60,3,2,215,67,0,0,0,1,0,11,
9307,1699,2012-06-19,3,2,0,42,18,0,60,1,2,215,67,0,0,0,1,0,11,15654.76472,14505.19543,11.2960
9308,1699,2012-06-19,4,2,0,49.6666679382324,10,0.333333343267441,60,3,2,215,67,0,0,0,1,0,11,
9309,1699,2012-06-20,0,3,0,11,43,6,60,1,2,215,67,0,0,0,1,0,11,15654.76472,14505.19543,11.2960
9310,1699,2012-06-20,1,3,0,26,32.3333320617676,1.66666662693024,60,3,2,215,67,0,0,0,1,0,11,1
9311,1699,2012-06-20,2,3,0,37.5,21,1.5,60,2,2,215,67,0,0,0,1,0,11,15654.76472,14505.19543,11.2
9312,1704,2011-10-13,0,4,0,22.5,21.5,1.5,45.5,2,2,215,78,1,1,0,1,0,6,17313.51415,15251.6154,11.1
9313,1704,2011-10-13,1,4,0,20.5,39.5,0,60,2,2,215,78,1,1,0,1,0,6,17313.51415,15251.6154,11.189
9314,1704,2011-10-13,2,4,0,22.3333339691162,37.6666679382324,0,60,3,2,215,78,1,1,0,1,0,6,173
9315,1704,2011-10-13,3,4,0,36.6666679382324,21,2.33333325386047,60,3,2,215,78,1,1,0,1,0,6,17
9316,1704,2011-10-13,4,4,0,25.6666660308838,33,1.33333337306976,60,3,2,215,78,1,1,0,1,0,6,17
9317,1704,2011-10-14,0,5,0,16,26.5,0,42.5,2,2,215,78,1,1,0,1,0,6,17313.51415,15251.6154,11.189
9318,1704,2011-10-14,1,5,0,20.3333339691162,39,0.666666686534882,60,3,2,215,78,1,1,0,1,0,6,1
9319,1704,2011-10-14,2,5,0,26.6666660308838,33.3333320617676,0,60,3,2,215,78,1,1,0,1,0,6,173
9320,1704,2011-10-14,3,5,0,50.3333320617676,9.66666698455811,0,60,3,2,215,78,1,1,0,1,0,6,173
9321,1704,2011-10-14,4,5,0,19.6666660308838,40.3333320617676,0,60,3,2,215,78,1,1,0,1,0,6,173
9322,1704,2011-10-15,0,6,1,9.5,28.5,0.5,38.5,2,2,215,78,1,1,0,1,0,6,17313.51415,15251.6154,11.1
9323,1704,2011-10-15,1,6,1,19.3999996185303,38.7999992370605,1.79999995231628,60,3,2,215,
9324,1704,2011-10-15,2,6,1,25.5,34,0.5,60,3,2,215,78,1,1,0,1,0,6,17313.51415,15251.6154,11.189
9325,1704,2011-10-15,3,6,1,22.6666660308838,35.6666679382324,1.66666662693024,60,3,2,215,
9326,1704,2011-10-15,4,6,1,31.3333339691162,28.6666660308838,0,60,3,2,215,78,1,1,0,1,0,6,173
9327,1704,2011-10-16,0,0,1,7.5,23,1,31.5,2,2,215,78,1,1,0,1,0,6,17313.51415,15251.6154,11.1890
9328,1704,2011-10-16,1,0,1,20.3333339691162,39.6666679382324,0,60,3,2,215,78,1,1,0,1,0,6,173
9329,1704,2011-10-16,2,0,1,24.7999992370605,35,0.200000002980232,60,3,2,215,78,1,1,0,1,0,6,1
9330,1704,2011-10-16,3,0,1,21.25,37.5,1.25,60,3,2,215,78,1,1,0,1,0,6,17313.51415,15251.6154,11.
9331,1704,2011-10-16,4,0,1,35.3333320617676,24.6666660308838,0,60,3,2,215,78,1,1,0,1,0,6,173
9332,1704,2011-10-17,3,1,0,20.5,39.5,0,60,2,2,215,78,1,1,0,1,0,6,17313.51415,15251.6154,11.189
9333,1704,2011-10-17,4,1,0,22.6666660308838,36.6666679382324,0.666666686534882,60,3,2,21
9334,1704,2012-06-19,0,2,0,50,6,4,60,1,2,215,78,1,1,0,1,0,6,17313.51415,15251.6154,11.1890882
9335,1704,2012-06-19,1,2,0,8,40.3333320617676,11.6666669845581,60,3,2,215,78,1,1,0,1,0,6,173
9336,1704,2012-06-19,2,2,0,15,43.3333320617676,1.66666662693024,60,3,2,215,78,1,1,0,1,0,6,17
9337,1704,2012-06-19,3,2,0,15.3333330154419,41,3.66666674613953,60,3,2,215,78,1,1,0,1,0,6,17
9338,1704,2012-06-19,4,2,0,34.6666679382324,25.3333339691162,0,60,3,2,215,78,1,1,0,1,0,6,173
9339,1704,2012-06-20,0,3,0,12.25,36.75,0.5,49.5,3,2,215,78,1,1,0,1,0,6,17313.51415,15251.6154,1
9340,1704,2012-06-20,1,3,0,22.2000007629395,36.7999992370605,1,60,3,2,215,78,1,1,0,1,0,6,173
9341,1704,2012-06-20,2,3,0,33.3333320617676,26.6666660308838,0,60,3,2,215,78,1,1,0,1,0,6,173
9342,1704,2012-06-20,3,3,0,46,14,0,60,3,2,215,78,1,1,0,1,0,6,17313.51415,15251.6154,11.189088
9343,1704,2012-06-20,4,3,0,32.6666679382324,27.3333339691162,0,60,3,2,215,78,1,1,0,1,0,6,173
9344,1713,2011-10-13,0,4,0,6,2,1,9,1,2,215,84,1,0,1,0,0,6,16429.98284,15617.35624,11.25687994,
9345,1713,2011-10-13,1,4,0,33.6666679382324,26.3333339691162,0,60,3,2,215,84,1,0,1,0,0,6,164
9346,1713,2011-10-13,2,4,0,39.3333320617676,20.6666660308838,0,60,3,2,215,84,1,0,1,0,0,6,164
9347,1713,2011-10-13,3,4,0,20.3333339691162,39.6666679382324,0,60,3,2,215,84,1,0,1,0,0,6,164
9348,1713,2011-10-13,4,4,0,23.6666660308838,22,0.333333343267441,46,3,2,215,84,1,0,1,0,0,6,1
9349,1713,2011-10-14,1,5,0,20.3333339691162,23.3333339691162,0,43.6666679382324,3,2,215,8

9350,1713,2011-10-14,2,5,0,36,24,0,60,3,2,215,84,1,0,1,0,0,6,16429.98284,15617.35624,11.256879
9351,1713,2011-10-14,3,5,0,40,20,0,60,3,2,215,84,1,0,1,0,0,6,16429.98284,15617.35624,11.256879
9352,1713,2011-10-14,4,5,0,43.3333320617676,8.66666698455811,0.333333343267441,52.333332
9353,1713,2011-10-15,2,6,1,26,29.6666660308838,1,56.6666679382324,3,2,215,84,1,0,1,0,0,6,164
9354,1713,2011-10-15,3,6,1,40.3333320617676,19.3333339691162,0.333333343267441,60,3,2,215
9355,1713,2011-10-15,4,6,1,24,36,0,60,3,2,215,84,1,0,1,0,0,6,16429.98284,15617.35624,11.256879
9356,1713,2011-10-17,0,1,0,32,28,0,60,1,2,215,84,1,0,1,0,0,6,16429.98284,15617.35624,11.256879
9357,1713,2011-10-17,1,1,0,26.3333339691162,33,0.666666686534882,60,3,2,215,84,1,0,1,0,0,6,1
9358,1713,2011-10-17,2,1,0,31.3333339691162,28.6666660308838,0,60,3,2,215,84,1,0,1,0,0,6,164
9359,1713,2011-10-17,3,1,0,43,17,0,60,1,2,215,84,1,0,1,0,0,6,16429.98284,15617.35624,11.256879
9360,1713,2011-10-18,0,2,0,28,24,1,53,1,2,215,84,1,0,1,0,0,6,16429.98284,15617.35624,11.256879
9361,1713,2011-10-18,1,2,0,29.6666660308838,30.3333339691162,0,60,3,2,215,84,1,0,1,0,0,6,164
9362,1713,2011-10-18,2,2,0,30.3333339691162,29.6666660308838,0,60,3,2,215,84,1,0,1,0,0,6,164
9363,1713,2011-10-18,3,2,0,37.5,22,0.5,60,3,2,215,84,1,0,1,0,0,6,16429.98284,15617.35624,11.256
9364,1713,2011-10-18,4,2,0,21.6666660308838,36.6666679382324,1.66666662693024,60,3,2,215,
9365,1713,2011-10-19,0,3,0,39,13,2,54,1,2,215,84,1,0,1,0,0,6,16429.98284,15617.35624,11.256879
9366,1713,2011-10-19,1,3,0,32,27,1,60,1,2,215,84,1,0,1,0,0,6,16429.98284,15617.35624,11.256879
9367,1713,2011-10-19,2,3,0,10.6666669845581,43.6666679382324,0.333333343267441,54.666667
9368,1713,2011-10-19,3,3,0,30.25,29.5,0.25,60,3,2,215,84,1,0,1,0,0,6,16429.98284,15617.35624,11
9369,1713,2011-10-19,4,3,0,25.3999996185303,23.6000003814697,0,49,3,2,215,84,1,0,1,0,0,6,164
9370,1731,2011-10-15,0,6,1,32,27,1,60,1,2,214,83,1,0,1,0,0,11,10716.6357,9934.080435,5.0134346
9371,1731,2011-10-15,1,6,1,17.3333339691162,41.3333320617676,1.33333337306976,60,3,2,214,
9372,1731,2011-10-16,0,0,1,20,36.5,0.5,57,2,2,214,83,1,0,1,0,0,11,10716.6357,9934.080435,5.0134
9373,1731,2011-10-16,1,0,1,35,25,0,60,1,2,214,83,1,0,1,0,0,11,10716.6357,9934.080435,5.0134346
9374,1731,2011-10-16,2,0,1,15,44.6666679382324,0.333333343267441,60,3,2,214,83,1,0,1,0,0,11,
9375,1731,2011-10-16,3,0,1,15.3333330154419,44.6666679382324,0,60,3,2,214,83,1,0,1,0,0,11,10
9376,1731,2011-10-16,4,0,1,35,24.6666660308838,0.333333343267441,60,3,2,214,83,1,0,1,0,0,11,
9377,1731,2011-10-17,0,1,0,0,2,0,2,1,2,214,83,1,0,1,0,0,11,10716.6357,9934.080435,5.013434684,
9378,1731,2011-10-17,1,1,0,22.5,37.5,0,60,2,2,214,83,1,0,1,0,0,11,10716.6357,9934.080435,5.0134
9379,1731,2011-10-17,2,1,0,30.6666660308838,29.3333339691162,0,60,3,2,214,83,1,0,1,0,0,11,10
9380,1731,2011-10-17,3,1,0,31.6666660308838,28.3333339691162,0,60,3,2,214,83,1,0,1,0,0,11,10
9381,1731,2011-10-17,4,1,0,21,38,1,60,3,2,214,83,1,0,1,0,0,11,10716.6357,9934.080435,5.0134346
9382,1731,2011-10-18,0,2,0,13,27.5,1,41.5,2,2,214,83,1,0,1,0,0,11,10716.6357,9934.080435,5.0134
9383,1731,2011-10-18,1,2,0,27.6666660308838,32.3333320617676,0,60,3,2,214,83,1,0,1,0,0,11,10
9384,1731,2011-10-18,2,2,0,11.6666669845581,48,0.333333343267441,60,3,2,214,83,1,0,1,0,0,11,
9385,1731,2011-10-18,3,2,0,30.3333339691162,29.6666660308838,0,60,3,2,214,83,1,0,1,0,0,11,10
9386,1731,2011-10-18,4,2,0,20.3333339691162,39.3333320617676,0.333333343267441,60,3,2,214
9387,1731,2011-10-20,0,4,0,15,28,3.5,46.5,2,2,214,83,1,0,1,0,0,11,10716.6357,9934.080435,5.0134
9388,1731,2011-10-20,1,4,0,11,44,5,60,3,2,214,83,1,0,1,0,0,11,10716.6357,9934.080435,5.0134346
9389,1731,2011-10-20,2,4,0,34.3333320617676,23,2.66666674613953,60,3,2,214,83,1,0,1,0,0,11,1
9390,1731,2011-10-20,3,4,0,33.3333320617676,26,0.666666686534882,60,3,2,214,83,1,0,1,0,0,11,
9391,1731,2011-10-20,4,4,0,39.6666679382324,19.6666660308838,0.666666686534882,60,3,2,214
9392,1731,2011-10-21,0,5,0,16,35,9,60,1,2,214,83,1,0,1,0,0,11,10716.6357,9934.080435,5.0134346
9393,1731,2011-10-21,1,5,0,11.3333330154419,43,5.66666650772095,60,3,2,214,83,1,0,1,0,0,11,1
9394,1731,2011-10-21,2,5,0,20.6666660308838,34.3333320617676,5,60,3,2,214,83,1,0,1,0,0,11,10
9395,1731,2011-10-21,3,5,0,14,35,11,60,3,2,214,83,1,0,1,0,0,11,10716.6357,9934.080435,5.013434
9396,1731,2011-10-21,4,5,0,23,34,3,60,3,2,214,83,1,0,1,0,0,11,10716.6357,9934.080435,5.0134346
9397,1731,2011-10-22,0,6,1,21.5,29,1,51.5,2,2,214,83,1,0,1,0,0,11,10716.6357,9934.080435,5.0134
9398,1731,2011-10-22,1,6,1,20.6666660308838,37,2.33333325386047,60,3,2,214,83,1,0,1,0,0,11,1
9399,1731,2011-10-22,2,6,1,21.6666660308838,38.3333320617676,0,60,3,2,214,83,1,0,1,0,0,11,10

9400,1731,2011-10-22,3,6,1,33.3333320617676,26.6666660308838,0,60,3,2,214,83,1,0,1,0,0,11,10
9401,1731,2011-10-22,4,6,1,39,21,0,60,1,2,214,83,1,0,1,0,0,11,10716.6357,9934.080435,5.013434
9402,1731,2011-10-24,0,1,0,6.5,31,2.5,40,2,2,214,83,1,0,1,0,0,11,10716.6357,9934.080435,5.01343
9403,1731,2011-10-24,1,1,0,25.3333339691162,31.6666660308838,3,60,3,2,214,83,1,0,1,0,0,11,10
9404,1731,2011-10-24,2,1,0,18,42,0,60,3,2,214,83,1,0,1,0,0,11,10716.6357,9934.080435,5.013434
9405,1731,2011-10-24,3,1,0,19.6666660308838,40.3333320617676,0,60,3,2,214,83,1,0,1,0,0,11,10
9406,1731,2011-10-24,4,1,0,27,33,0,60,2,2,214,83,1,0,1,0,0,11,10716.6357,9934.080435,5.013434
9407,1731,2011-10-25,4,2,0,29,31,0,60,3,2,214,83,1,0,1,0,0,11,10716.6357,9934.080435,5.013434
9408,1735,2011-10-13,0,4,0,3,1.5,1,5.5,2,2,214,72,0,1,0,1,0,11,10651.43018,9346.639037,4.69736
9409,1735,2011-10-13,1,4,0,21.75,37,1.25,60,3,2,214,72,0,1,0,1,0,11,10651.43018,9346.639037,4.
9410,1735,2011-10-13,2,4,0,21.6666660308838,37.3333320617676,1,60,3,2,214,72,0,1,0,1,0,11,10
9411,1735,2011-10-13,3,4,0,34.3333320617676,23.3333339691162,2.33333325386047,60,3,2,214,
9412,1735,2011-10-13,4,4,0,32.5,27.25,0.25,60,3,2,214,72,0,1,0,1,0,11,10651.43018,9346.639037,
9413,1735,2011-10-14,0,5,0,2,20,1,23,1,2,214,72,0,1,0,1,0,11,10651.43018,9346.639037,4.6973662
9414,1735,2011-10-14,1,5,0,11.1999998092651,45.4000015258789,3.40000009536743,60,3,2,214,
9415,1735,2011-10-14,2,5,0,15.75,43.5,0.75,60,3,2,214,72,0,1,0,1,0,11,10651.43018,9346.639037,
9416,1735,2011-10-14,3,5,0,27.3333339691162,30.3333339691162,2.33333325386047,60,3,2,214,
9417,1735,2011-10-14,4,5,0,28.3333339691162,31,0.666666686534882,60,3,2,214,72,0,1,0,1,0,11,
9418,1735,2011-10-15,0,6,1,52.6666679382324,7.33333349227905,0,60,3,2,214,72,0,1,0,1,0,11,10
9419,1735,2011-10-15,1,6,1,13.6666669845581,44.3333320617676,2,60,3,2,214,72,0,1,0,1,0,11,10
9420,1735,2011-10-15,2,6,1,20.3999996185303,36.7999992370605,2.79999995231628,60,3,2,214,
9421,1735,2011-10-15,3,6,1,28,30.3333339691162,1.66666662693024,60,3,2,214,72,0,1,0,1,0,11,1
9422,1735,2011-10-15,4,6,1,27.3333339691162,32.6666679382324,0,60,3,2,214,72,0,1,0,1,0,11,10
9423,1735,2011-10-16,1,0,1,11.3333330154419,42.6666679382324,1.33333337306976,55.333332
9424,1735,2011-10-16,2,0,1,17.3333339691162,41.3333320617676,1.33333337306976,60,3,2,214,
9425,1735,2011-10-16,3,0,1,23.3333339691162,33.8333320617676,2.83333325386047,60,3,2,214,
9426,1735,2011-10-16,4,0,1,36.5999984741211,23.3999996185303,0,60,3,2,214,72,0,1,0,1,0,11,10
9427,1735,2011-10-17,0,1,0,13,39.5,1,53.5,2,2,214,72,0,1,0,1,0,11,10651.43018,9346.639037,4.697
9428,1735,2011-10-17,1,1,0,10.75,42.25,7,60,3,2,214,72,0,1,0,1,0,11,10651.43018,9346.639037,4.
9429,1735,2011-10-17,2,1,0,24,33.6666679382324,2.33333325386047,60,3,2,214,72,0,1,0,1,0,11,1
9430,1735,2011-10-17,3,1,0,33.3333320617676,26.3333339691162,0.333333343267441,60,3,2,21
9431,1735,2011-10-17,4,1,0,28.75,31.25,0,60,3,2,214,72,0,1,0,1,0,11,10651.43018,9346.639037,4.
9432,1735,2011-10-18,1,2,0,14.6000003814697,35,2.40000009536743,52,3,2,214,72,0,1,0,1,0,11,1
9433,1735,2011-10-18,2,2,0,30.3999996185303,27.3999996185303,2.20000004768372,60,3,2,214,
9434,1735,2011-10-18,3,2,0,36.3333320617676,22,1.66666662693024,60,3,2,214,72,0,1,0,1,0,11,1
9435,1735,2011-10-18,4,2,0,16.6666660308838,41,2.33333325386047,60,3,2,214,72,0,1,0,1,0,11,1
9436,1735,2011-10-19,2,3,0,2,51,7,60,1,2,214,72,0,1,0,1,0,11,10651.43018,9346.639037,4.6973662
9437,1735,2011-10-19,3,3,0,12.3333330154419,44.3333320617676,3.33333325386047,60,3,2,214,
9438,1735,2011-10-19,4,3,0,24,35.3333320617676,0.666666686534882,60,3,2,214,72,0,1,0,1,0,11,
9439,1735,2011-10-25,0,2,0,59,1,0,60,1,2,214,72,0,1,0,1,0,11,10651.43018,9346.639037,4.6973662
9440,1735,2011-10-25,1,2,0,17.6666660308838,40,2.33333325386047,60,3,2,214,72,0,1,0,1,0,11,1
9441,1735,2011-10-25,2,2,0,15,45,0,60,3,2,214,72,0,1,0,1,0,11,10651.43018,9346.639037,4.69736
9442,1735,2011-10-25,3,2,0,21.3333339691162,38.6666679382324,0,60,3,2,214,72,0,1,0,1,0,11,10
9443,1735,2011-10-25,4,2,0,17.5,40.5,2,60,2,2,214,72,0,1,0,1,0,11,10651.43018,9346.639037,4.697
9444,1738,2011-10-15,1,6,1,11.75,32.25,0.5,44.5,3,2,214,75,1,0,0,1,0,11,10651.43018,9346.63903
9445,1738,2011-10-15,2,6,1,21,39,0,60,3,2,214,75,1,0,0,1,0,11,10651.43018,9346.639037,4.69736
9446,1738,2011-10-15,3,6,1,19.5,40.5,0,60,3,2,214,75,1,0,0,1,0,11,10651.43018,9346.639037,4.697
9447,1738,2011-10-15,4,6,1,12.6666669845581,45.8333320617676,1.5,60,3,2,214,75,1,0,0,1,0,11,
9448,1738,2011-10-16,1,0,1,12.1999998092651,45.4000015258789,2.40000009536743,60,3,2,214,
9449,1738,2011-10-16,2,0,1,18.5,40.8333320617676,0.666666686534882,60,3,2,214,75,1,0,0,1,0,1

9450,1738,2011-10-16,3,0,1,19.8333339691162,35.6666679382324,4.5,60,3,2,214,75,1,0,0,1,0,11,1
9451,1738,2011-10-16,4,0,1,17,38.3333320617676,4.66666650772095,60,3,2,214,75,1,0,0,1,0,11,1
9452,1738,2011-10-17,1,1,0,8.80000019073486,37.2000007629395,2.20000004768372,48.200000
9453,1738,2011-10-17,2,1,0,10,37.6666679382324,12.3333330154419,60,3,2,214,75,1,0,0,1,0,11,1
9454,1738,2011-10-17,3,1,0,11.6666669845581,25.3333339691162,23,60,3,2,214,75,1,0,0,1,0,11,1
9455,1738,2011-10-17,4,1,0,16.5,24.1666660308838,3,43.6666679382324,3,2,214,75,1,0,0,1,0,11,1
9456,1738,2011-10-18,1,2,0,3.5,37,9.5,50,3,2,214,75,1,0,0,1,0,11,10651.43018,9346.639037,4.697
9457,1738,2011-10-18,2,2,0,10.1666669845581,42.1666679382324,7.66666650772095,60,3,2,214,
9458,1738,2011-10-18,3,2,0,25.25,30.75,4,60,3,2,214,75,1,0,0,1,0,11,10651.43018,9346.639037,4.6
9459,1738,2011-10-18,4,2,0,21.6666660308838,36.3333320617676,2,60,3,2,214,75,1,0,0,1,0,11,10
9460,1738,2011-10-19,0,3,0,59,1,0,60,1,2,214,75,1,0,0,1,0,11,10651.43018,9346.639037,4.697366
9461,1738,2011-10-19,1,3,0,9.33333301544189,42.3333320617676,4.16666650772095,55.833332
9462,1738,2011-10-19,2,3,0,14.6666669845581,40.8333320617676,4.5,60,3,2,214,75,1,0,0,1,0,11,1
9463,1738,2011-10-19,3,3,0,21.2857151031494,35.5714302062988,3.14285707473755,60,3,2,214,
9464,1738,2011-10-19,4,3,0,15,39.2000007629395,5.80000019073486,60,3,2,214,75,1,0,0,1,0,11,1
9465,1738,2011-10-20,1,4,0,11.3333330154419,36,4,51.3333320617676,3,2,214,75,1,0,0,1,0,11,10
9466,1738,2011-10-20,2,4,0,5.66666650772095,40.3333320617676,14,60,3,2,214,75,1,0,0,1,0,11,1
9467,1738,2011-10-20,3,4,0,9.66666698455811,44,6.33333349227905,60,3,2,214,75,1,0,0,1,0,11,1
9468,1738,2011-10-20,4,4,0,25.5,30.25,4.25,60,3,2,214,75,1,0,0,1,0,11,10651.43018,9346.639037,4
9469,1739,2011-10-20,0,4,0,28,22,1.5,51.5,2,2,214,76,0,1,0,1,0,12,10932.45609,9087.120468,5.03
9470,1739,2011-10-20,1,4,0,20.6666660308838,35.5,3.83333325386047,60,3,2,214,76,0,1,0,1,0,12
9471,1739,2011-10-20,2,4,0,46.3333320617676,13.6666669845581,0,60,3,2,214,76,0,1,0,1,0,12,10
9472,1739,2011-10-20,3,4,0,25,34,1,60,3,2,214,76,0,1,0,1,0,12,10932.45609,9087.120468,5.03617
9473,1739,2011-10-20,4,4,0,47.6666679382324,12.3333330154419,0,60,3,2,214,76,0,1,0,1,0,12,10
9474,1739,2011-10-21,1,5,0,29.3333339691162,21.6666660308838,4.66666650772095,55.666667
9475,1739,2011-10-21,2,5,0,31.2000007629395,20.6000003814697,0.800000011920929,52.59999
9476,1739,2011-10-21,3,5,0,40.5999984741211,19,0.400000005960464,60,3,2,214,76,0,1,0,1,0,12,
9477,1739,2011-10-21,4,5,0,32.6666679382324,26.6666660308838,0.666666686534882,60,3,2,21
9478,1739,2011-10-22,0,6,1,3,22,1,26,1,2,214,76,0,1,0,1,0,12,10932.45609,9087.120468,5.036177
9479,1739,2011-10-22,1,6,1,36.3333320617676,17,6.66666650772095,60,3,2,214,76,0,1,0,1,0,12,1
9480,1739,2011-10-22,2,6,1,47.3333320617676,12.6666669845581,0,60,3,2,214,76,0,1,0,1,0,12,10
9481,1739,2011-10-22,3,6,1,30.25,28.25,1.5,60,3,2,214,76,0,1,0,1,0,12,10932.45609,9087.120468,5
9482,1739,2011-10-22,4,6,1,41.5,18.5,0,60,3,2,214,76,0,1,0,1,0,12,10932.45609,9087.120468,5.03
9483,1739,2011-10-23,0,0,1,4.5,16,0,20.5,2,2,214,76,0,1,0,1,0,12,10932.45609,9087.120468,5.036
9484,1739,2011-10-23,1,0,1,29.5,25.8333339691162,4.66666650772095,60,3,2,214,76,0,1,0,1,0,12
9485,1739,2011-10-23,2,0,1,48.6666679382324,11.3333330154419,0,60,3,2,214,76,0,1,0,1,0,12,10
9486,1739,2011-10-23,3,0,1,30.3333339691162,28.6666660308838,1,60,3,2,214,76,0,1,0,1,0,12,10
9487,1739,2011-10-23,4,0,1,48.4000015258789,11.6000003814697,0,60,3,2,214,76,0,1,0,1,0,12,10
9488,1739,2011-10-24,0,1,0,7.5,15.5,0.5,23.5,2,2,214,76,0,1,0,1,0,12,10932.45609,9087.120468,5.
9489,1739,2011-10-24,1,1,0,33.3333320617676,24,2.66666674613953,60,3,2,214,76,0,1,0,1,0,12,1
9490,1739,2011-10-24,2,1,0,47.5,12,0.5,60,3,2,214,76,0,1,0,1,0,12,10932.45609,9087.120468,5.03
9491,1739,2011-10-24,3,1,0,36.8333320617676,22,1.16666662693024,60,3,2,214,76,0,1,0,1,0,12,1
9492,1739,2011-10-24,4,1,0,48.3333320617676,11.6666669845581,0,60,3,2,214,76,0,1,0,1,0,12,10
9493,1739,2011-10-25,1,2,0,23,35,2,60,3,2,214,76,0,1,0,1,0,12,10932.45609,9087.120468,5.03617
9494,1739,2011-10-25,2,2,0,47.3333320617676,12.6666669845581,0,60,3,2,214,76,0,1,0,1,0,12,10
9495,1739,2011-10-25,3,2,0,44.3333320617676,14.3333330154419,1.33333337306976,60,3,2,214,
9496,1739,2011-10-25,4,2,0,36,10,2,48,2,2,214,76,0,1,0,1,0,12,10932.45609,9087.120468,5.03617
9497,1745,2011-10-15,0,6,1,28,26,0,54,1,2,214,83,1,1,1,0,0,7,10062.20229,9906.779852,5.120794
9498,1745,2011-10-15,1,6,1,29,30.6666660308838,0.333333343267441,60,3,2,214,83,1,1,1,0,0,7,1
9499,1745,2011-10-15,2,6,1,40.75,15,0,55.75,3,2,214,83,1,1,1,0,0,7,10062.20229,9906.779852,5.12

9500,1745,2011-10-15,3,6,1,38.4000015258789,11.3999996185303,0,49.7999992370605,3,2,214,8
9501,1745,2011-10-15,4,6,1,47.6666679382324,12.3333330154419,0,60,3,2,214,83,1,1,1,0,0,7,100
9502,1745,2011-10-16,0,0,1,23,26,1,50,1,2,214,83,1,1,1,0,0,7,10062.20229,9906.779852,5.120794
9503,1745,2011-10-16,1,0,1,37,22.3333339691162,0.666666686534882,60,3,2,214,83,1,1,1,0,0,7,1
9504,1745,2011-10-16,2,0,1,40.6666679382324,19.3333339691162,0,60,3,2,214,83,1,1,1,0,0,7,100
9505,1745,2011-10-16,3,0,1,39.75,18.25,2,60,3,2,214,83,1,1,1,0,0,7,10062.20229,9906.779852,5.12
9506,1745,2011-10-16,4,0,1,48,12,0,60,3,2,214,83,1,1,1,0,0,7,10062.20229,9906.779852,5.120794
9507,1745,2011-10-17,1,1,0,27,14.8000001907349,0,41.7999992370605,3,2,214,83,1,1,1,0,0,7,100
9508,1745,2011-10-17,2,1,0,45.5,14.5,0,60,3,2,214,83,1,1,1,0,0,7,10062.20229,9906.779852,5.1207
9509,1745,2011-10-17,3,1,0,46,14,0,60,3,2,214,83,1,1,1,0,0,7,10062.20229,9906.779852,5.120794
9510,1745,2011-10-17,4,1,0,53.7999992370605,6.19999980926514,0,60,3,2,214,83,1,1,1,0,0,7,100
9511,1745,2011-10-18,2,2,0,57,3,0,60,2,2,214,83,1,1,1,0,0,7,10062.20229,9906.779852,5.1207948
9512,1745,2011-10-18,3,2,0,49.6666679382324,10.3333330154419,0,60,3,2,214,83,1,1,1,0,0,7,100
9513,1745,2011-10-18,4,2,0,46,14,0,60,3,2,214,83,1,1,1,0,0,7,10062.20229,9906.779852,5.120794
9514,1745,2011-10-25,0,2,0,58,2,0,60,1,2,214,83,1,1,1,0,0,7,10062.20229,9906.779852,5.1207948
9515,1745,2011-10-25,1,2,0,19.6666660308838,40.3333320617676,0,60,3,2,214,83,1,1,1,0,0,7,100
9516,1745,2011-10-25,2,2,0,43,17,0,60,3,2,214,83,1,1,1,0,0,7,10062.20229,9906.779852,5.120794
9517,1745,2011-10-25,3,2,0,46,14,0,60,3,2,214,83,1,1,1,0,0,7,10062.20229,9906.779852,5.120794
9518,1745,2011-10-26,0,3,0,8,24,0.5,32.5,2,2,214,83,1,1,1,0,0,7,10062.20229,9906.779852,5.1207
9519,1745,2011-10-26,1,3,0,35.3333320617676,22.6666660308838,2,60,3,2,214,83,1,1,1,0,0,7,100
9520,1745,2011-10-26,2,3,0,36.75,22.25,1,60,3,2,214,83,1,1,1,0,0,7,10062.20229,9906.779852,5.12
9521,1745,2011-10-26,3,3,0,51,9,0,60,3,2,214,83,1,1,1,0,0,7,10062.20229,9906.779852,5.1207948
9522,1745,2011-10-26,4,3,0,51.1666679382324,8.83333301544189,0,60,3,2,214,83,1,1,1,0,0,7,100
9523,1752,2011-10-18,1,2,0,9,5,0,14,1,2,214,72,0,0,0,1,0,12,10097.91744,8850.796148,4.4026426
9524,1752,2011-10-18,2,2,0,29.3333339691162,28.3333339691162,2.33333325386047,60,3,2,214,
9525,1752,2011-10-18,3,2,0,38.3333320617676,21.6666660308838,0,60,3,2,214,72,0,0,0,1,0,12,10
9526,1752,2011-10-18,4,2,0,45,14,1,60,3,2,214,72,0,0,0,1,0,12,10097.91744,8850.796148,4.402642
9527,1752,2011-10-19,1,3,0,4,27,0.5,31.5,2,2,214,72,0,0,0,1,0,12,10097.91744,8850.796148,4.402
9528,1752,2011-10-19,2,3,0,21,37.8333320617676,1,59.8333320617676,3,2,214,72,0,0,0,1,0,12,10
9529,1752,2011-10-19,3,3,0,33.6666679382324,26,0.333333343267441,60,3,2,214,72,0,0,0,1,0,12,
9530,1752,2011-10-19,4,3,0,43.3333320617676,16.6666660308838,0,60,3,2,214,72,0,0,0,1,0,12,10
9531,1752,2011-10-20,0,4,0,60,0,0,60,1,2,214,72,0,0,0,1,0,12,10097.91744,8850.796148,4.402642
9532,1752,2011-10-20,1,4,0,13,22.25,4.25,39.5,3,2,214,72,0,0,0,1,0,12,10097.91744,8850.796148,4
9533,1752,2011-10-20,2,4,0,38.6666679382324,21.3333339691162,0,60,3,2,214,72,0,0,0,1,0,12,10
9534,1752,2011-10-20,3,4,0,30,28.6666660308838,1.33333337306976,60,3,2,214,72,0,0,0,1,0,12,1
9535,1752,2011-10-20,4,4,0,50,10,0,60,3,2,214,72,0,0,0,1,0,12,10097.91744,8850.796148,4.402642
9536,1752,2011-10-21,1,5,0,11,46.5,0.5,58,2,2,214,72,0,0,0,1,0,12,10097.91744,8850.796148,4.402
9537,1752,2011-10-21,2,5,0,29.1666660308838,30.8333339691162,0,60,3,2,214,72,0,0,0,1,0,12,10
9538,1752,2011-10-21,3,5,0,50.5,9.5,0,60,3,2,214,72,0,0,0,1,0,12,10097.91744,8850.796148,4.402
9539,1752,2011-10-21,4,5,0,40.6666679382324,19.3333339691162,0,60,3,2,214,72,0,0,0,1,0,12,10
9540,1752,2011-11-04,1,5,0,0,14,1,15,1,2,214,72,0,0,0,1,0,12,10097.91744,8850.796148,4.402642
9541,1752,2011-11-04,2,5,0,39.3333320617676,20,0.666666686534882,60,3,2,214,72,0,0,0,1,0,12,
9542,1752,2011-11-04,3,5,0,34.6666679382324,25,0.333333343267441,60,3,2,214,72,0,0,0,1,0,12,
9543,1752,2011-11-04,4,5,0,42.6666679382324,16.8333339691162,0.5,60,3,2,214,72,0,0,0,1,0,12,1
9544,1754,2011-11-04,1,5,0,38.3333320617676,13,0.333333343267441,51.6666679382324,3,2,214
9545,1754,2011-11-05,1,6,1,6,28,2,36,1,2,214,88,1,1,1,0,0,10,9846.217093,8628.786108,3.729225
9546,1754,2011-11-05,2,6,1,33,26.3333339691162,0.666666686534882,60,3,2,214,88,1,1,1,0,0,10,
9547,1754,2011-11-05,3,6,1,35.1666679382324,24.5,0.333333343267441,60,3,2,214,88,1,1,1,0,0,1
9548,1754,2011-11-05,4,6,1,45.2000007629395,14.8000001907349,0,60,3,2,214,88,1,1,1,0,0,10,98
9549,1754,2011-11-06,0,0,1,11,35,1,47,1,2,214,88,1,1,1,0,0,10,9846.217093,8628.786108,3.729225

9550,1754,2011-11-06,1,0,1,36.8333320617676,19,0.16666667163372,56,3,2,214,88,1,1,1,0,0,10,9
9551,1754,2011-11-06,2,0,1,46,14,0,60,3,2,214,88,1,1,1,0,0,10,9846.217093,8628.786108,3.729225
9552,1754,2011-11-06,3,0,1,49.3333320617676,10.6666669845581,0,60,3,2,214,88,1,1,1,0,0,10,98
9553,1754,2011-11-06,4,0,1,51.25,8.75,0,60,3,2,214,88,1,1,1,0,0,10,9846.217093,8628.786108,3.72
9554,1754,2011-11-07,1,1,0,17,33,0,50,1,2,214,88,1,1,1,0,0,10,9846.217093,8628.786108,3.729225
9555,1754,2011-11-07,2,1,0,37.8333320617676,22.1666660308838,0,60,3,2,214,88,1,1,1,0,0,10,98
9556,1754,2011-11-07,3,1,0,42.6666679382324,16.8333339691162,0.5,60,3,2,214,88,1,1,1,0,0,10,9
9557,1754,2011-11-07,4,1,0,31.5,28.25,0.25,60,3,2,214,88,1,1,1,0,0,10,9846.217093,8628.786108,3
9558,1754,2011-11-08,1,2,0,20.25,16,3,39.25,3,2,214,88,1,1,1,0,0,10,9846.217093,8628.786108,3.7
9559,1754,2011-11-08,2,2,0,48.25,11.75,0,60,3,2,214,88,1,1,1,0,0,10,9846.217093,8628.786108,3.7
9560,1754,2011-11-08,3,2,0,45,15,0,60,3,2,214,88,1,1,1,0,0,10,9846.217093,8628.786108,3.729225
9561,1754,2011-11-08,4,2,0,50,10,0,60,3,2,214,88,1,1,1,0,0,10,9846.217093,8628.786108,3.729225
9562,1754,2011-11-09,0,3,0,59,1,0,60,1,2,214,88,1,1,1,0,0,10,9846.217093,8628.786108,3.7292258
9563,1754,2011-11-09,1,3,0,38.3333320617676,20.6666660308838,1,60,3,2,214,88,1,1,1,0,0,10,98
9564,1754,2011-11-09,2,3,0,33,27,0,60,3,2,214,88,1,1,1,0,0,10,9846.217093,8628.786108,3.729225
9565,1754,2011-11-09,3,3,0,46,13.6000003814697,0.400000005960464,60,3,2,214,88,1,1,1,0,0,10,
9566,1754,2011-11-09,4,3,0,49,10.3333330154419,0.666666686534882,60,3,2,214,88,1,1,1,0,0,10,
9567,1754,2011-11-10,3,4,0,49,11,0,60,1,2,214,88,1,1,1,0,0,10,9846.217093,8628.786108,3.729225
9568,1754,2011-11-10,4,4,0,47,13,0,60,3,2,214,88,1,1,1,0,0,10,9846.217093,8628.786108,3.729225
9569,1758,2011-10-20,0,4,0,17,38,0,55,2,2,214,79,0,1,0,1,0,12,9886.352755,8558.573162,3.880603
9570,1758,2011-10-20,1,4,0,36,23.6666660308838,0.333333343267441,60,3,2,214,79,0,1,0,1,0,12,
9571,1758,2011-10-20,2,4,0,47.3333320617676,12.3333330154419,0.333333343267441,60,3,2,214
9572,1758,2011-10-20,3,4,0,43.3333320617676,13.6666669845581,3,60,3,2,214,79,0,1,0,1,0,12,98
9573,1758,2011-10-20,4,4,0,54,6,0,60,3,2,214,79,0,1,0,1,0,12,9886.352755,8558.573162,3.8806039
9574,1758,2011-10-21,0,5,0,26.6666660308838,14.6666669845581,1.33333337306976,42.6666679
9575,1758,2011-10-21,1,5,0,43.75,16.25,0,60,3,2,214,79,0,1,0,1,0,12,9886.352755,8558.573162,3.8
9576,1758,2011-10-21,2,5,0,44.6666679382324,15.3333330154419,0,60,3,2,214,79,0,1,0,1,0,12,98
9577,1758,2011-10-21,3,5,0,57,3,0,60,3,2,214,79,0,1,0,1,0,12,9886.352755,8558.573162,3.8806039
9578,1758,2011-10-21,4,5,0,46,14,0,60,3,2,214,79,0,1,0,1,0,12,9886.352755,8558.573162,3.880603
9579,1758,2011-10-22,0,6,1,33,10,4,47,1,2,214,79,0,1,0,1,0,12,9886.352755,8558.573162,3.880603
9580,1758,2011-10-22,1,6,1,34.2000007629395,25.7999992370605,0,60,3,2,214,79,0,1,0,1,0,12,98
9581,1758,2011-10-22,2,6,1,33.5,26.5,0,60,3,2,214,79,0,1,0,1,0,12,9886.352755,8558.573162,3.880
9582,1758,2011-10-22,3,6,1,32.3333320617676,27.6666660308838,0,60,3,2,214,79,0,1,0,1,0,12,98
9583,1758,2011-10-22,4,6,1,52,8,0,60,3,2,214,79,0,1,0,1,0,12,9886.352755,8558.573162,3.8806039
9584,1758,2011-10-23,0,0,1,28,20,1,49,1,2,214,79,0,1,0,1,0,12,9886.352755,8558.573162,3.880603
9585,1758,2011-10-23,1,0,1,40.3333320617676,19.6666660308838,0,60,3,2,214,79,0,1,0,1,0,12,98
9586,1758,2011-10-23,2,0,1,47.5999984741211,12.3999996185303,0,60,3,2,214,79,0,1,0,1,0,12,98
9587,1758,2011-10-23,3,0,1,36.75,20.75,2.5,60,3,2,214,79,0,1,0,1,0,12,9886.352755,8558.573162,3
9588,1758,2011-10-23,4,0,1,53,7,0,60,3,2,214,79,0,1,0,1,0,12,9886.352755,8558.573162,3.8806039
9589,1758,2011-10-24,0,1,0,23,21,1,45,2,2,214,79,0,1,0,1,0,12,9886.352755,8558.573162,3.880603
9590,1758,2011-10-24,1,1,0,38.3333320617676,21.3333339691162,0.333333343267441,60,3,2,214
9591,1758,2011-10-24,2,1,0,52.3333320617676,7.66666650772095,0,60,3,2,214,79,0,1,0,1,0,12,98
9592,1758,2011-10-24,3,1,0,35.4000015258789,23.3999996185303,1.20000004768372,60,3,2,214,
9593,1758,2011-10-24,4,1,0,48,12,0,60,3,2,214,79,0,1,0,1,0,12,9886.352755,8558.573162,3.880603
9594,1758,2011-10-25,4,2,0,51.5,8.5,0,60,2,2,214,79,0,1,0,1,0,12,9886.352755,8558.573162,3.8806
9595,1758,2011-11-10,0,4,0,45,14,1,60,1,2,214,79,0,1,0,1,0,12,9886.352755,8558.573162,3.880603
9596,1758,2011-11-10,1,4,0,25.3333339691162,34.6666679382324,0,60,3,2,214,79,0,1,0,1,0,12,98
9597,1758,2011-11-10,2,4,0,40.3333320617676,19.6666660308838,0,60,3,2,214,79,0,1,0,1,0,12,98
9598,1758,2011-11-10,3,4,0,24,35.6666679382324,0.333333343267441,60,3,2,214,79,0,1,0,1,0,12,
9599,1758,2011-11-10,4,4,0,56,4,0,60,1,2,214,79,0,1,0,1,0,12,9886.352755,8558.573162,3.8806039

9600,1760,2011-10-25,0,2,0,60,0,0,60,1,2,214,67,0,1,0,1,0,12,9841.054413,9468.898964,3.715097
9601,1760,2011-10-25,1,2,0,24,27.2000007629395,0.600000023841858,51.7999992370605,3,2,214
9602,1760,2011-10-25,2,2,0,31,22.6666660308838,6.33333349227905,60,3,2,214,67,0,1,0,1,0,12,9
9603,1760,2011-10-25,3,2,0,32.6666679382324,26.3333339691162,1,60,3,2,214,67,0,1,0,1,0,12,98
9604,1760,2011-10-25,4,2,0,24,33.6666679382324,2.33333325386047,60,3,2,214,67,0,1,0,1,0,12,9
9605,1760,2011-10-26,0,3,0,29,13.6666669845581,0.333333343267441,43,3,2,214,67,0,1,0,1,0,12,
9606,1760,2011-10-26,1,3,0,36.3333320617676,21.3333339691162,2.33333325386047,60,3,2,214,
9607,1760,2011-10-26,2,3,0,28,31.5,0.5,60,3,2,214,67,0,1,0,1,0,12,9841.054413,9468.898964,3.715
9608,1760,2011-10-26,3,3,0,14.3333330154419,42.3333320617676,3.33333325386047,60,3,2,214,
9609,1760,2011-10-26,4,3,0,13.3333330154419,46.6666679382324,0,60,3,2,214,67,0,1,0,1,0,12,98
9610,1760,2011-12-01,1,4,0,18.3333339691162,29.6666660308838,1.33333337306976,49.333332
9611,1760,2011-12-01,2,4,0,38,21.3333339691162,0.666666686534882,60,3,2,214,67,0,1,0,1,0,12,
9612,1760,2011-12-01,3,4,0,25.3333339691162,29,5.66666650772095,60,3,2,214,67,0,1,0,1,0,12,9
9613,1760,2011-12-01,4,4,0,5.66666650772095,48.6666679382324,5.66666650772095,60,3,2,214,
9614,1760,2011-12-02,0,5,0,14.6666669845581,25.6666660308838,1.33333337306976,41.666667
9615,1760,2011-12-02,1,5,0,20,40,0,60,1,2,214,67,0,1,0,1,0,12,9841.054413,9468.898964,3.715097
9616,1760,2011-12-02,2,5,0,28,29.5,2.5,60,2,2,214,67,0,1,0,1,0,12,9841.054413,9468.898964,3.715
9617,1760,2011-12-02,3,5,0,37.6666679382324,20,2.33333325386047,60,3,2,214,67,0,1,0,1,0,12,9
9618,1760,2011-12-02,4,5,0,28.3333339691162,30.3333339691162,1.33333337306976,60,3,2,214,
9619,1760,2011-12-04,1,0,1,18.25,31.5,3.5,53.25,3,2,214,67,0,1,0,1,0,12,9841.054413,9468.898964
9620,1760,2011-12-04,2,0,1,23,33,4,60,3,2,214,67,0,1,0,1,0,12,9841.054413,9468.898964,3.715097
9621,1760,2011-12-04,3,0,1,32.6666679382324,27.3333339691162,0,60,3,2,214,67,0,1,0,1,0,12,98
9622,1760,2011-12-04,4,0,1,9.33333301544189,49.3333320617676,1.33333337306976,60,3,2,214,
9623,1760,2011-12-05,2,1,0,36.6666679382324,22,1.33333337306976,60,3,2,214,67,0,1,0,1,0,12,9
9624,1760,2011-12-05,3,1,0,18.6666660308838,38,3.33333325386047,60,3,2,214,67,0,1,0,1,0,12,9
9625,1760,2011-12-05,4,1,0,12,41,7,60,3,2,214,67,0,1,0,1,0,12,9841.054413,9468.898964,3.715097
9626,1763,2010-10-07,0,4,0,0,4,0,4,1,2,214,71,1,0,0,1,0,12,10097.91744,8850.796148,4.402642633
9627,1763,2010-10-07,1,4,0,24.3333339691162,31.3333339691162,4.33333349227905,60,3,2,214,
9628,1763,2010-10-07,2,4,0,18.3333339691162,41.3333320617676,0.333333343267441,60,3,2,214
9629,1763,2010-10-07,3,4,0,28.6666660308838,30.6666660308838,0.666666686534882,60,3,2,214
9630,1763,2010-10-07,4,4,0,27.6666660308838,31,1.33333337306976,60,3,2,214,71,1,0,0,1,0,12,1
9631,1763,2010-10-08,0,5,0,48,11,1,60,1,2,214,71,1,0,0,1,0,12,10097.91744,8850.796148,4.402642
9632,1763,2010-10-08,1,5,0,20.3333339691162,30,9.66666698455811,60,3,2,214,71,1,0,0,1,0,12,1
9633,1763,2010-10-08,2,5,0,20.8333339691162,38.1666679382324,1,60,3,2,214,71,1,0,0,1,0,12,10
9634,1763,2010-10-08,3,5,0,16.6666660308838,42.6666679382324,0.666666686534882,60,3,2,214
9635,1763,2010-10-08,4,5,0,35.3333320617676,24.6666660308838,0,60,3,2,214,71,1,0,0,1,0,12,10
9636,1763,2010-10-09,1,6,1,17.6666660308838,33.6666679382324,7.66666650772095,59,3,2,214,
9637,1763,2010-10-09,2,6,1,33,27,0,60,3,2,214,71,1,0,0,1,0,12,10097.91744,8850.796148,4.402642
9638,1763,2010-10-09,3,6,1,30.2000007629395,29.3999996185303,0.400000005960464,60,3,2,214
9639,1763,2010-10-09,4,6,1,34.6666679382324,25,0.333333343267441,60,3,2,214,71,1,0,0,1,0,12,
9640,1763,2010-10-10,1,0,1,14,33.5,1.5,49,2,2,214,71,1,0,0,1,0,12,10097.91744,8850.796148,4.402
9641,1763,2010-10-10,2,0,1,33,27,0,60,3,2,214,71,1,0,0,1,0,12,10097.91744,8850.796148,4.402642
9642,1763,2010-10-10,3,0,1,43.25,16.5,0.25,60,3,2,214,71,1,0,0,1,0,12,10097.91744,8850.796148,4
9643,1763,2010-10-10,4,0,1,30.3999996185303,29.6000003814697,0,60,3,2,214,71,1,0,0,1,0,12,10
9644,1763,2010-10-11,0,1,0,0,3,0,3,1,2,214,71,1,0,0,1,0,12,10097.91744,8850.796148,4.402642633
9645,1763,2010-10-11,1,1,0,24,35.3333320617676,0.666666686534882,60,3,2,214,71,1,0,0,1,0,12,
9646,1763,2010-10-11,2,1,0,24.5,26,0.5,51,2,2,214,71,1,0,0,1,0,12,10097.91744,8850.796148,4.402
9647,1763,2010-10-11,3,1,0,32.3333320617676,20.3333339691162,0.333333343267441,53,3,2,214
9648,1763,2010-10-11,4,1,0,34.5,25.5,0,60,3,2,214,71,1,0,0,1,0,12,10097.91744,8850.796148,4.402
9649,1763,2011-12-05,0,1,0,4,3,0,7,1,2,214,71,1,0,0,1,0,12,10097.91744,8850.796148,4.402642633

9650,1763,2011-12-05,1,1,0,30,28,2,60,1,2,214,71,1,0,0,1,0,12,10097.91744,8850.796148,4.402642
9651,1763,2011-12-06,0,2,0,1,0,2,3,1,2,214,71,1,0,0,1,0,12,10097.91744,8850.796148,4.40264263
9652,1763,2011-12-06,1,2,0,24,33.5,2.5,60,2,2,214,71,1,0,0,1,0,12,10097.91744,8850.796148,4.402
9653,1763,2011-12-06,2,2,0,34,26,0,60,3,2,214,71,1,0,0,1,0,12,10097.91744,8850.796148,4.402642
9654,1763,2011-12-06,3,2,0,37,22.6666660308838,0.333333343267441,60,3,2,214,71,1,0,0,1,0,12,
9655,1763,2011-12-06,4,2,0,18,21.3333339691162,1,40.3333320617676,3,2,214,71,1,0,0,1,0,12,10
9656,1768,2010-10-08,0,5,0,12,24,5,41,2,2,214,65,1,1,1,1,0,10,9345.125699,7251.239604,4.627885
9657,1768,2010-10-08,1,5,0,30.3333339691162,29,0.666666686534882,60,3,2,214,65,1,1,1,1,0,10,
9658,1768,2010-10-08,2,5,0,16,40.3333320617676,3.66666674613953,60,3,2,214,65,1,1,1,1,0,10,9
9659,1768,2010-10-08,3,5,0,14.6666669845581,33.3333320617676,12,60,3,2,214,65,1,1,1,1,0,10,9
9660,1768,2010-10-08,4,5,0,41,19,0,60,3,2,214,65,1,1,1,1,0,10,9345.125699,7251.239604,4.627885
9661,1768,2010-10-09,0,6,1,0,25,7,32,1,2,214,65,1,1,1,1,0,10,9345.125699,7251.239604,4.6278858
9662,1768,2010-10-09,1,6,1,14,42,4,60,3,2,214,65,1,1,1,1,0,10,9345.125699,7251.239604,4.627885
9663,1768,2010-10-09,2,6,1,23.6666660308838,34.5,1.83333337306976,60,3,2,214,65,1,1,1,1,0,10
9664,1768,2010-10-09,3,6,1,31.6666660308838,22.3333339691162,6,60,3,2,214,65,1,1,1,1,0,10,93
9665,1768,2010-10-09,4,6,1,43.3333320617676,15,1.66666662693024,60,3,2,214,65,1,1,1,1,0,10,9
9666,1768,2010-10-10,0,0,1,11,30,8.5,49.5,2,2,214,65,1,1,1,1,0,10,9345.125699,7251.239604,4.627
9667,1768,2010-10-10,1,0,1,21,28.3333339691162,0.333333343267441,49.6666679382324,3,2,214
9668,1768,2010-10-10,2,0,1,13,23,5,41,1,2,214,65,1,1,1,1,0,10,9345.125699,7251.239604,4.627885
9669,1768,2010-10-10,3,0,1,24.3999996185303,27.2000007629395,8.39999961853027,60,3,2,214,
9670,1768,2010-10-10,4,0,1,43,16.6666660308838,0.333333343267441,60,3,2,214,65,1,1,1,1,0,10,
9671,1768,2010-10-11,0,1,0,27,30,3,60,1,2,214,65,1,1,1,1,0,10,9345.125699,7251.239604,4.627885
9672,1768,2010-10-11,1,1,0,6.5,48,5.5,60,2,2,214,65,1,1,1,1,0,10,9345.125699,7251.239604,4.6278
9673,1768,2010-10-11,3,1,0,31,29,0,60,1,2,214,65,1,1,1,1,0,10,9345.125699,7251.239604,4.627885
9674,1768,2010-10-11,4,1,0,28.6666660308838,30.3333339691162,1,60,3,2,214,65,1,1,1,1,0,10,93
9675,1768,2010-10-12,0,2,0,8,24,1,33,2,2,214,65,1,1,1,1,0,10,9345.125699,7251.239604,4.6278858
9676,1768,2010-10-12,1,2,0,19,37,4,60,3,2,214,65,1,1,1,1,0,10,9345.125699,7251.239604,4.627885
9677,1768,2010-10-12,2,2,0,31,27.3333339691162,1.66666662693024,60,3,2,214,65,1,1,1,1,0,10,9
9678,1768,2010-10-12,3,2,0,25.3333339691162,29.3333339691162,5.33333349227905,60,3,2,214,
9679,1768,2010-10-12,4,2,0,34.3333320617676,24.3333339691162,1.33333337306976,60,3,2,214,
9680,1768,2010-10-13,0,3,0,10.5,31.5,6,48,2,2,214,65,1,1,1,1,0,10,9345.125699,7251.239604,4.627
9681,1768,2010-10-13,1,3,0,3,43,14,60,3,2,214,65,1,1,1,1,0,10,9345.125699,7251.239604,4.627885
9682,1768,2010-10-13,2,3,0,42.3333320617676,17.6666660308838,0,60,3,2,214,65,1,1,1,1,0,10,93
9683,1768,2010-10-13,3,3,0,30.3333339691162,25.3333339691162,4.33333349227905,60,3,2,214,
9684,1768,2010-10-13,4,3,0,46.3333320617676,13.6666669845581,0,60,3,2,214,65,1,1,1,1,0,10,93
9685,1777,2010-10-11,0,1,0,15,24,21,60,1,2,214,67,0,0,0,1,0,12,10302.30577,8391.520881,4.24381
9686,1777,2010-10-11,1,1,0,1.33333337306976,37,21.6666660308838,60,3,2,214,67,0,0,0,1,0,12,1
9687,1777,2010-10-11,2,1,0,16,38.6666679382324,5.33333349227905,60,3,2,214,67,0,0,0,1,0,12,1
9688,1777,2010-10-11,3,1,0,40,17.5,2.5,60,2,2,214,67,0,0,0,1,0,12,10302.30577,8391.520881,4.243
9689,1777,2010-10-12,0,2,0,8.33333301544189,31.6666660308838,9,49,3,2,214,67,0,0,0,1,0,12,10
9690,1777,2010-10-12,1,2,0,12.6666669845581,45.6666679382324,1.66666662693024,60,3,2,214,
9691,1777,2010-10-12,2,2,0,10.3333330154419,47.6666679382324,2,60,3,2,214,67,0,0,0,1,0,12,10
9692,1777,2010-10-12,3,2,0,54,6,0,60,1,2,214,67,0,0,0,1,0,12,10302.30577,8391.520881,4.2438141
9693,1777,2010-10-12,4,2,0,42.6666679382324,8.33333301544189,0.333333343267441,51.333333
9694,1777,2010-10-13,0,3,0,6.33333349227905,26.3333339691162,8.33333301544189,41,3,2,214,
9695,1777,2010-10-13,1,3,0,15.3333330154419,43.6666679382324,1,60,3,2,214,67,0,0,0,1,0,12,10
9696,1777,2010-10-13,2,3,0,13,46.6666679382324,0.333333343267441,60,3,2,214,67,0,0,0,1,0,12,
9697,1777,2010-10-13,3,3,0,2.75,57.25,0,60,3,2,214,67,0,0,0,1,0,12,10302.30577,8391.520881,4.24
9698,1777,2010-10-13,4,3,0,22,38,0,60,3,2,214,67,0,0,0,1,0,12,10302.30577,8391.520881,4.243814
9699,1777,2010-10-14,0,4,0,28,25.6666660308838,6.33333349227905,60,3,2,214,67,0,0,0,1,0,12,1

9700,1777,2010-10-14,1,4,0,11,44,5,60,3,2,214,67,0,0,0,1,0,12,10302.30577,8391.520881,4.243814
9701,1777,2010-10-14,2,4,0,41.3333320617676,18.3333339691162,0.333333343267441,60,3,2,214
9702,1777,2010-10-14,3,4,0,24.25,33.75,2,60,3,2,214,67,0,0,0,1,0,12,10302.30577,8391.520881,4.2
9703,1777,2010-10-14,4,4,0,22.5,32,0.5,55,2,2,214,67,0,0,0,1,0,12,10302.30577,8391.520881,4.243
9704,1777,2010-10-20,0,3,0,3.66666674613953,32.3333320617676,24,60,3,2,214,67,0,0,0,1,0,12,1
9705,1777,2010-10-20,1,3,0,4.33333349227905,35.3333320617676,20.3333339691162,60,3,2,214,
9706,1777,2010-10-20,2,3,0,26,30.6666660308838,3.33333325386047,60,3,2,214,67,0,0,0,1,0,12,1
9707,1777,2010-10-20,3,3,0,30.5,29.5,0,60,3,2,214,67,0,0,0,1,0,12,10302.30577,8391.520881,4.243
9708,1777,2010-10-20,4,3,0,21,13.5,0,34.5,2,2,214,67,0,0,0,1,0,12,10302.30577,8391.520881,4.243
9709,1777,2010-10-21,0,4,0,4.5,33.25,22.25,60,3,2,214,67,0,0,0,1,0,12,10302.30577,8391.520881,4
9710,1777,2010-10-21,1,4,0,13.3333330154419,36,10.6666669845581,60,3,2,214,67,0,0,0,1,0,12,1
9711,1777,2010-10-21,2,4,0,21.3333339691162,37.3333320617676,1.33333337306976,60,3,2,214,
9712,1777,2010-10-21,3,4,0,38.6666679382324,19,2.33333325386047,60,3,2,214,67,0,0,0,1,0,12,1
9713,1777,2010-10-21,4,4,0,55,4.33333349227905,0,59.3333320617676,3,2,214,67,0,0,0,1,0,12,10
9714,1777,2010-10-22,0,5,0,0,32,28,60,2,2,214,67,0,0,0,1,0,12,10302.30577,8391.520881,4.243814
9715,1777,2010-10-22,1,5,0,3.66666674613953,37.3333320617676,19,60,3,2,214,67,0,0,0,1,0,12,1
9716,1777,2010-10-22,2,5,0,31,27.3333339691162,1.66666662693024,60,3,2,214,67,0,0,0,1,0,12,1
9717,1777,2010-10-22,3,5,0,26.6666660308838,31,2.33333325386047,60,3,2,214,67,0,0,0,1,0,12,1
9718,1777,2010-10-22,4,5,0,20,3,0,23,1,2,214,67,0,0,0,1,0,12,10302.30577,8391.520881,4.2438141
9719,1780,2010-10-22,0,5,0,19,2,0,21,1,2,214,81,1,1,1,0,0,9,10311.37256,8519.975953,3.9551960
9720,1780,2010-10-22,1,5,0,27.5,31,0.5,59,2,2,214,81,1,1,1,0,0,9,10311.37256,8519.975953,3.9551
9721,1780,2010-10-22,2,5,0,33,27,0,60,1,2,214,81,1,1,1,0,0,9,10311.37256,8519.975953,3.955196
9722,1780,2010-10-23,0,6,1,35.5,0,0.5,36,2,2,214,81,1,1,1,0,0,9,10311.37256,8519.975953,3.95519
9723,1780,2010-10-23,1,6,1,31.3333339691162,28,0.666666686534882,60,3,2,214,81,1,1,1,0,0,9,1
9724,1780,2010-10-23,2,6,1,49.2000007629395,10.8000001907349,0,60,3,2,214,81,1,1,1,0,0,9,103
9725,1780,2010-10-23,3,6,1,40,20,0,60,3,2,214,81,1,1,1,0,0,9,10311.37256,8519.975953,3.955196
9726,1780,2010-10-23,4,6,1,29.3333339691162,30.6666660308838,0,60,3,2,214,81,1,1,1,0,0,9,103
9727,1780,2010-10-24,0,0,1,10.5,31,1.5,43,2,2,214,81,1,1,1,0,0,9,10311.37256,8519.975953,3.9551
9728,1780,2010-10-24,1,0,1,32.3333320617676,27.6666660308838,0,60,3,2,214,81,1,1,1,0,0,9,103
9729,1780,2010-10-24,2,0,1,44.6666679382324,15,0.333333343267441,60,3,2,214,81,1,1,1,0,0,9,1
9730,1780,2010-10-24,3,0,1,36.4000015258789,23.6000003814697,0,60,3,2,214,81,1,1,1,0,0,9,103
9731,1780,2010-10-24,4,0,1,35.3333320617676,24,0.666666686534882,60,3,2,214,81,1,1,1,0,0,9,1
9732,1780,2010-10-25,0,1,0,31,24.5,1.5,57,2,2,214,81,1,1,1,0,0,9,10311.37256,8519.975953,3.9551
9733,1780,2010-10-25,1,1,0,26.3333339691162,33.3333320617676,0.333333343267441,60,3,2,214
9734,1780,2010-10-25,2,1,0,24.6666660308838,35.3333320617676,0,60,3,2,214,81,1,1,1,0,0,9,103
9735,1780,2010-10-25,3,1,0,32.25,27,0.75,60,3,2,214,81,1,1,1,0,0,9,10311.37256,8519.975953,3.95
9736,1780,2010-10-25,4,1,0,29.5,30,0.5,60,3,2,214,81,1,1,1,0,0,9,10311.37256,8519.975953,3.9551
9737,1780,2010-10-26,0,2,0,40.5,1,0.5,42,2,2,214,81,1,1,1,0,0,9,10311.37256,8519.975953,3.95519
9738,1780,2010-10-26,1,2,0,17,24.6666660308838,0,41.6666679382324,3,2,214,81,1,1,1,0,0,9,103
9739,1780,2010-10-26,2,2,0,45.6666679382324,14.3333330154419,0,60,3,2,214,81,1,1,1,0,0,9,103
9740,1780,2010-10-26,3,2,0,35.6666679382324,24.3333339691162,0,60,3,2,214,81,1,1,1,0,0,9,103
9741,1780,2010-10-26,4,2,0,42.7999992370605,16.6000003814697,0.600000023841858,60,3,2,214
9742,1780,2011-02-24,0,4,0,1,6,1,8,1,2,214,81,1,1,1,0,0,9,10311.37256,8519.975953,3.955196028,
9743,1780,2011-02-24,1,4,0,33,27,0,60,3,2,214,81,1,1,1,0,0,9,10311.37256,8519.975953,3.955196
9744,1780,2011-02-24,2,4,0,44.5,15.5,0,60,3,2,214,81,1,1,1,0,0,9,10311.37256,8519.975953,3.9551
9745,1780,2011-02-24,3,4,0,31.6666660308838,28.3333339691162,0,60,3,2,214,81,1,1,1,0,0,9,103
9746,1780,2011-02-24,4,4,0,20,12.5,1,33.5,2,2,214,81,1,1,1,0,0,9,10311.37256,8519.975953,3.9551
9747,1780,2011-02-25,0,5,0,6,33,1,40,1,2,214,81,1,1,1,0,0,9,10311.37256,8519.975953,3.9551960
9748,1780,2011-02-25,1,5,0,21.6666660308838,38.3333320617676,0,60,3,2,214,81,1,1,1,0,0,9,103
9749,1780,2011-02-25,2,5,0,41.6666679382324,18.3333339691162,0,60,3,2,214,81,1,1,1,0,0,9,103

9750,1780,2011-02-25,3,5,0,37.4000015258789,22.3999996185303,0.200000002980232,60,3,2,21
9751,1780,2011-02-25,4,5,0,28.5,19.5,0,48,2,2,214,81,1,1,1,0,0,9,10311.37256,8519.975953,3.955
9752,1780,2011-02-26,0,6,1,15.5,28.5,0.5,44.5,2,2,214,81,1,1,1,0,0,9,10311.37256,8519.975953,3.
9753,1780,2011-02-26,1,6,1,32.6666679382324,27.3333339691162,0,60,3,2,214,81,1,1,1,0,0,9,103
9754,1780,2011-02-26,2,6,1,34.6666679382324,25.3333339691162,0,60,3,2,214,81,1,1,1,0,0,9,103
9755,1780,2011-02-26,3,6,1,36.75,23.25,0,60,3,2,214,81,1,1,1,0,0,9,10311.37256,8519.975953,3.9
9756,1780,2011-02-26,4,6,1,27,28,0.333333343267441,55.3333320617676,3,2,214,81,1,1,1,0,0,9,1
9757,1780,2011-02-27,0,0,1,14.5,22,1,37.5,2,2,214,81,1,1,1,0,0,9,10311.37256,8519.975953,3.955
9758,1780,2011-02-27,1,0,1,33.3333320617676,26,0.666666686534882,60,3,2,214,81,1,1,1,0,0,9,1
9759,1780,2011-02-27,2,0,1,43.3333320617676,16.6666660308838,0,60,3,2,214,81,1,1,1,0,0,9,103
9760,1780,2011-02-27,3,0,1,31,29,0,60,3,2,214,81,1,1,1,0,0,9,10311.37256,8519.975953,3.955196
9761,1780,2011-02-27,4,0,1,29,10,0,39,2,2,214,81,1,1,1,0,0,9,10311.37256,8519.975953,3.955196
9762,1780,2011-03-01,0,2,0,17,13.5,0.5,31,2,2,214,81,1,1,1,0,0,9,10311.37256,8519.975953,3.955
9763,1780,2011-03-01,1,2,0,32.3333320617676,27.3333339691162,0.333333343267441,60,3,2,21
9764,1780,2011-03-01,2,2,0,44,16,0,60,3,2,214,81,1,1,1,0,0,9,10311.37256,8519.975953,3.955196
9765,1780,2011-03-01,3,2,0,38,22,0,60,3,2,214,81,1,1,1,0,0,9,10311.37256,8519.975953,3.955196
9766,1780,2011-03-01,4,2,0,28.5,8.5,0.5,37.5,2,2,214,81,1,1,1,0,0,9,10311.37256,8519.975953,3.9
9767,1780,2011-03-02,0,3,0,50.5,0.5,0,51,2,2,214,81,1,1,1,0,0,9,10311.37256,8519.975953,3.9551
9768,1780,2011-03-02,1,3,0,35.2000007629395,24.6000003814697,0.200000002980232,60,3,2,21
9769,1780,2011-03-02,2,3,0,40.3333320617676,19,0.666666686534882,60,3,2,214,81,1,1,1,0,0,9,1
9770,1780,2011-03-02,3,3,0,38,22,0,60,3,2,214,81,1,1,1,0,0,9,10311.37256,8519.975953,3.955196
9771,1780,2011-03-02,4,3,0,1,2,0,3,1,2,214,81,1,1,1,0,0,9,10311.37256,8519.975953,3.955196028,
9772,1851,2011-02-27,0,0,1,59,0,1,60,1,2,201,75,1,1,1,0,0,7,14723.99204,25206.33992,3.88656081
9773,1851,2011-02-27,1,0,1,25,33,2,60,1,2,201,75,1,1,1,0,0,7,14723.99204,25206.33992,3.886560
9774,1851,2011-02-28,0,1,0,15,24,1,40,1,2,201,75,1,1,1,0,0,7,14723.99204,25206.33992,3.886560
9775,1851,2011-02-28,1,1,0,17.2000007629395,41.5999984741211,1.20000004768372,60,3,2,201,
9776,1851,2011-02-28,2,1,0,46,14,0,60,3,2,201,75,1,1,1,0,0,7,14723.99204,25206.33992,3.886560
9777,1851,2011-02-28,3,1,0,24.6666660308838,32.3333320617676,3,60,3,2,201,75,1,1,1,0,0,7,147
9778,1851,2011-02-28,4,1,0,44.3333320617676,15.3333330154419,0.333333343267441,60,3,2,20
9779,1851,2011-05-01,0,0,1,6,27.5,2.5,36,2,2,201,75,1,1,1,0,0,7,14723.99204,25206.33992,3.8865
9780,1851,2011-05-01,1,0,1,26.3333339691162,27.3333339691162,6.33333349227905,60,3,2,201,
9781,1851,2011-05-01,2,0,1,41.7999992370605,16.7999992370605,1.39999997615814,60,3,2,201,
9782,1851,2011-05-01,3,0,1,47,12.6666669845581,0.333333343267441,60,3,2,201,75,1,1,1,0,0,7,1
9783,1851,2011-05-01,4,0,1,44.3333320617676,15.3333330154419,0.333333343267441,60,3,2,20
9784,1851,2011-05-02,0,1,0,19,13.5,0,32.5,2,2,201,75,1,1,1,0,0,7,14723.99204,25206.33992,3.886
9785,1851,2011-05-02,1,1,0,23,28,9,60,3,2,201,75,1,1,1,0,0,7,14723.99204,25206.33992,3.886560
9786,1851,2011-05-02,2,1,0,32.25,22.75,5,60,3,2,201,75,1,1,1,0,0,7,14723.99204,25206.33992,3.88
9787,1851,2011-05-02,3,1,0,34,23.6666660308838,2.33333325386047,60,3,2,201,75,1,1,1,0,0,7,14
9788,1851,2011-05-02,4,1,0,51.3333320617676,8.66666698455811,0,60,3,2,201,75,1,1,1,0,0,7,147
9789,1851,2011-05-03,0,2,0,31,21,0.5,52.5,2,2,201,75,1,1,1,0,0,7,14723.99204,25206.33992,3.886
9790,1851,2011-05-03,1,2,0,29.6666660308838,30,0.333333343267441,60,3,2,201,75,1,1,1,0,0,7,1
9791,1851,2011-05-03,2,2,0,21.6666660308838,38,0.333333343267441,60,3,2,201,75,1,1,1,0,0,7,1
9792,1851,2011-05-03,3,2,0,41,18,1,60,3,2,201,75,1,1,1,0,0,7,14723.99204,25206.33992,3.886560
9793,1851,2011-05-03,4,2,0,32.6666679382324,26.3333339691162,1,60,3,2,201,75,1,1,1,0,0,7,147
9794,1851,2011-05-04,0,3,0,24,30,1,55,1,2,201,75,1,1,1,0,0,7,14723.99204,25206.33992,3.886560
9795,1851,2011-05-04,1,3,0,17,41.6666679382324,1.33333337306976,60,3,2,201,75,1,1,1,0,0,7,14
9796,1851,2011-05-04,2,3,0,21,38.3333320617676,0.666666686534882,60,3,2,201,75,1,1,1,0,0,7,1
9797,1851,2011-05-04,3,3,0,25,33,2,60,3,2,201,75,1,1,1,0,0,7,14723.99204,25206.33992,3.886560
9798,1851,2011-05-04,4,3,0,48.3333320617676,10.3333330154419,1.33333337306976,60,3,2,201,
9799,1851,2011-05-05,0,4,0,16.5,37.5,1.5,55.5,2,2,201,75,1,1,1,0,0,7,14723.99204,25206.33992,3.

9800,1851,2011-05-05,1,4,0,6,45.3333320617676,8.66666698455811,60,3,2,201,75,1,1,1,0,0,7,147
9801,1851,2011-05-05,2,4,0,42.6666679382324,12.6666669845581,4.66666650772095,60,3,2,201,
9802,1851,2011-05-05,3,4,0,31.3333339691162,27,1.66666662693024,60,3,2,201,75,1,1,1,0,0,7,14
9803,1851,2011-05-05,4,4,0,39.7999992370605,20,0.200000002980232,60,3,2,201,75,1,1,1,0,0,7,1
9804,1851,2011-05-06,4,5,0,55,5,0,60,1,2,201,75,1,1,1,0,0,7,14723.99204,25206.33992,3.88656081
9805,1853,2010-11-01,0,1,0,12.5,18.5,0.5,31.5,2,2,201,77,0,0,0,0,0,12,14599.34199,25206.33992,3
9806,1853,2010-11-01,1,1,0,43,16.6666660308838,0.333333343267441,60,3,2,201,77,0,0,0,0,0,12,
9807,1853,2010-11-01,2,1,0,40.3333320617676,16.6666660308838,3,60,3,2,201,77,0,0,0,0,0,12,14
9808,1853,2010-11-01,3,1,0,26.25,26.25,7.5,60,3,2,201,77,0,0,0,0,0,12,14599.34199,25206.33992,3
9809,1853,2010-11-01,4,1,0,51.3333320617676,8,0.666666686534882,60,3,2,201,77,0,0,0,0,0,12,1
9810,1853,2010-11-02,0,2,0,15,40,0.5,55.5,2,2,201,77,0,0,0,0,0,12,14599.34199,25206.33992,3.831
9811,1853,2010-11-02,1,2,0,53.6666679382324,6,0.333333343267441,60,3,2,201,77,0,0,0,0,0,12,1
9812,1853,2010-11-02,2,2,0,23.3333339691162,35,1.66666662693024,60,3,2,201,77,0,0,0,0,0,12,1
9813,1853,2010-11-02,3,2,0,40.75,18.5,0.75,60,3,2,201,77,0,0,0,0,0,12,14599.34199,25206.33992,3
9814,1853,2010-11-02,4,2,0,52,7.66666650772095,0.333333343267441,60,3,2,201,77,0,0,0,0,0,12,
9815,1853,2010-11-03,4,3,0,50.3333320617676,9.33333301544189,0.333333343267441,60,3,2,201
9816,1853,2011-01-26,0,3,0,13.5,26.5,0.5,40.5,2,2,201,77,0,0,0,0,0,12,14599.34199,25206.33992,3
9817,1853,2011-01-26,1,3,0,21,16,0.5,37.5,2,2,201,77,0,0,0,0,0,12,14599.34199,25206.33992,3.831
9818,1853,2011-01-26,2,3,0,28.5,31.25,0.25,60,3,2,201,77,0,0,0,0,0,12,14599.34199,25206.33992,3
9819,1853,2011-01-26,3,3,0,28.3333339691162,30.3333339691162,1.33333337306976,60,3,2,201,
9820,1853,2011-01-26,4,3,0,41,18,0,59,3,2,201,77,0,0,0,0,0,12,14599.34199,25206.33992,3.83171
9821,1853,2011-01-27,0,4,0,40,20,0,60,1,2,201,77,0,0,0,0,0,12,14599.34199,25206.33992,3.83171
9822,1853,2011-01-27,1,4,0,35,22.6666660308838,2.33333325386047,60,3,2,201,77,0,0,0,0,0,12,1
9823,1853,2011-01-27,2,4,0,41.5999984741211,18.2000007629395,0.200000002980232,60,3,2,201
9824,1853,2011-01-27,3,4,0,38.3333320617676,21.3333339691162,0.333333343267441,60,3,2,201
9825,1853,2011-01-27,4,4,0,50,9.33333301544189,0.666666686534882,60,3,2,201,77,0,0,0,0,0,12,
9826,1853,2011-01-28,0,5,0,40,1.66666662693024,0,41.6666679382324,3,2,201,77,0,0,0,0,0,12,14
9827,1853,2011-01-28,1,5,0,16.3333339691162,39.3333320617676,4.33333349227905,60,3,2,201,
9828,1853,2011-01-28,2,5,0,25.5,16.5,0.5,42.5,2,2,201,77,0,0,0,0,0,12,14599.34199,25206.33992,3
9829,1853,2011-01-28,3,5,0,45.6666679382324,14.3333330154419,0,60,3,2,201,77,0,0,0,0,0,12,14
9830,1853,2011-01-28,4,5,0,47,12.3333330154419,0.666666686534882,60,3,2,201,77,0,0,0,0,0,12,
9831,1853,2011-01-29,0,6,1,21.5,21,0,42.5,2,2,201,77,0,0,0,0,0,12,14599.34199,25206.33992,3.831
9832,1853,2011-01-29,1,6,1,39.6666679382324,19.3333339691162,1,60,3,2,201,77,0,0,0,0,0,12,14
9833,1853,2011-01-29,2,6,1,34,24.6666660308838,1.33333337306976,60,3,2,201,77,0,0,0,0,0,12,1
9834,1853,2011-01-29,3,6,1,36.25,20.75,2.25,59.25,3,2,201,77,0,0,0,0,0,12,14599.34199,25206.33
9835,1853,2011-01-29,4,6,1,52.3333320617676,7.33333349227905,0.333333343267441,60,3,2,201
9836,1853,2011-02-01,0,2,0,28.6666660308838,16.3333339691162,0.666666686534882,45.66666
9837,1853,2011-02-01,1,2,0,35.25,11.25,0.5,47,3,2,201,77,0,0,0,0,0,12,14599.34199,25206.33992,3
9838,1853,2011-02-01,2,2,0,37.5,12.5,0.5,50.5,2,2,201,77,0,0,0,0,0,12,14599.34199,25206.33992,3
9839,1853,2011-02-01,3,2,0,52,7.66666650772095,0.333333343267441,60,3,2,201,77,0,0,0,0,0,12,
9840,1853,2011-02-01,4,2,0,48.3333320617676,11.3333330154419,0.333333343267441,60,3,2,201
9841,1853,2011-04-30,0,6,1,20.3333339691162,23.3333339691162,1.33333337306976,45,3,2,201,
9842,1853,2011-04-30,1,6,1,25.3333339691162,32.3333320617676,2.33333325386047,60,3,2,201,
9843,1853,2011-04-30,2,6,1,44,15.3333330154419,0.666666686534882,60,3,2,201,77,0,0,0,0,0,12,
9844,1853,2011-04-30,3,6,1,39,21,0,60,3,2,201,77,0,0,0,0,0,12,14599.34199,25206.33992,3.83171
9845,1853,2011-04-30,4,6,1,44.25,15,0.75,60,3,2,201,77,0,0,0,0,0,12,14599.34199,25206.33992,3.8
9846,1853,2011-05-06,0,5,0,21,20.5,0.5,42,2,2,201,77,0,0,0,0,0,12,14599.34199,25206.33992,3.831
9847,1853,2011-05-06,1,5,0,42,17.6666660308838,0.333333343267441,60,3,2,201,77,0,0,0,0,0,12,
9848,1853,2011-05-06,2,5,0,49.3333320617676,10.6666669845581,0,60,3,2,201,77,0,0,0,0,0,12,14
9849,1853,2011-05-06,3,5,0,43.3333320617676,16.3333339691162,0.333333343267441,60,3,2,201

9850,1853,2011-05-06,4,5,0,51.5,8.5,0,60,2,2,201,77,0,0,0,0,0,12,14599.34199,25206.33992,3.831
9851,1857,2010-10-29,0,5,0,26,14,0,40,1,2,201,71,1,1,0,1,0,10,15171.35669,32623.25073,4.24090
9852,1857,2010-10-29,1,5,0,23,31,6,60,3,2,201,71,1,1,0,1,0,10,15171.35669,32623.25073,4.24090
9853,1857,2010-10-29,2,5,0,19.3333339691162,36.6666679382324,4,60,3,2,201,71,1,1,0,1,0,10,15
9854,1857,2010-10-29,3,5,0,37.3333320617676,22.3333339691162,0.333333343267441,60,3,2,20
9855,1857,2010-10-29,4,5,0,35.6666679382324,24,0.333333343267441,60,3,2,201,71,1,1,0,1,0,10,
9856,1857,2010-10-30,0,6,1,22,22,0,44,1,2,201,71,1,1,0,1,0,10,15171.35669,32623.25073,4.24090
9857,1857,2010-10-30,1,6,1,12,47.6666679382324,0.333333343267441,60,3,2,201,71,1,1,0,1,0,10,
9858,1857,2010-10-30,2,6,1,27.3333339691162,32.1666679382324,0.5,60,3,2,201,71,1,1,0,1,0,10,
9859,1857,2010-10-30,3,6,1,12.6666669845581,33.3333320617676,0.666666686534882,46.66666
9860,1857,2010-10-30,4,6,1,22.6666660308838,19.3333339691162,0.666666686534882,42.66666
9861,1857,2010-10-31,0,0,1,19.5,28,0,47.5,2,2,201,71,1,1,0,1,0,10,15171.35669,32623.25073,4.24
9862,1857,2010-10-31,1,0,1,13,44.3333320617676,2.66666674613953,60,3,2,201,71,1,1,0,1,0,10,1
9863,1857,2010-10-31,2,0,1,8,49.6666679382324,2.33333325386047,60,3,2,201,71,1,1,0,1,0,10,15
9864,1857,2010-10-31,3,0,1,33.6666679382324,26,0.333333343267441,60,3,2,201,71,1,1,0,1,0,10,
9865,1857,2010-10-31,4,0,1,28,32,0,60,3,2,201,71,1,1,0,1,0,10,15171.35669,32623.25073,4.24090
9866,1857,2010-11-03,0,3,0,40,19,1,60,1,2,201,71,1,1,0,1,0,10,15171.35669,32623.25073,4.24090
9867,1857,2010-11-03,1,3,0,17.3333339691162,42.3333320617676,0.333333343267441,60,3,2,20
9868,1857,2010-11-03,2,3,0,32,27.6666660308838,0.333333343267441,60,3,2,201,71,1,1,0,1,0,10,
9869,1857,2010-11-03,3,3,0,15,45,0,60,3,2,201,71,1,1,0,1,0,10,15171.35669,32623.25073,4.24090
9870,1857,2010-11-03,4,3,0,33.6666679382324,26.3333339691162,0,60,3,2,201,71,1,1,0,1,0,10,15
9871,1857,2010-11-04,0,4,0,16,25,0.5,41.5,2,2,201,71,1,1,0,1,0,10,15171.35669,32623.25073,4.24
9872,1857,2010-11-04,1,4,0,10,45,5,60,3,2,201,71,1,1,0,1,0,10,15171.35669,32623.25073,4.24090
9873,1857,2010-11-04,2,4,0,38.3333320617676,21.6666660308838,0,60,3,2,201,71,1,1,0,1,0,10,15
9874,1857,2010-11-04,3,4,0,18.3333339691162,32.3333320617676,9.33333301544189,60,3,2,201,
9875,1857,2010-11-04,4,4,0,23.6666660308838,36.3333320617676,0,60,3,2,201,71,1,1,0,1,0,10,15
9876,1857,2010-11-11,0,4,0,19.5,24,1.5,45,2,2,201,71,1,1,0,1,0,10,15171.35669,32623.25073,4.24
9877,1857,2010-11-11,1,4,0,5,49.6666679382324,5.33333349227905,60,3,2,201,71,1,1,0,1,0,10,15
9878,1857,2010-11-11,2,4,0,28,32,0,60,3,2,201,71,1,1,0,1,0,10,15171.35669,32623.25073,4.24090
9879,1857,2010-11-11,3,4,0,37.3333320617676,21,1.66666662693024,60,3,2,201,71,1,1,0,1,0,10,1
9880,1857,2010-11-11,4,4,0,32,27.3333339691162,0.666666686534882,60,3,2,201,71,1,1,0,1,0,10,
9881,1857,2010-11-12,3,5,0,26.6666660308838,33.3333320617676,0,60,3,2,201,71,1,1,0,1,0,10,15
9882,1857,2010-11-12,4,5,0,30,30,0,60,3,2,201,71,1,1,0,1,0,10,15171.35669,32623.25073,4.24090
9883,1859,2010-11-02,0,2,0,11.5,20.5,1.5,33.5,2,2,201,77,0,1,0,1,0,8,15171.35669,32623.25073,4.
9884,1859,2010-11-02,1,2,0,44,15.3333330154419,0.666666686534882,60,3,2,201,77,0,1,0,1,0,8,1
9885,1859,2010-11-02,2,2,0,46.6666679382324,13,0.333333343267441,60,3,2,201,77,0,1,0,1,0,8,1
9886,1859,2010-11-02,3,2,0,35.6666679382324,24.3333339691162,0,60,3,2,201,77,0,1,0,1,0,8,151
9887,1859,2010-11-02,4,2,0,45.5,14.5,0,60,3,2,201,77,0,1,0,1,0,8,15171.35669,32623.25073,4.240
9888,1859,2010-11-12,0,5,0,45,15,0,60,1,2,201,77,0,1,0,1,0,8,15171.35669,32623.25073,4.2409064
9889,1859,2010-11-12,1,5,0,51,8,1,60,3,2,201,77,0,1,0,1,0,8,15171.35669,32623.25073,4.2409064
9890,1859,2010-11-12,2,5,0,41.5,18,0.5,60,2,2,201,77,0,1,0,1,0,8,15171.35669,32623.25073,4.240
9891,1859,2010-11-13,0,6,1,43,0,1,44,1,2,201,77,0,1,0,1,0,8,15171.35669,32623.25073,4.2409064
9892,1859,2010-11-13,1,6,1,45,13.6666669845581,1.33333337306976,60,3,2,201,77,0,1,0,1,0,8,15
9893,1859,2010-11-13,2,6,1,49.25,10.75,0,60,3,2,201,77,0,1,0,1,0,8,15171.35669,32623.25073,4.2
9894,1859,2010-11-13,3,6,1,53,7,0,60,3,2,201,77,0,1,0,1,0,8,15171.35669,32623.25073,4.2409064
9895,1859,2010-11-13,4,6,1,42.6666679382324,17,0.333333343267441,60,3,2,201,77,0,1,0,1,0,8,1
9896,1859,2010-11-14,0,0,1,19,10,0,29,1,2,201,77,0,1,0,1,0,8,15171.35669,32623.25073,4.240906
9897,1859,2010-11-14,1,0,1,50.6666679382324,9.33333301544189,0,60,3,2,201,77,0,1,0,1,0,8,151
9898,1859,2010-11-14,2,0,1,43.3333320617676,16.6666660308838,0,60,3,2,201,77,0,1,0,1,0,8,151
9899,1859,2010-11-14,3,0,1,50.5,9.5,0,60,3,2,201,77,0,1,0,1,0,8,15171.35669,32623.25073,4.2409

9900,1859,2010-11-14,4,0,1,44,16,0,60,3,2,201,77,0,1,0,1,0,8,15171.35669,32623.25073,4.2409064
9901,1859,2010-11-15,0,1,0,14,10,0,24,1,2,201,77,0,1,0,1,0,8,15171.35669,32623.25073,4.2409064
9902,1859,2010-11-15,1,1,0,47,12.3333330154419,0.666666686534882,60,3,2,201,77,0,1,0,1,0,8,1
9903,1859,2010-11-15,2,1,0,44.3333320617676,14,1.66666662693024,60,3,2,201,77,0,1,0,1,0,8,15
9904,1859,2010-11-15,3,1,0,41.4000015258789,18.3999996185303,0.200000002980232,60,3,2,20
9905,1859,2010-11-15,4,1,0,51.5,8.5,0,60,3,2,201,77,0,1,0,1,0,8,15171.35669,32623.25073,4.2409
9906,1859,2010-11-16,0,2,0,16,12,1,29,1,2,201,77,0,1,0,1,0,8,15171.35669,32623.25073,4.2409064
9907,1859,2010-11-16,1,2,0,32.6666679382324,26.6666660308838,0.666666686534882,60,3,2,20
9908,1859,2010-11-16,2,2,0,45.3333320617676,14.6666669845581,0,60,3,2,201,77,0,1,0,1,0,8,151
9909,1859,2010-11-16,3,2,0,51,9,0,60,3,2,201,77,0,1,0,1,0,8,15171.35669,32623.25073,4.24090647
9910,1859,2010-11-16,4,2,0,46,13.5,0.5,60,3,2,201,77,0,1,0,1,0,8,15171.35669,32623.25073,4.240
9911,1859,2010-11-17,0,3,0,17,9,1,27,1,2,201,77,0,1,0,1,0,8,15171.35669,32623.25073,4.24090647
9912,1859,2010-11-17,1,3,0,44.6666679382324,14.3333330154419,1,60,3,2,201,77,0,1,0,1,0,8,151
9913,1859,2010-11-17,2,3,0,26.6666660308838,32.6666679382324,0.666666686534882,60,3,2,20
9914,1859,2010-11-17,3,3,0,45,14,1,60,3,2,201,77,0,1,0,1,0,8,15171.35669,32623.25073,4.2409064
9915,1859,2010-11-17,4,3,0,47.6666679382324,11.6666669845581,0.666666686534882,60,3,2,20
9916,1865,2010-10-29,0,5,0,10,22,0.5,32.5,2,2,201,81,1,0,1,0,0,5,15305.22033,24521.32922,2.859
9917,1865,2010-10-29,1,5,0,20.6666660308838,38.3333320617676,1,60,3,2,201,81,1,0,1,0,0,5,153
9918,1865,2010-10-29,2,5,0,48,12,0,60,3,2,201,81,1,0,1,0,0,5,15305.22033,24521.32922,2.859575
9919,1865,2010-10-29,3,5,0,40,18.6666660308838,1.33333337306976,60,3,2,201,81,1,0,1,0,0,5,15
9920,1865,2010-10-29,4,5,0,36.6666679382324,23.3333339691162,0,60,3,2,201,81,1,0,1,0,0,5,153
9921,1865,2010-10-30,0,6,1,4,9,1,14,1,2,201,81,1,0,1,0,0,5,15305.22033,24521.32922,2.85957559
9922,1865,2010-10-30,1,6,1,26.25,23,1.25,50.5,3,2,201,81,1,0,1,0,0,5,15305.22033,24521.32922,2.
9923,1865,2010-10-30,2,6,1,33,25.3333339691162,1.66666662693024,60,3,2,201,81,1,0,1,0,0,5,15
9924,1865,2010-10-30,3,6,1,46.6666679382324,13.3333330154419,0,60,3,2,201,81,1,0,1,0,0,5,153
9925,1865,2010-10-30,4,6,1,44.3333320617676,15.6666669845581,0,60,3,2,201,81,1,0,1,0,0,5,153
9926,1865,2010-10-31,1,0,1,33,27,0,60,2,2,201,81,1,0,1,0,0,5,15305.22033,24521.32922,2.859575
9927,1865,2010-10-31,2,0,1,39.3333320617676,20.3333339691162,0.333333343267441,60,3,2,20
9928,1865,2010-10-31,3,0,1,44,15,1,60,3,2,201,81,1,0,1,0,0,5,15305.22033,24521.32922,2.859575
9929,1865,2010-10-31,4,0,1,42.3333320617676,16.6666660308838,1,60,3,2,201,81,1,0,1,0,0,5,153
9930,1865,2010-11-03,0,3,0,42,18,0,60,1,2,201,81,1,0,1,0,0,5,15305.22033,24521.32922,2.859575
9931,1865,2010-11-03,1,3,0,28.3333339691162,27,4.66666650772095,60,3,2,201,81,1,0,1,0,0,5,15
9932,1865,2010-11-03,2,3,0,31.6666660308838,27.6666660308838,0.666666686534882,60,3,2,20
9933,1865,2010-11-03,3,3,0,34.3333320617676,25.3333339691162,0.333333343267441,60,3,2,20
9934,1865,2010-11-03,4,3,0,31.3333339691162,28.6666660308838,0,60,3,2,201,81,1,0,1,0,0,5,153
9935,1865,2010-11-04,0,4,0,15.5,30,0,45.5,2,2,201,81,1,0,1,0,0,5,15305.22033,24521.32922,2.859
9936,1865,2010-11-04,1,4,0,17,41.3333320617676,1.66666662693024,60,3,2,201,81,1,0,1,0,0,5,15
9937,1865,2010-11-04,2,4,0,33,27,0,60,3,2,201,81,1,0,1,0,0,5,15305.22033,24521.32922,2.859575
9938,1865,2010-11-04,3,4,0,48.6666679382324,10.6666669845581,0.666666686534882,60,3,2,20
9939,1865,2010-11-04,4,4,0,45.3333320617676,14.3333330154419,0.333333343267441,60,3,2,20
9940,1867,2010-10-31,1,0,1,9,25.5,0.5,35,2,2,201,69,1,0,1,1,0,10,15645.84768,10370.8513,2.6628
9941,1867,2010-12-17,0,5,0,18,12,1,31,1,2,201,69,1,0,1,1,0,10,15645.84768,10370.8513,2.662862
9942,1867,2010-12-17,1,5,0,31.25,28.75,0,60,3,2,201,69,1,0,1,1,0,10,15645.84768,10370.8513,2.66
9943,1867,2010-12-17,2,5,0,35.5,24.5,0,60,3,2,201,69,1,0,1,1,0,10,15645.84768,10370.8513,2.662
9944,1867,2010-12-17,3,5,0,28.3333339691162,31.3333339691162,0.333333343267441,60,3,2,20
9945,1867,2010-12-17,4,5,0,39,21,0,60,3,2,201,69,1,0,1,1,0,10,15645.84768,10370.8513,2.662862
9946,1867,2010-12-18,1,6,1,25,27,0.333333343267441,52.3333320617676,3,2,201,69,1,0,1,1,0,10,
9947,1867,2010-12-18,2,6,1,39.5999984741211,20.3999996185303,0,60,3,2,201,69,1,0,1,1,0,10,15
9948,1867,2010-12-18,3,6,1,47.5,12.5,0,60,3,2,201,69,1,0,1,1,0,10,15645.84768,10370.8513,2.662
9949,1867,2010-12-18,4,6,1,47.6666679382324,12.3333330154419,0,60,3,2,201,69,1,0,1,1,0,10,15

9950,1867,2010-12-19,0,0,1,1,4,3,8,1,2,201,69,1,0,1,1,0,10,15645.84768,10370.8513,2.662862058,
9951,1867,2010-12-19,1,0,1,36.6666679382324,22,0.666666686534882,59.3333320617676,3,2,201
9952,1867,2010-12-19,2,0,1,29.6666660308838,29,1.33333337306976,60,3,2,201,69,1,0,1,1,0,10,1
9953,1867,2010-12-19,3,0,1,40.3333320617676,19,0.666666686534882,60,3,2,201,69,1,0,1,1,0,10,
9954,1867,2010-12-19,4,0,1,45.8333320617676,14.1666669845581,0,60,3,2,201,69,1,0,1,1,0,10,15
9955,1867,2010-12-20,0,1,0,15,11,1,27,1,2,201,69,1,0,1,1,0,10,15645.84768,10370.8513,2.662862
9956,1867,2010-12-20,1,1,0,34.2000007629395,25.7999992370605,0,60,3,2,201,69,1,0,1,1,0,10,15
9957,1867,2010-12-20,2,1,0,36.4000015258789,23.6000003814697,0,60,3,2,201,69,1,0,1,1,0,10,15
9958,1867,2010-12-20,3,1,0,40.3333320617676,19.6666660308838,0,60,3,2,201,69,1,0,1,1,0,10,15
9959,1867,2010-12-20,4,1,0,35.3333320617676,24.6666660308838,0,60,3,2,201,69,1,0,1,1,0,10,15
9960,1867,2010-12-21,2,2,0,33,27,0,60,1,2,201,69,1,0,1,1,0,10,15645.84768,10370.8513,2.662862
9961,1867,2010-12-21,3,2,0,42.6666679382324,16.6666660308838,0.666666686534882,60,3,2,201
9962,1867,2010-12-21,4,2,0,51,8.66666698455811,0.333333343267441,60,3,2,201,69,1,0,1,1,0,10,
9963,1869,2010-12-01,1,3,0,18,13.5,0.5,32,2,2,201,74,1,0,0,1,0,2,15185.18451,25273.22359,2.2932
9964,1869,2010-12-01,2,3,0,28.75,31.25,0,60,3,2,201,74,1,0,0,1,0,2,15185.18451,25273.22359,2.29
9965,1869,2010-12-01,3,3,0,35.8333320617676,23.8333339691162,0.333333343267441,60,3,2,201
9966,1869,2010-12-01,4,3,0,38.25,21.75,0,60,3,2,201,74,1,0,0,1,0,2,15185.18451,25273.22359,2.29
9967,1869,2010-12-02,1,4,0,18,29.5,1,48.5,2,2,201,74,1,0,0,1,0,2,15185.18451,25273.22359,2.2932
9968,1869,2010-12-02,2,4,0,36.7999992370605,23,0.200000002980232,60,3,2,201,74,1,0,0,1,0,2,1
9969,1869,2010-12-02,3,4,0,28,32,0,60,3,2,201,74,1,0,0,1,0,2,15185.18451,25273.22359,2.2932985
9970,1869,2010-12-02,4,4,0,39,21,0,60,3,2,201,74,1,0,0,1,0,2,15185.18451,25273.22359,2.2932985
9971,1869,2010-12-03,2,5,0,35,25,0,60,1,2,201,74,1,0,0,1,0,2,15185.18451,25273.22359,2.2932985
9972,1869,2010-12-03,3,5,0,39.3333320617676,20.6666660308838,0,60,3,2,201,74,1,0,0,1,0,2,151
9973,1869,2010-12-03,4,5,0,47,13,0,60,3,2,201,74,1,0,0,1,0,2,15185.18451,25273.22359,2.2932985
9974,1869,2010-12-21,1,2,0,14.5,28.5,0,43,2,2,201,74,1,0,0,1,0,2,15185.18451,25273.22359,2.2932
9975,1869,2010-12-21,2,2,0,27,33,0,60,3,2,201,74,1,0,0,1,0,2,15185.18451,25273.22359,2.2932985
9976,1869,2010-12-21,3,2,0,32.3333320617676,27.6666660308838,0,60,3,2,201,74,1,0,0,1,0,2,151
9977,1869,2010-12-21,4,2,0,29.5,30.5,0,60,2,2,201,74,1,0,0,1,0,2,15185.18451,25273.22359,2.2932
9978,1869,2010-12-22,1,3,0,16.7999992370605,24.3999996185303,1.39999997615814,42.599998
9979,1869,2010-12-22,2,3,0,20.25,29.75,0.25,50.25,3,2,201,74,1,0,0,1,0,2,15185.18451,25273.2235
9980,1869,2010-12-22,3,3,0,23.3333339691162,16.6666660308838,0.333333343267441,40.333332
9981,1869,2010-12-22,4,3,0,43.25,16.75,0,60,3,2,201,74,1,0,0,1,0,2,15185.18451,25273.22359,2.29
9982,1883,2010-12-03,0,5,0,8,50,2,60,1,2,201,77,0,1,0,1,0,12,15797.27079,7498.056802,2.626674
9983,1883,2010-12-03,1,5,0,9.33333301544189,43.6666679382324,7,60,3,2,201,77,0,1,0,1,0,12,15
9984,1883,2010-12-03,2,5,0,13.6666669845581,42.3333320617676,4,60,3,2,201,77,0,1,0,1,0,12,15
9985,1883,2010-12-03,3,5,0,17,40,3,60,1,2,201,77,0,1,0,1,0,12,15797.27079,7498.056802,2.626674
9986,1883,2010-12-04,0,6,1,12,41.5,4.5,58,2,2,201,77,0,1,0,1,0,12,15797.27079,7498.056802,2.626
9987,1883,2010-12-04,1,6,1,12,44,4,60,3,2,201,77,0,1,0,1,0,12,15797.27079,7498.056802,2.626674
9988,1883,2010-12-04,2,6,1,12.3333330154419,42.6666679382324,5,60,3,2,201,77,0,1,0,1,0,12,15
9989,1883,2010-12-04,3,6,1,25.5,34,0.5,60,3,2,201,77,0,1,0,1,0,12,15797.27079,7498.056802,2.626
9990,1883,2010-12-04,4,6,1,43.3333320617676,16.3333339691162,0.333333343267441,60,3,2,201
9991,1883,2010-12-05,0,0,1,6,47,5,58,2,2,201,77,0,1,0,1,0,12,15797.27079,7498.056802,2.626674
9992,1883,2010-12-05,1,0,1,20.3333339691162,30.6666660308838,9,60,3,2,201,77,0,1,0,1,0,12,15
9993,1883,2010-12-05,2,0,1,10.3333330154419,43.6666679382324,6,60,3,2,201,77,0,1,0,1,0,12,15
9994,1883,2010-12-05,3,0,1,12,44.3333320617676,3.66666674613953,60,3,2,201,77,0,1,0,1,0,12,1
9995,1883,2010-12-05,4,0,1,39.6666679382324,20,0.333333343267441,60,3,2,201,77,0,1,0,1,0,12,
9996,1883,2010-12-06,0,1,0,1,50,5,56,1,2,201,77,0,1,0,1,0,12,15797.27079,7498.056802,2.626674
9997,1883,2010-12-06,1,1,0,4.66666650772095,46.3333320617676,9,60,3,2,201,77,0,1,0,1,0,12,15
9998,1883,2010-12-06,2,1,0,17.6666660308838,37.3333320617676,5,60,3,2,201,77,0,1,0,1,0,12,15
9999,1883,2010-12-06,3,1,0,30.5,26.25,3.25,60,3,2,201,77,0,1,0,1,0,12,15797.27079,7498.056802,2

10000,1883,2010-12-06,4,1,0,43,15.1999998092651,1.79999995231628,60,3,2,201,77,0,1,0,1,0,12,
10001,1883,2010-12-07,0,2,0,8.5,41.5,6,56,2,2,201,77,0,1,0,1,0,12,15797.27079,7498.056802,2.626
10002,1883,2010-12-07,1,2,0,10.6666669845581,45.3333320617676,4,60,3,2,201,77,0,1,0,1,0,12,1
10003,1883,2010-12-07,2,2,0,19.6666660308838,37,3.33333325386047,60,3,2,201,77,0,1,0,1,0,12,
10004,1883,2010-12-07,3,2,0,30.6666660308838,25.6666660308838,3.66666674613953,60,3,2,201
10005,1883,2010-12-07,4,2,0,43,16.5,0.5,60,3,2,201,77,0,1,0,1,0,12,15797.27079,7498.056802,2.62
10006,1883,2010-12-09,0,4,0,8.39999961853027,31.6000003814697,2.79999995231628,42.79999
10007,1883,2010-12-09,1,4,0,12.1999998092651,42.5999984741211,5.1999980926514,60,3,2,201
10008,1883,2010-12-09,2,4,0,7,39,14,60,3,2,201,77,0,1,0,1,0,12,15797.27079,7498.056802,2.62667
10009,1883,2010-12-09,3,4,0,18.3333339691162,32.3333320617676,3.33333325386047,54,3,2,201
10010,1883,2010-12-10,1,5,0,3,50,7,60,1,2,201,77,0,1,0,1,0,12,15797.27079,7498.056802,2.626674
10011,1883,2010-12-10,2,5,0,10.6666669845581,44,5.33333349227905,60,3,2,201,77,0,1,0,1,0,12,
10012,1883,2010-12-10,3,5,0,25.3333339691162,34.3333320617676,0.333333343267441,60,3,2,20
10013,1883,2010-12-10,4,5,0,47.6666679382324,12.3333330154419,0,60,3,2,201,77,0,1,0,1,0,12,1
10014,1887,2010-12-10,0,5,0,13,45,2,60,1,2,201,76,1,0,0,1,0,12,15174.45332,23046.36336,2.32582
10015,1887,2010-12-10,1,5,0,23,33,4,60,3,2,201,76,1,0,0,1,0,12,15174.45332,23046.36336,2.32582
10016,1887,2010-12-10,2,5,0,23.3333339691162,34,2.66666674613953,60,3,2,201,76,1,0,0,1,0,12,
10017,1887,2010-12-11,0,6,1,11.5,31.5,6.5,49.5,2,2,201,76,1,0,0,1,0,12,15174.45332,23046.36336,
10018,1887,2010-12-11,1,6,1,49.6666679382324,9,1.33333337306976,60,3,2,201,76,1,0,0,1,0,12,1
10019,1887,2010-12-11,2,6,1,34.6666679382324,23.6666660308838,1.66666662693024,60,3,2,201
10020,1887,2010-12-11,3,6,1,17.25,38.5,4.25,60,3,2,201,76,1,0,0,1,0,12,15174.45332,23046.36336
10021,1887,2010-12-11,4,6,1,40.3333320617676,19.3333339691162,0.333333343267441,60,3,2,20
10022,1887,2010-12-12,0,0,1,8.66666698455811,31.6666660308838,6.33333349227905,46.66667
10023,1887,2010-12-12,1,0,1,17.3333339691162,36.6666679382324,6,60,3,2,201,76,1,0,0,1,0,12,1
10024,1887,2010-12-12,2,0,1,43.6666679382324,16.3333339691162,0,60,3,2,201,76,1,0,0,1,0,12,1
10025,1887,2010-12-12,3,0,1,26.7999992370605,30.7999992370605,2.40000009536743,60,3,2,201
10026,1887,2010-12-12,4,0,1,32,26.3333339691162,1.66666662693024,60,3,2,201,76,1,0,0,1,0,12,
10027,1887,2010-12-13,0,1,0,19.5,35.5,5,60,2,2,201,76,1,0,0,1,0,12,15174.45332,23046.36336,2.32
10028,1887,2010-12-13,1,1,0,11,39.7999992370605,9.1999980926514,60,3,2,201,76,1,0,0,1,0,12,
10029,1887,2010-12-13,2,1,0,31.6666660308838,26,2.33333325386047,60,3,2,201,76,1,0,0,1,0,12,
10030,1887,2010-12-13,3,1,0,28.6666660308838,21,10.3333330154419,60,3,2,201,76,1,0,0,1,0,12,
10031,1887,2010-12-13,4,1,0,44,15.3333330154419,0.666666686534882,60,3,2,201,76,1,0,0,1,0,12
10032,1887,2010-12-14,0,2,0,23,19.5,0,42.5,2,2,201,76,1,0,0,1,0,12,15174.45332,23046.36336,2.32
10033,1887,2010-12-14,1,2,0,11.6666669845581,35,13.3333330154419,60,3,2,201,76,1,0,0,1,0,12,
10034,1887,2010-12-14,2,2,0,36.6666679382324,22,1.33333337306976,60,3,2,201,76,1,0,0,1,0,12,
10035,1887,2010-12-14,3,2,0,47,13,0,60,3,2,201,76,1,0,0,1,0,12,15174.45332,23046.36336,2.32582
10036,1887,2010-12-14,4,2,0,36.6666679382324,23.3333339691162,0,60,3,2,201,76,1,0,0,1,0,12,1
10037,1887,2010-12-15,1,3,0,22,27,11,60,1,2,201,76,1,0,0,1,0,12,15174.45332,23046.36336,2.3258
10038,1887,2010-12-15,2,3,0,29,22.6666660308838,8.33333301544189,60,3,2,201,76,1,0,0,1,0,12,
10039,1887,2010-12-15,3,3,0,41.3333320617676,13.6666669845581,5,60,3,2,201,76,1,0,0,1,0,12,1
10040,1887,2010-12-15,4,3,0,48,11.6666669845581,0.333333343267441,60,3,2,201,76,1,0,0,1,0,12
10041,1902,2010-12-01,0,3,0,31,17.3333339691162,0.333333343267441,48.6666679382324,3,2,20
10042,1902,2010-12-01,1,3,0,17.3333339691162,42,0.666666686534882,60,3,2,201,76,1,1,1,0,0,10
10043,1902,2010-12-01,2,3,0,35.3333320617676,24,0.666666686534882,60,3,2,201,76,1,1,1,0,0,10
10044,1902,2010-12-01,3,3,0,44.25,15.75,0,60,3,2,201,76,1,1,1,0,0,10,16017.06919,26861.18907,2
10045,1902,2010-12-01,4,3,0,37.6666679382324,15.3333330154419,0,53,3,2,201,76,1,1,1,0,0,10,1
10046,1902,2010-12-02,0,4,0,38,12,4,54,1,2,201,76,1,1,1,0,0,10,16017.06919,26861.18907,2.52946
10047,1902,2010-12-02,1,4,0,46,14,0,60,3,2,201,76,1,1,1,0,0,10,16017.06919,26861.18907,2.52946
10048,1902,2010-12-02,2,4,0,47.6666679382324,12.3333330154419,0,60,3,2,201,76,1,1,1,0,0,10,1
10049,1902,2010-12-02,3,4,0,51,8,1,60,3,2,201,76,1,1,1,0,0,10,16017.06919,26861.18907,2.529468

10050,1902,2010-12-02,4,4,0,29.1666660308838,18.8333339691162,0.666666686534882,48.6666
10051,1902,2010-12-03,0,5,0,26.3333339691162,8.66666698455811,0.666666686534882,35.6666
10052,1902,2010-12-03,1,5,0,36.3333320617676,23.6666660308838,0,60,3,2,201,76,1,1,1,0,0,10,1
10053,1902,2010-12-03,2,5,0,44,16,0,60,3,2,201,76,1,1,1,0,0,10,16017.06919,26861.18907,2.5294
10054,1902,2010-12-03,3,5,0,42.3333320617676,17,0.666666686534882,60,3,2,201,76,1,1,1,0,0,10
10055,1902,2010-12-03,4,5,0,33,25.6666660308838,0.333333343267441,59,3,2,201,76,1,1,1,0,0,10
10056,1902,2010-12-04,0,6,1,39.5,12.5,0.5,52.5,2,2,201,76,1,1,1,0,0,10,16017.06919,26861.18907,
10057,1902,2010-12-04,1,6,1,39.25,20.5,0.25,60,3,2,201,76,1,1,1,0,0,10,16017.06919,26861.18907
10058,1902,2010-12-04,2,6,1,41.6666679382324,18.3333339691162,0,60,3,2,201,76,1,1,1,0,0,10,1
10059,1902,2010-12-04,3,6,1,43.6666679382324,16.3333339691162,0,60,3,2,201,76,1,1,1,0,0,10,1
10060,1902,2010-12-04,4,6,1,42,17.6666660308838,0.333333343267441,60,3,2,201,76,1,1,1,0,0,10
10061,1902,2010-12-05,1,0,1,35.4000015258789,20,0.400000005960464,55.7999992370605,3,2,20
10062,1902,2010-12-05,2,0,1,36.3333320617676,23,0.666666686534882,60,3,2,201,76,1,1,1,0,0,10
10063,1902,2010-12-05,3,0,1,34.6666679382324,24.6666660308838,0.666666686534882,60,3,2,20
10064,1902,2010-12-05,4,0,1,31.6666660308838,19,0.333333343267441,51,3,2,201,76,1,1,1,0,0,10
10065,1902,2010-12-06,1,1,0,22.5,30.5,0.5,53.5,2,2,201,76,1,1,1,0,0,10,16017.06919,26861.18907,
10066,1902,2010-12-06,2,1,0,41.5,18.3333339691162,0.16666667163372,60,3,2,201,76,1,1,1,0,0,1
10067,1902,2010-12-06,3,1,0,48.75,11,0.25,60,3,2,201,76,1,1,1,0,0,10,16017.06919,26861.18907,2
10068,1902,2010-12-06,4,1,0,45.3333320617676,14.6666669845581,0,60,3,2,201,76,1,1,1,0,0,10,1
10069,1902,2010-12-07,3,2,0,55,5,0,60,2,2,201,76,1,1,1,0,0,10,16017.06919,26861.18907,2.529468
10070,1902,2010-12-07,4,2,0,39,12,0,51,3,2,201,76,1,1,1,0,0,10,16017.06919,26861.18907,2.52946
10071,1902,2010-12-15,0,3,0,47,12,1,60,1,2,201,76,1,1,1,0,0,10,16017.06919,26861.18907,2.52946
10072,1902,2010-12-15,1,3,0,32.6666679382324,27.3333339691162,0,60,3,2,201,76,1,1,1,0,0,10,1
10073,1902,2010-12-15,2,3,0,41,18.6666660308838,0.333333343267441,60,3,2,201,76,1,1,1,0,0,10
10074,1902,2010-12-15,3,3,0,51,8.5,0.5,60,2,2,201,76,1,1,1,0,0,10,16017.06919,26861.18907,2.529
10075,1927,2010-12-07,1,2,0,7,36,0,43,2,2,201,84,1,1,1,0,0,10,16017.06919,26861.18907,2.529468
10076,1927,2010-12-07,2,2,0,36,23.6666660308838,0.333333343267441,60,3,2,201,84,1,1,1,0,0,10
10077,1927,2010-12-07,3,2,0,30,29,1,60,3,2,201,84,1,1,1,0,0,10,16017.06919,26861.18907,2.52946
10078,1927,2010-12-07,4,2,0,27,30,3,60,1,2,201,84,1,1,1,0,0,10,16017.06919,26861.18907,2.52946
10079,1927,2011-01-14,0,5,0,12,33.5,0.5,46,2,2,201,84,1,1,1,0,0,10,16017.06919,26861.18907,2.52
10080,1927,2011-01-14,1,5,0,32.3333320617676,27.3333339691162,0.333333343267441,60,3,2,20
10081,1927,2011-01-14,2,5,0,30.3333339691162,29.3333339691162,0.333333343267441,60,3,2,20
10082,1927,2011-01-14,3,5,0,36.6666679382324,23,0.333333343267441,60,3,2,201,84,1,1,1,0,0,10
10083,1927,2011-01-14,4,5,0,26.75,13,0.25,40,3,2,201,84,1,1,1,0,0,10,16017.06919,26861.18907,2
10084,1927,2011-01-15,0,6,1,7,25,1,33,1,2,201,84,1,1,1,0,0,10,16017.06919,26861.18907,2.529468
10085,1927,2011-01-15,1,6,1,30.2000007629395,27.6000003814697,2.20000004768372,60,3,2,201
10086,1927,2011-01-15,2,6,1,30.6666660308838,29,0.333333343267441,60,3,2,201,84,1,1,1,0,0,10
10087,1927,2011-01-15,3,6,1,40.6666679382324,19.3333339691162,0,60,3,2,201,84,1,1,1,0,0,10,1
10088,1927,2011-01-15,4,6,1,28.3333339691162,16.6666660308838,0.333333343267441,45.33333
10089,1927,2011-01-16,1,0,1,20,38.25,1.25,59.5,3,2,201,84,1,1,1,0,0,10,16017.06919,26861.18907
10090,1927,2011-01-16,2,0,1,28.3999996185303,31.3999996185303,0.200000002980232,60,3,2,20
10091,1927,2011-01-16,3,0,1,34,24,2,60,3,2,201,84,1,1,1,0,0,10,16017.06919,26861.18907,2.52946
10092,1927,2011-01-16,4,0,1,49,11,0,60,3,2,201,84,1,1,1,0,0,10,16017.06919,26861.18907,2.52946
10093,1927,2011-01-17,0,1,0,3,32,2,37,1,2,201,84,1,1,1,0,0,10,16017.06919,26861.18907,2.529468
10094,1927,2011-01-17,1,1,0,18,31.3333339691162,10.6666669845581,60,3,2,201,84,1,1,1,0,0,10,
10095,1927,2011-01-17,2,1,0,23.75,35.75,0.5,60,3,2,201,84,1,1,1,0,0,10,16017.06919,26861.18907
10096,1927,2011-01-17,3,1,0,25.75,34,0.25,60,3,2,201,84,1,1,1,0,0,10,16017.06919,26861.18907,2
10097,1927,2011-01-17,4,1,0,50.6666679382324,9,0.333333343267441,60,3,2,201,84,1,1,1,0,0,10,
10098,1927,2011-01-18,0,2,0,20,1,0,21,1,2,201,84,1,1,1,0,0,10,16017.06919,26861.18907,2.529468
10099,1927,2011-01-18,1,2,0,29,29.3333339691162,1.66666662693024,60,3,2,201,84,1,1,1,0,0,10,

10100,1927,2011-01-18,2,2,0,32,27.3333339691162,0.666666686534882,60,3,2,201,84,1,1,1,0,0,1
10101,1927,2011-01-18,3,2,0,38.7999992370605,20.7999992370605,0.400000005960464,60,3,2,2
10102,1927,2011-01-18,4,2,0,38,21,0.25,59.25,3,2,201,84,1,1,1,0,0,10,16017.06919,26861.18907,2
10103,1927,2011-01-19,0,3,0,0,6,0,6,1,2,201,84,1,1,1,0,0,10,16017.06919,26861.18907,2.5294680
10104,1927,2011-01-19,1,3,0,4.66666650772095,22,0.666666686534882,27.3333339691162,3,2,2
10105,1927,2011-01-19,2,3,0,29.6666660308838,30,0.333333343267441,60,3,2,201,84,1,1,1,0,0,1
10106,1927,2011-01-19,3,3,0,34.3333320617676,24,1.66666662693024,60,3,2,201,84,1,1,1,0,0,10,
10107,1927,2011-01-19,4,3,0,36.75,15.25,0.25,52.25,3,2,201,84,1,1,1,0,0,10,16017.06919,26861.1
10108,1935,2012-02-18,0,6,1,30,27,3,60,1,2,202,73,1,0,0,1,0,6,1260.35174,1703.831376,2.736642
10109,1935,2012-02-18,1,6,1,30.3333339691162,29.3333339691162,0.333333343267441,60,3,2,2
10110,1935,2012-02-18,2,6,1,42.3333320617676,17.6666660308838,0,60,3,2,202,73,1,0,0,1,0,6,12
10111,1935,2012-02-18,3,6,1,33.3333320617676,26,0.666666686534882,60,3,2,202,73,1,0,0,1,0,6,
10112,1935,2012-02-18,4,6,1,45.3333320617676,14.6666669845581,0,60,3,2,202,73,1,0,0,1,0,6,12
10113,1935,2012-02-19,0,7,1,17,25.6666660308838,1.66666662693024,44.3333320617676,3,2,20
10114,1935,2012-02-19,1,7,1,32.3333320617676,27.6666660308838,0,60,3,2,202,73,1,0,0,1,0,6,12
10115,1935,2012-02-19,2,7,1,43.3333320617676,16.6666660308838,0,60,3,2,202,73,1,0,0,1,0,6,12
10116,1935,2012-02-19,3,7,1,38,21.6666660308838,0.333333343267441,60,3,2,202,73,1,0,0,1,0,6,
10117,1935,2012-02-19,4,7,1,45,14.6666669845581,0.333333343267441,60,3,2,202,73,1,0,0,1,0,6,
10118,1935,2012-02-20,0,1,0,9.33333301544189,11.3333330154419,0.333333343267441,21,3,2,2
10119,1935,2012-02-20,1,1,0,25.6666660308838,34.3333320617676,0,60,3,2,202,73,1,0,0,1,0,6,12
10120,1935,2012-02-20,2,1,0,36,23.6666660308838,0.333333343267441,60,3,2,202,73,1,0,0,1,0,6,
10121,1935,2012-02-20,3,1,0,40,19.6666660308838,0.333333343267441,60,3,2,202,73,1,0,0,1,0,6,
10122,1935,2012-02-20,4,1,0,37,22.6666660308838,0.333333343267441,60,3,2,202,73,1,0,0,1,0,6,
10123,1935,2012-02-21,0,2,0,1.66666662693024,0.666666686534882,0,2.33333325386047,3,2,20
10124,1935,2012-02-21,1,2,0,26.6666660308838,33,0.333333343267441,60,3,2,202,73,1,0,0,1,0,6,
10125,1935,2012-02-21,2,2,0,41.6666679382324,18.3333339691162,0,60,3,2,202,73,1,0,0,1,0,6,12
10126,1935,2012-02-21,3,2,0,44.6666679382324,14.6666669845581,0.666666686534882,60,3,2,2
10127,1935,2012-02-21,4,2,0,50.6666679382324,9.33333301544189,0,60,3,2,202,73,1,0,0,1,0,6,12
10128,1935,2012-02-22,0,3,0,22.6666660308838,0.333333343267441,0,23,3,2,202,73,1,0,0,1,0,6,1
10129,1935,2012-02-22,1,3,0,28.6666660308838,31.3333339691162,0,60,3,2,202,73,1,0,0,1,0,6,12
10130,1935,2012-02-22,2,3,0,31,29,0,60,3,2,202,73,1,0,0,1,0,6,1260.35174,1703.831376,2.736642
10131,1935,2012-02-22,3,3,0,33,27,0,60,3,2,202,73,1,0,0,1,0,6,1260.35174,1703.831376,2.736642
10132,1935,2012-02-22,4,3,0,30.6666660308838,29.3333339691162,0,60,3,2,202,73,1,0,0,1,0,6,12
10133,1935,2012-02-23,0,4,0,2,4,0,6,3,2,202,73,1,0,0,1,0,6,1260.35174,1703.831376,2.736642575,
10134,1935,2012-02-23,1,4,0,30.3333339691162,29.6666660308838,0,60,3,2,202,73,1,0,0,1,0,6,12
10135,1935,2012-02-23,2,4,0,45.6666679382324,14.3333330154419,0,60,3,2,202,73,1,0,0,1,0,6,12
10136,1935,2012-02-23,3,4,0,32.3333320617676,27.6666660308838,0,60,3,2,202,73,1,0,0,1,0,6,12
10137,1935,2012-02-23,4,4,0,38,22,0,60,3,2,202,73,1,0,0,1,0,6,1260.35174,1703.831376,2.736642
10138,1935,2012-02-24,0,5,0,3.33333325386047,1,0.666666686534882,5,3,2,202,73,1,0,0,1,0,6,12
10139,1935,2012-02-24,1,5,0,24,36,0,60,3,2,202,73,1,0,0,1,0,6,1260.35174,1703.831376,2.736642
10140,1935,2012-02-24,2,5,0,29.6666660308838,30.3333339691162,0,60,3,2,202,73,1,0,0,1,0,6,12
10141,1935,2012-02-24,3,5,0,43,16.3333339691162,0.666666686534882,60,3,2,202,73,1,0,0,1,0,6,
10142,1935,2012-02-24,4,5,0,31.3333339691162,28.3333339691162,0.333333343267441,60,3,2,2
10143,1937,2011-01-14,0,5,0,37.5,7.5,13,58,2,2,202,75,1,0,0,1,0,6,1098.882961,2012.805128,3.87
10144,1937,2011-01-14,1,5,0,3,13.75,43.25,60,3,2,202,75,1,0,0,1,0,6,1098.882961,2012.805128,3.
10145,1937,2011-01-14,2,5,0,28.3333339691162,25.6666660308838,6,60,3,2,202,75,1,0,0,1,0,6,10
10146,1937,2011-01-14,3,5,0,29.3333339691162,30,0.666666686534882,60,3,2,202,75,1,0,0,1,0,6,
10147,1937,2011-01-14,4,5,0,6.33333349227905,51.3333320617676,2.33333325386047,60,3,2,20
10148,1937,2011-01-15,0,6,1,31.5,21.5,4.5,57.5,2,2,202,75,1,0,0,1,0,6,1098.882961,2012.805128,3
10149,1937,2011-01-15,1,6,1,11.25,43.25,5.5,60,3,2,202,75,1,0,0,1,0,6,1098.882961,2012.805128,

10150,1937,2011-01-15,2,6,1,23.75,28.75,7.5,60,3,2,202,75,1,0,0,1,0,6,1098.882961,2012.805128,3
10151,1937,2011-01-15,3,6,1,10.6666669845581,41.3333320617676,8,60,3,2,202,75,1,0,0,1,0,6,10
10152,1937,2011-01-15,4,6,1,18,34.6666679382324,7.33333349227905,60,3,2,202,75,1,0,0,1,0,6,1
10153,1937,2011-01-16,0,0,1,54,0,0,54,1,2,202,75,1,0,0,1,0,6,1098.882961,2012.805128,3.878621
10154,1937,2011-01-16,1,0,1,21.3333339691162,14,24.6666660308838,60,3,2,202,75,1,0,0,1,0,6,1
10155,1937,2011-01-16,2,0,1,22.6000003814697,35.5999984741211,1.79999995231628,60,3,2,202
10156,1937,2011-01-16,3,0,1,14,34.25,11.75,60,3,2,202,75,1,0,0,1,0,6,1098.882961,2012.805128,3
10157,1937,2011-01-16,4,0,1,37.6666679382324,22,0.333333343267441,60,3,2,202,75,1,0,0,1,0,6,
10158,1937,2011-01-17,0,1,0,32.5,3,1,36.5,2,2,202,75,1,0,0,1,0,6,1098.882961,2012.805128,3.878
10159,1937,2011-01-17,1,1,0,2.33333325386047,55.3333320617676,2.33333325386047,60,3,2,202
10160,1937,2011-01-17,2,1,0,16.6666660308838,40.6666679382324,2.66666674613953,60,3,2,202
10161,1937,2011-01-17,3,1,0,18.7999992370605,38,3.20000004768372,60,3,2,202,75,1,0,0,1,0,6,1
10162,1937,2011-01-17,4,1,0,16.3333339691162,29.6666660308838,1.33333337306976,47.333332
10163,1937,2011-01-18,0,2,0,13,2.66666674613953,0.666666686534882,16.3333339691162,3,2,20
10164,1937,2011-01-18,1,2,0,16,40,4,60,3,2,202,75,1,0,0,1,0,6,1098.882961,2012.805128,3.878621
10165,1937,2011-01-18,2,2,0,9.66666698455811,38,2.33333325386047,50,3,2,202,75,1,0,0,1,0,6,1
10166,1937,2011-01-18,3,2,0,8,44.3333320617676,7.66666650772095,60,3,2,202,75,1,0,0,1,0,6,10
10167,1937,2011-01-18,4,2,0,20,38.5,1.5,60,3,2,202,75,1,0,0,1,0,6,1098.882961,2012.805128,3.878
10168,1937,2011-01-19,0,3,0,2,34,24,60,1,2,202,75,1,0,0,1,0,6,1098.882961,2012.805128,3.878621
10169,1937,2011-01-19,1,3,0,9,27,24,60,1,2,202,75,1,0,0,1,0,6,1098.882961,2012.805128,3.878621
10170,1937,2011-01-19,2,3,0,40.5,19.5,0,60,2,2,202,75,1,0,0,1,0,6,1098.882961,2012.805128,3.878
10171,1937,2011-01-19,3,3,0,14.3333330154419,45.3333320617676,0.333333343267441,60,3,2,20
10172,1937,2011-01-19,4,3,0,19.3333339691162,40.3333320617676,0.333333343267441,60,3,2,20
10173,1939,2011-01-13,1,4,0,14.3333330154419,36,0.333333343267441,50.6666679382324,3,2,20
10174,1939,2011-01-13,2,4,0,47.3333320617676,12.6666669845581,0,60,3,2,202,77,0,1,0,1,0,11,1
10175,1939,2011-01-13,3,4,0,33,26.75,0.25,60,3,2,202,77,0,1,0,1,0,11,1137.045481,2013.377652,3
10176,1939,2011-01-13,4,4,0,46.6666679382324,13.3333330154419,0,60,3,2,202,77,0,1,0,1,0,11,1
10177,1939,2011-01-14,0,5,0,22,12,0,34,2,2,202,77,0,1,0,1,0,11,1137.045481,2013.377652,3.25348
10178,1939,2011-01-14,1,5,0,20,33.25,1.25,54.5,3,2,202,77,0,1,0,1,0,11,1137.045481,2013.377652
10179,1939,2011-01-14,2,5,0,43.6666679382324,16,0.333333343267441,60,3,2,202,77,0,1,0,1,0,11
10180,1939,2011-01-14,3,5,0,35,25,0,60,3,2,202,77,0,1,0,1,0,11,1137.045481,2013.377652,3.25348
10181,1939,2011-01-14,4,5,0,34.6666679382324,25.3333339691162,0,60,3,2,202,77,0,1,0,1,0,11,1
10182,1939,2011-01-15,1,6,1,40.2000007629395,14.6000003814697,4.80000019073486,59.599998
10183,1939,2011-01-15,2,6,1,37.75,21.5,0.75,60,3,2,202,77,0,1,0,1,0,11,1137.045481,2013.377652
10184,1939,2011-01-15,3,6,1,42,18,0,60,3,2,202,77,0,1,0,1,0,11,1137.045481,2013.377652,3.25348
10185,1939,2011-01-15,4,6,1,47.3333320617676,12.6666669845581,0,60,3,2,202,77,0,1,0,1,0,11,1
10186,1939,2011-01-16,0,0,1,39,20,1,60,1,2,202,77,0,1,0,1,0,11,1137.045481,2013.377652,3.25348
10187,1939,2011-01-16,1,0,1,22,37,1,60,3,2,202,77,0,1,0,1,0,11,1137.045481,2013.377652,3.25348
10188,1939,2011-01-16,2,0,1,33.7999992370605,15,11.1999998092651,60,3,2,202,77,0,1,0,1,0,11,
10189,1939,2011-01-16,3,0,1,44.6666679382324,7.33333349227905,8,60,3,2,202,77,0,1,0,1,0,11,1
10190,1939,2011-01-16,4,0,1,30.5,7.5,5.5,43.5,2,2,202,77,0,1,0,1,0,11,1137.045481,2013.377652,3
10191,1939,2011-01-17,0,1,0,30.6666660308838,21,0.333333343267441,52,3,2,202,77,0,1,0,1,0,1
10192,1939,2011-01-17,1,1,0,38.3333320617676,21,0.666666686534882,60,3,2,202,77,0,1,0,1,0,1
10193,1939,2011-01-17,2,1,0,36.3333320617676,23.3333339691162,0.333333343267441,60,3,2,20
10194,1939,2011-01-17,3,1,0,39.5,20.25,0.25,60,3,2,202,77,0,1,0,1,0,11,1137.045481,2013.377652
10195,1939,2011-01-17,4,1,0,43.6666679382324,16.3333339691162,0,60,3,2,202,77,0,1,0,1,0,11,1
10196,1939,2011-01-18,0,2,0,28,6,3,37,1,2,202,77,0,1,0,1,0,11,1137.045481,2013.377652,3.253484
10197,1939,2011-01-18,1,2,0,13.6666669845581,42.6666679382324,3.66666674613953,60,3,2,202
10198,1939,2011-01-18,2,2,0,36,23.3333339691162,0.666666686534882,60,3,2,202,77,0,1,0,1,0,1
10199,1939,2011-01-18,3,2,0,38,21.7999992370605,0.200000002980232,60,3,2,202,77,0,1,0,1,0,1

10200,1939,2011-01-18,4,2,0,44.5999984741211,15.1999998092651,0.200000002980232,60,3,2,2
10201,1939,2011-01-19,0,3,0,4,1,0,5,1,2,202,77,0,1,0,1,0,11,1137.045481,2013.377652,3.2534848
10202,1939,2011-01-19,1,3,0,28,31.6666660308838,0.333333343267441,60,3,2,202,77,0,1,0,1,0,1
10203,1939,2011-01-19,2,3,0,29,31,0,60,3,2,202,77,0,1,0,1,0,11,1137.045481,2013.377652,3.2534
10204,1939,2011-01-19,3,3,0,38,22,0,60,2,2,202,77,0,1,0,1,0,11,1137.045481,2013.377652,3.2534
10205,1939,2011-01-19,4,3,0,41,18,1,60,1,2,202,77,0,1,0,1,0,11,1137.045481,2013.377652,3.2534
10206,1939,2011-01-20,0,4,0,25.5,20.5,0,46,2,2,202,77,0,1,0,1,0,11,1137.045481,2013.377652,3.25
10207,1939,2011-01-20,1,4,0,32,28,0,60,3,2,202,77,0,1,0,1,0,11,1137.045481,2013.377652,3.2534
10208,1939,2011-01-20,2,4,0,49,11,0,60,3,2,202,77,0,1,0,1,0,11,1137.045481,2013.377652,3.2534
10209,1939,2011-01-20,3,4,0,35,24.6666660308838,0.333333343267441,60,3,2,202,77,0,1,0,1,0,1
10210,1939,2011-01-20,4,4,0,51,8.66666698455811,0.333333343267441,60,3,2,202,77,0,1,0,1,0,1
10211,1942,2011-01-13,1,4,0,6,15.5,24.5,46,2,2,202,72,0,0,0,1,0,12,1203.692795,1938.750116,3.4
10212,1942,2011-01-13,2,4,0,24.5,19,16.5,60,3,2,202,72,0,0,0,1,0,12,1203.692795,1938.750116,3.
10213,1942,2011-01-13,3,4,0,4.5,25.8333339691162,29.6666660308838,60,3,2,202,72,0,0,0,1,0,12
10214,1942,2011-01-13,4,4,0,13.25,35.75,11,60,3,2,202,72,0,0,0,1,0,12,1203.692795,1938.750116,
10215,1942,2011-01-14,1,5,0,10.3999996185303,26.6000003814697,8.60000038146973,45.59999
10216,1942,2011-01-14,2,5,0,13.6666669845581,32,14.3333330154419,60,3,2,202,72,0,0,0,1,0,12,
10217,1942,2011-01-14,3,5,0,7.33333349227905,26.3333339691162,26.3333339691162,60,3,2,20
10218,1942,2011-01-14,4,5,0,32.4000015258789,26.2000007629395,1.39999997615814,60,3,2,20
10219,1942,2011-01-15,1,6,1,23.6666660308838,27.6666660308838,3.33333325386047,54.66666
10220,1942,2011-01-15,2,6,1,18.3999996185303,37.5999984741211,4,60,3,2,202,72,0,0,0,1,0,12,
10221,1942,2011-01-15,3,6,1,20.6666660308838,38.3333320617676,1,60,3,2,202,72,0,0,0,1,0,12,
10222,1942,2011-01-15,4,6,1,32,23.3333339691162,4.66666650772095,60,3,2,202,72,0,0,0,1,0,12,
10223,1942,2011-01-16,2,0,1,2,58,0,60,1,2,202,72,0,0,0,1,0,12,1203.692795,1938.750116,3.48949
10224,1942,2011-01-16,3,0,1,0.333333343267441,54.3333320617676,5.33333349227905,60,3,2,2
10225,1942,2011-01-16,4,0,1,15.6666669845581,40.6666679382324,3.66666674613953,60,3,2,20
10226,1942,2011-01-19,0,3,0,3,11,4,18,1,2,202,72,0,0,0,1,0,12,1203.692795,1938.750116,3.48949
10227,1942,2011-01-19,1,3,0,13,29.25,5.25,47.5,3,2,202,72,0,0,0,1,0,12,1203.692795,1938.750116
10228,1942,2011-01-19,2,3,0,17,33.6666679382324,9.33333301544189,60,3,2,202,72,0,0,0,1,0,12,
10229,1942,2011-01-19,3,3,0,12,34.6666679382324,13.3333330154419,60,3,2,202,72,0,0,0,1,0,12,
10230,1942,2011-01-19,4,3,0,15.6666669845581,38.6666679382324,5.66666650772095,60,3,2,20
10231,1942,2011-01-20,1,4,0,6,32.5999984741211,11.8000001907349,50.4000015258789,3,2,202,
10232,1942,2011-01-20,2,4,0,11.75,31,17.25,60,3,2,202,72,0,0,0,1,0,12,1203.692795,1938.750116,
10233,1942,2011-01-20,3,4,0,30,15.3333330154419,14.6666669845581,60,3,2,202,72,0,0,0,1,0,12,
10234,1942,2011-01-20,4,4,0,13.6666669845581,37.6666679382324,8.66666698455811,60,3,2,20
10235,1942,2011-01-25,1,2,0,9.66666698455811,30.6666660308838,9,49.3333320617676,3,2,202,
10236,1942,2011-01-25,2,2,0,12.75,27.25,20,60,3,2,202,72,0,0,0,1,0,12,1203.692795,1938.750116,
10237,1942,2011-01-25,3,2,0,3.33333325386047,32.6666679382324,24,60,3,2,202,72,0,0,0,1,0,12,
10238,1942,2011-01-25,4,2,0,13.3333330154419,36.3333320617676,10.3333330154419,60,3,2,20
10239,1953,2011-01-16,0,0,1,15,39,0,54,1,2,202,84,0,0,0,0,0,8,1157.907736,1714.733628,1.74382
10240,1953,2011-01-16,1,0,1,5,55,0,60,3,2,202,84,0,0,0,0,0,8,1157.907736,1714.733628,1.743826
10241,1953,2011-01-16,2,0,1,21,38,1,60,3,2,202,84,0,0,0,0,0,8,1157.907736,1714.733628,1.74382
10242,1953,2011-01-16,3,0,1,1.5,56,2.5,60,2,2,202,84,0,0,0,0,0,8,1157.907736,1714.733628,1.743
10243,1953,2011-01-17,0,1,0,10,23.5,0.5,34,2,2,202,84,0,0,0,0,0,8,1157.907736,1714.733628,1.74
10244,1953,2011-01-17,1,1,0,17.3333339691162,39,3.66666674613953,60,3,2,202,84,0,0,0,0,0,8,
10245,1953,2011-01-17,2,1,0,26.6666660308838,33,0.333333343267441,60,3,2,202,84,0,0,0,0,0,8,
10246,1953,2011-01-17,3,1,0,16.25,42.25,1.5,60,3,2,202,84,0,0,0,0,0,8,1157.907736,1714.733628,
10247,1953,2011-01-17,4,1,0,46.3333320617676,13,0.666666686534882,60,3,2,202,84,0,0,0,0,0,8,
10248,1953,2011-01-18,0,2,0,10,23,0.5,33.5,2,2,202,84,0,0,0,0,0,8,1157.907736,1714.733628,1.74
10249,1953,2011-01-18,1,2,0,7,51,2,60,3,2,202,84,0,0,0,0,0,8,1157.907736,1714.733628,1.743826

10250,1953,2011-01-18,2,2,0,23,36,1,60,3,2,202,84,0,0,0,0,0,8,1157.907736,1714.733628,1.74382
10251,1953,2011-01-18,3,2,0,18.6666660308838,40,1.33333337306976,60,3,2,202,84,0,0,0,0,0,8,1
10252,1953,2011-01-18,4,2,0,40.75,18.25,1,60,3,2,202,84,0,0,0,0,0,8,1157.907736,1714.733628,1.
10253,1953,2011-01-19,1,3,0,49.7999992370605,3.79999995231628,4.59999990463257,58.20000
10254,1953,2011-01-19,2,3,0,43.5,6.66666650772095,9.83333301544189,60,3,2,202,84,0,0,0,0,0,8
10255,1953,2011-01-19,3,3,0,54.6666679382324,5.33333349227905,0,60,3,2,202,84,0,0,0,0,0,8,11
10256,1953,2011-01-19,4,3,0,45,8,0.25,53.25,3,2,202,84,0,0,0,0,0,8,1157.907736,1714.733628,1.74
10257,1953,2011-01-20,0,4,0,4,51,5,60,1,2,202,84,0,0,0,0,0,8,1157.907736,1714.733628,1.743826
10258,1953,2011-01-20,1,4,0,6,50.6666679382324,3.33333325386047,60,3,2,202,84,0,0,0,0,0,8,11
10259,1953,2011-01-20,2,4,0,12,41.3333320617676,6.66666650772095,60,3,2,202,84,0,0,0,0,0,8,1
10260,1953,2011-01-20,3,4,0,33,22.1666660308838,4.83333349227905,60,3,2,202,84,0,0,0,0,0,8,1
10261,1953,2011-01-20,4,4,0,35.5,11.25,5,51.75,3,2,202,84,0,0,0,0,0,8,1157.907736,1714.733628,1
10262,1953,2011-01-21,0,5,0,7.33333349227905,30.3333339691162,0.666666686534882,38.3333
10263,1953,2011-01-21,1,5,0,3,53.6666679382324,3.33333325386047,60,3,2,202,84,0,0,0,0,0,8,11
10264,1953,2011-01-21,2,5,0,12.6666669845581,46,1.33333337306976,60,3,2,202,84,0,0,0,0,0,8,1
10265,1953,2011-01-21,3,5,0,17,38.3333320617676,4.66666650772095,60,3,2,202,84,0,0,0,0,0,8,1
10266,1953,2011-01-21,4,5,0,47,11,2,60,3,2,202,84,0,0,0,0,0,8,1157.907736,1714.733628,1.74382
10267,1953,2011-01-22,0,6,1,11.3333330154419,27.6666660308838,1.66666662693024,40.66666
10268,1953,2011-01-22,1,6,1,16.3333339691162,42.6666679382324,1,60,3,2,202,84,0,0,0,0,0,8,11
10269,1953,2011-01-22,2,6,1,11,47.6666679382324,1.33333337306976,60,3,2,202,84,0,0,0,0,0,8,1
10270,1953,2011-01-22,3,6,1,24.6666660308838,33,2.33333325386047,60,3,2,202,84,0,0,0,0,0,8,1
10271,1953,2011-01-22,4,6,1,42,17.6666660308838,0.333333343267441,60,3,2,202,84,0,0,0,0,0,8,
10272,1953,2011-01-23,1,0,1,12.3333330154419,47.3333320617676,0.333333343267441,60,3,2,2
10273,1953,2011-01-23,2,0,1,15.6666669845581,43,1.33333337306976,60,3,2,202,84,0,0,0,0,0,8,1
10274,1953,2011-01-23,3,0,1,8.33333301544189,49.3333320617676,2.33333325386047,60,3,2,20
10275,1953,2011-01-23,4,0,1,47,12.3333330154419,0.666666686534882,60,3,2,202,84,0,0,0,0,0,8,
10276,1963,2011-01-13,0,4,0,27,20,0,47,1,2,202,76,0,0,1,1,0,10,1130.797571,1737.99405,2.505740
10277,1963,2011-01-13,1,4,0,13.6666669845581,38,8.33333301544189,60,3,2,202,76,0,0,1,1,0,10,
10278,1963,2011-01-13,2,4,0,28.3333339691162,31.3333339691162,0.333333343267441,60,3,2,2
10279,1963,2011-01-13,3,4,0,18.3999996185303,36.7999992370605,4.80000019073486,60,3,2,20
10280,1963,2011-01-13,4,4,0,19.3333339691162,30.6666660308838,0,50,3,2,202,76,0,0,1,1,0,10,1
10281,1963,2011-01-14,0,5,0,22,21,0,43,1,2,202,76,0,0,1,1,0,10,1130.797571,1737.99405,2.505740
10282,1963,2011-01-14,1,5,0,22.3333339691162,34.3333320617676,3.33333325386047,60,3,2,20
10283,1963,2011-01-14,2,5,0,33.6666679382324,24.6666660308838,1.66666662693024,60,3,2,20
10284,1963,2011-01-14,3,5,0,23.3333339691162,30,6.66666650772095,60,3,2,202,76,0,0,1,1,0,10,
10285,1963,2011-01-14,4,5,0,40.5999984741211,18.2000007629395,1.20000004768372,60,3,2,20
10286,1963,2011-01-15,0,6,1,29,16,15,60,1,2,202,76,0,0,1,1,0,10,1130.797571,1737.99405,2.5057
10287,1963,2011-01-15,1,6,1,31.6666660308838,24,4,59.6666679382324,3,2,202,76,0,0,1,1,0,10,1
10288,1963,2011-01-15,2,6,1,35.25,10.25,14.5,60,3,2,202,76,0,0,1,1,0,10,1130.797571,1737.99405
10289,1963,2011-01-15,3,6,1,45.6666679382324,6.33333349227905,8,60,3,2,202,76,0,0,1,1,0,10,1
10290,1963,2011-01-15,4,6,1,30.5,7,6,43.5,2,2,202,76,0,0,1,1,0,10,1130.797571,1737.99405,2.505
10291,1963,2011-01-16,1,0,1,18,35.6666679382324,5,58.6666679382324,3,2,202,76,0,0,1,1,0,10,1
10292,1963,2011-01-16,2,0,1,39.4000015258789,15.1999998092651,5.40000009536743,60,3,2,20
10293,1963,2011-01-16,3,0,1,21,29,10,60,3,2,202,76,0,0,1,1,0,10,1130.797571,1737.99405,2.5057
10294,1963,2011-01-16,4,0,1,44.3333320617676,15.6666669845581,0,60,3,2,202,76,0,0,1,1,0,10,1
10295,1963,2011-01-17,0,1,0,31,23,1,55,1,2,202,76,0,0,1,1,0,10,1130.797571,1737.99405,2.505740
10296,1963,2011-01-17,1,1,0,17,30.6666660308838,12.3333330154419,60,3,2,202,76,0,0,1,1,0,10,
10297,1963,2011-01-17,2,1,0,24.6666660308838,31.3333339691162,4,60,3,2,202,76,0,0,1,1,0,10,1
10298,1963,2011-01-17,3,1,0,24,25.3333339691162,10.6666669845581,60,3,2,202,76,0,0,1,1,0,10,
10299,1963,2011-01-17,4,1,0,34.75,22.5,2.75,60,3,2,202,76,0,0,1,1,0,10,1130.797571,1737.99405,

10300,1963,2011-01-18,4,2,0,16.5,43.5,0,60,2,2,202,76,0,0,1,1,0,10,1130.797571,1737.99405,2.505
10301,1963,2011-01-23,0,0,1,8,14,1,23,1,2,202,76,0,0,1,1,0,10,1130.797571,1737.99405,2.505740
10302,1963,2011-01-23,1,0,1,12.5,42.5,5,60,2,2,202,76,0,0,1,1,0,10,1130.797571,1737.99405,2.505
10303,1963,2011-01-24,0,1,0,23,28,0,51,1,2,202,76,0,0,1,1,0,10,1130.797571,1737.99405,2.50574
10304,1963,2011-01-24,1,1,0,11,38.25,10.75,60,3,2,202,76,0,0,1,1,0,10,1130.797571,1737.99405,2
10305,1963,2011-01-24,2,1,0,46,13.6666669845581,0.333333343267441,60,3,2,202,76,0,0,1,1,0,1
10306,1963,2011-01-24,3,1,0,39.3333320617676,16.3333339691162,4.33333349227905,60,3,2,20
10307,1963,2011-01-24,4,1,0,37,23,0,60,3,2,202,76,0,0,1,1,0,10,1130.797571,1737.99405,2.50574
10308,1963,2011-01-25,1,2,0,16.5,18,18,52.5,2,2,202,76,0,0,1,1,0,10,1130.797571,1737.99405,2.50
10309,1963,2011-01-25,2,2,0,28,28.5,3.5,60,3,2,202,76,0,0,1,1,0,10,1130.797571,1737.99405,2.505
10310,1963,2011-01-25,3,2,0,21.25,32.25,6.5,60,3,2,202,76,0,0,1,1,0,10,1130.797571,1737.99405,2
10311,1963,2011-01-25,4,2,0,34,22.6666660308838,3.33333325386047,60,3,2,202,76,0,0,1,1,0,10,
10312,1966,2011-01-18,0,2,0,26,19,1,46,2,2,202,70,0,1,0,1,0,9,1149.942557,1612.295291,3.241242
10313,1966,2011-01-18,1,2,0,32.3333320617676,27.3333339691162,0.333333343267441,60,3,2,2
10314,1966,2011-01-18,2,2,0,33.6666679382324,21.3333339691162,5,60,3,2,202,70,0,1,0,1,0,9,11
10315,1966,2011-01-18,3,2,0,48.3333320617676,11.6666669845581,0,60,3,2,202,70,0,1,0,1,0,9,11
10316,1966,2011-01-18,4,2,0,46.6666679382324,13.3333330154419,0,60,3,2,202,70,0,1,0,1,0,9,11
10317,1966,2011-01-19,1,3,0,32.6666679382324,27.3333339691162,0,60,3,2,202,70,0,1,0,1,0,9,11
10318,1966,2011-01-19,2,3,0,40,19.3333339691162,0.666666686534882,60,3,2,202,70,0,1,0,1,0,9,
10319,1966,2011-01-19,3,3,0,41.6666679382324,12.3333330154419,6,60,3,2,202,70,0,1,0,1,0,9,11
10320,1966,2011-01-19,4,3,0,44,14.3333330154419,1.66666662693024,60,3,2,202,70,0,1,0,1,0,9,1
10321,1966,2011-02-03,0,4,0,18.5,15,1,34.5,2,2,202,70,0,1,0,1,0,9,1149.942557,1612.295291,3.241
10322,1966,2011-02-03,1,4,0,35.5999984741211,20.3999996185303,4,60,3,2,202,70,0,1,0,1,0,9,11
10323,1966,2011-02-03,2,4,0,28.6666660308838,28.3333339691162,3,60,3,2,202,70,0,1,0,1,0,9,11
10324,1966,2011-02-03,3,4,0,24,34.3333320617676,1.66666662693024,60,3,2,202,70,0,1,0,1,0,9,1
10325,1966,2011-02-03,4,4,0,37.6666679382324,21.6666660308838,0.666666686534882,60,3,2,2
10326,1966,2011-02-04,0,5,0,15,25,0,40,1,2,202,70,0,1,0,1,0,9,1149.942557,1612.295291,3.241242
10327,1966,2011-02-04,1,5,0,29.75,26,4.25,60,3,2,202,70,0,1,0,1,0,9,1149.942557,1612.295291,3.
10328,1966,2011-02-04,2,5,0,39.4000015258789,16,4.59999990463257,60,3,2,202,70,0,1,0,1,0,9,1
10329,1966,2011-02-04,3,5,0,32.6666679382324,26.6666660308838,0.666666686534882,60,3,2,2
10330,1966,2011-02-04,4,5,0,37.3333320617676,22,0.666666686534882,60,3,2,202,70,0,1,0,1,0,9,
10331,1966,2011-02-05,0,6,1,20,25,3,48,1,2,202,70,0,1,0,1,0,9,1149.942557,1612.295291,3.241242
10332,1966,2011-02-05,1,6,1,35.6666679382324,22.6666660308838,1.66666662693024,60,3,2,20
10333,1966,2011-02-05,2,6,1,37.75,20.75,1.5,60,3,2,202,70,0,1,0,1,0,9,1149.942557,1612.295291,
10334,1966,2011-02-05,3,6,1,36,18.7999992370605,5.19999980926514,60,3,2,202,70,0,1,0,1,0,9,1
10335,1966,2011-02-05,4,6,1,46.6666679382324,11.6666669845581,1.66666662693024,60,3,2,20
10336,1966,2011-02-06,0,0,1,0,3,1,4,1,2,202,70,0,1,0,1,0,9,1149.942557,1612.295291,3.24124250
10337,1966,2011-02-06,1,0,1,40,16,4,60,3,2,202,70,0,1,0,1,0,9,1149.942557,1612.295291,3.241242
10338,1966,2011-02-06,2,0,1,39.3333320617676,18,2.66666674613953,60,3,2,202,70,0,1,0,1,0,9,1
10339,1966,2011-02-06,3,0,1,36.5,20.25,3.25,60,3,2,202,70,0,1,0,1,0,9,1149.942557,1612.295291,
10340,1966,2011-02-06,4,0,1,44,15,1,60,3,2,202,70,0,1,0,1,0,9,1149.942557,1612.295291,3.241242
10341,1966,2011-02-07,4,1,0,60,0,0,60,1,2,202,70,0,1,0,1,0,9,1149.942557,1612.295291,3.241242
10342,1968,2011-02-07,0,1,0,9.66666698455811,31,4,44.6666679382324,3,2,202,71,1,1,0,1,0,10,1
10343,1968,2011-02-07,1,1,0,33,26.6666660308838,0.333333343267441,60,3,2,202,71,1,1,0,1,0,1
10344,1968,2011-02-07,2,1,0,28.6666660308838,24.6666660308838,6.66666650772095,60,3,2,20
10345,1968,2011-02-07,3,1,0,36.6666679382324,22,1.33333337306976,60,3,2,202,71,1,1,0,1,0,10,
10346,1968,2011-02-07,4,1,0,56,4,0,60,1,2,202,71,1,1,0,1,0,10,1156.624345,1079.749992,3.97647
10347,1968,2011-02-08,0,2,0,14,41.6666679382324,4.33333349227905,60,3,2,202,71,1,1,0,1,0,10,
10348,1968,2011-02-08,1,2,0,33.6666679382324,19.3333339691162,7,60,3,2,202,71,1,1,0,1,0,10,1
10349,1968,2011-02-08,2,2,0,22.3333339691162,31.6666660308838,6,60,3,2,202,71,1,1,0,1,0,10,1

10350,1968,2011-02-08,3,2,0,31.3333339691162,28.6666660308838,0,60,3,2,202,71,1,1,0,1,0,10,1
10351,1968,2011-02-08,4,2,0,33.6666679382324,14.3333330154419,0,48,3,2,202,71,1,1,0,1,0,10,1
10352,1968,2011-02-09,0,3,0,11.8888893127441,43.5555572509766,4.22222232818604,59.66666
10353,1968,2011-02-09,1,3,0,21.75,35.375,2.875,60,3,2,202,71,1,1,0,1,0,10,1156.624345,1079.749
10354,1968,2011-02-09,2,3,0,19.1666660308838,39,1.83333337306976,60,3,2,202,71,1,1,0,1,0,10,
10355,1968,2011-02-09,3,3,0,33.6666679382324,15.6666669845581,1.5,50.8333320617676,3,2,20
10356,1968,2011-02-09,4,3,0,24,22,1,47,1,2,202,71,1,1,0,1,0,10,1156.624345,1079.749992,3.9764
10357,1968,2011-02-10,0,4,0,9,29,7,45,3,2,202,71,1,1,0,1,0,10,1156.624345,1079.749992,3.97647
10358,1968,2011-02-10,1,4,0,29.5,30.25,0.25,60,3,2,202,71,1,1,0,1,0,10,1156.624345,1079.749992
10359,1968,2011-02-10,2,4,0,19,41,0,60,3,2,202,71,1,1,0,1,0,10,1156.624345,1079.749992,3.9764
10360,1968,2011-02-10,3,4,0,15.3333330154419,41.6666679382324,3,60,3,2,202,71,1,1,0,1,0,10,1
10361,1968,2011-02-10,4,4,0,20,19,1.33333337306976,40.3333320617676,3,2,202,71,1,1,0,1,0,10,
10362,1968,2011-02-11,2,5,0,13.6666669845581,45,1.33333337306976,60,3,2,202,71,1,1,0,1,0,10,
10363,1968,2011-02-11,3,5,0,37.3333320617676,22.3333339691162,0.333333343267441,60,3,2,2
10364,1968,2011-02-11,4,5,0,39,12,1,52,1,2,202,71,1,1,0,1,0,10,1156.624345,1079.749992,3.9764
10365,1969,2011-02-11,0,5,0,40,20,0,60,1,2,202,74,1,1,0,1,0,9,1148.501832,1579.356671,3.16829
10366,1969,2011-02-11,1,5,0,15,26.6666660308838,18.3333339691162,60,3,2,202,74,1,1,0,1,0,9,1
10367,1969,2011-02-11,2,5,0,42,8.66666698455811,9.33333301544189,60,3,2,202,74,1,1,0,1,0,9,1
10368,1969,2011-02-12,0,6,1,24.5,13,1,38.5,2,2,202,74,1,1,0,1,0,9,1148.501832,1579.356671,3.16
10369,1969,2011-02-12,1,6,1,28.6666660308838,30,1.33333337306976,60,3,2,202,74,1,1,0,1,0,9,1
10370,1969,2011-02-12,2,6,1,9.5,22,3,34.5,2,2,202,74,1,1,0,1,0,9,1148.501832,1579.356671,3.168
10371,1969,2011-02-12,3,6,1,19.1666660308838,27.8333339691162,13,60,3,2,202,74,1,1,0,1,0,9,1
10372,1969,2011-02-12,4,6,1,47.3333320617676,11.6666669845581,1,60,3,2,202,74,1,1,0,1,0,9,11
10373,1969,2011-02-13,0,0,1,24,22,7,53,1,2,202,74,1,1,0,1,0,9,1148.501832,1579.356671,3.16829
10374,1969,2011-02-13,1,0,1,14.3333330154419,43,2.66666674613953,60,3,2,202,74,1,1,0,1,0,9,1
10375,1969,2011-02-13,2,0,1,22.6666660308838,36.3333320617676,1,60,3,2,202,74,1,1,0,1,0,9,11
10376,1969,2011-02-13,3,0,1,27.1666660308838,30.6666660308838,2.16666674613953,60,3,2,20
10377,1969,2011-02-13,4,0,1,31.4285717010498,26.8571434020996,1.71428573131561,60,3,2,20
10378,1969,2011-02-14,0,1,0,6,11,1,18,1,2,202,74,1,1,0,1,0,9,1148.501832,1579.356671,3.168290
10379,1969,2011-02-14,1,1,0,26,22.5,5.25,53.75,3,2,202,74,1,1,0,1,0,9,1148.501832,1579.356671,
10380,1969,2011-02-14,2,1,0,32.3333320617676,18,9.66666698455811,60,3,2,202,74,1,1,0,1,0,9,1
10381,1969,2011-02-14,3,1,0,8.66666698455811,24,27.3333339691162,60,3,2,202,74,1,1,0,1,0,9,1
10382,1969,2011-02-14,4,1,0,40.5999984741211,19,0.400000005960464,60,3,2,202,74,1,1,0,1,0,9,
10383,1969,2011-02-15,1,2,0,9.5,37,9.25,55.75,3,2,202,74,1,1,0,1,0,9,1148.501832,1579.356671,3.
10384,1969,2011-02-15,2,2,0,30.6666660308838,22.3333339691162,7,60,3,2,202,74,1,1,0,1,0,9,11
10385,1969,2011-02-15,3,2,0,9,36.6666679382324,14.3333330154419,60,3,2,202,74,1,1,0,1,0,9,11
10386,1969,2011-02-15,4,2,0,33.6666679382324,25.3333339691162,1,60,3,2,202,74,1,1,0,1,0,9,11
10387,1969,2011-02-16,0,3,0,17,37,4,58,1,2,202,74,1,1,0,1,0,9,1148.501832,1579.356671,3.16829
10388,1969,2011-02-16,1,3,0,33.75,22.5,3.75,60,3,2,202,74,1,1,0,1,0,9,1148.501832,1579.356671,
10389,1969,2011-02-16,2,3,0,27,30.8333339691162,2.16666674613953,60,3,2,202,74,1,1,0,1,0,9,1
10390,1969,2011-02-16,3,3,0,24.6666660308838,29.3333339691162,6,60,3,2,202,74,1,1,0,1,0,9,11
10391,1969,2011-02-16,4,3,0,31.6666660308838,27.3333339691162,1,60,3,2,202,74,1,1,0,1,0,9,11
10392,1972,2011-02-05,0,6,1,43,17,0,60,2,2,202,89,1,0,1,0,0,3,1159.32977,1836.274587,3.360730
10393,1972,2011-02-05,1,6,1,23.3333339691162,36.6666679382324,0,60,3,2,202,89,1,0,1,0,0,3,11
10394,1972,2011-02-05,2,6,1,34.6666679382324,25.3333339691162,0,60,3,2,202,89,1,0,1,0,0,3,11
10395,1972,2011-02-05,3,6,1,28,32,0,60,3,2,202,89,1,0,1,0,0,3,1159.32977,1836.274587,3.360730
10396,1972,2011-02-05,4,6,1,36,12.6666669845581,0,48.6666679382324,3,2,202,89,1,0,1,0,0,3,11
10397,1972,2011-02-13,0,0,1,38.3333320617676,20.6666660308838,0.666666686534882,59.66666
10398,1972,2011-02-13,1,0,1,34.6666679382324,25.3333339691162,0,60,3,2,202,89,1,0,1,0,0,3,11
10399,1972,2011-02-13,2,0,1,39,21,0,60,3,2,202,89,1,0,1,0,0,3,1159.32977,1836.274587,3.360730

10400,1972,2011-02-13,3,0,1,31,29,0,60,1,2,202,89,1,0,1,0,0,3,1159.32977,1836.274587,3.360730
10401,1972,2011-02-14,0,1,0,45.3333320617676,11.6666669845581,0,57,3,2,202,89,1,0,1,0,0,3,11
10402,1972,2011-02-14,1,1,0,43.6666679382324,16.3333339691162,0,60,3,2,202,89,1,0,1,0,0,3,11
10403,1972,2011-02-14,2,1,0,34.6666679382324,25.3333339691162,0,60,3,2,202,89,1,0,1,0,0,3,11
10404,1972,2011-02-14,3,1,0,29.6666660308838,30.3333339691162,0,60,3,2,202,89,1,0,1,0,0,3,11
10405,1972,2011-02-14,4,1,0,35.3333320617676,9.66666698455811,0.333333343267441,45.3333
10406,1972,2011-02-15,0,2,0,41.6666679382324,16.6666660308838,0,58.3333320617676,3,2,202,
10407,1972,2011-02-15,1,2,0,34.3333320617676,25.6666660308838,0,60,3,2,202,89,1,0,1,0,0,3,11
10408,1972,2011-02-15,2,2,0,37.3333320617676,22.6666660308838,0,60,3,2,202,89,1,0,1,0,0,3,11
10409,1972,2011-02-15,3,2,0,30,30,0,60,2,2,202,89,1,0,1,0,0,3,1159.32977,1836.274587,3.360730
10410,1972,2011-02-15,4,2,0,52.6666679382324,7.33333349227905,0,60,3,2,202,89,1,0,1,0,0,3,11
10411,1972,2011-02-16,0,3,0,25.3333339691162,34.6666679382324,0,60,3,2,202,89,1,0,1,0,0,3,11
10412,1972,2011-02-16,1,3,0,28,32,0,60,3,2,202,89,1,0,1,0,0,3,1159.32977,1836.274587,3.360730
10413,1972,2011-02-16,2,3,0,25.3333339691162,34.6666679382324,0,60,3,2,202,89,1,0,1,0,0,3,11
10414,1972,2011-02-16,3,3,0,34.8333320617676,25.1666660308838,0,60,3,2,202,89,1,0,1,0,0,3,11
10415,1972,2011-02-16,4,3,0,38.6666679382324,20.3333339691162,0,59,3,2,202,89,1,0,1,0,0,3,11
10416,1972,2011-02-17,0,4,0,32.6666679382324,21.6666660308838,0,54.3333320617676,3,2,202,
10417,1972,2011-02-17,1,4,0,40,18,0,58,3,2,202,89,1,0,1,0,0,3,1159.32977,1836.274587,3.360730
10418,1972,2011-02-17,2,4,0,17.5,28.5,0,46,2,2,202,89,1,0,1,0,0,3,1159.32977,1836.274587,3.360
10419,1972,2011-02-17,3,4,0,33,27,0,60,3,2,202,89,1,0,1,0,0,3,1159.32977,1836.274587,3.360730
10420,1972,2011-02-17,4,4,0,34.75,13.75,0.5,49,3,2,202,89,1,0,1,0,0,3,1159.32977,1836.274587,3.
10421,1972,2011-02-18,0,5,0,36,24,0,60,3,2,202,89,1,0,1,0,0,3,1159.32977,1836.274587,3.360730
10422,1972,2011-02-18,1,5,0,40.3333320617676,19.6666660308838,0,60,3,2,202,89,1,0,1,0,0,3,11
10423,1972,2011-02-18,2,5,0,41.3333320617676,18.6666660308838,0,60,3,2,202,89,1,0,1,0,0,3,11
10424,1972,2011-02-18,3,5,0,40,20,0,60,3,2,202,89,1,0,1,0,0,3,1159.32977,1836.274587,3.360730
10425,1972,2011-02-18,4,5,0,44,16,0,60,3,2,202,89,1,0,1,0,0,3,1159.32977,1836.274587,3.360730
10426,1974,2011-02-05,0,6,1,17.5,30,4.5,52,2,2,202,76,1,0,0,1,0,12,1183.431509,1071.129237,3.7
10427,1974,2011-02-06,0,0,1,4.75,34.25,21,60,3,2,202,76,1,0,0,1,0,12,1183.431509,1071.129237,3
10428,1974,2011-02-06,1,0,1,27.5,27.8333339691162,4.66666650772095,60,3,2,202,76,1,0,0,1,0,1
10429,1974,2011-02-06,2,0,1,22.3333339691162,32.6666679382324,5,60,3,2,202,76,1,0,0,1,0,12,1
10430,1974,2011-02-06,3,0,1,39,20.6666660308838,0.333333343267441,60,3,2,202,76,1,0,0,1,0,1
10431,1974,2011-02-06,4,0,1,39,20.6666660308838,0.333333343267441,60,3,2,202,76,1,0,0,1,0,1
10432,1974,2011-02-07,0,1,0,12.3333330154419,28.3333339691162,6.66666650772095,47.33333
10433,1974,2011-02-07,1,1,0,21,32.3333320617676,6.66666650772095,60,3,2,202,76,1,0,0,1,0,12,
10434,1974,2011-02-07,2,1,0,22.6000003814697,34.7999992370605,2.59999990463257,60,3,2,20
10435,1974,2011-02-07,3,1,0,12,38.3333320617676,9.66666698455811,60,3,2,202,76,1,0,0,1,0,12,
10436,1974,2011-02-07,4,1,0,20.3333339691162,38.6666679382324,1,60,3,2,202,76,1,0,0,1,0,12,1
10437,1974,2011-02-08,0,2,0,15,30.3333339691162,13.3333330154419,58.6666679382324,3,2,20
10438,1974,2011-02-08,1,2,0,26.6666660308838,31.6666660308838,1.66666662693024,60,3,2,20
10439,1974,2011-02-08,2,2,0,20.25,35.5,4.25,60,3,2,202,76,1,0,0,1,0,12,1183.431509,1071.129237
10440,1974,2011-02-08,3,2,0,26.7999992370605,28,5.19999980926514,60,3,2,202,76,1,0,0,1,0,12,
10441,1974,2011-02-08,4,2,0,27,28.6666660308838,0.666666686534882,56.3333320617676,3,2,2
10442,1974,2011-02-09,0,3,0,19.3333339691162,28.3333339691162,9.33333301544189,57,3,2,20
10443,1974,2011-02-09,1,3,0,24.3333339691162,32.3333320617676,3.33333325386047,60,3,2,20
10444,1974,2011-02-09,2,3,0,48,10.3333330154419,1.66666662693024,60,3,2,202,76,1,0,0,1,0,12,
10445,1974,2011-02-09,3,3,0,35,18.75,6.25,60,3,2,202,76,1,0,0,1,0,12,1183.431509,1071.129237,3
10446,1974,2011-02-09,4,3,0,43.25,15,1.75,60,3,2,202,76,1,0,0,1,0,12,1183.431509,1071.129237,3
10447,1974,2011-02-10,0,4,0,19.25,24.75,14,58,3,2,202,76,1,0,0,1,0,12,1183.431509,1071.129237,
10448,1974,2011-02-10,1,4,0,33.6666679382324,22.6666660308838,3.66666674613953,60,3,2,20
10449,1974,2011-02-10,2,4,0,46.6666679382324,13,0.333333343267441,60,3,2,202,76,1,0,0,1,0,1

10450,1974,2011-02-10,3,4,0,40.6666679382324,18.6666660308838,0.666666686534882,60,3,2,2
10451,1974,2011-02-10,4,4,0,48.4000015258789,10.6000003814697,1,60,3,2,202,76,1,0,0,1,0,12,1
10452,1974,2011-02-11,0,5,0,27.5,30,2.5,60,2,2,202,76,1,0,0,1,0,12,1183.431509,1071.129237,3.72
10453,1974,2011-02-11,1,5,0,23,28,9,60,3,2,202,76,1,0,0,1,0,12,1183.431509,1071.129237,3.72947
10454,1974,2011-02-11,2,5,0,28.3333339691162,30.6666660308838,1,60,3,2,202,76,1,0,0,1,0,12,1
10455,1974,2011-02-11,3,5,0,44.3333320617676,14.6666669845581,1,60,3,2,202,76,1,0,0,1,0,12,1
10456,1974,2011-02-11,4,5,0,48.6666679382324,9.66666698455811,1.66666662693024,60,3,2,20
10457,1977,2011-02-11,0,5,0,29,31,0,60,1,2,202,83,1,1,1,1,0,8,1174.845535,1875.468722,3.51725
10458,1977,2011-02-11,1,5,0,43,17,0,60,3,2,202,83,1,1,1,1,0,8,1174.845535,1875.468722,3.51725
10459,1977,2011-02-11,2,5,0,37,23,0,60,1,2,202,83,1,1,1,1,0,8,1174.845535,1875.468722,3.51725
10460,1977,2011-02-12,0,6,1,28,14,0.5,42.5,2,2,202,83,1,1,1,1,0,8,1174.845535,1875.468722,3.517
10461,1977,2011-02-12,1,6,1,36.3333320617676,22.6666660308838,1,60,3,2,202,83,1,1,1,1,0,8,11
10462,1977,2011-02-12,2,6,1,41.5999984741211,18.3999996185303,0,60,3,2,202,83,1,1,1,1,0,8,11
10463,1977,2011-02-12,3,6,1,38.5999984741211,20.6000003814697,0.800000011920929,60,3,2,2
10464,1977,2011-02-12,4,6,1,45.6666679382324,14.3333330154419,0,60,3,2,202,83,1,1,1,1,0,8,11
10465,1977,2011-02-13,0,0,1,28,11,0.5,39.5,2,2,202,83,1,1,1,1,0,8,1174.845535,1875.468722,3.517
10466,1977,2011-02-13,1,0,1,43.3333320617676,16.3333339691162,0.333333343267441,60,3,2,2
10467,1977,2011-02-13,2,0,1,43,17,0,60,3,2,202,83,1,1,1,1,0,8,1174.845535,1875.468722,3.51725
10468,1977,2011-02-13,3,0,1,43.25,16.75,0,60,3,2,202,83,1,1,1,1,0,8,1174.845535,1875.468722,3.
10469,1977,2011-02-13,4,0,1,41.5,18.5,0,60,3,2,202,83,1,1,1,1,0,8,1174.845535,1875.468722,3.517
10470,1977,2011-02-14,0,1,0,33.75,13.25,0.5,47.5,3,2,202,83,1,1,1,1,0,8,1174.845535,1875.46872
10471,1977,2011-02-14,1,1,0,43,16.75,0.25,60,3,2,202,83,1,1,1,1,0,8,1174.845535,1875.468722,3.
10472,1977,2011-02-14,2,1,0,54.3333320617676,5.66666650772095,0,60,3,2,202,83,1,1,1,1,0,8,11
10473,1977,2011-02-14,3,1,0,39.6666679382324,20.3333339691162,0,60,3,2,202,83,1,1,1,1,0,8,11
10474,1977,2011-02-14,4,1,0,46.3333320617676,13.6666669845581,0,60,3,2,202,83,1,1,1,1,0,8,11
10475,1977,2011-02-15,0,2,0,16.5,22,0,38.5,2,2,202,83,1,1,1,1,0,8,1174.845535,1875.468722,3.517
10476,1977,2011-02-15,1,2,0,39.2000007629395,20.7999992370605,0,60,3,2,202,83,1,1,1,1,0,8,11
10477,1977,2011-02-15,2,2,0,45.8333320617676,14,0.16666667163372,60,3,2,202,83,1,1,1,1,0,8,1
10478,1977,2011-02-15,3,2,0,39.3333320617676,20,0.666666686534882,60,3,2,202,83,1,1,1,1,0,8,
10479,1977,2011-02-15,4,2,0,46,14,0,60,3,2,202,83,1,1,1,1,0,8,1174.845535,1875.468722,3.51725
10480,1977,2011-02-16,0,3,0,2,2,2,6,1,2,202,83,1,1,1,1,0,8,1174.845535,1875.468722,3.51725885
10481,1977,2011-02-16,1,3,0,33.3333320617676,26.6666660308838,0,60,3,2,202,83,1,1,1,1,0,8,11
10482,1977,2011-02-16,2,3,0,48.6666679382324,11.3333330154419,0,60,3,2,202,83,1,1,1,1,0,8,11
10483,1977,2011-02-16,3,3,0,43,16.6666660308838,0.333333343267441,60,3,2,202,83,1,1,1,1,0,8,
10484,1977,2011-02-16,4,3,0,40.75,19.25,0,60,3,2,202,83,1,1,1,1,0,8,1174.845535,1875.468722,3.
10485,1977,2011-03-09,0,3,0,20,30,1,51,1,2,202,83,1,1,1,1,0,8,1174.845535,1875.468722,3.51725
10486,1977,2011-03-09,1,3,0,38.3333320617676,21.6666660308838,0,60,3,2,202,83,1,1,1,1,0,8,11
10487,1977,2011-03-09,2,3,0,37,21,2,60,3,2,202,83,1,1,1,1,0,8,1174.845535,1875.468722,3.51725
10488,1977,2011-03-09,3,3,0,36,23,1,60,3,2,202,83,1,1,1,1,0,8,1174.845535,1875.468722,3.51725
10489,1977,2011-03-09,4,3,0,50,10,0,60,3,2,202,83,1,1,1,1,0,8,1174.845535,1875.468722,3.51725
10490,2001,2011-03-10,0,4,0,34.3333320617676,13.6666669845581,0.666666686534882,48.6666
10491,2001,2011-03-10,1,4,0,41,15.75,3.25,60,3,2,202,85,0,1,1,1,0,8,992.8601953,1730.90574,2.1
10492,2001,2011-03-10,2,4,0,40,19.6666660308838,0.333333343267441,60,3,2,202,85,0,1,1,1,0,8,
10493,2001,2011-03-10,3,4,0,44.6666679382324,15.3333330154419,0,60,3,2,202,85,0,1,1,1,0,8,99
10494,2001,2011-03-10,4,4,0,41.6666679382324,17.3333339691162,0.333333343267441,59.3333
10495,2001,2011-03-11,0,5,0,25,5.5,0,30.5,2,2,202,85,0,1,1,1,0,8,992.8601953,1730.90574,2.1306
10496,2001,2011-03-11,1,5,0,49,9.19999980926514,0.200000002980232,58.4000015258789,3,2,2
10497,2001,2011-03-11,2,5,0,47.3333320617676,5.33333349227905,0,52.6666679382324,3,2,202,
10498,2001,2011-03-11,3,5,0,44.6666679382324,15.3333330154419,0,60,3,2,202,85,0,1,1,1,0,8,99
10499,2001,2011-03-11,4,5,0,45.3333320617676,14.6666669845581,0,60,3,2,202,85,0,1,1,1,0,8,99

10500,2001,2011-03-12,0,6,1,39.5,19.5,0.5,59.5,2,2,202,85,0,1,1,1,0,8,992.8601953,1730.90574,2.
10501,2001,2011-03-12,1,6,1,30.6666660308838,28,1.33333337306976,60,3,2,202,85,0,1,1,1,0,8,9
10502,2001,2011-03-12,2,6,1,43.2000007629395,16.3999996185303,0.400000005960464,60,3,2,2
10503,2001,2011-03-12,3,6,1,48.6666679382324,11.3333330154419,0,60,3,2,202,85,0,1,1,1,0,8,99
10504,2001,2011-03-12,4,6,1,50.3333320617676,9.66666698455811,0,60,3,2,202,85,0,1,1,1,0,8,99
10505,2001,2011-03-13,0,0,1,25.6666660308838,16.6666660308838,0.333333343267441,42.6666
10506,2001,2011-03-13,1,0,1,36,24,0,60,3,2,202,85,0,1,1,1,0,8,992.8601953,1730.90574,2.130682
10507,2001,2011-03-13,2,0,1,31,29,0,60,3,2,202,85,0,1,1,1,0,8,992.8601953,1730.90574,2.130682
10508,2001,2011-03-13,3,0,1,37.7999992370605,22,0.200000002980232,60,3,2,202,85,0,1,1,1,0,8,
10509,2001,2011-03-13,4,0,1,39,20,1,60,3,2,202,85,0,1,1,1,0,8,992.8601953,1730.90574,2.130682
10510,2001,2011-03-14,0,1,0,31.6666660308838,15,0.666666686534882,47.3333320617676,3,2,2
10511,2001,2011-03-14,1,1,0,52.6666679382324,7.33333349227905,0,60,3,2,202,85,0,1,1,1,0,8,99
10512,2001,2011-03-14,2,1,0,40.6666679382324,19.3333339691162,0,60,3,2,202,85,0,1,1,1,0,8,99
10513,2001,2011-03-14,3,1,0,47.75,12.25,0,60,3,2,202,85,0,1,1,1,0,8,992.8601953,1730.90574,2.1
10514,2001,2011-03-14,4,1,0,52.3333320617676,7.66666650772095,0,60,3,2,202,85,0,1,1,1,0,8,99
10515,2001,2011-03-15,0,2,0,33,21.6666660308838,0.333333343267441,55,3,2,202,85,0,1,1,1,0,8,
10516,2001,2011-03-15,1,2,0,44.6666679382324,15.3333330154419,0,60,3,2,202,85,0,1,1,1,0,8,99
10517,2001,2011-03-15,2,2,0,39.3333320617676,20.3333339691162,0.333333343267441,60,3,2,2
10518,2001,2011-03-15,3,2,0,40.3333320617676,19.6666660308838,0,60,3,2,202,85,0,1,1,1,0,8,99
10519,2001,2011-03-15,4,2,0,48.75,10.75,0.5,60,3,2,202,85,0,1,1,1,0,8,992.8601953,1730.90574,2.
10520,2001,2012-06-05,0,2,0,34,13.3333330154419,1,48.3333320617676,3,2,202,85,0,1,1,1,0,8,99
10521,2001,2012-06-05,1,2,0,47.6666679382324,12.3333330154419,0,60,3,2,202,85,0,1,1,1,0,8,99
10522,2001,2012-06-05,2,2,0,52.6666679382324,7.33333349227905,0,60,3,2,202,85,0,1,1,1,0,8,99
10523,2001,2012-06-05,3,2,0,50,10,0,60,1,2,202,85,0,1,1,1,0,8,992.8601953,1730.90574,2.130682
10524,2001,2012-06-05,4,2,0,46,9.5,0,55.5,2,2,202,85,0,1,1,1,0,8,992.8601953,1730.90574,2.13068
10525,2001,2012-06-06,0,3,0,20,4,1,25,1,2,202,85,0,1,1,1,0,8,992.8601953,1730.90574,2.1306827
10526,2001,2012-06-06,1,3,0,52.6666679382324,7.33333349227905,0,60,3,2,202,85,0,1,1,1,0,8,99
10527,2001,2012-06-06,2,3,0,47.6666679382324,12.3333330154419,0,60,3,2,202,85,0,1,1,1,0,8,99
10528,2001,2012-06-06,3,3,0,28.3333339691162,31.6666660308838,0,60,3,2,202,85,0,1,1,1,0,8,99
10529,2001,2012-06-06,4,3,0,45,13.6666669845581,0,58.6666679382324,3,2,202,85,0,1,1,1,0,8,99
10530,2001,2012-06-07,3,4,0,38,22,0,60,2,2,202,85,0,1,1,1,0,8,992.8601953,1730.90574,2.130682
10531,2001,2012-06-07,4,4,0,49.3333320617676,10.6666669845581,0,60,3,2,202,85,0,1,1,1,0,8,99
10532,2017,2012-06-07,0,4,0,48,11,1,60,1,2,202,91,0,0,1,1,0,10,1240.638285,2229.030142,0,0,0,0,
10533,2017,2012-06-07,1,4,0,16,43.3333320617676,0.666666686534882,60,3,2,202,91,0,0,1,1,0,1
10534,2017,2012-06-07,2,4,0,33.6666679382324,26.3333339691162,0,60,3,2,202,91,0,0,1,1,0,10,1
10535,2017,2012-06-07,3,4,0,5,55,0,60,1,2,202,91,0,0,1,1,0,10,1240.638285,2229.030142,0,0,0,0,0
10536,2017,2012-06-08,0,5,0,21,7,0,28,1,2,202,91,0,0,1,1,0,10,1240.638285,2229.030142,0,0,0,0,0
10537,2017,2012-06-08,1,5,0,6.66666650772095,51,2.33333325386047,60,3,2,202,91,0,0,1,1,0,10,
10538,2017,2012-06-08,2,5,0,46,14,0,60,3,2,202,91,0,0,1,1,0,10,1240.638285,2229.030142,0,0,0,0,
10539,2017,2012-06-08,3,5,0,13.5,46.5,0,60,2,2,202,91,0,0,1,1,0,10,1240.638285,2229.030142,0,0,
10540,2017,2012-06-08,4,5,0,40.3333320617676,19.6666660308838,0,60,3,2,202,91,0,0,1,1,0,10,1
10541,2017,2012-06-10,0,0,1,10,9,0,19,1,2,202,91,0,0,1,1,0,10,1240.638285,2229.030142,0,0,0,0,0
10542,2017,2012-06-10,1,0,1,25.6666660308838,33,1.33333337306976,60,3,2,202,91,0,0,1,1,0,10,
10543,2017,2012-06-10,2,0,1,30,29.5,0.5,60,2,2,202,91,0,0,1,1,0,10,1240.638285,2229.030142,0,0,
10544,2017,2012-06-10,3,0,1,23.3333339691162,36.6666679382324,0,60,3,2,202,91,0,0,1,1,0,10,1
10545,2017,2012-06-10,4,0,1,49,11,0,60,3,2,202,91,0,0,1,1,0,10,1240.638285,2229.030142,0,0,0,0,
10546,2017,2012-06-11,0,1,0,12,22,0,34,1,2,202,91,0,0,1,1,0,10,1240.638285,2229.030142,0,0,0,0,
10547,2017,2012-06-11,1,1,0,38.3333320617676,21.6666660308838,0,60,3,2,202,91,0,0,1,1,0,10,1
10548,2017,2012-06-11,2,1,0,40.6666679382324,19.3333339691162,0,60,3,2,202,91,0,0,1,1,0,10,1
10549,2017,2012-06-11,3,1,0,16.3333339691162,30.6666660308838,13,60,3,2,202,91,0,0,1,1,0,10,

10550,2017,2012-06-11,4,1,0,51.3333320617676,8.66666698455811,0,60,3,2,202,91,0,0,1,1,0,10,1
10551,2017,2012-06-23,0,6,1,27,12,0,39,1,2,202,91,0,0,1,1,0,10,1240.638285,2229.030142,0,0,0,0,
10552,2017,2012-06-23,1,6,1,9.66666698455811,49.6666679382324,0.666666686534882,60,3,2,20
10553,2017,2012-06-23,2,6,1,53.3333320617676,6.66666650772095,0,60,3,2,202,91,0,0,1,1,0,10,1
10554,2017,2012-06-23,3,6,1,40,20,0,60,3,2,202,91,0,0,1,1,0,10,1240.638285,2229.030142,0,0,0,0,
10555,2017,2012-06-23,4,6,1,44.3333320617676,15.6666669845581,0,60,3,2,202,91,0,0,1,1,0,10,1
10556,2017,2012-06-24,0,0,1,3,5,0,8,1,2,202,91,0,0,1,1,0,10,1240.638285,2229.030142,0,0,0,0,0,0,
10557,2017,2012-06-24,1,0,1,16.6666660308838,43,0.333333343267441,60,3,2,202,91,0,0,1,1,0,10
10558,2017,2012-06-24,2,0,1,34.75,24.5,0.75,60,3,2,202,91,0,0,1,1,0,10,1240.638285,2229.030142
10559,2017,2012-06-24,3,0,1,12.25,47.75,0,60,3,2,202,91,0,0,1,1,0,10,1240.638285,2229.030142,0
10560,2017,2012-06-24,4,0,1,50,10,0,60,3,2,202,91,0,0,1,1,0,10,1240.638285,2229.030142,0,0,0,0,
10561,2017,2012-06-25,0,1,0,24.5,7,0.5,32,2,2,202,91,0,0,1,1,0,10,1240.638285,2229.030142,0,0,0
10562,2017,2012-06-25,1,1,0,10.6666669845581,49.3333320617676,0,60,3,2,202,91,0,0,1,1,0,10,1
10563,2017,2012-06-25,2,1,0,35,25,0,60,3,2,202,91,0,0,1,1,0,10,1240.638285,2229.030142,0,0,0,0,
10564,2017,2012-06-25,3,1,0,7.66666650772095,52,0.333333343267441,60,3,2,202,91,0,0,1,1,0,10
10565,2017,2012-06-25,4,1,0,53.3333320617676,6.66666650772095,0,60,3,2,202,91,0,0,1,1,0,10,1
10566,2017,2012-06-26,0,2,0,17,13,0,30,1,2,202,91,0,0,1,1,0,10,1240.638285,2229.030142,0,0,0,0,
10567,2017,2012-06-26,1,2,0,29,31,0,60,3,2,202,91,0,0,1,1,0,10,1240.638285,2229.030142,0,0,0,0,
10568,2017,2012-06-26,2,2,0,27.3333339691162,32.3333320617676,0.333333343267441,60,3,2,20
10569,2017,2012-06-26,3,2,0,34.7999992370605,23.2000007629395,2,60,3,2,202,91,0,0,1,1,0,10,1
10570,2017,2012-06-26,4,2,0,56.6666679382324,3.33333325386047,0,60,3,2,202,91,0,0,1,1,0,10,1
10571,2017,2012-06-27,4,3,0,56.3333320617676,3.66666674613953,0,60,3,2,202,91,0,0,1,1,0,10,1
10572,2022,2012-06-23,1,6,1,22.3333339691162,33.3333320617676,0.333333343267441,56,3,2,20
10573,2022,2012-06-23,2,6,1,12.6666669845581,46.3333320617676,1,60,3,2,202,84,1,1,1,1,0,10,1
10574,2022,2012-06-23,3,6,1,16.6666660308838,43.3333320617676,0,60,3,2,202,84,1,1,1,1,0,10,1
10575,2022,2012-06-23,4,6,1,32,28,0,60,3,2,202,84,1,1,1,1,0,10,1105.805476,2202.503131,2.36393
10576,2022,2012-06-24,0,0,1,2,8,0,10,1,2,202,84,1,1,1,1,0,10,1105.805476,2202.503131,2.3639307
10577,2022,2012-06-24,1,0,1,18.6666660308838,41.3333320617676,0,60,3,2,202,84,1,1,1,1,0,10,1
10578,2022,2012-06-24,2,0,1,10.3333330154419,48.6666679382324,1,60,3,2,202,84,1,1,1,1,0,10,1
10579,2022,2012-06-24,3,0,1,4.33333349227905,53.6666679382324,2,60,3,2,202,84,1,1,1,1,0,10,1
10580,2022,2012-06-24,4,0,1,24,36,0,60,3,2,202,84,1,1,1,1,0,10,1105.805476,2202.503131,2.36393
10581,2022,2012-06-25,0,1,0,1,9.5,0,10.5,2,2,202,84,1,1,1,1,0,10,1105.805476,2202.503131,2.3639
10582,2022,2012-06-25,1,1,0,31.2000007629395,28.7999992370605,0,60,3,2,202,84,1,1,1,1,0,10,1
10583,2022,2012-06-25,2,1,0,28.3333339691162,31.6666660308838,0,60,3,2,202,84,1,1,1,1,0,10,1
10584,2022,2012-06-25,3,1,0,34.6666679382324,25.3333339691162,0,60,3,2,202,84,1,1,1,1,0,10,1
10585,2022,2012-06-25,4,1,0,25.5,34.5,0,60,3,2,202,84,1,1,1,1,0,10,1105.805476,2202.503131,2.36
10586,2022,2012-06-26,0,2,0,33.5,17.5,0,51,2,2,202,84,1,1,1,1,0,10,1105.805476,2202.503131,2.36
10587,2022,2012-06-26,1,2,0,30.5,29,0.5,60,3,2,202,84,1,1,1,1,0,10,1105.805476,2202.503131,2.36
10588,2022,2012-06-26,2,2,0,23,36.7999992370605,0.200000002980232,60,3,2,202,84,1,1,1,1,0,10
10589,2022,2012-06-26,3,2,0,35,25,0,60,3,2,202,84,1,1,1,1,0,10,1105.805476,2202.503131,2.36393
10590,2022,2012-06-26,4,2,0,32,28,0,60,3,2,202,84,1,1,1,1,0,10,1105.805476,2202.503131,2.36393
10591,2022,2012-06-27,0,3,0,42,18,0,60,1,2,202,84,1,1,1,1,0,10,1105.805476,2202.503131,2.36393
10592,2022,2012-06-27,1,3,0,18.3333339691162,41.6666679382324,0,60,3,2,202,84,1,1,1,1,0,10,1
10593,2022,2012-06-27,2,3,0,30,30,0,60,3,2,202,84,1,1,1,1,0,10,1105.805476,2202.503131,2.36393
10594,2022,2012-06-27,3,3,0,10,49.5,0.5,60,3,2,202,84,1,1,1,1,0,10,1105.805476,2202.503131,2.36
10595,2022,2012-06-27,4,3,0,20.5,25.5,0.5,46.5,3,2,202,84,1,1,1,1,0,10,1105.805476,2202.503131,
10596,2022,2012-06-28,0,4,0,4,38,0,42,1,2,202,84,1,1,1,1,0,10,1105.805476,2202.503131,2.363930
10597,2022,2012-06-28,1,4,0,21,39,0,60,3,2,202,84,1,1,1,1,0,10,1105.805476,2202.503131,2.36393
10598,2022,2012-06-28,2,4,0,32.3333320617676,27.6666660308838,0,60,3,2,202,84,1,1,1,1,0,10,1
10599,2022,2012-06-28,3,4,0,17,43,0,60,3,2,202,84,1,1,1,1,0,10,1105.805476,2202.503131,2.36393

10600,2022,2012-06-28,4,4,0,25.3999996185303,34.5999984741211,0,60,3,2,202,84,1,1,1,1,0,10,1
10601,2022,2012-06-29,0,5,0,0,7,0,7,1,2,202,84,1,1,1,1,0,10,1105.805476,2202.503131,2.3639307
10602,2022,2012-06-29,1,5,0,18.3333339691162,41,0.666666686534882,60,3,2,202,84,1,1,1,1,0,10
10603,2022,2012-06-29,2,5,0,27.3333339691162,32.6666679382324,0,60,3,2,202,84,1,1,1,1,0,10,1
10604,2022,2012-06-29,3,5,0,16.6666660308838,41.6666679382324,1.66666662693024,60,3,2,202
10605,2022,2012-06-29,4,5,0,30.5,29.5,0,60,2,2,202,84,1,1,1,1,0,10,1105.805476,2202.503131,2.36
10606,2029,2012-06-09,0,6,1,0,8,2,10,1,2,202,71,0,1,1,1,0,11,1112.926679,1822.091156,2.786485
10607,2029,2012-06-09,1,6,1,18.25,40.75,1,60,3,2,202,71,0,1,1,1,0,11,1112.926679,1822.091156,2
10608,2029,2012-06-09,2,6,1,16.3333339691162,42.3333320617676,1.33333337306976,60,3,2,202
10609,2029,2012-06-09,3,6,1,22.3333339691162,37,0.666666686534882,60,3,2,202,71,0,1,1,1,0,1
10610,2029,2012-06-09,4,6,1,20.6666660308838,37.3333320617676,2,60,3,2,202,71,0,1,1,1,0,11,1
10611,2029,2012-06-10,0,0,1,0,2,1,3,1,2,202,71,0,1,1,1,0,11,1112.926679,1822.091156,2.7864856
10612,2029,2012-06-10,1,0,1,13,38.6666679382324,8.33333301544189,60,3,2,202,71,0,1,1,1,0,11,
10613,2029,2012-06-10,2,0,1,13.3333330154419,43.5,3.16666674613953,60,3,2,202,71,0,1,1,1,0,1
10614,2029,2012-06-10,3,0,1,9.33333301544189,47.3333320617676,3.33333325386047,60,3,2,202
10615,2029,2012-06-10,4,0,1,20,34,6,60,3,2,202,71,0,1,1,1,0,11,1112.926679,1822.091156,2.78648
10616,2029,2012-06-11,0,1,0,5,16,2,23,1,2,202,71,0,1,1,1,0,11,1112.926679,1822.091156,2.786485
10617,2029,2012-06-11,1,1,0,13.3333330154419,45.6666679382324,1,60,3,2,202,71,0,1,1,1,0,11,1
10618,2029,2012-06-11,2,1,0,19.6666660308838,32.3333320617676,8,60,3,2,202,71,0,1,1,1,0,11,1
10619,2029,2012-06-11,3,1,0,10,29,21,60,3,2,202,71,0,1,1,1,0,11,1112.926679,1822.091156,2.7864
10620,2029,2012-06-11,4,1,0,27,32,1,60,3,2,202,71,0,1,1,1,0,11,1112.926679,1822.091156,2.78648
10621,2029,2012-06-12,0,2,0,2,47,11,60,1,2,202,71,0,1,1,1,0,11,1112.926679,1822.091156,2.78648
10622,2029,2012-06-12,1,2,0,16,29,15,60,3,2,202,71,0,1,1,1,0,11,1112.926679,1822.091156,2.7864
10623,2029,2012-06-12,2,2,0,28,30.3333339691162,1.66666662693024,60,3,2,202,71,0,1,1,1,0,11,
10624,2029,2012-06-12,3,2,0,2.33333325386047,55.6666679382324,2,60,3,2,202,71,0,1,1,1,0,11,1
10625,2029,2012-06-12,4,2,0,24,33.1666679382324,2.83333325386047,60,3,2,202,71,0,1,1,1,0,11,
10626,2029,2012-06-27,0,3,0,30,27,3,60,1,2,202,71,0,1,1,1,0,11,1112.926679,1822.091156,2.78648
10627,2029,2012-06-27,1,3,0,9.33333301544189,46,4.66666650772095,60,3,2,202,71,0,1,1,1,0,11,
10628,2029,2012-06-27,2,3,0,32.3333320617676,26.3333339691162,1.33333337306976,60,3,2,202
10629,2029,2012-06-27,3,3,0,2.5,23,34.5,60,2,2,202,71,0,1,1,1,0,11,1112.926679,1822.091156,2.78
10630,2029,2012-06-28,0,4,0,9,4,1.5,14.5,2,2,202,71,0,1,1,1,0,11,1112.926679,1822.091156,2.7864
10631,2029,2012-06-28,1,4,0,12,27,21,60,3,2,202,71,0,1,1,1,0,11,1112.926679,1822.091156,2.7864
10632,2029,2012-06-28,2,4,0,17.3333339691162,39,3.66666674613953,60,3,2,202,71,0,1,1,1,0,11,
10633,2029,2012-06-28,3,4,0,26,23.25,10.75,60,3,2,202,71,0,1,1,1,0,11,1112.926679,1822.091156,
10634,2029,2012-06-28,4,4,0,27.3999996185303,29,3.59999990463257,60,3,2,202,71,0,1,1,1,0,11,
10635,2029,2012-06-29,1,5,0,5.25,27.5,5.25,38,3,2,202,71,0,1,1,1,0,11,1112.926679,1822.091156,2
10636,2029,2012-06-29,2,5,0,14,36,10,60,3,2,202,71,0,1,1,1,0,11,1112.926679,1822.091156,2.7864
10637,2029,2012-06-29,3,5,0,4,11.3333330154419,44.6666679382324,60,3,2,202,71,0,1,1,1,0,11,1
10638,2029,2012-06-29,4,5,0,26.75,29.25,4,60,3,2,202,71,0,1,1,1,0,11,1112.926679,1822.091156,2
10639,2031,2012-06-08,0,5,0,19.6666660308838,20,1.66666662693024,41.3333320617676,3,2,202
10640,2031,2012-06-08,1,5,0,14.25,43,2.75,60,3,2,202,75,1,0,0,1,0,8,1137.045481,2013.377652,3.2
10641,2031,2012-06-08,2,5,0,36.6666679382324,22.6666660308838,0.666666686534882,60,3,2,20
10642,2031,2012-06-08,3,5,0,41.3333320617676,18.3333339691162,0.333333343267441,60,3,2,20
10643,2031,2012-06-08,4,5,0,36.3333320617676,22,1.66666662693024,60,3,2,202,75,1,0,0,1,0,8,1
10644,2031,2012-06-09,0,6,1,18.5,27,1,46.5,2,2,202,75,1,0,0,1,0,8,1137.045481,2013.377652,3.253
10645,2031,2012-06-09,1,6,1,16.3333339691162,42,1.66666662693024,60,3,2,202,75,1,0,0,1,0,8,1
10646,2031,2012-06-09,2,6,1,31.6000003814697,27.2000007629395,1.20000004768372,60,3,2,202
10647,2031,2012-06-09,3,6,1,35.6666679382324,24.3333339691162,0,60,3,2,202,75,1,0,0,1,0,8,11
10648,2031,2012-06-09,4,6,1,27.3333339691162,32.3333320617676,0.333333343267441,60,3,2,20
10649,2031,2012-06-10,0,0,1,26,28,2,56,2,2,202,75,1,0,0,1,0,8,1137.045481,2013.377652,3.253484

10650,2031,2012-06-10,1,0,1,18.3333339691162,40,1.66666662693024,60,3,2,202,75,1,0,0,1,0,8,1
10651,2031,2012-06-10,2,0,1,26,33,1,60,3,2,202,75,1,0,0,1,0,8,1137.045481,2013.377652,3.253484
10652,2031,2012-06-10,3,0,1,35.3333320617676,23.8333339691162,0.833333313465118,60,3,2,2
10653,2031,2012-06-10,4,0,1,31.7999992370605,27,1.20000004768372,60,3,2,202,75,1,0,0,1,0,8,1
10654,2031,2012-06-11,4,1,0,45,13,2,60,1,2,202,75,1,0,0,1,0,8,1137.045481,2013.377652,3.253484
10655,2031,2012-06-13,0,3,0,7.33333349227905,47.3333320617676,1,55.6666679382324,3,2,202,
10656,2031,2012-06-13,1,3,0,24,31.6000003814697,4.40000009536743,60,3,2,202,75,1,0,0,1,0,8,1
10657,2031,2012-06-13,2,3,0,32.3333320617676,25.3333339691162,2.33333325386047,60,3,2,20
10658,2031,2012-06-13,3,3,0,42,18,0,60,3,2,202,75,1,0,0,1,0,8,1137.045481,2013.377652,3.253484
10659,2031,2012-06-13,4,3,0,36,23,1,60,3,2,202,75,1,0,0,1,0,8,1137.045481,2013.377652,3.253484
10660,2031,2012-06-14,0,4,0,11.5,19.5,0.5,31.5,2,2,202,75,1,0,0,1,0,8,1137.045481,2013.377652,3
10661,2031,2012-06-14,1,4,0,23.25,35.75,1,60,3,2,202,75,1,0,0,1,0,8,1137.045481,2013.377652,3.2
10662,2031,2012-06-14,2,4,0,32,26.5,1.5,60,3,2,202,75,1,0,0,1,0,8,1137.045481,2013.377652,3.25
10663,2031,2012-06-14,3,4,0,42.3333320617676,17.3333339691162,0.333333343267441,60,3,2,2
10664,2031,2012-06-14,4,4,0,30.3333339691162,29,0.666666686534882,60,3,2,202,75,1,0,0,1,0,8,
10665,2031,2012-06-15,0,5,0,6,30.5,1.5,38,2,2,202,75,1,0,0,1,0,8,1137.045481,2013.377652,3.253
10666,2031,2012-06-15,1,5,0,33,26.5,0.5,60,2,2,202,75,1,0,0,1,0,8,1137.045481,2013.377652,3.25
10667,2031,2012-06-15,2,5,0,26,31,3,60,2,2,202,75,1,0,0,1,0,8,1137.045481,2013.377652,3.253484
10668,2031,2012-06-15,3,5,0,24,34,2,60,3,2,202,75,1,0,0,1,0,8,1137.045481,2013.377652,3.253484
10669,2031,2012-06-15,4,5,0,38,17.3333339691162,4.66666650772095,60,3,2,202,75,1,0,0,1,0,8,1
10670,2032,2012-02-18,0,6,1,22,35,3,60,1,2,202,78,0,0,0,1,0,8,1098.882961,2012.805128,3.87862
10671,2032,2012-02-18,1,6,1,6.66666650772095,51,2.33333325386047,60,3,2,202,78,0,0,0,1,0,8,1
10672,2032,2012-02-18,2,6,1,21.6666660308838,37.3333320617676,1,60,3,2,202,78,0,0,0,1,0,8,10
10673,2032,2012-02-18,3,6,1,13,46.3333320617676,0.666666686534882,60,3,2,202,78,0,0,0,1,0,8,
10674,2032,2012-02-18,4,6,1,33.6666679382324,23.6666660308838,2.66666674613953,60,3,2,20
10675,2032,2012-02-19,0,7,1,4.66666650772095,19.6666660308838,4.66666650772095,29,3,2,20
10676,2032,2012-02-19,1,7,1,12.3333330154419,42.3333320617676,5.33333349227905,60,3,2,20
10677,2032,2012-02-19,2,7,1,23,36,1,60,3,2,202,78,0,0,0,1,0,8,1098.882961,2012.805128,3.87862
10678,2032,2012-02-19,3,7,1,22.6666660308838,32.6666679382324,4.66666650772095,60,3,2,20
10679,2032,2012-02-19,4,7,1,36,23,1,60,3,2,202,78,0,0,0,1,0,8,1098.882961,2012.805128,3.87862
10680,2032,2012-02-20,0,1,0,8.33333301544189,22,4,34.3333320617676,3,2,202,78,0,0,0,1,0,8,10
10681,2032,2012-02-20,1,1,0,16,41.3333320617676,2.66666674613953,60,3,2,202,78,0,0,0,1,0,8,1
10682,2032,2012-02-20,2,1,0,38.6666679382324,18,3.33333325386047,60,3,2,202,78,0,0,0,1,0,8,1
10683,2032,2012-02-20,3,1,0,34.3333320617676,23,2.66666674613953,60,3,2,202,78,0,0,0,1,0,8,1
10684,2032,2012-02-20,4,1,0,49.3333320617676,10.6666669845581,0,60,3,2,202,78,0,0,0,1,0,8,10
10685,2032,2012-02-21,0,2,0,12.3333330154419,33.3333320617676,1,46.6666679382324,3,2,202,
10686,2032,2012-02-21,1,2,0,18,35.6666679382324,6.33333349227905,60,3,2,202,78,0,0,0,1,0,8,1
10687,2032,2012-02-21,2,2,0,28.3333339691162,27.6666660308838,4,60,3,2,202,78,0,0,0,1,0,8,10
10688,2032,2012-02-21,3,2,0,17,42.6666679382324,0.333333343267441,60,3,2,202,78,0,0,0,1,0,8,
10689,2032,2012-02-21,4,2,0,42,17,1,60,3,2,202,78,0,0,0,1,0,8,1098.882961,2012.805128,3.87862
10690,2032,2012-02-22,0,3,0,19.6666660308838,21.6666660308838,2,43.3333320617676,3,2,202,
10691,2032,2012-02-22,1,3,0,11,45.6666679382324,3.33333325386047,60,3,2,202,78,0,0,0,1,0,8,1
10692,2032,2012-02-22,2,3,0,24.3333339691162,32.3333320617676,3.33333325386047,60,3,2,20
10693,2032,2012-02-22,3,3,0,12.6666669845581,46.6666679382324,0.666666686534882,60,3,2,2
10694,2032,2012-02-22,4,3,0,0.333333343267441,0.666666686534882,0.666666686534882,1.666
10695,2032,2012-02-23,0,4,0,14.3333330154419,29.3333339691162,4,47.6666679382324,3,2,202,
10696,2032,2012-02-23,1,4,0,21.6666660308838,37.3333320617676,1,60,3,2,202,78,0,0,0,1,0,8,10
10697,2032,2012-02-23,2,4,0,27.3333339691162,28.6666660308838,4,60,3,2,202,78,0,0,0,1,0,8,10
10698,2032,2012-02-23,3,4,0,13.6666669845581,39,7.33333349227905,60,3,2,202,78,0,0,0,1,0,8,1
10699,2032,2012-02-23,4,4,0,33.6666679382324,25,1.33333337306976,60,3,2,202,78,0,0,0,1,0,8,1

10700,2034,2012-06-09,0,6,1,34,6,1,41,1,2,202,82,0,0,0,1,0,9,1171.963246,1137.826309,3.7592851
10701,2034,2012-06-09,1,6,1,26.5,33.3333320617676,0.16666667163372,60,3,2,202,82,0,0,0,1,0,9
10702,2034,2012-06-09,2,6,1,49.3333320617676,10.6666669845581,0,60,3,2,202,82,0,0,0,1,0,9,11
10703,2034,2012-06-09,3,6,1,24,35.6666679382324,0.333333343267441,60,3,2,202,82,0,0,0,1,0,9,
10704,2034,2012-06-09,4,6,1,42,17.6666660308838,0.333333343267441,60,3,2,202,82,0,0,0,1,0,9,
10705,2034,2012-06-10,2,0,1,29.3333339691162,30.6666660308838,0,60,3,2,202,82,0,0,0,1,0,9,11
10706,2034,2012-06-10,3,0,1,21.3333339691162,38.3333320617676,0.333333343267441,60,3,2,20
10707,2034,2012-06-10,4,0,1,22.3333339691162,22,0.666666686534882,45,3,2,202,82,0,0,0,1,0,9,
10708,2034,2012-06-11,0,1,0,24,16.3333339691162,1,41.3333320617676,3,2,202,82,0,0,0,1,0,9,11
10709,2034,2012-06-11,1,1,0,34.25,25.75,0,60,3,2,202,82,0,0,0,1,0,9,1171.963246,1137.826309,3.7
10710,2034,2012-06-11,2,1,0,35,25,0,60,3,2,202,82,0,0,0,1,0,9,1171.963246,1137.826309,3.759285
10711,2034,2012-06-11,3,1,0,21.3333339691162,38.3333320617676,0.333333343267441,60,3,2,20
10712,2034,2012-06-11,4,1,0,35.3333320617676,24.3333339691162,0.333333343267441,60,3,2,20
10713,2034,2012-06-12,1,2,0,27,27.2000007629395,0.600000023841858,54.7999992370605,3,2,20
10714,2034,2012-06-12,2,2,0,39,19.5,1.5,60,3,2,202,82,0,0,0,1,0,9,1171.963246,1137.826309,3.759
10715,2034,2012-06-12,3,2,0,25.6666660308838,31.3333339691162,3,60,3,2,202,82,0,0,0,1,0,9,11
10716,2034,2012-06-12,4,2,0,38,22,0,60,3,2,202,82,0,0,0,1,0,9,1171.963246,1137.826309,3.759285
10717,2034,2012-06-13,0,3,0,24,7,1,32,1,2,202,82,0,0,0,1,0,9,1171.963246,1137.826309,3.7592851
10718,2034,2012-06-13,1,3,0,33,27,0,60,3,2,202,82,0,0,0,1,0,9,1171.963246,1137.826309,3.759285
10719,2034,2012-06-13,2,3,0,31.3333339691162,28,0.666666686534882,60,3,2,202,82,0,0,0,1,0,9,
10720,2034,2012-06-13,3,3,0,32,26.1666660308838,1.83333337306976,60,3,2,202,82,0,0,0,1,0,9,1
10721,2034,2012-06-13,4,3,0,30,21.75,0.5,52.25,3,2,202,82,0,0,0,1,0,9,1171.963246,1137.826309,3
10722,2034,2012-06-14,0,4,0,28,13,1,42,2,2,202,82,0,0,0,1,0,9,1171.963246,1137.826309,3.759285
10723,2034,2012-06-14,1,4,0,30.3333339691162,29.6666660308838,0,60,3,2,202,82,0,0,0,1,0,9,11
10724,2034,2012-06-14,2,4,0,46,14,0,60,3,2,202,82,0,0,0,1,0,9,1171.963246,1137.826309,3.759285
10725,2034,2012-06-14,3,4,0,48,12,0,60,3,2,202,82,0,0,0,1,0,9,1171.963246,1137.826309,3.759285
10726,2034,2012-06-14,4,4,0,37.7999992370605,22.2000007629395,0,60,3,2,202,82,0,0,0,1,0,9,11
10727,2051,2012-06-10,0,0,1,48,11,1,60,1,2,204,70,1,0,0,1,0,10,917.3631994,985.5490508,0.80693
10728,2051,2012-06-10,1,0,1,23.6666660308838,35.3333320617676,1,60,3,2,204,70,1,0,0,1,0,10,9
10729,2051,2012-06-10,2,0,1,24.6666660308838,33.6666679382324,1.66666662693024,60,3,2,204
10730,2051,2012-06-11,0,1,0,14,22,1,37,1,2,204,70,1,0,0,1,0,10,917.3631994,985.5490508,0.80693
10731,2051,2012-06-11,1,1,0,5.66666650772095,52.3333320617676,2,60,3,2,204,70,1,0,0,1,0,10,9
10732,2051,2012-06-11,2,1,0,22.6666660308838,36.3333320617676,1,60,3,2,204,70,1,0,0,1,0,10,9
10733,2051,2012-06-11,3,1,0,18.6666660308838,40,1.33333337306976,60,3,2,204,70,1,0,0,1,0,10,
10734,2051,2012-06-11,4,1,0,12.3333330154419,47.3333320617676,0.333333343267441,60,3,2,20
10735,2051,2012-06-12,0,2,0,16,6,2,24,2,2,204,70,1,0,0,1,0,10,917.3631994,985.5490508,0.806932
10736,2051,2012-06-12,1,2,0,13.6666669845581,45.3333320617676,1,60,3,2,204,70,1,0,0,1,0,10,9
10737,2051,2012-06-12,2,2,0,26.3333339691162,31.6666660308838,2,60,3,2,204,70,1,0,0,1,0,10,9
10738,2051,2012-06-12,3,2,0,30.6666660308838,27.6666660308838,1.66666662693024,60,3,2,204
10739,2051,2012-06-12,4,2,0,18.5,40.6666679382324,0.833333313465118,60,3,2,204,70,1,0,0,1,0,
10740,2051,2012-06-13,0,3,0,5,9,1,15,1,2,204,70,1,0,0,1,0,10,917.3631994,985.5490508,0.8069329
10741,2051,2012-06-13,1,3,0,10.8333330154419,48.3333320617676,0.833333313465118,60,3,2,20
10742,2051,2012-06-13,2,3,0,30.25,28.75,1,60,3,2,204,70,1,0,0,1,0,10,917.3631994,985.5490508,0
10743,2051,2012-06-13,3,3,0,17.6666660308838,39,3.33333325386047,60,3,2,204,70,1,0,0,1,0,10,
10744,2051,2012-06-13,4,3,0,24,34,2,60,3,2,204,70,1,0,0,1,0,10,917.3631994,985.5490508,0.80693
10745,2051,2012-06-14,0,4,0,1,5,0,6,1,2,204,70,1,0,0,1,0,10,917.3631994,985.5490508,0.80693295
10746,2051,2012-06-14,1,4,0,12,47.6666679382324,0.333333343267441,60,3,2,204,70,1,0,0,1,0,10
10747,2051,2012-06-14,2,4,0,38,21.3999996185303,0.600000023841858,60,3,2,204,70,1,0,0,1,0,10
10748,2051,2012-06-14,3,4,0,19,40.2000007629395,0.800000011920929,60,3,2,204,70,1,0,0,1,0,10
10749,2051,2012-06-14,4,4,0,31,29,0,60,3,2,204,70,1,0,0,1,0,10,917.3631994,985.5490508,0.80693

10750,2051,2012-06-15,0,5,0,8,25,2,35,1,2,204,70,1,0,0,1,0,10,917.3631994,985.5490508,0.80693
10751,2051,2012-06-15,1,5,0,13.3333330154419,38.6666679382324,8,60,3,2,204,70,1,0,0,1,0,10,9
10752,2051,2012-06-15,2,5,0,27.3333339691162,32,0.666666686534882,60,3,2,204,70,1,0,0,1,0,1
10753,2051,2012-06-15,3,5,0,14.5,44.5,1,60,3,2,204,70,1,0,0,1,0,10,917.3631994,985.5490508,0.8
10754,2051,2012-06-15,4,5,0,21.1666660308838,37.6666679382324,1.16666662693024,60,3,2,20
10755,2052,2012-06-07,1,4,0,15.8000001907349,35,0.600000023841858,51.4000015258789,3,2,2
10756,2052,2012-06-07,2,4,0,35.2000007629395,22.7999992370605,2,60,3,2,204,81,0,0,1,1,0,8,96
10757,2052,2012-06-07,3,4,0,41,19,0,60,3,2,204,81,0,0,1,1,0,8,965.2049896,1143.499645,0.89699
10758,2052,2012-06-07,4,4,0,49.6666679382324,10.3333330154419,0,60,3,2,204,81,0,0,1,1,0,8,96
10759,2052,2012-06-08,1,5,0,7,35,4.66666650772095,46.6666679382324,3,2,204,81,0,0,1,1,0,8,96
10760,2052,2012-06-08,2,5,0,30.75,29.25,0,60,3,2,204,81,0,0,1,1,0,8,965.2049896,1143.499645,0.
10761,2052,2012-06-08,3,5,0,22.6666660308838,33.6666679382324,3.66666674613953,60,3,2,20
10762,2052,2012-06-08,4,5,0,45.75,14.25,0,60,3,2,204,81,0,0,1,1,0,8,965.2049896,1143.499645,0.
10763,2052,2012-06-09,1,6,1,11.3999996185303,32.2000007629395,5.80000019073486,49.40000
10764,2052,2012-06-09,2,6,1,32.5,25.5,2,60,3,2,204,81,0,0,1,1,0,8,965.2049896,1143.499645,0.89
10765,2052,2012-06-09,3,6,1,24.6666660308838,29.3333339691162,6,60,3,2,204,81,0,0,1,1,0,8,96
10766,2052,2012-06-09,4,6,1,28.6000003814697,31,0.400000005960464,60,3,2,204,81,0,0,1,1,0,8,
10767,2052,2012-06-10,1,0,1,26.5,3.5,0.5,30.5,2,2,204,81,0,0,1,1,0,8,965.2049896,1143.499645,0.
10768,2052,2012-06-10,2,0,1,36,22.3999996185303,1.60000002384186,60,3,2,204,81,0,0,1,1,0,8,9
10769,2052,2012-06-10,3,0,1,25,33.3333320617676,1.66666662693024,60,3,2,204,81,0,0,1,1,0,8,9
10770,2052,2012-06-10,4,0,1,35.75,24.25,0,60,3,2,204,81,0,0,1,1,0,8,965.2049896,1143.499645,0.
10771,2052,2012-06-11,1,1,0,22.6666660308838,19.3333339691162,0,42,3,2,204,81,0,0,1,1,0,8,96
10772,2052,2012-06-11,2,1,0,35.6666679382324,21,3.33333325386047,60,3,2,204,81,0,0,1,1,0,8,9
10773,2052,2012-06-11,3,1,0,12,39.3333320617676,8.66666698455811,60,3,2,204,81,0,0,1,1,0,8,9
10774,2052,2012-06-11,4,1,0,28.6000003814697,25.6000003814697,5.80000019073486,60,3,2,20
10775,2053,2012-06-08,0,5,0,20.5,20,1,41.5,2,2,204,65,1,1,0,1,0,9,965.2049896,1143.499645,0.89
10776,2053,2012-06-08,1,5,0,4.66666650772095,53,2.33333325386047,60,3,2,204,65,1,1,0,1,0,9,9
10777,2053,2012-06-08,2,5,0,27.6666660308838,30.3333339691162,2,60,3,2,204,65,1,1,0,1,0,9,96
10778,2053,2012-06-08,3,5,0,9.33333301544189,49,1.66666662693024,60,3,2,204,65,1,1,0,1,0,9,9
10779,2053,2012-06-08,4,5,0,28,30.3333339691162,1.66666662693024,60,3,2,204,65,1,1,0,1,0,9,9
10780,2053,2012-06-09,0,6,1,21,26.5,4,51.5,2,2,204,65,1,1,0,1,0,9,965.2049896,1143.499645,0.89
10781,2053,2012-06-09,1,6,1,3.33333325386047,54.6666679382324,2,60,3,2,204,65,1,1,0,1,0,9,96
10782,2053,2012-06-09,2,6,1,26.6666660308838,32.3333320617676,1,60,3,2,204,65,1,1,0,1,0,9,96
10783,2053,2012-06-09,3,6,1,20.3333339691162,39.6666679382324,0,60,3,2,204,65,1,1,0,1,0,9,96
10784,2053,2012-06-09,4,6,1,3,51,6,60,1,2,204,65,1,1,0,1,0,9,965.2049896,1143.499645,0.892294
10785,2053,2012-06-10,0,0,1,14,28,3.5,45.5,2,2,204,65,1,1,0,1,0,9,965.2049896,1143.499645,0.89
10786,2053,2012-06-10,1,0,1,11.3333330154419,43.6666679382324,5,60,3,2,204,65,1,1,0,1,0,9,96
10787,2053,2012-06-10,2,0,1,26.3333339691162,32.3333320617676,1.33333337306976,60,3,2,20
10788,2053,2012-06-10,3,0,1,23.5,36,0.5,60,2,2,204,65,1,1,0,1,0,9,965.2049896,1143.499645,0.89
10789,2053,2012-06-10,4,0,1,29,30.5,0.5,60,2,2,204,65,1,1,0,1,0,9,965.2049896,1143.499645,0.89
10790,2053,2012-06-11,0,1,0,6,43.3333320617676,2.66666674613953,52,3,2,204,65,1,1,0,1,0,9,96
10791,2053,2012-06-11,1,1,0,1.60000002384186,55.5999984741211,2.79999995231628,60,3,2,20
10792,2053,2012-06-11,2,1,0,11,48,1,60,3,2,204,65,1,1,0,1,0,9,965.2049896,1143.499645,0.89229
10793,2053,2012-06-11,3,1,0,16,43.6666679382324,0.333333343267441,60,3,2,204,65,1,1,0,1,0,9,
10794,2053,2012-06-11,4,1,0,25.3333339691162,32.6666679382324,2,60,3,2,204,65,1,1,0,1,0,9,96
10795,2053,2012-06-12,0,2,0,16.6666660308838,27.6666660308838,0.333333343267441,44.6666
10796,2053,2012-06-12,1,2,0,5,54,1,60,3,2,204,65,1,1,0,1,0,9,965.2049896,1143.499645,0.892294
10797,2053,2012-06-12,2,2,0,18.3333339691162,40.8333320617676,0.833333313465118,60,3,2,2
10798,2053,2012-06-12,3,2,0,14.8000001907349,44,1.20000004768372,60,3,2,204,65,1,1,0,1,0,9,9
10799,2053,2012-06-12,4,2,0,10.5,44,2,56.5,2,2,204,65,1,1,0,1,0,9,965.2049896,1143.499645,0.89

10800,2053,2012-06-13,0,3,0,9.5,31,2,42.5,2,2,204,65,1,1,0,1,0,9,965.2049896,1143.499645,0.8922
10801,2053,2012-06-13,1,3,0,12.6666669845581,45.6666679382324,1.66666662693024,60,3,2,204
10802,2053,2012-06-13,2,3,0,24.6666660308838,33.3333320617676,2,60,3,2,204,65,1,1,0,1,0,9,96
10803,2053,2012-06-13,3,3,0,25.6000003814697,34.2000007629395,0.200000002980232,60,3,2,20
10804,2053,2012-06-13,4,3,0,31,28.5,0.25,59.75,3,2,204,65,1,1,0,1,0,9,965.2049896,1143.499645,0
10805,2054,2010-11-04,0,4,0,32,7.5,0,39.5,2,2,204,82,1,1,0,1,0,8,915.4053599,1026.576328,0.8917
10806,2054,2010-11-04,1,4,0,44.3333320617676,15.6666669845581,0,60,3,2,204,82,1,1,0,1,0,8,91
10807,2054,2010-11-04,2,4,0,39.6666679382324,13,0.333333343267441,53,3,2,204,82,1,1,0,1,0,8,
10808,2054,2010-11-04,3,4,0,52,8,0,60,2,2,204,82,1,1,0,1,0,8,915.4053599,1026.576328,0.8917286
10809,2054,2010-11-04,4,4,0,47.1666679382324,10.8333330154419,0,58,3,2,204,82,1,1,0,1,0,8,91
10810,2054,2010-11-05,0,5,0,39.75,10.25,0,50,3,2,204,82,1,1,0,1,0,8,915.4053599,1026.576328,0.8
10811,2054,2010-11-05,1,5,0,35.6666679382324,24.3333339691162,0,60,3,2,204,82,1,1,0,1,0,8,91
10812,2054,2010-11-05,2,5,0,45.3333320617676,14.6666669845581,0,60,3,2,204,82,1,1,0,1,0,8,91
10813,2054,2010-11-05,3,5,0,33,27,0,60,3,2,204,82,1,1,0,1,0,8,915.4053599,1026.576328,0.891728
10814,2054,2010-11-05,4,5,0,47,13,0,60,3,2,204,82,1,1,0,1,0,8,915.4053599,1026.576328,0.891728
10815,2054,2010-11-06,0,6,1,24.5,15.5,0,40,2,2,204,82,1,1,0,1,0,8,915.4053599,1026.576328,0.891
10816,2054,2010-11-06,1,6,1,38.5999984741211,21.3999996185303,0,60,3,2,204,82,1,1,0,1,0,8,91
10817,2054,2010-11-06,2,6,1,19.3333339691162,10,0,29.3333339691162,3,2,204,82,1,1,0,1,0,8,91
10818,2054,2010-11-06,3,6,1,40,20,0,60,3,2,204,82,1,1,0,1,0,8,915.4053599,1026.576328,0.891728
10819,2054,2010-11-06,4,6,1,43.3333320617676,16.6666660308838,0,60,3,2,204,82,1,1,0,1,0,8,91
10820,2054,2010-11-07,0,0,1,33.5,6.5,0,40,2,2,204,82,1,1,0,1,0,8,915.4053599,1026.576328,0.8917
10821,2054,2010-11-07,1,0,1,32.75,27.25,0,60,3,2,204,82,1,1,0,1,0,8,915.4053599,1026.576328,0.8
10822,2054,2010-11-07,2,0,1,43.5,16.5,0,60,3,2,204,82,1,1,0,1,0,8,915.4053599,1026.576328,0.891
10823,2054,2010-11-07,3,0,1,53.3333320617676,6.33333349227905,0.333333343267441,60,3,2,20
10824,2054,2010-11-07,4,0,1,51,9,0,60,3,2,204,82,1,1,0,1,0,8,915.4053599,1026.576328,0.8917286
10825,2054,2010-11-08,2,1,0,51.5,8.5,0,60,2,2,204,82,1,1,0,1,0,8,915.4053599,1026.576328,0.8917
10826,2054,2010-11-08,3,1,0,43.3333320617676,16.6666660308838,0,60,3,2,204,82,1,1,0,1,0,8,91
10827,2054,2010-11-08,4,1,0,42,18,0,60,3,2,204,82,1,1,0,1,0,8,915.4053599,1026.576328,0.891728
10828,2054,2012-06-12,0,2,0,24.5,14,0,38.5,2,2,204,82,1,1,0,1,0,8,915.4053599,1026.576328,0.891
10829,2054,2012-06-12,1,2,0,39.6666679382324,20.3333339691162,0,60,3,2,204,82,1,1,0,1,0,8,91
10830,2054,2012-06-12,2,2,0,47,13,0,60,3,2,204,82,1,1,0,1,0,8,915.4053599,1026.576328,0.891728
10831,2054,2012-06-12,3,2,0,39.6666679382324,20.3333339691162,0,60,3,2,204,82,1,1,0,1,0,8,91
10832,2054,2012-06-12,4,2,0,45,15,0,60,1,2,204,82,1,1,0,1,0,8,915.4053599,1026.576328,0.891728
10833,2054,2012-06-13,0,3,0,34.5,5.5,0,40,2,2,204,82,1,1,0,1,0,8,915.4053599,1026.576328,0.8917
10834,2054,2012-06-13,1,3,0,29.3333339691162,30.6666660308838,0,60,3,2,204,82,1,1,0,1,0,8,91
10835,2054,2012-06-13,2,3,0,40,20,0,60,3,2,204,82,1,1,0,1,0,8,915.4053599,1026.576328,0.891728
10836,2054,2012-06-13,3,3,0,11,49,0,60,1,2,204,82,1,1,0,1,0,8,915.4053599,1026.576328,0.891728
10837,2054,2012-06-13,4,3,0,42,18,0,60,2,2,204,82,1,1,0,1,0,8,915.4053599,1026.576328,0.891728
10838,2058,2010-10-22,0,5,0,36,23,1,60,1,2,204,78,0,0,0,1,0,10,970.0171289,1143.499645,0.88467
10839,2058,2010-10-22,1,5,0,19,32.75,8.25,60,3,2,204,78,0,0,0,1,0,10,970.0171289,1143.499645,0
10840,2058,2010-10-22,2,5,0,30,20.7999992370605,0,50.7999992370605,3,2,204,78,0,0,0,1,0,10,9
10841,2058,2010-10-22,3,5,0,22.6666660308838,16.3333339691162,21,60,3,2,204,78,0,0,0,1,0,10,
10842,2058,2010-10-22,4,5,0,35.6666679382324,23,1.33333337306976,60,3,2,204,78,0,0,0,1,0,10,
10843,2058,2010-10-23,0,6,1,0,20,1,21,1,2,204,78,0,0,0,1,0,10,970.0171289,1143.499645,0.884675
10844,2058,2010-10-23,1,6,1,22.6666660308838,37.3333320617676,0,60,3,2,204,78,0,0,0,1,0,10,9
10845,2058,2010-10-23,2,6,1,29,29.6666660308838,1.33333337306976,60,3,2,204,78,0,0,0,1,0,10,
10846,2058,2010-10-23,3,6,1,27.5,29.6666660308838,2.83333325386047,60,3,2,204,78,0,0,0,1,0,1
10847,2058,2010-10-23,4,6,1,29.25,26.25,4.5,60,3,2,204,78,0,0,0,1,0,10,970.0171289,1143.499645
10848,2058,2010-10-24,0,0,1,3,9,0,12,1,2,204,78,0,0,0,1,0,10,970.0171289,1143.499645,0.8846750
10849,2058,2010-10-24,1,0,1,30.6666660308838,26.6666660308838,2.66666674613953,60,3,2,204

10850,2058,2010-10-24,2,0,1,35,25,0,60,3,2,204,78,0,0,0,1,0,10,970.0171289,1143.499645,0.88467
10851,2058,2010-10-24,3,0,1,17.6666660308838,41.6666679382324,0.666666686534882,60,3,2,2
10852,2058,2010-10-24,4,0,1,36.75,21.25,2,60,3,2,204,78,0,0,0,1,0,10,970.0171289,1143.499645,0
10853,2058,2010-10-25,4,1,0,51,9,0,60,1,2,204,78,0,0,0,1,0,10,970.0171289,1143.499645,0.884675
10854,2058,2010-11-08,0,1,0,10,29,0,39,1,2,204,78,0,0,0,1,0,10,970.0171289,1143.499645,0.88467
10855,2058,2010-11-08,1,1,0,22.3333339691162,37.3333320617676,0.333333343267441,60,3,2,2
10856,2058,2010-11-08,2,1,0,28,30.6666660308838,1.33333337306976,60,3,2,204,78,0,0,0,1,0,10,
10857,2058,2010-11-08,3,1,0,32,28,0,60,1,2,204,78,0,0,0,1,0,10,970.0171289,1143.499645,0.88467
10858,2058,2010-11-09,0,2,0,0,0,1,1,1,2,204,78,0,0,0,1,0,10,970.0171289,1143.499645,0.8846750
10859,2058,2010-11-09,1,2,0,24.6666660308838,35.3333320617676,0,60,3,2,204,78,0,0,0,1,0,10,9
10860,2058,2010-11-09,2,2,0,48,12,0,60,3,2,204,78,0,0,0,1,0,10,970.0171289,1143.499645,0.88467
10861,2058,2010-11-09,3,2,0,29.3999996185303,30.2000007629395,0.400000005960464,60,3,2,2
10862,2058,2010-11-09,4,2,0,22.75,36.25,1,60,3,2,204,78,0,0,0,1,0,10,970.0171289,1143.499645,0
10863,2058,2010-11-10,1,3,0,28,29.6000003814697,2.40000009536743,60,3,2,204,78,0,0,0,1,0,10,
10864,2058,2010-11-10,2,3,0,16.3333339691162,38,5.66666650772095,60,3,2,204,78,0,0,0,1,0,10,
10865,2058,2010-11-10,3,3,0,19,35,6,60,3,2,204,78,0,0,0,1,0,10,970.0171289,1143.499645,0.88467
10866,2058,2010-11-10,4,3,0,39.7999992370605,18,2.20000004768372,60,3,2,204,78,0,0,0,1,0,10,
10867,2060,2010-10-25,0,1,0,27.5,18,1,46.5,2,2,204,78,0,0,0,1,0,11,917.3631994,985.5490508,0.8
10868,2060,2010-10-25,1,1,0,30.75,27.75,1.5,60,3,2,204,78,0,0,0,1,0,11,917.3631994,985.5490508
10869,2060,2010-10-25,2,1,0,37.3333320617676,22.3333339691162,0.333333343267441,60,3,2,2
10870,2060,2010-10-25,3,1,0,41.6666679382324,16.6666660308838,1.66666662693024,60,3,2,20
10871,2060,2010-10-25,4,1,0,33.3333320617676,26.6666660308838,0,60,3,2,204,78,0,0,0,1,0,11,9
10872,2060,2010-10-26,0,2,0,9,24,2,35,1,2,204,78,0,0,0,1,0,11,917.3631994,985.5490508,0.80693
10873,2060,2010-10-26,1,2,0,18,40.5999984741211,1.39999997615814,60,3,2,204,78,0,0,0,1,0,11,
10874,2060,2010-10-26,2,2,0,48.8333320617676,11,0.16666667163372,60,3,2,204,78,0,0,0,1,0,11,
10875,2060,2010-10-26,3,2,0,41.6666679382324,18,0.333333343267441,60,3,2,204,78,0,0,0,1,0,1
10876,2060,2010-10-26,4,2,0,50.3333320617676,9.5,0.16666667163372,60,3,2,204,78,0,0,0,1,0,11
10877,2060,2010-10-27,0,3,0,8,8,0,16,2,2,204,78,0,0,0,1,0,11,917.3631994,985.5490508,0.806932
10878,2060,2010-10-27,1,3,0,23.3333339691162,34,2.66666674613953,60,3,2,204,78,0,0,0,1,0,11,
10879,2060,2010-10-27,2,3,0,30,28,2,60,3,2,204,78,0,0,0,1,0,11,917.3631994,985.5490508,0.8069
10880,2060,2010-10-27,3,3,0,11.6666669845581,43.3333320617676,5,60,3,2,204,78,0,0,0,1,0,11,9
10881,2060,2010-10-27,4,3,0,47.6666679382324,12.3333330154419,0,60,3,2,204,78,0,0,0,1,0,11,9
10882,2060,2010-10-28,0,4,0,1,12,1,14,1,2,204,78,0,0,0,1,0,11,917.3631994,985.5490508,0.80693
10883,2060,2010-10-28,1,4,0,24.3999996185303,34.5999984741211,1,60,3,2,204,78,0,0,0,1,0,11,9
10884,2060,2010-10-28,2,4,0,47.6666679382324,12,0.333333343267441,60,3,2,204,78,0,0,0,1,0,1
10885,2060,2010-10-28,3,4,0,32.6666679382324,25.6666660308838,1.66666662693024,60,3,2,20
10886,2060,2010-10-28,4,4,0,50.3333320617676,9.66666698455811,0,60,3,2,204,78,0,0,0,1,0,11,9
10887,2060,2010-10-29,1,5,0,18,42,0,60,1,2,204,78,0,0,0,1,0,11,917.3631994,985.5490508,0.8069
10888,2060,2010-10-29,2,5,0,42.3333320617676,17.6666660308838,0,60,3,2,204,78,0,0,0,1,0,11,9
10889,2060,2010-10-29,3,5,0,43,17,0,60,3,2,204,78,0,0,0,1,0,11,917.3631994,985.5490508,0.8069
10890,2060,2010-10-29,4,5,0,41.3333320617676,18.6666660308838,0,60,3,2,204,78,0,0,0,1,0,11,9
10891,2066,2010-10-22,0,5,0,9.5,31.5,8,49,2,2,204,76,0,0,0,1,0,12,1033.724599,618.6679038,0,0,0
10892,2066,2010-10-22,1,5,0,12.6666669845581,34.3333320617676,13,60,3,2,204,76,0,0,0,1,0,12,
10893,2066,2010-10-22,2,5,0,32.25,26,1.75,60,3,2,204,76,0,0,0,1,0,12,1033.724599,618.6679038,0
10894,2066,2010-10-22,3,5,0,29,30,1,60,3,2,204,76,0,0,0,1,0,12,1033.724599,618.6679038,0,0,0,0,
10895,2066,2010-10-22,4,5,0,29.3333339691162,28.3333339691162,2.33333325386047,60,3,2,20
10896,2066,2010-10-23,0,6,1,8,33,18,59,1,2,204,76,0,0,0,1,0,12,1033.724599,618.6679038,0,0,0,0,
10897,2066,2010-10-23,1,6,1,10.6666669845581,28.3333339691162,21,60,3,2,204,76,0,0,0,1,0,12,
10898,2066,2010-10-23,2,6,1,23,23.3333339691162,13.6666669845581,60,3,2,204,76,0,0,0,1,0,12,
10899,2066,2010-10-23,3,6,1,22.3333339691162,36.6666679382324,1,60,3,2,204,76,0,0,0,1,0,12,1

10900,2066,2010-10-23,4,6,1,46.6666679382324,13.3333330154419,0,60,3,2,204,76,0,0,0,1,0,12,1
10901,2066,2010-10-24,0,0,1,12.5,24,5,41.5,2,2,204,76,0,0,0,1,0,12,1033.724599,618.6679038,0,0,
10902,2066,2010-10-24,1,0,1,8,44.3333320617676,7.66666650772095,60,3,2,204,76,0,0,0,1,0,12,1
10903,2066,2010-10-24,2,0,1,15,39,6,60,3,2,204,76,0,0,0,1,0,12,1033.724599,618.6679038,0,0,0,0,
10904,2066,2010-10-24,3,0,1,1.66666662693024,18.3333339691162,40,60,3,2,204,76,0,0,0,1,0,12,
10905,2066,2010-10-24,4,0,1,35.3333320617676,22.6666660308838,2,60,3,2,204,76,0,0,0,1,0,12,1
10906,2066,2010-10-25,0,1,0,8,35,11,54,1,2,204,76,0,0,0,1,0,12,1033.724599,618.6679038,0,0,0,0,
10907,2066,2010-10-25,1,1,0,7.66666650772095,40.6666679382324,11.6666669845581,60,3,2,204
10908,2066,2010-10-25,2,1,0,23,28,9,60,2,2,204,76,0,0,0,1,0,12,1033.724599,618.6679038,0,0,0,0,
10909,2066,2010-10-25,3,1,0,18.3333339691162,37.6666679382324,4,60,3,2,204,76,0,0,0,1,0,12,1
10910,2066,2010-10-25,4,1,0,33.3333320617676,26.3333339691162,0.333333343267441,60,3,2,20
10911,2066,2010-10-26,0,2,0,8,34,11,53,2,2,204,76,0,0,0,1,0,12,1033.724599,618.6679038,0,0,0,0,
10912,2066,2010-10-26,1,2,0,17,40,3,60,1,2,204,76,0,0,0,1,0,12,1033.724599,618.6679038,0,0,0,0,
10913,2066,2010-10-26,2,2,0,15.3333330154419,30.3333339691162,14.3333330154419,60,3,2,204
10914,2066,2010-10-26,3,2,0,11.6666669845581,36,12.3333330154419,60,3,2,204,76,0,0,0,1,0,12,
10915,2066,2010-10-26,4,2,0,33.6666679382324,22.6666660308838,3.66666674613953,60,3,2,204
10916,2066,2010-10-27,0,3,0,53,5,0,58,1,2,204,76,0,0,0,1,0,12,1033.724599,618.6679038,0,0,0,0,0
10917,2066,2010-10-27,1,3,0,19.6666660308838,29.3333339691162,11,60,3,2,204,76,0,0,0,1,0,12,
10918,2066,2010-10-27,2,3,0,21.6666660308838,33.6666679382324,4.66666650772095,60,3,2,204
10919,2066,2010-10-27,3,3,0,8.66666698455811,40.6666679382324,10.6666669845581,60,3,2,204
10920,2066,2010-10-27,4,3,0,16.6666660308838,19.3333339691162,10,46,3,2,204,76,0,0,0,1,0,12,
10921,2066,2010-10-28,1,4,0,50,10,0,60,2,2,204,76,0,0,0,1,0,12,1033.724599,618.6679038,0,0,0,0,
10922,2066,2010-10-28,2,4,0,55,5,0,60,3,2,204,76,0,0,0,1,0,12,1033.724599,618.6679038,0,0,0,0,0
10923,2066,2010-10-28,3,4,0,53.6666679382324,6.33333349227905,0,60,3,2,204,76,0,0,0,1,0,12,1
10924,2066,2010-10-28,4,4,0,24,6,0,30,1,2,204,76,0,0,0,1,0,12,1033.724599,618.6679038,0,0,0,0,0
10925,2066,2010-10-29,0,5,0,8,50,2,60,1,2,204,76,0,0,0,1,0,12,1033.724599,618.6679038,0,0,0,0,0
10926,2066,2010-10-29,1,5,0,7.66666650772095,48.6666679382324,3.66666674613953,60,3,2,204
10927,2066,2010-10-29,2,5,0,13,40,7,60,1,2,204,76,0,0,0,1,0,12,1033.724599,618.6679038,0,0,0,0,
10928,2066,2010-10-30,0,6,1,7.33333349227905,32,12,51.3333320617676,3,2,204,76,0,0,0,1,0,12,
10929,2066,2010-10-30,1,6,1,19,30.6666660308838,10.3333330154419,60,3,2,204,76,0,0,0,1,0,12,
10930,2066,2010-10-30,2,6,1,35.5,23,1.5,60,2,2,204,76,0,0,0,1,0,12,1033.724599,618.6679038,0,0,
10931,2066,2010-10-30,3,6,1,26.6666660308838,28,5.33333349227905,60,3,2,204,76,0,0,0,1,0,12,
10932,2066,2010-10-30,4,6,1,34,21.3333339691162,4.66666650772095,60,3,2,204,76,0,0,0,1,0,12,
10933,2069,2010-10-28,0,4,0,15,43,2,60,1,2,204,72,1,0,1,1,0,11,910.8110909,1316.677662,1.2023
10934,2069,2010-10-28,1,4,0,24.3333339691162,31.6666660308838,4,60,3,2,204,72,1,0,1,1,0,11,9
10935,2069,2010-10-28,2,4,0,10,48,2,60,1,2,204,72,1,0,1,1,0,11,910.8110909,1316.677662,1.2023
10936,2069,2010-10-29,0,5,0,22.5,29.5,1,53,2,2,204,72,1,0,1,1,0,11,910.8110909,1316.677662,1.20
10937,2069,2010-10-29,1,5,0,8.33333301544189,50,1.66666662693024,60,3,2,204,72,1,0,1,1,0,11,
10938,2069,2010-10-29,2,5,0,23,36.6666679382324,0.333333343267441,60,3,2,204,72,1,0,1,1,0,1
10939,2069,2010-10-29,3,5,0,32,25.6666660308838,2.33333325386047,60,3,2,204,72,1,0,1,1,0,11,
10940,2069,2010-10-29,4,5,0,37,23,0,60,3,2,204,72,1,0,1,1,0,11,910.8110909,1316.677662,1.2023
10941,2069,2010-10-30,0,6,1,20.5,33,1.5,55,2,2,204,72,1,0,1,1,0,11,910.8110909,1316.677662,1.20
10942,2069,2010-10-30,1,6,1,21,35,4,60,3,2,204,72,1,0,1,1,0,11,910.8110909,1316.677662,1.2023
10943,2069,2010-10-30,2,6,1,25.6666660308838,32.6666679382324,1.66666662693024,60,3,2,204
10944,2069,2010-10-30,3,6,1,33,24,3,60,3,2,204,72,1,0,1,1,0,11,910.8110909,1316.677662,1.2023
10945,2069,2010-10-30,4,6,1,44,15.3333330154419,0.666666686534882,60,3,2,204,72,1,0,1,1,0,1
10946,2069,2010-11-19,0,5,0,19.5,25,1,45.5,2,2,204,72,1,0,1,1,0,11,910.8110909,1316.677662,1.20
10947,2069,2010-11-19,1,5,0,22.6666660308838,34.3333320617676,3,60,3,2,204,72,1,0,1,1,0,11,9
10948,2069,2010-11-19,2,5,0,30.25,29,0.75,60,3,2,204,72,1,0,1,1,0,11,910.8110909,1316.677662,1
10949,2069,2010-11-19,3,5,0,13.3333330154419,46.3333320617676,0.333333343267441,60,3,2,20

```
10950,2069,2010-11-19,4,5,0,34.6666679382324,22.6666660308838,2.66666674613953,60,3,2,204
10951,2069,2010-11-20,0,6,1,23.5,20.5,1.5,45.5,2,2,204,72,1,0,1,1,0,11,910.8110909,1316.677662,
10952,2069,2010-11-20,1,6,1,14.3333330154419,44.3333320617676,1.33333337306976,60,3,2,204
10953,2069,2010-11-20,2,6,1,21,35.25,3.75,60,3,2,204,72,1,0,1,1,0,11,910.8110909,1316.677662,1
10954,2069,2010-11-20,3,6,1,25.6666660308838,31.6666660308838,2.66666674613953,60,3,2,204
10955,2069,2010-11-20,4,6,1,45,14.3333330154419,0.666666686534882,60,3,2,204,72,1,0,1,1,0,11
10956,2069,2010-11-21,0,0,1,21,35,1,57,2,2,204,72,1,0,1,1,0,11,910.8110909,1316.677662,1.20236
10957,2069,2010-11-21,1,0,1,46.6666679382324,12,1.33333337306976,60,3,2,204,72,1,0,1,1,0,11,
10958,2069,2010-11-21,2,0,1,38.3333320617676,19.6666660308838,2,60,3,2,204,72,1,0,1,1,0,11,9
10959,2069,2010-11-21,3,0,1,29.1666660308838,28.6666660308838,2.16666674613953,60,3,2,204
10960,2069,2010-11-21,4,0,1,48,12,0,60,3,2,204,72,1,0,1,1,0,11,910.8110909,1316.677662,1.20236
10961,2069,2010-11-22,0,1,0,18.5,23,1,42.5,2,2,204,72,1,0,1,1,0,11,910.8110909,1316.677662,1.20
10962,2069,2010-11-22,1,1,0,11,31.3333339691162,17.6666660308838,60,3,2,204,72,1,0,1,1,0,11,
10963,2069,2010-11-22,2,1,0,26.6666660308838,23,10.3333330154419,60,3,2,204,72,1,0,1,1,0,11,
10964,2069,2010-11-22,3,1,0,34.3333320617676,18.3333339691162,7.33333349227905,60,3,2,204
10965,2069,2010-11-22,4,1,0,41.25,18.75,0,60,3,2,204,72,1,0,1,1,0,11,910.8110909,1316.677662,1
10966,2069,2010-11-23,4,2,0,42.5,17.5,0,60,2,2,204,72,1,0,1,1,0,11,910.8110909,1316.677662,1.20
10967,2070,2010-11-05,0,5,0,15,34,11,60,1,2,204,81,0,0,0,1,0,11,1026.690314,874.471072,0,0,0,0,
10968,2070,2010-11-05,1,5,0,23.6666660308838,34,2.33333325386047,60,3,2,204,81,0,0,0,1,0,11,
10969,2070,2010-11-05,2,5,0,24.6666660308838,34.6666679382324,0.666666686534882,60,3,2,20
10970,2070,2010-11-05,3,5,0,36.6666679382324,22.6666660308838,0.666666686534882,60,3,2,20
10971,2070,2010-11-05,4,5,0,58.75,1.25,0,60,3,2,204,81,0,0,0,1,0,11,1026.690314,874.471072,0,0,
10972,2070,2010-11-06,0,6,1,11.5,23,1,35.5,2,2,204,81,0,0,0,1,0,11,1026.690314,874.471072,0,0,0
10973,2070,2010-11-06,1,6,1,23.3333339691162,34,2.66666674613953,60,3,2,204,81,0,0,0,1,0,11,
10974,2070,2010-11-06,2,6,1,38,22,0,60,3,2,204,81,0,0,0,1,0,11,1026.690314,874.471072,0,0,0,0,0
10975,2070,2010-11-06,3,6,1,29,30.6666660308838,0.333333343267441,60,3,2,204,81,0,0,0,1,0,11
10976,2070,2010-11-06,4,6,1,56.6666679382324,3.33333325386047,0,60,3,2,204,81,0,0,0,1,0,11,1
10977,2070,2010-11-07,0,0,1,16,18.5,0,34.5,2,2,204,81,0,0,0,1,0,11,1026.690314,874.471072,0,0,0
10978,2070,2010-11-07,1,0,1,8,50,2,60,3,2,204,81,0,0,0,1,0,11,1026.690314,874.471072,0,0,0,0,0,0
10979,2070,2010-11-07,2,0,1,26,34,0,60,3,2,204,81,0,0,0,1,0,11,1026.690314,874.471072,0,0,0,0,0
10980,2070,2010-11-07,3,0,1,17.3333339691162,34.3333320617676,8.33333301544189,60,3,2,204
10981,2070,2010-11-07,4,0,1,47.5,7.5,5,60,2,2,204,81,0,0,0,1,0,11,1026.690314,874.471072,0,0,0,0
10982,2070,2010-11-08,4,1,0,58,2,0,60,1,2,204,81,0,0,0,1,0,11,1026.690314,874.471072,0,0,0,0,0,0
10983,2070,2010-11-23,0,2,0,10.5,17.5,4.5,32.5,2,2,204,81,0,0,0,1,0,11,1026.690314,874.471072,0
10984,2070,2010-11-23,1,2,0,31.6666660308838,27,1.33333337306976,60,3,2,204,81,0,0,0,1,0,11,
10985,2070,2010-11-23,2,2,0,33,25,2,60,3,2,204,81,0,0,0,1,0,11,1026.690314,874.471072,0,0,0,0,0
10986,2070,2010-11-23,3,2,0,47.6666679382324,12.3333330154419,0,60,3,2,204,81,0,0,0,1,0,11,1
10987,2070,2010-11-23,4,2,0,54.5,5.5,0,60,2,2,204,81,0,0,0,1,0,11,1026.690314,874.471072,0,0,0,0
10988,2070,2010-11-24,0,3,0,19,11,0.5,30.5,2,2,204,81,0,0,0,1,0,11,1026.690314,874.471072,0,0,0
10989,2070,2010-11-24,1,3,0,13,41,6,60,3,2,204,81,0,0,0,1,0,11,1026.690314,874.471072,0,0,0,0,0
10990,2070,2010-11-24,2,3,0,14,37.6666679382324,8.33333301544189,60,3,2,204,81,0,0,0,1,0,11,
10991,2070,2010-11-24,3,3,0,37,22.6666660308838,0.333333343267441,60,3,2,204,81,0,0,0,1,0,11
10992,2070,2010-11-24,4,3,0,53.25,6.5,0.25,60,3,2,204,81,0,0,0,1,0,11,1026.690314,874.471072,0,
10993,2070,2010-11-25,0,4,0,17.3333339691162,14.6666669845581,2,34,3,2,204,81,0,0,0,1,0,11,1
10994,2070,2010-11-25,1,4,0,26,29.6666660308838,4.33333349227905,60,3,2,204,81,0,0,0,1,0,11,
10995,2070,2010-11-25,2,4,0,31,25.6666660308838,3.33333325386047,60,3,2,204,81,0,0,0,1,0,11,
10996,2070,2010-11-25,3,4,0,26.6666660308838,33,0.333333343267441,60,3,2,204,81,0,0,0,1,0,11
10997,2070,2010-11-25,4,4,0,41,19,0,60,3,2,204,81,0,0,0,1,0,11,1026.690314,874.471072,0,0,0,0,0
10998,2070,2010-11-26,0,5,0,22,28,10,60,1,2,204,81,0,0,0,1,0,11,1026.690314,874.471072,0,0,0,0,
10999,2070,2010-11-26,1,5,0,27.3333339691162,23.3333339691162,9.33333301544189,60,3,2,204
```

11000,2070,2010-11-26,2,5,0,17.6666660308838,34.3333320617676,8,60,3,2,204,81,0,0,0,1,0,11,1
11001,2070,2010-11-26,3,5,0,8.33333301544189,46.6666679382324,5,60,3,2,204,81,0,0,0,1,0,11,1
11002,2070,2010-11-26,4,5,0,34.5,23.5,2,60,2,2,204,81,0,0,0,1,0,11,1026.690314,874.471072,0,0,0
11003,2072,2010-11-08,0,1,0,14,16.5,0,30.5,2,2,204,66,1,0,0,1,0,12,965.0874247,908.8277497,0,0,
11004,2072,2010-11-08,1,1,0,7,47,6,60,3,2,204,66,1,0,0,1,0,12,965.0874247,908.8277497,0,0,0,0,0
11005,2072,2010-11-08,2,1,0,9.33333301544189,47.3333320617676,3.33333325386047,60,3,2,20
11006,2072,2010-11-08,3,1,0,39,19,2,60,3,2,204,66,1,0,0,1,0,12,965.0874247,908.8277497,0,0,0,0,
11007,2072,2010-11-08,4,1,0,30.5,25,4.5,60,2,2,204,66,1,0,0,1,0,12,965.0874247,908.8277497,0,0,
11008,2072,2010-11-09,0,2,0,11,23,1,35,1,2,204,66,1,0,0,1,0,12,965.0874247,908.8277497,0,0,0,0,
11009,2072,2010-11-09,1,2,0,0.666666686534882,57.3333320617676,2,60,3,2,204,66,1,0,0,1,0,12,
11010,2072,2010-11-09,2,2,0,10.3333330154419,47,2.66666674613953,60,3,2,204,66,1,0,0,1,0,12,
11011,2072,2010-11-09,3,2,0,7.66666650772095,50.3333320617676,2,60,3,2,204,66,1,0,0,1,0,12,9
11012,2072,2010-11-09,4,2,0,36.5,23.25,0.25,60,3,2,204,66,1,0,0,1,0,12,965.0874247,908.8277497
11013,2072,2010-11-10,0,3,0,15,19,3,37,1,2,204,66,1,0,0,1,0,12,965.0874247,908.8277497,0,0,0,0,
11014,2072,2010-11-10,1,3,0,6.66666650772095,51,2.33333325386047,60,3,2,204,66,1,0,0,1,0,12,
11015,2072,2010-11-10,2,3,0,16.3333339691162,43,0.666666686534882,60,3,2,204,66,1,0,0,1,0,1
11016,2072,2010-11-10,3,3,0,13,43,4,60,3,2,204,66,1,0,0,1,0,12,965.0874247,908.8277497,0,0,0,0,
11017,2072,2010-11-10,4,3,0,14.5,38.5,7,60,2,2,204,66,1,0,0,1,0,12,965.0874247,908.8277497,0,0,
11018,2072,2010-11-11,0,4,0,12,35,2,49,1,2,204,66,1,0,0,1,0,12,965.0874247,908.8277497,0,0,0,0,
11019,2072,2010-11-11,1,4,0,11,40,9,60,3,2,204,66,1,0,0,1,0,12,965.0874247,908.8277497,0,0,0,0,
11020,2072,2010-11-11,2,4,0,14,44.6666679382324,1.33333337306976,60,3,2,204,66,1,0,0,1,0,12,
11021,2072,2010-11-11,3,4,0,6.33333349227905,46,7.66666650772095,60,3,2,204,66,1,0,0,1,0,12,
11022,2072,2010-11-11,4,4,0,25.75,30.75,3.5,60,3,2,204,66,1,0,0,1,0,12,965.0874247,908.8277497
11023,2072,2010-12-01,0,3,0,4,54,2,60,1,2,204,66,1,0,0,1,0,12,965.0874247,908.8277497,0,0,0,0,0
11024,2072,2010-12-01,1,3,0,27,32,1,60,3,2,204,66,1,0,0,1,0,12,965.0874247,908.8277497,0,0,0,0,
11025,2072,2010-12-01,2,3,0,42.3333320617676,17,0.666666686534882,60,3,2,204,66,1,0,0,1,0,1
11026,2072,2010-12-01,3,3,0,47.3333320617676,12.6666669845581,0,60,3,2,204,66,1,0,0,1,0,12,9
11027,2072,2010-12-01,4,3,0,22.3333339691162,34.3333320617676,3.33333325386047,60,3,2,20
11028,2072,2010-12-02,0,4,0,10.3333330154419,21.6666660308838,10,42,3,2,204,66,1,0,0,1,0,12,
11029,2072,2010-12-02,1,4,0,5.66666650772095,49.6666679382324,4.66666650772095,60,3,2,20
11030,2072,2010-12-02,2,4,0,18,41,1,60,3,2,204,66,1,0,0,1,0,12,965.0874247,908.8277497,0,0,0,0,
11031,2072,2010-12-02,3,4,0,35.6666679382324,23.3333339691162,1,60,3,2,204,66,1,0,0,1,0,12,9
11032,2072,2010-12-02,4,4,0,37.3333320617676,17.3333339691162,5.33333349227905,60,3,2,20
11033,2072,2010-12-03,1,5,0,12,39,9,60,3,2,204,66,1,0,0,1,0,12,965.0874247,908.8277497,0,0,0,0,
11034,2072,2010-12-03,2,5,0,12.3333330154419,47.3333320617676,0.333333343267441,60,3,2,2
11035,2072,2010-12-03,3,5,0,4.33333349227905,55,0.666666686534882,60,3,2,204,66,1,0,0,1,0,1
11036,2072,2010-12-03,4,5,0,30,30,0,60,3,2,204,66,1,0,0,1,0,12,965.0874247,908.8277497,0,0,0,0,
11037,2082,2010-11-27,0,6,1,6.66666650772095,36.6666679382324,0.666666686534882,44,3,2,2
11038,2082,2010-11-27,1,6,1,10.6666669845581,49.3333320617676,0,60,3,2,204,75,1,0,1,1,0,11,1
11039,2082,2010-11-27,2,6,1,29,31,0,60,3,2,204,75,1,0,1,1,0,11,1000.21565,895.8999621,0,0,0,0,0
11040,2082,2010-11-27,3,6,1,37,22.6666660308838,0.333333343267441,60,3,2,204,75,1,0,1,1,0,1
11041,2082,2010-11-27,4,6,1,37.6666679382324,21.6666660308838,0.666666686534882,60,3,2,2
11042,2082,2010-11-28,0,0,1,1,44,2,47,1,2,204,75,1,0,1,1,0,11,1000.21565,895.8999621,0,0,0,0,0,
11043,2082,2010-11-28,1,0,1,11.8000001907349,47,1.20000004768372,60,3,2,204,75,1,0,1,1,0,11,
11044,2082,2010-11-28,2,0,1,35.6666679382324,21.6666660308838,2.66666674613953,60,3,2,20
11045,2082,2010-11-28,3,0,1,29.6666660308838,29.3333339691162,1,60,3,2,204,75,1,0,1,1,0,11,1
11046,2082,2010-11-28,4,0,1,36.6666679382324,22,1.33333337306976,60,3,2,204,75,1,0,1,1,0,11,
11047,2082,2010-11-29,0,1,0,21,32,1,54,1,2,204,75,1,0,1,1,0,11,1000.21565,895.8999621,0,0,0,0,0
11048,2082,2010-11-29,1,1,0,23,36.75,0.25,60,3,2,204,75,1,0,1,1,0,11,1000.21565,895.8999621,0,0
11049,2082,2010-11-29,2,1,0,28.3333339691162,31.3333339691162,0.333333343267441,60,3,2,2

11050,2082,2010-11-29,3,1,0,12,48,0,60,3,2,204,75,1,0,1,1,0,11,1000.21565,895.8999621,0,0,0,0,0
11051,2082,2010-11-29,4,1,0,41.3333320617676,18,0.666666686534882,60,3,2,204,75,1,0,1,1,0,1
11052,2082,2010-11-30,0,2,0,7,32,1,40,1,2,204,75,1,0,1,1,0,11,1000.21565,895.8999621,0,0,0,0,0,
11053,2082,2010-11-30,1,2,0,10.5,49.5,0,60,2,2,204,75,1,0,1,1,0,11,1000.21565,895.8999621,0,0,0
11054,2082,2010-11-30,2,2,0,27.3333339691162,32.6666679382324,0,60,3,2,204,75,1,0,1,1,0,11,1
11055,2082,2010-11-30,3,2,0,28.3333339691162,31.3333339691162,0.333333343267441,60,3,2,2
11056,2082,2010-11-30,4,2,0,48.3333320617676,11.6666669845581,0,60,3,2,204,75,1,0,1,1,0,11,1
11057,2082,2010-12-01,0,3,0,22.5,23.5,1,47,2,2,204,75,1,0,1,1,0,11,1000.21565,895.8999621,0,0,0
11058,2082,2010-12-01,1,3,0,38,22,0,60,3,2,204,75,1,0,1,1,0,11,1000.21565,895.8999621,0,0,0,0,0
11059,2082,2010-12-01,2,3,0,22.6666660308838,37.3333320617676,0,60,3,2,204,75,1,0,1,1,0,11,1
11060,2082,2010-12-01,3,3,0,37,23,0,60,3,2,204,75,1,0,1,1,0,11,1000.21565,895.8999621,0,0,0,0,0
11061,2082,2010-12-01,4,3,0,39.3333320617676,20.3333339691162,0.333333343267441,60,3,2,2
11062,2082,2010-12-02,0,4,0,13,5,2,20,1,2,204,75,1,0,1,1,0,11,1000.21565,895.8999621,0,0,0,0,0,
11063,2082,2010-12-02,1,4,0,30.3333339691162,29.6666660308838,0,60,3,2,204,75,1,0,1,1,0,11,1
11064,2082,2010-12-02,2,4,0,42.3333320617676,17.3333339691162,0.333333343267441,60,3,2,2
11065,2082,2010-12-02,3,4,0,46.3333320617676,13.6666669845581,0,60,3,2,204,75,1,0,1,1,0,11,1
11066,2082,2010-12-02,4,4,0,50,10,0,60,3,2,204,75,1,0,1,1,0,11,1000.21565,895.8999621,0,0,0,0,0
11067,2082,2010-12-03,0,5,0,5,51,2,58,1,2,204,75,1,0,1,1,0,11,1000.21565,895.8999621,0,0,0,0,0,
11068,2082,2010-12-03,1,5,0,16.6666660308838,43,0.333333343267441,60,3,2,204,75,1,0,1,1,0,1
11069,2082,2010-12-03,2,5,0,26,34,0,60,3,2,204,75,1,0,1,1,0,11,1000.21565,895.8999621,0,0,0,0,0
11070,2082,2010-12-03,3,5,0,46.3333320617676,13.6666669845581,0,60,3,2,204,75,1,0,1,1,0,11,1
11071,2082,2010-12-03,4,5,0,42.3333320617676,17.6666660308838,0,60,3,2,204,75,1,0,1,1,0,11,1
11072,2082,2010-12-04,0,6,1,37,2.5,0,39.5,2,2,204,75,1,0,1,1,0,11,1000.21565,895.8999621,0,0,0,
11073,2082,2010-12-04,1,6,1,50.6666679382324,9.33333301544189,0,60,3,2,204,75,1,0,1,1,0,11,1
11074,2082,2010-12-04,2,6,1,55.3333320617676,4.66666650772095,0,60,3,2,204,75,1,0,1,1,0,11,1
11075,2082,2010-12-04,3,6,1,38.3333320617676,21.1666660308838,0.5,60,3,2,204,75,1,0,1,1,0,11
11076,2082,2010-12-04,4,6,1,39,20.3333339691162,0.666666686534882,60,3,2,204,75,1,0,1,1,0,1
11077,2082,2010-12-05,4,0,1,25,4,0,29,1,2,204,75,1,0,1,1,0,11,1000.21565,895.8999621,0,0,0,0,0,
11078,2083,2010-11-19,0,5,0,32,22,1,55,2,2,204,71,1,1,1,1,0,6,969.0113626,923.8817825,0,0,0,0,0
11079,2083,2010-11-19,1,5,0,26.3333339691162,33.3333320617676,0.333333343267441,60,3,2,2
11080,2083,2010-11-19,2,5,0,30.7999992370605,28.6000003814697,0.600000023841858,60,3,2,2
11081,2083,2010-11-19,3,5,0,26.3333339691162,33.3333320617676,0.333333343267441,60,3,2,2
11082,2083,2010-11-19,4,5,0,50.3333320617676,9.66666698455811,0,60,3,2,204,71,1,1,1,1,0,6,96
11083,2083,2010-11-20,0,6,1,10,30.3333339691162,2,42.3333320617676,3,2,204,71,1,1,1,1,0,6,96
11084,2083,2010-11-20,1,6,1,9.33333301544189,50.6666679382324,0,60,3,2,204,71,1,1,1,1,0,6,96
11085,2083,2010-11-20,2,6,1,23.75,36,0.25,60,3,2,204,71,1,1,1,1,0,6,969.0113626,923.8817825,0,
11086,2083,2010-11-20,3,6,1,20.25,39,0.75,60,3,2,204,71,1,1,1,1,0,6,969.0113626,923.8817825,0,
11087,2083,2010-11-20,4,6,1,34.6666679382324,25.3333339691162,0,60,3,2,204,71,1,1,1,1,0,6,96
11088,2083,2010-11-21,3,0,1,13.5,46,0.5,60,2,2,204,71,1,1,1,1,0,6,969.0113626,923.8817825,0,0,0
11089,2083,2010-11-21,4,0,1,45.6666679382324,14.3333330154419,0,60,3,2,204,71,1,1,1,1,0,6,96
11090,2083,2010-11-30,0,2,0,29,31,0,60,1,2,204,71,1,1,1,1,0,6,969.0113626,923.8817825,0,0,0,0,0
11091,2083,2010-11-30,1,2,0,13.6666669845581,37,1.5,52.1666679382324,3,2,204,71,1,1,1,1,0,6,
11092,2083,2010-11-30,2,2,0,23,33,4,60,3,2,204,71,1,1,1,1,0,6,969.0113626,923.8817825,0,0,0,0,0
11093,2083,2010-11-30,3,2,0,3,49.3333320617676,7.66666650772095,60,3,2,204,71,1,1,1,1,0,6,96
11094,2083,2010-11-30,4,2,0,43,16.3333339691162,0.666666686534882,60,3,2,204,71,1,1,1,1,0,6,
11095,2083,2010-12-05,0,0,1,22,38,0,60,1,2,204,71,1,1,1,1,0,6,969.0113626,923.8817825,0,0,0,0,0
11096,2083,2010-12-05,1,0,1,28.3333339691162,31.3333339691162,0.333333343267441,60,3,2,2
11097,2083,2010-12-05,2,0,1,35.3333320617676,24.3333339691162,0.333333343267441,60,3,2,2
11098,2083,2010-12-05,3,0,1,31.6666660308838,27.3333339691162,1,60,3,2,204,71,1,1,1,1,0,6,96
11099,2083,2010-12-05,4,0,1,39,20.3333339691162,0.666666686534882,60,3,2,204,71,1,1,1,1,0,6,

11100,2083,2010-12-06,0,1,0,33.5,19.5,0.5,53.5,2,2,204,71,1,1,1,1,0,6,969.0113626,923.8817825,0
11101,2083,2010-12-06,1,1,0,29.3333339691162,30.6666660308838,0,60,3,2,204,71,1,1,1,1,0,6,96
11102,2083,2010-12-06,2,1,0,19.3333339691162,39.3333320617676,1.33333337306976,60,3,2,204
11103,2083,2010-12-06,3,1,0,17.6666660308838,41.6666679382324,0.666666686534882,60,3,2,20
11104,2083,2010-12-06,4,1,0,23.3333339691162,36.3333320617676,0.333333343267441,60,3,2,20
11105,2083,2010-12-07,0,2,0,10,24.3333339691162,1.66666662693024,36,3,2,204,71,1,1,1,1,0,6,9
11106,2083,2010-12-07,1,2,0,21,38.6666679382324,0.333333343267441,60,3,2,204,71,1,1,1,1,0,6,
11107,2083,2010-12-07,2,2,0,44,16,0,60,3,2,204,71,1,1,1,1,0,6,969.0113626,923.8817825,0,0,0,0,0
11108,2083,2010-12-07,3,2,0,35.6666679382324,24,0.333333343267441,60,3,2,204,71,1,1,1,1,0,6,
11109,2083,2010-12-07,4,2,0,36,23.6666660308838,0.333333343267441,60,3,2,204,71,1,1,1,1,0,6,
11110,2084,2010-11-19,0,5,0,14.5,29,0.5,44,2,2,204,74,0,0,1,1,0,8,983.6669379,873.9286972,0,0,0
11111,2084,2010-11-19,1,5,0,29,30.3333339691162,0.666666686534882,60,3,2,204,74,0,0,1,1,0,8,
11112,2084,2010-11-19,2,5,0,33.3333320617676,24,2.66666674613953,60,3,2,204,74,0,0,1,1,0,8,9
11113,2084,2010-11-19,3,5,0,26.6000003814697,32.4000015258789,1,60,3,2,204,74,0,0,1,1,0,8,98
11114,2084,2010-11-19,4,5,0,48.3333320617676,11.6666669845581,0,60,3,2,204,74,0,0,1,1,0,8,98
11115,2084,2010-11-20,0,6,1,36.5,3,0,39.5,2,2,204,74,0,0,1,1,0,8,983.6669379,873.9286972,0,0,0,0
11116,2084,2010-11-20,1,6,1,43.6666679382324,16.3333339691162,0,60,3,2,204,74,0,0,1,1,0,8,98
11117,2084,2010-11-20,2,6,1,54.6666679382324,5.33333349227905,0,60,3,2,204,74,0,0,1,1,0,8,98
11118,2084,2010-11-20,3,6,1,51.5,8.5,0,60,3,2,204,74,0,0,1,1,0,8,983.6669379,873.9286972,0,0,0,0
11119,2084,2010-11-20,4,6,1,44,8.5,0,52.5,3,2,204,74,0,0,1,1,0,8,983.6669379,873.9286972,0,0,0,0
11120,2084,2010-11-21,0,0,1,41,17,2,60,1,2,204,74,0,0,1,1,0,8,983.6669379,873.9286972,0,0,0,0,0
11121,2084,2010-11-21,1,0,1,25.3333339691162,34.3333320617676,0.333333343267441,60,3,2,20
11122,2084,2010-11-21,2,0,1,36,24,0,60,3,2,204,74,0,0,1,1,0,8,983.6669379,873.9286972,0,0,0,0,0
11123,2084,2010-11-21,3,0,1,21.3333339691162,38,0.666666686534882,60,3,2,204,74,0,0,1,1,0,8,
11124,2084,2010-11-21,4,0,1,51,9,0,60,1,2,204,74,0,0,1,1,0,8,983.6669379,873.9286972,0,0,0,0,0,0
11125,2084,2010-11-22,0,1,0,20.3333339691162,16,1.33333337306976,37.6666679382324,3,2,204
11126,2084,2010-11-22,1,1,0,16,43,1,60,3,2,204,74,0,0,1,1,0,8,983.6669379,873.9286972,0,0,0,0,0
11127,2084,2010-11-22,2,1,0,34,25,1,60,3,2,204,74,0,0,1,1,0,8,983.6669379,873.9286972,0,0,0,0,0
11128,2084,2010-11-22,3,1,0,20.6666660308838,38.3333320617676,1,60,3,2,204,74,0,0,1,1,0,8,98
11129,2084,2010-11-22,4,1,0,53,7,0,60,3,2,204,74,0,0,1,1,0,8,983.6669379,873.9286972,0,0,0,0,0,0
11130,2084,2010-11-23,0,2,0,31.3333339691162,20,1,52.3333320617676,3,2,204,74,0,0,1,1,0,8,98
11131,2084,2010-11-23,1,2,0,19.75,37.5,2.75,60,3,2,204,74,0,0,1,1,0,8,983.6669379,873.9286972,0
11132,2084,2010-11-23,2,2,0,45,14,1,60,3,2,204,74,0,0,1,1,0,8,983.6669379,873.9286972,0,0,0,0,0
11133,2084,2010-11-23,3,2,0,33.3333320617676,26.3333339691162,0.333333343267441,60,3,2,20
11134,2084,2010-11-23,4,2,0,49.3333320617676,10.6666669845581,0,60,3,2,204,74,0,0,1,1,0,8,98
11135,2084,2010-11-24,0,3,0,11.5,33,2,46.5,2,2,204,74,0,0,1,1,0,8,983.6669379,873.9286972,0,0,0
11136,2084,2010-11-24,1,3,0,20.3999996185303,34.5999984741211,5,60,3,2,204,74,0,0,1,1,0,8,98
11137,2084,2010-11-24,2,3,0,24.6666660308838,33.3333320617676,2,60,3,2,204,74,0,0,1,1,0,8,98
11138,2084,2010-11-24,3,3,0,31.6666660308838,27,1.33333337306976,60,3,2,204,74,0,0,1,1,0,8,9
11139,2084,2010-11-24,4,3,0,52,8,0,60,3,2,204,74,0,0,1,1,0,8,983.6669379,873.9286972,0,0,0,0,0,0
11140,2084,2010-11-25,0,4,0,24,24,0.5,48.5,2,2,204,74,0,0,1,1,0,8,983.6669379,873.9286972,0,0,0
11141,2084,2010-11-25,1,4,0,40.3333320617676,18.6666660308838,1,60,3,2,204,74,0,0,1,1,0,8,98
11142,2084,2010-11-25,2,4,0,30.3333339691162,28.3333339691162,1.33333337306976,60,3,2,204
11143,2084,2010-11-25,3,4,0,25,34.3333320617676,0.666666686534882,60,3,2,204,74,0,0,1,1,0,8,
11144,2084,2010-11-25,4,4,0,46.3333320617676,13.6666669845581,0,60,3,2,204,74,0,0,1,1,0,8,98
11145,2086,2010-11-22,0,1,0,10,46,1.66666662693024,57.6666679382324,3,2,204,80,1,1,0,1,0,10,
11146,2086,2010-11-22,1,1,0,26.6000003814697,30.2000007629395,3.20000004768372,60,3,2,204
11147,2086,2010-11-22,2,1,0,26.3333339691162,32,1.66666662693024,60,3,2,204,80,1,1,0,1,0,10,
11148,2086,2010-11-22,3,1,0,33,25.3333339691162,1.66666662693024,60,3,2,204,80,1,1,0,1,0,10,
11149,2086,2010-11-22,4,1,0,49.3333320617676,10.6666669845581,0,60,3,2,204,80,1,1,0,1,0,10,9

11150,2086,2010-11-23,0,2,0,17,24.3333339691162,0,41.3333320617676,3,2,204,80,1,1,0,1,0,10,9
11151,2086,2010-11-23,1,2,0,24.25,35.75,0,60,3,2,204,80,1,1,0,1,0,10,966.1098346,892.9819487,0
11152,2086,2010-11-23,2,2,0,30.3333339691162,29,0.666666686534882,60,3,2,204,80,1,1,0,1,0,1
11153,2086,2010-11-23,3,2,0,37.3333320617676,22.6666660308838,0,60,3,2,204,80,1,1,0,1,0,10,9
11154,2086,2010-11-23,4,2,0,34,3,0.5,37.5,2,2,204,80,1,1,0,1,0,10,966.1098346,892.9819487,0,0,0
11155,2086,2010-11-24,0,3,0,20.5,27.5,0.5,48.5,2,2,204,80,1,1,0,1,0,10,966.1098346,892.9819487,
11156,2086,2010-11-24,1,3,0,18.6666660308838,41.3333320617676,0,60,3,2,204,80,1,1,0,1,0,10,9
11157,2086,2010-11-24,2,3,0,33.1666679382324,25.8333339691162,1,60,3,2,204,80,1,1,0,1,0,10,9
11158,2086,2010-11-24,3,3,0,31.1666660308838,27.5,0.16666667163372,58.8333320617676,3,2,2
11159,2086,2010-11-25,0,4,0,15,33.5,3.5,52,2,2,204,80,1,1,0,1,0,10,966.1098346,892.9819487,0,0,
11160,2086,2010-11-25,1,4,0,31.3333339691162,28,0.666666686534882,60,3,2,204,80,1,1,0,1,0,1
11161,2086,2010-11-25,2,4,0,33.6666679382324,26,0.333333343267441,60,3,2,204,80,1,1,0,1,0,1
11162,2086,2010-11-25,3,4,0,26.3333339691162,31,2.66666674613953,60,3,2,204,80,1,1,0,1,0,10,
11163,2086,2010-11-25,4,4,0,39.5,8,0,47.5,2,2,204,80,1,1,0,1,0,10,966.1098346,892.9819487,0,0,0
11164,2086,2010-12-01,0,3,0,19,22.5,1.5,43,2,2,204,80,1,1,0,1,0,10,966.1098346,892.9819487,0,0,
11165,2086,2010-12-01,1,3,0,11,47.6666679382324,1.33333337306976,60,3,2,204,80,1,1,0,1,0,10,
11166,2086,2010-12-01,2,3,0,37.3333320617676,20.6666660308838,2,60,3,2,204,80,1,1,0,1,0,10,9
11167,2086,2010-12-01,3,3,0,23,34.6666679382324,1.33333337306976,59,3,2,204,80,1,1,0,1,0,10,
11168,2086,2010-12-01,4,3,0,40.5,9,0.5,50,2,2,204,80,1,1,0,1,0,10,966.1098346,892.9819487,0,0,0
11169,2088,2012-02-19,0,7,1,38,20,2,60,1,2,204,73,0,0,1,1,0,12,981.7107917,932.9623739,0,0,0,0,
11170,2088,2012-02-19,1,7,1,42,18,0,60,3,2,204,73,0,0,1,1,0,12,981.7107917,932.9623739,0,0,0,0,
11171,2088,2012-02-19,2,7,1,47.3333320617676,10.6666669845581,2,60,3,2,204,73,0,0,1,1,0,12,9
11172,2088,2012-02-19,3,7,1,52.3333320617676,7.33333349227905,0.333333343267441,60,3,2,2
11173,2088,2012-02-19,4,7,1,46,8.33333301544189,0.666666686534882,55,3,2,204,73,0,0,1,1,0,1
11174,2088,2012-02-20,0,1,0,14.6666669845581,9.33333301544189,1.33333337306976,25.33333
11175,2088,2012-02-20,1,1,0,28.3333339691162,29.6666660308838,2,60,3,2,204,73,0,0,1,1,0,12,9
11176,2088,2012-02-20,2,1,0,18.6666660308838,40.3333320617676,1,60,3,2,204,73,0,0,1,1,0,12,9
11177,2088,2012-02-20,3,1,0,19.3333339691162,39.6666679382324,1,60,3,2,204,73,0,0,1,1,0,12,9
11178,2088,2012-02-20,4,1,0,16,9.66666698455811,1.33333337306976,27,3,2,204,73,0,0,1,1,0,12,
11179,2088,2012-02-21,0,2,0,29.6666660308838,8.33333301544189,3,41,3,2,204,73,0,0,1,1,0,12,9
11180,2088,2012-02-21,1,2,0,32.6666679382324,24.6666660308838,2.66666674613953,60,3,2,20
11181,2088,2012-02-21,2,2,0,8,46,6,60,3,2,204,73,0,0,1,1,0,12,981.7107917,932.9623739,0,0,0,0,0
11182,2088,2012-02-21,3,2,0,16.3333339691162,43,0.666666686534882,60,3,2,204,73,0,0,1,1,0,1
11183,2088,2012-02-21,4,2,0,29,11.3333330154419,1,41.3333320617676,3,2,204,73,0,0,1,1,0,12,9
11184,2088,2012-02-22,0,3,0,14.3333330154419,4,1.33333337306976,19.6666660308838,3,2,204,
11185,2088,2012-02-22,1,3,0,35.3333320617676,21.3333339691162,3.33333325386047,60,3,2,20
11186,2088,2012-02-22,2,3,0,17,42.3333320617676,0.666666686534882,60,3,2,204,73,0,0,1,1,0,1
11187,2088,2012-02-22,3,3,0,23,36.3333320617676,0.666666686534882,60,3,2,204,73,0,0,1,1,0,1
11188,2088,2012-02-22,4,3,0,47,11.6666669845581,0.666666686534882,59.3333320617676,3,2,2
11189,2088,2012-02-23,0,4,0,28,5.33333349227905,1,34.3333320617676,3,2,204,73,0,0,1,1,0,12,9
11190,2088,2012-02-23,1,4,0,31.6666660308838,27.3333339691162,1,60,3,2,204,73,0,0,1,1,0,12,9
11191,2088,2012-02-23,2,4,0,19.6666660308838,39.3333320617676,1,60,3,2,204,73,0,0,1,1,0,12,9
11192,2088,2012-02-23,3,4,0,28.3333339691162,31.6666660308838,0,60,3,2,204,73,0,0,1,1,0,12,9
11193,2088,2012-02-23,4,4,0,37,14,1.33333337306976,52.3333320617676,3,2,204,73,0,0,1,1,0,12,
11194,2088,2012-02-24,0,5,0,11.6666669845581,4.33333349227905,1.33333337306976,17.33333
11195,2088,2012-02-24,1,5,0,34.3333320617676,25.6666660308838,0,60,3,2,204,73,0,0,1,1,0,12,9
11196,2088,2012-02-24,2,5,0,19.6666660308838,36.6666679382324,3.66666674613953,60,3,2,20
11197,2088,2012-02-24,3,5,0,24.3333339691162,34.6666679382324,1,60,3,2,204,73,0,0,1,1,0,12,9
11198,2088,2012-02-24,4,5,0,22.3333339691162,9.66666698455811,1.66666662693024,33.66666
11199,2088,2012-02-25,0,6,1,5.33333349227905,2.33333325386047,1.66666662693024,9.333333

11200,2088,2012-02-25,1,6,1,30,28.3333339691162,1.66666662693024,60,3,2,204,73,0,0,1,1,0,12,
11201,2088,2012-02-25,2,6,1,17.3333339691162,40.6666679382324,2,60,3,2,204,73,0,0,1,1,0,12,9
11202,2088,2012-02-25,3,6,1,27,30.3333339691162,2.66666674613953,60,3,2,204,73,0,0,1,1,0,12,
11203,2088,2012-02-25,4,6,1,24,6.33333349227905,0.666666686534882,31,3,2,204,73,0,0,1,1,0,1
11204,2096,2010-11-27,0,6,1,27.3333339691162,15,0,42.3333320617676,3,2,204,75,1,1,0,1,0,11,9
11205,2096,2010-11-27,1,6,1,5.66666650772095,49.3333320617676,5,60,3,2,204,75,1,1,0,1,0,11,9
11206,2096,2010-11-27,2,6,1,15.25,44.25,0.5,60,3,2,204,75,1,1,0,1,0,11,965.5013092,1078.474113
11207,2096,2010-11-27,3,6,1,17.3333339691162,42,0.666666686534882,60,3,2,204,75,1,1,0,1,0,1
11208,2096,2010-11-27,4,6,1,21,39,0,60,3,2,204,75,1,1,0,1,0,11,965.5013092,1078.474113,0,0,0,0,
11209,2096,2010-11-28,1,0,1,33.6666679382324,19.6666660308838,0,53.3333320617676,3,2,204,
11210,2096,2010-11-28,2,0,1,27.75,31,1.25,60,3,2,204,75,1,1,0,1,0,11,965.5013092,1078.474113,0
11211,2096,2010-11-28,3,0,1,14.3333330154419,42.6666679382324,3,60,3,2,204,75,1,1,0,1,0,11,9
11212,2096,2010-11-28,4,0,1,44,15.6666669845581,0.333333343267441,60,3,2,204,75,1,1,0,1,0,1
11213,2096,2010-11-29,1,1,0,24.3333339691162,32.3333320617676,0.333333343267441,57,3,2,2
11214,2096,2010-11-29,2,1,0,38.6666679382324,21.3333339691162,0,60,3,2,204,75,1,1,0,1,0,11,9
11215,2096,2010-11-29,3,1,0,48,12,0,60,3,2,204,75,1,1,0,1,0,11,965.5013092,1078.474113,0,0,0,0,
11216,2096,2010-11-29,4,1,0,39,21,0,60,3,2,204,75,1,1,0,1,0,11,965.5013092,1078.474113,0,0,0,0,
11217,2096,2010-11-30,0,2,0,27,32.6666679382324,0.333333343267441,60,3,2,204,75,1,1,0,1,0,1
11218,2096,2010-11-30,1,2,0,22.3333339691162,37.6666679382324,0,60,3,2,204,75,1,1,0,1,0,11,9
11219,2096,2010-11-30,2,2,0,18.6666660308838,41.3333320617676,0,60,3,2,204,75,1,1,0,1,0,11,9
11220,2096,2010-11-30,3,2,0,15.5,40.5,4,60,2,2,204,75,1,1,0,1,0,11,965.5013092,1078.474113,0,0,
11221,2096,2010-11-30,4,2,0,21.3333339691162,37,1.66666662693024,60,3,2,204,75,1,1,0,1,0,11,
11222,2096,2010-12-02,0,4,0,29.5,22.5,0.5,52.5,2,2,204,75,1,1,0,1,0,11,965.5013092,1078.474113,
11223,2096,2010-12-02,1,4,0,27.25,32.75,0,60,3,2,204,75,1,1,0,1,0,11,965.5013092,1078.474113,0
11224,2096,2010-12-02,2,4,0,29.3333339691162,30.6666660308838,0,60,3,2,204,75,1,1,0,1,0,11,9
11225,2096,2010-12-02,3,4,0,28.6666660308838,30,1.33333337306976,60,3,2,204,75,1,1,0,1,0,11,
11226,2096,2010-12-02,4,4,0,46.6666679382324,13.3333330154419,0,60,3,2,204,75,1,1,0,1,0,11,9
11227,2096,2010-12-03,0,5,0,0,6,0,6,1,2,204,75,1,1,0,1,0,11,965.5013092,1078.474113,0,0,0,0,0,0,
11228,2096,2010-12-03,1,5,0,20.2000007629395,39.7999992370605,0,60,3,2,204,75,1,1,0,1,0,11,9
11229,2096,2010-12-03,2,5,0,28,31.5,0.5,60,3,2,204,75,1,1,0,1,0,11,965.5013092,1078.474113,0,0,
11230,2096,2010-12-03,3,5,0,17.3333339691162,42,0.666666686534882,60,3,2,204,75,1,1,0,1,0,1
11231,2096,2010-12-03,4,5,0,43.3333320617676,16,0.666666686534882,60,3,2,204,75,1,1,0,1,0,1
11232,2096,2011-03-02,0,3,0,54,4,0,58,1,2,204,75,1,1,0,1,0,11,965.5013092,1078.474113,0,0,0,0,0
11233,2096,2011-03-02,1,3,0,53,7,0,60,3,2,204,75,1,1,0,1,0,11,965.5013092,1078.474113,0,0,0,0,0
11234,2096,2011-03-02,2,3,0,56.3333320617676,3.66666674613953,0,60,3,2,204,75,1,1,0,1,0,11,9
11235,2096,2011-03-02,3,3,0,35.4000015258789,24.3999996185303,0.200000002980232,60,3,2,2
11236,2096,2011-03-02,4,3,0,37,23,0,60,3,2,204,75,1,1,0,1,0,11,965.5013092,1078.474113,0,0,0,0,
11237,2096,2011-03-03,3,4,0,58,2,0,60,1,2,204,75,1,1,0,1,0,11,965.5013092,1078.474113,0,0,0,0,0
11238,2096,2011-03-03,4,4,0,26,4,0,30,1,2,204,75,1,1,0,1,0,11,965.5013092,1078.474113,0,0,0,0,0
11239,2099,2011-03-03,0,4,0,7,22,2.5,31.5,2,2,204,69,0,1,1,1,0,12,971.2336739,1070.418446,0,0,0
11240,2099,2011-03-03,1,4,0,24,30.3333339691162,5.66666650772095,60,3,2,204,69,0,1,1,1,0,12,
11241,2099,2011-03-03,2,4,0,23.6666660308838,34.3333320617676,2,60,3,2,204,69,0,1,1,1,0,12,9
11242,2099,2011-03-03,3,4,0,24,33.3333320617676,2.66666674613953,60,3,2,204,69,0,1,1,1,0,12,
11243,2099,2011-03-03,4,4,0,33.6666679382324,25.6666660308838,0.666666686534882,60,3,2,2
11244,2099,2011-03-04,0,5,0,11,22,1.33333337306976,34.3333320617676,3,2,204,69,0,1,1,1,0,12,
11245,2099,2011-03-04,1,5,0,16,40.125,3.875,60,3,2,204,69,0,1,1,1,0,12,971.2336739,1070.41844
11246,2099,2011-03-04,2,5,0,21,34,5,60,3,2,204,69,0,1,1,1,0,12,971.2336739,1070.418446,0,0,0,0,
11247,2099,2011-03-04,3,5,0,12.1666669845581,41.3333320617676,6.5,60,3,2,204,69,0,1,1,1,0,12
11248,2099,2011-03-04,4,5,0,24.8333339691162,32,3.16666674613953,60,3,2,204,69,0,1,1,1,0,12,
11249,2099,2011-03-05,0,6,1,3.5,25.5,2.5,31.5,2,2,204,69,0,1,1,1,0,12,971.2336739,1070.418446,0

11250,2099,2011-03-05,1,6,1,14.25,38.25,7.5,60,3,2,204,69,0,1,1,1,0,12,971.2336739,1070.418446
11251,2099,2011-03-05,2,6,1,13.25,37.5,9.25,60,3,2,204,69,0,1,1,1,0,12,971.2336739,1070.418446
11252,2099,2011-03-05,3,6,1,5.19999980926514,38,16.7999992370605,60,3,2,204,69,0,1,1,1,0,12,
11253,2099,2011-03-05,4,6,1,25.6666660308838,29.8333339691162,4.5,60,3,2,204,69,0,1,1,1,0,12
11254,2099,2011-03-06,1,0,1,21,29.6666660308838,5.33333349227905,56,3,2,204,69,0,1,1,1,0,12,
11255,2099,2011-03-06,2,0,1,10.8000001907349,40,9.19999980926514,60,3,2,204,69,0,1,1,1,0,12,
11256,2099,2011-03-06,3,0,1,13,43,4,60,3,2,204,69,0,1,1,1,0,12,971.2336739,1070.418446,0,0,0,0,
11257,2099,2011-03-06,4,0,1,25,30.6666660308838,4.33333349227905,60,3,2,204,69,0,1,1,1,0,12,
11258,2099,2011-03-08,0,2,0,10,11,1,22,1,2,204,69,0,1,1,1,0,12,971.2336739,1070.418446,0,0,0,0,
11259,2099,2011-03-08,1,2,0,11,43.6666679382324,5.33333349227905,60,3,2,204,69,0,1,1,1,0,12,
11260,2099,2011-03-08,2,2,0,19.3333339691162,37.6666679382324,3,60,3,2,204,69,0,1,1,1,0,12,9
11261,2099,2011-03-08,3,2,0,22.25,31.75,6,60,3,2,204,69,0,1,1,1,0,12,971.2336739,1070.418446,0
11262,2099,2011-03-08,4,2,0,11.6666669845581,33,15.3333330154419,60,3,2,204,69,0,1,1,1,0,12,
11263,2101,2011-03-05,0,6,1,43,17,0,60,1,2,204,79,0,0,1,1,0,9,1003.1582,1149.004123,0,0,0,0,0,0,
11264,2101,2011-03-05,1,6,1,24.6666660308838,21,14.3333330154419,60,3,2,204,79,0,0,1,1,0,9,1
11265,2101,2011-03-05,2,6,1,30.6666660308838,28.3333339691162,1,60,3,2,204,79,0,0,1,1,0,9,10
11266,2101,2011-03-05,3,6,1,35,23.6666660308838,1.33333337306976,60,3,2,204,79,0,0,1,1,0,9,1
11267,2101,2011-03-05,4,6,1,44.3333320617676,15.6666669845581,0,60,3,2,204,79,0,0,1,1,0,9,10
11268,2101,2011-03-06,0,0,1,28.5,17,2,47.5,2,2,204,79,0,0,1,1,0,9,1003.1582,1149.004123,0,0,0,0,
11269,2101,2011-03-06,1,0,1,14.3333330154419,44,1.66666662693024,60,3,2,204,79,0,0,1,1,0,9,1
11270,2101,2011-03-06,2,0,1,34.3333320617676,16.6666660308838,9,60,3,2,204,79,0,0,1,1,0,9,10
11271,2101,2011-03-06,3,0,1,40.6666679382324,18.6666660308838,0.666666686534882,60,3,2,2
11272,2101,2011-03-06,4,0,1,44.3333320617676,15.3333330154419,0.333333343267441,60,3,2,2
11273,2101,2011-03-07,0,1,0,25.75,16,2.5,44.25,3,2,204,79,0,0,1,1,0,9,1003.1582,1149.004123,0,0
11274,2101,2011-03-07,1,1,0,43.3333320617676,13,3.66666674613953,60,3,2,204,79,0,0,1,1,0,9,1
11275,2101,2011-03-07,2,1,0,54,6,0,60,3,2,204,79,0,0,1,1,0,9,1003.1582,1149.004123,0,0,0,0,0,0,3
11276,2101,2011-03-07,3,1,0,42.6666679382324,17.3333339691162,0,60,3,2,204,79,0,0,1,1,0,9,10
11277,2101,2011-03-07,4,1,0,53,7,0,60,3,2,204,79,0,0,1,1,0,9,1003.1582,1149.004123,0,0,0,0,0,0,3
11278,2101,2011-03-08,0,2,0,35.5,16.5,1.5,53.5,2,2,204,79,0,0,1,1,0,9,1003.1582,1149.004123,0,0,
11279,2101,2011-03-08,1,2,0,9,47.25,3.75,60,3,2,204,79,0,0,1,1,0,9,1003.1582,1149.004123,0,0,0,
11280,2101,2011-03-08,2,2,0,33,18.3333339691162,8.66666698455811,60,3,2,204,79,0,0,1,1,0,9,1
11281,2101,2011-03-08,3,2,0,34.3333320617676,25,0.666666686534882,60,3,2,204,79,0,0,1,1,0,9,
11282,2101,2011-03-08,4,2,0,40.6666679382324,18.6666660308838,0.666666686534882,60,3,2,2
11283,2101,2011-03-09,0,3,0,36.5,9.5,1.5,47.5,2,2,204,79,0,0,1,1,0,9,1003.1582,1149.004123,0,0,0
11284,2101,2011-03-09,1,3,0,28.5,25.75,5.75,60,3,2,204,79,0,0,1,1,0,9,1003.1582,1149.004123,0,0
11285,2101,2011-03-09,2,3,0,42.6666679382324,16.6666660308838,0.666666686534882,60,3,2,2
11286,2101,2011-03-09,3,3,0,41,19,0,60,3,2,204,79,0,0,1,1,0,9,1003.1582,1149.004123,0,0,0,0,0,0,
11287,2101,2011-03-09,4,3,0,38.6666679382324,21,0.333333343267441,60,3,2,204,79,0,0,1,1,0,9,
11288,2101,2011-03-10,0,4,0,32,16,2,50,2,2,204,79,0,0,1,1,0,9,1003.1582,1149.004123,0,0,0,0,0,0,
11289,2101,2011-03-10,1,4,0,24.5,28,7.5,60,3,2,204,79,0,0,1,1,0,9,1003.1582,1149.004123,0,0,0,0,
11290,2101,2011-03-10,2,4,0,17.3333339691162,38.6666679382324,4,60,3,2,204,79,0,0,1,1,0,9,10
11291,2101,2011-03-10,3,4,0,25.3333339691162,34.6666679382324,0,60,3,2,204,79,0,0,1,1,0,9,10
11292,2101,2011-03-10,4,4,0,25.6666660308838,33.6666679382324,0.666666686534882,60,3,2,2
11293,2101,2011-03-12,2,6,1,37,22.6666660308838,0.333333343267441,60,3,2,204,79,0,0,1,1,0,9,
11294,2101,2011-03-12,3,6,1,29.6666660308838,30.3333339691162,0,60,3,2,204,79,0,0,1,1,0,9,10
11295,2101,2011-03-12,4,6,1,38.6666679382324,19.6666660308838,1.66666662693024,60,3,2,20
11296,2110,2011-03-12,0,6,1,43,16,1,60,1,2,204,75,1,1,0,1,0,8,1033.793772,1181.093387,0,0,0,0,0
11297,2110,2011-03-12,1,6,1,12.6666669845581,40,7.33333349227905,60,3,2,204,75,1,1,0,1,0,8,1
11298,2110,2011-03-12,2,6,1,35.5,24.5,0,60,2,2,204,75,1,1,0,1,0,8,1033.793772,1181.093387,0,0,0
11299,2110,2011-03-13,0,0,1,31,23,4,58,1,2,204,75,1,1,0,1,0,8,1033.793772,1181.093387,0,0,0,0,0

11300,2110,2011-03-13,1,0,1,42.3333320617676,17.6666660308838,0,60,3,2,204,75,1,1,0,1,0,8,10
11301,2110,2011-03-13,2,0,1,50.75,9.25,0,60,3,2,204,75,1,1,0,1,0,8,1033.793772,1181.093387,0,0,
11302,2110,2011-03-13,3,0,1,46.8333320617676,13.1666669845581,0,60,3,2,204,75,1,1,0,1,0,8,10
11303,2110,2011-03-13,4,0,1,43,17,0,60,3,2,204,75,1,1,0,1,0,8,1033.793772,1181.093387,0,0,0,0,0
11304,2110,2011-03-14,0,1,0,3,6.5,1,10.5,2,2,204,75,1,1,0,1,0,8,1033.793772,1181.093387,0,0,0,0,
11305,2110,2011-03-14,1,1,0,38,22,0,60,3,2,204,75,1,1,0,1,0,8,1033.793772,1181.093387,0,0,0,0,0
11306,2110,2011-03-14,2,1,0,20,18,0,38,2,2,204,75,1,1,0,1,0,8,1033.793772,1181.093387,0,0,0,0,0
11307,2110,2011-03-14,3,1,0,42.3333320617676,17.6666660308838,0,60,3,2,204,75,1,1,0,1,0,8,10
11308,2110,2011-03-14,4,1,0,48.5,11.3333330154419,0.16666667163372,60,3,2,204,75,1,1,0,1,0,8
11309,2110,2011-03-15,0,2,0,1,7,0,8,1,2,204,75,1,1,0,1,0,8,1033.793772,1181.093387,0,0,0,0,0,0,4
11310,2110,2011-03-15,1,2,0,29.2000007629395,30.7999992370605,0,60,3,2,204,75,1,1,0,1,0,8,10
11311,2110,2011-03-15,2,2,0,28.2000007629395,31.7999992370605,0,60,3,2,204,75,1,1,0,1,0,8,10
11312,2110,2011-03-15,3,2,0,27.6666660308838,32.3333320617676,0,60,3,2,204,75,1,1,0,1,0,8,10
11313,2110,2011-03-15,4,2,0,43.3333320617676,16.6666660308838,0,60,3,2,204,75,1,1,0,1,0,8,10
11314,2110,2011-03-16,1,3,0,10.3333330154419,45.3333320617676,0.666666686534882,56.3333
11315,2110,2011-03-16,2,3,0,11.5,48.5,0,60,3,2,204,75,1,1,0,1,0,8,1033.793772,1181.093387,0,0,0
11316,2110,2011-03-16,3,3,0,38.75,21.25,0,60,3,2,204,75,1,1,0,1,0,8,1033.793772,1181.093387,0,
11317,2110,2011-03-16,4,3,0,46.6666679382324,13.3333330154419,0,60,3,2,204,75,1,1,0,1,0,8,10
11318,2110,2011-03-17,0,4,0,4,12,0,16,1,2,204,75,1,1,0,1,0,8,1033.793772,1181.093387,0,0,0,0,0,
11319,2110,2011-03-17,1,4,0,30,30,0,60,3,2,204,75,1,1,0,1,0,8,1033.793772,1181.093387,0,0,0,0,0
11320,2110,2011-03-17,2,4,0,33,27,0,60,3,2,204,75,1,1,0,1,0,8,1033.793772,1181.093387,0,0,0,0,0
11321,2110,2011-03-17,3,4,0,37.6666679382324,22,0.333333343267441,60,3,2,204,75,1,1,0,1,0,8,
11322,2110,2011-03-17,4,4,0,42,18,0,60,3,2,204,75,1,1,0,1,0,8,1033.793772,1181.093387,0,0,0,0,0
11323,2110,2011-03-18,3,5,0,15,45,0,60,2,2,204,75,1,1,0,1,0,8,1033.793772,1181.093387,0,0,0,0,0
11324,2110,2011-03-18,4,5,0,49.6666679382324,10.3333330154419,0,60,3,2,204,75,1,1,0,1,0,8,10
11325,2113,2010-12-01,0,3,0,0,1,0,1,1,2,204,73,0,0,0,1,0,9,1033.793772,1181.093387,0,0,0,0,0,0,4
11326,2113,2010-12-01,1,3,0,12,27.5,0,39.5,2,2,204,73,0,0,0,1,0,9,1033.793772,1181.093387,0,0,0
11327,2113,2010-12-01,2,3,0,39.6666679382324,20.3333339691162,0,60,3,2,204,73,0,0,0,1,0,9,10
11328,2113,2010-12-01,3,3,0,42,18,0,60,3,2,204,73,0,0,0,1,0,9,1033.793772,1181.093387,0,0,0,0,0
11329,2113,2010-12-01,4,3,0,49,11,0,60,3,2,204,73,0,0,0,1,0,9,1033.793772,1181.093387,0,0,0,0,0
11330,2113,2011-03-18,1,5,0,18.3333339691162,23,0.333333343267441,41.6666679382324,3,2,2
11331,2113,2011-03-18,2,5,0,40.3333320617676,19.6666660308838,0,60,3,2,204,73,0,0,0,1,0,9,10
11332,2113,2011-03-18,3,5,0,47,13,0,60,1,2,204,73,0,0,0,1,0,9,1033.793772,1181.093387,0,0,0,0,0
11333,2113,2011-03-19,1,6,1,22,15,2,39,1,2,204,73,0,0,0,1,0,9,1033.793772,1181.093387,0,0,0,0,0
11334,2113,2011-03-19,2,6,1,37,22.6666660308838,0.333333343267441,60,3,2,204,73,0,0,0,1,0,9,
11335,2113,2011-03-19,3,6,1,42.2000007629395,17.7999992370605,0,60,3,2,204,73,0,0,0,1,0,9,10
11336,2113,2011-03-19,4,6,1,36.75,23.25,0,60,3,2,204,73,0,0,0,1,0,9,1033.793772,1181.093387,0,
11337,2113,2011-03-21,1,1,0,9,33,1,43,2,2,204,73,0,0,0,1,0,9,1033.793772,1181.093387,0,0,0,0,0,
11338,2113,2011-03-21,2,1,0,44.6666679382324,15.3333330154419,0,60,3,2,204,73,0,0,0,1,0,9,10
11339,2113,2011-03-21,3,1,0,28.6666660308838,31.3333339691162,0,60,3,2,204,73,0,0,0,1,0,9,10
11340,2113,2011-03-21,4,1,0,51,9,0,60,3,2,204,73,0,0,0,1,0,9,1033.793772,1181.093387,0,0,0,0,0,
11341,2113,2011-03-22,0,2,0,4,5,1,10,1,2,204,73,0,0,0,1,0,9,1033.793772,1181.093387,0,0,0,0,0,0,
11342,2113,2011-03-22,1,2,0,31.6666660308838,27.6666660308838,0.666666686534882,60,3,2,2
11343,2113,2011-03-22,2,2,0,30.3333339691162,29.6666660308838,0,60,3,2,204,73,0,0,0,1,0,9,10
11344,2113,2011-03-22,3,2,0,22,38,0,60,3,2,204,73,0,0,0,1,0,9,1033.793772,1181.093387,0,0,0,0,0
11345,2113,2011-03-22,4,2,0,41.5,18.5,0,60,2,2,204,73,0,0,0,1,0,9,1033.793772,1181.093387,0,0,0
11346,2113,2011-03-23,1,3,0,28.5,14,0,42.5,2,2,204,73,0,0,0,1,0,9,1033.793772,1181.093387,0,0,0
11347,2113,2011-03-23,2,3,0,33.3333320617676,26.6666660308838,0,60,3,2,204,73,0,0,0,1,0,9,10
11348,2113,2011-03-23,3,3,0,29,31,0,60,3,2,204,73,0,0,0,1,0,9,1033.793772,1181.093387,0,0,0,0,0
11349,2113,2011-03-23,4,3,0,44.7999992370605,15.1999998092651,0,60,3,2,204,73,0,0,0,1,0,9,10

11350,2113,2011-03-24,1,4,0,9.66666698455811,33.3333320617676,2.33333325386047,45.333332
11351,2113,2011-03-24,2,4,0,24.6666660308838,35.3333320617676,0,60,3,2,204,73,0,0,0,1,0,9,10
11352,2113,2011-03-24,3,4,0,18.6666660308838,39.3333320617676,2,60,3,2,204,73,0,0,0,1,0,9,10
11353,2113,2011-03-24,4,4,0,43.3333320617676,16.6666660308838,0,60,3,2,204,73,0,0,0,1,0,9,10
11354,2120,2010-12-01,0,3,0,18,7,1,26,1,2,204,69,0,1,0,1,0,11,1001.784696,1133.824623,0,0,0,0,0
11355,2120,2010-12-01,1,3,0,17.3333339691162,41.3333320617676,1.33333337306976,60,3,2,204
11356,2120,2010-12-02,0,4,0,22.5,11,0,33.5,2,2,204,69,0,1,0,1,0,11,1001.784696,1133.824623,0,0,
11357,2120,2010-12-02,1,4,0,25.3333339691162,33.3333320617676,1.33333337306976,60,3,2,204
11358,2120,2010-12-02,2,4,0,26.3333339691162,33,0.666666686534882,60,3,2,204,69,0,1,0,1,0,11
11359,2120,2010-12-02,3,4,0,27.3333339691162,32.3333320617676,0.333333343267441,60,3,2,20
11360,2120,2010-12-02,4,4,0,32,27,1,60,3,2,204,69,0,1,0,1,0,11,1001.784696,1133.824623,0,0,0,0,
11361,2120,2010-12-03,0,5,0,7,12,0,19,1,2,204,69,0,1,0,1,0,11,1001.784696,1133.824623,0,0,0,0,0
11362,2120,2010-12-03,1,5,0,54.6666679382324,5.33333349227905,0,60,3,2,204,69,0,1,0,1,0,11,1
11363,2120,2010-12-03,2,5,0,50.6666679382324,9,0.333333343267441,60,3,2,204,69,0,1,0,1,0,11,
11364,2120,2010-12-03,3,5,0,27.1666660308838,32.3333320617676,0.5,60,3,2,204,69,0,1,0,1,0,11
11365,2120,2010-12-03,4,5,0,23,36,1,60,3,2,204,69,0,1,0,1,0,11,1001.784696,1133.824623,0,0,0,0,
11366,2120,2010-12-04,0,6,1,41,10.5,0,51.5,2,2,204,69,0,1,0,1,0,11,1001.784696,1133.824623,0,0,
11367,2120,2010-12-04,1,6,1,23.25,35.25,1.5,60,3,2,204,69,0,1,0,1,0,11,1001.784696,1133.824623
11368,2120,2010-12-04,2,6,1,31,27.6666660308838,1.33333337306976,60,3,2,204,69,0,1,0,1,0,11,
11369,2120,2010-12-04,3,6,1,18.6666660308838,41,0.333333343267441,60,3,2,204,69,0,1,0,1,0,11
11370,2120,2010-12-04,4,6,1,46.7999992370605,12.1999998092651,1,60,3,2,204,69,0,1,0,1,0,11,1
11371,2120,2010-12-05,0,0,1,27.5,2.5,0.5,30.5,2,2,204,69,0,1,0,1,0,11,1001.784696,1133.824623,0
11372,2120,2010-12-05,1,0,1,21.2000007629395,36,2.79999995231628,60,3,2,204,69,0,1,0,1,0,11,
11373,2120,2010-12-05,2,0,1,26.75,32.75,0.5,60,3,2,204,69,0,1,0,1,0,11,1001.784696,1133.824623
11374,2120,2010-12-05,3,0,1,25.6666660308838,33.6666679382324,0.666666686534882,60,3,2,20
11375,2120,2010-12-05,4,0,1,45,15,0,60,3,2,204,69,0,1,0,1,0,11,1001.784696,1133.824623,0,0,0,0,
11376,2120,2010-12-06,0,1,0,31,13,2,46,1,2,204,69,0,1,0,1,0,11,1001.784696,1133.824623,0,0,0,0,
11377,2120,2010-12-06,1,1,0,36.6666679382324,22.3333339691162,1,60,3,2,204,69,0,1,0,1,0,11,1
11378,2120,2010-12-06,2,1,0,22.2000007629395,31.7999992370605,6,60,3,2,204,69,0,1,0,1,0,11,1
11379,2120,2010-12-06,3,1,0,31,24.7999992370605,4.19999980926514,60,3,2,204,69,0,1,0,1,0,11,
11380,2120,2010-12-06,4,1,0,30.3333339691162,29.3333339691162,0.333333343267441,60,3,2,20
11381,2120,2010-12-07,3,2,0,9,44,7,60,1,2,204,69,0,1,0,1,0,11,1001.784696,1133.824623,0,0,0,0,0
11382,2120,2010-12-07,4,2,0,29,27.6666660308838,3.33333325386047,60,3,2,204,69,0,1,0,1,0,11,
11383,2133,2010-12-01,0,3,0,7,16,2.5,25.5,2,2,204,67,0,1,0,1,0,12,976.5929193,951.4739826,0.764
11384,2133,2010-12-01,1,3,0,17.3333339691162,38.3333320617676,4.33333349227905,60,3,2,204
11385,2133,2010-12-01,2,3,0,27.75,29,3.25,60,3,2,204,67,0,1,0,1,0,12,976.5929193,951.4739826,0
11386,2133,2010-12-01,3,3,0,38.6666679382324,18.6666660308838,2.66666674613953,60,3,2,204
11387,2133,2010-12-01,4,3,0,50.3333320617676,9.33333301544189,0.333333343267441,60,3,2,20
11388,2133,2010-12-02,0,4,0,23,29,1,53,1,2,204,67,0,1,0,1,0,12,976.5929193,951.4739826,0.7641
11389,2133,2010-12-02,1,4,0,9,46,5,60,3,2,204,67,0,1,0,1,0,12,976.5929193,951.4739826,0.764117
11390,2133,2010-12-02,2,4,0,11.1999998092651,41.2000007629395,7.59999990463257,60,3,2,204
11391,2133,2010-12-02,3,4,0,43,16.1666660308838,0.833333313465118,60,3,2,204,67,0,1,0,1,0,12
11392,2133,2010-12-02,4,4,0,35.6666679382324,23,1.33333337306976,60,3,2,204,67,0,1,0,1,0,12,
11393,2133,2010-12-03,0,5,0,20.3333339691162,36,3.66666674613953,60,3,2,204,67,0,1,0,1,0,12,
11394,2133,2010-12-03,1,5,0,7.33333349227905,49.3333320617676,3.33333325386047,60,3,2,204
11395,2133,2010-12-03,2,5,0,11.3333330154419,44.6666679382324,4,60,3,2,204,67,0,1,0,1,0,12,9
11396,2133,2010-12-03,3,5,0,17,40.3333320617676,2.66666674613953,60,3,2,204,67,0,1,0,1,0,12,
11397,2133,2010-12-03,4,5,0,29,28.6666660308838,2.33333325386047,60,3,2,204,67,0,1,0,1,0,12,
11398,2133,2010-12-04,0,6,1,7,32,1.5,40.5,2,2,204,67,0,1,0,1,0,12,976.5929193,951.4739826,0.764
11399,2133,2010-12-04,1,6,1,14.3333330154419,40.6666679382324,5,60,3,2,204,67,0,1,0,1,0,12,9

11400,2133,2010-12-04,2,6,1,45.6666679382324,12,2.33333325386047,60,3,2,204,67,0,1,0,1,0,12,
11401,2133,2010-12-04,3,6,1,22.6666660308838,31.6666660308838,5.66666650772095,60,3,2,204
11402,2133,2010-12-04,4,6,1,24.6000003814697,34.2000007629395,1.20000004768372,60,3,2,204
11403,2133,2010-12-05,0,0,1,15.5,18.5,0,34,2,2,204,67,0,1,0,1,0,12,976.5929193,951.4739826,0.76
11404,2133,2010-12-05,1,0,1,16.3333339691162,41,2.66666674613953,60,3,2,204,67,0,1,0,1,0,12,
11405,2133,2010-12-05,2,0,1,16.3333339691162,42.6666679382324,1,60,3,2,204,67,0,1,0,1,0,12,9
11406,2133,2010-12-05,3,0,1,16,38.3333320617676,5.66666650772095,60,3,2,204,67,0,1,0,1,0,12,
11407,2133,2010-12-05,4,0,1,23.25,31.25,5.5,60,3,2,204,67,0,1,0,1,0,12,976.5929193,951.4739826
11408,2133,2010-12-07,0,2,0,5,52,3,60,1,2,204,67,0,1,0,1,0,12,976.5929193,951.4739826,0.764117
11409,2133,2010-12-07,1,2,0,6,42,12,60,3,2,204,67,0,1,0,1,0,12,976.5929193,951.4739826,0.76411
11410,2133,2010-12-07,2,2,0,6.33333349227905,47,6.66666650772095,60,3,2,204,67,0,1,0,1,0,12,
11411,2133,2010-12-07,3,2,0,9.66666698455811,42.6666679382324,7.66666650772095,60,3,2,204
11412,2133,2010-12-07,4,2,0,17.6666660308838,27.3333339691162,15,60,3,2,204,67,0,1,0,1,0,12,
11413,2139,2012-02-19,0,7,1,35,24,1,60,1,2,204,76,1,0,1,0,0,9,1023.13803,761.9923468,0.7422345
11414,2139,2012-02-19,1,7,1,24.3333339691162,34.3333320617676,1.33333337306976,60,3,2,204
11415,2139,2012-02-19,2,7,1,38.6666679382324,18.6666660308838,2.66666674613953,60,3,2,204
11416,2139,2012-02-19,3,7,1,50.3333320617676,9.66666698455811,0,60,3,2,204,76,1,0,1,0,0,9,10
11417,2139,2012-02-19,4,7,1,20,9.33333301544189,0.333333343267441,29.6666660308838,3,2,20
11418,2139,2012-02-20,0,1,0,9,14.3333330154419,1,24.3333339691162,3,2,204,76,1,0,1,0,0,9,102
11419,2139,2012-02-20,1,1,0,32.6666679382324,25.6666660308838,1.66666662693024,60,3,2,204
11420,2139,2012-02-20,2,1,0,19.3333339691162,33,7.66666650772095,60,3,2,204,76,1,0,1,0,0,9,1
11421,2139,2012-02-20,3,1,0,10,38.6666679382324,11.3333330154419,60,3,2,204,76,1,0,1,0,0,9,1
11422,2139,2012-02-20,4,1,0,13.3333330154419,8.33333301544189,0.333333343267441,22,3,2,20
11423,2139,2012-02-21,0,2,0,10.3333330154419,19,0,29.3333339691162,3,2,204,76,1,0,1,0,0,9,10
11424,2139,2012-02-21,1,2,0,30.6666660308838,28,1.33333337306976,60,3,2,204,76,1,0,1,0,0,9,1
11425,2139,2012-02-21,2,2,0,37,23,0,60,3,2,204,76,1,0,1,0,0,9,1023.13803,761.9923468,0.7422345
11426,2139,2012-02-21,3,2,0,26,32.3333320617676,1.66666662693024,60,3,2,204,76,1,0,1,0,0,9,1
11427,2139,2012-02-21,4,2,0,20,11,0.666666686534882,31.6666660308838,3,2,204,76,1,0,1,0,0,9,
11428,2139,2012-02-22,0,3,0,3,17.3333339691162,1.33333337306976,21.6666660308838,3,2,204,
11429,2139,2012-02-22,1,3,0,7.66666650772095,44.6666679382324,7.66666650772095,60,3,2,204
11430,2139,2012-02-22,2,3,0,27,30,3,60,3,2,204,76,1,0,1,0,0,9,1023.13803,761.9923468,0.7422345
11431,2139,2012-02-22,3,3,0,35.6666679382324,22.3333339691162,2,60,3,2,204,76,1,0,1,0,0,9,10
11432,2139,2012-02-22,4,3,0,39.6666679382324,14,0.666666686534882,54.3333320617676,3,2,20
11433,2139,2012-02-23,0,4,0,10,18.6666660308838,0.666666686534882,29.3333339691162,3,2,20
11434,2139,2012-02-23,1,4,0,19.3333339691162,39,1.66666662693024,60,3,2,204,76,1,0,1,0,0,9,1
11435,2139,2012-02-23,2,4,0,34.3333320617676,23.3333339691162,2.33333325386047,60,3,2,204
11436,2139,2012-02-23,3,4,0,22,33.3333320617676,4.66666650772095,60,3,2,204,76,1,0,1,0,0,9,1
11437,2139,2012-02-23,4,4,0,6,11.3333330154419,1.33333337306976,18.6666660308838,3,2,204,
11438,2139,2012-02-24,0,5,0,13.3333330154419,15.3333330154419,1.66666662693024,30.333333
11439,2139,2012-02-24,1,5,0,14,43,3,60,3,2,204,76,1,0,1,0,0,9,1023.13803,761.9923468,0.7422345
11440,2139,2012-02-24,2,5,0,20.6666660308838,34.6666679382324,4.66666650772095,60,3,2,204
11441,2139,2012-02-24,3,5,0,12.3333330154419,40,7.66666650772095,60,3,2,204,76,1,0,1,0,0,9,1
11442,2139,2012-02-24,4,5,0,15.3333330154419,8.66666698455811,0.666666686534882,24.66666
11443,2139,2012-02-25,0,6,1,10,17.3333339691162,2.66666674613953,30,3,2,204,76,1,0,1,0,0,9,1
11444,2139,2012-02-25,1,6,1,10.3333330154419,46.6666679382324,3,60,3,2,204,76,1,0,1,0,0,9,10
11445,2139,2012-02-25,2,6,1,12.3333330154419,42.6666679382324,5,60,3,2,204,76,1,0,1,0,0,9,10
11446,2139,2012-02-25,3,6,1,16.6666660308838,34.6666679382324,8.66666698455811,60,3,2,204
11447,2139,2012-02-25,4,6,1,17,9.66666698455811,1,27.6666660308838,3,2,204,76,1,0,1,0,0,9,10
11448,2141,2010-11-25,0,4,0,5,31,0.5,36.5,2,2,204,76,1,0,1,0,0,6,951.6206306,913.5382497,0.7631
11449,2141,2010-11-25,1,4,0,16.6000003814697,38.4000015258789,5,60,3,2,204,76,1,0,1,0,0,6,95

11450,2141,2010-11-25,2,4,0,10,49,1,60,3,2,204,76,1,0,1,0,0,6,951.6206306,913.5382497,0.76313
11451,2141,2010-11-25,3,4,0,24.6666660308838,34.6666679382324,0.666666686534882,60,3,2,2
11452,2141,2010-11-25,4,4,0,27.6666660308838,32.3333320617676,0,60,3,2,204,76,1,0,1,0,0,6,95
11453,2141,2010-11-26,0,5,0,13.5,40,0.5,54,2,2,204,76,1,0,1,0,0,6,951.6206306,913.5382497,0.76
11454,2141,2010-11-26,1,5,0,8,49.3333320617676,2.66666674613953,60,3,2,204,76,1,0,1,0,0,6,95
11455,2141,2010-11-26,2,5,0,33.6666679382324,25.1666660308838,1.16666662693024,60,3,2,20
11456,2141,2010-11-26,3,5,0,19.3333339691162,38.3333320617676,2.33333325386047,60,3,2,20
11457,2141,2010-11-26,4,5,0,15.6666669845581,44,0.333333343267441,60,3,2,204,76,1,0,1,0,0,6,
11458,2141,2010-11-27,0,6,1,14.5,23,1,38.5,2,2,204,76,1,0,1,0,0,6,951.6206306,913.5382497,0.76
11459,2141,2010-11-27,1,6,1,18.6666660308838,41.3333320617676,0,60,3,2,204,76,1,0,1,0,0,6,95
11460,2141,2010-11-27,2,6,1,23,35.6666679382324,1.33333337306976,60,3,2,204,76,1,0,1,0,0,6,9
11461,2141,2010-11-27,3,6,1,31.3333339691162,28,0.666666686534882,60,3,2,204,76,1,0,1,0,0,6,
11462,2141,2010-11-27,4,6,1,28.75,31.25,0,60,3,2,204,76,1,0,1,0,0,6,951.6206306,913.5382497,0.
11463,2141,2010-11-28,0,0,1,8,29.25,1.5,38.75,3,2,204,76,1,0,1,0,0,6,951.6206306,913.5382497,0.
11464,2141,2010-11-28,1,0,1,9.66666698455811,43.6666679382324,6.66666650772095,60,3,2,20
11465,2141,2010-11-28,2,0,1,10.6666669845581,31.6666660308838,17.6666660308838,60,3,2,20
11466,2141,2010-11-28,3,0,1,25.3333339691162,27.3333339691162,7.33333349227905,60,3,2,20
11467,2141,2010-11-28,4,0,1,31,28.7999992370605,0.200000002980232,60,3,2,204,76,1,0,1,0,0,6,
11468,2141,2010-11-30,1,2,0,7.66666650772095,46.3333320617676,6,60,3,2,204,76,1,0,1,0,0,6,95
11469,2141,2010-11-30,2,2,0,12.3333330154419,46.6666679382324,1,60,3,2,204,76,1,0,1,0,0,6,95
11470,2141,2010-11-30,3,2,0,17.3333339691162,41.6666679382324,1,60,3,2,204,76,1,0,1,0,0,6,95
11471,2141,2010-11-30,4,2,0,38.3333320617676,21,0.666666686534882,60,3,2,204,76,1,0,1,0,0,6,
11472,2141,2010-12-05,0,0,1,16,44,0,60,1,2,204,76,1,0,1,0,0,6,951.6206306,913.5382497,0.76313
11473,2141,2010-12-05,1,0,1,1,51,8,60,1,2,204,76,1,0,1,0,0,6,951.6206306,913.5382497,0.763136
11474,2150,2010-11-30,0,2,0,33,27,0,60,1,2,211,85,1,1,1,0,0,9,7646.054312,6932.311462,10.2166
11475,2150,2010-11-30,1,2,0,17.3333339691162,32.6666679382324,10,60,3,2,211,85,1,1,1,0,0,9,7
11476,2150,2010-11-30,2,2,0,30,29,1,60,2,2,211,85,1,1,1,0,0,9,7646.054312,6932.311462,10.2166
11477,2150,2010-12-08,0,3,0,5,29,0,34,1,2,211,85,1,1,1,0,0,9,7646.054312,6932.311462,10.21661
11478,2150,2010-12-08,1,3,0,19,36.3333320617676,4.66666650772095,60,3,2,211,85,1,1,1,0,0,9,7
11479,2150,2010-12-08,2,3,0,23.25,34.5,2.25,60,3,2,211,85,1,1,1,0,0,9,7646.054312,6932.311462,
11480,2150,2010-12-08,3,3,0,33.2000007629395,23,3.79999995231628,60,3,2,211,85,1,1,1,0,0,9,7
11481,2150,2010-12-08,4,3,0,42.6666679382324,17.3333339691162,0,60,3,2,211,85,1,1,1,0,0,9,76
11482,2150,2010-12-09,0,4,0,22,19,2.66666674613953,43.6666679382324,3,2,211,85,1,1,1,0,0,9,7
11483,2150,2010-12-09,1,4,0,24.3333339691162,33.6666679382324,2,60,3,2,211,85,1,1,1,0,0,9,76
11484,2150,2010-12-09,2,4,0,36.6666679382324,23.3333339691162,0,60,3,2,211,85,1,1,1,0,0,9,76
11485,2150,2010-12-09,3,4,0,19.5,38.25,2.25,60,3,2,211,85,1,1,1,0,0,9,7646.054312,6932.311462,
11486,2150,2010-12-09,4,4,0,31,15,0.666666686534882,46.6666679382324,3,2,211,85,1,1,1,0,0,9,
11487,2150,2010-12-10,0,5,0,22.5,32,1,55.5,2,2,211,85,1,1,1,0,0,9,7646.054312,6932.311462,10.2
11488,2150,2010-12-10,1,5,0,32.3333320617676,24.3333339691162,3.33333325386047,60,3,2,21
11489,2150,2010-12-10,2,5,0,18,38,4,60,3,2,211,85,1,1,1,0,0,9,7646.054312,6932.311462,10.2166
11490,2150,2010-12-10,3,5,0,13.3333330154419,46.3333320617676,0.333333343267441,60,3,2,2
11491,2150,2010-12-10,4,5,0,28.1666660308838,31.3333339691162,0.5,60,3,2,211,85,1,1,1,0,0,9,
11492,2150,2010-12-11,0,6,1,8,23,1,32,1,2,211,85,1,1,1,0,0,9,7646.054312,6932.311462,10.21661
11493,2150,2010-12-11,1,6,1,31,25.1666660308838,3.83333325386047,60,3,2,211,85,1,1,1,0,0,9,7
11494,2150,2010-12-11,2,6,1,21.7999992370605,25.6000003814697,12.6000003814697,60,3,2,21
11495,2150,2010-12-11,3,6,1,25,29.3333339691162,5.66666650772095,60,3,2,211,85,1,1,1,0,0,9,7
11496,2150,2010-12-11,4,6,1,7,18,1,26,1,2,211,85,1,1,1,0,0,9,7646.054312,6932.311462,10.21661
11497,2150,2010-12-12,0,0,1,24,22.3333339691162,5,51.3333320617676,3,2,211,85,1,1,1,0,0,9,76
11498,2150,2010-12-12,1,0,1,17,23,20,60,3,2,211,85,1,1,1,0,0,9,7646.054312,6932.311462,10.216
11499,2150,2010-12-12,2,0,1,28.5,26.25,5.25,60,3,2,211,85,1,1,1,0,0,9,7646.054312,6932.311462,

11500,2150,2010-12-12,3,0,1,10.6666669845581,33.6666679382324,15.6666669845581,60,3,2,21
11501,2150,2010-12-12,4,0,1,23,24,1.66666662693024,48.6666679382324,3,2,211,85,1,1,1,0,0,9,7
11502,2150,2010-12-13,3,1,0,30.6666660308838,25.6666660308838,3.66666674613953,60,3,2,21
11503,2150,2010-12-13,4,1,0,29.3333339691162,21.3333339691162,0.333333343267441,51,3,2,2
11504,2151,2010-12-08,0,3,0,15,16.5,0.5,32,2,2,211,67,1,1,0,1,0,12,6404.974985,6820.172329,12.0
11505,2151,2010-12-08,1,3,0,4.66666650772095,25,30.3333339691162,60,3,2,211,67,1,1,0,1,0,12,
11506,2151,2010-12-08,2,3,0,16.2000007629395,41.4000015258789,2.40000009536743,60,3,2,21
11507,2151,2010-12-08,3,3,0,5.33333349227905,51.6666679382324,3,60,3,2,211,67,1,1,0,1,0,12,6
11508,2151,2010-12-08,4,3,0,15.3333330154419,42.6666679382324,2,60,3,2,211,67,1,1,0,1,0,12,6
11509,2151,2010-12-09,0,4,0,32.6666679382324,17.6666660308838,3,53.3333320617676,3,2,211,
11510,2151,2010-12-09,1,4,0,4,20.3333339691162,35.6666679382324,60,3,2,211,67,1,1,0,1,0,12,6
11511,2151,2010-12-09,2,4,0,14.25,18,27.75,60,3,2,211,67,1,1,0,1,0,12,6404.974985,6820.172329,
11512,2151,2010-12-09,3,4,0,9.5,47,3.5,60,3,2,211,67,1,1,0,1,0,12,6404.974985,6820.172329,12.02
11513,2151,2010-12-09,4,4,0,22.3333339691162,36,1.66666662693024,60,3,2,211,67,1,1,0,1,0,12,
11514,2151,2010-12-10,0,5,0,25.5,24,1.5,51,2,2,211,67,1,1,0,1,0,12,6404.974985,6820.172329,12.0
11515,2151,2010-12-10,1,5,0,32.3333320617676,27,0.666666686534882,60,3,2,211,67,1,1,0,1,0,1
11516,2151,2010-12-10,2,5,0,28.3333339691162,31.6666660308838,0,60,3,2,211,67,1,1,0,1,0,12,6
11517,2151,2010-12-10,3,5,0,15.1999998092651,27.3999996185303,17.3999996185303,60,3,2,21
11518,2151,2010-12-10,4,5,0,31.5,27.25,1.25,60,3,2,211,67,1,1,0,1,0,12,6404.974985,6820.172329
11519,2151,2010-12-11,0,6,1,3,5,1,9,1,2,211,67,1,1,0,1,0,12,6404.974985,6820.172329,12.027803
11520,2151,2010-12-11,1,6,1,23.3333339691162,30.6666660308838,6,60,3,2,211,67,1,1,0,1,0,12,6
11521,2151,2010-12-11,2,6,1,23,35.6666679382324,1.33333337306976,60,3,2,211,67,1,1,0,1,0,12,
11522,2151,2010-12-11,3,6,1,16.3333339691162,37,6.66666650772095,60,3,2,211,67,1,1,0,1,0,12,
11523,2151,2010-12-11,4,6,1,16.3999996185303,32.5999984741211,11,60,3,2,211,67,1,1,0,1,0,12,
11524,2151,2010-12-13,0,1,0,18,40,2,60,1,2,211,67,1,1,0,1,0,12,6404.974985,6820.172329,12.027
11525,2151,2010-12-13,1,1,0,16.6666660308838,42.3333320617676,1,60,3,2,211,67,1,1,0,1,0,12,6
11526,2151,2010-12-13,2,1,0,18,34.3333320617676,7.66666650772095,60,3,2,211,67,1,1,0,1,0,12,
11527,2151,2010-12-13,3,1,0,6,51.3333320617676,2.66666674613953,60,3,2,211,67,1,1,0,1,0,12,6
11528,2151,2010-12-13,4,1,0,14,46,0,60,1,2,211,67,1,1,0,1,0,12,6404.974985,6820.172329,12.027
11529,2151,2010-12-14,0,2,0,18,10,1,29,1,2,211,67,1,1,0,1,0,12,6404.974985,6820.172329,12.027
11530,2151,2010-12-14,1,2,0,20.8333339691162,35.8333320617676,1.16666662693024,57.83333
11531,2151,2010-12-14,2,2,0,10.6666669845581,46.3333320617676,3,60,3,2,211,67,1,1,0,1,0,12,6
11532,2151,2010-12-14,3,2,0,14.6666669845581,39.3333320617676,6,60,3,2,211,67,1,1,0,1,0,12,6
11533,2151,2010-12-14,4,2,0,30.7999992370605,28,1.20000004768372,60,3,2,211,67,1,1,0,1,0,12,
11534,2155,2010-12-11,0,6,1,39,21,0,60,1,2,211,65,0,1,0,1,0,12,6754.511113,6227.733429,11.752
11535,2155,2010-12-11,1,6,1,41,19,0,60,1,2,211,65,0,1,0,1,0,12,6754.511113,6227.733429,11.752
11536,2155,2010-12-12,1,0,1,7,44.2000007629395,7.19999980926514,58.4000015258789,3,2,211,
11537,2155,2010-12-12,2,0,1,22.6666660308838,31.5,5.83333349227905,60,3,2,211,65,0,1,0,1,0,1
11538,2155,2010-12-12,3,0,1,34.3333320617676,23.6666660308838,2,60,3,2,211,65,0,1,0,1,0,12,6
11539,2155,2010-12-12,4,0,1,33.6666679382324,25.6666660308838,0.666666686534882,60,3,2,2
11540,2155,2010-12-13,0,1,0,6,35,3,44,2,2,211,65,0,1,0,1,0,12,6754.511113,6227.733429,11.7524
11541,2155,2010-12-13,1,1,0,19.6666660308838,37.6666679382324,2.66666674613953,60,3,2,21
11542,2155,2010-12-13,2,1,0,18.3333339691162,38.3333320617676,3.33333325386047,60,3,2,21
11543,2155,2010-12-13,3,1,0,21.3999996185303,31,7.59999990463257,60,3,2,211,65,0,1,0,1,0,12,
11544,2155,2010-12-13,4,1,0,28.3333339691162,30.3333339691162,1.33333337306976,60,3,2,21
11545,2155,2010-12-14,0,2,0,6,36,9,51,1,2,211,65,0,1,0,1,0,12,6754.511113,6227.733429,11.7524
11546,2155,2010-12-14,1,2,0,11,43.5,2.75,57.25,3,2,211,65,0,1,0,1,0,12,6754.511113,6227.733429
11547,2155,2010-12-14,2,2,0,20,35.3333320617676,4.66666650772095,60,3,2,211,65,0,1,0,1,0,12,
11548,2155,2010-12-14,3,2,0,17,37.75,5.25,60,3,2,211,65,0,1,0,1,0,12,6754.511113,6227.733429,1
11549,2155,2010-12-14,4,2,0,29.5,26.6666660308838,3.83333325386047,60,3,2,211,65,0,1,0,1,0,1

11550,2155,2011-01-12,0,3,0,8.5,38,6.5,53,2,2,211,65,0,1,0,1,0,12,6754.511113,6227.733429,11.75
11551,2155,2011-01-12,1,3,0,19.75,38.5,1.75,60,3,2,211,65,0,1,0,1,0,12,6754.511113,6227.733429
11552,2155,2011-01-12,2,3,0,35,25,0,60,3,2,211,65,0,1,0,1,0,12,6754.511113,6227.733429,11.752
11553,2155,2011-01-12,3,3,0,7.66666650772095,46,6.33333349227905,60,3,2,211,65,0,1,0,1,0,12,
11554,2155,2011-01-12,4,3,0,37.6666679382324,21,1.33333337306976,60,3,2,211,65,0,1,0,1,0,12,
11555,2155,2011-01-13,0,4,0,15.5,26,0,41.5,2,2,211,65,0,1,0,1,0,12,6754.511113,6227.733429,11.
11556,2155,2011-01-13,1,4,0,20.5,36.5,3,60,3,2,211,65,0,1,0,1,0,12,6754.511113,6227.733429,11.
11557,2155,2011-01-13,2,4,0,16.25,43,0.75,60,3,2,211,65,0,1,0,1,0,12,6754.511113,6227.733429,1
11558,2155,2011-01-13,3,4,0,16.3333339691162,33,10.6666669845581,60,3,2,211,65,0,1,0,1,0,12,
11559,2155,2011-01-13,4,4,0,35,23.6666660308838,1.33333337306976,60,3,2,211,65,0,1,0,1,0,12,
11560,2155,2011-01-14,2,5,0,23,35.5,1.5,60,2,2,211,65,0,1,0,1,0,12,6754.511113,6227.733429,11.
11561,2155,2011-01-14,3,5,0,18.3333339691162,37.3333320617676,4.33333349227905,60,3,2,21
11562,2155,2011-01-14,4,5,0,16,22,2.33333325386047,40.3333320617676,3,2,211,65,0,1,0,1,0,12,
11563,2157,2011-01-14,0,5,0,33,25,2,60,1,2,211,79,0,1,0,1,0,12,7642.77211,6897.902098,8.917496
11564,2157,2011-01-14,1,5,0,41.6666679382324,15.6666669845581,2.66666674613953,60,3,2,21
11565,2157,2011-01-14,2,5,0,29.6666660308838,24.6666660308838,5.66666650772095,60,3,2,21
11566,2157,2011-01-15,1,6,1,7.66666650772095,33,1,41.6666679382324,3,2,211,79,0,1,0,1,0,12,7
11567,2157,2011-01-15,2,6,1,18.6666660308838,40,1.33333337306976,60,3,2,211,79,0,1,0,1,0,12,
11568,2157,2011-01-15,3,6,1,32,26.2000007629395,1.79999995231628,60,3,2,211,79,0,1,0,1,0,12,
11569,2157,2011-01-15,4,6,1,37.3333320617676,18.3333339691162,4.33333349227905,60,3,2,21
11570,2157,2011-01-17,1,1,0,5.33333349227905,39,0.333333343267441,44.6666679382324,3,2,2
11571,2157,2011-01-17,2,1,0,16,43.3333320617676,0.666666686534882,60,3,2,211,79,0,1,0,1,0,1
11572,2157,2011-01-17,3,1,0,34,24.75,1.25,60,3,2,211,79,0,1,0,1,0,12,7642.77211,6897.902098,8.
11573,2157,2011-01-17,4,1,0,28.2000007629395,31,0.800000011920929,60,3,2,211,79,0,1,0,1,0,1
11574,2157,2011-02-02,0,3,0,1,26,2,29,1,2,211,79,0,1,0,1,0,12,7642.77211,6897.902098,8.9174961
11575,2157,2011-02-02,1,3,0,4.33333349227905,50.6666679382324,5,60,3,2,211,79,0,1,0,1,0,12,7
11576,2157,2011-02-02,2,3,0,30.6666660308838,29.3333339691162,0,60,3,2,211,79,0,1,0,1,0,12,7
11577,2157,2011-02-02,3,3,0,13.6666669845581,45.3333320617676,1,60,3,2,211,79,0,1,0,1,0,12,7
11578,2157,2011-02-02,4,3,0,37.25,21.75,1,60,3,2,211,79,0,1,0,1,0,12,7642.77211,6897.902098,8.
11579,2157,2011-02-03,0,4,0,9.5,25.5,2.5,37.5,2,2,211,79,0,1,0,1,0,12,7642.77211,6897.902098,8.
11580,2157,2011-02-03,1,4,0,5.5,47.75,6.75,60,3,2,211,79,0,1,0,1,0,12,7642.77211,6897.902098,8.
11581,2157,2011-02-03,2,4,0,32,26.3333339691162,1.66666662693024,60,3,2,211,79,0,1,0,1,0,12,
11582,2157,2011-02-03,3,4,0,38,14,8,60,3,2,211,79,0,1,0,1,0,12,7642.77211,6897.902098,8.917496
11583,2157,2011-02-03,4,4,0,35,23.6666660308838,1.33333337306976,60,3,2,211,79,0,1,0,1,0,12,
11584,2157,2011-02-04,0,5,0,10,32,0,42,1,2,211,79,0,1,0,1,0,12,7642.77211,6897.902098,8.917496
11585,2157,2011-02-04,1,5,0,4.75,44.5,10.75,60,3,2,211,79,0,1,0,1,0,12,7642.77211,6897.902098,
11586,2157,2011-02-04,2,5,0,15.3333330154419,32.6666679382324,12,60,3,2,211,79,0,1,0,1,0,12,
11587,2157,2011-02-04,3,5,0,7.66666650772095,46.3333320617676,6,60,3,2,211,79,0,1,0,1,0,12,7
11588,2157,2011-02-04,4,5,0,25.6666660308838,34,0.333333343267441,60,3,2,211,79,0,1,0,1,0,1
11589,2157,2011-02-05,1,6,1,9,48,3,60,1,2,211,79,0,1,0,1,0,12,7642.77211,6897.902098,8.9174961
11590,2157,2011-02-05,2,6,1,15.3333330154419,39.6666679382324,5,60,3,2,211,79,0,1,0,1,0,12,7
11591,2157,2011-02-05,3,6,1,25.6666660308838,33.6666679382324,0.666666686534882,60,3,2,2
11592,2157,2011-02-05,4,6,1,7.66666650772095,44,8.33333301544189,60,3,2,211,79,0,1,0,1,0,12,
11593,2158,2011-01-12,1,3,0,25.5,23.5,0.5,49.5,3,2,211,80,1,0,0,1,0,10,7642.77211,6897.902098,8
11594,2158,2011-01-12,2,3,0,37.6666679382324,22,0.333333343267441,60,3,2,211,80,1,0,0,1,0,1
11595,2158,2011-01-12,3,3,0,41.3333320617676,11.3333330154419,7.33333349227905,60,3,2,21
11596,2158,2011-01-12,4,3,0,44.75,15,0.25,60,3,2,211,80,1,0,0,1,0,10,7642.77211,6897.902098,8.
11597,2158,2011-01-14,1,5,0,34.6666679382324,19.6666660308838,0.666666686534882,55,3,2,2
11598,2158,2011-01-14,2,5,0,37.25,22.75,0,60,3,2,211,80,1,0,0,1,0,10,7642.77211,6897.902098,8.
11599,2158,2011-01-14,3,5,0,32.3333320617676,27.6666660308838,0,60,3,2,211,80,1,0,0,1,0,10,7

11600,2158,2011-01-14,4,5,0,26.6666660308838,33.3333320617676,0,60,3,2,211,80,1,0,0,1,0,10,7
11601,2158,2011-01-15,2,6,1,43.4000015258789,16.2000007629395,0.200000002980232,59.7999
11602,2158,2011-01-15,3,6,1,39.6666679382324,20.1666660308838,0.16666667163372,60,3,2,21
11603,2158,2011-01-15,4,6,1,34.5,25.25,0.25,60,3,2,211,80,1,0,0,1,0,10,7642.77211,6897.902098,8
11604,2158,2011-01-16,4,0,1,36.5,23.5,0,60,2,2,211,80,1,0,0,1,0,10,7642.77211,6897.902098,8.917
11605,2158,2011-02-05,0,6,1,15,16,0,31,1,2,211,80,1,0,0,1,0,10,7642.77211,6897.902098,8.91749
11606,2158,2011-02-05,1,6,1,34,13,1,48,1,2,211,80,1,0,0,1,0,10,7642.77211,6897.902098,8.91749
11607,2158,2011-02-05,2,6,1,24,36,0,60,1,2,211,80,1,0,0,1,0,10,7642.77211,6897.902098,8.91749
11608,2158,2011-02-07,1,1,0,14.6666669845581,24.6666660308838,1.33333337306976,40.66666
11609,2158,2011-02-07,2,1,0,34.4000015258789,19.3999996185303,0,53.7999992370605,3,2,211,
11610,2158,2011-02-07,3,1,0,30,15,0.25,45.25,3,2,211,80,1,0,0,1,0,10,7642.77211,6897.902098,8.9
11611,2158,2011-02-07,4,1,0,42,18,0,60,3,2,211,80,1,0,0,1,0,10,7642.77211,6897.902098,8.91749
11612,2158,2011-02-08,1,2,0,32,14.6666669845581,0,46.6666679382324,3,2,211,80,1,0,0,1,0,10,7
11613,2158,2011-02-08,2,2,0,34.6666679382324,25.3333339691162,0,60,3,2,211,80,1,0,0,1,0,10,7
11614,2158,2011-02-08,3,2,0,43.4000015258789,16.3999996185303,0.200000002980232,60,3,2,2
11615,2158,2011-02-08,4,2,0,39,20.6000003814697,0.400000005960464,60,3,2,211,80,1,0,0,1,0,1
11616,2159,2011-01-16,0,0,1,34,26,0,60,1,2,211,66,0,1,0,1,0,11,5682.297247,6632.858577,6.1600
11617,2159,2011-01-16,1,0,1,31.6666660308838,26.6666660308838,1.66666662693024,60,3,2,21
11618,2159,2011-01-16,2,0,1,23.6666660308838,33.6666679382324,2.66666674613953,60,3,2,21
11619,2159,2011-01-16,3,0,1,21,33.6666679382324,5.33333349227905,60,3,2,211,66,0,1,0,1,0,11,
11620,2159,2011-01-17,0,1,0,27,10,1,38,1,2,211,66,0,1,0,1,0,11,5682.297247,6632.858577,6.1600
11621,2159,2011-01-17,1,1,0,31,27,2,60,3,2,211,66,0,1,0,1,0,11,5682.297247,6632.858577,6.1600
11622,2159,2011-01-17,2,1,0,23.3333339691162,35.3333320617676,1.33333337306976,60,3,2,21
11623,2159,2011-01-17,3,1,0,39.3333320617676,20.3333339691162,0.333333343267441,60,3,2,2
11624,2159,2011-01-17,4,1,0,18,40.6666679382324,1.33333337306976,60,3,2,211,66,0,1,0,1,0,11,
11625,2159,2011-01-18,0,2,0,19.5,11.5,0.5,31.5,2,2,211,66,0,1,0,1,0,11,5682.297247,6632.858577,
11626,2159,2011-01-18,1,2,0,28.3333339691162,26.6666660308838,5,60,3,2,211,66,0,1,0,1,0,11,5
11627,2159,2011-01-18,2,2,0,30,28.6666660308838,1.33333337306976,60,3,2,211,66,0,1,0,1,0,11,
11628,2159,2011-01-18,4,2,0,10,22.6666660308838,27.3333339691162,60,3,2,211,66,0,1,0,1,0,11,
11629,2159,2011-02-02,0,3,0,40,7,2,49,1,2,211,66,0,1,0,1,0,11,5682.297247,6632.858577,6.16008
11630,2159,2011-02-02,1,3,0,20.3333339691162,37.6666679382324,2,60,3,2,211,66,0,1,0,1,0,11,5
11631,2159,2011-02-02,2,3,0,24,35.6666679382324,0.333333343267441,60,3,2,211,66,0,1,0,1,0,1
11632,2159,2011-02-02,3,3,0,21.5,35,3.5,60,3,2,211,66,0,1,0,1,0,11,5682.297247,6632.858577,6.1
11633,2159,2011-02-02,4,3,0,31.6666660308838,25.6666660308838,2.66666674613953,60,3,2,21
11634,2159,2011-02-03,0,4,0,27.5,10.5,0.5,38.5,2,2,211,66,0,1,0,1,0,11,5682.297247,6632.858577,
11635,2159,2011-02-03,1,4,0,32.6666679382324,23.3333339691162,4,60,3,2,211,66,0,1,0,1,0,11,5
11636,2159,2011-02-03,2,4,0,24.3333339691162,35.3333320617676,0.333333343267441,60,3,2,2
11637,2159,2011-02-03,3,4,0,33,25.75,1.25,60,3,2,211,66,0,1,0,1,0,11,5682.297247,6632.858577,6
11638,2159,2011-02-03,4,4,0,20.6666660308838,27.6666660308838,11.6666669845581,60,3,2,21
11639,2159,2011-02-04,0,5,0,28,20,2,50,1,2,211,66,0,1,0,1,0,11,5682.297247,6632.858577,6.1600
11640,2159,2011-02-04,1,5,0,22,35,3,60,3,2,211,66,0,1,0,1,0,11,5682.297247,6632.858577,6.1600
11641,2159,2011-02-04,2,5,0,22.3333339691162,35,2.66666674613953,60,3,2,211,66,0,1,0,1,0,11,
11642,2159,2011-02-04,3,5,0,36.5,23,0.5,60,3,2,211,66,0,1,0,1,0,11,5682.297247,6632.858577,6.1
11643,2159,2011-02-04,4,5,0,16.5,43.3333320617676,0.16666667163372,60,3,2,211,66,0,1,0,1,0,1
11644,2159,2011-02-05,0,6,1,15,14.5,1,30.5,2,2,211,66,0,1,0,1,0,11,5682.297247,6632.858577,6.1
11645,2159,2011-02-05,1,6,1,35.3333320617676,20,4.66666650772095,60,3,2,211,66,0,1,0,1,0,11,
11646,2159,2011-02-05,2,6,1,29.3333339691162,29,1.66666662693024,60,3,2,211,66,0,1,0,1,0,11,
11647,2159,2011-02-05,3,6,1,26.6666660308838,30.6666660308838,2.66666674613953,60,3,2,21
11648,2159,2011-02-05,4,6,1,31,25.6666660308838,3.33333325386047,60,3,2,211,66,0,1,0,1,0,11,
11649,2160,2011-02-03,0,4,0,9,35,2,46,2,2,211,67,1,0,0,1,0,11,6264.139122,7626.774783,10.8929

11650,2160,2011-02-03,1,4,0,20,40,0,60,3,2,211,67,1,0,0,1,0,11,6264.139122,7626.774783,10.892
11651,2160,2011-02-03,2,4,0,17.5,38,4.5,60,3,2,211,67,1,0,0,1,0,11,6264.139122,7626.774783,10.
11652,2160,2011-02-03,3,4,0,7.33333349227905,45,7.66666650772095,60,3,2,211,67,1,0,0,1,0,11,
11653,2160,2011-02-03,4,4,0,33,26,1,60,3,2,211,67,1,0,0,1,0,11,6264.139122,7626.774783,10.892
11654,2160,2011-02-04,0,5,0,10.3333330154419,41.3333320617676,6.33333349227905,58,3,2,21
11655,2160,2011-02-04,1,5,0,1,42,17,60,3,2,211,67,1,0,0,1,0,11,6264.139122,7626.774783,10.892
11656,2160,2011-02-04,2,5,0,32.4000015258789,24.3999996185303,3.20000004768372,60,3,2,21
11657,2160,2011-02-04,3,5,0,28.3333339691162,24.3333339691162,7.33333349227905,60,3,2,21
11658,2160,2011-02-04,4,5,0,35,24.3333339691162,0.666666686534882,60,3,2,211,67,1,0,0,1,0,1
11659,2160,2011-02-05,0,6,1,13.3333330154419,40.3333320617676,4.33333349227905,58,3,2,21
11660,2160,2011-02-05,1,6,1,11,45.6666679382324,3.33333325386047,60,3,2,211,67,1,0,0,1,0,11,
11661,2160,2011-02-05,2,6,1,13.6666669845581,39.3333320617676,7,60,3,2,211,67,1,0,0,1,0,11,6
11662,2160,2011-02-05,3,6,1,29,28.5,2.5,60,3,2,211,67,1,0,0,1,0,11,6264.139122,7626.774783,10.
11663,2160,2011-02-05,4,6,1,39,20.6666660308838,0.333333343267441,60,3,2,211,67,1,0,0,1,0,1
11664,2160,2011-02-06,0,0,1,7,42,3.5,52.5,2,2,211,67,1,0,0,1,0,11,6264.139122,7626.774783,10.8
11665,2160,2011-02-06,1,0,1,3.33333325386047,39,17.6666660308838,60,3,2,211,67,1,0,0,1,0,11,
11666,2160,2011-02-06,2,0,1,24.3333339691162,29,6.66666650772095,60,3,2,211,67,1,0,0,1,0,11,
11667,2160,2011-02-06,3,0,1,20.6666660308838,27.6666660308838,11.6666669845581,60,3,2,21
11668,2160,2011-02-06,4,0,1,9.5,46,4.5,60,3,2,211,67,1,0,0,1,0,11,6264.139122,7626.774783,10.8
11669,2160,2011-02-07,0,1,0,12,34.5,9,55.5,2,2,211,67,1,0,0,1,0,11,6264.139122,7626.774783,10.
11670,2160,2011-02-07,1,1,0,9,30,21,60,1,2,211,67,1,0,0,1,0,11,6264.139122,7626.774783,10.892
11671,2160,2011-02-07,2,1,0,10.6666669845581,48,1.33333337306976,60,3,2,211,67,1,0,0,1,0,11,
11672,2160,2011-02-07,3,1,0,17,41.6666679382324,1.33333337306976,60,3,2,211,67,1,0,0,1,0,11,
11673,2160,2011-02-07,4,1,0,41.3333320617676,18.6666660308838,0,60,3,2,211,67,1,0,0,1,0,11,6
11674,2160,2011-02-08,0,2,0,20,22,5.5,47.5,2,2,211,67,1,0,0,1,0,11,6264.139122,7626.774783,10.
11675,2160,2011-02-08,1,2,0,17.6000003814697,35,7.40000009536743,60,3,2,211,67,1,0,0,1,0,11,
11676,2160,2011-02-08,2,2,0,26.3333339691162,30.6666660308838,3,60,3,2,211,67,1,0,0,1,0,11,6
11677,2160,2011-02-08,3,2,0,15,44,1,60,3,2,211,67,1,0,0,1,0,11,6264.139122,7626.774783,10.892
11678,2160,2011-02-08,4,2,0,44.3333320617676,15.6666669845581,0,60,3,2,211,67,1,0,0,1,0,11,6
11679,2163,2011-02-06,0,0,1,48,11,1,60,1,2,211,65,1,1,0,1,0,12,6428.213466,6382.16461,10.8735
11680,2163,2011-02-06,1,0,1,15.5,23,7,45.5,3,2,211,65,1,1,0,1,0,12,6428.213466,6382.16461,10.87
11681,2163,2011-02-06,2,0,1,28.6666660308838,30,1.33333337306976,60,3,2,211,65,1,1,0,1,0,12,
11682,2163,2011-02-06,3,0,1,21.6666660308838,22.3333339691162,16,60,3,2,211,65,1,1,0,1,0,12,
11683,2163,2011-02-06,4,0,1,38.6666679382324,17.3333339691162,4,60,3,2,211,65,1,1,0,1,0,12,6
11684,2163,2011-02-07,1,1,0,19,34,4.25,57.25,3,2,211,65,1,1,0,1,0,12,6428.213466,6382.16461,10
11685,2163,2011-02-07,2,1,0,31,26.8333339691162,2.16666674613953,60,3,2,211,65,1,1,0,1,0,12,
11686,2163,2011-02-07,3,1,0,32.3333320617676,23,4.66666650772095,60,3,2,211,65,1,1,0,1,0,12,
11687,2163,2011-02-07,4,1,0,42.3333320617676,16,1.66666662693024,60,3,2,211,65,1,1,0,1,0,12,
11688,2163,2011-02-08,1,2,0,28.6666660308838,24.3333339691162,2,55,3,2,211,65,1,1,0,1,0,12,6
11689,2163,2011-02-08,2,2,0,31,27,2,60,3,2,211,65,1,1,0,1,0,12,6428.213466,6382.16461,10.8735
11690,2163,2011-02-08,3,2,0,15.6666669845581,26.3333339691162,18,60,3,2,211,65,1,1,0,1,0,12,
11691,2163,2011-02-08,4,2,0,25,33.75,1.25,60,3,2,211,65,1,1,0,1,0,12,6428.213466,6382.16461,10
11692,2163,2011-02-09,0,3,0,4,28,9,41,1,2,211,65,1,1,0,1,0,12,6428.213466,6382.16461,10.87352
11693,2163,2011-02-09,1,3,0,5,36.3333320617676,18.6666660308838,60,3,2,211,65,1,1,0,1,0,12,6
11694,2163,2011-02-09,2,3,0,46.6666679382324,12.3333330154419,1,60,3,2,211,65,1,1,0,1,0,12,6
11695,2163,2011-02-09,3,3,0,14.3333330154419,42.3333320617676,3.33333325386047,60,3,2,21
11696,2163,2011-02-09,4,3,0,47,12.6666669845581,0.333333343267441,60,3,2,211,65,1,1,0,1,0,1
11697,2163,2011-02-10,0,4,0,11,34,11,56,1,2,211,65,1,1,0,1,0,12,6428.213466,6382.16461,10.873
11698,2163,2011-02-10,1,4,0,22,28,4.66666650772095,54.6666679382324,3,2,211,65,1,1,0,1,0,12,
11699,2163,2011-02-10,2,4,0,37,22,1,60,3,2,211,65,1,1,0,1,0,12,6428.213466,6382.16461,10.8735

11700,2163,2011-02-10,3,4,0,5.33333349227905,44.6666679382324,10,60,3,2,211,65,1,1,0,1,0,12,
11701,2163,2011-02-10,4,4,0,40.4000015258789,15.1999998092651,3.40000009536743,59,3,2,211
11702,2163,2011-02-11,1,5,0,12.5,41,6.5,60,2,2,211,65,1,1,0,1,0,12,6428.213466,6382.16461,10.87
11703,2163,2011-02-11,2,5,0,26,27.3333339691162,6.66666650772095,60,3,2,211,65,1,1,0,1,0,12,
11704,2163,2011-02-11,3,5,0,43.3333320617676,11,5.66666650772095,60,3,2,211,65,1,1,0,1,0,12,
11705,2163,2011-02-11,4,5,0,46.6666679382324,13.3333330154419,0,60,3,2,211,65,1,1,0,1,0,12,6
11706,2164,2011-02-05,0,6,1,25,29.5,1,55.5,2,2,211,83,1,0,1,0,0,7,6497.743938,6363.971652,10.94
11707,2164,2011-02-05,1,6,1,14.3333330154419,43.3333320617676,2.33333325386047,60,3,2,211
11708,2164,2011-02-05,2,6,1,29.6666660308838,27.3333339691162,3,60,3,2,211,83,1,0,1,0,0,7,64
11709,2164,2011-02-05,3,6,1,4.5,47.5,8,60,2,2,211,83,1,0,1,0,0,7,6497.743938,6363.971652,10.943
11710,2164,2011-02-05,4,6,1,50,10,0,60,1,2,211,83,1,0,1,0,0,7,6497.743938,6363.971652,10.94384
11711,2164,2011-02-06,3,0,1,19,33,8,60,1,2,211,83,1,0,1,0,0,7,6497.743938,6363.971652,10.94384
11712,2164,2011-02-06,4,0,1,40,20,0,60,3,2,211,83,1,0,1,0,0,7,6497.743938,6363.971652,10.94384
11713,2164,2011-02-11,0,5,0,21,37,2,60,1,2,211,83,1,0,1,0,0,7,6497.743938,6363.971652,10.94384
11714,2164,2011-02-11,1,5,0,2,53,5,60,1,2,211,83,1,0,1,0,0,7,6497.743938,6363.971652,10.943847
11715,2164,2011-02-12,0,6,1,36,20.5,2.5,59,2,2,211,83,1,0,1,0,0,7,6497.743938,6363.971652,10.94
11716,2164,2011-02-12,1,6,1,15,41.3333320617676,3.66666674613953,60,3,2,211,83,1,0,1,0,0,7,6
11717,2164,2011-02-12,2,6,1,33,25.6666660308838,1.33333337306976,60,3,2,211,83,1,0,1,0,0,7,6
11718,2164,2011-02-12,3,6,1,23.3333339691162,36,0.666666686534882,60,3,2,211,83,1,0,1,0,0,7,
11719,2164,2011-02-12,4,6,1,36.6666679382324,23,0.333333343267441,60,3,2,211,83,1,0,1,0,0,7,
11720,2164,2011-02-13,0,0,1,29.5,24,0.5,54,2,2,211,83,1,0,1,0,0,7,6497.743938,6363.971652,10.94
11721,2164,2011-02-13,1,0,1,11,47.3333320617676,1.66666662693024,60,3,2,211,83,1,0,1,0,0,7,6
11722,2164,2011-02-13,2,0,1,34.6666679382324,25.3333339691162,0,60,3,2,211,83,1,0,1,0,0,7,64
11723,2164,2011-02-13,3,0,1,41.6666679382324,18.3333339691162,0,60,3,2,211,83,1,0,1,0,0,7,64
11724,2164,2011-02-13,4,0,1,31,28.6666660308838,0.333333343267441,60,3,2,211,83,1,0,1,0,0,7,
11725,2164,2011-02-14,0,1,0,14.5,33,0.5,48,2,2,211,83,1,0,1,0,0,7,6497.743938,6363.971652,10.94
11726,2164,2011-02-14,1,1,0,13,45.3333320617676,1.66666662693024,60,3,2,211,83,1,0,1,0,0,7,6
11727,2164,2011-02-14,2,1,0,27,32.3333320617676,0.666666686534882,60,3,2,211,83,1,0,1,0,0,7,
11728,2164,2011-02-14,3,1,0,19.3333339691162,40.3333320617676,0.333333343267441,60,3,2,21
11729,2164,2011-02-14,4,1,0,36.6666679382324,23.3333339691162,0,60,3,2,211,83,1,0,1,0,0,7,64
11730,2164,2011-02-15,0,2,0,32.5,15,2,49.5,2,2,211,83,1,0,1,0,0,7,6497.743938,6363.971652,10.94
11731,2164,2011-02-15,1,2,0,18.5,40.5,1,60,2,2,211,83,1,0,1,0,0,7,6497.743938,6363.971652,10.94
11732,2164,2011-02-15,2,2,0,41,19,0,60,3,2,211,83,1,0,1,0,0,7,6497.743938,6363.971652,10.94384
11733,2164,2011-02-15,3,2,0,21.3333339691162,38.6666679382324,0,60,3,2,211,83,1,0,1,0,0,7,64
11734,2164,2011-02-15,4,2,0,34.6666679382324,25.3333339691162,0,60,3,2,211,83,1,0,1,0,0,7,64
11735,2164,2011-02-16,1,3,0,12,46.6666679382324,1.33333337306976,60,3,2,211,83,1,0,1,0,0,7,6
11736,2164,2011-02-16,2,3,0,27.3333339691162,32.6666679382324,0,60,3,2,211,83,1,0,1,0,0,7,64
11737,2164,2011-02-16,3,3,0,13.3333330154419,43.6666679382324,3,60,3,2,211,83,1,0,1,0,0,7,64
11738,2164,2011-02-16,4,3,0,22.5,36,1.5,60,2,2,211,83,1,0,1,0,0,7,6497.743938,6363.971652,10.94
11739,2173,2011-02-06,0,0,1,30,28,2,60,1,2,211,71,1,1,1,0,0,11,7915.454486,6944.300393,9.8432
11740,2173,2011-02-06,1,0,1,16.6666660308838,43,0.333333343267441,60,3,2,211,71,1,1,1,0,0,1
11741,2173,2011-02-06,2,0,1,22.6666660308838,36.6666679382324,0.666666686534882,60,3,2,2
11742,2173,2011-02-06,3,0,1,22.3333339691162,37.6666679382324,0,60,3,2,211,71,1,1,1,0,0,11,7
11743,2173,2011-02-07,0,1,0,8,22,9,39,1,2,211,71,1,1,1,0,0,11,7915.454486,6944.300393,9.843203
11744,2173,2011-02-07,1,1,0,13,42.6666679382324,4.33333349227905,60,3,2,211,71,1,1,1,0,0,11,
11745,2173,2011-02-07,2,1,0,17.6666660308838,42,0.333333343267441,60,3,2,211,71,1,1,1,0,0,1
11746,2173,2011-02-07,3,1,0,28.3333339691162,31,0.666666686534882,60,3,2,211,71,1,1,1,0,0,1
11747,2173,2011-02-07,4,1,0,15,29.6666660308838,0.666666686534882,45.3333320617676,3,2,2
11748,2173,2011-02-08,0,2,0,21,24,9,54,1,2,211,71,1,1,1,0,0,11,7915.454486,6944.300393,9.8432
11749,2173,2011-02-08,1,2,0,26,33.6666679382324,0.333333343267441,60,3,2,211,71,1,1,1,0,0,1

11750,2173,2011-02-08,2,2,0,26,32.6666679382324,1.33333337306976,60,3,2,211,71,1,1,1,0,0,11,
11751,2173,2011-02-08,3,2,0,17.6666660308838,41.6666679382324,0.666666686534882,60,3,2,2
11752,2173,2011-02-08,4,2,0,23.3999996185303,31.2000007629395,0.200000002980232,54.7999
11753,2173,2011-02-09,0,3,0,29,0.5,0,29.5,2,2,211,71,1,1,1,0,0,11,7915.454486,6944.300393,9.84
11754,2173,2011-02-09,1,3,0,25.6666660308838,34,0.333333343267441,60,3,2,211,71,1,1,1,0,0,1
11755,2173,2011-02-09,2,3,0,17.3333339691162,42,0.666666686534882,60,3,2,211,71,1,1,1,0,0,1
11756,2173,2011-02-09,3,3,0,16,43.3333320617676,0.666666686534882,60,3,2,211,71,1,1,1,0,0,1
11757,2173,2011-02-09,4,3,0,21.3333339691162,25.6666660308838,1,48,3,2,211,71,1,1,1,0,0,11,7
11758,2173,2011-02-10,0,4,0,32,19,0.5,51.5,2,2,211,71,1,1,1,0,0,11,7915.454486,6944.300393,9.8
11759,2173,2011-02-10,1,4,0,22.6666660308838,36.3333320617676,1,60,3,2,211,71,1,1,1,0,0,11,7
11760,2173,2011-02-10,2,4,0,22.3333339691162,37,0.666666686534882,60,3,2,211,71,1,1,1,0,0,1
11761,2173,2011-02-10,3,4,0,22.3333339691162,37,0.666666686534882,60,3,2,211,71,1,1,1,0,0,1
11762,2173,2011-02-10,4,4,0,21,28,0.5,49.5,2,2,211,71,1,1,1,0,0,11,7915.454486,6944.300393,9.8
11763,2173,2011-02-11,1,5,0,18.6666660308838,39.3333320617676,2,60,3,2,211,71,1,1,1,0,0,11,7
11764,2173,2011-02-11,2,5,0,32.6666679382324,24.6666660308838,2.66666674613953,60,3,2,21
11765,2173,2011-02-11,3,5,0,30.3333339691162,29.3333339691162,0.333333343267441,60,3,2,2
11766,2173,2011-02-11,4,5,0,18.5,34,0,52.5,2,2,211,71,1,1,1,0,0,11,7915.454486,6944.300393,9.8
11767,2183,2011-02-11,0,5,0,19,35,6,60,1,2,211,77,0,1,0,1,0,9,6215.884477,6022.890984,13.4208
11768,2183,2011-02-11,1,5,0,17,36,7,60,1,2,211,77,0,1,0,1,0,9,6215.884477,6022.890984,13.4208
11769,2183,2011-02-12,0,6,1,20.6666660308838,24,0.666666686534882,45.3333320617676,3,2,2
11770,2183,2011-02-12,1,6,1,36.5,16,7.5,60,2,2,211,77,0,1,0,1,0,9,6215.884477,6022.890984,13.4
11771,2183,2011-02-12,2,6,1,25.6666660308838,27,7.33333349227905,60,3,2,211,77,0,1,0,1,0,9,6
11772,2183,2011-02-12,3,6,1,21.3333339691162,27.3333339691162,11.3333330154419,60,3,2,21
11773,2183,2011-02-12,4,6,1,48.6666679382324,11,0.333333343267441,60,3,2,211,77,0,1,0,1,0,9,
11774,2183,2011-11-03,0,4,0,20,24.6666660308838,0.666666686534882,45.3333320617676,3,2,2
11775,2183,2011-11-03,1,4,0,15.75,36.25,8,60,3,2,211,77,0,1,0,1,0,9,6215.884477,6022.890984,13
11776,2183,2011-11-03,2,4,0,16.6666660308838,34.6666679382324,8.66666698455811,60,3,2,21
11777,2183,2011-11-03,3,4,0,43.3333320617676,16.6666660308838,0,60,3,2,211,77,0,1,0,1,0,9,62
11778,2183,2011-11-03,4,4,0,35.6666679382324,24.3333339691162,0,60,3,2,211,77,0,1,0,1,0,9,62
11779,2183,2011-11-04,0,5,0,11,28,1.33333337306976,40.3333320617676,3,2,211,77,0,1,0,1,0,9,6
11780,2183,2011-11-04,1,5,0,6.59999990463257,47.4000015258789,6,60,3,2,211,77,0,1,0,1,0,9,62
11781,2183,2011-11-04,2,5,0,24.6666660308838,33.6666679382324,1.66666662693024,60,3,2,21
11782,2183,2011-11-04,3,5,0,21.6666660308838,38.3333320617676,0,60,3,2,211,77,0,1,0,1,0,9,62
11783,2183,2011-11-04,4,5,0,48.6666679382324,11.3333330154419,0,60,3,2,211,77,0,1,0,1,0,9,62
11784,2183,2011-11-05,0,6,1,13.3333330154419,38,4.66666650772095,56,3,2,211,77,0,1,0,1,0,9,6
11785,2183,2011-11-05,1,6,1,18.3333339691162,39.6666679382324,2,60,3,2,211,77,0,1,0,1,0,9,62
11786,2183,2011-11-05,2,6,1,29,30.2000007629395,0.800000011920929,60,3,2,211,77,0,1,0,1,0,9,
11787,2183,2011-11-05,3,6,1,21.6666660308838,25.3333339691162,13,60,3,2,211,77,0,1,0,1,0,9,6
11788,2183,2011-11-05,4,6,1,47,13,0,60,3,2,211,77,0,1,0,1,0,9,6215.884477,6022.890984,13.4208
11789,2183,2011-11-06,0,0,1,9.33333301544189,30.6666660308838,1.33333337306976,41.33333
11790,2183,2011-11-06,1,0,1,13.6666669845581,37,9.33333301544189,60,3,2,211,77,0,1,0,1,0,9,6
11791,2183,2011-11-06,2,0,1,30,29,1,60,1,2,211,77,0,1,0,1,0,9,6215.884477,6022.890984,13.4208
11792,2183,2011-11-06,3,0,1,31.3333339691162,24,4.66666650772095,60,3,2,211,77,0,1,0,1,0,9,6
11793,2183,2011-11-06,4,0,1,41.6666679382324,18.3333339691162,0,60,3,2,211,77,0,1,0,1,0,9,62
11794,2183,2011-11-07,0,1,0,17,40.5,1,58.5,2,2,211,77,0,1,0,1,0,9,6215.884477,6022.890984,13.4
11795,2183,2011-11-07,1,1,0,19,33,8,60,3,2,211,77,0,1,0,1,0,9,6215.884477,6022.890984,13.4208
11796,2183,2011-11-07,2,1,0,21.3999996185303,34.5999984741211,4,60,3,2,211,77,0,1,0,1,0,9,62
11797,2183,2011-11-07,3,1,0,42,16.6666660308838,1.33333337306976,60,3,2,211,77,0,1,0,1,0,9,6
11798,2183,2011-11-07,4,1,0,43.3333320617676,16.6666660308838,0,60,3,2,211,77,0,1,0,1,0,9,62
11799,2183,2011-11-08,2,2,0,26,27,7,60,1,2,211,77,0,1,0,1,0,9,6215.884477,6022.890984,13.4208

11800,2183,2011-11-08,3,2,0,26.3333339691162,31.6666660308838,2,60,3,2,211,77,0,1,0,1,0,9,62
11801,2183,2011-11-08,4,2,0,40.6666679382324,16,3.33333325386047,60,3,2,211,77,0,1,0,1,0,9,6
11802,2186,2011-11-01,0,2,0,2,37,1.5,40.5,2,2,211,66,1,0,0,0,0,10,6153.166165,6268.102285,12.9
11803,2186,2011-11-01,1,2,0,2,55.6666679382324,2.33333325386047,60,3,2,211,66,1,0,0,0,0,10,6
11804,2186,2011-11-01,2,2,0,15.3333330154419,41,3.66666674613953,60,3,2,211,66,1,0,0,0,0,10,
11805,2186,2011-11-01,3,2,0,11,47.3333320617676,1.66666662693024,60,3,2,211,66,1,0,0,0,0,10,
11806,2186,2011-11-01,4,2,0,13.5,28.75,0.25,42.5,3,2,211,66,1,0,0,0,0,10,6153.166165,6268.1022
11807,2186,2011-11-02,0,3,0,3,44.5,6.5,54,2,2,211,66,1,0,0,0,0,10,6153.166165,6268.102285,12.9
11808,2186,2011-11-02,1,3,0,9.83333301544189,43.8333320617676,2.83333325386047,56.5,3,2,2
11809,2186,2011-11-02,2,3,0,13.6666669845581,45,1.33333337306976,60,3,2,211,66,1,0,0,0,0,10,
11810,2186,2011-11-02,3,3,0,8.66666698455811,48.3333320617676,3,60,3,2,211,66,1,0,0,0,0,10,6
11811,2186,2011-11-02,4,3,0,12,23.5,1.5,37,2,2,211,66,1,0,0,0,0,10,6153.166165,6268.102285,12.
11812,2186,2011-11-03,0,4,0,0.5,41,6.5,48,2,2,211,66,1,0,0,0,0,10,6153.166165,6268.102285,12.9
11813,2186,2011-11-03,1,4,0,17.6666660308838,31.3333339691162,11,60,3,2,211,66,1,0,0,0,0,10,
11814,2186,2011-11-03,2,4,0,23.6666660308838,35.3333320617676,1,60,3,2,211,66,1,0,0,0,0,10,6
11815,2186,2011-11-03,3,4,0,17.6666660308838,36.6666679382324,5.66666650772095,60,3,2,21
11816,2186,2011-11-03,4,4,0,24.3333339691162,34.6666679382324,1,60,3,2,211,66,1,0,0,0,0,10,6
11817,2186,2011-11-04,0,5,0,1.5,27.5,2,31,2,2,211,66,1,0,0,0,0,10,6153.166165,6268.102285,12.9
11818,2186,2011-11-04,1,5,0,8,50,2,60,3,2,211,66,1,0,0,0,0,10,6153.166165,6268.102285,12.9843
11819,2186,2011-11-04,2,5,0,20.1666660308838,37.1666679382324,2.66666674613953,60,3,2,21
11820,2186,2011-11-04,3,5,0,21.3333339691162,32.3333320617676,6.33333349227905,60,3,2,21
11821,2186,2011-11-04,4,5,0,11.5,24,0.5,36,2,2,211,66,1,0,0,0,0,10,6153.166165,6268.102285,12.
11822,2186,2011-11-05,0,6,1,3,47,4,54,1,2,211,66,1,0,0,0,0,10,6153.166165,6268.102285,12.9843
11823,2186,2011-11-05,1,6,1,14.6666669845581,41,4.33333349227905,60,3,2,211,66,1,0,0,0,0,10,
11824,2186,2011-11-05,2,6,1,8.66666698455811,47,4.33333349227905,60,3,2,211,66,1,0,0,0,0,10,
11825,2186,2011-11-05,3,6,1,20.1666660308838,27,12.8333330154419,60,3,2,211,66,1,0,0,0,0,10,
11826,2186,2011-11-05,4,6,1,19.5,27,1,47.5,2,2,211,66,1,0,0,0,0,10,6153.166165,6268.102285,12.
11827,2186,2011-11-08,0,2,0,0,56,4,60,1,2,211,66,1,0,0,0,0,10,6153.166165,6268.102285,12.9843
11828,2186,2011-11-08,1,2,0,8,47.6666679382324,4.33333349227905,60,3,2,211,66,1,0,0,0,0,10,6
11829,2186,2011-11-08,2,2,0,8.66666698455811,45.6666679382324,5.66666650772095,60,3,2,21
11830,2186,2011-11-08,3,2,0,31,28,1,60,1,2,211,66,1,0,0,0,0,10,6153.166165,6268.102285,12.984
11831,2186,2011-11-09,0,3,0,3,45,2,50,1,2,211,66,1,0,0,0,0,10,6153.166165,6268.102285,12.9843
11832,2186,2011-11-09,1,3,0,0.666666686534882,54.6666679382324,4.66666650772095,60,3,2,2
11833,2186,2011-11-09,2,3,0,13.3333330154419,43.3333320617676,3.33333325386047,60,3,2,21
11834,2186,2011-11-09,3,3,0,9.60000038146973,47,3.40000009536743,60,3,2,211,66,1,0,0,0,0,10,
11835,2186,2011-11-09,4,3,0,22,36.25,0.5,58.75,3,2,211,66,1,0,0,0,0,10,6153.166165,6268.102285
11836,2186,2011-11-16,4,3,0,40,14.5,1.5,56,2,2,211,66,1,0,0,0,0,10,6153.166165,6268.102285,12.
11837,2188,2011-10-29,0,6,1,32.6666679382324,27.3333339691162,0,60,3,2,211,79,0,0,1,0,0,10,6
11838,2188,2011-10-29,1,6,1,31.6666660308838,28.3333339691162,0,60,3,2,211,79,0,0,1,0,0,10,6
11839,2188,2011-10-29,2,6,1,34.6666679382324,25,0.333333343267441,60,3,2,211,79,0,0,1,0,0,1
11840,2188,2011-10-29,3,6,1,46,14,0,60,3,2,211,79,0,0,1,0,0,10,6054.643207,6412.502755,11.579
11841,2188,2011-10-29,4,6,1,46,14,0,60,3,2,211,79,0,0,1,0,0,10,6054.643207,6412.502755,11.579
11842,2188,2011-10-30,0,0,1,8,38,14,60,3,2,211,79,0,0,1,0,0,10,6054.643207,6412.502755,11.579
11843,2188,2011-10-30,1,0,1,36.3333320617676,22.3333339691162,1.33333337306976,60,3,2,21
11844,2188,2011-10-30,2,0,1,39.3333320617676,19.6666660308838,1,60,3,2,211,79,0,0,1,0,0,10,6
11845,2188,2011-10-30,3,0,1,32.6666679382324,26.6666660308838,0.666666686534882,60,3,2,2
11846,2188,2011-10-30,4,0,1,49.7999992370605,10.1999998092651,0,60,3,2,211,79,0,0,1,0,0,10,6
11847,2188,2011-10-31,0,1,0,13.5,35,3.5,52,3,2,211,79,0,0,1,0,0,10,6054.643207,6412.502755,11.
11848,2188,2011-10-31,1,1,0,26.3333339691162,32.6666679382324,1,60,3,2,211,79,0,0,1,0,0,10,6
11849,2188,2011-10-31,2,1,0,52.6666679382324,7.33333349227905,0,60,3,2,211,79,0,0,1,0,0,10,6

11850,2188,2011-10-31,3,1,0,36.6666679382324,23,0.333333343267441,60,3,2,211,79,0,0,1,0,0,10
11851,2188,2011-10-31,4,1,0,49.3333320617676,10.6666669845581,0,60,3,2,211,79,0,0,1,0,0,10,6
11852,2188,2011-11-01,0,2,0,19.75,24.25,4,48,3,2,211,79,0,0,1,0,0,10,6054.643207,6412.502755,1
11853,2188,2011-11-01,1,2,0,22.3333339691162,34,3.66666674613953,60,3,2,211,79,0,0,1,0,0,10,
11854,2188,2011-11-01,2,2,0,43,17,0,60,3,2,211,79,0,0,1,0,0,10,6054.643207,6412.502755,11.5793
11855,2188,2011-11-01,3,2,0,33.3333320617676,26.3333339691162,0.333333343267441,60,3,2,21
11856,2188,2011-11-01,4,2,0,54.3333320617676,5.66666650772095,0,60,3,2,211,79,0,0,1,0,0,10,6
11857,2188,2011-11-02,0,3,0,34,26,0,60,1,2,211,79,0,0,1,0,0,10,6054.643207,6412.502755,11.5793
11858,2188,2011-11-02,1,3,0,32.6666679382324,27.3333339691162,0,60,3,2,211,79,0,0,1,0,0,10,6
11859,2188,2011-11-02,2,3,0,30,27.6666660308838,2.33333325386047,60,3,2,211,79,0,0,1,0,0,10,
11860,2188,2011-11-02,3,3,0,26,32,2,60,3,2,211,79,0,0,1,0,0,10,6054.643207,6412.502755,11.5793
11861,2188,2011-11-02,4,3,0,47,12.3333330154419,0.666666686534882,60,3,2,211,79,0,0,1,0,0,10
11862,2188,2011-11-16,0,3,0,36,23,0,59,2,2,211,79,0,0,1,0,0,10,6054.643207,6412.502755,11.5793
11863,2188,2011-11-16,1,3,0,24,36,0,60,3,2,211,79,0,0,1,0,0,10,6054.643207,6412.502755,11.5793
11864,2188,2011-11-16,2,3,0,31.3333339691162,17.3333339691162,11.3333330154419,60,3,2,211
11865,2188,2011-11-16,3,3,0,34,25,1,60,3,2,211,79,0,0,1,0,0,10,6054.643207,6412.502755,11.5793
11866,2188,2011-11-16,4,3,0,44,16,0,60,1,2,211,79,0,0,1,0,0,10,6054.643207,6412.502755,11.5793
11867,2196,2011-10-29,0,6,1,8,4,0,12,1,2,211,69,0,1,0,1,0,12,6418.681482,7157.706632,12.380809
11868,2196,2011-10-29,1,6,1,30.25,24,0.5,54.75,3,2,211,69,0,1,0,1,0,12,6418.681482,7157.706632
11869,2196,2011-10-29,2,6,1,25,18,17,60,3,2,211,69,0,1,0,1,0,12,6418.681482,7157.706632,12.380
11870,2196,2011-10-29,3,6,1,35.6666679382324,22.3333339691162,2,60,3,2,211,69,0,1,0,1,0,12,6
11871,2196,2011-10-29,4,6,1,48.75,11.25,0,60,3,2,211,69,0,1,0,1,0,12,6418.681482,7157.706632,1
11872,2196,2011-10-30,0,0,1,0,7,0,7,1,2,211,69,0,1,0,1,0,12,6418.681482,7157.706632,12.3808098
11873,2196,2011-10-30,1,0,1,28,10.6000003814697,21.3999996185303,60,3,2,211,69,0,1,0,1,0,12,
11874,2196,2011-10-30,2,0,1,26.5,16,17.5,60,3,2,211,69,0,1,0,1,0,12,6418.681482,7157.706632,12
11875,2196,2011-10-30,3,0,1,43,15.3333330154419,1.66666662693024,60,3,2,211,69,0,1,0,1,0,12,
11876,2196,2011-10-30,4,0,1,52.6666679382324,7.33333349227905,0,60,3,2,211,69,0,1,0,1,0,12,6
11877,2196,2011-10-31,0,1,0,2,7,4,13,1,2,211,69,0,1,0,1,0,12,6418.681482,7157.706632,12.380809
11878,2196,2011-10-31,1,1,0,21.6666660308838,14.3333330154419,24,60,3,2,211,69,0,1,0,1,0,12,
11879,2196,2011-10-31,2,1,0,25.25,12.75,12.25,50.25,3,2,211,69,0,1,0,1,0,12,6418.681482,7157.70
11880,2196,2011-10-31,3,1,0,25.5,16,11,52.5,3,2,211,69,0,1,0,1,0,12,6418.681482,7157.706632,12
11881,2196,2011-10-31,4,1,0,32.3333320617676,26.3333339691162,1.33333337306976,60,3,2,211
11882,2196,2011-11-01,3,2,0,39,21,0,60,2,2,211,69,0,1,0,1,0,12,6418.681482,7157.706632,12.3808
11883,2196,2011-11-01,4,2,0,49.3333320617676,10.6666669845581,0,60,3,2,211,69,0,1,0,1,0,12,6
11884,2196,2011-11-02,0,3,0,28,32,0,60,1,2,211,69,0,1,0,1,0,12,6418.681482,7157.706632,12.3808
11885,2196,2011-11-02,1,3,0,22.3333339691162,37.6666679382324,0,60,3,2,211,69,0,1,0,1,0,12,6
11886,2196,2011-11-03,0,4,0,10,32,1,43,1,2,211,69,0,1,0,1,0,12,6418.681482,7157.706632,12.3808
11887,2196,2011-11-03,1,4,0,23.3333339691162,36.3333320617676,0.333333343267441,60,3,2,21
11888,2196,2011-11-03,2,4,0,32.3333320617676,22.5,5.16666650772095,60,3,2,211,69,0,1,0,1,0,1
11889,2196,2011-11-03,3,4,0,37.6666679382324,22,0.333333343267441,60,3,2,211,69,0,1,0,1,0,12
11890,2196,2011-11-03,4,4,0,39,20.6666660308838,0.333333343267441,60,3,2,211,69,0,1,0,1,0,12
11891,2196,2011-11-04,0,5,0,19,15,6,40,1,2,211,69,0,1,0,1,0,12,6418.681482,7157.706632,12.3808
11892,2196,2011-11-04,1,5,0,39,15.3333330154419,5.66666650772095,60,3,2,211,69,0,1,0,1,0,12,
11893,2196,2011-11-04,2,5,0,18.3333339691162,20,21.6666660308838,60,3,2,211,69,0,1,0,1,0,12,
11894,2196,2011-11-04,3,5,0,43,16.8333339691162,0.16666667163372,60,3,2,211,69,0,1,0,1,0,12,
11895,2196,2011-11-04,4,5,0,40.3333320617676,19.3333339691162,0.333333343267441,60,3,2,21
11896,2196,2011-11-05,0,6,1,24,9,0,33,1,2,211,69,0,1,0,1,0,12,6418.681482,7157.706632,12.38080
11897,2196,2011-11-05,1,6,1,33.3333320617676,9.33333301544189,17.3333339691162,60,3,2,211
11898,2196,2011-11-05,2,6,1,23,23,14,60,3,2,211,69,0,1,0,1,0,12,6418.681482,7157.706632,12.380
11899,2196,2011-11-05,3,6,1,34.6666679382324,14.3333330154419,11,60,3,2,211,69,0,1,0,1,0,12,

11900,2196,2011-11-05,4,6,1,48.4000015258789,11.6000003814697,0,60,3,2,211,69,0,1,0,1,0,12,6
11901,2203,2011-10-28,0,5,0,0,15,0,15,1,2,211,76,0,0,1,1,0,11,6563.588314,6943.719838,11.3882
11902,2203,2011-10-28,1,5,0,17.6666660308838,38,4.33333349227905,60,3,2,211,76,0,0,1,1,0,11,
11903,2203,2011-10-28,2,5,0,8.60000038146973,49,2.40000009536743,60,3,2,211,76,0,0,1,1,0,11,
11904,2203,2011-10-28,3,5,0,25.3333339691162,33,1.66666662693024,60,3,2,211,76,0,0,1,1,0,11,
11905,2203,2011-10-28,4,5,0,8.66666698455811,51.3333320617676,0,60,3,2,211,76,0,0,1,1,0,11,6
11906,2203,2011-10-29,0,6,1,9,36,2,47,1,2,211,76,0,0,1,1,0,11,6563.588314,6943.719838,11.3882
11907,2203,2011-10-29,1,6,1,17.6666660308838,40.6666679382324,1.66666662693024,60,3,2,21
11908,2203,2011-10-29,2,6,1,9.33333301544189,48,2.66666674613953,60,3,2,211,76,0,0,1,1,0,11,
11909,2203,2011-10-29,3,6,1,11.1666669845581,47.1666679382324,1.66666662693024,60,3,2,21
11910,2203,2011-10-29,4,6,1,9.66666698455811,46.6666679382324,3.66666674613953,60,3,2,21
11911,2203,2011-10-30,4,0,1,30,29.6666660308838,0.333333343267441,60,3,2,211,76,0,0,1,1,0,1
11912,2203,2011-11-01,0,2,0,11,47,2,60,1,2,211,76,0,0,1,1,0,11,6563.588314,6943.719838,11.388
11913,2203,2011-11-01,1,2,0,14,45,1,60,3,2,211,76,0,0,1,1,0,11,6563.588314,6943.719838,11.388
11914,2203,2011-11-01,2,2,0,7.33333349227905,47.6666679382324,5,60,3,2,211,76,0,0,1,1,0,11,6
11915,2203,2011-11-01,3,2,0,12,44,4,60,3,2,211,76,0,0,1,1,0,11,6563.588314,6943.719838,11.388
11916,2203,2011-11-02,0,3,0,2.5,30,6,38.5,2,2,211,76,0,0,1,1,0,11,6563.588314,6943.719838,11.3
11917,2203,2011-11-02,1,3,0,5,48.6666679382324,6.33333349227905,60,3,2,211,76,0,0,1,1,0,11,6
11918,2203,2011-11-02,2,3,0,15,43.6666679382324,1.33333337306976,60,3,2,211,76,0,0,1,1,0,11,
11919,2203,2011-11-02,3,3,0,14.3333330154419,43.6666679382324,2,60,3,2,211,76,0,0,1,1,0,11,6
11920,2203,2011-11-02,4,3,0,14,42.2000007629395,3.79999995231628,60,3,2,211,76,0,0,1,1,0,11,
11921,2203,2011-11-03,0,4,0,3,3,0,6,1,2,211,76,0,0,1,1,0,11,6563.588314,6943.719838,11.388222
11922,2203,2011-11-03,1,4,0,12.6000003814697,43.4000015258789,2.20000004768372,58.20000
11923,2203,2011-11-03,2,4,0,8.66666698455811,49.6666679382324,1.66666662693024,60,3,2,21
11924,2203,2011-11-03,3,4,0,8,49,3,60,3,2,211,76,0,0,1,1,0,11,6563.588314,6943.719838,11.3882
11925,2203,2011-11-03,4,4,0,29.25,30.75,0,60,3,2,211,76,0,0,1,1,0,11,6563.588314,6943.719838,1
11926,2203,2011-11-10,1,4,0,4.33333349227905,45.3333320617676,8.66666698455811,58.33333
11927,2203,2011-11-10,2,4,0,12,46.3333320617676,1.66666662693024,60,3,2,211,76,0,0,1,1,0,11,
11928,2203,2011-11-10,3,4,0,19,37.3333320617676,3.66666674613953,60,3,2,211,76,0,0,1,1,0,11,
11929,2203,2011-11-10,4,4,0,20,38,2,60,3,2,211,76,0,0,1,1,0,11,6563.588314,6943.719838,11.388
11930,2203,2011-11-16,1,3,0,6.75,43.75,4,54.5,3,2,211,76,0,0,1,1,0,11,6563.588314,6943.719838,1
11931,2203,2011-11-16,2,3,0,7,50.75,2.25,60,3,2,211,76,0,0,1,1,0,11,6563.588314,6943.719838,11
11932,2203,2011-11-16,3,3,0,1.66666662693024,56.3333320617676,2,60,3,2,211,76,0,0,1,1,0,11,6
11933,2203,2011-11-16,4,3,0,16.3333339691162,39.6666679382324,4,60,3,2,211,76,0,0,1,1,0,11,6
11934,2204,2011-10-30,0,0,1,17.5,21,0.5,39,2,2,211,72,1,1,0,1,0,12,6515.435413,7055.317455,11.1
11935,2204,2011-10-30,1,0,1,24.3333339691162,28.6666660308838,7,60,3,2,211,72,1,1,0,1,0,12,6
11936,2204,2011-10-30,2,0,1,18,42,0,60,3,2,211,72,1,1,0,1,0,12,6515.435413,7055.317455,11.1047
11937,2204,2011-10-30,3,0,1,8,51.6666679382324,0.333333343267441,60,3,2,211,72,1,1,0,1,0,12,
11938,2204,2011-10-30,4,0,1,36.6666679382324,22,1.33333337306976,60,3,2,211,72,1,1,0,1,0,12,
11939,2204,2011-10-31,0,1,0,23.3333339691162,24,1.66666662693024,49,3,2,211,72,1,1,0,1,0,12,
11940,2204,2011-10-31,1,1,0,14.6000003814697,43.7999992370605,1.60000002384186,60,3,2,21
11941,2204,2011-10-31,2,1,0,28.3333339691162,30.6666660308838,1,60,3,2,211,72,1,1,0,1,0,12,6
11942,2204,2011-10-31,3,1,0,7,50,3,60,3,2,211,72,1,1,0,1,0,12,6515.435413,7055.317455,11.1047
11943,2204,2011-10-31,4,1,0,38.3333320617676,20.3333339691162,1.33333337306976,60,3,2,21
11944,2204,2011-11-01,0,2,0,15,20,1,36,2,2,211,72,1,1,0,1,0,12,6515.435413,7055.317455,11.1047
11945,2204,2011-11-01,1,2,0,10,48.5,1.5,60,3,2,211,72,1,1,0,1,0,12,6515.435413,7055.317455,11.1
11946,2204,2011-11-01,2,2,0,30.2000007629395,29.7999992370605,0,60,3,2,211,72,1,1,0,1,0,12,6
11947,2204,2011-11-01,3,2,0,11.6666669845581,48.3333320617676,0,60,3,2,211,72,1,1,0,1,0,12,6
11948,2204,2011-11-01,4,2,0,45,15,0,60,3,2,211,72,1,1,0,1,0,12,6515.435413,7055.317455,11.1047
11949,2204,2011-11-02,0,3,0,17.5,15,0.5,33,2,2,211,72,1,1,0,1,0,12,6515.435413,7055.317455,11.1

11950,2204,2011-11-02,1,3,0,17.6666660308838,39,3.33333325386047,60,3,2,211,72,1,1,0,1,0,12,
11951,2204,2011-11-02,2,3,0,36.25,23.5,0.25,60,3,2,211,72,1,1,0,1,0,12,6515.435413,7055.317455
11952,2204,2011-11-02,3,3,0,8,50.4000015258789,1.60000002384186,60,3,2,211,72,1,1,0,1,0,12,6
11953,2204,2011-11-02,4,3,0,29.6666660308838,30.3333339691162,0,60,3,2,211,72,1,1,0,1,0,12,6
11954,2204,2011-11-03,0,4,0,19,19,0.5,38.5,2,2,211,72,1,1,0,1,0,12,6515.435413,7055.317455,11.1
11955,2204,2011-11-03,1,4,0,17.3333339691162,33,9.66666698455811,60,3,2,211,72,1,1,0,1,0,12,
11956,2204,2011-11-03,2,4,0,44,16,0,60,3,2,211,72,1,1,0,1,0,12,6515.435413,7055.317455,11.104
11957,2204,2011-11-03,3,4,0,4,54,2,60,1,2,211,72,1,1,0,1,0,12,6515.435413,7055.317455,11.1047
11958,2204,2011-11-03,4,4,0,27.6666660308838,32.3333320617676,0,60,3,2,211,72,1,1,0,1,0,12,6
11959,2204,2011-11-04,0,5,0,36.6666679382324,17.6666660308838,0.666666686534882,55,3,2,2
11960,2204,2011-11-04,1,5,0,26.3333339691162,33.3333320617676,0.333333343267441,60,3,2,2
11961,2204,2011-11-04,2,5,0,35.3333320617676,24.6666660308838,0,60,3,2,211,72,1,1,0,1,0,12,6
11962,2204,2011-11-04,3,5,0,31.75,28.25,0,60,3,2,211,72,1,1,0,1,0,12,6515.435413,7055.317455,1
11963,2204,2011-11-04,4,5,0,27.6666660308838,32.3333320617676,0,60,3,2,211,72,1,1,0,1,0,12,6
11964,2204,2011-11-05,3,6,1,10,48.5,1.5,60,2,2,211,72,1,1,0,1,0,12,6515.435413,7055.317455,11.1
11965,2204,2011-11-05,4,6,1,40.6666679382324,19.3333339691162,0,60,3,2,211,72,1,1,0,1,0,12,6
11966,2212,2011-10-29,0,6,1,36.6666679382324,23.3333339691162,0,60,3,2,211,82,0,0,0,0,0,6,65
11967,2212,2011-10-29,1,6,1,34,26,0,60,3,2,211,82,0,0,0,0,0,6,6560.469415,6833.273684,10.9833
11968,2212,2011-10-29,2,6,1,19,40.3333320617676,0.666666686534882,60,3,2,211,82,0,0,0,0,0,6,
11969,2212,2011-10-29,3,6,1,27.3333339691162,29.6666660308838,3,60,3,2,211,82,0,0,0,0,0,6,65
11970,2212,2011-10-29,4,6,1,37.25,22.75,0,60,3,2,211,82,0,0,0,0,0,6,6560.469415,6833.273684,10
11971,2212,2011-10-30,0,0,1,37.6666679382324,20.3333339691162,0.333333343267441,58.3333
11972,2212,2011-10-30,1,0,1,26.3333339691162,32.3333320617676,1.33333337306976,60,3,2,21
11973,2212,2011-10-30,2,0,1,38,21,1,60,3,2,211,82,0,0,0,0,0,6,6560.469415,6833.273684,10.9833
11974,2212,2011-10-30,3,0,1,31,27,2,60,3,2,211,82,0,0,0,0,0,6,6560.469415,6833.273684,10.9833
11975,2212,2011-10-30,4,0,1,42,16.75,0,58.75,3,2,211,82,0,0,0,0,0,6,6560.469415,6833.273684,10
11976,2212,2011-10-31,0,1,0,41.6666679382324,7,0.333333343267441,49,3,2,211,82,0,0,0,0,0,6,6
11977,2212,2011-10-31,1,1,0,40,20,0,60,3,2,211,82,0,0,0,0,0,6,6560.469415,6833.273684,10.9833
11978,2212,2011-10-31,2,1,0,54.6666679382324,5.33333349227905,0,60,3,2,211,82,0,0,0,0,0,6,65
11979,2212,2011-10-31,3,1,0,50.3333320617676,9.66666698455811,0,60,3,2,211,82,0,0,0,0,0,6,65
11980,2212,2011-10-31,4,1,0,46.75,13.25,0,60,3,2,211,82,0,0,0,0,0,6,6560.469415,6833.273684,10
11981,2212,2011-11-03,0,4,0,42,16.6666660308838,1.33333337306976,60,3,2,211,82,0,0,0,0,0,6,6
11982,2212,2011-11-03,1,4,0,32,27.6666660308838,0.333333343267441,60,3,2,211,82,0,0,0,0,0,6,
11983,2212,2011-11-03,2,4,0,39.6666679382324,20,0.333333343267441,60,3,2,211,82,0,0,0,0,0,6,
11984,2212,2011-11-03,3,4,0,35.6666679382324,23,1.33333337306976,60,3,2,211,82,0,0,0,0,0,6,6
11985,2212,2011-11-03,4,4,0,43.3333320617676,16,0.666666686534882,60,3,2,211,82,0,0,0,0,0,6,
11986,2212,2011-11-04,0,5,0,29,17,4.66666650772095,50.6666679382324,3,2,211,82,0,0,0,0,0,6,6
11987,2212,2011-11-04,1,5,0,29.3333339691162,30,0.666666686534882,60,3,2,211,82,0,0,0,0,0,6,
11988,2212,2011-11-04,2,5,0,44.6666679382324,15.3333330154419,0,60,3,2,211,82,0,0,0,0,0,6,65
11989,2212,2011-11-04,3,5,0,49,10.3333330154419,0.666666686534882,60,3,2,211,82,0,0,0,0,0,6,
11990,2212,2011-11-04,4,5,0,52,8,0,60,3,2,211,82,0,0,0,0,0,6,6560.469415,6833.273684,10.98335
11991,2212,2011-11-05,0,6,1,36,19,5,60,1,2,211,82,0,0,0,0,0,6,6560.469415,6833.273684,10.9833
11992,2212,2011-11-05,1,6,1,25.6666660308838,27,7.33333349227905,60,3,2,211,82,0,0,0,0,0,6,6
11993,2212,2011-11-05,2,6,1,37.6666679382324,21,1.33333337306976,60,3,2,211,82,0,0,0,0,0,6,6
11994,2212,2011-11-05,3,6,1,41.3333320617676,18.6666660308838,0,60,3,2,211,82,0,0,0,0,0,6,65
11995,2212,2011-11-05,4,6,1,37,23,0,60,1,2,211,82,0,0,0,0,0,6,6560.469415,6833.273684,10.9833
11996,2212,2011-11-16,0,3,0,31.3333339691162,28,0.666666686534882,60,3,2,211,82,0,0,0,0,0,6,
11997,2212,2011-11-16,1,3,0,16.6666660308838,41,2.33333325386047,60,3,2,211,82,0,0,0,0,0,6,6
11998,2212,2011-11-16,2,3,0,13,45,2,60,3,2,211,82,0,0,0,0,0,6,6560.469415,6833.273684,10.9833
11999,2212,2011-11-16,3,3,0,42.3333320617676,17,0.666666686534882,60,3,2,211,82,0,0,0,0,0,6,

12000,2212,2011-11-16,4,3,0,42.5,17.5,0,60,2,2,211,82,0,0,0,0,0,6,6560.469415,6833.273684,10.98
12001,2213,2011-11-05,0,6,1,14,45.5,0.5,60,2,2,211,74,1,0,0,0,0,12,6560.469415,6833.273684,10.
12002,2213,2011-11-05,1,6,1,18.6666660308838,36,5.33333349227905,60,3,2,211,74,1,0,0,0,0,12,
12003,2213,2011-11-05,2,6,1,23.3333339691162,35,1.66666662693024,60,3,2,211,74,1,0,0,0,0,12,
12004,2213,2011-11-05,3,6,1,14.6666669845581,41.6666679382324,3.66666674613953,60,3,2,21
12005,2213,2011-11-05,4,6,1,25.6666660308838,34.3333320617676,0,60,3,2,211,74,1,0,0,0,0,12,6
12006,2213,2011-11-06,0,0,1,11.3333330154419,47.6666679382324,1,60,3,2,211,74,1,0,0,0,0,12,6
12007,2213,2011-11-06,1,0,1,21.6666660308838,38.3333320617676,0,60,3,2,211,74,1,0,0,0,0,12,6
12008,2213,2011-11-06,2,0,1,26,33.3333320617676,0.666666686534882,60,3,2,211,74,1,0,0,0,0,1
12009,2213,2011-11-06,3,0,1,17.6666660308838,41.6666679382324,0.666666686534882,60,3,2,2
12010,2213,2011-11-06,4,0,1,29,31,0,60,3,2,211,74,1,0,0,0,0,12,6560.469415,6833.273684,10.983
12011,2213,2011-11-07,0,1,0,8.66666698455811,49.3333320617676,0.666666686534882,58.6666
12012,2213,2011-11-07,1,1,0,6.33333349227905,53,0.666666686534882,60,3,2,211,74,1,0,0,0,0,1
12013,2213,2011-11-07,2,1,0,16.3333339691162,43.3333320617676,0.333333343267441,60,3,2,2
12014,2213,2011-11-07,3,1,0,17.3333339691162,42.6666679382324,0,60,3,2,211,74,1,0,0,0,0,12,6
12015,2213,2011-11-07,4,1,0,38.6666679382324,19,0,57.6666679382324,3,2,211,74,1,0,0,0,0,12,6
12016,2213,2011-11-08,0,2,0,5,54,1,60,3,2,211,74,1,0,0,0,0,12,6560.469415,6833.273684,10.9833
12017,2213,2011-11-08,1,2,0,5.66666650772095,54.3333320617676,0,60,3,2,211,74,1,0,0,0,0,12,6
12018,2213,2011-11-08,2,2,0,26,33.6666679382324,0.333333343267441,60,3,2,211,74,1,0,0,0,0,1
12019,2213,2011-11-08,3,2,0,12.6666669845581,45,2.33333325386047,60,3,2,211,74,1,0,0,0,0,12,
12020,2213,2011-11-08,4,2,0,34,26,0,60,3,2,211,74,1,0,0,0,0,12,6560.469415,6833.273684,10.983
12021,2213,2011-11-09,0,3,0,15.6666669845581,39,5.33333349227905,60,3,2,211,74,1,0,0,0,0,12,
12022,2213,2011-11-09,1,3,0,8,50.3333320617676,1.66666662693024,60,3,2,211,74,1,0,0,0,0,12,6
12023,2213,2011-11-09,2,3,0,23.6666660308838,31.3333339691162,5,60,3,2,211,74,1,0,0,0,0,12,6
12024,2213,2011-11-09,3,3,0,17.6666660308838,42.3333320617676,0,60,3,2,211,74,1,0,0,0,0,12,6
12025,2213,2011-11-09,4,3,0,42.6666679382324,17.3333339691162,0,60,3,2,211,74,1,0,0,0,0,12,6
12026,2213,2011-11-23,0,3,0,15.6666669845581,37,5,57.6666679382324,3,2,211,74,1,0,0,0,0,12,6
12027,2213,2011-11-23,1,3,0,6.33333349227905,50.6666679382324,3,60,3,2,211,74,1,0,0,0,0,12,6
12028,2213,2011-11-23,2,3,0,24.6666660308838,33,2.33333325386047,60,3,2,211,74,1,0,0,0,0,12,
12029,2213,2011-11-23,3,3,0,31,26.6666660308838,2.33333325386047,60,3,2,211,74,1,0,0,0,0,12,
12030,2213,2011-11-23,4,3,0,42,17.3333339691162,0.666666686534882,60,3,2,211,74,1,0,0,0,0,1
12031,2213,2011-11-24,0,4,0,12,37,11,60,1,2,211,74,1,0,0,0,0,12,6560.469415,6833.273684,10.98
12032,2213,2011-11-24,1,4,0,22.3333339691162,37.6666679382324,0,60,3,2,211,74,1,0,0,0,0,12,6
12033,2213,2011-11-24,2,4,0,36.6666679382324,23.3333339691162,0,60,3,2,211,74,1,0,0,0,0,12,6
12034,2213,2011-11-24,3,4,0,28.6666660308838,30.6666660308838,0.666666686534882,60,3,2,2
12035,2213,2011-11-24,4,4,0,41.3333320617676,18.6666660308838,0,60,3,2,211,74,1,0,0,0,0,12,6
12036,2222,2011-11-11,1,5,0,36,12,2,50,1,2,211,78,1,1,1,1,0,11,6521.98519,6649.167495,11.3614
12037,2222,2011-11-11,2,5,0,38.7999992370605,21,0.200000002980232,60,3,2,211,78,1,1,1,1,0,1
12038,2222,2011-11-11,3,5,0,30.3333339691162,29.3333339691162,0.333333343267441,60,3,2,2
12039,2222,2011-11-11,4,5,0,26.75,33,0.25,60,3,2,211,78,1,1,1,1,0,11,6521.98519,6649.167495,11
12040,2222,2011-11-12,4,6,1,16,44,0,60,2,2,211,78,1,1,1,1,0,11,6521.98519,6649.167495,11.3614
12041,2222,2011-11-24,1,4,0,19.5,13.5,0.5,33.5,2,2,211,78,1,1,1,1,0,11,6521.98519,6649.167495,1
12042,2222,2011-11-25,1,5,0,29,18,0.333333343267441,47.3333320617676,3,2,211,78,1,1,1,1,0,1
12043,2222,2011-11-25,2,5,0,45.5999984741211,14.1999998092651,0.200000002980232,60,3,2,2
12044,2222,2011-11-25,3,5,0,23.6666660308838,35,1.33333337306976,60,3,2,211,78,1,1,1,1,0,11,
12045,2222,2011-11-25,4,5,0,29.6666660308838,30.3333339691162,0,60,3,2,211,78,1,1,1,1,0,11,6
12046,2222,2011-11-26,1,6,1,26,18,1,45,1,2,211,78,1,1,1,1,0,11,6521.98519,6649.167495,11.3614
12047,2222,2011-11-26,2,6,1,47.25,12.75,0,60,3,2,211,78,1,1,1,1,0,11,6521.98519,6649.167495,11
12048,2222,2011-11-26,3,6,1,26,34,0,60,3,2,211,78,1,1,1,1,0,11,6521.98519,6649.167495,11.3614
12049,2222,2011-11-26,4,6,1,41.3333320617676,18.6666660308838,0,60,3,2,211,78,1,1,1,1,0,11,6

12050,2222,2011-11-27,1,0,1,15.3333330154419,22.6666660308838,0.333333343267441,38.3333
12051,2222,2011-11-27,2,0,1,18.3333339691162,41.6666679382324,0,60,3,2,211,78,1,1,1,1,0,11,6
12052,2222,2011-11-27,3,0,1,38.6666679382324,21.3333339691162,0,60,3,2,211,78,1,1,1,1,0,11,6
12053,2222,2011-11-27,4,0,1,32.6666679382324,27.1666660308838,0.16666667163372,60,3,2,21
12054,2222,2011-11-28,1,1,0,23.3333339691162,18,0,41.3333320617676,3,2,211,78,1,1,1,1,0,11,6
12055,2222,2011-11-28,2,1,0,26.5,33.5,0,60,3,2,211,78,1,1,1,1,0,11,6521.98519,6649.167495,11.3
12056,2222,2011-11-28,3,1,0,27.3333339691162,32.6666679382324,0,60,3,2,211,78,1,1,1,1,0,11,6
12057,2222,2011-11-28,4,1,0,28.6666660308838,31.3333339691162,0,60,3,2,211,78,1,1,1,1,0,11,6
12058,2223,2011-11-12,0,6,1,5.5,13.5,0,19,2,2,211,89,0,1,0,1,0,11,6935.36468,6274.928883,11.55
12059,2223,2011-11-12,1,6,1,26.6666660308838,25.6666660308838,7.66666650772095,60,3,2,21
12060,2223,2011-11-12,2,6,1,36,23.3333339691162,0.666666686534882,60,3,2,211,89,0,1,0,1,0,1
12061,2223,2011-11-12,3,6,1,30.3333339691162,29.6666660308838,0,60,3,2,211,89,0,1,0,1,0,11,6
12062,2223,2011-11-12,4,6,1,44,16,0,60,3,2,211,89,0,1,0,1,0,11,6935.36468,6274.928883,11.5514
12063,2223,2011-11-13,0,0,1,38,1,0,39,1,2,211,89,0,1,0,1,0,11,6935.36468,6274.928883,11.551438
12064,2223,2011-11-13,1,0,1,38.4000015258789,21.6000003814697,0,60,3,2,211,89,0,1,0,1,0,11,6
12065,2223,2011-11-13,2,0,1,39.6666679382324,20.3333339691162,0,60,3,2,211,89,0,1,0,1,0,11,6
12066,2223,2011-11-13,3,0,1,37.6666679382324,22.3333339691162,0,60,3,2,211,89,0,1,0,1,0,11,6
12067,2223,2011-11-13,4,0,1,45,15,0,60,3,2,211,89,0,1,0,1,0,11,6935.36468,6274.928883,11.5514
12068,2223,2011-11-14,0,1,0,23,22,0,45,2,2,211,89,0,1,0,1,0,11,6935.36468,6274.928883,11.5514
12069,2223,2011-11-14,1,1,0,39.75,20.25,0,60,3,2,211,89,0,1,0,1,0,11,6935.36468,6274.928883,11
12070,2223,2011-11-14,2,1,0,38,22,0,60,3,2,211,89,0,1,0,1,0,11,6935.36468,6274.928883,11.5514
12071,2223,2011-11-14,3,1,0,42.6666679382324,17.3333339691162,0,60,3,2,211,89,0,1,0,1,0,11,6
12072,2223,2011-11-14,4,1,0,49.6666679382324,10.3333330154419,0,60,3,2,211,89,0,1,0,1,0,11,6
12073,2223,2011-11-15,0,2,0,0,2,0,2,1,2,211,89,0,1,0,1,0,11,6935.36468,6274.928883,11.5514382
12074,2223,2011-11-15,2,2,0,47.7999992370605,11.6000003814697,0,59.4000015258789,3,2,211,
12075,2223,2011-11-15,3,2,0,46.5999984741211,9.80000019073486,3.59999990463257,60,3,2,21
12076,2223,2011-11-15,4,2,0,46.3333320617676,13.6666669845581,0,60,3,2,211,89,0,1,0,1,0,11,6
12077,2223,2011-11-16,0,3,0,1,0,0,1,1,2,211,89,0,1,0,1,0,11,6935.36468,6274.928883,11.5514382
12078,2223,2011-11-16,1,3,0,30,14,0,44,1,2,211,89,0,1,0,1,0,11,6935.36468,6274.928883,11.5514
12079,2223,2011-11-16,2,3,0,25.3333339691162,34.6666679382324,0,60,3,2,211,89,0,1,0,1,0,11,6
12080,2223,2011-11-16,3,3,0,24.75,35.25,0,60,3,2,211,89,0,1,0,1,0,11,6935.36468,6274.928883,11
12081,2223,2011-11-16,4,3,0,48.1666679382324,11.8333330154419,0,60,3,2,211,89,0,1,0,1,0,11,6
12082,2223,2011-11-17,0,4,0,38,22,0,60,1,2,211,89,0,1,0,1,0,11,6935.36468,6274.928883,11.5514
12083,2223,2011-11-17,1,4,0,39,21,0,60,3,2,211,89,0,1,0,1,0,11,6935.36468,6274.928883,11.5514
12084,2223,2011-11-17,2,4,0,41,19,0,60,3,2,211,89,0,1,0,1,0,11,6935.36468,6274.928883,11.5514
12085,2223,2011-11-17,3,4,0,34,18.6666660308838,7.33333349227905,60,3,2,211,89,0,1,0,1,0,11,
12086,2223,2011-11-17,4,4,0,46.3333320617676,13.6666669845581,0,60,3,2,211,89,0,1,0,1,0,11,6
12087,2226,2011-11-17,0,4,0,17,34,9,60,1,2,211,70,0,1,0,1,0,12,6866.602256,6341.883492,12.154
12088,2226,2011-11-17,1,4,0,5.5,30.5,24,60,2,2,211,70,0,1,0,1,0,12,6866.602256,6341.883492,12.1
12089,2226,2011-11-23,0,3,0,12,27,8.33333301544189,47.3333320617676,3,2,211,70,0,1,0,1,0,12,
12090,2226,2011-11-23,1,3,0,12.3333330154419,34.3333320617676,13.3333330154419,60,3,2,21
12091,2226,2011-11-23,2,3,0,33,24.6666660308838,2.33333325386047,60,3,2,211,70,0,1,0,1,0,12,
12092,2226,2011-11-23,3,3,0,45,14.6666669845581,0.333333343267441,60,3,2,211,70,0,1,0,1,0,1
12093,2226,2011-11-23,4,3,0,41,18.3333339691162,0.666666686534882,60,3,2,211,70,0,1,0,1,0,1
12094,2226,2011-11-24,0,4,0,7.5,22.5,28.5,58.5,2,2,211,70,0,1,0,1,0,12,6866.602256,6341.883492,
12095,2226,2011-11-24,1,4,0,18.75,36.25,5,60,3,2,211,70,0,1,0,1,0,12,6866.602256,6341.883492,1
12096,2226,2011-11-24,2,4,0,23,33,4,60,3,2,211,70,0,1,0,1,0,12,6866.602256,6341.883492,12.154
12097,2226,2011-11-24,3,4,0,35.3333320617676,23,1.66666662693024,60,3,2,211,70,0,1,0,1,0,12,
12098,2226,2011-11-24,4,4,0,14,42.3333320617676,3.66666674613953,60,3,2,211,70,0,1,0,1,0,12,
12099,2226,2011-11-25,0,5,0,26,27,2,55,1,2,211,70,0,1,0,1,0,12,6866.602256,6341.883492,12.154

12100,2226,2011-11-25,1,5,0,6.66666650772095,33,20.3333339691162,60,3,2,211,70,0,1,0,1,0,12,
12101,2226,2011-11-25,2,5,0,33,25.75,1.25,60,3,2,211,70,0,1,0,1,0,12,6866.602256,6341.883492,1
12102,2226,2011-11-25,3,5,0,29.75,23.75,6.5,60,3,2,211,70,0,1,0,1,0,12,6866.602256,6341.883492
12103,2226,2011-11-25,4,5,0,37,21.6666660308838,1.33333337306976,60,3,2,211,70,0,1,0,1,0,12,
12104,2226,2011-11-26,0,6,1,5.33333349227905,15,3.66666674613953,24,3,2,211,70,0,1,0,1,0,12,
12105,2226,2011-11-26,1,6,1,16,22.6666660308838,21.3333339691162,60,3,2,211,70,0,1,0,1,0,12,
12106,2226,2011-11-26,2,6,1,37.3333320617676,21.3333339691162,1.33333337306976,60,3,2,21
12107,2226,2011-11-26,3,6,1,32.2000007629395,22.7999992370605,5,60,3,2,211,70,0,1,0,1,0,12,6
12108,2226,2011-11-26,4,6,1,34.5,22.8333339691162,2.66666674613953,60,3,2,211,70,0,1,0,1,0,1
12109,2226,2011-11-27,0,0,1,14.6666669845581,22,1.33333337306976,38,3,2,211,70,0,1,0,1,0,12,
12110,2226,2011-11-27,1,0,1,17.6666660308838,31.3333339691162,11,60,3,2,211,70,0,1,0,1,0,12,
12111,2226,2011-11-27,2,0,1,25.3333339691162,33,1.66666662693024,60,3,2,211,70,0,1,0,1,0,12,
12112,2226,2011-11-27,3,0,1,24.3333339691162,26.6666660308838,9,60,3,2,211,70,0,1,0,1,0,12,6
12113,2226,2011-11-27,4,0,1,39.6666679382324,18,2.33333325386047,60,3,2,211,70,0,1,0,1,0,12,
12114,2226,2011-11-28,0,1,0,17.6666660308838,23.3333339691162,19,60,3,2,211,70,0,1,0,1,0,12,
12115,2226,2011-11-28,1,1,0,15.6666669845581,28.5,15.8333330154419,60,3,2,211,70,0,1,0,1,0,1
12116,2226,2011-11-28,2,1,0,27.3333339691162,28.3333339691162,4.33333349227905,60,3,2,21
12117,2226,2011-11-28,3,1,0,32,25,3,60,3,2,211,70,0,1,0,1,0,12,6866.602256,6341.883492,12.154
12118,2226,2011-11-28,4,1,0,33,25,2,60,3,2,211,70,0,1,0,1,0,12,6866.602256,6341.883492,12.154
12119,2226,2011-11-29,2,2,0,20.3333339691162,36.3333320617676,3.33333325386047,60,3,2,21
12120,2226,2011-11-29,3,2,0,6,0,0,6,1,2,211,70,0,1,0,1,0,12,6866.602256,6341.883492,12.154897
12121,2227,2011-11-19,0,6,1,20.5,17.25,1.25,39,3,2,211,65,0,1,0,1,0,12,6305.540777,7135.856018
12122,2227,2011-11-19,1,6,1,41.3333320617676,17.3333339691162,1.33333337306976,60,3,2,21
12123,2227,2011-11-19,2,6,1,30.6666660308838,27.3333339691162,2,60,3,2,211,65,0,1,0,1,0,12,6
12124,2227,2011-11-19,3,6,1,33.3333320617676,21.6666660308838,5,60,3,2,211,65,0,1,0,1,0,12,6
12125,2227,2011-11-19,4,6,1,43.5,16,0.5,60,3,2,211,65,0,1,0,1,0,12,6305.540777,7135.856018,10.
12126,2227,2011-11-20,0,0,1,21,24,3.40000009536743,48.4000015258789,3,2,211,65,0,1,0,1,0,12,
12127,2227,2011-11-20,1,0,1,15.6000003814697,41,3.40000009536743,60,3,2,211,65,0,1,0,1,0,12,
12128,2227,2011-11-20,2,0,1,34,24,2,60,3,2,211,65,0,1,0,1,0,12,6305.540777,7135.856018,10.753
12129,2227,2011-11-20,3,0,1,29.6666660308838,29.3333339691162,1,60,3,2,211,65,0,1,0,1,0,12,6
12130,2227,2011-11-20,4,0,1,33.3333320617676,21.3333339691162,5.33333349227905,60,3,2,21
12131,2227,2011-11-21,0,1,0,2,23,0,25,1,2,211,65,0,1,0,1,0,12,6305.540777,7135.856018,10.7530
12132,2227,2011-11-21,1,1,0,37.25,16.75,6,60,3,2,211,65,0,1,0,1,0,12,6305.540777,7135.856018,1
12133,2227,2011-11-21,2,1,0,16,39.25,4.75,60,3,2,211,65,0,1,0,1,0,12,6305.540777,7135.856018,1
12134,2227,2011-11-21,3,1,0,47,9.33333301544189,3.66666674613953,60,3,2,211,65,0,1,0,1,0,12,
12135,2227,2011-11-21,4,1,0,40.3333320617676,15.6666669845581,4,60,3,2,211,65,0,1,0,1,0,12,6
12136,2227,2011-11-22,1,2,0,18.3333339691162,39.6666679382324,0.666666686534882,58.6666
12137,2227,2011-11-22,2,2,0,23.5,29,2.75,55.25,3,2,211,65,0,1,0,1,0,12,6305.540777,7135.856018
12138,2227,2011-11-22,3,2,0,7.66666650772095,26.6666660308838,23.8333339691162,58.16666
12139,2227,2011-11-22,4,2,0,32.5,27.25,0.25,60,3,2,211,65,0,1,0,1,0,12,6305.540777,7135.856018
12140,2227,2011-11-23,0,3,0,14.6666669845581,23,3.66666674613953,41.3333320617676,3,2,21
12141,2227,2011-11-23,1,3,0,31,20.6666660308838,8.33333301544189,60,3,2,211,65,0,1,0,1,0,12,
12142,2227,2011-11-23,2,3,0,36.3333320617676,21,2.66666674613953,60,3,2,211,65,0,1,0,1,0,12,
12143,2227,2011-11-23,3,3,0,41,18,1,60,3,2,211,65,0,1,0,1,0,12,6305.540777,7135.856018,10.753
12144,2227,2011-11-23,4,3,0,38.2000007629395,18.7999992370605,3,60,3,2,211,65,0,1,0,1,0,12,6
12145,2227,2011-11-29,0,2,0,6,53,1,60,1,2,211,65,0,1,0,1,0,12,6305.540777,7135.856018,10.7530
12146,2227,2011-11-29,1,2,0,19,34.6666679382324,6.33333349227905,60,3,2,211,65,0,1,0,1,0,12,
12147,2227,2011-11-29,2,2,0,24,31.3333339691162,4.66666650772095,60,3,2,211,65,0,1,0,1,0,12,
12148,2227,2011-11-29,3,2,0,31,27,2,60,3,2,211,65,0,1,0,1,0,12,6305.540777,7135.856018,10.753
12149,2227,2011-11-29,4,2,0,35,24.5,0.5,60,2,2,211,65,0,1,0,1,0,12,6305.540777,7135.856018,10.

12150,2242,2011-11-11,0,5,0,20.5,26.5,2.5,49.5,2,2,211,79,0,0,0,1,0,12,6773.535653,6414.204939,
12151,2242,2011-11-11,1,5,0,35.3333320617676,24,0.666666686534882,60,3,2,211,79,0,0,0,1,0,12
12152,2242,2011-11-11,2,5,0,35,24.5,0.5,60,3,2,211,79,0,0,0,1,0,12,6773.535653,6414.204939,11.7
12153,2242,2011-11-11,3,5,0,10.6666669845581,47.3333320617676,2,60,3,2,211,79,0,0,0,1,0,12,6
12154,2242,2011-11-11,4,5,0,33,26.3333339691162,0.666666686534882,60,3,2,211,79,0,0,0,1,0,12
12155,2242,2011-11-12,0,6,1,13,25.5,1,39.5,2,2,211,79,0,0,0,1,0,12,6773.535653,6414.204939,11.7
12156,2242,2011-11-12,1,6,1,23.3333339691162,29.6666660308838,7,60,3,2,211,79,0,0,0,1,0,12,6
12157,2242,2011-11-12,2,6,1,33.2000007629395,24.2000007629395,2.5999999046325 7,60,3,2,211
12158,2242,2011-11-12,3,6,1,45.6666679382324,14.3333330154419,0,60,3,2,211,79,0,0,0,1,0,12,6
12159,2242,2011-11-12,4,6,1,36.6666679382324,23,0.333333343267441,60,3,2,211,79,0,0,0,1,0,12
12160,2242,2011-11-13,0,0,1,13.5,31,6.5,51,2,2,211,79,0,0,0,1,0,12,6773.535653,6414.204939,11.7
12161,2242,2011-11-13,1,0,1,0.666666686534882,51.6666679382324,7.66666650772095,60,3,2,21
12162,2242,2011-11-13,2,0,1,35,24,1,60,3,2,211,79,0,0,0,1,0,12,6773.535653,6414.204939,11.7181
12163,2242,2011-11-13,3,0,1,18.7999992370605,38.5999984741211,2.59999990463257,60,3,2,211
12164,2242,2011-11-13,4,0,1,43.3333320617676,16.6666660308838,0,60,3,2,211,79,0,0,0,1,0,12,6
12165,2242,2011-11-14,0,1,0,35,24,1,60,1,2,211,79,0,0,0,1,0,12,6773.535653,6414.204939,11.7181
12166,2242,2011-11-14,1,1,0,11.6666669845581,43.6666679382324,4.66666650772095,60,3,2,211
12167,2242,2011-11-14,2,1,0,38,17.6666660308838,4.33333349227905,60,3,2,211,79,0,0,0,1,0,12,
12168,2242,2011-11-14,3,1,0,37.75,21.75,0.5,60,3,2,211,79,0,0,0,1,0,12,6773.535653,6414.204939
12169,2242,2011-11-14,4,1,0,42.75,17.25,0,60,3,2,211,79,0,0,0,1,0,12,6773.535653,6414.204939,1
12170,2242,2011-11-15,4,2,0,49,11,0,60,2,2,211,79,0,0,0,1,0,12,6773.535653,6414.204939,11.7181
12171,2242,2011-11-24,0,4,0,26.25,24.5,0.75,51.5,3,2,211,79,0,0,0,1,0,12,6773.535653,6414.20493
12172,2242,2011-11-24,1,4,0,33.3333320617676,25,1.66666662693024,60,3,2,211,79,0,0,0,1,0,12,
12173,2242,2011-11-24,2,4,0,36.3333320617676,23.6666660308838,0,60,3,2,211,79,0,0,0,1,0,12,6
12174,2242,2011-11-24,3,4,0,29,30.3333339691162,0.666666686534882,60,3,2,211,79,0,0,0,1,0,12
12175,2242,2011-11-24,4,4,0,39.6666679382324,20.3333339691162,0,60,3,2,211,79,0,0,0,1,0,12,6
12176,2242,2011-11-25,0,5,0,18,25,1.5,44.5,2,2,211,79,0,0,0,1,0,12,6773.535653,6414.204939,11.7
12177,2242,2011-11-25,1,5,0,24,34.8333320617676,1.16666662693024,60,3,2,211,79,0,0,0,1,0,12,
12178,2242,2011-11-25,2,5,0,25.6666660308838,30.6666660308838,3.66666674613953,60,3,2,211
12179,2242,2011-11-25,3,5,0,34,19.6666660308838,6.33333349227905,60,3,2,211,79,0,0,0,1,0,12,
12180,2242,2011-11-25,4,5,0,34.3333320617676,24.6666660308838,1,60,3,2,211,79,0,0,0,1,0,12,6
12181,2245,2011-11-15,0,2,0,60,0,0,60,1,2,213,67,1,1,0,1,0,12,4411.981245,4720.916013,6.205275
12182,2245,2011-11-15,1,2,0,32,26.3333339691162,1.66666662693024,60,3,2,213,67,1,1,0,1,0,12,
12183,2245,2011-11-15,2,2,0,28,30.6666660308838,1.33333337306976,60,3,2,213,67,1,1,0,1,0,12,
12184,2245,2011-11-15,3,2,0,10,49,1,60,3,2,213,67,1,1,0,1,0,12,4411.981245,4720.916013,6.20527
12185,2245,2011-11-15,4,2,0,23.3333339691162,35,1.66666662693024,60,3,2,213,67,1,1,0,1,0,12,
12186,2245,2011-11-16,0,3,0,13.6666669845581,21,0.666666686534882,35.3333320617676,3,2,21
12187,2245,2011-11-16,1,3,0,27,32.3333320617676,0.666666686534882,60,3,2,213,67,1,1,0,1,0,12
12188,2245,2011-11-16,2,3,0,21,37.6666679382324,1.33333337306976,60,3,2,213,67,1,1,0,1,0,12,
12189,2245,2011-11-16,3,3,0,31.3333339691162,28.3333339691162,0.333333343267441,60,3,2,21
12190,2245,2011-11-16,4,3,0,31.6666660308838,28,0.333333343267441,60,3,2,213,67,1,1,0,1,0,12
12191,2245,2011-11-17,0,4,0,7.5,20.5,5,33,2,2,213,67,1,1,0,1,0,12,4411.981245,4720.916013,6.205
12192,2245,2011-11-17,1,4,0,20.6666660308838,37.6666679382324,1.66666662693024,60,3,2,213
12193,2245,2011-11-17,2,4,0,17,42.3333320617676,0.666666686534882,60,3,2,213,67,1,1,0,1,0,12
12194,2245,2011-11-17,3,4,0,14.3333330154419,44.6666679382324,1,60,3,2,213,67,1,1,0,1,0,12,4
12195,2245,2011-11-17,4,4,0,29,30.6666660308838,0.333333343267441,60,3,2,213,67,1,1,0,1,0,12
12196,2245,2011-11-18,0,5,0,14,13,1,28,2,2,213,67,1,1,0,1,0,12,4411.981245,4720.916013,6.20527
12197,2245,2011-11-18,1,5,0,24.8571434020996,29.5714282989502,5.57142877578735,60,3,2,213
12198,2245,2011-11-18,2,5,0,15.8333330154419,37.8333320617676,6.33333349227905,60,3,2,213
12199,2245,2011-11-18,3,5,0,31.1666660308838,24.3333339691162,4.5,60,3,2,213,67,1,1,0,1,0,12

12200,2245,2011-11-18,4,5,0,30.5,25.6666660308838,3.83333325386047,60,3,2,213,67,1,1,0,1,0,1
12201,2245,2011-11-19,1,6,1,15.75,35,1.5,52.25,3,2,213,67,1,1,0,1,0,12,4411.981245,4720.916013
12202,2245,2011-11-19,2,6,1,23.5,36.25,0.25,60,3,2,213,67,1,1,0,1,0,12,4411.981245,4720.916013
12203,2245,2011-11-19,3,6,1,24.6666660308838,34.3333320617676,1,60,3,2,213,67,1,1,0,1,0,12,4
12204,2245,2011-11-19,4,6,1,35.3333320617676,24.6666660308838,0,60,3,2,213,67,1,1,0,1,0,12,4
12205,2245,2011-11-20,2,0,1,24,35,1,60,2,2,213,67,1,1,0,1,0,12,4411.981245,4720.916013,6.20527
12206,2245,2011-11-20,3,0,1,25.3333339691162,33.3333320617676,1.33333337306976,60,3,2,213
12207,2245,2011-11-20,4,0,1,35.6666679382324,23.6666660308838,0.666666686534882,60,3,2,21
12208,2246,2011-11-20,0,0,1,43,12,5,60,1,2,213,70,0,1,1,1,0,12,4411.981245,4720.916013,6.20527
12209,2246,2011-11-20,1,0,1,10.6666669845581,44,5.33333349227905,60,3,2,213,70,0,1,1,1,0,12,
12210,2246,2011-11-20,2,0,1,20.6666660308838,35.6666679382324,3.66666674613953,60,3,2,213
12211,2246,2011-11-21,0,1,0,5,24.5,7,36.5,2,2,213,70,0,1,1,1,0,12,4411.981245,4720.916013,6.205
12212,2246,2011-11-21,1,1,0,25.6666660308838,17,17.3333339691162,60,3,2,213,70,0,1,1,1,0,12,
12213,2246,2011-11-21,2,1,0,31.6666660308838,16.6666660308838,11.6666669845581,60,3,2,213
12214,2246,2011-11-21,3,1,0,34.5,20.25,5.25,60,3,2,213,70,0,1,1,1,0,12,4411.981245,4720.916013
12215,2246,2011-11-21,4,1,0,41.3333320617676,18,0.666666686534882,60,3,2,213,70,0,1,1,1,0,12
12216,2246,2011-11-22,0,2,0,15,21,0,36,1,2,213,70,0,1,1,1,0,12,4411.981245,4720.916013,6.20527
12217,2246,2011-11-22,1,2,0,8,44,8,60,3,2,213,70,0,1,1,1,0,12,4411.981245,4720.916013,6.205275
12218,2246,2011-11-22,2,2,0,5.33333349227905,35,19.6666660308838,60,3,2,213,70,0,1,1,1,0,12,
12219,2246,2011-11-22,3,2,0,43.5999984741211,5.59999990463257,10.8000001907349,60,3,2,213
12220,2246,2011-11-22,4,2,0,41.5,7.75,0.5,49.75,3,2,213,70,0,1,1,1,0,12,4411.981245,4720.916013
12221,2246,2011-11-23,0,3,0,5,15,2,22,1,2,213,70,0,1,1,1,0,12,4411.981245,4720.916013,6.205275
12222,2246,2011-11-23,1,3,0,21.75,31,5.5,58.25,3,2,213,70,0,1,1,1,0,12,4411.981245,4720.916013
12223,2246,2011-11-23,2,3,0,40.3333320617676,17.6666660308838,2,60,3,2,213,70,0,1,1,1,0,12,4
12224,2246,2011-11-23,3,3,0,42.6666679382324,15.3333330154419,2,60,3,2,213,70,0,1,1,1,0,12,4
12225,2246,2011-11-23,4,3,0,42.7999992370605,12.3999996185303,4.80000019073486,60,3,2,213
12226,2246,2011-11-24,0,4,0,4,18,4,26,1,2,213,70,0,1,1,1,0,12,4411.981245,4720.916013,6.205275
12227,2246,2011-11-24,1,4,0,19.6000003814697,30,10.3999996185303,60,3,2,213,70,0,1,1,1,0,12,
12228,2246,2011-11-24,2,4,0,31,21.3333339691162,7.66666650772095,60,3,2,213,70,0,1,1,1,0,12,
12229,2246,2011-11-24,3,4,0,38.6666679382324,17.6666660308838,3.66666674613953,60,3,2,213
12230,2246,2011-11-24,4,4,0,51.6666679382324,7.33333349227905,1,60,3,2,213,70,0,1,1,1,0,12,4
12231,2246,2011-11-25,2,5,0,31,28,1,60,3,2,213,70,0,1,1,1,0,12,4411.981245,4720.916013,6.20527
12232,2246,2011-11-25,3,5,0,34.3333320617676,20.3333339691162,5.33333349227905,60,3,2,213
12233,2246,2011-11-25,4,5,0,40,13.6666669845581,0.666666686534882,54.3333320617676,3,2,21
12234,2250,2011-11-03,0,4,0,25,28,3,56,1,2,213,81,0,1,0,1,0,12,5042.89648,4618.750633,9.842397
12235,2250,2011-11-03,1,4,0,36,24,0,60,3,2,213,81,0,1,0,1,0,12,5042.89648,4618.750633,9.842397
12236,2250,2011-11-03,2,4,0,42.3333320617676,17.6666660308838,0,60,3,2,213,81,0,1,0,1,0,12,5
12237,2250,2011-11-03,3,4,0,42.5,17.3333339691162,0.16666667163372,60,3,2,213,81,0,1,0,1,0,1
12238,2250,2011-11-03,4,4,0,21.3333339691162,37.6666679382324,1,60,3,2,213,81,0,1,0,1,0,12,5
12239,2250,2011-11-04,0,5,0,12,20,1.5,33.5,2,2,213,81,0,1,0,1,0,12,5042.89648,4618.750633,9.842
12240,2250,2011-11-04,1,5,0,19.3333339691162,39.3333320617676,1.33333337306976,60,3,2,213
12241,2250,2011-11-04,2,5,0,36,23,1,60,3,2,213,81,0,1,0,1,0,12,5042.89648,4618.750633,9.842397
12242,2250,2011-11-04,3,5,0,25.3333339691162,33.6666679382324,1,60,3,2,213,81,0,1,0,1,0,12,5
12243,2250,2011-11-04,4,5,0,36.25,23.75,0,60,3,2,213,81,0,1,0,1,0,12,5042.89648,4618.750633,9.8
12244,2250,2011-11-05,0,6,1,25,16,0,41,1,2,213,81,0,1,0,1,0,12,5042.89648,4618.750633,9.842397
12245,2250,2011-11-05,1,6,1,11,42,7,60,3,2,213,81,0,1,0,1,0,12,5042.89648,4618.750633,9.842397
12246,2250,2011-11-05,2,6,1,27,29,4,60,3,2,213,81,0,1,0,1,0,12,5042.89648,4618.750633,9.842397
12247,2250,2011-11-05,3,6,1,20.6666660308838,37,2.33333325386047,60,3,2,213,81,0,1,0,1,0,12,
12248,2250,2011-11-05,4,6,1,34.5999984741211,24.2000007629395,1.20000004768372,60,3,2,213
12249,2250,2011-11-06,0,0,1,18.5,20.5,1,40,2,2,213,81,0,1,0,1,0,12,5042.89648,4618.750633,9.842

```
12250,2250,2011-11-06,1,0,1,9.5,35.75,14.75,60,3,2,213,81,0,1,0,1,0,12,5042.89648,4618.750633,9
12251,2250,2011-11-06,2,0,1,28.3333339691162,28.6666660308838,3,60,3,2,213,81,0,1,0,1,0,12,5
12252,2250,2011-11-06,3,0,1,9,42.6666679382324,8.33333301544189,60,3,2,213,81,0,1,0,1,0,12,5
12253,2250,2011-11-06,4,0,1,48,12,0,60,3,2,213,81,0,1,0,1,0,12,5042.89648,4618.750633,9.842397
12254,2250,2011-11-07,1,1,0,18,26,16,60,2,2,213,81,0,1,0,1,0,12,5042.89648,4618.750633,9.84239
12255,2250,2011-11-07,2,1,0,25,31.6666660308838,3.33333325386047,60,3,2,213,81,0,1,0,1,0,12,
12256,2250,2011-11-07,3,1,0,13.6666669845581,41.6666679382324,4.66666650772095,60,3,2,213
12257,2250,2011-11-07,4,1,0,49,10.6666669845581,0.333333343267441,60,3,2,213,81,0,1,0,1,0,12
12258,2250,2011-11-25,0,5,0,32,28,0,60,1,2,213,81,0,1,0,1,0,12,5042.89648,4618.750633,9.842397
12259,2250,2011-11-25,1,5,0,26.3333339691162,32.6666679382324,1,60,3,2,213,81,0,1,0,1,0,12,5
12260,2250,2011-11-25,2,5,0,35,25,0,60,2,2,213,81,0,1,0,1,0,12,5042.89648,4618.750633,9.842397
12261,2250,2011-11-26,0,6,1,0,9,2,11,1,2,213,81,0,1,0,1,0,12,5042.89648,4618.750633,9.8423973
12262,2250,2011-11-26,1,6,1,38.3333320617676,21.6666660308838,0,60,3,2,213,81,0,1,0,1,0,12,5
12263,2250,2011-11-26,2,6,1,46,13.75,0.25,60,3,2,213,81,0,1,0,1,0,12,5042.89648,4618.750633,9.8
12264,2250,2011-11-26,3,6,1,51,9,0,60,3,2,213,81,0,1,0,1,0,12,5042.89648,4618.750633,9.842397
12265,2250,2011-11-26,4,6,1,39.6666679382324,19.3333339691162,1,60,3,2,213,81,0,1,0,1,0,12,5
12266,2251,2011-11-07,0,1,0,24,36,0,60,1,2,213,80,1,1,1,1,0,7,4963.509813,4341.46778,10.30825
12267,2251,2011-11-07,1,1,0,32.6666679382324,27.3333339691162,0,60,3,2,213,80,1,1,1,1,0,7,49
12268,2251,2011-11-08,0,2,0,30.5,13,0.5,44,2,2,213,80,1,1,1,1,0,7,4963.509813,4341.46778,10.30
12269,2251,2011-11-08,1,2,0,18,40.6666679382324,1.33333337306976,60,3,2,213,80,1,1,1,1,0,7,4
12270,2251,2011-11-08,2,2,0,31.8333339691162,27,1.16666662693024,60,3,2,213,80,1,1,1,1,0,7,4
12271,2251,2011-11-08,3,2,0,31.6666660308838,28.3333339691162,0,60,3,2,213,80,1,1,1,1,0,7,49
12272,2251,2011-11-08,4,2,0,21,20,1,42,1,2,213,80,1,1,1,1,0,7,4963.509813,4341.46778,10.30825
12273,2251,2011-11-09,0,3,0,22,26,0.5,48.5,2,2,213,80,1,1,1,1,0,7,4963.509813,4341.46778,10.30
12274,2251,2011-11-09,1,3,0,24,36,0,60,3,2,213,80,1,1,1,1,0,7,4963.509813,4341.46778,10.30825
12275,2251,2011-11-09,2,3,0,23.6666660308838,36,0.333333343267441,60,3,2,213,80,1,1,1,1,0,7,
12276,2251,2011-11-09,3,3,0,25,32.3333320617676,2.66666674613953,60,3,2,213,80,1,1,1,1,0,7,4
12277,2251,2011-11-09,4,3,0,11.5,22,1.5,35,2,2,213,80,1,1,1,1,0,7,4963.509813,4341.46778,10.30
12278,2251,2011-11-10,0,4,0,26.5,16.5,0.5,43.5,2,2,213,80,1,1,1,1,0,7,4963.509813,4341.46778,10
12279,2251,2011-11-10,1,4,0,27,33,0,60,3,2,213,80,1,1,1,1,0,7,4963.509813,4341.46778,10.30825
12280,2251,2011-11-10,2,4,0,27.6666660308838,32.3333320617676,0,60,3,2,213,80,1,1,1,1,0,7,49
12281,2251,2011-11-10,3,4,0,41.3333320617676,18.6666660308838,0,60,3,2,213,80,1,1,1,1,0,7,49
12282,2251,2011-11-10,4,4,0,17.25,27,0.75,45,3,2,213,80,1,1,1,1,0,7,4963.509813,4341.46778,10.3
12283,2251,2012-02-09,0,4,0,32.5,15.75,0.25,48.5,3,2,213,80,1,1,1,1,0,7,4963.509813,4341.46778,
12284,2251,2012-02-09,1,4,0,24,36,0,60,3,2,213,80,1,1,1,1,0,7,4963.509813,4341.46778,10.30825
12285,2251,2012-02-09,2,4,0,36,23,1,60,3,2,213,80,1,1,1,1,0,7,4963.509813,4341.46778,10.30825
12286,2251,2012-02-09,3,4,0,57.6666679382324,2.33333325386047,0,60,3,2,213,80,1,1,1,1,0,7,49
12287,2251,2012-02-09,4,4,0,43,17,0,60,3,2,213,80,1,1,1,1,0,7,4963.509813,4341.46778,10.30825
12288,2251,2012-02-10,0,5,0,23.6666660308838,28.3333339691162,1,53,3,2,213,80,1,1,1,1,0,7,49
12289,2251,2012-02-10,1,5,0,43.5,16.1666660308838,0.333333343267441,60,3,2,213,80,1,1,1,1,0,
12290,2251,2012-02-10,2,5,0,29.3333339691162,30.3333339691162,0.333333343267441,60,3,2,2
12291,2251,2012-02-10,3,5,0,36.3333320617676,23.6666660308838,0,60,3,2,213,80,1,1,1,1,0,7,49
12292,2251,2012-02-10,4,5,0,13,42,1,56,1,2,213,80,1,1,1,1,0,7,4963.509813,4341.46778,10.30825
12293,2251,2012-02-11,2,6,1,42.3333320617676,17.6666660308838,0,60,3,2,213,80,1,1,1,1,0,7,49
12294,2251,2012-02-11,3,6,1,39.6666679382324,20.3333339691162,0,60,3,2,213,80,1,1,1,1,0,7,49
12295,2251,2012-02-11,4,6,1,18,28,0.5,46.5,2,2,213,80,1,1,1,1,0,7,4963.509813,4341.46778,10.30
12296,2254,2012-02-10,3,5,0,14.6666669845581,39.3333320617676,6,60,3,2,213,72,1,1,0,1,0,12,5
12297,2254,2012-02-10,4,5,0,24.6666660308838,33.3333320617676,2,60,3,2,213,72,1,1,0,1,0,12,5
12298,2254,2012-02-11,0,6,1,5,48.5,1.5,55,2,2,213,72,1,1,0,1,0,12,5024.935837,4662.789107,11.30
12299,2254,2012-02-11,1,6,1,25.6666660308838,33.3333320617676,1,60,3,2,213,72,1,1,0,1,0,12,5
```

12300,2254,2012-02-11,2,6,1,19.3333339691162,40.6666679382324,0,60,3,2,213,72,1,1,0,1,0,12,5
12301,2254,2012-02-11,3,6,1,11,47,2,60,1,2,213,72,1,1,0,1,0,12,5024.935837,4662.789107,11.3065
12302,2254,2012-02-12,0,0,1,13.5,20.5,0.5,34.5,2,2,213,72,1,1,0,1,0,12,5024.935837,4662.789107,
12303,2254,2012-02-12,1,0,1,12.3333330154419,45.6666679382324,2,60,3,2,213,72,1,1,0,1,0,12,5
12304,2254,2012-02-12,2,0,1,16.6666660308838,43.3333320617676,0,60,3,2,213,72,1,1,0,1,0,12,5
12305,2254,2012-02-12,3,0,1,27,30,3,60,3,2,213,72,1,1,0,1,0,12,5024.935837,4662.789107,11.3065
12306,2254,2012-02-12,4,0,1,18.25,41,0.75,60,3,2,213,72,1,1,0,1,0,12,5024.935837,4662.789107,1
12307,2254,2012-02-13,0,1,0,8,30,3.33333325386047,41.3333320617676,3,2,213,72,1,1,0,1,0,12,5
12308,2254,2012-02-13,1,1,0,24.6666660308838,33,2.33333325386047,60,3,2,213,72,1,1,0,1,0,12,
12309,2254,2012-02-13,2,1,0,34.6666679382324,23.3333339691162,2,60,3,2,213,72,1,1,0,1,0,12,5
12310,2254,2012-02-13,3,1,0,12.3333330154419,45.3333320617676,2.33333325386047,60,3,2,213
12311,2254,2012-02-13,4,1,0,25,34.75,0.25,60,3,2,213,72,1,1,0,1,0,12,5024.935837,4662.789107,1
12312,2254,2012-02-14,0,2,0,34,18.5,0.5,53,2,2,213,72,1,1,0,1,0,12,5024.935837,4662.789107,11.3
12313,2254,2012-02-14,1,2,0,11.1666669845581,46,2.83333325386047,60,3,2,213,72,1,1,0,1,0,12,
12314,2254,2012-02-14,2,2,0,24,35,1,60,3,2,213,72,1,1,0,1,0,12,5024.935837,4662.789107,11.3065
12315,2254,2012-02-14,3,2,0,24.6666660308838,31.6666660308838,3.66666674613953,60,3,2,213
12316,2254,2012-02-14,4,2,0,43.25,15.5,1.25,60,3,2,213,72,1,1,0,1,0,12,5024.935837,4662.789107
12317,2254,2012-02-15,0,3,0,36,19,1,56,1,2,213,72,1,1,0,1,0,12,5024.935837,4662.789107,11.3065
12318,2254,2012-02-15,1,3,0,6,49.3333320617676,4.66666650772095,60,3,2,213,72,1,1,0,1,0,12,5
12319,2254,2012-02-15,2,3,0,24.3999996185303,31.7999992370605,3.79999995231628,60,3,2,213
12320,2254,2012-02-15,3,3,0,32,27.6666660308838,0.333333343267441,60,3,2,213,72,1,1,0,1,0,12
12321,2254,2012-02-15,4,3,0,18,42,0,60,3,2,213,72,1,1,0,1,0,12,5024.935837,4662.789107,11.3065
12322,2254,2012-02-16,0,4,0,13,13,2,28,1,2,213,72,1,1,0,1,0,12,5024.935837,4662.789107,11.3065
12323,2254,2012-02-16,1,4,0,19.6666660308838,39.3333320617676,1,60,3,2,213,72,1,1,0,1,0,12,5
12324,2254,2012-02-16,2,4,0,23.5,34,2.5,60,3,2,213,72,1,1,0,1,0,12,5024.935837,4662.789107,11.3
12325,2254,2012-02-16,3,4,0,22.3333339691162,37.6666679382324,0,60,3,2,213,72,1,1,0,1,0,12,5
12326,2254,2012-02-16,4,4,0,27,31.6666660308838,1.33333337306976,60,3,2,213,72,1,1,0,1,0,12,
12327,2257,2012-02-10,0,5,0,21,36,3,60,1,2,213,74,0,1,0,1,0,12,4367.922972,4753.3769,6.1251318
12328,2257,2012-02-10,1,5,0,14,41,5,60,3,2,213,74,0,1,0,1,0,12,4367.922972,4753.3769,6.1251318
12329,2257,2012-02-10,2,5,0,31.3333339691162,28.3333339691162,0.333333343267441,60,3,2,21
12330,2257,2012-02-10,3,5,0,11.6666669845581,47.3333320617676,1,60,3,2,213,74,0,1,0,1,0,12,4
12331,2257,2012-02-11,0,6,1,10.5,22.75,1,34.25,3,2,213,74,0,1,0,1,0,12,4367.922972,4753.3769,6.
12332,2257,2012-02-11,1,6,1,24.3333339691162,35.3333320617676,0.333333343267441,60,3,2,21
12333,2257,2012-02-11,2,6,1,31,28.25,0.75,60,3,2,213,74,0,1,0,1,0,12,4367.922972,4753.3769,6.12
12334,2257,2012-02-11,3,6,1,1.66666662693024,52.3333320617676,6,60,3,2,213,74,0,1,0,1,0,12,4
12335,2257,2012-02-11,4,6,1,24.6666660308838,33.3333320617676,2,60,3,2,213,74,0,1,0,1,0,12,4
12336,2257,2012-02-12,0,0,1,16,33,1,50,1,2,213,74,0,1,0,1,0,12,4367.922972,4753.3769,6.1251318
12337,2257,2012-02-12,1,0,1,12.3333330154419,43.3333320617676,4.33333349227905,60,3,2,213
12338,2257,2012-02-12,2,0,1,28,29.6000003814697,2.40000009536743,60,3,2,213,74,0,1,0,1,0,12,
12339,2257,2012-02-12,3,0,1,22.3333339691162,34.3333320617676,3.33333325386047,60,3,2,213
12340,2257,2012-02-12,4,0,1,43.3333320617676,16.6666660308838,0,60,3,2,213,74,0,1,0,1,0,12,4
12341,2257,2012-02-13,0,1,0,20,34,2,56,1,2,213,74,0,1,0,1,0,12,4367.922972,4753.3769,6.1251318
12342,2257,2012-02-13,1,1,0,31,25.3333339691162,3.66666674613953,60,3,2,213,74,0,1,0,1,0,12,
12343,2257,2012-02-13,2,1,0,27.3333339691162,32.6666679382324,0,60,3,2,213,74,0,1,0,1,0,12,4
12344,2257,2012-02-13,3,1,0,31.7999992370605,26.7999992370605,1.39999997615814,60,3,2,213
12345,2257,2012-02-13,4,1,0,48.6666679382324,11,0.333333343267441,60,3,2,213,74,0,1,0,1,0,12
12346,2257,2012-02-14,0,2,0,9,37,1,47,1,2,213,74,0,1,0,1,0,12,4367.922972,4753.3769,6.12513185
12347,2257,2012-02-14,1,2,0,8.66666698455811,48.6666679382324,2.66666674613953,60,3,2,213
12348,2257,2012-02-14,2,2,0,16.3333339691162,41.3333320617676,2.33333325386047,60,3,2,213
12349,2257,2012-02-14,3,2,0,13.25,42.25,4.5,60,3,2,213,74,0,1,0,1,0,12,4367.922972,4753.3769,6.

12350,2257,2012-02-14,4,2,0,38.3333320617676,21.3333339691162,0.333333343267441,60,3,2,2
12351,2257,2012-02-15,0,3,0,3,37,0,40,1,2,213,74,0,1,0,1,0,12,4367.922972,4753.3769,6.1251318
12352,2257,2012-02-15,1,3,0,20,33.3333320617676,6.66666650772095,60,3,2,213,74,0,1,0,1,0,12,
12353,2257,2012-02-15,2,3,0,21.6666660308838,34,4.33333349227905,60,3,2,213,74,0,1,0,1,0,12,
12354,2257,2012-02-15,3,3,0,20,34.3333320617676,5.66666650772095,60,3,2,213,74,0,1,0,1,0,12,
12355,2257,2012-02-15,4,3,0,43.6666679382324,16,0.333333343267441,60,3,2,213,74,0,1,0,1,0,1
12356,2261,2012-02-15,0,3,0,21,36,3,60,1,2,213,80,1,1,1,1,0,10,5085.652081,4386.068339,9.6860
12357,2261,2012-02-15,1,3,0,14,41,5,60,3,2,213,80,1,1,1,1,0,10,5085.652081,4386.068339,9.6860
12358,2261,2012-02-16,0,4,0,9.5,21,1,31.5,2,2,213,80,1,1,1,1,0,10,5085.652081,4386.068339,9.68
12359,2261,2012-02-16,1,4,0,28,31.6666660308838,0.333333343267441,60,3,2,213,80,1,1,1,1,0,1
12360,2261,2012-02-16,2,4,0,34.5999984741211,25.2000007629395,0.200000002980232,60,3,2,2
12361,2261,2012-02-16,3,4,0,11.6666669845581,47.3333320617676,1,60,3,2,213,80,1,1,1,1,0,10,5
12362,2261,2012-02-16,4,4,0,23.6666660308838,33.6666679382324,2.66666674613953,60,3,2,21
12363,2261,2012-02-17,0,5,0,11.5,24.5,1,37,2,2,213,80,1,1,1,1,0,10,5085.652081,4386.068339,9.6
12364,2261,2012-02-17,1,5,0,20.6666660308838,39,0.333333343267441,60,3,2,213,80,1,1,1,1,0,1
12365,2261,2012-02-17,2,5,0,26,31,3,60,3,2,213,80,1,1,1,1,0,10,5085.652081,4386.068339,9.6860
12366,2261,2012-02-17,3,5,0,3.5,51,5.5,60,3,2,213,80,1,1,1,1,0,10,5085.652081,4386.068339,9.68
12367,2261,2012-02-17,4,5,0,25.6666660308838,33,1.33333337306976,60,3,2,213,80,1,1,1,1,0,10,
12368,2261,2012-03-01,0,4,0,16,33,1,50,1,2,213,80,1,1,1,1,0,10,5085.652081,4386.068339,9.6860
12369,2261,2012-03-01,1,4,0,12.3333330154419,43.3333320617676,4.33333349227905,60,3,2,21
12370,2261,2012-03-01,2,4,0,27,32,1,60,3,2,213,80,1,1,1,1,0,10,5085.652081,4386.068339,9.6860
12371,2261,2012-03-01,3,4,0,20.75,36,3.25,60,3,2,213,80,1,1,1,1,0,10,5085.652081,4386.068339,9
12372,2261,2012-03-01,4,4,0,43.3333320617676,16.6666660308838,0,60,3,2,213,80,1,1,1,1,0,10,5
12373,2261,2012-03-02,0,5,0,20,34,2,56,1,2,213,80,1,1,1,1,0,10,5085.652081,4386.068339,9.6860
12374,2261,2012-03-02,1,5,0,31,25.3333339691162,3.66666674613953,60,3,2,213,80,1,1,1,1,0,10,
12375,2261,2012-03-02,2,5,0,27.3333339691162,32.6666679382324,0,60,3,2,213,80,1,1,1,1,0,10,5
12376,2261,2012-03-02,3,5,0,40.25,19,0.75,60,3,2,213,80,1,1,1,1,0,10,5085.652081,4386.068339,9
12377,2261,2012-03-02,4,5,0,45.25,14.5,0.25,60,3,2,213,80,1,1,1,1,0,10,5085.652081,4386.068339
12378,2261,2012-03-03,0,6,1,6,37,0.5,43.5,2,2,213,80,1,1,1,1,0,10,5085.652081,4386.068339,9.68
12379,2261,2012-03-03,1,6,1,8.66666698455811,48.6666679382324,2.66666674613953,60,3,2,21
12380,2261,2012-03-03,2,6,1,16.3333339691162,41.3333320617676,2.33333325386047,60,3,2,21
12381,2261,2012-03-03,3,6,1,8.66666698455811,46.3333320617676,5,60,3,2,213,80,1,1,1,1,0,10,5
12382,2261,2012-03-03,4,6,1,39,20.6000003814697,0.400000005960464,60,3,2,213,80,1,1,1,1,0,1
12383,2261,2012-03-04,1,0,1,20,33.3333320617676,6.66666650772095,60,3,2,213,80,1,1,1,1,0,10,
12384,2261,2012-03-04,2,0,1,21.6666660308838,34,4.33333349227905,60,3,2,213,80,1,1,1,1,0,10,
12385,2261,2012-03-04,3,0,1,20,34.3333320617676,5.66666650772095,60,3,2,213,80,1,1,1,1,0,10,
12386,2261,2012-03-04,4,0,1,43.6666679382324,16,0.333333343267441,60,3,2,213,80,1,1,1,1,0,1
12387,2262,2012-02-10,0,5,0,13,20.5,4.5,38,2,2,213,76,0,1,0,1,0,10,5040.680316,5190.013031,11.
12388,2262,2012-02-10,1,5,0,35.25,9.75,5.5,50.5,3,2,213,76,0,1,0,1,0,10,5040.680316,5190.01303
12389,2262,2012-02-10,2,5,0,42.6666679382324,17,0.333333343267441,60,3,2,213,76,0,1,0,1,0,1
12390,2262,2012-02-10,3,5,0,46.3333320617676,13,0.666666686534882,60,3,2,213,76,0,1,0,1,0,1
12391,2262,2012-02-10,4,5,0,43.5,9,0.5,53,2,2,213,76,0,1,0,1,0,10,5040.680316,5190.013031,11.7
12392,2262,2012-02-11,1,6,1,44.75,14.25,0.25,59.25,3,2,213,76,0,1,0,1,0,10,5040.680316,5190.01
12393,2262,2012-02-11,2,6,1,44.4000015258789,14.6000003814697,1,60,3,2,213,76,0,1,0,1,0,10,5
12394,2262,2012-02-11,3,6,1,35.6666679382324,24.3333339691162,0,60,3,2,213,76,0,1,0,1,0,10,5
12395,2262,2012-02-11,4,6,1,51.6666679382324,8.33333301544189,0,60,3,2,213,76,0,1,0,1,0,10,5
12396,2262,2012-02-12,2,0,1,42,11,7,60,1,2,213,76,0,1,0,1,0,10,5040.680316,5190.013031,11.7500
12397,2262,2012-02-12,3,0,1,35,17.6666660308838,7.33333349227905,60,3,2,213,76,0,1,0,1,0,10,
12398,2262,2012-02-12,4,0,1,48.3333320617676,11.6666669845581,0,60,3,2,213,76,0,1,0,1,0,10,5
12399,2262,2012-03-04,0,0,1,54,5,1,60,1,2,213,76,0,1,0,1,0,10,5040.680316,5190.013031,11.7500

12400,2262,2012-03-04,1,0,1,27,32.5,0.5,60,2,2,213,76,0,1,0,1,0,10,5040.680316,5190.013031,11.7
12401,2262,2012-03-05,0,1,0,0,1,0,1,1,2,213,76,0,1,0,1,0,10,5040.680316,5190.013031,11.7500813
12402,2262,2012-03-05,1,1,0,38.5,11.75,0.25,50.5,3,2,213,76,0,1,0,1,0,10,5040.680316,5190.01303
12403,2262,2012-03-05,2,1,0,45.5999984741211,14.3999996185303,0,60,3,2,213,76,0,1,0,1,0,10,5
12404,2262,2012-03-05,3,1,0,39,21,0,60,3,2,213,76,0,1,0,1,0,10,5040.680316,5190.013031,11.7500
12405,2262,2012-03-05,4,1,0,50.6666679382324,9.33333301544189,0,60,3,2,213,76,0,1,0,1,0,10,5
12406,2262,2012-03-06,0,2,0,41,5,0,46,1,2,213,76,0,1,0,1,0,10,5040.680316,5190.013031,11.75008
12407,2262,2012-03-06,1,2,0,31.6666660308838,27,1.33333337306976,60,3,2,213,76,0,1,0,1,0,10,
12408,2262,2012-03-06,2,2,0,43.5,16.5,0,60,3,2,213,76,0,1,0,1,0,10,5040.680316,5190.013031,11.7
12409,2262,2012-03-06,3,2,0,32.2000007629395,27,0.800000011920929,60,3,2,213,76,0,1,0,1,0,10
12410,2262,2012-03-06,4,2,0,52.6666679382324,7.33333349227905,0,60,3,2,213,76,0,1,0,1,0,10,5
12411,2262,2012-03-07,0,3,0,42,0,0,42,1,2,213,76,0,1,0,1,0,10,5040.680316,5190.013031,11.75008
12412,2262,2012-03-07,1,3,0,43.6666679382324,16,0.333333343267441,60,3,2,213,76,0,1,0,1,0,10
12413,2262,2012-03-07,2,3,0,46.3333320617676,13.3333330154419,0.333333343267441,60,3,2,21
12414,2262,2012-03-07,3,3,0,26,33.5,0.5,60,3,2,213,76,0,1,0,1,0,10,5040.680316,5190.013031,11.7
12415,2262,2012-03-07,4,3,0,40.8333320617676,19.1666660308838,0,60,3,2,213,76,0,1,0,1,0,10,5
12416,2266,2011-11-01,1,2,0,38.5,20.5,1,60,2,2,213,71,1,1,0,1,0,12,3842.949796,4261.47191,8.397
12417,2266,2011-11-01,2,2,0,25.6666660308838,34.3333320617676,0,60,3,2,213,71,1,1,0,1,0,12,3
12418,2266,2011-11-01,3,2,0,29,25,6,60,3,2,213,71,1,1,0,1,0,12,3842.949796,4261.47191,8.397895
12419,2266,2011-11-01,4,2,0,9.66666698455811,45,5.33333349227905,60,3,2,213,71,1,1,0,1,0,12,
12420,2266,2012-02-12,1,0,1,0,3,2,5,1,2,213,71,1,1,0,1,0,12,3842.949796,4261.47191,8.397895312
12421,2266,2012-02-12,2,0,1,10.3333330154419,34.3333320617676,9.33333301544189,54,3,2,213
12422,2266,2012-02-12,3,0,1,23,37,0,60,1,2,213,71,1,1,0,1,0,12,3842.949796,4261.47191,8.397895
12423,2266,2012-02-13,1,1,0,18.5,31,1.5,51,2,2,213,71,1,1,0,1,0,12,3842.949796,4261.47191,8.397
12424,2266,2012-02-13,2,1,0,26.3333339691162,32.3333320617676,1.33333337306976,60,3,2,213
12425,2266,2012-02-13,3,1,0,41.7999992370605,17.3999996185303,0.800000011920929,60,3,2,21
12426,2266,2012-02-13,4,1,0,23.75,32,4.25,60,3,2,213,71,1,1,0,1,0,12,3842.949796,4261.47191,8.3
12427,2266,2012-02-14,0,2,0,28,32,0,60,1,2,213,71,1,1,0,1,0,12,3842.949796,4261.47191,8.397895
12428,2266,2012-02-14,1,2,0,12,23.5,1,36.5,2,2,213,71,1,1,0,1,0,12,3842.949796,4261.47191,8.397
12429,2266,2012-02-14,2,2,0,15,45,0,60,3,2,213,71,1,1,0,1,0,12,3842.949796,4261.47191,8.397895
12430,2266,2012-02-14,3,2,0,30.6666660308838,27.3333339691162,2,60,3,2,213,71,1,1,0,1,0,12,3
12431,2266,2012-02-14,4,2,0,30.6000003814697,28.2000007629395,1.20000004768372,60,3,2,213
12432,2266,2012-02-15,0,3,0,17.5,15.5,1,34,2,2,213,71,1,1,0,1,0,12,3842.949796,4261.47191,8.397
12433,2266,2012-02-15,1,3,0,10.6666669845581,48,1.33333337306976,60,3,2,213,71,1,1,0,1,0,12,
12434,2266,2012-02-15,2,3,0,24,35,1,60,3,2,213,71,1,1,0,1,0,12,3842.949796,4261.47191,8.397895
12435,2266,2012-02-15,3,3,0,18.6666660308838,38.6666679382324,2.66666674613953,60,3,2,213
12436,2266,2012-02-15,4,3,0,20,39.6666679382324,0.333333343267441,60,3,2,213,71,1,1,0,1,0,12
12437,2266,2012-02-16,1,4,0,14.5,31.75,2.25,48.5,3,2,213,71,1,1,0,1,0,12,3842.949796,4261.47191
12438,2266,2012-02-16,2,4,0,18.6666660308838,33.3333320617676,8,60,3,2,213,71,1,1,0,1,0,12,3
12439,2266,2012-02-16,3,4,0,14.3333330154419,30,15.6666669845581,60,3,2,213,71,1,1,0,1,0,12,
12440,2266,2012-02-16,4,4,0,15.6666669845581,40,2,57.6666679382324,3,2,213,71,1,1,0,1,0,12,3
12441,2270,2011-11-01,0,2,0,46,11,3,60,1,2,213,77,1,1,0,0,0,7,4004.197693,4565.006347,7.85556
12442,2270,2011-11-01,1,2,0,39,20.3333339691162,0.666666686534882,60,3,2,213,77,1,1,0,0,0,7,
12443,2270,2011-11-01,2,2,0,27,33,0,60,1,2,213,77,1,1,0,0,0,7,4004.197693,4565.006347,7.85556
12444,2270,2011-11-02,1,3,0,32.3333320617676,26.3333339691162,0,58.6666679382324,3,2,213,
12445,2270,2011-11-02,2,3,0,36.4000015258789,23,0.600000023841858,60,3,2,213,77,1,1,0,0,0,7,
12446,2270,2011-11-02,3,3,0,28.75,31.25,0,60,3,2,213,77,1,1,0,0,0,7,4004.197693,4565.006347,7.8
12447,2270,2011-11-02,4,3,0,37.3333320617676,22.3333339691162,0.333333343267441,60,3,2,21
12448,2270,2011-11-03,1,4,0,27.3333339691162,19.3333339691162,0.333333343267441,47,3,2,21
12449,2270,2011-11-03,2,4,0,37,22.6666660308838,0.333333343267441,60,3,2,213,77,1,1,0,0,0,7,

12450,2270,2011-11-03,3,4,0,38.4000015258789,21.3999996185303,0.200000002980232,60,3,2,21
12451,2270,2011-11-03,4,4,0,35.75,24.25,0,60,3,2,213,77,1,1,0,0,0,7,4004.197693,4565.006347,7.8
12452,2270,2011-11-04,0,5,0,4,16,1,21,1,2,213,77,1,1,0,0,0,7,4004.197693,4565.006347,7.855560
12453,2270,2011-11-04,1,5,0,32.3333320617676,27.3333339691162,0.333333343267441,60,3,2,21
12454,2270,2011-11-04,2,5,0,34.6666679382324,25.3333339691162,0,60,3,2,213,77,1,1,0,0,0,7,40
12455,2270,2011-11-04,3,5,0,30,29.6666660308838,0.333333343267441,60,3,2,213,77,1,1,0,0,0,7,
12456,2270,2011-11-04,4,5,0,40.5,19.5,0,60,3,2,213,77,1,1,0,0,0,7,4004.197693,4565.006347,7.855
12457,2270,2011-11-05,0,6,1,0,2,0,2,1,2,213,77,1,1,0,0,0,7,4004.197693,4565.006347,7.85556098
12458,2270,2011-11-05,1,6,1,29,30.6666660308838,0.333333343267441,60,3,2,213,77,1,1,0,0,0,7,
12459,2270,2011-11-05,2,6,1,30.3333339691162,29.6666660308838,0,60,3,2,213,77,1,1,0,0,0,7,40
12460,2270,2011-11-05,3,6,1,24.3333339691162,35.6666679382324,0,60,3,2,213,77,1,1,0,0,0,7,40
12461,2270,2011-11-05,4,6,1,38.75,21.25,0,60,3,2,213,77,1,1,0,0,0,7,4004.197693,4565.006347,7.8
12462,2270,2011-11-06,0,0,1,8,22.5,0.5,31,2,2,213,77,1,1,0,0,0,7,4004.197693,4565.006347,7.855
12463,2270,2011-11-06,1,0,1,32.7999992370605,27.2000007629395,0,60,3,2,213,77,1,1,0,0,0,7,40
12464,2270,2011-11-06,2,0,1,38,22,0,60,3,2,213,77,1,1,0,0,0,7,4004.197693,4565.006347,7.85556
12465,2270,2011-11-06,3,0,1,30,30,0,60,3,2,213,77,1,1,0,0,0,7,4004.197693,4565.006347,7.85556
12466,2270,2011-11-06,4,0,1,35,25,0,60,3,2,213,77,1,1,0,0,0,7,4004.197693,4565.006347,7.85556
12467,2270,2011-11-07,1,1,0,26,34,0,60,1,2,213,77,1,1,0,0,0,7,4004.197693,4565.006347,7.85556
12468,2270,2011-11-07,2,1,0,33,26.6666660308838,0.333333343267441,60,3,2,213,77,1,1,0,0,0,7,
12469,2270,2011-11-07,3,1,0,32.6666679382324,26.3333339691162,1,60,3,2,213,77,1,1,0,0,0,7,40
12470,2270,2011-11-07,4,1,0,52.3333320617676,7.33333349227905,0.333333343267441,60,3,2,21
12471,2271,2011-10-28,0,5,0,11.5,23,0.5,35,2,2,213,84,1,1,0,0,0,12,3636.179789,4007.877029,7.55
12472,2271,2011-10-28,1,5,0,32.3333320617676,27.6666660308838,0,60,3,2,213,84,1,1,0,0,0,12,3
12473,2271,2011-10-28,2,5,0,24.6666660308838,32,3.33333325386047,60,3,2,213,84,1,1,0,0,0,12,
12474,2271,2011-10-28,3,5,0,31.3333339691162,25,3.66666674613953,60,3,2,213,84,1,1,0,0,0,12,
12475,2271,2011-10-28,4,5,0,44.5,15.5,0,60,3,2,213,84,1,1,0,0,0,12,3636.179789,4007.877029,7.55
12476,2271,2011-10-29,0,6,1,11,18,5,34,1,2,213,84,1,1,0,0,0,12,3636.179789,4007.877029,7.5543
12477,2271,2011-10-29,1,6,1,33.6666679382324,24.6666660308838,1.66666662693024,60,3,2,213
12478,2271,2011-10-29,2,6,1,37.3333320617676,22,0.666666686534882,60,3,2,213,84,1,1,0,0,0,12
12479,2271,2011-10-29,3,6,1,27.5,10.5,0,38,2,2,213,84,1,1,0,0,0,12,3636.179789,4007.877029,7.55
12480,2271,2011-10-29,4,6,1,42,17,1,60,3,2,213,84,1,1,0,0,0,12,3636.179789,4007.877029,7.5543
12481,2271,2011-10-30,0,0,1,1,29,1,31,2,2,213,84,1,1,0,0,0,12,3636.179789,4007.877029,7.554347
12482,2271,2011-10-30,1,0,1,29.6666660308838,28.3333339691162,2,60,3,2,213,84,1,1,0,0,0,12,3
12483,2271,2011-10-30,2,0,1,34.6666679382324,23,2.33333325386047,60,3,2,213,84,1,1,0,0,0,12,
12484,2271,2011-10-30,3,0,1,38.3333320617676,15.6666669845581,6,60,3,2,213,84,1,1,0,0,0,12,3
12485,2271,2011-10-30,4,0,1,52.5,7.5,0,60,2,2,213,84,1,1,0,0,0,12,3636.179789,4007.877029,7.554
12486,2271,2011-10-31,4,1,0,33,27,0,60,2,2,213,84,1,1,0,0,0,12,3636.179789,4007.877029,7.5543
12487,2271,2011-11-01,0,2,0,22,38,0,60,1,2,213,84,1,1,0,0,0,12,3636.179789,4007.877029,7.5543
12488,2271,2011-11-01,1,2,0,25,35,0,60,3,2,213,84,1,1,0,0,0,12,3636.179789,4007.877029,7.5543
12489,2271,2011-11-01,2,2,0,26.7999992370605,33,0.200000002980232,60,3,2,213,84,1,1,0,0,0,12
12490,2271,2011-11-01,3,2,0,30.6666660308838,28,1.33333337306976,60,3,2,213,84,1,1,0,0,0,12,
12491,2271,2011-11-01,4,2,0,36.3333320617676,23.6666660308838,0,60,3,2,213,84,1,1,0,0,0,12,3
12492,2271,2011-11-02,0,3,0,17.5,26.5,0.5,44.5,2,2,213,84,1,1,0,0,0,12,3636.179789,4007.877029,
12493,2271,2011-11-02,1,3,0,31.3333339691162,23,5.66666650772095,60,3,2,213,84,1,1,0,0,0,12,
12494,2271,2011-11-02,2,3,0,42,18,0,60,3,2,213,84,1,1,0,0,0,12,3636.179789,4007.877029,7.5543
12495,2271,2011-11-02,3,3,0,33.7999992370605,26.2000007629395,0,60,3,2,213,84,1,1,0,0,0,12,3
12496,2271,2011-11-02,4,3,0,40,20,0,60,3,2,213,84,1,1,0,0,0,12,3636.179789,4007.877029,7.5543
12497,2271,2011-11-03,0,4,0,15,23.5,0.5,39,2,2,213,84,1,1,0,0,0,12,3636.179789,4007.877029,7.55
12498,2271,2011-11-03,1,4,0,20.6666660308838,38.3333320617676,1,60,3,2,213,84,1,1,0,0,0,12,3
12499,2271,2011-11-03,2,4,0,49,11,0,60,3,2,213,84,1,1,0,0,0,12,3636.179789,4007.877029,7.5543

12500,2271,2011-11-03,3,4,0,22,28.5,9.5,60,3,2,213,84,1,1,0,0,0,12,3636.179789,4007.877029,7.55
12501,2271,2011-11-03,4,4,0,35.6666679382324,24,0.333333343267441,60,3,2,213,84,1,1,0,0,0,12
12502,2271,2011-11-07,0,1,0,3,28,1,32,1,2,213,84,1,1,0,0,0,12,3636.179789,4007.877029,7.554347
12503,2271,2011-11-07,1,1,0,27.3333339691162,31,1.66666662693024,60,3,2,213,84,1,1,0,0,0,12,
12504,2271,2011-11-07,2,1,0,44,16,0,60,1,2,213,84,1,1,0,0,0,12,3636.179789,4007.877029,7.55434
12505,2272,2011-10-31,0,1,0,60,0,0,60,1,2,213,68,1,1,0,1,0,11,3977.776118,4443.472002,8.154075
12506,2272,2011-10-31,1,1,0,9.66666698455811,33.3333320617676,1.33333337306976,44.333332
12507,2272,2011-10-31,2,1,0,28,32,0,60,3,2,213,68,1,1,0,1,0,11,3977.776118,4443.472002,8.15407
12508,2272,2011-10-31,3,1,0,39,21,0,60,3,2,213,68,1,1,0,1,0,11,3977.776118,4443.472002,8.15407
12509,2272,2011-10-31,4,1,0,51,9,0,60,3,2,213,68,1,1,0,1,0,11,3977.776118,4443.472002,8.154075
12510,2272,2012-02-17,1,5,0,18,37.6666679382324,4,59.6666679382324,3,2,213,68,1,1,0,1,0,11,3
12511,2272,2012-02-17,2,5,0,28.75,31,0.25,60,3,2,213,68,1,1,0,1,0,11,3977.776118,4443.472002,8
12512,2272,2012-02-17,3,5,0,20.3333339691162,37.6666679382324,2,60,3,2,213,68,1,1,0,1,0,11,3
12513,2272,2012-02-17,4,5,0,38.6666679382324,21.3333339691162,0,60,3,2,213,68,1,1,0,1,0,11,3
12514,2272,2012-02-18,1,6,1,15,14,2,31,1,2,213,68,1,1,0,1,0,11,3977.776118,4443.472002,8.15407
12515,2272,2012-02-18,2,6,1,27.2000007629395,31.2000007629395,1.60000002384186,60,3,2,213
12516,2272,2012-02-18,3,6,1,20.5,36,3.5,60,3,2,213,68,1,1,0,1,0,11,3977.776118,4443.472002,8.15
12517,2272,2012-02-18,4,6,1,22.6000003814697,33.7999992370605,3.59999990463257,60,3,2,213
12518,2272,2012-02-19,1,0,1,13.5,27.5,1,42,2,2,213,68,1,1,0,1,0,11,3977.776118,4443.472002,8.15
12519,2272,2012-02-19,2,0,1,11,47.6666679382324,1.33333337306976,60,3,2,213,68,1,1,0,1,0,11,
12520,2272,2012-02-19,3,0,1,15.6666669845581,43.3333320617676,1,60,3,2,213,68,1,1,0,1,0,11,3
12521,2272,2012-02-19,4,0,1,46,13.25,0.75,60,3,2,213,68,1,1,0,1,0,11,3977.776118,4443.472002,8
12522,2272,2012-02-20,1,1,0,10.75,29.25,3.5,43.5,3,2,213,68,1,1,0,1,0,11,3977.776118,4443.47200
12523,2272,2012-02-20,2,1,0,8.39999961853027,46.7999992370605,4.80000019073486,60,3,2,213
12524,2272,2012-02-20,3,1,0,38.6666679382324,14.6666669845581,6.66666650772095,60,3,2,213
12525,2272,2012-02-20,4,1,0,47,12.3333330154419,0.666666686534882,60,3,2,213,68,1,1,0,1,0,1
12526,2272,2012-02-21,2,2,0,22,37,1,60,1,2,213,68,1,1,0,1,0,11,3977.776118,4443.472002,8.15407
12527,2272,2012-02-21,3,2,0,24.6666660308838,33.3333320617676,2,60,3,2,213,68,1,1,0,1,0,11,3
12528,2272,2012-02-21,4,2,0,42.3333320617676,17.3333339691162,0.333333343267441,60,3,2,2
12529,2274,2011-11-01,1,2,0,21.5,26,4.5,52,3,2,213,70,0,1,0,1,0,12,3679.903175,3955.17504,7.571
12530,2274,2011-11-01,2,2,0,30.3333339691162,27.1666660308838,2.5,60,3,2,213,70,0,1,0,1,0,12
12531,2274,2011-11-01,3,2,0,22,33.25,4.75,60,3,2,213,70,0,1,0,1,0,12,3679.903175,3955.17504,7.5
12532,2274,2011-11-01,4,2,0,31.3333339691162,25.6666660308838,3,60,3,2,213,70,0,1,0,1,0,12,3
12533,2274,2011-11-02,1,3,0,5.33333349227905,27.6666660308838,9.66666698455811,42.666667
12534,2274,2011-11-02,2,3,0,6.66666650772095,29.6666660308838,23.6666660308838,60,3,2,213
12535,2274,2011-11-02,3,3,0,9.80000019073486,40.7999992370605,9.39999961853027,60,3,2,213
12536,2274,2011-11-02,4,3,0,31.5,27.8333339691162,0.666666686534882,60,3,2,213,70,0,1,0,1,0,
12537,2274,2011-11-03,1,4,0,5.33333349227905,33.6666679382324,20.3333339691162,59.333332
12538,2274,2011-11-03,2,4,0,15.3333330154419,33,11.6666669845581,60,3,2,213,70,0,1,0,1,0,12,
12539,2274,2011-11-03,3,4,0,16.3333339691162,25.3333339691162,18.3333339691162,60,3,2,213
12540,2274,2011-11-03,4,4,0,28.6666660308838,26.6666660308838,4.66666650772095,60,3,2,213
12541,2274,2012-02-21,1,2,0,14.3333330154419,35.3333320617676,8.66666698455811,58.333332
12542,2274,2012-02-21,2,2,0,17,30,13,60,3,2,213,70,0,1,0,1,0,12,3679.903175,3955.17504,7.5710
12543,2274,2012-02-21,3,2,0,1.5,36,22.5,60,2,2,213,70,0,1,0,1,0,12,3679.903175,3955.17504,7.571
12544,2274,2012-02-22,1,3,0,29,23.75,2,54.75,3,2,213,70,0,1,0,1,0,12,3679.903175,3955.17504,7.5
12545,2274,2012-02-22,2,3,0,21.6666660308838,37,1.33333337306976,60,3,2,213,70,0,1,0,1,0,12,
12546,2274,2012-02-22,3,3,0,14.75,39.75,5.5,60,3,2,213,70,0,1,0,1,0,12,3679.903175,3955.17504,7
12547,2274,2012-02-22,4,3,0,23,35.3333320617676,1.66666662693024,60,3,2,213,70,0,1,0,1,0,12,
12548,2274,2012-02-23,1,4,0,18.6666660308838,22,1,41.6666679382324,3,2,213,70,0,1,0,1,0,12,3
12549,2274,2012-02-23,2,4,0,31,28.3333339691162,0.666666686534882,60,3,2,213,70,0,1,0,1,0,12

12550,2274,2012-02-23,3,4,0,24.6666660308838,33.6666679382324,1.66666662693024,60,3,2,213
12551,2274,2012-02-23,4,4,0,28.6666660308838,30.3333339691162,1,60,3,2,213,70,0,1,0,1,0,12,3
12552,2275,2011-10-28,1,5,0,26,31.75,1.5,59.25,3,2,213,72,0,1,0,1,0,12,3842.949796,4261.47191,8
12553,2275,2011-10-28,2,5,0,12.3999996185303,44.2000007629395,3.40000009536743,60,3,2,213
12554,2275,2011-10-28,3,5,0,14.3333330154419,41.3333320617676,4.33333349227905,60,3,2,213
12555,2275,2011-10-28,4,5,0,33.3333320617676,26.6666660308838,0,60,3,2,213,72,0,1,0,1,0,12,3
12556,2275,2011-10-29,0,6,1,8.5,22,0,30.5,2,2,213,72,0,1,0,1,0,12,3842.949796,4261.47191,8.3978
12557,2275,2011-10-29,1,6,1,16,43.3333320617676,0.666666686534882,60,3,2,213,72,0,1,0,1,0,12
12558,2275,2011-10-29,2,6,1,29.25,29.75,1,60,3,2,213,72,0,1,0,1,0,12,3842.949796,4261.47191,8.3
12559,2275,2011-10-29,3,6,1,16.3999996185303,30,13.6000003814697,60,3,2,213,72,0,1,0,1,0,12,
12560,2275,2011-10-29,4,6,1,39.6666679382324,20,0.333333343267441,60,3,2,213,72,0,1,0,1,0,12
12561,2275,2011-10-30,0,0,1,11,20,0,31,1,2,213,72,0,1,0,1,0,12,3842.949796,4261.47191,8.397895
12562,2275,2011-10-30,1,0,1,15.6666669845581,42.3333320617676,2,60,3,2,213,72,0,1,0,1,0,12,3
12563,2275,2011-10-30,2,0,1,33.6666679382324,26.3333339691162,0,60,3,2,213,72,0,1,0,1,0,12,3
12564,2275,2011-10-30,3,0,1,26.25,31.5,2.25,60,3,2,213,72,0,1,0,1,0,12,3842.949796,4261.47191,8
12565,2275,2011-10-30,4,0,1,45.2000007629395,14.8000001907349,0,60,3,2,213,72,0,1,0,1,0,12,3
12566,2275,2011-10-31,0,1,0,20.6666660308838,27,1,48.6666679382324,3,2,213,72,0,1,0,1,0,12,3
12567,2275,2011-10-31,1,1,0,16,43,1,60,3,2,213,72,0,1,0,1,0,12,3842.949796,4261.47191,8.397895
12568,2275,2011-10-31,2,1,0,31.3333339691162,21.6666660308838,7,60,3,2,213,72,0,1,0,1,0,12,3
12569,2275,2011-10-31,3,1,0,23,23.6666660308838,13.3333330154419,60,3,2,213,72,0,1,0,1,0,12,
12570,2275,2011-10-31,4,1,0,36.75,22.75,0.5,60,3,2,213,72,0,1,0,1,0,12,3842.949796,4261.47191,8
12571,2275,2011-11-01,0,2,0,17.5,14.5,0,32,2,2,213,72,0,1,0,1,0,12,3842.949796,4261.47191,8.397
12572,2275,2011-11-01,1,2,0,13.8000001907349,42.7999992370605,3.40000009536743,60,3,2,213
12573,2275,2011-11-01,2,2,0,15.6666669845581,39.6666679382324,4.66666650772095,60,3,2,213
12574,2275,2011-11-01,3,2,0,22,37,1,60,3,2,213,72,0,1,0,1,0,12,3842.949796,4261.47191,8.397895
12575,2275,2011-11-01,4,2,0,38,22,0,60,3,2,213,72,0,1,0,1,0,12,3842.949796,4261.47191,8.397895
12576,2275,2011-11-02,1,3,0,23,35,2,60,1,2,213,72,0,1,0,1,0,12,3842.949796,4261.47191,8.397895
12577,2275,2011-11-02,2,3,0,18,40.3333320617676,1.66666662693024,60,3,2,213,72,0,1,0,1,0,12,
12578,2275,2011-11-02,3,3,0,14.6666669845581,43,2.33333325386047,60,3,2,213,72,0,1,0,1,0,12,
12579,2275,2011-11-02,4,3,0,41.6666679382324,18,0.333333343267441,60,3,2,213,72,0,1,0,1,0,12
12580,2275,2011-11-03,0,4,0,53,6,1,60,1,2,213,72,0,1,0,1,0,12,3842.949796,4261.47191,8.3978953
12581,2275,2011-11-03,1,4,0,22,36,2,60,1,2,213,72,0,1,0,1,0,12,3842.949796,4261.47191,8.397895
12582,2276,2011-10-29,0,6,1,27,18.5,0,45.5,2,2,213,82,0,1,0,1,0,8,3613.639402,3969.628686,7.546
12583,2276,2011-10-29,1,6,1,27.6666660308838,28.6666660308838,3.66666674613953,60,3,2,213
12584,2276,2011-10-29,2,6,1,30.3333339691162,29.6666660308838,0,60,3,2,213,82,0,1,0,1,0,8,36
12585,2276,2011-10-29,3,6,1,28.6666660308838,31.3333339691162,0,60,3,2,213,82,0,1,0,1,0,8,36
12586,2276,2011-10-29,4,6,1,40,20,0,60,3,2,213,82,0,1,0,1,0,8,3613.639402,3969.628686,7.546676
12587,2276,2011-10-30,0,0,1,12,16,2.66666674613953,30.6666660308838,3,2,213,82,0,1,0,1,0,8,3
12588,2276,2011-10-30,1,0,1,30,27.6666660308838,2.33333325386047,60,3,2,213,82,0,1,0,1,0,8,3
12589,2276,2011-10-30,2,0,1,23.6666660308838,36,0.333333343267441,60,3,2,213,82,0,1,0,1,0,8,
12590,2276,2011-10-30,3,0,1,28,32,0,60,3,2,213,82,0,1,0,1,0,8,3613.639402,3969.628686,7.546676
12591,2276,2011-10-30,4,0,1,37.25,22.75,0,60,3,2,213,82,0,1,0,1,0,8,3613.639402,3969.628686,7.5
12592,2276,2011-10-31,0,1,0,21,21,0.333333343267441,42.3333320617676,3,2,213,82,0,1,0,1,0,8,
12593,2276,2011-10-31,1,1,0,35.2000007629395,24.2000007629395,0.600000023841858,60,3,2,21
12594,2276,2011-10-31,2,1,0,33.3333320617676,26.6666660308838,0,60,3,2,213,82,0,1,0,1,0,8,36
12595,2276,2011-10-31,3,1,0,25.6666660308838,34,0.333333343267441,60,3,2,213,82,0,1,0,1,0,8,
12596,2276,2011-10-31,4,1,0,37.3333320617676,22.6666660308838,0,60,3,2,213,82,0,1,0,1,0,8,36
12597,2276,2011-11-01,1,2,0,9,50,1,60,1,2,213,82,0,1,0,1,0,8,3613.639402,3969.628686,7.5466766
12598,2276,2011-11-01,2,2,0,39,21,0,60,3,2,213,82,0,1,0,1,0,8,3613.639402,3969.628686,7.546676
12599,2276,2011-11-01,3,2,0,34.3333320617676,25.3333339691162,0.333333343267441,60,3,2,21

12600,2276,2011-11-01,4,2,0,13,28.3333339691162,0,41.3333320617676,3,2,213,82,0,1,0,1,0,8,36
12601,2276,2011-11-02,0,3,0,25,31,4,60,1,2,213,82,0,1,0,1,0,8,3613.639402,3969.628686,7.54667
12602,2276,2011-11-02,1,3,0,47.6666679382324,12,0.333333343267441,60,3,2,213,82,0,1,0,1,0,8,
12603,2276,2011-11-02,2,3,0,34,26,0,60,2,2,213,82,0,1,0,1,0,8,3613.639402,3969.628686,7.54667
12604,2276,2011-11-03,0,4,0,15.5,18,0,33.5,2,2,213,82,0,1,0,1,0,8,3613.639402,3969.628686,7.54
12605,2276,2011-11-03,1,4,0,36.3333320617676,23.6666660308838,0,60,3,2,213,82,0,1,0,1,0,8,36
12606,2276,2011-11-03,2,4,0,37.75,22,0.25,60,3,2,213,82,0,1,0,1,0,8,3613.639402,3969.628686,7.5
12607,2276,2011-11-03,3,4,0,25,34.75,0.25,60,3,2,213,82,0,1,0,1,0,8,3613.639402,3969.628686,7.5
12608,2276,2011-11-03,4,4,0,38.6666679382324,21.3333339691162,0,60,3,2,213,82,0,1,0,1,0,8,36
12609,2276,2011-11-04,0,5,0,20,28,0,48,1,2,213,82,0,1,0,1,0,8,3613.639402,3969.628686,7.54667
12610,2276,2011-11-04,1,5,0,38.6666679382324,21.3333339691162,0,60,3,2,213,82,0,1,0,1,0,8,36
12611,2276,2011-11-04,2,5,0,37.6666679382324,22.3333339691162,0,60,3,2,213,82,0,1,0,1,0,8,36
12612,2276,2011-11-04,3,5,0,27.2000007629395,32.2000007629395,0.600000023841858,60,3,2,2
12613,2276,2011-11-04,4,5,0,34.25,25.5,0.25,60,3,2,213,82,0,1,0,1,0,8,3613.639402,3969.628686,
12614,2278,2011-11-01,0,2,0,60,0,0,60,1,2,213,67,1,1,0,1,0,12,3744.904747,4134.503682,7.53781
12615,2278,2011-11-01,1,2,0,25.3333339691162,19,15.6666669845581,60,3,2,213,67,1,1,0,1,0,12,
12616,2278,2011-11-01,2,2,0,7,36.5,16.5,60,2,2,213,67,1,1,0,1,0,12,3744.904747,4134.503682,7.5
12617,2278,2011-11-02,0,3,0,27,1,1,29,1,2,213,67,1,1,0,1,0,12,3744.904747,4134.503682,7.53781
12618,2278,2011-11-02,1,3,0,11,47.6666679382324,1.33333337306976,60,3,2,213,67,1,1,0,1,0,12,
12619,2278,2011-11-02,2,3,0,17,42.25,0.75,60,3,2,213,67,1,1,0,1,0,12,3744.904747,4134.503682,
12620,2278,2011-11-02,3,3,0,28.3333339691162,26,5.66666650772095,60,3,2,213,67,1,1,0,1,0,12,
12621,2278,2011-11-02,4,3,0,42.6666679382324,17.3333339691162,0,60,3,2,213,67,1,1,0,1,0,12,3
12622,2278,2011-11-03,0,4,0,0,4,0,4,1,2,213,67,1,1,0,1,0,12,3744.904747,4134.503682,7.5378157
12623,2278,2011-11-03,1,4,0,16,44,0,60,3,2,213,67,1,1,0,1,0,12,3744.904747,4134.503682,7.5378
12624,2278,2011-11-03,2,4,0,26.3333339691162,33.6666679382324,0,60,3,2,213,67,1,1,0,1,0,12,3
12625,2278,2011-11-03,3,4,0,36.5999984741211,14,0.800000011920929,51.4000015258789,3,2,2
12626,2278,2011-11-03,4,4,0,17,33.6666679382324,1.66666662693024,52.3333320617676,3,2,21
12627,2278,2011-11-04,0,5,0,1,2,1,4,1,2,213,67,1,1,0,1,0,12,3744.904747,4134.503682,7.5378157
12628,2278,2011-11-04,1,5,0,15,33.6666679382324,5.66666650772095,54.3333320617676,3,2,21
12629,2278,2011-11-04,2,5,0,16,43,1,60,3,2,213,67,1,1,0,1,0,12,3744.904747,4134.503682,7.5378
12630,2278,2011-11-04,3,5,0,15.6666669845581,39.3333320617676,5,60,3,2,213,67,1,1,0,1,0,12,3
12631,2278,2011-11-04,4,5,0,27.3333339691162,31.8333339691162,0.833333313465118,60,3,2,2
12632,2278,2011-11-05,1,6,1,19.1666660308838,37,3.5,59.6666679382324,3,2,213,67,1,1,0,1,0,12
12633,2278,2011-11-05,2,6,1,18.6666660308838,41.3333320617676,0,60,3,2,213,67,1,1,0,1,0,12,3
12634,2278,2011-11-05,3,6,1,21.3333339691162,22.6666660308838,16,60,3,2,213,67,1,1,0,1,0,12,
12635,2278,2011-11-05,4,6,1,40,19.6666660308838,0.333333343267441,60,3,2,213,67,1,1,0,1,0,1
12636,2278,2011-11-06,1,0,1,5.33333349227905,34.6666679382324,5.33333349227905,45.33333
12637,2278,2011-11-06,2,0,1,13.8333330154419,45.5,0.666666686534882,60,3,2,213,67,1,1,0,1,0,
12638,2278,2011-11-06,3,0,1,29.75,29.5,0.75,60,3,2,213,67,1,1,0,1,0,12,3744.904747,4134.503682
12639,2278,2011-11-06,4,0,1,23.6666660308838,36,0.333333343267441,60,3,2,213,67,1,1,0,1,0,1
12640,2278,2011-11-13,3,0,1,24,36,0,60,2,2,213,67,1,1,0,1,0,12,3744.904747,4134.503682,7.5378
12641,2278,2011-11-13,4,0,1,19.5,38,0,57.5,2,2,213,67,1,1,0,1,0,12,3744.904747,4134.503682,7.5
12642,2282,2011-11-13,0,0,1,56,4,0,60,1,2,213,84,0,1,0,1,0,11,3744.904747,4134.503682,7.53781
12643,2282,2011-11-13,1,0,1,31.3333339691162,27.6666660308838,1,60,3,2,213,84,0,1,0,1,0,11,3
12644,2282,2011-11-13,2,0,1,45,15,0,60,3,2,213,84,0,1,0,1,0,11,3744.904747,4134.503682,7.5378
12645,2282,2011-11-13,3,0,1,53.6666679382324,6.33333349227905,0,60,3,2,213,84,0,1,0,1,0,11,3
12646,2282,2011-11-14,0,1,0,4,21,4,29,1,2,213,84,0,1,0,1,0,11,3744.904747,4134.503682,7.53781
12647,2282,2011-11-14,1,1,0,34.6666679382324,25,0.333333343267441,60,3,2,213,84,0,1,0,1,0,1
12648,2282,2011-11-14,2,1,0,50,10,0,60,3,2,213,84,0,1,0,1,0,11,3744.904747,4134.503682,7.5378
12649,2282,2011-11-14,3,1,0,49.3333320617676,10.3333330154419,0.333333343267441,60,3,2,2

12650,2282,2011-11-14,4,1,0,51.8333320617676,8.16666698455811,0,60,3,2,213,84,0,1,0,1,0,11,3
12651,2282,2011-11-15,0,2,0,1.5,9.5,2.5,13.5,2,2,213,84,0,1,0,1,0,11,3744.904747,4134.503682,7.5
12652,2282,2011-11-15,1,2,0,31.25,27.75,1,60,3,2,213,84,0,1,0,1,0,11,3744.904747,4134.503682,7
12653,2282,2011-11-15,2,2,0,53.6666679382324,6.33333349227905,0,60,3,2,213,84,0,1,0,1,0,11,3
12654,2282,2011-11-15,3,2,0,51.3333320617676,8.66666698455811,0,60,3,2,213,84,0,1,0,1,0,11,3
12655,2282,2011-11-15,4,2,0,51.6666679382324,8.33333301544189,0,60,3,2,213,84,0,1,0,1,0,11,3
12656,2282,2011-11-16,1,3,0,26.3999996185303,23.2000007629395,0.800000011920929,50.40000
12657,2282,2011-11-16,2,3,0,48,12,0,60,3,2,213,84,0,1,0,1,0,11,3744.904747,4134.503682,7.53781
12658,2282,2011-11-16,3,3,0,29.6666660308838,16.3333339691162,14,60,3,2,213,84,0,1,0,1,0,11,
12659,2282,2011-11-16,4,3,0,55,5,0,60,3,2,213,84,0,1,0,1,0,11,3744.904747,4134.503682,7.537815
12660,2282,2011-11-17,0,4,0,3,11,0,14,1,2,213,84,0,1,0,1,0,11,3744.904747,4134.503682,7.537815
12661,2282,2011-11-17,1,4,0,16.3333339691162,39,4.66666650772095,60,3,2,213,84,0,1,0,1,0,11,
12662,2282,2011-11-17,2,4,0,44.5999984741211,13.6000003814697,1.79999995231628,60,3,2,213
12663,2282,2011-11-17,3,4,0,49.6666679382324,10,0.333333343267441,60,3,2,213,84,0,1,0,1,0,11
12664,2282,2011-11-17,4,4,0,45,13.3333330154419,1.66666662693024,60,3,2,213,84,0,1,0,1,0,11,
12665,2282,2011-11-18,0,5,0,0,16,4,20,1,2,213,84,0,1,0,1,0,11,3744.904747,4134.503682,7.537815
12666,2282,2011-11-18,1,5,0,27.3333339691162,30.6666660308838,2,60,3,2,213,84,0,1,0,1,0,11,3
12667,2282,2011-11-18,2,5,0,42.25,17.75,0,60,3,2,213,84,0,1,0,1,0,11,3744.904747,4134.503682,7
12668,2282,2011-11-18,3,5,0,44,16,0,60,3,2,213,84,0,1,0,1,0,11,3744.904747,4134.503682,7.53781
12669,2282,2011-11-18,4,5,0,50.6666679382324,9.33333301544189,0,60,3,2,213,84,0,1,0,1,0,11,3
12670,2282,2011-11-19,3,6,1,46.3333320617676,13.3333330154419,0.333333343267441,60,3,2,21
12671,2282,2011-11-19,4,6,1,46.6666679382324,13,0.333333343267441,60,3,2,213,84,0,1,0,1,0,11
12672,2286,2011-11-01,0,2,0,7.33333349227905,36.3333320617676,3,46.6666679382324,3,2,213,
12673,2286,2011-11-01,1,2,0,19.75,39.5,0.75,60,3,2,213,67,0,0,0,1,0,12,3698.034125,3974.195381
12674,2286,2011-11-01,2,2,0,36.3333320617676,21.3333339691162,2.33333325386047,60,3,2,213
12675,2286,2011-11-01,3,2,0,40.3333320617676,19.3333339691162,0.333333343267441,60,3,2,21
12676,2286,2011-11-01,4,2,0,44.3333320617676,15.6666669845581,0,60,3,2,213,67,0,0,0,1,0,12,3
12677,2286,2011-11-02,0,3,0,23,27,1.66666662693024,51.6666679382324,3,2,213,67,0,0,0,1,0,12,
12678,2286,2011-11-02,1,3,0,32,28,0,60,3,2,213,67,0,0,0,1,0,12,3698.034125,3974.195381,7.59750
12679,2286,2011-11-02,2,3,0,31,27.7999992370605,1.20000004768372,60,3,2,213,67,0,0,0,1,0,12,
12680,2286,2011-11-02,3,3,0,28.6666660308838,28.6666660308838,2.66666674613953,60,3,2,213
12681,2286,2011-11-02,4,3,0,39.3333320617676,20.6666660308838,0,60,3,2,213,67,0,0,0,1,0,12,3
12682,2286,2011-11-03,0,4,0,23.5,36,0.5,60,2,2,213,67,0,0,0,1,0,12,3698.034125,3974.195381,7.59
12683,2286,2011-11-03,1,4,0,29.6666660308838,27.6666660308838,2.66666674613953,60,3,2,213
12684,2286,2011-11-03,2,4,0,23,34.75,2.25,60,3,2,213,67,0,0,0,1,0,12,3698.034125,3974.195381,7
12685,2286,2011-11-03,3,4,0,26.3999996185303,32.5999984741211,1,60,3,2,213,67,0,0,0,1,0,12,3
12686,2286,2011-11-03,4,4,0,45,15,0,60,3,2,213,67,0,0,0,1,0,12,3698.034125,3974.195381,7.59750
12687,2286,2011-11-04,0,5,0,3.66666674613953,39.6666679382324,3.33333325386047,46.666667
12688,2286,2011-11-04,1,5,0,21.6666660308838,37.3333320617676,1,60,3,2,213,67,0,0,0,1,0,12,3
12689,2286,2011-11-04,2,5,0,15.6666669845581,39.6666679382324,4.66666650772095,60,3,2,213
12690,2286,2011-11-04,3,5,0,39.5,17.25,3.25,60,3,2,213,67,0,0,0,1,0,12,3698.034125,3974.195381
12691,2286,2011-11-04,4,5,0,43,16.5,0.5,60,3,2,213,67,0,0,0,1,0,12,3698.034125,3974.195381,7.59
12692,2286,2011-11-05,0,6,1,1,23,1,25,1,2,213,67,0,0,0,1,0,12,3698.034125,3974.195381,7.597507
12693,2286,2011-11-05,1,6,1,30.6666660308838,28.3333339691162,1,60,3,2,213,67,0,0,0,1,0,12,3
12694,2286,2011-11-05,2,6,1,30.3333339691162,29,0.666666686534882,60,3,2,213,67,0,0,0,1,0,12
12695,2286,2011-11-05,3,6,1,34,25.3333339691162,0.666666686534882,60,3,2,213,67,0,0,0,1,0,12
12696,2286,2011-11-05,4,6,1,40.7999992370605,19,0.200000002980232,60,3,2,213,67,0,0,0,1,0,12
12697,2286,2011-11-19,0,6,1,32.6666679382324,15.3333330154419,0.666666686534882,48.66666
12698,2286,2011-11-19,1,6,1,33,25.3333339691162,1.66666662693024,60,3,2,213,67,0,0,0,1,0,12,
12699,2286,2011-11-19,2,6,1,38.5,21.5,0,60,2,2,213,67,0,0,0,1,0,12,3698.034125,3974.195381,7.59

12700,2286,2011-11-24,0,4,0,21,19,1.5,41.5,2,2,213,67,0,0,0,1,0,12,3698.034125,3974.195381,7.59
12701,2286,2011-11-24,1,4,0,37,22.5,0.5,60,2,2,213,67,0,0,0,1,0,12,3698.034125,3974.195381,7.59
12702,2286,2011-11-24,2,4,0,34,19,7,60,1,2,213,67,0,0,0,1,0,12,3698.034125,3974.195381,7.59750
12703,2286,2011-11-24,3,4,0,17.6666660308838,38.3333320617676,4,60,3,2,213,67,0,0,0,1,0,12,3
12704,2286,2011-11-24,4,4,0,40,17.6666660308838,2.33333325386047,60,3,2,213,67,0,0,0,1,0,12,
12705,2287,2011-10-29,4,6,1,33,27,0,60,2,2,213,72,1,1,1,1,0,11,3929.917032,4531.717293,9.06842
12706,2287,2011-11-01,0,2,0,16.3333339691162,38.3333320617676,4,58.6666679382324,3,2,213,
12707,2287,2011-11-01,1,2,0,18.3333339691162,41.3333320617676,0.333333343267441,60,3,2,21
12708,2287,2011-11-01,2,2,0,30,26.6666660308838,3.33333325386047,60,3,2,213,72,1,1,1,1,0,11,
12709,2287,2011-11-01,3,2,0,19,38.75,2.25,60,3,2,213,72,1,1,1,1,0,11,3929.917032,4531.717293,9
12710,2287,2011-11-01,4,2,0,22.3333339691162,37.6666679382324,0,60,3,2,213,72,1,1,1,1,0,11,3
12711,2287,2011-11-02,0,3,0,10.5,43.5,4,58,2,2,213,72,1,1,1,1,0,11,3929.917032,4531.717293,9.06
12712,2287,2011-11-02,1,3,0,25.3333339691162,33.3333320617676,1.33333337306976,60,3,2,213
12713,2287,2011-11-02,2,3,0,17,39,4,60,3,2,213,72,1,1,1,1,0,11,3929.917032,4531.717293,9.06842
12714,2287,2011-11-02,3,3,0,19.75,40,0.25,60,3,2,213,72,1,1,1,1,0,11,3929.917032,4531.717293,9
12715,2287,2011-11-02,4,3,0,29.6666660308838,30.3333339691162,0,60,3,2,213,72,1,1,1,1,0,11,3
12716,2287,2011-11-03,0,4,0,13.3333330154419,39.3333320617676,1,53.6666679382324,3,2,213,
12717,2287,2011-11-03,1,4,0,25.3333339691162,34.6666679382324,0,60,3,2,213,72,1,1,1,1,0,11,3
12718,2287,2011-11-03,2,4,0,38.6666679382324,19,2.33333325386047,60,3,2,213,72,1,1,1,1,0,11,
12719,2287,2011-11-03,3,4,0,32,24.3333339691162,3.66666674613953,60,3,2,213,72,1,1,1,1,0,11,
12720,2287,2011-11-03,4,4,0,20.3333339691162,39.3333320617676,0.333333343267441,60,3,2,21
12721,2287,2011-11-04,0,5,0,17.5,29.5,1.5,48.5,2,2,213,72,1,1,1,1,0,11,3929.917032,4531.717293,
12722,2287,2011-11-04,1,5,0,4,53,3,60,3,2,213,72,1,1,1,1,0,11,3929.917032,4531.717293,9.068429
12723,2287,2011-11-04,2,5,0,22,38,0,60,3,2,213,72,1,1,1,1,0,11,3929.917032,4531.717293,9.06842
12724,2287,2011-11-04,3,5,0,33.6666679382324,25.3333339691162,1,60,3,2,213,72,1,1,1,1,0,11,3
12725,2287,2011-11-04,4,5,0,33.25,26.75,0,60,3,2,213,72,1,1,1,1,0,11,3929.917032,4531.717293,9
12726,2287,2011-11-05,0,6,1,15.5,37,1,53.5,2,2,213,72,1,1,1,1,0,11,3929.917032,4531.717293,9.06
12727,2287,2011-11-06,0,0,1,14,30,1.5,45.5,2,2,213,72,1,1,1,1,0,11,3929.917032,4531.717293,9.06
12728,2287,2011-11-06,1,0,1,19,39,2,60,3,2,213,72,1,1,1,1,0,11,3929.917032,4531.717293,9.06842
12729,2287,2011-11-06,2,0,1,30.3333339691162,29.3333339691162,0.333333343267441,60,3,2,21
12730,2287,2011-11-06,3,0,1,26.3333339691162,33.6666679382324,0,60,3,2,213,72,1,1,1,1,0,11,3
12731,2287,2011-11-06,4,0,1,26.6666660308838,33.3333320617676,0,60,3,2,213,72,1,1,1,1,0,11,3
12732,2287,2011-11-07,1,1,0,7.5,47,3.5,58,3,2,213,72,1,1,1,1,0,11,3929.917032,4531.717293,9.068
12733,2287,2011-11-07,2,1,0,18.1666660308838,41,0.833333313465118,60,3,2,213,72,1,1,1,1,0,11
12734,2287,2011-11-07,3,1,0,43.5,16.5,0,60,3,2,213,72,1,1,1,1,0,11,3929.917032,4531.717293,9.06
12735,2287,2011-11-07,4,1,0,37,23,0,60,3,2,213,72,1,1,1,1,0,11,3929.917032,4531.717293,9.06842
12736,2294,2011-10-29,0,6,1,14.3333330154419,44,1.66666662693024,60,3,2,213,80,1,1,1,0,0,12,
12737,2294,2011-10-29,1,6,1,14.6666669845581,43,2.33333325386047,60,3,2,213,80,1,1,1,0,0,12,
12738,2294,2011-10-29,2,6,1,47.6666679382324,12,0.333333343267441,60,3,2,213,80,1,1,1,0,0,12
12739,2294,2011-10-29,3,6,1,43.6666679382324,16,0.333333343267441,60,3,2,213,80,1,1,1,0,0,12
12740,2294,2011-10-30,0,0,1,26,26,3,55,1,2,213,80,1,1,1,0,0,12,3610.019635,4338.117003,9.49021
12741,2294,2011-10-30,1,0,1,39.3333320617676,20.6666660308838,0,60,3,2,213,80,1,1,1,0,0,12,3
12742,2294,2011-10-30,2,0,1,44.6666679382324,15.3333330154419,0,60,3,2,213,80,1,1,1,0,0,12,3
12743,2294,2011-10-30,3,0,1,24.6666660308838,35.3333320617676,0,60,3,2,213,80,1,1,1,0,0,12,3
12744,2294,2011-10-30,4,0,1,28.5,30.5,0,59,3,2,213,80,1,1,1,0,0,12,3610.019635,4338.117003,9.49
12745,2294,2011-10-31,0,1,0,26.3333339691162,29,0.333333343267441,55.6666679382324,3,2,21
12746,2294,2011-10-31,1,1,0,38.6666679382324,21.3333339691162,0,60,3,2,213,80,1,1,1,0,0,12,3
12747,2294,2011-10-31,2,1,0,11.3333330154419,47.3333320617676,1.33333337306976,60,3,2,213
12748,2294,2011-10-31,3,1,0,37,22.6666660308838,0.333333343267441,60,3,2,213,80,1,1,1,0,0,12
12749,2294,2011-10-31,4,1,0,53.6666679382324,6.33333349227905,0,60,3,2,213,80,1,1,1,0,0,12,3

12750,2294,2011-11-01,0,2,0,28,15,2,45,1,2,213,80,1,1,1,0,0,12,3610.019635,4338.117003,9.49021
12751,2294,2011-11-01,1,2,0,31.3333339691162,28.1666660308838,0.5,60,3,2,213,80,1,1,1,0,0,12
12752,2294,2011-11-01,2,2,0,49,10.6666669845581,0.333333343267441,60,3,2,213,80,1,1,1,0,0,1
12753,2294,2011-11-01,3,2,0,25,31.6666660308838,3.33333325386047,60,3,2,213,80,1,1,1,0,0,12,
12754,2294,2011-11-01,4,2,0,38,22,0,60,3,2,213,80,1,1,1,0,0,12,3610.019635,4338.117003,9.49021
12755,2294,2011-11-02,0,3,0,1,9,4,14,1,2,213,80,1,1,1,0,0,12,3610.019635,4338.117003,9.490212
12756,2294,2011-11-02,1,3,0,38.6666679382324,21.3333339691162,0,60,3,2,213,80,1,1,1,0,0,12,3
12757,2294,2011-11-02,2,3,0,41.6666679382324,18.3333339691162,0,60,3,2,213,80,1,1,1,0,0,12,3
12758,2294,2011-11-02,3,3,0,50.75,9.25,0,60,3,2,213,80,1,1,1,0,0,12,3610.019635,4338.117003,9.
12759,2294,2011-11-02,4,3,0,50.5,9,0.5,60,2,2,213,80,1,1,1,0,0,12,3610.019635,4338.117003,9.49
12760,2294,2011-11-03,3,4,0,46,14,0,60,2,2,213,80,1,1,1,0,0,12,3610.019635,4338.117003,9.49021
12761,2294,2011-11-03,4,4,0,51,9,0,60,3,2,213,80,1,1,1,0,0,12,3610.019635,4338.117003,9.490212
12762,2296,2011-10-29,1,6,1,5.80000019073486,44,2.20000004768372,52,3,2,213,74,1,1,0,1,0,11,
12763,2296,2011-10-29,2,6,1,19.75,35.5,4.75,60,3,2,213,74,1,1,0,1,0,11,4210.818063,4534.188674
12764,2296,2011-10-29,3,6,1,48.6666679382324,11.3333330154419,0,60,3,2,213,74,1,1,0,1,0,11,4
12765,2296,2011-10-29,4,6,1,11.5,29,6.5,47,2,2,213,74,1,1,0,1,0,11,4210.818063,4534.188674,8.4
12766,2296,2011-10-30,0,0,1,0,1,0,1,1,2,213,74,1,1,0,1,0,11,4210.818063,4534.188674,8.4844356
12767,2296,2011-10-30,1,0,1,14,42.6666679382324,3.33333325386047,60,3,2,213,74,1,1,0,1,0,11,
12768,2296,2011-10-30,2,0,1,24.6000003814697,33.7999992370605,1.60000002384186,60,3,2,21
12769,2296,2011-10-30,3,0,1,44.25,15.25,0.5,60,3,2,213,74,1,1,0,1,0,11,4210.818063,4534.188674
12770,2296,2011-10-30,4,0,1,40.3333320617676,19.6666660308838,0,60,3,2,213,74,1,1,0,1,0,11,4
12771,2296,2011-10-31,0,1,0,8,2,0,10,1,2,213,74,1,1,0,1,0,11,4210.818063,4534.188674,8.484435
12772,2296,2011-10-31,1,1,0,38.3333320617676,19.3333339691162,2.33333325386047,60,3,2,21
12773,2296,2011-10-31,2,1,0,22,36.6666679382324,1.33333337306976,60,3,2,213,74,1,1,0,1,0,11,
12774,2296,2011-10-31,3,1,0,37.7999992370605,22,0.200000002980232,60,3,2,213,74,1,1,0,1,0,1
12775,2296,2011-10-31,4,1,0,35.3333320617676,21.6666660308838,1,58,3,2,213,74,1,1,0,1,0,11,4
12776,2296,2011-11-03,0,4,0,56,2,2,60,1,2,213,74,1,1,0,1,0,11,4210.818063,4534.188674,8.48443
12777,2296,2011-11-03,1,4,0,7.33333349227905,44.3333320617676,8.33333301544189,60,3,2,21
12778,2296,2011-11-03,2,4,0,24,35.6666679382324,0.333333343267441,60,3,2,213,74,1,1,0,1,0,1
12779,2296,2011-11-03,3,4,0,10.3333330154419,46,3.66666674613953,60,3,2,213,74,1,1,0,1,0,11,
12780,2296,2011-11-03,4,4,0,51,9,0,60,1,2,213,74,1,1,0,1,0,11,4210.818063,4534.188674,8.48443
12781,2296,2011-11-04,0,5,0,1,24,5,30,1,2,213,74,1,1,0,1,0,11,4210.818063,4534.188674,8.48443
12782,2296,2011-11-04,1,5,0,6,48.75,4.25,59,3,2,213,74,1,1,0,1,0,11,4210.818063,4534.188674,8.
12783,2296,2011-11-04,2,5,0,13.6666669845581,40.6666679382324,5.66666650772095,60,3,2,21
12784,2296,2011-11-04,3,5,0,38,22,0,60,3,2,213,74,1,1,0,1,0,11,4210.818063,4534.188674,8.4844
12785,2296,2011-11-04,4,5,0,38,12.1999998092651,1.39999997615814,51.5999984741211,3,2,21
12786,2296,2011-11-15,1,2,0,2,40.5,1.5,44,2,2,213,74,1,1,0,1,0,11,4210.818063,4534.188674,8.48
12787,2296,2011-11-15,2,2,0,24.6666660308838,34.6666679382324,0.666666686534882,60,3,2,2
12788,2296,2011-11-15,3,2,0,36.3333320617676,22,1.66666662693024,60,3,2,213,74,1,1,0,1,0,11,
12789,2296,2011-11-15,4,2,0,38.5999984741211,12.1999998092651,0.200000002980232,51,3,2,2
12790,2300,2011-11-15,1,2,0,9,24,1,34,1,2,213,76,1,0,1,0,0,10,3836.977126,4271.982547,7.64361
12791,2300,2011-11-16,1,3,0,21.6000003814697,36.7999992370605,0.400000005960464,58.7999
12792,2300,2011-11-16,2,3,0,40,20,0,60,3,2,213,76,1,0,1,0,0,10,3836.977126,4271.982547,7.64361
12793,2300,2011-11-16,3,3,0,48.6666679382324,11.3333330154419,0,60,3,2,213,76,1,0,1,0,0,10,3
12794,2300,2011-11-16,4,3,0,43,17,0,60,3,2,213,76,1,0,1,0,0,10,3836.977126,4271.982547,7.64361
12795,2300,2011-11-17,1,4,0,28.3333339691162,23.6666660308838,0.333333343267441,52.33333
12796,2300,2011-11-17,2,4,0,42.2000007629395,17.6000003814697,0.200000002980232,60,3,2,2
12797,2300,2011-11-17,3,4,0,40,20,0,60,3,2,213,76,1,0,1,0,0,10,3836.977126,4271.982547,7.64361
12798,2300,2011-11-17,4,4,0,17.6666660308838,26,0,43.6666679382324,3,2,213,76,1,0,1,0,0,10,3
12799,2300,2011-11-18,1,5,0,19,33.3333320617676,0.333333343267441,52.6666679382324,3,2,2

12800,2300,2011-11-18,2,5,0,42.3333320617676,17.6666660308838,0,60,3,2,213,76,1,0,1,0,0,10,3
12801,2300,2011-11-18,3,5,0,44.2000007629395,15.8000001907349,0,60,3,2,213,76,1,0,1,0,0,10,3
12802,2300,2011-11-18,4,5,0,48,11.6666669845581,0.333333343267441,60,3,2,213,76,1,0,1,0,0,1
12803,2300,2011-11-19,1,6,1,21.3333339691162,36,0.666666686534882,58,3,2,213,76,1,0,1,0,0,1
12804,2300,2011-11-19,2,6,1,38,22,0,60,3,2,213,76,1,0,1,0,0,10,3836.977126,4271.982547,7.64361
12805,2300,2011-11-19,3,6,1,34.3333320617676,25.6666660308838,0,60,3,2,213,76,1,0,1,0,0,10,3
12806,2300,2011-11-19,4,6,1,37,23,0,60,3,2,213,76,1,0,1,0,0,10,3836.977126,4271.982547,7.64361
12807,2300,2011-11-20,0,0,1,21,17.5,2.5,41,2,2,213,76,1,0,1,0,0,10,3836.977126,4271.982547,7.64
12808,2300,2011-11-20,1,0,1,21,38.7999992370605,0.200000002980232,60,3,2,213,76,1,0,1,0,0,1
12809,2300,2011-11-20,2,0,1,40.6666679382324,19.3333339691162,0,60,3,2,213,76,1,0,1,0,0,10,3
12810,2300,2011-11-20,3,0,1,48.6666679382324,11.3333330154419,0,60,3,2,213,76,1,0,1,0,0,10,3
12811,2300,2011-11-20,4,0,1,36.6666679382324,23.3333339691162,0,60,3,2,213,76,1,0,1,0,0,10,3
12812,2300,2011-11-21,1,1,0,17,43,0,60,1,2,213,76,1,0,1,0,0,10,3836.977126,4271.982547,7.64361
12813,2300,2011-11-21,2,1,0,41.3333320617676,18.6666660308838,0,60,3,2,213,76,1,0,1,0,0,10,3
12814,2300,2011-11-21,3,1,0,41.3333320617676,18.6666660308838,0,60,3,2,213,76,1,0,1,0,0,10,3
12815,2300,2011-11-21,4,1,0,51.3333320617676,8.66666698455811,0,60,3,2,213,76,1,0,1,0,0,10,3
12816,2302,2011-11-11,1,5,0,38.6666679382324,17,0.333333343267441,56,3,2,213,66,0,0,0,0,0,9,
12817,2302,2011-11-11,2,5,0,35.8333320617676,22,2.16666674613953,60,3,2,213,66,0,0,0,0,0,9,3
12818,2302,2011-11-11,3,5,0,42,18,0,60,3,2,213,66,0,0,0,0,0,9,3762.281777,4211.152746,7.53854
12819,2302,2011-11-11,4,5,0,32,27.3333339691162,0.666666686534882,60,3,2,213,66,0,0,0,0,0,9,
12820,2302,2011-11-12,0,6,1,13,31,7,51,1,2,213,66,0,0,0,0,0,9,3762.281777,4211.152746,7.53854
12821,2302,2011-11-12,1,6,1,13.6666669845581,43,3.33333325386047,60,3,2,213,66,0,0,0,0,0,9,3
12822,2302,2011-11-12,2,6,1,26.6666660308838,32.3333320617676,1,60,3,2,213,66,0,0,0,0,0,9,37
12823,2302,2011-11-12,3,6,1,32,26,2,60,3,2,213,66,0,0,0,0,0,9,3762.281777,4211.152746,7.53854
12824,2302,2011-11-12,4,6,1,26.75,11.5,0.5,38.75,3,2,213,66,0,0,0,0,0,9,3762.281777,4211.15274
12825,2302,2011-11-21,0,1,0,33,25,2,60,1,2,213,66,0,0,0,0,0,9,3762.281777,4211.152746,7.53854
12826,2302,2011-11-21,1,1,0,17.6666660308838,36,6.33333349227905,60,3,2,213,66,0,0,0,0,0,9,3
12827,2302,2011-11-21,2,1,0,21,39,0,60,1,2,213,66,0,0,0,0,0,9,3762.281777,4211.152746,7.53854
12828,2302,2011-11-22,0,2,0,2.5,45.5,2.5,50.5,2,2,213,66,0,0,0,0,0,9,3762.281777,4211.152746,7.
12829,2302,2011-11-22,1,2,0,18,36.3333320617676,5.66666650772095,60,3,2,213,66,0,0,0,0,0,9,3
12830,2302,2011-11-22,2,2,0,27.25,26,6.75,60,3,2,213,66,0,0,0,0,0,9,3762.281777,4211.152746,7.
12831,2302,2011-11-22,3,2,0,40,19.6666660308838,0.333333343267441,60,3,2,213,66,0,0,0,0,0,9,
12832,2302,2011-11-22,4,2,0,19.5,29.5,1,50,2,2,213,66,0,0,0,0,0,9,3762.281777,4211.152746,7.53
12833,2302,2011-11-24,0,4,0,11,29,1,41,2,2,213,66,0,0,0,0,0,9,3762.281777,4211.152746,7.53854
12834,2302,2011-11-24,1,4,0,43.3333320617676,16,0.666666686534882,60,3,2,213,66,0,0,0,0,0,9,
12835,2302,2011-11-24,2,4,0,25,30,5,60,1,2,213,66,0,0,0,0,0,9,3762.281777,4211.152746,7.53854
12836,2302,2011-11-24,3,4,0,31,27,2,60,3,2,213,66,0,0,0,0,0,9,3762.281777,4211.152746,7.53854
12837,2302,2011-11-24,4,4,0,33,8.66666698455811,3,44.6666679382324,3,2,213,66,0,0,0,0,0,9,37
12838,2306,2011-11-11,3,5,0,32,28,0,60,3,2,213,88,1,1,1,1,0,10,4209.071569,4630.200383,8.5452
12839,2306,2011-11-11,4,5,0,50.6666679382324,9.33333301544189,0,60,3,2,213,88,1,1,1,1,0,10,4
12840,2306,2011-11-12,0,6,1,48,12,0,60,1,2,213,88,1,1,1,1,0,10,4209.071569,4630.200383,8.5452
12841,2306,2011-11-12,1,6,1,23,37,0,60,1,2,213,88,1,1,1,1,0,10,4209.071569,4630.200383,8.5452
12842,2306,2011-11-13,0,0,1,24.5,24,0,48.5,2,2,213,88,1,1,1,1,0,10,4209.071569,4630.200383,8.5
12843,2306,2011-11-13,1,0,1,32.7999992370605,27.2000007629395,0,60,3,2,213,88,1,1,1,1,0,10,4
12844,2306,2011-11-13,2,0,1,37.75,22.25,0,60,3,2,213,88,1,1,1,1,0,10,4209.071569,4630.200383,8
12845,2306,2011-11-13,3,0,1,33.6666679382324,26.3333339691162,0,60,3,2,213,88,1,1,1,1,0,10,4
12846,2306,2011-11-13,4,0,1,42.3333320617676,17.6666660308838,0,60,3,2,213,88,1,1,1,1,0,10,4
12847,2306,2011-11-14,0,1,0,24,12,0,36,2,2,213,88,1,1,1,1,0,10,4209.071569,4630.200383,8.5452
12848,2306,2011-11-14,1,1,0,41.6666679382324,18.3333339691162,0,60,3,2,213,88,1,1,1,1,0,10,4
12849,2306,2011-11-14,2,1,0,33.7999992370605,26.2000007629395,0,60,3,2,213,88,1,1,1,1,0,10,4

12850,2306,2011-11-14,3,1,0,27.8333339691162,32.1666679382324,0,60,3,2,213,88,1,1,1,1,0,10,4
12851,2306,2011-11-14,4,1,0,43.6666679382324,16.3333339691162,0,60,3,2,213,88,1,1,1,1,0,10,4
12852,2306,2011-11-15,0,2,0,24,17.5,0.5,42,2,2,213,88,1,1,1,1,0,10,4209.071569,4630.200383,8.54
12853,2306,2011-11-15,1,2,0,29.6666660308838,30.3333339691162,0,60,3,2,213,88,1,1,1,1,0,10,4
12854,2306,2011-11-15,2,2,0,38,22,0,60,3,2,213,88,1,1,1,1,0,10,4209.071569,4630.200383,8.54524
12855,2306,2011-11-15,3,2,0,32,28,0,60,3,2,213,88,1,1,1,1,0,10,4209.071569,4630.200383,8.54524
12856,2306,2011-11-15,4,2,0,41.7999992370605,18.2000007629395,0,60,3,2,213,88,1,1,1,1,0,10,4
12857,2306,2011-11-16,0,3,0,26.5,18,0,44.5,3,2,213,88,1,1,1,1,0,10,4209.071569,4630.200383,8.54
12858,2306,2011-11-16,1,3,0,40,20,0,60,3,2,213,88,1,1,1,1,0,10,4209.071569,4630.200383,8.54524
12859,2306,2011-11-16,2,3,0,33,27,0,60,3,2,213,88,1,1,1,1,0,10,4209.071569,4630.200383,8.54524
12860,2306,2011-11-16,3,3,0,42,18,0,60,3,2,213,88,1,1,1,1,0,10,4209.071569,4630.200383,8.54524
12861,2306,2011-11-16,4,3,0,45.75,14.25,0,60,3,2,213,88,1,1,1,1,0,10,4209.071569,4630.200383,8
12862,2306,2011-11-17,0,4,0,32.5,21,0,53.5,2,2,213,88,1,1,1,1,0,10,4209.071569,4630.200383,8.54
12863,2306,2011-11-17,1,4,0,37,23,0,60,3,2,213,88,1,1,1,1,0,10,4209.071569,4630.200383,8.54524
12864,2306,2011-11-17,2,4,0,37,23,0,60,3,2,213,88,1,1,1,1,0,10,4209.071569,4630.200383,8.54524
12865,2306,2011-11-17,3,4,0,45,15,0,60,3,2,213,88,1,1,1,1,0,10,4209.071569,4630.200383,8.54524
12866,2306,2011-11-17,4,4,0,42.6666679382324,17.3333339691162,0,60,3,2,213,88,1,1,1,1,0,10,4
12867,2313,2011-11-11,0,5,0,53,5,2,60,1,2,213,78,1,0,0,1,0,8,3413.081726,4326.920155,7.3224194
12868,2313,2011-11-11,1,5,0,23,35,2,60,3,2,213,78,1,0,0,1,0,8,3413.081726,4326.920155,7.322419
12869,2313,2011-11-11,2,5,0,42.6666679382324,17.3333339691162,0,60,3,2,213,78,1,0,0,1,0,8,34
12870,2313,2011-11-11,3,5,0,22,37,1,60,1,2,213,78,1,0,0,1,0,8,3413.081726,4326.920155,7.322419
12871,2313,2011-11-12,0,6,1,1,11,1,13,1,2,213,78,1,0,0,1,0,8,3413.081726,4326.920155,7.3224194
12872,2313,2011-11-12,1,6,1,16.3333339691162,43.6666679382324,0,60,3,2,213,78,1,0,0,1,0,8,34
12873,2313,2011-11-12,2,6,1,31.3333339691162,28.3333339691162,0.333333343267441,60,3,2,21
12874,2313,2011-11-12,3,6,1,35,25,0,60,3,2,213,78,1,0,0,1,0,8,3413.081726,4326.920155,7.322419
12875,2313,2011-11-12,4,6,1,33.5,26.5,0,60,3,2,213,78,1,0,0,1,0,8,3413.081726,4326.920155,7.322
12876,2313,2011-11-13,1,0,1,14.6000003814697,32.5999984741211,1,48.2000007629395,3,2,213,
12877,2313,2011-11-13,2,0,1,27,32.3333320617676,0.666666686534882,60,3,2,213,78,1,0,0,1,0,8,
12878,2313,2011-11-13,3,0,1,42.6666679382324,17.3333339691162,0,60,3,2,213,78,1,0,0,1,0,8,34
12879,2313,2011-11-13,4,0,1,36,24,0,60,3,2,213,78,1,0,0,1,0,8,3413.081726,4326.920155,7.322419
12880,2313,2011-11-14,1,1,0,25.5,33.5,1,60,3,2,213,78,1,0,0,1,0,8,3413.081726,4326.920155,7.322
12881,2313,2011-11-14,2,1,0,36,23.6666660308838,0.333333343267441,60,3,2,213,78,1,0,0,1,0,8,
12882,2313,2011-11-14,3,1,0,35.3333320617676,24,0.666666686534882,60,3,2,213,78,1,0,0,1,0,8,
12883,2313,2011-11-14,4,1,0,33,27,0,60,3,2,213,78,1,0,0,1,0,8,3413.081726,4326.920155,7.322419
12884,2313,2011-11-15,1,2,0,30,26,0.666666686534882,56.6666679382324,3,2,213,78,1,0,0,1,0,8,
12885,2313,2011-11-15,2,2,0,30.6666660308838,29.3333339691162,0,60,3,2,213,78,1,0,0,1,0,8,34
12886,2313,2011-11-15,3,2,0,26.6666660308838,32.8333320617676,0.5,60,3,2,213,78,1,0,0,1,0,8,3
12887,2313,2011-11-15,4,2,0,32.75,27.25,0,60,3,2,213,78,1,0,0,1,0,8,3413.081726,4326.920155,7.3
12888,2313,2011-11-16,0,3,0,29,8.5,1,38.5,2,2,213,78,1,0,0,1,0,8,3413.081726,4326.920155,7.3224
12889,2313,2011-11-16,1,3,0,21.3333339691162,38.6666679382324,0,60,3,2,213,78,1,0,0,1,0,8,34
12890,2313,2011-11-16,2,3,0,42.3333320617676,17.3333339691162,0.333333343267441,60,3,2,21
12891,2313,2011-11-16,3,3,0,34.6666679382324,25.3333339691162,0,60,3,2,213,78,1,0,0,1,0,8,34
12892,2313,2011-11-16,4,3,0,35,24.7999992370605,0.200000002980232,60,3,2,213,78,1,0,0,1,0,8,
12893,2313,2011-11-17,0,4,0,8,17,2,27,1,2,213,78,1,0,0,1,0,8,3413.081726,4326.920155,7.3224194
12894,2313,2011-11-17,1,4,0,24.3333339691162,35,0.666666686534882,60,3,2,213,78,1,0,0,1,0,8,
12895,2313,2011-11-17,2,4,0,24.6666660308838,29.3333339691162,6,60,3,2,213,78,1,0,0,1,0,8,34
12896,2313,2011-11-17,3,4,0,33,21,6,60,3,2,213,78,1,0,0,1,0,8,3413.081726,4326.920155,7.322419
12897,2313,2011-11-17,4,4,0,31.3333339691162,11.6666669845581,3,46,3,2,213,78,1,0,0,1,0,8,34
12898,2322,2011-11-11,0,5,0,14.5,45.5,0,60,2,2,213,74,1,1,0,1,0,12,4130.013622,3921.627795,8.48
12899,2322,2011-11-11,1,5,0,39.6666679382324,19,1.33333337306976,60,3,2,213,74,1,1,0,1,0,12,

12900,2322,2011-11-11,2,5,0,36.6666679382324,22,1.33333337306976,60,3,2,213,74,1,1,0,1,0,12,
12901,2322,2011-11-11,3,5,0,25.3333339691162,33.6666679382324,1,60,3,2,213,74,1,1,0,1,0,12,4
12902,2322,2011-11-11,4,5,0,39,21,0,60,3,2,213,74,1,1,0,1,0,12,4130.013622,3921.627795,8.4891
12903,2322,2011-11-12,0,6,1,11,36,1.66666662693024,48.6666679382324,3,2,213,74,1,1,0,1,0,12,
12904,2322,2011-11-12,1,6,1,21,35.6666679382324,3.33333325386047,60,3,2,213,74,1,1,0,1,0,12,
12905,2322,2011-11-12,2,6,1,49.6666679382324,10.3333330154419,0,60,3,2,213,74,1,1,0,1,0,12,4
12906,2322,2011-11-12,3,6,1,40,19.6666660308838,0.333333343267441,60,3,2,213,74,1,1,0,1,0,1
12907,2322,2011-11-12,4,6,1,38.5,20.5,1,60,2,2,213,74,1,1,0,1,0,12,4130.013622,3921.627795,8.4
12908,2322,2011-11-13,0,0,1,30.3333339691162,17,1,48.3333320617676,3,2,213,74,1,1,0,1,0,12,4
12909,2322,2011-11-13,1,0,1,34.7999992370605,24.3999996185303,0.800000011920929,60,3,2,2
12910,2322,2011-11-13,2,0,1,28.6666660308838,29.3333339691162,2,60,3,2,213,74,1,1,0,1,0,12,4
12911,2322,2011-11-13,3,0,1,27.7999992370605,25,7.19999980926514,60,3,2,213,74,1,1,0,1,0,12,
12912,2322,2011-11-13,4,0,1,45.75,14.25,0,60,3,2,213,74,1,1,0,1,0,12,4130.013622,3921.627795,8
12913,2322,2011-11-16,0,3,0,34,13.6666669845581,0,47.6666679382324,3,2,213,74,1,1,0,1,0,12,4
12914,2322,2011-11-16,1,3,0,33.3333320617676,25,1.66666662693024,60,3,2,213,74,1,1,0,1,0,12,
12915,2322,2011-11-16,2,3,0,25.6666660308838,32.3333320617676,2,60,3,2,213,74,1,1,0,1,0,12,4
12916,2322,2011-11-16,3,3,0,42,18,0,60,1,2,213,74,1,1,0,1,0,12,4130.013622,3921.627795,8.4891
12917,2322,2011-11-16,4,3,0,49,10.6666669845581,0.333333343267441,60,3,2,213,74,1,1,0,1,0,1
12918,2322,2011-11-17,0,4,0,13,44,3,60,1,2,213,74,1,1,0,1,0,12,4130.013622,3921.627795,8.4891
12919,2322,2011-11-17,1,4,0,22,37,1,60,1,2,213,74,1,1,0,1,0,12,4130.013622,3921.627795,8.4891
12920,2322,2011-11-18,0,5,0,22,27,1,50,3,2,213,74,1,1,0,1,0,12,4130.013622,3921.627795,8.4891
12921,2322,2011-11-18,1,5,0,24,34.6666679382324,1.33333337306976,60,3,2,213,74,1,1,0,1,0,12,
12922,2322,2011-11-18,2,5,0,26,34,0,60,1,2,213,74,1,1,0,1,0,12,4130.013622,3921.627795,8.4891
12923,2322,2011-11-18,3,5,0,22.6666660308838,37.3333320617676,0,60,3,2,213,74,1,1,0,1,0,12,4
12924,2322,2011-11-18,4,5,0,28.6666660308838,31,0.333333343267441,60,3,2,213,74,1,1,0,1,0,1
12925,2322,2011-11-19,0,6,1,11,45,1.5,57.5,2,2,213,74,1,1,0,1,0,12,4130.013622,3921.627795,8.4
12926,2322,2011-11-19,1,6,1,30,29,1,60,1,2,213,74,1,1,0,1,0,12,4130.013622,3921.627795,8.4891
12927,2322,2011-11-19,2,6,1,20,37.5,2.5,60,2,2,213,74,1,1,0,1,0,12,4130.013622,3921.627795,8.4
12928,2322,2011-11-19,3,6,1,16,42.3333320617676,1.66666662693024,60,3,2,213,74,1,1,0,1,0,12,
12929,2322,2011-11-19,4,6,1,47.3333320617676,12.3333330154419,0.333333343267441,60,3,2,2
12930,2322,2011-11-24,0,4,0,4,13,0,17,1,2,213,74,1,1,0,1,0,12,4130.013622,3921.627795,8.48918
12931,2322,2011-11-24,1,4,0,45.5,14,0.5,60,2,2,213,74,1,1,0,1,0,12,4130.013622,3921.627795,8.4
12932,2322,2011-11-24,2,4,0,30.6666660308838,28.3333339691162,1,60,3,2,213,74,1,1,0,1,0,12,4
12933,2322,2011-11-24,3,4,0,28.3333339691162,30.6666660308838,1,60,3,2,213,74,1,1,0,1,0,12,4
12934,2322,2011-11-24,4,4,0,34,26,0,60,3,2,213,74,1,1,0,1,0,12,4130.013622,3921.627795,8.4891
12935,2369,2011-11-13,0,0,1,4,50,6,60,1,2,219,66,0,1,0,1,0,12,4548.754931,4206.514908,4.72148
12936,2369,2011-11-13,1,0,1,5,46,9,60,3,2,219,66,0,1,0,1,0,12,4548.754931,4206.514908,4.72148
12937,2369,2011-11-14,0,1,0,10,41,9,60,2,2,219,66,0,1,0,1,0,12,4548.754931,4206.514908,4.7214
12938,2369,2011-11-14,1,1,0,4,47,9,60,3,2,219,66,0,1,0,1,0,12,4548.754931,4206.514908,4.72148
12939,2369,2011-11-14,2,1,0,3.66666674613953,35.3333320617676,21,60,3,2,219,66,0,1,0,1,0,12,
12940,2369,2011-11-14,3,1,0,10.3333330154419,35.6666679382324,14,60,3,2,219,66,0,1,0,1,0,12,
12941,2369,2011-11-14,4,1,0,35.3333320617676,24.6666660308838,0,60,3,2,219,66,0,1,0,1,0,12,4
12942,2369,2011-11-15,0,2,0,7.5,28.5,5,41,2,2,219,66,0,1,0,1,0,12,4548.754931,4206.514908,4.72
12943,2369,2011-11-15,1,2,0,3,44.6666679382324,12.3333330154419,60,3,2,219,66,0,1,0,1,0,12,4
12944,2369,2011-11-15,2,2,0,2,43,15,60,3,2,219,66,0,1,0,1,0,12,4548.754931,4206.514908,4.7214
12945,2369,2011-11-15,3,2,0,38.3333320617676,14.3333330154419,7.33333349227905,60,3,2,21
12946,2369,2011-11-15,4,2,0,42,17.6666660308838,0.333333343267441,60,3,2,219,66,0,1,0,1,0,1
12947,2369,2011-11-16,0,3,0,7,34,11.5,52.5,2,2,219,66,0,1,0,1,0,12,4548.754931,4206.514908,4.7
12948,2369,2011-11-16,1,3,0,10,43,7,60,3,2,219,66,0,1,0,1,0,12,4548.754931,4206.514908,4.7214
12949,2369,2011-11-16,2,3,0,36.4000015258789,20.6000003814697,3,60,3,2,219,66,0,1,0,1,0,12,4

12950,2369,2011-11-16,3,3,0,40,19,1,60,3,2,219,66,0,1,0,1,0,12,4548.754931,4206.514908,4.72148
12951,2369,2011-11-16,4,3,0,42,17.3333339691162,0.666666686534882,60,3,2,219,66,0,1,0,1,0,12
12952,2369,2011-11-17,0,4,0,10,39,4.5,53.5,2,2,219,66,0,1,0,1,0,12,4548.754931,4206.514908,4.72
12953,2369,2011-11-17,1,4,0,2.33333325386047,45.3333320617676,12.3333330154419,60,3,2,219
12954,2369,2011-11-17,2,4,0,18,31.3333339691162,10.6666669845581,60,3,2,219,66,0,1,0,1,0,12,
12955,2369,2011-11-17,3,4,0,36.3333320617676,18,5.66666650772095,60,3,2,219,66,0,1,0,1,0,12,
12956,2369,2011-11-17,4,4,0,41,18,1,60,3,2,219,66,0,1,0,1,0,12,4548.754931,4206.514908,4.72148
12957,2369,2011-11-18,0,5,0,4,29.6666660308838,7.66666650772095,41.3333320617676,3,2,219,
12958,2369,2011-11-18,1,5,0,6.33333349227905,46,7.66666650772095,60,3,2,219,66,0,1,0,1,0,12,
12959,2369,2011-11-18,2,5,0,0,22.5,37.5,60,2,2,219,66,0,1,0,1,0,12,4548.754931,4206.514908,4.72
12960,2369,2011-11-18,3,5,0,37.5,15,7.5,60,2,2,219,66,0,1,0,1,0,12,4548.754931,4206.514908,4.72
12961,2369,2011-11-18,4,5,0,39,21,0,60,3,2,219,66,0,1,0,1,0,12,4548.754931,4206.514908,4.72148
12962,2369,2011-11-19,0,6,1,9.66666698455811,30.6666660308838,2,42.3333320617676,3,2,219,
12963,2369,2011-11-19,1,6,1,24.6666660308838,35.3333320617676,0,60,3,2,219,66,0,1,0,1,0,12,4
12964,2369,2011-11-19,2,6,1,26.25,30.25,3.5,60,3,2,219,66,0,1,0,1,0,12,4548.754931,4206.514908
12965,2369,2011-11-19,3,6,1,18,29.5,12.5,60,3,2,219,66,0,1,0,1,0,12,4548.754931,4206.514908,4.7
12966,2369,2011-11-19,4,6,1,40.6666679382324,19.3333339691162,0,60,3,2,219,66,0,1,0,1,0,12,4
12967,2369,2011-11-24,3,4,0,41.5,15,3.5,60,2,2,219,66,0,1,0,1,0,12,4548.754931,4206.514908,4.72
12968,2369,2011-11-24,4,4,0,35.3333320617676,24.6666660308838,0,60,3,2,219,66,0,1,0,1,0,12,4
12969,2372,2011-11-15,0,2,0,18.5,12.5,0.5,31.5,2,2,219,65,0,0,0,1,0,11,2944.239331,2944.239331,
12970,2372,2011-11-15,1,2,0,32.6666679382324,27.3333339691162,0,60,3,2,219,65,0,0,0,1,0,11,2
12971,2372,2011-11-15,2,2,0,34.75,19.75,5.5,60,3,2,219,65,0,0,0,1,0,11,2944.239331,2944.239331
12972,2372,2011-11-15,3,2,0,25.3333339691162,11,23.6666660308838,60,3,2,219,65,0,0,0,1,0,11,
12973,2372,2011-11-15,4,2,0,19,10,1.5,30.5,2,2,219,65,0,0,0,1,0,11,2944.239331,2944.239331,0,0,
12974,2372,2011-11-16,0,3,0,15.5,19,0.5,35,2,2,219,65,0,0,0,1,0,11,2944.239331,2944.239331,0,0,
12975,2372,2011-11-16,1,3,0,19.6666660308838,40.3333320617676,0,60,3,2,219,65,0,0,0,1,0,11,2
12976,2372,2011-11-16,2,3,0,22,37.3333320617676,0.666666686534882,60,3,2,219,65,0,0,0,1,0,1
12977,2372,2011-11-16,3,3,0,24.5,22.3333339691162,13.1666669845581,60,3,2,219,65,0,0,0,1,0,1
12978,2372,2011-11-16,4,3,0,33.5,7.5,0,41,2,2,219,65,0,0,0,1,0,11,2944.239331,2944.239331,0,0,0
12979,2372,2011-11-17,0,4,0,30,15,0,45,3,2,219,65,0,0,0,1,0,11,2944.239331,2944.239331,0,0,0,0,
12980,2372,2011-11-17,1,4,0,50,10,0,60,3,2,219,65,0,0,0,1,0,11,2944.239331,2944.239331,0,0,0,0,
12981,2372,2011-11-17,2,4,0,26.3333339691162,14.3333330154419,19.3333339691162,60,3,2,219
12982,2372,2011-11-17,3,4,0,53.6666679382324,6.33333349227905,0,60,3,2,219,65,0,0,0,1,0,11,2
12983,2372,2011-11-17,4,4,0,44.5,13.25,0.25,58,3,2,219,65,0,0,0,1,0,11,2944.239331,2944.239331
12984,2372,2011-11-18,0,5,0,21,11,1,33,3,2,219,65,0,0,0,1,0,11,2944.239331,2944.239331,0,0,0,0,
12985,2372,2011-11-18,1,5,0,32,22.3333339691162,5.66666650772095,60,3,2,219,65,0,0,0,1,0,11,
12986,2372,2011-11-18,2,5,0,42,16,2,60,3,2,219,65,0,0,0,1,0,11,2944.239331,2944.239331,0,0,0,0,
12987,2372,2011-11-18,3,5,0,38.3333320617676,20,1.66666662693024,60,3,2,219,65,0,0,0,1,0,11,
12988,2372,2011-11-18,4,5,0,35.2000007629395,19.3999996185303,0.400000005960464,55,3,2,2
12989,2372,2011-11-20,0,0,1,1.33333337306976,58.6666679382324,0,60,3,2,219,65,0,0,0,1,0,11,2
12990,2372,2011-11-20,1,0,1,13.3333330154419,46.3333320617676,0.333333343267441,60,3,2,2
12991,2372,2011-11-20,2,0,1,22.3333339691162,37.3333320617676,0.333333343267441,60,3,2,2
12992,2372,2011-11-20,3,0,1,45.6666679382324,14.3333330154419,0,60,3,2,219,65,0,0,0,1,0,11,2
12993,2372,2011-11-20,4,0,1,15.6666669845581,29.3333339691162,0,45,3,2,219,65,0,0,0,1,0,11,2
12994,2372,2011-11-21,0,1,0,36,6,0.5,42.5,2,2,219,65,0,0,0,1,0,11,2944.239331,2944.239331,0,0,0
12995,2372,2011-11-21,1,1,0,25.8333339691162,34.1666679382324,0,60,3,2,219,65,0,0,0,1,0,11,2
12996,2372,2011-11-21,2,1,0,39.6666679382324,20.3333339691162,0,60,3,2,219,65,0,0,0,1,0,11,2
12997,2372,2011-11-21,3,1,0,47,13,0,60,3,2,219,65,0,0,0,1,0,11,2944.239331,2944.239331,0,0,0,0,
12998,2372,2011-11-21,4,1,0,30,14.5,0.5,45,2,2,219,65,0,0,0,1,0,11,2944.239331,2944.239331,0,0,
12999,2372,2011-11-22,2,2,0,20.3333339691162,16,23.6666660308838,60,3,2,219,65,0,0,0,1,0,11,

13000,2372,2011-11-22,3,2,0,36,11,13,60,3,2,219,65,0,0,0,1,0,11,2944.239331,2944.239331,0,0,0,
13001,2372,2011-11-22,4,2,0,35.6666679382324,23.3333339691162,1,60,3,2,219,65,0,0,0,1,0,11,2
13002,2372,2011-11-24,0,4,0,54,6,0,60,1,2,219,65,0,0,0,1,0,11,2944.239331,2944.239331,0,0,0,0,0,
13003,2372,2011-11-24,1,4,0,45,15,0,60,3,2,219,65,0,0,0,1,0,11,2944.239331,2944.239331,0,0,0,0,
13004,2372,2011-11-24,2,4,0,34.5,25,0.5,60,2,2,219,65,0,0,0,1,0,11,2944.239331,2944.239331,0,0,
13005,2408,2011-11-22,0,2,0,24,36,0,60,1,2,219,80,0,0,1,0,0,10,12892.93159,13338.25583,4.8463
13006,2408,2011-11-22,1,2,0,25.3333339691162,33.6666679382324,1,60,3,2,219,80,0,0,1,0,0,10,1
13007,2408,2011-11-22,2,2,0,42.3333320617676,17.6666660308838,0,60,3,2,219,80,0,0,1,0,0,10,1
13008,2408,2011-11-22,3,2,0,34,26,0,60,1,2,219,80,0,0,1,0,0,10,12892.93159,13338.25583,4.8463
13009,2408,2012-02-10,0,5,0,17.3333339691162,29.6666660308838,12.3333330154419,59.33333
13010,2408,2012-02-10,1,5,0,24.3333339691162,35.6666679382324,0,60,3,2,219,80,0,0,1,0,0,10,1
13011,2408,2012-02-10,2,5,0,13,47,0,60,3,2,219,80,0,0,1,0,0,10,12892.93159,13338.25583,4.8463
13012,2408,2012-02-10,3,5,0,37.3333320617676,17.3333339691162,5.33333349227905,60,3,2,21
13013,2408,2012-02-10,4,5,0,39,19.3333339691162,1.66666662693024,60,3,2,219,80,0,0,1,0,0,10,
13014,2408,2012-02-11,0,6,1,20,22.3333339691162,0.666666686534882,43,3,2,219,80,0,0,1,0,0,1
13015,2408,2012-02-11,1,6,1,25,34,1,60,3,2,219,80,0,0,1,0,0,10,12892.93159,13338.25583,4.8463
13016,2408,2012-02-11,2,6,1,27.6666660308838,31.3333339691162,1,60,3,2,219,80,0,0,1,0,0,10,1
13017,2408,2012-02-11,3,6,1,41,16.25,2.75,60,3,2,219,80,0,0,1,0,0,10,12892.93159,13338.25583,4
13018,2408,2012-02-11,4,6,1,36.3333320617676,5.66666650772095,0,42,3,2,219,80,0,0,1,0,0,10,1
13019,2408,2012-02-12,0,0,1,10,20,1,31,1,2,219,80,0,0,1,0,0,10,12892.93159,13338.25583,4.8463
13020,2408,2012-02-12,1,0,1,16.6666660308838,43,0.333333343267441,60,3,2,219,80,0,0,1,0,0,1
13021,2408,2012-02-12,2,0,1,8,52,0,60,3,2,219,80,0,0,1,0,0,10,12892.93159,13338.25583,4.84636
13022,2408,2012-02-12,3,0,1,34.5,25,0.5,60,3,2,219,80,0,0,1,0,0,10,12892.93159,13338.25583,4.8
13023,2408,2012-02-12,4,0,1,40.7999992370605,18.2000007629395,1,60,3,2,219,80,0,0,1,0,0,10,1
13024,2408,2012-02-13,0,1,0,12.3333330154419,21.3333339691162,1,34.6666679382324,3,2,219,
13025,2408,2012-02-13,1,1,0,26.6666660308838,27,6.33333349227905,60,3,2,219,80,0,0,1,0,0,10,
13026,2408,2012-02-13,2,1,0,21.3333339691162,37.3333320617676,1.33333337306976,60,3,2,21
13027,2408,2012-02-13,3,1,0,21,36.3333320617676,2.66666674613953,60,3,2,219,80,0,0,1,0,0,10,
13028,2408,2012-02-13,4,1,0,56.5,3.5,0,60,3,2,219,80,0,0,1,0,0,10,12892.93159,13338.25583,4.84
13029,2408,2012-02-14,0,2,0,16.5,28.5,0.25,45.25,3,2,219,80,0,0,1,0,0,10,12892.93159,13338.255
13030,2408,2012-02-14,1,2,0,28.6666660308838,31.3333339691162,0,60,3,2,219,80,0,0,1,0,0,10,1
13031,2408,2012-02-14,2,2,0,28.6666660308838,25.3333339691162,6,60,3,2,219,80,0,0,1,0,0,10,1
13032,2408,2012-02-14,3,2,0,28.3333339691162,29.3333339691162,2.33333325386047,60,3,2,21
13033,2408,2012-02-14,4,2,0,38.6666679382324,20,1.33333337306976,60,3,2,219,80,0,0,1,0,0,10,
13034,2408,2012-02-15,1,3,0,14,46,0,60,3,2,219,80,0,0,1,0,0,10,12892.93159,13338.25583,4.8463
13035,2408,2012-02-15,2,3,0,29,30.3333339691162,0.666666686534882,60,3,2,219,80,0,0,1,0,0,1
13036,2408,2012-02-15,3,3,0,44,14.3333330154419,1.66666662693024,60,3,2,219,80,0,0,1,0,0,10,
13037,2408,2012-02-15,4,3,0,38.3333320617676,20.3333339691162,1.33333337306976,60,3,2,21
13038,2410,2012-02-11,0,6,1,11.3333330154419,39.6666679382324,3.66666674613953,54.66666
13039,2410,2012-02-11,1,6,1,13.6666669845581,41.6666679382324,4.66666650772095,60,3,2,21
13040,2410,2012-02-11,2,6,1,44.75,15,0.25,60,3,2,219,67,0,0,0,1,0,10,13159.36434,13338.25583,6
13041,2410,2012-02-11,3,6,1,42.6666679382324,17.3333339691162,0,60,3,2,219,67,0,0,0,1,0,10,1
13042,2410,2012-02-11,4,6,1,9,22,0.5,31.5,2,2,219,67,0,0,0,1,0,10,13159.36434,13338.25583,6.03
13043,2410,2012-02-12,0,0,1,20.5,24.5,1.5,46.5,2,2,219,67,0,0,0,1,0,10,13159.36434,13338.25583,
13044,2410,2012-02-12,1,0,1,24,29,7,60,3,2,219,67,0,0,0,1,0,10,13159.36434,13338.25583,6.0368
13045,2410,2012-02-12,2,0,1,42,14.6666669845581,3.33333325386047,60,3,2,219,67,0,0,0,1,0,10,
13046,2410,2012-02-12,3,0,1,35.1666679382324,17.1666660308838,0.16666667163372,52.5,3,2,2
13047,2410,2012-02-13,0,1,0,9.33333301544189,44.6666679382324,2,56,3,2,219,67,0,0,0,1,0,10,1
13048,2410,2012-02-13,1,1,0,21.6666660308838,31.3333339691162,7,60,3,2,219,67,0,0,0,1,0,10,1
13049,2410,2012-02-13,2,1,0,16.6666660308838,35,8.33333301544189,60,3,2,219,67,0,0,0,1,0,10,

13050,2410,2012-02-13,3,1,0,37,20.6666660308838,2.33333325386047,60,3,2,219,67,0,0,0,1,0,10,
13051,2410,2012-02-13,4,1,0,28,29,1,58,1,2,219,67,0,0,0,1,0,10,13159.36434,13338.25583,6.03684
13052,2410,2012-02-14,4,2,0,21,9,0,30,1,2,219,67,0,0,0,1,0,10,13159.36434,13338.25583,6.036843
13053,2410,2012-02-15,0,3,0,8,49,3,60,1,2,219,67,0,0,0,1,0,10,13159.36434,13338.25583,6.036843
13054,2410,2012-02-15,1,3,0,36,23.6666660308838,0.333333343267441,60,3,2,219,67,0,0,0,1,0,10
13055,2410,2012-02-16,0,4,0,18,37,5,60,3,2,219,67,0,0,0,1,0,10,13159.36434,13338.25583,6.03684
13056,2410,2012-02-16,1,4,0,36.3333320617676,23.6666660308838,0,60,3,2,219,67,0,0,0,1,0,10,1
13057,2410,2012-02-16,2,4,0,12.3999996185303,37.4000015258789,10.1999998092651,60,3,2,219
13058,2410,2012-02-16,3,4,0,29,30.6666660308838,0.333333343267441,60,3,2,219,67,0,0,0,1,0,10
13059,2410,2012-02-16,4,4,0,32.6666679382324,9.66666698455811,0.666666686534882,43,3,2,2

ite","Neigh_ID","Age","Sex","Education","Marital","Car","Neigh_SES","SPPB","DwDens_1km","DwDe
23969881,0.087382224,0.087382224,1,1,7.477139225,12.97190964,166.6766968,17.44665819,38.9
23969881,0.087382224,0.087382224,1,1,7.477139225,12.97190964,166.6766968,17.44665819,38.9
3969881,0.087382224,0.087382224,1,1,7.477139225,12.97190964,166.6766968,17.44665819,38.91
23969881,0.087382224,0.087382224,1,1,7.477139225,12.97190964,166.6766968,17.44665819,38.9
3969881,0.087382224,0.087382224,1,1,7.477139225,12.97190964,166.6766968,17.44665819,38.91
247,4.323969881,0.087382224,0.087382224,1,1,7.477139225,12.97190964,166.6766968,17.44665
23969881,0.087382224,0.087382224,1,1,7.477139225,12.97190964,166.6766968,17.44665819,38.9
23969881,0.087382224,0.087382224,1,1,7.477139225,12.97190964,166.6766968,17.44665819,38.9
23969881,0.087382224,0.087382224,1,1,7.477139225,12.97190964,166.6766968,17.44665819,38.9
907,1145.351723,0.830793247,4.323969881,0.087382224,0.087382224,1,1,7.477139225,12.971909
,1,1,1,11,971.525907,1145.351723,0.830793247,4.323969881,0.087382224,0.087382224,1,1,7.477
3247,4.323969881,0.087382224,0.087382224,1,1,7.477139225,12.97190964,166.6766968,17.4466
5907,1145.351723,0.830793247,4.323969881,0.087382224,0.087382224,1,1,7.477139225,12.97190
5907,1145.351723,0.830793247,4.323969881,0.087382224,0.087382224,1,1,7.477139225,12.97190
25907,1145.351723,0.830793247,4.323969881,0.087382224,0.087382224,1,1,7.477139225,12.9719
7,4.323969881,0.087382224,0.087382224,1,1,7.477139225,12.97190964,166.6766968,17.44665819
247,4.323969881,0.087382224,0.087382224,1,1,7.477139225,12.97190964,166.6766968,17.446658
5907,1145.351723,0.830793247,4.323969881,0.087382224,0.087382224,1,1,7.477139225,12.97190
,1,1,1,11,971.525907,1145.351723,0.830793247,4.323969881,0.087382224,0.087382224,1,1,7.477
323969881,0.087382224,0.087382224,1,1,7.477139225,12.97190964,166.6766968,17.44665819,38.
24,3,2,207,76,0,0,1,1,1,11,971.525907,1145.351723,0.830793247,4.323969881,0.087382224,0.0873
,1,1,1,11,971.525907,1145.351723,0.830793247,4.323969881,0.087382224,0.087382224,1,1,7.477
5907,1145.351723,0.830793247,4.323969881,0.087382224,0.087382224,1,1,7.477139225,12.97190
907,1145.351723,0.830793247,4.323969881,0.087382224,0.087382224,1,1,7.477139225,12.971909
907,1145.351723,0.830793247,4.323969881,0.087382224,0.087382224,1,1,7.477139225,12.971909
907,1145.351723,0.830793247,4.323969881,0.087382224,0.087382224,1,1,7.477139225,12.971909
47,4.323969881,0.087382224,0.087382224,1,1,7.477139225,12.97190964,166.6766968,17.4466581
5907,1145.351723,0.830793247,4.323969881,0.087382224,0.087382224,1,1,7.477139225,12.97190
5907,1145.351723,0.830793247,4.323969881,0.087382224,0.087382224,1,1,7.477139225,12.97190
907,1145.351723,0.830793247,4.323969881,0.087382224,0.087382224,1,1,7.477139225,12.971909
47,4.323969881,0.087382224,0.087382224,1,1,7.477139225,12.97190964,166.6766968,17.4466581
5907,1145.351723,0.830793247,4.323969881,0.087382224,0.087382224,1,1,7.477139225,12.97190
,1,1,1,11,971.525907,1145.351723,0.830793247,4.323969881,0.087382224,0.087382224,1,1,7.477
323969881,0.087382224,0.087382224,1,1,7.477139225,12.97190964,166.6766968,17.44665819,38.
907,1145.351723,0.830793247,4.323969881,0.087382224,0.087382224,1,1,7.477139225,12.971909
25907,1145.351723,0.830793247,4.323969881,0.087382224,0.087382224,1,1,7.477139225,12.9719
0,1,1,1,11,971.525907,1145.351723,0.830793247,4.323969881,0.087382224,0.087382224,1,1,7.477
247,4.323969881,0.087382224,0.087382224,1,1,7.477139225,12.97190964,166.6766968,17.446658
247,4.323969881,0.087382224,0.087382224,1,1,7.477139225,12.97190964,166.6766968,17.446658
323969881,0.087382224,0.087382224,1,1,7.477139225,12.97190964,166.6766968,17.44665819,38.
7,4.323969881,0.087382224,0.087382224,1,1,7.477139225,12.97190964,166.6766968,17.44665819
23969881,0.087382224,0.087382224,1,1,7.477139225,12.97190964,166.6766968,17.44665819,38.9
0.087382224,0,1,0,3.477608592,0,583.2837124,14.4900358,47.67723573,5.216412888,25.6723577,
7,1004.655036,0.579601432,0,0.087382224,0,1,0,3.477608592,0,583.2837124,14.4900358,47.6772
7,1004.655036,0.579601432,0,0.087382224,0,1,0,3.477608592,0,583.2837124,14.4900358,47.6772
17,1004.655036,0.579601432,0,0.087382224,0,1,0,3.477608592,0,583.2837124,14.4900358,47.677
32,0,0.087382224,0,1,0,3.477608592,0,583.2837124,14.4900358,47.67723573,5.216412888,25.672
,0,1,1,8,859.58217,1004.655036,0.579601432,0,0.087382224,0,1,0,3.477608592,0,583.2837124,14.
7,1004.655036,0.579601432,0,0.087382224,0,1,0,3.477608592,0,583.2837124,14.4900358,47.6772

87382224,0,1,0,3.477608592,0,583.2837124,14.4900358,47.67723573,5.216412888,25.6723577,2.3
2,0,0.087382224,0,1,0,3.477608592,0,583.2837124,14.4900358,47.67723573,5.216412888,25.67235
87382224,0,1,0,3.477608592,0,583.2837124,14.4900358,47.67723573,5.216412888,25.6723577,2.3
2,0,0.087382224,0,1,0,3.477608592,0,583.2837124,14.4900358,47.67723573,5.216412888,25.67235
7382224,0,1,0,3.477608592,0,583.2837124,14.4900358,47.67723573,5.216412888,25.6723577,2.31
217,1004.655036,0.579601432,0,0.087382224,0,1,0,3.477608592,0,583.2837124,14.4900358,47.67
7,1004.655036,0.579601432,0,0.087382224,0,1,0,3.477608592,0,583.2837124,14.4900358,47.6772
0,0,1,1,8,859.58217,1004.655036,0.579601432,0,0.087382224,0,1,0,3.477608592,0,583.2837124,14
7,1004.655036,0.579601432,0,0.087382224,0,1,0,3.477608592,0,583.2837124,14.4900358,47.6772
82224,0,1,0,3.477608592,0,583.2837124,14.4900358,47.67723573,5.216412888,25.6723577,2.318
.087382224,0,1,0,3.477608592,0,583.2837124,14.4900358,47.67723573,5.216412888,25.6723577,
,0,1,1,8,859.58217,1004.655036,0.579601432,0,0.087382224,0,1,0,3.477608592,0,583.2837124,14.
7,1004.655036,0.579601432,0,0.087382224,0,1,0,3.477608592,0,583.2837124,14.4900358,47.6772
7,1004.655036,0.579601432,0,0.087382224,0,1,0,3.477608592,0,583.2837124,14.4900358,47.6772
7,1004.655036,0.579601432,0,0.087382224,0,1,0,3.477608592,0,583.2837124,14.4900358,47.6772
0,0,1,1,8,859.58217,1004.655036,0.579601432,0,0.087382224,0,1,0,3.477608592,0,583.2837124,14
7,1004.655036,0.579601432,0,0.087382224,0,1,0,3.477608592,0,583.2837124,14.4900358,47.6772
87382224,0,1,0,3.477608592,0,583.2837124,14.4900358,47.67723573,5.216412888,25.6723577,2.3
87382224,0,1,0,3.477608592,0,583.2837124,14.4900358,47.67723573,5.216412888,25.6723577,2.3
32,0,0.087382224,0,1,0,3.477608592,0,583.2837124,14.4900358,47.67723573,5.216412888,25.672
217,1004.655036,0.579601432,0,0.087382224,0,1,0,3.477608592,0,583.2837124,14.4900358,47.67
217,1004.655036,0.579601432,0,0.087382224,0,1,0,3.477608592,0,583.2837124,14.4900358,47.67
,0.087382224,0,1,0,3.477608592,0,583.2837124,14.4900358,47.67723573,5.216412888,25.672357
143,1249.63125,0.892739755,6.923609605,0.087382224,0.087382224,1,1,7.141918039,13.8472192
23609605,0.087382224,0.087382224,1,1,7.141918039,13.84721921,276.4445999,17.8547951,62.31
23609605,0.087382224,0.087382224,1,1,7.141918039,13.84721921,276.4445999,17.8547951,62.31
23609605,0.087382224,0.087382224,1,1,7.141918039,13.84721921,276.4445999,17.8547951,62.31
3609605,0.087382224,0.087382224,1,1,7.141918039,13.84721921,276.4445999,17.8547951,62.312
6.923609605,0.087382224,0.087382224,1,1,7.141918039,13.84721921,276.4445999,17.8547951,6
143,1249.63125,0.892739755,6.923609605,0.087382224,0.087382224,1,1,7.141918039,13.8472192
143,1249.63125,0.892739755,6.923609605,0.087382224,0.087382224,1,1,7.141918039,13.8472192
143,1249.63125,0.892739755,6.923609605,0.087382224,0.087382224,1,1,7.141918039,13.8472192
9605,0.087382224,0.087382224,1,1,7.141918039,13.84721921,276.4445999,17.8547951,62.31248
143,1249.63125,0.892739755,6.923609605,0.087382224,0.087382224,1,1,7.141918039,13.8472192
6.923609605,0.087382224,0.087382224,1,1,7.141918039,13.84721921,276.4445999,17.8547951,6
1,0,1,1,9,942.3840143,1249.63125,0.892739755,6.923609605,0.087382224,0.087382224,1,1,7.141
143,1249.63125,0.892739755,6.923609605,0.087382224,0.087382224,1,1,7.141918039,13.8472192
9605,0.087382224,0.087382224,1,1,7.141918039,13.84721921,276.4445999,17.8547951,62.31248
143,1249.63125,0.892739755,6.923609605,0.087382224,0.087382224,1,1,7.141918039,13.8472192
0143,1249.63125,0.892739755,6.923609605,0.087382224,0.087382224,1,1,7.141918039,13.847219
143,1249.63125,0.892739755,6.923609605,0.087382224,0.087382224,1,1,7.141918039,13.8472192
143,1249.63125,0.892739755,6.923609605,0.087382224,0.087382224,1,1,7.141918039,13.8472192
143,1249.63125,0.892739755,6.923609605,0.087382224,0.087382224,1,1,7.141918039,13.8472192
23609605,0.087382224,0.087382224,1,1,7.141918039,13.84721921,276.4445999,17.8547951,62.31
23609605,0.087382224,0.087382224,1,1,7.141918039,13.84721921,276.4445999,17.8547951,62.31
0143,1249.63125,0.892739755,6.923609605,0.087382224,0.087382224,1,1,7.141918039,13.847219
23609605,0.087382224,0.087382224,1,1,7.141918039,13.84721921,276.4445999,17.8547951,62.31
0143,1249.63125,0.892739755,6.923609605,0.087382224,0.087382224,1,1,7.141918039,13.847219
40143,1249.63125,0.892739755,6.923609605,0.087382224,0.087382224,1,1,7.141918039,13.84721
,0,1,1,9,942.3840143,1249.63125,0.892739755,6.923609605,0.087382224,0.087382224,1,1,7.1419

,6.923609605,0.087382224,0.087382224,1,1,7.141918039,13.84721921,276.4445999,17.8547951,
1,0,1,1,9,942.3840143,1249.63125,0.892739755,6.923609605,0.087382224,0.087382224,1,1,7.141
40143,1249.63125,0.892739755,6.923609605,0.087382224,0.087382224,1,1,7.141918039,13.84721
0143,1249.63125,0.892739755,6.923609605,0.087382224,0.087382224,1,1,7.141918039,13.847219
40143,1249.63125,0.892739755,6.923609605,0.087382224,0.087382224,1,1,7.141918039,13.84721
0,0.087382224,0,1,0,4.122887796,0,444.0798154,13.74295932,54.35852644,6.184331694,22.6493
1,0,1,1,11,881.3318984,1314.527243,0.687147966,0,0.087382224,0,1,0,4.122887796,0,444.079815
,1,0,1,1,11,881.3318984,1314.527243,0.687147966,0,0.087382224,0,1,0,4.122887796,0,444.07981
318984,1314.527243,0.687147966,0,0.087382224,0,1,0,4.122887796,0,444.0798154,13.74295932,5
1,0,1,1,11,881.3318984,1314.527243,0.687147966,0,0.087382224,0,1,0,4.122887796,0,444.079815
966,0,0.087382224,0,1,0,4.122887796,0,444.0798154,13.74295932,54.35852644,6.184331694,22.6
318984,1314.527243,0.687147966,0,0.087382224,0,1,0,4.122887796,0,444.0798154,13.74295932,5
47966,0,0.087382224,0,1,0,4.122887796,0,444.0798154,13.74295932,54.35852644,6.184331694,22
18984,1314.527243,0.687147966,0,0.087382224,0,1,0,4.122887796,0,444.0798154,13.74295932,54
18984,1314.527243,0.687147966,0,0.087382224,0,1,0,4.122887796,0,444.0798154,13.74295932,54
0,0.087382224,0,1,0,4.122887796,0,444.0798154,13.74295932,54.35852644,6.184331694,22.6493
318984,1314.527243,0.687147966,0,0.087382224,0,1,0,4.122887796,0,444.0798154,13.74295932,5
318984,1314.527243,0.687147966,0,0.087382224,0,1,0,4.122887796,0,444.0798154,13.74295932,5
3318984,1314.527243,0.687147966,0,0.087382224,0,1,0,4.122887796,0,444.0798154,13.74295932,
966,0,0.087382224,0,1,0,4.122887796,0,444.0798154,13.74295932,54.35852644,6.184331694,22.6
18984,1314.527243,0.687147966,0,0.087382224,0,1,0,4.122887796,0,444.0798154,13.74295932,54
0,0.087382224,0,1,0,4.122887796,0,444.0798154,13.74295932,54.35852644,6.184331694,22.6493
1,0,1,1,11,881.3318984,1314.527243,0.687147966,0,0.087382224,0,1,0,4.122887796,0,444.079815
18984,1314.527243,0.687147966,0,0.087382224,0,1,0,4.122887796,0,444.0798154,13.74295932,54
,0.087382224,0,1,0,4.122887796,0,444.0798154,13.74295932,54.35852644,6.184331694,22.649386
966,0,0.087382224,0,1,0,4.122887796,0,444.0798154,13.74295932,54.35852644,6.184331694,22.6
18984,1314.527243,0.687147966,0,0.087382224,0,1,0,4.122887796,0,444.0798154,13.74295932,54
1,0,1,1,11,881.3318984,1314.527243,0.687147966,0,0.087382224,0,1,0,4.122887796,0,444.079815
,1,0,1,1,11,881.3318984,1314.527243,0.687147966,0,0.087382224,0,1,0,4.122887796,0,444.07981
,1,0,1,1,11,881.3318984,1314.527243,0.687147966,0,0.087382224,0,1,0,4.122887796,0,444.07981
0,0.087382224,0,1,0,4.122887796,0,444.0798154,13.74295932,54.35852644,6.184331694,22.6493
1,0,1,1,11,881.3318984,1314.527243,0.687147966,0,0.087382224,0,1,0,4.122887796,0,444.079815
,1,0,1,1,11,881.3318984,1314.527243,0.687147966,0,0.087382224,0,1,0,4.122887796,0,444.07981
318984,1314.527243,0.687147966,0,0.087382224,0,1,0,4.122887796,0,444.0798154,13.74295932,5
18984,1314.527243,0.687147966,0,0.087382224,0,1,0,4.122887796,0,444.0798154,13.74295932,54
0,0.087382224,0,1,0,4.122887796,0,444.0798154,13.74295932,54.35852644,6.184331694,22.6493
1,0,1,1,11,881.3318984,1314.527243,0.687147966,0,0.087382224,0,1,0,4.122887796,0,444.079815
3318984,1314.527243,0.687147966,0,0.087382224,0,1,0,4.122887796,0,444.0798154,13.74295932,
3318984,1314.527243,0.687147966,0,0.087382224,0,1,0,4.122887796,0,444.0798154,13.74295932,
0,0.087382224,0,1,0,4.122887796,0,444.0798154,13.74295932,54.35852644,6.184331694,22.6493
7363,4.91111611,0.087382224,0.087382224,1,1,5.789018902,9.822232219,380.9483472,15.919801
3593828,1234.444667,0.723627363,4.91111611,0.087382224,0.087382224,1,1,5.789018902,9.8222
1,1,1,1,1,12,907.3593828,1234.444667,0.723627363,4.91111611,0.087382224,0.087382224,1,1,5.7
1,1,1,1,1,12,907.3593828,1234.444667,0.723627363,4.91111611,0.087382224,0.087382224,1,1,5.7
593828,1234.444667,0.723627363,4.91111611,0.087382224,0.087382224,1,1,5.789018902,9.82223
3627363,4.91111611,0.087382224,0.087382224,1,1,5.789018902,9.822232219,380.9483472,15.919
3593828,1234.444667,0.723627363,4.91111611,0.087382224,0.087382224,1,1,5.789018902,9.8222
3593828,1234.444667,0.723627363,4.91111611,0.087382224,0.087382224,1,1,5.789018902,9.8222
3593828,1234.444667,0.723627363,4.91111611,0.087382224,0.087382224,1,1,5.789018902,9.8222
593828,1234.444667,0.723627363,4.91111611,0.087382224,0.087382224,1,1,5.789018902,9.82223

4.91111611,0.087382224,0.087382224,1,1,5.789018902,9.822232219,380.9483472,15.91980198,5
527363,4.91111611,0.087382224,0.087382224,1,1,5.789018902,9.822232219,380.9483472,15.9198
3593828,1234.444667,0.723627363,4.91111611,0.087382224,0.087382224,1,1,5.789018902,9.8222
593828,1234.444667,0.723627363,4.91111611,0.087382224,0.087382224,1,1,5.789018902,9.82223
593828,1234.444667,0.723627363,4.91111611,0.087382224,0.087382224,1,1,5.789018902,9.82223
7676,3,2,207,69,1,1,1,1,1,12,907.3593828,1234.444667,0.723627363,4.91111611,0.087382224,0.08
3593828,1234.444667,0.723627363,4.91111611,0.087382224,0.087382224,1,1,5.789018902,9.8222
3593828,1234.444667,0.723627363,4.91111611,0.087382224,0.087382224,1,1,5.789018902,9.8222
1,1,1,1,1,12,907.3593828,1234.444667,0.723627363,4.91111611,0.087382224,0.087382224,1,1,5.78
3593828,1234.444667,0.723627363,4.91111611,0.087382224,0.087382224,1,1,5.789018902,9.8222
.3593828,1234.444667,0.723627363,4.91111611,0.087382224,0.087382224,1,1,5.789018902,9.822
3593828,1234.444667,0.723627363,4.91111611,0.087382224,0.087382224,1,1,5.789018902,9.8222
3593828,1234.444667,0.723627363,4.91111611,0.087382224,0.087382224,1,1,5.789018902,9.8222
1,1,1,1,1,12,907.3593828,1234.444667,0.723627363,4.91111611,0.087382224,0.087382224,1,1,5.78
.723627363,4.91111611,0.087382224,0.087382224,1,1,5.789018902,9.822232219,380.9483472,15
4.91111611,0.087382224,0.087382224,1,1,5.789018902,9.822232219,380.9483472,15.91980198,5
2,0,0.087382224,0,1,0,6.594522672,0,512.7643317,19.78356801,58.68589025,9.891784007,22.007
1,0,1,1,10,905.5895508,1438.075827,1.099087112,0,0.087382224,0,1,0,6.594522672,0,512.764331
.5895508,1438.075827,1.099087112,0,0.087382224,0,1,0,6.594522672,0,512.7643317,19.7835680
5895508,1438.075827,1.099087112,0,0.087382224,0,1,0,6.594522672,0,512.7643317,19.78356801,
.5895508,1438.075827,1.099087112,0,0.087382224,0,1,0,6.594522672,0,512.7643317,19.7835680
0,0.087382224,0,1,0,6.594522672,0,512.7643317,19.78356801,58.68589025,9.891784007,22.0072
895508,1438.075827,1.099087112,0,0.087382224,0,1,0,6.594522672,0,512.7643317,19.78356801,5
1,1,0,1,1,10,905.5895508,1438.075827,1.099087112,0,0.087382224,0,1,0,6.594522672,0,512.76433
1,1,0,1,1,10,905.5895508,1438.075827,1.099087112,0,0.087382224,0,1,0,6.594522672,0,512.76433
,1,1,0,1,1,10,905.5895508,1438.075827,1.099087112,0,0.087382224,0,1,0,6.594522672,0,512.7643
87112,0,0.087382224,0,1,0,6.594522672,0,512.7643317,19.78356801,58.68589025,9.891784007,22
5895508,1438.075827,1.099087112,0,0.087382224,0,1,0,6.594522672,0,512.7643317,19.78356801,
7112,0,0.087382224,0,1,0,6.594522672,0,512.7643317,19.78356801,58.68589025,9.891784007,22.0
5,1,1,0,1,1,10,905.5895508,1438.075827,1.099087112,0,0.087382224,0,1,0,6.594522672,0,512.7643
2,0,0.087382224,0,1,0,6.594522672,0,512.7643317,19.78356801,58.68589025,9.891784007,22.007
87112,0,0.087382224,0,1,0,6.594522672,0,512.7643317,19.78356801,58.68589025,9.891784007,22
1,1,0,1,1,10,905.5895508,1438.075827,1.099087112,0,0.087382224,0,1,0,6.594522672,0,512.76433
5895508,1438.075827,1.099087112,0,0.087382224,0,1,0,6.594522672,0,512.7643317,19.78356801,
0,0.087382224,0,1,0,6.594522672,0,512.7643317,19.78356801,58.68589025,9.891784007,22.0072
7112,0,0.087382224,0,1,0,6.594522672,0,512.7643317,19.78356801,58.68589025,9.891784007,22.0
087112,0,0.087382224,0,1,0,6.594522672,0,512.7643317,19.78356801,58.68589025,9.891784007,2
1,1,0,1,1,10,905.5895508,1438.075827,1.099087112,0,0.087382224,0,1,0,6.594522672,0,512.76433
.5895508,1438.075827,1.099087112,0,0.087382224,0,1,0,6.594522672,0,512.7643317,19.7835680
,0.087382224,0,1,0,6.594522672,0,512.7643317,19.78356801,58.68589025,9.891784007,22.00720
895508,1438.075827,1.099087112,0,0.087382224,0,1,0,6.594522672,0,512.7643317,19.78356801,5
895508,1438.075827,1.099087112,0,0.087382224,0,1,0,6.594522672,0,512.7643317,19.78356801,5
5.5895508,1438.075827,1.099087112,0,0.087382224,0,1,0,6.594522672,0,512.7643317,19.7835680
0,0.087382224,0,1,0,5.484783008,0,442.0247934,16.45434902,55.27968473,8.227174512,24.5687
1,0,0.087382224,0,1,0,5.484783008,0,442.0247934,16.45434902,55.27968473,8.227174512,24.568
1,0,1,1,1,11,900.4003243,1487.145876,0.914130501,0,0.087382224,0,1,0,5.484783008,0,442.02479
1,0,0.087382224,0,1,0,5.484783008,0,442.0247934,16.45434902,55.27968473,8.227174512,24.568
003243,1487.145876,0.914130501,0,0.087382224,0,1,0,5.484783008,0,442.0247934,16.45434902,5
501,0,0.087382224,0,1,0,5.484783008,0,442.0247934,16.45434902,55.27968473,8.227174512,24.5
0,0.087382224,0,1,0,5.484783008,0,442.0247934,16.45434902,55.27968473,8.227174512,24.5687

003243,1487.145876,0.914130501,0,0.087382224,0,1,0,5.484783008,0,442.0247934,16.45434902,5
003243,1487.145876,0.914130501,0,0.087382224,0,1,0,5.484783008,0,442.0247934,16.45434902,5
1,0,0.087382224,0,1,0,5.484783008,0,442.0247934,16.45434902,55.27968473,8.227174512,24.5687
1,0,0.087382224,0,1,0,5.484783008,0,442.0247934,16.45434902,55.27968473,8.227174512,24.5687
1,0,1,1,1,11,900.4003243,1487.145876,0.914130501,0,0.087382224,0,1,0,5.484783008,0,442.02479
003243,1487.145876,0.914130501,0,0.087382224,0,1,0,5.484783008,0,442.0247934,16.45434902,5
1,0,1,1,1,11,900.4003243,1487.145876,0.914130501,0,0.087382224,0,1,0,5.484783008,0,442.02479
4003243,1487.145876,0.914130501,0,0.087382224,0,1,0,5.484783008,0,442.0247934,16.45434902,
0501,0,0.087382224,0,1,0,5.484783008,0,442.0247934,16.45434902,55.27968473,8.227174512,24.5
1,0,0.087382224,0,1,0,5.484783008,0,442.0247934,16.45434902,55.27968473,8.227174512,24.5687
003243,1487.145876,0.914130501,0,0.087382224,0,1,0,5.484783008,0,442.0247934,16.45434902,5
0,0.087382224,0,1,0,5.484783008,0,442.0247934,16.45434902,55.27968473,8.227174512,24.5687
1,0,1,1,1,11,900.4003243,1487.145876,0.914130501,0,0.087382224,0,1,0,5.484783008,0,442.02479
0,0.087382224,0,1,0,5.484783008,0,442.0247934,16.45434902,55.27968473,8.227174512,24.5687
0501,0,0.087382224,0,1,0,5.484783008,0,442.0247934,16.45434902,55.27968473,8.227174512,24.5
1,0,0.087382224,0,1,0,5.484783008,0,442.0247934,16.45434902,55.27968473,8.227174512,24.5687
.4003243,1487.145876,0.914130501,0,0.087382224,0,1,0,5.484783008,0,442.0247934,16.4543490
003243,1487.145876,0.914130501,0,0.087382224,0,1,0,5.484783008,0,442.0247934,16.45434902,5
0,0.087382224,0,1,0,5.484783008,0,442.0247934,16.45434902,55.27968473,8.227174512,24.5687
1,0,1,1,1,11,900.4003243,1487.145876,0.914130501,0,0.087382224,0,1,0,5.484783008,0,442.02479
1,0,1,1,1,11,900.4003243,1487.145876,0.914130501,0,0.087382224,0,1,0,5.484783008,0,442.02479
003243,1487.145876,0.914130501,0,0.087382224,0,1,0,5.484783008,0,442.0247934,16.45434902,5
4003243,1487.145876,0.914130501,0,0.087382224,0,1,0,5.484783008,0,442.0247934,16.45434902,
1,0,1,1,1,11,900.4003243,1487.145876,0.914130501,0,0.087382224,0,1,0,5.484783008,0,442.02479
1,0,1,1,1,11,900.4003243,1487.145876,0.914130501,0,0.087382224,0,1,0,5.484783008,0,442.02479
1,0,0.087382224,0,1,0,5.484783008,0,442.0247934,16.45434902,55.27968473,8.227174512,24.5687
1,0,0.087382224,0,1,0,5.484783008,0,442.0247934,16.45434902,55.27968473,8.227174512,24.5687
1,0,0.087382224,0,1,0,5.484783008,0,442.0247934,16.45434902,55.27968473,8.227174512,24.5687
087382224,0,1,0,4.137128055,4.026808167,351.5420099,14.47994819,28.18765717,6.205692082,
47608,1090.12904,0.689521342,0,0.087382224,0,1,0,4.137128055,4.026808167,351.5420099,14.47
,0.087382224,0,1,0,4.137128055,4.026808167,351.5420099,14.47994819,28.18765717,6.20569208
0.087382224,0,1,0,4.137128055,4.026808167,351.5420099,14.47994819,28.18765717,6.205692082
,0.087382224,0,1,0,4.137128055,4.026808167,351.5420099,14.47994819,28.18765717,6.20569208
1,1,0,1,1,9,879.5047608,1090.12904,0.689521342,0,0.087382224,0,1,0,4.137128055,4.026808167,3
42,0,0.087382224,0,1,0,4.137128055,4.026808167,351.5420099,14.47994819,28.18765717,6.20569
,0.087382224,0,1,0,4.137128055,4.026808167,351.5420099,14.47994819,28.18765717,6.20569208
47608,1090.12904,0.689521342,0,0.087382224,0,1,0,4.137128055,4.026808167,351.5420099,14.47
047608,1090.12904,0.689521342,0,0.087382224,0,1,0,4.137128055,4.026808167,351.5420099,14.4
1342,0,0.087382224,0,1,0,4.137128055,4.026808167,351.5420099,14.47994819,28.18765717,6.205
5047608,1090.12904,0.689521342,0,0.087382224,0,1,0,4.137128055,4.026808167,351.5420099,14.
,0.087382224,0,1,0,4.137128055,4.026808167,351.5420099,14.47994819,28.18765717,6.20569208
47608,1090.12904,0.689521342,0,0.087382224,0,1,0,4.137128055,4.026808167,351.5420099,14.47
1,1,0,1,1,9,879.5047608,1090.12904,0.689521342,0,0.087382224,0,1,0,4.137128055,4.026808167,3
047608,1090.12904,0.689521342,0,0.087382224,0,1,0,4.137128055,4.026808167,351.5420099,14.4
,1,1,0,1,1,9,879.5047608,1090.12904,0.689521342,0,0.087382224,0,1,0,4.137128055,4.026808167,
47608,1090.12904,0.689521342,0,0.087382224,0,1,0,4.137128055,4.026808167,351.5420099,14.47
1,1,0,1,1,9,879.5047608,1090.12904,0.689521342,0,0.087382224,0,1,0,4.137128055,4.026808167,3
1,1,0,1,1,9,879.5047608,1090.12904,0.689521342,0,0.087382224,0,1,0,4.137128055,4.026808167,3
047608,1090.12904,0.689521342,0,0.087382224,0,1,0,4.137128055,4.026808167,351.5420099,14.4
1342,0,0.087382224,0,1,0,4.137128055,4.026808167,351.5420099,14.47994819,28.18765717,6.205

42,0,0.087382224,0,1,0,4.137128055,4.026808167,351.5420099,14.47994819,28.18765717,6.20569
1,1,0,1,1,9,879.5047608,1090.12904,0.689521342,0,0.087382224,0,1,0,4.137128055,4.026808167,3
47608,1090.12904,0.689521342,0,0.087382224,0,1,0,4.137128055,4.026808167,351.5420099,14.47
47608,1090.12904,0.689521342,0,0.087382224,0,1,0,4.137128055,4.026808167,351.5420099,14.47
,0.087382224,0,1,0,4.137128055,4.026808167,351.5420099,14.47994819,28.18765717,6.20569208
42,0,0.087382224,0,1,0,4.137128055,4.026808167,351.5420099,14.47994819,28.18765717,6.20569
21342,0,0.087382224,0,1,0,4.137128055,4.026808167,351.5420099,14.47994819,28.18765717,6.2
5047608,1090.12904,0.689521342,0,0.087382224,0,1,0,4.137128055,4.026808167,351.5420099,14.
1,1,0,1,1,9,879.5047608,1090.12904,0.689521342,0,0.087382224,0,1,0,4.137128055,4.026808167,3
47608,1090.12904,0.689521342,0,0.087382224,0,1,0,4.137128055,4.026808167,351.5420099,14.47
,0.087382224,0,1,0,4.137128055,4.026808167,351.5420099,14.47994819,28.18765717,6.20569208
047608,1090.12904,0.689521342,0,0.087382224,0,1,0,4.137128055,4.026808167,351.5420099,14.4
0.087382224,0,1,0,4.137128055,4.026808167,351.5420099,14.47994819,28.18765717,6.205692082
,0,1,1,1,1,12,888.4749243,927.3234014,0.760073641,3.873942715,0.087382224,0.087382224,1,1,6
0,1,1,1,1,12,888.4749243,927.3234014,0.760073641,3.873942715,0.087382224,0.087382224,1,1,6.0
0073641,3.873942715,0.087382224,0.087382224,1,1,6.080589131,7.74788543,195.6414555,14.441
,0,1,1,1,1,12,888.4749243,927.3234014,0.760073641,3.873942715,0.087382224,0.087382224,1,1,6
1,1,1,1,12,888.4749243,927.3234014,0.760073641,3.873942715,0.087382224,0.087382224,1,1,6.08
1,3.873942715,0.087382224,0.087382224,1,1,6.080589131,7.74788543,195.6414555,14.44139919,1
0,1,1,1,1,12,888.4749243,927.3234014,0.760073641,3.873942715,0.087382224,0.087382224,1,1,6.0
.4749243,927.3234014,0.760073641,3.873942715,0.087382224,0.087382224,1,1,6.080589131,7.74
749243,927.3234014,0.760073641,3.873942715,0.087382224,0.087382224,1,1,6.080589131,7.7478
1,3.873942715,0.087382224,0.087382224,1,1,6.080589131,7.74788543,195.6414555,14.44139919,1
4749243,927.3234014,0.760073641,3.873942715,0.087382224,0.087382224,1,1,6.080589131,7.747
0073641,3.873942715,0.087382224,0.087382224,1,1,6.080589131,7.74788543,195.6414555,14.441
,0,1,1,1,1,12,888.4749243,927.3234014,0.760073641,3.873942715,0.087382224,0.087382224,1,1,6
.4749243,927.3234014,0.760073641,3.873942715,0.087382224,0.087382224,1,1,6.080589131,7.74
749243,927.3234014,0.760073641,3.873942715,0.087382224,0.087382224,1,1,6.080589131,7.7478
749243,927.3234014,0.760073641,3.873942715,0.087382224,0.087382224,1,1,6.080589131,7.7478
4749243,927.3234014,0.760073641,3.873942715,0.087382224,0.087382224,1,1,6.080589131,7.747
,0,1,1,1,1,12,888.4749243,927.3234014,0.760073641,3.873942715,0.087382224,0.087382224,1,1,6
,0,1,1,1,1,12,888.4749243,927.3234014,0.760073641,3.873942715,0.087382224,0.087382224,1,1,6
0,1,1,1,1,12,888.4749243,927.3234014,0.760073641,3.873942715,0.087382224,0.087382224,1,1,6.0
749243,927.3234014,0.760073641,3.873942715,0.087382224,0.087382224,1,1,6.080589131,7.7478
4749243,927.3234014,0.760073641,3.873942715,0.087382224,0.087382224,1,1,6.080589131,7.747
3641,3.873942715,0.087382224,0.087382224,1,1,6.080589131,7.74788543,195.6414555,14.441399
541,3.873942715,0.087382224,0.087382224,1,1,6.080589131,7.74788543,195.6414555,14.4413991
0,1,1,1,1,12,888.4749243,927.3234014,0.760073641,3.873942715,0.087382224,0.087382224,1,1,6.
4749243,927.3234014,0.760073641,3.873942715,0.087382224,0.087382224,1,1,6.080589131,7.747
.4749243,927.3234014,0.760073641,3.873942715,0.087382224,0.087382224,1,1,6.080589131,7.74
073641,3.873942715,0.087382224,0.087382224,1,1,6.080589131,7.74788543,195.6414555,14.4413
727891498,4.193449154,0.087382224,0.087382224,1,1,5.823131984,8.386898308,254.9606396,15.
2661652,1098.183314,0.727891498,4.193449154,0.087382224,0.087382224,1,1,5.823131984,8.386
561652,1098.183314,0.727891498,4.193449154,0.087382224,0.087382224,1,1,5.823131984,8.3868
,0,1,0,1,1,12,889.2661652,1098.183314,0.727891498,4.193449154,0.087382224,0.087382224,1,1,5
,0,1,0,1,1,12,889.2661652,1098.183314,0.727891498,4.193449154,0.087382224,0.087382224,1,1,5
1498,4.193449154,0.087382224,0.087382224,1,1,5.823131984,8.386898308,254.9606396,15.28572
.2661652,1098.183314,0.727891498,4.193449154,0.087382224,0.087382224,1,1,5.823131984,8.38
561652,1098.183314,0.727891498,4.193449154,0.087382224,0.087382224,1,1,5.823131984,8.3868
1498,4.193449154,0.087382224,0.087382224,1,1,5.823131984,8.386898308,254.9606396,15.28572

2661652,1098.183314,0.727891498,4.193449154,0.087382224,0.087382224,1,1,5.823131984,8.386
.2661652,1098.183314,0.727891498,4.193449154,0.087382224,0.087382224,1,1,5.823131984,8.38
0,1,0,1,1,12,889.2661652,1098.183314,0.727891498,4.193449154,0.087382224,0.087382224,1,1,5.8
2661652,1098.183314,0.727891498,4.193449154,0.087382224,0.087382224,1,1,5.823131984,8.386
4.193449154,0.087382224,0.087382224,1,1,5.823131984,8.386898308,254.9606396,15.28572146,2
661652,1098.183314,0.727891498,4.193449154,0.087382224,0.087382224,1,1,5.823131984,8.3868
661652,1098.183314,0.727891498,4.193449154,0.087382224,0.087382224,1,1,5.823131984,8.3868
4.193449154,0.087382224,0.087382224,1,1,5.823131984,8.386898308,254.9606396,15.28572146,2
0,1,0,1,1,12,889.2661652,1098.183314,0.727891498,4.193449154,0.087382224,0.087382224,1,1,5.8
,0,1,0,1,1,12,889.2661652,1098.183314,0.727891498,4.193449154,0.087382224,0.087382224,1,1,5
2661652,1098.183314,0.727891498,4.193449154,0.087382224,0.087382224,1,1,5.823131984,8.386
8,4.193449154,0.087382224,0.087382224,1,1,5.823131984,8.386898308,254.9606396,15.28572146
98,4.193449154,0.087382224,0.087382224,1,1,5.823131984,8.386898308,254.9606396,15.2857214
.2661652,1098.183314,0.727891498,4.193449154,0.087382224,0.087382224,1,1,5.823131984,8.38
.2661652,1098.183314,0.727891498,4.193449154,0.087382224,0.087382224,1,1,5.823131984,8.38
661652,1098.183314,0.727891498,4.193449154,0.087382224,0.087382224,1,1,5.823131984,8.3868
661652,1098.183314,0.727891498,4.193449154,0.087382224,0.087382224,1,1,5.823131984,8.3868
727891498,4.193449154,0.087382224,0.087382224,1,1,5.823131984,8.386898308,254.9606396,15.
8,4.193449154,0.087382224,0.087382224,1,1,5.823131984,8.386898308,254.9606396,15.28572146
.2661652,1098.183314,0.727891498,4.193449154,0.087382224,0.087382224,1,1,5.823131984,8.38
.2661652,1098.183314,0.727891498,4.193449154,0.087382224,0.087382224,1,1,5.823131984,8.38
4.193449154,0.087382224,0.087382224,1,1,5.823131984,8.386898308,254.9606396,15.28572146,2
.193449154,0.087382224,0.087382224,1,1,5.823131984,8.386898308,254.9606396,15.28572146,25
2661652,1098.183314,0.727891498,4.193449154,0.087382224,0.087382224,1,1,5.823131984,8.386
,1,1,0,1,1,11,889.2661652,1098.183314,0.727891498,4.193449154,0.087382224,0.087382224,1,1,5
2661652,1098.183314,0.727891498,4.193449154,0.087382224,0.087382224,1,1,5.823131984,8.386
2661652,1098.183314,0.727891498,4.193449154,0.087382224,0.087382224,1,1,5.823131984,8.386
,1,1,0,1,1,11,889.2661652,1098.183314,0.727891498,4.193449154,0.087382224,0.087382224,1,1,5
2661652,1098.183314,0.727891498,4.193449154,0.087382224,0.087382224,1,1,5.823131984,8.386
661652,1098.183314,0.727891498,4.193449154,0.087382224,0.087382224,1,1,5.823131984,8.3868
2661652,1098.183314,0.727891498,4.193449154,0.087382224,0.087382224,1,1,5.823131984,8.386
.2661652,1098.183314,0.727891498,4.193449154,0.087382224,0.087382224,1,1,5.823131984,8.38
661652,1098.183314,0.727891498,4.193449154,0.087382224,0.087382224,1,1,5.823131984,8.3868
8,4.193449154,0.087382224,0.087382224,1,1,5.823131984,8.386898308,254.9606396,15.28572146
1498,4.193449154,0.087382224,0.087382224,1,1,5.823131984,8.386898308,254.9606396,15.28572
1,1,0,1,1,11,889.2661652,1098.183314,0.727891498,4.193449154,0.087382224,0.087382224,1,1,5.8
2661652,1098.183314,0.727891498,4.193449154,0.087382224,0.087382224,1,1,5.823131984,8.386
661652,1098.183314,0.727891498,4.193449154,0.087382224,0.087382224,1,1,5.823131984,8.3868
0605,3,2,207,70,1,1,0,1,1,11,889.2661652,1098.183314,0.727891498,4.193449154,0.087382224,0.0
1,0,1,1,11,889.2661652,1098.183314,0.727891498,4.193449154,0.087382224,0.087382224,1,1,5.8
9.2661652,1098.183314,0.727891498,4.193449154,0.087382224,0.087382224,1,1,5.823131984,8.38
7891498,4.193449154,0.087382224,0.087382224,1,1,5.823131984,8.386898308,254.9606396,15.28
661652,1098.183314,0.727891498,4.193449154,0.087382224,0.087382224,1,1,5.823131984,8.3868
1498,4.193449154,0.087382224,0.087382224,1,1,5.823131984,8.386898308,254.9606396,15.28572
1,1,0,1,1,11,889.2661652,1098.183314,0.727891498,4.193449154,0.087382224,0.087382224,1,1,5.8
,1,1,0,1,1,11,889.2661652,1098.183314,0.727891498,4.193449154,0.087382224,0.087382224,1,1,5
,1,1,0,1,1,11,889.2661652,1098.183314,0.727891498,4.193449154,0.087382224,0.087382224,1,1,5
661652,1098.183314,0.727891498,4.193449154,0.087382224,0.087382224,1,1,5.823131984,8.3868
1,0,1,1,11,889.2661652,1098.183314,0.727891498,4.193449154,0.087382224,0.087382224,1,1,5.8
661652,1098.183314,0.727891498,4.193449154,0.087382224,0.087382224,1,1,5.823131984,8.3868

2661652,1098.183314,0.727891498,4.193449154,0.087382224,0.087382224,1,1,5.823131984,8.386
0605,3,2,207,70,1,1,0,1,1,11,889.2661652,1098.183314,0.727891498,4.193449154,0.087382224,0.0
.854085911,0.087382224,0.087382224,1,1,6.163126868,7.708171823,175.1336159,14.63742631,1
3.854085911,0.087382224,0.087382224,1,1,6.163126868,7.708171823,175.1336159,14.63742631,
.854085911,0.087382224,0.087382224,1,1,6.163126868,7.708171823,175.1336159,14.63742631,1
,0,1,0,1,1,12,896.823243,929.6955618,0.770390858,3.854085911,0.087382224,0.087382224,1,1,6.
390858,3.854085911,0.087382224,0.087382224,1,1,6.163126868,7.708171823,175.1336159,14.63
823243,929.6955618,0.770390858,3.854085911,0.087382224,0.087382224,1,1,6.163126868,7.7081
3.854085911,0.087382224,0.087382224,1,1,6.163126868,7.708171823,175.1336159,14.63742631,
.854085911,0.087382224,0.087382224,1,1,6.163126868,7.708171823,175.1336159,14.63742631,1
.823243,929.6955618,0.770390858,3.854085911,0.087382224,0.087382224,1,1,6.163126868,7.708
.823243,929.6955618,0.770390858,3.854085911,0.087382224,0.087382224,1,1,6.163126868,7.708
,0,1,0,1,1,12,896.823243,929.6955618,0.770390858,3.854085911,0.087382224,0.087382224,1,1,6.
3.854085911,0.087382224,0.087382224,1,1,6.163126868,7.708171823,175.1336159,14.63742631,
54085911,0.087382224,0.087382224,1,1,6.163126868,7.708171823,175.1336159,14.63742631,15.4
,0,1,0,1,1,12,896.823243,929.6955618,0.770390858,3.854085911,0.087382224,0.087382224,1,1,6.
3.854085911,0.087382224,0.087382224,1,1,6.163126868,7.708171823,175.1336159,14.63742631,
90858,3.854085911,0.087382224,0.087382224,1,1,6.163126868,7.708171823,175.1336159,14.6374
,0,1,0,1,1,12,896.823243,929.6955618,0.770390858,3.854085911,0.087382224,0.087382224,1,1,6.
823243,929.6955618,0.770390858,3.854085911,0.087382224,0.087382224,1,1,6.163126868,7.7081
.823243,929.6955618,0.770390858,3.854085911,0.087382224,0.087382224,1,1,6.163126868,7.708
23243,929.6955618,0.770390858,3.854085911,0.087382224,0.087382224,1,1,6.163126868,7.70817
90858,3.854085911,0.087382224,0.087382224,1,1,6.163126868,7.708171823,175.1336159,14.6374
,0,1,0,1,1,12,896.823243,929.6955618,0.770390858,3.854085911,0.087382224,0.087382224,1,1,6.
858,3.854085911,0.087382224,0.087382224,1,1,6.163126868,7.708171823,175.1336159,14.637426
390858,3.854085911,0.087382224,0.087382224,1,1,6.163126868,7.708171823,175.1336159,14.63
23243,929.6955618,0.770390858,3.854085911,0.087382224,0.087382224,1,1,6.163126868,7.70817
858,3.854085911,0.087382224,0.087382224,1,1,6.163126868,7.708171823,175.1336159,14.637426
823243,929.6955618,0.770390858,3.854085911,0.087382224,0.087382224,1,1,6.163126868,7.7081
.823243,929.6955618,0.770390858,3.854085911,0.087382224,0.087382224,1,1,6.163126868,7.708
23243,929.6955618,0.770390858,3.854085911,0.087382224,0.087382224,1,1,6.163126868,7.70817
823243,929.6955618,0.770390858,3.854085911,0.087382224,0.087382224,1,1,6.163126868,7.708
418,4.163684608,0.087382224,0.087382224,1,1,5.752843348,8.327369216,277.5642176,15.101213
367729,1073.063478,0.719105418,4.163684608,0.087382224,0.087382224,1,1,5.752843348,8.3273
367729,1073.063478,0.719105418,4.163684608,0.087382224,0.087382224,1,1,5.752843348,8.3273
2367729,1073.063478,0.719105418,4.163684608,0.087382224,0.087382224,1,1,5.752843348,8.327
,4.163684608,0.087382224,0.087382224,1,1,5.752843348,8.327369216,277.5642176,15.10121379
4.163684608,0.087382224,0.087382224,1,1,5.752843348,8.327369216,277.5642176,15.10121379,
105418,4.163684608,0.087382224,0.087382224,1,1,5.752843348,8.327369216,277.5642176,15.101
.2367729,1073.063478,0.719105418,4.163684608,0.087382224,0.087382224,1,1,5.752843348,8.32
,4.163684608,0.087382224,0.087382224,1,1,5.752843348,8.327369216,277.5642176,15.10121379
,4.163684608,0.087382224,0.087382224,1,1,5.752843348,8.327369216,277.5642176,15.10121379
163684608,0.087382224,0.087382224,1,1,5.752843348,8.327369216,277.5642176,15.10121379,29.
,0,1,0,1,1,12,888.2367729,1073.063478,0.719105418,4.163684608,0.087382224,0.087382224,1,1,5
5418,4.163684608,0.087382224,0.087382224,1,1,5.752843348,8.327369216,277.5642176,15.10121
9105418,4.163684608,0.087382224,0.087382224,1,1,5.752843348,8.327369216,277.5642176,15.1
367729,1073.063478,0.719105418,4.163684608,0.087382224,0.087382224,1,1,5.752843348,8.3273
367729,1073.063478,0.719105418,4.163684608,0.087382224,0.087382224,1,1,5.752843348,8.3273
.2367729,1073.063478,0.719105418,4.163684608,0.087382224,0.087382224,1,1,5.752843348,8.32
.2367729,1073.063478,0.719105418,4.163684608,0.087382224,0.087382224,1,1,5.752843348,8.32

.2367729,1073.063478,0.719105418,4.163684608,0.087382224,0.087382224,1,1,5.752843348,8.32
418,4.163684608,0.087382224,0.087382224,1,1,5.752843348,8.327369216,277.5642176,15.101213
0,1,0,1,1,12,888.2367729,1073.063478,0.719105418,4.163684608,0.087382224,0.087382224,1,1,5.
9105418,4.163684608,0.087382224,0.087382224,1,1,5.752843348,8.327369216,277.5642176,15.10
,0,1,0,1,1,12,888.2367729,1073.063478,0.719105418,4.163684608,0.087382224,0.087382224,1,1,5
,0,1,0,1,1,12,888.2367729,1073.063478,0.719105418,4.163684608,0.087382224,0.087382224,1,1,5
0,1,0,1,1,12,888.2367729,1073.063478,0.719105418,4.163684608,0.087382224,0.087382224,1,1,5.
105418,4.163684608,0.087382224,0.087382224,1,1,5.752843348,8.327369216,277.5642176,15.101
0,1,0,1,1,12,888.2367729,1073.063478,0.719105418,4.163684608,0.087382224,0.087382224,1,1,5.
5418,4.163684608,0.087382224,0.087382224,1,1,5.752843348,8.327369216,277.5642176,15.10121
2367729,1073.063478,0.719105418,4.163684608,0.087382224,0.087382224,1,1,5.752843348,8.327
.2367729,1073.063478,0.719105418,4.163684608,0.087382224,0.087382224,1,1,5.752843348,8.32
0,1,0,1,1,12,888.2367729,1073.063478,0.719105418,4.163684608,0.087382224,0.087382224,1,1,5.
5418,4.163684608,0.087382224,0.087382224,1,1,5.752843348,8.327369216,277.5642176,15.10121
87382224,0,1,0,4.137128055,4.026808167,351.5420099,14.47994819,28.18765717,6.205692082,1
5047608,1090.12904,0.689521342,0,0.087382224,0,1,0,4.137128055,4.026808167,351.5420099,14.
21342,0,0.087382224,0,1,0,4.137128055,4.026808167,351.5420099,14.47994819,28.18765717,6.2
47608,1090.12904,0.689521342,0,0.087382224,0,1,0,4.137128055,4.026808167,351.5420099,14.47
47608,1090.12904,0.689521342,0,0.087382224,0,1,0,4.137128055,4.026808167,351.5420099,14.47
47608,1090.12904,0.689521342,0,0.087382224,0,1,0,4.137128055,4.026808167,351.5420099,14.47
,0,1,0,1,1,9,879.5047608,1090.12904,0.689521342,0,0.087382224,0,1,0,4.137128055,4.026808167,
,0,1,0,1,1,9,879.5047608,1090.12904,0.689521342,0,0.087382224,0,1,0,4.137128055,4.026808167,
5047608,1090.12904,0.689521342,0,0.087382224,0,1,0,4.137128055,4.026808167,351.5420099,14.
7676,3,2,207,76,0,1,0,1,1,9,879.5047608,1090.12904,0.689521342,0,0.087382224,0,1,0,4.13712805
0,1,0,1,1,9,879.5047608,1090.12904,0.689521342,0,0.087382224,0,1,0,4.137128055,4.026808167,3
21342,0,0.087382224,0,1,0,4.137128055,4.026808167,351.5420099,14.47994819,28.18765717,6.2
047608,1090.12904,0.689521342,0,0.087382224,0,1,0,4.137128055,4.026808167,351.5420099,14.4
42,0,0.087382224,0,1,0,4.137128055,4.026808167,351.5420099,14.47994819,28.18765717,6.20569
0,1,0,1,1,9,879.5047608,1090.12904,0.689521342,0,0.087382224,0,1,0,4.137128055,4.026808167,3
21342,0,0.087382224,0,1,0,4.137128055,4.026808167,351.5420099,14.47994819,28.18765717,6.2
5047608,1090.12904,0.689521342,0,0.087382224,0,1,0,4.137128055,4.026808167,351.5420099,14.
5047608,1090.12904,0.689521342,0,0.087382224,0,1,0,4.137128055,4.026808167,351.5420099,14.
42,0,0.087382224,0,1,0,4.137128055,4.026808167,351.5420099,14.47994819,28.18765717,6.20569
0,1,0,1,1,9,879.5047608,1090.12904,0.689521342,0,0.087382224,0,1,0,4.137128055,4.026808167,3
5047608,1090.12904,0.689521342,0,0.087382224,0,1,0,4.137128055,4.026808167,351.5420099,14.
0.087382224,0,1,0,4.137128055,4.026808167,351.5420099,14.47994819,28.18765717,6.205692082
0.087382224,0,1,0,4.137128055,4.026808167,351.5420099,14.47994819,28.18765717,6.205692082
47608,1090.12904,0.689521342,0,0.087382224,0,1,0,4.137128055,4.026808167,351.5420099,14.47
47608,1090.12904,0.689521342,0,0.087382224,0,1,0,4.137128055,4.026808167,351.5420099,14.47
047608,1090.12904,0.689521342,0,0.087382224,0,1,0,4.137128055,4.026808167,351.5420099,14.4
,0.087382224,0,1,0,4.137128055,4.026808167,351.5420099,14.47994819,28.18765717,6.20569208
0.087382224,0,1,0,4.137128055,4.026808167,351.5420099,14.47994819,28.18765717,6.205692082
,0.087382224,0,1,0,4.137128055,4.026808167,351.5420099,14.47994819,28.18765717,6.20569208
,0,1,0,1,1,9,879.5047608,1090.12904,0.689521342,0,0.087382224,0,1,0,4.137128055,4.026808167,
,0.087382224,0,1,0,4.137128055,4.026808167,351.5420099,14.47994819,28.18765717,6.20569208
,0.087382224,0,1,0,4.137128055,4.026808167,351.5420099,14.47994819,28.18765717,6.20569208
38,4.167750163,0.087382224,0.087382224,1,1,5.722605504,8.335500326,288.8242349,15.0218394
1,0,0,1,1,9,875.0711037,1066.556794,0.715325688,4.167750163,0.087382224,0.087382224,1,1,5.7
711037,1066.556794,0.715325688,4.167750163,0.087382224,0.087382224,1,1,5.722605504,8.335
1,0,0,1,1,9,875.0711037,1066.556794,0.715325688,4.167750163,0.087382224,0.087382224,1,1,5.7

588,4.167750163,0.087382224,0.087382224,1,1,5.722605504,8.335500326,288.8242349,15.021839
25688,4.167750163,0.087382224,0.087382224,1,1,5.722605504,8.335500326,288.8242349,15.0218
1,0,0,1,1,9,875.0711037,1066.556794,0.715325688,4.167750163,0.087382224,0.087382224,1,1,5.72
0711037,1066.556794,0.715325688,4.167750163,0.087382224,0.087382224,1,1,5.722605504,8.335
588,4.167750163,0.087382224,0.087382224,1,1,5.722605504,8.335500326,288.8242349,15.021839
4.167750163,0.087382224,0.087382224,1,1,5.722605504,8.335500326,288.8242349,15.02183945,2
0711037,1066.556794,0.715325688,4.167750163,0.087382224,0.087382224,1,1,5.722605504,8.335
1,0,0,1,1,9,875.0711037,1066.556794,0.715325688,4.167750163,0.087382224,0.087382224,1,1,5.72
0711037,1066.556794,0.715325688,4.167750163,0.087382224,0.087382224,1,1,5.722605504,8.335
.167750163,0.087382224,0.087382224,1,1,5.722605504,8.335500326,288.8242349,15.02183945,29
11037,1066.556794,0.715325688,4.167750163,0.087382224,0.087382224,1,1,5.722605504,8.33550
11037,1066.556794,0.715325688,4.167750163,0.087382224,0.087382224,1,1,5.722605504,8.33550
4.167750163,0.087382224,0.087382224,1,1,5.722605504,8.335500326,288.8242349,15.02183945,2
.167750163,0.087382224,0.087382224,1,1,5.722605504,8.335500326,288.8242349,15.02183945,29
1,0,0,1,1,9,875.0711037,1066.556794,0.715325688,4.167750163,0.087382224,0.087382224,1,1,5.72
,1,0,0,1,1,9,875.0711037,1066.556794,0.715325688,4.167750163,0.087382224,0.087382224,1,1,5.7
0711037,1066.556794,0.715325688,4.167750163,0.087382224,0.087382224,1,1,5.722605504,8.335
4.167750163,0.087382224,0.087382224,1,1,5.722605504,8.335500326,288.8242349,15.02183945,2
.167750163,0.087382224,0.087382224,1,1,5.722605504,8.335500326,288.8242349,15.02183945,29
0711037,1066.556794,0.715325688,4.167750163,0.087382224,0.087382224,1,1,5.722605504,8.335
0711037,1066.556794,0.715325688,4.167750163,0.087382224,0.087382224,1,1,5.722605504,8.335
11037,1066.556794,0.715325688,4.167750163,0.087382224,0.087382224,1,1,5.722605504,8.33550
11037,1066.556794,0.715325688,4.167750163,0.087382224,0.087382224,1,1,5.722605504,8.33550
67750163,0.087382224,0.087382224,1,1,5.722605504,8.335500326,288.8242349,15.02183945,29.1
0711037,1066.556794,0.715325688,4.167750163,0.087382224,0.087382224,1,1,5.722605504,8.335
711037,1066.556794,0.715325688,4.167750163,0.087382224,0.087382224,1,1,5.722605504,8.3355
0711037,1066.556794,0.715325688,4.167750163,0.087382224,0.087382224,1,1,5.722605504,8.335
0711037,1066.556794,0.715325688,4.167750163,0.087382224,0.087382224,1,1,5.722605504,8.335
.15226782,0.087382224,0.087382224,1,1,6.193493999,10.30453564,266.0728929,14.70954825,30.
3488221,1129.61513,0.77418675,5.15226782,0.087382224,0.087382224,1,1,6.193493999,10.30453
.8488221,1129.61513,0.77418675,5.15226782,0.087382224,0.087382224,1,1,6.193493999,10.3045
1,1,1,1,1,10,892.8488221,1129.61513,0.77418675,5.15226782,0.087382224,0.087382224,1,1,6.1934
.8488221,1129.61513,0.77418675,5.15226782,0.087382224,0.087382224,1,1,6.193493999,10.3045
15226782,0.087382224,0.087382224,1,1,6.193493999,10.30453564,266.0728929,14.70954825,30.9
488221,1129.61513,0.77418675,5.15226782,0.087382224,0.087382224,1,1,6.193493999,10.304535
.15226782,0.087382224,0.087382224,1,1,6.193493999,10.30453564,266.0728929,14.70954825,30.
15226782,0.087382224,0.087382224,1,1,6.193493999,10.30453564,266.0728929,14.70954825,30.9
1,1,1,1,1,10,892.8488221,1129.61513,0.77418675,5.15226782,0.087382224,0.087382224,1,1,6.1934
1,1,1,1,1,10,892.8488221,1129.61513,0.77418675,5.15226782,0.087382224,0.087382224,1,1,6.1934
3488221,1129.61513,0.77418675,5.15226782,0.087382224,0.087382224,1,1,6.193493999,10.30453
.15226782,0.087382224,0.087382224,1,1,6.193493999,10.30453564,266.0728929,14.70954825,30.
15226782,0.087382224,0.087382224,1,1,6.193493999,10.30453564,266.0728929,14.70954825,30.9
.8488221,1129.61513,0.77418675,5.15226782,0.087382224,0.087382224,1,1,6.193493999,10.3045
.8488221,1129.61513,0.77418675,5.15226782,0.087382224,0.087382224,1,1,6.193493999,10.3045
3675,5.15226782,0.087382224,0.087382224,1,1,6.193493999,10.30453564,266.0728929,14.709548
488221,1129.61513,0.77418675,5.15226782,0.087382224,0.087382224,1,1,6.193493999,10.304535
226782,0.087382224,0.087382224,1,1,6.193493999,10.30453564,266.0728929,14.70954825,30.913
7418675,5.15226782,0.087382224,0.087382224,1,1,6.193493999,10.30453564,266.0728929,14.709
.15226782,0.087382224,0.087382224,1,1,6.193493999,10.30453564,266.0728929,14.70954825,30.
.8488221,1129.61513,0.77418675,5.15226782,0.087382224,0.087382224,1,1,6.193493999,10.3045

18675,5.15226782,0.087382224,0.087382224,1,1,6.193493999,10.30453564,266.0728929,14.7095
7418675,5.15226782,0.087382224,0.087382224,1,1,6.193493999,10.30453564,266.0728929,14.709
488221,1129.61513,0.77418675,5.15226782,0.087382224,0.087382224,1,1,6.193493999,10.304535
488221,1129.61513,0.77418675,5.15226782,0.087382224,0.087382224,1,1,6.193493999,10.304535
,1,1,1,1,1,10,892.8488221,1129.61513,0.77418675,5.15226782,0.087382224,0.087382224,1,1,6.193
.15226782,0.087382224,0.087382224,1,1,6.193493999,10.30453564,266.0728929,14.70954825,30.
,1,1,1,1,1,10,892.8488221,1129.61513,0.77418675,5.15226782,0.087382224,0.087382224,1,1,6.193
1,1,1,1,1,10,892.8488221,1129.61513,0.77418675,5.15226782,0.087382224,0.087382224,1,1,6.193
.8488221,1129.61513,0.77418675,5.15226782,0.087382224,0.087382224,1,1,6.193493999,10.3045
2.8488221,1129.61513,0.77418675,5.15226782,0.087382224,0.087382224,1,1,6.193493999,10.304
0,0.087382224,0,1,0,3.411950086,0,544.7688991,14.21645869,28.62678212,4.549266782,9.54226
348,0,0.087382224,0,1,0,3.411950086,0,544.7688991,14.21645869,28.62678212,4.549266782,9.5
0,0.087382224,0,1,0,3.411950086,0,544.7688991,14.21645869,28.62678212,4.549266782,9.54226
58348,0,0.087382224,0,1,0,3.411950086,0,544.7688991,14.21645869,28.62678212,4.549266782,9
284267,1084.406933,0.568658348,0,0.087382224,0,1,0,3.411950086,0,544.7688991,14.21645869,
284267,1084.406933,0.568658348,0,0.087382224,0,1,0,3.411950086,0,544.7688991,14.21645869,
,0,0.087382224,0,1,0,3.411950086,0,544.7688991,14.21645869,28.62678212,4.549266782,9.5422
0,0.087382224,0,1,0,3.411950086,0,544.7688991,14.21645869,28.62678212,4.549266782,9.54226
.0284267,1084.406933,0.568658348,0,0.087382224,0,1,0,3.411950086,0,544.7688991,14.2164586
58348,0,0.087382224,0,1,0,3.411950086,0,544.7688991,14.21645869,28.62678212,4.549266782,9
,0,1,0,1,1,12,875.0284267,1084.406933,0.568658348,0,0.087382224,0,1,0,3.411950086,0,544.7688
,0,0.087382224,0,1,0,3.411950086,0,544.7688991,14.21645869,28.62678212,4.549266782,9.5422
.087382224,0,1,0,3.411950086,0,544.7688991,14.21645869,28.62678212,4.549266782,9.5422607
,0,1,0,1,1,12,875.0284267,1084.406933,0.568658348,0,0.087382224,0,1,0,3.411950086,0,544.7688
,0,0.087382224,0,1,0,3.411950086,0,544.7688991,14.21645869,28.62678212,4.549266782,9.5422
58348,0,0.087382224,0,1,0,3.411950086,0,544.7688991,14.21645869,28.62678212,4.549266782,9
,0,1,0,1,1,12,875.0284267,1084.406933,0.568658348,0,0.087382224,0,1,0,3.411950086,0,544.7688
284267,1084.406933,0.568658348,0,0.087382224,0,1,0,3.411950086,0,544.7688991,14.21645869,
284267,1084.406933,0.568658348,0,0.087382224,0,1,0,3.411950086,0,544.7688991,14.21645869,
84267,1084.406933,0.568658348,0,0.087382224,0,1,0,3.411950086,0,544.7688991,14.21645869,2
,0,1,0,1,1,12,875.0284267,1084.406933,0.568658348,0,0.087382224,0,1,0,3.411950086,0,544.7688
,0,1,0,1,1,12,875.0284267,1084.406933,0.568658348,0,0.087382224,0,1,0,3.411950086,0,544.7688
348,0,0.087382224,0,1,0,3.411950086,0,544.7688991,14.21645869,28.62678212,4.549266782,9.5
,0,1,0,1,1,12,875.0284267,1084.406933,0.568658348,0,0.087382224,0,1,0,3.411950086,0,544.7688
84267,1084.406933,0.568658348,0,0.087382224,0,1,0,3.411950086,0,544.7688991,14.21645869,2
348,0,0.087382224,0,1,0,3.411950086,0,544.7688991,14.21645869,28.62678212,4.549266782,9.5
284267,1084.406933,0.568658348,0,0.087382224,0,1,0,3.411950086,0,544.7688991,14.21645869,
.0284267,1084.406933,0.568658348,0,0.087382224,0,1,0,3.411950086,0,544.7688991,14.2164586
58348,0,0.087382224,0,1,0,3.411950086,0,544.7688991,14.21645869,28.62678212,4.549266782,9
348,0,0.087382224,0,1,0,3.411950086,0,544.7688991,14.21645869,28.62678212,4.549266782,9.5
348,0,0.087382224,0,1,0,3.411950086,0,544.7688991,14.21645869,28.62678212,4.549266782,9.5
5.098517308,0.087382224,0.087382224,1,1,5.885925733,10.19703462,311.2187622,15.45055505,
.7902768,1152.711043,0.735740717,5.098517308,0.087382224,0.087382224,1,1,5.885925733,10.1
.7902768,1152.711043,0.735740717,5.098517308,0.087382224,0.087382224,1,1,5.885925733,10.1
,5.098517308,0.087382224,0.087382224,1,1,5.885925733,10.19703462,311.2187622,15.45055505
,5.098517308,0.087382224,0.087382224,1,1,5.885925733,10.19703462,311.2187622,15.45055505
98517308,0.087382224,0.087382224,1,1,5.885925733,10.19703462,311.2187622,15.45055505,35.
,0,0,0,1,1,10,892.7902768,1152.711043,0.735740717,5.098517308,0.087382224,0.087382224,1,1,5
,5.098517308,0.087382224,0.087382224,1,1,5.885925733,10.19703462,311.2187622,15.45055505
,0,0,0,1,1,10,892.7902768,1152.711043,0.735740717,5.098517308,0.087382224,0.087382224,1,1,5

902768,1152.711043,0.735740717,5.098517308,0.087382224,0.087382224,1,1,5.885925733,10.197
0717,5.098517308,0.087382224,0.087382224,1,1,5.885925733,10.19703462,311.2187622,15.45055
.7902768,1152.711043,0.735740717,5.098517308,0.087382224,0.087382224,1,1,5.885925733,10.1
902768,1152.711043,0.735740717,5.098517308,0.087382224,0.087382224,1,1,5.885925733,10.197
7902768,1152.711043,0.735740717,5.098517308,0.087382224,0.087382224,1,1,5.885925733,10.1
0.735740717,5.098517308,0.087382224,0.087382224,1,1,5.885925733,10.19703462,311.2187622,
0717,5.098517308,0.087382224,0.087382224,1,1,5.885925733,10.19703462,311.2187622,15.45055
5740717,5.098517308,0.087382224,0.087382224,1,1,5.885925733,10.19703462,311.2187622,15.4
902768,1152.711043,0.735740717,5.098517308,0.087382224,0.087382224,1,1,5.885925733,10.197
0717,5.098517308,0.087382224,0.087382224,1,1,5.885925733,10.19703462,311.2187622,15.45055
7902768,1152.711043,0.735740717,5.098517308,0.087382224,0.087382224,1,1,5.885925733,10.19
.7902768,1152.711043,0.735740717,5.098517308,0.087382224,0.087382224,1,1,5.885925733,10.1
902768,1152.711043,0.735740717,5.098517308,0.087382224,0.087382224,1,1,5.885925733,10.197
.7902768,1152.711043,0.735740717,5.098517308,0.087382224,0.087382224,1,1,5.885925733,10.1
.7902768,1152.711043,0.735740717,5.098517308,0.087382224,0.087382224,1,1,5.885925733,10.1
717,5.098517308,0.087382224,0.087382224,1,1,5.885925733,10.19703462,311.2187622,15.450555
.7902768,1152.711043,0.735740717,5.098517308,0.087382224,0.087382224,1,1,5.885925733,10.1
902768,1152.711043,0.735740717,5.098517308,0.087382224,0.087382224,1,1,5.885925733,10.197
7902768,1152.711043,0.735740717,5.098517308,0.087382224,0.087382224,1,1,5.885925733,10.19
7,5.098517308,0.087382224,0.087382224,1,1,5.885925733,10.19703462,311.2187622,15.45055505
707363,599.2627876,1.035745602,4.831730494,0.087382224,0.087382224,1,1,8.285964815,19.326
.0707363,599.2627876,1.035745602,4.831730494,0.087382224,0.087382224,1,1,8.285964815,19.3
.0707363,599.2627876,1.035745602,4.831730494,0.087382224,0.087382224,1,1,8.285964815,19.3
.0707363,599.2627876,1.035745602,4.831730494,0.087382224,0.087382224,1,1,8.285964815,19.3
1211,3,2,207,73,0,1,1,1,1,11,908.0707363,599.2627876,1.035745602,4.831730494,0.087382224,0.0
35745602,4.831730494,0.087382224,0.087382224,1,1,8.285964815,19.32692198,95.12704748,20.
2,4.831730494,0.087382224,0.087382224,1,1,8.285964815,19.32692198,95.12704748,20.71491204
5745602,4.831730494,0.087382224,0.087382224,1,1,8.285964815,19.32692198,95.12704748,20.7
5602,4.831730494,0.087382224,0.087382224,1,1,8.285964815,19.32692198,95.12704748,20.71491
0707363,599.2627876,1.035745602,4.831730494,0.087382224,0.087382224,1,1,8.285964815,19.32
0707363,599.2627876,1.035745602,4.831730494,0.087382224,0.087382224,1,1,8.285964815,19.32
.0707363,599.2627876,1.035745602,4.831730494,0.087382224,0.087382224,1,1,8.285964815,19.3
.0707363,599.2627876,1.035745602,4.831730494,0.087382224,0.087382224,1,1,8.285964815,19.3
0,1,1,1,1,11,908.0707363,599.2627876,1.035745602,4.831730494,0.087382224,0.087382224,1,1,8.
.0707363,599.2627876,1.035745602,4.831730494,0.087382224,0.087382224,1,1,8.285964815,19.3
4.831730494,0.087382224,0.087382224,1,1,8.285964815,19.32692198,95.12704748,20.71491204,
707363,599.2627876,1.035745602,4.831730494,0.087382224,0.087382224,1,1,8.285964815,19.326
707363,599.2627876,1.035745602,4.831730494,0.087382224,0.087382224,1,1,8.285964815,19.326
0707363,599.2627876,1.035745602,4.831730494,0.087382224,0.087382224,1,1,8.285964815,19.32
2,4.831730494,0.087382224,0.087382224,1,1,8.285964815,19.32692198,95.12704748,20.71491204
602,4.831730494,0.087382224,0.087382224,1,1,8.285964815,19.32692198,95.12704748,20.714912
745602,4.831730494,0.087382224,0.087382224,1,1,8.285964815,19.32692198,95.12704748,20.714
,0,1,1,1,1,11,908.0707363,599.2627876,1.035745602,4.831730494,0.087382224,0.087382224,1,1,8
2,4.831730494,0.087382224,0.087382224,1,1,8.285964815,19.32692198,95.12704748,20.71491204
745602,4.831730494,0.087382224,0.087382224,1,1,8.285964815,19.32692198,95.12704748,20.714
831730494,0.087382224,0.087382224,1,1,8.285964815,19.32692198,95.12704748,20.71491204,19.
.0707363,599.2627876,1.035745602,4.831730494,0.087382224,0.087382224,1,1,8.285964815,19.3
8.0707363,599.2627876,1.035745602,4.831730494,0.087382224,0.087382224,1,1,8.285964815,19.3
5745602,4.831730494,0.087382224,0.087382224,1,1,8.285964815,19.32692198,95.12704748,20.7
707363,599.2627876,1.035745602,4.831730494,0.087382224,0.087382224,1,1,8.285964815,19.326

348,0,0.087382224,0,1,0,3.411950086,0,544.7688991,14.21645869,28.62678212,4.549266782,9.542
0,0.087382224,0,1,0,3.411950086,0,544.7688991,14.21645869,28.62678212,4.549266782,9.54226
658348,0,0.087382224,0,1,0,3.411950086,0,544.7688991,14.21645869,28.62678212,4.549266782,
0284267,1084.406933,0.568658348,0,0.087382224,0,1,0,3.411950086,0,544.7688991,14.21645869,
0284267,1084.406933,0.568658348,0,0.087382224,0,1,0,3.411950086,0,544.7688991,14.21645869,
348,0,0.087382224,0,1,0,3.411950086,0,544.7688991,14.21645869,28.62678212,4.549266782,9.542
1,0,0,1,1,9,875.0284267,1084.406933,0.568658348,0,0.087382224,0,1,0,3.411950086,0,544.768899
0284267,1084.406933,0.568658348,0,0.087382224,0,1,0,3.411950086,0,544.7688991,14.21645869,
,0.087382224,0,1,0,3.411950086,0,544.7688991,14.21645869,28.62678212,4.549266782,9.542260
34267,1084.406933,0.568658348,0,0.087382224,0,1,0,3.411950086,0,544.7688991,14.21645869,28
34267,1084.406933,0.568658348,0,0.087382224,0,1,0,3.411950086,0,544.7688991,14.21645869,28
284267,1084.406933,0.568658348,0,0.087382224,0,1,0,3.411950086,0,544.7688991,14.21645869,2
0,0.087382224,0,1,0,3.411950086,0,544.7688991,14.21645869,28.62678212,4.549266782,9.54226
48,0,0.087382224,0,1,0,3.411950086,0,544.7688991,14.21645869,28.62678212,4.549266782,9.542
1,0,0,1,1,9,875.0284267,1084.406933,0.568658348,0,0.087382224,0,1,0,3.411950086,0,544.768899
,1,0,0,1,1,9,875.0284267,1084.406933,0.568658348,0,0.087382224,0,1,0,3.411950086,0,544.76889
0,0.087382224,0,1,0,3.411950086,0,544.7688991,14.21645869,28.62678212,4.549266782,9.54226
1,0,0,1,1,9,875.0284267,1084.406933,0.568658348,0,0.087382224,0,1,0,3.411950086,0,544.768899
087382224,0,1,0,3.411950086,0,544.7688991,14.21645869,28.62678212,4.549266782,9.54226070
0284267,1084.406933,0.568658348,0,0.087382224,0,1,0,3.411950086,0,544.7688991,14.21645869,
0284267,1084.406933,0.568658348,0,0.087382224,0,1,0,3.411950086,0,544.7688991,14.21645869,
34267,1084.406933,0.568658348,0,0.087382224,0,1,0,3.411950086,0,544.7688991,14.21645869,28
34267,1084.406933,0.568658348,0,0.087382224,0,1,0,3.411950086,0,544.7688991,14.21645869,28
658348,0,0.087382224,0,1,0,3.411950086,0,544.7688991,14.21645869,28.62678212,4.549266782,
284267,1084.406933,0.568658348,0,0.087382224,0,1,0,3.411950086,0,544.7688991,14.21645869,2
0284267,1084.406933,0.568658348,0,0.087382224,0,1,0,3.411950086,0,544.7688991,14.21645869,
0284267,1084.406933,0.568658348,0,0.087382224,0,1,0,3.411950086,0,544.7688991,14.21645869,
58348,0,0.087382224,0,1,0,3.411950086,0,544.7688991,14.21645869,28.62678212,4.549266782,9.
1,0,0,1,1,9,875.0284267,1084.406933,0.568658348,0,0.087382224,0,1,0,3.411950086,0,544.768899
284267,1084.406933,0.568658348,0,0.087382224,0,1,0,3.411950086,0,544.7688991,14.21645869,
284267,1084.406933,0.568658348,0,0.087382224,0,1,0,3.411950086,0,544.7688991,14.21645869,2
.098517308,0.087382224,0.087382224,1,1,5.885925733,10.19703462,311.2187622,15.45055505,3
02768,1152.711043,0.735740717,5.098517308,0.087382224,0.087382224,1,1,5.885925733,10.1970
02768,1152.711043,0.735740717,5.098517308,0.087382224,0.087382224,1,1,5.885925733,10.1970
5.098517308,0.087382224,0.087382224,1,1,5.885925733,10.19703462,311.2187622,15.45055505,
.098517308,0.087382224,0.087382224,1,1,5.885925733,10.19703462,311.2187622,15.45055505,3
1,1,0,1,1,9,892.7902768,1152.711043,0.735740717,5.098517308,0.087382224,0.087382224,1,1,5.8
,1,1,0,1,1,9,892.7902768,1152.711043,0.735740717,5.098517308,0.087382224,0.087382224,1,1,5.
7902768,1152.711043,0.735740717,5.098517308,0.087382224,0.087382224,1,1,5.885925733,10.19
717,5.098517308,0.087382224,0.087382224,1,1,5.885925733,10.19703462,311.2187622,15.450555
7,5.098517308,0.087382224,0.087382224,1,1,5.885925733,10.19703462,311.2187622,15.45055505
717,5.098517308,0.087382224,0.087382224,1,1,5.885925733,10.19703462,311.2187622,15.450555
7902768,1152.711043,0.735740717,5.098517308,0.087382224,0.087382224,1,1,5.885925733,10.19
02768,1152.711043,0.735740717,5.098517308,0.087382224,0.087382224,1,1,5.885925733,10.1970
02768,1152.711043,0.735740717,5.098517308,0.087382224,0.087382224,1,1,5.885925733,10.1970
9395,3,2,207,73,1,1,0,1,1,9,892.7902768,1152.711043,0.735740717,5.098517308,0.087382224,0.08
5.098517308,0.087382224,0.087382224,1,1,5.885925733,10.19703462,311.2187622,15.45055505,
7902768,1152.711043,0.735740717,5.098517308,0.087382224,0.087382224,1,1,5.885925733,10.19
7902768,1152.711043,0.735740717,5.098517308,0.087382224,0.087382224,1,1,5.885925733,10.19
17,5.098517308,0.087382224,0.087382224,1,1,5.885925733,10.19703462,311.2187622,15.4505550

5740717,5.098517308,0.087382224,0.087382224,1,1,5.885925733,10.19703462,311.2187622,15.4
902768,1152.711043,0.735740717,5.098517308,0.087382224,0.087382224,1,1,5.885925733,10.19
,1,1,0,1,1,9,892.7902768,1152.711043,0.735740717,5.098517308,0.087382224,0.087382224,1,1,5.
5.098517308,0.087382224,0.087382224,1,1,5.885925733,10.19703462,311.2187622,15.45055505,
,1,1,0,1,1,9,892.7902768,1152.711043,0.735740717,5.098517308,0.087382224,0.087382224,1,1,5.
1,1,0,1,1,9,892.7902768,1152.711043,0.735740717,5.098517308,0.087382224,0.087382224,1,1,5.8
7902768,1152.711043,0.735740717,5.098517308,0.087382224,0.087382224,1,1,5.885925733,10.19
7902768,1152.711043,0.735740717,5.098517308,0.087382224,0.087382224,1,1,5.885925733,10.1
5.098517308,0.087382224,0.087382224,1,1,5.885925733,10.19703462,311.2187622,15.45055505,
902768,1152.711043,0.735740717,5.098517308,0.087382224,0.087382224,1,1,5.885925733,10.197
7902768,1152.711043,0.735740717,5.098517308,0.087382224,0.087382224,1,1,5.885925733,10.19
1,1,0,1,1,9,892.7902768,1152.711043,0.735740717,5.098517308,0.087382224,0.087382224,1,1,5.8
7902768,1152.711043,0.735740717,5.098517308,0.087382224,0.087382224,1,1,5.885925733,10.19
4.430494159,0.087382224,0.087382224,1,1,6.888452469,8.860988317,196.7984776,16.36007461,
0,1,0,1,1,10,891.2532305,915.9845891,0.861056559,4.430494159,0.087382224,0.087382224,1,1,6.
1056559,4.430494159,0.087382224,0.087382224,1,1,6.888452469,8.860988317,196.7984776,16.3
2532305,915.9845891,0.861056559,4.430494159,0.087382224,0.087382224,1,1,6.888452469,8.860
9,4.430494159,0.087382224,0.087382224,1,1,6.888452469,8.860988317,196.7984776,16.36007461
4.430494159,0.087382224,0.087382224,1,1,6.888452469,8.860988317,196.7984776,16.36007461,
.2532305,915.9845891,0.861056559,4.430494159,0.087382224,0.087382224,1,1,6.888452469,8.86
.2532305,915.9845891,0.861056559,4.430494159,0.087382224,0.087382224,1,1,6.888452469,8.86
056559,4.430494159,0.087382224,0.087382224,1,1,6.888452469,8.860988317,196.7984776,16.360
532305,915.9845891,0.861056559,4.430494159,0.087382224,0.087382224,1,1,6.888452469,8.8609
430494159,0.087382224,0.087382224,1,1,6.888452469,8.860988317,196.7984776,16.36007461,17.
9395,3,2,207,68,0,1,0,1,1,10,891.2532305,915.9845891,0.861056559,4.430494159,0.087382224,0.0
9,4.430494159,0.087382224,0.087382224,1,1,6.888452469,8.860988317,196.7984776,16.36007461
.2532305,915.9845891,0.861056559,4.430494159,0.087382224,0.087382224,1,1,6.888452469,8.86
1056559,4.430494159,0.087382224,0.087382224,1,1,6.888452469,8.860988317,196.7984776,16.3
0,1,0,1,1,10,891.2532305,915.9845891,0.861056559,4.430494159,0.087382224,0.087382224,1,1,6.
532305,915.9845891,0.861056559,4.430494159,0.087382224,0.087382224,1,1,6.888452469,8.8609
,0,1,0,1,1,10,891.2532305,915.9845891,0.861056559,4.430494159,0.087382224,0.087382224,1,1,6
,0,1,0,1,1,10,891.2532305,915.9845891,0.861056559,4.430494159,0.087382224,0.087382224,1,1,6
,0,1,0,1,1,10,891.2532305,915.9845891,0.861056559,4.430494159,0.087382224,0.087382224,1,1,6
0,1,0,1,1,10,891.2532305,915.9845891,0.861056559,4.430494159,0.087382224,0.087382224,1,1,6.
056559,4.430494159,0.087382224,0.087382224,1,1,6.888452469,8.860988317,196.7984776,16.360
1.2532305,915.9845891,0.861056559,4.430494159,0.087382224,0.087382224,1,1,6.888452469,8.8
5559,4.430494159,0.087382224,0.087382224,1,1,6.888452469,8.860988317,196.7984776,16.36007
2532305,915.9845891,0.861056559,4.430494159,0.087382224,0.087382224,1,1,6.888452469,8.860
.2532305,915.9845891,0.861056559,4.430494159,0.087382224,0.087382224,1,1,6.888452469,8.86
0,1,0,1,1,10,891.2532305,915.9845891,0.861056559,4.430494159,0.087382224,0.087382224,1,1,6.
.2532305,915.9845891,0.861056559,4.430494159,0.087382224,0.087382224,1,1,6.888452469,8.86
4.430494159,0.087382224,0.087382224,1,1,6.888452469,8.860988317,196.7984776,16.36007461,
532305,915.9845891,0.861056559,4.430494159,0.087382224,0.087382224,1,1,6.888452469,8.8609
9,4.430494159,0.087382224,0.087382224,1,1,6.888452469,8.860988317,196.7984776,16.36007461
,0.087382224,0,1,0,5.835945816,0,468.113051,17.50783745,52.11747468,8.753918724,26.058737
.7001648,1467.00565,0.972657636,0,0.087382224,0,1,0,5.835945816,0,468.113051,17.50783745,5
,1,1,1,0,1,11,900.7001648,1467.00565,0.972657636,0,0.087382224,0,1,0,5.835945816,0,468.11305
1,1,1,0,1,11,900.7001648,1467.00565,0.972657636,0,0.087382224,0,1,0,5.835945816,0,468.113051
001648,1467.00565,0.972657636,0,0.087382224,0,1,0,5.835945816,0,468.113051,17.50783745,52.
,0.087382224,0,1,0,5.835945816,0,468.113051,17.50783745,52.11747468,8.753918724,26.058737

57636,0,0.087382224,0,1,0,5.835945816,0,468.113051,17.50783745,52.11747468,8.753918724,26.0
.7001648,1467.00565,0.972657636,0,0.087382224,0,1,0,5.835945816,0,468.113051,17.50783745,5
7001648,1467.00565,0.972657636,0,0.087382224,0,1,0,5.835945816,0,468.113051,17.50783745,52
001648,1467.00565,0.972657636,0,0.087382224,0,1,0,5.835945816,0,468.113051,17.50783745,52.1
001648,1467.00565,0.972657636,0,0.087382224,0,1,0,5.835945816,0,468.113051,17.50783745,52.1
57636,0,0.087382224,0,1,0,5.835945816,0,468.113051,17.50783745,52.11747468,8.753918724,26.0
0,0.087382224,0,1,0,5.835945816,0,468.113051,17.50783745,52.11747468,8.753918724,26.058737
001648,1467.00565,0.972657636,0,0.087382224,0,1,0,5.835945816,0,468.113051,17.50783745,52.1
0,0.087382224,0,1,0,5.835945816,0,468.113051,17.50783745,52.11747468,8.753918724,26.058737
0,0.087382224,0,1,0,5.835945816,0,468.113051,17.50783745,52.11747468,8.753918724,26.058737
001648,1467.00565,0.972657636,0,0.087382224,0,1,0,5.835945816,0,468.113051,17.50783745,52.1
.7001648,1467.00565,0.972657636,0,0.087382224,0,1,0,5.835945816,0,468.113051,17.50783745,5
001648,1467.00565,0.972657636,0,0.087382224,0,1,0,5.835945816,0,468.113051,17.50783745,52.1
,0.087382224,0,1,0,5.835945816,0,468.113051,17.50783745,52.11747468,8.753918724,26.0587373
087382224,0,1,0,5.835945816,0,468.113051,17.50783745,52.11747468,8.753918724,26.05873734,
00.7001648,1467.00565,0.972657636,0,0.087382224,0,1,0,5.835945816,0,468.113051,17.50783745
0,0.087382224,0,1,0,5.835945816,0,468.113051,17.50783745,52.11747468,8.753918724,26.058737
001648,1467.00565,0.972657636,0,0.087382224,0,1,0,5.835945816,0,468.113051,17.50783745,52.1
.7001648,1467.00565,0.972657636,0,0.087382224,0,1,0,5.835945816,0,468.113051,17.50783745,5
,0.087382224,0,1,0,5.835945816,0,468.113051,17.50783745,52.11747468,8.753918724,26.0587373
7001648,1467.00565,0.972657636,0,0.087382224,0,1,0,5.835945816,0,468.113051,17.50783745,52
0,0.087382224,0,1,0,5.835945816,0,468.113051,17.50783745,52.11747468,8.753918724,26.058737
0.7001648,1467.00565,0.972657636,0,0.087382224,0,1,0,5.835945816,0,468.113051,17.50783745,5
0,0.087382224,0,1,0,5.835945816,0,468.113051,17.50783745,52.11747468,8.753918724,26.058737
5.034992018,0.087382224,0.087382224,1,1,6.340471293,10.06998404,229.8802174,15.05861932,2
3043179,1138.335613,0.792558912,5.034992018,0.087382224,0.087382224,1,1,6.340471293,10.06
3043179,1138.335613,0.792558912,5.034992018,0.087382224,0.087382224,1,1,6.340471293,10.06
3043179,1138.335613,0.792558912,5.034992018,0.087382224,0.087382224,1,1,6.340471293,10.06
558912,5.034992018,0.087382224,0.087382224,1,1,6.340471293,10.06998404,229.8802174,15.058
8912,5.034992018,0.087382224,0.087382224,1,1,6.340471293,10.06998404,229.8802174,15.05861
2324,3,2,207,76,0,1,0,1,1,10,887.3043179,1138.335613,0.792558912,5.034992018,0.087382224,0.0
.3043179,1138.335613,0.792558912,5.034992018,0.087382224,0.087382224,1,1,6.340471293,10.0
5.034992018,0.087382224,0.087382224,1,1,6.340471293,10.06998404,229.8802174,15.05861932,2
912,5.034992018,0.087382224,0.087382224,1,1,6.340471293,10.06998404,229.8802174,15.058619
0,1,0,1,1,10,887.3043179,1138.335613,0.792558912,5.034992018,0.087382224,0.087382224,1,1,6.3
2,5.034992018,0.087382224,0.087382224,1,1,6.340471293,10.06998404,229.8802174,15.05861932
3043179,1138.335613,0.792558912,5.034992018,0.087382224,0.087382224,1,1,6.340471293,10.06
.3043179,1138.335613,0.792558912,5.034992018,0.087382224,0.087382224,1,1,6.340471293,10.0
5.034992018,0.087382224,0.087382224,1,1,6.340471293,10.06998404,229.8802174,15.05861932,2
0,1,0,1,1,10,887.3043179,1138.335613,0.792558912,5.034992018,0.087382224,0.087382224,1,1,6.3
043179,1138.335613,0.792558912,5.034992018,0.087382224,0.087382224,1,1,6.340471293,10.069
.3043179,1138.335613,0.792558912,5.034992018,0.087382224,0.087382224,1,1,6.340471293,10.0
,0,1,0,1,1,10,887.3043179,1138.335613,0.792558912,5.034992018,0.087382224,0.087382224,1,1,6
8912,5.034992018,0.087382224,0.087382224,1,1,6.340471293,10.06998404,229.8802174,15.05861
58912,5.034992018,0.087382224,0.087382224,1,1,6.340471293,10.06998404,229.8802174,15.0586
3043179,1138.335613,0.792558912,5.034992018,0.087382224,0.087382224,1,1,6.340471293,10.06
2,5.034992018,0.087382224,0.087382224,1,1,6.340471293,10.06998404,229.8802174,15.05861932
2558912,5.034992018,0.087382224,0.087382224,1,1,6.340471293,10.06998404,229.8802174,15.05
5.034992018,0.087382224,0.087382224,1,1,6.340471293,10.06998404,229.8802174,15.05861932,2
0,1,0,1,1,10,887.3043179,1138.335613,0.792558912,5.034992018,0.087382224,0.087382224,1,1,6.3

3043179,1138.335613,0.792558912,5.034992018,0.087382224,0.087382224,1,1,6.340471293,10.0
558912,5.034992018,0.087382224,0.087382224,1,1,6.340471293,10.06998404,229.8802174,15.05
,0,1,0,1,1,10,887.3043179,1138.335613,0.792558912,5.034992018,0.087382224,0.087382224,1,1,6
0605,3,2,207,79,1,1,0,1,1,11,1156.426087,924.9865309,1.875889181,3.30519943,0.087382224,0.08
,1,1,0,1,1,11,1156.426087,924.9865309,1.875889181,3.30519943,0.087382224,0.087382224,1,1,9.
1,3.30519943,0.087382224,0.087382224,1,1,9.379445904,16.52599715,42.28989021,22.51067017,
181,3.30519943,0.087382224,0.087382224,1,1,9.379445904,16.52599715,42.28989021,22.5106701
,1,1,0,1,1,11,1156.426087,924.9865309,1.875889181,3.30519943,0.087382224,0.087382224,1,1,9.
5889181,3.30519943,0.087382224,0.087382224,1,1,9.379445904,16.52599715,42.28989021,22.51
389181,3.30519943,0.087382224,0.087382224,1,1,9.379445904,16.52599715,42.28989021,22.5106
.426087,924.9865309,1.875889181,3.30519943,0.087382224,0.087382224,1,1,9.379445904,16.525
6.426087,924.9865309,1.875889181,3.30519943,0.087382224,0.087382224,1,1,9.379445904,16.52
30519943,0.087382224,0.087382224,1,1,9.379445904,16.52599715,42.28989021,22.51067017,19.8
1,1,0,1,1,11,1156.426087,924.9865309,1.875889181,3.30519943,0.087382224,0.087382224,1,1,9.3
,1,1,0,1,1,11,1156.426087,924.9865309,1.875889181,3.30519943,0.087382224,0.087382224,1,1,9.
,1,1,0,1,1,11,1156.426087,924.9865309,1.875889181,3.30519943,0.087382224,0.087382224,1,1,9.
6.426087,924.9865309,1.875889181,3.30519943,0.087382224,0.087382224,1,1,9.379445904,16.52
9181,3.30519943,0.087382224,0.087382224,1,1,9.379445904,16.52599715,42.28989021,22.510670
.426087,924.9865309,1.875889181,3.30519943,0.087382224,0.087382224,1,1,9.379445904,16.525
6.426087,924.9865309,1.875889181,3.30519943,0.087382224,0.087382224,1,1,9.379445904,16.52
1,3.30519943,0.087382224,0.087382224,1,1,9.379445904,16.52599715,42.28989021,22.51067017,
389181,3.30519943,0.087382224,0.087382224,1,1,9.379445904,16.52599715,42.28989021,22.5106
30519943,0.087382224,0.087382224,1,1,9.379445904,16.52599715,42.28989021,22.51067017,19.8
,1,1,0,1,1,11,1156.426087,924.9865309,1.875889181,3.30519943,0.087382224,0.087382224,1,1,9.
6.426087,924.9865309,1.875889181,3.30519943,0.087382224,0.087382224,1,1,9.379445904,16.52
1,1,0,1,1,11,1156.426087,924.9865309,1.875889181,3.30519943,0.087382224,0.087382224,1,1,9.3
6.426087,924.9865309,1.875889181,3.30519943,0.087382224,0.087382224,1,1,9.379445904,16.52
.426087,924.9865309,1.875889181,3.30519943,0.087382224,0.087382224,1,1,9.379445904,16.525
6.426087,924.9865309,1.875889181,3.30519943,0.087382224,0.087382224,1,1,9.379445904,16.52
426087,924.9865309,1.875889181,3.30519943,0.087382224,0.087382224,1,1,9.379445904,16.5259
426087,924.9865309,1.875889181,3.30519943,0.087382224,0.087382224,1,1,9.379445904,16.5259
9395,3,2,207,81,0,1,1,1,1,9,1188.075336,883.5809344,2.886997997,3.379547207,0.087382224,0.08
75336,883.5809344,2.886997997,3.379547207,0.087382224,0.087382224,1,1,9.623326657,16.8977
.075336,883.5809344,2.886997997,3.379547207,0.087382224,0.087382224,1,1,9.623326657,16.89
.075336,883.5809344,2.886997997,3.379547207,0.087382224,0.087382224,1,1,9.623326657,16.89
.379547207,0.087382224,0.087382224,1,1,9.623326657,16.89773604,83.56300311,24.05831664,1
075336,883.5809344,2.886997997,3.379547207,0.087382224,0.087382224,1,1,9.623326657,16.897
38.075336,883.5809344,2.886997997,3.379547207,0.087382224,0.087382224,1,1,9.623326657,16.
3.379547207,0.087382224,0.087382224,1,1,9.623326657,16.89773604,83.56300311,24.05831664,
3.379547207,0.087382224,0.087382224,1,1,9.623326657,16.89773604,83.56300311,24.05831664,
.379547207,0.087382224,0.087382224,1,1,9.623326657,16.89773604,83.56300311,24.05831664,1
075336,883.5809344,2.886997997,3.379547207,0.087382224,0.087382224,1,1,9.623326657,16.897
,0,1,1,1,1,9,1188.075336,883.5809344,2.886997997,3.379547207,0.087382224,0.087382224,1,1,9.
38.075336,883.5809344,2.886997997,3.379547207,0.087382224,0.087382224,1,1,9.623326657,16.
3.379547207,0.087382224,0.087382224,1,1,9.623326657,16.89773604,83.56300311,24.05831664,
.379547207,0.087382224,0.087382224,1,1,9.623326657,16.89773604,83.56300311,24.05831664,1
997997,3.379547207,0.087382224,0.087382224,1,1,9.623326657,16.89773604,83.56300311,24.05
075336,883.5809344,2.886997997,3.379547207,0.087382224,0.087382224,1,1,9.623326657,16.897
,0,1,1,1,1,9,1188.075336,883.5809344,2.886997997,3.379547207,0.087382224,0.087382224,1,1,9.
075336,883.5809344,2.886997997,3.379547207,0.087382224,0.087382224,1,1,9.623326657,16.89

97,3.379547207,0.087382224,0.087382224,1,1,9.623326657,16.89773604,83.56300311,24.0583166
,0,1,1,1,1,9,1188.075336,883.5809344,2.886997997,3.379547207,0.087382224,0.087382224,1,1,9.6
.075336,883.5809344,2.886997997,3.379547207,0.087382224,0.087382224,1,1,9.623326657,16.89
75336,883.5809344,2.886997997,3.379547207,0.087382224,0.087382224,1,1,9.623326657,16.8977
7676,3,2,207,81,0,1,1,1,1,9,1188.075336,883.5809344,2.886997997,3.379547207,0.087382224,0.08
.379547207,0.087382224,0.087382224,1,1,9.623326657,16.89773604,83.56300311,24.05831664,1
.379547207,0.087382224,0.087382224,1,1,9.623326657,16.89773604,83.56300311,24.05831664,1
0,1,1,1,1,9,1188.075336,883.5809344,2.886997997,3.379547207,0.087382224,0.087382224,1,1,9.62
075336,883.5809344,2.886997997,3.379547207,0.087382224,0.087382224,1,1,9.623326657,16.897
997,3.379547207,0.087382224,0.087382224,1,1,9.623326657,16.89773604,83.56300311,24.058316
.886997997,3.379547207,0.087382224,0.087382224,1,1,9.623326657,16.89773604,83.56300311,24
938199243,0.087382224,0.087382224,1,1,9.120009698,15.75279697,85.88035757,19.15202037,31.
.255269,937.178978,0.91200097,3.938199243,0.087382224,0.087382224,1,1,9.120009698,15.7527
,0,1,0,1,1,12,1142.255269,937.178978,0.91200097,3.938199243,0.087382224,0.087382224,1,1,9.1
.938199243,0.087382224,0.087382224,1,1,9.120009698,15.75279697,85.88035757,19.15202037,3
.938199243,0.087382224,0.087382224,1,1,9.120009698,15.75279697,85.88035757,19.15202037,3
938199243,0.087382224,0.087382224,1,1,9.120009698,15.75279697,85.88035757,19.15202037,31.
0,1,0,1,1,12,1142.255269,937.178978,0.91200097,3.938199243,0.087382224,0.087382224,1,1,9.12
.938199243,0.087382224,0.087382224,1,1,9.120009698,15.75279697,85.88035757,19.15202037,3
255269,937.178978,0.91200097,3.938199243,0.087382224,0.087382224,1,1,9.120009698,15.75279
.938199243,0.087382224,0.087382224,1,1,9.120009698,15.75279697,85.88035757,19.15202037,3
255269,937.178978,0.91200097,3.938199243,0.087382224,0.087382224,1,1,9.120009698,15.75279
.938199243,0.087382224,0.087382224,1,1,9.120009698,15.75279697,85.88035757,19.15202037,3
0,1,0,1,1,12,1142.255269,937.178978,0.91200097,3.938199243,0.087382224,0.087382224,1,1,9.12
938199243,0.087382224,0.087382224,1,1,9.120009698,15.75279697,85.88035757,19.15202037,31.
938199243,0.087382224,0.087382224,1,1,9.120009698,15.75279697,85.88035757,19.15202037,31.
0,1,0,1,1,12,1142.255269,937.178978,0.91200097,3.938199243,0.087382224,0.087382224,1,1,9.12
0,1,0,1,1,12,1142.255269,937.178978,0.91200097,3.938199243,0.087382224,0.087382224,1,1,9.12
.255269,937.178978,0.91200097,3.938199243,0.087382224,0.087382224,1,1,9.120009698,15.7527
255269,937.178978,0.91200097,3.938199243,0.087382224,0.087382224,1,1,9.120009698,15.75279
7,3.938199243,0.087382224,0.087382224,1,1,9.120009698,15.75279697,85.88035757,19.15202037
.255269,937.178978,0.91200097,3.938199243,0.087382224,0.087382224,1,1,9.120009698,15.7527
0,1,0,1,1,12,1142.255269,937.178978,0.91200097,3.938199243,0.087382224,0.087382224,1,1,9.12
255269,937.178978,0.91200097,3.938199243,0.087382224,0.087382224,1,1,9.120009698,15.75279
.255269,937.178978,0.91200097,3.938199243,0.087382224,0.087382224,1,1,9.120009698,15.7527
.938199243,0.087382224,0.087382224,1,1,9.120009698,15.75279697,85.88035757,19.15202037,3
255269,937.178978,0.91200097,3.938199243,0.087382224,0.087382224,1,1,9.120009698,15.75279
97,3.938199243,0.087382224,0.087382224,1,1,9.120009698,15.75279697,85.88035757,19.1520203
97,3.938199243,0.087382224,0.087382224,1,1,9.120009698,15.75279697,85.88035757,19.1520203
142.255269,937.178978,0.91200097,3.938199243,0.087382224,0.087382224,1,1,9.120009698,15.7
3,0,0.087382224,0,1,0,2.753491072,0,872.107512,15.83257367,20.84816944,5.506982145,10.4240
8338653,855.9636594,0.688372768,0,0.087382224,0,1,0,2.753491072,0,872.107512,15.83257367,2
1,1,0,1,1,11,948.8338653,855.9636594,0.688372768,0,0.087382224,0,1,0,2.753491072,0,872.10751
8338653,855.9636594,0.688372768,0,0.087382224,0,1,0,2.753491072,0,872.107512,15.83257367,2
3,0,0.087382224,0,1,0,2.753491072,0,872.107512,15.83257367,20.84816944,5.506982145,10.4240
688372768,0,0.087382224,0,1,0,2.753491072,0,872.107512,15.83257367,20.84816944,5.50698214
8372768,0,0.087382224,0,1,0,2.753491072,0,872.107512,15.83257367,20.84816944,5.506982145,
.8338653,855.9636594,0.688372768,0,0.087382224,0,1,0,2.753491072,0,872.107512,15.83257367,
.8338653,855.9636594,0.688372768,0,0.087382224,0,1,0,2.753491072,0,872.107512,15.83257367,
.8338653,855.9636594,0.688372768,0,0.087382224,0,1,0,2.753491072,0,872.107512,15.83257367,

3372768,0,0.087382224,0,1,0,2.753491072,0,872.107512,15.83257367,20.84816944,5.506982145,1
1,1,0,1,1,11,948.8338653,855.9636594,0.688372768,0,0.087382224,0,1,0,2.753491072,0,872.10751
3,0,0.087382224,0,1,0,2.753491072,0,872.107512,15.83257367,20.84816944,5.506982145,10.42408
3338653,855.9636594,0.688372768,0,0.087382224,0,1,0,2.753491072,0,872.107512,15.83257367,2
3,0,0.087382224,0,1,0,2.753491072,0,872.107512,15.83257367,20.84816944,5.506982145,10.42408
768,0,0.087382224,0,1,0,2.753491072,0,872.107512,15.83257367,20.84816944,5.506982145,10.424
1,1,0,1,1,11,948.8338653,855.9636594,0.688372768,0,0.087382224,0,1,0,2.753491072,0,872.10751
3372768,0,0.087382224,0,1,0,2.753491072,0,872.107512,15.83257367,20.84816944,5.506982145,1
1,1,0,1,1,11,948.8338653,855.9636594,0.688372768,0,0.087382224,0,1,0,2.753491072,0,872.10751
3,0,0.087382224,0,1,0,2.753491072,0,872.107512,15.83257367,20.84816944,5.506982145,10.42408
338653,855.9636594,0.688372768,0,0.087382224,0,1,0,2.753491072,0,872.107512,15.83257367,20
338653,855.9636594,0.688372768,0,0.087382224,0,1,0,2.753491072,0,872.107512,15.83257367,20
1,1,0,1,1,11,948.8338653,855.9636594,0.688372768,0,0.087382224,0,1,0,2.753491072,0,872.10751
1,1,0,1,1,11,948.8338653,855.9636594,0.688372768,0,0.087382224,0,1,0,2.753491072,0,872.10751
3372768,0,0.087382224,0,1,0,2.753491072,0,872.107512,15.83257367,20.84816944,5.506982145,1
338653,855.9636594,0.688372768,0,0.087382224,0,1,0,2.753491072,0,872.107512,15.83257367,20
1,1,0,1,1,11,948.8338653,855.9636594,0.688372768,0,0.087382224,0,1,0,2.753491072,0,872.10751
72768,0,0.087382224,0,1,0,2.753491072,0,872.107512,15.83257367,20.84816944,5.506982145,10.4
3.8338653,855.9636594,0.688372768,0,0.087382224,0,1,0,2.753491072,0,872.107512,15.83257367
,1.441879636,0,4,0,6.872974338,12.67107482,80.21009103,28.35101914,46.46060766,4.29560896
52546,0,1.441879636,0,4,0,6.872974338,12.67107482,80.21009103,28.35101914,46.46060766,4.29
,1,1,0,1,1,8,1010.290313,1074.18078,6.013852546,0,1.441879636,0,4,0,6.872974338,12.67107482,
1,1,0,1,1,8,1010.290313,1074.18078,6.013852546,0,1.441879636,0,4,0,6.872974338,12.67107482,8
290313,1074.18078,6.013852546,0,1.441879636,0,4,0,6.872974338,12.67107482,80.21009103,28.3
,1.441879636,0,4,0,6.872974338,12.67107482,80.21009103,28.35101914,46.46060766,4.29560896
46,0,1.441879636,0,4,0,6.872974338,12.67107482,80.21009103,28.35101914,46.46060766,4.29560
2546,0,1.441879636,0,4,0,6.872974338,12.67107482,80.21009103,28.35101914,46.46060766,4.295
.290313,1074.18078,6.013852546,0,1.441879636,0,4,0,6.872974338,12.67107482,80.21009103,28.
.290313,1074.18078,6.013852546,0,1.441879636,0,4,0,6.872974338,12.67107482,80.21009103,28.
.290313,1074.18078,6.013852546,0,1.441879636,0,4,0,6.872974338,12.67107482,80.21009103,28.
52546,0,1.441879636,0,4,0,6.872974338,12.67107482,80.21009103,28.35101914,46.46060766,4.29
,1.441879636,0,4,0,6.872974338,12.67107482,80.21009103,28.35101914,46.46060766,4.29560896
46,0,1.441879636,0,4,0,6.872974338,12.67107482,80.21009103,28.35101914,46.46060766,4.29560
290313,1074.18078,6.013852546,0,1.441879636,0,4,0,6.872974338,12.67107482,80.21009103,28.3
,1.441879636,0,4,0,6.872974338,12.67107482,80.21009103,28.35101914,46.46060766,4.29560896
5,0,1.441879636,0,4,0,6.872974338,12.67107482,80.21009103,28.35101914,46.46060766,4.295608
1,1,0,1,1,8,1010.290313,1074.18078,6.013852546,0,1.441879636,0,4,0,6.872974338,12.67107482,8
290313,1074.18078,6.013852546,0,1.441879636,0,4,0,6.872974338,12.67107482,80.21009103,28.3
1,1,0,1,1,8,1010.290313,1074.18078,6.013852546,0,1.441879636,0,4,0,6.872974338,12.67107482,8
,1.441879636,0,4,0,6.872974338,12.67107482,80.21009103,28.35101914,46.46060766,4.29560896
90313,1074.18078,6.013852546,0,1.441879636,0,4,0,6.872974338,12.67107482,80.21009103,28.35
290313,1074.18078,6.013852546,0,1.441879636,0,4,0,6.872974338,12.67107482,80.21009103,28.3
).290313,1074.18078,6.013852546,0,1.441879636,0,4,0,6.872974338,12.67107482,80.21009103,28
290313,1074.18078,6.013852546,0,1.441879636,0,4,0,6.872974338,12.67107482,80.21009103,28.3
52546,0,1.441879636,0,4,0,6.872974338,12.67107482,80.21009103,28.35101914,46.46060766,4.29
90313,1074.18078,6.013852546,0,1.441879636,0,4,0,6.872974338,12.67107482,80.21009103,28.35
1,1,0,1,1,8,1010.290313,1074.18078,6.013852546,0,1.441879636,0,4,0,6.872974338,12.67107482,8
290313,1074.18078,6.013852546,0,1.441879636,0,4,0,6.872974338,12.67107482,80.21009103,28.3
2546,0,1.441879636,0,4,0,6.872974338,12.67107482,80.21009103,28.35101914,46.46060766,4.295
7906,0,0.912181185,0,2,0,6.550526335,10.05063835,210.3506739,27.13789482,40.2025534,4.6789

5374285,1006.186545,3.743157906,0,0.912181185,0,2,0,6.550526335,10.05063835,210.3506739,27
,1,1,0,1,1,11,869.5374285,1006.186545,3.743157906,0,0.912181185,0,2,0,6.550526335,10.050638
5374285,1006.186545,3.743157906,0,0.912181185,0,2,0,6.550526335,10.05063835,210.3506739,27
5374285,1006.186545,3.743157906,0,0.912181185,0,2,0,6.550526335,10.05063835,210.3506739,27
5374285,1006.186545,3.743157906,0,0.912181185,0,2,0,6.550526335,10.05063835,210.3506739,27
5374285,1006.186545,3.743157906,0,0.912181185,0,2,0,6.550526335,10.05063835,210.3506739,27
0,0.912181185,0,2,0,6.550526335,10.05063835,210.3506739,27.13789482,40.2025534,4.67894738
5374285,1006.186545,3.743157906,0,0.912181185,0,2,0,6.550526335,10.05063835,210.3506739,27
5374285,1006.186545,3.743157906,0,0.912181185,0,2,0,6.550526335,10.05063835,210.3506739,27
157906,0,0.912181185,0,2,0,6.550526335,10.05063835,210.3506739,27.13789482,40.2025534,4.67
59.5374285,1006.186545,3.743157906,0,0.912181185,0,2,0,6.550526335,10.05063835,210.350673
7906,0,0.912181185,0,2,0,6.550526335,10.05063835,210.3506739,27.13789482,40.2025534,4.6789
1,1,0,1,1,11,869.5374285,1006.186545,3.743157906,0,0.912181185,0,2,0,6.550526335,10.0506383
374285,1006.186545,3.743157906,0,0.912181185,0,2,0,6.550526335,10.05063835,210.3506739,27.
06,0,0.912181185,0,2,0,6.550526335,10.05063835,210.3506739,27.13789482,40.2025534,4.678947
5374285,1006.186545,3.743157906,0,0.912181185,0,2,0,6.550526335,10.05063835,210.3506739,27
7906,0,0.912181185,0,2,0,6.550526335,10.05063835,210.3506739,27.13789482,40.2025534,4.6789
157906,0,0.912181185,0,2,0,6.550526335,10.05063835,210.3506739,27.13789482,40.2025534,4.67
7906,0,0.912181185,0,2,0,6.550526335,10.05063835,210.3506739,27.13789482,40.2025534,4.6789
5,0,0.912181185,0,2,0,6.550526335,10.05063835,210.3506739,27.13789482,40.2025534,4.6789473
,1,1,0,1,1,11,869.5374285,1006.186545,3.743157906,0,0.912181185,0,2,0,6.550526335,10.050638
0,0.912181185,0,2,0,6.550526335,10.05063835,210.3506739,27.13789482,40.2025534,4.67894738
5,0,0.912181185,0,2,0,6.550526335,10.05063835,210.3506739,27.13789482,40.2025534,4.6789473
5374285,1006.186545,3.743157906,0,0.912181185,0,2,0,6.550526335,10.05063835,210.3506739,27
5374285,1006.186545,3.743157906,0,0.912181185,0,2,0,6.550526335,10.05063835,210.3506739,27
157906,0,0.912181185,0,2,0,6.550526335,10.05063835,210.3506739,27.13789482,40.2025534,4.67
1,1,0,1,1,11,869.5374285,1006.186545,3.743157906,0,0.912181185,0,2,0,6.550526335,10.0506383
,1,1,0,1,1,11,869.5374285,1006.186545,3.743157906,0,0.912181185,0,2,0,6.550526335,10.050638
37,3.112407601,1.303188786,0.66596937,3,1,7.604801895,9.337222802,53.60775925,28.13776701
.4619183,941.7441145,4.562881137,3.112407601,1.303188786,0.66596937,3,1,7.604801895,9.337
4619183,941.7441145,4.562881137,3.112407601,1.303188786,0.66596937,3,1,7.604801895,9.3372
37,3.112407601,1.303188786,0.66596937,3,1,7.604801895,9.337222802,53.60775925,28.13776701
4619183,941.7441145,4.562881137,3.112407601,1.303188786,0.66596937,3,1,7.604801895,9.3372
.4619183,941.7441145,4.562881137,3.112407601,1.303188786,0.66596937,3,1,7.604801895,9.337
,0,1,0,1,1,12,953.4619183,941.7441145,4.562881137,3.112407601,1.303188786,0.66596937,3,1,7.
3.4619183,941.7441145,4.562881137,3.112407601,1.303188786,0.66596937,3,1,7.604801895,9.33
.4619183,941.7441145,4.562881137,3.112407601,1.303188786,0.66596937,3,1,7.604801895,9.337
4619183,941.7441145,4.562881137,3.112407601,1.303188786,0.66596937,3,1,7.604801895,9.3372
4619183,941.7441145,4.562881137,3.112407601,1.303188786,0.66596937,3,1,7.604801895,9.3372
.4619183,941.7441145,4.562881137,3.112407601,1.303188786,0.66596937,3,1,7.604801895,9.337
.4619183,941.7441145,4.562881137,3.112407601,1.303188786,0.66596937,3,1,7.604801895,9.337
3.4619183,941.7441145,4.562881137,3.112407601,1.303188786,0.66596937,3,1,7.604801895,9.33
,0,1,0,1,1,12,953.4619183,941.7441145,4.562881137,3.112407601,1.303188786,0.66596937,3,1,7.
0,1,0,1,1,12,953.4619183,941.7441145,4.562881137,3.112407601,1.303188786,0.66596937,3,1,7.6
3.4619183,941.7441145,4.562881137,3.112407601,1.303188786,0.66596937,3,1,7.604801895,9.33
4619183,941.7441145,4.562881137,3.112407601,1.303188786,0.66596937,3,1,7.604801895,9.3372
37,3.112407601,1.303188786,0.66596937,3,1,7.604801895,9.337222802,53.60775925,28.13776701
4619183,941.7441145,4.562881137,3.112407601,1.303188786,0.66596937,3,1,7.604801895,9.3372
.4619183,941.7441145,4.562881137,3.112407601,1.303188786,0.66596937,3,1,7.604801895,9.337
4619183,941.7441145,4.562881137,3.112407601,1.303188786,0.66596937,3,1,7.604801895,9.3372

137,3.112407601,1.303188786,0.66596937,3,1,7.604801895,9.337222802,53.60775925,28.1377670
.4619183,941.7441145,4.562881137,3.112407601,1.303188786,0.66596937,3,1,7.604801895,9.337
.4619183,941.7441145,4.562881137,3.112407601,1.303188786,0.66596937,3,1,7.604801895,9.337
.4619183,941.7441145,4.562881137,3.112407601,1.303188786,0.66596937,3,1,7.604801895,9.337
4619183,941.7441145,4.562881137,3.112407601,1.303188786,0.66596937,3,1,7.604801895,9.3372
,0,1,0,1,1,12,953.4619183,941.7441145,4.562881137,3.112407601,1.303188786,0.66596937,3,1,7.6
4619183,941.7441145,4.562881137,3.112407601,1.303188786,0.66596937,3,1,7.604801895,9.3372
.4619183,941.7441145,4.562881137,3.112407601,1.303188786,0.66596937,3,1,7.604801895,9.337
37,3.112407601,1.303188786,0.66596937,3,1,7.604801895,9.337222802,53.60775925,28.13776701
.4619183,941.7441145,4.562881137,3.112407601,1.303188786,0.66596937,3,1,7.604801895,9.337
.4619183,941.7441145,4.562881137,3.112407601,1.303188786,0.66596937,3,1,7.604801895,9.337
,0,1,0,1,1,12,953.4619183,941.7441145,4.562881137,3.112407601,1.303188786,0.66596937,3,1,7.6
4,0,1,0,1,1,12,953.4619183,941.7441145,4.562881137,3.112407601,1.303188786,0.66596937,3,1,7
6.892706758,1.441879636,0,4,0,6.809673638,10.33906014,107.1105298,35.561629,41.35624055,5
0.970266,1082.673038,5.29641283,6.892706758,1.441879636,0,4,0,6.809673638,10.33906014,107
,1,1,1,1,1,12,1110.970266,1082.673038,5.29641283,6.892706758,1.441879636,0,4,0,6.809673638,1
0.970266,1082.673038,5.29641283,6.892706758,1.441879636,0,4,0,6.809673638,10.33906014,107
0.970266,1082.673038,5.29641283,6.892706758,1.441879636,0,4,0,6.809673638,10.33906014,107
.970266,1082.673038,5.29641283,6.892706758,1.441879636,0,4,0,6.809673638,10.33906014,107.1
3,6.892706758,1.441879636,0,4,0,6.809673638,10.33906014,107.1105298,35.561629,41.35624055,
0.970266,1082.673038,5.29641283,6.892706758,1.441879636,0,4,0,6.809673638,10.33906014,107
0.970266,1082.673038,5.29641283,6.892706758,1.441879636,0,4,0,6.809673638,10.33906014,107
0.970266,1082.673038,5.29641283,6.892706758,1.441879636,0,4,0,6.809673638,10.33906014,107
08838,3,2,208,70,1,1,1,1,1,12,1110.970266,1082.673038,5.29641283,6.892706758,1.441879636,0,4
0.970266,1082.673038,5.29641283,6.892706758,1.441879636,0,4,0,6.809673638,10.33906014,107
,1,1,1,1,1,12,1110.970266,1082.673038,5.29641283,6.892706758,1.441879636,0,4,0,6.809673638,1
,1,1,1,1,1,12,1110.970266,1082.673038,5.29641283,6.892706758,1.441879636,0,4,0,6.809673638,1
0.970266,1082.673038,5.29641283,6.892706758,1.441879636,0,4,0,6.809673638,10.33906014,107
.970266,1082.673038,5.29641283,6.892706758,1.441879636,0,4,0,6.809673638,10.33906014,107.1
0.970266,1082.673038,5.29641283,6.892706758,1.441879636,0,4,0,6.809673638,10.33906014,107
0.970266,1082.673038,5.29641283,6.892706758,1.441879636,0,4,0,6.809673638,10.33906014,107
33,6.892706758,1.441879636,0,4,0,6.809673638,10.33906014,107.1105298,35.561629,41.35624055
0.970266,1082.673038,5.29641283,6.892706758,1.441879636,0,4,0,6.809673638,10.33906014,107
91162,3,2,208,70,1,1,1,1,1,12,1110.970266,1082.673038,5.29641283,6.892706758,1.441879636,0,4
0.970266,1082.673038,5.29641283,6.892706758,1.441879636,0,4,0,6.809673638,10.33906014,107
0.970266,1082.673038,5.29641283,6.892706758,1.441879636,0,4,0,6.809673638,10.33906014,107
0.970266,1082.673038,5.29641283,6.892706758,1.441879636,0,4,0,6.809673638,10.33906014,107
1,1,1,1,1,12,1110.970266,1082.673038,5.29641283,6.892706758,1.441879636,0,4,0,6.809673638,10
.970266,1082.673038,5.29641283,6.892706758,1.441879636,0,4,0,6.809673638,10.33906014,107.1
.970266,1082.673038,5.29641283,6.892706758,1.441879636,0,4,0,6.809673638,10.33906014,107.1
,1,1,1,1,1,12,1110.970266,1082.673038,5.29641283,6.892706758,1.441879636,0,4,0,6.809673638,1
0.970266,1082.673038,5.29641283,6.892706758,1.441879636,0,4,0,6.809673638,10.33906014,107
0.970266,1082.673038,5.29641283,6.892706758,1.441879636,0,4,0,6.809673638,10.33906014,107
9737,4.309060355,0.912181185,0.66596937,2,1,5.168954606,8.61812071,330.056779,26.70626546
4.309060355,0.912181185,0.66596937,2,1,5.168954606,8.61812071,330.056779,26.70626546,34.4
951192,873.9591819,3.445969737,4.309060355,0.912181185,0.66596937,2,1,5.168954606,8.61812
2,1,1,1,0,1,8,842.6951192,873.9591819,3.445969737,4.309060355,0.912181185,0.66596937,2,1,5.1
2,1,1,1,0,1,8,842.6951192,873.9591819,3.445969737,4.309060355,0.912181185,0.66596937,2,1,5.1
2,1,1,1,0,1,8,842.6951192,873.9591819,3.445969737,4.309060355,0.912181185,0.66596937,2,1,5.1
7,4.309060355,0.912181185,0.66596937,2,1,5.168954606,8.61812071,330.056779,26.70626546,34.

7,4.309060355,0.912181185,0.66596937,2,1,5.168954606,8.61812071,330.056779,26.70626546,34.
37,4.309060355,0.912181185,0.66596937,2,1,5.168954606,8.61812071,330.056779,26.70626546,34
5951192,873.9591819,3.445969737,4.309060355,0.912181185,0.66596937,2,1,5.168954606,8.6181
5951192,873.9591819,3.445969737,4.309060355,0.912181185,0.66596937,2,1,5.168954606,8.6181
.6951192,873.9591819,3.445969737,4.309060355,0.912181185,0.66596937,2,1,5.168954606,8.618
951192,873.9591819,3.445969737,4.309060355,0.912181185,0.66596937,2,1,5.168954606,8.61812
5951192,873.9591819,3.445969737,4.309060355,0.912181185,0.66596937,2,1,5.168954606,8.6181
.309060355,0.912181185,0.66596937,2,1,5.168954606,8.61812071,330.056779,26.70626546,34.47
5951192,873.9591819,3.445969737,4.309060355,0.912181185,0.66596937,2,1,5.168954606,8.6181
2,1,1,1,0,1,8,842.6951192,873.9591819,3.445969737,4.309060355,0.912181185,0.66596937,2,1,5.
517676,3,2,208,82,1,1,1,0,1,8,842.6951192,873.9591819,3.445969737,4.309060355,0.912181185,0.
45117,3,2,208,82,1,1,1,0,1,8,842.6951192,873.9591819,3.445969737,4.309060355,0.912181185,0.6
.6951192,873.9591819,3.445969737,4.309060355,0.912181185,0.66596937,2,1,5.168954606,8.618
,1,1,1,0,1,8,842.6951192,873.9591819,3.445969737,4.309060355,0.912181185,0.66596937,2,1,5.1
5951192,873.9591819,3.445969737,4.309060355,0.912181185,0.66596937,2,1,5.168954606,8.6181
,1,1,1,0,1,8,842.6951192,873.9591819,3.445969737,4.309060355,0.912181185,0.66596937,2,1,5.1
37,4.309060355,0.912181185,0.66596937,2,1,5.168954606,8.61812071,330.056779,26.70626546,34
2,1,1,1,0,1,8,842.6951192,873.9591819,3.445969737,4.309060355,0.912181185,0.66596937,2,1,5.
19,3.445969737,4.309060355,0.912181185,0.66596937,2,1,5.168954606,8.61812071,330.056779,2
2.6951192,873.9591819,3.445969737,4.309060355,0.912181185,0.66596937,2,1,5.168954606,8.61
82,1,1,1,0,1,8,842.6951192,873.9591819,3.445969737,4.309060355,0.912181185,0.66596937,2,1,5
37,4.309060355,0.912181185,0.66596937,2,1,5.168954606,8.61812071,330.056779,26.70626546,34
2,1,1,1,0,1,8,842.6951192,873.9591819,3.445969737,4.309060355,0.912181185,0.66596937,2,1,5.
37,4.309060355,0.912181185,0.66596937,2,1,5.168954606,8.61812071,330.056779,26.70626546,34
5951192,873.9591819,3.445969737,4.309060355,0.912181185,0.66596937,2,1,5.168954606,8.6181
.6951192,873.9591819,3.445969737,4.309060355,0.912181185,0.66596937,2,1,5.168954606,8.618
37,4.309060355,0.912181185,0.66596937,2,1,5.168954606,8.61812071,330.056779,26.70626546,34
2,1,1,1,0,1,8,842.6951192,873.9591819,3.445969737,4.309060355,0.912181185,0.66596937,2,1,5.
5951192,873.9591819,3.445969737,4.309060355,0.912181185,0.66596937,2,1,5.168954606,8.6181
37,4.309060355,0.912181185,0.66596937,2,1,5.168954606,8.61812071,330.056779,26.70626546,34
5258789,3,2,208,82,1,1,1,0,1,8,842.6951192,873.9591819,3.445969737,4.309060355,0.912181185,
9382324,3,2,208,82,1,1,1,0,1,8,842.6951192,873.9591819,3.445969737,4.309060355,0.912181185,
37,4.309060355,0.912181185,0.66596937,2,1,5.168954606,8.61812071,330.056779,26.70626546,34
59737,4.309060355,0.912181185,0.66596937,2,1,5.168954606,8.61812071,330.056779,26.7062654
7,4.309060355,0.912181185,0.66596937,2,1,5.168954606,8.61812071,330.056779,26.70626546,34.
37,4.309060355,0.912181185,0.66596937,2,1,5.168954606,8.61812071,330.056779,26.70626546,34
7,4.309060355,0.912181185,0.66596937,2,1,5.168954606,8.61812071,330.056779,26.70626546,34.
.6951192,873.9591819,3.445969737,4.309060355,0.912181185,0.66596937,2,1,5.168954606,8.618
.6951192,873.9591819,3.445969737,4.309060355,0.912181185,0.66596937,2,1,5.168954606,8.618
37,4.309060355,0.912181185,0.66596937,2,1,5.168954606,8.61812071,330.056779,26.70626546,34
5951192,873.9591819,3.445969737,4.309060355,0.912181185,0.66596937,2,1,5.168954606,8.6181
45,3.394503284,0.66596937,0.66596937,1,1,5.847365782,3.394503284,78.64506909,21.92762168,
.9416786,959.2939377,1.461841445,3.394503284,0.66596937,0.66596937,1,1,5.847365782,3.3945
9416786,959.2939377,1.461841445,3.394503284,0.66596937,0.66596937,1,1,5.847365782,3.39450
.9416786,959.2939377,1.461841445,3.394503284,0.66596937,0.66596937,1,1,5.847365782,3.3945
45,3.394503284,0.66596937,0.66596937,1,1,5.847365782,3.394503284,78.64506909,21.92762168,
591162,3,2,208,78,1,0,1,0,1,8,871.9416786,959.2939377,1.461841445,3.394503284,0.66596937,0.6
.9416786,959.2939377,1.461841445,3.394503284,0.66596937,0.66596937,1,1,5.847365782,3.3945
9416786,959.2939377,1.461841445,3.394503284,0.66596937,0.66596937,1,1,5.847365782,3.39450
.9416786,959.2939377,1.461841445,3.394503284,0.66596937,0.66596937,1,1,5.847365782,3.3945

9416786,959.2939377,1.461841445,3.394503284,0.66596937,0.66596937,1,1,5.847365782,3.39450
78,1,0,1,0,1,8,871.9416786,959.2939377,1.461841445,3.394503284,0.66596937,0.66596937,1,1,5.8
78,1,0,1,0,1,8,871.9416786,959.2939377,1.461841445,3.394503284,0.66596937,0.66596937,1,1,5.8
.9416786,959.2939377,1.461841445,3.394503284,0.66596937,0.66596937,1,1,5.847365782,3.3945
9416786,959.2939377,1.461841445,3.394503284,0.66596937,0.66596937,1,1,5.847365782,3.39450
78,1,0,1,0,1,8,871.9416786,959.2939377,1.461841445,3.394503284,0.66596937,0.66596937,1,1,5.8
382324,3,2,208,78,1,0,1,0,1,8,871.9416786,959.2939377,1.461841445,3.394503284,0.66596937,0.6
9416786,959.2939377,1.461841445,3.394503284,0.66596937,0.66596937,1,1,5.847365782,3.39450
.9416786,959.2939377,1.461841445,3.394503284,0.66596937,0.66596937,1,1,5.847365782,3.3945
1.9416786,959.2939377,1.461841445,3.394503284,0.66596937,0.66596937,1,1,5.847365782,3.394
1.9416786,959.2939377,1.461841445,3.394503284,0.66596937,0.66596937,1,1,5.847365782,3.394
517676,3,2,208,78,1,0,1,0,1,8,871.9416786,959.2939377,1.461841445,3.394503284,0.66596937,0.6
9416786,959.2939377,1.461841445,3.394503284,0.66596937,0.66596937,1,1,5.847365782,3.39450
.9416786,959.2939377,1.461841445,3.394503284,0.66596937,0.66596937,1,1,5.847365782,3.3945
.9416786,959.2939377,1.461841445,3.394503284,0.66596937,0.66596937,1,1,5.847365782,3.3945
1.9416786,959.2939377,1.461841445,3.394503284,0.66596937,0.66596937,1,1,5.847365782,3.394
.9416786,959.2939377,1.461841445,3.394503284,0.66596937,0.66596937,1,1,5.847365782,3.3945
.9416786,959.2939377,1.461841445,3.394503284,0.66596937,0.66596937,1,1,5.847365782,3.3945
78,1,0,1,0,1,8,871.9416786,959.2939377,1.461841445,3.394503284,0.66596937,0.66596937,1,1,5.8
1.9416786,959.2939377,1.461841445,3.394503284,0.66596937,0.66596937,1,1,5.847365782,3.394
1.9416786,959.2939377,1.461841445,3.394503284,0.66596937,0.66596937,1,1,5.847365782,3.394
2,11.64124681,2.169722626,2.169722626,2,2,0,0,1754.141195,6.172836482,3.880415602,1.543209
52,11.64124681,2.169722626,2.169722626,2,2,0,0,1754.141195,6.172836482,3.880415602,1.54320
29627362,11.64124681,2.169722626,2.169722626,2,2,0,0,1754.141195,6.172836482,3.880415602,
.6355938,331.6605803,4.629627362,11.64124681,2.169722626,2.169722626,2,2,0,0,1754.141195,6
4,0,1,0,1,1,9,368.6355938,331.6605803,4.629627362,11.64124681,2.169722626,2.169722626,2,2,0
52,11.64124681,2.169722626,2.169722626,2,2,0,0,1754.141195,6.172836482,3.880415602,1.54320
.6355938,331.6605803,4.629627362,11.64124681,2.169722626,2.169722626,2,2,0,0,1754.141195,6
.6355938,331.6605803,4.629627362,11.64124681,2.169722626,2.169722626,2,2,0,0,1754.141195,6
29627362,11.64124681,2.169722626,2.169722626,2,2,0,0,1754.141195,6.172836482,3.880415602,1
6355938,331.6605803,4.629627362,11.64124681,2.169722626,2.169722626,2,2,0,0,1754.141195,6.
2,11.64124681,2.169722626,2.169722626,2,2,0,0,1754.141195,6.172836482,3.880415602,1.543209
6355938,331.6605803,4.629627362,11.64124681,2.169722626,2.169722626,2,2,0,0,1754.141195,6.
52,11.64124681,2.169722626,2.169722626,2,2,0,0,1754.141195,6.172836482,3.880415602,1.54320
4,0,1,0,1,1,9,368.6355938,331.6605803,4.629627362,11.64124681,2.169722626,2.169722626,2,2,0
6355938,331.6605803,4.629627362,11.64124681,2.169722626,2.169722626,2,2,0,0,1754.141195,6.
52,11.64124681,2.169722626,2.169722626,2,2,0,0,1754.141195,6.172836482,3.880415602,1.54320
52,11.64124681,2.169722626,2.169722626,2,2,0,0,1754.141195,6.172836482,3.880415602,1.54320
362,11.64124681,2.169722626,2.169722626,2,2,0,0,1754.141195,6.172836482,3.880415602,1.5432
8.6355938,331.6605803,4.629627362,11.64124681,2.169722626,2.169722626,2,2,0,0,1754.141195
9627362,11.64124681,2.169722626,2.169722626,2,2,0,0,1754.141195,6.172836482,3.880415602,1.
8.6355938,331.6605803,4.629627362,11.64124681,2.169722626,2.169722626,2,2,0,0,1754.141195
74,0,1,0,1,1,9,368.6355938,331.6605803,4.629627362,11.64124681,2.169722626,2.169722626,2,2,
52,11.64124681,2.169722626,2.169722626,2,2,0,0,1754.141195,6.172836482,3.880415602,1.54320
6355938,331.6605803,4.629627362,11.64124681,2.169722626,2.169722626,2,2,0,0,1754.141195,6.
.6355938,331.6605803,4.629627362,11.64124681,2.169722626,2.169722626,2,2,0,0,1754.141195,6
.6355938,331.6605803,4.629627362,11.64124681,2.169722626,2.169722626,2,2,0,0,1754.141195,6
6355938,331.6605803,4.629627362,11.64124681,2.169722626,2.169722626,2,2,0,0,1754.141195,6.
27362,11.64124681,2.169722626,2.169722626,2,2,0,0,1754.141195,6.172836482,3.880415602,1.54
.6355938,331.6605803,4.629627362,11.64124681,2.169722626,2.169722626,2,2,0,0,1754.141195,6

4,0,1,0,1,1,9,368.6355938,331.6605803,4.629627362,11.64124681,2.169722626,2.169722626,2,2,0
355938,331.6605803,4.629627362,11.64124681,2.169722626,2.169722626,2,2,0,0,1754.141195,6.
8.6355938,331.6605803,4.629627362,11.64124681,2.169722626,2.169722626,2,2,0,0,1754.14119
425,0,0,0,0,0,0,1442.331006,5.088631274,5.483716388,1.696210425,5.483716388,1.696210425,0
1.0141299,378.9749549,1.696210425,0,0,0,0,0,0,1442.331006,5.088631274,5.483716388,1.6962
425,0,0,0,0,0,0,1442.331006,5.088631274,5.483716388,1.696210425,5.483716388,1.696210425,0
25,0,0,0,0,0,0,1442.331006,5.088631274,5.483716388,1.696210425,5.483716388,1.696210425,0,
1.0141299,378.9749549,1.696210425,0,0,0,0,0,0,1442.331006,5.088631274,5.483716388,1.6962
.0141299,378.9749549,1.696210425,0,0,0,0,0,0,1442.331006,5.088631274,5.483716388,1.69621
425,0,0,0,0,0,0,1442.331006,5.088631274,5.483716388,1.696210425,5.483716388,1.696210425,0
1.0141299,378.9749549,1.696210425,0,0,0,0,0,0,1442.331006,5.088631274,5.483716388,1.6962
.0141299,378.9749549,1.696210425,0,0,0,0,0,0,1442.331006,5.088631274,5.483716388,1.69621
1.0141299,378.9749549,1.696210425,0,0,0,0,0,0,1442.331006,5.088631274,5.483716388,1.6962
,0,0,0,0,0,0,1442.331006,5.088631274,5.483716388,1.696210425,5.483716388,1.696210425,0,0,
1.0141299,378.9749549,1.696210425,0,0,0,0,0,0,1442.331006,5.088631274,5.483716388,1.6962
8,0,1,0,1,1,10,351.0141299,378.9749549,1.696210425,0,0,0,0,0,0,1442.331006,5.088631274,5.48
1.0141299,378.9749549,1.696210425,0,0,0,0,0,0,1442.331006,5.088631274,5.483716388,1.6962
68,0,1,0,1,1,10,351.0141299,378.9749549,1.696210425,0,0,0,0,0,0,1442.331006,5.088631274,5.4
1.0141299,378.9749549,1.696210425,0,0,0,0,0,0,1442.331006,5.088631274,5.483716388,1.6962
.0141299,378.9749549,1.696210425,0,0,0,0,0,0,1442.331006,5.088631274,5.483716388,1.69621
1.0141299,378.9749549,1.696210425,0,0,0,0,0,0,1442.331006,5.088631274,5.483716388,1.6962
1.0141299,378.9749549,1.696210425,0,0,0,0,0,0,1442.331006,5.088631274,5.483716388,1.6962
.0141299,378.9749549,1.696210425,0,0,0,0,0,0,1442.331006,5.088631274,5.483716388,1.69621
.0141299,378.9749549,1.696210425,0,0,0,0,0,0,1442.331006,5.088631274,5.483716388,1.69621
1.0141299,378.9749549,1.696210425,0,0,0,0,0,0,1442.331006,5.088631274,5.483716388,1.6962
1.0141299,378.9749549,1.696210425,0,0,0,0,0,0,1442.331006,5.088631274,5.483716388,1.6962
210425,0,0,0,0,0,0,1442.331006,5.088631274,5.483716388,1.696210425,5.483716388,1.69621042
9,25.47967795,9.480286879,3.856306804,7,3,7.08887058,7.279907986,284.4315777,61.43687836
,25.47967795,9.480286879,3.856306804,7,3,7.08887058,7.279907986,284.4315777,61.43687836,
09,25.47967795,9.480286879,3.856306804,7,3,7.08887058,7.279907986,284.4315777,61.4368783
7.648785,1974.724251,8.270349009,25.47967795,9.480286879,3.856306804,7,3,7.08887058,7.279
.648785,1974.724251,8.270349009,25.47967795,9.480286879,3.856306804,7,3,7.08887058,7.2799
.648785,1974.724251,8.270349009,25.47967795,9.480286879,3.856306804,7,3,7.08887058,7.2799
9,25.47967795,9.480286879,3.856306804,7,3,7.08887058,7.279907986,284.4315777,61.43687836
6,1,1,0,1,1,8,1927.648785,1974.724251,8.270349009,25.47967795,9.480286879,3.856306804,7,3,7
7.648785,1974.724251,8.270349009,25.47967795,9.480286879,3.856306804,7,3,7.08887058,7.279
.648785,1974.724251,8.270349009,25.47967795,9.480286879,3.856306804,7,3,7.08887058,7.2799
27.648785,1974.724251,8.270349009,25.47967795,9.480286879,3.856306804,7,3,7.08887058,7.27
,1,1,0,1,1,8,1927.648785,1974.724251,8.270349009,25.47967795,9.480286879,3.856306804,7,3,7.
.648785,1974.724251,8.270349009,25.47967795,9.480286879,3.856306804,7,3,7.08887058,7.2799
.648785,1974.724251,8.270349009,25.47967795,9.480286879,3.856306804,7,3,7.08887058,7.2799
27.648785,1974.724251,8.270349009,25.47967795,9.480286879,3.856306804,7,3,7.08887058,7.27
,1,1,0,1,1,8,1927.648785,1974.724251,8.270349009,25.47967795,9.480286879,3.856306804,7,3,7.
6,1,1,0,1,1,8,1927.648785,1974.724251,8.270349009,25.47967795,9.480286879,3.856306804,7,3,7
349009,25.47967795,9.480286879,3.856306804,7,3,7.08887058,7.279907986,284.4315777,61.436
7.648785,1974.724251,8.270349009,25.47967795,9.480286879,3.856306804,7,3,7.08887058,7.279
27.648785,1974.724251,8.270349009,25.47967795,9.480286879,3.856306804,7,3,7.08887058,7.27
9,25.47967795,9.480286879,3.856306804,7,3,7.08887058,7.279907986,284.4315777,61.43687836
7.648785,1974.724251,8.270349009,25.47967795,9.480286879,3.856306804,7,3,7.08887058,7.279
.648785,1974.724251,8.270349009,25.47967795,9.480286879,3.856306804,7,3,7.08887058,7.2799

7.648785,1974.724251,8.270349009,25.47967795,9.480286879,3.856306804,7,3,7.08887058,7.279
6,1,1,0,1,1,8,1927.648785,1974.724251,8.270349009,25.47967795,9.480286879,3.856306804,7,3,7
9,25.47967795,9.480286879,3.856306804,7,3,7.08887058,7.279907986,284.4315777,61.43687836
7.648785,1974.724251,8.270349009,25.47967795,9.480286879,3.856306804,7,3,7.08887058,7.279
.648785,1974.724251,8.270349009,25.47967795,9.480286879,3.856306804,7,3,7.08887058,7.2799
6,1,1,0,1,1,8,1927.648785,1974.724251,8.270349009,25.47967795,9.480286879,3.856306804,7,3,7
349009,25.47967795,9.480286879,3.856306804,7,3,7.08887058,7.279907986,284.4315777,61.436
009,25.47967795,9.480286879,3.856306804,7,3,7.08887058,7.279907986,284.4315777,61.436878
63,7.384249633,9.238650271,8.14418704,5,2,7.521652625,0,657.6776245,57.16455995,40.61337
71.466853,1124.491448,6.769487363,7.384249633,9.238650271,8.14418704,5,2,7.521652625,0,65
71.466853,1124.491448,6.769487363,7.384249633,9.238650271,8.14418704,5,2,7.521652625,0,65
1.466853,1124.491448,6.769487363,7.384249633,9.238650271,8.14418704,5,2,7.521652625,0,657
71.466853,1124.491448,6.769487363,7.384249633,9.238650271,8.14418704,5,2,7.521652625,0,65
,0,1,0,1,1,12,1671.466853,1124.491448,6.769487363,7.384249633,9.238650271,8.14418704,5,2,7.
71.466853,1124.491448,6.769487363,7.384249633,9.238650271,8.14418704,5,2,7.521652625,0,65
4,0,1,0,1,1,12,1671.466853,1124.491448,6.769487363,7.384249633,9.238650271,8.14418704,5,2,7
71.466853,1124.491448,6.769487363,7.384249633,9.238650271,8.14418704,5,2,7.521652625,0,65
4,0,1,0,1,1,12,1671.466853,1124.491448,6.769487363,7.384249633,9.238650271,8.14418704,5,2,7
,0,1,0,1,1,12,1671.466853,1124.491448,6.769487363,7.384249633,9.238650271,8.14418704,5,2,7.
4,0,1,0,1,1,12,1671.466853,1124.491448,6.769487363,7.384249633,9.238650271,8.14418704,5,2,7
71.466853,1124.491448,6.769487363,7.384249633,9.238650271,8.14418704,5,2,7.521652625,0,65
1.466853,1124.491448,6.769487363,7.384249633,9.238650271,8.14418704,5,2,7.521652625,0,657
71.466853,1124.491448,6.769487363,7.384249633,9.238650271,8.14418704,5,2,7.521652625,0,65
.466853,1124.491448,6.769487363,7.384249633,9.238650271,8.14418704,5,2,7.521652625,0,657.
71.466853,1124.491448,6.769487363,7.384249633,9.238650271,8.14418704,5,2,7.521652625,0,65
71.466853,1124.491448,6.769487363,7.384249633,9.238650271,8.14418704,5,2,7.521652625,0,65
71.466853,1124.491448,6.769487363,7.384249633,9.238650271,8.14418704,5,2,7.521652625,0,65
4,0,1,0,1,1,12,1671.466853,1124.491448,6.769487363,7.384249633,9.238650271,8.14418704,5,2,7
455811,3,2,208,74,0,1,0,1,1,12,1671.466853,1124.491448,6.769487363,7.384249633,9.238650271,
71.466853,1124.491448,6.769487363,7.384249633,9.238650271,8.14418704,5,2,7.521652625,0,65
4,0,1,0,1,1,12,1671.466853,1124.491448,6.769487363,7.384249633,9.238650271,8.14418704,5,2,7
4,0,1,0,1,1,12,1671.466853,1124.491448,6.769487363,7.384249633,9.238650271,8.14418704,5,2,7
4,0,1,0,1,1,12,1671.466853,1124.491448,6.769487363,7.384249633,9.238650271,8.14418704,5,2,7
1.466853,1124.491448,6.769487363,7.384249633,9.238650271,8.14418704,5,2,7.521652625,0,657
4,0,1,0,1,1,12,1671.466853,1124.491448,6.769487363,7.384249633,9.238650271,8.14418704,5,2,7
71.466853,1124.491448,6.769487363,7.384249633,9.238650271,8.14418704,5,2,7.521652625,0,65
517676,3,2,208,74,0,1,0,1,1,12,1671.466853,1124.491448,6.769487363,7.384249633,9.238650271,
9845581,3,2,208,74,0,1,0,1,1,12,1671.466853,1124.491448,6.769487363,7.384249633,9.238650271
71.466853,1124.491448,6.769487363,7.384249633,9.238650271,8.14418704,5,2,7.521652625,0,65
71.466853,1124.491448,6.769487363,7.384249633,9.238650271,8.14418704,5,2,7.521652625,0,65
71.466853,1124.491448,6.769487363,7.384249633,9.238650271,8.14418704,5,2,7.521652625,0,65
71.466853,1124.491448,6.769487363,7.384249633,9.238650271,8.14418704,5,2,7.521652625,0,65
.9987918,897.5603387,3.534787397,4.326261809,0.912181185,0.66596937,2,1,3.534787397,8.652
5.9987918,897.5603387,3.534787397,4.326261809,0.912181185,0.66596937,2,1,3.534787397,8.65
7,4.326261809,0.912181185,0.66596937,2,1,3.534787397,8.652523618,298.8198122,27.39460232
9987918,897.5603387,3.534787397,4.326261809,0.912181185,0.66596937,2,1,3.534787397,8.6525
9987918,897.5603387,3.534787397,4.326261809,0.912181185,0.66596937,2,1,3.534787397,8.6525
7,4.326261809,0.912181185,0.66596937,2,1,3.534787397,8.652523618,298.8198122,27.39460232,
9987918,897.5603387,3.534787397,4.326261809,0.912181185,0.66596937,2,1,3.534787397,8.6525
5.9987918,897.5603387,3.534787397,4.326261809,0.912181185,0.66596937,2,1,3.534787397,8.65

.9987918,897.5603387,3.534787397,4.326261809,0.912181185,0.66596937,2,1,3.534787397,8.652
79,1,1,1,1,1,5,835.9987918,897.5603387,3.534787397,4.326261809,0.912181185,0.66596937,2,1,3
79,1,1,1,1,1,5,835.9987918,897.5603387,3.534787397,4.326261809,0.912181185,0.66596937,2,1,3
97,4.326261809,0.912181185,0.66596937,2,1,3.534787397,8.652523618,298.8198122,27.39460232
5.9987918,897.5603387,3.534787397,4.326261809,0.912181185,0.66596937,2,1,3.534787397,8.65
5.9987918,897.5603387,3.534787397,4.326261809,0.912181185,0.66596937,2,1,3.534787397,8.65
97,4.326261809,0.912181185,0.66596937,2,1,3.534787397,8.652523618,298.8198122,27.39460232
97,4.326261809,0.912181185,0.66596937,2,1,3.534787397,8.652523618,298.8198122,27.39460232
9987918,897.5603387,3.534787397,4.326261809,0.912181185,0.66596937,2,1,3.534787397,8.6525
5.9987918,897.5603387,3.534787397,4.326261809,0.912181185,0.66596937,2,1,3.534787397,8.65
9987918,897.5603387,3.534787397,4.326261809,0.912181185,0.66596937,2,1,3.534787397,8.6525
97,4.326261809,0.912181185,0.66596937,2,1,3.534787397,8.652523618,298.8198122,27.39460232
9987918,897.5603387,3.534787397,4.326261809,0.912181185,0.66596937,2,1,3.534787397,8.6525
9,1,1,1,1,1,5,835.9987918,897.5603387,3.534787397,4.326261809,0.912181185,0.66596937,2,1,3.
97,4.326261809,0.912181185,0.66596937,2,1,3.534787397,8.652523618,298.8198122,27.39460232
9987918,897.5603387,3.534787397,4.326261809,0.912181185,0.66596937,2,1,3.534787397,8.6525
97,4.326261809,0.912181185,0.66596937,2,1,3.534787397,8.652523618,298.8198122,27.39460232
79,1,1,1,1,1,5,835.9987918,897.5603387,3.534787397,4.326261809,0.912181185,0.66596937,2,1,3
0617676,3,2,208,79,1,1,1,1,1,5,835.9987918,897.5603387,3.534787397,4.326261809,0.912181185,
97,4.326261809,0.912181185,0.66596937,2,1,3.534787397,8.652523618,298.8198122,27.39460232
97,4.326261809,0.912181185,0.66596937,2,1,3.534787397,8.652523618,298.8198122,27.39460232
9987918,897.5603387,3.534787397,4.326261809,0.912181185,0.66596937,2,1,3.534787397,8.6525
382324,3,2,208,72,1,1,1,1,1,9,850.1233058,827.6911942,2.487157465,3.900147936,0.912181185,0.
.1233058,827.6911942,2.487157465,3.900147936,0.912181185,0.66596937,2,1,4.974314929,0,441
.1233058,827.6911942,2.487157465,3.900147936,0.912181185,0.66596937,2,1,4.974314929,0,441
.1233058,827.6911942,2.487157465,3.900147936,0.912181185,0.66596937,2,1,4.974314929,0,441
258789,3,2,208,72,1,1,1,1,1,9,850.1233058,827.6911942,2.487157465,3.900147936,0.912181185,0.
55,3.900147936,0.912181185,0.66596937,2,1,4.974314929,0,441.1079392,22.38441718,31.201183
2,1,1,1,1,1,9,850.1233058,827.6911942,2.487157465,3.900147936,0.912181185,0.66596937,2,1,4.
0.1233058,827.6911942,2.487157465,3.900147936,0.912181185,0.66596937,2,1,4.974314929,0,44
2,1,1,1,1,1,9,850.1233058,827.6911942,2.487157465,3.900147936,0.912181185,0.66596937,2,1,4.
1233058,827.6911942,2.487157465,3.900147936,0.912181185,0.66596937,2,1,4.974314929,0,441.
.1233058,827.6911942,2.487157465,3.900147936,0.912181185,0.66596937,2,1,4.974314929,0,441
2,1,1,1,1,1,9,850.1233058,827.6911942,2.487157465,3.900147936,0.912181185,0.66596937,2,1,4.
2,1,1,1,1,1,9,850.1233058,827.6911942,2.487157465,3.900147936,0.912181185,0.66596937,2,1,4.
1233058,827.6911942,2.487157465,3.900147936,0.912181185,0.66596937,2,1,4.974314929,0,441.
55,3.900147936,0.912181185,0.66596937,2,1,4.974314929,0,441.1079392,22.38441718,31.201183
.1233058,827.6911942,2.487157465,3.900147936,0.912181185,0.66596937,2,1,4.974314929,0,441
1233058,827.6911942,2.487157465,3.900147936,0.912181185,0.66596937,2,1,4.974314929,0,441.
55,3.900147936,0.912181185,0.66596937,2,1,4.974314929,0,441.1079392,22.38441718,31.201183
2,1,1,1,1,1,9,850.1233058,827.6911942,2.487157465,3.900147936,0.912181185,0.66596937,2,1,4.
1233058,827.6911942,2.487157465,3.900147936,0.912181185,0.66596937,2,1,4.974314929,0,441.
1233058,827.6911942,2.487157465,3.900147936,0.912181185,0.66596937,2,1,4.974314929,0,441.
2,1,1,1,1,1,9,850.1233058,827.6911942,2.487157465,3.900147936,0.912181185,0.66596937,2,1,4.
72,1,1,1,1,1,9,850.1233058,827.6911942,2.487157465,3.900147936,0.912181185,0.66596937,2,1,4
1233058,827.6911942,2.487157465,3.900147936,0.912181185,0.66596937,2,1,4.974314929,0,441.
382324,3,2,208,72,1,1,1,1,1,9,850.1233058,827.6911942,2.487157465,3.900147936,0.912181185,0.
7465,3.900147936,0.912181185,0.66596937,2,1,4.974314929,0,441.1079392,22.38441718,31.2011
2,1,1,1,1,1,9,850.1233058,827.6911942,2.487157465,3.900147936,0.912181185,0.66596937,2,1,4.
87157465,3.900147936,0.912181185,0.66596937,2,1,4.974314929,0,441.1079392,22.38441718,31.

2,1,1,1,1,1,9,850.1233058,827.6911942,2.487157465,3.900147936,0.912181185,0.66596937,2,1,4.
57465,3.900147936,0.912181185,0.66596937,2,1,4.974314929,0,441.1079392,22.38441718,31.201
6,0,1,0,1,1,9,1927.366351,1998.711604,7.76583867,10.39454007,9.629657873,3.303534583,6,1,8.
7,10.39454007,9.629657873,3.303534583,6,1,8.960583081,3.46484669,318.582845,54.36087069,7
27.366351,1998.711604,7.76583867,10.39454007,9.629657873,3.303534583,6,1,8.960583081,3.46
6,0,1,0,1,1,9,1927.366351,1998.711604,7.76583867,10.39454007,9.629657873,3.303534583,6,1,8.
3867,10.39454007,9.629657873,3.303534583,6,1,8.960583081,3.46484669,318.582845,54.3608706
6,0,1,0,1,1,9,1927.366351,1998.711604,7.76583867,10.39454007,9.629657873,3.303534583,6,1,8.
7.366351,1998.711604,7.76583867,10.39454007,9.629657873,3.303534583,6,1,8.960583081,3.464
6,0,1,0,1,1,9,1927.366351,1998.711604,7.76583867,10.39454007,9.629657873,3.303534583,6,1,8.
3867,10.39454007,9.629657873,3.303534583,6,1,8.960583081,3.46484669,318.582845,54.3608706
66,0,1,0,1,1,9,1927.366351,1998.711604,7.76583867,10.39454007,9.629657873,3.303534583,6,1,8
6,0,1,0,1,1,9,1927.366351,1998.711604,7.76583867,10.39454007,9.629657873,3.303534583,6,1,8.
.366351,1998.711604,7.76583867,10.39454007,9.629657873,3.303534583,6,1,8.960583081,3.4648
6,0,1,0,1,1,9,1927.366351,1998.711604,7.76583867,10.39454007,9.629657873,3.303534583,6,1,8.
745117,3,2,208,66,0,1,0,1,1,9,1927.366351,1998.711604,7.76583867,10.39454007,9.629657873,3.3
,7.76583867,10.39454007,9.629657873,3.303534583,6,1,8.960583081,3.46484669,318.582845,54.
1,14.56934394,9.238650271,8.392871128,5,3,6.115987283,0,583.0990781,51.37429318,54.635039
1,1,0,0,1,1,7,1780.078908,1508.454241,6.727586011,14.56934394,9.238650271,8.392871128,5,3,6
.078908,1508.454241,6.727586011,14.56934394,9.238650271,8.392871128,5,3,6.115987283,0,583
80.078908,1508.454241,6.727586011,14.56934394,9.238650271,8.392871128,5,3,6.115987283,0,5
.078908,1508.454241,6.727586011,14.56934394,9.238650271,8.392871128,5,3,6.115987283,0,583
.078908,1508.454241,6.727586011,14.56934394,9.238650271,8.392871128,5,3,6.115987283,0,583
.078908,1508.454241,6.727586011,14.56934394,9.238650271,8.392871128,5,3,6.115987283,0,583
0.078908,1508.454241,6.727586011,14.56934394,9.238650271,8.392871128,5,3,6.115987283,0,58
11,14.56934394,9.238650271,8.392871128,5,3,6.115987283,0,583.0990781,51.37429318,54.63503
.078908,1508.454241,6.727586011,14.56934394,9.238650271,8.392871128,5,3,6.115987283,0,583
9227905,3,2,208,81,1,0,0,1,1,7,1780.078908,1508.454241,6.727586011,14.56934394,9.238650271,
0.078908,1508.454241,6.727586011,14.56934394,9.238650271,8.392871128,5,3,6.115987283,0,58
1,1,0,0,1,1,7,1780.078908,1508.454241,6.727586011,14.56934394,9.238650271,8.392871128,5,3,6
0.078908,1508.454241,6.727586011,14.56934394,9.238650271,8.392871128,5,3,6.115987283,0,58
0.078908,1508.454241,6.727586011,14.56934394,9.238650271,8.392871128,5,3,6.115987283,0,58
382324,3,2,208,81,1,0,0,1,1,7,1780.078908,1508.454241,6.727586011,14.56934394,9.238650271,8.
.078908,1508.454241,6.727586011,14.56934394,9.238650271,8.392871128,5,3,6.115987283,0,583
.078908,1508.454241,6.727586011,14.56934394,9.238650271,8.392871128,5,3,6.115987283,0,583
11,14.56934394,9.238650271,8.392871128,5,3,6.115987283,0,583.0990781,51.37429318,54.63503
1,1,0,0,1,1,7,1780.078908,1508.454241,6.727586011,14.56934394,9.238650271,8.392871128,5,3,6
1,1,0,0,1,1,7,1780.078908,1508.454241,6.727586011,14.56934394,9.238650271,8.392871128,5,3,6
80.078908,1508.454241,6.727586011,14.56934394,9.238650271,8.392871128,5,3,6.115987283,0,5
1,1,0,0,1,1,7,1780.078908,1508.454241,6.727586011,14.56934394,9.238650271,8.392871128,5,3,6
1,1,0,0,1,1,7,1780.078908,1508.454241,6.727586011,14.56934394,9.238650271,8.392871128,5,3,6
1,1,0,0,1,1,7,1780.078908,1508.454241,6.727586011,14.56934394,9.238650271,8.392871128,5,3,6
0.078908,1508.454241,6.727586011,14.56934394,9.238650271,8.392871128,5,3,6.115987283,0,58
1,1,0,0,1,1,7,1780.078908,1508.454241,6.727586011,14.56934394,9.238650271,8.392871128,5,3,6
1,1,0,0,1,1,7,1780.078908,1508.454241,6.727586011,14.56934394,9.238650271,8.392871128,5,3,6
.078908,1508.454241,6.727586011,14.56934394,9.238650271,8.392871128,5,3,6.115987283,0,583
.078908,1508.454241,6.727586011,14.56934394,9.238650271,8.392871128,5,3,6.115987283,0,583
,1,0,0,1,1,7,1780.078908,1508.454241,6.727586011,14.56934394,9.238650271,8.392871128,5,3,6.
1,1,0,0,1,1,7,1780.078908,1508.454241,6.727586011,14.56934394,9.238650271,8.392871128,5,3,6
0.078908,1508.454241,6.727586011,14.56934394,9.238650271,8.392871128,5,3,6.115987283,0,58

.078908,1508.454241,6.727586011,14.56934394,9.238650271,8.392871128,5,3,6.115987283,0,583
0.078908,1508.454241,6.727586011,14.56934394,9.238650271,8.392871128,5,3,6.115987283,0,58
2,8.187359634,9.238650271,8.14418704,5,2,6.452168528,0,642.9522589,60.22023959,40.936798
69076142,8.187359634,9.238650271,8.14418704,5,2,6.452168528,0,642.9522589,60.22023959,40.
51.96764,963.7080832,7.169076142,8.187359634,9.238650271,8.14418704,5,2,6.452168528,0,642
42,8.187359634,9.238650271,8.14418704,5,2,6.452168528,0,642.9522589,60.22023959,40.936798
73,1,1,0,1,1,11,1751.96764,963.7080832,7.169076142,8.187359634,9.238650271,8.14418704,5,2,6
169076142,8.187359634,9.238650271,8.14418704,5,2,6.452168528,0,642.9522589,60.22023959,40
3,1,1,0,1,1,11,1751.96764,963.7080832,7.169076142,8.187359634,9.238650271,8.14418704,5,2,6.
3,1,1,0,1,1,11,1751.96764,963.7080832,7.169076142,8.187359634,9.238650271,8.14418704,5,2,6.
3,1,1,0,1,1,11,1751.96764,963.7080832,7.169076142,8.187359634,9.238650271,8.14418704,5,2,6.
751.96764,963.7080832,7.169076142,8.187359634,9.238650271,8.14418704,5,2,6.452168528,0,64
42,8.187359634,9.238650271,8.14418704,5,2,6.452168528,0,642.9522589,60.22023959,40.936798
751.96764,963.7080832,7.169076142,8.187359634,9.238650271,8.14418704,5,2,6.452168528,0,64
1.96764,963.7080832,7.169076142,8.187359634,9.238650271,8.14418704,5,2,6.452168528,0,642.
751.96764,963.7080832,7.169076142,8.187359634,9.238650271,8.14418704,5,2,6.452168528,0,64
1751.96764,963.7080832,7.169076142,8.187359634,9.238650271,8.14418704,5,2,6.452168528,0,6
01,8.467815326,9.238650271,8.14418704,5,2,6.910161121,0,696.1895841,58.35247169,29.63735
6.306832,932.8440704,7.677956801,8.467815326,9.238650271,8.14418704,5,2,6.910161121,0,696
01,8.467815326,9.238650271,8.14418704,5,2,6.910161121,0,696.1895841,58.35247169,29.637353
8,1,1,0,1,1,9,1736.306832,932.8440704,7.677956801,8.467815326,9.238650271,8.14418704,5,2,6.
01,8.467815326,9.238650271,8.14418704,5,2,6.910161121,0,696.1895841,58.35247169,29.637353
,1,1,0,1,1,9,1736.306832,932.8440704,7.677956801,8.467815326,9.238650271,8.14418704,5,2,6.9
6.306832,932.8440704,7.677956801,8.467815326,9.238650271,8.14418704,5,2,6.910161121,0,696
6.306832,932.8440704,7.677956801,8.467815326,9.238650271,8.14418704,5,2,6.910161121,0,696
6.306832,932.8440704,7.677956801,8.467815326,9.238650271,8.14418704,5,2,6.910161121,0,696
68,1,1,0,1,1,9,1736.306832,932.8440704,7.677956801,8.467815326,9.238650271,8.14418704,5,2,6
68,1,1,0,1,1,9,1736.306832,932.8440704,7.677956801,8.467815326,9.238650271,8.14418704,5,2,6
01,8.467815326,9.238650271,8.14418704,5,2,6.910161121,0,696.1895841,58.35247169,29.637353
.306832,932.8440704,7.677956801,8.467815326,9.238650271,8.14418704,5,2,6.910161121,0,696.
.306832,932.8440704,7.677956801,8.467815326,9.238650271,8.14418704,5,2,6.910161121,0,696.
.306832,932.8440704,7.677956801,8.467815326,9.238650271,8.14418704,5,2,6.910161121,0,696.
382324,3,2,208,68,1,1,0,1,1,9,1736.306832,932.8440704,7.677956801,8.467815326,9.238650271,8.
.306832,932.8440704,7.677956801,8.467815326,9.238650271,8.14418704,5,2,6.910161121,0,696.
.306832,932.8440704,7.677956801,8.467815326,9.238650271,8.14418704,5,2,6.910161121,0,696.
6.306832,932.8440704,7.677956801,8.467815326,9.238650271,8.14418704,5,2,6.910161121,0,696
01,8.467815326,9.238650271,8.14418704,5,2,6.910161121,0,696.1895841,58.35247169,29.637353
.306832,932.8440704,7.677956801,8.467815326,9.238650271,8.14418704,5,2,6.910161121,0,696.
8,1,1,0,1,1,9,1736.306832,932.8440704,7.677956801,8.467815326,9.238650271,8.14418704,5,2,6.
6.306832,932.8440704,7.677956801,8.467815326,9.238650271,8.14418704,5,2,6.910161121,0,696
6.306832,932.8440704,7.677956801,8.467815326,9.238650271,8.14418704,5,2,6.910161121,0,696
6.306832,932.8440704,7.677956801,8.467815326,9.238650271,8.14418704,5,2,6.910161121,0,696
.306832,932.8440704,7.677956801,8.467815326,9.238650271,8.14418704,5,2,6.910161121,0,696.
8,1,1,0,1,1,9,1736.306832,932.8440704,7.677956801,8.467815326,9.238650271,8.14418704,5,2,6.
6.306832,932.8440704,7.677956801,8.467815326,9.238650271,8.14418704,5,2,6.910161121,0,696
6.306832,932.8440704,7.677956801,8.467815326,9.238650271,8.14418704,5,2,6.910161121,0,696
01,8.467815326,9.238650271,8.14418704,5,2,6.910161121,0,696.1895841,58.35247169,29.637353
8,1,1,0,1,1,9,1736.306832,932.8440704,7.677956801,8.467815326,9.238650271,8.14418704,5,2,6.
.306832,932.8440704,7.677956801,8.467815326,9.238650271,8.14418704,5,2,6.910161121,0,696.
6.306832,932.8440704,7.677956801,8.467815326,9.238650271,8.14418704,5,2,6.910161121,0,696

6.306832,932.8440704,7.677956801,8.467815326,9.238650271,8.14418704,5,2,6.910161121,0,696
.306832,932.8440704,7.677956801,8.467815326,9.238650271,8.14418704,5,2,6.910161121,0,696.
6.306832,932.8440704,7.677956801,8.467815326,9.238650271,8.14418704,5,2,6.910161121,0,696
6.306832,932.8440704,7.677956801,8.467815326,9.238650271,8.14418704,5,2,6.910161121,0,696
6.306832,932.8440704,7.677956801,8.467815326,9.238650271,8.14418704,5,2,6.910161121,0,696
6.306832,932.8440704,7.677956801,8.467815326,9.238650271,8.14418704,5,2,6.910161121,0,696
.306832,932.8440704,7.677956801,8.467815326,9.238650271,8.14418704,5,2,6.910161121,0,696.
9,12.1091724,8.14418704,4.840652457,2,1,7.814206185,0,621.8115244,52.46681296,133.2008964,
.5752385,682.1134234,2.232630339,12.1091724,8.14418704,4.840652457,2,1,7.814206185,0,621.8
39,12.1091724,8.14418704,4.840652457,2,1,7.814206185,0,621.8115244,52.46681296,133.200896
.5752385,682.1134234,2.232630339,12.1091724,8.14418704,4.840652457,2,1,7.814206185,0,621.8
0.5752385,682.1134234,2.232630339,12.1091724,8.14418704,4.840652457,2,1,7.814206185,0,621
0,1,1,0,1,1,7,980.5752385,682.1134234,2.232630339,12.1091724,8.14418704,4.840652457,2,1,7.81
.5752385,682.1134234,2.232630339,12.1091724,8.14418704,4.840652457,2,1,7.814206185,0,621.8
.5752385,682.1134234,2.232630339,12.1091724,8.14418704,4.840652457,2,1,7.814206185,0,621.8
5752385,682.1134234,2.232630339,12.1091724,8.14418704,4.840652457,2,1,7.814206185,0,621.81
39,12.1091724,8.14418704,4.840652457,2,1,7.814206185,0,621.8115244,52.46681296,133.200896
752385,682.1134234,2.232630339,12.1091724,8.14418704,4.840652457,2,1,7.814206185,0,621.81
.5752385,682.1134234,2.232630339,12.1091724,8.14418704,4.840652457,2,1,7.814206185,0,621.8
.5752385,682.1134234,2.232630339,12.1091724,8.14418704,4.840652457,2,1,7.814206185,0,621.8
0,1,1,0,1,1,7,980.5752385,682.1134234,2.232630339,12.1091724,8.14418704,4.840652457,2,1,7.81
70,1,1,0,1,1,7,980.5752385,682.1134234,2.232630339,12.1091724,8.14418704,4.840652457,2,1,7.8
0.5752385,682.1134234,2.232630339,12.1091724,8.14418704,4.840652457,2,1,7.814206185,0,621
.5752385,682.1134234,2.232630339,12.1091724,8.14418704,4.840652457,2,1,7.814206185,0,621.8
.5752385,682.1134234,2.232630339,12.1091724,8.14418704,4.840652457,2,1,7.814206185,0,621.8
.5752385,682.1134234,2.232630339,12.1091724,8.14418704,4.840652457,2,1,7.814206185,0,621.8
0,1,1,0,1,1,7,980.5752385,682.1134234,2.232630339,12.1091724,8.14418704,4.840652457,2,1,7.81
39,12.1091724,8.14418704,4.840652457,2,1,7.814206185,0,621.8115244,52.46681296,133.200896
0,1,1,0,1,1,7,980.5752385,682.1134234,2.232630339,12.1091724,8.14418704,4.840652457,2,1,7.81
.5752385,682.1134234,2.232630339,12.1091724,8.14418704,4.840652457,2,1,7.814206185,0,621.8
0,1,1,0,1,1,7,980.5752385,682.1134234,2.232630339,12.1091724,8.14418704,4.840652457,2,1,7.81
5752385,682.1134234,2.232630339,12.1091724,8.14418704,4.840652457,2,1,7.814206185,0,621.81
.5752385,682.1134234,2.232630339,12.1091724,8.14418704,4.840652457,2,1,7.814206185,0,621.8
5752385,682.1134234,2.232630339,12.1091724,8.14418704,4.840652457,2,1,7.814206185,0,621.81
5752385,682.1134234,2.232630339,12.1091724,8.14418704,4.840652457,2,1,7.814206185,0,621.81
5752385,682.1134234,2.232630339,12.1091724,8.14418704,4.840652457,2,1,7.814206185,0,621.81
.5752385,682.1134234,2.232630339,12.1091724,8.14418704,4.840652457,2,1,7.814206185,0,621.8
517676,3,2,208,70,1,1,0,1,1,7,980.5752385,682.1134234,2.232630339,12.1091724,8.14418704,4.84
.5752385,682.1134234,2.232630339,12.1091724,8.14418704,4.840652457,2,1,7.814206185,0,621.8
5752385,682.1134234,2.232630339,12.1091724,8.14418704,4.840652457,2,1,7.814206185,0,621.81
0,1,1,0,1,1,7,980.5752385,682.1134234,2.232630339,12.1091724,8.14418704,4.840652457,2,1,7.81
.5752385,682.1134234,2.232630339,12.1091724,8.14418704,4.840652457,2,1,7.814206185,0,621.8
20996,3,2,208,76,0,1,0,1,1,9,1907.586859,2188.868396,7.815693215,20.79503493,9.480286879,0.8
7.586859,2188.868396,7.815693215,20.79503493,9.480286879,0.845779144,7,2,7.257429414,6.93
76,0,1,0,1,1,9,1907.586859,2188.868396,7.815693215,20.79503493,9.480286879,0.845779144,7,2,
6,0,1,0,1,1,9,1907.586859,2188.868396,7.815693215,20.79503493,9.480286879,0.845779144,7,2,7
76,0,1,0,1,1,9,1907.586859,2188.868396,7.815693215,20.79503493,9.480286879,0.845779144,7,2,
96,7.815693215,20.79503493,9.480286879,0.845779144,7,2,7.257429414,6.93167831,223.3726584
1907.586859,2188.868396,7.815693215,20.79503493,9.480286879,0.845779144,7,2,7.257429414,6
5693215,20.79503493,9.480286879,0.845779144,7,2,7.257429414,6.93167831,223.3726584,61.967

1907.586859,2188.868396,7.815693215,20.79503493,9.480286879,0.845779144,7,2,7.257429414,6
6,0,1,0,1,1,9,1907.586859,2188.868396,7.815693215,20.79503493,9.480286879,0.845779144,7,2,7
.586859,2188.868396,7.815693215,20.79503493,9.480286879,0.845779144,7,2,7.257429414,6.931
07.586859,2188.868396,7.815693215,20.79503493,9.480286879,0.845779144,7,2,7.257429414,6.9
15,20.79503493,9.480286879,0.845779144,7,2,7.257429414,6.93167831,223.3726584,61.96728192
5,1,1,1,1,1,5,1891.832834,2156.541812,7.705414444,12.38558837,9.363325868,3.303534583,6,1,7
.832834,2156.541812,7.705414444,12.38558837,9.363325868,3.303534583,6,1,7.112690256,3.096
91.832834,2156.541812,7.705414444,12.38558837,9.363325868,3.303534583,6,1,7.112690256,3.0
91.832834,2156.541812,7.705414444,12.38558837,9.363325868,3.303534583,6,1,7.112690256,3.0
1.832834,2156.541812,7.705414444,12.38558837,9.363325868,3.303534583,6,1,7.112690256,3.09
91.832834,2156.541812,7.705414444,12.38558837,9.363325868,3.303534583,6,1,7.112690256,3.0
1.832834,2156.541812,7.705414444,12.38558837,9.363325868,3.303534583,6,1,7.112690256,3.09
5,1,1,1,1,1,5,1891.832834,2156.541812,7.705414444,12.38558837,9.363325868,3.303534583,6,1,7
85,1,1,1,1,1,5,1891.832834,2156.541812,7.705414444,12.38558837,9.363325868,3.303534583,6,1,
.832834,2156.541812,7.705414444,12.38558837,9.363325868,3.303534583,6,1,7.112690256,3.096
9382324,3,2,208,85,1,1,1,1,1,5,1891.832834,2156.541812,7.705414444,12.38558837,9.363325868,3
91.832834,2156.541812,7.705414444,12.38558837,9.363325868,3.303534583,6,1,7.112690256,3.0
.832834,2156.541812,7.705414444,12.38558837,9.363325868,3.303534583,6,1,7.112690256,3.096
.832834,2156.541812,7.705414444,12.38558837,9.363325868,3.303534583,6,1,7.112690256,3.096
,1,1,1,1,1,5,1891.832834,2156.541812,7.705414444,12.38558837,9.363325868,3.303534583,6,1,7.1
44,12.38558837,9.363325868,3.303534583,6,1,7.112690256,3.096397093,400.6875765,53.9379011
91.832834,2156.541812,7.705414444,12.38558837,9.363325868,3.303534583,6,1,7.112690256,3.0
1.832834,2156.541812,7.705414444,12.38558837,9.363325868,3.303534583,6,1,7.112690256,3.09
85,1,1,1,1,1,5,1891.832834,2156.541812,7.705414444,12.38558837,9.363325868,3.303534583,6,1,
8,85,1,1,1,1,1,5,1891.832834,2156.541812,7.705414444,12.38558837,9.363325868,3.303534583,6,1
1.832834,2156.541812,7.705414444,12.38558837,9.363325868,3.303534583,6,1,7.112690256,3.09
85,1,1,1,1,1,5,1891.832834,2156.541812,7.705414444,12.38558837,9.363325868,3.303534583,6,1,
85,1,1,1,1,1,5,1891.832834,2156.541812,7.705414444,12.38558837,9.363325868,3.303534583,6,1,
1.832834,2156.541812,7.705414444,12.38558837,9.363325868,3.303534583,6,1,7.112690256,3.09
37,8.770435381,9.363325868,3.303534583,6,1,6.97283588,0,426.0545012,51.71519944,64.316526
9,1,1,0,1,1,9,1861.339337,2023.637127,7.553905537,8.770435381,9.363325868,3.303534583,6,1,6
37,8.770435381,9.363325868,3.303534583,6,1,6.97283588,0,426.0545012,51.71519944,64.316526
9,1,1,0,1,1,9,1861.339337,2023.637127,7.553905537,8.770435381,9.363325868,3.303534583,6,1,6
.339337,2023.637127,7.553905537,8.770435381,9.363325868,3.303534583,6,1,6.97283588,0,426.0
382324,3,2,208,69,1,1,0,1,1,9,1861.339337,2023.637127,7.553905537,8.770435381,9.363325868,3.
9,1,1,0,1,1,9,1861.339337,2023.637127,7.553905537,8.770435381,9.363325868,3.303534583,6,1,6
61.339337,2023.637127,7.553905537,8.770435381,9.363325868,3.303534583,6,1,6.97283588,0,42
9,1,1,0,1,1,9,1861.339337,2023.637127,7.553905537,8.770435381,9.363325868,3.303534583,6,1,6
37,8.770435381,9.363325868,3.303534583,6,1,6.97283588,0,426.0545012,51.71519944,64.316526
1.339337,2023.637127,7.553905537,8.770435381,9.363325868,3.303534583,6,1,6.97283588,0,426
.339337,2023.637127,7.553905537,8.770435381,9.363325868,3.303534583,6,1,6.97283588,0,426.0
1.339337,2023.637127,7.553905537,8.770435381,9.363325868,3.303534583,6,1,6.97283588,0,426
1.339337,2023.637127,7.553905537,8.770435381,9.363325868,3.303534583,6,1,6.97283588,0,426
1.339337,2023.637127,7.553905537,8.770435381,9.363325868,3.303534583,6,1,6.97283588,0,426
69,1,1,0,1,1,9,1861.339337,2023.637127,7.553905537,8.770435381,9.363325868,3.303534583,6,1,
61.339337,2023.637127,7.553905537,8.770435381,9.363325868,3.303534583,6,1,6.97283588,0,42
1.339337,2023.637127,7.553905537,8.770435381,9.363325868,3.303534583,6,1,6.97283588,0,426
1.339337,2023.637127,7.553905537,8.770435381,9.363325868,3.303534583,6,1,6.97283588,0,426
61.339337,2023.637127,7.553905537,8.770435381,9.363325868,3.303534583,6,1,6.97283588,0,42
0308838,3,2,208,69,1,1,0,1,1,9,1861.339337,2023.637127,7.553905537,8.770435381,9.363325868,3

.339337,2023.637127,7.553905537,8.770435381,9.363325868,3.303534583,6,1,6.97283588,0,426.
9,1,1,0,1,1,9,1861.339337,2023.637127,7.553905537,8.770435381,9.363325868,3.303534583,6,1,6
61.339337,2023.637127,7.553905537,8.770435381,9.363325868,3.303534583,6,1,6.97283588,0,42
1.339337,2023.637127,7.553905537,8.770435381,9.363325868,3.303534583,6,1,6.97283588,0,426
.339337,2023.637127,7.553905537,8.770435381,9.363325868,3.303534583,6,1,6.97283588,0,426.
61.339337,2023.637127,7.553905537,8.770435381,9.363325868,3.303534583,6,1,6.97283588,0,42
37,8.770435381,9.363325868,3.303534583,6,1,6.97283588,0,426.0545012,51.71519944,64.316526
.339337,2023.637127,7.553905537,8.770435381,9.363325868,3.303534583,6,1,6.97283588,0,426.
.339337,2023.637127,7.553905537,8.770435381,9.363325868,3.303534583,6,1,6.97283588,0,426.
.339337,2023.637127,7.553905537,8.770435381,9.363325868,3.303534583,6,1,6.97283588,0,426.
.339337,2023.637127,7.553905537,8.770435381,9.363325868,3.303534583,6,1,6.97283588,0,426.
.339337,2023.637127,7.553905537,8.770435381,9.363325868,3.303534583,6,1,6.97283588,0,426.
1.339337,2023.637127,7.553905537,8.770435381,9.363325868,3.303534583,6,1,6.97283588,0,426
9691162,3,2,208,69,1,1,0,1,1,9,1861.339337,2023.637127,7.553905537,8.770435381,9.363325868,
0617676,3,2,208,68,0,1,0,1,1,9,1692.707591,1190.550145,8.30638742,8.365285804,9.238650271,4.
2.707591,1190.550145,8.30638742,8.365285804,9.238650271,4.840652457,5,1,7.383455485,0,689
8,0,1,0,1,1,9,1692.707591,1190.550145,8.30638742,8.365285804,9.238650271,4.840652457,5,1,7.
2.707591,1190.550145,8.30638742,8.365285804,9.238650271,4.840652457,5,1,7.383455485,0,689
592.707591,1190.550145,8.30638742,8.365285804,9.238650271,4.840652457,5,1,7.383455485,0,6
2.707591,1190.550145,8.30638742,8.365285804,9.238650271,4.840652457,5,1,7.383455485,0,689
8,0,1,0,1,1,9,1692.707591,1190.550145,8.30638742,8.365285804,9.238650271,4.840652457,5,1,7.
1692.707591,1190.550145,8.30638742,8.365285804,9.238650271,4.840652457,5,1,7.383455485,0,
0638742,8.365285804,9.238650271,4.840652457,5,1,7.383455485,0,689.4250427,61.83643968,50.
382324,3,2,208,68,0,1,0,1,1,9,1692.707591,1190.550145,8.30638742,8.365285804,9.238650271,4.8
5,11.48140328,9.238650271,4.840652457,5,1,6.58130697,0,753.4117283,63.61930071,57.4070164
2.103134,1212.737065,9.871960455,11.48140328,9.238650271,4.840652457,5,1,6.58130697,0,753
4,1,1,0,1,1,8,1622.103134,1212.737065,9.871960455,11.48140328,9.238650271,4.840652457,5,1,6
2.103134,1212.737065,9.871960455,11.48140328,9.238650271,4.840652457,5,1,6.58130697,0,753
55,11.48140328,9.238650271,4.840652457,5,1,6.58130697,0,753.4117283,63.61930071,57.407016
.103134,1212.737065,9.871960455,11.48140328,9.238650271,4.840652457,5,1,6.58130697,0,753.
.103134,1212.737065,9.871960455,11.48140328,9.238650271,4.840652457,5,1,6.58130697,0,753.
.103134,1212.737065,9.871960455,11.48140328,9.238650271,4.840652457,5,1,6.58130697,0,753.
2.103134,1212.737065,9.871960455,11.48140328,9.238650271,4.840652457,5,1,6.58130697,0,753
22.103134,1212.737065,9.871960455,11.48140328,9.238650271,4.840652457,5,1,6.58130697,0,75
,1,1,0,1,1,8,1622.103134,1212.737065,9.871960455,11.48140328,9.238650271,4.840652457,5,1,6.
55,11.48140328,9.238650271,4.840652457,5,1,6.58130697,0,753.4117283,63.61930071,57.407016
55,11.48140328,9.238650271,4.840652457,5,1,6.58130697,0,753.4117283,63.61930071,57.407016
55,11.48140328,9.238650271,4.840652457,5,1,6.58130697,0,753.4117283,63.61930071,57.407016
2.103134,1212.737065,9.871960455,11.48140328,9.238650271,4.840652457,5,1,6.58130697,0,753
517676,3,2,208,74,1,1,0,1,1,8,1622.103134,1212.737065,9.871960455,11.48140328,9.238650271,4.
4,1,1,0,1,1,8,1622.103134,1212.737065,9.871960455,11.48140328,9.238650271,4.840652457,5,1,6
.103134,1212.737065,9.871960455,11.48140328,9.238650271,4.840652457,5,1,6.58130697,0,753.
4,1,1,0,1,1,8,1622.103134,1212.737065,9.871960455,11.48140328,9.238650271,4.840652457,5,1,6
1.48140328,9.238650271,4.840652457,5,1,6.58130697,0,753.4117283,63.61930071,57.40701641,1
517676,3,2,208,74,1,1,0,1,1,8,1622.103134,1212.737065,9.871960455,11.48140328,9.238650271,4.
55,11.48140328,9.238650271,4.840652457,5,1,6.58130697,0,753.4117283,63.61930071,57.407016
4,1,1,0,1,1,8,1622.103134,1212.737065,9.871960455,11.48140328,9.238650271,4.840652457,5,1,6
2.103134,1212.737065,9.871960455,11.48140328,9.238650271,4.840652457,5,1,6.58130697,0,753
4,1,1,0,1,1,8,1622.103134,1212.737065,9.871960455,11.48140328,9.238650271,4.840652457,5,1,6
517676,3,2,208,74,1,1,0,1,1,8,1622.103134,1212.737065,9.871960455,11.48140328,9.238650271,4.

2.103134,1212.737065,9.871960455,11.48140328,9.238650271,4.840652457,5,1,6.58130697,0,753
.103134,1212.737065,9.871960455,11.48140328,9.238650271,4.840652457,5,1,6.58130697,0,753.4
2.103134,1212.737065,9.871960455,11.48140328,9.238650271,4.840652457,5,1,6.58130697,0,753
.103134,1212.737065,9.871960455,11.48140328,9.238650271,4.840652457,5,1,6.58130697,0,753.4
382324,3,2,208,74,1,1,0,1,1,8,1622.103134,1212.737065,9.871960455,11.48140328,9.238650271,4.
2.103134,1212.737065,9.871960455,11.48140328,9.238650271,4.840652457,5,1,6.58130697,0,753
2.103134,1212.737065,9.871960455,11.48140328,9.238650271,4.840652457,5,1,6.58130697,0,753
2.103134,1212.737065,9.871960455,11.48140328,9.238650271,4.840652457,5,1,6.58130697,0,753
103134,1212.737065,9.871960455,11.48140328,9.238650271,4.840652457,5,1,6.58130697,0,753.41
29.955856,1212.392101,8.951293992,10.94451335,8.69695926,4.840652457,4,1,7.832382243,0,77
51293992,10.94451335,8.69695926,4.840652457,4,1,7.832382243,0,775.7688647,62.65905794,54.
7,0,1,0,1,1,9,1629.955856,1212.392101,8.951293992,10.94451335,8.69695926,4.840652457,4,1,7.8
9.955856,1212.392101,8.951293992,10.94451335,8.69695926,4.840652457,4,1,7.832382243,0,775
29.955856,1212.392101,8.951293992,10.94451335,8.69695926,4.840652457,4,1,7.832382243,0,77
93992,10.94451335,8.69695926,4.840652457,4,1,7.832382243,0,775.7688647,62.65905794,54.7225
7,0,1,0,1,1,9,1629.955856,1212.392101,8.951293992,10.94451335,8.69695926,4.840652457,4,1,7.8
.955856,1212.392101,8.951293992,10.94451335,8.69695926,4.840652457,4,1,7.832382243,0,775.7
9.955856,1212.392101,8.951293992,10.94451335,8.69695926,4.840652457,4,1,7.832382243,0,775
93992,10.94451335,8.69695926,4.840652457,4,1,7.832382243,0,775.7688647,62.65905794,54.7225
93992,10.94451335,8.69695926,4.840652457,4,1,7.832382243,0,775.7688647,62.65905794,54.7225
9.955856,1212.392101,8.951293992,10.94451335,8.69695926,4.840652457,4,1,7.832382243,0,775
7,0,1,0,1,1,9,1629.955856,1212.392101,8.951293992,10.94451335,8.69695926,4.840652457,4,1,7.8
77,0,1,0,1,1,9,1629.955856,1212.392101,8.951293992,10.94451335,8.69695926,4.840652457,4,1,7
93992,10.94451335,8.69695926,4.840652457,4,1,7.832382243,0,775.7688647,62.65905794,54.7225
93992,10.94451335,8.69695926,4.840652457,4,1,7.832382243,0,775.7688647,62.65905794,54.7225
92,10.94451335,8.69695926,4.840652457,4,1,7.832382243,0,775.7688647,62.65905794,54.7225667
1293992,10.94451335,8.69695926,4.840652457,4,1,7.832382243,0,775.7688647,62.65905794,54.72
9.955856,1212.392101,8.951293992,10.94451335,8.69695926,4.840652457,4,1,7.832382243,0,775
7,0,1,0,1,1,9,1629.955856,1212.392101,8.951293992,10.94451335,8.69695926,4.840652457,4,1,7.8
93992,10.94451335,8.69695926,4.840652457,4,1,7.832382243,0,775.7688647,62.65905794,54.7225
.955856,1212.392101,8.951293992,10.94451335,8.69695926,4.840652457,4,1,7.832382243,0,775.7
92,10.94451335,8.69695926,4.840652457,4,1,7.832382243,0,775.7688647,62.65905794,54.7225667
51293992,10.94451335,8.69695926,4.840652457,4,1,7.832382243,0,775.7688647,62.65905794,54.
.955856,1212.392101,8.951293992,10.94451335,8.69695926,4.840652457,4,1,7.832382243,0,775.7
93992,10.94451335,8.69695926,4.840652457,4,1,7.832382243,0,775.7688647,62.65905794,54.7225
9.955856,1212.392101,8.951293992,10.94451335,8.69695926,4.840652457,4,1,7.832382243,0,775
.955856,1212.392101,8.951293992,10.94451335,8.69695926,4.840652457,4,1,7.832382243,0,775.7
93992,10.94451335,8.69695926,4.840652457,4,1,7.832382243,0,775.7688647,62.65905794,54.7225
77,0,1,0,1,1,9,1629.955856,1212.392101,8.951293992,10.94451335,8.69695926,4.840652457,4,1,7
93992,10.94451335,8.69695926,4.840652457,4,1,7.832382243,0,775.7688647,62.65905794,54.7225
9.955856,1212.392101,8.951293992,10.94451335,8.69695926,4.840652457,4,1,7.832382243,0,775
92,10.94451335,8.69695926,4.840652457,4,1,7.832382243,0,775.7688647,62.65905794,54.7225667
51293992,10.94451335,8.69695926,4.840652457,4,1,7.832382243,0,775.7688647,62.65905794,54.
9.955856,1212.392101,8.951293992,10.94451335,8.69695926,4.840652457,4,1,7.832382243,0,775
.955856,1212.392101,8.951293992,10.94451335,8.69695926,4.840652457,4,1,7.832382243,0,775.7
801512,10.16167518,8.69695926,4.840652457,4,1,8.095326323,0,816.0582008,64.76261058,40.64
71,1,1,0,1,1,9,1480.891505,1147.10447,9.251801512,10.16167518,8.69695926,4.840652457,4,1,8.0
.891505,1147.10447,9.251801512,10.16167518,8.69695926,4.840652457,4,1,8.095326323,0,816.05
.891505,1147.10447,9.251801512,10.16167518,8.69695926,4.840652457,4,1,8.095326323,0,816.05
0617676,3,2,208,71,1,1,0,1,1,9,1480.891505,1147.10447,9.251801512,10.16167518,8.69695926,4.8

,1,1,0,1,1,9,1480.891505,1147.10447,9.251801512,10.16167518,8.69695926,4.840652457,4,1,8.09
1512,10.16167518,8.69695926,4.840652457,4,1,8.095326323,0,816.0582008,64.76261058,40.6467
0.891505,1147.10447,9.251801512,10.16167518,8.69695926,4.840652457,4,1,8.095326323,0,816.
0.891505,1147.10447,9.251801512,10.16167518,8.69695926,4.840652457,4,1,8.095326323,0,816.
1512,10.16167518,8.69695926,4.840652457,4,1,8.095326323,0,816.0582008,64.76261058,40.6467
0.891505,1147.10447,9.251801512,10.16167518,8.69695926,4.840652457,4,1,8.095326323,0,816.
1,1,1,0,1,1,9,1480.891505,1147.10447,9.251801512,10.16167518,8.69695926,4.840652457,4,1,8.0
71,1,1,0,1,1,9,1480.891505,1147.10447,9.251801512,10.16167518,8.69695926,4.840652457,4,1,8.
1,1,1,0,1,1,9,1480.891505,1147.10447,9.251801512,10.16167518,8.69695926,4.840652457,4,1,8.0
2,10.16167518,8.69695926,4.840652457,4,1,8.095326323,0,816.0582008,64.76261058,40.6467007
801512,10.16167518,8.69695926,4.840652457,4,1,8.095326323,0,816.0582008,64.76261058,40.64
0.891505,1147.10447,9.251801512,10.16167518,8.69695926,4.840652457,4,1,8.095326323,0,816.
1,1,1,0,1,1,9,1480.891505,1147.10447,9.251801512,10.16167518,8.69695926,4.840652457,4,1,8.0
.891505,1147.10447,9.251801512,10.16167518,8.69695926,4.840652457,4,1,8.095326323,0,816.0
9382324,3,2,208,71,1,1,0,1,1,9,1480.891505,1147.10447,9.251801512,10.16167518,8.69695926,4.8
,1,1,0,1,1,9,1480.891505,1147.10447,9.251801512,10.16167518,8.69695926,4.840652457,4,1,8.09
80.891505,1147.10447,9.251801512,10.16167518,8.69695926,4.840652457,4,1,8.095326323,0,816
0.891505,1147.10447,9.251801512,10.16167518,8.69695926,4.840652457,4,1,8.095326323,0,816.
2,10.16167518,8.69695926,4.840652457,4,1,8.095326323,0,816.0582008,64.76261058,40.6467007
80.891505,1147.10447,9.251801512,10.16167518,8.69695926,4.840652457,4,1,8.095326323,0,816
2,10.16167518,8.69695926,4.840652457,4,1,8.095326323,0,816.0582008,64.76261058,40.6467007
.891505,1147.10447,9.251801512,10.16167518,8.69695926,4.840652457,4,1,8.095326323,0,816.0
0.891505,1147.10447,9.251801512,10.16167518,8.69695926,4.840652457,4,1,8.095326323,0,816.
80.891505,1147.10447,9.251801512,10.16167518,8.69695926,4.840652457,4,1,8.095326323,0,816
10.16167518,8.69695926,4.840652457,4,1,8.095326323,0,816.0582008,64.76261058,40.64670073,
0.891505,1147.10447,9.251801512,10.16167518,8.69695926,4.840652457,4,1,8.095326323,0,816.
2,10.16167518,8.69695926,4.840652457,4,1,8.095326323,0,816.0582008,64.76261058,40.6467007
71,1,1,0,1,1,9,1480.891505,1147.10447,9.251801512,10.16167518,8.69695926,4.840652457,4,1,8.
10.16167518,8.69695926,4.840652457,4,1,8.095326323,0,816.0582008,64.76261058,40.64670073,
0.891505,1147.10447,9.251801512,10.16167518,8.69695926,4.840652457,4,1,8.095326323,0,816.
0.891505,1147.10447,9.251801512,10.16167518,8.69695926,4.840652457,4,1,8.095326323,0,816.
0.891505,1147.10447,9.251801512,10.16167518,8.69695926,4.840652457,4,1,8.095326323,0,816.
0.891505,1147.10447,9.251801512,10.16167518,8.69695926,4.840652457,4,1,8.095326323,0,816.
71,1,1,0,1,1,9,1480.891505,1147.10447,9.251801512,10.16167518,8.69695926,4.840652457,4,1,8.
0617676,3,2,208,75,1,1,0,1,1,12,1689.442246,1177.284027,7.598358616,6.21949779,9.238650271,
516,6.21949779,9.238650271,4.840652457,5,1,7.598358616,0,654.9142338,60.78686892,49.75598
598358616,6.21949779,9.238650271,4.840652457,5,1,7.598358616,0,654.9142338,60.78686892,49
689.442246,1177.284027,7.598358616,6.21949779,9.238650271,4.840652457,5,1,7.598358616,0,6
598358616,6.21949779,9.238650271,4.840652457,5,1,7.598358616,0,654.9142338,60.78686892,4
9.442246,1177.284027,7.598358616,6.21949779,9.238650271,4.840652457,5,1,7.598358616,0,654
75,1,1,0,1,1,12,1689.442246,1177.284027,7.598358616,6.21949779,9.238650271,4.840652457,5,1,
1689.442246,1177.284027,7.598358616,6.21949779,9.238650271,4.840652457,5,1,7.598358616,0,
89.442246,1177.284027,7.598358616,6.21949779,9.238650271,4.840652457,5,1,7.598358616,0,65
89.442246,1177.284027,7.598358616,6.21949779,9.238650271,4.840652457,5,1,7.598358616,0,65
1689.442246,1177.284027,7.598358616,6.21949779,9.238650271,4.840652457,5,1,7.598358616,0,
9.442246,1177.284027,7.598358616,6.21949779,9.238650271,4.840652457,5,1,7.598358616,0,654
689.442246,1177.284027,7.598358616,6.21949779,9.238650271,4.840652457,5,1,7.598358616,0,6
89.442246,1177.284027,7.598358616,6.21949779,9.238650271,4.840652457,5,1,7.598358616,0,65
89.442246,1177.284027,7.598358616,6.21949779,9.238650271,4.840652457,5,1,7.598358616,0,65
98358616,6.21949779,9.238650271,4.840652457,5,1,7.598358616,0,654.9142338,60.78686892,49.

16,6.21949779,9.238650271,4.840652457,5,1,7.598358616,0,654.9142338,60.78686892,49.7559823
517676,3,2,208,75,1,1,0,1,1,12,1689.442246,1177.284027,7.598358616,6.21949779,9.238650271,4.
89.442246,1177.284027,7.598358616,6.21949779,9.238650271,4.840652457,5,1,7.598358616,0,65
9.442246,1177.284027,7.598358616,6.21949779,9.238650271,4.840652457,5,1,7.598358616,0,654
516,6.21949779,9.238650271,4.840652457,5,1,7.598358616,0,654.9142338,60.78686892,49.755982
16,6.21949779,9.238650271,4.840652457,5,1,7.598358616,0,654.9142338,60.78686892,49.7559823
89.442246,1177.284027,7.598358616,6.21949779,9.238650271,4.840652457,5,1,7.598358616,0,65
5,1,1,0,1,1,12,1689.442246,1177.284027,7.598358616,6.21949779,9.238650271,4.840652457,5,1,7
358616,6.21949779,9.238650271,4.840652457,5,1,7.598358616,0,654.9142338,60.78686892,49.755
16,6.21949779,9.238650271,4.840652457,5,1,7.598358616,0,654.9142338,60.78686892,49.7559823
89.442246,1177.284027,7.598358616,6.21949779,9.238650271,4.840652457,5,1,7.598358616,0,65
9.442246,1177.284027,7.598358616,6.21949779,9.238650271,4.840652457,5,1,7.598358616,0,654
9.442246,1177.284027,7.598358616,6.21949779,9.238650271,4.840652457,5,1,7.598358616,0,654
16,6.21949779,9.238650271,4.840652457,5,1,7.598358616,0,654.9142338,60.78686892,49.7559823
75,1,1,0,1,1,12,1689.442246,1177.284027,7.598358616,6.21949779,9.238650271,4.840652457,5,1,
689.442246,1177.284027,7.598358616,6.21949779,9.238650271,4.840652457,5,1,7.598358616,0,6
689.442246,1177.284027,7.598358616,6.21949779,9.238650271,4.840652457,5,1,7.598358616,0,6
75,1,1,0,1,1,12,1689.442246,1177.284027,7.598358616,6.21949779,9.238650271,4.840652457,5,1,
89.442246,1177.284027,7.598358616,6.21949779,9.238650271,4.840652457,5,1,7.598358616,0,65
58616,6.21949779,9.238650271,4.840652457,5,1,7.598358616,0,654.9142338,60.78686892,49.7559
.442246,1177.284027,7.598358616,6.21949779,9.238650271,4.840652457,5,1,7.598358616,0,654.9
89.442246,1177.284027,7.598358616,6.21949779,9.238650271,4.840652457,5,1,7.598358616,0,65
58616,6.21949779,9.238650271,4.840652457,5,1,7.598358616,0,654.9142338,60.78686892,49.7559
8,0,1,0,1,1,9,1689.442246,1177.284027,7.598358616,6.21949779,9.238650271,4.840652457,5,1,7.5
58616,6.21949779,9.238650271,4.840652457,5,1,7.598358616,0,654.9142338,60.78686892,49.7559
16,6.21949779,9.238650271,4.840652457,5,1,7.598358616,0,654.9142338,60.78686892,49.7559823
.442246,1177.284027,7.598358616,6.21949779,9.238650271,4.840652457,5,1,7.598358616,0,654.9
58616,6.21949779,9.238650271,4.840652457,5,1,7.598358616,0,654.9142338,60.78686892,49.7559
98358616,6.21949779,9.238650271,4.840652457,5,1,7.598358616,0,654.9142338,60.78686892,49.
58616,6.21949779,9.238650271,4.840652457,5,1,7.598358616,0,654.9142338,60.78686892,49.7559
16,6.21949779,9.238650271,4.840652457,5,1,7.598358616,0,654.9142338,60.78686892,49.7559823
9.442246,1177.284027,7.598358616,6.21949779,9.238650271,4.840652457,5,1,7.598358616,0,654
78,0,1,0,1,1,9,1689.442246,1177.284027,7.598358616,6.21949779,9.238650271,4.840652457,5,1,7
.442246,1177.284027,7.598358616,6.21949779,9.238650271,4.840652457,5,1,7.598358616,0,654.9
7,7.598358616,6.21949779,9.238650271,4.840652457,5,1,7.598358616,0,654.9142338,60.7868689
.442246,1177.284027,7.598358616,6.21949779,9.238650271,4.840652457,5,1,7.598358616,0,654.9
16,6.21949779,9.238650271,4.840652457,5,1,7.598358616,0,654.9142338,60.78686892,49.7559823
.442246,1177.284027,7.598358616,6.21949779,9.238650271,4.840652457,5,1,7.598358616,0,654.9
89.442246,1177.284027,7.598358616,6.21949779,9.238650271,4.840652457,5,1,7.598358616,0,65
78,0,1,0,1,1,9,1689.442246,1177.284027,7.598358616,6.21949779,9.238650271,4.840652457,5,1,7
8,0,1,0,1,1,9,1689.442246,1177.284027,7.598358616,6.21949779,9.238650271,4.840652457,5,1,7.5
9.442246,1177.284027,7.598358616,6.21949779,9.238650271,4.840652457,5,1,7.598358616,0,654
89.442246,1177.284027,7.598358616,6.21949779,9.238650271,4.840652457,5,1,7.598358616,0,65
16,6.21949779,9.238650271,4.840652457,5,1,7.598358616,0,654.9142338,60.78686892,49.7559823
16,6.21949779,9.238650271,4.840652457,5,1,7.598358616,0,654.9142338,60.78686892,49.7559823
89.442246,1177.284027,7.598358616,6.21949779,9.238650271,4.840652457,5,1,7.598358616,0,65
78,0,1,0,1,1,9,1689.442246,1177.284027,7.598358616,6.21949779,9.238650271,4.840652457,5,1,7
.442246,1177.284027,7.598358616,6.21949779,9.238650271,4.840652457,5,1,7.598358616,0,654.9
89.442246,1177.284027,7.598358616,6.21949779,9.238650271,4.840652457,5,1,7.598358616,0,65
55,8.508504792,9.238650271,4.840652457,5,1,7.496295693,0,695.3278607,60.90740251,51.05102

9,0,1,1,1,1,9,1691.551281,1190.550145,8.433332655,8.508504792,9.238650271,4.840652457,5,1,7
9,0,1,1,1,1,9,1691.551281,1190.550145,8.433332655,8.508504792,9.238650271,4.840652457,5,1,7
69,0,1,1,1,1,9,1691.551281,1190.550145,8.433332655,8.508504792,9.238650271,4.840652457,5,1,
9,0,1,1,1,1,9,1691.551281,1190.550145,8.433332655,8.508504792,9.238650271,4.840652457,5,1,7
91.551281,1190.550145,8.433332655,8.508504792,9.238650271,4.840652457,5,1,7.496295693,0,6
5,8.508504792,9.238650271,4.840652457,5,1,7.496295693,0,695.3278607,60.90740251,51.05102
5,8.508504792,9.238650271,4.840652457,5,1,7.496295693,0,695.3278607,60.90740251,51.05102
2655,8.508504792,9.238650271,4.840652457,5,1,7.496295693,0,695.3278607,60.90740251,51.05
1.551281,1190.550145,8.433332655,8.508504792,9.238650271,4.840652457,5,1,7.496295693,0,69
9,0,1,1,1,1,9,1691.551281,1190.550145,8.433332655,8.508504792,9.238650271,4.840652457,5,1,7
1.551281,1190.550145,8.433332655,8.508504792,9.238650271,4.840652457,5,1,7.496295693,0,69
5,8.508504792,9.238650271,4.840652457,5,1,7.496295693,0,695.3278607,60.90740251,51.05102
1.551281,1190.550145,8.433332655,8.508504792,9.238650271,4.840652457,5,1,7.496295693,0,69
1691.551281,1190.550145,8.433332655,8.508504792,9.238650271,4.840652457,5,1,7.496295693,
5,8.508504792,9.238650271,4.840652457,5,1,7.496295693,0,695.3278607,60.90740251,51.05102
,0,1,1,1,1,9,1691.551281,1190.550145,8.433332655,8.508504792,9.238650271,4.840652457,5,1,7.
.551281,1190.550145,8.433332655,8.508504792,9.238650271,4.840652457,5,1,7.496295693,0,695
1.551281,1190.550145,8.433332655,8.508504792,9.238650271,4.840652457,5,1,7.496295693,0,69
1.551281,1190.550145,8.433332655,8.508504792,9.238650271,4.840652457,5,1,7.496295693,0,69
9,0,1,1,1,1,9,1691.551281,1190.550145,8.433332655,8.508504792,9.238650271,4.840652457,5,1,7
9,7.900222428,8.392871128,8.14418704,3,2,6.259959928,0,482.5528431,35.77119959,23.70066
71,0,1,0,1,1,7,996.2592061,724.3738923,2.682839969,7.900222428,8.392871128,8.14418704,3,2,6
.2592061,724.3738923,2.682839969,7.900222428,8.392871128,8.14418704,3,2,6.259959928,0,482
71,0,1,0,1,1,7,996.2592061,724.3738923,2.682839969,7.900222428,8.392871128,8.14418704,3,2,6
9,7.900222428,8.392871128,8.14418704,3,2,6.259959928,0,482.5528431,35.77119959,23.70066
2592061,724.3738923,2.682839969,7.900222428,8.392871128,8.14418704,3,2,6.259959928,0,482.
1,0,1,0,1,1,7,996.2592061,724.3738923,2.682839969,7.900222428,8.392871128,8.14418704,3,2,6.
71,0,1,0,1,1,7,996.2592061,724.3738923,2.682839969,7.900222428,8.392871128,8.14418704,3,2,6
2592061,724.3738923,2.682839969,7.900222428,8.392871128,8.14418704,3,2,6.259959928,0,482.
2592061,724.3738923,2.682839969,7.900222428,8.392871128,8.14418704,3,2,6.259959928,0,482.
.2592061,724.3738923,2.682839969,7.900222428,8.392871128,8.14418704,3,2,6.259959928,0,482
71,0,1,0,1,1,7,996.2592061,724.3738923,2.682839969,7.900222428,8.392871128,8.14418704,3,2,6
.2592061,724.3738923,2.682839969,7.900222428,8.392871128,8.14418704,3,2,6.259959928,0,482
6.2592061,724.3738923,2.682839969,7.900222428,8.392871128,8.14418704,3,2,6.259959928,0,48
6.2592061,724.3738923,2.682839969,7.900222428,8.392871128,8.14418704,3,2,6.259959928,0,48
.2592061,724.3738923,2.682839969,7.900222428,8.392871128,8.14418704,3,2,6.259959928,0,482
1,0,1,0,1,1,7,996.2592061,724.3738923,2.682839969,7.900222428,8.392871128,8.14418704,3,2,6.
71,0,1,0,1,1,7,996.2592061,724.3738923,2.682839969,7.900222428,8.392871128,8.14418704,3,2,6
.2592061,724.3738923,2.682839969,7.900222428,8.392871128,8.14418704,3,2,6.259959928,0,482
.2592061,724.3738923,2.682839969,7.900222428,8.392871128,8.14418704,3,2,6.259959928,0,482
71,0,1,0,1,1,7,996.2592061,724.3738923,2.682839969,7.900222428,8.392871128,8.14418704,3,2,6
9,7.900222428,8.392871128,8.14418704,3,2,6.259959928,0,482.5528431,35.77119959,23.70066
9,7.900222428,8.392871128,8.14418704,3,2,6.259959928,0,482.5528431,35.77119959,23.70066
71,0,1,0,1,1,7,996.2592061,724.3738923,2.682839969,7.900222428,8.392871128,8.14418704,3,2,6
2592061,724.3738923,2.682839969,7.900222428,8.392871128,8.14418704,3,2,6.259959928,0,482.
592061,724.3738923,2.682839969,7.900222428,8.392871128,8.14418704,3,2,6.259959928,0,482.5
9,7.900222428,8.392871128,8.14418704,3,2,6.259959928,0,482.5528431,35.77119959,23.70066
2592061,724.3738923,2.682839969,7.900222428,8.392871128,8.14418704,3,2,6.259959928,0,482.
2592061,724.3738923,2.682839969,7.900222428,8.392871128,8.14418704,3,2,6.259959928,0,482.
2592061,724.3738923,2.682839969,7.900222428,8.392871128,8.14418704,3,2,6.259959928,0,482.

592061,724.3738923,2.682839969,7.900222428,8.392871128,8.14418704,3,2,6.259959928,0,482.5
.2592061,724.3738923,2.682839969,7.900222428,8.392871128,8.14418704,3,2,6.259959928,0,482
59,7.900222428,8.392871128,8.14418704,3,2,6.259959928,0,482.5528431,35.77119959,23.700667
2592061,724.3738923,2.682839969,7.900222428,8.392871128,8.14418704,3,2,6.259959928,0,482.
59,7.900222428,8.392871128,8.14418704,3,2,6.259959928,0,482.5528431,35.77119959,23.700667
.900222428,8.392871128,8.14418704,3,2,6.259959928,0,482.5528431,35.77119959,23.70066728,2
71,0,1,0,1,1,7,996.2592061,724.3738923,2.682839969,7.900222428,8.392871128,8.14418704,3,2,6
2592061,724.3738923,2.682839969,7.900222428,8.392871128,8.14418704,3,2,6.259959928,0,482.
2592061,724.3738923,2.682839969,7.900222428,8.392871128,8.14418704,3,2,6.259959928,0,482.
2592061,724.3738923,2.682839969,7.900222428,8.392871128,8.14418704,3,2,6.259959928,0,482.
592061,724.3738923,2.682839969,7.900222428,8.392871128,8.14418704,3,2,6.259959928,0,482.5
2592061,724.3738923,2.682839969,7.900222428,8.392871128,8.14418704,3,2,6.259959928,0,482.
6.2592061,724.3738923,2.682839969,7.900222428,8.392871128,8.14418704,3,2,6.259959928,0,48
2592061,724.3738923,2.682839969,7.900222428,8.392871128,8.14418704,3,2,6.259959928,0,482.
6.2592061,724.3738923,2.682839969,7.900222428,8.392871128,8.14418704,3,2,6.259959928,0,48
33,7.610789149,8.392871128,4.840652457,3,1,3.234539833,0,482.4452367,34.50175822,45.66473
112.162054,1132.545046,3.234539833,7.610789149,8.392871128,4.840652457,3,1,3.234539833,0,
333,7.610789149,8.392871128,4.840652457,3,1,3.234539833,0,482.4452367,34.50175822,45.6647
12.162054,1132.545046,3.234539833,7.610789149,8.392871128,4.840652457,3,1,3.234539833,0,4
112.162054,1132.545046,3.234539833,7.610789149,8.392871128,4.840652457,3,1,3.234539833,0,
,1,1,0,1,1,11,1112.162054,1132.545046,3.234539833,7.610789149,8.392871128,4.840652457,3,1,3
333,7.610789149,8.392871128,4.840652457,3,1,3.234539833,0,482.4452367,34.50175822,45.6647
333,7.610789149,8.392871128,4.840652457,3,1,3.234539833,0,482.4452367,34.50175822,45.6647
12.162054,1132.545046,3.234539833,7.610789149,8.392871128,4.840652457,3,1,3.234539833,0,4
112.162054,1132.545046,3.234539833,7.610789149,8.392871128,4.840652457,3,1,3.234539833,0,
12.162054,1132.545046,3.234539833,7.610789149,8.392871128,4.840652457,3,1,3.234539833,0,4
112.162054,1132.545046,3.234539833,7.610789149,8.392871128,4.840652457,3,1,3.234539833,0,
112.162054,1132.545046,3.234539833,7.610789149,8.392871128,4.840652457,3,1,3.234539833,0,
12.162054,1132.545046,3.234539833,7.610789149,8.392871128,4.840652457,3,1,3.234539833,0,4
333,7.610789149,8.392871128,4.840652457,3,1,3.234539833,0,482.4452367,34.50175822,45.6647
76,1,1,0,1,1,11,1112.162054,1132.545046,3.234539833,7.610789149,8.392871128,4.840652457,3,
12.162054,1132.545046,3.234539833,7.610789149,8.392871128,4.840652457,3,1,3.234539833,0,4
333,7.610789149,8.392871128,4.840652457,3,1,3.234539833,0,482.4452367,34.50175822,45.6647
2.162054,1132.545046,3.234539833,7.610789149,8.392871128,4.840652457,3,1,3.234539833,0,48
2.162054,1132.545046,3.234539833,7.610789149,8.392871128,4.840652457,3,1,3.234539833,0,48
,1,1,0,1,1,11,1112.162054,1132.545046,3.234539833,7.610789149,8.392871128,4.840652457,3,1,3
333,7.610789149,8.392871128,4.840652457,3,1,3.234539833,0,482.4452367,34.50175822,45.6647
112.162054,1132.545046,3.234539833,7.610789149,8.392871128,4.840652457,3,1,3.234539833,0,
112.162054,1132.545046,3.234539833,7.610789149,8.392871128,4.840652457,3,1,3.234539833,0,
2.162054,1132.545046,3.234539833,7.610789149,8.392871128,4.840652457,3,1,3.234539833,0,48
2.162054,1132.545046,3.234539833,7.610789149,8.392871128,4.840652457,3,1,3.234539833,0,48
112.162054,1132.545046,3.234539833,7.610789149,8.392871128,4.840652457,3,1,3.234539833,0,
112.162054,1132.545046,3.234539833,7.610789149,8.392871128,4.840652457,3,1,3.234539833,0,
2.162054,1132.545046,3.234539833,7.610789149,8.392871128,4.840652457,3,1,3.234539833,0,48
2.162054,1132.545046,3.234539833,7.610789149,8.392871128,4.840652457,3,1,3.234539833,0,48
2.162054,1132.545046,3.234539833,7.610789149,8.392871128,4.840652457,3,1,3.234539833,0,48
112.162054,1132.545046,3.234539833,7.610789149,8.392871128,4.840652457,3,1,3.234539833,0,
333,7.610789149,8.392871128,4.840652457,3,1,3.234539833,0,482.4452367,34.50175822,45.6647
76,1,1,0,1,1,11,1112.162054,1132.545046,3.234539833,7.610789149,8.392871128,4.840652457,3,
12.162054,1132.545046,3.234539833,7.610789149,8.392871128,4.840652457,3,1,3.234539833,0,4

112.162054,1132.545046,3.234539833,7.610789149,8.392871128,4.840652457,3,1,3.234539833,0,
112.162054,1132.545046,3.234539833,7.610789149,8.392871128,4.840652457,3,1,3.234539833,0,
33,7.610789149,8.392871128,4.840652457,3,1,3.234539833,0,482.4452367,34.50175822,45.6647
12.162054,1132.545046,3.234539833,7.610789149,8.392871128,4.840652457,3,1,3.234539833,0,4
112.162054,1132.545046,3.234539833,7.610789149,8.392871128,4.840652457,3,1,3.234539833,0,
98,7.155835616,237.8059422,10.70238059,4,2,5.093187396,7.155835616,280.5783101,18.462804
.5354949,705.9742577,2.546593698,7.155835616,237.8059422,10.70238059,4,2,5.093187396,7.15
98,7.155835616,237.8059422,10.70238059,4,2,5.093187396,7.155835616,280.5783101,18.4628043
98,7.155835616,237.8059422,10.70238059,4,2,5.093187396,7.155835616,280.5783101,18.4628043
46593698,7.155835616,237.8059422,10.70238059,4,2,5.093187396,7.155835616,280.5783101,18.
.5354949,705.9742577,2.546593698,7.155835616,237.8059422,10.70238059,4,2,5.093187396,7.15
8.5354949,705.9742577,2.546593698,7.155835616,237.8059422,10.70238059,4,2,5.093187396,7.1
.5354949,705.9742577,2.546593698,7.155835616,237.8059422,10.70238059,4,2,5.093187396,7.15
29395,3,2,209,73,1,1,1,1,1,9,938.5354949,705.9742577,2.546593698,7.155835616,237.8059422,1
.5354949,705.9742577,2.546593698,7.155835616,237.8059422,10.70238059,4,2,5.093187396,7.15
354949,705.9742577,2.546593698,7.155835616,237.8059422,10.70238059,4,2,5.093187396,7.155
354949,705.9742577,2.546593698,7.155835616,237.8059422,10.70238059,4,2,5.093187396,7.155
98,7.155835616,237.8059422,10.70238059,4,2,5.093187396,7.155835616,280.5783101,18.4628043
354949,705.9742577,2.546593698,7.155835616,237.8059422,10.70238059,4,2,5.093187396,7.155
46593698,7.155835616,237.8059422,10.70238059,4,2,5.093187396,7.155835616,280.5783101,18.
46593698,7.155835616,237.8059422,10.70238059,4,2,5.093187396,7.155835616,280.5783101,18.
.5354949,705.9742577,2.546593698,7.155835616,237.8059422,10.70238059,4,2,5.093187396,7.15
.5354949,705.9742577,2.546593698,7.155835616,237.8059422,10.70238059,4,2,5.093187396,7.15
98,7.155835616,237.8059422,10.70238059,4,2,5.093187396,7.155835616,280.5783101,18.4628043
3,1,1,1,1,1,9,938.5354949,705.9742577,2.546593698,7.155835616,237.8059422,10.70238059,4,2,5
354949,705.9742577,2.546593698,7.155835616,237.8059422,10.70238059,4,2,5.093187396,7.155
3,1,1,1,1,1,9,938.5354949,705.9742577,2.546593698,7.155835616,237.8059422,10.70238059,4,2,5
46593698,7.155835616,237.8059422,10.70238059,4,2,5.093187396,7.155835616,280.5783101,18.
3,1,1,1,1,1,9,938.5354949,705.9742577,2.546593698,7.155835616,237.8059422,10.70238059,4,2,5
98,7.155835616,237.8059422,10.70238059,4,2,5.093187396,7.155835616,280.5783101,18.4628043
593698,7.155835616,237.8059422,10.70238059,4,2,5.093187396,7.155835616,280.5783101,18.46
98,7.155835616,237.8059422,10.70238059,4,2,5.093187396,7.155835616,280.5783101,18.4628043
3,1,1,1,1,1,9,938.5354949,705.9742577,2.546593698,7.155835616,237.8059422,10.70238059,4,2,5
593698,7.155835616,237.8059422,10.70238059,4,2,5.093187396,7.155835616,280.5783101,18.46
.5354949,705.9742577,2.546593698,7.155835616,237.8059422,10.70238059,4,2,5.093187396,7.15
98,7.155835616,237.8059422,10.70238059,4,2,5.093187396,7.155835616,280.5783101,18.4628043
7,7.988583773,4.657041108,2.452816392,5,2,1.112770585,0,749.2027532,34.49588815,7.988583
.9786762,2605.764446,5.563852927,7.988583773,4.657041108,2.452816392,5,2,1.112770585,0,74
.9786762,2605.764446,5.563852927,7.988583773,4.657041108,2.452816392,5,2,1.112770585,0,74
27,7.988583773,4.657041108,2.452816392,5,2,1.112770585,0,749.2027532,34.49588815,7.988583
.9786762,2605.764446,5.563852927,7.988583773,4.657041108,2.452816392,5,2,1.112770585,0,74
.9786762,2605.764446,5.563852927,7.988583773,4.657041108,2.452816392,5,2,1.112770585,0,74
0.9786762,2605.764446,5.563852927,7.988583773,4.657041108,2.452816392,5,2,1.112770585,0,
0.9786762,2605.764446,5.563852927,7.988583773,4.657041108,2.452816392,5,2,1.112770585,0,7
27,7.988583773,4.657041108,2.452816392,5,2,1.112770585,0,749.2027532,34.49588815,7.988583
9786762,2605.764446,5.563852927,7.988583773,4.657041108,2.452816392,5,2,1.112770585,0,749
0.9786762,2605.764446,5.563852927,7.988583773,4.657041108,2.452816392,5,2,1.112770585,0,7
7,1,1,1,1,1,9,700.9786762,2605.764446,5.563852927,7.988583773,4.657041108,2.452816392,5,2,1
52927,7.988583773,4.657041108,2.452816392,5,2,1.112770585,0,749.2027532,34.49588815,7.988
27,7.988583773,4.657041108,2.452816392,5,2,1.112770585,0,749.2027532,34.49588815,7.988583

63852927,7.988583773,4.657041108,2.452816392,5,2,1.112770585,0,749.2027532,34.49588815,7.
352927,7.988583773,4.657041108,2.452816392,5,2,1.112770585,0,749.2027532,34.49588815,7.988
0.9786762,2605.764446,5.563852927,7.988583773,4.657041108,2.452816392,5,2,1.112770585,0,7
67,1,1,1,1,1,9,700.9786762,2605.764446,5.563852927,7.988583773,4.657041108,2.452816392,5,2,
.988583773,4.657041108,2.452816392,5,2,1.112770585,0,749.2027532,34.49588815,7.988583773,
.9786762,2605.764446,5.563852927,7.988583773,4.657041108,2.452816392,5,2,1.112770585,0,74
.9786762,2605.764446,5.563852927,7.988583773,4.657041108,2.452816392,5,2,1.112770585,0,74
63852927,7.988583773,4.657041108,2.452816392,5,2,1.112770585,0,749.2027532,34.49588815,7.
9786762,2605.764446,5.563852927,7.988583773,4.657041108,2.452816392,5,2,1.112770585,0,749
852927,7.988583773,4.657041108,2.452816392,5,2,1.112770585,0,749.2027532,34.49588815,7.98
9786762,2605.764446,5.563852927,7.988583773,4.657041108,2.452816392,5,2,1.112770585,0,749
.9786762,2605.764446,5.563852927,7.988583773,4.657041108,2.452816392,5,2,1.112770585,0,74
.9786762,2605.764446,5.563852927,7.988583773,4.657041108,2.452816392,5,2,1.112770585,0,74
.9786762,2605.764446,5.563852927,7.988583773,4.657041108,2.452816392,5,2,1.112770585,0,74
27,7.988583773,4.657041108,2.452816392,5,2,1.112770585,0,749.2027532,34.49588815,7.988583
.9786762,2605.764446,5.563852927,7.988583773,4.657041108,2.452816392,5,2,1.112770585,0,74
7,1,1,1,1,1,9,700.9786762,2605.764446,5.563852927,7.988583773,4.657041108,2.452816392,5,2,1
7,1,1,1,1,1,9,700.9786762,2605.764446,5.563852927,7.988583773,4.657041108,2.452816392,5,2,1
0.9786762,2605.764446,5.563852927,7.988583773,4.657041108,2.452816392,5,2,1.112770585,0,
4.420022,674.8695037,0,0,0,0,0,0,1.416288754,0,986.3422464,7.789588148,6.102517046,1.416288
420022,674.8695037,0,0,0,0,0,0,1.416288754,0,986.3422464,7.789588148,6.102517046,1.4162887
420022,674.8695037,0,0,0,0,0,0,1.416288754,0,986.3422464,7.789588148,6.102517046,1.4162887
4.420022,674.8695037,0,0,0,0,0,0,1.416288754,0,986.3422464,7.789588148,6.102517046,1.416288
416288754,0,986.3422464,7.789588148,6.102517046,1.416288754,0,2.832577508,3.051258523,2.8
,0,1.416288754,0,986.3422464,7.789588148,6.102517046,1.416288754,0,2.832577508,3.05125852
420022,674.8695037,0,0,0,0,0,0,1.416288754,0,986.3422464,7.789588148,6.102517046,1.4162887
.420022,674.8695037,0,0,0,0,0,0,1.416288754,0,986.3422464,7.789588148,6.102517046,1.416288
.416288754,0,986.3422464,7.789588148,6.102517046,1.416288754,0,2.832577508,3.051258523,2.
69,1,1,0,1,1,9,854.420022,674.8695037,0,0,0,0,0,0,1.416288754,0,986.3422464,7.789588148,6.102
69,1,1,0,1,1,9,854.420022,674.8695037,0,0,0,0,0,0,1.416288754,0,986.3422464,7.789588148,6.102
420022,674.8695037,0,0,0,0,0,0,1.416288754,0,986.3422464,7.789588148,6.102517046,1.4162887
.416288754,0,986.3422464,7.789588148,6.102517046,1.416288754,0,2.832577508,3.051258523,2.
.416288754,0,986.3422464,7.789588148,6.102517046,1.416288754,0,2.832577508,3.051258523,2.
0,1.416288754,0,986.3422464,7.789588148,6.102517046,1.416288754,0,2.832577508,3.051258523
.416288754,0,986.3422464,7.789588148,6.102517046,1.416288754,0,2.832577508,3.051258523,2.
.416288754,0,986.3422464,7.789588148,6.102517046,1.416288754,0,2.832577508,3.051258523,2.
420022,674.8695037,0,0,0,0,0,0,1.416288754,0,986.3422464,7.789588148,6.102517046,1.4162887
.420022,674.8695037,0,0,0,0,0,0,1.416288754,0,986.3422464,7.789588148,6.102517046,1.416288
420022,674.8695037,0,0,0,0,0,0,1.416288754,0,986.3422464,7.789588148,6.102517046,1.4162887
416288754,0,986.3422464,7.789588148,6.102517046,1.416288754,0,2.832577508,3.051258523,2.8
.416288754,0,986.3422464,7.789588148,6.102517046,1.416288754,0,2.832577508,3.051258523,2.
420022,674.8695037,0,0,0,0,0,0,1.416288754,0,986.3422464,7.789588148,6.102517046,1.4162887
9,1,1,0,1,1,9,854.420022,674.8695037,0,0,0,0,0,0,1.416288754,0,986.3422464,7.789588148,6.1025
4.420022,674.8695037,0,0,0,0,0,0,1.416288754,0,986.3422464,7.789588148,6.102517046,1.416288
517676,3,2,209,69,1,1,0,1,1,9,854.420022,674.8695037,0,0,0,0,0,0,1.416288754,0,986.3422464,7.78
.420022,674.8695037,0,0,0,0,0,0,1.416288754,0,986.3422464,7.789588148,6.102517046,1.416288
9,1,1,0,1,1,9,854.420022,674.8695037,0,0,0,0,0,0,1.416288754,0,986.3422464,7.789588148,6.1025
420022,674.8695037,0,0,0,0,0,0,1.416288754,0,986.3422464,7.789588148,6.102517046,1.4162887
.416288754,0,986.3422464,7.789588148,6.102517046,1.416288754,0,2.832577508,3.051258523,2.
,0,1.416288754,0,986.3422464,7.789588148,6.102517046,1.416288754,0,2.832577508,3.05125852

420022,674.8695037,0,0,0,0,0,0,1.416288754,0,986.3422464,7.789588148,6.102517046,1.41628875
420022,674.8695037,0,0,0,0,0,0,1.416288754,0,986.3422464,7.789588148,6.102517046,1.41628875
420022,674.8695037,0,0,0,0,0,0,1.416288754,0,986.3422464,7.789588148,6.102517046,1.41628875
0,0,0,0,1.416288754,0,986.3422464,7.789588148,6.102517046,1.416288754,0,2.832577508,3.05125
420022,674.8695037,0,0,0,0,0,0,1.416288754,0,986.3422464,7.789588148,6.102517046,1.41628875
420022,674.8695037,0,0,0,0,0,0,1.416288754,0,986.3422464,7.789588148,6.102517046,1.41628875
69,1,1,0,1,1,9,854.420022,674.8695037,0,0,0,0,0,0,1.416288754,0,986.3422464,7.789588148,6.102
9,1,1,0,1,1,9,854.420022,674.8695037,0,0,0,0,0,0,1.416288754,0,986.3422464,7.789588148,6.1025
420022,674.8695037,0,0,0,0,0,0,1.416288754,0,986.3422464,7.789588148,6.102517046,1.41628875
5.979757177,0,652.1366776,13.95276675,30.78836857,1.993252392,0,0.996626196,6.157673714,0
3.790738,1043.749955,0,0,0,0,0,0,5.979757177,0,652.1366776,13.95276675,30.78836857,1.993252
.790738,1043.749955,0,0,0,0,0,0,5.979757177,0,652.1366776,13.95276675,30.78836857,1.9932523
7,0,1,0,1,1,9,1343.790738,1043.749955,0,0,0,0,0,0,5.979757177,0,652.1366776,13.95276675,30.78
0617676,3,2,209,77,0,1,0,1,1,9,1343.790738,1043.749955,0,0,0,0,0,0,5.979757177,0,652.1366776,1
0,0,5.979757177,0,652.1366776,13.95276675,30.78836857,1.993252392,0,0.996626196,6.1576737
.790738,1043.749955,0,0,0,0,0,0,5.979757177,0,652.1366776,13.95276675,30.78836857,1.9932523
7,0,1,0,1,1,9,1343.790738,1043.749955,0,0,0,0,0,0,5.979757177,0,652.1366776,13.95276675,30.78
3.790738,1043.749955,0,0,0,0,0,0,5.979757177,0,652.1366776,13.95276675,30.78836857,1.993252
3.790738,1043.749955,0,0,0,0,0,0,5.979757177,0,652.1366776,13.95276675,30.78836857,1.993252
0,0,5.979757177,0,652.1366776,13.95276675,30.78836857,1.993252392,0,0.996626196,6.1576737
0,5.979757177,0,652.1366776,13.95276675,30.78836857,1.993252392,0,0.996626196,6.157673714
0,0,5.979757177,0,652.1366776,13.95276675,30.78836857,1.993252392,0,0.996626196,6.1576737
7,0,1,0,1,1,9,1343.790738,1043.749955,0,0,0,0,0,0,5.979757177,0,652.1366776,13.95276675,30.78
.790738,1043.749955,0,0,0,0,0,0,5.979757177,0,652.1366776,13.95276675,30.78836857,1.9932523
0,5.979757177,0,652.1366776,13.95276675,30.78836857,1.993252392,0,0.996626196,6.157673714
3.790738,1043.749955,0,0,0,0,0,0,5.979757177,0,652.1366776,13.95276675,30.78836857,1.993252
0,5.979757177,0,652.1366776,13.95276675,30.78836857,1.993252392,0,0.996626196,6.157673714
7,0,1,0,1,1,9,1343.790738,1043.749955,0,0,0,0,0,0,5.979757177,0,652.1366776,13.95276675,30.78
43.790738,1043.749955,0,0,0,0,0,0,5.979757177,0,652.1366776,13.95276675,30.78836857,1.99325
0,0,5.979757177,0,652.1366776,13.95276675,30.78836857,1.993252392,0,0.996626196,6.1576737
7,0,1,0,1,1,9,1343.790738,1043.749955,0,0,0,0,0,0,5.979757177,0,652.1366776,13.95276675,30.78
.790738,1043.749955,0,0,0,0,0,0,5.979757177,0,652.1366776,13.95276675,30.78836857,1.9932523
.790738,1043.749955,0,0,0,0,0,0,5.979757177,0,652.1366776,13.95276675,30.78836857,1.9932523
.790738,1043.749955,0,0,0,0,0,0,5.979757177,0,652.1366776,13.95276675,30.78836857,1.9932523
5.979757177,0,652.1366776,13.95276675,30.78836857,1.993252392,0,0.996626196,6.157673714,0
7,0,1,0,1,1,9,1343.790738,1043.749955,0,0,0,0,0,0,5.979757177,0,652.1366776,13.95276675,30.78
.790738,1043.749955,0,0,0,0,0,0,5.979757177,0,652.1366776,13.95276675,30.78836857,1.9932523
0,0,5.979757177,0,652.1366776,13.95276675,30.78836857,1.993252392,0,0.996626196,6.1576737
0,0,5.979757177,0,652.1366776,13.95276675,30.78836857,1.993252392,0,0.996626196,6.1576737
343.790738,1043.749955,0,0,0,0,0,0,5.979757177,0,652.1366776,13.95276675,30.78836857,1.9932
77,0,1,0,1,1,9,1343.790738,1043.749955,0,0,0,0,0,0,5.979757177,0,652.1366776,13.95276675,30.7
.790738,1043.749955,0,0,0,0,0,0,5.979757177,0,652.1366776,13.95276675,30.78836857,1.9932523
.790738,1043.749955,0,0,0,0,0,0,5.979757177,0,652.1366776,13.95276675,30.78836857,1.9932523
617676,3,2,209,68,0,1,0,1,1,9,918.0759661,1049.940098,4.133700946,0,7.798250346,0,4,0,0,0,103
.0759661,1049.940098,4.133700946,0,7.798250346,0,4,0,0,0,1036.240537,11.02320252,15.649944
00946,0,7.798250346,0,4,0,0,0,1036.240537,11.02320252,15.64994466,1.377900315,2.60832411,3.
46,0,7.798250346,0,4,0,0,0,1036.240537,11.02320252,15.64994466,1.377900315,2.60832411,3.444
0759661,1049.940098,4.133700946,0,7.798250346,0,4,0,0,0,1036.240537,11.02320252,15.6499446
46,0,7.798250346,0,4,0,0,0,1036.240537,11.02320252,15.64994466,1.377900315,2.60832411,3.444
8,0,1,0,1,1,9,918.0759661,1049.940098,4.133700946,0,7.798250346,0,4,0,0,0,1036.240537,11.0232

8.0759661,1049.940098,4.133700946,0,7.798250346,0,4,0,0,0,1036.240537,11.02320252,15.64994
17676,3,2,209,68,0,1,0,1,1,9,918.0759661,1049.940098,4.133700946,0,7.798250346,0,4,0,0,0,103
8.0759661,1049.940098,4.133700946,0,7.798250346,0,4,0,0,0,1036.240537,11.02320252,15.64994
0946,0,7.798250346,0,4,0,0,0,1036.240537,11.02320252,15.64994466,1.377900315,2.60832411,3.
8,0,1,0,1,1,9,918.0759661,1049.940098,4.133700946,0,7.798250346,0,4,0,0,0,1036.240537,11.023
8,0,1,0,1,1,9,918.0759661,1049.940098,4.133700946,0,7.798250346,0,4,0,0,0,1036.240537,11.023
133700946,0,7.798250346,0,4,0,0,0,1036.240537,11.02320252,15.64994466,1.377900315,2.60832
33700946,0,7.798250346,0,4,0,0,0,1036.240537,11.02320252,15.64994466,1.377900315,2.608324
.0759661,1049.940098,4.133700946,0,7.798250346,0,4,0,0,0,1036.240537,11.02320252,15.649944
.0759661,1049.940098,4.133700946,0,7.798250346,0,4,0,0,0,1036.240537,11.02320252,15.649944
.0759661,1049.940098,4.133700946,0,7.798250346,0,4,0,0,0,1036.240537,11.02320252,15.649944
8.0759661,1049.940098,4.133700946,0,7.798250346,0,4,0,0,0,1036.240537,11.02320252,15.64994
8,0,1,0,1,1,9,918.0759661,1049.940098,4.133700946,0,7.798250346,0,4,0,0,0,1036.240537,11.023
8.0759661,1049.940098,4.133700946,0,7.798250346,0,4,0,0,0,1036.240537,11.02320252,15.64994
17676,3,2,209,68,0,1,0,1,1,9,918.0759661,1049.940098,4.133700946,0,7.798250346,0,4,0,0,0,103
.0759661,1049.940098,4.133700946,0,7.798250346,0,4,0,0,0,1036.240537,11.02320252,15.649944
.0759661,1049.940098,4.133700946,0,7.798250346,0,4,0,0,0,1036.240537,11.02320252,15.649944
.0759661,1049.940098,4.133700946,0,7.798250346,0,4,0,0,0,1036.240537,11.02320252,15.649944
3700946,0,7.798250346,0,4,0,0,0,1036.240537,11.02320252,15.64994466,1.377900315,2.6083241
,0,1,0,1,1,9,918.0759661,1049.940098,4.133700946,0,7.798250346,0,4,0,0,0,1036.240537,11.0232
759661,1049.940098,4.133700946,0,7.798250346,0,4,0,0,0,1036.240537,11.02320252,15.6499446
6,0,7.798250346,0,4,0,0,0,1036.240537,11.02320252,15.64994466,1.377900315,2.60832411,3.444
8,0,1,0,1,1,9,918.0759661,1049.940098,4.133700946,0,7.798250346,0,4,0,0,0,1036.240537,11.023
8.0759661,1049.940098,4.133700946,0,7.798250346,0,4,0,0,0,1036.240537,11.02320252,15.64994
617676,3,2,209,68,0,1,0,1,1,9,918.0759661,1049.940098,4.133700946,0,7.798250346,0,4,0,0,0,103
46,0,7.798250346,0,4,0,0,0,1036.240537,11.02320252,15.64994466,1.377900315,2.60832411,3.44
.0759661,1049.940098,4.133700946,0,7.798250346,0,4,0,0,0,1036.240537,11.02320252,15.649944
46,0,7.798250346,0,4,0,0,0,1036.240537,11.02320252,15.64994466,1.377900315,2.60832411,3.44
8.0759661,1049.940098,4.133700946,0,7.798250346,0,4,0,0,0,1036.240537,11.02320252,15.64994
5,0,5.594025631,0,1,0,0,0,1104.238439,12.6063071,3.166898957,1.146027918,0,0,0,0,0,1.146027
0.548676,1138.897852,3.438083755,0,5.594025631,0,1,0,0,0,1104.238439,12.6063071,3.16689895
0.548676,1138.897852,3.438083755,0,5.594025631,0,1,0,0,0,1104.238439,12.6063071,3.16689895
5,0,0,0,1,1,9,1000.548676,1138.897852,3.438083755,0,5.594025631,0,1,0,0,0,1104.238439,12.606
5,0,0,0,1,1,9,1000.548676,1138.897852,3.438083755,0,5.594025631,0,1,0,0,0,1104.238439,12.606
.548676,1138.897852,3.438083755,0,5.594025631,0,1,0,0,0,1104.238439,12.6063071,3.166898957
.548676,1138.897852,3.438083755,0,5.594025631,0,1,0,0,0,1104.238439,12.6063071,3.166898957
.548676,1138.897852,3.438083755,0,5.594025631,0,1,0,0,0,1104.238439,12.6063071,3.166898957
3755,0,5.594025631,0,1,0,0,0,1104.238439,12.6063071,3.166898957,1.146027918,0,0,0,0,0,1.146
.548676,1138.897852,3.438083755,0,5.594025631,0,1,0,0,0,1104.238439,12.6063071,3.166898957
0.548676,1138.897852,3.438083755,0,5.594025631,0,1,0,0,0,1104.238439,12.6063071,3.16689895
3755,0,5.594025631,0,1,0,0,0,1104.238439,12.6063071,3.166898957,1.146027918,0,0,0,0,0,1.146
,5.594025631,0,1,0,0,0,1104.238439,12.6063071,3.166898957,1.146027918,0,0,0,0,0,1.146027918,
5,0,5.594025631,0,1,0,0,0,1104.238439,12.6063071,3.166898957,1.146027918,0,0,0,0,0,1.146027
0.548676,1138.897852,3.438083755,0,5.594025631,0,1,0,0,0,1104.238439,12.6063071,3.16689895
83755,0,5.594025631,0,1,0,0,0,1104.238439,12.6063071,3.166898957,1.146027918,0,0,0,0,0,1.14
38083755,0,5.594025631,0,1,0,0,0,1104.238439,12.6063071,3.166898957,1.146027918,0,0,0,0,0,1.
,0,5.594025631,0,1,0,0,0,1104.238439,12.6063071,3.166898957,1.146027918,0,0,0,0,0,1.1460279
,0,5.594025631,0,1,0,0,0,1104.238439,12.6063071,3.166898957,1.146027918,0,0,0,0,0,1.1460279
5,0,0,0,1,1,9,1000.548676,1138.897852,3.438083755,0,5.594025631,0,1,0,0,0,1104.238439,12.606
75,0,0,0,1,1,9,1000.548676,1138.897852,3.438083755,0,5.594025631,0,1,0,0,0,1104.238439,12.60

.548676,1138.897852,3.438083755,0,5.594025631,0,1,0,0,0,1104.238439,12.6063071,3.166898957
33755,0,5.594025631,0,1,0,0,0,1104.238439,12.6063071,3.166898957,1.146027918,0,0,0,0,0,1.1460
,0,0,0,1,1,9,1000.548676,1138.897852,3.438083755,0,5.594025631,0,1,0,0,0,1104.238439,12.60630
00.548676,1138.897852,3.438083755,0,5.594025631,0,1,0,0,0,1104.238439,12.6063071,3.16689895
5,0,0,0,1,1,9,1000.548676,1138.897852,3.438083755,0,5.594025631,0,1,0,0,0,1104.238439,12.6063
3,0,7.805690249,0,5,0,0,0,1141.136848,10.06244028,13.17871256,1.341658704,0,2.683317408,3.29
113,0,7.805690249,0,5,0,0,0,1141.136848,10.06244028,13.17871256,1.341658704,0,2.683317408,3
113,0,7.805690249,0,5,0,0,0,1141.136848,10.06244028,13.17871256,1.341658704,0,2.683317408,3
976113,0,7.805690249,0,5,0,0,0,1141.136848,10.06244028,13.17871256,1.341658704,0,2.68331740
9.3757817,1123.12525,4.024976113,0,7.805690249,0,5,0,0,0,1141.136848,10.06244028,13.178712
3,0,0,1,1,1,9,899.3757817,1123.12525,4.024976113,0,7.805690249,0,5,0,0,0,1141.136848,10.06244
3757817,1123.12525,4.024976113,0,7.805690249,0,5,0,0,0,1141.136848,10.06244028,13.17871256
9.3757817,1123.12525,4.024976113,0,7.805690249,0,5,0,0,0,1141.136848,10.06244028,13.178712
9.3757817,1123.12525,4.024976113,0,7.805690249,0,5,0,0,0,1141.136848,10.06244028,13.178712
5113,0,7.805690249,0,5,0,0,0,1141.136848,10.06244028,13.17871256,1.341658704,0,2.683317408,
4976113,0,7.805690249,0,5,0,0,0,1141.136848,10.06244028,13.17871256,1.341658704,0,2.683317
3,0,7.805690249,0,5,0,0,0,1141.136848,10.06244028,13.17871256,1.341658704,0,2.683317408,3.29
3,0,7.805690249,0,5,0,0,0,1141.136848,10.06244028,13.17871256,1.341658704,0,2.683317408,3.29
3,0,0,1,1,1,9,899.3757817,1123.12525,4.024976113,0,7.805690249,0,5,0,0,0,1141.136848,10.06244
.3757817,1123.12525,4.024976113,0,7.805690249,0,5,0,0,0,1141.136848,10.06244028,13.1787125
3,0,0,1,1,1,9,899.3757817,1123.12525,4.024976113,0,7.805690249,0,5,0,0,0,1141.136848,10.06244
73,0,0,1,1,1,9,899.3757817,1123.12525,4.024976113,0,7.805690249,0,5,0,0,0,1141.136848,10.0624
0,7.805690249,0,5,0,0,0,1141.136848,10.06244028,13.17871256,1.341658704,0,2.683317408,3.294
73,0,0,1,1,1,9,899.3757817,1123.12525,4.024976113,0,7.805690249,0,5,0,0,0,1141.136848,10.0624
3757817,1123.12525,4.024976113,0,7.805690249,0,5,0,0,0,1141.136848,10.06244028,13.17871256
3757817,1123.12525,4.024976113,0,7.805690249,0,5,0,0,0,1141.136848,10.06244028,13.17871256
5113,0,7.805690249,0,5,0,0,0,1141.136848,10.06244028,13.17871256,1.341658704,0,2.683317408,
5113,0,7.805690249,0,5,0,0,0,1141.136848,10.06244028,13.17871256,1.341658704,0,2.683317408,
.3757817,1123.12525,4.024976113,0,7.805690249,0,5,0,0,0,1141.136848,10.06244028,13.1787125
99.3757817,1123.12525,4.024976113,0,7.805690249,0,5,0,0,0,1141.136848,10.06244028,13.178712
3,0,7.805690249,0,5,0,0,0,1141.136848,10.06244028,13.17871256,1.341658704,0,2.683317408,3.29
9.3757817,1123.12525,4.024976113,0,7.805690249,0,5,0,0,0,1141.136848,10.06244028,13.178712
0,7.805690249,0,5,0,0,0,1141.136848,10.06244028,13.17871256,1.341658704,0,2.683317408,3.294
9.3757817,1123.12525,4.024976113,0,7.805690249,0,5,0,0,0,1141.136848,10.06244028,13.178712
048,0,7.798250346,0,4,0,0,0,1007.104596,13.26971012,19.95747876,1.769294683,3.991495751,4.4
34048,0,7.798250346,0,4,0,0,0,1007.104596,13.26971012,19.95747876,1.769294683,3.991495751,4
68,1,1,0,1,1,9,908.46098,986.9408127,5.307884048,0,7.798250346,0,4,0,0,0,1007.104596,13.26971
46098,986.9408127,5.307884048,0,7.798250346,0,4,0,0,0,1007.104596,13.26971012,19.95747876,1
34048,0,7.798250346,0,4,0,0,0,1007.104596,13.26971012,19.95747876,1.769294683,3.991495751,4
258789,3,2,209,68,1,1,0,1,1,9,908.46098,986.9408127,5.307884048,0,7.798250346,0,4,0,0,0,1007.1
884048,0,7.798250346,0,4,0,0,0,1007.104596,13.26971012,19.95747876,1.769294683,3.991495751
46098,986.9408127,5.307884048,0,7.798250346,0,4,0,0,0,1007.104596,13.26971012,19.95747876,1
0,7.798250346,0,4,0,0,0,1007.104596,13.26971012,19.95747876,1.769294683,3.991495751,4.4232
0617676,3,2,209,68,1,1,0,1,1,9,908.46098,986.9408127,5.307884048,0,7.798250346,0,4,0,0,0,1007.
.46098,986.9408127,5.307884048,0,7.798250346,0,4,0,0,0,1007.104596,13.26971012,19.95747876
.46098,986.9408127,5.307884048,0,7.798250346,0,4,0,0,0,1007.104596,13.26971012,19.95747876
68,1,1,0,1,1,9,908.46098,986.9408127,5.307884048,0,7.798250346,0,4,0,0,0,1007.104596,13.26971
.46098,986.9408127,5.307884048,0,7.798250346,0,4,0,0,0,1007.104596,13.26971012,19.95747876
8.46098,986.9408127,5.307884048,0,7.798250346,0,4,0,0,0,1007.104596,13.26971012,19.9574787
34048,0,7.798250346,0,4,0,0,0,1007.104596,13.26971012,19.95747876,1.769294683,3.991495751,4

68,1,1,0,1,1,9,908.46098,986.9408127,5.307884048,0,7.798250346,0,4,0,0,0,1007.104596,13.2697
884048,0,7.798250346,0,4,0,0,0,1007.104596,13.26971012,19.95747876,1.769294683,3.991495751
0,7.798250346,0,4,0,0,0,1007.104596,13.26971012,19.95747876,1.769294683,3.991495751,4.4232
8.46098,986.9408127,5.307884048,0,7.798250346,0,4,0,0,0,1007.104596,13.26971012,19.9574787
46098,986.9408127,5.307884048,0,7.798250346,0,4,0,0,0,1007.104596,13.26971012,19.95747876,
7884048,0,7.798250346,0,4,0,0,0,1007.104596,13.26971012,19.95747876,1.769294683,3.9914957
,7.798250346,0,4,0,0,0,1007.104596,13.26971012,19.95747876,1.769294683,3.991495751,4.42323
4048,0,7.798250346,0,4,0,0,0,1007.104596,13.26971012,19.95747876,1.769294683,3.991495751,4.
8.46098,986.9408127,5.307884048,0,7.798250346,0,4,0,0,0,1007.104596,13.26971012,19.9574787
8.46098,986.9408127,5.307884048,0,7.798250346,0,4,0,0,0,1007.104596,13.26971012,19.9574787
048,0,7.798250346,0,4,0,0,0,1007.104596,13.26971012,19.95747876,1.769294683,3.991495751,4.4
0,7.798250346,0,4,0,0,0,1007.104596,13.26971012,19.95747876,1.769294683,3.991495751,4.4232
3,0,7.798250346,0,4,0,0,0,1007.104596,13.26971012,19.95747876,1.769294683,3.991495751,4.423
4048,0,7.798250346,0,4,0,0,0,1007.104596,13.26971012,19.95747876,1.769294683,3.991495751,4.
.46098,986.9408127,5.307884048,0,7.798250346,0,4,0,0,0,1007.104596,13.26971012,19.95747876
.46098,986.9408127,5.307884048,0,7.798250346,0,4,0,0,0,1007.104596,13.26971012,19.95747876
8.46098,986.9408127,5.307884048,0,7.798250346,0,4,0,0,0,1007.104596,13.26971012,19.9574787
3,0,7.798250346,0,4,0,0,0,1007.104596,13.26971012,19.95747876,1.769294683,3.991495751,4.423
.46098,986.9408127,5.307884048,0,7.798250346,0,4,0,0,0,1007.104596,13.26971012,19.95747876
08.46098,986.9408127,5.307884048,0,7.798250346,0,4,0,0,0,1007.104596,13.26971012,19.9574787
8.46098,986.9408127,5.307884048,0,7.798250346,0,4,0,0,0,1007.104596,13.26971012,19.9574787
8,0,1,0,1,1,12,908.46098,986.9408127,5.307884048,0,7.798250346,0,4,0,0,0,1007.104596,13.2697
84048,0,7.798250346,0,4,0,0,0,1007.104596,13.26971012,19.95747876,1.769294683,3.991495751,
8.46098,986.9408127,5.307884048,0,7.798250346,0,4,0,0,0,1007.104596,13.26971012,19.9574787
8,0,1,0,1,1,12,908.46098,986.9408127,5.307884048,0,7.798250346,0,4,0,0,0,1007.104596,13.2697
8.46098,986.9408127,5.307884048,0,7.798250346,0,4,0,0,0,1007.104596,13.26971012,19.9574787
4048,0,7.798250346,0,4,0,0,0,1007.104596,13.26971012,19.95747876,1.769294683,3.991495751,4.
08.46098,986.9408127,5.307884048,0,7.798250346,0,4,0,0,0,1007.104596,13.26971012,19.957478
3,0,7.798250346,0,4,0,0,0,1007.104596,13.26971012,19.95747876,1.769294683,3.991495751,4.423
7884048,0,7.798250346,0,4,0,0,0,1007.104596,13.26971012,19.95747876,1.769294683,3.9914957
0.7987446,991.1770082,4.87239487,0,7.798250346,0,4,0,1.624131623,0,948.3584568,13.8051188,
30.7987446,991.1770082,4.87239487,0,7.798250346,0,4,0,1.624131623,0,948.3584568,13.805118
30.7987446,991.1770082,4.87239487,0,7.798250346,0,4,0,1.624131623,0,948.3584568,13.8051188
87239487,0,7.798250346,0,4,0,1.624131623,0,948.3584568,13.8051188,13.73743922,1.624131623
487,0,7.798250346,0,4,0,1.624131623,0,948.3584568,13.8051188,13.73743922,1.624131623,2.747
1,0,1,0,1,1,12,930.7987446,991.1770082,4.87239487,0,7.798250346,0,4,0,1.624131623,0,948.3584
39487,0,7.798250346,0,4,0,1.624131623,0,948.3584568,13.8051188,13.73743922,1.624131623,2.74
37,0,7.798250346,0,4,0,1.624131623,0,948.3584568,13.8051188,13.73743922,1.624131623,2.7474
0.7987446,991.1770082,4.87239487,0,7.798250346,0,4,0,1.624131623,0,948.3584568,13.8051188,
9487,0,7.798250346,0,4,0,1.624131623,0,948.3584568,13.8051188,13.73743922,1.624131623,2.747
0.7987446,991.1770082,4.87239487,0,7.798250346,0,4,0,1.624131623,0,948.3584568,13.8051188,
930.7987446,991.1770082,4.87239487,0,7.798250346,0,4,0,1.624131623,0,948.3584568,13.805118
39487,0,7.798250346,0,4,0,1.624131623,0,948.3584568,13.8051188,13.73743922,1.624131623,2.74
37,0,7.798250346,0,4,0,1.624131623,0,948.3584568,13.8051188,13.73743922,1.624131623,2.7474
0.7987446,991.1770082,4.87239487,0,7.798250346,0,4,0,1.624131623,0,948.3584568,13.8051188,
.7987446,991.1770082,4.87239487,0,7.798250346,0,4,0,1.624131623,0,948.3584568,13.8051188,1
.7987446,991.1770082,4.87239487,0,7.798250346,0,4,0,1.624131623,0,948.3584568,13.8051188,1
1,0,1,0,1,1,12,930.7987446,991.1770082,4.87239487,0,7.798250346,0,4,0,1.624131623,0,948.3584
,7.798250346,0,4,0,1.624131623,0,948.3584568,13.8051188,13.73743922,1.624131623,2.747487
487,0,7.798250346,0,4,0,1.624131623,0,948.3584568,13.8051188,13.73743922,1.624131623,2.747

370605,3,2,209,81,0,1,0,1,1,12,930.7987446,991.1770082,4.87239487,0,7.798250346,0,4,0,1.62413
1,0,1,0,1,1,12,930.7987446,991.1770082,4.87239487,0,7.798250346,0,4,0,1.624131623,0,948.3584
1,0,1,0,1,1,12,930.7987446,991.1770082,4.87239487,0,7.798250346,0,4,0,1.624131623,0,948.3584
37,0,7.798250346,0,4,0,1.624131623,0,948.3584568,13.8051188,13.73743922,1.624131623,2.7474
37,0,7.798250346,0,4,0,1.624131623,0,948.3584568,13.8051188,13.73743922,1.624131623,2.7474
,7.798250346,0,4,0,1.624131623,0,948.3584568,13.8051188,13.73743922,1.624131623,2.7474878
1,0,1,0,1,1,12,930.7987446,991.1770082,4.87239487,0,7.798250346,0,4,0,1.624131623,0,948.3584
30.7987446,991.1770082,4.87239487,0,7.798250346,0,4,0,1.624131623,0,948.3584568,13.805118
.7987446,991.1770082,4.87239487,0,7.798250346,0,4,0,1.624131623,0,948.3584568,13.8051188,1
.7987446,991.1770082,4.87239487,0,7.798250346,0,4,0,1.624131623,0,948.3584568,13.8051188,1
3,0,7.798250346,0,4,0,1.705987927,0,979.0953444,14.50089738,18.61962985,1.705987927,3.7239
66,1,1,0,1,1,12,926.2617828,991.1770082,5.11796378,0,7.798250346,0,4,0,1.705987927,0,979.095
66,1,1,0,1,1,12,926.2617828,991.1770082,5.11796378,0,7.798250346,0,4,0,1.705987927,0,979.095
26.2617828,991.1770082,5.11796378,0,7.798250346,0,4,0,1.705987927,0,979.0953444,14.500897
78,0,7.798250346,0,4,0,1.705987927,0,979.0953444,14.50089738,18.61962985,1.705987927,3.723
.2617828,991.1770082,5.11796378,0,7.798250346,0,4,0,1.705987927,0,979.0953444,14.50089738,
6,1,1,0,1,1,12,926.2617828,991.1770082,5.11796378,0,7.798250346,0,4,0,1.705987927,0,979.0953
6,1,1,0,1,1,12,926.2617828,991.1770082,5.11796378,0,7.798250346,0,4,0,1.705987927,0,979.0953
66,1,1,0,1,1,12,926.2617828,991.1770082,5.11796378,0,7.798250346,0,4,0,1.705987927,0,979.0953
26.2617828,991.1770082,5.11796378,0,7.798250346,0,4,0,1.705987927,0,979.0953444,14.500897
,1,1,0,1,1,12,926.2617828,991.1770082,5.11796378,0,7.798250346,0,4,0,1.705987927,0,979.09534
78,0,7.798250346,0,4,0,1.705987927,0,979.0953444,14.50089738,18.61962985,1.705987927,3.723
6.2617828,991.1770082,5.11796378,0,7.798250346,0,4,0,1.705987927,0,979.0953444,14.5008973
6.2617828,991.1770082,5.11796378,0,7.798250346,0,4,0,1.705987927,0,979.0953444,14.5008973
66,1,1,0,1,1,12,926.2617828,991.1770082,5.11796378,0,7.798250346,0,4,0,1.705987927,0,979.095
6,1,1,0,1,1,12,926.2617828,991.1770082,5.11796378,0,7.798250346,0,4,0,1.705987927,0,979.0953
6,1,1,0,1,1,12,926.2617828,991.1770082,5.11796378,0,7.798250346,0,4,0,1.705987927,0,979.0953
6.2617828,991.1770082,5.11796378,0,7.798250346,0,4,0,1.705987927,0,979.0953444,14.5008973
78,0,7.798250346,0,4,0,1.705987927,0,979.0953444,14.50089738,18.61962985,1.705987927,3.723
0154419,3,2,209,66,1,1,0,1,1,12,926.2617828,991.1770082,5.11796378,0,7.798250346,0,4,0,1.7059
6.2617828,991.1770082,5.11796378,0,7.798250346,0,4,0,1.705987927,0,979.0953444,14.5008973
6.2617828,991.1770082,5.11796378,0,7.798250346,0,4,0,1.705987927,0,979.0953444,14.5008973
78,0,7.798250346,0,4,0,1.705987927,0,979.0953444,14.50089738,18.61962985,1.705987927,3.723
6.2617828,991.1770082,5.11796378,0,7.798250346,0,4,0,1.705987927,0,979.0953444,14.5008973
2617828,991.1770082,5.11796378,0,7.798250346,0,4,0,1.705987927,0,979.0953444,14.50089738,1
6,1,1,0,1,1,12,926.2617828,991.1770082,5.11796378,0,7.798250346,0,4,0,1.705987927,0,979.0953
6.2617828,991.1770082,5.11796378,0,7.798250346,0,4,0,1.705987927,0,979.0953444,14.5008973
78,0,7.798250346,0,4,0,1.705987927,0,979.0953444,14.50089738,18.61962985,1.705987927,3.723
78,0,7.798250346,0,4,0,1.705987927,0,979.0953444,14.50089738,18.61962985,1.705987927,3.723
,1,1,0,1,1,12,926.2617828,991.1770082,5.11796378,0,7.798250346,0,4,0,1.705987927,0,979.09534
66,1,1,0,1,1,12,926.2617828,991.1770082,5.11796378,0,7.798250346,0,4,0,1.705987927,0,979.0953
78,0,7.798250346,0,4,0,1.705987927,0,979.0953444,14.50089738,18.61962985,1.705987927,3.723
.2617828,991.1770082,5.11796378,0,7.798250346,0,4,0,1.705987927,0,979.0953444,14.50089738,
66,1,1,0,1,1,12,926.2617828,991.1770082,5.11796378,0,7.798250346,0,4,0,1.705987927,0,979.095
0154419,3,2,209,66,1,1,0,1,1,12,926.2617828,991.1770082,5.11796378,0,7.798250346,0,4,0,1.7059
6,1,1,0,1,1,12,926.2617828,991.1770082,5.11796378,0,7.798250346,0,4,0,1.705987927,0,979.0953
6.2617828,991.1770082,5.11796378,0,7.798250346,0,4,0,1.705987927,0,979.0953444,14.5008973
6.2617828,991.1770082,5.11796378,0,7.798250346,0,4,0,1.705987927,0,979.0953444,14.5008973
6.2617828,991.1770082,5.11796378,0,7.798250346,0,4,0,1.705987927,0,979.0953444,14.5008973
2617828,991.1770082,5.11796378,0,7.798250346,0,4,0,1.705987927,0,979.0953444,14.50089738,1

6.2617828,991.1770082,5.11796378,0,7.798250346,0,4,0,1.705987927,0,979.0953444,14.5008973
6,1,1,0,1,1,12,926.2617828,991.1770082,5.11796378,0,7.798250346,0,4,0,1.705987927,0,979.0953
6.2617828,991.1770082,5.11796378,0,7.798250346,0,4,0,1.705987927,0,979.0953444,14.5008973
6.2617828,991.1770082,5.11796378,0,7.798250346,0,4,0,1.705987927,0,979.0953444,14.5008973
517676,3,2,210,74,1,1,0,1,1,9,2927.93979,3071.899213,3.789943871,4.797249814,10.68658397,10.
.93979,3071.899213,3.789943871,4.797249814,10.68658397,10.5424273,2,1,6.632401774,19.1889
4,1,1,0,1,1,9,2927.93979,3071.899213,3.789943871,4.797249814,10.68658397,10.5424273,2,1,6.6
4,1,1,0,1,1,9,2927.93979,3071.899213,3.789943871,4.797249814,10.68658397,10.5424273,2,1,6.6
,74,1,1,0,1,1,9,2927.93979,3071.899213,3.789943871,4.797249814,10.68658397,10.5424273,2,1,6
.93979,3071.899213,3.789943871,4.797249814,10.68658397,10.5424273,2,1,6.632401774,19.1889
.93979,3071.899213,3.789943871,4.797249814,10.68658397,10.5424273,2,1,6.632401774,19.1889
7.93979,3071.899213,3.789943871,4.797249814,10.68658397,10.5424273,2,1,6.632401774,19.188
.93979,3071.899213,3.789943871,4.797249814,10.68658397,10.5424273,2,1,6.632401774,19.1889
93979,3071.899213,3.789943871,4.797249814,10.68658397,10.5424273,2,1,6.632401774,19.18899
4,1,1,0,1,1,9,2927.93979,3071.899213,3.789943871,4.797249814,10.68658397,10.5424273,2,1,6.6
7.93979,3071.899213,3.789943871,4.797249814,10.68658397,10.5424273,2,1,6.632401774,19.188
4,1,1,0,1,1,9,2927.93979,3071.899213,3.789943871,4.797249814,10.68658397,10.5424273,2,1,6.6
,4.797249814,10.68658397,10.5424273,2,1,6.632401774,19.18899926,270.5364,49.26927032,52.7
0308838,3,2,210,74,1,1,0,1,1,9,2927.93979,3071.899213,3.789943871,4.797249814,10.68658397,1
.93979,3071.899213,3.789943871,4.797249814,10.68658397,10.5424273,2,1,6.632401774,19.1889
27.93979,3071.899213,3.789943871,4.797249814,10.68658397,10.5424273,2,1,6.632401774,19.18
27.93979,3071.899213,3.789943871,4.797249814,10.68658397,10.5424273,2,1,6.632401774,19.18
74,1,1,0,1,1,9,2927.93979,3071.899213,3.789943871,4.797249814,10.68658397,10.5424273,2,1,6.
7.93979,3071.899213,3.789943871,4.797249814,10.68658397,10.5424273,2,1,6.632401774,19.188
,1,1,0,1,1,9,2927.93979,3071.899213,3.789943871,4.797249814,10.68658397,10.5424273,2,1,6.63
,4.797249814,10.68658397,10.5424273,2,1,6.632401774,19.18899926,270.5364,49.26927032,52.7
7.93979,3071.899213,3.789943871,4.797249814,10.68658397,10.5424273,2,1,6.632401774,19.188
74,1,1,0,1,1,9,2927.93979,3071.899213,3.789943871,4.797249814,10.68658397,10.5424273,2,1,6.
.93979,3071.899213,3.789943871,4.797249814,10.68658397,10.5424273,2,1,6.632401774,19.1889
27.93979,3071.899213,3.789943871,4.797249814,10.68658397,10.5424273,2,1,6.632401774,19.18
.93979,3071.899213,3.789943871,4.797249814,10.68658397,10.5424273,2,1,6.632401774,19.1889
4,1,1,0,1,1,9,2927.93979,3071.899213,3.789943871,4.797249814,10.68658397,10.5424273,2,1,6.6
.93979,3071.899213,3.789943871,4.797249814,10.68658397,10.5424273,2,1,6.632401774,19.1889
.93979,3071.899213,3.789943871,4.797249814,10.68658397,10.5424273,2,1,6.632401774,19.1889
4,1,1,0,1,1,9,2927.93979,3071.899213,3.789943871,4.797249814,10.68658397,10.5424273,2,1,6.6
,4.797249814,10.68658397,10.5424273,2,1,6.632401774,19.18899926,270.5364,49.26927032,52.7
7.93979,3071.899213,3.789943871,4.797249814,10.68658397,10.5424273,2,1,6.632401774,19.188
.93979,3071.899213,3.789943871,4.797249814,10.68658397,10.5424273,2,1,6.632401774,19.1889
7.93979,3071.899213,3.789943871,4.797249814,10.68658397,10.5424273,2,1,6.632401774,19.188
7.93979,3071.899213,3.789943871,4.797249814,10.68658397,10.5424273,2,1,6.632401774,19.188
7.93979,3071.899213,3.789943871,4.797249814,10.68658397,10.5424273,2,1,6.632401774,19.188
4,1,1,0,1,1,9,2927.93979,3071.899213,3.789943871,4.797249814,10.68658397,10.5424273,2,1,6.6
,4.797249814,10.68658397,10.5424273,2,1,6.632401774,19.18899926,270.5364,49.26927032,52.7
,0,10.68658397,0,2,0,7.997152826,7.604851999,287.3988754,65.31008141,45.62911199,17.32716
9,0,10.68658397,0,2,0,7.997152826,7.604851999,287.3988754,65.31008141,45.62911199,17.3271
7,0,1,0,1,1,9,2798.385313,5615.175703,2.665717609,0,10.68658397,0,2,0,7.997152826,7.604851
8.385313,5615.175703,2.665717609,0,10.68658397,0,2,0,7.997152826,7.604851999,287.3988754,
8.385313,5615.175703,2.665717609,0,10.68658397,0,2,0,7.997152826,7.604851999,287.3988754,
98.385313,5615.175703,2.665717609,0,10.68658397,0,2,0,7.997152826,7.604851999,287.3988754
8.385313,5615.175703,2.665717609,0,10.68658397,0,2,0,7.997152826,7.604851999,287.3988754,

7,0,1,0,1,1,9,2798.385313,5615.175703,2.665717609,0,10.68658397,0,2,0,7.997152826,7.6048519
.385313,5615.175703,2.665717609,0,10.68658397,0,2,0,7.997152826,7.604851999,287.3988754,6
154419,3,2,210,77,0,1,0,1,1,9,2798.385313,5615.175703,2.665717609,0,10.68658397,0,2,0,7.99715
8.385313,5615.175703,2.665717609,0,10.68658397,0,2,0,7.997152826,7.604851999,287.3988754,
.385313,5615.175703,2.665717609,0,10.68658397,0,2,0,7.997152826,7.604851999,287.3988754,6
98.385313,5615.175703,2.665717609,0,10.68658397,0,2,0,7.997152826,7.604851999,287.3988754
8.385313,5615.175703,2.665717609,0,10.68658397,0,2,0,7.997152826,7.604851999,287.3988754,
98.385313,5615.175703,2.665717609,0,10.68658397,0,2,0,7.997152826,7.604851999,287.3988754
.385313,5615.175703,2.665717609,0,10.68658397,0,2,0,7.997152826,7.604851999,287.3988754,6
8.385313,5615.175703,2.665717609,0,10.68658397,0,2,0,7.997152826,7.604851999,287.3988754,
.385313,5615.175703,2.665717609,0,10.68658397,0,2,0,7.997152826,7.604851999,287.3988754,6
617676,3,2,210,77,0,1,0,1,1,9,2798.385313,5615.175703,2.665717609,0,10.68658397,0,2,0,7.9971
.385313,5615.175703,2.665717609,0,10.68658397,0,2,0,7.997152826,7.604851999,287.3988754,6
7,0,1,0,1,1,9,2798.385313,5615.175703,2.665717609,0,10.68658397,0,2,0,7.997152826,7.6048519
9,0,10.68658397,0,2,0,7.997152826,7.604851999,287.3988754,65.31008141,45.62911199,17.3271
98.385313,5615.175703,2.665717609,0,10.68658397,0,2,0,7.997152826,7.604851999,287.3988754
9,0,10.68658397,0,2,0,7.997152826,7.604851999,287.3988754,65.31008141,45.62911199,17.3271
154419,3,2,210,77,0,1,0,1,1,9,2798.385313,5615.175703,2.665717609,0,10.68658397,0,2,0,7.9971
77,0,1,0,1,1,9,2798.385313,5615.175703,2.665717609,0,10.68658397,0,2,0,7.997152826,7.604851
7,0,1,0,1,1,9,2798.385313,5615.175703,2.665717609,0,10.68658397,0,2,0,7.997152826,7.6048519
9,0,10.68658397,0,2,0,7.997152826,7.604851999,287.3988754,65.31008141,45.62911199,17.3271
382324,3,2,210,77,0,1,0,1,1,9,2798.385313,5615.175703,2.665717609,0,10.68658397,0,2,0,7.99715
8.385313,5615.175703,2.665717609,0,10.68658397,0,2,0,7.997152826,7.604851999,287.3988754,
.385313,5615.175703,2.665717609,0,10.68658397,0,2,0,7.997152826,7.604851999,287.3988754,6
.385313,5615.175703,2.665717609,0,10.68658397,0,2,0,7.997152826,7.604851999,287.3988754,6
98.385313,5615.175703,2.665717609,0,10.68658397,0,2,0,7.997152826,7.604851999,287.3988754
8.385313,5615.175703,2.665717609,0,10.68658397,0,2,0,7.997152826,7.604851999,287.3988754,
.385313,5615.175703,2.665717609,0,10.68658397,0,2,0,7.997152826,7.604851999,287.3988754,6
.385313,5615.175703,2.665717609,0,10.68658397,0,2,0,7.997152826,7.604851999,287.3988754,6
98.385313,5615.175703,2.665717609,0,10.68658397,0,2,0,7.997152826,7.604851999,287.3988754
.385313,5615.175703,2.665717609,0,10.68658397,0,2,0,7.997152826,7.604851999,287.3988754,6
9,0,10.68658397,0,2,0,7.997152826,7.604851999,287.3988754,65.31008141,45.62911199,17.3271
7,0,1,0,1,1,10,2798.385313,5615.175703,2.665717609,0,10.68658397,0,2,0,7.997152826,7.604851
77,0,1,0,1,1,10,2798.385313,5615.175703,2.665717609,0,10.68658397,0,2,0,7.997152826,7.60485
8.385313,5615.175703,2.665717609,0,10.68658397,0,2,0,7.997152826,7.604851999,287.3988754,
798.385313,5615.175703,2.665717609,0,10.68658397,0,2,0,7.997152826,7.604851999,287.398875
7,0,1,0,1,1,10,2798.385313,5615.175703,2.665717609,0,10.68658397,0,2,0,7.997152826,7.604851
8.385313,5615.175703,2.665717609,0,10.68658397,0,2,0,7.997152826,7.604851999,287.3988754,
798.385313,5615.175703,2.665717609,0,10.68658397,0,2,0,7.997152826,7.604851999,287.398875
7,0,1,0,1,1,10,2798.385313,5615.175703,2.665717609,0,10.68658397,0,2,0,7.997152826,7.604851
8.385313,5615.175703,2.665717609,0,10.68658397,0,2,0,7.997152826,7.604851999,287.3988754,
98.385313,5615.175703,2.665717609,0,10.68658397,0,2,0,7.997152826,7.604851999,287.3988754
7,0,1,0,1,1,10,2798.385313,5615.175703,2.665717609,0,10.68658397,0,2,0,7.997152826,7.604851
8.385313,5615.175703,2.665717609,0,10.68658397,0,2,0,7.997152826,7.604851999,287.3988754,
7,0,1,0,1,1,10,2798.385313,5615.175703,2.665717609,0,10.68658397,0,2,0,7.997152826,7.604851
798.385313,5615.175703,2.665717609,0,10.68658397,0,2,0,7.997152826,7.604851999,287.398875
8.385313,5615.175703,2.665717609,0,10.68658397,0,2,0,7.997152826,7.604851999,287.3988754,
98.385313,5615.175703,2.665717609,0,10.68658397,0,2,0,7.997152826,7.604851999,287.3988754
798.385313,5615.175703,2.665717609,0,10.68658397,0,2,0,7.997152826,7.604851999,287.398875
98.385313,5615.175703,2.665717609,0,10.68658397,0,2,0,7.997152826,7.604851999,287.3988754

798.385313,5615.175703,2.665717609,0,10.68658397,0,2,0,7.997152826,7.604851999,287.398875
09,0,10.68658397,0,2,0,7.997152826,7.604851999,287.3988754,65.31008141,45.62911199,17.327
98.385313,5615.175703,2.665717609,0,10.68658397,0,2,0,7.997152826,7.604851999,287.3988754
8.385313,5615.175703,2.665717609,0,10.68658397,0,2,0,7.997152826,7.604851999,287.3988754,
798.385313,5615.175703,2.665717609,0,10.68658397,0,2,0,7.997152826,7.604851999,287.398875
98.385313,5615.175703,2.665717609,0,10.68658397,0,2,0,7.997152826,7.604851999,287.3988754
382324,3,2,210,68,0,1,0,1,1,9,1581.717696,1731.878306,2.319614214,5.931154457,6.951031909,
4,5.931154457,6.951031909,6.951031909,1,1,4.639228428,0,583.5918747,71.90804064,41.51808
81.717696,1731.878306,2.319614214,5.931154457,6.951031909,6.951031909,1,1,4.639228428,0,5
1.717696,1731.878306,2.319614214,5.931154457,6.951031909,6.951031909,1,1,4.639228428,0,58
68,0,1,0,1,1,9,1581.717696,1731.878306,2.319614214,5.931154457,6.951031909,6.951031909,1,1,
1.717696,1731.878306,2.319614214,5.931154457,6.951031909,6.951031909,1,1,4.639228428,0,5
81.717696,1731.878306,2.319614214,5.931154457,6.951031909,6.951031909,1,1,4.639228428,0,5
1.717696,1731.878306,2.319614214,5.931154457,6.951031909,6.951031909,1,1,4.639228428,0,58
4,5.931154457,6.951031909,6.951031909,1,1,4.639228428,0,583.5918747,71.90804064,41.51808
.717696,1731.878306,2.319614214,5.931154457,6.951031909,6.951031909,1,1,4.639228428,0,583
81.717696,1731.878306,2.319614214,5.931154457,6.951031909,6.951031909,1,1,4.639228428,0,5
614214,5.931154457,6.951031909,6.951031909,1,1,4.639228428,0,583.5918747,71.90804064,41.
1.717696,1731.878306,2.319614214,5.931154457,6.951031909,6.951031909,1,1,4.639228428,0,5
81.717696,1731.878306,2.319614214,5.931154457,6.951031909,6.951031909,1,1,4.639228428,0,5
214,5.931154457,6.951031909,6.951031909,1,1,4.639228428,0,583.5918747,71.90804064,41.518
.717696,1731.878306,2.319614214,5.931154457,6.951031909,6.951031909,1,1,4.639228428,0,583
8,0,1,0,1,1,9,1581.717696,1731.878306,2.319614214,5.931154457,6.951031909,6.951031909,1,1,4
81.717696,1731.878306,2.319614214,5.931154457,6.951031909,6.951031909,1,1,4.639228428,0,5
81.717696,1731.878306,2.319614214,5.931154457,6.951031909,6.951031909,1,1,4.639228428,0,5
617676,3,2,210,68,0,1,0,1,1,9,1581.717696,1731.878306,2.319614214,5.931154457,6.951031909,
4214,5.931154457,6.951031909,6.951031909,1,1,4.639228428,0,583.5918747,71.90804064,41.51
.717696,1731.878306,2.319614214,5.931154457,6.951031909,6.951031909,1,1,4.639228428,0,583
1.717696,1731.878306,2.319614214,5.931154457,6.951031909,6.951031909,1,1,4.639228428,0,58
81.717696,1731.878306,2.319614214,5.931154457,6.951031909,6.951031909,1,1,4.639228428,0,5
4,5.931154457,6.951031909,6.951031909,1,1,4.639228428,0,583.5918747,71.90804064,41.51808
.717696,1731.878306,2.319614214,5.931154457,6.951031909,6.951031909,1,1,4.639228428,0,583
81.717696,1731.878306,2.319614214,5.931154457,6.951031909,6.951031909,1,1,4.639228428,0,5
4,5.931154457,6.951031909,6.951031909,1,1,4.639228428,0,583.5918747,71.90804064,41.51808
8,0,1,0,1,1,12,2968.891659,3289.410553,3.816546022,4.73789714,10.68658397,10.5424273,2,1,6.
22,4.73789714,10.68658397,10.5424273,2,1,6.678955538,18.95158856,257.0536626,48.66096178
68.891659,3289.410553,3.816546022,4.73789714,10.68658397,10.5424273,2,1,6.678955538,18.95
68.891659,3289.410553,3.816546022,4.73789714,10.68658397,10.5424273,2,1,6.678955538,18.95
8.891659,3289.410553,3.816546022,4.73789714,10.68658397,10.5424273,2,1,6.678955538,18.951
68.891659,3289.410553,3.816546022,4.73789714,10.68658397,10.5424273,2,1,6.678955538,18.95
22,4.73789714,10.68658397,10.5424273,2,1,6.678955538,18.95158856,257.0536626,48.66096178
68.891659,3289.410553,3.816546022,4.73789714,10.68658397,10.5424273,2,1,6.678955538,18.95
68.891659,3289.410553,3.816546022,4.73789714,10.68658397,10.5424273,2,1,6.678955538,18.95
68.891659,3289.410553,3.816546022,4.73789714,10.68658397,10.5424273,2,1,6.678955538,18.95
8.891659,3289.410553,3.816546022,4.73789714,10.68658397,10.5424273,2,1,6.678955538,18.951
68.891659,3289.410553,3.816546022,4.73789714,10.68658397,10.5424273,2,1,6.678955538,18.95
8.891659,3289.410553,3.816546022,4.73789714,10.68658397,10.5424273,2,1,6.678955538,18.951
8.891659,3289.410553,3.816546022,4.73789714,10.68658397,10.5424273,2,1,6.678955538,18.951
68.891659,3289.410553,3.816546022,4.73789714,10.68658397,10.5424273,2,1,6.678955538,18.95
68.891659,3289.410553,3.816546022,4.73789714,10.68658397,10.5424273,2,1,6.678955538,18.95

968.891659,3289.410553,3.816546022,4.73789714,10.68658397,10.5424273,2,1,6.678955538,18.9
68.891659,3289.410553,3.816546022,4.73789714,10.68658397,10.5424273,2,1,6.678955538,18.95
8,0,1,0,1,1,12,2968.891659,3289.410553,3.816546022,4.73789714,10.68658397,10.5424273,2,1,6.
8,0,1,0,1,1,12,2968.891659,3289.410553,3.816546022,4.73789714,10.68658397,10.5424273,2,1,6.
8,0,1,0,1,1,12,2968.891659,3289.410553,3.816546022,4.73789714,10.68658397,10.5424273,2,1,6.
8.891659,3289.410553,3.816546022,4.73789714,10.68658397,10.5424273,2,1,6.678955538,18.951
.891659,3289.410553,3.816546022,4.73789714,10.68658397,10.5424273,2,1,6.678955538,18.9515
68.891659,3289.410553,3.816546022,4.73789714,10.68658397,10.5424273,2,1,6.678955538,18.95
68,0,1,0,1,1,12,2968.891659,3289.410553,3.816546022,4.73789714,10.68658397,10.5424273,2,1,6
8.891659,3289.410553,3.816546022,4.73789714,10.68658397,10.5424273,2,1,6.678955538,18.951
68.891659,3289.410553,3.816546022,4.73789714,10.68658397,10.5424273,2,1,6.678955538,18.95
8.891659,3289.410553,3.816546022,4.73789714,10.68658397,10.5424273,2,1,6.678955538,18.951
68.891659,3289.410553,3.816546022,4.73789714,10.68658397,10.5424273,2,1,6.678955538,18.95
68.891659,3289.410553,3.816546022,4.73789714,10.68658397,10.5424273,2,1,6.678955538,18.95
68.891659,3289.410553,3.816546022,4.73789714,10.68658397,10.5424273,2,1,6.678955538,18.95
8,0,1,0,1,1,12,2968.891659,3289.410553,3.816546022,4.73789714,10.68658397,10.5424273,2,1,6.
793,4.506871037,7.192668516,6.951031909,3,1,8.998145586,9.013742074,318.097831,74.9845465
00.527392,1604.306334,4.499072793,4.506871037,7.192668516,6.951031909,3,1,8.998145586,9.0
00.527392,1604.306334,4.499072793,4.506871037,7.192668516,6.951031909,3,1,8.998145586,9.0
072793,4.506871037,7.192668516,6.951031909,3,1,8.998145586,9.013742074,318.097831,74.9845
93,4.506871037,7.192668516,6.951031909,3,1,8.998145586,9.013742074,318.097831,74.98454655
00.527392,1604.306334,4.499072793,4.506871037,7.192668516,6.951031909,3,1,8.998145586,9.0
00.527392,1604.306334,4.499072793,4.506871037,7.192668516,6.951031909,3,1,8.998145586,9.0
499072793,4.506871037,7.192668516,6.951031909,3,1,8.998145586,9.013742074,318.097831,74.
793,4.506871037,7.192668516,6.951031909,3,1,8.998145586,9.013742074,318.097831,74.9845465
7,0,1,0,1,1,12,1800.527392,1604.306334,4.499072793,4.506871037,7.192668516,6.951031909,3,1,
793,4.506871037,7.192668516,6.951031909,3,1,8.998145586,9.013742074,318.097831,74.9845465
0.527392,1604.306334,4.499072793,4.506871037,7.192668516,6.951031909,3,1,8.998145586,9.01
793,4.506871037,7.192668516,6.951031909,3,1,8.998145586,9.013742074,318.097831,74.9845465
99072793,4.506871037,7.192668516,6.951031909,3,1,8.998145586,9.013742074,318.097831,74.98
793,4.506871037,7.192668516,6.951031909,3,1,8.998145586,9.013742074,318.097831,74.9845465
800.527392,1604.306334,4.499072793,4.506871037,7.192668516,6.951031909,3,1,8.998145586,9.
800.527392,1604.306334,4.499072793,4.506871037,7.192668516,6.951031909,3,1,8.998145586,9.
67,0,1,0,1,1,12,1800.527392,1604.306334,4.499072793,4.506871037,7.192668516,6.951031909,3,
800.527392,1604.306334,4.499072793,4.506871037,7.192668516,6.951031909,3,1,8.998145586,9.
793,4.506871037,7.192668516,6.951031909,3,1,8.998145586,9.013742074,318.097831,74.9845465
00.527392,1604.306334,4.499072793,4.506871037,7.192668516,6.951031909,3,1,8.998145586,9.0
00.527392,1604.306334,4.499072793,4.506871037,7.192668516,6.951031909,3,1,8.998145586,9.0
00.527392,1604.306334,4.499072793,4.506871037,7.192668516,6.951031909,3,1,8.998145586,9.0
67,0,1,0,1,1,12,1800.527392,1604.306334,4.499072793,4.506871037,7.192668516,6.951031909,3,
0.527392,1604.306334,4.499072793,4.506871037,7.192668516,6.951031909,3,1,8.998145586,9.01
7,0,1,0,1,1,12,1800.527392,1604.306334,4.499072793,4.506871037,7.192668516,6.951031909,3,1,
9072793,4.506871037,7.192668516,6.951031909,3,1,8.998145586,9.013742074,318.097831,74.984
072793,4.506871037,7.192668516,6.951031909,3,1,8.998145586,9.013742074,318.097831,74.9845
072793,4.506871037,7.192668516,6.951031909,3,1,8.998145586,9.013742074,318.097831,74.9845
00.66293,4548.083033,2.658589063,0,10.68658397,0,2,0,9.305061719,0,437.1424751,59.8182539
0.66293,4548.083033,2.658589063,0,10.68658397,0,2,0,9.305061719,0,437.1424751,59.81825391,
53,0,10.68658397,0,2,0,9.305061719,0,437.1424751,59.81825391,51.48669138,17.28082891,34.32
7,0,1,0,1,1,12,2900.66293,4548.083033,2.658589063,0,10.68658397,0,2,0,9.305061719,0,437.1424
9382324,3,2,210,77,0,1,0,1,1,12,2900.66293,4548.083033,2.658589063,0,10.68658397,0,2,0,9.3050

63,0,10.68658397,0,2,0,9.305061719,0,437.1424751,59.81825391,51.48669138,17.28082891,34.324
00.66293,4548.083033,2.658589063,0,10.68658397,0,2,0,9.305061719,0,437.1424751,59.81825391
63,0,10.68658397,0,2,0,9.305061719,0,437.1424751,59.81825391,51.48669138,17.28082891,34.324
00.66293,4548.083033,2.658589063,0,10.68658397,0,2,0,9.305061719,0,437.1424751,59.81825391
7,0,1,0,1,1,12,2900.66293,4548.083033,2.658589063,0,10.68658397,0,2,0,9.305061719,0,437.1424
63,0,10.68658397,0,2,0,9.305061719,0,437.1424751,59.81825391,51.48669138,17.28082891,34.324
63,0,10.68658397,0,2,0,9.305061719,0,437.1424751,59.81825391,51.48669138,17.28082891,34.324
00.66293,4548.083033,2.658589063,0,10.68658397,0,2,0,9.305061719,0,437.1424751,59.81825391
0.66293,4548.083033,2.658589063,0,10.68658397,0,2,0,9.305061719,0,437.1424751,59.81825391,
63,0,10.68658397,0,2,0,9.305061719,0,437.1424751,59.81825391,51.48669138,17.28082891,34.324
89063,0,10.68658397,0,2,0,9.305061719,0,437.1424751,59.81825391,51.48669138,17.28082891,34
2900.66293,4548.083033,2.658589063,0,10.68658397,0,2,0,9.305061719,0,437.1424751,59.818253
58589063,0,10.68658397,0,2,0,9.305061719,0,437.1424751,59.81825391,51.48669138,17.2808289
00.66293,4548.083033,2.658589063,0,10.68658397,0,2,0,9.305061719,0,437.1424751,59.81825391
63,0,10.68658397,0,2,0,9.305061719,0,437.1424751,59.81825391,51.48669138,17.28082891,34.324
63,0,10.68658397,0,2,0,9.305061719,0,437.1424751,59.81825391,51.48669138,17.28082891,34.324
900.66293,4548.083033,2.658589063,0,10.68658397,0,2,0,9.305061719,0,437.1424751,59.8182539
00.66293,4548.083033,2.658589063,0,10.68658397,0,2,0,9.305061719,0,437.1424751,59.81825391
00.66293,4548.083033,2.658589063,0,10.68658397,0,2,0,9.305061719,0,437.1424751,59.81825391
7,0,1,0,1,1,12,2900.66293,4548.083033,2.658589063,0,10.68658397,0,2,0,9.305061719,0,437.1424
63,0,10.68658397,0,2,0,9.305061719,0,437.1424751,59.81825391,51.48669138,17.28082891,34.324
7,0,1,0,1,1,12,2900.66293,4548.083033,2.658589063,0,10.68658397,0,2,0,9.305061719,0,437.1424
7,0,1,0,1,1,12,2900.66293,4548.083033,2.658589063,0,10.68658397,0,2,0,9.305061719,0,437.1424
89063,0,10.68658397,0,2,0,9.305061719,0,437.1424751,59.81825391,51.48669138,17.28082891,34
89063,0,10.68658397,0,2,0,9.305061719,0,437.1424751,59.81825391,51.48669138,17.28082891,34
.491757375,0,10.68658397,0,2,0,8.721150812,5.966160793,224.659089,62.29393437,59.66160793
6,1,1,1,1,1,8,2795.864645,4992.769149,2.491757375,0,10.68658397,0,2,0,8.721150812,5.9661607
795.864645,4992.769149,2.491757375,0,10.68658397,0,2,0,8.721150812,5.966160793,224.659089,
.864645,4992.769149,2.491757375,0,10.68658397,0,2,0,8.721150812,5.966160793,224.659089,62.
0617676,3,2,210,86,1,1,1,1,1,8,2795.864645,4992.769149,2.491757375,0,10.68658397,0,2,0,8.7211
75,0,10.68658397,0,2,0,8.721150812,5.966160793,224.659089,62.29393437,59.66160793,17.44230
91757375,0,10.68658397,0,2,0,8.721150812,5.966160793,224.659089,62.29393437,59.66160793,1
6,1,1,1,1,1,8,2795.864645,4992.769149,2.491757375,0,10.68658397,0,2,0,8.721150812,5.9661607
5.864645,4992.769149,2.491757375,0,10.68658397,0,2,0,8.721150812,5.966160793,224.659089,62
.864645,4992.769149,2.491757375,0,10.68658397,0,2,0,8.721150812,5.966160793,224.659089,62.
57375,0,10.68658397,0,2,0,8.721150812,5.966160793,224.659089,62.29393437,59.66160793,17.44
.864645,4992.769149,2.491757375,0,10.68658397,0,2,0,8.721150812,5.966160793,224.659089,62.
1757375,0,10.68658397,0,2,0,8.721150812,5.966160793,224.659089,62.29393437,59.66160793,17.
1757375,0,10.68658397,0,2,0,8.721150812,5.966160793,224.659089,62.29393437,59.66160793,17.
75,0,10.68658397,0,2,0,8.721150812,5.966160793,224.659089,62.29393437,59.66160793,17.44230
57375,0,10.68658397,0,2,0,8.721150812,5.966160793,224.659089,62.29393437,59.66160793,17.44
5.864645,4992.769149,2.491757375,0,10.68658397,0,2,0,8.721150812,5.966160793,224.659089,62
5.864645,4992.769149,2.491757375,0,10.68658397,0,2,0,8.721150812,5.966160793,224.659089,62
1757375,0,10.68658397,0,2,0,8.721150812,5.966160793,224.659089,62.29393437,59.66160793,17.
6,1,1,1,1,1,8,2795.864645,4992.769149,2.491757375,0,10.68658397,0,2,0,8.721150812,5.9661607
57375,0,10.68658397,0,2,0,8.721150812,5.966160793,224.659089,62.29393437,59.66160793,17.44
.864645,4992.769149,2.491757375,0,10.68658397,0,2,0,8.721150812,5.966160793,224.659089,62.
.864645,4992.769149,2.491757375,0,10.68658397,0,2,0,8.721150812,5.966160793,224.659089,62.
1757375,0,10.68658397,0,2,0,8.721150812,5.966160793,224.659089,62.29393437,59.66160793,17.
5.864645,4992.769149,2.491757375,0,10.68658397,0,2,0,8.721150812,5.966160793,224.659089,62

91757375,0,10.68658397,0,2,0,8.721150812,5.966160793,224.659089,62.29393437,59.66160793,17
.864645,4992.769149,2.491757375,0,10.68658397,0,2,0,8.721150812,5.966160793,224.659089,62.
.864645,4992.769149,2.491757375,0,10.68658397,0,2,0,8.721150812,5.966160793,224.659089,62.
.864645,4992.769149,2.491757375,0,10.68658397,0,2,0,8.721150812,5.966160793,224.659089,62.
.864645,4992.769149,2.491757375,0,10.68658397,0,2,0,8.721150812,5.966160793,224.659089,62.
86,1,1,1,1,1,8,2795.864645,4992.769149,2.491757375,0,10.68658397,0,2,0,8.721150812,5.966160
6,1,1,1,1,1,8,2795.864645,4992.769149,2.491757375,0,10.68658397,0,2,0,8.721150812,5.9661607
86,1,1,1,1,1,8,2795.864645,4992.769149,2.491757375,0,10.68658397,0,2,0,8.721150812,5.966160
86,1,1,1,1,1,8,2795.864645,4992.769149,2.491757375,0,10.68658397,0,2,0,8.721150812,5.966160
95.864645,4992.769149,2.491757375,0,10.68658397,0,2,0,8.721150812,5.966160793,224.659089,6
75,0,10.68658397,0,2,0,8.721150812,5.966160793,224.659089,62.29393437,59.66160793,17.44230
1757375,0,10.68658397,0,2,0,8.721150812,5.966160793,224.659089,62.29393437,59.66160793,17.
5,0,10.68658397,0,2,0,8.721150812,5.966160793,224.659089,62.29393437,59.66160793,17.442301
5.864645,4992.769149,2.491757375,0,10.68658397,0,2,0,8.721150812,5.966160793,224.659089,62
.864645,4992.769149,2.491757375,0,10.68658397,0,2,0,8.721150812,5.966160793,224.659089,62.
3,0,10.68658397,0,2,0,8.535961499,5.482382777,175.4893329,65.84884585,60.30621054,17.07192
46143,0,10.68658397,0,2,0,8.535961499,5.482382777,175.4893329,65.84884585,60.30621054,17.0
3.034449,4670.450585,2.438846143,0,10.68658397,0,2,0,8.535961499,5.482382777,175.4893329,6
3.034449,4670.450585,2.438846143,0,10.68658397,0,2,0,8.535961499,5.482382777,175.4893329,6
.034449,4670.450585,2.438846143,0,10.68658397,0,2,0,8.535961499,5.482382777,175.4893329,65
43,0,10.68658397,0,2,0,8.535961499,5.482382777,175.4893329,65.84884585,60.30621054,17.0719
76,0,1,0,1,1,9,2743.034449,4670.450585,2.438846143,0,10.68658397,0,2,0,8.535961499,5.482382
3.034449,4670.450585,2.438846143,0,10.68658397,0,2,0,8.535961499,5.482382777,175.4893329,6
3.034449,4670.450585,2.438846143,0,10.68658397,0,2,0,8.535961499,5.482382777,175.4893329,6
76,0,1,0,1,1,9,2743.034449,4670.450585,2.438846143,0,10.68658397,0,2,0,8.535961499,5.482382
0,10.68658397,0,2,0,8.535961499,5.482382777,175.4893329,65.84884585,60.30621054,17.071923
3.034449,4670.450585,2.438846143,0,10.68658397,0,2,0,8.535961499,5.482382777,175.4893329,6
6,0,1,0,1,1,9,2743.034449,4670.450585,2.438846143,0,10.68658397,0,2,0,8.535961499,5.4823827
.034449,4670.450585,2.438846143,0,10.68658397,0,2,0,8.535961499,5.482382777,175.4893329,65
.034449,4670.450585,2.438846143,0,10.68658397,0,2,0,8.535961499,5.482382777,175.4893329,65
517676,3,2,210,76,0,1,0,1,1,9,2743.034449,4670.450585,2.438846143,0,10.68658397,0,2,0,8.53596
38846143,0,10.68658397,0,2,0,8.535961499,5.482382777,175.4893329,65.84884585,60.30621054,
76,0,1,0,1,1,9,2743.034449,4670.450585,2.438846143,0,10.68658397,0,2,0,8.535961499,5.482382
43,0,10.68658397,0,2,0,8.535961499,5.482382777,175.4893329,65.84884585,60.30621054,17.0719
6143,0,10.68658397,0,2,0,8.535961499,5.482382777,175.4893329,65.84884585,60.30621054,17.07
3.034449,4670.450585,2.438846143,0,10.68658397,0,2,0,8.535961499,5.482382777,175.4893329,6
.034449,4670.450585,2.438846143,0,10.68658397,0,2,0,8.535961499,5.482382777,175.4893329,65
846143,0,10.68658397,0,2,0,8.535961499,5.482382777,175.4893329,65.84884585,60.30621054,17
3.034449,4670.450585,2.438846143,0,10.68658397,0,2,0,8.535961499,5.482382777,175.4893329,6
6,0,1,0,1,1,9,2743.034449,4670.450585,2.438846143,0,10.68658397,0,2,0,8.535961499,5.4823827
.034449,4670.450585,2.438846143,0,10.68658397,0,2,0,8.535961499,5.482382777,175.4893329,65
.034449,4670.450585,2.438846143,0,10.68658397,0,2,0,8.535961499,5.482382777,175.4893329,65
143,0,10.68658397,0,2,0,8.535961499,5.482382777,175.4893329,65.84884585,60.30621054,17.071
3.034449,4670.450585,2.438846143,0,10.68658397,0,2,0,8.535961499,5.482382777,175.4893329,6
143,0,10.68658397,0,2,0,8.535961499,5.482382777,175.4893329,65.84884585,60.30621054,17.071
743.034449,4670.450585,2.438846143,0,10.68658397,0,2,0,8.535961499,5.482382777,175.489332
3.034449,4670.450585,2.438846143,0,10.68658397,0,2,0,8.535961499,5.482382777,175.4893329,6
3.034449,4670.450585,2.438846143,0,10.68658397,0,2,0,8.535961499,5.482382777,175.4893329,6
143,0,10.68658397,0,2,0,8.535961499,5.482382777,175.4893329,65.84884585,60.30621054,17.071
,1,1,0,1,1,12,2743.034449,4670.450585,2.438846143,0,10.68658397,0,2,0,8.535961499,5.4823827

8,1,1,0,1,1,12,2743.034449,4670.450585,2.438846143,0,10.68658397,0,2,0,8.535961499,5.482382
143,0,10.68658397,0,2,0,8.535961499,5.482382777,175.4893329,65.84884585,60.30621054,17.071
43.034449,4670.450585,2.438846143,0,10.68658397,0,2,0,8.535961499,5.482382777,175.4893329
743.034449,4670.450585,2.438846143,0,10.68658397,0,2,0,8.535961499,5.482382777,175.489332
3.034449,4670.450585,2.438846143,0,10.68658397,0,2,0,8.535961499,5.482382777,175.4893329,
8,1,1,0,1,1,12,2743.034449,4670.450585,2.438846143,0,10.68658397,0,2,0,8.535961499,5.482382
3.034449,4670.450585,2.438846143,0,10.68658397,0,2,0,8.535961499,5.482382777,175.4893329,
743.034449,4670.450585,2.438846143,0,10.68658397,0,2,0,8.535961499,5.482382777,175.489332
154419,3,2,210,68,1,1,0,1,1,12,2743.034449,4670.450585,2.438846143,0,10.68658397,0,2,0,8.5359
8,1,1,0,1,1,12,2743.034449,4670.450585,2.438846143,0,10.68658397,0,2,0,8.535961499,5.482382
143,0,10.68658397,0,2,0,8.535961499,5.482382777,175.4893329,65.84884585,60.30621054,17.071
743.034449,4670.450585,2.438846143,0,10.68658397,0,2,0,8.535961499,5.482382777,175.489332
3.034449,4670.450585,2.438846143,0,10.68658397,0,2,0,8.535961499,5.482382777,175.4893329,
,1,1,0,1,1,12,2743.034449,4670.450585,2.438846143,0,10.68658397,0,2,0,8.535961499,5.4823827
143,0,10.68658397,0,2,0,8.535961499,5.482382777,175.4893329,65.84884585,60.30621054,17.071
143,0,10.68658397,0,2,0,8.535961499,5.482382777,175.4893329,65.84884585,60.30621054,17.071
43.034449,4670.450585,2.438846143,0,10.68658397,0,2,0,8.535961499,5.482382777,175.4893329
3.034449,4670.450585,2.438846143,0,10.68658397,0,2,0,8.535961499,5.482382777,175.4893329,
8,1,1,0,1,1,12,2743.034449,4670.450585,2.438846143,0,10.68658397,0,2,0,8.535961499,5.482382
3.034449,4670.450585,2.438846143,0,10.68658397,0,2,0,8.535961499,5.482382777,175.4893329,
43.034449,4670.450585,2.438846143,0,10.68658397,0,2,0,8.535961499,5.482382777,175.4893329
43.034449,4670.450585,2.438846143,0,10.68658397,0,2,0,8.535961499,5.482382777,175.4893329
3.034449,4670.450585,2.438846143,0,10.68658397,0,2,0,8.535961499,5.482382777,175.4893329,
3.034449,4670.450585,2.438846143,0,10.68658397,0,2,0,8.535961499,5.482382777,175.4893329,
3.034449,4670.450585,2.438846143,0,10.68658397,0,2,0,8.535961499,5.482382777,175.4893329,
43.034449,4670.450585,2.438846143,0,10.68658397,0,2,0,8.535961499,5.482382777,175.4893329
43.034449,4670.450585,2.438846143,0,10.68658397,0,2,0,8.535961499,5.482382777,175.4893329
8,1,1,0,1,1,12,2743.034449,4670.450585,2.438846143,0,10.68658397,0,2,0,8.535961499,5.482382
73,0,1,0,1,1,12,2660.644625,4420.10785,2.357478342,4.947354918,10.68658397,0.144156668,2,1,
660.644625,4420.10785,2.357478342,4.947354918,10.68658397,0.144156668,2,1,8.251174197,4.9
78342,4.947354918,10.68658397,0.144156668,2,1,8.251174197,4.947354918,118.8534403,64.8306
0.644625,4420.10785,2.357478342,4.947354918,10.68658397,0.144156668,2,1,8.251174197,4.947
0.644625,4420.10785,2.357478342,4.947354918,10.68658397,0.144156668,2,1,8.251174197,4.947
2,4.947354918,10.68658397,0.144156668,2,1,8.251174197,4.947354918,118.8534403,64.83065441
60.644625,4420.10785,2.357478342,4.947354918,10.68658397,0.144156668,2,1,8.251174197,4.94
0.644625,4420.10785,2.357478342,4.947354918,10.68658397,0.144156668,2,1,8.251174197,4.947
73,0,1,0,1,1,12,2660.644625,4420.10785,2.357478342,4.947354918,10.68658397,0.144156668,2,1,
60.644625,4420.10785,2.357478342,4.947354918,10.68658397,0.144156668,2,1,8.251174197,4.94
42,4.947354918,10.68658397,0.144156668,2,1,8.251174197,4.947354918,118.8534403,64.8306544
42,4.947354918,10.68658397,0.144156668,2,1,8.251174197,4.947354918,118.8534403,64.8306544
3,0,1,0,1,1,12,2660.644625,4420.10785,2.357478342,4.947354918,10.68658397,0.144156668,2,1,8
478342,4.947354918,10.68658397,0.144156668,2,1,8.251174197,4.947354918,118.8534403,64.830
660.644625,4420.10785,2.357478342,4.947354918,10.68658397,0.144156668,2,1,8.251174197,4.9
.357478342,4.947354918,10.68658397,0.144156668,2,1,8.251174197,4.947354918,118.8534403,6
78342,4.947354918,10.68658397,0.144156668,2,1,8.251174197,4.947354918,118.8534403,64.8306
660.644625,4420.10785,2.357478342,4.947354918,10.68658397,0.144156668,2,1,8.251174197,4.9
660.644625,4420.10785,2.357478342,4.947354918,10.68658397,0.144156668,2,1,8.251174197,4.9
0.644625,4420.10785,2.357478342,4.947354918,10.68658397,0.144156668,2,1,8.251174197,4.947
42,4.947354918,10.68658397,0.144156668,2,1,8.251174197,4.947354918,118.8534403,64.8306544
42,4.947354918,10.68658397,0.144156668,2,1,8.251174197,4.947354918,118.8534403,64.8306544

3,0,1,0,1,1,12,2660.644625,4420.10785,2.357478342,4.947354918,10.68658397,0.144156668,2,1,8
0.644625,4420.10785,2.357478342,4.947354918,10.68658397,0.144156668,2,1,8.251174197,4.947
660.644625,4420.10785,2.357478342,4.947354918,10.68658397,0.144156668,2,1,8.251174197,4.9
3,0,1,0,1,1,12,2660.644625,4420.10785,2.357478342,4.947354918,10.68658397,0.144156668,2,1,8
3,0,1,0,1,1,12,2660.644625,4420.10785,2.357478342,4.947354918,10.68658397,0.144156668,2,1,8
0.644625,4420.10785,2.357478342,4.947354918,10.68658397,0.144156668,2,1,8.251174197,4.947
478342,4.947354918,10.68658397,0.144156668,2,1,8.251174197,4.947354918,118.8534403,64.83
0.644625,4420.10785,2.357478342,4.947354918,10.68658397,0.144156668,2,1,8.251174197,4.947
78342,4.947354918,10.68658397,0.144156668,2,1,8.251174197,4.947354918,118.8534403,64.8306
3,0,1,0,1,1,12,2660.644625,4420.10785,2.357478342,4.947354918,10.68658397,0.144156668,2,1,8
3,0,1,0,1,1,12,2660.644625,4420.10785,2.357478342,4.947354918,10.68658397,0.144156668,2,1,8
357478342,4.947354918,10.68658397,0.144156668,2,1,8.251174197,4.947354918,118.8534403,64.
60.644625,4420.10785,2.357478342,4.947354918,10.68658397,0.144156668,2,1,8.251174197,4.9
,4.947354918,10.68658397,0.144156668,2,1,8.251174197,4.947354918,118.8534403,64.83065441
0.644625,4420.10785,2.357478342,4.947354918,10.68658397,0.144156668,2,1,8.251174197,4.947
42,4.947354918,10.68658397,0.144156668,2,1,8.251174197,4.947354918,118.8534403,64.8306544
42,4.947354918,10.68658397,0.144156668,2,1,8.251174197,4.947354918,118.8534403,64.8306544
0.644625,4420.10785,2.357478342,4.947354918,10.68658397,0.144156668,2,1,8.251174197,4.947
0.644625,4420.10785,2.357478342,4.947354918,10.68658397,0.144156668,2,1,8.251174197,4.947
42,4.947354918,10.68658397,0.144156668,2,1,8.251174197,4.947354918,118.8534403,64.8306544
,4.947354918,10.68658397,0.144156668,2,1,8.251174197,4.947354918,118.8534403,64.83065441
7,0,1,0,1,1,11,2660.644625,4420.10785,2.357478342,4.947354918,10.68658397,0.144156668,2,1,8
2660.644625,4420.10785,2.357478342,4.947354918,10.68658397,0.144156668,2,1,8.251174197,4.
60.644625,4420.10785,2.357478342,4.947354918,10.68658397,0.144156668,2,1,8.251174197,4.94
42,4.947354918,10.68658397,0.144156668,2,1,8.251174197,4.947354918,118.8534403,64.8306544
342,4.947354918,10.68658397,0.144156668,2,1,8.251174197,4.947354918,118.8534403,64.83065
2660.644625,4420.10785,2.357478342,4.947354918,10.68658397,0.144156668,2,1,8.251174197,4.
60.644625,4420.10785,2.357478342,4.947354918,10.68658397,0.144156668,2,1,8.251174197,4.94
42,4.947354918,10.68658397,0.144156668,2,1,8.251174197,4.947354918,118.8534403,64.8306544
60.644625,4420.10785,2.357478342,4.947354918,10.68658397,0.144156668,2,1,8.251174197,4.94
42,4.947354918,10.68658397,0.144156668,2,1,8.251174197,4.947354918,118.8534403,64.8306544
2660.644625,4420.10785,2.357478342,4.947354918,10.68658397,0.144156668,2,1,8.251174197,4.
57478342,4.947354918,10.68658397,0.144156668,2,1,8.251174197,4.947354918,118.8534403,64.
0.644625,4420.10785,2.357478342,4.947354918,10.68658397,0.144156668,2,1,8.251174197,4.947
.357478342,4.947354918,10.68658397,0.144156668,2,1,8.251174197,4.947354918,118.8534403,6
43,0,10.68658397,0,2,0,8.535961499,5.482382777,175.4893329,65.84884585,60.30621054,17.0719
3.034449,4670.450585,2.438846143,0,10.68658397,0,2,0,8.535961499,5.482382777,175.4893329,
3.034449,4670.450585,2.438846143,0,10.68658397,0,2,0,8.535961499,5.482382777,175.4893329,
46143,0,10.68658397,0,2,0,8.535961499,5.482382777,175.4893329,65.84884585,60.30621054,17.0
46143,0,10.68658397,0,2,0,8.535961499,5.482382777,175.4893329,65.84884585,60.30621054,17.0
38846143,0,10.68658397,0,2,0,8.535961499,5.482382777,175.4893329,65.84884585,60.30621054,
.034449,4670.450585,2.438846143,0,10.68658397,0,2,0,8.535961499,5.482382777,175.4893329,6
846143,0,10.68658397,0,2,0,8.535961499,5.482382777,175.4893329,65.84884585,60.30621054,1
77,1,1,1,1,1,8,2743.034449,4670.450585,2.438846143,0,10.68658397,0,2,0,8.535961499,5.48238
,0,10.68658397,0,2,0,8.535961499,5.482382777,175.4893329,65.84884585,60.30621054,17.07192
7,1,1,1,1,1,8,2743.034449,4670.450585,2.438846143,0,10.68658397,0,2,0,8.535961499,5.482382
743.034449,4670.450585,2.438846143,0,10.68658397,0,2,0,8.535961499,5.482382777,175.489332
3.034449,4670.450585,2.438846143,0,10.68658397,0,2,0,8.535961499,5.482382777,175.4893329,
43,0,10.68658397,0,2,0,8.535961499,5.482382777,175.4893329,65.84884585,60.30621054,17.0719
,0,10.68658397,0,2,0,8.535961499,5.482382777,175.4893329,65.84884585,60.30621054,17.07192

3.034449,4670.450585,2.438846143,0,10.68658397,0,2,0,8.535961499,5.482382777,175.4893329,6
77,1,1,1,1,1,8,2743.034449,4670.450585,2.438846143,0,10.68658397,0,2,0,8.535961499,5.482382
3846143,0,10.68658397,0,2,0,8.535961499,5.482382777,175.4893329,65.84884585,60.30621054,17
.034449,4670.450585,2.438846143,0,10.68658397,0,2,0,8.535961499,5.482382777,175.4893329,65
3,0,10.68658397,0,2,0,8.535961499,5.482382777,175.4893329,65.84884585,60.30621054,17.07192
77,1,1,1,1,1,8,2743.034449,4670.450585,2.438846143,0,10.68658397,0,2,0,8.535961499,5.482382
43,0,10.68658397,0,2,0,8.535961499,5.482382777,175.4893329,65.84884585,60.30621054,17.0719
43,0,10.68658397,0,2,0,8.535961499,5.482382777,175.4893329,65.84884585,60.30621054,17.0719
7,1,1,1,1,1,8,2743.034449,4670.450585,2.438846143,0,10.68658397,0,2,0,8.535961499,5.4823827
43,0,10.68658397,0,2,0,8.535961499,5.482382777,175.4893329,65.84884585,60.30621054,17.0719
3,0,10.68658397,0,2,0,8.535961499,5.482382777,175.4893329,65.84884585,60.30621054,17.07192
2,0,10.68658397,0,2,0,8.483216776,5.257339373,156.8475415,66.65384609,57.8307331,16.966433
.68103,4687.400677,2.423776222,0,10.68658397,0,2,0,8.483216776,5.257339373,156.8475415,66.
4.68103,4687.400677,2.423776222,0,10.68658397,0,2,0,8.483216776,5.257339373,156.8475415,66
7,0,1,0,1,1,8,2694.68103,4687.400677,2.423776222,0,10.68658397,0,2,0,8.483216776,5.25733937
7,0,1,0,1,1,8,2694.68103,4687.400677,2.423776222,0,10.68658397,0,2,0,8.483216776,5.25733937
4.68103,4687.400677,2.423776222,0,10.68658397,0,2,0,8.483216776,5.257339373,156.8475415,66
.68103,4687.400677,2.423776222,0,10.68658397,0,2,0,8.483216776,5.257339373,156.8475415,66.
.68103,4687.400677,2.423776222,0,10.68658397,0,2,0,8.483216776,5.257339373,156.8475415,66.
94.68103,4687.400677,2.423776222,0,10.68658397,0,2,0,8.483216776,5.257339373,156.8475415,6
7,0,1,0,1,1,8,2694.68103,4687.400677,2.423776222,0,10.68658397,0,2,0,8.483216776,5.25733937
9691162,3,2,210,77,0,1,0,1,1,8,2694.68103,4687.400677,2.423776222,0,10.68658397,0,2,0,8.48321
94.68103,4687.400677,2.423776222,0,10.68658397,0,2,0,8.483216776,5.257339373,156.8475415,6
.68103,4687.400677,2.423776222,0,10.68658397,0,2,0,8.483216776,5.257339373,156.8475415,66.
4.68103,4687.400677,2.423776222,0,10.68658397,0,2,0,8.483216776,5.257339373,156.8475415,66
382324,3,2,210,77,0,1,0,1,1,8,2694.68103,4687.400677,2.423776222,0,10.68658397,0,2,0,8.483216
.68103,4687.400677,2.423776222,0,10.68658397,0,2,0,8.483216776,5.257339373,156.8475415,66.
.68103,4687.400677,2.423776222,0,10.68658397,0,2,0,8.483216776,5.257339373,156.8475415,66.
382324,3,2,210,77,0,1,0,1,1,8,2694.68103,4687.400677,2.423776222,0,10.68658397,0,2,0,8.483216
4.68103,4687.400677,2.423776222,0,10.68658397,0,2,0,8.483216776,5.257339373,156.8475415,66
7,0,1,0,1,1,8,2694.68103,4687.400677,2.423776222,0,10.68658397,0,2,0,8.483216776,5.25733937
4.68103,4687.400677,2.423776222,0,10.68658397,0,2,0,8.483216776,5.257339373,156.8475415,66
7,0,1,0,1,1,8,2694.68103,4687.400677,2.423776222,0,10.68658397,0,2,0,8.483216776,5.25733937
77,0,1,0,1,1,8,2694.68103,4687.400677,2.423776222,0,10.68658397,0,2,0,8.483216776,5.2573393
.68103,4687.400677,2.423776222,0,10.68658397,0,2,0,8.483216776,5.257339373,156.8475415,66.
94.68103,4687.400677,2.423776222,0,10.68658397,0,2,0,8.483216776,5.257339373,156.8475415,6
94.68103,4687.400677,2.423776222,0,10.68658397,0,2,0,8.483216776,5.257339373,156.8475415,6
9691162,3,2,210,77,0,1,0,1,1,8,2694.68103,4687.400677,2.423776222,0,10.68658397,0,2,0,8.48321
7,0,1,0,1,1,8,2694.68103,4687.400677,2.423776222,0,10.68658397,0,2,0,8.483216776,5.25733937
94.68103,4687.400677,2.423776222,0,10.68658397,0,2,0,8.483216776,5.257339373,156.8475415,6
4.68103,4687.400677,2.423776222,0,10.68658397,0,2,0,8.483216776,5.257339373,156.8475415,66
0617676,3,2,210,77,0,1,0,1,1,8,2694.68103,4687.400677,2.423776222,0,10.68658397,0,2,0,8.48321
.68103,4687.400677,2.423776222,0,10.68658397,0,2,0,8.483216776,5.257339373,156.8475415,66.
2,0,10.68658397,0,2,0,8.483216776,5.257339373,156.8475415,66.65384609,57.8307331,16.966433
2,0,10.68658397,0,2,0,8.483216776,5.257339373,156.8475415,66.65384609,57.8307331,16.966433
94.68103,4687.400677,2.423776222,0,10.68658397,0,2,0,8.483216776,5.257339373,156.8475415,6
94.68103,4687.400677,2.423776222,0,10.68658397,0,2,0,8.483216776,5.257339373,156.8475415,6
4.68103,4687.400677,2.423776222,0,10.68658397,0,2,0,8.483216776,5.257339373,156.8475415,66
4.68103,4687.400677,2.423776222,0,10.68658397,0,2,0,8.483216776,5.257339373,156.8475415,66
.68103,4687.400677,2.423776222,0,10.68658397,0,2,0,8.483216776,5.257339373,156.8475415,66.

464,0,10.39093842,0,4,0,0,0,1638.578802,27.03959719,45.29254342,2.575199732,0,0,0,3.8627995
0,1,1,0,1,1,8,936.7231122,992.443142,5.150399464,0,10.39093842,0,4,0,0,0,1638.578802,27.0395
.7231122,992.443142,5.150399464,0,10.39093842,0,4,0,0,0,1638.578802,27.03959719,45.2925434
.7231122,992.443142,5.150399464,0,10.39093842,0,4,0,0,0,1638.578802,27.03959719,45.2925434
0399464,0,10.39093842,0,4,0,0,0,1638.578802,27.03959719,45.29254342,2.575199732,0,0,0,3.862
399464,0,10.39093842,0,4,0,0,0,1638.578802,27.03959719,45.29254342,2.575199732,0,0,0,3.8627
0,1,1,0,1,1,8,936.7231122,992.443142,5.150399464,0,10.39093842,0,4,0,0,0,1638.578802,27.0395
0,1,1,0,1,1,8,936.7231122,992.443142,5.150399464,0,10.39093842,0,4,0,0,0,1638.578802,27.0395
.7231122,992.443142,5.150399464,0,10.39093842,0,4,0,0,0,1638.578802,27.03959719,45.2925434
382324,3,2,210,70,1,1,0,1,1,8,936.7231122,992.443142,5.150399464,0,10.39093842,0,4,0,0,0,1638.
4,0,10.39093842,0,4,0,0,0,1638.578802,27.03959719,45.29254342,2.575199732,0,0,0,3.862799598,
0,1,1,0,1,1,8,936.7231122,992.443142,5.150399464,0,10.39093842,0,4,0,0,0,1638.578802,27.0395
.7231122,992.443142,5.150399464,0,10.39093842,0,4,0,0,0,1638.578802,27.03959719,45.2925434
7231122,992.443142,5.150399464,0,10.39093842,0,4,0,0,0,1638.578802,27.03959719,45.29254342
9464,0,10.39093842,0,4,0,0,0,1638.578802,27.03959719,45.29254342,2.575199732,0,0,0,3.862799
0,10.39093842,0,4,0,0,0,1638.578802,27.03959719,45.29254342,2.575199732,0,0,0,3.862799598,9
70,1,1,0,1,1,8,936.7231122,992.443142,5.150399464,0,10.39093842,0,4,0,0,0,1638.578802,27.039
.7231122,992.443142,5.150399464,0,10.39093842,0,4,0,0,0,1638.578802,27.03959719,45.2925434
.7231122,992.443142,5.150399464,0,10.39093842,0,4,0,0,0,1638.578802,27.03959719,45.2925434
9464,0,10.39093842,0,4,0,0,0,1638.578802,27.03959719,45.29254342,2.575199732,0,0,0,3.862799
4,0,10.39093842,0,4,0,0,0,1638.578802,27.03959719,45.29254342,2.575199732,0,0,0,3.862799598,
7231122,992.443142,5.150399464,0,10.39093842,0,4,0,0,0,1638.578802,27.03959719,45.29254342
4,0,10.39093842,0,4,0,0,0,1638.578802,27.03959719,45.29254342,2.575199732,0,0,0,3.862799598,
.7231122,992.443142,5.150399464,0,10.39093842,0,4,0,0,0,1638.578802,27.03959719,45.2925434
4,0,10.39093842,0,4,0,0,0,1638.578802,27.03959719,45.29254342,2.575199732,0,0,0,3.862799598,
0,10.39093842,0,4,0,0,0,1638.578802,27.03959719,45.29254342,2.575199732,0,0,0,3.862799598,9
7231122,992.443142,5.150399464,0,10.39093842,0,4,0,0,0,1638.578802,27.03959719,45.29254342
70,1,1,0,1,1,8,936.7231122,992.443142,5.150399464,0,10.39093842,0,4,0,0,0,1638.578802,27.039
7231122,992.443142,5.150399464,0,10.39093842,0,4,0,0,0,1638.578802,27.03959719,45.29254342
7231122,992.443142,5.150399464,0,10.39093842,0,4,0,0,0,1638.578802,27.03959719,45.29254342
4,0,10.39093842,0,4,0,0,0,1638.578802,27.03959719,45.29254342,2.575199732,0,0,0,3.862799598,
.7231122,992.443142,5.150399464,0,10.39093842,0,4,0,0,0,1638.578802,27.03959719,45.2925434
.7231122,992.443142,5.150399464,0,10.39093842,0,4,0,0,0,1638.578802,27.03959719,45.2925434
7231122,992.443142,5.150399464,0,10.39093842,0,4,0,0,0,1638.578802,27.03959719,45.29254342
0399464,0,10.39093842,0,4,0,0,0,1638.578802,27.03959719,45.29254342,2.575199732,0,0,0,3.862
3605,0,10.39093842,0,4,0,0,0,1611.595968,24.92478233,49.79797938,3.398833954,0,0,0,3.398833
5,1,1,1,1,1,9,978.1989598,1005.05473,4.531778605,0,10.39093842,0,4,0,0,0,1611.595968,24.9247
75,1,1,1,1,1,9,978.1989598,1005.05473,4.531778605,0,10.39093842,0,4,0,0,0,1611.595968,24.924
8.1989598,1005.05473,4.531778605,0,10.39093842,0,4,0,0,0,1611.595968,24.92478233,49.797979
1989598,1005.05473,4.531778605,0,10.39093842,0,4,0,0,0,1611.595968,24.92478233,49.79797938
75,1,1,1,1,1,9,978.1989598,1005.05473,4.531778605,0,10.39093842,0,4,0,0,0,1611.595968,24.924
778605,0,10.39093842,0,4,0,0,0,1611.595968,24.92478233,49.79797938,3.398833954,0,0,0,3.3988
.1989598,1005.05473,4.531778605,0,10.39093842,0,4,0,0,0,1611.595968,24.92478233,49.7979793
.1989598,1005.05473,4.531778605,0,10.39093842,0,4,0,0,0,1611.595968,24.92478233,49.7979793
5,0,10.39093842,0,4,0,0,0,1611.595968,24.92478233,49.79797938,3.398833954,0,0,0,3.398833954,
5,1,1,1,1,1,9,978.1989598,1005.05473,4.531778605,0,10.39093842,0,4,0,0,0,1611.595968,24.9247
5,1,1,1,1,1,9,978.1989598,1005.05473,4.531778605,0,10.39093842,0,4,0,0,0,1611.595968,24.9247
3605,0,10.39093842,0,4,0,0,0,1611.595968,24.92478233,49.79797938,3.398833954,0,0,0,3.398833
8.1989598,1005.05473,4.531778605,0,10.39093842,0,4,0,0,0,1611.595968,24.92478233,49.797979
75,1,1,1,1,1,9,978.1989598,1005.05473,4.531778605,0,10.39093842,0,4,0,0,0,1611.595968,24.924

3605,0,10.39093842,0,4,0,0,0,1611.595968,24.92478233,49.79797938,3.398833954,0,0,0,3.3988339
1989598,1005.05473,4.531778605,0,10.39093842,0,4,0,0,0,1611.595968,24.92478233,49.79797938
.1989598,1005.05473,4.531778605,0,10.39093842,0,4,0,0,0,1611.595968,24.92478233,49.7979793
5,1,1,1,1,1,9,978.1989598,1005.05473,4.531778605,0,10.39093842,0,4,0,0,0,1611.595968,24.9247
5,0,10.39093842,0,4,0,0,0,1611.595968,24.92478233,49.79797938,3.398833954,0,0,0,3.398833954,
8.1989598,1005.05473,4.531778605,0,10.39093842,0,4,0,0,0,1611.595968,24.92478233,49.797979
5,1,1,1,1,1,9,978.1989598,1005.05473,4.531778605,0,10.39093842,0,4,0,0,0,1611.595968,24.9247
3.1989598,1005.05473,4.531778605,0,10.39093842,0,4,0,0,0,1611.595968,24.92478233,49.797979
3.1989598,1005.05473,4.531778605,0,10.39093842,0,4,0,0,0,1611.595968,24.92478233,49.797979
78.1989598,1005.05473,4.531778605,0,10.39093842,0,4,0,0,0,1611.595968,24.92478233,49.797979
67,0,1,0,1,1,11,957.6965115,900.2575528,2.781128148,4.65863564,3.439906506,1.561264204,3,1,
7.6965115,900.2575528,2.781128148,4.65863564,3.439906506,1.561264204,3,1,0,0,1444.168296,
7.6965115,900.2575528,2.781128148,4.65863564,3.439906506,1.561264204,3,1,0,0,1444.168296,
57.6965115,900.2575528,2.781128148,4.65863564,3.439906506,1.561264204,3,1,0,0,1444.168296
.6965115,900.2575528,2.781128148,4.65863564,3.439906506,1.561264204,3,1,0,0,1444.168296,14
.6965115,900.2575528,2.781128148,4.65863564,3.439906506,1.561264204,3,1,0,0,1444.168296,14
.6965115,900.2575528,2.781128148,4.65863564,3.439906506,1.561264204,3,1,0,0,1444.168296,14
57.6965115,900.2575528,2.781128148,4.65863564,3.439906506,1.561264204,3,1,0,0,1444.168296
7.6965115,900.2575528,2.781128148,4.65863564,3.439906506,1.561264204,3,1,0,0,1444.168296,
.6965115,900.2575528,2.781128148,4.65863564,3.439906506,1.561264204,3,1,0,0,1444.168296,14
67,0,1,0,1,1,11,957.6965115,900.2575528,2.781128148,4.65863564,3.439906506,1.561264204,3,1,
.6965115,900.2575528,2.781128148,4.65863564,3.439906506,1.561264204,3,1,0,0,1444.168296,14
.6965115,900.2575528,2.781128148,4.65863564,3.439906506,1.561264204,3,1,0,0,1444.168296,14
7.6965115,900.2575528,2.781128148,4.65863564,3.439906506,1.561264204,3,1,0,0,1444.168296,
.6965115,900.2575528,2.781128148,4.65863564,3.439906506,1.561264204,3,1,0,0,1444.168296,14
57.6965115,900.2575528,2.781128148,4.65863564,3.439906506,1.561264204,3,1,0,0,1444.168296
7.6965115,900.2575528,2.781128148,4.65863564,3.439906506,1.561264204,3,1,0,0,1444.168296,
57.6965115,900.2575528,2.781128148,4.65863564,3.439906506,1.561264204,3,1,0,0,1444.168296
57.6965115,900.2575528,2.781128148,4.65863564,3.439906506,1.561264204,3,1,0,0,1444.168296
0308838,3,2,210,67,0,1,0,1,1,11,957.6965115,900.2575528,2.781128148,4.65863564,3.439906506,
7.6965115,900.2575528,2.781128148,4.65863564,3.439906506,1.561264204,3,1,0,0,1444.168296,
.6965115,900.2575528,2.781128148,4.65863564,3.439906506,1.561264204,3,1,0,0,1444.168296,14
.6965115,900.2575528,2.781128148,4.65863564,3.439906506,1.561264204,3,1,0,0,1444.168296,14
5965115,900.2575528,2.781128148,4.65863564,3.439906506,1.561264204,3,1,0,0,1444.168296,14.
.6965115,900.2575528,2.781128148,4.65863564,3.439906506,1.561264204,3,1,0,0,1444.168296,14
7.6965115,900.2575528,2.781128148,4.65863564,3.439906506,1.561264204,3,1,0,0,1444.168296,
7.6965115,900.2575528,2.781128148,4.65863564,3.439906506,1.561264204,3,1,0,0,1444.168296,
1222,4.240622436,3.439906506,1.561264204,3,1,0,0,1505.189736,16.31557985,21.20311218,2.039
.8623022,826.5964981,3.059171222,4.240622436,3.439906506,1.561264204,3,1,0,0,1505.189736,
8,1,1,0,1,1,9,982.8623022,826.5964981,3.059171222,4.240622436,3.439906506,1.561264204,3,1,0
.8623022,826.5964981,3.059171222,4.240622436,3.439906506,1.561264204,3,1,0,0,1505.189736,
8,1,1,0,1,1,9,982.8623022,826.5964981,3.059171222,4.240622436,3.439906506,1.561264204,3,1,0
.8623022,826.5964981,3.059171222,4.240622436,3.439906506,1.561264204,3,1,0,0,1505.189736,
59171222,4.240622436,3.439906506,1.561264204,3,1,0,0,1505.189736,16.31557985,21.20311218,
32.8623022,826.5964981,3.059171222,4.240622436,3.439906506,1.561264204,3,1,0,0,1505.18973
1222,4.240622436,3.439906506,1.561264204,3,1,0,0,1505.189736,16.31557985,21.20311218,2.039
71222,4.240622436,3.439906506,1.561264204,3,1,0,0,1505.189736,16.31557985,21.20311218,2.03
32.8623022,826.5964981,3.059171222,4.240622436,3.439906506,1.561264204,3,1,0,0,1505.18973
22,4.240622436,3.439906506,1.561264204,3,1,0,0,1505.189736,16.31557985,21.20311218,2.03944
1222,4.240622436,3.439906506,1.561264204,3,1,0,0,1505.189736,16.31557985,21.20311218,2.039

8,1,1,0,1,1,9,982.8623022,826.5964981,3.059171222,4.240622436,3.439906506,1.561264204,3,1,0
.8623022,826.5964981,3.059171222,4.240622436,3.439906506,1.561264204,3,1,0,0,1505.189736,
3623022,826.5964981,3.059171222,4.240622436,3.439906506,1.561264204,3,1,0,0,1505.189736,1
3623022,826.5964981,3.059171222,4.240622436,3.439906506,1.561264204,3,1,0,0,1505.189736,1
2,4.240622436,3.439906506,1.561264204,3,1,0,0,1505.189736,16.31557985,21.20311218,2.039447
.8623022,826.5964981,3.059171222,4.240622436,3.439906506,1.561264204,3,1,0,0,1505.189736,
07,3.68568057,3.439906506,1.561264204,3,1,0,0,1239.9595,12.73001583,33.17112513,1.41444620
.3434571,1028.103305,2.828892407,3.68568057,3.439906506,1.561264204,3,1,0,0,1239.9595,12.7
.3434571,1028.103305,2.828892407,3.68568057,3.439906506,1.561264204,3,1,0,0,1239.9595,12.7
2.3434571,1028.103305,2.828892407,3.68568057,3.439906506,1.561264204,3,1,0,0,1239.9595,12.
28892407,3.68568057,3.439906506,1.561264204,3,1,0,0,1239.9595,12.73001583,33.17112513,1.41
2.3434571,1028.103305,2.828892407,3.68568057,3.439906506,1.561264204,3,1,0,0,1239.9595,12.
828892407,3.68568057,3.439906506,1.561264204,3,1,0,0,1239.9595,12.73001583,33.17112513,1.4
2.3434571,1028.103305,2.828892407,3.68568057,3.439906506,1.561264204,3,1,0,0,1239.9595,12.
2.3434571,1028.103305,2.828892407,3.68568057,3.439906506,1.561264204,3,1,0,0,1239.9595,12.
942.3434571,1028.103305,2.828892407,3.68568057,3.439906506,1.561264204,3,1,0,0,1239.9595,1
3434571,1028.103305,2.828892407,3.68568057,3.439906506,1.561264204,3,1,0,0,1239.9595,12.73
9,1,1,1,1,1,12,942.3434571,1028.103305,2.828892407,3.68568057,3.439906506,1.561264204,3,1,0
407,3.68568057,3.439906506,1.561264204,3,1,0,0,1239.9595,12.73001583,33.17112513,1.4144462
407,3.68568057,3.439906506,1.561264204,3,1,0,0,1239.9595,12.73001583,33.17112513,1.4144462
92407,3.68568057,3.439906506,1.561264204,3,1,0,0,1239.9595,12.73001583,33.17112513,1.41444
9,1,1,1,1,1,12,942.3434571,1028.103305,2.828892407,3.68568057,3.439906506,1.561264204,3,1,0
828892407,3.68568057,3.439906506,1.561264204,3,1,0,0,1239.9595,12.73001583,33.17112513,1.4
9,1,1,1,1,1,12,942.3434571,1028.103305,2.828892407,3.68568057,3.439906506,1.561264204,3,1,0
2.3434571,1028.103305,2.828892407,3.68568057,3.439906506,1.561264204,3,1,0,0,1239.9595,12.
0233,3.420483313,3.439906506,1.561264204,3,1,0,0,1387.136576,13.02047099,23.94338319,1.531
2.8407981,946.0953896,3.063640233,3.420483313,3.439906506,1.561264204,3,1,0,0,1387.136576
.8407981,946.0953896,3.063640233,3.420483313,3.439906506,1.561264204,3,1,0,0,1387.136576,
2.8407981,946.0953896,3.063640233,3.420483313,3.439906506,1.561264204,3,1,0,0,1387.136576
233,3.420483313,3.439906506,1.561264204,3,1,0,0,1387.136576,13.02047099,23.94338319,1.5318
063640233,3.420483313,3.439906506,1.561264204,3,1,0,0,1387.136576,13.02047099,23.9433831
540233,3.420483313,3.439906506,1.561264204,3,1,0,0,1387.136576,13.02047099,23.94338319,1.5
2.8407981,946.0953896,3.063640233,3.420483313,3.439906506,1.561264204,3,1,0,0,1387.136576
2.8407981,946.0953896,3.063640233,3.420483313,3.439906506,1.561264204,3,1,0,0,1387.136576
53640233,3.420483313,3.439906506,1.561264204,3,1,0,0,1387.136576,13.02047099,23.94338319,
33,3.420483313,3.439906506,1.561264204,3,1,0,0,1387.136576,13.02047099,23.94338319,1.53182
2,1,1,0,1,1,12,952.8407981,946.0953896,3.063640233,3.420483313,3.439906506,1.561264204,3,1,
2,1,1,0,1,1,12,952.8407981,946.0953896,3.063640233,3.420483313,3.439906506,1.561264204,3,1,
3640233,3.420483313,3.439906506,1.561264204,3,1,0,0,1387.136576,13.02047099,23.94338319,1.
3.420483313,3.439906506,1.561264204,3,1,0,0,1387.136576,13.02047099,23.94338319,1.5318201
53640233,3.420483313,3.439906506,1.561264204,3,1,0,0,1387.136576,13.02047099,23.94338319,
952.8407981,946.0953896,3.063640233,3.420483313,3.439906506,1.561264204,3,1,0,0,1387.13657
53640233,3.420483313,3.439906506,1.561264204,3,1,0,0,1387.136576,13.02047099,23.94338319,
2.8407981,946.0953896,3.063640233,3.420483313,3.439906506,1.561264204,3,1,0,0,1387.136576
52.8407981,946.0953896,3.063640233,3.420483313,3.439906506,1.561264204,3,1,0,0,1387.136576
.8407981,946.0953896,3.063640233,3.420483313,3.439906506,1.561264204,3,1,0,0,1387.136576,
.8407981,946.0953896,3.063640233,3.420483313,3.439906506,1.561264204,3,1,0,0,1387.136576,
3640233,3.420483313,3.439906506,1.561264204,3,1,0,0,1387.136576,13.02047099,23.94338319,1.
.8407981,946.0953896,3.063640233,3.420483313,3.439906506,1.561264204,3,1,0,0,1387.136576,
.8407981,946.0953896,3.063640233,3.420483313,3.439906506,1.561264204,3,1,0,0,1387.136576,

233,3.420483313,3.439906506,1.561264204,3,1,0,0,1387.136576,13.02047099,23.94338319,1.5318
2.8407981,946.0953896,3.063640233,3.420483313,3.439906506,1.561264204,3,1,0,0,1387.136576
52.8407981,946.0953896,3.063640233,3.420483313,3.439906506,1.561264204,3,1,0,0,1387.13657
63699,0,10.39093842,0,4,0,0,0,1375.352924,17.28568206,59.79422004,1.81954548,0,0,0,2.729318
25.3891482,1300.175058,4.548863699,0,10.39093842,0,4,0,0,0,1375.352924,17.28568206,59.7942
89,0,1,0,1,1,10,925.3891482,1300.175058,4.548863699,0,10.39093842,0,4,0,0,0,1375.352924,17.2
9,0,1,0,1,1,10,925.3891482,1300.175058,4.548863699,0,10.39093842,0,4,0,0,0,1375.352924,17.28
.3891482,1300.175058,4.548863699,0,10.39093842,0,4,0,0,0,1375.352924,17.28568206,59.794220
5.3891482,1300.175058,4.548863699,0,10.39093842,0,4,0,0,0,1375.352924,17.28568206,59.79422
.3891482,1300.175058,4.548863699,0,10.39093842,0,4,0,0,0,1375.352924,17.28568206,59.794220
89,0,1,0,1,1,10,925.3891482,1300.175058,4.548863699,0,10.39093842,0,4,0,0,0,1375.352924,17.2
9,0,1,0,1,1,10,925.3891482,1300.175058,4.548863699,0,10.39093842,0,4,0,0,0,1375.352924,17.28
.3891482,1300.175058,4.548863699,0,10.39093842,0,4,0,0,0,1375.352924,17.28568206,59.794220
89,0,1,0,1,1,10,925.3891482,1300.175058,4.548863699,0,10.39093842,0,4,0,0,0,1375.352924,17.2
.3891482,1300.175058,4.548863699,0,10.39093842,0,4,0,0,0,1375.352924,17.28568206,59.794220
25.3891482,1300.175058,4.548863699,0,10.39093842,0,4,0,0,0,1375.352924,17.28568206,59.7942
.3891482,1300.175058,4.548863699,0,10.39093842,0,4,0,0,0,1375.352924,17.28568206,59.794220
25.3891482,1300.175058,4.548863699,0,10.39093842,0,4,0,0,0,1375.352924,17.28568206,59.7942
63699,0,10.39093842,0,4,0,0,0,1375.352924,17.28568206,59.79422004,1.81954548,0,0,0,2.729318
.3891482,1300.175058,4.548863699,0,10.39093842,0,4,0,0,0,1375.352924,17.28568206,59.794220
48863699,0,10.39093842,0,4,0,0,0,1375.352924,17.28568206,59.79422004,1.81954548,0,0,0,2.729
699,0,10.39093842,0,4,0,0,0,1375.352924,17.28568206,59.79422004,1.81954548,0,0,0,2.72931821
89,0,1,0,1,1,10,925.3891482,1300.175058,4.548863699,0,10.39093842,0,4,0,0,0,1375.352924,17.2
548863699,0,10.39093842,0,4,0,0,0,1375.352924,17.28568206,59.79422004,1.81954548,0,0,0,2.72
25.3891482,1300.175058,4.548863699,0,10.39093842,0,4,0,0,0,1375.352924,17.28568206,59.7942
48863699,0,10.39093842,0,4,0,0,0,1375.352924,17.28568206,59.79422004,1.81954548,0,0,0,2.729
.3891482,1300.175058,4.548863699,0,10.39093842,0,4,0,0,0,1375.352924,17.28568206,59.794220
699,0,10.39093842,0,4,0,0,0,1375.352924,17.28568206,59.79422004,1.81954548,0,0,0,2.72931821
63699,0,10.39093842,0,4,0,0,0,1375.352924,17.28568206,59.79422004,1.81954548,0,0,0,2.729318
89,0,1,0,1,1,10,925.3891482,1300.175058,4.548863699,0,10.39093842,0,4,0,0,0,1375.352924,17.2
548863699,0,10.39093842,0,4,0,0,0,1375.352924,17.28568206,59.79422004,1.81954548,0,0,0,2.72
25.3891482,1300.175058,4.548863699,0,10.39093842,0,4,0,0,0,1375.352924,17.28568206,59.7942
699,0,10.39093842,0,4,0,0,0,1375.352924,17.28568206,59.79422004,1.81954548,0,0,0,2.72931821
699,0,10.39093842,0,4,0,0,0,1375.352924,17.28568206,59.79422004,1.81954548,0,0,0,2.72931821
25.3891482,1300.175058,4.548863699,0,10.39093842,0,4,0,0,0,1375.352924,17.28568206,59.7942
.3891482,1300.175058,4.548863699,0,10.39093842,0,4,0,0,0,1375.352924,17.28568206,59.794220
.3891482,1300.175058,4.548863699,0,10.39093842,0,4,0,0,0,1375.352924,17.28568206,59.794220
89,0,1,0,1,1,10,925.3891482,1300.175058,4.548863699,0,10.39093842,0,4,0,0,0,1375.352924,17.2
699,0,10.39093842,0,4,0,0,0,1375.352924,17.28568206,59.79422004,1.81954548,0,0,0,2.72931821
548863699,0,10.39093842,0,4,0,0,0,1375.352924,17.28568206,59.79422004,1.81954548,0,0,0,2.72
.3891482,1300.175058,4.548863699,0,10.39093842,0,4,0,0,0,1375.352924,17.28568206,59.794220
863699,0,10.39093842,0,4,0,0,0,1375.352924,17.28568206,59.79422004,1.81954548,0,0,0,2.72931
25.3891482,1300.175058,4.548863699,0,10.39093842,0,4,0,0,0,1375.352924,17.28568206,59.7942
25.3891482,1300.175058,4.548863699,0,10.39093842,0,4,0,0,0,1375.352924,17.28568206,59.7942
2370605,3,2,210,89,0,1,0,1,1,10,925.3891482,1300.175058,4.548863699,0,10.39093842,0,4,0,0,0,1
925.3891482,1300.175058,4.548863699,0,10.39093842,0,4,0,0,0,1375.352924,17.28568206,59.794
.3891482,1300.175058,4.548863699,0,10.39093842,0,4,0,0,0,1375.352924,17.28568206,59.794220
9,0,1,0,1,1,10,925.3891482,1300.175058,4.548863699,0,10.39093842,0,4,0,0,0,1375.352924,17.28
699,0,10.39093842,0,4,0,0,0,1375.352924,17.28568206,59.79422004,1.81954548,0,0,0,2.72931821
63699,0,10.39093842,0,4,0,0,0,1375.352924,17.28568206,59.79422004,1.81954548,0,0,0,2.729318

.3891482,1300.175058,4.548863699,0,10.39093842,0,4,0,0,0,1375.352924,17.28568206,59.794220
599,0,10.39093842,0,4,0,0,0,1375.352924,17.28568206,59.79422004,1.81954548,0,0,0,2.72931821
.3891482,1300.175058,4.548863699,0,10.39093842,0,4,0,0,0,1375.352924,17.28568206,59.794220
.3891482,1300.175058,4.548863699,0,10.39093842,0,4,0,0,0,1375.352924,17.28568206,59.794220
863699,0,10.39093842,0,4,0,0,0,1375.352924,17.28568206,59.79422004,1.81954548,0,0,0,2.729318
.3891482,1300.175058,4.548863699,0,10.39093842,0,4,0,0,0,1375.352924,17.28568206,59.794220
.3891482,1300.175058,4.548863699,0,10.39093842,0,4,0,0,0,1375.352924,17.28568206,59.794220
48863699,0,10.39093842,0,4,0,0,0,1375.352924,17.28568206,59.79422004,1.81954548,0,0,0,2.7293
.3891482,1300.175058,4.548863699,0,10.39093842,0,4,0,0,0,1375.352924,17.28568206,59.794220
599,0,10.39093842,0,4,0,0,0,1375.352924,17.28568206,59.79422004,1.81954548,0,0,0,2.72931821
9,0,1,0,1,1,10,925.3891482,1300.175058,4.548863699,0,10.39093842,0,4,0,0,0,1375.352924,17.285
.3891482,1300.175058,4.548863699,0,10.39093842,0,4,0,0,0,1375.352924,17.28568206,59.794220
25.3891482,1300.175058,4.548863699,0,10.39093842,0,4,0,0,0,1375.352924,17.28568206,59.7942
25.3891482,1300.175058,4.548863699,0,10.39093842,0,4,0,0,0,1375.352924,17.28568206,59.7942
863699,0,10.39093842,0,4,0,0,0,1375.352924,17.28568206,59.79422004,1.81954548,0,0,0,2.729318
599,0,10.39093842,0,4,0,0,0,1375.352924,17.28568206,59.79422004,1.81954548,0,0,0,2.72931821
599,0,10.39093842,0,4,0,0,0,1375.352924,17.28568206,59.79422004,1.81954548,0,0,0,2.72931821
599,0,10.39093842,0,4,0,0,0,1375.352924,17.28568206,59.79422004,1.81954548,0,0,0,2.72931821
.3891482,1300.175058,4.548863699,0,10.39093842,0,4,0,0,0,1375.352924,17.28568206,59.794220
863699,0,10.39093842,0,4,0,0,0,1375.352924,17.28568206,59.79422004,1.81954548,0,0,0,2.729318
.3891482,1300.175058,4.548863699,0,10.39093842,0,4,0,0,0,1375.352924,17.28568206,59.794220
599,0,10.39093842,0,4,0,0,0,1375.352924,17.28568206,59.79422004,1.81954548,0,0,0,2.72931821
599,0,10.39093842,0,4,0,0,0,1375.352924,17.28568206,59.79422004,1.81954548,0,0,0,2.72931821
.3891482,1300.175058,4.548863699,0,10.39093842,0,4,0,0,0,1375.352924,17.28568206,59.794220
5,6.609550438,1.690198102,1.690198102,1,1,10.26743712,19.82865132,233.9039137,51.33718562
07.130336,1787.467877,4.10697485,6.609550438,1.690198102,1.690198102,1,1,10.26743712,19.8
1607.130336,1787.467877,4.10697485,6.609550438,1.690198102,1.690198102,1,1,10.26743712,19.
85,6.609550438,1.690198102,1.690198102,1,1,10.26743712,19.82865132,233.9039137,51.3371856
85,6.609550438,1.690198102,1.690198102,1,1,10.26743712,19.82865132,233.9039137,51.3371856
7,1,0,0,1,1,10,1607.130336,1787.467877,4.10697485,6.609550438,1.690198102,1.690198102,1,1,1
07.130336,1787.467877,4.10697485,6.609550438,1.690198102,1.690198102,1,1,10.26743712,19.8
1607.130336,1787.467877,4.10697485,6.609550438,1.690198102,1.690198102,1,1,10.26743712,19.
7.130336,1787.467877,4.10697485,6.609550438,1.690198102,1.690198102,1,1,10.26743712,19.82
07.130336,1787.467877,4.10697485,6.609550438,1.690198102,1.690198102,1,1,10.26743712,19.8
97485,6.609550438,1.690198102,1.690198102,1,1,10.26743712,19.82865132,233.9039137,51.3371
10697485,6.609550438,1.690198102,1.690198102,1,1,10.26743712,19.82865132,233.9039137,51.
1607.130336,1787.467877,4.10697485,6.609550438,1.690198102,1.690198102,1,1,10.26743712,19.
7.130336,1787.467877,4.10697485,6.609550438,1.690198102,1.690198102,1,1,10.26743712,19.82
7.130336,1787.467877,4.10697485,6.609550438,1.690198102,1.690198102,1,1,10.26743712,19.82
97485,6.609550438,1.690198102,1.690198102,1,1,10.26743712,19.82865132,233.9039137,51.3371
0697485,6.609550438,1.690198102,1.690198102,1,1,10.26743712,19.82865132,233.9039137,51.33
07.130336,1787.467877,4.10697485,6.609550438,1.690198102,1.690198102,1,1,10.26743712,19.8
7,1,0,0,1,1,10,1607.130336,1787.467877,4.10697485,6.609550438,1.690198102,1.690198102,1,1,1
07.130336,1787.467877,4.10697485,6.609550438,1.690198102,1.690198102,1,1,10.26743712,19.8
7485,6.609550438,1.690198102,1.690198102,1,1,10.26743712,19.82865132,233.9039137,51.33718
10697485,6.609550438,1.690198102,1.690198102,1,1,10.26743712,19.82865132,233.9039137,51.
10697485,6.609550438,1.690198102,1.690198102,1,1,10.26743712,19.82865132,233.9039137,51.
07.130336,1787.467877,4.10697485,6.609550438,1.690198102,1.690198102,1,1,10.26743712,19.8
1607.130336,1787.467877,4.10697485,6.609550438,1.690198102,1.690198102,1,1,10.26743712,19.
85,6.609550438,1.690198102,1.690198102,1,1,10.26743712,19.82865132,233.9039137,51.3371856

07.130336,1787.467877,4.10697485,6.609550438,1.690198102,1.690198102,1,1,10.26743712,19.8
07.130336,1787.467877,4.10697485,6.609550438,1.690198102,1.690198102,1,1,10.26743712,19.8
10697485,6.609550438,1.690198102,1.690198102,1,1,10.26743712,19.82865132,233.9039137,51.3
07.130336,1787.467877,4.10697485,6.609550438,1.690198102,1.690198102,1,1,10.26743712,19.8
35,6.609550438,1.690198102,1.690198102,1,1,10.26743712,19.82865132,233.9039137,51.3371856
7.130336,1787.467877,4.10697485,6.609550438,1.690198102,1.690198102,1,1,10.26743712,19.82
7.130336,1787.467877,4.10697485,6.609550438,1.690198102,1.690198102,1,1,10.26743712,19.82
7.130336,1787.467877,4.10697485,6.609550438,1.690198102,1.690198102,1,1,10.26743712,19.82
7,4.10697485,6.609550438,1.690198102,1.690198102,1,1,10.26743712,19.82865132,233.9039137,5
97485,6.609550438,1.690198102,1.690198102,1,1,10.26743712,19.82865132,233.9039137,51.3371
009862056,4.17405127,1.690198102,1.690198102,1,1,12.69789651,16.69620508,137.9299365,53.4
9,0,0,1,0,1,12,1625.561504,1874.953605,4.009862056,4.17405127,1.690198102,1.690198102,1,1,1
056,4.17405127,1.690198102,1.690198102,1,1,12.69789651,16.69620508,137.9299365,53.4648274
625.561504,1874.953605,4.009862056,4.17405127,1.690198102,1.690198102,1,1,12.69789651,16.
5.561504,1874.953605,4.009862056,4.17405127,1.690198102,1.690198102,1,1,12.69789651,16.69
79,0,0,1,0,1,12,1625.561504,1874.953605,4.009862056,4.17405127,1.690198102,1.690198102,1,1,
625.561504,1874.953605,4.009862056,4.17405127,1.690198102,1.690198102,1,1,12.69789651,16.
79,0,0,1,0,1,12,1625.561504,1874.953605,4.009862056,4.17405127,1.690198102,1.690198102,1,1,
9,0,0,1,0,1,12,1625.561504,1874.953605,4.009862056,4.17405127,1.690198102,1.690198102,1,1,1
5.561504,1874.953605,4.009862056,4.17405127,1.690198102,1.690198102,1,1,12.69789651,16.69
5.561504,1874.953605,4.009862056,4.17405127,1.690198102,1.690198102,1,1,12.69789651,16.69
625.561504,1874.953605,4.009862056,4.17405127,1.690198102,1.690198102,1,1,12.69789651,16.
25.561504,1874.953605,4.009862056,4.17405127,1.690198102,1.690198102,1,1,12.69789651,16.6
5.561504,1874.953605,4.009862056,4.17405127,1.690198102,1.690198102,1,1,12.69789651,16.69
5.561504,1874.953605,4.009862056,4.17405127,1.690198102,1.690198102,1,1,12.69789651,16.69
862056,4.17405127,1.690198102,1.690198102,1,1,12.69789651,16.69620508,137.9299365,53.4648
79,0,0,1,0,1,12,1625.561504,1874.953605,4.009862056,4.17405127,1.690198102,1.690198102,1,1,
5,4.009862056,4.17405127,1.690198102,1.690198102,1,1,12.69789651,16.69620508,137.9299365,
862056,4.17405127,1.690198102,1.690198102,1,1,12.69789651,16.69620508,137.9299365,53.4648
5.561504,1874.953605,4.009862056,4.17405127,1.690198102,1.690198102,1,1,12.69789651,16.69
79,0,0,1,0,1,12,1625.561504,1874.953605,4.009862056,4.17405127,1.690198102,1.690198102,1,1,
056,4.17405127,1.690198102,1.690198102,1,1,12.69789651,16.69620508,137.9299365,53.4648274
09862056,4.17405127,1.690198102,1.690198102,1,1,12.69789651,16.69620508,137.9299365,53.46
625.561504,1874.953605,4.009862056,4.17405127,1.690198102,1.690198102,1,1,12.69789651,16.
2,79,0,0,1,0,1,12,1625.561504,1874.953605,4.009862056,4.17405127,1.690198102,1.690198102,1,
862056,4.17405127,1.690198102,1.690198102,1,1,12.69789651,16.69620508,137.9299365,53.4648
25.561504,1874.953605,4.009862056,4.17405127,1.690198102,1.690198102,1,1,12.69789651,16.6
5.561504,1874.953605,4.009862056,4.17405127,1.690198102,1.690198102,1,1,12.69789651,16.69
9,0,0,1,0,1,12,1625.561504,1874.953605,4.009862056,4.17405127,1.690198102,1.690198102,1,1,1
09862056,4.17405127,1.690198102,1.690198102,1,1,12.69789651,16.69620508,137.9299365,53.46
5.870272852,1.690198102,1.690198102,1,1,10.31562919,29.35136426,218.06741,47.45189429,99.
7,1,0,0,1,1,9,1548.69824,1731.923397,2.063125839,5.870272852,1.690198102,1.690198102,1,1,10
3125839,5.870272852,1.690198102,1.690198102,1,1,10.31562919,29.35136426,218.06741,47.4518
9,5.870272852,1.690198102,1.690198102,1,1,10.31562919,29.35136426,218.06741,47.45189429,99
8.69824,1731.923397,2.063125839,5.870272852,1.690198102,1.690198102,1,1,10.31562919,29.35
9,5.870272852,1.690198102,1.690198102,1,1,10.31562919,29.35136426,218.06741,47.45189429,99
9,5.870272852,1.690198102,1.690198102,1,1,10.31562919,29.35136426,218.06741,47.45189429,99
67,1,0,0,1,1,9,1548.69824,1731.923397,2.063125839,5.870272852,1.690198102,1.690198102,1,1,1
7,1,0,0,1,1,9,1548.69824,1731.923397,2.063125839,5.870272852,1.690198102,1.690198102,1,1,10
.69824,1731.923397,2.063125839,5.870272852,1.690198102,1.690198102,1,1,10.31562919,29.351

48.69824,1731.923397,2.063125839,5.870272852,1.690198102,1.690198102,1,1,10.31562919,29.3
,5.870272852,1.690198102,1.690198102,1,1,10.31562919,29.35136426,218.06741,47.45189429,9
.69824,1731.923397,2.063125839,5.870272852,1.690198102,1.690198102,1,1,10.31562919,29.351
67,1,0,0,1,1,9,1548.69824,1731.923397,2.063125839,5.870272852,1.690198102,1.690198102,1,1,1
,5.870272852,1.690198102,1.690198102,1,1,10.31562919,29.35136426,218.06741,47.45189429,9
870272852,1.690198102,1.690198102,1,1,10.31562919,29.35136426,218.06741,47.45189429,99.79
.69824,1731.923397,2.063125839,5.870272852,1.690198102,1.690198102,1,1,10.31562919,29.351
39,5.870272852,1.690198102,1.690198102,1,1,10.31562919,29.35136426,218.06741,47.45189429
67,1,0,0,1,1,9,1548.69824,1731.923397,2.063125839,5.870272852,1.690198102,1.690198102,1,1,1
548.69824,1731.923397,2.063125839,5.870272852,1.690198102,1.690198102,1,1,10.31562919,29
,5.870272852,1.690198102,1.690198102,1,1,10.31562919,29.35136426,218.06741,47.45189429,9
67,1,0,0,1,1,9,1548.69824,1731.923397,2.063125839,5.870272852,1.690198102,1.690198102,1,1,1
67,1,0,0,1,1,9,1548.69824,1731.923397,2.063125839,5.870272852,1.690198102,1.690198102,1,1,1
8.69824,1731.923397,2.063125839,5.870272852,1.690198102,1.690198102,1,1,10.31562919,29.35
.69824,1731.923397,2.063125839,5.870272852,1.690198102,1.690198102,1,1,10.31562919,29.351
617676,3,2,212,75,0,0,0,1,1,10,1607.130336,1787.467877,4.10697485,6.609550438,1.690198102,
07.130336,1787.467877,4.10697485,6.609550438,1.690198102,1.690198102,1,1,10.26743712,19.8
07.130336,1787.467877,4.10697485,6.609550438,1.690198102,1.690198102,1,1,10.26743712,19.8
07.130336,1787.467877,4.10697485,6.609550438,1.690198102,1.690198102,1,1,10.26743712,19.8
5,6.609550438,1.690198102,1.690198102,1,1,10.26743712,19.82865132,233.9039137,51.3371856
7,4.10697485,6.609550438,1.690198102,1.690198102,1,1,10.26743712,19.82865132,233.9039137
07.130336,1787.467877,4.10697485,6.609550438,1.690198102,1.690198102,1,1,10.26743712,19.8
5,6.609550438,1.690198102,1.690198102,1,1,10.26743712,19.82865132,233.9039137,51.3371856
07.130336,1787.467877,4.10697485,6.609550438,1.690198102,1.690198102,1,1,10.26743712,19.8
07.130336,1787.467877,4.10697485,6.609550438,1.690198102,1.690198102,1,1,10.26743712,19.8
697485,6.609550438,1.690198102,1.690198102,1,1,10.26743712,19.82865132,233.9039137,51.33
10697485,6.609550438,1.690198102,1.690198102,1,1,10.26743712,19.82865132,233.9039137,51.
75,0,0,0,1,1,10,1607.130336,1787.467877,4.10697485,6.609550438,1.690198102,1.690198102,1,1,
607.130336,1787.467877,4.10697485,6.609550438,1.690198102,1.690198102,1,1,10.26743712,19.
607.130336,1787.467877,4.10697485,6.609550438,1.690198102,1.690198102,1,1,10.26743712,19.
,6.609550438,1.690198102,1.690198102,1,1,10.26743712,19.82865132,233.9039137,51.33718562
5,0,0,0,1,1,10,1607.130336,1787.467877,4.10697485,6.609550438,1.690198102,1.690198102,1,1,1
5,0,0,0,1,1,10,1607.130336,1787.467877,4.10697485,6.609550438,1.690198102,1.690198102,1,1,1
07.130336,1787.467877,4.10697485,6.609550438,1.690198102,1.690198102,1,1,10.26743712,19.8
7.130336,1787.467877,4.10697485,6.609550438,1.690198102,1.690198102,1,1,10.26743712,19.82
485,6.609550438,1.690198102,1.690198102,1,1,10.26743712,19.82865132,233.9039137,51.33718
5,0,0,0,1,1,10,1607.130336,1787.467877,4.10697485,6.609550438,1.690198102,1.690198102,1,1,1
10697485,6.609550438,1.690198102,1.690198102,1,1,10.26743712,19.82865132,233.9039137,51.
10697485,6.609550438,1.690198102,1.690198102,1,1,10.26743712,19.82865132,233.9039137,51.
7.130336,1787.467877,4.10697485,6.609550438,1.690198102,1.690198102,1,1,10.26743712,19.82
07.130336,1787.467877,4.10697485,6.609550438,1.690198102,1.690198102,1,1,10.26743712,19.8
07.130336,1787.467877,4.10697485,6.609550438,1.690198102,1.690198102,1,1,10.26743712,19.8
607.130336,1787.467877,4.10697485,6.609550438,1.690198102,1.690198102,1,1,10.26743712,19.
10697485,6.609550438,1.690198102,1.690198102,1,1,10.26743712,19.82865132,233.9039137,51.
7.130336,1787.467877,4.10697485,6.609550438,1.690198102,1.690198102,1,1,10.26743712,19.82
,6.609550438,1.690198102,1.690198102,1,1,10.26743712,19.82865132,233.9039137,51.33718562
5,0,0,0,1,1,10,1607.130336,1787.467877,4.10697485,6.609550438,1.690198102,1.690198102,1,1,1
5,0,0,0,1,1,10,1607.130336,1787.467877,4.10697485,6.609550438,1.690198102,1.690198102,1,1,1
697485,6.609550438,1.690198102,1.690198102,1,1,10.26743712,19.82865132,233.9039137,51.33
5,6.609550438,1.690198102,1.690198102,1,1,10.26743712,19.82865132,233.9039137,51.3371856

5,6.609550438,1.690198102,1.690198102,1,1,10.26743712,19.82865132,233.9039137,51.33718562
39,5.870272852,1.690198102,1.690198102,1,1,10.31562919,29.35136426,218.06741,47.45189429,9
3125839,5.870272852,1.690198102,1.690198102,1,1,10.31562919,29.35136426,218.06741,47.4518
8.69824,1731.923397,2.063125839,5.870272852,1.690198102,1.690198102,1,1,10.31562919,29.35
548.69824,1731.923397,2.063125839,5.870272852,1.690198102,1.690198102,1,1,10.31562919,29.
39,5.870272852,1.690198102,1.690198102,1,1,10.31562919,29.35136426,218.06741,47.45189429,9
39,5.870272852,1.690198102,1.690198102,1,1,10.31562919,29.35136426,218.06741,47.45189429,9
548.69824,1731.923397,2.063125839,5.870272852,1.690198102,1.690198102,1,1,10.31562919,29.
3125839,5.870272852,1.690198102,1.690198102,1,1,10.31562919,29.35136426,218.06741,47.4518
48.69824,1731.923397,2.063125839,5.870272852,1.690198102,1.690198102,1,1,10.31562919,29.3
39,5.870272852,1.690198102,1.690198102,1,1,10.31562919,29.35136426,218.06741,47.45189429,9
39,5.870272852,1.690198102,1.690198102,1,1,10.31562919,29.35136426,218.06741,47.45189429,9
48.69824,1731.923397,2.063125839,5.870272852,1.690198102,1.690198102,1,1,10.31562919,29.3
25839,5.870272852,1.690198102,1.690198102,1,1,10.31562919,29.35136426,218.06741,47.451894
39,5.870272852,1.690198102,1.690198102,1,1,10.31562919,29.35136426,218.06741,47.45189429,9
8.69824,1731.923397,2.063125839,5.870272852,1.690198102,1.690198102,1,1,10.31562919,29.35
5839,5.870272852,1.690198102,1.690198102,1,1,10.31562919,29.35136426,218.06741,47.4518942
66,1,1,0,1,1,11,1548.69824,1731.923397,2.063125839,5.870272852,1.690198102,1.690198102,1,1,
63125839,5.870272852,1.690198102,1.690198102,1,1,10.31562919,29.35136426,218.06741,47.451
25839,5.870272852,1.690198102,1.690198102,1,1,10.31562919,29.35136426,218.06741,47.451894
48.69824,1731.923397,2.063125839,5.870272852,1.690198102,1.690198102,1,1,10.31562919,29.3
25839,5.870272852,1.690198102,1.690198102,1,1,10.31562919,29.35136426,218.06741,47.451894
8.69824,1731.923397,2.063125839,5.870272852,1.690198102,1.690198102,1,1,10.31562919,29.35
66,1,1,0,1,1,11,1548.69824,1731.923397,2.063125839,5.870272852,1.690198102,1.690198102,1,1,
8.69824,1731.923397,2.063125839,5.870272852,1.690198102,1.690198102,1,1,10.31562919,29.35
3125839,5.870272852,1.690198102,1.690198102,1,1,10.31562919,29.35136426,218.06741,47.4518
9382324,3,2,212,66,1,1,0,1,1,11,1548.69824,1731.923397,2.063125839,5.870272852,1.690198102,1
8.69824,1731.923397,2.063125839,5.870272852,1.690198102,1.690198102,1,1,10.31562919,29.35
548.69824,1731.923397,2.063125839,5.870272852,1.690198102,1.690198102,1,1,10.31562919,29.
66,1,1,0,1,1,11,1548.69824,1731.923397,2.063125839,5.870272852,1.690198102,1.690198102,1,1,
469,4.553126573,1.690198102,1.690198102,1,1,11.67389192,9.106253147,173.8260445,52.532513
377709469,4.553126573,1.690198102,1.690198102,1,1,11.67389192,9.106253147,173.8260445,52
2.358901,1940.685086,4.377709469,4.553126573,1.690198102,1.690198102,1,1,11.67389192,9.10
469,4.553126573,1.690198102,1.690198102,1,1,11.67389192,9.106253147,173.8260445,52.532513
2.358901,1940.685086,4.377709469,4.553126573,1.690198102,1.690198102,1,1,11.67389192,9.10
09469,4.553126573,1.690198102,1.690198102,1,1,11.67389192,9.106253147,173.8260445,52.5325
66,1,0,0,1,1,12,1652.358901,1940.685086,4.377709469,4.553126573,1.690198102,1.690198102,1,1
52.358901,1940.685086,4.377709469,4.553126573,1.690198102,1.690198102,1,1,11.67389192,9.1
469,4.553126573,1.690198102,1.690198102,1,1,11.67389192,9.106253147,173.8260445,52.532513
52.358901,1940.685086,4.377709469,4.553126573,1.690198102,1.690198102,1,1,11.67389192,9.1
709469,4.553126573,1.690198102,1.690198102,1,1,11.67389192,9.106253147,173.8260445,52.532
2.358901,1940.685086,4.377709469,4.553126573,1.690198102,1.690198102,1,1,11.67389192,9.10
709469,4.553126573,1.690198102,1.690198102,1,1,11.67389192,9.106253147,173.8260445,52.532
66,1,0,0,1,1,12,1652.358901,1940.685086,4.377709469,4.553126573,1.690198102,1.690198102,1,1
2.358901,1940.685086,4.377709469,4.553126573,1.690198102,1.690198102,1,1,11.67389192,9.10
469,4.553126573,1.690198102,1.690198102,1,1,11.67389192,9.106253147,173.8260445,52.532513
36,4.377709469,4.553126573,1.690198102,1.690198102,1,1,11.67389192,9.106253147,173.826044
2.358901,1940.685086,4.377709469,4.553126573,1.690198102,1.690198102,1,1,11.67389192,9.10
709469,4.553126573,1.690198102,1.690198102,1,1,11.67389192,9.106253147,173.8260445,52.532
52.358901,1940.685086,4.377709469,4.553126573,1.690198102,1.690198102,1,1,11.67389192,9.1

9,4.553126573,1.690198102,1.690198102,1,1,11.67389192,9.106253147,173.8260445,52.5325136
66,1,0,0,1,1,12,1652.358901,1940.685086,4.377709469,4.553126573,1.690198102,1.690198102,1,
2.358901,1940.685086,4.377709469,4.553126573,1.690198102,1.690198102,1,1,11.67389192,9.10
652.358901,1940.685086,4.377709469,4.553126573,1.690198102,1.690198102,1,1,11.67389192,9.
469,4.553126573,1.690198102,1.690198102,1,1,11.67389192,9.106253147,173.8260445,52.532513
469,4.553126573,1.690198102,1.690198102,1,1,11.67389192,9.106253147,173.8260445,52.532513
709469,4.553126573,1.690198102,1.690198102,1,1,11.67389192,9.106253147,173.8260445,52.532
469,4.553126573,1.690198102,1.690198102,1,1,11.67389192,9.106253147,173.8260445,52.532513
52.358901,1940.685086,4.377709469,4.553126573,1.690198102,1.690198102,1,1,11.67389192,9.1
469,4.553126573,1.690198102,1.690198102,1,1,11.67389192,9.106253147,173.8260445,52.532513
22,4.788694755,1.690198102,1.690198102,1,1,11.81561856,9.577389511,203.0368945,52.7764295
72,0,1,0,1,1,12,1644.295588,2001.848272,4.726247422,4.788694755,1.690198102,1.690198102,1,
4.295588,2001.848272,4.726247422,4.788694755,1.690198102,1.690198102,1,1,11.81561856,9.57
422,4.788694755,1.690198102,1.690198102,1,1,11.81561856,9.577389511,203.0368945,52.776429
4.295588,2001.848272,4.726247422,4.788694755,1.690198102,1.690198102,1,1,11.81561856,9.57
2,0,1,0,1,1,12,1644.295588,2001.848272,4.726247422,4.788694755,1.690198102,1.690198102,1,1,
2,4.726247422,4.788694755,1.690198102,1.690198102,1,1,11.81561856,9.577389511,203.036894
1644.295588,2001.848272,4.726247422,4.788694755,1.690198102,1.690198102,1,1,11.81561856,9
644.295588,2001.848272,4.726247422,4.788694755,1.690198102,1.690198102,1,1,11.81561856,9.
44.295588,2001.848272,4.726247422,4.788694755,1.690198102,1.690198102,1,1,11.81561856,9.5
517676,3,2,212,72,0,1,0,1,1,12,1644.295588,2001.848272,4.726247422,4.788694755,1.690198102,
422,4.788694755,1.690198102,1.690198102,1,1,11.81561856,9.577389511,203.0368945,52.776429
2,0,1,0,1,1,12,1644.295588,2001.848272,4.726247422,4.788694755,1.690198102,1.690198102,1,1,
4.295588,2001.848272,4.726247422,4.788694755,1.690198102,1.690198102,1,1,11.81561856,9.57
4.295588,2001.848272,4.726247422,4.788694755,1.690198102,1.690198102,1,1,11.81561856,9.57
9382324,3,2,212,72,0,1,0,1,1,12,1644.295588,2001.848272,4.726247422,4.788694755,1.690198102
44.295588,2001.848272,4.726247422,4.788694755,1.690198102,1.690198102,1,1,11.81561856,9.5
2,0,1,0,1,1,12,1644.295588,2001.848272,4.726247422,4.788694755,1.690198102,1.690198102,1,1,
4.295588,2001.848272,4.726247422,4.788694755,1.690198102,1.690198102,1,1,11.81561856,9.57
422,4.788694755,1.690198102,1.690198102,1,1,11.81561856,9.577389511,203.0368945,52.776429
72,0,1,0,1,1,12,1644.295588,2001.848272,4.726247422,4.788694755,1.690198102,1.690198102,1,
49443,4.344568588,1.690198102,1.690198102,1,1,12.48254833,8.689137177,153.8723418,53.3975
349443,4.344568588,1.690198102,1.690198102,1,1,12.48254833,8.689137177,153.8723418,53.397
4,0,0,0,1,1,12,1633.984958,1934.234114,4.160849443,4.344568588,1.690198102,1.690198102,1,1,
443,4.344568588,1.690198102,1.690198102,1,1,12.48254833,8.689137177,153.8723418,53.397567
33.984958,1934.234114,4.160849443,4.344568588,1.690198102,1.690198102,1,1,12.48254833,8.6
349443,4.344568588,1.690198102,1.690198102,1,1,12.48254833,8.689137177,153.8723418,53.397
3.984958,1934.234114,4.160849443,4.344568588,1.690198102,1.690198102,1,1,12.48254833,8.68
74,0,0,0,1,1,12,1633.984958,1934.234114,4.160849443,4.344568588,1.690198102,1.690198102,1,
160849443,4.344568588,1.690198102,1.690198102,1,1,12.48254833,8.689137177,153.8723418,53
3.984958,1934.234114,4.160849443,4.344568588,1.690198102,1.690198102,1,1,12.48254833,8.68
9382324,3,2,212,74,0,0,0,1,1,12,1633.984958,1934.234114,4.160849443,4.344568588,1.690198102
3.984958,1934.234114,4.160849443,4.344568588,1.690198102,1.690198102,1,1,12.48254833,8.68
4,0,0,0,1,1,12,1633.984958,1934.234114,4.160849443,4.344568588,1.690198102,1.690198102,1,1,
33.984958,1934.234114,4.160849443,4.344568588,1.690198102,1.690198102,1,1,12.48254833,8.6
443,4.344568588,1.690198102,1.690198102,1,1,12.48254833,8.689137177,153.8723418,53.397567
633.984958,1934.234114,4.160849443,4.344568588,1.690198102,1.690198102,1,1,12.48254833,8.
3.984958,1934.234114,4.160849443,4.344568588,1.690198102,1.690198102,1,1,12.48254833,8.68
633.984958,1934.234114,4.160849443,4.344568588,1.690198102,1.690198102,1,1,12.48254833,8.
349443,4.344568588,1.690198102,1.690198102,1,1,12.48254833,8.689137177,153.8723418,53.397

60849443,4.344568588,1.690198102,1.690198102,1,1,12.48254833,8.689137177,153.8723418,53.3
443,4.344568588,1.690198102,1.690198102,1,1,12.48254833,8.689137177,153.8723418,53.397567
443,4.344568588,1.690198102,1.690198102,1,1,12.48254833,8.689137177,153.8723418,53.397567
33.984958,1934.234114,4.160849443,4.344568588,1.690198102,1.690198102,1,1,12.48254833,8.6
849443,4.344568588,1.690198102,1.690198102,1,1,12.48254833,8.689137177,153.8723418,53.397
443,4.344568588,1.690198102,1.690198102,1,1,12.48254833,8.689137177,153.8723418,53.397567
3.984958,1934.234114,4.160849443,4.344568588,1.690198102,1.690198102,1,1,12.48254833,8.68
3.984958,1934.234114,4.160849443,4.344568588,1.690198102,1.690198102,1,1,12.48254833,8.68
443,4.344568588,1.690198102,1.690198102,1,1,12.48254833,8.689137177,153.8723418,53.397567
3.984958,1934.234114,4.160849443,4.344568588,1.690198102,1.690198102,1,1,12.48254833,8.68
0.995833,2054.845439,4.321171776,5.232528323,1.690198102,1.690198102,1,1,12.09928097,10.4
650.995833,2054.845439,4.321171776,5.232528323,1.690198102,1.690198102,1,1,12.09928097,10
69,1,0,0,1,1,11,1650.995833,2054.845439,4.321171776,5.232528323,1.690198102,1.690198102,1,
2370605,3,2,212,69,1,0,0,1,1,11,1650.995833,2054.845439,4.321171776,5.232528323,1.690198102
776,5.232528323,1.690198102,1.690198102,1,1,12.09928097,10.46505665,259.7371842,56.175233
9,1,0,0,1,1,11,1650.995833,2054.845439,4.321171776,5.232528323,1.690198102,1.690198102,1,1,
321171776,5.232528323,1.690198102,1.690198102,1,1,12.09928097,10.46505665,259.7371842,56
69,1,0,0,1,1,11,1650.995833,2054.845439,4.321171776,5.232528323,1.690198102,1.690198102,1,
650.995833,2054.845439,4.321171776,5.232528323,1.690198102,1.690198102,1,1,12.09928097,10
9,1,0,0,1,1,11,1650.995833,2054.845439,4.321171776,5.232528323,1.690198102,1.690198102,1,1,
650.995833,2054.845439,4.321171776,5.232528323,1.690198102,1.690198102,1,1,12.09928097,10
50.995833,2054.845439,4.321171776,5.232528323,1.690198102,1.690198102,1,1,12.09928097,10.
171776,5.232528323,1.690198102,1.690198102,1,1,12.09928097,10.46505665,259.7371842,56.175
382324,3,2,212,69,1,0,0,1,1,11,1650.995833,2054.845439,4.321171776,5.232528323,1.690198102,
21171776,5.232528323,1.690198102,1.690198102,1,1,12.09928097,10.46505665,259.7371842,56.1
9,1,0,0,1,1,11,1650.995833,2054.845439,4.321171776,5.232528323,1.690198102,1.690198102,1,1,
4741211,3,2,212,69,1,0,0,1,1,11,1650.995833,2054.845439,4.321171776,5.232528323,1.690198102
0.995833,2054.845439,4.321171776,5.232528323,1.690198102,1.690198102,1,1,12.09928097,10.4
9,1,0,0,1,1,11,1650.995833,2054.845439,4.321171776,5.232528323,1.690198102,1.690198102,1,1,
0.995833,2054.845439,4.321171776,5.232528323,1.690198102,1.690198102,1,1,12.09928097,10.4
776,5.232528323,1.690198102,1.690198102,1,1,12.09928097,10.46505665,259.7371842,56.175233
9382324,3,2,212,68,1,1,0,1,1,12,1644.295588,2001.848272,4.726247422,4.788694755,1.690198102
68,1,1,0,1,1,12,1644.295588,2001.848272,4.726247422,4.788694755,1.690198102,1.690198102,1,
4.295588,2001.848272,4.726247422,4.788694755,1.690198102,1.690198102,1,1,11.81561856,9.57
4.295588,2001.848272,4.726247422,4.788694755,1.690198102,1.690198102,1,1,11.81561856,9.57
422,4.788694755,1.690198102,1.690198102,1,1,11.81561856,9.577389511,203.0368945,52.776429
26247422,4.788694755,1.690198102,1.690198102,1,1,11.81561856,9.577389511,203.0368945,52.7
4.295588,2001.848272,4.726247422,4.788694755,1.690198102,1.690198102,1,1,11.81561856,9.57
68,1,1,0,1,1,12,1644.295588,2001.848272,4.726247422,4.788694755,1.690198102,1.690198102,1,
8,1,1,0,1,1,12,1644.295588,2001.848272,4.726247422,4.788694755,1.690198102,1.690198102,1,1,
422,4.788694755,1.690198102,1.690198102,1,1,11.81561856,9.577389511,203.0368945,52.776429
644.295588,2001.848272,4.726247422,4.788694755,1.690198102,1.690198102,1,1,11.81561856,9.
422,4.788694755,1.690198102,1.690198102,1,1,11.81561856,9.577389511,203.0368945,52.776429
44.295588,2001.848272,4.726247422,4.788694755,1.690198102,1.690198102,1,1,11.81561856,9.5
422,4.788694755,1.690198102,1.690198102,1,1,11.81561856,9.577389511,203.0368945,52.776429
422,4.788694755,1.690198102,1.690198102,1,1,11.81561856,9.577389511,203.0368945,52.776429
44.295588,2001.848272,4.726247422,4.788694755,1.690198102,1.690198102,1,1,11.81561856,9.5
4.295588,2001.848272,4.726247422,4.788694755,1.690198102,1.690198102,1,1,11.81561856,9.57
422,4.788694755,1.690198102,1.690198102,1,1,11.81561856,9.577389511,203.0368945,52.776429
4.295588,2001.848272,4.726247422,4.788694755,1.690198102,1.690198102,1,1,11.81561856,9.57

47422,4.788694755,1.690198102,1.690198102,1,1,11.81561856,9.577389511,203.0368945,52.7764
68,1,1,0,1,1,12,1644.295588,2001.848272,4.726247422,4.788694755,1.690198102,1.690198102,1,
8,1,1,0,1,1,12,1644.295588,2001.848272,4.726247422,4.788694755,1.690198102,1.690198102,1,1,
422,4.788694755,1.690198102,1.690198102,1,1,11.81561856,9.577389511,203.0368945,52.776429
44.295588,2001.848272,4.726247422,4.788694755,1.690198102,1.690198102,1,1,11.81561856,9.5
247422,4.788694755,1.690198102,1.690198102,1,1,11.81561856,9.577389511,203.0368945,52.776
8,1,1,0,1,1,12,1644.295588,2001.848272,4.726247422,4.788694755,1.690198102,1.690198102,1,1,
26247422,4.788694755,1.690198102,1.690198102,1,1,11.81561856,9.577389511,203.0368945,52.7
422,4.788694755,1.690198102,1.690198102,1,1,11.81561856,9.577389511,203.0368945,52.776429
4.295588,2001.848272,4.726247422,4.788694755,1.690198102,1.690198102,1,1,11.81561856,9.57
776,5.232528323,1.690198102,1.690198102,1,1,12.09928097,10.46505665,259.7371842,56.175233
650.995833,2054.845439,4.321171776,5.232528323,1.690198102,1.690198102,1,1,12.09928097,1
0.995833,2054.845439,4.321171776,5.232528323,1.690198102,1.690198102,1,1,12.09928097,10.4
776,5.232528323,1.690198102,1.690198102,1,1,12.09928097,10.46505665,259.7371842,56.175233
76,5.232528323,1.690198102,1.690198102,1,1,12.09928097,10.46505665,259.7371842,56.1752330
776,5.232528323,1.690198102,1.690198102,1,1,12.09928097,10.46505665,259.7371842,56.175233
776,5.232528323,1.690198102,1.690198102,1,1,12.09928097,10.46505665,259.7371842,56.175233
50.995833,2054.845439,4.321171776,5.232528323,1.690198102,1.690198102,1,1,12.09928097,10.
776,5.232528323,1.690198102,1.690198102,1,1,12.09928097,10.46505665,259.7371842,56.175233
776,5.232528323,1.690198102,1.690198102,1,1,12.09928097,10.46505665,259.7371842,56.175233
71,0,0,0,1,1,12,1650.995833,2054.845439,4.321171776,5.232528323,1.690198102,1.690198102,1,
0.995833,2054.845439,4.321171776,5.232528323,1.690198102,1.690198102,1,1,12.09928097,10.4
21171776,5.232528323,1.690198102,1.690198102,1,1,12.09928097,10.46505665,259.7371842,56.1
776,5.232528323,1.690198102,1.690198102,1,1,12.09928097,10.46505665,259.7371842,56.175233
4.321171776,5.232528323,1.690198102,1.690198102,1,1,12.09928097,10.46505665,259.7371842,5
39,4.321171776,5.232528323,1.690198102,1.690198102,1,1,12.09928097,10.46505665,259.737184
776,5.232528323,1.690198102,1.690198102,1,1,12.09928097,10.46505665,259.7371842,56.175233
550.995833,2054.845439,4.321171776,5.232528323,1.690198102,1.690198102,1,1,12.09928097,10
0.995833,2054.845439,4.321171776,5.232528323,1.690198102,1.690198102,1,1,12.09928097,10.4
71776,5.232528323,1.690198102,1.690198102,1,1,12.09928097,10.46505665,259.7371842,56.1752
321171776,5.232528323,1.690198102,1.690198102,1,1,12.09928097,10.46505665,259.7371842,56
,1650.995833,2054.845439,4.321171776,5.232528323,1.690198102,1.690198102,1,1,12.09928097,
,1650.995833,2054.845439,4.321171776,5.232528323,1.690198102,1.690198102,1,1,12.09928097,
71,0,0,0,1,1,12,1650.995833,2054.845439,4.321171776,5.232528323,1.690198102,1.690198102,1,
469,4.553126573,1.690198102,1.690198102,1,1,11.67389192,9.106253147,173.8260445,52.532513
52.358901,1940.685086,4.377709469,4.553126573,1.690198102,1.690198102,1,1,11.67389192,9.1
709469,4.553126573,1.690198102,1.690198102,1,1,11.67389192,9.106253147,173.8260445,52.532
59,4.553126573,1.690198102,1.690198102,1,1,11.67389192,9.106253147,173.8260445,52.5325136
469,4.553126573,1.690198102,1.690198102,1,1,11.67389192,9.106253147,173.8260445,52.532513
09469,4.553126573,1.690198102,1.690198102,1,1,11.67389192,9.106253147,173.8260445,52.5325
469,4.553126573,1.690198102,1.690198102,1,1,11.67389192,9.106253147,173.8260445,52.532513
652.358901,1940.685086,4.377709469,4.553126573,1.690198102,1.690198102,1,1,11.67389192,9.
469,4.553126573,1.690198102,1.690198102,1,1,11.67389192,9.106253147,173.8260445,52.532513
469,4.553126573,1.690198102,1.690198102,1,1,11.67389192,9.106253147,173.8260445,52.532513
9,0,0,0,1,1,12,1652.358901,1940.685086,4.377709469,4.553126573,1.690198102,1.690198102,1,1,
2.358901,1940.685086,4.377709469,4.553126573,1.690198102,1.690198102,1,1,11.67389192,9.10
652.358901,1940.685086,4.377709469,4.553126573,1.690198102,1.690198102,1,1,11.67389192,9.
469,4.553126573,1.690198102,1.690198102,1,1,11.67389192,9.106253147,173.8260445,52.532513
9,0,0,0,1,1,12,1652.358901,1940.685086,4.377709469,4.553126573,1.690198102,1.690198102,1,1,
9,0,0,0,1,1,12,1652.358901,1940.685086,4.377709469,4.553126573,1.690198102,1.690198102,1,1,

77709469,4.553126573,1.690198102,1.690198102,1,1,11.67389192,9.106253147,173.8260445,52.5
2.358901,1940.685086,4.377709469,4.553126573,1.690198102,1.690198102,1,1,11.67389192,9.10
59,4.553126573,1.690198102,1.690198102,1,1,11.67389192,9.106253147,173.8260445,52.5325136
52.358901,1940.685086,4.377709469,4.553126573,1.690198102,1.690198102,1,1,11.67389192,9.1
652.358901,1940.685086,4.377709469,4.553126573,1.690198102,1.690198102,1,1,11.67389192,9.
377709469,4.553126573,1.690198102,1.690198102,1,1,11.67389192,9.106253147,173.8260445,52
652.358901,1940.685086,4.377709469,4.553126573,1.690198102,1.690198102,1,1,11.67389192,9.
09469,4.553126573,1.690198102,1.690198102,1,1,11.67389192,9.106253147,173.8260445,52.5325
59,4.553126573,1.690198102,1.690198102,1,1,11.67389192,9.106253147,173.8260445,52.5325136
469,4.553126573,1.690198102,1.690198102,1,1,11.67389192,9.106253147,173.8260445,52.532513
77709469,4.553126573,1.690198102,1.690198102,1,1,11.67389192,9.106253147,173.8260445,52.5
652.358901,1940.685086,4.377709469,4.553126573,1.690198102,1.690198102,1,1,11.67389192,9.
09469,4.553126573,1.690198102,1.690198102,1,1,11.67389192,9.106253147,173.8260445,52.5325
52.358901,1940.685086,4.377709469,4.553126573,1.690198102,1.690198102,1,1,11.67389192,9.1
9,0,0,0,1,1,12,1652.358901,1940.685086,4.377709469,4.553126573,1.690198102,1.690198102,1,1,
9,0,0,0,1,1,12,1652.358901,1940.685086,4.377709469,4.553126573,1.690198102,1.690198102,1,1,
377709469,4.553126573,1.690198102,1.690198102,1,1,11.67389192,9.106253147,173.8260445,52
6,0,0,0,1,1,12,1633.984958,1934.234114,4.160849443,4.344568588,1.690198102,1.690198102,1,1,
849443,4.344568588,1.690198102,1.690198102,1,1,12.48254833,8.689137177,153.8723418,53.397
76,0,0,0,1,1,12,1633.984958,1934.234114,4.160849443,4.344568588,1.690198102,1.690198102,1,
3.984958,1934.234114,4.160849443,4.344568588,1.690198102,1.690198102,1,1,12.48254833,8.68
633.984958,1934.234114,4.160849443,4.344568588,1.690198102,1.690198102,1,1,12.48254833,8.
849443,4.344568588,1.690198102,1.690198102,1,1,12.48254833,8.689137177,153.8723418,53.397
76,0,0,0,1,1,12,1633.984958,1934.234114,4.160849443,4.344568588,1.690198102,1.690198102,1,
3.984958,1934.234114,4.160849443,4.344568588,1.690198102,1.690198102,1,1,12.48254833,8.68
633.984958,1934.234114,4.160849443,4.344568588,1.690198102,1.690198102,1,1,12.48254833,8.
3.984958,1934.234114,4.160849443,4.344568588,1.690198102,1.690198102,1,1,12.48254833,8.68
849443,4.344568588,1.690198102,1.690198102,1,1,12.48254833,8.689137177,153.8723418,53.397
33.984958,1934.234114,4.160849443,4.344568588,1.690198102,1.690198102,1,1,12.48254833,8.6
33.984958,1934.234114,4.160849443,4.344568588,1.690198102,1.690198102,1,1,12.48254833,8.6
849443,4.344568588,1.690198102,1.690198102,1,1,12.48254833,8.689137177,153.8723418,53.397
633.984958,1934.234114,4.160849443,4.344568588,1.690198102,1.690198102,1,1,12.48254833,8.
849443,4.344568588,1.690198102,1.690198102,1,1,12.48254833,8.689137177,153.8723418,53.397
6,0,0,0,1,1,12,1633.984958,1934.234114,4.160849443,4.344568588,1.690198102,1.690198102,1,1,
33.984958,1934.234114,4.160849443,4.344568588,1.690198102,1.690198102,1,1,12.48254833,8.6
3.984958,1934.234114,4.160849443,4.344568588,1.690198102,1.690198102,1,1,12.48254833,8.68
3.984958,1934.234114,4.160849443,4.344568588,1.690198102,1.690198102,1,1,12.48254833,8.68
,0,0,0,1,1,12,1633.984958,1934.234114,4.160849443,4.344568588,1.690198102,1.690198102,1,1,1
33.984958,1934.234114,4.160849443,4.344568588,1.690198102,1.690198102,1,1,12.48254833,8.6
76,0,0,0,1,1,12,1633.984958,1934.234114,4.160849443,4.344568588,1.690198102,1.690198102,1,
633.984958,1934.234114,4.160849443,4.344568588,1.690198102,1.690198102,1,1,12.48254833,8.
160849443,4.344568588,1.690198102,1.690198102,1,1,12.48254833,8.689137177,153.8723418,53
50849443,4.344568588,1.690198102,1.690198102,1,1,12.48254833,8.689137177,153.8723418,53.3
633.984958,1934.234114,4.160849443,4.344568588,1.690198102,1.690198102,1,1,12.48254833,8.
3.984958,1934.234114,4.160849443,4.344568588,1.690198102,1.690198102,1,1,12.48254833,8.68
3.984958,1934.234114,4.160849443,4.344568588,1.690198102,1.690198102,1,1,12.48254833,8.68
3.984958,1934.234114,4.160849443,4.344568588,1.690198102,1.690198102,1,1,12.48254833,8.68
6,0,0,0,1,1,12,1633.984958,1934.234114,4.160849443,4.344568588,1.690198102,1.690198102,1,1,
3.984958,1934.234114,4.160849443,4.344568588,1.690198102,1.690198102,1,1,12.48254833,8.68
76,0,0,0,1,1,12,1633.984958,1934.234114,4.160849443,4.344568588,1.690198102,1.690198102,1,

6,0,0,0,1,1,12,1633.984958,1934.234114,4.160849443,4.344568588,1.690198102,1.690198102,1,1,
767,14.36694006,7.523155123,4.29308169,7,2,15.16388935,7.183470031,380.0578983,60.6555573
363.404579,1219.513101,9.649747767,14.36694006,7.523155123,4.29308169,7,2,15.16388935,7.1
747767,14.36694006,7.523155123,4.29308169,7,2,15.16388935,7.183470031,380.0578983,60.6555
63.404579,1219.513101,9.649747767,14.36694006,7.523155123,4.29308169,7,2,15.16388935,7.18
63.404579,1219.513101,9.649747767,14.36694006,7.523155123,4.29308169,7,2,15.16388935,7.18
3.404579,1219.513101,9.649747767,14.36694006,7.523155123,4.29308169,7,2,15.16388935,7.183
363.404579,1219.513101,9.649747767,14.36694006,7.523155123,4.29308169,7,2,15.16388935,7.1
747767,14.36694006,7.523155123,4.29308169,7,2,15.16388935,7.183470031,380.0578983,60.6555
363.404579,1219.513101,9.649747767,14.36694006,7.523155123,4.29308169,7,2,15.16388935,7.1
747767,14.36694006,7.523155123,4.29308169,7,2,15.16388935,7.183470031,380.0578983,60.6555
649747767,14.36694006,7.523155123,4.29308169,7,2,15.16388935,7.183470031,380.0578983,60.
3.404579,1219.513101,9.649747767,14.36694006,7.523155123,4.29308169,7,2,15.16388935,7.183
747767,14.36694006,7.523155123,4.29308169,7,2,15.16388935,7.183470031,380.0578983,60.6555
63.404579,1219.513101,9.649747767,14.36694006,7.523155123,4.29308169,7,2,15.16388935,7.18
363.404579,1219.513101,9.649747767,14.36694006,7.523155123,4.29308169,7,2,15.16388935,7.1
3.404579,1219.513101,9.649747767,14.36694006,7.523155123,4.29308169,7,2,15.16388935,7.183
649747767,14.36694006,7.523155123,4.29308169,7,2,15.16388935,7.183470031,380.0578983,60.
747767,14.36694006,7.523155123,4.29308169,7,2,15.16388935,7.183470031,380.0578983,60.6555
1,0,1,0,1,1,12,2363.404579,1219.513101,9.649747767,14.36694006,7.523155123,4.29308169,7,2,1
63.404579,1219.513101,9.649747767,14.36694006,7.523155123,4.29308169,7,2,15.16388935,7.18
3.404579,1219.513101,9.649747767,14.36694006,7.523155123,4.29308169,7,2,15.16388935,7.183
3.404579,1219.513101,9.649747767,14.36694006,7.523155123,4.29308169,7,2,15.16388935,7.183
649747767,14.36694006,7.523155123,4.29308169,7,2,15.16388935,7.183470031,380.0578983,60.
63.404579,1219.513101,9.649747767,14.36694006,7.523155123,4.29308169,7,2,15.16388935,7.18
71,0,1,0,1,1,12,2363.404579,1219.513101,9.649747767,14.36694006,7.523155123,4.29308169,7,2,
363.404579,1219.513101,9.649747767,14.36694006,7.523155123,4.29308169,7,2,15.16388935,7.1
71,0,1,0,1,1,12,2363.404579,1219.513101,9.649747767,14.36694006,7.523155123,4.29308169,7,2,
3.404579,1219.513101,9.649747767,14.36694006,7.523155123,4.29308169,7,2,15.16388935,7.183
649747767,14.36694006,7.523155123,4.29308169,7,2,15.16388935,7.183470031,380.0578983,60.
3.404579,1219.513101,9.649747767,14.36694006,7.523155123,4.29308169,7,2,15.16388935,7.183
3.404579,1219.513101,9.649747767,14.36694006,7.523155123,4.29308169,7,2,15.16388935,7.183
3.404579,1219.513101,9.649747767,14.36694006,7.523155123,4.29308169,7,2,15.16388935,7.183
747767,14.36694006,7.523155123,4.29308169,7,2,15.16388935,7.183470031,380.0578983,60.6555
3.404579,1219.513101,9.649747767,14.36694006,7.523155123,4.29308169,7,2,15.16388935,7.183
71,0,1,0,1,1,12,2363.404579,1219.513101,9.649747767,14.36694006,7.523155123,4.29308169,7,2,
1,0,1,0,1,1,12,2363.404579,1219.513101,9.649747767,14.36694006,7.523155123,4.29308169,7,2,1
911868,6.158447685,6.532031315,1.690198102,3,1,14.02298848,18.47534305,136.7821393,59.597
63.879042,1585.449327,5.842911868,6.158447685,6.532031315,1.690198102,3,1,14.02298848,18.
763.879042,1585.449327,5.842911868,6.158447685,6.532031315,1.690198102,3,1,14.02298848,18
868,6.158447685,6.532031315,1.690198102,3,1,14.02298848,18.47534305,136.7821393,59.597701
763.879042,1585.449327,5.842911868,6.158447685,6.532031315,1.690198102,3,1,14.02298848,18
911868,6.158447685,6.532031315,1.690198102,3,1,14.02298848,18.47534305,136.7821393,59.597
8,1,0,1,1,1,10,1763.879042,1585.449327,5.842911868,6.158447685,6.532031315,1.690198102,3,1,
63.879042,1585.449327,5.842911868,6.158447685,6.532031315,1.690198102,3,1,14.02298848,18.
3.879042,1585.449327,5.842911868,6.158447685,6.532031315,1.690198102,3,1,14.02298848,18.4
3.879042,1585.449327,5.842911868,6.158447685,6.532031315,1.690198102,3,1,14.02298848,18.4
,1,0,1,1,1,10,1763.879042,1585.449327,5.842911868,6.158447685,6.532031315,1.690198102,3,1,1
63.879042,1585.449327,5.842911868,6.158447685,6.532031315,1.690198102,3,1,14.02298848,18.
78,1,0,1,1,1,10,1763.879042,1585.449327,5.842911868,6.158447685,6.532031315,1.690198102,3,

763.879042,1585.449327,5.842911868,6.158447685,6.532031315,1.690198102,3,1,14.02298848,18
78,1,0,1,1,1,10,1763.879042,1585.449327,5.842911868,6.158447685,6.532031315,1.690198102,3,
0617676,3,2,212,78,1,0,1,1,1,10,1763.879042,1585.449327,5.842911868,6.158447685,6.532031315
763.879042,1585.449327,5.842911868,6.158447685,6.532031315,1.690198102,3,1,14.02298848,18
3.879042,1585.449327,5.842911868,6.158447685,6.532031315,1.690198102,3,1,14.02298848,18.4
3.879042,1585.449327,5.842911868,6.158447685,6.532031315,1.690198102,3,1,14.02298848,18.4
3.879042,1585.449327,5.842911868,6.158447685,6.532031315,1.690198102,3,1,14.02298848,18.4
868,6.158447685,6.532031315,1.690198102,3,1,14.02298848,18.47534305,136.7821393,59.597701
8,1,0,1,1,1,10,1763.879042,1585.449327,5.842911868,6.158447685,6.532031315,1.690198102,3,1,
3.879042,1585.449327,5.842911868,6.158447685,6.532031315,1.690198102,3,1,14.02298848,18.4
78,1,0,1,1,1,10,1763.879042,1585.449327,5.842911868,6.158447685,6.532031315,1.690198102,3,
8,1,0,1,1,1,10,1763.879042,1585.449327,5.842911868,6.158447685,6.532031315,1.690198102,3,1,
868,6.158447685,6.532031315,1.690198102,3,1,14.02298848,18.47534305,136.7821393,59.597701
763.879042,1585.449327,5.842911868,6.158447685,6.532031315,1.690198102,3,1,14.02298848,18
868,6.158447685,6.532031315,1.690198102,3,1,14.02298848,18.47534305,136.7821393,59.597701
911868,6.158447685,6.532031315,1.690198102,3,1,14.02298848,18.47534305,136.7821393,59.597
63.879042,1585.449327,5.842911868,6.158447685,6.532031315,1.690198102,3,1,14.02298848,18.
8,1,0,1,1,1,10,1763.879042,1585.449327,5.842911868,6.158447685,6.532031315,1.690198102,3,1,
3.879042,1585.449327,5.842911868,6.158447685,6.532031315,1.690198102,3,1,14.02298848,18.4
763.879042,1585.449327,5.842911868,6.158447685,6.532031315,1.690198102,3,1,14.02298848,18
911868,6.158447685,6.532031315,1.690198102,3,1,14.02298848,18.47534305,136.7821393,59.597
763.879042,1585.449327,5.842911868,6.158447685,6.532031315,1.690198102,3,1,14.02298848,18
3.879042,1585.449327,5.842911868,6.158447685,6.532031315,1.690198102,3,1,14.02298848,18.4
78,1,0,1,1,1,10,1763.879042,1585.449327,5.842911868,6.158447685,6.532031315,1.690198102,3,
3.879042,1585.449327,5.842911868,6.158447685,6.532031315,1.690198102,3,1,14.02298848,18.4
562563,4.780028828,1.690198102,1.690198102,1,1,12.11589304,33.4602018,101.7980106,51.4925
2,1,1,0,1,1,10,1625.079858,1580.795093,4.240562563,4.780028828,1.690198102,1.690198102,1,1,
25.079858,1580.795093,4.240562563,4.780028828,1.690198102,1.690198102,1,1,12.11589304,33.
563,4.780028828,1.690198102,1.690198102,1,1,12.11589304,33.4602018,101.7980106,51.4925454
5.079858,1580.795093,4.240562563,4.780028828,1.690198102,1.690198102,1,1,12.11589304,33.4
563,4.780028828,1.690198102,1.690198102,1,1,12.11589304,33.4602018,101.7980106,51.4925454
25.079858,1580.795093,4.240562563,4.780028828,1.690198102,1.690198102,1,1,12.11589304,33.
72,1,1,0,1,1,10,1625.079858,1580.795093,4.240562563,4.780028828,1.690198102,1.690198102,1,
1625.079858,1580.795093,4.240562563,4.780028828,1.690198102,1.690198102,1,1,12.11589304,3
563,4.780028828,1.690198102,1.690198102,1,1,12.11589304,33.4602018,101.7980106,51.4925454
562563,4.780028828,1.690198102,1.690198102,1,1,12.11589304,33.4602018,101.7980106,51.4925
625.079858,1580.795093,4.240562563,4.780028828,1.690198102,1.690198102,1,1,12.11589304,33
5.079858,1580.795093,4.240562563,4.780028828,1.690198102,1.690198102,1,1,12.11589304,33.4
5.079858,1580.795093,4.240562563,4.780028828,1.690198102,1.690198102,1,1,12.11589304,33.4
625.079858,1580.795093,4.240562563,4.780028828,1.690198102,1.690198102,1,1,12.11589304,33
5.079858,1580.795093,4.240562563,4.780028828,1.690198102,1.690198102,1,1,12.11589304,33.4
2,1,1,0,1,1,10,1625.079858,1580.795093,4.240562563,4.780028828,1.690198102,1.690198102,1,1,
5.079858,1580.795093,4.240562563,4.780028828,1.690198102,1.690198102,1,1,12.11589304,33.4
72,1,1,0,1,1,10,1625.079858,1580.795093,4.240562563,4.780028828,1.690198102,1.690198102,1,
40562563,4.780028828,1.690198102,1.690198102,1,1,12.11589304,33.4602018,101.7980106,51.49
562563,4.780028828,1.690198102,1.690198102,1,1,12.11589304,33.4602018,101.7980106,51.4925
25.079858,1580.795093,4.240562563,4.780028828,1.690198102,1.690198102,1,1,12.11589304,33.
40562563,4.780028828,1.690198102,1.690198102,1,1,12.11589304,33.4602018,101.7980106,51.49
562563,4.780028828,1.690198102,1.690198102,1,1,12.11589304,33.4602018,101.7980106,51.4925
625.079858,1580.795093,4.240562563,4.780028828,1.690198102,1.690198102,1,1,12.11589304,33

563,4.780028828,1.690198102,1.690198102,1,1,12.11589304,33.4602018,101.7980106,51.4925454
563,4.780028828,1.690198102,1.690198102,1,1,12.11589304,33.4602018,101.7980106,51.4925454
562563,4.780028828,1.690198102,1.690198102,1,1,12.11589304,33.4602018,101.7980106,51.4925
25.079858,1580.795093,4.240562563,4.780028828,1.690198102,1.690198102,1,1,12.11589304,33.
240562563,4.780028828,1.690198102,1.690198102,1,1,12.11589304,33.4602018,101.7980106,51.
5.079858,1580.795093,4.240562563,4.780028828,1.690198102,1.690198102,1,1,12.11589304,33.4
625.079858,1580.795093,4.240562563,4.780028828,1.690198102,1.690198102,1,1,12.11589304,33
562563,4.780028828,1.690198102,1.690198102,1,1,12.11589304,33.4602018,101.7980106,51.4925
625.079858,1580.795093,4.240562563,4.780028828,1.690198102,1.690198102,1,1,12.11589304,33
40562563,4.780028828,1.690198102,1.690198102,1,1,12.11589304,33.4602018,101.7980106,51.49
625.079858,1580.795093,4.240562563,4.780028828,1.690198102,1.690198102,1,1,12.11589304,33
5.079858,1580.795093,4.240562563,4.780028828,1.690198102,1.690198102,1,1,12.11589304,33.4
563,4.780028828,1.690198102,1.690198102,1,1,12.11589304,33.4602018,101.7980106,51.4925454
25.079858,1580.795093,4.240562563,4.780028828,1.690198102,1.690198102,1,1,12.11589304,33.
74,0,1,0,1,1,12,1625.079858,1580.795093,4.240562563,4.780028828,1.690198102,1.690198102,1,
625.079858,1580.795093,4.240562563,4.780028828,1.690198102,1.690198102,1,1,12.11589304,33
625.079858,1580.795093,4.240562563,4.780028828,1.690198102,1.690198102,1,1,12.11589304,33
562563,4.780028828,1.690198102,1.690198102,1,1,12.11589304,33.4602018,101.7980106,51.4925
625.079858,1580.795093,4.240562563,4.780028828,1.690198102,1.690198102,1,1,12.11589304,33
5.079858,1580.795093,4.240562563,4.780028828,1.690198102,1.690198102,1,1,12.11589304,33.4
5.079858,1580.795093,4.240562563,4.780028828,1.690198102,1.690198102,1,1,12.11589304,33.4
40562563,4.780028828,1.690198102,1.690198102,1,1,12.11589304,33.4602018,101.7980106,51.49
5.079858,1580.795093,4.240562563,4.780028828,1.690198102,1.690198102,1,1,12.11589304,33.4
4,0,1,0,1,1,12,1625.079858,1580.795093,4.240562563,4.780028828,1.690198102,1.690198102,1,1,
5.079858,1580.795093,4.240562563,4.780028828,1.690198102,1.690198102,1,1,12.11589304,33.4
74,0,1,0,1,1,12,1625.079858,1580.795093,4.240562563,4.780028828,1.690198102,1.690198102,1,
4,0,1,0,1,1,12,1625.079858,1580.795093,4.240562563,4.780028828,1.690198102,1.690198102,1,1,
563,4.780028828,1.690198102,1.690198102,1,1,12.11589304,33.4602018,101.7980106,51.4925454
562563,4.780028828,1.690198102,1.690198102,1,1,12.11589304,33.4602018,101.7980106,51.4925
25.079858,1580.795093,4.240562563,4.780028828,1.690198102,1.690198102,1,1,12.11589304,33.
74,0,1,0,1,1,12,1625.079858,1580.795093,4.240562563,4.780028828,1.690198102,1.690198102,1,
5.079858,1580.795093,4.240562563,4.780028828,1.690198102,1.690198102,1,1,12.11589304,33.4
625.079858,1580.795093,4.240562563,4.780028828,1.690198102,1.690198102,1,1,12.11589304,33
562563,4.780028828,1.690198102,1.690198102,1,1,12.11589304,33.4602018,101.7980106,51.4925
40562563,4.780028828,1.690198102,1.690198102,1,1,12.11589304,33.4602018,101.7980106,51.49
563,4.780028828,1.690198102,1.690198102,1,1,12.11589304,33.4602018,101.7980106,51.4925454
625.079858,1580.795093,4.240562563,4.780028828,1.690198102,1.690198102,1,1,12.11589304,33
5.079858,1580.795093,4.240562563,4.780028828,1.690198102,1.690198102,1,1,12.11589304,33.4
562563,4.780028828,1.690198102,1.690198102,1,1,12.11589304,33.4602018,101.7980106,51.4925
25.079858,1580.795093,4.240562563,4.780028828,1.690198102,1.690198102,1,1,12.11589304,33.
74,0,1,0,1,1,12,1625.079858,1580.795093,4.240562563,4.780028828,1.690198102,1.690198102,1,
562563,4.780028828,1.690198102,1.690198102,1,1,12.11589304,33.4602018,101.7980106,51.4925
625.079858,1580.795093,4.240562563,4.780028828,1.690198102,1.690198102,1,1,12.11589304,33
562563,4.780028828,1.690198102,1.690198102,1,1,12.11589304,33.4602018,101.7980106,51.4925
4,0,1,0,1,1,12,1625.079858,1580.795093,4.240562563,4.780028828,1.690198102,1.690198102,1,1,
25.079858,1580.795093,4.240562563,4.780028828,1.690198102,1.690198102,1,1,12.11589304,33.
5.079858,1580.795093,4.240562563,4.780028828,1.690198102,1.690198102,1,1,12.11589304,33.4
5.079858,1580.795093,4.240562563,4.780028828,1.690198102,1.690198102,1,1,12.11589304,33.4
3067,4.166740389,1.690198102,1.690198102,1,1,9.818768037,12.50022117,221.2736075,46.63914
559.30899,1705.458039,0.81823067,4.166740389,1.690198102,1.690198102,1,1,9.818768037,12.5

80,0,1,0,1,1,12,1559.30899,1705.458039,0.81823067,4.166740389,1.690198102,1.690198102,1,1,9
9.30899,1705.458039,0.81823067,4.166740389,1.690198102,1.690198102,1,1,9.818768037,12.500
559.30899,1705.458039,0.81823067,4.166740389,1.690198102,1.690198102,1,1,9.818768037,12.5
382324,3,2,212,80,0,1,0,1,1,12,1559.30899,1705.458039,0.81823067,4.166740389,1.690198102,1.6
1823067,4.166740389,1.690198102,1.690198102,1,1,9.818768037,12.50022117,221.2736075,46.63
9.30899,1705.458039,0.81823067,4.166740389,1.690198102,1.690198102,1,1,9.818768037,12.500
559.30899,1705.458039,0.81823067,4.166740389,1.690198102,1.690198102,1,1,9.818768037,12.5
9.30899,1705.458039,0.81823067,4.166740389,1.690198102,1.690198102,1,1,9.818768037,12.500
3067,4.166740389,1.690198102,1.690198102,1,1,9.818768037,12.50022117,221.2736075,46.63914
323067,4.166740389,1.690198102,1.690198102,1,1,9.818768037,12.50022117,221.2736075,46.639
9.30899,1705.458039,0.81823067,4.166740389,1.690198102,1.690198102,1,1,9.818768037,12.500
9.30899,1705.458039,0.81823067,4.166740389,1.690198102,1.690198102,1,1,9.818768037,12.500
559.30899,1705.458039,0.81823067,4.166740389,1.690198102,1.690198102,1,1,9.818768037,12.5
3067,4.166740389,1.690198102,1.690198102,1,1,9.818768037,12.50022117,221.2736075,46.63914
3067,4.166740389,1.690198102,1.690198102,1,1,9.818768037,12.50022117,221.2736075,46.63914
59.30899,1705.458039,0.81823067,4.166740389,1.690198102,1.690198102,1,1,9.818768037,12.50
7,4.166740389,1.690198102,1.690198102,1,1,9.818768037,12.50022117,221.2736075,46.63914818
9.30899,1705.458039,0.81823067,4.166740389,1.690198102,1.690198102,1,1,9.818768037,12.500
7,4.166740389,1.690198102,1.690198102,1,1,9.818768037,12.50022117,221.2736075,46.63914818
59.30899,1705.458039,0.81823067,4.166740389,1.690198102,1.690198102,1,1,9.818768037,12.50
1823067,4.166740389,1.690198102,1.690198102,1,1,9.818768037,12.50022117,221.2736075,46.63
323067,4.166740389,1.690198102,1.690198102,1,1,9.818768037,12.50022117,221.2736075,46.639
7,4.166740389,1.690198102,1.690198102,1,1,9.818768037,12.50022117,221.2736075,46.63914818
3067,4.166740389,1.690198102,1.690198102,1,1,9.818768037,12.50022117,221.2736075,46.63914
559.30899,1705.458039,0.81823067,4.166740389,1.690198102,1.690198102,1,1,9.818768037,12.5
9.30899,1705.458039,0.81823067,4.166740389,1.690198102,1.690198102,1,1,9.818768037,12.500
559.30899,1705.458039,0.81823067,4.166740389,1.690198102,1.690198102,1,1,9.818768037,12.5
80,0,1,0,1,1,12,1559.30899,1705.458039,0.81823067,4.166740389,1.690198102,1.690198102,1,1,9
9.30899,1705.458039,0.81823067,4.166740389,1.690198102,1.690198102,1,1,9.818768037,12.500
0,0,1,0,1,1,12,1559.30899,1705.458039,0.81823067,4.166740389,1.690198102,1.690198102,1,1,9.8
9.30899,1705.458039,0.81823067,4.166740389,1.690198102,1.690198102,1,1,9.818768037,12.500
80,0,1,0,1,1,12,1559.30899,1705.458039,0.81823067,4.166740389,1.690198102,1.690198102,1,1,9
7,4.166740389,1.690198102,1.690198102,1,1,9.818768037,12.50022117,221.2736075,46.63914818
3067,4.166740389,1.690198102,1.690198102,1,1,9.818768037,12.50022117,221.2736075,46.63914
087,17.93030573,15.57149594,5.71399711,6,2,18.52779617,14.34424458,160.9745089,46.3194904
31.418007,2273.983634,9.263898087,17.93030573,15.57149594,5.71399711,6,2,18.52779617,14.3
74,0,1,0,1,1,12,1731.418007,2273.983634,9.263898087,17.93030573,15.57149594,5.71399711,6,2,
1.418007,2273.983634,9.263898087,17.93030573,15.57149594,5.71399711,6,2,18.52779617,14.34
087,17.93030573,15.57149594,5.71399711,6,2,18.52779617,14.34424458,160.9745089,46.3194904
398087,17.93030573,15.57149594,5.71399711,6,2,18.52779617,14.34424458,160.9745089,46.3194
731.418007,2273.983634,9.263898087,17.93030573,15.57149594,5.71399711,6,2,18.52779617,14.
1.418007,2273.983634,9.263898087,17.93030573,15.57149594,5.71399711,6,2,18.52779617,14.34
31.418007,2273.983634,9.263898087,17.93030573,15.57149594,5.71399711,6,2,18.52779617,14.3
74,0,1,0,1,1,12,1731.418007,2273.983634,9.263898087,17.93030573,15.57149594,5.71399711,6,2,
1.418007,2273.983634,9.263898087,17.93030573,15.57149594,5.71399711,6,2,18.52779617,14.34
4,0,1,0,1,1,12,1731.418007,2273.983634,9.263898087,17.93030573,15.57149594,5.71399711,6,2,1
1.418007,2273.983634,9.263898087,17.93030573,15.57149594,5.71399711,6,2,18.52779617,14.34
74,0,1,0,1,1,12,1731.418007,2273.983634,9.263898087,17.93030573,15.57149594,5.71399711,6,2,
4,0,1,0,1,1,12,1731.418007,2273.983634,9.263898087,17.93030573,15.57149594,5.71399711,6,2,1
087,17.93030573,15.57149594,5.71399711,6,2,18.52779617,14.34424458,160.9745089,46.3194904

398087,17.93030573,15.57149594,5.71399711,6,2,18.52779617,14.34424458,160.9745089,46.3194
4,0,1,0,1,1,12,1731.418007,2273.983634,9.263898087,17.93030573,15.57149594,5.71399711,6,2,1
398087,17.93030573,15.57149594,5.71399711,6,2,18.52779617,14.34424458,160.9745089,46.3194
087,17.93030573,15.57149594,5.71399711,6,2,18.52779617,14.34424458,160.9745089,46.3194904
1.418007,2273.983634,9.263898087,17.93030573,15.57149594,5.71399711,6,2,18.52779617,14.34
087,17.93030573,15.57149594,5.71399711,6,2,18.52779617,14.34424458,160.9745089,46.3194904
731.418007,2273.983634,9.263898087,17.93030573,15.57149594,5.71399711,6,2,18.52779617,14.
74,0,1,0,1,1,12,1731.418007,2273.983634,9.263898087,17.93030573,15.57149594,5.71399711,6,2,
1.418007,2273.983634,9.263898087,17.93030573,15.57149594,5.71399711,6,2,18.52779617,14.34
731.418007,2273.983634,9.263898087,17.93030573,15.57149594,5.71399711,6,2,18.52779617,14.
34,9.263898087,17.93030573,15.57149594,5.71399711,6,2,18.52779617,14.34424458,160.9745089
31.418007,2273.983634,9.263898087,17.93030573,15.57149594,5.71399711,6,2,18.52779617,14.3
1.418007,2273.983634,9.263898087,17.93030573,15.57149594,5.71399711,6,2,18.52779617,14.34
731.418007,2273.983634,9.263898087,17.93030573,15.57149594,5.71399711,6,2,18.52779617,14.
1.418007,2273.983634,9.263898087,17.93030573,15.57149594,5.71399711,6,2,18.52779617,14.34
398087,17.93030573,15.57149594,5.71399711,6,2,18.52779617,14.34424458,160.9745089,46.3194
731.418007,2273.983634,9.263898087,17.93030573,15.57149594,5.71399711,6,2,18.52779617,14.
1.418007,2273.983634,9.263898087,17.93030573,15.57149594,5.71399711,6,2,18.52779617,14.34
1.418007,2273.983634,9.263898087,17.93030573,15.57149594,5.71399711,6,2,18.52779617,14.34
731.418007,2273.983634,9.263898087,17.93030573,15.57149594,5.71399711,6,2,18.52779617,14.
,1,0,0,1,1,12,1731.418007,2273.983634,9.263898087,17.93030573,15.57149594,5.71399711,6,2,18
31.418007,2273.983634,9.263898087,17.93030573,15.57149594,5.71399711,6,2,18.52779617,14.3
69,1,0,0,1,1,12,1731.418007,2273.983634,9.263898087,17.93030573,15.57149594,5.71399711,6,2,
731.418007,2273.983634,9.263898087,17.93030573,15.57149594,5.71399711,6,2,18.52779617,14.
263898087,17.93030573,15.57149594,5.71399711,6,2,18.52779617,14.34424458,160.9745089,46.3
53898087,17.93030573,15.57149594,5.71399711,6,2,18.52779617,14.34424458,160.9745089,46.31
263898087,17.93030573,15.57149594,5.71399711,6,2,18.52779617,14.34424458,160.9745089,46.3
1.418007,2273.983634,9.263898087,17.93030573,15.57149594,5.71399711,6,2,18.52779617,14.34
1.418007,2273.983634,9.263898087,17.93030573,15.57149594,5.71399711,6,2,18.52779617,14.34
1.418007,2273.983634,9.263898087,17.93030573,15.57149594,5.71399711,6,2,18.52779617,14.34
398087,17.93030573,15.57149594,5.71399711,6,2,18.52779617,14.34424458,160.9745089,46.3194
1.418007,2273.983634,9.263898087,17.93030573,15.57149594,5.71399711,6,2,18.52779617,14.34
69,1,0,0,1,1,12,1731.418007,2273.983634,9.263898087,17.93030573,15.57149594,5.71399711,6,2,
9,1,0,0,1,1,12,1731.418007,2273.983634,9.263898087,17.93030573,15.57149594,5.71399711,6,2,1
9,1,0,0,1,1,12,1731.418007,2273.983634,9.263898087,17.93030573,15.57149594,5.71399711,6,2,1
31.418007,2273.983634,9.263898087,17.93030573,15.57149594,5.71399711,6,2,18.52779617,14.3
69,1,0,0,1,1,12,1731.418007,2273.983634,9.263898087,17.93030573,15.57149594,5.71399711,6,2,
1.418007,2273.983634,9.263898087,17.93030573,15.57149594,5.71399711,6,2,18.52779617,14.34
731.418007,2273.983634,9.263898087,17.93030573,15.57149594,5.71399711,6,2,18.52779617,14.
398087,17.93030573,15.57149594,5.71399711,6,2,18.52779617,14.34424458,160.9745089,46.3194
53898087,17.93030573,15.57149594,5.71399711,6,2,18.52779617,14.34424458,160.9745089,46.31
087,17.93030573,15.57149594,5.71399711,6,2,18.52779617,14.34424458,160.9745089,46.3194904
731.418007,2273.983634,9.263898087,17.93030573,15.57149594,5.71399711,6,2,18.52779617,14.
1.418007,2273.983634,9.263898087,17.93030573,15.57149594,5.71399711,6,2,18.52779617,14.34
398087,17.93030573,15.57149594,5.71399711,6,2,18.52779617,14.34424458,160.9745089,46.3194
31.418007,2273.983634,9.263898087,17.93030573,15.57149594,5.71399711,6,2,18.52779617,14.3
731.418007,2273.983634,9.263898087,17.93030573,15.57149594,5.71399711,6,2,18.52779617,14.
1.418007,2273.983634,9.263898087,17.93030573,15.57149594,5.71399711,6,2,18.52779617,14.34
731.418007,2273.983634,9.263898087,17.93030573,15.57149594,5.71399711,6,2,18.52779617,14.
398087,17.93030573,15.57149594,5.71399711,6,2,18.52779617,14.34424458,160.9745089,46.3194

9,1,0,0,1,1,12,1731.418007,2273.983634,9.263898087,17.93030573,15.57149594,5.71399711,6,2,1
31.418007,2273.983634,9.263898087,17.93030573,15.57149594,5.71399711,6,2,18.52779617,14.3
1.418007,2273.983634,9.263898087,17.93030573,15.57149594,5.71399711,6,2,18.52779617,14.34
1.418007,2273.983634,9.263898087,17.93030573,15.57149594,5.71399711,6,2,18.52779617,14.34
732591,16.18119566,6.532031315,1.690198102,3,1,17.7976149,16.18119566,232.1353286,57.1397
778.425352,1840.084206,7.493732591,16.18119566,6.532031315,1.690198102,3,1,17.7976149,16.
77,1,0,1,0,1,12,1778.425352,1840.084206,7.493732591,16.18119566,6.532031315,1.690198102,3,
778.425352,1840.084206,7.493732591,16.18119566,6.532031315,1.690198102,3,1,17.7976149,16.
77,1,0,1,0,1,12,1778.425352,1840.084206,7.493732591,16.18119566,6.532031315,1.690198102,3,
8.425352,1840.084206,7.493732591,16.18119566,6.532031315,1.690198102,3,1,17.7976149,16.18
7,1,0,1,0,1,12,1778.425352,1840.084206,7.493732591,16.18119566,6.532031315,1.690198102,3,1,
8.425352,1840.084206,7.493732591,16.18119566,6.532031315,1.690198102,3,1,17.7976149,16.18
8.425352,1840.084206,7.493732591,16.18119566,6.532031315,1.690198102,3,1,17.7976149,16.18
8.425352,1840.084206,7.493732591,16.18119566,6.532031315,1.690198102,3,1,17.7976149,16.18
591,16.18119566,6.532031315,1.690198102,3,1,17.7976149,16.18119566,232.1353286,57.139711,
778.425352,1840.084206,7.493732591,16.18119566,6.532031315,1.690198102,3,1,17.7976149,16.
732591,16.18119566,6.532031315,1.690198102,3,1,17.7976149,16.18119566,232.1353286,57.1397
591,16.18119566,6.532031315,1.690198102,3,1,17.7976149,16.18119566,232.1353286,57.139711,
7,1,0,1,0,1,12,1778.425352,1840.084206,7.493732591,16.18119566,6.532031315,1.690198102,3,1,
732591,16.18119566,6.532031315,1.690198102,3,1,17.7976149,16.18119566,232.1353286,57.1397
78.425352,1840.084206,7.493732591,16.18119566,6.532031315,1.690198102,3,1,17.7976149,16.1
93732591,16.18119566,6.532031315,1.690198102,3,1,17.7976149,16.18119566,232.1353286,57.13
778.425352,1840.084206,7.493732591,16.18119566,6.532031315,1.690198102,3,1,17.7976149,16.
778.425352,1840.084206,7.493732591,16.18119566,6.532031315,1.690198102,3,1,17.7976149,16.
0617676,3,2,212,77,1,0,1,0,1,12,1778.425352,1840.084206,7.493732591,16.18119566,6.532031315
778.425352,1840.084206,7.493732591,16.18119566,6.532031315,1.690198102,3,1,17.7976149,16.
8.425352,1840.084206,7.493732591,16.18119566,6.532031315,1.690198102,3,1,17.7976149,16.18
8.425352,1840.084206,7.493732591,16.18119566,6.532031315,1.690198102,3,1,17.7976149,16.18
7,1,0,1,0,1,12,1778.425352,1840.084206,7.493732591,16.18119566,6.532031315,1.690198102,3,1,
778.425352,1840.084206,7.493732591,16.18119566,6.532031315,1.690198102,3,1,17.7976149,16.
78.425352,1840.084206,7.493732591,16.18119566,6.532031315,1.690198102,3,1,17.7976149,16.1
778.425352,1840.084206,7.493732591,16.18119566,6.532031315,1.690198102,3,1,17.7976149,16.
591,16.18119566,6.532031315,1.690198102,3,1,17.7976149,16.18119566,232.1353286,57.139711,
8.425352,1840.084206,7.493732591,16.18119566,6.532031315,1.690198102,3,1,17.7976149,16.18
591,16.18119566,6.532031315,1.690198102,3,1,17.7976149,16.18119566,232.1353286,57.139711,
93732591,16.18119566,6.532031315,1.690198102,3,1,17.7976149,16.18119566,232.1353286,57.13
493732591,16.18119566,6.532031315,1.690198102,3,1,17.7976149,16.18119566,232.1353286,57.
8.425352,1840.084206,7.493732591,16.18119566,6.532031315,1.690198102,3,1,17.7976149,16.18
591,16.18119566,6.532031315,1.690198102,3,1,17.7976149,16.18119566,232.1353286,57.139711,
9.997799,1810.372266,6.064885082,11.00703395,6.610192116,1.690198102,4,1,15.16221271,38.5
799.997799,1810.372266,6.064885082,11.00703395,6.610192116,1.690198102,4,1,15.16221271,38
9.997799,1810.372266,6.064885082,11.00703395,6.610192116,1.690198102,4,1,15.16221271,38.5
9.997799,1810.372266,6.064885082,11.00703395,6.610192116,1.690198102,4,1,15.16221271,38.5
54885082,11.00703395,6.610192116,1.690198102,4,1,15.16221271,38.52461883,120.917111,53.06
385082,11.00703395,6.610192116,1.690198102,4,1,15.16221271,38.52461883,120.917111,53.0677
9,0,0,0,1,1,12,1799.997799,1810.372266,6.064885082,11.00703395,6.610192116,1.690198102,4,1,
9.997799,1810.372266,6.064885082,11.00703395,6.610192116,1.690198102,4,1,15.16221271,38.5
69,0,0,0,1,1,12,1799.997799,1810.372266,6.064885082,11.00703395,6.610192116,1.690198102,4,
9,0,0,0,1,1,12,1799.997799,1810.372266,6.064885082,11.00703395,6.610192116,1.690198102,4,1,
082,11.00703395,6.610192116,1.690198102,4,1,15.16221271,38.52461883,120.917111,53.0677444

799.997799,1810.372266,6.064885082,11.00703395,6.610192116,1.690198102,4,1,15.16221271,3
69,0,0,0,1,1,12,1799.997799,1810.372266,6.064885082,11.00703395,6.610192116,1.690198102,4,
9.997799,1810.372266,6.064885082,11.00703395,6.610192116,1.690198102,4,1,15.16221271,38.5
799.997799,1810.372266,6.064885082,11.00703395,6.610192116,1.690198102,4,1,15.16221271,3
56,6.064885082,11.00703395,6.610192116,1.690198102,4,1,15.16221271,38.52461883,120.917111
9,0,0,0,1,1,12,1799.997799,1810.372266,6.064885082,11.00703395,6.610192116,1.690198102,4,1,
9.997799,1810.372266,6.064885082,11.00703395,6.610192116,1.690198102,4,1,15.16221271,38.5
799.997799,1810.372266,6.064885082,11.00703395,6.610192116,1.690198102,4,1,15.16221271,3
9.997799,1810.372266,6.064885082,11.00703395,6.610192116,1.690198102,4,1,15.16221271,38.5
385082,11.00703395,6.610192116,1.690198102,4,1,15.16221271,38.52461883,120.917111,53.0677
54885082,11.00703395,6.610192116,1.690198102,4,1,15.16221271,38.52461883,120.917111,53.06
99.997799,1810.372266,6.064885082,11.00703395,6.610192116,1.690198102,4,1,15.16221271,38.
9.997799,1810.372266,6.064885082,11.00703395,6.610192116,1.690198102,4,1,15.16221271,38.5
799.997799,1810.372266,6.064885082,11.00703395,6.610192116,1.690198102,4,1,15.16221271,3
385082,11.00703395,6.610192116,1.690198102,4,1,15.16221271,38.52461883,120.917111,53.0677
9,0,0,0,1,1,12,1799.997799,1810.372266,6.064885082,11.00703395,6.610192116,1.690198102,4,1,
99.997799,1810.372266,6.064885082,11.00703395,6.610192116,1.690198102,4,1,15.16221271,38.
9.997799,1810.372266,6.064885082,11.00703395,6.610192116,1.690198102,4,1,15.16221271,38.5
9.997799,1810.372266,6.064885082,11.00703395,6.610192116,1.690198102,4,1,15.16221271,38.5
082,11.00703395,6.610192116,1.690198102,4,1,15.16221271,38.52461883,120.917111,53.0677444
99.997799,1810.372266,6.064885082,11.00703395,6.610192116,1.690198102,4,1,15.16221271,38.
69,0,0,0,1,1,12,1799.997799,1810.372266,6.064885082,11.00703395,6.610192116,1.690198102,4,
799.997799,1810.372266,6.064885082,11.00703395,6.610192116,1.690198102,4,1,15.16221271,3
799.997799,1810.372266,6.064885082,11.00703395,6.610192116,1.690198102,4,1,15.16221271,3
34,3.236890549,1.690198102,1.690198102,1,1,10.43801951,12.9475622,116.7174749,46.6231538,
35934,3.236890549,1.690198102,1.690198102,1,1,10.43801951,12.9475622,116.7174749,46.62315
35934,3.236890549,1.690198102,1.690198102,1,1,10.43801951,12.9475622,116.7174749,46.62315
34,3.236890549,1.690198102,1.690198102,1,1,10.43801951,12.9475622,116.7174749,46.6231538,
5.387563,1587.706318,1.391735934,3.236890549,1.690198102,1.690198102,1,1,10.43801951,12.9
.387563,1587.706318,1.391735934,3.236890549,1.690198102,1.690198102,1,1,10.43801951,12.94
95.387563,1587.706318,1.391735934,3.236890549,1.690198102,1.690198102,1,1,10.43801951,12.
35934,3.236890549,1.690198102,1.690198102,1,1,10.43801951,12.9475622,116.7174749,46.62315
95.387563,1587.706318,1.391735934,3.236890549,1.690198102,1.690198102,1,1,10.43801951,12.
35934,3.236890549,1.690198102,1.690198102,1,1,10.43801951,12.9475622,116.7174749,46.62315
78,1,0,1,1,1,9,1595.387563,1587.706318,1.391735934,3.236890549,1.690198102,1.690198102,1,1,
.387563,1587.706318,1.391735934,3.236890549,1.690198102,1.690198102,1,1,10.43801951,12.94
.387563,1587.706318,1.391735934,3.236890549,1.690198102,1.690198102,1,1,10.43801951,12.94
5.387563,1587.706318,1.391735934,3.236890549,1.690198102,1.690198102,1,1,10.43801951,12.9
95.387563,1587.706318,1.391735934,3.236890549,1.690198102,1.690198102,1,1,10.43801951,12.
34,3.236890549,1.690198102,1.690198102,1,1,10.43801951,12.9475622,116.7174749,46.6231538,
595.387563,1587.706318,1.391735934,3.236890549,1.690198102,1.690198102,1,1,10.43801951,12
.387563,1587.706318,1.391735934,3.236890549,1.690198102,1.690198102,1,1,10.43801951,12.94
.387563,1587.706318,1.391735934,3.236890549,1.690198102,1.690198102,1,1,10.43801951,12.94
5.387563,1587.706318,1.391735934,3.236890549,1.690198102,1.690198102,1,1,10.43801951,12.9
.387563,1587.706318,1.391735934,3.236890549,1.690198102,1.690198102,1,1,10.43801951,12.94
34,3.236890549,1.690198102,1.690198102,1,1,10.43801951,12.9475622,116.7174749,46.6231538,
35934,3.236890549,1.690198102,1.690198102,1,1,10.43801951,12.9475622,116.7174749,46.62315
1735934,3.236890549,1.690198102,1.690198102,1,1,10.43801951,12.9475622,116.7174749,46.623
91735934,3.236890549,1.690198102,1.690198102,1,1,10.43801951,12.9475622,116.7174749,46.6
95.387563,1587.706318,1.391735934,3.236890549,1.690198102,1.690198102,1,1,10.43801951,12.

78,1,0,1,1,1,9,1595.387563,1587.706318,1.391735934,3.236890549,1.690198102,1.690198102,1,1,
.387563,1587.706318,1.391735934,3.236890549,1.690198102,1.690198102,1,1,10.43801951,12.94
8,1,0,1,1,1,9,1595.387563,1587.706318,1.391735934,3.236890549,1.690198102,1.690198102,1,1,1
.387563,1587.706318,1.391735934,3.236890549,1.690198102,1.690198102,1,1,10.43801951,12.94
.387563,1587.706318,1.391735934,3.236890549,1.690198102,1.690198102,1,1,10.43801951,12.94
.387563,1587.706318,1.391735934,3.236890549,1.690198102,1.690198102,1,1,10.43801951,12.94
34,3.236890549,1.690198102,1.690198102,1,1,10.43801951,12.9475622,116.7174749,46.6231538,5
78,1,0,1,1,1,9,1595.387563,1587.706318,1.391735934,3.236890549,1.690198102,1.690198102,1,1,
8,1,0,1,1,1,9,1595.387563,1587.706318,1.391735934,3.236890549,1.690198102,1.690198102,1,1,1
5,17.71853878,37.1511708,0,8,0,28.51045016,7.087415511,237.379928,95.66839944,81.50527838,
6,0,1,0,1,1,9,11624.47064,9150.85946,10.13704895,17.71853878,37.1511708,0,8,0,28.51045016,7.
6,0,1,0,1,1,9,11624.47064,9150.85946,10.13704895,17.71853878,37.1511708,0,8,0,28.51045016,7.
5,17.71853878,37.1511708,0,8,0,28.51045016,7.087415511,237.379928,95.66839944,81.50527838,
5,17.71853878,37.1511708,0,8,0,28.51045016,7.087415511,237.379928,95.66839944,81.50527838,
4895,17.71853878,37.1511708,0,8,0,28.51045016,7.087415511,237.379928,95.66839944,81.505278
4.47064,9150.85946,10.13704895,17.71853878,37.1511708,0,8,0,28.51045016,7.087415511,237.37
6,0,1,0,1,1,9,11624.47064,9150.85946,10.13704895,17.71853878,37.1511708,0,8,0,28.51045016,7.
4.47064,9150.85946,10.13704895,17.71853878,37.1511708,0,8,0,28.51045016,7.087415511,237.37
24.47064,9150.85946,10.13704895,17.71853878,37.1511708,0,8,0,28.51045016,7.087415511,237.3
24.47064,9150.85946,10.13704895,17.71853878,37.1511708,0,8,0,28.51045016,7.087415511,237.3
24.47064,9150.85946,10.13704895,17.71853878,37.1511708,0,8,0,28.51045016,7.087415511,237.3
5,17.71853878,37.1511708,0,8,0,28.51045016,7.087415511,237.379928,95.66839944,81.50527838,
24.47064,9150.85946,10.13704895,17.71853878,37.1511708,0,8,0,28.51045016,7.087415511,237.3
4895,17.71853878,37.1511708,0,8,0,28.51045016,7.087415511,237.379928,95.66839944,81.505278
895,17.71853878,37.1511708,0,8,0,28.51045016,7.087415511,237.379928,95.66839944,81.5052783
4.47064,9150.85946,10.13704895,17.71853878,37.1511708,0,8,0,28.51045016,7.087415511,237.37
17.71853878,37.1511708,0,8,0,28.51045016,7.087415511,237.379928,95.66839944,81.50527838,3
4.47064,9150.85946,10.13704895,17.71853878,37.1511708,0,8,0,28.51045016,7.087415511,237.37
4.47064,9150.85946,10.13704895,17.71853878,37.1511708,0,8,0,28.51045016,7.087415511,237.37
24.47064,9150.85946,10.13704895,17.71853878,37.1511708,0,8,0,28.51045016,7.087415511,237.3
4895,17.71853878,37.1511708,0,8,0,28.51045016,7.087415511,237.379928,95.66839944,81.505278
66,0,1,0,1,1,9,11624.47064,9150.85946,10.13704895,17.71853878,37.1511708,0,8,0,28.51045016,7
6,0,1,0,1,1,9,11624.47064,9150.85946,10.13704895,17.71853878,37.1511708,0,8,0,28.51045016,7.
6,0,1,0,1,1,9,11624.47064,9150.85946,10.13704895,17.71853878,37.1511708,0,8,0,28.51045016,7.
704895,17.71853878,37.1511708,0,8,0,28.51045016,7.087415511,237.379928,95.66839944,81.505
6,0,1,0,1,1,9,11624.47064,9150.85946,10.13704895,17.71853878,37.1511708,0,8,0,28.51045016,7.
624.47064,9150.85946,10.13704895,17.71853878,37.1511708,0,8,0,28.51045016,7.087415511,237
24.47064,9150.85946,10.13704895,17.71853878,37.1511708,0,8,0,28.51045016,7.087415511,237.3
3704895,17.71853878,37.1511708,0,8,0,28.51045016,7.087415511,237.379928,95.66839944,81.50
66,0,1,0,1,1,9,11624.47064,9150.85946,10.13704895,17.71853878,37.1511708,0,8,0,28.51045016,7
5,17.71853878,37.1511708,0,8,0,28.51045016,7.087415511,237.379928,95.66839944,81.50527838,
24.47064,9150.85946,10.13704895,17.71853878,37.1511708,0,8,0,28.51045016,7.087415511,237.3
24.47064,9150.85946,10.13704895,17.71853878,37.1511708,0,8,0,28.51045016,7.087415511,237.3
17.71853878,37.1511708,0,8,0,28.51045016,7.087415511,237.379928,95.66839944,81.50527838,3
5,17.71853878,37.1511708,0,8,0,28.51045016,7.087415511,237.379928,95.66839944,81.50527838,
624.47064,9150.85946,10.13704895,17.71853878,37.1511708,0,8,0,28.51045016,7.087415511,237
6,0,1,0,1,1,9,11624.47064,9150.85946,10.13704895,17.71853878,37.1511708,0,8,0,28.51045016,7.
4.47064,9150.85946,10.13704895,17.71853878,37.1511708,0,8,0,28.51045016,7.087415511,237.37
35,17.60489667,34.43361432,0,8,0,27.65420446,17.60489667,317.8116776,95.53270632,80.98252
48.26986,8704.323754,10.05607435,17.60489667,34.43361432,0,8,0,27.65420446,17.60489667,31

88,1,1,0,0,1,9,11448.26986,8704.323754,10.05607435,17.60489667,34.43361432,0,8,0,27.6542044
5607435,17.60489667,34.43361432,0,8,0,27.65420446,17.60489667,317.8116776,95.53270632,80.
8.26986,8704.323754,10.05607435,17.60489667,34.43361432,0,8,0,27.65420446,17.60489667,317
5,17.60489667,34.43361432,0,8,0,27.65420446,17.60489667,317.8116776,95.53270632,80.982524
8.26986,8704.323754,10.05607435,17.60489667,34.43361432,0,8,0,27.65420446,17.60489667,317
48.26986,8704.323754,10.05607435,17.60489667,34.43361432,0,8,0,27.65420446,17.60489667,31
35,17.60489667,34.43361432,0,8,0,27.65420446,17.60489667,317.8116776,95.53270632,80.98252
35,17.60489667,34.43361432,0,8,0,27.65420446,17.60489667,317.8116776,95.53270632,80.98252
07435,17.60489667,34.43361432,0,8,0,27.65420446,17.60489667,317.8116776,95.53270632,80.98
8.26986,8704.323754,10.05607435,17.60489667,34.43361432,0,8,0,27.65420446,17.60489667,317
8.26986,8704.323754,10.05607435,17.60489667,34.43361432,0,8,0,27.65420446,17.60489667,317
07435,17.60489667,34.43361432,0,8,0,27.65420446,17.60489667,317.8116776,95.53270632,80.98
,88,1,1,0,0,1,9,11448.26986,8704.323754,10.05607435,17.60489667,34.43361432,0,8,0,27.654204
0.05607435,17.60489667,34.43361432,0,8,0,27.65420446,17.60489667,317.8116776,95.53270632,
8,1,1,0,0,1,9,11448.26986,8704.323754,10.05607435,17.60489667,34.43361432,0,8,0,27.65420446
35,17.60489667,34.43361432,0,8,0,27.65420446,17.60489667,317.8116776,95.53270632,80.98252
35,17.60489667,34.43361432,0,8,0,27.65420446,17.60489667,317.8116776,95.53270632,80.98252
8.26986,8704.323754,10.05607435,17.60489667,34.43361432,0,8,0,27.65420446,17.60489667,317
07435,17.60489667,34.43361432,0,8,0,27.65420446,17.60489667,317.8116776,95.53270632,80.98
5607435,17.60489667,34.43361432,0,8,0,27.65420446,17.60489667,317.8116776,95.53270632,80.
8,1,1,0,0,1,9,11448.26986,8704.323754,10.05607435,17.60489667,34.43361432,0,8,0,27.65420446
48.26986,8704.323754,10.05607435,17.60489667,34.43361432,0,8,0,27.65420446,17.60489667,31
448.26986,8704.323754,10.05607435,17.60489667,34.43361432,0,8,0,27.65420446,17.60489667,3
35,17.60489667,34.43361432,0,8,0,27.65420446,17.60489667,317.8116776,95.53270632,80.98252
48.26986,8704.323754,10.05607435,17.60489667,34.43361432,0,8,0,27.65420446,17.60489667,31
07435,17.60489667,34.43361432,0,8,0,27.65420446,17.60489667,317.8116776,95.53270632,80.98
448.26986,8704.323754,10.05607435,17.60489667,34.43361432,0,8,0,27.65420446,17.60489667,3
8.26986,8704.323754,10.05607435,17.60489667,34.43361432,0,8,0,27.65420446,17.60489667,317
5,17.60489667,34.43361432,0,8,0,27.65420446,17.60489667,317.8116776,95.53270632,80.982524
448.26986,8704.323754,10.05607435,17.60489667,34.43361432,0,8,0,27.65420446,17.60489667,3
8.26986,8704.323754,10.05607435,17.60489667,34.43361432,0,8,0,27.65420446,17.60489667,317
448.26986,8704.323754,10.05607435,17.60489667,34.43361432,0,8,0,27.65420446,17.60489667,3
448.26986,8704.323754,10.05607435,17.60489667,34.43361432,0,8,0,27.65420446,17.60489667,3
8.26986,8704.323754,10.05607435,17.60489667,34.43361432,0,8,0,27.65420446,17.60489667,317
448.26986,8704.323754,10.05607435,17.60489667,34.43361432,0,8,0,27.65420446,17.60489667,3
448.26986,8704.323754,10.05607435,17.60489667,34.43361432,0,8,0,27.65420446,17.60489667,3
0617676,3,2,206,84,1,0,1,0,1,9,11448.26986,8704.323754,10.05607435,17.60489667,34.43361432,
7629395,3,2,206,84,1,0,1,0,1,9,11448.26986,8704.323754,10.05607435,17.60489667,34.43361432,
84,1,0,1,0,1,9,11448.26986,8704.323754,10.05607435,17.60489667,34.43361432,0,8,0,27.6542044
5607435,17.60489667,34.43361432,0,8,0,27.65420446,17.60489667,317.8116776,95.53270632,80.
11448.26986,8704.323754,10.05607435,17.60489667,34.43361432,0,8,0,27.65420446,17.60489667
5607435,17.60489667,34.43361432,0,8,0,27.65420446,17.60489667,317.8116776,95.53270632,80.
11448.26986,8704.323754,10.05607435,17.60489667,34.43361432,0,8,0,27.65420446,17.60489667
8.26986,8704.323754,10.05607435,17.60489667,34.43361432,0,8,0,27.65420446,17.60489667,317
8.26986,8704.323754,10.05607435,17.60489667,34.43361432,0,8,0,27.65420446,17.60489667,317
7.60489667,34.43361432,0,8,0,27.65420446,17.60489667,317.8116776,95.53270632,80.9825247,3
5,17.60489667,34.43361432,0,8,0,27.65420446,17.60489667,317.8116776,95.53270632,80.982524
8.26986,8704.323754,10.05607435,17.60489667,34.43361432,0,8,0,27.65420446,17.60489667,317
5607435,17.60489667,34.43361432,0,8,0,27.65420446,17.60489667,317.8116776,95.53270632,80.
8.26986,8704.323754,10.05607435,17.60489667,34.43361432,0,8,0,27.65420446,17.60489667,317

5,17.60489667,34.43361432,0,8,0,27.65420446,17.60489667,317.8116776,95.53270632,80.9825247
35,17.60489667,34.43361432,0,8,0,27.65420446,17.60489667,317.8116776,95.53270632,80.982524
8.26986,8704.323754,10.05607435,17.60489667,34.43361432,0,8,0,27.65420446,17.60489667,317
8.26986,8704.323754,10.05607435,17.60489667,34.43361432,0,8,0,27.65420446,17.60489667,317
84,1,0,1,0,1,9,11448.26986,8704.323754,10.05607435,17.60489667,34.43361432,0,8,0,27.6542044
8.71557,8725.475982,8.785242555,15.28883562,18.095824,0.838306773,6,1,29.28414185,0,414.5
8.71557,8725.475982,8.785242555,15.28883562,18.095824,0.838306773,6,1,29.28414185,0,414.5
3,1,1,0,1,1,8,10408.71557,8725.475982,8.785242555,15.28883562,18.095824,0.838306773,6,1,29.2
55,15.28883562,18.095824,0.838306773,6,1,29.28414185,0,414.5518898,89.31663264,64.9775513
8.71557,8725.475982,8.785242555,15.28883562,18.095824,0.838306773,6,1,29.28414185,0,414.5
42555,15.28883562,18.095824,0.838306773,6,1,29.28414185,0,414.5518898,89.31663264,64.9775
8.71557,8725.475982,8.785242555,15.28883562,18.095824,0.838306773,6,1,29.28414185,0,414.5
0408.71557,8725.475982,8.785242555,15.28883562,18.095824,0.838306773,6,1,29.28414185,0,41
55,15.28883562,18.095824,0.838306773,6,1,29.28414185,0,414.5518898,89.31663264,64.9775513
2,8.785242555,15.28883562,18.095824,0.838306773,6,1,29.28414185,0,414.5518898,89.31663264,
8.71557,8725.475982,8.785242555,15.28883562,18.095824,0.838306773,6,1,29.28414185,0,414.5
8.71557,8725.475982,8.785242555,15.28883562,18.095824,0.838306773,6,1,29.28414185,0,414.5
8.71557,8725.475982,8.785242555,15.28883562,18.095824,0.838306773,6,1,29.28414185,0,414.5
408.71557,8725.475982,8.785242555,15.28883562,18.095824,0.838306773,6,1,29.28414185,0,414
42555,15.28883562,18.095824,0.838306773,6,1,29.28414185,0,414.5518898,89.31663264,64.9775
8.71557,8725.475982,8.785242555,15.28883562,18.095824,0.838306773,6,1,29.28414185,0,414.5
8.71557,8725.475982,8.785242555,15.28883562,18.095824,0.838306773,6,1,29.28414185,0,414.5
55,15.28883562,18.095824,0.838306773,6,1,29.28414185,0,414.5518898,89.31663264,64.9775513
8.71557,8725.475982,8.785242555,15.28883562,18.095824,0.838306773,6,1,29.28414185,0,414.5
435,17.60489667,34.43361432,0,8,0,27.65420446,17.60489667,317.8116776,95.53270632,80.9825
1448.26986,8704.323754,10.05607435,17.60489667,34.43361432,0,8,0,27.65420446,17.60489667,
448.26986,8704.323754,10.05607435,17.60489667,34.43361432,0,8,0,27.65420446,17.60489667,3
48.26986,8704.323754,10.05607435,17.60489667,34.43361432,0,8,0,27.65420446,17.60489667,31
07435,17.60489667,34.43361432,0,8,0,27.65420446,17.60489667,317.8116776,95.53270632,80.98
48.26986,8704.323754,10.05607435,17.60489667,34.43361432,0,8,0,27.65420446,17.60489667,31
448.26986,8704.323754,10.05607435,17.60489667,34.43361432,0,8,0,27.65420446,17.60489667,3
448.26986,8704.323754,10.05607435,17.60489667,34.43361432,0,8,0,27.65420446,17.60489667,3
07435,17.60489667,34.43361432,0,8,0,27.65420446,17.60489667,317.8116776,95.53270632,80.98
07435,17.60489667,34.43361432,0,8,0,27.65420446,17.60489667,317.8116776,95.53270632,80.98
48.26986,8704.323754,10.05607435,17.60489667,34.43361432,0,8,0,27.65420446,17.60489667,31
435,17.60489667,34.43361432,0,8,0,27.65420446,17.60489667,317.8116776,95.53270632,80.9825
48.26986,8704.323754,10.05607435,17.60489667,34.43361432,0,8,0,27.65420446,17.60489667,31
5607435,17.60489667,34.43361432,0,8,0,27.65420446,17.60489667,317.8116776,95.53270632,80.
35,17.60489667,34.43361432,0,8,0,27.65420446,17.60489667,317.8116776,95.53270632,80.982524
,0,0,0,0,1,11,11448.26986,8704.323754,10.05607435,17.60489667,34.43361432,0,8,0,27.65420446
48.26986,8704.323754,10.05607435,17.60489667,34.43361432,0,8,0,27.65420446,17.60489667,31
48.26986,8704.323754,10.05607435,17.60489667,34.43361432,0,8,0,27.65420446,17.60489667,31
05607435,17.60489667,34.43361432,0,8,0,27.65420446,17.60489667,317.8116776,95.53270632,8
607435,17.60489667,34.43361432,0,8,0,27.65420446,17.60489667,317.8116776,95.53270632,80.9
0617676,3,2,206,73,0,0,0,0,1,11,11448.26986,8704.323754,10.05607435,17.60489667,34.43361432
11448.26986,8704.323754,10.05607435,17.60489667,34.43361432,0,8,0,27.65420446,17.60489667
48.26986,8704.323754,10.05607435,17.60489667,34.43361432,0,8,0,27.65420446,17.60489667,31
35,17.60489667,34.43361432,0,8,0,27.65420446,17.60489667,317.8116776,95.53270632,80.982524
5,17.60489667,34.43361432,0,8,0,27.65420446,17.60489667,317.8116776,95.53270632,80.9825247
73,0,0,0,0,1,11,11448.26986,8704.323754,10.05607435,17.60489667,34.43361432,0,8,0,27.654204

1448.26986,8704.323754,10.05607435,17.60489667,34.43361432,0,8,0,27.65420446,17.60489667,
448.26986,8704.323754,10.05607435,17.60489667,34.43361432,0,8,0,27.65420446,17.60489667,3
48.26986,8704.323754,10.05607435,17.60489667,34.43361432,0,8,0,27.65420446,17.60489667,31
435,17.60489667,34.43361432,0,8,0,27.65420446,17.60489667,317.8116776,95.53270632,80.9825
435,17.60489667,34.43361432,0,8,0,27.65420446,17.60489667,317.8116776,95.53270632,80.9825
48.26986,8704.323754,10.05607435,17.60489667,34.43361432,0,8,0,27.65420446,17.60489667,31
435,17.60489667,34.43361432,0,8,0,27.65420446,17.60489667,317.8116776,95.53270632,80.9825
48.26986,8704.323754,10.05607435,17.60489667,34.43361432,0,8,0,27.65420446,17.60489667,31
,18.29803756,32.83297439,0,7,0,28.0439024,7.319215024,213.5200422,98.15365838,87.8305802
744.4059,9715.281987,9.560421271,18.29803756,32.83297439,0,7,0,28.0439024,7.319215024,213
1744.4059,9715.281987,9.560421271,18.29803756,32.83297439,0,7,0,28.0439024,7.319215024,21
44.4059,9715.281987,9.560421271,18.29803756,32.83297439,0,7,0,28.0439024,7.319215024,213.
1744.4059,9715.281987,9.560421271,18.29803756,32.83297439,0,7,0,28.0439024,7.319215024,21
,18.29803756,32.83297439,0,7,0,28.0439024,7.319215024,213.5200422,98.15365838,87.8305802
1744.4059,9715.281987,9.560421271,18.29803756,32.83297439,0,7,0,28.0439024,7.319215024,21
44.4059,9715.281987,9.560421271,18.29803756,32.83297439,0,7,0,28.0439024,7.319215024,213.
73,1,1,1,0,1,10,11744.4059,9715.281987,9.560421271,18.29803756,32.83297439,0,7,0,28.0439024
3,1,1,1,0,1,10,11744.4059,9715.281987,9.560421271,18.29803756,32.83297439,0,7,0,28.0439024,
,18.29803756,32.83297439,0,7,0,28.0439024,7.319215024,213.5200422,98.15365838,87.8305802
71,18.29803756,32.83297439,0,7,0,28.0439024,7.319215024,213.5200422,98.15365838,87.830580
44.4059,9715.281987,9.560421271,18.29803756,32.83297439,0,7,0,28.0439024,7.319215024,213.
44.4059,9715.281987,9.560421271,18.29803756,32.83297439,0,7,0,28.0439024,7.319215024,213.
60421271,18.29803756,32.83297439,0,7,0,28.0439024,7.319215024,213.5200422,98.15365838,87.
71,18.29803756,32.83297439,0,7,0,28.0439024,7.319215024,213.5200422,98.15365838,87.830580
5275121,21.78870481,18.095824,0.838306773,6,1,28.38093118,14.5258032,319.0755373,92.88304
85275121,21.78870481,18.095824,0.838306773,6,1,28.38093118,14.5258032,319.0755373,92.883
21,21.78870481,18.095824,0.838306773,6,1,28.38093118,14.5258032,319.0755373,92.8830475,76.
0.38103,8849.234909,8.385275121,21.78870481,18.095824,0.838306773,6,1,28.38093118,14.5258
,1,0,1,0,1,8,11130.38103,8849.234909,8.385275121,21.78870481,18.095824,0.838306773,6,1,28.3
75121,21.78870481,18.095824,0.838306773,6,1,28.38093118,14.5258032,319.0755373,92.8830475
130.38103,8849.234909,8.385275121,21.78870481,18.095824,0.838306773,6,1,28.38093118,14.52
130.38103,8849.234909,8.385275121,21.78870481,18.095824,0.838306773,6,1,28.38093118,14.52
0.38103,8849.234909,8.385275121,21.78870481,18.095824,0.838306773,6,1,28.38093118,14.5258
75121,21.78870481,18.095824,0.838306773,6,1,28.38093118,14.5258032,319.0755373,92.8830475
,21.78870481,18.095824,0.838306773,6,1,28.38093118,14.5258032,319.0755373,92.8830475,76.2
30.38103,8849.234909,8.385275121,21.78870481,18.095824,0.838306773,6,1,28.38093118,14.525
90,1,0,1,0,1,8,11130.38103,8849.234909,8.385275121,21.78870481,18.095824,0.838306773,6,1,28
85275121,21.78870481,18.095824,0.838306773,6,1,28.38093118,14.5258032,319.0755373,92.883
0.38103,8849.234909,8.385275121,21.78870481,18.095824,0.838306773,6,1,28.38093118,14.5258
.385275121,21.78870481,18.095824,0.838306773,6,1,28.38093118,14.5258032,319.0755373,92.88
0.38103,8849.234909,8.385275121,21.78870481,18.095824,0.838306773,6,1,28.38093118,14.5258
21,21.78870481,18.095824,0.838306773,6,1,28.38093118,14.5258032,319.0755373,92.8830475,76.
0.38103,8849.234909,8.385275121,21.78870481,18.095824,0.838306773,6,1,28.38093118,14.5258
75121,21.78870481,18.095824,0.838306773,6,1,28.38093118,14.5258032,319.0755373,92.8830475
0.38103,8849.234909,8.385275121,21.78870481,18.095824,0.838306773,6,1,28.38093118,14.5258
0.38103,8849.234909,8.385275121,21.78870481,18.095824,0.838306773,6,1,28.38093118,14.5258
0.38103,8849.234909,8.385275121,21.78870481,18.095824,0.838306773,6,1,28.38093118,14.5258
9382324,3,2,206,90,1,0,1,0,1,8,11130.38103,8849.234909,8.385275121,21.78870481,18.095824,0.8
517676,3,2,206,90,1,0,1,0,1,8,11130.38103,8849.234909,8.385275121,21.78870481,18.095824,0.83
30.38103,8849.234909,8.385275121,21.78870481,18.095824,0.838306773,6,1,28.38093118,14.525

0.38103,8849.234909,8.385275121,21.78870481,18.095824,0.838306773,6,1,28.38093118,14.5258
21,21.78870481,18.095824,0.838306773,6,1,28.38093118,14.5258032,319.0755373,92.8830475,76.
0.38103,8849.234909,8.385275121,21.78870481,18.095824,0.838306773,6,1,28.38093118,14.5258
38176,18.4733174,33.88062318,0,7,0,29.87642628,14.77865392,307.648664,94.27672292,81.2825
356.30923,9081.389159,9.294888176,18.4733174,33.88062318,0,7,0,29.87642628,14.77865392,30
356.30923,9081.389159,9.294888176,18.4733174,33.88062318,0,7,0,29.87642628,14.77865392,30
356.30923,9081.389159,9.294888176,18.4733174,33.88062318,0,7,0,29.87642628,14.77865392,30
356.30923,9081.389159,9.294888176,18.4733174,33.88062318,0,7,0,29.87642628,14.77865392,30
38176,18.4733174,33.88062318,0,7,0,29.87642628,14.77865392,307.648664,94.27672292,81.2825
6,0,1,0,1,1,12,11356.30923,9081.389159,9.294888176,18.4733174,33.88062318,0,7,0,29.87642628
6,0,1,0,1,1,12,11356.30923,9081.389159,9.294888176,18.4733174,33.88062318,0,7,0,29.87642628
94888176,18.4733174,33.88062318,0,7,0,29.87642628,14.77865392,307.648664,94.27672292,81.28
356.30923,9081.389159,9.294888176,18.4733174,33.88062318,0,7,0,29.87642628,14.77865392,30
176,18.4733174,33.88062318,0,7,0,29.87642628,14.77865392,307.648664,94.27672292,81.282596
176,18.4733174,33.88062318,0,7,0,29.87642628,14.77865392,307.648664,94.27672292,81.282596
56.30923,9081.389159,9.294888176,18.4733174,33.88062318,0,7,0,29.87642628,14.77865392,307
176,18.4733174,33.88062318,0,7,0,29.87642628,14.77865392,307.648664,94.27672292,81.282596
38176,18.4733174,33.88062318,0,7,0,29.87642628,14.77865392,307.648664,94.27672292,81.2825
356.30923,9081.389159,9.294888176,18.4733174,33.88062318,0,7,0,29.87642628,14.77865392,30
6,0,1,0,1,1,12,11356.30923,9081.389159,9.294888176,18.4733174,33.88062318,0,7,0,29.87642628
11356.30923,9081.389159,9.294888176,18.4733174,33.88062318,0,7,0,29.87642628,14.77865392,3
11356.30923,9081.389159,9.294888176,18.4733174,33.88062318,0,7,0,29.87642628,14.77865392,3
382324,3,2,206,66,0,1,0,1,1,12,11356.30923,9081.389159,9.294888176,18.4733174,33.88062318,0,
6,0,1,0,1,1,12,11356.30923,9081.389159,9.294888176,18.4733174,33.88062318,0,7,0,29.87642628
56.30923,9081.389159,9.294888176,18.4733174,33.88062318,0,7,0,29.87642628,14.77865392,307
356.30923,9081.389159,9.294888176,18.4733174,33.88062318,0,7,0,29.87642628,14.77865392,30
356.30923,9081.389159,9.294888176,18.4733174,33.88062318,0,7,0,29.87642628,14.77865392,30
388176,18.4733174,33.88062318,0,7,0,29.87642628,14.77865392,307.648664,94.27672292,81.282
294888176,18.4733174,33.88062318,0,7,0,29.87642628,14.77865392,307.648664,94.27672292,81.
1356.30923,9081.389159,9.294888176,18.4733174,33.88062318,0,7,0,29.87642628,14.77865392,30
356.30923,9081.389159,9.294888176,18.4733174,33.88062318,0,7,0,29.87642628,14.77865392,30
356.30923,9081.389159,9.294888176,18.4733174,33.88062318,0,7,0,29.87642628,14.77865392,30
8.57331359,34.26006023,0,8,0,31.91350515,14.85865088,269.0177205,97.77754768,74.29325438,
5,1,1,0,0,1,9,11573.04335,9045.961934,10.18516122,18.57331359,34.26006023,0,8,0,31.91350515
5,1,1,0,0,1,9,11573.04335,9045.961934,10.18516122,18.57331359,34.26006023,0,8,0,31.91350515
573.04335,9045.961934,10.18516122,18.57331359,34.26006023,0,8,0,31.91350515,14.85865088,2
73.04335,9045.961934,10.18516122,18.57331359,34.26006023,0,8,0,31.91350515,14.85865088,26
8.57331359,34.26006023,0,8,0,31.91350515,14.85865088,269.0177205,97.77754768,74.29325438,
18516122,18.57331359,34.26006023,0,8,0,31.91350515,14.85865088,269.0177205,97.77754768,74
5,1,1,0,0,1,9,11573.04335,9045.961934,10.18516122,18.57331359,34.26006023,0,8,0,31.91350515
73.04335,9045.961934,10.18516122,18.57331359,34.26006023,0,8,0,31.91350515,14.85865088,26
3.04335,9045.961934,10.18516122,18.57331359,34.26006023,0,8,0,31.91350515,14.85865088,269
22,18.57331359,34.26006023,0,8,0,31.91350515,14.85865088,269.0177205,97.77754768,74.29325
73.04335,9045.961934,10.18516122,18.57331359,34.26006023,0,8,0,31.91350515,14.85865088,26
65,1,1,0,0,1,9,11573.04335,9045.961934,10.18516122,18.57331359,34.26006023,0,8,0,31.9135051
1573.04335,9045.961934,10.18516122,18.57331359,34.26006023,0,8,0,31.91350515,14.85865088,2
3516122,18.57331359,34.26006023,0,8,0,31.91350515,14.85865088,269.0177205,97.77754768,74.2
3516122,18.57331359,34.26006023,0,8,0,31.91350515,14.85865088,269.0177205,97.77754768,74.2
16122,18.57331359,34.26006023,0,8,0,31.91350515,14.85865088,269.0177205,97.77754768,74.29
16122,18.57331359,34.26006023,0,8,0,31.91350515,14.85865088,269.0177205,97.77754768,74.29

73.04335,9045.961934,10.18516122,18.57331359,34.26006023,0,8,0,31.91350515,14.85865088,26
22,18.57331359,34.26006023,0,8,0,31.91350515,14.85865088,269.0177205,97.77754768,74.293254
2,18.57331359,34.26006023,0,8,0,31.91350515,14.85865088,269.0177205,97.77754768,74.2932543
73.04335,9045.961934,10.18516122,18.57331359,34.26006023,0,8,0,31.91350515,14.85865088,26
5,1,1,0,0,1,9,11573.04335,9045.961934,10.18516122,18.57331359,34.26006023,0,8,0,31.91350515
5,1,1,0,0,1,9,11573.04335,9045.961934,10.18516122,18.57331359,34.26006023,0,8,0,31.91350515
73.04335,9045.961934,10.18516122,18.57331359,34.26006023,0,8,0,31.91350515,14.85865088,26
8.57331359,34.26006023,0,8,0,31.91350515,14.85865088,269.0177205,97.77754768,74.29325438,
73.04335,9045.961934,10.18516122,18.57331359,34.26006023,0,8,0,31.91350515,14.85865088,26
22,18.57331359,34.26006023,0,8,0,31.91350515,14.85865088,269.0177205,97.77754768,74.293254
8516122,18.57331359,34.26006023,0,8,0,31.91350515,14.85865088,269.0177205,97.77754768,74.2
73.04335,9045.961934,10.18516122,18.57331359,34.26006023,0,8,0,31.91350515,14.85865088,26
7,10.62623746,18.14838725,0.838306773,7,1,29.47630351,7.084158305,222.3913544,91.53167933
966.128766,7792.199074,10.85969077,10.62623746,18.14838725,0.838306773,7,1,29.47630351,7.0
.128766,7792.199074,10.85969077,10.62623746,18.14838725,0.838306773,7,1,29.47630351,7.084
.128766,7792.199074,10.85969077,10.62623746,18.14838725,0.838306773,7,1,29.47630351,7.084
.128766,7792.199074,10.85969077,10.62623746,18.14838725,0.838306773,7,1,29.47630351,7.084
7,10.62623746,18.14838725,0.838306773,7,1,29.47630351,7.084158305,222.3913544,91.53167933
.128766,7792.199074,10.85969077,10.62623746,18.14838725,0.838306773,7,1,29.47630351,7.084
66.128766,7792.199074,10.85969077,10.62623746,18.14838725,0.838306773,7,1,29.47630351,7.08
5969077,10.62623746,18.14838725,0.838306773,7,1,29.47630351,7.084158305,222.3913544,91.53
77,10.62623746,18.14838725,0.838306773,7,1,29.47630351,7.084158305,222.3913544,91.5316793
7,10.62623746,18.14838725,0.838306773,7,1,29.47630351,7.084158305,222.3913544,91.53167933
.128766,7792.199074,10.85969077,10.62623746,18.14838725,0.838306773,7,1,29.47630351,7.084
.128766,7792.199074,10.85969077,10.62623746,18.14838725,0.838306773,7,1,29.47630351,7.084
77,10.62623746,18.14838725,0.838306773,7,1,29.47630351,7.084158305,222.3913544,91.5316793
85969077,10.62623746,18.14838725,0.838306773,7,1,29.47630351,7.084158305,222.3913544,91.5
7,10.62623746,18.14838725,0.838306773,7,1,29.47630351,7.084158305,222.3913544,91.53167933
2370605,3,2,206,79,1,1,0,0,1,8,9966.128766,7792.199074,10.85969077,10.62623746,18.14838725,0
77,10.62623746,18.14838725,0.838306773,7,1,29.47630351,7.084158305,222.3913544,91.5316793
9,1,1,0,0,1,8,9966.128766,7792.199074,10.85969077,10.62623746,18.14838725,0.838306773,7,1,2
7629395,3,2,206,79,1,1,0,0,1,8,9966.128766,7792.199074,10.85969077,10.62623746,18.14838725,0
66.128766,7792.199074,10.85969077,10.62623746,18.14838725,0.838306773,7,1,29.47630351,7.0
966.128766,7792.199074,10.85969077,10.62623746,18.14838725,0.838306773,7,1,29.47630351,7.0
79,1,1,0,0,1,8,9966.128766,7792.199074,10.85969077,10.62623746,18.14838725,0.838306773,7,1,
.128766,7792.199074,10.85969077,10.62623746,18.14838725,0.838306773,7,1,29.47630351,7.084
77,10.62623746,18.14838725,0.838306773,7,1,29.47630351,7.084158305,222.3913544,91.5316793
66.128766,7792.199074,10.85969077,10.62623746,18.14838725,0.838306773,7,1,29.47630351,7.0
77,10.62623746,18.14838725,0.838306773,7,1,29.47630351,7.084158305,222.3913544,91.5316793
97,3.507796988,18.14838725,0.838306773,7,1,30.58659135,7.015593976,203.8917857,90.9951092
872.508308,7702.139997,10.70530697,3.507796988,18.14838725,0.838306773,7,1,30.58659135,7.
72.508308,7702.139997,10.70530697,3.507796988,18.14838725,0.838306773,7,1,30.58659135,7.0
70,1,1,1,0,1,10,9872.508308,7702.139997,10.70530697,3.507796988,18.14838725,0.838306773,7,1
70530697,3.507796988,18.14838725,0.838306773,7,1,30.58659135,7.015593976,203.8917857,90.9
872.508308,7702.139997,10.70530697,3.507796988,18.14838725,0.838306773,7,1,30.58659135,7.
0,1,1,1,0,1,10,9872.508308,7702.139997,10.70530697,3.507796988,18.14838725,0.838306773,7,1,
72.508308,7702.139997,10.70530697,3.507796988,18.14838725,0.838306773,7,1,30.58659135,7.0
72.508308,7702.139997,10.70530697,3.507796988,18.14838725,0.838306773,7,1,30.58659135,7.0
70,1,1,1,0,1,10,9872.508308,7702.139997,10.70530697,3.507796988,18.14838725,0.838306773,7,1
10.70530697,3.507796988,18.14838725,0.838306773,7,1,30.58659135,7.015593976,203.8917857,9

2.508308,7702.139997,10.70530697,3.507796988,18.14838725,0.838306773,7,1,30.58659135,7.01
0,1,1,1,0,1,10,9872.508308,7702.139997,10.70530697,3.507796988,18.14838725,0.838306773,7,1,
0,1,1,1,0,1,10,9872.508308,7702.139997,10.70530697,3.507796988,18.14838725,0.838306773,7,1,
2.508308,7702.139997,10.70530697,3.507796988,18.14838725,0.838306773,7,1,30.58659135,7.01
2.508308,7702.139997,10.70530697,3.507796988,18.14838725,0.838306773,7,1,30.58659135,7.01
70,1,1,1,0,1,10,9872.508308,7702.139997,10.70530697,3.507796988,18.14838725,0.838306773,7,1
70,1,1,1,0,1,10,9872.508308,7702.139997,10.70530697,3.507796988,18.14838725,0.838306773,7,1
2.508308,7702.139997,10.70530697,3.507796988,18.14838725,0.838306773,7,1,30.58659135,7.01
872.508308,7702.139997,10.70530697,3.507796988,18.14838725,0.838306773,7,1,30.58659135,7.
530697,3.507796988,18.14838725,0.838306773,7,1,30.58659135,7.015593976,203.8917857,90.995
0.70530697,3.507796988,18.14838725,0.838306773,7,1,30.58659135,7.015593976,203.8917857,90
72.508308,7702.139997,10.70530697,3.507796988,18.14838725,0.838306773,7,1,30.58659135,7.0
2.508308,7702.139997,10.70530697,3.507796988,18.14838725,0.838306773,7,1,30.58659135,7.01
597,3.507796988,18.14838725,0.838306773,7,1,30.58659135,7.015593976,203.8917857,90.995109
0,1,1,1,0,1,10,9872.508308,7702.139997,10.70530697,3.507796988,18.14838725,0.838306773,7,1,
,10.70530697,3.507796988,18.14838725,0.838306773,7,1,30.58659135,7.015593976,203.8917857,
2.508308,7702.139997,10.70530697,3.507796988,18.14838725,0.838306773,7,1,30.58659135,7.01
0,1,1,1,0,1,10,9872.508308,7702.139997,10.70530697,3.507796988,18.14838725,0.838306773,7,1,
9872.508308,7702.139997,10.70530697,3.507796988,18.14838725,0.838306773,7,1,30.58659135,7
72.508308,7702.139997,10.70530697,3.507796988,18.14838725,0.838306773,7,1,30.58659135,7.0
597,3.507796988,18.14838725,0.838306773,7,1,30.58659135,7.015593976,203.8917857,90.995109
597,3.507796988,18.14838725,0.838306773,7,1,30.58659135,7.015593976,203.8917857,90.995109
72.508308,7702.139997,10.70530697,3.507796988,18.14838725,0.838306773,7,1,30.58659135,7.0
597,3.507796988,18.14838725,0.838306773,7,1,30.58659135,7.015593976,203.8917857,90.995109
872.508308,7702.139997,10.70530697,3.507796988,18.14838725,0.838306773,7,1,30.58659135,7.
0,1,1,1,0,1,10,9872.508308,7702.139997,10.70530697,3.507796988,18.14838725,0.838306773,7,1,
72.508308,7702.139997,10.70530697,3.507796988,18.14838725,0.838306773,7,1,30.58659135,7.0
530697,3.507796988,18.14838725,0.838306773,7,1,30.58659135,7.015593976,203.8917857,90.995
872.508308,7702.139997,10.70530697,3.507796988,18.14838725,0.838306773,7,1,30.58659135,7.
245221,4.122005076,18.14838725,0.838306773,7,1,19.40617149,16.48802031,331.9702158,85.218
44.103282,5540.207145,11.81245221,4.122005076,18.14838725,0.838306773,7,1,19.40617149,16.
444.103282,5540.207145,11.81245221,4.122005076,18.14838725,0.838306773,7,1,19.40617149,16
221,4.122005076,18.14838725,0.838306773,7,1,19.40617149,16.48802031,331.9702158,85.218405
444.103282,5540.207145,11.81245221,4.122005076,18.14838725,0.838306773,7,1,19.40617149,16
11.81245221,4.122005076,18.14838725,0.838306773,7,1,19.40617149,16.48802031,331.9702158,8
81245221,4.122005076,18.14838725,0.838306773,7,1,19.40617149,16.48802031,331.9702158,85.2
44.103282,5540.207145,11.81245221,4.122005076,18.14838725,0.838306773,7,1,19.40617149,16.
5,0,1,0,1,1,12,9444.103282,5540.207145,11.81245221,4.122005076,18.14838725,0.838306773,7,1,
5,0,1,0,1,1,12,9444.103282,5540.207145,11.81245221,4.122005076,18.14838725,0.838306773,7,1,
245221,4.122005076,18.14838725,0.838306773,7,1,19.40617149,16.48802031,331.9702158,85.218
5,0,1,0,1,1,12,9444.103282,5540.207145,11.81245221,4.122005076,18.14838725,0.838306773,7,1,
44.103282,5540.207145,11.81245221,4.122005076,18.14838725,0.838306773,7,1,19.40617149,16.
44.103282,5540.207145,11.81245221,4.122005076,18.14838725,0.838306773,7,1,19.40617149,16.
44.103282,5540.207145,11.81245221,4.122005076,18.14838725,0.838306773,7,1,19.40617149,16.
245221,4.122005076,18.14838725,0.838306773,7,1,19.40617149,16.48802031,331.9702158,85.218
44.103282,5540.207145,11.81245221,4.122005076,18.14838725,0.838306773,7,1,19.40617149,16.
5,0,1,0,1,1,12,9444.103282,5540.207145,11.81245221,4.122005076,18.14838725,0.838306773,7,1,
5,0,1,0,1,1,12,9444.103282,5540.207145,11.81245221,4.122005076,18.14838725,0.838306773,7,1,
65,0,1,0,1,1,12,9444.103282,5540.207145,11.81245221,4.122005076,18.14838725,0.838306773,7,1
245221,4.122005076,18.14838725,0.838306773,7,1,19.40617149,16.48802031,331.9702158,85.218

44.103282,5540.207145,11.81245221,4.122005076,18.14838725,0.838306773,7,1,19.40617149,16.
44.103282,5540.207145,11.81245221,4.122005076,18.14838725,0.838306773,7,1,19.40617149,16.
44.103282,5540.207145,11.81245221,4.122005076,18.14838725,0.838306773,7,1,19.40617149,16.
44.103282,5540.207145,11.81245221,4.122005076,18.14838725,0.838306773,7,1,19.40617149,16.
44.103282,5540.207145,11.81245221,4.122005076,18.14838725,0.838306773,7,1,19.40617149,16.
4.103282,5540.207145,11.81245221,4.122005076,18.14838725,0.838306773,7,1,19.40617149,16.4
44.103282,5540.207145,11.81245221,4.122005076,18.14838725,0.838306773,7,1,19.40617149,16.
44.103282,5540.207145,11.81245221,4.122005076,18.14838725,0.838306773,7,1,19.40617149,16.
5,0,1,0,1,1,12,9444.103282,5540.207145,11.81245221,4.122005076,18.14838725,0.838306773,7,1,
245221,4.122005076,18.14838725,0.838306773,7,1,19.40617149,16.48802031,331.9702158,85.218
5,0,1,0,1,1,12,9444.103282,5540.207145,11.81245221,4.122005076,18.14838725,0.838306773,7,1,
245221,4.122005076,18.14838725,0.838306773,7,1,19.40617149,16.48802031,331.9702158,85.218
4.103282,5540.207145,11.81245221,4.122005076,18.14838725,0.838306773,7,1,19.40617149,16.4
444.103282,5540.207145,11.81245221,4.122005076,18.14838725,0.838306773,7,1,19.40617149,1
221,4.122005076,18.14838725,0.838306773,7,1,19.40617149,16.48802031,331.9702158,85.218405
65,0,1,0,1,1,12,9444.103282,5540.207145,11.81245221,4.122005076,18.14838725,0.838306773,7,
5,0,1,0,1,1,12,9444.103282,5540.207145,11.81245221,4.122005076,18.14838725,0.838306773,7,1,
44.103282,5540.207145,11.81245221,4.122005076,18.14838725,0.838306773,7,1,19.40617149,16.
31245221,4.122005076,18.14838725,0.838306773,7,1,19.40617149,16.48802031,331.9702158,85.2
523.924014,5736.686029,12.33389753,3.829590412,18.14838725,0.838306773,7,1,18.91197621,15
12.33389753,3.829590412,18.14838725,0.838306773,7,1,18.91197621,15.31836165,324.079005,86
23.924014,5736.686029,12.33389753,3.829590412,18.14838725,0.838306773,7,1,18.91197621,15.
3.924014,5736.686029,12.33389753,3.829590412,18.14838725,0.838306773,7,1,18.91197621,15.3
389753,3.829590412,18.14838725,0.838306773,7,1,18.91197621,15.31836165,324.079005,86.3372
3.924014,5736.686029,12.33389753,3.829590412,18.14838725,0.838306773,7,1,18.91197621,15.3
9753,3.829590412,18.14838725,0.838306773,7,1,18.91197621,15.31836165,324.079005,86.337282
12.33389753,3.829590412,18.14838725,0.838306773,7,1,18.91197621,15.31836165,324.079005,86
523.924014,5736.686029,12.33389753,3.829590412,18.14838725,0.838306773,7,1,18.91197621,1
517676,3,2,206,71,1,1,0,1,1,11,9523.924014,5736.686029,12.33389753,3.829590412,18.14838725,
23.924014,5736.686029,12.33389753,3.829590412,18.14838725,0.838306773,7,1,18.91197621,15.
1,1,1,0,1,1,11,9523.924014,5736.686029,12.33389753,3.829590412,18.14838725,0.838306773,7,1,
1,1,1,0,1,1,11,9523.924014,5736.686029,12.33389753,3.829590412,18.14838725,0.838306773,7,1,
753,3.829590412,18.14838725,0.838306773,7,1,18.91197621,15.31836165,324.079005,86.3372826
53,3.829590412,18.14838725,0.838306773,7,1,18.91197621,15.31836165,324.079005,86.33728268
9753,3.829590412,18.14838725,0.838306773,7,1,18.91197621,15.31836165,324.079005,86.337282
9753,3.829590412,18.14838725,0.838306773,7,1,18.91197621,15.31836165,324.079005,86.337282
1,1,1,0,1,1,11,9523.924014,5736.686029,12.33389753,3.829590412,18.14838725,0.838306773,7,1,
23.924014,5736.686029,12.33389753,3.829590412,18.14838725,0.838306773,7,1,18.91197621,15.
23.924014,5736.686029,12.33389753,3.829590412,18.14838725,0.838306773,7,1,18.91197621,15.
3.924014,5736.686029,12.33389753,3.829590412,18.14838725,0.838306773,7,1,18.91197621,15.3
23.924014,5736.686029,12.33389753,3.829590412,18.14838725,0.838306773,7,1,18.91197621,15.
3.924014,5736.686029,12.33389753,3.829590412,18.14838725,0.838306773,7,1,18.91197621,15.3
23.924014,5736.686029,12.33389753,3.829590412,18.14838725,0.838306773,7,1,18.91197621,15.
23.924014,5736.686029,12.33389753,3.829590412,18.14838725,0.838306773,7,1,18.91197621,15.
523.924014,5736.686029,12.33389753,3.829590412,18.14838725,0.838306773,7,1,18.91197621,1
11.66527225,34.43361432,0,8,0,28.1716831,11.66527225,344.1910237,96.03982874,85.54532986,
8.42662,8628.066712,10.2442484,11.66527225,34.43361432,0,8,0,28.1716831,11.66527225,344.1
0617676,3,2,206,80,1,1,1,0,1,4,11308.42662,8628.066712,10.2442484,11.66527225,34.43361432,0,
4,11.66527225,34.43361432,0,8,0,28.1716831,11.66527225,344.1910237,96.03982874,85.5453298
8.42662,8628.066712,10.2442484,11.66527225,34.43361432,0,8,0,28.1716831,11.66527225,344.1

11.66527225,34.43361432,0,8,0,28.1716831,11.66527225,344.1910237,96.03982874,85.54532986,
8.42662,8628.066712,10.2442484,11.66527225,34.43361432,0,8,0,28.1716831,11.66527225,344.1
308.42662,8628.066712,10.2442484,11.66527225,34.43361432,0,8,0,28.1716831,11.66527225,344
4,11.66527225,34.43361432,0,8,0,28.1716831,11.66527225,344.1910237,96.03982874,85.5453298
2484,11.66527225,34.43361432,0,8,0,28.1716831,11.66527225,344.1910237,96.03982874,85.5453
0617676,3,2,206,80,1,1,1,0,1,4,11308.42662,8628.066712,10.2442484,11.66527225,34.43361432,0,
8.42662,8628.066712,10.2442484,11.66527225,34.43361432,0,8,0,28.1716831,11.66527225,344.1
1308.42662,8628.066712,10.2442484,11.66527225,34.43361432,0,8,0,28.1716831,11.66527225,344
8.42662,8628.066712,10.2442484,11.66527225,34.43361432,0,8,0,28.1716831,11.66527225,344.1
11.66527225,34.43361432,0,8,0,28.1716831,11.66527225,344.1910237,96.03982874,85.54532986,
308.42662,8628.066712,10.2442484,11.66527225,34.43361432,0,8,0,28.1716831,11.66527225,344
8.42662,8628.066712,10.2442484,11.66527225,34.43361432,0,8,0,28.1716831,11.66527225,344.1
8.42662,8628.066712,10.2442484,11.66527225,34.43361432,0,8,0,28.1716831,11.66527225,344.1
4,11.66527225,34.43361432,0,8,0,28.1716831,11.66527225,344.1910237,96.03982874,85.5453298
095251,15.00033862,18.095824,0,6,0,27.47729256,11.25025396,321.1691824,93.29499333,86.2519
15.10319,8466.706382,8.946095251,15.00033862,18.095824,0,6,0,27.47729256,11.25025396,321.
251,15.00033862,18.095824,0,6,0,27.47729256,11.25025396,321.1691824,93.29499333,86.251947
15.10319,8466.706382,8.946095251,15.00033862,18.095824,0,6,0,27.47729256,11.25025396,321.
51,15.00033862,18.095824,0,6,0,27.47729256,11.25025396,321.1691824,93.29499333,86.2519470
251,15.00033862,18.095824,0,6,0,27.47729256,11.25025396,321.1691824,93.29499333,86.251947
15.10319,8466.706382,8.946095251,15.00033862,18.095824,0,6,0,27.47729256,11.25025396,321.
15.10319,8466.706382,8.946095251,15.00033862,18.095824,0,6,0,27.47729256,11.25025396,321.
946095251,15.00033862,18.095824,0,6,0,27.47729256,11.25025396,321.1691824,93.29499333,86.
5095251,15.00033862,18.095824,0,6,0,27.47729256,11.25025396,321.1691824,93.29499333,86.251
946095251,15.00033862,18.095824,0,6,0,27.47729256,11.25025396,321.1691824,93.29499333,86.
15.10319,8466.706382,8.946095251,15.00033862,18.095824,0,6,0,27.47729256,11.25025396,321.
251,15.00033862,18.095824,0,6,0,27.47729256,11.25025396,321.1691824,93.29499333,86.251947
1315.10319,8466.706382,8.946095251,15.00033862,18.095824,0,6,0,27.47729256,11.25025396,32
15.10319,8466.706382,8.946095251,15.00033862,18.095824,0,6,0,27.47729256,11.25025396,321.
315.10319,8466.706382,8.946095251,15.00033862,18.095824,0,6,0,27.47729256,11.25025396,321
315.10319,8466.706382,8.946095251,15.00033862,18.095824,0,6,0,27.47729256,11.25025396,321
,0,1,0,1,1,12,11315.10319,8466.706382,8.946095251,15.00033862,18.095824,0,6,0,27.47729256,1
5251,15.00033862,18.095824,0,6,0,27.47729256,11.25025396,321.1691824,93.29499333,86.25194
79,0,1,0,1,1,12,11315.10319,8466.706382,8.946095251,15.00033862,18.095824,0,6,0,27.47729256
15.10319,8466.706382,8.946095251,15.00033862,18.095824,0,6,0,27.47729256,11.25025396,321.
15.10319,8466.706382,8.946095251,15.00033862,18.095824,0,6,0,27.47729256,11.25025396,321.
1315.10319,8466.706382,8.946095251,15.00033862,18.095824,0,6,0,27.47729256,11.25025396,32
251,15.00033862,18.095824,0,6,0,27.47729256,11.25025396,321.1691824,93.29499333,86.251947
315.10319,8466.706382,8.946095251,15.00033862,18.095824,0,6,0,27.47729256,11.25025396,321
15.10319,8466.706382,8.946095251,15.00033862,18.095824,0,6,0,27.47729256,11.25025396,321.
15.10319,8466.706382,8.946095251,15.00033862,18.095824,0,6,0,27.47729256,11.25025396,321.
11315.10319,8466.706382,8.946095251,15.00033862,18.095824,0,6,0,27.47729256,11.25025396,3
9,0,1,0,1,1,12,11315.10319,8466.706382,8.946095251,15.00033862,18.095824,0,6,0,27.47729256,
79,0,1,0,1,1,12,11315.10319,8466.706382,8.946095251,15.00033862,18.095824,0,6,0,27.47729256
50.3735,5224.914772,12.24689595,4.063221959,105.5820322,0.838306773,8,1,20.66663691,16.25
,1,1,1,1,1,12,10250.3735,5224.914772,12.24689595,4.063221959,105.5820322,0.838306773,8,1,20
,1,1,1,1,1,12,10250.3735,5224.914772,12.24689595,4.063221959,105.5820322,0.838306773,8,1,20
66,1,1,1,1,1,12,10250.3735,5224.914772,12.24689595,4.063221959,105.5820322,0.838306773,8,1,
.063221959,105.5820322,0.838306773,8,1,20.66663691,16.25288783,245.9306413,90.32085761,8
4689595,4.063221959,105.5820322,0.838306773,8,1,20.66663691,16.25288783,245.9306413,90.32

95,4.063221959,105.5820322,0.838306773,8,1,20.66663691,16.25288783,245.9306413,90.3208576
0250.3735,5224.914772,12.24689595,4.063221959,105.5820322,0.838306773,8,1,20.66663691,16.
,66,1,1,1,1,1,12,10250.3735,5224.914772,12.24689595,4.063221959,105.5820322,0.838306773,8,1
24689595,4.063221959,105.5820322,0.838306773,8,1,20.66663691,16.25288783,245.9306413,90.3
250.3735,5224.914772,12.24689595,4.063221959,105.5820322,0.838306773,8,1,20.66663691,16.2
24689595,4.063221959,105.5820322,0.838306773,8,1,20.66663691,16.25288783,245.9306413,90.3
89595,4.063221959,105.5820322,0.838306773,8,1,20.66663691,16.25288783,245.9306413,90.3208
.063221959,105.5820322,0.838306773,8,1,20.66663691,16.25288783,245.9306413,90.32085761,8
50.3735,5224.914772,12.24689595,4.063221959,105.5820322,0.838306773,8,1,20.66663691,16.25
50.3735,5224.914772,12.24689595,4.063221959,105.5820322,0.838306773,8,1,20.66663691,16.25
0250.3735,5224.914772,12.24689595,4.063221959,105.5820322,0.838306773,8,1,20.66663691,16.
95,4.063221959,105.5820322,0.838306773,8,1,20.66663691,16.25288783,245.9306413,90.3208576
5,4.063221959,105.5820322,0.838306773,8,1,20.66663691,16.25288783,245.9306413,90.32085761
50.3735,5224.914772,12.24689595,4.063221959,105.5820322,0.838306773,8,1,20.66663691,16.25
50.3735,5224.914772,12.24689595,4.063221959,105.5820322,0.838306773,8,1,20.66663691,16.25
95,4.063221959,105.5820322,0.838306773,8,1,20.66663691,16.25288783,245.9306413,90.3208576
5,4.063221959,105.5820322,0.838306773,8,1,20.66663691,16.25288783,245.9306413,90.32085761
,66,1,1,1,1,1,12,10250.3735,5224.914772,12.24689595,4.063221959,105.5820322,0.838306773,8,1
6,1,1,1,1,1,12,10250.3735,5224.914772,12.24689595,4.063221959,105.5820322,0.838306773,8,1,2
6,1,1,1,1,1,12,10250.3735,5224.914772,12.24689595,4.063221959,105.5820322,0.838306773,8,1,2
0250.3735,5224.914772,12.24689595,4.063221959,105.5820322,0.838306773,8,1,20.66663691,16.
0250.3735,5224.914772,12.24689595,4.063221959,105.5820322,0.838306773,8,1,20.66663691,16.
95,4.063221959,105.5820322,0.838306773,8,1,20.66663691,16.25288783,245.9306413,90.3208576
250.3735,5224.914772,12.24689595,4.063221959,105.5820322,0.838306773,8,1,20.66663691,16.2
0250.3735,5224.914772,12.24689595,4.063221959,105.5820322,0.838306773,8,1,20.66663691,16.
89595,4.063221959,105.5820322,0.838306773,8,1,20.66663691,16.25288783,245.9306413,90.3208
6,1,1,1,1,1,12,10250.3735,5224.914772,12.24689595,4.063221959,105.5820322,0.838306773,8,1,2
24689595,4.063221959,105.5820322,0.838306773,8,1,20.66663691,16.25288783,245.9306413,90.3
50.3735,5224.914772,12.24689595,4.063221959,105.5820322,0.838306773,8,1,20.66663691,16.25
50.3735,5224.914772,12.24689595,4.063221959,105.5820322,0.838306773,8,1,20.66663691,16.25
39,12.67756223,6.175828578,0,3,0,27.62812943,0,441.1810005,88.70083659,67.61366525,31.2634
,1,1,0,1,1,12,10396.67341,9785.089064,6.543504339,12.67756223,6.175828578,0,3,0,27.62812943
8,1,1,0,1,1,12,10396.67341,9785.089064,6.543504339,12.67756223,6.175828578,0,3,0,27.6281294
396.67341,9785.089064,6.543504339,12.67756223,6.175828578,0,3,0,27.62812943,0,441.1810005
68,1,1,0,1,1,12,10396.67341,9785.089064,6.543504339,12.67756223,6.175828578,0,3,0,27.628129
339,12.67756223,6.175828578,0,3,0,27.62812943,0,441.1810005,88.70083659,67.61366525,31.263
396.67341,9785.089064,6.543504339,12.67756223,6.175828578,0,3,0,27.62812943,0,441.1810005
396.67341,9785.089064,6.543504339,12.67756223,6.175828578,0,3,0,27.62812943,0,441.1810005
396.67341,9785.089064,6.543504339,12.67756223,6.175828578,0,3,0,27.62812943,0,441.1810005
96.67341,9785.089064,6.543504339,12.67756223,6.175828578,0,3,0,27.62812943,0,441.1810005,8
39,12.67756223,6.175828578,0,3,0,27.62812943,0,441.1810005,88.70083659,67.61366525,31.2634
517676,3,2,206,68,1,1,0,1,1,12,10396.67341,9785.089064,6.543504339,12.67756223,6.175828578,0
8,1,1,0,1,1,12,10396.67341,9785.089064,6.543504339,12.67756223,6.175828578,0,3,0,27.6281294
68,1,1,0,1,1,12,10396.67341,9785.089064,6.543504339,12.67756223,6.175828578,0,3,0,27.628129
396.67341,9785.089064,6.543504339,12.67756223,6.175828578,0,3,0,27.62812943,0,441.1810005
339,12.67756223,6.175828578,0,3,0,27.62812943,0,441.1810005,88.70083659,67.61366525,31.263
8,1,1,0,1,1,12,10396.67341,9785.089064,6.543504339,12.67756223,6.175828578,0,3,0,27.6281294
96.67341,9785.089064,6.543504339,12.67756223,6.175828578,0,3,0,27.62812943,0,441.1810005,8
,10396.67341,9785.089064,6.543504339,12.67756223,6.175828578,0,3,0,27.62812943,0,441.1810
10396.67341,9785.089064,6.543504339,12.67756223,6.175828578,0,3,0,27.62812943,0,441.18100

,1,1,0,1,1,12,10396.67341,9785.089064,6.543504339,12.67756223,6.175828578,0,3,0,27.62812943
8,1,1,0,1,1,12,10396.67341,9785.089064,6.543504339,12.67756223,6.175828578,0,3,0,27.6281294
396.67341,9785.089064,6.543504339,12.67756223,6.175828578,0,3,0,27.62812943,0,441.1810005
8,1,1,0,1,1,12,10396.67341,9785.089064,6.543504339,12.67756223,6.175828578,0,3,0,27.6281294
39,12.67756223,6.175828578,0,3,0,27.62812943,0,441.1810005,88.70083659,67.61366525,31.2634
339,12.67756223,6.175828578,0,3,0,27.62812943,0,441.1810005,88.70083659,67.61366525,31.263
543504339,12.67756223,6.175828578,0,3,0,27.62812943,0,441.1810005,88.70083659,67.6136652
396.67341,9785.089064,6.543504339,12.67756223,6.175828578,0,3,0,27.62812943,0,441.1810005
543504339,12.67756223,6.175828578,0,3,0,27.62812943,0,441.1810005,88.70083659,67.6136652
04,16.28422943,20.94852046,0,5,0,29.72926762,89.56326187,172.2709306,89.18780285,118.0606
16904,16.28422943,20.94852046,0,5,0,29.72926762,89.56326187,172.2709306,89.18780285,118.06
04,16.28422943,20.94852046,0,5,0,29.72926762,89.56326187,172.2709306,89.18780285,118.0606
2316904,16.28422943,20.94852046,0,5,0,29.72926762,89.56326187,172.2709306,89.18780285,118
5.62003,12899.68011,7.432316904,16.28422943,20.94852046,0,5,0,29.72926762,89.56326187,172
5.62003,12899.68011,7.432316904,16.28422943,20.94852046,0,5,0,29.72926762,89.56326187,172
04,16.28422943,20.94852046,0,5,0,29.72926762,89.56326187,172.2709306,89.18780285,118.0606
65.62003,12899.68011,7.432316904,16.28422943,20.94852046,0,5,0,29.72926762,89.56326187,17
4,1,1,0,1,1,6,10965.62003,12899.68011,7.432316904,16.28422943,20.94852046,0,5,0,29.72926762
32316904,16.28422943,20.94852046,0,5,0,29.72926762,89.56326187,172.2709306,89.18780285,1
2316904,16.28422943,20.94852046,0,5,0,29.72926762,89.56326187,172.2709306,89.18780285,118
965.62003,12899.68011,7.432316904,16.28422943,20.94852046,0,5,0,29.72926762,89.56326187,1
5.62003,12899.68011,7.432316904,16.28422943,20.94852046,0,5,0,29.72926762,89.56326187,172
5.62003,12899.68011,7.432316904,16.28422943,20.94852046,0,5,0,29.72926762,89.56326187,172
5.62003,12899.68011,7.432316904,16.28422943,20.94852046,0,5,0,29.72926762,89.56326187,172
5.62003,12899.68011,7.432316904,16.28422943,20.94852046,0,5,0,29.72926762,89.56326187,172
74,1,1,0,1,1,6,10965.62003,12899.68011,7.432316904,16.28422943,20.94852046,0,5,0,29.7292676
16904,16.28422943,20.94852046,0,5,0,29.72926762,89.56326187,172.2709306,89.18780285,118.06
74,1,1,0,1,1,6,10965.62003,12899.68011,7.432316904,16.28422943,20.94852046,0,5,0,29.7292676
2316904,16.28422943,20.94852046,0,5,0,29.72926762,89.56326187,172.2709306,89.18780285,118
04,16.28422943,20.94852046,0,5,0,29.72926762,89.56326187,172.2709306,89.18780285,118.0606
965.62003,12899.68011,7.432316904,16.28422943,20.94852046,0,5,0,29.72926762,89.56326187,1
16904,16.28422943,20.94852046,0,5,0,29.72926762,89.56326187,172.2709306,89.18780285,118.06
5.62003,12899.68011,7.432316904,16.28422943,20.94852046,0,5,0,29.72926762,89.56326187,172
74,1,1,0,1,1,6,10965.62003,12899.68011,7.432316904,16.28422943,20.94852046,0,5,0,29.7292676
32316904,16.28422943,20.94852046,0,5,0,29.72926762,89.56326187,172.2709306,89.18780285,1
5.62003,12899.68011,7.432316904,16.28422943,20.94852046,0,5,0,29.72926762,89.56326187,172
16904,16.28422943,20.94852046,0,5,0,29.72926762,89.56326187,172.2709306,89.18780285,118.06
04,16.28422943,20.94852046,0,5,0,29.72926762,89.56326187,172.2709306,89.18780285,118.0606
931.26621,8228.046922,10.25373802,11.63377748,18.14838725,0.838306773,7,1,30.76121407,7.7
3,1,1,1,1,1,12,10931.26621,8228.046922,10.25373802,11.63377748,18.14838725,0.838306773,7,1,
.25373802,11.63377748,18.14838725,0.838306773,7,1,30.76121407,7.755851655,113.743523,88.1
31.26621,8228.046922,10.25373802,11.63377748,18.14838725,0.838306773,7,1,30.76121407,7.75
02,11.63377748,18.14838725,0.838306773,7,1,30.76121407,7.755851655,113.743523,88.18214701
802,11.63377748,18.14838725,0.838306773,7,1,30.76121407,7.755851655,113.743523,88.1821470
31.26621,8228.046922,10.25373802,11.63377748,18.14838725,0.838306773,7,1,30.76121407,7.75
802,11.63377748,18.14838725,0.838306773,7,1,30.76121407,7.755851655,113.743523,88.1821470
931.26621,8228.046922,10.25373802,11.63377748,18.14838725,0.838306773,7,1,30.76121407,7.7
802,11.63377748,18.14838725,0.838306773,7,1,30.76121407,7.755851655,113.743523,88.1821470
0931.26621,8228.046922,10.25373802,11.63377748,18.14838725,0.838306773,7,1,30.76121407,7.
0931.26621,8228.046922,10.25373802,11.63377748,18.14838725,0.838306773,7,1,30.76121407,7.

931.26621,8228.046922,10.25373802,11.63377748,18.14838725,0.838306773,7,1,30.76121407,7.7
31.26621,8228.046922,10.25373802,11.63377748,18.14838725,0.838306773,7,1,30.76121407,7.75
73,1,1,1,1,1,12,10931.26621,8228.046922,10.25373802,11.63377748,18.14838725,0.838306773,7,
10931.26621,8228.046922,10.25373802,11.63377748,18.14838725,0.838306773,7,1,30.76121407,7
931.26621,8228.046922,10.25373802,11.63377748,18.14838725,0.838306773,7,1,30.76121407,7.7
73,1,1,1,1,1,12,10931.26621,8228.046922,10.25373802,11.63377748,18.14838725,0.838306773,7,
25373802,11.63377748,18.14838725,0.838306773,7,1,30.76121407,7.755851655,113.743523,88.18
73,1,1,1,1,1,12,10931.26621,8228.046922,10.25373802,11.63377748,18.14838725,0.838306773,7,
373802,11.63377748,18.14838725,0.838306773,7,1,30.76121407,7.755851655,113.743523,88.1821
31.26621,8228.046922,10.25373802,11.63377748,18.14838725,0.838306773,7,1,30.76121407,7.75
0.25373802,11.63377748,18.14838725,0.838306773,7,1,30.76121407,7.755851655,113.743523,88.
31.26621,8228.046922,10.25373802,11.63377748,18.14838725,0.838306773,7,1,30.76121407,7.75
31.26621,8228.046922,10.25373802,11.63377748,18.14838725,0.838306773,7,1,30.76121407,7.75
3,1,1,1,1,1,12,10931.26621,8228.046922,10.25373802,11.63377748,18.14838725,0.838306773,7,1,
8.139381995,105.6213574,5.982691169,9,2,30.41811338,16.27876399,106.6760007,94.01962318,
7524529,8.139381995,105.6213574,5.982691169,9,2,30.41811338,16.27876399,106.6760007,94.0
9,8.139381995,105.6213574,5.982691169,9,2,30.41811338,16.27876399,106.6760007,94.01962318
9,8.139381995,105.6213574,5.982691169,9,2,30.41811338,16.27876399,106.6760007,94.01962318
9,8.139381995,105.6213574,5.982691169,9,2,30.41811338,16.27876399,106.6760007,94.01962318
8.03366,5832.334896,11.7524529,8.139381995,105.6213574,5.982691169,9,2,30.41811338,16.278
524529,8.139381995,105.6213574,5.982691169,9,2,30.41811338,16.27876399,106.6760007,94.019
08.03366,5832.334896,11.7524529,8.139381995,105.6213574,5.982691169,9,2,30.41811338,16.27
8.03366,5832.334896,11.7524529,8.139381995,105.6213574,5.982691169,9,2,30.41811338,16.278
8.03366,5832.334896,11.7524529,8.139381995,105.6213574,5.982691169,9,2,30.41811338,16.278
7524529,8.139381995,105.6213574,5.982691169,9,2,30.41811338,16.27876399,106.6760007,94.0
208.03366,5832.334896,11.7524529,8.139381995,105.6213574,5.982691169,9,2,30.41811338,16.2
4529,8.139381995,105.6213574,5.982691169,9,2,30.41811338,16.27876399,106.6760007,94.01962
524529,8.139381995,105.6213574,5.982691169,9,2,30.41811338,16.27876399,106.6760007,94.01
8.139381995,105.6213574,5.982691169,9,2,30.41811338,16.27876399,106.6760007,94.01962318,
29,8.139381995,105.6213574,5.982691169,9,2,30.41811338,16.27876399,106.6760007,94.0196231
29,8.139381995,105.6213574,5.982691169,9,2,30.41811338,16.27876399,106.6760007,94.0196231
9,8.139381995,105.6213574,5.982691169,9,2,30.41811338,16.27876399,106.6760007,94.01962318
6,0,1,0,0,1,9,11208.03366,5832.334896,11.7524529,8.139381995,105.6213574,5.982691169,9,2,30
6,0,1,0,0,1,9,11208.03366,5832.334896,11.7524529,8.139381995,105.6213574,5.982691169,9,2,30
76,0,1,0,0,1,9,11208.03366,5832.334896,11.7524529,8.139381995,105.6213574,5.982691169,9,2,3
8.139381995,105.6213574,5.982691169,9,2,30.41811338,16.27876399,106.6760007,94.01962318,
208.03366,5832.334896,11.7524529,8.139381995,105.6213574,5.982691169,9,2,30.41811338,16.2
8.03366,5832.334896,11.7524529,8.139381995,105.6213574,5.982691169,9,2,30.41811338,16.278
8.03366,5832.334896,11.7524529,8.139381995,105.6213574,5.982691169,9,2,30.41811338,16.278
8.03366,5832.334896,11.7524529,8.139381995,105.6213574,5.982691169,9,2,30.41811338,16.278
4529,8.139381995,105.6213574,5.982691169,9,2,30.41811338,16.27876399,106.6760007,94.01962
6,0,1,0,0,1,9,11208.03366,5832.334896,11.7524529,8.139381995,105.6213574,5.982691169,9,2,30
6,0,1,0,0,1,9,11208.03366,5832.334896,11.7524529,8.139381995,105.6213574,5.982691169,9,2,30
524529,8.139381995,105.6213574,5.982691169,9,2,30.41811338,16.27876399,106.6760007,94.019
781,3.88364735,105.5820322,0.838306773,8,1,31.9625492,15.5345894,41.46717819,93.16743066,
975.77993,7092.243956,10.88086781,3.88364735,105.5820322,0.838306773,8,1,31.9625492,15.53
31,3.88364735,105.5820322,0.838306773,8,1,31.9625492,15.5345894,41.46717819,93.16743066,8
370605,3,2,206,70,0,1,0,1,1,12,10975.77993,7092.243956,10.88086781,3.88364735,105.5820322,0.
0975.77993,7092.243956,10.88086781,3.88364735,105.5820322,0.838306773,8,1,31.9625492,15.5
975.77993,7092.243956,10.88086781,3.88364735,105.5820322,0.838306773,8,1,31.9625492,15.53

781,3.88364735,105.5820322,0.838306773,8,1,31.9625492,15.5345894,41.46717819,93.16743066,8
3.88364735,105.5820322,0.838306773,8,1,31.9625492,15.5345894,41.46717819,93.16743066,81.5
88086781,3.88364735,105.5820322,0.838306773,8,1,31.9625492,15.5345894,41.46717819,93.1674
0,0,1,0,1,1,12,10975.77993,7092.243956,10.88086781,3.88364735,105.5820322,0.838306773,8,1,3
0975.77993,7092.243956,10.88086781,3.88364735,105.5820322,0.838306773,8,1,31.9625492,15.5
975.77993,7092.243956,10.88086781,3.88364735,105.5820322,0.838306773,8,1,31.9625492,15.53
31,3.88364735,105.5820322,0.838306773,8,1,31.9625492,15.5345894,41.46717819,93.16743066,8
975.77993,7092.243956,10.88086781,3.88364735,105.5820322,0.838306773,8,1,31.9625492,15.53
975.77993,7092.243956,10.88086781,3.88364735,105.5820322,0.838306773,8,1,31.9625492,15.53
10975.77993,7092.243956,10.88086781,3.88364735,105.5820322,0.838306773,8,1,31.9625492,15.5
086781,3.88364735,105.5820322,0.838306773,8,1,31.9625492,15.5345894,41.46717819,93.167430
31,3.88364735,105.5820322,0.838306773,8,1,31.9625492,15.5345894,41.46717819,93.16743066,8
,70,0,1,0,1,1,12,10975.77993,7092.243956,10.88086781,3.88364735,105.5820322,0.838306773,8,1
975.77993,7092.243956,10.88086781,3.88364735,105.5820322,0.838306773,8,1,31.9625492,15.53
975.77993,7092.243956,10.88086781,3.88364735,105.5820322,0.838306773,8,1,31.9625492,15.53
75.77993,7092.243956,10.88086781,3.88364735,105.5820322,0.838306773,8,1,31.9625492,15.534
,0,1,0,1,1,12,10975.77993,7092.243956,10.88086781,3.88364735,105.5820322,0.838306773,8,1,31
086781,3.88364735,105.5820322,0.838306773,8,1,31.9625492,15.5345894,41.46717819,93.167430
975.77993,7092.243956,10.88086781,3.88364735,105.5820322,0.838306773,8,1,31.9625492,15.53
70,0,1,0,1,1,12,10975.77993,7092.243956,10.88086781,3.88364735,105.5820322,0.838306773,8,1,
517676,3,2,206,76,0,1,0,1,1,10,10901.90647,8355.935604,10.22935892,11.53342193,18.14838725,
01.90647,8355.935604,10.22935892,11.53342193,18.14838725,0.838306773,7,1,31.37003401,7.68
01.90647,8355.935604,10.22935892,11.53342193,18.14838725,0.838306773,7,1,31.37003401,7.68
892,11.53342193,18.14838725,0.838306773,7,1,31.37003401,7.68894795,133.4418285,88.6544439
01.90647,8355.935604,10.22935892,11.53342193,18.14838725,0.838306773,7,1,31.37003401,7.68
92,11.53342193,18.14838725,0.838306773,7,1,31.37003401,7.68894795,133.4418285,88.65444394
6,0,1,0,1,1,10,10901.90647,8355.935604,10.22935892,11.53342193,18.14838725,0.838306773,7,1,
0901.90647,8355.935604,10.22935892,11.53342193,18.14838725,0.838306773,7,1,31.37003401,7.
6,0,1,0,1,1,10,10901.90647,8355.935604,10.22935892,11.53342193,18.14838725,0.838306773,7,1,
892,11.53342193,18.14838725,0.838306773,7,1,31.37003401,7.68894795,133.4418285,88.6544439
901.90647,8355.935604,10.22935892,11.53342193,18.14838725,0.838306773,7,1,31.37003401,7.6
01.90647,8355.935604,10.22935892,11.53342193,18.14838725,0.838306773,7,1,31.37003401,7.68
76,0,1,0,1,1,10,10901.90647,8355.935604,10.22935892,11.53342193,18.14838725,0.838306773,7,1
901.90647,8355.935604,10.22935892,11.53342193,18.14838725,0.838306773,7,1,31.37003401,7.6
.22935892,11.53342193,18.14838725,0.838306773,7,1,31.37003401,7.68894795,133.4418285,88.6
6,0,1,0,1,1,10,10901.90647,8355.935604,10.22935892,11.53342193,18.14838725,0.838306773,7,1,
901.90647,8355.935604,10.22935892,11.53342193,18.14838725,0.838306773,7,1,31.37003401,7.6
01.90647,8355.935604,10.22935892,11.53342193,18.14838725,0.838306773,7,1,31.37003401,7.68
901.90647,8355.935604,10.22935892,11.53342193,18.14838725,0.838306773,7,1,31.37003401,7.6
01.90647,8355.935604,10.22935892,11.53342193,18.14838725,0.838306773,7,1,31.37003401,7.68
6,0,1,0,1,1,10,10901.90647,8355.935604,10.22935892,11.53342193,18.14838725,0.838306773,7,1,
6,0,1,0,1,1,10,10901.90647,8355.935604,10.22935892,11.53342193,18.14838725,0.838306773,7,1,
901.90647,8355.935604,10.22935892,11.53342193,18.14838725,0.838306773,7,1,31.37003401,7.6
01.90647,8355.935604,10.22935892,11.53342193,18.14838725,0.838306773,7,1,31.37003401,7.68
35892,11.53342193,18.14838725,0.838306773,7,1,31.37003401,7.68894795,133.4418285,88.6544
01.90647,8355.935604,10.22935892,11.53342193,18.14838725,0.838306773,7,1,31.37003401,7.68
01.90647,8355.935604,10.22935892,11.53342193,18.14838725,0.838306773,7,1,31.37003401,7.68
892,11.53342193,18.14838725,0.838306773,7,1,31.37003401,7.68894795,133.4418285,88.6544439
76,0,1,0,1,1,10,10901.90647,8355.935604,10.22935892,11.53342193,18.14838725,0.838306773,7,1
35892,11.53342193,18.14838725,0.838306773,7,1,31.37003401,7.68894795,133.4418285,88.6544

0901.90647,8355.935604,10.22935892,11.53342193,18.14838725,0.838306773,7,1,31.37003401,7.
01.90647,8355.935604,10.22935892,11.53342193,18.14838725,0.838306773,7,1,31.37003401,7.68
392,11.53342193,18.14838725,0.838306773,7,1,31.37003401,7.68894795,133.4418285,88.6544439
935892,11.53342193,18.14838725,0.838306773,7,1,31.37003401,7.68894795,133.4418285,88.6544
0901.90647,8355.935604,10.22935892,11.53342193,18.14838725,0.838306773,7,1,31.37003401,7.
76,0,1,0,1,1,10,10901.90647,8355.935604,10.22935892,11.53342193,18.14838725,0.838306773,7,
392,11.53342193,18.14838725,0.838306773,7,1,31.37003401,7.68894795,133.4418285,88.6544439
532,11.45243417,18.14838725,0.838306773,7,1,31.25315232,7.634956115,105.3328027,88.324126
9,1,1,1,1,1,12,10711.84153,8490.900718,10.19124532,11.45243417,18.14838725,0.838306773,7,1,
11.84153,8490.900718,10.19124532,11.45243417,18.14838725,0.838306773,7,1,31.25315232,7.63
532,11.45243417,18.14838725,0.838306773,7,1,31.25315232,7.634956115,105.3328027,88.324126
9,1,1,1,1,1,12,10711.84153,8490.900718,10.19124532,11.45243417,18.14838725,0.838306773,7,1,
124532,11.45243417,18.14838725,0.838306773,7,1,31.25315232,7.634956115,105.3328027,88.324
711.84153,8490.900718,10.19124532,11.45243417,18.14838725,0.838306773,7,1,31.25315232,7.6
711.84153,8490.900718,10.19124532,11.45243417,18.14838725,0.838306773,7,1,31.25315232,7.6
19124532,11.45243417,18.14838725,0.838306773,7,1,31.25315232,7.634956115,105.3328027,88.3
11.84153,8490.900718,10.19124532,11.45243417,18.14838725,0.838306773,7,1,31.25315232,7.63
532,11.45243417,18.14838725,0.838306773,7,1,31.25315232,7.634956115,105.3328027,88.324126
711.84153,8490.900718,10.19124532,11.45243417,18.14838725,0.838306773,7,1,31.25315232,7.6
532,11.45243417,18.14838725,0.838306773,7,1,31.25315232,7.634956115,105.3328027,88.324126
124532,11.45243417,18.14838725,0.838306773,7,1,31.25315232,7.634956115,105.3328027,88.324
532,11.45243417,18.14838725,0.838306773,7,1,31.25315232,7.634956115,105.3328027,88.324126
711.84153,8490.900718,10.19124532,11.45243417,18.14838725,0.838306773,7,1,31.25315232,7.6
711.84153,8490.900718,10.19124532,11.45243417,18.14838725,0.838306773,7,1,31.25315232,7.6
11.84153,8490.900718,10.19124532,11.45243417,18.14838725,0.838306773,7,1,31.25315232,7.63
32,11.45243417,18.14838725,0.838306773,7,1,31.25315232,7.634956115,105.3328027,88.3241261
.19124532,11.45243417,18.14838725,0.838306773,7,1,31.25315232,7.634956115,105.3328027,88
11.84153,8490.900718,10.19124532,11.45243417,18.14838725,0.838306773,7,1,31.25315232,7.63
711.84153,8490.900718,10.19124532,11.45243417,18.14838725,0.838306773,7,1,31.25315232,7.6
9,1,1,1,1,1,12,10711.84153,8490.900718,10.19124532,11.45243417,18.14838725,0.838306773,7,1,
19124532,11.45243417,18.14838725,0.838306773,7,1,31.25315232,7.634956115,105.3328027,88.3
711.84153,8490.900718,10.19124532,11.45243417,18.14838725,0.838306773,7,1,31.25315232,7.6
711.84153,8490.900718,10.19124532,11.45243417,18.14838725,0.838306773,7,1,31.25315232,7.6
532,11.45243417,18.14838725,0.838306773,7,1,31.25315232,7.634956115,105.3328027,88.324126
124532,11.45243417,18.14838725,0.838306773,7,1,31.25315232,7.634956115,105.3328027,88.324
19124532,11.45243417,18.14838725,0.838306773,7,1,31.25315232,7.634956115,105.3328027,88.3
.19124532,11.45243417,18.14838725,0.838306773,7,1,31.25315232,7.634956115,105.3328027,88
9,1,1,1,1,1,12,10711.84153,8490.900718,10.19124532,11.45243417,18.14838725,0.838306773,7,1,
11.84153,8490.900718,10.19124532,11.45243417,18.14838725,0.838306773,7,1,31.25315232,7.63
24532,11.45243417,18.14838725,0.838306773,7,1,31.25315232,7.634956115,105.3328027,88.3241
11.84153,8490.900718,10.19124532,11.45243417,18.14838725,0.838306773,7,1,31.25315232,7.63
711.84153,8490.900718,10.19124532,11.45243417,18.14838725,0.838306773,7,1,31.25315232,7.6
11.84153,8490.900718,10.19124532,11.45243417,18.14838725,0.838306773,7,1,31.25315232,7.63
47.69049,8090.101009,10.00346709,18.22938741,18.14838725,0.838306773,7,1,28.67660565,7.29
047.69049,8090.101009,10.00346709,18.22938741,18.14838725,0.838306773,7,1,28.67660565,7.2
.00346709,18.22938741,18.14838725,0.838306773,7,1,28.67660565,7.291754962,234.8123646,91
047.69049,8090.101009,10.00346709,18.22938741,18.14838725,0.838306773,7,1,28.67660565,7.2
047.69049,8090.101009,10.00346709,18.22938741,18.14838725,0.838306773,7,1,28.67660565,7.2
517676,3,2,206,66,0,1,1,1,1,12,11047.69049,8090.101009,10.00346709,18.22938741,18.14838725,
6,0,1,1,1,1,12,11047.69049,8090.101009,10.00346709,18.22938741,18.14838725,0.838306773,7,1,

047.69049,8090.101009,10.00346709,18.22938741,18.14838725,0.838306773,7,1,28.67660565,7.2
00346709,18.22938741,18.14838725,0.838306773,7,1,28.67660565,7.291754962,234.8123646,91.3
047.69049,8090.101009,10.00346709,18.22938741,18.14838725,0.838306773,7,1,28.67660565,7.2
6,0,1,1,1,1,12,11047.69049,8090.101009,10.00346709,18.22938741,18.14838725,0.838306773,7,1,
47.69049,8090.101009,10.00346709,18.22938741,18.14838725,0.838306773,7,1,28.67660565,7.29
1047.69049,8090.101009,10.00346709,18.22938741,18.14838725,0.838306773,7,1,28.67660565,7.
00346709,18.22938741,18.14838725,0.838306773,7,1,28.67660565,7.291754962,234.8123646,91.3
709,18.22938741,18.14838725,0.838306773,7,1,28.67660565,7.291754962,234.8123646,91.364999
346709,18.22938741,18.14838725,0.838306773,7,1,28.67660565,7.291754962,234.8123646,91.364
047.69049,8090.101009,10.00346709,18.22938741,18.14838725,0.838306773,7,1,28.67660565,7.2
47.69049,8090.101009,10.00346709,18.22938741,18.14838725,0.838306773,7,1,28.67660565,7.29
.00346709,18.22938741,18.14838725,0.838306773,7,1,28.67660565,7.291754962,234.8123646,91.
709,18.22938741,18.14838725,0.838306773,7,1,28.67660565,7.291754962,234.8123646,91.364999
709,18.22938741,18.14838725,0.838306773,7,1,28.67660565,7.291754962,234.8123646,91.364999
6,0,1,1,1,1,12,11047.69049,8090.101009,10.00346709,18.22938741,18.14838725,0.838306773,7,1,
66,0,1,1,1,1,12,11047.69049,8090.101009,10.00346709,18.22938741,18.14838725,0.838306773,7,
047.69049,8090.101009,10.00346709,18.22938741,18.14838725,0.838306773,7,1,28.67660565,7.2
6,0,1,1,1,1,12,11047.69049,8090.101009,10.00346709,18.22938741,18.14838725,0.838306773,7,1,
517676,3,2,206,66,0,1,1,1,1,12,11047.69049,8090.101009,10.00346709,18.22938741,18.14838725,
66,0,1,1,1,1,12,11047.69049,8090.101009,10.00346709,18.22938741,18.14838725,0.838306773,7,
47.69049,8090.101009,10.00346709,18.22938741,18.14838725,0.838306773,7,1,28.67660565,7.29
6,0,1,1,1,1,12,11047.69049,8090.101009,10.00346709,18.22938741,18.14838725,0.838306773,7,1,
6,0,1,1,1,1,12,11047.69049,8090.101009,10.00346709,18.22938741,18.14838725,0.838306773,7,1,
346709,18.22938741,18.14838725,0.838306773,7,1,28.67660565,7.291754962,234.8123646,91.364
47.69049,8090.101009,10.00346709,18.22938741,18.14838725,0.838306773,7,1,28.67660565,7.29
47.69049,8090.101009,10.00346709,18.22938741,18.14838725,0.838306773,7,1,28.67660565,7.29
47.69049,8090.101009,10.00346709,18.22938741,18.14838725,0.838306773,7,1,28.67660565,7.29
047.69049,8090.101009,10.00346709,18.22938741,18.14838725,0.838306773,7,1,28.67660565,7.2
6,0,1,1,1,1,12,11047.69049,8090.101009,10.00346709,18.22938741,18.14838725,0.838306773,7,1,
709,18.22938741,18.14838725,0.838306773,7,1,28.67660565,7.291754962,234.8123646,91.364999
709,18.22938741,18.14838725,0.838306773,7,1,28.67660565,7.291754962,234.8123646,91.364999
047.69049,8090.101009,10.00346709,18.22938741,18.14838725,0.838306773,7,1,28.67660565,7.2
047.69049,8090.101009,10.00346709,18.22938741,18.14838725,0.838306773,7,1,28.67660565,7.2
66,0,1,1,1,1,12,11047.69049,8090.101009,10.00346709,18.22938741,18.14838725,0.838306773,7,
346709,18.22938741,18.14838725,0.838306773,7,1,28.67660565,7.291754962,234.8123646,91.364
47.69049,8090.101009,10.00346709,18.22938741,18.14838725,0.838306773,7,1,28.67660565,7.29
47.69049,8090.101009,10.00346709,18.22938741,18.14838725,0.838306773,7,1,28.67660565,7.29
047.69049,8090.101009,10.00346709,18.22938741,18.14838725,0.838306773,7,1,28.67660565,7.2
709,18.22938741,18.14838725,0.838306773,7,1,28.67660565,7.291754962,234.8123646,91.364999
709,18.22938741,18.14838725,0.838306773,7,1,28.67660565,7.291754962,234.8123646,91.364999
047.69049,8090.101009,10.00346709,18.22938741,18.14838725,0.838306773,7,1,28.67660565,7.2
09,18.22938741,18.14838725,0.838306773,7,1,28.67660565,7.291754962,234.8123646,91.3649994
6,0,1,1,1,1,12,11047.69049,8090.101009,10.00346709,18.22938741,18.14838725,0.838306773,7,1,
047.69049,8090.101009,10.00346709,18.22938741,18.14838725,0.838306773,7,1,28.67660565,7.2
346709,18.22938741,18.14838725,0.838306773,7,1,28.67660565,7.291754962,234.8123646,91.364
66,0,1,1,1,1,12,11047.69049,8090.101009,10.00346709,18.22938741,18.14838725,0.838306773,7,
346709,18.22938741,18.14838725,0.838306773,7,1,28.67660565,7.291754962,234.8123646,91.364
047.69049,8090.101009,10.00346709,18.22938741,18.14838725,0.838306773,7,1,28.67660565,7.2
709,18.22938741,18.14838725,0.838306773,7,1,28.67660565,7.291754962,234.8123646,91.364999
47.69049,8090.101009,10.00346709,18.22938741,18.14838725,0.838306773,7,1,28.67660565,7.29

047.69049,8090.101009,10.00346709,18.22938741,18.14838725,0.838306773,7,1,28.67660565,7.2
346709,18.22938741,18.14838725,0.838306773,7,1,28.67660565,7.291754962,234.8123646,91.364
47.69049,8090.101009,10.00346709,18.22938741,18.14838725,0.838306773,7,1,28.67660565,7.29
.00346709,18.22938741,18.14838725,0.838306773,7,1,28.67660565,7.291754962,234.8123646,91.
047.69049,8090.101009,10.00346709,18.22938741,18.14838725,0.838306773,7,1,28.67660565,7.2
346709,18.22938741,18.14838725,0.838306773,7,1,28.67660565,7.291754962,234.8123646,91.364
6,0,1,1,1,1,12,11047.69049,8090.101009,10.00346709,18.22938741,18.14838725,0.838306773,7,1,
47.69049,8090.101009,10.00346709,18.22938741,18.14838725,0.838306773,7,1,28.67660565,7.29
95973,3.97685837,105.5820322,0.838306773,8,1,32.9038792,15.90743348,54.91907942,95.969647
932.06855,6902.306226,10.96795973,3.97685837,105.5820322,0.838306773,8,1,32.9038792,15.90
32.06855,6902.306226,10.96795973,3.97685837,105.5820322,0.838306773,8,1,32.9038792,15.907
8,0,1,0,1,1,12,10932.06855,6902.306226,10.96795973,3.97685837,105.5820322,0.838306773,8,1,3
32.06855,6902.306226,10.96795973,3.97685837,105.5820322,0.838306773,8,1,32.9038792,15.907
8,0,1,0,1,1,12,10932.06855,6902.306226,10.96795973,3.97685837,105.5820322,0.838306773,8,1,3
8,0,1,0,1,1,12,10932.06855,6902.306226,10.96795973,3.97685837,105.5820322,0.838306773,8,1,3
8,0,1,0,1,1,12,10932.06855,6902.306226,10.96795973,3.97685837,105.5820322,0.838306773,8,1,3
5795973,3.97685837,105.5820322,0.838306773,8,1,32.9038792,15.90743348,54.91907942,95.9696
932.06855,6902.306226,10.96795973,3.97685837,105.5820322,0.838306773,8,1,32.9038792,15.90
932.06855,6902.306226,10.96795973,3.97685837,105.5820322,0.838306773,8,1,32.9038792,15.90
932.06855,6902.306226,10.96795973,3.97685837,105.5820322,0.838306773,8,1,32.9038792,15.90
5973,3.97685837,105.5820322,0.838306773,8,1,32.9038792,15.90743348,54.91907942,95.9696476
973,3.97685837,105.5820322,0.838306773,8,1,32.9038792,15.90743348,54.91907942,95.96964766
973,3.97685837,105.5820322,0.838306773,8,1,32.9038792,15.90743348,54.91907942,95.96964766
932.06855,6902.306226,10.96795973,3.97685837,105.5820322,0.838306773,8,1,32.9038792,15.90
32.06855,6902.306226,10.96795973,3.97685837,105.5820322,0.838306773,8,1,32.9038792,15.907
932.06855,6902.306226,10.96795973,3.97685837,105.5820322,0.838306773,8,1,32.9038792,15.90
32.06855,6902.306226,10.96795973,3.97685837,105.5820322,0.838306773,8,1,32.9038792,15.907
32.06855,6902.306226,10.96795973,3.97685837,105.5820322,0.838306773,8,1,32.9038792,15.907
8,0,1,0,1,1,12,10932.06855,6902.306226,10.96795973,3.97685837,105.5820322,0.838306773,8,1,3
32.06855,6902.306226,10.96795973,3.97685837,105.5820322,0.838306773,8,1,32.9038792,15.907
795973,3.97685837,105.5820322,0.838306773,8,1,32.9038792,15.90743348,54.91907942,95.96964
0932.06855,6902.306226,10.96795973,3.97685837,105.5820322,0.838306773,8,1,32.9038792,15.9
8,0,1,0,1,1,12,10932.06855,6902.306226,10.96795973,3.97685837,105.5820322,0.838306773,8,1,3
73,3.97685837,105.5820322,0.838306773,8,1,32.9038792,15.90743348,54.91907942,95.96964766,8
932.06855,6902.306226,10.96795973,3.97685837,105.5820322,0.838306773,8,1,32.9038792,15.90
932.06855,6902.306226,10.96795973,3.97685837,105.5820322,0.838306773,8,1,32.9038792,15.90
973,3.97685837,105.5820322,0.838306773,8,1,32.9038792,15.90743348,54.91907942,95.96964766
8,0,1,0,1,1,12,10932.06855,6902.306226,10.96795973,3.97685837,105.5820322,0.838306773,8,1,3
8,0,1,0,1,1,12,10932.06855,6902.306226,10.96795973,3.97685837,105.5820322,0.838306773,8,1,3
973,3.97685837,105.5820322,0.838306773,8,1,32.9038792,15.90743348,54.91907942,95.96964766
932.06855,6902.306226,10.96795973,3.97685837,105.5820322,0.838306773,8,1,32.9038792,15.90
32.06855,6902.306226,10.96795973,3.97685837,105.5820322,0.838306773,8,1,32.9038792,15.907
795973,3.97685837,105.5820322,0.838306773,8,1,32.9038792,15.90743348,54.91907942,95.96964
5973,3.97685837,105.5820322,0.838306773,8,1,32.9038792,15.90743348,54.91907942,95.9696476
932.06855,6902.306226,10.96795973,3.97685837,105.5820322,0.838306773,8,1,32.9038792,15.90
0617676,3,2,206,73,1,1,0,1,1,11,11052.36828,6452.864273,11.06717302,8.160197173,105.5820322
.06717302,8.160197173,105.5820322,5.982691169,8,2,31.12642412,16.32039435,89.17318888,95.
06717302,8.160197173,105.5820322,5.982691169,8,2,31.12642412,16.32039435,89.17318888,95.4
052.36828,6452.864273,11.06717302,8.160197173,105.5820322,5.982691169,8,2,31.12642412,16.
717302,8.160197173,105.5820322,5.982691169,8,2,31.12642412,16.32039435,89.17318888,95.454

302,8.160197173,105.5820322,5.982691169,8,2,31.12642412,16.32039435,89.17318888,95.454367
052.36828,6452.864273,11.06717302,8.160197173,105.5820322,5.982691169,8,2,31.12642412,16.
02,8.160197173,105.5820322,5.982691169,8,2,31.12642412,16.32039435,89.17318888,95.4543673
717302,8.160197173,105.5820322,5.982691169,8,2,31.12642412,16.32039435,89.17318888,95.454
3,1,1,0,1,1,11,11052.36828,6452.864273,11.06717302,8.160197173,105.5820322,5.982691169,8,2,
302,8.160197173,105.5820322,5.982691169,8,2,31.12642412,16.32039435,89.17318888,95.454367
302,8.160197173,105.5820322,5.982691169,8,2,31.12642412,16.32039435,89.17318888,95.454367
052.36828,6452.864273,11.06717302,8.160197173,105.5820322,5.982691169,8,2,31.12642412,16.
302,8.160197173,105.5820322,5.982691169,8,2,31.12642412,16.32039435,89.17318888,95.454367
52.36828,6452.864273,11.06717302,8.160197173,105.5820322,5.982691169,8,2,31.12642412,16.3
52.36828,6452.864273,11.06717302,8.160197173,105.5820322,5.982691169,8,2,31.12642412,16.3
1052.36828,6452.864273,11.06717302,8.160197173,105.5820322,5.982691169,8,2,31.12642412,1
302,8.160197173,105.5820322,5.982691169,8,2,31.12642412,16.32039435,89.17318888,95.454367
1.06717302,8.160197173,105.5820322,5.982691169,8,2,31.12642412,16.32039435,89.17318888,95
3,1,1,0,1,1,11,11052.36828,6452.864273,11.06717302,8.160197173,105.5820322,5.982691169,8,2,
02,8.160197173,105.5820322,5.982691169,8,2,31.12642412,16.32039435,89.17318888,95.4543673
52.36828,6452.864273,11.06717302,8.160197173,105.5820322,5.982691169,8,2,31.12642412,16.3
717302,8.160197173,105.5820322,5.982691169,8,2,31.12642412,16.32039435,89.17318888,95.454
02,8.160197173,105.5820322,5.982691169,8,2,31.12642412,16.32039435,89.17318888,95.4543673
52.36828,6452.864273,11.06717302,8.160197173,105.5820322,5.982691169,8,2,31.12642412,16.3
52.36828,6452.864273,11.06717302,8.160197173,105.5820322,5.982691169,8,2,31.12642412,16.3
.06717302,8.160197173,105.5820322,5.982691169,8,2,31.12642412,16.32039435,89.17318888,95.
302,8.160197173,105.5820322,5.982691169,8,2,31.12642412,16.32039435,89.17318888,95.454367
052.36828,6452.864273,11.06717302,8.160197173,105.5820322,5.982691169,8,2,31.12642412,16.
3,1,1,0,1,1,11,11052.36828,6452.864273,11.06717302,8.160197173,105.5820322,5.982691169,8,2,
73,1,1,0,1,1,11,11052.36828,6452.864273,11.06717302,8.160197173,105.5820322,5.982691169,8,
717302,8.160197173,105.5820322,5.982691169,8,2,31.12642412,16.32039435,89.17318888,95.454
052.36828,6452.864273,11.06717302,8.160197173,105.5820322,5.982691169,8,2,31.12642412,16.
1052.36828,6452.864273,11.06717302,8.160197173,105.5820322,5.982691169,8,2,31.12642412,1
52.36828,6452.864273,11.06717302,8.160197173,105.5820322,5.982691169,8,2,31.12642412,16.3
52.36828,6452.864273,11.06717302,8.160197173,105.5820322,5.982691169,8,2,31.12642412,16.3
47,15.0314925,18.14838725,0.838306773,7,1,30.41354242,7.515746251,201.1025148,93.94405327
021.59726,7908.312506,10.13784747,15.0314925,18.14838725,0.838306773,7,1,30.41354242,7.51
21.59726,7908.312506,10.13784747,15.0314925,18.14838725,0.838306773,7,1,30.41354242,7.515
021.59726,7908.312506,10.13784747,15.0314925,18.14838725,0.838306773,7,1,30.41354242,7.51
747,15.0314925,18.14838725,0.838306773,7,1,30.41354242,7.515746251,201.1025148,93.9440532
34747,15.0314925,18.14838725,0.838306773,7,1,30.41354242,7.515746251,201.1025148,93.94405
0.13784747,15.0314925,18.14838725,0.838306773,7,1,30.41354242,7.515746251,201.1025148,93.9
1021.59726,7908.312506,10.13784747,15.0314925,18.14838725,0.838306773,7,1,30.41354242,7.5
021.59726,7908.312506,10.13784747,15.0314925,18.14838725,0.838306773,7,1,30.41354242,7.51
1021.59726,7908.312506,10.13784747,15.0314925,18.14838725,0.838306773,7,1,30.41354242,7.5
4747,15.0314925,18.14838725,0.838306773,7,1,30.41354242,7.515746251,201.1025148,93.944053
7,0,1,1,1,1,12,11021.59726,7908.312506,10.13784747,15.0314925,18.14838725,0.838306773,7,1,3
21.59726,7908.312506,10.13784747,15.0314925,18.14838725,0.838306773,7,1,30.41354242,7.515
7,0,1,1,1,1,12,11021.59726,7908.312506,10.13784747,15.0314925,18.14838725,0.838306773,7,1,3
7,0,1,1,1,1,12,11021.59726,7908.312506,10.13784747,15.0314925,18.14838725,0.838306773,7,1,3
13784747,15.0314925,18.14838725,0.838306773,7,1,30.41354242,7.515746251,201.1025148,93.94
21.59726,7908.312506,10.13784747,15.0314925,18.14838725,0.838306773,7,1,30.41354242,7.515
0.13784747,15.0314925,18.14838725,0.838306773,7,1,30.41354242,7.515746251,201.1025148,93.9
1021.59726,7908.312506,10.13784747,15.0314925,18.14838725,0.838306773,7,1,30.41354242,7.5

747,15.0314925,18.14838725,0.838306773,7,1,30.41354242,7.515746251,201.1025148,93.9440532
784747,15.0314925,18.14838725,0.838306773,7,1,30.41354242,7.515746251,201.1025148,93.9440
7,0,1,1,1,1,12,11021.59726,7908.312506,10.13784747,15.0314925,18.14838725,0.838306773,7,1,3
1021.59726,7908.312506,10.13784747,15.0314925,18.14838725,0.838306773,7,1,30.41354242,7.5
13784747,15.0314925,18.14838725,0.838306773,7,1,30.41354242,7.515746251,201.1025148,93.94
,11021.59726,7908.312506,10.13784747,15.0314925,18.14838725,0.838306773,7,1,30.41354242,7
7,0,1,1,1,1,12,11021.59726,7908.312506,10.13784747,15.0314925,18.14838725,0.838306773,7,1,3
1021.59726,7908.312506,10.13784747,15.0314925,18.14838725,0.838306773,7,1,30.41354242,7.5
67,0,1,1,1,1,12,11021.59726,7908.312506,10.13784747,15.0314925,18.14838725,0.838306773,7,1,
21.59726,7908.312506,10.13784747,15.0314925,18.14838725,0.838306773,7,1,30.41354242,7.515
744,17.33518797,18.14838725,0.838306773,7,1,26.88455923,13.86815037,261.0988264,90.895414
1237.29409,8012.061644,8.961519744,17.33518797,18.14838725,0.838306773,7,1,26.88455923,13
9744,17.33518797,18.14838725,0.838306773,7,1,26.88455923,13.86815037,261.0988264,90.89541
44,17.33518797,18.14838725,0.838306773,7,1,26.88455923,13.86815037,261.0988264,90.8954145
237.29409,8012.061644,8.961519744,17.33518797,18.14838725,0.838306773,7,1,26.88455923,13.
6,0,1,1,1,1,11,11237.29409,8012.061644,8.961519744,17.33518797,18.14838725,0.838306773,7,1,
6,0,1,1,1,1,11,11237.29409,8012.061644,8.961519744,17.33518797,18.14838725,0.838306773,7,1,
19744,17.33518797,18.14838725,0.838306773,7,1,26.88455923,13.86815037,261.0988264,90.8954
37.29409,8012.061644,8.961519744,17.33518797,18.14838725,0.838306773,7,1,26.88455923,13.8
237.29409,8012.061644,8.961519744,17.33518797,18.14838725,0.838306773,7,1,26.88455923,13.
237.29409,8012.061644,8.961519744,17.33518797,18.14838725,0.838306773,7,1,26.88455923,13.
519744,17.33518797,18.14838725,0.838306773,7,1,26.88455923,13.86815037,261.0988264,90.895
6,0,1,1,1,1,11,11237.29409,8012.061644,8.961519744,17.33518797,18.14838725,0.838306773,7,1,
37.29409,8012.061644,8.961519744,17.33518797,18.14838725,0.838306773,7,1,26.88455923,13.8
237.29409,8012.061644,8.961519744,17.33518797,18.14838725,0.838306773,7,1,26.88455923,13.
519744,17.33518797,18.14838725,0.838306773,7,1,26.88455923,13.86815037,261.0988264,90.895
519744,17.33518797,18.14838725,0.838306773,7,1,26.88455923,13.86815037,261.0988264,90.895
519744,17.33518797,18.14838725,0.838306773,7,1,26.88455923,13.86815037,261.0988264,90.895
519744,17.33518797,18.14838725,0.838306773,7,1,26.88455923,13.86815037,261.0988264,90.895
1237.29409,8012.061644,8.961519744,17.33518797,18.14838725,0.838306773,7,1,26.88455923,13
1009,11.15369087,18.5278243,0.838306773,8,1,32.1318734,7.435793912,114.0261071,91.6100220
9,11.15369087,18.5278243,0.838306773,8,1,32.1318734,7.435793912,114.0261071,91.61002203,63
9,1,1,1,1,1,12,11113.2294,8146.685575,10.93851009,11.15369087,18.5278243,0.838306773,8,1,32
113.2294,8146.685575,10.93851009,11.15369087,18.5278243,0.838306773,8,1,32.1318734,7.4357
13.2294,8146.685575,10.93851009,11.15369087,18.5278243,0.838306773,8,1,32.1318734,7.43579
51009,11.15369087,18.5278243,0.838306773,8,1,32.1318734,7.435793912,114.0261071,91.610022
9,11.15369087,18.5278243,0.838306773,8,1,32.1318734,7.435793912,114.0261071,91.61002203,63
51009,11.15369087,18.5278243,0.838306773,8,1,32.1318734,7.435793912,114.0261071,91.610022
9,1,1,1,1,1,12,11113.2294,8146.685575,10.93851009,11.15369087,18.5278243,0.838306773,8,1,32
1113.2294,8146.685575,10.93851009,11.15369087,18.5278243,0.838306773,8,1,32.1318734,7.435
13.2294,8146.685575,10.93851009,11.15369087,18.5278243,0.838306773,8,1,32.1318734,7.43579
517676,3,2,206,69,1,1,1,1,1,12,11113.2294,8146.685575,10.93851009,11.15369087,18.5278243,0.8
1113.2294,8146.685575,10.93851009,11.15369087,18.5278243,0.838306773,8,1,32.1318734,7.4357
1.15369087,18.5278243,0.838306773,8,1,32.1318734,7.435793912,114.0261071,91.61002203,63.2
0617676,3,2,206,69,1,1,1,1,1,12,11113.2294,8146.685575,10.93851009,11.15369087,18.5278243,0.
113.2294,8146.685575,10.93851009,11.15369087,18.5278243,0.838306773,8,1,32.1318734,7.4357
113.2294,8146.685575,10.93851009,11.15369087,18.5278243,0.838306773,8,1,32.1318734,7.4357
51009,11.15369087,18.5278243,0.838306773,8,1,32.1318734,7.435793912,114.0261071,91.610022
51009,11.15369087,18.5278243,0.838306773,8,1,32.1318734,7.435793912,114.0261071,91.610022
046,3.183522097,18.14838725,0.838306773,7,1,30.15636138,6.367044193,292.2531463,92.144437

4,0,1,0,1,1,11,9447.379463,7203.209823,10.05212046,3.183522097,18.14838725,0.838306773,7,1,
7.379463,7203.209823,10.05212046,3.183522097,18.14838725,0.838306773,7,1,30.15636138,6.36
4,0,1,0,1,1,11,9447.379463,7203.209823,10.05212046,3.183522097,18.14838725,0.838306773,7,1,
7.379463,7203.209823,10.05212046,3.183522097,18.14838725,0.838306773,7,1,30.15636138,6.36
517676,3,2,206,74,0,1,0,1,1,11,9447.379463,7203.209823,10.05212046,3.183522097,18.14838725,
05212046,3.183522097,18.14838725,0.838306773,7,1,30.15636138,6.367044193,292.2531463,92.1
046,3.183522097,18.14838725,0.838306773,7,1,30.15636138,6.367044193,292.2531463,92.144437
47.379463,7203.209823,10.05212046,3.183522097,18.14838725,0.838306773,7,1,30.15636138,6.3
.05212046,3.183522097,18.14838725,0.838306773,7,1,30.15636138,6.367044193,292.2531463,92.
4,0,1,0,1,1,11,9447.379463,7203.209823,10.05212046,3.183522097,18.14838725,0.838306773,7,1,
47.379463,7203.209823,10.05212046,3.183522097,18.14838725,0.838306773,7,1,30.15636138,6.3
47.379463,7203.209823,10.05212046,3.183522097,18.14838725,0.838306773,7,1,30.15636138,6.3
2046,3.183522097,18.14838725,0.838306773,7,1,30.15636138,6.367044193,292.2531463,92.14443
47.379463,7203.209823,10.05212046,3.183522097,18.14838725,0.838306773,7,1,30.15636138,6.3
05212046,3.183522097,18.14838725,0.838306773,7,1,30.15636138,6.367044193,292.2531463,92.1
4,0,1,0,1,1,11,9447.379463,7203.209823,10.05212046,3.183522097,18.14838725,0.838306773,7,1,
212046,3.183522097,18.14838725,0.838306773,7,1,30.15636138,6.367044193,292.2531463,92.144
47.379463,7203.209823,10.05212046,3.183522097,18.14838725,0.838306773,7,1,30.15636138,6.3
4,0,1,0,1,1,11,9447.379463,7203.209823,10.05212046,3.183522097,18.14838725,0.838306773,7,1,
47.379463,7203.209823,10.05212046,3.183522097,18.14838725,0.838306773,7,1,30.15636138,6.3
05212046,3.183522097,18.14838725,0.838306773,7,1,30.15636138,6.367044193,292.2531463,92.1
46,3.183522097,18.14838725,0.838306773,7,1,30.15636138,6.367044193,292.2531463,92.1444375
4,0,1,0,1,1,11,9447.379463,7203.209823,10.05212046,3.183522097,18.14838725,0.838306773,7,1,
47.379463,7203.209823,10.05212046,3.183522097,18.14838725,0.838306773,7,1,30.15636138,6.3
4,0,1,0,1,1,11,9447.379463,7203.209823,10.05212046,3.183522097,18.14838725,0.838306773,7,1,
4,0,1,0,1,1,11,9447.379463,7203.209823,10.05212046,3.183522097,18.14838725,0.838306773,7,1,
7.379463,7203.209823,10.05212046,3.183522097,18.14838725,0.838306773,7,1,30.15636138,6.36
4,0,1,0,1,1,11,9447.379463,7203.209823,10.05212046,3.183522097,18.14838725,0.838306773,7,1,
046,3.183522097,18.14838725,0.838306773,7,1,30.15636138,6.367044193,292.2531463,92.144437
,12.08331668,29.39298478,19.91707628,5,3,30.98245248,40.27772228,227.1793737,105.7337664
71.981,8368.769594,4.9178496,12.08331668,29.39298478,19.91707628,5,3,30.98245248,40.27772
71,0,1,0,1,1,10,10471.981,8368.769594,4.9178496,12.08331668,29.39298478,19.91707628,5,3,30.
71.981,8368.769594,4.9178496,12.08331668,29.39298478,19.91707628,5,3,30.98245248,40.27772
71.981,8368.769594,4.9178496,12.08331668,29.39298478,19.91707628,5,3,30.98245248,40.27772
71,0,1,0,1,1,10,10471.981,8368.769594,4.9178496,12.08331668,29.39298478,19.91707628,5,3,30.
496,12.08331668,29.39298478,19.91707628,5,3,30.98245248,40.27772228,227.1793737,105.7337
2.08331668,29.39298478,19.91707628,5,3,30.98245248,40.27772228,227.1793737,105.7337664,1
71.981,8368.769594,4.9178496,12.08331668,29.39298478,19.91707628,5,3,30.98245248,40.27772
331668,29.39298478,19.91707628,5,3,30.98245248,40.27772228,227.1793737,105.7337664,104.7
82324,3,2,216,71,0,1,0,1,1,10,10471.981,8368.769594,4.9178496,12.08331668,29.39298478,19.91
71,0,1,0,1,1,10,10471.981,8368.769594,4.9178496,12.08331668,29.39298478,19.91707628,5,3,30.
0471.981,8368.769594,4.9178496,12.08331668,29.39298478,19.91707628,5,3,30.98245248,40.277
2.08331668,29.39298478,19.91707628,5,3,30.98245248,40.27772228,227.1793737,105.7337664,1
9382324,3,2,216,71,0,1,0,1,1,10,10471.981,8368.769594,4.9178496,12.08331668,29.39298478,19.9
496,12.08331668,29.39298478,19.91707628,5,3,30.98245248,40.27772228,227.1793737,105.7337
1,0,1,0,1,1,10,10471.981,8368.769594,4.9178496,12.08331668,29.39298478,19.91707628,5,3,30.9
71.981,8368.769594,4.9178496,12.08331668,29.39298478,19.91707628,5,3,30.98245248,40.27772
6,12.08331668,29.39298478,19.91707628,5,3,30.98245248,40.27772228,227.1793737,105.733766
1,0,1,0,1,1,10,10471.981,8368.769594,4.9178496,12.08331668,29.39298478,19.91707628,5,3,30.9
471.981,8368.769594,4.9178496,12.08331668,29.39298478,19.91707628,5,3,30.98245248,40.2777

0471.981,8368.769594,4.9178496,12.08331668,29.39298478,19.91707628,5,3,30.98245248,40.277
96,12.08331668,29.39298478,19.91707628,5,3,30.98245248,40.27772228,227.1793737,105.733766
603857,7.817043319,32.94084014,4.132277098,6,2,31.82867025,27.35965162,163.8718626,98.012
857,7.817043319,32.94084014,4.132277098,6,2,31.82867025,27.35965162,163.8718626,98.012095
062603857,7.817043319,32.94084014,4.132277098,6,2,31.82867025,27.35965162,163.8718626,98
57,7.817043319,32.94084014,4.132277098,6,2,31.82867025,27.35965162,163.8718626,98.0120956
62603857,7.817043319,32.94084014,4.132277098,6,2,31.82867025,27.35965162,163.8718626,98.0
55.35641,7919.353612,6.062603857,7.817043319,32.94084014,4.132277098,6,2,31.82867025,27.3
857,7.817043319,32.94084014,4.132277098,6,2,31.82867025,27.35965162,163.8718626,98.012095
71,1,1,0,1,1,12,10255.35641,7919.353612,6.062603857,7.817043319,32.94084014,4.132277098,6,
71,1,1,0,1,1,12,10255.35641,7919.353612,6.062603857,7.817043319,32.94084014,4.132277098,6,
1,1,1,0,1,1,12,10255.35641,7919.353612,6.062603857,7.817043319,32.94084014,4.132277098,6,2,
10255.35641,7919.353612,6.062603857,7.817043319,32.94084014,4.132277098,6,2,31.82867025,2
7.817043319,32.94084014,4.132277098,6,2,31.82867025,27.35965162,163.8718626,98.01209569,
,1,1,0,1,1,12,10255.35641,7919.353612,6.062603857,7.817043319,32.94084014,4.132277098,6,2,3
55.35641,7919.353612,6.062603857,7.817043319,32.94084014,4.132277098,6,2,31.82867025,27.3
0255.35641,7919.353612,6.062603857,7.817043319,32.94084014,4.132277098,6,2,31.82867025,2
857,7.817043319,32.94084014,4.132277098,6,2,31.82867025,27.35965162,163.8718626,98.012095
1,1,1,0,1,1,12,10255.35641,7919.353612,6.062603857,7.817043319,32.94084014,4.132277098,6,2,
857,7.817043319,32.94084014,4.132277098,6,2,31.82867025,27.35965162,163.8718626,98.012095
10255.35641,7919.353612,6.062603857,7.817043319,32.94084014,4.132277098,6,2,31.82867025,2
062603857,7.817043319,32.94084014,4.132277098,6,2,31.82867025,27.35965162,163.8718626,98
603857,7.817043319,32.94084014,4.132277098,6,2,31.82867025,27.35965162,163.8718626,98.012
76,7.587064008,32.94084014,4.132277098,6,2,30.7743746,26.55472403,186.7852366,96.8636053,
25.09005,8010.835743,6.558473276,7.587064008,32.94084014,4.132277098,6,2,30.7743746,26.55
125.09005,8010.835743,6.558473276,7.587064008,32.94084014,4.132277098,6,2,30.7743746,26.5
276,7.587064008,32.94084014,4.132277098,6,2,30.7743746,26.55472403,186.7852366,96.8636053
9,0,1,0,1,1,12,10125.09005,8010.835743,6.558473276,7.587064008,32.94084014,4.132277098,6,2,
125.09005,8010.835743,6.558473276,7.587064008,32.94084014,4.132277098,6,2,30.7743746,26.5
25.09005,8010.835743,6.558473276,7.587064008,32.94084014,4.132277098,6,2,30.7743746,26.55
0125.09005,8010.835743,6.558473276,7.587064008,32.94084014,4.132277098,6,2,30.7743746,26.
25.09005,8010.835743,6.558473276,7.587064008,32.94084014,4.132277098,6,2,30.7743746,26.55
276,7.587064008,32.94084014,4.132277098,6,2,30.7743746,26.55472403,186.7852366,96.8636053
125.09005,8010.835743,6.558473276,7.587064008,32.94084014,4.132277098,6,2,30.7743746,26.5
,0,1,0,1,1,12,10125.09005,8010.835743,6.558473276,7.587064008,32.94084014,4.132277098,6,2,3
125.09005,8010.835743,6.558473276,7.587064008,32.94084014,4.132277098,6,2,30.7743746,26.5
125.09005,8010.835743,6.558473276,7.587064008,32.94084014,4.132277098,6,2,30.7743746,26.5
125.09005,8010.835743,6.558473276,7.587064008,32.94084014,4.132277098,6,2,30.7743746,26.5
9,0,1,0,1,1,12,10125.09005,8010.835743,6.558473276,7.587064008,32.94084014,4.132277098,6,2,
125.09005,8010.835743,6.558473276,7.587064008,32.94084014,4.132277098,6,2,30.7743746,26.5
69,0,1,0,1,1,12,10125.09005,8010.835743,6.558473276,7.587064008,32.94084014,4.132277098,6,
9,0,1,0,1,1,12,10125.09005,8010.835743,6.558473276,7.587064008,32.94084014,4.132277098,6,2,
9,0,1,0,1,1,12,10125.09005,8010.835743,6.558473276,7.587064008,32.94084014,4.132277098,6,2,
125.09005,8010.835743,6.558473276,7.587064008,32.94084014,4.132277098,6,2,30.7743746,26.5
69,0,1,0,1,1,12,10125.09005,8010.835743,6.558473276,7.587064008,32.94084014,4.132277098,6,
9,0,1,0,1,1,12,10125.09005,8010.835743,6.558473276,7.587064008,32.94084014,4.132277098,6,2,
,0,1,0,1,1,12,10125.09005,8010.835743,6.558473276,7.587064008,32.94084014,4.132277098,6,2,3
0125.09005,8010.835743,6.558473276,7.587064008,32.94084014,4.132277098,6,2,30.7743746,26.
276,7.587064008,32.94084014,4.132277098,6,2,30.7743746,26.55472403,186.7852366,96.8636053
25.09005,8010.835743,6.558473276,7.587064008,32.94084014,4.132277098,6,2,30.7743746,26.55

76,7.587064008,32.94084014,4.132277098,6,2,30.7743746,26.55472403,186.7852366,96.8636053,
9,0,1,0,1,1,12,10125.09005,8010.835743,6.558473276,7.587064008,32.94084014,4.132277098,6,2,
76,7.587064008,32.94084014,4.132277098,6,2,30.7743746,26.55472403,186.7852366,96.8636053,
25.09005,8010.835743,6.558473276,7.587064008,32.94084014,4.132277098,6,2,30.7743746,26.55
9,0,1,0,1,1,12,10125.09005,8010.835743,6.558473276,7.587064008,32.94084014,4.132277098,6,2,
25.09005,8010.835743,6.558473276,7.587064008,32.94084014,4.132277098,6,2,30.7743746,26.55
0125.09005,8010.835743,6.558473276,7.587064008,32.94084014,4.132277098,6,2,30.7743746,26.
125.09005,8010.835743,6.558473276,7.587064008,32.94084014,4.132277098,6,2,30.7743746,26.5
,7.587064008,32.94084014,4.132277098,6,2,30.7743746,26.55472403,186.7852366,96.8636053,1
125.09005,8010.835743,6.558473276,7.587064008,32.94084014,4.132277098,6,2,30.7743746,26.5
125.09005,8010.835743,6.558473276,7.587064008,32.94084014,4.132277098,6,2,30.7743746,26.5
9,0,1,0,1,1,12,10125.09005,8010.835743,6.558473276,7.587064008,32.94084014,4.132277098,6,2,
,0,1,0,1,1,12,10125.09005,8010.835743,6.558473276,7.587064008,32.94084014,4.132277098,6,2,3
125.09005,8010.835743,6.558473276,7.587064008,32.94084014,4.132277098,6,2,30.7743746,26.5
276,7.587064008,32.94084014,4.132277098,6,2,30.7743746,26.55472403,186.7852366,96.8636053
9,0,1,0,1,1,12,10125.09005,8010.835743,6.558473276,7.587064008,32.94084014,4.132277098,6,2,
25.09005,8010.835743,6.558473276,7.587064008,32.94084014,4.132277098,6,2,30.7743746,26.55
9,0,1,0,1,1,12,10125.09005,8010.835743,6.558473276,7.587064008,32.94084014,4.132277098,6,2,
19,8.83804263,29.39298478,4.132277098,5,2,31.28737546,22.09510657,269.8908966,106.3770766
83.68808,8008.09681,4.693106319,8.83804263,29.39298478,4.132277098,5,2,31.28737546,22.095
383.68808,8008.09681,4.693106319,8.83804263,29.39298478,4.132277098,5,2,31.28737546,22.09
383.68808,8008.09681,4.693106319,8.83804263,29.39298478,4.132277098,5,2,31.28737546,22.09
83.68808,8008.09681,4.693106319,8.83804263,29.39298478,4.132277098,5,2,31.28737546,22.095
,78,1,1,0,1,1,11,10383.68808,8008.09681,4.693106319,8.83804263,29.39298478,4.132277098,5,2,
383.68808,8008.09681,4.693106319,8.83804263,29.39298478,4.132277098,5,2,31.28737546,22.09
0383.68808,8008.09681,4.693106319,8.83804263,29.39298478,4.132277098,5,2,31.28737546,22.0
383.68808,8008.09681,4.693106319,8.83804263,29.39298478,4.132277098,5,2,31.28737546,22.09
383.68808,8008.09681,4.693106319,8.83804263,29.39298478,4.132277098,5,2,31.28737546,22.09
8,1,1,0,1,1,11,10383.68808,8008.09681,4.693106319,8.83804263,29.39298478,4.132277098,5,2,31
383.68808,8008.09681,4.693106319,8.83804263,29.39298478,4.132277098,5,2,31.28737546,22.09
383.68808,8008.09681,4.693106319,8.83804263,29.39298478,4.132277098,5,2,31.28737546,22.09
383.68808,8008.09681,4.693106319,8.83804263,29.39298478,4.132277098,5,2,31.28737546,22.09
0383.68808,8008.09681,4.693106319,8.83804263,29.39298478,4.132277098,5,2,31.28737546,22.0
591162,3,2,216,78,1,1,0,1,1,11,10383.68808,8008.09681,4.693106319,8.83804263,29.39298478,4.1
383.68808,8008.09681,4.693106319,8.83804263,29.39298478,4.132277098,5,2,31.28737546,22.09
383.68808,8008.09681,4.693106319,8.83804263,29.39298478,4.132277098,5,2,31.28737546,22.09
19,8.83804263,29.39298478,4.132277098,5,2,31.28737546,22.09510657,269.8908966,106.3770766
8,1,1,0,1,1,11,10383.68808,8008.09681,4.693106319,8.83804263,29.39298478,4.132277098,5,2,31
,1,1,0,1,1,11,10383.68808,8008.09681,4.693106319,8.83804263,29.39298478,4.132277098,5,2,31.
8,1,1,0,1,1,11,10383.68808,8008.09681,4.693106319,8.83804263,29.39298478,4.132277098,5,2,31
8,1,1,0,1,1,11,10383.68808,8008.09681,4.693106319,8.83804263,29.39298478,4.132277098,5,2,31
83.68808,8008.09681,4.693106319,8.83804263,29.39298478,4.132277098,5,2,31.28737546,22.095
19,8.83804263,29.39298478,4.132277098,5,2,31.28737546,22.09510657,269.8908966,106.3770766
8,1,1,0,1,1,11,10383.68808,8008.09681,4.693106319,8.83804263,29.39298478,4.132277098,5,2,31
78,1,1,0,1,1,11,10383.68808,8008.09681,4.693106319,8.83804263,29.39298478,4.132277098,5,2,3
8,1,1,0,1,1,11,10383.68808,8008.09681,4.693106319,8.83804263,29.39298478,4.132277098,5,2,31
83.68808,8008.09681,4.693106319,8.83804263,29.39298478,4.132277098,5,2,31.28737546,22.095
78,1,1,0,1,1,11,10383.68808,8008.09681,4.693106319,8.83804263,29.39298478,4.132277098,5,2,3
3.68808,8008.09681,4.693106319,8.83804263,29.39298478,4.132277098,5,2,31.28737546,22.0951
83.68808,8008.09681,4.693106319,8.83804263,29.39298478,4.132277098,5,2,31.28737546,22.095

19,8.83804263,29.39298478,4.132277098,5,2,31.28737546,22.09510657,269.8908966,106.3770766
78,1,1,0,1,1,11,10383.68808,8008.09681,4.693106319,8.83804263,29.39298478,4.132277098,5,2,31
0308838,3,2,216,78,1,1,0,1,1,11,10383.68808,8008.09681,4.693106319,8.83804263,29.39298478,4.
3.68808,8008.09681,4.693106319,8.83804263,29.39298478,4.132277098,5,2,31.28737546,22.0951
383.68808,8008.09681,4.693106319,8.83804263,29.39298478,4.132277098,5,2,31.28737546,22.09
0383.68808,8008.09681,4.693106319,8.83804263,29.39298478,4.132277098,5,2,31.28737546,22.0
383.68808,8008.09681,4.693106319,8.83804263,29.39298478,4.132277098,5,2,31.28737546,22.09
19,8.83804263,29.39298478,4.132277098,5,2,31.28737546,22.09510657,269.8908966,106.3770766
383.68808,8008.09681,4.693106319,8.83804263,29.39298478,4.132277098,5,2,31.28737546,22.09
19,8.83804263,29.39298478,4.132277098,5,2,31.28737546,22.09510657,269.8908966,106.3770766
78,1,1,0,1,1,11,10383.68808,8008.09681,4.693106319,8.83804263,29.39298478,4.132277098,5,2,31
78,1,1,0,1,1,11,10383.68808,8008.09681,4.693106319,8.83804263,29.39298478,4.132277098,5,2,31
3.318114,5769.177813,5.458318323,7.168945896,28.76111656,7.573324183,5,1,21.83327329,7.16
3.318114,5769.177813,5.458318323,7.168945896,28.76111656,7.573324183,5,1,21.83327329,7.16
73.318114,5769.177813,5.458318323,7.168945896,28.76111656,7.573324183,5,1,21.83327329,7.1
318323,7.168945896,28.76111656,7.573324183,5,1,21.83327329,7.168945896,257.0870829,85.513
23,7.168945896,28.76111656,7.573324183,5,1,21.83327329,7.168945896,257.0870829,85.5136537
23,7.168945896,28.76111656,7.573324183,5,1,21.83327329,7.168945896,257.0870829,85.5136537
323,7.168945896,28.76111656,7.573324183,5,1,21.83327329,7.168945896,257.0870829,85.513653
458318323,7.168945896,28.76111656,7.573324183,5,1,21.83327329,7.168945896,257.0870829,85
7.168945896,28.76111656,7.573324183,5,1,21.83327329,7.168945896,257.0870829,85.51365372,7
6973.318114,5769.177813,5.458318323,7.168945896,28.76111656,7.573324183,5,1,21.83327329,7
973.318114,5769.177813,5.458318323,7.168945896,28.76111656,7.573324183,5,1,21.83327329,7.
73.318114,5769.177813,5.458318323,7.168945896,28.76111656,7.573324183,5,1,21.83327329,7.1
73.318114,5769.177813,5.458318323,7.168945896,28.76111656,7.573324183,5,1,21.83327329,7.1
323,7.168945896,28.76111656,7.573324183,5,1,21.83327329,7.168945896,257.0870829,85.513653
73.318114,5769.177813,5.458318323,7.168945896,28.76111656,7.573324183,5,1,21.83327329,7.1
5973.318114,5769.177813,5.458318323,7.168945896,28.76111656,7.573324183,5,1,21.83327329,7
458318323,7.168945896,28.76111656,7.573324183,5,1,21.83327329,7.168945896,257.0870829,85.
3.318114,5769.177813,5.458318323,7.168945896,28.76111656,7.573324183,5,1,21.83327329,7.16
323,7.168945896,28.76111656,7.573324183,5,1,21.83327329,7.168945896,257.0870829,85.513653
9,0,1,0,1,1,11,6973.318114,5769.177813,5.458318323,7.168945896,28.76111656,7.573324183,5,1,
3.318114,5769.177813,5.458318323,7.168945896,28.76111656,7.573324183,5,1,21.83327329,7.16
73.318114,5769.177813,5.458318323,7.168945896,28.76111656,7.573324183,5,1,21.83327329,7.1
3.318114,5769.177813,5.458318323,7.168945896,28.76111656,7.573324183,5,1,21.83327329,7.16
3,7.168945896,28.76111656,7.573324183,5,1,21.83327329,7.168945896,257.0870829,85.51365372
73.318114,5769.177813,5.458318323,7.168945896,28.76111656,7.573324183,5,1,21.83327329,7.1
973.318114,5769.177813,5.458318323,7.168945896,28.76111656,7.573324183,5,1,21.83327329,7.
3.318114,5769.177813,5.458318323,7.168945896,28.76111656,7.573324183,5,1,21.83327329,7.16
9,0,1,0,1,1,11,6973.318114,5769.177813,5.458318323,7.168945896,28.76111656,7.573324183,5,1,
3,7.168945896,28.76111656,7.573324183,5,1,21.83327329,7.168945896,257.0870829,85.51365372
3.318114,5769.177813,5.458318323,7.168945896,28.76111656,7.573324183,5,1,21.83327329,7.16
73.318114,5769.177813,5.458318323,7.168945896,28.76111656,7.573324183,5,1,21.83327329,7.1
9,1,1,0,1,1,12,6973.318114,5769.177813,5.458318323,7.168945896,28.76111656,7.573324183,5,1,
973.318114,5769.177813,5.458318323,7.168945896,28.76111656,7.573324183,5,1,21.83327329,7.
9,1,1,0,1,1,12,6973.318114,5769.177813,5.458318323,7.168945896,28.76111656,7.573324183,5,1,
458318323,7.168945896,28.76111656,7.573324183,5,1,21.83327329,7.168945896,257.0870829,85
323,7.168945896,28.76111656,7.573324183,5,1,21.83327329,7.168945896,257.0870829,85.513653
458318323,7.168945896,28.76111656,7.573324183,5,1,21.83327329,7.168945896,257.0870829,85
23,7.168945896,28.76111656,7.573324183,5,1,21.83327329,7.168945896,257.0870829,85.5136537

13,5.458318323,7.168945896,28.76111656,7.573324183,5,1,21.83327329,7.168945896,257.087082
73.318114,5769.177813,5.458318323,7.168945896,28.76111656,7.573324183,5,1,21.83327329,7.1
3323,7.168945896,28.76111656,7.573324183,5,1,21.83327329,7.168945896,257.0870829,85.51365
9,1,1,0,1,1,12,6973.318114,5769.177813,5.458318323,7.168945896,28.76111656,7.573324183,5,1,
9,1,1,0,1,1,12,6973.318114,5769.177813,5.458318323,7.168945896,28.76111656,7.573324183,5,1,
69,1,1,0,1,1,12,6973.318114,5769.177813,5.458318323,7.168945896,28.76111656,7.573324183,5,
9,1,1,0,1,1,12,6973.318114,5769.177813,5.458318323,7.168945896,28.76111656,7.573324183,5,1,
73.318114,5769.177813,5.458318323,7.168945896,28.76111656,7.573324183,5,1,21.83327329,7.1
23,7.168945896,28.76111656,7.573324183,5,1,21.83327329,7.168945896,257.0870829,85.5136537
73.318114,5769.177813,5.458318323,7.168945896,28.76111656,7.573324183,5,1,21.83327329,7.1
973.318114,5769.177813,5.458318323,7.168945896,28.76111656,7.573324183,5,1,21.83327329,7.
73.318114,5769.177813,5.458318323,7.168945896,28.76111656,7.573324183,5,1,21.83327329,7.1
973.318114,5769.177813,5.458318323,7.168945896,28.76111656,7.573324183,5,1,21.83327329,7.
323,7.168945896,28.76111656,7.573324183,5,1,21.83327329,7.168945896,257.0870829,85.513653
981.848715,6792.212536,5.541889635,9.96804764,46.44850006,11.15261014,7,2,24.68659928,19.
981.848715,6792.212536,5.541889635,9.96804764,46.44850006,11.15261014,7,2,24.68659928,19.
0,0,1,0,1,1,12,8981.848715,6792.212536,5.541889635,9.96804764,46.44850006,11.15261014,7,2,2
1.848715,6792.212536,5.541889635,9.96804764,46.44850006,11.15261014,7,2,24.68659928,19.93
382324,3,2,216,70,0,1,0,1,1,12,8981.848715,6792.212536,5.541889635,9.96804764,46.44850006,1
81.848715,6792.212536,5.541889635,9.96804764,46.44850006,11.15261014,7,2,24.68659928,19.9
1.848715,6792.212536,5.541889635,9.96804764,46.44850006,11.15261014,7,2,24.68659928,19.93
0,0,1,0,1,1,12,8981.848715,6792.212536,5.541889635,9.96804764,46.44850006,11.15261014,7,2,2
,0,1,0,1,1,12,8981.848715,6792.212536,5.541889635,9.96804764,46.44850006,11.15261014,7,2,24
1.848715,6792.212536,5.541889635,9.96804764,46.44850006,11.15261014,7,2,24.68659928,19.93
1.848715,6792.212536,5.541889635,9.96804764,46.44850006,11.15261014,7,2,24.68659928,19.93
0,0,1,0,1,1,12,8981.848715,6792.212536,5.541889635,9.96804764,46.44850006,11.15261014,7,2,2
0,0,1,0,1,1,12,8981.848715,6792.212536,5.541889635,9.96804764,46.44850006,11.15261014,7,2,2
0,0,1,0,1,1,12,8981.848715,6792.212536,5.541889635,9.96804764,46.44850006,11.15261014,7,2,2
81.848715,6792.212536,5.541889635,9.96804764,46.44850006,11.15261014,7,2,24.68659928,19.9
0,0,1,0,1,1,12,8981.848715,6792.212536,5.541889635,9.96804764,46.44850006,11.15261014,7,2,2
81.848715,6792.212536,5.541889635,9.96804764,46.44850006,11.15261014,7,2,24.68659928,19.9
1.848715,6792.212536,5.541889635,9.96804764,46.44850006,11.15261014,7,2,24.68659928,19.93
81.848715,6792.212536,5.541889635,9.96804764,46.44850006,11.15261014,7,2,24.68659928,19.9
81.848715,6792.212536,5.541889635,9.96804764,46.44850006,11.15261014,7,2,24.68659928,19.9
535,9.96804764,46.44850006,11.15261014,7,2,24.68659928,19.93609528,61.8142828,90.18165861
981.848715,6792.212536,5.541889635,9.96804764,46.44850006,11.15261014,7,2,24.68659928,19.
81.848715,6792.212536,5.541889635,9.96804764,46.44850006,11.15261014,7,2,24.68659928,19.9
0,0,1,0,1,1,12,8981.848715,6792.212536,5.541889635,9.96804764,46.44850006,11.15261014,7,2,2
81.848715,6792.212536,5.541889635,9.96804764,46.44850006,11.15261014,7,2,24.68659928,19.9
81.848715,6792.212536,5.541889635,9.96804764,46.44850006,11.15261014,7,2,24.68659928,19.9
1.848715,6792.212536,5.541889635,9.96804764,46.44850006,11.15261014,7,2,24.68659928,19.93
0,0,1,0,1,1,12,8981.848715,6792.212536,5.541889635,9.96804764,46.44850006,11.15261014,7,2,2
81.848715,6792.212536,5.541889635,9.96804764,46.44850006,11.15261014,7,2,24.68659928,19.9
81.848715,6792.212536,5.541889635,9.96804764,46.44850006,11.15261014,7,2,24.68659928,19.9
36,10.84100596,29.39298478,19.91707628,5,3,28.29778918,28.90934923,8.794031042,103.598685
4,0,1,0,1,1,12,10401.12111,12174.39974,6.714729636,10.84100596,29.39298478,19.91707628,5,3,
729636,10.84100596,29.39298478,19.91707628,5,3,28.29778918,28.90934923,8.794031042,103.59
4,0,1,0,1,1,12,10401.12111,12174.39974,6.714729636,10.84100596,29.39298478,19.91707628,5,3,
401.12111,12174.39974,6.714729636,10.84100596,29.39298478,19.91707628,5,3,28.29778918,28.
01.12111,12174.39974,6.714729636,10.84100596,29.39298478,19.91707628,5,3,28.29778918,28.9

4,0,1,0,1,1,12,10401.12111,12174.39974,6.714729636,10.84100596,29.39298478,19.91707628,5,3,
401.12111,12174.39974,6.714729636,10.84100596,29.39298478,19.91707628,5,3,28.29778918,28.
01.12111,12174.39974,6.714729636,10.84100596,29.39298478,19.91707628,5,3,28.29778918,28.9
36,10.84100596,29.39298478,19.91707628,5,3,28.29778918,28.90934923,8.794031042,103.598685
536,10.84100596,29.39298478,19.91707628,5,3,28.29778918,28.90934923,8.794031042,103.59868
536,10.84100596,29.39298478,19.91707628,5,3,28.29778918,28.90934923,8.794031042,103.59868
10401.12111,12174.39974,6.714729636,10.84100596,29.39298478,19.91707628,5,3,28.29778918,2
714729636,10.84100596,29.39298478,19.91707628,5,3,28.29778918,28.90934923,8.794031042,10
714729636,10.84100596,29.39298478,19.91707628,5,3,28.29778918,28.90934923,8.794031042,10
0401.12111,12174.39974,6.714729636,10.84100596,29.39298478,19.91707628,5,3,28.29778918,2
401.12111,12174.39974,6.714729636,10.84100596,29.39298478,19.91707628,5,3,28.29778918,28.
4,0,1,0,1,1,12,10401.12111,12174.39974,6.714729636,10.84100596,29.39298478,19.91707628,5,3,
729636,10.84100596,29.39298478,19.91707628,5,3,28.29778918,28.90934923,8.794031042,103.59
401.12111,12174.39974,6.714729636,10.84100596,29.39298478,19.91707628,5,3,28.29778918,28.
536,10.84100596,29.39298478,19.91707628,5,3,28.29778918,28.90934923,8.794031042,103.59868
0401.12111,12174.39974,6.714729636,10.84100596,29.39298478,19.91707628,5,3,28.29778918,2
36,10.84100596,29.39298478,19.91707628,5,3,28.29778918,28.90934923,8.794031042,103.598685
.714729636,10.84100596,29.39298478,19.91707628,5,3,28.29778918,28.90934923,8.794031042,1
01.12111,12174.39974,6.714729636,10.84100596,29.39298478,19.91707628,5,3,28.29778918,28.9
4,0,1,0,1,1,12,10401.12111,12174.39974,6.714729636,10.84100596,29.39298478,19.91707628,5,3,
01.12111,12174.39974,6.714729636,10.84100596,29.39298478,19.91707628,5,3,28.29778918,28.9
36,10.84100596,29.39298478,19.91707628,5,3,28.29778918,28.90934923,8.794031042,103.598685
729636,10.84100596,29.39298478,19.91707628,5,3,28.29778918,28.90934923,8.794031042,103.59
3,1,1,0,1,1,12,10401.12111,12174.39974,6.714729636,10.84100596,29.39298478,19.91707628,5,3,
3,1,1,0,1,1,12,10401.12111,12174.39974,6.714729636,10.84100596,29.39298478,19.91707628,5,3,
3,1,1,0,1,1,12,10401.12111,12174.39974,6.714729636,10.84100596,29.39298478,19.91707628,5,3,
73,1,1,0,1,1,12,10401.12111,12174.39974,6.714729636,10.84100596,29.39298478,19.91707628,5,
29636,10.84100596,29.39298478,19.91707628,5,3,28.29778918,28.90934923,8.794031042,103.598
73,1,1,0,1,1,12,10401.12111,12174.39974,6.714729636,10.84100596,29.39298478,19.91707628,5,
401.12111,12174.39974,6.714729636,10.84100596,29.39298478,19.91707628,5,3,28.29778918,28.
3,1,1,0,1,1,12,10401.12111,12174.39974,6.714729636,10.84100596,29.39298478,19.91707628,5,3,
10401.12111,12174.39974,6.714729636,10.84100596,29.39298478,19.91707628,5,3,28.29778918,2
714729636,10.84100596,29.39298478,19.91707628,5,3,28.29778918,28.90934923,8.794031042,10
73,1,1,0,1,1,12,10401.12111,12174.39974,6.714729636,10.84100596,29.39298478,19.91707628,5,
401.12111,12174.39974,6.714729636,10.84100596,29.39298478,19.91707628,5,3,28.29778918,28.
,73,1,1,0,1,1,12,10401.12111,12174.39974,6.714729636,10.84100596,29.39298478,19.91707628,5,
729636,10.84100596,29.39298478,19.91707628,5,3,28.29778918,28.90934923,8.794031042,103.59
01.12111,12174.39974,6.714729636,10.84100596,29.39298478,19.91707628,5,3,28.29778918,28.9
0401.12111,12174.39974,6.714729636,10.84100596,29.39298478,19.91707628,5,3,28.29778918,2
01.12111,12174.39974,6.714729636,10.84100596,29.39298478,19.91707628,5,3,28.29778918,28.9
363606,8.724005515,28.76111656,7.573324183,5,1,22.13462615,4.362002757,370.2604664,88.538
0,1,1,0,1,1,11,7243.419952,4348.015304,6.225363606,8.724005515,28.76111656,7.573324183,5,1,
506,8.724005515,28.76111656,7.573324183,5,1,22.13462615,4.362002757,370.2604664,88.538504
3.419952,4348.015304,6.225363606,8.724005515,28.76111656,7.573324183,5,1,22.13462615,4.36
506,8.724005515,28.76111656,7.573324183,5,1,22.13462615,4.362002757,370.2604664,88.538504
225363606,8.724005515,28.76111656,7.573324183,5,1,22.13462615,4.362002757,370.2604664,88
243.419952,4348.015304,6.225363606,8.724005515,28.76111656,7.573324183,5,1,22.13462615,4.
506,8.724005515,28.76111656,7.573324183,5,1,22.13462615,4.362002757,370.2604664,88.538504
243.419952,4348.015304,6.225363606,8.724005515,28.76111656,7.573324183,5,1,22.13462615,4.
06,8.724005515,28.76111656,7.573324183,5,1,22.13462615,4.362002757,370.2604664,88.5385046

43.419952,4348.015304,6.225363606,8.724005515,28.76111656,7.573324183,5,1,22.13462615,4.3
243.419952,4348.015304,6.225363606,8.724005515,28.76111656,7.573324183,5,1,22.13462615,4.
0,1,1,0,1,1,11,7243.419952,4348.015304,6.225363606,8.724005515,28.76111656,7.573324183,5,1,
0,1,1,0,1,1,11,7243.419952,4348.015304,6.225363606,8.724005515,28.76111656,7.573324183,5,1,
,1,1,0,1,1,11,7243.419952,4348.015304,6.225363606,8.724005515,28.76111656,7.573324183,5,1,2
43.419952,4348.015304,6.225363606,8.724005515,28.76111656,7.573324183,5,1,22.13462615,4.3
506,8.724005515,28.76111656,7.573324183,5,1,22.13462615,4.362002757,370.2604664,88.538504
6.225363606,8.724005515,28.76111656,7.573324183,5,1,22.13462615,4.362002757,370.2604664,8
506,8.724005515,28.76111656,7.573324183,5,1,22.13462615,4.362002757,370.2604664,88.538504
506,8.724005515,28.76111656,7.573324183,5,1,22.13462615,4.362002757,370.2604664,88.538504
43.419952,4348.015304,6.225363606,8.724005515,28.76111656,7.573324183,5,1,22.13462615,4.3
3.419952,4348.015304,6.225363606,8.724005515,28.76111656,7.573324183,5,1,22.13462615,4.36
0,1,1,0,1,1,11,7243.419952,4348.015304,6.225363606,8.724005515,28.76111656,7.573324183,5,1,
506,8.724005515,28.76111656,7.573324183,5,1,22.13462615,4.362002757,370.2604664,88.538504
363606,8.724005515,28.76111656,7.573324183,5,1,22.13462615,4.362002757,370.2604664,88.538
70,1,1,0,1,1,11,7243.419952,4348.015304,6.225363606,8.724005515,28.76111656,7.573324183,5,
43.419952,4348.015304,6.225363606,8.724005515,28.76111656,7.573324183,5,1,22.13462615,4.3
3.419952,4348.015304,6.225363606,8.724005515,28.76111656,7.573324183,5,1,22.13462615,4.36
3.419952,4348.015304,6.225363606,8.724005515,28.76111656,7.573324183,5,1,22.13462615,4.36
43.419952,4348.015304,6.225363606,8.724005515,28.76111656,7.573324183,5,1,22.13462615,4.3
3.419952,4348.015304,6.225363606,8.724005515,28.76111656,7.573324183,5,1,22.13462615,4.36
0,1,1,0,1,1,11,7243.419952,4348.015304,6.225363606,8.724005515,28.76111656,7.573324183,5,1,
5,8.104738141,28.76111656,7.573324183,5,1,18.80419039,0,406.1274115,84.29464658,72.942643
5,8.104738141,28.76111656,7.573324183,5,1,18.80419039,0,406.1274115,84.29464658,72.942643
5,8.104738141,28.76111656,7.573324183,5,1,18.80419039,0,406.1274115,84.29464658,72.942643
5,8.104738141,28.76111656,7.573324183,5,1,18.80419039,0,406.1274115,84.29464658,72.942643
5,8.104738141,28.76111656,7.573324183,5,1,18.80419039,0,406.1274115,84.29464658,72.942643
9.02886,5483.54693,5.835783225,8.104738141,28.76111656,7.573324183,5,1,18.80419039,0,406.1
2,0,0,0,1,1,12,7129.02886,5483.54693,5.835783225,8.104738141,28.76111656,7.573324183,5,1,18
2,0,0,0,1,1,12,7129.02886,5483.54693,5.835783225,8.104738141,28.76111656,7.573324183,5,1,18
3225,8.104738141,28.76111656,7.573324183,5,1,18.80419039,0,406.1274115,84.29464658,72.942
5,8.104738141,28.76111656,7.573324183,5,1,18.80419039,0,406.1274115,84.29464658,72.942643
5783225,8.104738141,28.76111656,7.573324183,5,1,18.80419039,0,406.1274115,84.29464658,72.
370605,3,2,216,72,0,0,0,1,1,12,7129.02886,5483.54693,5.835783225,8.104738141,28.76111656,7.5
9.02886,5483.54693,5.835783225,8.104738141,28.76111656,7.573324183,5,1,18.80419039,0,406.1
2,0,0,0,1,1,12,7129.02886,5483.54693,5.835783225,8.104738141,28.76111656,7.573324183,5,1,18
225,8.104738141,28.76111656,7.573324183,5,1,18.80419039,0,406.1274115,84.29464658,72.9426
5783225,8.104738141,28.76111656,7.573324183,5,1,18.80419039,0,406.1274115,84.29464658,72.
2,0,0,0,1,1,12,7129.02886,5483.54693,5.835783225,8.104738141,28.76111656,7.573324183,5,1,18
5783225,8.104738141,28.76111656,7.573324183,5,1,18.80419039,0,406.1274115,84.29464658,72.
5,8.104738141,28.76111656,7.573324183,5,1,18.80419039,0,406.1274115,84.29464658,72.942643
35783225,8.104738141,28.76111656,7.573324183,5,1,18.80419039,0,406.1274115,84.29464658,7
5,8.104738141,28.76111656,7.573324183,5,1,18.80419039,0,406.1274115,84.29464658,72.942643
9.02886,5483.54693,5.835783225,8.104738141,28.76111656,7.573324183,5,1,18.80419039,0,406.1
72,0,0,0,1,1,12,7129.02886,5483.54693,5.835783225,8.104738141,28.76111656,7.573324183,5,1,1
225,8.104738141,28.76111656,7.573324183,5,1,18.80419039,0,406.1274115,84.29464658,72.9426
72,0,0,0,1,1,12,7129.02886,5483.54693,5.835783225,8.104738141,28.76111656,7.573324183,5,1,1
72,0,0,0,1,1,12,7129.02886,5483.54693,5.835783225,8.104738141,28.76111656,7.573324183,5,1,1
5783225,8.104738141,28.76111656,7.573324183,5,1,18.80419039,0,406.1274115,84.29464658,72.9
104738141,28.76111656,7.573324183,5,1,18.80419039,0,406.1274115,84.29464658,72.94264327,3

5,8.104738141,28.76111656,7.573324183,5,1,18.80419039,0,406.1274115,84.29464658,72.9426432
29.02886,5483.54693,5.835783225,8.104738141,28.76111656,7.573324183,5,1,18.80419039,0,406
8,1,0,0,1,1,12,7129.02886,5483.54693,5.835783225,8.104738141,28.76111656,7.573324183,5,1,18
9.02886,5483.54693,5.835783225,8.104738141,28.76111656,7.573324183,5,1,18.80419039,0,406.1
.104738141,28.76111656,7.573324183,5,1,18.80419039,0,406.1274115,84.29464658,72.94264327,
3225,8.104738141,28.76111656,7.573324183,5,1,18.80419039,0,406.1274115,84.29464658,72.9426
29.02886,5483.54693,5.835783225,8.104738141,28.76111656,7.573324183,5,1,18.80419039,0,406
29.02886,5483.54693,5.835783225,8.104738141,28.76111656,7.573324183,5,1,18.80419039,0,406
29.02886,5483.54693,5.835783225,8.104738141,28.76111656,7.573324183,5,1,18.80419039,0,406
3225,8.104738141,28.76111656,7.573324183,5,1,18.80419039,0,406.1274115,84.29464658,72.9426
29.02886,5483.54693,5.835783225,8.104738141,28.76111656,7.573324183,5,1,18.80419039,0,406
9.02886,5483.54693,5.835783225,8.104738141,28.76111656,7.573324183,5,1,18.80419039,0,406.1
8,1,0,0,1,1,12,7129.02886,5483.54693,5.835783225,8.104738141,28.76111656,7.573324183,5,1,18
29.02886,5483.54693,5.835783225,8.104738141,28.76111656,7.573324183,5,1,18.80419039,0,406
104738141,28.76111656,7.573324183,5,1,18.80419039,0,406.1274115,84.29464658,72.94264327,3
9.02886,5483.54693,5.835783225,8.104738141,28.76111656,7.573324183,5,1,18.80419039,0,406.1
5,8.104738141,28.76111656,7.573324183,5,1,18.80419039,0,406.1274115,84.29464658,72.9426432
29.02886,5483.54693,5.835783225,8.104738141,28.76111656,7.573324183,5,1,18.80419039,0,406
8,1,0,0,1,1,12,7129.02886,5483.54693,5.835783225,8.104738141,28.76111656,7.573324183,5,1,18
9.02886,5483.54693,5.835783225,8.104738141,28.76111656,7.573324183,5,1,18.80419039,0,406.1
29.02886,5483.54693,5.835783225,8.104738141,28.76111656,7.573324183,5,1,18.80419039,0,406
5783225,8.104738141,28.76111656,7.573324183,5,1,18.80419039,0,406.1274115,84.29464658,72.9
3225,8.104738141,28.76111656,7.573324183,5,1,18.80419039,0,406.1274115,84.29464658,72.9426
225,8.104738141,28.76111656,7.573324183,5,1,18.80419039,0,406.1274115,84.29464658,72.94264
3225,8.104738141,28.76111656,7.573324183,5,1,18.80419039,0,406.1274115,84.29464658,72.9426
5,8.104738141,28.76111656,7.573324183,5,1,18.80419039,0,406.1274115,84.29464658,72.9426432
29.02886,5483.54693,5.835783225,8.104738141,28.76111656,7.573324183,5,1,18.80419039,0,406
68,1,0,0,1,1,12,7129.02886,5483.54693,5.835783225,8.104738141,28.76111656,7.573324183,5,1,1
5,8.104738141,28.76111656,7.573324183,5,1,18.80419039,0,406.1274115,84.29464658,72.9426432
5,8.104738141,28.76111656,7.573324183,5,1,18.80419039,0,406.1274115,84.29464658,72.9426432
225,8.104738141,28.76111656,7.573324183,5,1,18.80419039,0,406.1274115,84.29464658,72.94264
8,1,0,0,1,1,12,7129.02886,5483.54693,5.835783225,8.104738141,28.76111656,7.573324183,5,1,18
5,8.104738141,28.76111656,7.573324183,5,1,18.80419039,0,406.1274115,84.29464658,72.9426432
9.02886,5483.54693,5.835783225,8.104738141,28.76111656,7.573324183,5,1,18.80419039,0,406.1
907,12.55709636,28.20812542,7.573324183,4,1,23.52260693,0,465.0830518,83.2338399,25.114192
1.496661,4023.456929,5.428293907,12.55709636,28.20812542,7.573324183,4,1,23.52260693,0,46
71,1,1,0,1,1,12,7091.496661,4023.456929,5.428293907,12.55709636,28.20812542,7.573324183,4,1
91.496661,4023.456929,5.428293907,12.55709636,28.20812542,7.573324183,4,1,23.52260693,0,4
91.496661,4023.456929,5.428293907,12.55709636,28.20812542,7.573324183,4,1,23.52260693,0,4
,1,1,0,1,1,12,7091.496661,4023.456929,5.428293907,12.55709636,28.20812542,7.573324183,4,1,2
91.496661,4023.456929,5.428293907,12.55709636,28.20812542,7.573324183,4,1,23.52260693,0,4
71,1,1,0,1,1,12,7091.496661,4023.456929,5.428293907,12.55709636,28.20812542,7.573324183,4,1
91.496661,4023.456929,5.428293907,12.55709636,28.20812542,7.573324183,4,1,23.52260693,0,4
1.496661,4023.456929,5.428293907,12.55709636,28.20812542,7.573324183,4,1,23.52260693,0,46
0154419,3,2,216,71,1,1,0,1,1,12,7091.496661,4023.456929,5.428293907,12.55709636,28.20812542
3907,12.55709636,28.20812542,7.573324183,4,1,23.52260693,0,465.0830518,83.2338399,25.11419
91.496661,4023.456929,5.428293907,12.55709636,28.20812542,7.573324183,4,1,23.52260693,0,4
1,1,1,0,1,1,12,7091.496661,4023.456929,5.428293907,12.55709636,28.20812542,7.573324183,4,1,
091.496661,4023.456929,5.428293907,12.55709636,28.20812542,7.573324183,4,1,23.52260693,0,
1,1,1,0,1,1,12,7091.496661,4023.456929,5.428293907,12.55709636,28.20812542,7.573324183,4,1,

91.496661,4023.456929,5.428293907,12.55709636,28.20812542,7.573324183,4,1,23.52260693,0,4
091.496661,4023.456929,5.428293907,12.55709636,28.20812542,7.573324183,4,1,23.52260693,0,
907,12.55709636,28.20812542,7.573324183,4,1,23.52260693,0,465.0830518,83.2338399,25.11419
91.496661,4023.456929,5.428293907,12.55709636,28.20812542,7.573324183,4,1,23.52260693,0,4
091.496661,4023.456929,5.428293907,12.55709636,28.20812542,7.573324183,4,1,23.52260693,0,
91.496661,4023.456929,5.428293907,12.55709636,28.20812542,7.573324183,4,1,23.52260693,0,4
1,1,1,0,1,1,12,7091.496661,4023.456929,5.428293907,12.55709636,28.20812542,7.573324183,4,1,
1.496661,4023.456929,5.428293907,12.55709636,28.20812542,7.573324183,4,1,23.52260693,0,46
3907,12.55709636,28.20812542,7.573324183,4,1,23.52260693,0,465.0830518,83.2338399,25.1141
07,12.55709636,28.20812542,7.573324183,4,1,23.52260693,0,465.0830518,83.2338399,25.114192
1,1,1,0,1,1,12,7091.496661,4023.456929,5.428293907,12.55709636,28.20812542,7.573324183,4,1,
1.496661,4023.456929,5.428293907,12.55709636,28.20812542,7.573324183,4,1,23.52260693,0,46
091.496661,4023.456929,5.428293907,12.55709636,28.20812542,7.573324183,4,1,23.52260693,0,
091.496661,4023.456929,5.428293907,12.55709636,28.20812542,7.573324183,4,1,23.52260693,0,
1544189,3,2,216,71,1,1,0,1,1,12,7091.496661,4023.456929,5.428293907,12.55709636,28.20812542
1,1,1,0,1,1,12,7091.496661,4023.456929,5.428293907,12.55709636,28.20812542,7.573324183,4,1,
91.496661,4023.456929,5.428293907,12.55709636,28.20812542,7.573324183,4,1,23.52260693,0,4
1,1,1,0,1,1,12,7091.496661,4023.456929,5.428293907,12.55709636,28.20812542,7.573324183,4,1,
91.496661,4023.456929,5.428293907,12.55709636,28.20812542,7.573324183,4,1,23.52260693,0,4
506,8.724005515,28.76111656,7.573324183,5,1,22.13462615,4.362002757,370.2604664,88.538504
43.419952,4348.015304,6.225363606,8.724005515,28.76111656,7.573324183,5,1,22.13462615,4.3
25363606,8.724005515,28.76111656,7.573324183,5,1,22.13462615,4.362002757,370.2604664,88.5
69,0,1,0,1,1,11,7243.419952,4348.015304,6.225363606,8.724005515,28.76111656,7.573324183,5,
3.419952,4348.015304,6.225363606,8.724005515,28.76111656,7.573324183,5,1,22.13462615,4.36
363606,8.724005515,28.76111656,7.573324183,5,1,22.13462615,4.362002757,370.2604664,88.538
243.419952,4348.015304,6.225363606,8.724005515,28.76111656,7.573324183,5,1,22.13462615,4.
43.419952,4348.015304,6.225363606,8.724005515,28.76111656,7.573324183,5,1,22.13462615,4.3
225363606,8.724005515,28.76111656,7.573324183,5,1,22.13462615,4.362002757,370.2604664,88
06,8.724005515,28.76111656,7.573324183,5,1,22.13462615,4.362002757,370.2604664,88.5385046
506,8.724005515,28.76111656,7.573324183,5,1,22.13462615,4.362002757,370.2604664,88.538504
43.419952,4348.015304,6.225363606,8.724005515,28.76111656,7.573324183,5,1,22.13462615,4.3
506,8.724005515,28.76111656,7.573324183,5,1,22.13462615,4.362002757,370.2604664,88.538504
43.419952,4348.015304,6.225363606,8.724005515,28.76111656,7.573324183,5,1,22.13462615,4.3
25363606,8.724005515,28.76111656,7.573324183,5,1,22.13462615,4.362002757,370.2604664,88.5
25363606,8.724005515,28.76111656,7.573324183,5,1,22.13462615,4.362002757,370.2604664,88.
3.419952,4348.015304,6.225363606,8.724005515,28.76111656,7.573324183,5,1,22.13462615,4.36
43.419952,4348.015304,6.225363606,8.724005515,28.76111656,7.573324183,5,1,22.13462615,4.3
3.419952,4348.015304,6.225363606,8.724005515,28.76111656,7.573324183,5,1,22.13462615,4.36
43.419952,4348.015304,6.225363606,8.724005515,28.76111656,7.573324183,5,1,22.13462615,4.3
243.419952,4348.015304,6.225363606,8.724005515,28.76111656,7.573324183,5,1,22.13462615,4.
25363606,8.724005515,28.76111656,7.573324183,5,1,22.13462615,4.362002757,370.2604664,88.5
3.419952,4348.015304,6.225363606,8.724005515,28.76111656,7.573324183,5,1,22.13462615,4.36
243.419952,4348.015304,6.225363606,8.724005515,28.76111656,7.573324183,5,1,22.13462615,4.
506,8.724005515,28.76111656,7.573324183,5,1,22.13462615,4.362002757,370.2604664,88.538504
43.419952,4348.015304,6.225363606,8.724005515,28.76111656,7.573324183,5,1,22.13462615,4.3
382324,3,2,216,69,0,1,0,1,1,11,7243.419952,4348.015304,6.225363606,8.724005515,28.76111656,
506,8.724005515,28.76111656,7.573324183,5,1,22.13462615,4.362002757,370.2604664,88.538504
506,8.724005515,28.76111656,7.573324183,5,1,22.13462615,4.362002757,370.2604664,88.538504
06,8.724005515,28.76111656,7.573324183,5,1,22.13462615,4.362002757,370.2604664,88.5385046
9,0,1,0,1,1,11,7243.419952,4348.015304,6.225363606,8.724005515,28.76111656,7.573324183,5,1,

3.419952,4348.015304,6.225363606,8.724005515,28.76111656,7.573324183,5,1,22.13462615,4.36
243.419952,4348.015304,6.225363606,8.724005515,28.76111656,7.573324183,5,1,22.13462615,4.
3.419952,4348.015304,6.225363606,8.724005515,28.76111656,7.573324183,5,1,22.13462615,4.36
84.239553,5642.713735,4.768430145,7.879167063,42.9006447,3.579285958,6,1,24.3719763,7.879
3,1,1,1,1,1,12,9684.239553,5642.713735,4.768430145,7.879167063,42.9006447,3.579285958,6,1,2
73,1,1,1,1,1,12,9684.239553,5642.713735,4.768430145,7.879167063,42.9006447,3.579285958,6,1,
4.239553,5642.713735,4.768430145,7.879167063,42.9006447,3.579285958,6,1,24.3719763,7.8791
0145,7.879167063,42.9006447,3.579285958,6,1,24.3719763,7.879167063,334.4098598,99.6072074
3,1,1,1,1,1,12,9684.239553,5642.713735,4.768430145,7.879167063,42.9006447,3.579285958,6,1,2
73,1,1,1,1,1,12,9684.239553,5642.713735,4.768430145,7.879167063,42.9006447,3.579285958,6,1,
84.239553,5642.713735,4.768430145,7.879167063,42.9006447,3.579285958,6,1,24.3719763,7.879
145,7.879167063,42.9006447,3.579285958,6,1,24.3719763,7.879167063,334.4098598,99.60720747
430145,7.879167063,42.9006447,3.579285958,6,1,24.3719763,7.879167063,334.4098598,99.60720
5,7.879167063,42.9006447,3.579285958,6,1,24.3719763,7.879167063,334.4098598,99.60720747,1
4.239553,5642.713735,4.768430145,7.879167063,42.9006447,3.579285958,6,1,24.3719763,7.8791
4.239553,5642.713735,4.768430145,7.879167063,42.9006447,3.579285958,6,1,24.3719763,7.8791
768430145,7.879167063,42.9006447,3.579285958,6,1,24.3719763,7.879167063,334.4098598,99.60
3,1,1,1,1,1,12,9684.239553,5642.713735,4.768430145,7.879167063,42.9006447,3.579285958,6,1,2
45,7.879167063,42.9006447,3.579285958,6,1,24.3719763,7.879167063,334.4098598,99.60720747,1
4.239553,5642.713735,4.768430145,7.879167063,42.9006447,3.579285958,6,1,24.3719763,7.8791
9684.239553,5642.713735,4.768430145,7.879167063,42.9006447,3.579285958,6,1,24.3719763,7.87
0145,7.879167063,42.9006447,3.579285958,6,1,24.3719763,7.879167063,334.4098598,99.6072074
430145,7.879167063,42.9006447,3.579285958,6,1,24.3719763,7.879167063,334.4098598,99.60720
.168761,7176.567982,6.779973524,3.678924666,46.44850006,3.579285958,7,1,31.81372192,18.39
2.168761,7176.567982,6.779973524,3.678924666,46.44850006,3.579285958,7,1,31.81372192,18.3
.168761,7176.567982,6.779973524,3.678924666,46.44850006,3.579285958,7,1,31.81372192,18.39
75,1,1,0,1,1,7,9332.168761,7176.567982,6.779973524,3.678924666,46.44850006,3.579285958,7,1,
168761,7176.567982,6.779973524,3.678924666,46.44850006,3.579285958,7,1,31.81372192,18.394
75,1,1,0,1,1,7,9332.168761,7176.567982,6.779973524,3.678924666,46.44850006,3.579285958,7,1,
5,1,1,0,1,1,7,9332.168761,7176.567982,6.779973524,3.678924666,46.44850006,3.579285958,7,1,3
2.168761,7176.567982,6.779973524,3.678924666,46.44850006,3.579285958,7,1,31.81372192,18.3
.168761,7176.567982,6.779973524,3.678924666,46.44850006,3.579285958,7,1,31.81372192,18.39
,1,1,0,1,1,7,9332.168761,7176.567982,6.779973524,3.678924666,46.44850006,3.579285958,7,1,31
.168761,7176.567982,6.779973524,3.678924666,46.44850006,3.579285958,7,1,31.81372192,18.39
32.168761,7176.567982,6.779973524,3.678924666,46.44850006,3.579285958,7,1,31.81372192,18.
.168761,7176.567982,6.779973524,3.678924666,46.44850006,3.579285958,7,1,31.81372192,18.39
2.168761,7176.567982,6.779973524,3.678924666,46.44850006,3.579285958,7,1,31.81372192,18.3
168761,7176.567982,6.779973524,3.678924666,46.44850006,3.579285958,7,1,31.81372192,18.394
32.168761,7176.567982,6.779973524,3.678924666,46.44850006,3.579285958,7,1,31.81372192,18.
32.168761,7176.567982,6.779973524,3.678924666,46.44850006,3.579285958,7,1,31.81372192,18.
2.168761,7176.567982,6.779973524,3.678924666,46.44850006,3.579285958,7,1,31.81372192,18.3
.168761,7176.567982,6.779973524,3.678924666,46.44850006,3.579285958,7,1,31.81372192,18.39
.168761,7176.567982,6.779973524,3.678924666,46.44850006,3.579285958,7,1,31.81372192,18.39
.168761,7176.567982,6.779973524,3.678924666,46.44850006,3.579285958,7,1,31.81372192,18.39
24,3.678924666,46.44850006,3.579285958,7,1,31.81372192,18.39462333,26.2155059,88.66119224
.168761,7176.567982,6.779973524,3.678924666,46.44850006,3.579285958,7,1,31.81372192,18.39
,1,1,0,1,1,7,9332.168761,7176.567982,6.779973524,3.678924666,46.44850006,3.579285958,7,1,31
.168761,7176.567982,6.779973524,3.678924666,46.44850006,3.579285958,7,1,31.81372192,18.39
5,1,1,0,1,1,7,9332.168761,7176.567982,6.779973524,3.678924666,46.44850006,3.579285958,7,1,3
.168761,7176.567982,6.779973524,3.678924666,46.44850006,3.579285958,7,1,31.81372192,18.39

0617676,3,2,216,75,1,1,0,1,1,7,9332.168761,7176.567982,6.779973524,3.678924666,46.44850006,3
.168761,7176.567982,6.779973524,3.678924666,46.44850006,3.579285958,7,1,31.81372192,18.39
75,1,1,0,1,1,7,9332.168761,7176.567982,6.779973524,3.678924666,46.44850006,3.579285958,7,1,
.168761,7176.567982,6.779973524,3.678924666,46.44850006,3.579285958,7,1,31.81372192,18.39
973524,3.678924666,46.44850006,3.579285958,7,1,31.81372192,18.39462333,26.2155059,88.6611
8,1,0,1,1,1,11,9332.168761,7176.567982,6.779973524,3.678924666,46.44850006,3.579285958,7,1,
973524,3.678924666,46.44850006,3.579285958,7,1,31.81372192,18.39462333,26.2155059,88.6611
3524,3.678924666,46.44850006,3.579285958,7,1,31.81372192,18.39462333,26.2155059,88.661192
68,1,0,1,1,1,11,9332.168761,7176.567982,6.779973524,3.678924666,46.44850006,3.579285958,7,1
779973524,3.678924666,46.44850006,3.579285958,7,1,31.81372192,18.39462333,26.2155059,88.6
32.168761,7176.567982,6.779973524,3.678924666,46.44850006,3.579285958,7,1,31.81372192,18.
779973524,3.678924666,46.44850006,3.579285958,7,1,31.81372192,18.39462333,26.2155059,88.
32.168761,7176.567982,6.779973524,3.678924666,46.44850006,3.579285958,7,1,31.81372192,18.
32.168761,7176.567982,6.779973524,3.678924666,46.44850006,3.579285958,7,1,31.81372192,18.
973524,3.678924666,46.44850006,3.579285958,7,1,31.81372192,18.39462333,26.2155059,88.6611
332.168761,7176.567982,6.779973524,3.678924666,46.44850006,3.579285958,7,1,31.81372192,18
32.168761,7176.567982,6.779973524,3.678924666,46.44850006,3.579285958,7,1,31.81372192,18.
79973524,3.678924666,46.44850006,3.579285958,7,1,31.81372192,18.39462333,26.2155059,88.66
2.168761,7176.567982,6.779973524,3.678924666,46.44850006,3.579285958,7,1,31.81372192,18.3
332.168761,7176.567982,6.779973524,3.678924666,46.44850006,3.579285958,7,1,31.81372192,18
8,1,0,1,1,1,11,9332.168761,7176.567982,6.779973524,3.678924666,46.44850006,3.579285958,7,1,
32.168761,7176.567982,6.779973524,3.678924666,46.44850006,3.579285958,7,1,31.81372192,18.
8,1,0,1,1,1,11,9332.168761,7176.567982,6.779973524,3.678924666,46.44850006,3.579285958,7,1,
524,3.678924666,46.44850006,3.579285958,7,1,31.81372192,18.39462333,26.2155059,88.6611922
32.168761,7176.567982,6.779973524,3.678924666,46.44850006,3.579285958,7,1,31.81372192,18.
73524,3.678924666,46.44850006,3.579285958,7,1,31.81372192,18.39462333,26.2155059,88.66119
2.168761,7176.567982,6.779973524,3.678924666,46.44850006,3.579285958,7,1,31.81372192,18.3
779973524,3.678924666,46.44850006,3.579285958,7,1,31.81372192,18.39462333,26.2155059,88.
779973524,3.678924666,46.44850006,3.579285958,7,1,31.81372192,18.39462333,26.2155059,88.6
8,1,0,1,1,1,11,9332.168761,7176.567982,6.779973524,3.678924666,46.44850006,3.579285958,7,1,
24,3.678924666,46.44850006,3.579285958,7,1,31.81372192,18.39462333,26.2155059,88.66119224
332.168761,7176.567982,6.779973524,3.678924666,46.44850006,3.579285958,7,1,31.81372192,18
524,3.678924666,46.44850006,3.579285958,7,1,31.81372192,18.39462333,26.2155059,88.6611922
8,1,0,1,1,1,11,9332.168761,7176.567982,6.779973524,3.678924666,46.44850006,3.579285958,7,1,
973524,3.678924666,46.44850006,3.579285958,7,1,31.81372192,18.39462333,26.2155059,88.6611
9973524,3.678924666,46.44850006,3.579285958,7,1,31.81372192,18.39462333,26.2155059,88.661
2.168761,7176.567982,6.779973524,3.678924666,46.44850006,3.579285958,7,1,31.81372192,18.3
8,1,0,1,1,1,11,9332.168761,7176.567982,6.779973524,3.678924666,46.44850006,3.579285958,7,1,
524,3.678924666,46.44850006,3.579285958,7,1,31.81372192,18.39462333,26.2155059,88.6611922
,1,0,1,1,1,11,9332.168761,7176.567982,6.779973524,3.678924666,46.44850006,3.579285958,7,1,3
973524,3.678924666,46.44850006,3.579285958,7,1,31.81372192,18.39462333,26.2155059,88.6611
32.168761,7176.567982,6.779973524,3.678924666,46.44850006,3.579285958,7,1,31.81372192,18.
32.168761,7176.567982,6.779973524,3.678924666,46.44850006,3.579285958,7,1,31.81372192,18.
9382324,3,2,216,68,1,0,1,1,1,11,9332.168761,7176.567982,6.779973524,3.678924666,46.44850006
2.168761,7176.567982,6.779973524,3.678924666,46.44850006,3.579285958,7,1,31.81372192,18.3
32.168761,7176.567982,6.779973524,3.678924666,46.44850006,3.579285958,7,1,31.81372192,18.
2.168761,7176.567982,6.779973524,3.678924666,46.44850006,3.579285958,7,1,31.81372192,18.3
32.168761,7176.567982,6.779973524,3.678924666,46.44850006,3.579285958,7,1,31.81372192,18.
382324,3,2,216,87,1,1,1,1,1,7,9139.1861,6612.240396,6.253846161,8.618838429,46.44850006,11.1
39.1861,6612.240396,6.253846161,8.618838429,46.44850006,11.15261014,7,2,22.74125877,17.23

.1861,6612.240396,6.253846161,8.618838429,46.44850006,11.15261014,7,2,22.74125877,17.2376
39.1861,6612.240396,6.253846161,8.618838429,46.44850006,11.15261014,7,2,22.74125877,17.23
.618838429,46.44850006,11.15261014,7,2,22.74125877,17.23767686,115.512728,84.14265744,99.
39.1861,6612.240396,6.253846161,8.618838429,46.44850006,11.15261014,7,2,22.74125877,17.23
9.1861,6612.240396,6.253846161,8.618838429,46.44850006,11.15261014,7,2,22.74125877,17.237
39.1861,6612.240396,6.253846161,8.618838429,46.44850006,11.15261014,7,2,22.74125877,17.23
.1861,6612.240396,6.253846161,8.618838429,46.44850006,11.15261014,7,2,22.74125877,17.2376
18838429,46.44850006,11.15261014,7,2,22.74125877,17.23767686,115.512728,84.14265744,99.11
8.618838429,46.44850006,11.15261014,7,2,22.74125877,17.23767686,115.512728,84.14265744,9
8.618838429,46.44850006,11.15261014,7,2,22.74125877,17.23767686,115.512728,84.14265744,9
.1861,6612.240396,6.253846161,8.618838429,46.44850006,11.15261014,7,2,22.74125877,17.2376
39.1861,6612.240396,6.253846161,8.618838429,46.44850006,11.15261014,7,2,22.74125877,17.23
18838429,46.44850006,11.15261014,7,2,22.74125877,17.23767686,115.512728,84.14265744,99.11
.1861,6612.240396,6.253846161,8.618838429,46.44850006,11.15261014,7,2,22.74125877,17.2376
.1861,6612.240396,6.253846161,8.618838429,46.44850006,11.15261014,7,2,22.74125877,17.2376
46161,8.618838429,46.44850006,11.15261014,7,2,22.74125877,17.23767686,115.512728,84.14265
39.1861,6612.240396,6.253846161,8.618838429,46.44850006,11.15261014,7,2,22.74125877,17.23
18838429,46.44850006,11.15261014,7,2,22.74125877,17.23767686,115.512728,84.14265744,99.11
18838429,46.44850006,11.15261014,7,2,22.74125877,17.23767686,115.512728,84.14265744,99.11
39.1861,6612.240396,6.253846161,8.618838429,46.44850006,11.15261014,7,2,22.74125877,17.23
.1861,6612.240396,6.253846161,8.618838429,46.44850006,11.15261014,7,2,22.74125877,17.2376
.1861,6612.240396,6.253846161,8.618838429,46.44850006,11.15261014,7,2,22.74125877,17.2376
9139.1861,6612.240396,6.253846161,8.618838429,46.44850006,11.15261014,7,2,22.74125877,17.
39.1861,6612.240396,6.253846161,8.618838429,46.44850006,11.15261014,7,2,22.74125877,17.23
.1861,6612.240396,6.253846161,8.618838429,46.44850006,11.15261014,7,2,22.74125877,17.2376
39.1861,6612.240396,6.253846161,8.618838429,46.44850006,11.15261014,7,2,22.74125877,17.23
.1861,6612.240396,6.253846161,8.618838429,46.44850006,11.15261014,7,2,22.74125877,17.2376
574,4.396556935,46.44850006,3.579285958,7,1,25.16039987,21.98278467,257.4577895,91.890156
94.005866,6308.025976,6.563582574,4.396556935,46.44850006,3.579285958,7,1,25.16039987,21.
582574,4.396556935,46.44850006,3.579285958,7,1,25.16039987,21.98278467,257.4577895,91.890
574,4.396556935,46.44850006,3.579285958,7,1,25.16039987,21.98278467,257.4577895,91.890156
94.005866,6308.025976,6.563582574,4.396556935,46.44850006,3.579285958,7,1,25.16039987,21.
94.005866,6308.025976,6.563582574,4.396556935,46.44850006,3.579285958,7,1,25.16039987,21.
9,0,1,0,1,1,11,9694.005866,6308.025976,6.563582574,4.396556935,46.44850006,3.579285958,7,1,
382324,3,2,216,69,0,1,0,1,1,11,9694.005866,6308.025976,6.563582574,4.396556935,46.44850006,
9694.005866,6308.025976,6.563582574,4.396556935,46.44850006,3.579285958,7,1,25.16039987,2
9,0,1,0,1,1,11,9694.005866,6308.025976,6.563582574,4.396556935,46.44850006,3.579285958,7,1,
4.005866,6308.025976,6.563582574,4.396556935,46.44850006,3.579285958,7,1,25.16039987,21.9
,0,1,0,1,1,11,9694.005866,6308.025976,6.563582574,4.396556935,46.44850006,3.579285958,7,1,2
694.005866,6308.025976,6.563582574,4.396556935,46.44850006,3.579285958,7,1,25.16039987,2
94.005866,6308.025976,6.563582574,4.396556935,46.44850006,3.579285958,7,1,25.16039987,21.
4.005866,6308.025976,6.563582574,4.396556935,46.44850006,3.579285958,7,1,25.16039987,21.9
94.005866,6308.025976,6.563582574,4.396556935,46.44850006,3.579285958,7,1,25.16039987,21.
94.005866,6308.025976,6.563582574,4.396556935,46.44850006,3.579285958,7,1,25.16039987,21.
574,4.396556935,46.44850006,3.579285958,7,1,25.16039987,21.98278467,257.4577895,91.890156
9694.005866,6308.025976,6.563582574,4.396556935,46.44850006,3.579285958,7,1,25.16039987,2
94.005866,6308.025976,6.563582574,4.396556935,46.44850006,3.579285958,7,1,25.16039987,21.
32574,4.396556935,46.44850006,3.579285958,7,1,25.16039987,21.98278467,257.4577895,91.8901
74,4.396556935,46.44850006,3.579285958,7,1,25.16039987,21.98278467,257.4577895,91.8901560
94.005866,6308.025976,6.563582574,4.396556935,46.44850006,3.579285958,7,1,25.16039987,21.

694.005866,6308.025976,6.563582574,4.396556935,46.44850006,3.579285958,7,1,25.16039987,2
2574,4.396556935,46.44850006,3.579285958,7,1,25.16039987,21.98278467,257.4577895,91.89015
574,4.396556935,46.44850006,3.579285958,7,1,25.16039987,21.98278467,257.4577895,91.890156
94.005866,6308.025976,6.563582574,4.396556935,46.44850006,3.579285958,7,1,25.16039987,21.
574,4.396556935,46.44850006,3.579285958,7,1,25.16039987,21.98278467,257.4577895,91.890156
582574,4.396556935,46.44850006,3.579285958,7,1,25.16039987,21.98278467,257.4577895,91.890
905,4.35553425,46.44850006,3.579285958,7,1,32.06887341,17.422137,211.1094277,93.5342141,1
31.316406,6591.464379,6.948255905,4.35553425,46.44850006,3.579285958,7,1,32.06887341,17.4
9,1,1,0,0,1,10,9731.316406,6591.464379,6.948255905,4.35553425,46.44850006,3.579285958,7,1,3
31.316406,6591.464379,6.948255905,4.35553425,46.44850006,3.579285958,7,1,32.06887341,17.4
05,4.35553425,46.44850006,3.579285958,7,1,32.06887341,17.422137,211.1094277,93.5342141,10
255905,4.35553425,46.44850006,3.579285958,7,1,32.06887341,17.422137,211.1094277,93.534214
31.316406,6591.464379,6.948255905,4.35553425,46.44850006,3.579285958,7,1,32.06887341,17.4
31.316406,6591.464379,6.948255905,4.35553425,46.44850006,3.579285958,7,1,32.06887341,17.4
31.316406,6591.464379,6.948255905,4.35553425,46.44850006,3.579285958,7,1,32.06887341,17.4
31.316406,6591.464379,6.948255905,4.35553425,46.44850006,3.579285958,7,1,32.06887341,17.4
948255905,4.35553425,46.44850006,3.579285958,7,1,32.06887341,17.422137,211.1094277,93.53
31.316406,6591.464379,6.948255905,4.35553425,46.44850006,3.579285958,7,1,32.06887341,17.4
31.316406,6591.464379,6.948255905,4.35553425,46.44850006,3.579285958,7,1,32.06887341,17.4
731.316406,6591.464379,6.948255905,4.35553425,46.44850006,3.579285958,7,1,32.06887341,17.
905,4.35553425,46.44850006,3.579285958,7,1,32.06887341,17.422137,211.1094277,93.5342141,1
948255905,4.35553425,46.44850006,3.579285958,7,1,32.06887341,17.422137,211.1094277,93.53
9,1,1,0,0,1,10,9731.316406,6591.464379,6.948255905,4.35553425,46.44850006,3.579285958,7,1,3
1.316406,6591.464379,6.948255905,4.35553425,46.44850006,3.579285958,7,1,32.06887341,17.42
1.316406,6591.464379,6.948255905,4.35553425,46.44850006,3.579285958,7,1,32.06887341,17.42
255905,4.35553425,46.44850006,3.579285958,7,1,32.06887341,17.422137,211.1094277,93.534214
948255905,4.35553425,46.44850006,3.579285958,7,1,32.06887341,17.422137,211.1094277,93.53
948255905,4.35553425,46.44850006,3.579285958,7,1,32.06887341,17.422137,211.1094277,93.53
1.316406,6591.464379,6.948255905,4.35553425,46.44850006,3.579285958,7,1,32.06887341,17.42
69,1,1,0,0,1,10,9731.316406,6591.464379,6.948255905,4.35553425,46.44850006,3.579285958,7,1,
255905,4.35553425,46.44850006,3.579285958,7,1,32.06887341,17.422137,211.1094277,93.534214
731.316406,6591.464379,6.948255905,4.35553425,46.44850006,3.579285958,7,1,32.06887341,17.
948255905,4.35553425,46.44850006,3.579285958,7,1,32.06887341,17.422137,211.1094277,93.53
9,1,1,0,0,1,10,9731.316406,6591.464379,6.948255905,4.35553425,46.44850006,3.579285958,7,1,3
69,1,1,0,0,1,10,9731.316406,6591.464379,6.948255905,4.35553425,46.44850006,3.579285958,7,1,
905,4.35553425,46.44850006,3.579285958,7,1,32.06887341,17.422137,211.1094277,93.5342141,1
1.316406,6591.464379,6.948255905,4.35553425,46.44850006,3.579285958,7,1,32.06887341,17.42
31.316406,6591.464379,6.948255905,4.35553425,46.44850006,3.579285958,7,1,32.06887341,17.4
4.35553425,46.44850006,3.579285958,7,1,32.06887341,17.422137,211.1094277,93.5342141,108.8
31.316406,6591.464379,6.948255905,4.35553425,46.44850006,3.579285958,7,1,32.06887341,17.4
31.316406,6591.464379,6.948255905,4.35553425,46.44850006,3.579285958,7,1,32.06887341,17.4
31.316406,6591.464379,6.948255905,4.35553425,46.44850006,3.579285958,7,1,32.06887341,17.4
1.316406,6591.464379,6.948255905,4.35553425,46.44850006,3.579285958,7,1,32.06887341,17.42
731.316406,6591.464379,6.948255905,4.35553425,46.44850006,3.579285958,7,1,32.06887341,17.
31.316406,6591.464379,6.948255905,4.35553425,46.44850006,3.579285958,7,1,32.06887341,17.4
31.316406,6591.464379,6.948255905,4.35553425,46.44850006,3.579285958,7,1,32.06887341,17.4
905,4.35553425,46.44850006,3.579285958,7,1,32.06887341,17.422137,211.1094277,93.5342141,1
48255905,4.35553425,46.44850006,3.579285958,7,1,32.06887341,17.422137,211.1094277,93.5342
255905,4.35553425,46.44850006,3.579285958,7,1,32.06887341,17.422137,211.1094277,93.534214
9,0,1,0,0,1,12,9731.316406,6591.464379,6.948255905,4.35553425,46.44850006,3.579285958,7,1,3

31.316406,6591.464379,6.948255905,4.35553425,46.44850006,3.579285958,7,1,32.06887341,17.4
05,4.35553425,46.44850006,3.579285958,7,1,32.06887341,17.422137,211.1094277,93.5342141,10
1.316406,6591.464379,6.948255905,4.35553425,46.44850006,3.579285958,7,1,32.06887341,17.42
905,4.35553425,46.44850006,3.579285958,7,1,32.06887341,17.422137,211.1094277,93.5342141,1
31.316406,6591.464379,6.948255905,4.35553425,46.44850006,3.579285958,7,1,32.06887341,17.4
1.316406,6591.464379,6.948255905,4.35553425,46.44850006,3.579285958,7,1,32.06887341,17.42
731.316406,6591.464379,6.948255905,4.35553425,46.44850006,3.579285958,7,1,32.06887341,17.
905,4.35553425,46.44850006,3.579285958,7,1,32.06887341,17.422137,211.1094277,93.5342141,1
31.316406,6591.464379,6.948255905,4.35553425,46.44850006,3.579285958,7,1,32.06887341,17.4
31.316406,6591.464379,6.948255905,4.35553425,46.44850006,3.579285958,7,1,32.06887341,17.4
905,4.35553425,46.44850006,3.579285958,7,1,32.06887341,17.422137,211.1094277,93.5342141,1
255905,4.35553425,46.44850006,3.579285958,7,1,32.06887341,17.422137,211.1094277,93.534214
31.316406,6591.464379,6.948255905,4.35553425,46.44850006,3.579285958,7,1,32.06887341,17.4
31.316406,6591.464379,6.948255905,4.35553425,46.44850006,3.579285958,7,1,32.06887341,17.4
1.316406,6591.464379,6.948255905,4.35553425,46.44850006,3.579285958,7,1,32.06887341,17.42
69,0,1,0,0,1,12,9731.316406,6591.464379,6.948255905,4.35553425,46.44850006,3.579285958,7,1,
905,4.35553425,46.44850006,3.579285958,7,1,32.06887341,17.422137,211.1094277,93.5342141,1
31.316406,6591.464379,6.948255905,4.35553425,46.44850006,3.579285958,7,1,32.06887341,17.4
31.316406,6591.464379,6.948255905,4.35553425,46.44850006,3.579285958,7,1,32.06887341,17.4
905,4.35553425,46.44850006,3.579285958,7,1,32.06887341,17.422137,211.1094277,93.5342141,1
69,0,1,0,0,1,12,9731.316406,6591.464379,6.948255905,4.35553425,46.44850006,3.579285958,7,1,
22547,4.495387381,46.44850006,3.579285958,7,1,25.02446976,22.47693691,243.5836295,92.4817
1,0,1,0,1,1,11,9646.36136,6458.018467,6.528122547,4.495387381,46.44850006,3.579285958,7,1,2
646.36136,6458.018467,6.528122547,4.495387381,46.44850006,3.579285958,7,1,25.02446976,22.
46.36136,6458.018467,6.528122547,4.495387381,46.44850006,3.579285958,7,1,25.02446976,22.4
546.36136,6458.018467,6.528122547,4.495387381,46.44850006,3.579285958,7,1,25.02446976,22.
1,0,1,0,1,1,11,9646.36136,6458.018467,6.528122547,4.495387381,46.44850006,3.579285958,7,1,2
646.36136,6458.018467,6.528122547,4.495387381,46.44850006,3.579285958,7,1,25.02446976,22.
8122547,4.495387381,46.44850006,3.579285958,7,1,25.02446976,22.47693691,243.5836295,92.48
6.36136,6458.018467,6.528122547,4.495387381,46.44850006,3.579285958,7,1,25.02446976,22.47
46.36136,6458.018467,6.528122547,4.495387381,46.44850006,3.579285958,7,1,25.02446976,22.4
9646.36136,6458.018467,6.528122547,4.495387381,46.44850006,3.579285958,7,1,25.02446976,22
28122547,4.495387381,46.44850006,3.579285958,7,1,25.02446976,22.47693691,243.5836295,92.
6.36136,6458.018467,6.528122547,4.495387381,46.44850006,3.579285958,7,1,25.02446976,22.47
28122547,4.495387381,46.44850006,3.579285958,7,1,25.02446976,22.47693691,243.5836295,92.
46.36136,6458.018467,6.528122547,4.495387381,46.44850006,3.579285958,7,1,25.02446976,22.4
6.36136,6458.018467,6.528122547,4.495387381,46.44850006,3.579285958,7,1,25.02446976,22.47
6.36136,6458.018467,6.528122547,4.495387381,46.44850006,3.579285958,7,1,25.02446976,22.47
7,4.495387381,46.44850006,3.579285958,7,1,25.02446976,22.47693691,243.5836295,92.48173608
1,0,1,0,1,1,11,9646.36136,6458.018467,6.528122547,4.495387381,46.44850006,3.579285958,7,1,2
646.36136,6458.018467,6.528122547,4.495387381,46.44850006,3.579285958,7,1,25.02446976,22.
6.36136,6458.018467,6.528122547,4.495387381,46.44850006,3.579285958,7,1,25.02446976,22.47
6.36136,6458.018467,6.528122547,4.495387381,46.44850006,3.579285958,7,1,25.02446976,22.47
7,4.495387381,46.44850006,3.579285958,7,1,25.02446976,22.47693691,243.5836295,92.48173608
7,4.495387381,46.44850006,3.579285958,7,1,25.02446976,22.47693691,243.5836295,92.48173608
46.36136,6458.018467,6.528122547,4.495387381,46.44850006,3.579285958,7,1,25.02446976,22.4
1,0,1,0,1,1,11,9646.36136,6458.018467,6.528122547,4.495387381,46.44850006,3.579285958,7,1,2
0617676,3,2,216,81,0,1,0,1,1,11,9646.36136,6458.018467,6.528122547,4.495387381,46.44850006,
22547,4.495387381,46.44850006,3.579285958,7,1,25.02446976,22.47693691,243.5836295,92.4817
6.36136,6458.018467,6.528122547,4.495387381,46.44850006,3.579285958,7,1,25.02446976,22.47

646.36136,6458.018467,6.528122547,4.495387381,46.44850006,3.579285958,7,1,25.02446976,22.
6.36136,6458.018467,6.528122547,4.495387381,46.44850006,3.579285958,7,1,25.02446976,22.47
74,4.396556935,46.44850006,3.579285958,7,1,25.16039987,21.98278467,257.4577895,91.8901560
574,4.396556935,46.44850006,3.579285958,7,1,25.16039987,21.98278467,257.4577895,91.890156
694.005866,6308.025976,6.563582574,4.396556935,46.44850006,3.579285958,7,1,25.16039987,21
9,1,1,0,1,1,11,9694.005866,6308.025976,6.563582574,4.396556935,46.44850006,3.579285958,7,1,
4.005866,6308.025976,6.563582574,4.396556935,46.44850006,3.579285958,7,1,25.16039987,21.9
4.005866,6308.025976,6.563582574,4.396556935,46.44850006,3.579285958,7,1,25.16039987,21.9
94.005866,6308.025976,6.563582574,4.396556935,46.44850006,3.579285958,7,1,25.16039987,21.
694.005866,6308.025976,6.563582574,4.396556935,46.44850006,3.579285958,7,1,25.16039987,21
4.005866,6308.025976,6.563582574,4.396556935,46.44850006,3.579285958,7,1,25.16039987,21.9
694.005866,6308.025976,6.563582574,4.396556935,46.44850006,3.579285958,7,1,25.16039987,21
4.005866,6308.025976,6.563582574,4.396556935,46.44850006,3.579285958,7,1,25.16039987,21.9
694.005866,6308.025976,6.563582574,4.396556935,46.44850006,3.579285958,7,1,25.16039987,21
4.005866,6308.025976,6.563582574,4.396556935,46.44850006,3.579285958,7,1,25.16039987,21.9
9382324,3,2,216,69,1,1,0,1,1,11,9694.005866,6308.025976,6.563582574,4.396556935,46.44850006
4.005866,6308.025976,6.563582574,4.396556935,46.44850006,3.579285958,7,1,25.16039987,21.9
94.005866,6308.025976,6.563582574,4.396556935,46.44850006,3.579285958,7,1,25.16039987,21.
94.005866,6308.025976,6.563582574,4.396556935,46.44850006,3.579285958,7,1,25.16039987,21.
4.005866,6308.025976,6.563582574,4.396556935,46.44850006,3.579285958,7,1,25.16039987,21.9
94.005866,6308.025976,6.563582574,4.396556935,46.44850006,3.579285958,7,1,25.16039987,21.
9,1,1,0,1,1,11,9694.005866,6308.025976,6.563582574,4.396556935,46.44850006,3.579285958,7,1,
4.005866,6308.025976,6.563582574,4.396556935,46.44850006,3.579285958,7,1,25.16039987,21.9
4.005866,6308.025976,6.563582574,4.396556935,46.44850006,3.579285958,7,1,25.16039987,21.9
9,1,1,0,1,1,11,9694.005866,6308.025976,6.563582574,4.396556935,46.44850006,3.579285958,7,1,
0308838,3,2,216,69,1,1,0,1,1,11,9694.005866,6308.025976,6.563582574,4.396556935,46.44850006
4,4.396556935,46.44850006,3.579285958,7,1,25.16039987,21.98278467,257.4577895,91.89015604
694.005866,6308.025976,6.563582574,4.396556935,46.44850006,3.579285958,7,1,25.16039987,21
4.005866,6308.025976,6.563582574,4.396556935,46.44850006,3.579285958,7,1,25.16039987,21.9
4.005866,6308.025976,6.563582574,4.396556935,46.44850006,3.579285958,7,1,25.16039987,21.9
4.005866,6308.025976,6.563582574,4.396556935,46.44850006,3.579285958,7,1,25.16039987,21.9
974073,12.24173132,46.44850006,11.15261014,7,2,22.51609111,8.161154211,207.6498451,91.672
094.195764,5760.148353,5.360974073,12.24173132,46.44850006,11.15261014,7,2,22.51609111,8.
073,12.24173132,46.44850006,11.15261014,7,2,22.51609111,8.161154211,207.6498451,91.672656
073,12.24173132,46.44850006,11.15261014,7,2,22.51609111,8.161154211,207.6498451,91.672656
073,12.24173132,46.44850006,11.15261014,7,2,22.51609111,8.161154211,207.6498451,91.672656
4.195764,5760.148353,5.360974073,12.24173132,46.44850006,11.15261014,7,2,22.51609111,8.16
60974073,12.24173132,46.44850006,11.15261014,7,2,22.51609111,8.161154211,207.6498451,91.6
73,1,1,1,1,1,11,9094.195764,5760.148353,5.360974073,12.24173132,46.44850006,11.15261014,7,2
73,12.24173132,46.44850006,11.15261014,7,2,22.51609111,8.161154211,207.6498451,91.6726566
974073,12.24173132,46.44850006,11.15261014,7,2,22.51609111,8.161154211,207.6498451,91.672
4.195764,5760.148353,5.360974073,12.24173132,46.44850006,11.15261014,7,2,22.51609111,8.16
4.195764,5760.148353,5.360974073,12.24173132,46.44850006,11.15261014,7,2,22.51609111,8.16
73,1,1,1,1,1,11,9094.195764,5760.148353,5.360974073,12.24173132,46.44850006,11.15261014,7,2
974073,12.24173132,46.44850006,11.15261014,7,2,22.51609111,8.161154211,207.6498451,91.672
0308838,3,2,216,73,1,1,1,1,1,11,9094.195764,5760.148353,5.360974073,12.24173132,46.44850006
,1,1,1,1,1,11,9094.195764,5760.148353,5.360974073,12.24173132,46.44850006,11.15261014,7,2,2
073,12.24173132,46.44850006,11.15261014,7,2,22.51609111,8.161154211,207.6498451,91.672656
974073,12.24173132,46.44850006,11.15261014,7,2,22.51609111,8.161154211,207.6498451,91.672
5.360974073,12.24173132,46.44850006,11.15261014,7,2,22.51609111,8.161154211,207.6498451,9

,1,1,1,1,1,11,9094.195764,5760.148353,5.360974073,12.24173132,46.44850006,11.15261014,7,2,2
382324,3,2,216,73,1,1,1,1,1,11,9094.195764,5760.148353,5.360974073,12.24173132,46.44850006,
094.195764,5760.148353,5.360974073,12.24173132,46.44850006,11.15261014,7,2,22.51609111,8.
3,12.24173132,46.44850006,11.15261014,7,2,22.51609111,8.161154211,207.6498451,91.67265665
974073,12.24173132,46.44850006,11.15261014,7,2,22.51609111,8.161154211,207.6498451,91.672
4.195764,5760.148353,5.360974073,12.24173132,46.44850006,11.15261014,7,2,22.51609111,8.16
517676,3,2,216,67,1,1,0,1,1,12,10241.16811,9088.747035,6.276338387,8.842318954,29.39298478,
241.16811,9088.747035,6.276338387,8.842318954,29.39298478,4.132277098,5,2,29.81260734,22.
241.16811,9088.747035,6.276338387,8.842318954,29.39298478,4.132277098,5,2,29.81260734,22.
241.16811,9088.747035,6.276338387,8.842318954,29.39298478,4.132277098,5,2,29.81260734,22.
41.16811,9088.747035,6.276338387,8.842318954,29.39298478,4.132277098,5,2,29.81260734,22.1
387,8.842318954,29.39298478,4.132277098,5,2,29.81260734,22.10579739,169.8487829,104.60563
7,1,1,0,1,1,12,10241.16811,9088.747035,6.276338387,8.842318954,29.39298478,4.132277098,5,2,
241.16811,9088.747035,6.276338387,8.842318954,29.39298478,4.132277098,5,2,29.81260734,22.
7,1,1,0,1,1,12,10241.16811,9088.747035,6.276338387,8.842318954,29.39298478,4.132277098,5,2,
41.16811,9088.747035,6.276338387,8.842318954,29.39298478,4.132277098,5,2,29.81260734,22.1
41.16811,9088.747035,6.276338387,8.842318954,29.39298478,4.132277098,5,2,29.81260734,22.1
241.16811,9088.747035,6.276338387,8.842318954,29.39298478,4.132277098,5,2,29.81260734,22.
241.16811,9088.747035,6.276338387,8.842318954,29.39298478,4.132277098,5,2,29.81260734,22.
387,8.842318954,29.39298478,4.132277098,5,2,29.81260734,22.10579739,169.8487829,104.60563
41.16811,9088.747035,6.276338387,8.842318954,29.39298478,4.132277098,5,2,29.81260734,22.1
3387,8.842318954,29.39298478,4.132277098,5,2,29.81260734,22.10579739,169.8487829,104.6056
241.16811,9088.747035,6.276338387,8.842318954,29.39298478,4.132277098,5,2,29.81260734,22.
241.16811,9088.747035,6.276338387,8.842318954,29.39298478,4.132277098,5,2,29.81260734,22.
7,1,1,0,1,1,12,10241.16811,9088.747035,6.276338387,8.842318954,29.39298478,4.132277098,5,2,
241.16811,9088.747035,6.276338387,8.842318954,29.39298478,4.132277098,5,2,29.81260734,22.
7,1,1,0,1,1,12,10241.16811,9088.747035,6.276338387,8.842318954,29.39298478,4.132277098,5,2,
7,1,1,0,1,1,12,10241.16811,9088.747035,6.276338387,8.842318954,29.39298478,4.132277098,5,2,
7,1,1,0,1,1,12,10241.16811,9088.747035,6.276338387,8.842318954,29.39298478,4.132277098,5,2,
7,1,1,0,1,1,12,10241.16811,9088.747035,6.276338387,8.842318954,29.39298478,4.132277098,5,2,
9845581,3,2,216,67,1,1,0,1,1,12,10241.16811,9088.747035,6.276338387,8.842318954,29.39298478
3387,8.842318954,29.39298478,4.132277098,5,2,29.81260734,22.10579739,169.8487829,104.6056
241.16811,9088.747035,6.276338387,8.842318954,29.39298478,4.132277098,5,2,29.81260734,22.
7,1,1,0,1,1,12,10241.16811,9088.747035,6.276338387,8.842318954,29.39298478,4.132277098,5,2,
7,1,1,0,1,1,12,10241.16811,9088.747035,6.276338387,8.842318954,29.39298478,4.132277098,5,2,
387,8.842318954,29.39298478,4.132277098,5,2,29.81260734,22.10579739,169.8487829,104.60563
7,1,1,0,1,1,12,10241.16811,9088.747035,6.276338387,8.842318954,29.39298478,4.132277098,5,2,
308838,3,2,216,67,1,1,0,1,1,12,10241.16811,9088.747035,6.276338387,8.842318954,29.39298478,
241.16811,9088.747035,6.276338387,8.842318954,29.39298478,4.132277098,5,2,29.81260734,22.
41.16811,9088.747035,6.276338387,8.842318954,29.39298478,4.132277098,5,2,29.81260734,22.1
41.16811,9088.747035,6.276338387,8.842318954,29.39298478,4.132277098,5,2,29.81260734,22.1
67,1,1,0,1,1,12,10241.16811,9088.747035,6.276338387,8.842318954,29.39298478,4.132277098,5,
41.16811,9088.747035,6.276338387,8.842318954,29.39298478,4.132277098,5,2,29.81260734,22.1
154419,3,2,216,67,1,1,0,1,1,12,10241.16811,9088.747035,6.276338387,8.842318954,29.39298478,
305,5.473544327,15.25345664,0,4,0,27.37454762,10.94708865,359.4661544,72.99879366,60.2089
28.818269,5923.880539,6.083232805,5.473544327,15.25345664,0,4,0,27.37454762,10.94708865,3
083232805,5.473544327,15.25345664,0,4,0,27.37454762,10.94708865,359.4661544,72.99879366,
7,0,1,0,1,1,12,6628.818269,5923.880539,6.083232805,5.473544327,15.25345664,0,4,0,27.3745476
628.818269,5923.880539,6.083232805,5.473544327,15.25345664,0,4,0,27.37454762,10.94708865,
232805,5.473544327,15.25345664,0,4,0,27.37454762,10.94708865,359.4661544,72.99879366,60.2

7,0,1,0,1,1,12,6628.818269,5923.880539,6.083232805,5.473544327,15.25345664,0,4,0,27.3745476
67,0,1,0,1,1,12,6628.818269,5923.880539,6.083232805,5.473544327,15.25345664,0,4,0,27.374547
083232805,5.473544327,15.25345664,0,4,0,27.37454762,10.94708865,359.4661544,72.99879366,
67,0,1,0,1,1,12,6628.818269,5923.880539,6.083232805,5.473544327,15.25345664,0,4,0,27.374547
232805,5.473544327,15.25345664,0,4,0,27.37454762,10.94708865,359.4661544,72.99879366,60.2
3232805,5.473544327,15.25345664,0,4,0,27.37454762,10.94708865,359.4661544,72.99879366,60
67,0,1,0,1,1,12,6628.818269,5923.880539,6.083232805,5.473544327,15.25345664,0,4,0,27.374547
28.818269,5923.880539,6.083232805,5.473544327,15.25345664,0,4,0,27.37454762,10.94708865,3
3232805,5.473544327,15.25345664,0,4,0,27.37454762,10.94708865,359.4661544,72.99879366,60
05,5.473544327,15.25345664,0,4,0,27.37454762,10.94708865,359.4661544,72.99879366,60.2089
28.818269,5923.880539,6.083232805,5.473544327,15.25345664,0,4,0,27.37454762,10.94708865,3
28.818269,5923.880539,6.083232805,5.473544327,15.25345664,0,4,0,27.37454762,10.94708865,3
28.818269,5923.880539,6.083232805,5.473544327,15.25345664,0,4,0,27.37454762,10.94708865,3
8.818269,5923.880539,6.083232805,5.473544327,15.25345664,0,4,0,27.37454762,10.94708865,35
083232805,5.473544327,15.25345664,0,4,0,27.37454762,10.94708865,359.4661544,72.99879366,6
2805,5.473544327,15.25345664,0,4,0,27.37454762,10.94708865,359.4661544,72.99879366,60.208
3232805,5.473544327,15.25345664,0,4,0,27.37454762,10.94708865,359.4661544,72.99879366,60
083232805,5.473544327,15.25345664,0,4,0,27.37454762,10.94708865,359.4661544,72.99879366,
628.818269,5923.880539,6.083232805,5.473544327,15.25345664,0,4,0,27.37454762,10.94708865,
05,5.473544327,15.25345664,0,4,0,27.37454762,10.94708865,359.4661544,72.99879366,60.2089
628.818269,5923.880539,6.083232805,5.473544327,15.25345664,0,4,0,27.37454762,10.94708865,
8.818269,5923.880539,6.083232805,5.473544327,15.25345664,0,4,0,27.37454762,10.94708865,35
67,0,1,0,1,1,12,6628.818269,5923.880539,6.083232805,5.473544327,15.25345664,0,4,0,27.374547
28.818269,5923.880539,6.083232805,5.473544327,15.25345664,0,4,0,27.37454762,10.94708865,3
05,5.473544327,15.25345664,0,4,0,27.37454762,10.94708865,359.4661544,72.99879366,60.2089
2805,5.473544327,15.25345664,0,4,0,27.37454762,10.94708865,359.4661544,72.99879366,60.208
232805,5.473544327,15.25345664,0,4,0,27.37454762,10.94708865,359.4661544,72.99879366,60.2
238.843127,6192.89729,6.937282421,5.45904552,15.25345664,0,4,0,15.41618316,10.91809104,28
76,1,1,0,1,1,11,6238.843127,6192.89729,6.937282421,5.45904552,15.25345664,0,4,0,15.41618316
76,1,1,0,1,1,11,6238.843127,6192.89729,6.937282421,5.45904552,15.25345664,0,4,0,15.41618316
76,1,1,0,1,1,11,6238.843127,6192.89729,6.937282421,5.45904552,15.25345664,0,4,0,15.41618316
8.843127,6192.89729,6.937282421,5.45904552,15.25345664,0,4,0,15.41618316,10.91809104,280.
2421,5.45904552,15.25345664,0,4,0,15.41618316,10.91809104,280.1350133,69.37282421,49.131
2421,5.45904552,15.25345664,0,4,0,15.41618316,10.91809104,280.1350133,69.37282421,49.131
238.843127,6192.89729,6.937282421,5.45904552,15.25345664,0,4,0,15.41618316,10.91809104,28
8.843127,6192.89729,6.937282421,5.45904552,15.25345664,0,4,0,15.41618316,10.91809104,280.
21,5.45904552,15.25345664,0,4,0,15.41618316,10.91809104,280.1350133,69.37282421,49.131409
,5.45904552,15.25345664,0,4,0,15.41618316,10.91809104,280.1350133,69.37282421,49.1314096
,1,1,0,1,1,11,6238.843127,6192.89729,6.937282421,5.45904552,15.25345664,0,4,0,15.41618316,1
76,1,1,0,1,1,11,6238.843127,6192.89729,6.937282421,5.45904552,15.25345664,0,4,0,15.41618316
238.843127,6192.89729,6.937282421,5.45904552,15.25345664,0,4,0,15.41618316,10.91809104,28
.937282421,5.45904552,15.25345664,0,4,0,15.41618316,10.91809104,280.1350133,69.37282421,4
21,5.45904552,15.25345664,0,4,0,15.41618316,10.91809104,280.1350133,69.37282421,49.131409
9382324,3,2,217,76,1,1,0,1,1,11,6238.843127,6192.89729,6.937282421,5.45904552,15.25345664,0,
76,1,1,0,1,1,11,6238.843127,6192.89729,6.937282421,5.45904552,15.25345664,0,4,0,15.41618316
238.843127,6192.89729,6.937282421,5.45904552,15.25345664,0,4,0,15.41618316,10.91809104,28
2421,5.45904552,15.25345664,0,4,0,15.41618316,10.91809104,280.1350133,69.37282421,49.131
2421,5.45904552,15.25345664,0,4,0,15.41618316,10.91809104,280.1350133,69.37282421,49.131
21,5.45904552,15.25345664,0,4,0,15.41618316,10.91809104,280.1350133,69.37282421,49.131409
7282421,5.45904552,15.25345664,0,4,0,15.41618316,10.91809104,280.1350133,69.37282421,49.13

21,5.45904552,15.25345664,0,4,0,15.41618316,10.91809104,280.1350133,69.37282421,49.131409
0617676,3,2,217,76,1,1,0,1,1,11,6238.843127,6192.89729,6.937282421,5.45904552,15.25345664,0,
21,5.45904552,15.25345664,0,4,0,15.41618316,10.91809104,280.1350133,69.37282421,49.131409
8.843127,6192.89729,6.937282421,5.45904552,15.25345664,0,4,0,15.41618316,10.91809104,280.
19331,9.640917568,15.25345664,3.579285958,4,1,25.00014708,0,405.9133213,75.00044123,67.48
0.489604,6321.696741,5.714319331,9.640917568,15.25345664,3.579285958,4,1,25.00014708,0,40
0.489604,6321.696741,5.714319331,9.640917568,15.25345664,3.579285958,4,1,25.00014708,0,40
331,9.640917568,15.25345664,3.579285958,4,1,25.00014708,0,405.9133213,75.00044123,67.4864
070.489604,6321.696741,5.714319331,9.640917568,15.25345664,3.579285958,4,1,25.00014708,0,
31,9.640917568,15.25345664,3.579285958,4,1,25.00014708,0,405.9133213,75.00044123,67.48642
14319331,9.640917568,15.25345664,3.579285958,4,1,25.00014708,0,405.9133213,75.00044123,67
70.489604,6321.696741,5.714319331,9.640917568,15.25345664,3.579285958,4,1,25.00014708,0,4
14319331,9.640917568,15.25345664,3.579285958,4,1,25.00014708,0,405.9133213,75.00044123,67
70.489604,6321.696741,5.714319331,9.640917568,15.25345664,3.579285958,4,1,25.00014708,0,4
19331,9.640917568,15.25345664,3.579285958,4,1,25.00014708,0,405.9133213,75.00044123,67.48
070.489604,6321.696741,5.714319331,9.640917568,15.25345664,3.579285958,4,1,25.00014708,0,
331,9.640917568,15.25345664,3.579285958,4,1,25.00014708,0,405.9133213,75.00044123,67.4864
14319331,9.640917568,15.25345664,3.579285958,4,1,25.00014708,0,405.9133213,75.00044123,67
070.489604,6321.696741,5.714319331,9.640917568,15.25345664,3.579285958,4,1,25.00014708,0,
31,9.640917568,15.25345664,3.579285958,4,1,25.00014708,0,405.9133213,75.00044123,67.48642
70.489604,6321.696741,5.714319331,9.640917568,15.25345664,3.579285958,4,1,25.00014708,0,4
0,1,1,0,1,1,12,7070.489604,6321.696741,5.714319331,9.640917568,15.25345664,3.579285958,4,1,
714319331,9.640917568,15.25345664,3.579285958,4,1,25.00014708,0,405.9133213,75.00044123,
0.489604,6321.696741,5.714319331,9.640917568,15.25345664,3.579285958,4,1,25.00014708,0,40
19331,9.640917568,15.25345664,3.579285958,4,1,25.00014708,0,405.9133213,75.00044123,67.48
0.489604,6321.696741,5.714319331,9.640917568,15.25345664,3.579285958,4,1,25.00014708,0,40
70.489604,6321.696741,5.714319331,9.640917568,15.25345664,3.579285958,4,1,25.00014708,0,4
0.489604,6321.696741,5.714319331,9.640917568,15.25345664,3.579285958,4,1,25.00014708,0,40
31,9.640917568,15.25345664,3.579285958,4,1,25.00014708,0,405.9133213,75.00044123,67.48642
70.489604,6321.696741,5.714319331,9.640917568,15.25345664,3.579285958,4,1,25.00014708,0,4
0.489604,6321.696741,5.714319331,9.640917568,15.25345664,3.579285958,4,1,25.00014708,0,40
70.489604,6321.696741,5.714319331,9.640917568,15.25345664,3.579285958,4,1,25.00014708,0,4
0.489604,6321.696741,5.714319331,9.640917568,15.25345664,3.579285958,4,1,25.00014708,0,40
9.640917568,15.25345664,3.579285958,4,1,25.00014708,0,405.9133213,75.00044123,67.4864229
70.489604,6321.696741,5.714319331,9.640917568,15.25345664,3.579285958,4,1,25.00014708,0,4
70.489604,6321.696741,5.714319331,9.640917568,15.25345664,3.579285958,4,1,25.00014708,0,4
70.489604,6321.696741,5.714319331,9.640917568,15.25345664,3.579285958,4,1,25.00014708,0,4
2,0,1,0,1,1,12,7070.489604,6321.696741,5.714319331,9.640917568,15.25345664,3.579285958,4,1,
517676,3,2,217,72,0,1,0,1,1,12,7070.489604,6321.696741,5.714319331,9.640917568,15.25345664,
0.489604,6321.696741,5.714319331,9.640917568,15.25345664,3.579285958,4,1,25.00014708,0,40
2,0,1,0,1,1,12,7070.489604,6321.696741,5.714319331,9.640917568,15.25345664,3.579285958,4,1,
0.489604,6321.696741,5.714319331,9.640917568,15.25345664,3.579285958,4,1,25.00014708,0,40
14319331,9.640917568,15.25345664,3.579285958,4,1,25.00014708,0,405.9133213,75.00044123,67
258789,3,2,217,72,0,1,0,1,1,12,7070.489604,6321.696741,5.714319331,9.640917568,15.25345664,
,0,1,0,1,1,12,7070.489604,6321.696741,5.714319331,9.640917568,15.25345664,3.579285958,4,1,2
9.640917568,15.25345664,3.579285958,4,1,25.00014708,0,405.9133213,75.00044123,67.4864229
19331,9.640917568,15.25345664,3.579285958,4,1,25.00014708,0,405.9133213,75.00044123,67.48
9331,9.640917568,15.25345664,3.579285958,4,1,25.00014708,0,405.9133213,75.00044123,67.486
70.489604,6321.696741,5.714319331,9.640917568,15.25345664,3.579285958,4,1,25.00014708,0,4
0.489604,6321.696741,5.714319331,9.640917568,15.25345664,3.579285958,4,1,25.00014708,0,40

331,9.640917568,15.25345664,3.579285958,4,1,25.00014708,0,405.9133213,75.00044123,67.4864
2,0,1,0,1,1,12,7070.489604,6321.696741,5.714319331,9.640917568,15.25345664,3.579285958,4,1,
070.489604,6321.696741,5.714319331,9.640917568,15.25345664,3.579285958,4,1,25.00014708,0,
331,9.640917568,15.25345664,3.579285958,4,1,25.00014708,0,405.9133213,75.00044123,67.4864
0.489604,6321.696741,5.714319331,9.640917568,15.25345664,3.579285958,4,1,25.00014708,0,40
319331,9.640917568,15.25345664,3.579285958,4,1,25.00014708,0,405.9133213,75.00044123,67.48
714319331,9.640917568,15.25345664,3.579285958,4,1,25.00014708,0,405.9133213,75.00044123,6
2,0,1,0,1,1,12,7070.489604,6321.696741,5.714319331,9.640917568,15.25345664,3.579285958,4,1,
0.489604,6321.696741,5.714319331,9.640917568,15.25345664,3.579285958,4,1,25.00014708,0,40
331,9.640917568,15.25345664,3.579285958,4,1,25.00014708,0,405.9133213,75.00044123,67.4864
1,9.640917568,15.25345664,3.579285958,4,1,25.00014708,0,405.9133213,75.00044123,67.486422
331,9.640917568,15.25345664,3.579285958,4,1,25.00014708,0,405.9133213,75.00044123,67.4864
319331,9.640917568,15.25345664,3.579285958,4,1,25.00014708,0,405.9133213,75.00044123,67.48
070.489604,6321.696741,5.714319331,9.640917568,15.25345664,3.579285958,4,1,25.00014708,0,
70.489604,6321.696741,5.714319331,9.640917568,15.25345664,3.579285958,4,1,25.00014708,0,4
18,5.641042587,15.25345664,0,4,0,15.38714537,11.28208517,301.3900047,70.78086872,50.76938
18,5.641042587,15.25345664,0,4,0,15.38714537,11.28208517,301.3900047,70.78086872,50.76938
18.759237,6244.984202,6.924215418,5.641042587,15.25345664,0,4,0,15.38714537,11.28208517,3
4,0,1,0,1,1,11,6318.759237,6244.984202,6.924215418,5.641042587,15.25345664,0,4,0,15.3871453
318.759237,6244.984202,6.924215418,5.641042587,15.25345664,0,4,0,15.38714537,11.28208517,
4,0,1,0,1,1,11,6318.759237,6244.984202,6.924215418,5.641042587,15.25345664,0,4,0,15.3871453
924215418,5.641042587,15.25345664,0,4,0,15.38714537,11.28208517,301.3900047,70.78086872,
8.759237,6244.984202,6.924215418,5.641042587,15.25345664,0,4,0,15.38714537,11.28208517,30
924215418,5.641042587,15.25345664,0,4,0,15.38714537,11.28208517,301.3900047,70.78086872,
8.759237,6244.984202,6.924215418,5.641042587,15.25345664,0,4,0,15.38714537,11.28208517,30
4,0,1,0,1,1,11,6318.759237,6244.984202,6.924215418,5.641042587,15.25345664,0,4,0,15.3871453
8.759237,6244.984202,6.924215418,5.641042587,15.25345664,0,4,0,15.38714537,11.28208517,30
8.759237,6244.984202,6.924215418,5.641042587,15.25345664,0,4,0,15.38714537,11.28208517,30
924215418,5.641042587,15.25345664,0,4,0,15.38714537,11.28208517,301.3900047,70.78086872,
4,0,1,0,1,1,11,6318.759237,6244.984202,6.924215418,5.641042587,15.25345664,0,4,0,15.3871453
8.759237,6244.984202,6.924215418,5.641042587,15.25345664,0,4,0,15.38714537,11.28208517,30
418,5.641042587,15.25345664,0,4,0,15.38714537,11.28208517,301.3900047,70.78086872,50.7693
418,5.641042587,15.25345664,0,4,0,15.38714537,11.28208517,301.3900047,70.78086872,50.7693
18.759237,6244.984202,6.924215418,5.641042587,15.25345664,0,4,0,15.38714537,11.28208517,3
8.759237,6244.984202,6.924215418,5.641042587,15.25345664,0,4,0,15.38714537,11.28208517,30
18.759237,6244.984202,6.924215418,5.641042587,15.25345664,0,4,0,15.38714537,11.28208517,3
15418,5.641042587,15.25345664,0,4,0,15.38714537,11.28208517,301.3900047,70.78086872,50.76
18.759237,6244.984202,6.924215418,5.641042587,15.25345664,0,4,0,15.38714537,11.28208517,3
5418,5.641042587,15.25345664,0,4,0,15.38714537,11.28208517,301.3900047,70.78086872,50.769
8.759237,6244.984202,6.924215418,5.641042587,15.25345664,0,4,0,15.38714537,11.28208517,30
,6318.759237,6244.984202,6.924215418,5.641042587,15.25345664,0,4,0,15.38714537,11.2820851
18,5.641042587,15.25345664,0,4,0,15.38714537,11.28208517,301.3900047,70.78086872,50.76938
418,5.641042587,15.25345664,0,4,0,15.38714537,11.28208517,301.3900047,70.78086872,50.7693
8.759237,6244.984202,6.924215418,5.641042587,15.25345664,0,4,0,15.38714537,11.28208517,30
18.759237,6244.984202,6.924215418,5.641042587,15.25345664,0,4,0,15.38714537,11.28208517,3
8.759237,6244.984202,6.924215418,5.641042587,15.25345664,0,4,0,15.38714537,11.28208517,30
15418,5.641042587,15.25345664,0,4,0,15.38714537,11.28208517,301.3900047,70.78086872,50.76
8.759237,6244.984202,6.924215418,5.641042587,15.25345664,0,4,0,15.38714537,11.28208517,30
18.759237,6244.984202,6.924215418,5.641042587,15.25345664,0,4,0,15.38714537,11.28208517,3
418,5.641042587,15.25345664,0,4,0,15.38714537,11.28208517,301.3900047,70.78086872,50.7693

215418,5.641042587,15.25345664,0,4,0,15.38714537,11.28208517,301.3900047,70.78086872,50.7
420028,6.274353898,15.25345664,0,4,0,27.88371262,0,406.2354929,76.48104033,50.19483118,26.
733.220527,5884.297864,6.373420028,6.274353898,15.25345664,0,4,0,27.88371262,0,406.235492
3.220527,5884.297864,6.373420028,6.274353898,15.25345664,0,4,0,27.88371262,0,406.2354929,
3.220527,5884.297864,6.373420028,6.274353898,15.25345664,0,4,0,27.88371262,0,406.2354929,
382324,3,2,217,76,0,1,0,1,1,11,6733.220527,5884.297864,6.373420028,6.274353898,15.25345664,
028,6.274353898,15.25345664,0,4,0,27.88371262,0,406.2354929,76.48104033,50.19483118,26.290
733.220527,5884.297864,6.373420028,6.274353898,15.25345664,0,4,0,27.88371262,0,406.235492
3.220527,5884.297864,6.373420028,6.274353898,15.25345664,0,4,0,27.88371262,0,406.2354929,
20028,6.274353898,15.25345664,0,4,0,27.88371262,0,406.2354929,76.48104033,50.19483118,26.2
,0,1,0,1,1,11,6733.220527,5884.297864,6.373420028,6.274353898,15.25345664,0,4,0,27.88371262
420028,6.274353898,15.25345664,0,4,0,27.88371262,0,406.2354929,76.48104033,50.19483118,26.
3.220527,5884.297864,6.373420028,6.274353898,15.25345664,0,4,0,27.88371262,0,406.2354929,
3.220527,5884.297864,6.373420028,6.274353898,15.25345664,0,4,0,27.88371262,0,406.2354929,
028,6.274353898,15.25345664,0,4,0,27.88371262,0,406.2354929,76.48104033,50.19483118,26.290
3.220527,5884.297864,6.373420028,6.274353898,15.25345664,0,4,0,27.88371262,0,406.2354929,
33.220527,5884.297864,6.373420028,6.274353898,15.25345664,0,4,0,27.88371262,0,406.2354929
28,6.274353898,15.25345664,0,4,0,27.88371262,0,406.2354929,76.48104033,50.19483118,26.2903
733.220527,5884.297864,6.373420028,6.274353898,15.25345664,0,4,0,27.88371262,0,406.235492
028,6.274353898,15.25345664,0,4,0,27.88371262,0,406.2354929,76.48104033,50.19483118,26.290
73420028,6.274353898,15.25345664,0,4,0,27.88371262,0,406.2354929,76.48104033,50.19483118,
028,6.274353898,15.25345664,0,4,0,27.88371262,0,406.2354929,76.48104033,50.19483118,26.290
28,6.274353898,15.25345664,0,4,0,27.88371262,0,406.2354929,76.48104033,50.19483118,26.2903
733.220527,5884.297864,6.373420028,6.274353898,15.25345664,0,4,0,27.88371262,0,406.235492
028,6.274353898,15.25345664,0,4,0,27.88371262,0,406.2354929,76.48104033,50.19483118,26.290
3.220527,5884.297864,6.373420028,6.274353898,15.25345664,0,4,0,27.88371262,0,406.2354929,
3.220527,5884.297864,6.373420028,6.274353898,15.25345664,0,4,0,27.88371262,0,406.2354929,
3.220527,5884.297864,6.373420028,6.274353898,15.25345664,0,4,0,27.88371262,0,406.2354929,
028,6.274353898,15.25345664,0,4,0,27.88371262,0,406.2354929,76.48104033,50.19483118,26.290
526,5.905567886,15.25345664,0,4,0,27.23748792,5.905567886,395.7503572,74.70853831,64.9612
5.377771,5969.745349,6.225711526,5.905567886,15.25345664,0,4,0,27.23748792,5.905567886,39
5.377771,5969.745349,6.225711526,5.905567886,15.25345664,0,4,0,27.23748792,5.905567886,39
73,1,1,0,1,1,10,6715.377771,5969.745349,6.225711526,5.905567886,15.25345664,0,4,0,27.237487
526,5.905567886,15.25345664,0,4,0,27.23748792,5.905567886,395.7503572,74.70853831,64.9612
225711526,5.905567886,15.25345664,0,4,0,27.23748792,5.905567886,395.7503572,74.70853831,
73,1,1,0,1,1,10,6715.377771,5969.745349,6.225711526,5.905567886,15.25345664,0,4,0,27.237487
526,5.905567886,15.25345664,0,4,0,27.23748792,5.905567886,395.7503572,74.70853831,64.9612
5.377771,5969.745349,6.225711526,5.905567886,15.25345664,0,4,0,27.23748792,5.905567886,39
5.377771,5969.745349,6.225711526,5.905567886,15.25345664,0,4,0,27.23748792,5.905567886,39
11526,5.905567886,15.25345664,0,4,0,27.23748792,5.905567886,395.7503572,74.70853831,64.961
715.377771,5969.745349,6.225711526,5.905567886,15.25345664,0,4,0,27.23748792,5.905567886,
5.377771,5969.745349,6.225711526,5.905567886,15.25345664,0,4,0,27.23748792,5.905567886,39
526,5.905567886,15.25345664,0,4,0,27.23748792,5.905567886,395.7503572,74.70853831,64.9612
5.377771,5969.745349,6.225711526,5.905567886,15.25345664,0,4,0,27.23748792,5.905567886,39
5.377771,5969.745349,6.225711526,5.905567886,15.25345664,0,4,0,27.23748792,5.905567886,39
526,5.905567886,15.25345664,0,4,0,27.23748792,5.905567886,395.7503572,74.70853831,64.9612
5.377771,5969.745349,6.225711526,5.905567886,15.25345664,0,4,0,27.23748792,5.905567886,39
5.377771,5969.745349,6.225711526,5.905567886,15.25345664,0,4,0,27.23748792,5.905567886,39
225711526,5.905567886,15.25345664,0,4,0,27.23748792,5.905567886,395.7503572,74.70853831,6
5.377771,5969.745349,6.225711526,5.905567886,15.25345664,0,4,0,27.23748792,5.905567886,39

526,5.905567886,15.25345664,0,4,0,27.23748792,5.905567886,395.7503572,74.70853831,64.9612
5.377771,5969.745349,6.225711526,5.905567886,15.25345664,0,4,0,27.23748792,5.905567886,39
526,5.905567886,15.25345664,0,4,0,27.23748792,5.905567886,395.7503572,74.70853831,64.9612
715.377771,5969.745349,6.225711526,5.905567886,15.25345664,0,4,0,27.23748792,5.905567886,
526,5.905567886,15.25345664,0,4,0,27.23748792,5.905567886,395.7503572,74.70853831,64.9612
5.377771,5969.745349,6.225711526,5.905567886,15.25345664,0,4,0,27.23748792,5.905567886,39
5.377771,5969.745349,6.225711526,5.905567886,15.25345664,0,4,0,27.23748792,5.905567886,39
526,5.905567886,15.25345664,0,4,0,27.23748792,5.905567886,395.7503572,74.70853831,64.9612
711526,5.905567886,15.25345664,0,4,0,27.23748792,5.905567886,395.7503572,74.70853831,64.96
26,5.905567886,15.25345664,0,4,0,27.23748792,5.905567886,395.7503572,74.70853831,64.96124
526,5.905567886,15.25345664,0,4,0,27.23748792,5.905567886,395.7503572,74.70853831,64.9612
526,5.905567886,15.25345664,0,4,0,27.23748792,5.905567886,395.7503572,74.70853831,64.9612
5.377771,5969.745349,6.225711526,5.905567886,15.25345664,0,4,0,27.23748792,5.905567886,39
526,5.905567886,15.25345664,0,4,0,27.23748792,5.905567886,395.7503572,74.70853831,64.9612
711526,5.905567886,15.25345664,0,4,0,27.23748792,5.905567886,395.7503572,74.70853831,64.96
5.377771,5969.745349,6.225711526,5.905567886,15.25345664,0,4,0,27.23748792,5.905567886,39
715.377771,5969.745349,6.225711526,5.905567886,15.25345664,0,4,0,27.23748792,5.905567886,
5.377771,5969.745349,6.225711526,5.905567886,15.25345664,0,4,0,27.23748792,5.905567886,39
5.377771,5969.745349,6.225711526,5.905567886,15.25345664,0,4,0,27.23748792,5.905567886,39
121,6.413678016,15.25345664,0,4,0,27.48493326,0,413.1314071,75.98775667,51.30942412,26.676
0.192015,5928.880252,6.467043121,6.413678016,15.25345664,0,4,0,27.48493326,0,413.1314071,
0.192015,5928.880252,6.467043121,6.413678016,15.25345664,0,4,0,27.48493326,0,413.1314071,
81,0,1,0,1,1,12,6690.192015,5928.880252,6.467043121,6.413678016,15.25345664,0,4,0,27.484933
121,6.413678016,15.25345664,0,4,0,27.48493326,0,413.1314071,75.98775667,51.30942412,26.676
043121,6.413678016,15.25345664,0,4,0,27.48493326,0,413.1314071,75.98775667,51.30942412,26.
81,0,1,0,1,1,12,6690.192015,5928.880252,6.467043121,6.413678016,15.25345664,0,4,0,27.484933
121,6.413678016,15.25345664,0,4,0,27.48493326,0,413.1314071,75.98775667,51.30942412,26.676
121,6.413678016,15.25345664,0,4,0,27.48493326,0,413.1314071,75.98775667,51.30942412,26.676
043121,6.413678016,15.25345664,0,4,0,27.48493326,0,413.1314071,75.98775667,51.30942412,26.
043121,6.413678016,15.25345664,0,4,0,27.48493326,0,413.1314071,75.98775667,51.30942412,26.
690.192015,5928.880252,6.467043121,6.413678016,15.25345664,0,4,0,27.48493326,0,413.131407
0.192015,5928.880252,6.467043121,6.413678016,15.25345664,0,4,0,27.48493326,0,413.1314071,
043121,6.413678016,15.25345664,0,4,0,27.48493326,0,413.1314071,75.98775667,51.30942412,26.
67043121,6.413678016,15.25345664,0,4,0,27.48493326,0,413.1314071,75.98775667,51.30942412,2
43121,6.413678016,15.25345664,0,4,0,27.48493326,0,413.1314071,75.98775667,51.30942412,26.6
0.192015,5928.880252,6.467043121,6.413678016,15.25345664,0,4,0,27.48493326,0,413.1314071,
121,6.413678016,15.25345664,0,4,0,27.48493326,0,413.1314071,75.98775667,51.30942412,26.676
0.192015,5928.880252,6.467043121,6.413678016,15.25345664,0,4,0,27.48493326,0,413.1314071,
0.192015,5928.880252,6.467043121,6.413678016,15.25345664,0,4,0,27.48493326,0,413.1314071,
043121,6.413678016,15.25345664,0,4,0,27.48493326,0,413.1314071,75.98775667,51.30942412,26.
121,6.413678016,15.25345664,0,4,0,27.48493326,0,413.1314071,75.98775667,51.30942412,26.676
0.192015,5928.880252,6.467043121,6.413678016,15.25345664,0,4,0,27.48493326,0,413.1314071,
121,6.413678016,15.25345664,0,4,0,27.48493326,0,413.1314071,75.98775667,51.30942412,26.676
0.192015,5928.880252,6.467043121,6.413678016,15.25345664,0,4,0,27.48493326,0,413.1314071,
043121,6.413678016,15.25345664,0,4,0,27.48493326,0,413.1314071,75.98775667,51.30942412,26.
0.192015,5928.880252,6.467043121,6.413678016,15.25345664,0,4,0,27.48493326,0,413.1314071,
121,6.413678016,15.25345664,0,4,0,27.48493326,0,413.1314071,75.98775667,51.30942412,26.676
0.192015,5928.880252,6.467043121,6.413678016,15.25345664,0,4,0,27.48493326,0,413.1314071,
0.192015,5928.880252,6.467043121,6.413678016,15.25345664,0,4,0,27.48493326,0,413.1314071,
467043121,6.413678016,15.25345664,0,4,0,27.48493326,0,413.1314071,75.98775667,51.30942412

121,6.413678016,15.25345664,0,4,0,27.48493326,0,413.1314071,75.98775667,51.30942412,26.676
690.192015,5928.880252,6.467043121,6.413678016,15.25345664,0,4,0,27.48493326,0,413.131407
0.192015,5928.880252,6.467043121,6.413678016,15.25345664,0,4,0,27.48493326,0,413.1314071,
121,6.413678016,15.25345664,0,4,0,27.48493326,0,413.1314071,75.98775667,51.30942412,26.676
0.192015,5928.880252,6.467043121,6.413678016,15.25345664,0,4,0,27.48493326,0,413.1314071,
121,6.413678016,15.25345664,0,4,0,27.48493326,0,413.1314071,75.98775667,51.30942412,26.676
0.192015,5928.880252,6.467043121,6.413678016,15.25345664,0,4,0,27.48493326,0,413.1314071,
690.192015,5928.880252,6.467043121,6.413678016,15.25345664,0,4,0,27.48493326,0,413.131407
32421,5.45904552,15.25345664,0,4,0,15.41618316,10.91809104,280.1350133,69.37282421,49.1314
3,0,1,0,1,1,11,6238.843127,6192.89729,6.937282421,5.45904552,15.25345664,0,4,0,15.41618316,
32421,5.45904552,15.25345664,0,4,0,15.41618316,10.91809104,280.1350133,69.37282421,49.1314
38.843127,6192.89729,6.937282421,5.45904552,15.25345664,0,4,0,15.41618316,10.91809104,280
21,5.45904552,15.25345664,0,4,0,15.41618316,10.91809104,280.1350133,69.37282421,49.131409
1,5.45904552,15.25345664,0,4,0,15.41618316,10.91809104,280.1350133,69.37282421,49.1314096
32421,5.45904552,15.25345664,0,4,0,15.41618316,10.91809104,280.1350133,69.37282421,49.1314
37282421,5.45904552,15.25345664,0,4,0,15.41618316,10.91809104,280.1350133,69.37282421,49.
37282421,5.45904552,15.25345664,0,4,0,15.41618316,10.91809104,280.1350133,69.37282421,49.
3,0,1,0,1,1,11,6238.843127,6192.89729,6.937282421,5.45904552,15.25345664,0,4,0,15.41618316,
38.843127,6192.89729,6.937282421,5.45904552,15.25345664,0,4,0,15.41618316,10.91809104,280
238.843127,6192.89729,6.937282421,5.45904552,15.25345664,0,4,0,15.41618316,10.91809104,28
382324,3,2,217,73,0,1,0,1,1,11,6238.843127,6192.89729,6.937282421,5.45904552,15.25345664,0,4
3,0,1,0,1,1,11,6238.843127,6192.89729,6.937282421,5.45904552,15.25345664,0,4,0,15.41618316,
8.843127,6192.89729,6.937282421,5.45904552,15.25345664,0,4,0,15.41618316,10.91809104,280.
8.843127,6192.89729,6.937282421,5.45904552,15.25345664,0,4,0,15.41618316,10.91809104,280.
32421,5.45904552,15.25345664,0,4,0,15.41618316,10.91809104,280.1350133,69.37282421,49.1314
8.843127,6192.89729,6.937282421,5.45904552,15.25345664,0,4,0,15.41618316,10.91809104,280.
8.843127,6192.89729,6.937282421,5.45904552,15.25345664,0,4,0,15.41618316,10.91809104,280.
238.843127,6192.89729,6.937282421,5.45904552,15.25345664,0,4,0,15.41618316,10.91809104,28
5238.843127,6192.89729,6.937282421,5.45904552,15.25345664,0,4,0,15.41618316,10.91809104,28
2421,5.45904552,15.25345664,0,4,0,15.41618316,10.91809104,280.1350133,69.37282421,49.1314
21,5.45904552,15.25345664,0,4,0,15.41618316,10.91809104,280.1350133,69.37282421,49.131409
3,0,1,0,1,1,11,6238.843127,6192.89729,6.937282421,5.45904552,15.25345664,0,4,0,15.41618316,
8.843127,6192.89729,6.937282421,5.45904552,15.25345664,0,4,0,15.41618316,10.91809104,280.
8.843127,6192.89729,6.937282421,5.45904552,15.25345664,0,4,0,15.41618316,10.91809104,280.
21,5.45904552,15.25345664,0,4,0,15.41618316,10.91809104,280.1350133,69.37282421,49.131409
21,5.45904552,15.25345664,0,4,0,15.41618316,10.91809104,280.1350133,69.37282421,49.131409
8.843127,6192.89729,6.937282421,5.45904552,15.25345664,0,4,0,15.41618316,10.91809104,280.
238.843127,6192.89729,6.937282421,5.45904552,15.25345664,0,4,0,15.41618316,10.91809104,28
238.843127,6192.89729,6.937282421,5.45904552,15.25345664,0,4,0,15.41618316,10.91809104,28
335092,4.795450847,29.39298478,3.579285958,5,1,27.84134037,9.590901693,305.5400506,82.258
092,4.795450847,29.39298478,3.579285958,5,1,27.84134037,9.590901693,305.5400506,82.258505
98.075199,8995.118758,6.960335092,4.795450847,29.39298478,3.579285958,5,1,27.84134037,9.5
298.075199,8995.118758,6.960335092,4.795450847,29.39298478,3.579285958,5,1,27.84134037,9.
8.075199,8995.118758,6.960335092,4.795450847,29.39298478,3.579285958,5,1,27.84134037,9.59
298.075199,8995.118758,6.960335092,4.795450847,29.39298478,3.579285958,5,1,27.84134037,9.
092,4.795450847,29.39298478,3.579285958,5,1,27.84134037,9.590901693,305.5400506,82.258505
960335092,4.795450847,29.39298478,3.579285958,5,1,27.84134037,9.590901693,305.5400506,82.
8,1,1,0,1,1,12,9298.075199,8995.118758,6.960335092,4.795450847,29.39298478,3.579285958,5,1,
8.075199,8995.118758,6.960335092,4.795450847,29.39298478,3.579285958,5,1,27.84134037,9.59
960335092,4.795450847,29.39298478,3.579285958,5,1,27.84134037,9.590901693,305.5400506,82

8,1,1,0,1,1,12,9298.075199,8995.118758,6.960335092,4.795450847,29.39298478,3.579285958,5,1,
98.075199,8995.118758,6.960335092,4.795450847,29.39298478,3.579285958,5,1,27.84134037,9.5
8,1,1,0,1,1,12,9298.075199,8995.118758,6.960335092,4.795450847,29.39298478,3.579285958,5,1,
,9298.075199,8995.118758,6.960335092,4.795450847,29.39298478,3.579285958,5,1,27.84134037,
092,4.795450847,29.39298478,3.579285958,5,1,27.84134037,9.590901693,305.5400506,82.258505
60335092,4.795450847,29.39298478,3.579285958,5,1,27.84134037,9.590901693,305.5400506,82.2
8,1,1,0,1,1,12,9298.075199,8995.118758,6.960335092,4.795450847,29.39298478,3.579285958,5,1,
8,1,1,0,1,1,12,9298.075199,8995.118758,6.960335092,4.795450847,29.39298478,3.579285958,5,1,
298.075199,8995.118758,6.960335092,4.795450847,29.39298478,3.579285958,5,1,27.84134037,9.
335092,4.795450847,29.39298478,3.579285958,5,1,27.84134037,9.590901693,305.5400506,82.258
98.075199,8995.118758,6.960335092,4.795450847,29.39298478,3.579285958,5,1,27.84134037,9.5
98.075199,8995.118758,6.960335092,4.795450847,29.39298478,3.579285958,5,1,27.84134037,9.5
98.075199,8995.118758,6.960335092,4.795450847,29.39298478,3.579285958,5,1,27.84134037,9.5
298.075199,8995.118758,6.960335092,4.795450847,29.39298478,3.579285958,5,1,27.84134037,9.
3,0,19.91707628,0,3,0,32.09710211,34.08329335,242.167884,81.70171446,102.2498801,31.367622
33,0,19.91707628,0,3,0,32.09710211,34.08329335,242.167884,81.70171446,102.2498801,31.36762
.015989,9642.992356,7.294795933,0,19.91707628,0,3,0,32.09710211,34.08329335,242.167884,81.
33,0,19.91707628,0,3,0,32.09710211,34.08329335,242.167884,81.70171446,102.2498801,31.36762
33,0,19.91707628,0,3,0,32.09710211,34.08329335,242.167884,81.70171446,102.2498801,31.36762
33,0,19.91707628,0,3,0,32.09710211,34.08329335,242.167884,81.70171446,102.2498801,31.36762
.015989,9642.992356,7.294795933,0,19.91707628,0,3,0,32.09710211,34.08329335,242.167884,81.
17.015989,9642.992356,7.294795933,0,19.91707628,0,3,0,32.09710211,34.08329335,242.167884,8
95933,0,19.91707628,0,3,0,32.09710211,34.08329335,242.167884,81.70171446,102.2498801,31.36
.015989,9642.992356,7.294795933,0,19.91707628,0,3,0,32.09710211,34.08329335,242.167884,81.
33,0,19.91707628,0,3,0,32.09710211,34.08329335,242.167884,81.70171446,102.2498801,31.36762
.015989,9642.992356,7.294795933,0,19.91707628,0,3,0,32.09710211,34.08329335,242.167884,81.
33,0,19.91707628,0,3,0,32.09710211,34.08329335,242.167884,81.70171446,102.2498801,31.36762
7.015989,9642.992356,7.294795933,0,19.91707628,0,3,0,32.09710211,34.08329335,242.167884,8
33,0,19.91707628,0,3,0,32.09710211,34.08329335,242.167884,81.70171446,102.2498801,31.36762
33,0,19.91707628,0,3,0,32.09710211,34.08329335,242.167884,81.70171446,102.2498801,31.36762
.015989,9642.992356,7.294795933,0,19.91707628,0,3,0,32.09710211,34.08329335,242.167884,81.
.015989,9642.992356,7.294795933,0,19.91707628,0,3,0,32.09710211,34.08329335,242.167884,81.
.015989,9642.992356,7.294795933,0,19.91707628,0,3,0,32.09710211,34.08329335,242.167884,81.
33,0,19.91707628,0,3,0,32.09710211,34.08329335,242.167884,81.70171446,102.2498801,31.36762
95933,0,19.91707628,0,3,0,32.09710211,34.08329335,242.167884,81.70171446,102.2498801,31.36
7.015989,9642.992356,7.294795933,0,19.91707628,0,3,0,32.09710211,34.08329335,242.167884,8
94795933,0,19.91707628,0,3,0,32.09710211,34.08329335,242.167884,81.70171446,102.2498801,3
95933,0,19.91707628,0,3,0,32.09710211,34.08329335,242.167884,81.70171446,102.2498801,31.36
95933,0,19.91707628,0,3,0,32.09710211,34.08329335,242.167884,81.70171446,102.2498801,31.36
95933,0,19.91707628,0,3,0,32.09710211,34.08329335,242.167884,81.70171446,102.2498801,31.36
7.015989,9642.992356,7.294795933,0,19.91707628,0,3,0,32.09710211,34.08329335,242.167884,8
33,0,19.91707628,0,3,0,32.09710211,34.08329335,242.167884,81.70171446,102.2498801,31.36762
33,0,19.91707628,0,3,0,32.09710211,34.08329335,242.167884,81.70171446,102.2498801,31.36762
33,0,19.91707628,0,3,0,32.09710211,34.08329335,242.167884,81.70171446,102.2498801,31.36762
54,0,19.91707628,0,3,0,31.42437847,36.59842846,296.4198648,77.73398886,134.1942377,33.0782
15954,0,19.91707628,0,3,0,31.42437847,36.59842846,296.4198648,77.73398886,134.1942377,33.0
54,0,19.91707628,0,3,0,31.42437847,36.59842846,296.4198648,77.73398886,134.1942377,33.0782
6.30886,10482.39374,7.442615954,0,19.91707628,0,3,0,31.42437847,36.59842846,296.4198648,7
4,0,1,0,1,1,12,9856.30886,10482.39374,7.442615954,0,19.91707628,0,3,0,31.42437847,36.59842
54,0,19.91707628,0,3,0,31.42437847,36.59842846,296.4198648,77.73398886,134.1942377,33.0782

4,0,19.91707628,0,3,0,31.42437847,36.59842846,296.4198648,77.73398886,134.1942377,33.0782
6.30886,10482.39374,7.442615954,0,19.91707628,0,3,0,31.42437847,36.59842846,296.4198648,7
856.30886,10482.39374,7.442615954,0,19.91707628,0,3,0,31.42437847,36.59842846,296.4198648
,0,19.91707628,0,3,0,31.42437847,36.59842846,296.4198648,77.73398886,134.1942377,33.07829
56.30886,10482.39374,7.442615954,0,19.91707628,0,3,0,31.42437847,36.59842846,296.4198648,
6.30886,10482.39374,7.442615954,0,19.91707628,0,3,0,31.42437847,36.59842846,296.4198648,7
4,0,1,0,1,1,12,9856.30886,10482.39374,7.442615954,0,19.91707628,0,3,0,31.42437847,36.598428
4,0,19.91707628,0,3,0,31.42437847,36.59842846,296.4198648,77.73398886,134.1942377,33.0782
856.30886,10482.39374,7.442615954,0,19.91707628,0,3,0,31.42437847,36.59842846,296.4198648
6.30886,10482.39374,7.442615954,0,19.91707628,0,3,0,31.42437847,36.59842846,296.4198648,7
4,0,19.91707628,0,3,0,31.42437847,36.59842846,296.4198648,77.73398886,134.1942377,33.0782
4,0,1,0,1,1,12,9856.30886,10482.39374,7.442615954,0,19.91707628,0,3,0,31.42437847,36.598428
954,0,19.91707628,0,3,0,31.42437847,36.59842846,296.4198648,77.73398886,134.1942377,33.07
4,0,19.91707628,0,3,0,31.42437847,36.59842846,296.4198648,77.73398886,134.1942377,33.0782
4,0,1,0,1,1,12,9856.30886,10482.39374,7.442615954,0,19.91707628,0,3,0,31.42437847,36.598428
856.30886,10482.39374,7.442615954,0,19.91707628,0,3,0,31.42437847,36.59842846,296.4198648
4,0,19.91707628,0,3,0,31.42437847,36.59842846,296.4198648,77.73398886,134.1942377,33.0782
5954,0,19.91707628,0,3,0,31.42437847,36.59842846,296.4198648,77.73398886,134.1942377,33.0
6.30886,10482.39374,7.442615954,0,19.91707628,0,3,0,31.42437847,36.59842846,296.4198648,7
4,0,19.91707628,0,3,0,31.42437847,36.59842846,296.4198648,77.73398886,134.1942377,33.0782
56.30886,10482.39374,7.442615954,0,19.91707628,0,3,0,31.42437847,36.59842846,296.4198648,
4,0,19.91707628,0,3,0,31.42437847,36.59842846,296.4198648,77.73398886,134.1942377,33.0782
5954,0,19.91707628,0,3,0,31.42437847,36.59842846,296.4198648,77.73398886,134.1942377,33.0
5954,0,19.91707628,0,3,0,31.42437847,36.59842846,296.4198648,77.73398886,134.1942377,33.0
856.30886,10482.39374,7.442615954,0,19.91707628,0,3,0,31.42437847,36.59842846,296.4198648
56.30886,10482.39374,7.442615954,0,19.91707628,0,3,0,31.42437847,36.59842846,296.4198648,
84,0,1,0,1,1,12,9856.30886,10482.39374,7.442615954,0,19.91707628,0,3,0,31.42437847,36.59842
,0,19.91707628,0,3,0,31.42437847,36.59842846,296.4198648,77.73398886,134.1942377,33.07829
954,0,19.91707628,0,3,0,31.42437847,36.59842846,296.4198648,77.73398886,134.1942377,33.07
,0,19.91707628,0,3,0,31.42437847,36.59842846,296.4198648,77.73398886,134.1942377,33.07829
4,1,1,0,1,1,8,9856.30886,10482.39374,7.442615954,0,19.91707628,0,3,0,31.42437847,36.5984284
,0,19.91707628,0,3,0,31.42437847,36.59842846,296.4198648,77.73398886,134.1942377,33.07829
4,1,1,0,1,1,8,9856.30886,10482.39374,7.442615954,0,19.91707628,0,3,0,31.42437847,36.5984284
.30886,10482.39374,7.442615954,0,19.91707628,0,3,0,31.42437847,36.59842846,296.4198648,77.
0,19.91707628,0,3,0,31.42437847,36.59842846,296.4198648,77.73398886,134.1942377,33.078293
,0,19.91707628,0,3,0,31.42437847,36.59842846,296.4198648,77.73398886,134.1942377,33.07829
954,0,19.91707628,0,3,0,31.42437847,36.59842846,296.4198648,77.73398886,134.1942377,33.07
.30886,10482.39374,7.442615954,0,19.91707628,0,3,0,31.42437847,36.59842846,296.4198648,77.
6.30886,10482.39374,7.442615954,0,19.91707628,0,3,0,31.42437847,36.59842846,296.4198648,7
954,0,19.91707628,0,3,0,31.42437847,36.59842846,296.4198648,77.73398886,134.1942377,33.078
6.30886,10482.39374,7.442615954,0,19.91707628,0,3,0,31.42437847,36.59842846,296.4198648,7
6.30886,10482.39374,7.442615954,0,19.91707628,0,3,0,31.42437847,36.59842846,296.4198648,7
.30886,10482.39374,7.442615954,0,19.91707628,0,3,0,31.42437847,36.59842846,296.4198648,77.
.30886,10482.39374,7.442615954,0,19.91707628,0,3,0,31.42437847,36.59842846,296.4198648,77.
6.30886,10482.39374,7.442615954,0,19.91707628,0,3,0,31.42437847,36.59842846,296.4198648,7
4,1,1,0,1,1,8,9856.30886,10482.39374,7.442615954,0,19.91707628,0,3,0,31.42437847,36.5984284
4,0,19.91707628,0,3,0,31.42437847,36.59842846,296.4198648,77.73398886,134.1942377,33.0782
6.30886,10482.39374,7.442615954,0,19.91707628,0,3,0,31.42437847,36.59842846,296.4198648,7
0,19.91707628,0,3,0,31.42437847,36.59842846,296.4198648,77.73398886,134.1942377,33.078293
6.30886,10482.39374,7.442615954,0,19.91707628,0,3,0,31.42437847,36.59842846,296.4198648,7

56.30886,10482.39374,7.442615954,0,19.91707628,0,3,0,31.42437847,36.59842846,296.4198648,
.30886,10482.39374,7.442615954,0,19.91707628,0,3,0,31.42437847,36.59842846,296.4198648,77.
56.30886,10482.39374,7.442615954,0,19.91707628,0,3,0,31.42437847,36.59842846,296.4198648,
954,0,19.91707628,0,3,0,31.42437847,36.59842846,296.4198648,77.73398886,134.1942377,33.078
4,1,1,0,1,1,8,9856.30886,10482.39374,7.442615954,0,19.91707628,0,3,0,31.42437847,36.5984284
.30886,10482.39374,7.442615954,0,19.91707628,0,3,0,31.42437847,36.59842846,296.4198648,77.
2615954,0,19.91707628,0,3,0,31.42437847,36.59842846,296.4198648,77.73398886,134.1942377,3
4,0,19.91707628,0,3,0,31.42437847,36.59842846,296.4198648,77.73398886,134.1942377,33.07829
5954,0,19.91707628,0,3,0,31.42437847,36.59842846,296.4198648,77.73398886,134.1942377,33.07
53,9.015873668,31.03825582,3.579285958,5,1,31.10430668,9.015873668,370.5641308,84.2092205
.436902,7499.037717,7.586416263,9.015873668,31.03825582,3.579285958,5,1,31.10430668,9.015
2.436902,7499.037717,7.586416263,9.015873668,31.03825582,3.579285958,5,1,31.10430668,9.01
5263,9.015873668,31.03825582,3.579285958,5,1,31.10430668,9.015873668,370.5641308,84.20922
3,9.015873668,31.03825582,3.579285958,5,1,31.10430668,9.015873668,370.5641308,84.20922052
.436902,7499.037717,7.586416263,9.015873668,31.03825582,3.579285958,5,1,31.10430668,9.015
81,1,1,1,0,1,7,7962.436902,7499.037717,7.586416263,9.015873668,31.03825582,3.579285958,5,1,
62.436902,7499.037717,7.586416263,9.015873668,31.03825582,3.579285958,5,1,31.10430668,9.0
62.436902,7499.037717,7.586416263,9.015873668,31.03825582,3.579285958,5,1,31.10430668,9.0
62.436902,7499.037717,7.586416263,9.015873668,31.03825582,3.579285958,5,1,31.10430668,9.0
.436902,7499.037717,7.586416263,9.015873668,31.03825582,3.579285958,5,1,31.10430668,9.015
62.436902,7499.037717,7.586416263,9.015873668,31.03825582,3.579285958,5,1,31.10430668,9.0
2.436902,7499.037717,7.586416263,9.015873668,31.03825582,3.579285958,5,1,31.10430668,9.01
53,9.015873668,31.03825582,3.579285958,5,1,31.10430668,9.015873668,370.5641308,84.2092205
53,9.015873668,31.03825582,3.579285958,5,1,31.10430668,9.015873668,370.5641308,84.2092205
62.436902,7499.037717,7.586416263,9.015873668,31.03825582,3.579285958,5,1,31.10430668,9.0
.436902,7499.037717,7.586416263,9.015873668,31.03825582,3.579285958,5,1,31.10430668,9.015
.436902,7499.037717,7.586416263,9.015873668,31.03825582,3.579285958,5,1,31.10430668,9.015
62.436902,7499.037717,7.586416263,9.015873668,31.03825582,3.579285958,5,1,31.10430668,9.0
5263,9.015873668,31.03825582,3.579285958,5,1,31.10430668,9.015873668,370.5641308,84.20922
53,9.015873668,31.03825582,3.579285958,5,1,31.10430668,9.015873668,370.5641308,84.2092205
62.436902,7499.037717,7.586416263,9.015873668,31.03825582,3.579285958,5,1,31.10430668,9.0
1,1,1,1,0,1,7,7962.436902,7499.037717,7.586416263,9.015873668,31.03825582,3.579285958,5,1,3
53,9.015873668,31.03825582,3.579285958,5,1,31.10430668,9.015873668,370.5641308,84.2092205
5416263,9.015873668,31.03825582,3.579285958,5,1,31.10430668,9.015873668,370.5641308,84.20
.436902,7499.037717,7.586416263,9.015873668,31.03825582,3.579285958,5,1,31.10430668,9.015
62.436902,7499.037717,7.586416263,9.015873668,31.03825582,3.579285958,5,1,31.10430668,9.0
53,9.015873668,31.03825582,3.579285958,5,1,31.10430668,9.015873668,370.5641308,84.2092205
.436902,7499.037717,7.586416263,9.015873668,31.03825582,3.579285958,5,1,31.10430668,9.015
5263,9.015873668,31.03825582,3.579285958,5,1,31.10430668,9.015873668,370.5641308,84.20922
1,1,1,1,0,1,7,7962.436902,7499.037717,7.586416263,9.015873668,31.03825582,3.579285958,5,1,3
.436902,7499.037717,7.586416263,9.015873668,31.03825582,3.579285958,5,1,31.10430668,9.015
2.436902,7499.037717,7.586416263,9.015873668,31.03825582,3.579285958,5,1,31.10430668,9.01
53,9.015873668,31.03825582,3.579285958,5,1,31.10430668,9.015873668,370.5641308,84.2092205
11.31195642,28.76111656,11.12117954,5,2,17.21800517,15.08260856,279.5717928,82.11664002,
.960078711,11.31195642,28.76111656,11.12117954,5,2,17.21800517,15.08260856,279.5717928,82
90,1,1,0,1,1,7,6800.099454,6688.081755,5.960078711,11.31195642,28.76111656,11.12117954,5,2,
.099454,6688.081755,5.960078711,11.31195642,28.76111656,11.12117954,5,2,17.21800517,15.08
.099454,6688.081755,5.960078711,11.31195642,28.76111656,11.12117954,5,2,17.21800517,15.08
78711,11.31195642,28.76111656,11.12117954,5,2,17.21800517,15.08260856,279.5717928,82.1166
.099454,6688.081755,5.960078711,11.31195642,28.76111656,11.12117954,5,2,17.21800517,15.08

0078711,11.31195642,28.76111656,11.12117954,5,2,17.21800517,15.08260856,279.5717928,82.11
.099454,6688.081755,5.960078711,11.31195642,28.76111656,11.12117954,5,2,17.21800517,15.08
1,11.31195642,28.76111656,11.12117954,5,2,17.21800517,15.08260856,279.5717928,82.11664002
00.099454,6688.081755,5.960078711,11.31195642,28.76111656,11.12117954,5,2,17.21800517,15.
.099454,6688.081755,5.960078711,11.31195642,28.76111656,11.12117954,5,2,17.21800517,15.08
.099454,6688.081755,5.960078711,11.31195642,28.76111656,11.12117954,5,2,17.21800517,15.08
.099454,6688.081755,5.960078711,11.31195642,28.76111656,11.12117954,5,2,17.21800517,15.08
1.31195642,28.76111656,11.12117954,5,2,17.21800517,15.08260856,279.5717928,82.11664002,7
.099454,6688.081755,5.960078711,11.31195642,28.76111656,11.12117954,5,2,17.21800517,15.08
.099454,6688.081755,5.960078711,11.31195642,28.76111656,11.12117954,5,2,17.21800517,15.08
.099454,6688.081755,5.960078711,11.31195642,28.76111656,11.12117954,5,2,17.21800517,15.08
78711,11.31195642,28.76111656,11.12117954,5,2,17.21800517,15.08260856,279.5717928,82.1166
1.31195642,28.76111656,11.12117954,5,2,17.21800517,15.08260856,279.5717928,82.11664002,7
78711,11.31195642,28.76111656,11.12117954,5,2,17.21800517,15.08260856,279.5717928,82.1166
11,11.31195642,28.76111656,11.12117954,5,2,17.21800517,15.08260856,279.5717928,82.1166400
.099454,6688.081755,5.960078711,11.31195642,28.76111656,11.12117954,5,2,17.21800517,15.08
11,11.31195642,28.76111656,11.12117954,5,2,17.21800517,15.08260856,279.5717928,82.1166400
1.31195642,28.76111656,11.12117954,5,2,17.21800517,15.08260856,279.5717928,82.11664002,7
.099454,6688.081755,5.960078711,11.31195642,28.76111656,11.12117954,5,2,17.21800517,15.08
0078711,11.31195642,28.76111656,11.12117954,5,2,17.21800517,15.08260856,279.5717928,82.11
0.099454,6688.081755,5.960078711,11.31195642,28.76111656,11.12117954,5,2,17.21800517,15.0
11,11.31195642,28.76111656,11.12117954,5,2,17.21800517,15.08260856,279.5717928,82.1166400
1.31195642,28.76111656,11.12117954,5,2,17.21800517,15.08260856,279.5717928,82.11664002,7
78711,11.31195642,28.76111656,11.12117954,5,2,17.21800517,15.08260856,279.5717928,82.1166
0078711,11.31195642,28.76111656,11.12117954,5,2,17.21800517,15.08260856,279.5717928,82.11
.099454,6688.081755,5.960078711,11.31195642,28.76111656,11.12117954,5,2,17.21800517,15.08
11,11.31195642,28.76111656,11.12117954,5,2,17.21800517,15.08260856,279.5717928,82.1166400
60078711,11.31195642,28.76111656,11.12117954,5,2,17.21800517,15.08260856,279.5717928,82.1
7,90,1,1,0,1,1,7,6800.099454,6688.081755,5.960078711,11.31195642,28.76111656,11.12117954,5,
00.099454,6688.081755,5.960078711,11.31195642,28.76111656,11.12117954,5,2,17.21800517,15.
11,11.31195642,28.76111656,11.12117954,5,2,17.21800517,15.08260856,279.5717928,82.1166400
00.099454,6688.081755,5.960078711,11.31195642,28.76111656,11.12117954,5,2,17.21800517,15.
00.099454,6688.081755,5.960078711,11.31195642,28.76111656,11.12117954,5,2,17.21800517,15.
.099454,6688.081755,5.960078711,11.31195642,28.76111656,11.12117954,5,2,17.21800517,15.08
.099454,6688.081755,5.960078711,11.31195642,28.76111656,11.12117954,5,2,17.21800517,15.08
11.31195642,28.76111656,11.12117954,5,2,17.21800517,15.08260856,279.5717928,82.11664002,7
6800.099454,6688.081755,5.960078711,11.31195642,28.76111656,11.12117954,5,2,17.21800517,1
.099454,6688.081755,5.960078711,11.31195642,28.76111656,11.12117954,5,2,17.21800517,15.08
.099454,6688.081755,5.960078711,11.31195642,28.76111656,11.12117954,5,2,17.21800517,15.08
.099454,6688.081755,5.960078711,11.31195642,28.76111656,11.12117954,5,2,17.21800517,15.08
11,11.31195642,28.76111656,11.12117954,5,2,17.21800517,15.08260856,279.5717928,82.1166400
00.099454,6688.081755,5.960078711,11.31195642,28.76111656,11.12117954,5,2,17.21800517,15.
.099454,6688.081755,5.960078711,11.31195642,28.76111656,11.12117954,5,2,17.21800517,15.08
11,11.31195642,28.76111656,11.12117954,5,2,17.21800517,15.08260856,279.5717928,82.1166400
00.099454,6688.081755,5.960078711,11.31195642,28.76111656,11.12117954,5,2,17.21800517,15.
78711,11.31195642,28.76111656,11.12117954,5,2,17.21800517,15.08260856,279.5717928,82.1166
11,11.31195642,28.76111656,11.12117954,5,2,17.21800517,15.08260856,279.5717928,82.1166400
11,11.31195642,28.76111656,11.12117954,5,2,17.21800517,15.08260856,279.5717928,82.1166400
.099454,6688.081755,5.960078711,11.31195642,28.76111656,11.12117954,5,2,17.21800517,15.08
11,11.31195642,28.76111656,11.12117954,5,2,17.21800517,15.08260856,279.5717928,82.1166400

11,11.31195642,28.76111656,11.12117954,5,2,17.21800517,15.08260856,279.5717928,82.1166400
.099454,6688.081755,5.960078711,11.31195642,28.76111656,11.12117954,5,2,17.21800517,15.08
.099454,6688.081755,5.960078711,11.31195642,28.76111656,11.12117954,5,2,17.21800517,15.08
00.099454,6688.081755,5.960078711,11.31195642,28.76111656,11.12117954,5,2,17.21800517,15.
.099454,6688.081755,5.960078711,11.31195642,28.76111656,11.12117954,5,2,17.21800517,15.08
11.31195642,28.76111656,11.12117954,5,2,17.21800517,15.08260856,279.5717928,82.11664002,
0,1,1,0,1,1,7,6800.099454,6688.081755,5.960078711,11.31195642,28.76111656,11.12117954,5,2,1
6800.099454,6688.081755,5.960078711,11.31195642,28.76111656,11.12117954,5,2,17.21800517,1
00.099454,6688.081755,5.960078711,11.31195642,28.76111656,11.12117954,5,2,17.21800517,15.0
78711,11.31195642,28.76111656,11.12117954,5,2,17.21800517,15.08260856,279.5717928,82.1166
78711,11.31195642,28.76111656,11.12117954,5,2,17.21800517,15.08260856,279.5717928,82.1166
00.099454,6688.081755,5.960078711,11.31195642,28.76111656,11.12117954,5,2,17.21800517,15.
78711,11.31195642,28.76111656,11.12117954,5,2,17.21800517,15.08260856,279.5717928,82.1166
1,11.31195642,28.76111656,11.12117954,5,2,17.21800517,15.08260856,279.5717928,82.11664002
11,11.31195642,28.76111656,11.12117954,5,2,17.21800517,15.08260856,279.5717928,82.1166400
11,11.31195642,28.76111656,11.12117954,5,2,17.21800517,15.08260856,279.5717928,82.1166400
.099454,6688.081755,5.960078711,11.31195642,28.76111656,11.12117954,5,2,17.21800517,15.08
43.104683,6803.830615,6.443889833,9.111138868,31.03825582,11.15261014,5,2,21.26483645,9.1
7,0,1,0,1,1,12,7843.104683,6803.830615,6.443889833,9.111138868,31.03825582,11.15261014,5,2,
43.104683,6803.830615,6.443889833,9.111138868,31.03825582,11.15261014,5,2,21.26483645,9.1
3.104683,6803.830615,6.443889833,9.111138868,31.03825582,11.15261014,5,2,21.26483645,9.11
43.104683,6803.830615,6.443889833,9.111138868,31.03825582,11.15261014,5,2,21.26483645,9.1
333,9.111138868,31.03825582,11.15261014,5,2,21.26483645,9.111138868,289.3734978,85.059345
,7843.104683,6803.830615,6.443889833,9.111138868,31.03825582,11.15261014,5,2,21.26483645,
7,0,1,0,1,1,12,7843.104683,6803.830615,6.443889833,9.111138868,31.03825582,11.15261014,5,2,
443889833,9.111138868,31.03825582,11.15261014,5,2,21.26483645,9.111138868,289.3734978,85
43.104683,6803.830615,6.443889833,9.111138868,31.03825582,11.15261014,5,2,21.26483645,9.1
333,9.111138868,31.03825582,11.15261014,5,2,21.26483645,9.111138868,289.3734978,85.059345
843.104683,6803.830615,6.443889833,9.111138868,31.03825582,11.15261014,5,2,21.26483645,9.
7,0,1,0,1,1,12,7843.104683,6803.830615,6.443889833,9.111138868,31.03825582,11.15261014,5,2,
7,0,1,0,1,1,12,7843.104683,6803.830615,6.443889833,9.111138868,31.03825582,11.15261014,5,2,
43.104683,6803.830615,6.443889833,9.111138868,31.03825582,11.15261014,5,2,21.26483645,9.1
39833,9.111138868,31.03825582,11.15261014,5,2,21.26483645,9.111138868,289.3734978,85.0593
6.443889833,9.111138868,31.03825582,11.15261014,5,2,21.26483645,9.111138868,289.3734978,8
,67,0,1,0,1,1,12,7843.104683,6803.830615,6.443889833,9.111138868,31.03825582,11.15261014,5,
389833,9.111138868,31.03825582,11.15261014,5,2,21.26483645,9.111138868,289.3734978,85.059
,7843.104683,6803.830615,6.443889833,9.111138868,31.03825582,11.15261014,5,2,21.26483645,
0308838,3,2,217,67,0,1,0,1,1,12,7843.104683,6803.830615,6.443889833,9.111138868,31.03825582
7,0,1,0,1,1,12,7843.104683,6803.830615,6.443889833,9.111138868,31.03825582,11.15261014,5,2,
3.104683,6803.830615,6.443889833,9.111138868,31.03825582,11.15261014,5,2,21.26483645,9.11
43.104683,6803.830615,6.443889833,9.111138868,31.03825582,11.15261014,5,2,21.26483645,9.1
67,0,1,0,1,1,12,7843.104683,6803.830615,6.443889833,9.111138868,31.03825582,11.15261014,5,
43.104683,6803.830615,6.443889833,9.111138868,31.03825582,11.15261014,5,2,21.26483645,9.1
333,9.111138868,31.03825582,11.15261014,5,2,21.26483645,9.111138868,289.3734978,85.059345
7,0,1,0,1,1,12,7843.104683,6803.830615,6.443889833,9.111138868,31.03825582,11.15261014,5,2,
3.104683,6803.830615,6.443889833,9.111138868,31.03825582,11.15261014,5,2,21.26483645,9.11
333,9.111138868,31.03825582,11.15261014,5,2,21.26483645,9.111138868,289.3734978,85.059345
389833,9.111138868,31.03825582,11.15261014,5,2,21.26483645,9.111138868,289.3734978,85.059
7,0,1,0,1,1,12,7843.104683,6803.830615,6.443889833,9.111138868,31.03825582,11.15261014,5,2,
843.104683,6803.830615,6.443889833,9.111138868,31.03825582,11.15261014,5,2,21.26483645,9.

333,9.111138868,31.03825582,11.15261014,5,2,21.26483645,9.111138868,289.3734978,85.059345
333,9.111138868,31.03825582,11.15261014,5,2,21.26483645,9.111138868,289.3734978,85.059345
3.104683,6803.830615,6.443889833,9.111138868,31.03825582,11.15261014,5,2,21.26483645,9.11
843.104683,6803.830615,6.443889833,9.111138868,31.03825582,11.15261014,5,2,21.26483645,9.
3.104683,6803.830615,6.443889833,9.111138868,31.03825582,11.15261014,5,2,21.26483645,9.11
89,0,1,1,1,1,10,7843.104683,6803.830615,6.443889833,9.111138868,31.03825582,11.15261014,5,
3.104683,6803.830615,6.443889833,9.111138868,31.03825582,11.15261014,5,2,21.26483645,9.11
3.104683,6803.830615,6.443889833,9.111138868,31.03825582,11.15261014,5,2,21.26483645,9.11
389833,9.111138868,31.03825582,11.15261014,5,2,21.26483645,9.111138868,289.3734978,85.059
15,6.443889833,9.111138868,31.03825582,11.15261014,5,2,21.26483645,9.111138868,289.373497
43889833,9.111138868,31.03825582,11.15261014,5,2,21.26483645,9.111138868,289.3734978,85.0
333,9.111138868,31.03825582,11.15261014,5,2,21.26483645,9.111138868,289.3734978,85.059345
333,9.111138868,31.03825582,11.15261014,5,2,21.26483645,9.111138868,289.3734978,85.059345
9.111138868,31.03825582,11.15261014,5,2,21.26483645,9.111138868,289.3734978,85.05934579,
843.104683,6803.830615,6.443889833,9.111138868,31.03825582,11.15261014,5,2,21.26483645,9.
9,0,1,1,1,1,10,7843.104683,6803.830615,6.443889833,9.111138868,31.03825582,11.15261014,5,2,
443889833,9.111138868,31.03825582,11.15261014,5,2,21.26483645,9.111138868,289.3734978,85
89,0,1,1,1,1,10,7843.104683,6803.830615,6.443889833,9.111138868,31.03825582,11.15261014,5,
333,9.111138868,31.03825582,11.15261014,5,2,21.26483645,9.111138868,289.3734978,85.059345
3.104683,6803.830615,6.443889833,9.111138868,31.03825582,11.15261014,5,2,21.26483645,9.11
89,0,1,1,1,1,10,7843.104683,6803.830615,6.443889833,9.111138868,31.03825582,11.15261014,5,
6.443889833,9.111138868,31.03825582,11.15261014,5,2,21.26483645,9.111138868,289.3734978,
333,9.111138868,31.03825582,11.15261014,5,2,21.26483645,9.111138868,289.3734978,85.059345
9,0,1,1,1,1,10,7843.104683,6803.830615,6.443889833,9.111138868,31.03825582,11.15261014,5,2,
3.104683,6803.830615,6.443889833,9.111138868,31.03825582,11.15261014,5,2,21.26483645,9.11
0617676,3,2,217,89,0,1,1,1,1,10,7843.104683,6803.830615,6.443889833,9.111138868,31.03825582
333,9.111138868,31.03825582,11.15261014,5,2,21.26483645,9.111138868,289.3734978,85.059345
843.104683,6803.830615,6.443889833,9.111138868,31.03825582,11.15261014,5,2,21.26483645,9.
43889833,9.111138868,31.03825582,11.15261014,5,2,21.26483645,9.111138868,289.3734978,85.0
7843.104683,6803.830615,6.443889833,9.111138868,31.03825582,11.15261014,5,2,21.26483645,9
389833,9.111138868,31.03825582,11.15261014,5,2,21.26483645,9.111138868,289.3734978,85.059
33,9.111138868,31.03825582,11.15261014,5,2,21.26483645,9.111138868,289.3734978,85.0593457
43.104683,6803.830615,6.443889833,9.111138868,31.03825582,11.15261014,5,2,21.26483645,9.1
3.104683,6803.830615,6.443889833,9.111138868,31.03825582,11.15261014,5,2,21.26483645,9.11
333,9.111138868,31.03825582,11.15261014,5,2,21.26483645,9.111138868,289.3734978,85.059345
389833,9.111138868,31.03825582,11.15261014,5,2,21.26483645,9.111138868,289.3734978,85.059
,15.20648111,28.76111656,14.7004655,5,3,18.00042323,22.80972167,325.8186058,81.00190455,
27.904603,6699.99811,5.785850325,15.20648111,28.76111656,14.7004655,5,3,18.00042323,22.80
6,1,1,1,1,1,11,7227.904603,6699.99811,5.785850325,15.20648111,28.76111656,14.7004655,5,3,18
,15.20648111,28.76111656,14.7004655,5,3,18.00042323,22.80972167,325.8186058,81.00190455,
,15.20648111,28.76111656,14.7004655,5,3,18.00042323,22.80972167,325.8186058,81.00190455,
6,1,1,1,1,1,11,7227.904603,6699.99811,5.785850325,15.20648111,28.76111656,14.7004655,5,3,18
27.904603,6699.99811,5.785850325,15.20648111,28.76111656,14.7004655,5,3,18.00042323,22.80
27.904603,6699.99811,5.785850325,15.20648111,28.76111656,14.7004655,5,3,18.00042323,22.80
,15.20648111,28.76111656,14.7004655,5,3,18.00042323,22.80972167,325.8186058,81.00190455,
6,1,1,1,1,1,11,7227.904603,6699.99811,5.785850325,15.20648111,28.76111656,14.7004655,5,3,18
27.904603,6699.99811,5.785850325,15.20648111,28.76111656,14.7004655,5,3,18.00042323,22.80
25,15.20648111,28.76111656,14.7004655,5,3,18.00042323,22.80972167,325.8186058,81.00190455
27.904603,6699.99811,5.785850325,15.20648111,28.76111656,14.7004655,5,3,18.00042323,22.80
382324,3,2,217,76,1,1,1,1,1,11,7227.904603,6699.99811,5.785850325,15.20648111,28.76111656,1

27.904603,6699.99811,5.785850325,15.20648111,28.76111656,14.7004655,5,3,18.00042323,22.80
27.904603,6699.99811,5.785850325,15.20648111,28.76111656,14.7004655,5,3,18.00042323,22.80
76,1,1,1,1,1,11,7227.904603,6699.99811,5.785850325,15.20648111,28.76111656,14.7004655,5,3,1
25,15.20648111,28.76111656,14.7004655,5,3,18.00042323,22.80972167,325.8186058,81.00190455
5850325,15.20648111,28.76111656,14.7004655,5,3,18.00042323,22.80972167,325.8186058,81.001
25,15.20648111,28.76111656,14.7004655,5,3,18.00042323,22.80972167,325.8186058,81.00190455
6,1,1,1,1,1,11,7227.904603,6699.99811,5.785850325,15.20648111,28.76111656,14.7004655,5,3,18
76,1,1,1,1,1,11,7227.904603,6699.99811,5.785850325,15.20648111,28.76111656,14.7004655,5,3,1
6,1,1,1,1,1,11,7227.904603,6699.99811,5.785850325,15.20648111,28.76111656,14.7004655,5,3,18
27.904603,6699.99811,5.785850325,15.20648111,28.76111656,14.7004655,5,3,18.00042323,22.80
6,1,1,1,1,1,11,7227.904603,6699.99811,5.785850325,15.20648111,28.76111656,14.7004655,5,3,18
7.904603,6699.99811,5.785850325,15.20648111,28.76111656,14.7004655,5,3,18.00042323,22.809
5,15.20648111,28.76111656,14.7004655,5,3,18.00042323,22.80972167,325.8186058,81.00190455,
6,1,1,1,1,1,11,7227.904603,6699.99811,5.785850325,15.20648111,28.76111656,14.7004655,5,3,18
7.904603,6699.99811,5.785850325,15.20648111,28.76111656,14.7004655,5,3,18.00042323,22.809
6,1,1,1,1,1,11,7227.904603,6699.99811,5.785850325,15.20648111,28.76111656,14.7004655,5,3,18
27.904603,6699.99811,5.785850325,15.20648111,28.76111656,14.7004655,5,3,18.00042323,22.80
5,15.20648111,28.76111656,14.7004655,5,3,18.00042323,22.80972167,325.8186058,81.00190455,
227.904603,6699.99811,5.785850325,15.20648111,28.76111656,14.7004655,5,3,18.00042323,22.8
25,15.20648111,28.76111656,14.7004655,5,3,18.00042323,22.80972167,325.8186058,81.00190455
3,9.111138868,31.03825582,11.15261014,5,2,21.26483645,9.111138868,289.3734978,85.05934579
3,9.111138868,31.03825582,11.15261014,5,2,21.26483645,9.111138868,289.3734978,85.05934579
33,9.111138868,31.03825582,11.15261014,5,2,21.26483645,9.111138868,289.3734978,85.0593457
.104683,6803.830615,6.443889833,9.111138868,31.03825582,11.15261014,5,2,21.26483645,9.111
3.104683,6803.830615,6.443889833,9.111138868,31.03825582,11.15261014,5,2,21.26483645,9.11
3,9.111138868,31.03825582,11.15261014,5,2,21.26483645,9.111138868,289.3734978,85.05934579
3.104683,6803.830615,6.443889833,9.111138868,31.03825582,11.15261014,5,2,21.26483645,9.11
33,9.111138868,31.03825582,11.15261014,5,2,21.26483645,9.111138868,289.3734978,85.0593457
3,9.111138868,31.03825582,11.15261014,5,2,21.26483645,9.111138868,289.3734978,85.05934579
9,1,1,0,1,1,8,7843.104683,6803.830615,6.443889833,9.111138868,31.03825582,11.15261014,5,2,2
43889833,9.111138868,31.03825582,11.15261014,5,2,21.26483645,9.111138868,289.3734978,85.
.104683,6803.830615,6.443889833,9.111138868,31.03825582,11.15261014,5,2,21.26483645,9.111
3.104683,6803.830615,6.443889833,9.111138868,31.03825582,11.15261014,5,2,21.26483645,9.11
382324,3,2,217,79,1,1,0,1,1,8,7843.104683,6803.830615,6.443889833,9.111138868,31.03825582,1
3.104683,6803.830615,6.443889833,9.111138868,31.03825582,11.15261014,5,2,21.26483645,9.11
89833,9.111138868,31.03825582,11.15261014,5,2,21.26483645,9.111138868,289.3734978,85.0593
79,1,1,0,1,1,8,7843.104683,6803.830615,6.443889833,9.111138868,31.03825582,11.15261014,5,2,
889833,9.111138868,31.03825582,11.15261014,5,2,21.26483645,9.111138868,289.3734978,85.05
33,9.111138868,31.03825582,11.15261014,5,2,21.26483645,9.111138868,289.3734978,85.0593457
33,9.111138868,31.03825582,11.15261014,5,2,21.26483645,9.111138868,289.3734978,85.0593457
889833,9.111138868,31.03825582,11.15261014,5,2,21.26483645,9.111138868,289.3734978,85.05
33,9.111138868,31.03825582,11.15261014,5,2,21.26483645,9.111138868,289.3734978,85.0593457
9,1,1,0,1,1,8,7843.104683,6803.830615,6.443889833,9.111138868,31.03825582,11.15261014,5,2,2
3.104683,6803.830615,6.443889833,9.111138868,31.03825582,11.15261014,5,2,21.26483645,9.11
9,1,1,0,1,1,8,7843.104683,6803.830615,6.443889833,9.111138868,31.03825582,11.15261014,5,2,2
529395,3,2,217,79,1,1,0,1,1,8,7843.104683,6803.830615,6.443889833,9.111138868,31.03825582,1
3,9.111138868,31.03825582,11.15261014,5,2,21.26483645,9.111138868,289.3734978,85.05934579
517676,3,2,217,79,1,1,0,1,1,8,7843.104683,6803.830615,6.443889833,9.111138868,31.03825582,1
.104683,6803.830615,6.443889833,9.111138868,31.03825582,11.15261014,5,2,21.26483645,9.111
9,1,1,0,1,1,8,7843.104683,6803.830615,6.443889833,9.111138868,31.03825582,11.15261014,5,2,2

3.104683,6803.830615,6.443889833,9.111138868,31.03825582,11.15261014,5,2,21.26483645,9.11
9,1,1,0,1,1,8,7843.104683,6803.830615,6.443889833,9.111138868,31.03825582,11.15261014,5,2,2
3,9.111138868,31.03825582,11.15261014,5,2,21.26483645,9.111138868,289.3734978,85.05934579
43.104683,6803.830615,6.443889833,9.111138868,31.03825582,11.15261014,5,2,21.26483645,9.1
33,9.111138868,31.03825582,11.15261014,5,2,21.26483645,9.111138868,289.3734978,85.0593457
6.303897,6782.338417,5.835366132,8.177644167,31.03825582,3.579285958,5,1,25.93496059,12.2
.303897,6782.338417,5.835366132,8.177644167,31.03825582,3.579285958,5,1,25.93496059,12.26
6.303897,6782.338417,5.835366132,8.177644167,31.03825582,3.579285958,5,1,25.93496059,12.2
.303897,6782.338417,5.835366132,8.177644167,31.03825582,3.579285958,5,1,25.93496059,12.26
0617676,3,2,217,76,1,1,1,1,1,9,7396.303897,6782.338417,5.835366132,8.177644167,31.03825582,3
6.303897,6782.338417,5.835366132,8.177644167,31.03825582,3.579285958,5,1,25.93496059,12.2
2,8.177644167,31.03825582,3.579285958,5,1,25.93496059,12.26646625,333.2993211,74.56301168
382324,3,2,217,76,1,1,1,1,1,9,7396.303897,6782.338417,5.835366132,8.177644167,31.03825582,3.
.303897,6782.338417,5.835366132,8.177644167,31.03825582,3.579285958,5,1,25.93496059,12.26
5366132,8.177644167,31.03825582,3.579285958,5,1,25.93496059,12.26646625,333.2993211,74.56
6,1,1,1,1,1,9,7396.303897,6782.338417,5.835366132,8.177644167,31.03825582,3.579285958,5,1,2
56132,8.177644167,31.03825582,3.579285958,5,1,25.93496059,12.26646625,333.2993211,74.5630
96.303897,6782.338417,5.835366132,8.177644167,31.03825582,3.579285958,5,1,25.93496059,12.
6.303897,6782.338417,5.835366132,8.177644167,31.03825582,3.579285958,5,1,25.93496059,12.2
6.303897,6782.338417,5.835366132,8.177644167,31.03825582,3.579285958,5,1,25.93496059,12.2
6.303897,6782.338417,5.835366132,8.177644167,31.03825582,3.579285958,5,1,25.93496059,12.2
.303897,6782.338417,5.835366132,8.177644167,31.03825582,3.579285958,5,1,25.93496059,12.26
6.303897,6782.338417,5.835366132,8.177644167,31.03825582,3.579285958,5,1,25.93496059,12.2
6.303897,6782.338417,5.835366132,8.177644167,31.03825582,3.579285958,5,1,25.93496059,12.2
529395,3,2,217,76,1,1,1,1,1,9,7396.303897,6782.338417,5.835366132,8.177644167,31.03825582,3.
6.303897,6782.338417,5.835366132,8.177644167,31.03825582,3.579285958,5,1,25.93496059,12.2
6,1,1,1,1,1,9,7396.303897,6782.338417,5.835366132,8.177644167,31.03825582,3.579285958,5,1,2
2,8.177644167,31.03825582,3.579285958,5,1,25.93496059,12.26646625,333.2993211,74.56301168
6.303897,6782.338417,5.835366132,8.177644167,31.03825582,3.579285958,5,1,25.93496059,12.2
32,8.177644167,31.03825582,3.579285958,5,1,25.93496059,12.26646625,333.2993211,74.5630116
396.303897,6782.338417,5.835366132,8.177644167,31.03825582,3.579285958,5,1,25.93496059,12
6.303897,6782.338417,5.835366132,8.177644167,31.03825582,3.579285958,5,1,25.93496059,12.2
6.303897,6782.338417,5.835366132,8.177644167,31.03825582,3.579285958,5,1,25.93496059,12.2
32,8.177644167,31.03825582,3.579285958,5,1,25.93496059,12.26646625,333.2993211,74.5630116
5132,8.177644167,31.03825582,3.579285958,5,1,25.93496059,12.26646625,333.2993211,74.56301
56132,8.177644167,31.03825582,3.579285958,5,1,25.93496059,12.26646625,333.2993211,74.5630
6.303897,6782.338417,5.835366132,8.177644167,31.03825582,3.579285958,5,1,25.93496059,12.2
517676,3,2,217,76,1,1,1,1,1,9,7396.303897,6782.338417,5.835366132,8.177644167,31.03825582,3.
6.303897,6782.338417,5.835366132,8.177644167,31.03825582,3.579285958,5,1,25.93496059,12.2
6.303897,6782.338417,5.835366132,8.177644167,31.03825582,3.579285958,5,1,25.93496059,12.2
56132,8.177644167,31.03825582,3.579285958,5,1,25.93496059,12.26646625,333.2993211,74.5630
76,1,1,1,1,1,9,7396.303897,6782.338417,5.835366132,8.177644167,31.03825582,3.579285958,5,1,
54896,7.141534467,32.94084014,3.579285958,6,1,28.19728122,14.28306893,118.1575943,87.8453
.788545,6986.262374,6.507064896,7.141534467,32.94084014,3.579285958,6,1,28.19728122,14.28
90.788545,6986.262374,6.507064896,7.141534467,32.94084014,3.579285958,6,1,28.19728122,14.
.788545,6986.262374,6.507064896,7.141534467,32.94084014,3.579285958,6,1,28.19728122,14.28
5,7.141534467,32.94084014,3.579285958,6,1,28.19728122,14.28306893,118.1575943,87.8453761,
54896,7.141534467,32.94084014,3.579285958,6,1,28.19728122,14.28306893,118.1575943,87.8453
96,7.141534467,32.94084014,3.579285958,6,1,28.19728122,14.28306893,118.1575943,87.8453761
.788545,6986.262374,6.507064896,7.141534467,32.94084014,3.579285958,6,1,28.19728122,14.28

```
96,7.141534467,32.94084014,3.579285958,6,1,28.19728122,14.28306893,118.1575943,87.8453761
7064896,7.141534467,32.94084014,3.579285958,6,1,28.19728122,14.28306893,118.1575943,87.84
4896,7.141534467,32.94084014,3.579285958,6,1,28.19728122,14.28306893,118.1575943,87.84537
.788545,6986.262374,6.507064896,7.141534467,32.94084014,3.579285958,6,1,28.19728122,14.28
.788545,6986.262374,6.507064896,7.141534467,32.94084014,3.579285958,6,1,28.19728122,14.28
90.788545,6986.262374,6.507064896,7.141534467,32.94084014,3.579285958,6,1,28.19728122,14.
7064896,7.141534467,32.94084014,3.579285958,6,1,28.19728122,14.28306893,118.1575943,87.84
75,1,0,0,1,1,6,9090.788545,6986.262374,6.507064896,7.141534467,32.94084014,3.579285958,6,1,
7064896,7.141534467,32.94084014,3.579285958,6,1,28.19728122,14.28306893,118.1575943,87.84
.788545,6986.262374,6.507064896,7.141534467,32.94084014,3.579285958,6,1,28.19728122,14.28
75,1,0,0,1,1,6,9090.788545,6986.262374,6.507064896,7.141534467,32.94084014,3.579285958,6,1,
.788545,6986.262374,6.507064896,7.141534467,32.94084014,3.579285958,6,1,28.19728122,14.28
6,7.141534467,32.94084014,3.579285958,6,1,28.19728122,14.28306893,118.1575943,87.8453761,
07064896,7.141534467,32.94084014,3.579285958,6,1,28.19728122,14.28306893,118.1575943,87.8
.788545,6986.262374,6.507064896,7.141534467,32.94084014,3.579285958,6,1,28.19728122,14.28
.788545,6986.262374,6.507064896,7.141534467,32.94084014,3.579285958,6,1,28.19728122,14.28
.788545,6986.262374,6.507064896,7.141534467,32.94084014,3.579285958,6,1,28.19728122,14.28
96,7.141534467,32.94084014,3.579285958,6,1,28.19728122,14.28306893,118.1575943,87.8453761
96,7.141534467,32.94084014,3.579285958,6,1,28.19728122,14.28306893,118.1575943,87.8453761
96,7.141534467,32.94084014,3.579285958,6,1,28.19728122,14.28306893,118.1575943,87.8453761
90.788545,6986.262374,6.507064896,7.141534467,32.94084014,3.579285958,6,1,28.19728122,14.
.788545,6986.262374,6.507064896,7.141534467,32.94084014,3.579285958,6,1,28.19728122,14.28
64896,7.141534467,32.94084014,3.579285958,6,1,28.19728122,14.28306893,118.1575943,87.8453
0.788545,6986.262374,6.507064896,7.141534467,32.94084014,3.579285958,6,1,28.19728122,14.2
75,1,0,0,1,1,6,9090.788545,6986.262374,6.507064896,7.141534467,32.94084014,3.579285958,6,1,
3,10.21873537,29.39298478,4.132277098,5,2,28.20093736,30.65620611,326.4372456,89.11496205
2.740955,9707.536865,7.332243713,10.21873537,29.39298478,4.132277098,5,2,28.20093736,30.6
922.740955,9707.536865,7.332243713,10.21873537,29.39298478,4.132277098,5,2,28.20093736,30
.740955,9707.536865,7.332243713,10.21873537,29.39298478,4.132277098,5,2,28.20093736,30.65
2.740955,9707.536865,7.332243713,10.21873537,29.39298478,4.132277098,5,2,28.20093736,30.6
,0,1,0,1,1,8,9922.740955,9707.536865,7.332243713,10.21873537,29.39298478,4.132277098,5,2,28
7,0,1,0,1,1,8,9922.740955,9707.536865,7.332243713,10.21873537,29.39298478,4.132277098,5,2,2
77,0,1,0,1,1,8,9922.740955,9707.536865,7.332243713,10.21873537,29.39298478,4.132277098,5,2,
77,0,1,0,1,1,8,9922.740955,9707.536865,7.332243713,10.21873537,29.39298478,4.132277098,5,2,
22.740955,9707.536865,7.332243713,10.21873537,29.39298478,4.132277098,5,2,28.20093736,30.
10.21873537,29.39298478,4.132277098,5,2,28.20093736,30.65620611,326.4372456,89.11496205,
2243713,10.21873537,29.39298478,4.132277098,5,2,28.20093736,30.65620611,326.4372456,89.11
77,0,1,0,1,1,8,9922.740955,9707.536865,7.332243713,10.21873537,29.39298478,4.132277098,5,2,
2.740955,9707.536865,7.332243713,10.21873537,29.39298478,4.132277098,5,2,28.20093736,30.6
13,10.21873537,29.39298478,4.132277098,5,2,28.20093736,30.65620611,326.4372456,89.1149620
13,10.21873537,29.39298478,4.132277098,5,2,28.20093736,30.65620611,326.4372456,89.1149620
2.740955,9707.536865,7.332243713,10.21873537,29.39298478,4.132277098,5,2,28.20093736,30.6
.740955,9707.536865,7.332243713,10.21873537,29.39298478,4.132277098,5,2,28.20093736,30.65
2243713,10.21873537,29.39298478,4.132277098,5,2,28.20093736,30.65620611,326.4372456,89.11
2.740955,9707.536865,7.332243713,10.21873537,29.39298478,4.132277098,5,2,28.20093736,30.6
2243713,10.21873537,29.39298478,4.132277098,5,2,28.20093736,30.65620611,326.4372456,89.11
7,0,1,0,1,1,8,9922.740955,9707.536865,7.332243713,10.21873537,29.39298478,4.132277098,5,2,2
.740955,9707.536865,7.332243713,10.21873537,29.39298478,4.132277098,5,2,28.20093736,30.65
22.740955,9707.536865,7.332243713,10.21873537,29.39298478,4.132277098,5,2,28.20093736,30.
2243713,10.21873537,29.39298478,4.132277098,5,2,28.20093736,30.65620611,326.4372456,89.11
```

```
13,10.21873537,29.39298478,4.132277098,5,2,28.20093736,30.65620611,326.4372456,89.1149620
2243713,10.21873537,29.39298478,4.132277098,5,2,28.20093736,30.65620611,326.4372456,89.11
22.740955,9707.536865,7.332243713,10.21873537,29.39298478,4.132277098,5,2,28.20093736,30.
22.740955,9707.536865,7.332243713,10.21873537,29.39298478,4.132277098,5,2,28.20093736,30.
.740955,9707.536865,7.332243713,10.21873537,29.39298478,4.132277098,5,2,28.20093736,30.65
228,16.89527049,26.2140549,10.66364303,12,4,16.4793237,16.89527049,336.7526759,64.0158343
512.627589,4528.536465,10.14112228,16.89527049,26.2140549,10.66364303,12,4,16.4793237,16.
12.627589,4528.536465,10.14112228,16.89527049,26.2140549,10.66364303,12,4,16.4793237,16.8
2.627589,4528.536465,10.14112228,16.89527049,26.2140549,10.66364303,12,4,16.4793237,16.89
2.627589,4528.536465,10.14112228,16.89527049,26.2140549,10.66364303,12,4,16.4793237,16.89
2.627589,4528.536465,10.14112228,16.89527049,26.2140549,10.66364303,12,4,16.4793237,16.89
3,90,1,0,1,0,1,4,4512.627589,4528.536465,10.14112228,16.89527049,26.2140549,10.66364303,12,4
228,16.89527049,26.2140549,10.66364303,12,4,16.4793237,16.89527049,336.7526759,64.0158343
2.627589,4528.536465,10.14112228,16.89527049,26.2140549,10.66364303,12,4,16.4793237,16.89
12.627589,4528.536465,10.14112228,16.89527049,26.2140549,10.66364303,12,4,16.4793237,16.8
90,1,0,1,0,1,4,4512.627589,4528.536465,10.14112228,16.89527049,26.2140549,10.66364303,12,4,
512.627589,4528.536465,10.14112228,16.89527049,26.2140549,10.66364303,12,4,16.4793237,16.
2.627589,4528.536465,10.14112228,16.89527049,26.2140549,10.66364303,12,4,16.4793237,16.89
12.627589,4528.536465,10.14112228,16.89527049,26.2140549,10.66364303,12,4,16.4793237,16.8
228,16.89527049,26.2140549,10.66364303,12,4,16.4793237,16.89527049,336.7526759,64.0158343
12.627589,4528.536465,10.14112228,16.89527049,26.2140549,10.66364303,12,4,16.4793237,16.8
512.627589,4528.536465,10.14112228,16.89527049,26.2140549,10.66364303,12,4,16.4793237,16.
512.627589,4528.536465,10.14112228,16.89527049,26.2140549,10.66364303,12,4,16.4793237,16.
28,16.89527049,26.2140549,10.66364303,12,4,16.4793237,16.89527049,336.7526759,64.01583439
3,90,1,0,1,0,1,4,4512.627589,4528.536465,10.14112228,16.89527049,26.2140549,10.66364303,12,4
12.627589,4528.536465,10.14112228,16.89527049,26.2140549,10.66364303,12,4,16.4793237,16.8
228,16.89527049,26.2140549,10.66364303,12,4,16.4793237,16.89527049,336.7526759,64.0158343
2.627589,4528.536465,10.14112228,16.89527049,26.2140549,10.66364303,12,4,16.4793237,16.89
12.627589,4528.536465,10.14112228,16.89527049,26.2140549,10.66364303,12,4,16.4793237,16.8
2.627589,4528.536465,10.14112228,16.89527049,26.2140549,10.66364303,12,4,16.4793237,16.89
3,90,1,0,1,0,1,4,4512.627589,4528.536465,10.14112228,16.89527049,26.2140549,10.66364303,12,4
2.627589,4528.536465,10.14112228,16.89527049,26.2140549,10.66364303,12,4,16.4793237,16.89
512.627589,4528.536465,10.14112228,16.89527049,26.2140549,10.66364303,12,4,16.4793237,16.
2.627589,4528.536465,10.14112228,16.89527049,26.2140549,10.66364303,12,4,16.4793237,16.89
512.627589,4528.536465,10.14112228,16.89527049,26.2140549,10.66364303,12,4,16.4793237,16.
39691162,3,2,218,90,1,0,1,0,1,4,4512.627589,4528.536465,10.14112228,16.89527049,26.2140549,1
2.627589,4528.536465,10.14112228,16.89527049,26.2140549,10.66364303,12,4,16.4793237,16.89
2.627589,4528.536465,10.14112228,16.89527049,26.2140549,10.66364303,12,4,16.4793237,16.89
12.627589,4528.536465,10.14112228,16.89527049,26.2140549,10.66364303,12,4,16.4793237,16.8
2.627589,4528.536465,10.14112228,16.89527049,26.2140549,10.66364303,12,4,16.4793237,16.89
13552,41.3076248,34.141976,10.03789582,14,9,15.33913552,11.26571585,201.1785198,59.551937
777.074849,4408.728467,15.33913552,41.3076248,34.141976,10.03789582,14,9,15.33913552,11.2
77.074849,4408.728467,15.33913552,41.3076248,34.141976,10.03789582,14,9,15.33913552,11.26
3913552,41.3076248,34.141976,10.03789582,14,9,15.33913552,11.26571585,201.1785198,59.5519
777.074849,4408.728467,15.33913552,41.3076248,34.141976,10.03789582,14,9,15.33913552,11.2
777.074849,4408.728467,15.33913552,41.3076248,34.141976,10.03789582,14,9,15.33913552,11.2
3,69,0,1,0,1,1,12,4777.074849,4408.728467,15.33913552,41.3076248,34.141976,10.03789582,14,9,
77.074849,4408.728467,15.33913552,41.3076248,34.141976,10.03789582,14,9,15.33913552,11.26
33913552,41.3076248,34.141976,10.03789582,14,9,15.33913552,11.26571585,201.1785198,59.551
77.074849,4408.728467,15.33913552,41.3076248,34.141976,10.03789582,14,9,15.33913552,11.26
```

777.074849,4408.728467,15.33913552,41.3076248,34.141976,10.03789582,14,9,15.33913552,11.2
777.074849,4408.728467,15.33913552,41.3076248,34.141976,10.03789582,14,9,15.33913552,11.2
13552,41.3076248,34.141976,10.03789582,14,9,15.33913552,11.26571585,201.1785198,59.551937
3552,41.3076248,34.141976,10.03789582,14,9,15.33913552,11.26571585,201.1785198,59.5519379
777.074849,4408.728467,15.33913552,41.3076248,34.141976,10.03789582,14,9,15.33913552,11.2
777.074849,4408.728467,15.33913552,41.3076248,34.141976,10.03789582,14,9,15.33913552,11.2
777.074849,4408.728467,15.33913552,41.3076248,34.141976,10.03789582,14,9,15.33913552,11.2
69,0,1,0,1,1,12,4777.074849,4408.728467,15.33913552,41.3076248,34.141976,10.03789582,14,9,1
3913552,41.3076248,34.141976,10.03789582,14,9,15.33913552,11.26571585,201.1785198,59.5519
777.074849,4408.728467,15.33913552,41.3076248,34.141976,10.03789582,14,9,15.33913552,11.2
3,69,0,1,0,1,1,12,4777.074849,4408.728467,15.33913552,41.3076248,34.141976,10.03789582,14,9,
777.074849,4408.728467,15.33913552,41.3076248,34.141976,10.03789582,14,9,15.33913552,11.2
3913552,41.3076248,34.141976,10.03789582,14,9,15.33913552,11.26571585,201.1785198,59.5519
3552,41.3076248,34.141976,10.03789582,14,9,15.33913552,11.26571585,201.1785198,59.5519379
5.33913552,41.3076248,34.141976,10.03789582,14,9,15.33913552,11.26571585,201.1785198,59.5
777.074849,4408.728467,15.33913552,41.3076248,34.141976,10.03789582,14,9,15.33913552,11.2
77.074849,4408.728467,15.33913552,41.3076248,34.141976,10.03789582,14,9,15.33913552,11.26
777.074849,4408.728467,15.33913552,41.3076248,34.141976,10.03789582,14,9,15.33913552,11.2
69,0,1,0,1,1,12,4777.074849,4408.728467,15.33913552,41.3076248,34.141976,10.03789582,14,9,1
3552,41.3076248,34.141976,10.03789582,14,9,15.33913552,11.26571585,201.1785198,59.5519379
5003,20.5390953,26.2140549,10.66364303,12,4,15.7769688,16.43127624,325.7125217,64.3700327
42.304285,4594.983269,10.09726003,20.5390953,26.2140549,10.66364303,12,4,15.7769688,16.43
4542.304285,4594.983269,10.09726003,20.5390953,26.2140549,10.66364303,12,4,15.7769688,16.
84,0,1,1,1,1,12,4542.304285,4594.983269,10.09726003,20.5390953,26.2140549,10.66364303,12,4,
42.304285,4594.983269,10.09726003,20.5390953,26.2140549,10.66364303,12,4,15.7769688,16.43
9726003,20.5390953,26.2140549,10.66364303,12,4,15.7769688,16.43127624,325.7125217,64.3700
5003,20.5390953,26.2140549,10.66364303,12,4,15.7769688,16.43127624,325.7125217,64.3700327
4542.304285,4594.983269,10.09726003,20.5390953,26.2140549,10.66364303,12,4,15.7769688,16.
42.304285,4594.983269,10.09726003,20.5390953,26.2140549,10.66364303,12,4,15.7769688,16.43
42.304285,4594.983269,10.09726003,20.5390953,26.2140549,10.66364303,12,4,15.7769688,16.43
003,20.5390953,26.2140549,10.66364303,12,4,15.7769688,16.43127624,325.7125217,64.3700327,
9,10.09726003,20.5390953,26.2140549,10.66364303,12,4,15.7769688,16.43127624,325.7125217,6
10.09726003,20.5390953,26.2140549,10.66364303,12,4,15.7769688,16.43127624,325.7125217,64.
5003,20.5390953,26.2140549,10.66364303,12,4,15.7769688,16.43127624,325.7125217,64.3700327
5003,20.5390953,26.2140549,10.66364303,12,4,15.7769688,16.43127624,325.7125217,64.3700327
42.304285,4594.983269,10.09726003,20.5390953,26.2140549,10.66364303,12,4,15.7769688,16.43
42.304285,4594.983269,10.09726003,20.5390953,26.2140549,10.66364303,12,4,15.7769688,16.43
4542.304285,4594.983269,10.09726003,20.5390953,26.2140549,10.66364303,12,4,15.7769688,16.
5003,20.5390953,26.2140549,10.66364303,12,4,15.7769688,16.43127624,325.7125217,64.3700327
26003,20.5390953,26.2140549,10.66364303,12,4,15.7769688,16.43127624,325.7125217,64.370032
542.304285,4594.983269,10.09726003,20.5390953,26.2140549,10.66364303,12,4,15.7769688,16.4
542.304285,4594.983269,10.09726003,20.5390953,26.2140549,10.66364303,12,4,15.7769688,16.4
.09726003,20.5390953,26.2140549,10.66364303,12,4,15.7769688,16.43127624,325.7125217,64.37
0617676,3,2,218,84,0,1,1,1,1,12,4542.304285,4594.983269,10.09726003,20.5390953,26.2140549,1
542.304285,4594.983269,10.09726003,20.5390953,26.2140549,10.66364303,12,4,15.7769688,16.4
542.304285,4594.983269,10.09726003,20.5390953,26.2140549,10.66364303,12,4,15.7769688,16.4
84,0,1,1,1,1,12,4542.304285,4594.983269,10.09726003,20.5390953,26.2140549,10.66364303,12,4,
5003,20.5390953,26.2140549,10.66364303,12,4,15.7769688,16.43127624,325.7125217,64.3700327
9,10.09726003,20.5390953,26.2140549,10.66364303,12,4,15.7769688,16.43127624,325.7125217,6
9726003,20.5390953,26.2140549,10.66364303,12,4,15.7769688,16.43127624,325.7125217,64.3700

9,10.09726003,20.5390953,26.2140549,10.66364303,12,4,15.7769688,16.43127624,325.7125217,64
542.304285,4594.983269,10.09726003,20.5390953,26.2140549,10.66364303,12,4,15.7769688,16.4
5258789,3,2,218,84,0,1,1,1,1,12,4542.304285,4594.983269,10.09726003,20.5390953,26.2140549,10
9726003,20.5390953,26.2140549,10.66364303,12,4,15.7769688,16.43127624,325.7125217,64.3700
9005615,5.459584892,18.65493977,1.983195325,9,1,16.08075983,10.91916978,166.7189452,82.70
78,1,1,0,0,1,12,4092.313692,3394.355042,9.189005615,5.459584892,18.65493977,1.983195325,9,1
092.313692,3394.355042,9.189005615,5.459584892,18.65493977,1.983195325,9,1,16.08075983,10
092.313692,3394.355042,9.189005615,5.459584892,18.65493977,1.983195325,9,1,16.08075983,10
5615,5.459584892,18.65493977,1.983195325,9,1,16.08075983,10.91916978,166.7189452,82.70105
092.313692,3394.355042,9.189005615,5.459584892,18.65493977,1.983195325,9,1,16.08075983,10
5615,5.459584892,18.65493977,1.983195325,9,1,16.08075983,10.91916978,166.7189452,82.70105
092.313692,3394.355042,9.189005615,5.459584892,18.65493977,1.983195325,9,1,16.08075983,10
92.313692,3394.355042,9.189005615,5.459584892,18.65493977,1.983195325,9,1,16.08075983,10.
92.313692,3394.355042,9.189005615,5.459584892,18.65493977,1.983195325,9,1,16.08075983,10.
189005615,5.459584892,18.65493977,1.983195325,9,1,16.08075983,10.91916978,166.7189452,82
189005615,5.459584892,18.65493977,1.983195325,9,1,16.08075983,10.91916978,166.7189452,82.
78,1,1,0,0,1,12,4092.313692,3394.355042,9.189005615,5.459584892,18.65493977,1.983195325,9,1
05615,5.459584892,18.65493977,1.983195325,9,1,16.08075983,10.91916978,166.7189452,82.7010
92.313692,3394.355042,9.189005615,5.459584892,18.65493977,1.983195325,9,1,16.08075983,10.
9005615,5.459584892,18.65493977,1.983195325,9,1,16.08075983,10.91916978,166.7189452,82.70
189005615,5.459584892,18.65493977,1.983195325,9,1,16.08075983,10.91916978,166.7189452,82.
78,1,1,0,0,1,12,4092.313692,3394.355042,9.189005615,5.459584892,18.65493977,1.983195325,9,1
05615,5.459584892,18.65493977,1.983195325,9,1,16.08075983,10.91916978,166.7189452,82.7010
092.313692,3394.355042,9.189005615,5.459584892,18.65493977,1.983195325,9,1,16.08075983,10
5615,5.459584892,18.65493977,1.983195325,9,1,16.08075983,10.91916978,166.7189452,82.70105
189005615,5.459584892,18.65493977,1.983195325,9,1,16.08075983,10.91916978,166.7189452,82.
092.313692,3394.355042,9.189005615,5.459584892,18.65493977,1.983195325,9,1,16.08075983,10
92.313692,3394.355042,9.189005615,5.459584892,18.65493977,1.983195325,9,1,16.08075983,10.
78,1,1,0,0,1,12,4092.313692,3394.355042,9.189005615,5.459584892,18.65493977,1.983195325,9,1
5615,5.459584892,18.65493977,1.983195325,9,1,16.08075983,10.91916978,166.7189452,82.70105
92.313692,3394.355042,9.189005615,5.459584892,18.65493977,1.983195325,9,1,16.08075983,10.
92.313692,3394.355042,9.189005615,5.459584892,18.65493977,1.983195325,9,1,16.08075983,10.
05615,5.459584892,18.65493977,1.983195325,9,1,16.08075983,10.91916978,166.7189452,82.7010
092.313692,3394.355042,9.189005615,5.459584892,18.65493977,1.983195325,9,1,16.08075983,10
3,78,1,1,0,0,1,12,4092.313692,3394.355042,9.189005615,5.459584892,18.65493977,1.983195325,9
5615,5.459584892,18.65493977,1.983195325,9,1,16.08075983,10.91916978,166.7189452,82.70105
70,1,1,0,0,1,12,3278.02934,3573.773391,9.892468299,0,13.14713297,0,13,0,15.16845139,17.7744
92468299,0,13.14713297,0,13,0,15.16845139,17.77446542,207.1366211,79.79924427,71.09786169
278.02934,3573.773391,9.892468299,0,13.14713297,0,13,0,15.16845139,17.77446542,207.136621
892468299,0,13.14713297,0,13,0,15.16845139,17.77446542,207.1366211,79.79924427,71.097861
99,0,13.14713297,0,13,0,15.16845139,17.77446542,207.1366211,79.79924427,71.09786169,14.508
70,1,1,0,0,1,12,3278.02934,3573.773391,9.892468299,0,13.14713297,0,13,0,15.16845139,17.7744
70,1,1,0,0,1,12,3278.02934,3573.773391,9.892468299,0,13.14713297,0,13,0,15.16845139,17.7744
,3278.02934,3573.773391,9.892468299,0,13.14713297,0,13,0,15.16845139,17.77446542,207.1366
92468299,0,13.14713297,0,13,0,15.16845139,17.77446542,207.1366211,79.79924427,71.09786169
468299,0,13.14713297,0,13,0,15.16845139,17.77446542,207.1366211,79.79924427,71.09786169,14
,3278.02934,3573.773391,9.892468299,0,13.14713297,0,13,0,15.16845139,17.77446542,207.1366
278.02934,3573.773391,9.892468299,0,13.14713297,0,13,0,15.16845139,17.77446542,207.136621
78.02934,3573.773391,9.892468299,0,13.14713297,0,13,0,15.16845139,17.77446542,207.1366211
468299,0,13.14713297,0,13,0,15.16845139,17.77446542,207.1366211,79.79924427,71.09786169,14

70,1,1,0,0,1,12,3278.02934,3573.773391,9.892468299,0,13.14713297,0,13,0,15.16845139,17.7744
78.02934,3573.773391,9.892468299,0,13.14713297,0,13,0,15.16845139,17.77446542,207.1366211
0,1,1,0,0,1,12,3278.02934,3573.773391,9.892468299,0,13.14713297,0,13,0,15.16845139,17.77446
70,1,1,0,0,1,12,3278.02934,3573.773391,9.892468299,0,13.14713297,0,13,0,15.16845139,17.7744
299,0,13.14713297,0,13,0,15.16845139,17.77446542,207.1366211,79.79924427,71.09786169,14.50
,3278.02934,3573.773391,9.892468299,0,13.14713297,0,13,0,15.16845139,17.77446542,207.1366
299,0,13.14713297,0,13,0,15.16845139,17.77446542,207.1366211,79.79924427,71.09786169,14.50
468299,0,13.14713297,0,13,0,15.16845139,17.77446542,207.1366211,79.79924427,71.09786169,1
70,1,1,0,0,1,12,3278.02934,3573.773391,9.892468299,0,13.14713297,0,13,0,15.16845139,17.7744
70,1,1,0,0,1,12,3278.02934,3573.773391,9.892468299,0,13.14713297,0,13,0,15.16845139,17.7744
278.02934,3573.773391,9.892468299,0,13.14713297,0,13,0,15.16845139,17.77446542,207.136621
78.02934,3573.773391,9.892468299,0,13.14713297,0,13,0,15.16845139,17.77446542,207.1366211
468299,0,13.14713297,0,13,0,15.16845139,17.77446542,207.1366211,79.79924427,71.09786169,1
278.02934,3573.773391,9.892468299,0,13.14713297,0,13,0,15.16845139,17.77446542,207.136621
299,0,13.14713297,0,13,0,15.16845139,17.77446542,207.1366211,79.79924427,71.09786169,14.50
,21.9512875,26.18626234,20.57036637,11,7,14.60139147,21.9512875,17.06123605,57.82151022,
3,83,1,0,1,0,1,5,4988.767506,5107.342735,10.51300186,21.9512875,26.18626234,20.57036637,11,
186,21.9512875,26.18626234,20.57036637,11,7,14.60139147,21.9512875,17.06123605,57.8215102
8.767506,5107.342735,10.51300186,21.9512875,26.18626234,20.57036637,11,7,14.60139147,21.9
3,1,0,1,0,1,5,4988.767506,5107.342735,10.51300186,21.9512875,26.18626234,20.57036637,11,7,1
3,1,0,1,0,1,5,4988.767506,5107.342735,10.51300186,21.9512875,26.18626234,20.57036637,11,7,1
8.767506,5107.342735,10.51300186,21.9512875,26.18626234,20.57036637,11,7,14.60139147,21.9
186,21.9512875,26.18626234,20.57036637,11,7,14.60139147,21.9512875,17.06123605,57.8215102
3,1,0,1,0,1,5,4988.767506,5107.342735,10.51300186,21.9512875,26.18626234,20.57036637,11,7,1
300186,21.9512875,26.18626234,20.57036637,11,7,14.60139147,21.9512875,17.06123605,57.8215
51300186,21.9512875,26.18626234,20.57036637,11,7,14.60139147,21.9512875,17.06123605,57.82
8.767506,5107.342735,10.51300186,21.9512875,26.18626234,20.57036637,11,7,14.60139147,21.9
35,10.51300186,21.9512875,26.18626234,20.57036637,11,7,14.60139147,21.9512875,17.06123605
51300186,21.9512875,26.18626234,20.57036637,11,7,14.60139147,21.9512875,17.06123605,57.82
1300186,21.9512875,26.18626234,20.57036637,11,7,14.60139147,21.9512875,17.06123605,57.821
186,21.9512875,26.18626234,20.57036637,11,7,14.60139147,21.9512875,17.06123605,57.8215102
36,21.9512875,26.18626234,20.57036637,11,7,14.60139147,21.9512875,17.06123605,57.82151022
8.767506,5107.342735,10.51300186,21.9512875,26.18626234,20.57036637,11,7,14.60139147,21.9
8.767506,5107.342735,10.51300186,21.9512875,26.18626234,20.57036637,11,7,14.60139147,21.9
8.767506,5107.342735,10.51300186,21.9512875,26.18626234,20.57036637,11,7,14.60139147,21.9
3,83,1,0,1,0,1,5,4988.767506,5107.342735,10.51300186,21.9512875,26.18626234,20.57036637,11,
0.51300186,21.9512875,26.18626234,20.57036637,11,7,14.60139147,21.9512875,17.06123605,57.
186,21.9512875,26.18626234,20.57036637,11,7,14.60139147,21.9512875,17.06123605,57.8215102
51300186,21.9512875,26.18626234,20.57036637,11,7,14.60139147,21.9512875,17.06123605,57.82
186,21.9512875,26.18626234,20.57036637,11,7,14.60139147,21.9512875,17.06123605,57.8215102
.51300186,21.9512875,26.18626234,20.57036637,11,7,14.60139147,21.9512875,17.06123605,57.8
8.767506,5107.342735,10.51300186,21.9512875,26.18626234,20.57036637,11,7,14.60139147,21.9
8.767506,5107.342735,10.51300186,21.9512875,26.18626234,20.57036637,11,7,14.60139147,21.9
8.767506,5107.342735,10.51300186,21.9512875,26.18626234,20.57036637,11,7,14.60139147,21.9
.51300186,21.9512875,26.18626234,20.57036637,11,7,14.60139147,21.9512875,17.06123605,57.8
8.767506,5107.342735,10.51300186,21.9512875,26.18626234,20.57036637,11,7,14.60139147,21.9
8.767506,5107.342735,10.51300186,21.9512875,26.18626234,20.57036637,11,7,14.60139147,21.9
186,21.9512875,26.18626234,20.57036637,11,7,14.60139147,21.9512875,17.06123605,57.8215102
36,21.9512875,26.18626234,20.57036637,11,7,14.60139147,21.9512875,17.06123605,57.82151022
988.767506,5107.342735,10.51300186,21.9512875,26.18626234,20.57036637,11,7,14.60139147,2

8.767506,5107.342735,10.51300186,21.9512875,26.18626234,20.57036637,11,7,14.60139147,21.9
36,21.9512875,26.18626234,20.57036637,11,7,14.60139147,21.9512875,17.06123605,57.82151022
186,21.9512875,26.18626234,20.57036637,11,7,14.60139147,21.9512875,17.06123605,57.8215102
8.767506,5107.342735,10.51300186,21.9512875,26.18626234,20.57036637,11,7,14.60139147,21.9
8.767506,5107.342735,10.51300186,21.9512875,26.18626234,20.57036637,11,7,14.60139147,21.9
3,83,1,0,1,0,1,5,4988.767506,5107.342735,10.51300186,21.9512875,26.18626234,20.57036637,11,
35,10.51300186,21.9512875,26.18626234,20.57036637,11,7,14.60139147,21.9512875,17.06123605
3518,18.93627635,41.28400737,10.68573,12,5,16.55043723,18.93627635,196.2574913,59.9239968
63.058463,5668.318631,10.27268518,18.93627635,41.28400737,10.68573,12,5,16.55043723,18.93
63.058463,5668.318631,10.27268518,18.93627635,41.28400737,10.68573,12,5,16.55043723,18.93
518,18.93627635,41.28400737,10.68573,12,5,16.55043723,18.93627635,196.2574913,59.92399686
3.058463,5668.318631,10.27268518,18.93627635,41.28400737,10.68573,12,5,16.55043723,18.936
7268518,18.93627635,41.28400737,10.68573,12,5,16.55043723,18.93627635,196.2574913,59.9239
3.058463,5668.318631,10.27268518,18.93627635,41.28400737,10.68573,12,5,16.55043723,18.936
63.058463,5668.318631,10.27268518,18.93627635,41.28400737,10.68573,12,5,16.55043723,18.93
63.058463,5668.318631,10.27268518,18.93627635,41.28400737,10.68573,12,5,16.55043723,18.93
18,18.93627635,41.28400737,10.68573,12,5,16.55043723,18.93627635,196.2574913,59.92399686,
72,0,0,0,1,1,9,4963.058463,5668.318631,10.27268518,18.93627635,41.28400737,10.68573,12,5,16
518,18.93627635,41.28400737,10.68573,12,5,16.55043723,18.93627635,196.2574913,59.92399686
518,18.93627635,41.28400737,10.68573,12,5,16.55043723,18.93627635,196.2574913,59.92399686
63.058463,5668.318631,10.27268518,18.93627635,41.28400737,10.68573,12,5,16.55043723,18.93
268518,18.93627635,41.28400737,10.68573,12,5,16.55043723,18.93627635,196.2574913,59.92399
72,0,0,0,1,1,9,4963.058463,5668.318631,10.27268518,18.93627635,41.28400737,10.68573,12,5,16
72,0,0,0,1,1,9,4963.058463,5668.318631,10.27268518,18.93627635,41.28400737,10.68573,12,5,16
963.058463,5668.318631,10.27268518,18.93627635,41.28400737,10.68573,12,5,16.55043723,18.9
963.058463,5668.318631,10.27268518,18.93627635,41.28400737,10.68573,12,5,16.55043723,18.9
58518,18.93627635,41.28400737,10.68573,12,5,16.55043723,18.93627635,196.2574913,59.923996
3.058463,5668.318631,10.27268518,18.93627635,41.28400737,10.68573,12,5,16.55043723,18.936
63.058463,5668.318631,10.27268518,18.93627635,41.28400737,10.68573,12,5,16.55043723,18.93
3.058463,5668.318631,10.27268518,18.93627635,41.28400737,10.68573,12,5,16.55043723,18.936
268518,18.93627635,41.28400737,10.68573,12,5,16.55043723,18.93627635,196.2574913,59.92399
3518,18.93627635,41.28400737,10.68573,12,5,16.55043723,18.93627635,196.2574913,59.9239968
63.058463,5668.318631,10.27268518,18.93627635,41.28400737,10.68573,12,5,16.55043723,18.93
72,0,0,0,1,1,9,4963.058463,5668.318631,10.27268518,18.93627635,41.28400737,10.68573,12,5,16
63.058463,5668.318631,10.27268518,18.93627635,41.28400737,10.68573,12,5,16.55043723,18.93
3,72,0,0,0,1,1,9,4963.058463,5668.318631,10.27268518,18.93627635,41.28400737,10.68573,12,5,1
3518,18.93627635,41.28400737,10.68573,12,5,16.55043723,18.93627635,196.2574913,59.9239968
72,0,0,0,1,1,9,4963.058463,5668.318631,10.27268518,18.93627635,41.28400737,10.68573,12,5,16
63.058463,5668.318631,10.27268518,18.93627635,41.28400737,10.68573,12,5,16.55043723,18.93
3.058463,5668.318631,10.27268518,18.93627635,41.28400737,10.68573,12,5,16.55043723,18.936
58518,18.93627635,41.28400737,10.68573,12,5,16.55043723,18.93627635,196.2574913,59.923996
18,18.93627635,41.28400737,10.68573,12,5,16.55043723,18.93627635,196.2574913,59.92399686,
63.058463,5668.318631,10.27268518,18.93627635,41.28400737,10.68573,12,5,16.55043723,18.93
3.058463,5668.318631,10.27268518,18.93627635,41.28400737,10.68573,12,5,16.55043723,18.936
3.058463,5668.318631,10.27268518,18.93627635,41.28400737,10.68573,12,5,16.55043723,18.936
7268518,18.93627635,41.28400737,10.68573,12,5,16.55043723,18.93627635,196.2574913,59.9239
5738323,14.22615519,21.2492004,9.793979412,9,3,17.51103524,18.96820692,214.806331,72.8459
280.803481,3942.205668,9.105738323,14.22615519,21.2492004,9.793979412,9,3,17.51103524,18.
80.803481,3942.205668,9.105738323,14.22615519,21.2492004,9.793979412,9,3,17.51103524,18.9
80.803481,3942.205668,9.105738323,14.22615519,21.2492004,9.793979412,9,3,17.51103524,18.9

80.803481,3942.205668,9.105738323,14.22615519,21.2492004,9.793979412,9,3,17.51103524,18.9
323,14.22615519,21.2492004,9.793979412,9,3,17.51103524,18.96820692,214.806331,72.84590659
3323,14.22615519,21.2492004,9.793979412,9,3,17.51103524,18.96820692,214.806331,72.8459065
3323,14.22615519,21.2492004,9.793979412,9,3,17.51103524,18.96820692,214.806331,72.8459065
80,1,1,0,0,1,12,4280.803481,3942.205668,9.105738323,14.22615519,21.2492004,9.793979412,9,3,
280.803481,3942.205668,9.105738323,14.22615519,21.2492004,9.793979412,9,3,17.51103524,18.
738323,14.22615519,21.2492004,9.793979412,9,3,17.51103524,18.96820692,214.806331,72.84590
80.803481,3942.205668,9.105738323,14.22615519,21.2492004,9.793979412,9,3,17.51103524,18.9
3,80,1,1,0,0,1,12,4280.803481,3942.205668,9.105738323,14.22615519,21.2492004,9.793979412,9,
280.803481,3942.205668,9.105738323,14.22615519,21.2492004,9.793979412,9,3,17.51103524,18.
4280.803481,3942.205668,9.105738323,14.22615519,21.2492004,9.793979412,9,3,17.51103524,1
105738323,14.22615519,21.2492004,9.793979412,9,3,17.51103524,18.96820692,214.806331,72.84
323,14.22615519,21.2492004,9.793979412,9,3,17.51103524,18.96820692,214.806331,72.84590659
9.105738323,14.22615519,21.2492004,9.793979412,9,3,17.51103524,18.96820692,214.806331,72.
3,80,1,1,0,0,1,12,4280.803481,3942.205668,9.105738323,14.22615519,21.2492004,9.793979412,9,
3323,14.22615519,21.2492004,9.793979412,9,3,17.51103524,18.96820692,214.806331,72.8459065
80,1,1,0,0,1,12,4280.803481,3942.205668,9.105738323,14.22615519,21.2492004,9.793979412,9,3,
80.803481,3942.205668,9.105738323,14.22615519,21.2492004,9.793979412,9,3,17.51103524,18.9
80.803481,3942.205668,9.105738323,14.22615519,21.2492004,9.793979412,9,3,17.51103524,18.9
5738323,14.22615519,21.2492004,9.793979412,9,3,17.51103524,18.96820692,214.806331,72.8459
9.105738323,14.22615519,21.2492004,9.793979412,9,3,17.51103524,18.96820692,214.806331,72.
738323,14.22615519,21.2492004,9.793979412,9,3,17.51103524,18.96820692,214.806331,72.84590
280.803481,3942.205668,9.105738323,14.22615519,21.2492004,9.793979412,9,3,17.51103524,18.
80.803481,3942.205668,9.105738323,14.22615519,21.2492004,9.793979412,9,3,17.51103524,18.9
80,1,1,0,0,1,12,4280.803481,3942.205668,9.105738323,14.22615519,21.2492004,9.793979412,9,3,
80.803481,3942.205668,9.105738323,14.22615519,21.2492004,9.793979412,9,3,17.51103524,18.9
105738323,14.22615519,21.2492004,9.793979412,9,3,17.51103524,18.96820692,214.806331,72.84
,4280.803481,3942.205668,9.105738323,14.22615519,21.2492004,9.793979412,9,3,17.51103524,1
280.803481,3942.205668,9.105738323,14.22615519,21.2492004,9.793979412,9,3,17.51103524,18.
280.803481,3942.205668,9.105738323,14.22615519,21.2492004,9.793979412,9,3,17.51103524,18.
80.803481,3942.205668,9.105738323,14.22615519,21.2492004,9.793979412,9,3,17.51103524,18.9
4942,29.05075638,26.18626234,16.32446614,11,7,14.50486021,24.90064832,138.3514828,58.0194
77,1,1,1,0,1,11,5009.894787,6013.015951,9.863304942,29.05075638,26.18626234,16.32446614,11
4942,29.05075638,26.18626234,16.32446614,11,7,14.50486021,24.90064832,138.3514828,58.0194
4942,29.05075638,26.18626234,16.32446614,11,7,14.50486021,24.90064832,138.3514828,58.0194
09.894787,6013.015951,9.863304942,29.05075638,26.18626234,16.32446614,11,7,14.50486021,2
7,1,1,1,0,1,11,5009.894787,6013.015951,9.863304942,29.05075638,26.18626234,16.32446614,11,
09.894787,6013.015951,9.863304942,29.05075638,26.18626234,16.32446614,11,7,14.50486021,2
009.894787,6013.015951,9.863304942,29.05075638,26.18626234,16.32446614,11,7,14.50486021,
009.894787,6013.015951,9.863304942,29.05075638,26.18626234,16.32446614,11,7,14.50486021,
5009.894787,6013.015951,9.863304942,29.05075638,26.18626234,16.32446614,11,7,14.50486021
009.894787,6013.015951,9.863304942,29.05075638,26.18626234,16.32446614,11,7,14.50486021,
4942,29.05075638,26.18626234,16.32446614,11,7,14.50486021,24.90064832,138.3514828,58.0194
77,1,1,1,0,1,11,5009.894787,6013.015951,9.863304942,29.05075638,26.18626234,16.32446614,11
009.894787,6013.015951,9.863304942,29.05075638,26.18626234,16.32446614,11,7,14.50486021,
4942,29.05075638,26.18626234,16.32446614,11,7,14.50486021,24.90064832,138.3514828,58.0194
9.894787,6013.015951,9.863304942,29.05075638,26.18626234,16.32446614,11,7,14.50486021,24.
009.894787,6013.015951,9.863304942,29.05075638,26.18626234,16.32446614,11,7,14.50486021,
09.894787,6013.015951,9.863304942,29.05075638,26.18626234,16.32446614,11,7,14.50486021,2
009.894787,6013.015951,9.863304942,29.05075638,26.18626234,16.32446614,11,7,14.50486021,

09.894787,6013.015951,9.863304942,29.05075638,26.18626234,16.32446614,11,7,14.50486021,2
9691162,3,2,218,77,1,1,1,0,1,11,5009.894787,6013.015951,9.863304942,29.05075638,26.18626234
942,29.05075638,26.18626234,16.32446614,11,7,14.50486021,24.90064832,138.3514828,58.0194
5009.894787,6013.015951,9.863304942,29.05075638,26.18626234,16.32446614,11,7,14.50486021
5009.894787,6013.015951,9.863304942,29.05075638,26.18626234,16.32446614,11,7,14.50486021
,77,1,1,1,0,1,11,5009.894787,6013.015951,9.863304942,29.05075638,26.18626234,16.32446614,1
009.894787,6013.015951,9.863304942,29.05075638,26.18626234,16.32446614,11,7,14.50486021,
09.894787,6013.015951,9.863304942,29.05075638,26.18626234,16.32446614,11,7,14.50486021,2
09.894787,6013.015951,9.863304942,29.05075638,26.18626234,16.32446614,11,7,14.50486021,2
942,29.05075638,26.18626234,16.32446614,11,7,14.50486021,24.90064832,138.3514828,58.0194
09.894787,6013.015951,9.863304942,29.05075638,26.18626234,16.32446614,11,7,14.50486021,2
77,1,1,1,0,1,11,5009.894787,6013.015951,9.863304942,29.05075638,26.18626234,16.32446614,11
09.894787,6013.015951,9.863304942,29.05075638,26.18626234,16.32446614,11,7,14.50486021,2
77,1,1,1,0,1,11,5009.894787,6013.015951,9.863304942,29.05075638,26.18626234,16.32446614,11
009.894787,6013.015951,9.863304942,29.05075638,26.18626234,16.32446614,11,7,14.50486021,
009.894787,6013.015951,9.863304942,29.05075638,26.18626234,16.32446614,11,7,14.50486021,
92,11.11269543,24.2865642,3.821901933,10,2,18.55408421,18.52115904,54.3566739,73.617818,
1.101314,2851.382529,7.780744992,11.11269543,24.2865642,3.821901933,10,2,18.55408421,18.5
01.101314,2851.382529,7.780744992,11.11269543,24.2865642,3.821901933,10,2,18.55408421,18.
92,11.11269543,24.2865642,3.821901933,10,2,18.55408421,18.52115904,54.3566739,73.617818,
,79,1,1,1,0,1,9,3901.101314,2851.382529,7.780744992,11.11269543,24.2865642,3.821901933,10,
901.101314,2851.382529,7.780744992,11.11269543,24.2865642,3.821901933,10,2,18.55408421,1
,79,1,1,1,0,1,9,3901.101314,2851.382529,7.780744992,11.11269543,24.2865642,3.821901933,10,
01.101314,2851.382529,7.780744992,11.11269543,24.2865642,3.821901933,10,2,18.55408421,18.
,3901.101314,2851.382529,7.780744992,11.11269543,24.2865642,3.821901933,10,2,18.55408421,
,79,1,1,1,0,1,9,3901.101314,2851.382529,7.780744992,11.11269543,24.2865642,3.821901933,10,
79,1,1,1,0,1,9,3901.101314,2851.382529,7.780744992,11.11269543,24.2865642,3.821901933,10,2,
92,11.11269543,24.2865642,3.821901933,10,2,18.55408421,18.52115904,54.3566739,73.617818,
01.101314,2851.382529,7.780744992,11.11269543,24.2865642,3.821901933,10,2,18.55408421,18.
4992,11.11269543,24.2865642,3.821901933,10,2,18.55408421,18.52115904,54.3566739,73.61781
,79,1,1,1,0,1,9,3901.101314,2851.382529,7.780744992,11.11269543,24.2865642,3.821901933,10,
780744992,11.11269543,24.2865642,3.821901933,10,2,18.55408421,18.52115904,54.3566739,73.
,79,1,1,1,0,1,9,3901.101314,2851.382529,7.780744992,11.11269543,24.2865642,3.821901933,10,
1.101314,2851.382529,7.780744992,11.11269543,24.2865642,3.821901933,10,2,18.55408421,18.5
901.101314,2851.382529,7.780744992,11.11269543,24.2865642,3.821901933,10,2,18.55408421,1
2,11.11269543,24.2865642,3.821901933,10,2,18.55408421,18.52115904,54.3566739,73.617818,8
1.101314,2851.382529,7.780744992,11.11269543,24.2865642,3.821901933,10,2,18.55408421,18.5
44992,11.11269543,24.2865642,3.821901933,10,2,18.55408421,18.52115904,54.3566739,73.6178
01.101314,2851.382529,7.780744992,11.11269543,24.2865642,3.821901933,10,2,18.55408421,18.
92,11.11269543,24.2865642,3.821901933,10,2,18.55408421,18.52115904,54.3566739,73.617818,
901.101314,2851.382529,7.780744992,11.11269543,24.2865642,3.821901933,10,2,18.55408421,1
92,11.11269543,24.2865642,3.821901933,10,2,18.55408421,18.52115904,54.3566739,73.617818,
1.101314,2851.382529,7.780744992,11.11269543,24.2865642,3.821901933,10,2,18.55408421,18.5
92,11.11269543,24.2865642,3.821901933,10,2,18.55408421,18.52115904,54.3566739,73.617818,
780744992,11.11269543,24.2865642,3.821901933,10,2,18.55408421,18.52115904,54.3566739,73.
76,14.85238083,24.2865642,3.821901933,10,2,17.02406255,14.85238083,144.2624806,73.966616
55.192467,3124.816474,7.044439676,14.85238083,24.2865642,3.821901933,10,2,17.02406255,14.
855.192467,3124.816474,7.044439676,14.85238083,24.2865642,3.821901933,10,2,17.02406255,1
55.192467,3124.816474,7.044439676,14.85238083,24.2865642,3.821901933,10,2,17.02406255,14.
76,14.85238083,24.2865642,3.821901933,10,2,17.02406255,14.85238083,144.2624806,73.966616

55.192467,3124.816474,7.044439676,14.85238083,24.2865642,3.821901933,10,2,17.02406255,14.
0617676,3,2,218,77,0,0,0,1,1,7,3855.192467,3124.816474,7.044439676,14.85238083,24.2865642,3.
576,14.85238083,24.2865642,3.821901933,10,2,17.02406255,14.85238083,144.2624806,73.966616
576,14.85238083,24.2865642,3.821901933,10,2,17.02406255,14.85238083,144.2624806,73.966616
5.192467,3124.816474,7.044439676,14.85238083,24.2865642,3.821901933,10,2,17.02406255,14.8
5.192467,3124.816474,7.044439676,14.85238083,24.2865642,3.821901933,10,2,17.02406255,14.8
55.192467,3124.816474,7.044439676,14.85238083,24.2865642,3.821901933,10,2,17.02406255,14.
855.192467,3124.816474,7.044439676,14.85238083,24.2865642,3.821901933,10,2,17.02406255,14
576,14.85238083,24.2865642,3.821901933,10,2,17.02406255,14.85238083,144.2624806,73.966616
855.192467,3124.816474,7.044439676,14.85238083,24.2865642,3.821901933,10,2,17.02406255,14
3,77,0,0,0,1,1,7,3855.192467,3124.816474,7.044439676,14.85238083,24.2865642,3.821901933,10,
5.192467,3124.816474,7.044439676,14.85238083,24.2865642,3.821901933,10,2,17.02406255,14.8
5.192467,3124.816474,7.044439676,14.85238083,24.2865642,3.821901933,10,2,17.02406255,14.8
855.192467,3124.816474,7.044439676,14.85238083,24.2865642,3.821901933,10,2,17.02406255,14
79382324,3,2,218,77,0,0,0,1,1,7,3855.192467,3124.816474,7.044439676,14.85238083,24.2865642,
576,14.85238083,24.2865642,3.821901933,10,2,17.02406255,14.85238083,144.2624806,73.966616
77,0,0,0,1,1,7,3855.192467,3124.816474,7.044439676,14.85238083,24.2865642,3.821901933,10,2,
77,0,0,0,1,1,7,3855.192467,3124.816474,7.044439676,14.85238083,24.2865642,3.821901933,10,2,
855.192467,3124.816474,7.044439676,14.85238083,24.2865642,3.821901933,10,2,17.02406255,14
55.192467,3124.816474,7.044439676,14.85238083,24.2865642,3.821901933,10,2,17.02406255,14.
7,0,0,0,1,1,7,3855.192467,3124.816474,7.044439676,14.85238083,24.2865642,3.821901933,10,2,1
5.192467,3124.816474,7.044439676,14.85238083,24.2865642,3.821901933,10,2,17.02406255,14.8
5.192467,3124.816474,7.044439676,14.85238083,24.2865642,3.821901933,10,2,17.02406255,14.8
5.192467,3124.816474,7.044439676,14.85238083,24.2865642,3.821901933,10,2,17.02406255,14.8
5.192467,3124.816474,7.044439676,14.85238083,24.2865642,3.821901933,10,2,17.02406255,14.8
5.192467,3124.816474,7.044439676,14.85238083,24.2865642,3.821901933,10,2,17.02406255,14.8
5.192467,3124.816474,7.044439676,14.85238083,24.2865642,3.821901933,10,2,17.02406255,14.8
855.192467,3124.816474,7.044439676,14.85238083,24.2865642,3.821901933,10,2,17.02406255,14
576,14.85238083,24.2865642,3.821901933,10,2,17.02406255,14.85238083,144.2624806,73.966616
576,14.85238083,24.2865642,3.821901933,10,2,17.02406255,14.85238083,144.2624806,73.966616
5.192467,3124.816474,7.044439676,14.85238083,24.2865642,3.821901933,10,2,17.02406255,14.8
855.192467,3124.816474,7.044439676,14.85238083,24.2865642,3.821901933,10,2,17.02406255,14
5.192467,3124.816474,7.044439676,14.85238083,24.2865642,3.821901933,10,2,17.02406255,14.8
5.192467,3124.816474,7.044439676,14.85238083,24.2865642,3.821901933,10,2,17.02406255,14.8
7,0,0,0,1,1,7,3855.192467,3124.816474,7.044439676,14.85238083,24.2865642,3.821901933,10,2,1
0617676,3,2,218,67,0,1,0,1,1,11,4331.19471,4305.44311,9.364784769,13.90691692,23.14889855,9.
64784769,13.90691692,23.14889855,9.793979412,10,3,18.06065634,18.54255589,271.2847128,69
,4331.19471,4305.44311,9.364784769,13.90691692,23.14889855,9.793979412,10,3,18.06065634,1
4331.19471,4305.44311,9.364784769,13.90691692,23.14889855,9.793979412,10,3,18.06065634,18
.364784769,13.90691692,23.14889855,9.793979412,10,3,18.06065634,18.54255589,271.2847128,6
3,67,0,1,0,1,1,11,4331.19471,4305.44311,9.364784769,13.90691692,23.14889855,9.793979412,10,3
31.19471,4305.44311,9.364784769,13.90691692,23.14889855,9.793979412,10,3,18.06065634,18.5
3,67,0,1,0,1,1,11,4331.19471,4305.44311,9.364784769,13.90691692,23.14889855,9.793979412,10,3
59,13.90691692,23.14889855,9.793979412,10,3,18.06065634,18.54255589,271.2847128,69.566972
31.19471,4305.44311,9.364784769,13.90691692,23.14889855,9.793979412,10,3,18.06065634,18.5
4331.19471,4305.44311,9.364784769,13.90691692,23.14889855,9.793979412,10,3,18.06065634,18
67,0,1,0,1,1,11,4331.19471,4305.44311,9.364784769,13.90691692,23.14889855,9.793979412,10,3,
331.19471,4305.44311,9.364784769,13.90691692,23.14889855,9.793979412,10,3,18.06065634,18.
34769,13.90691692,23.14889855,9.793979412,10,3,18.06065634,18.54255589,271.2847128,69.566
4331.19471,4305.44311,9.364784769,13.90691692,23.14889855,9.793979412,10,3,18.06065634,18

,4331.19471,4305.44311,9.364784769,13.90691692,23.14889855,9.793979412,10,3,18.06065634,1
4331.19471,4305.44311,9.364784769,13.90691692,23.14889855,9.793979412,10,3,18.06065634,18
59,13.90691692,23.14889855,9.793979412,10,3,18.06065634,18.54255589,271.2847128,69.566972
9,13.90691692,23.14889855,9.793979412,10,3,18.06065634,18.54255589,271.2847128,69.5669725
59,13.90691692,23.14889855,9.793979412,10,3,18.06065634,18.54255589,271.2847128,69.566972
13.90691692,23.14889855,9.793979412,10,3,18.06065634,18.54255589,271.2847128,69.56697257
67,0,1,0,1,1,11,4331.19471,4305.44311,9.364784769,13.90691692,23.14889855,9.793979412,10,3,
31.19471,4305.44311,9.364784769,13.90691692,23.14889855,9.793979412,10,3,18.06065634,18.5
331.19471,4305.44311,9.364784769,13.90691692,23.14889855,9.793979412,10,3,18.06065634,18.
4331.19471,4305.44311,9.364784769,13.90691692,23.14889855,9.793979412,10,3,18.06065634,18
4577.866569,4611.536619,11.01513096,27.79283881,24.2865642,17.53300257,10,6,15.55077312,2
77.866569,4611.536619,11.01513096,27.79283881,24.2865642,17.53300257,10,6,15.55077312,23.
82,1,0,1,1,1,10,4577.866569,4611.536619,11.01513096,27.79283881,24.2865642,17.53300257,10,6
4577.866569,4611.536619,11.01513096,27.79283881,24.2865642,17.53300257,10,6,15.55077312,2
1513096,27.79283881,24.2865642,17.53300257,10,6,15.55077312,23.82243327,63.80648284,62.20
577.866569,4611.536619,11.01513096,27.79283881,24.2865642,17.53300257,10,6,15.55077312,23
77.866569,4611.536619,11.01513096,27.79283881,24.2865642,17.53300257,10,6,15.55077312,23.
577.866569,4611.536619,11.01513096,27.79283881,24.2865642,17.53300257,10,6,15.55077312,23
20617676,3,2,218,82,1,0,1,1,1,10,4577.866569,4611.536619,11.01513096,27.79283881,24.2865642
3096,27.79283881,24.2865642,17.53300257,10,6,15.55077312,23.82243327,63.80648284,62.20309
4577.866569,4611.536619,11.01513096,27.79283881,24.2865642,17.53300257,10,6,15.55077312,2
77.866569,4611.536619,11.01513096,27.79283881,24.2865642,17.53300257,10,6,15.55077312,23.
3,82,1,0,1,1,1,10,4577.866569,4611.536619,11.01513096,27.79283881,24.2865642,17.53300257,10
3,82,1,0,1,1,1,10,4577.866569,4611.536619,11.01513096,27.79283881,24.2865642,17.53300257,10
3096,27.79283881,24.2865642,17.53300257,10,6,15.55077312,23.82243327,63.80648284,62.20309
,4577.866569,4611.536619,11.01513096,27.79283881,24.2865642,17.53300257,10,6,15.55077312,
77.866569,4611.536619,11.01513096,27.79283881,24.2865642,17.53300257,10,6,15.55077312,23.
3,82,1,0,1,1,1,10,4577.866569,4611.536619,11.01513096,27.79283881,24.2865642,17.53300257,10
4577.866569,4611.536619,11.01513096,27.79283881,24.2865642,17.53300257,10,6,15.55077312,2
3096,27.79283881,24.2865642,17.53300257,10,6,15.55077312,23.82243327,63.80648284,62.20309
1513096,27.79283881,24.2865642,17.53300257,10,6,15.55077312,23.82243327,63.80648284,62.20
4577.866569,4611.536619,11.01513096,27.79283881,24.2865642,17.53300257,10,6,15.55077312,2
77.866569,4611.536619,11.01513096,27.79283881,24.2865642,17.53300257,10,6,15.55077312,23.
4577.866569,4611.536619,11.01513096,27.79283881,24.2865642,17.53300257,10,6,15.55077312,2
1513096,27.79283881,24.2865642,17.53300257,10,6,15.55077312,23.82243327,63.80648284,62.20
01513096,27.79283881,24.2865642,17.53300257,10,6,15.55077312,23.82243327,63.80648284,62.2
1513096,27.79283881,24.2865642,17.53300257,10,6,15.55077312,23.82243327,63.80648284,62.20
82,1,0,1,1,1,10,4577.866569,4611.536619,11.01513096,27.79283881,24.2865642,17.53300257,10,6
77.866569,4611.536619,11.01513096,27.79283881,24.2865642,17.53300257,10,6,15.55077312,23.
20617676,3,2,218,82,1,0,1,1,1,10,4577.866569,4611.536619,11.01513096,27.79283881,24.2865642
77.866569,4611.536619,11.01513096,27.79283881,24.2865642,17.53300257,10,6,15.55077312,23.
.01513096,27.79283881,24.2865642,17.53300257,10,6,15.55077312,23.82243327,63.80648284,62.
4577.866569,4611.536619,11.01513096,27.79283881,24.2865642,17.53300257,10,6,15.55077312,2
53,37.83250639,17.25834534,1.442051805,23,9,28.09955754,33.62889457,277.8907759,222.59257
67,1,1,0,1,0,8,16797.86577,13188.21368,14.32526463,37.83250639,17.25834534,1.442051805,23,9
797.86577,13188.21368,14.32526463,37.83250639,17.25834534,1.442051805,23,9,28.09955754,33
97.86577,13188.21368,14.32526463,37.83250639,17.25834534,1.442051805,23,9,28.09955754,33.
463,37.83250639,17.25834534,1.442051805,23,9,28.09955754,33.62889457,277.8907759,222.5925
97.86577,13188.21368,14.32526463,37.83250639,17.25834534,1.442051805,23,9,28.09955754,33.
67,1,1,0,1,0,8,16797.86577,13188.21368,14.32526463,37.83250639,17.25834534,1.442051805,23,9

67,1,1,0,1,0,8,16797.86577,13188.21368,14.32526463,37.83250639,17.25834534,1.442051805,23,
97.86577,13188.21368,14.32526463,37.83250639,17.25834534,1.442051805,23,9,28.09955754,33.
97.86577,13188.21368,14.32526463,37.83250639,17.25834534,1.442051805,23,9,28.09955754,33.
3,37.83250639,17.25834534,1.442051805,23,9,28.09955754,33.62889457,277.8907759,222.592573
797.86577,13188.21368,14.32526463,37.83250639,17.25834534,1.442051805,23,9,28.09955754,3
797.86577,13188.21368,14.32526463,37.83250639,17.25834534,1.442051805,23,9,28.09955754,3
67,1,1,0,1,0,8,16797.86577,13188.21368,14.32526463,37.83250639,17.25834534,1.442051805,23,
797.86577,13188.21368,14.32526463,37.83250639,17.25834534,1.442051805,23,9,28.09955754,3
67,1,1,0,1,0,8,16797.86577,13188.21368,14.32526463,37.83250639,17.25834534,1.442051805,23,
7,1,1,0,1,0,8,16797.86577,13188.21368,14.32526463,37.83250639,17.25834534,1.442051805,23,9,
9382324,3,2,203,67,1,1,0,1,0,8,16797.86577,13188.21368,14.32526463,37.83250639,17.25834534,
797.86577,13188.21368,14.32526463,37.83250639,17.25834534,1.442051805,23,9,28.09955754,3
797.86577,13188.21368,14.32526463,37.83250639,17.25834534,1.442051805,23,9,28.09955754,3
7,1,1,0,1,0,8,16797.86577,13188.21368,14.32526463,37.83250639,17.25834534,1.442051805,23,9,
797.86577,13188.21368,14.32526463,37.83250639,17.25834534,1.442051805,23,9,28.09955754,3
67,1,1,0,1,0,8,16797.86577,13188.21368,14.32526463,37.83250639,17.25834534,1.442051805,23,
67,1,1,0,1,0,8,16797.86577,13188.21368,14.32526463,37.83250639,17.25834534,1.442051805,23,
97.86577,13188.21368,14.32526463,37.83250639,17.25834534,1.442051805,23,9,28.09955754,33.
97.86577,13188.21368,14.32526463,37.83250639,17.25834534,1.442051805,23,9,28.09955754,33.
97.86577,13188.21368,14.32526463,37.83250639,17.25834534,1.442051805,23,9,28.09955754,33.
67,1,1,0,1,0,8,16797.86577,13188.21368,14.32526463,37.83250639,17.25834534,1.442051805,23,
67,1,1,0,1,0,8,16797.86577,13188.21368,14.32526463,37.83250639,17.25834534,1.442051805,23,
97.86577,13188.21368,14.32526463,37.83250639,17.25834534,1.442051805,23,9,28.09955754,33.
0308838,3,2,203,67,1,1,0,1,0,8,16797.86577,13188.21368,14.32526463,37.83250639,17.25834534,
6797.86577,13188.21368,14.32526463,37.83250639,17.25834534,1.442051805,23,9,28.09955754,3
463,37.83250639,17.25834534,1.442051805,23,9,28.09955754,33.62889457,277.8907759,222.5925
97.86577,13188.21368,14.32526463,37.83250639,17.25834534,1.442051805,23,9,28.09955754,33.
797.86577,13188.21368,14.32526463,37.83250639,17.25834534,1.442051805,23,9,28.09955754,3
842,21.4448468,16.09081317,1.284477451,24,4,30.86193616,25.73381615,242.5805243,220.10965
575.50861,12563.64133,15.72211842,21.4448468,16.09081317,1.284477451,24,4,30.86193616,25.
67,1,1,0,1,0,9,16575.50861,12563.64133,15.72211842,21.4448468,16.09081317,1.284477451,24,4,
75.50861,12563.64133,15.72211842,21.4448468,16.09081317,1.284477451,24,4,30.86193616,25.7
6575.50861,12563.64133,15.72211842,21.4448468,16.09081317,1.284477451,24,4,30.86193616,2
7,1,1,0,1,0,9,16575.50861,12563.64133,15.72211842,21.4448468,16.09081317,1.284477451,24,4,3
6575.50861,12563.64133,15.72211842,21.4448468,16.09081317,1.284477451,24,4,30.86193616,2
67,1,1,0,1,0,9,16575.50861,12563.64133,15.72211842,21.4448468,16.09081317,1.284477451,24,4,
1842,21.4448468,16.09081317,1.284477451,24,4,30.86193616,25.73381615,242.5805243,220.1096
75.50861,12563.64133,15.72211842,21.4448468,16.09081317,1.284477451,24,4,30.86193616,25.7
67,1,1,0,1,0,9,16575.50861,12563.64133,15.72211842,21.4448468,16.09081317,1.284477451,24,4,
75.50861,12563.64133,15.72211842,21.4448468,16.09081317,1.284477451,24,4,30.86193616,25.7
575.50861,12563.64133,15.72211842,21.4448468,16.09081317,1.284477451,24,4,30.86193616,25.
75.50861,12563.64133,15.72211842,21.4448468,16.09081317,1.284477451,24,4,30.86193616,25.7
3,67,1,1,0,1,0,9,16575.50861,12563.64133,15.72211842,21.4448468,16.09081317,1.284477451,24,
0308838,3,2,203,67,1,1,0,1,0,9,16575.50861,12563.64133,15.72211842,21.4448468,16.09081317,1.
67,1,1,0,1,0,9,16575.50861,12563.64133,15.72211842,21.4448468,16.09081317,1.284477451,24,4,
575.50861,12563.64133,15.72211842,21.4448468,16.09081317,1.284477451,24,4,30.86193616,25.
75.50861,12563.64133,15.72211842,21.4448468,16.09081317,1.284477451,24,4,30.86193616,25.7
842,21.4448468,16.09081317,1.284477451,24,4,30.86193616,25.73381615,242.5805243,220.10965
59845581,3,2,203,67,1,1,0,1,0,9,16575.50861,12563.64133,15.72211842,21.4448468,16.09081317,
575.50861,12563.64133,15.72211842,21.4448468,16.09081317,1.284477451,24,4,30.86193616,25.

6575.50861,12563.64133,15.72211842,21.4448468,16.09081317,1.284477451,24,4,30.86193616,2
6575.50861,12563.64133,15.72211842,21.4448468,16.09081317,1.284477451,24,4,30.86193616,2
575.50861,12563.64133,15.72211842,21.4448468,16.09081317,1.284477451,24,4,30.86193616,25.
67,1,1,0,1,0,9,16575.50861,12563.64133,15.72211842,21.4448468,16.09081317,1.284477451,24,4,
575.50861,12563.64133,15.72211842,21.4448468,16.09081317,1.284477451,24,4,30.86193616,25.
575.50861,12563.64133,15.72211842,21.4448468,16.09081317,1.284477451,24,4,30.86193616,25.
6575.50861,12563.64133,15.72211842,21.4448468,16.09081317,1.284477451,24,4,30.86193616,2
67,1,1,0,1,0,9,16575.50861,12563.64133,15.72211842,21.4448468,16.09081317,1.284477451,24,4,
67,1,1,0,1,0,9,16575.50861,12563.64133,15.72211842,21.4448468,16.09081317,1.284477451,24,4,
575.50861,12563.64133,15.72211842,21.4448468,16.09081317,1.284477451,24,4,30.86193616,25.
67,1,1,0,1,0,9,16575.50861,12563.64133,15.72211842,21.4448468,16.09081317,1.284477451,24,4,
67,1,1,0,1,0,9,16575.50861,12563.64133,15.72211842,21.4448468,16.09081317,1.284477451,24,4,
6575.50861,12563.64133,15.72211842,21.4448468,16.09081317,1.284477451,24,4,30.86193616,2
205,7.636912303,15.46659136,0.910871208,24,2,25.8613194,49.63992997,69.21157369,200.80553
6375.70692,14655.37573,13.18420205,7.636912303,15.46659136,0.910871208,24,2,25.8613194,4
205,7.636912303,15.46659136,0.910871208,24,2,25.8613194,49.63992997,69.21157369,200.8055
74,1,1,1,0,0,12,16375.70692,14655.37573,13.18420205,7.636912303,15.46659136,0.910871208,24
205,7.636912303,15.46659136,0.910871208,24,2,25.8613194,49.63992997,69.21157369,200.8055
375.70692,14655.37573,13.18420205,7.636912303,15.46659136,0.910871208,24,2,25.8613194,49.
74,1,1,1,0,0,12,16375.70692,14655.37573,13.18420205,7.636912303,15.46659136,0.910871208,24
375.70692,14655.37573,13.18420205,7.636912303,15.46659136,0.910871208,24,2,25.8613194,49.
375.70692,14655.37573,13.18420205,7.636912303,15.46659136,0.910871208,24,2,25.8613194,49.
3,74,1,1,1,0,0,12,16375.70692,14655.37573,13.18420205,7.636912303,15.46659136,0.910871208,2
375.70692,14655.37573,13.18420205,7.636912303,15.46659136,0.910871208,24,2,25.8613194,49.
6375.70692,14655.37573,13.18420205,7.636912303,15.46659136,0.910871208,24,2,25.8613194,4
6375.70692,14655.37573,13.18420205,7.636912303,15.46659136,0.910871208,24,2,25.8613194,4
6375.70692,14655.37573,13.18420205,7.636912303,15.46659136,0.910871208,24,2,25.8613194,4
6375.70692,14655.37573,13.18420205,7.636912303,15.46659136,0.910871208,24,2,25.8613194,4
16375.70692,14655.37573,13.18420205,7.636912303,15.46659136,0.910871208,24,2,25.8613194,4
205,7.636912303,15.46659136,0.910871208,24,2,25.8613194,49.63992997,69.21157369,200.8055
205,7.636912303,15.46659136,0.910871208,24,2,25.8613194,49.63992997,69.21157369,200.8055
375.70692,14655.37573,13.18420205,7.636912303,15.46659136,0.910871208,24,2,25.8613194,49.
375.70692,14655.37573,13.18420205,7.636912303,15.46659136,0.910871208,24,2,25.8613194,49.
205,7.636912303,15.46659136,0.910871208,24,2,25.8613194,49.63992997,69.21157369,200.8055
74,1,1,1,0,0,12,16375.70692,14655.37573,13.18420205,7.636912303,15.46659136,0.910871208,24
0617676,3,2,203,74,1,1,1,0,0,12,16375.70692,14655.37573,13.18420205,7.636912303,15.46659136
3,74,1,1,1,0,0,12,16375.70692,14655.37573,13.18420205,7.636912303,15.46659136,0.910871208,2
6375.70692,14655.37573,13.18420205,7.636912303,15.46659136,0.910871208,24,2,25.8613194,4
74,1,1,1,0,0,12,16375.70692,14655.37573,13.18420205,7.636912303,15.46659136,0.910871208,24
001,22.1288926,16.17549357,3.372279327,22,4,26.76260836,30.98044964,146.5262651,188.50185
001,22.1288926,16.17549357,3.372279327,22,4,26.76260836,30.98044964,146.5262651,188.50185
001,22.1288926,16.17549357,3.372279327,22,4,26.76260836,30.98044964,146.5262651,188.50185
32.62164,15040.94259,13.96310001,22.1288926,16.17549357,3.372279327,22,4,26.76260836,30.9
32.62164,15040.94259,13.96310001,22.1288926,16.17549357,3.372279327,22,4,26.76260836,30.9
01,22.1288926,16.17549357,3.372279327,22,4,26.76260836,30.98044964,146.5262651,188.501850
001,22.1288926,16.17549357,3.372279327,22,4,26.76260836,30.98044964,146.5262651,188.50185
001,22.1288926,16.17549357,3.372279327,22,4,26.76260836,30.98044964,146.5262651,188.50185
832.62164,15040.94259,13.96310001,22.1288926,16.17549357,3.372279327,22,4,26.76260836,30.
001,22.1288926,16.17549357,3.372279327,22,4,26.76260836,30.98044964,146.5262651,188.50185
3,83,0,1,0,1,0,9,16832.62164,15040.94259,13.96310001,22.1288926,16.17549357,3.372279327,22,

32.62164,15040.94259,13.96310001,22.1288926,16.17549357,3.372279327,22,4,26.76260836,30.9
001,22.1288926,16.17549357,3.372279327,22,4,26.76260836,30.98044964,146.5262651,188.50185
832.62164,15040.94259,13.96310001,22.1288926,16.17549357,3.372279327,22,4,26.76260836,30.
3.96310001,22.1288926,16.17549357,3.372279327,22,4,26.76260836,30.98044964,146.5262651,18
001,22.1288926,16.17549357,3.372279327,22,4,26.76260836,30.98044964,146.5262651,188.50185
6832.62164,15040.94259,13.96310001,22.1288926,16.17549357,3.372279327,22,4,26.76260836,3
0001,22.1288926,16.17549357,3.372279327,22,4,26.76260836,30.98044964,146.5262651,188.5018
32.62164,15040.94259,13.96310001,22.1288926,16.17549357,3.372279327,22,4,26.76260836,30.9
0001,22.1288926,16.17549357,3.372279327,22,4,26.76260836,30.98044964,146.5262651,188.5018
83,0,1,0,1,0,9,16832.62164,15040.94259,13.96310001,22.1288926,16.17549357,3.372279327,22,4,
32.62164,15040.94259,13.96310001,22.1288926,16.17549357,3.372279327,22,4,26.76260836,30.9
3,83,0,1,0,1,0,9,16832.62164,15040.94259,13.96310001,22.1288926,16.17549357,3.372279327,22,
832.62164,15040.94259,13.96310001,22.1288926,16.17549357,3.372279327,22,4,26.76260836,30.
32.62164,15040.94259,13.96310001,22.1288926,16.17549357,3.372279327,22,4,26.76260836,30.9
32.62164,15040.94259,13.96310001,22.1288926,16.17549357,3.372279327,22,4,26.76260836,30.9
6832.62164,15040.94259,13.96310001,22.1288926,16.17549357,3.372279327,22,4,26.76260836,3
310001,22.1288926,16.17549357,3.372279327,22,4,26.76260836,30.98044964,146.5262651,188.50
3,83,0,1,0,1,0,9,16832.62164,15040.94259,13.96310001,22.1288926,16.17549357,3.372279327,22,
6832.62164,15040.94259,13.96310001,22.1288926,16.17549357,3.372279327,22,4,26.76260836,3
10001,22.1288926,16.17549357,3.372279327,22,4,26.76260836,30.98044964,146.5262651,188.501
6832.62164,15040.94259,13.96310001,22.1288926,16.17549357,3.372279327,22,4,26.76260836,3
310001,22.1288926,16.17549357,3.372279327,22,4,26.76260836,30.98044964,146.5262651,188.50
32.62164,15040.94259,13.96310001,22.1288926,16.17549357,3.372279327,22,4,26.76260836,30.9
6832.62164,15040.94259,13.96310001,22.1288926,16.17549357,3.372279327,22,4,26.76260836,3
32.62164,15040.94259,13.96310001,22.1288926,16.17549357,3.372279327,22,4,26.76260836,30.9
001,22.1288926,16.17549357,3.372279327,22,4,26.76260836,30.98044964,146.5262651,188.50185
001,22.1288926,16.17549357,3.372279327,22,4,26.76260836,30.98044964,146.5262651,188.50185
32.62164,15040.94259,13.96310001,22.1288926,16.17549357,3.372279327,22,4,26.76260836,30.9
425,21.36500481,15.53201678,6.842211452,22,5,28.57747154,28.48667308,108.8062333,183.8663
6858.17065,18090.34459,12.40154425,21.36500481,15.53201678,6.842211452,22,5,28.57747154,
858.17065,18090.34459,12.40154425,21.36500481,15.53201678,6.842211452,22,5,28.57747154,2
4425,21.36500481,15.53201678,6.842211452,22,5,28.57747154,28.48667308,108.8062333,183.866
4425,21.36500481,15.53201678,6.842211452,22,5,28.57747154,28.48667308,108.8062333,183.866
6858.17065,18090.34459,12.40154425,21.36500481,15.53201678,6.842211452,22,5,28.57747154,
16858.17065,18090.34459,12.40154425,21.36500481,15.53201678,6.842211452,22,5,28.57747154
16858.17065,18090.34459,12.40154425,21.36500481,15.53201678,6.842211452,22,5,28.57747154
858.17065,18090.34459,12.40154425,21.36500481,15.53201678,6.842211452,22,5,28.57747154,2
67,0,1,1,1,0,11,16858.17065,18090.34459,12.40154425,21.36500481,15.53201678,6.842211452,22
858.17065,18090.34459,12.40154425,21.36500481,15.53201678,6.842211452,22,5,28.57747154,2
858.17065,18090.34459,12.40154425,21.36500481,15.53201678,6.842211452,22,5,28.57747154,2
858.17065,18090.34459,12.40154425,21.36500481,15.53201678,6.842211452,22,5,28.57747154,2
67,0,1,1,1,0,11,16858.17065,18090.34459,12.40154425,21.36500481,15.53201678,6.842211452,22
67,0,1,1,1,0,11,16858.17065,18090.34459,12.40154425,21.36500481,15.53201678,6.842211452,22
67,0,1,1,1,0,11,16858.17065,18090.34459,12.40154425,21.36500481,15.53201678,6.842211452,22
6858.17065,18090.34459,12.40154425,21.36500481,15.53201678,6.842211452,22,5,28.57747154,
3,67,0,1,1,1,0,11,16858.17065,18090.34459,12.40154425,21.36500481,15.53201678,6.842211452,2
67,0,1,1,1,0,11,16858.17065,18090.34459,12.40154425,21.36500481,15.53201678,6.842211452,22
3,67,0,1,1,1,0,11,16858.17065,18090.34459,12.40154425,21.36500481,15.53201678,6.842211452,2
858.17065,18090.34459,12.40154425,21.36500481,15.53201678,6.842211452,22,5,28.57747154,2
4425,21.36500481,15.53201678,6.842211452,22,5,28.57747154,28.48667308,108.8062333,183.866

9382324,3,2,203,67,0,1,1,1,0,11,16858.17065,18090.34459,12.40154425,21.36500481,15.53201678
858.17065,18090.34459,12.40154425,21.36500481,15.53201678,6.842211452,22,5,28.57747154,2
858.17065,18090.34459,12.40154425,21.36500481,15.53201678,6.842211452,22,5,28.57747154,2
4425,21.36500481,15.53201678,6.842211452,22,5,28.57747154,28.48667308,108.8062333,183.866
858.17065,18090.34459,12.40154425,21.36500481,15.53201678,6.842211452,22,5,28.57747154,2
6858.17065,18090.34459,12.40154425,21.36500481,15.53201678,6.842211452,22,5,28.57747154,
858.17065,18090.34459,12.40154425,21.36500481,15.53201678,6.842211452,22,5,28.57747154,2
858.17065,18090.34459,12.40154425,21.36500481,15.53201678,6.842211452,22,5,28.57747154,2
16858.17065,18090.34459,12.40154425,21.36500481,15.53201678,6.842211452,22,5,28.57747154
5,1,0,1,0,0,11,16793.12444,14125.37203,11.70147614,19.921472,14.360437,0.689113848,21,5,24.
7614,19.921472,14.360437,0.689113848,21,5,24.42047193,47.81153279,110.9636583,220.2930072
793.12444,14125.37203,11.70147614,19.921472,14.360437,0.689113848,21,5,24.42047193,47.811
793.12444,14125.37203,11.70147614,19.921472,14.360437,0.689113848,21,5,24.42047193,47.811
3,75,1,0,1,0,0,11,16793.12444,14125.37203,11.70147614,19.921472,14.360437,0.689113848,21,5,2
3,75,1,0,1,0,0,11,16793.12444,14125.37203,11.70147614,19.921472,14.360437,0.689113848,21,5,2
16793.12444,14125.37203,11.70147614,19.921472,14.360437,0.689113848,21,5,24.42047193,47.8
793.12444,14125.37203,11.70147614,19.921472,14.360437,0.689113848,21,5,24.42047193,47.811
793.12444,14125.37203,11.70147614,19.921472,14.360437,0.689113848,21,5,24.42047193,47.811
793.12444,14125.37203,11.70147614,19.921472,14.360437,0.689113848,21,5,24.42047193,47.811
93.12444,14125.37203,11.70147614,19.921472,14.360437,0.689113848,21,5,24.42047193,47.8115
793.12444,14125.37203,11.70147614,19.921472,14.360437,0.689113848,21,5,24.42047193,47.811
16793.12444,14125.37203,11.70147614,19.921472,14.360437,0.689113848,21,5,24.42047193,47.8
793.12444,14125.37203,11.70147614,19.921472,14.360437,0.689113848,21,5,24.42047193,47.811
793.12444,14125.37203,11.70147614,19.921472,14.360437,0.689113848,21,5,24.42047193,47.811
20617676,3,2,203,75,1,0,1,0,0,11,16793.12444,14125.37203,11.70147614,19.921472,14.360437,0.6
793.12444,14125.37203,11.70147614,19.921472,14.360437,0.689113848,21,5,24.42047193,47.811
793.12444,14125.37203,11.70147614,19.921472,14.360437,0.689113848,21,5,24.42047193,47.811
16793.12444,14125.37203,11.70147614,19.921472,14.360437,0.689113848,21,5,24.42047193,47.8
16793.12444,14125.37203,11.70147614,19.921472,14.360437,0.689113848,21,5,24.42047193,47.8
793.12444,14125.37203,11.70147614,19.921472,14.360437,0.689113848,21,5,24.42047193,47.811
6793.12444,14125.37203,11.70147614,19.921472,14.360437,0.689113848,21,5,24.42047193,47.81
3,75,1,0,1,0,0,11,16793.12444,14125.37203,11.70147614,19.921472,14.360437,0.689113848,21,5,2
93.12444,14125.37203,11.70147614,19.921472,14.360437,0.689113848,21,5,24.42047193,47.8115
7614,19.921472,14.360437,0.689113848,21,5,24.42047193,47.81153279,110.9636583,220.2930072
93.12444,14125.37203,11.70147614,19.921472,14.360437,0.689113848,21,5,24.42047193,47.8115
793.12444,14125.37203,11.70147614,19.921472,14.360437,0.689113848,21,5,24.42047193,47.811
7614,19.921472,14.360437,0.689113848,21,5,24.42047193,47.81153279,110.9636583,220.2930072
793.12444,14125.37203,11.70147614,19.921472,14.360437,0.689113848,21,5,24.42047193,47.811
16793.12444,14125.37203,11.70147614,19.921472,14.360437,0.689113848,21,5,24.42047193,47.8
3,75,1,0,1,0,0,11,16793.12444,14125.37203,11.70147614,19.921472,14.360437,0.689113848,21,5,2
6793.12444,14125.37203,11.70147614,19.921472,14.360437,0.689113848,21,5,24.42047193,47.81
3,75,1,0,1,0,0,11,16793.12444,14125.37203,11.70147614,19.921472,14.360437,0.689113848,21,5,2
793.12444,14125.37203,11.70147614,19.921472,14.360437,0.689113848,21,5,24.42047193,47.811
16793.12444,14125.37203,11.70147614,19.921472,14.360437,0.689113848,21,5,24.42047193,47.8
3,75,1,0,1,0,0,11,16793.12444,14125.37203,11.70147614,19.921472,14.360437,0.689113848,21,5,2
7614,19.921472,14.360437,0.689113848,21,5,24.42047193,47.81153279,110.9636583,220.2930072
16793.12444,14125.37203,11.70147614,19.921472,14.360437,0.689113848,21,5,24.42047193,47.8
7614,19.921472,14.360437,0.689113848,21,5,24.42047193,47.81153279,110.9636583,220.2930072
6793.12444,14125.37203,11.70147614,19.921472,14.360437,0.689113848,21,5,24.42047193,47.81
20617676,3,2,203,75,1,0,1,0,0,11,16793.12444,14125.37203,11.70147614,19.921472,14.360437,0.6

75,1,0,1,0,0,11,16793.12444,14125.37203,11.70147614,19.921472,14.360437,0.689113848,21,5,24
793.12444,14125.37203,11.70147614,19.921472,14.360437,0.689113848,21,5,24.42047193,47.811
16793.12444,14125.37203,11.70147614,19.921472,14.360437,0.689113848,21,5,24.42047193,47.8
3,75,1,0,1,0,0,11,16793.12444,14125.37203,11.70147614,19.921472,14.360437,0.689113848,21,5,2
3,75,1,0,1,0,0,11,16793.12444,14125.37203,11.70147614,19.921472,14.360437,0.689113848,21,5,2
793.12444,14125.37203,11.70147614,19.921472,14.360437,0.689113848,21,5,24.42047193,47.811
16793.12444,14125.37203,11.70147614,19.921472,14.360437,0.689113848,21,5,24.42047193,47.8
7614,19.921472,14.360437,0.689113848,21,5,24.42047193,47.81153279,110.9636583,220.2930072
7614,19.921472,14.360437,0.689113848,21,5,24.42047193,47.81153279,110.9636583,220.2930072
161,21.13231005,15.75264075,3.372279327,20,4,26.83495225,54.94400613,145.7617909,183.9312
9161,21.13231005,15.75264075,3.372279327,20,4,26.83495225,54.94400613,145.7617909,183.931
042.39645,14407.10233,11.74029161,21.13231005,15.75264075,3.372279327,20,4,26.83495225,54
65,0,1,1,1,0,12,17042.39645,14407.10233,11.74029161,21.13231005,15.75264075,3.372279327,20
9161,21.13231005,15.75264075,3.372279327,20,4,26.83495225,54.94400613,145.7617909,183.931
042.39645,14407.10233,11.74029161,21.13231005,15.75264075,3.372279327,20,4,26.83495225,54
9161,21.13231005,15.75264075,3.372279327,20,4,26.83495225,54.94400613,145.7617909,183.931
042.39645,14407.10233,11.74029161,21.13231005,15.75264075,3.372279327,20,4,26.83495225,54
042.39645,14407.10233,11.74029161,21.13231005,15.75264075,3.372279327,20,4,26.83495225,54
042.39645,14407.10233,11.74029161,21.13231005,15.75264075,3.372279327,20,4,26.83495225,54
042.39645,14407.10233,11.74029161,21.13231005,15.75264075,3.372279327,20,4,26.83495225,54
042.39645,14407.10233,11.74029161,21.13231005,15.75264075,3.372279327,20,4,26.83495225,54
042.39645,14407.10233,11.74029161,21.13231005,15.75264075,3.372279327,20,4,26.83495225,54
7042.39645,14407.10233,11.74029161,21.13231005,15.75264075,3.372279327,20,4,26.83495225,5
042.39645,14407.10233,11.74029161,21.13231005,15.75264075,3.372279327,20,4,26.83495225,54
9382324,3,2,203,65,0,1,1,1,0,12,17042.39645,14407.10233,11.74029161,21.13231005,15.75264075
17042.39645,14407.10233,11.74029161,21.13231005,15.75264075,3.372279327,20,4,26.83495225
17042.39645,14407.10233,11.74029161,21.13231005,15.75264075,3.372279327,20,4,26.83495225
3,65,0,1,1,1,0,12,17042.39645,14407.10233,11.74029161,21.13231005,15.75264075,3.372279327,2
042.39645,14407.10233,11.74029161,21.13231005,15.75264075,3.372279327,20,4,26.83495225,54
9161,21.13231005,15.75264075,3.372279327,20,4,26.83495225,54.94400613,145.7617909,183.931
3,65,0,1,1,1,0,12,17042.39645,14407.10233,11.74029161,21.13231005,15.75264075,3.372279327,2
3,65,0,1,1,1,0,12,17042.39645,14407.10233,11.74029161,21.13231005,15.75264075,3.372279327,2
9161,21.13231005,15.75264075,3.372279327,20,4,26.83495225,54.94400613,145.7617909,183.931
042.39645,14407.10233,11.74029161,21.13231005,15.75264075,3.372279327,20,4,26.83495225,54
042.39645,14407.10233,11.74029161,21.13231005,15.75264075,3.372279327,20,4,26.83495225,54
9382324,3,2,203,65,0,1,1,1,0,12,17042.39645,14407.10233,11.74029161,21.13231005,15.75264075
042.39645,14407.10233,11.74029161,21.13231005,15.75264075,3.372279327,20,4,26.83495225,54
9161,21.13231005,15.75264075,3.372279327,20,4,26.83495225,54.94400613,145.7617909,183.931
3,65,0,1,1,1,0,12,17042.39645,14407.10233,11.74029161,21.13231005,15.75264075,3.372279327,2
17042.39645,14407.10233,11.74029161,21.13231005,15.75264075,3.372279327,20,4,26.83495225
54,31.77898652,18.89179516,1.442051805,25,9,29.05173935,28.24798801,211.9999443,227.65999
792.78982,13837.30984,14.7899764,31.77898652,18.89179516,1.442051805,25,9,29.05173935,28.
6792.78982,13837.30984,14.7899764,31.77898652,18.89179516,1.442051805,25,9,29.05173935,28
764,31.77898652,18.89179516,1.442051805,25,9,29.05173935,28.24798801,211.9999443,227.6599
899764,31.77898652,18.89179516,1.442051805,25,9,29.05173935,28.24798801,211.9999443,227.6
77,1,1,0,1,0,11,16792.78982,13837.30984,14.7899764,31.77898652,18.89179516,1.442051805,25,9
77,1,1,0,1,0,11,16792.78982,13837.30984,14.7899764,31.77898652,18.89179516,1.442051805,25,9
6792.78982,13837.30984,14.7899764,31.77898652,18.89179516,1.442051805,25,9,29.05173935,28
14.7899764,31.77898652,18.89179516,1.442051805,25,9,29.05173935,28.24798801,211.9999443,2
792.78982,13837.30984,14.7899764,31.77898652,18.89179516,1.442051805,25,9,29.05173935,28.

14.7899764,31.77898652,18.89179516,1.442051805,25,9,29.05173935,28.24798801,211.9999443,2
16792.78982,13837.30984,14.7899764,31.77898652,18.89179516,1.442051805,25,9,29.05173935,
792.78982,13837.30984,14.7899764,31.77898652,18.89179516,1.442051805,25,9,29.05173935,28.
3,77,1,1,0,1,0,11,16792.78982,13837.30984,14.7899764,31.77898652,18.89179516,1.442051805,25
4,31.77898652,18.89179516,1.442051805,25,9,29.05173935,28.24798801,211.9999443,227.659993
6792.78982,13837.30984,14.7899764,31.77898652,18.89179516,1.442051805,25,9,29.05173935,28
6792.78982,13837.30984,14.7899764,31.77898652,18.89179516,1.442051805,25,9,29.05173935,28
6792.78982,13837.30984,14.7899764,31.77898652,18.89179516,1.442051805,25,9,29.05173935,28
792.78982,13837.30984,14.7899764,31.77898652,18.89179516,1.442051805,25,9,29.05173935,28.
16792.78982,13837.30984,14.7899764,31.77898652,18.89179516,1.442051805,25,9,29.05173935,
,16792.78982,13837.30984,14.7899764,31.77898652,18.89179516,1.442051805,25,9,29.05173935,
6792.78982,13837.30984,14.7899764,31.77898652,18.89179516,1.442051805,25,9,29.05173935,28
764,31.77898652,18.89179516,1.442051805,25,9,29.05173935,28.24798801,211.9999443,227.6599
6792.78982,13837.30984,14.7899764,31.77898652,18.89179516,1.442051805,25,9,29.05173935,28
6792.78982,13837.30984,14.7899764,31.77898652,18.89179516,1.442051805,25,9,29.05173935,28
764,31.77898652,18.89179516,1.442051805,25,9,29.05173935,28.24798801,211.9999443,227.6599
764,31.77898652,18.89179516,1.442051805,25,9,29.05173935,28.24798801,211.9999443,227.6599
77,1,1,0,1,0,11,16792.78982,13837.30984,14.7899764,31.77898652,18.89179516,1.442051805,25,
784,14.91831674,15.65748524,0.976042153,24,4,25.86575568,37.29579184,46.11938681,211.4028
77,1,1,1,0,0,7,16726.97049,13921.3779,12.93287784,14.91831674,15.65748524,0.976042153,24,4,
34,14.91831674,15.65748524,0.976042153,24,4,25.86575568,37.29579184,46.11938681,211.40281
726.97049,13921.3779,12.93287784,14.91831674,15.65748524,0.976042153,24,4,25.86575568,37.
726.97049,13921.3779,12.93287784,14.91831674,15.65748524,0.976042153,24,4,25.86575568,37.
7,1,1,1,0,0,7,16726.97049,13921.3779,12.93287784,14.91831674,15.65748524,0.976042153,24,4,2
726.97049,13921.3779,12.93287784,14.91831674,15.65748524,0.976042153,24,4,25.86575568,37.
726.97049,13921.3779,12.93287784,14.91831674,15.65748524,0.976042153,24,4,25.86575568,37.
726.97049,13921.3779,12.93287784,14.91831674,15.65748524,0.976042153,24,4,25.86575568,37.
726.97049,13921.3779,12.93287784,14.91831674,15.65748524,0.976042153,24,4,25.86575568,37.
50308838,3,2,203,77,1,1,1,0,0,7,16726.97049,13921.3779,12.93287784,14.91831674,15.65748524,0
726.97049,13921.3779,12.93287784,14.91831674,15.65748524,0.976042153,24,4,25.86575568,37.
77,1,1,1,0,0,7,16726.97049,13921.3779,12.93287784,14.91831674,15.65748524,0.976042153,24,4,
726.97049,13921.3779,12.93287784,14.91831674,15.65748524,0.976042153,24,4,25.86575568,37.
26.97049,13921.3779,12.93287784,14.91831674,15.65748524,0.976042153,24,4,25.86575568,37.2
0308838,3,2,203,77,1,1,1,0,0,7,16726.97049,13921.3779,12.93287784,14.91831674,15.65748524,0.
26.97049,13921.3779,12.93287784,14.91831674,15.65748524,0.976042153,24,4,25.86575568,37.2
726.97049,13921.3779,12.93287784,14.91831674,15.65748524,0.976042153,24,4,25.86575568,37.
726.97049,13921.3779,12.93287784,14.91831674,15.65748524,0.976042153,24,4,25.86575568,37.
26.97049,13921.3779,12.93287784,14.91831674,15.65748524,0.976042153,24,4,25.86575568,37.2
77,1,1,1,0,0,7,16726.97049,13921.3779,12.93287784,14.91831674,15.65748524,0.976042153,24,4,
77,1,1,1,0,0,7,16726.97049,13921.3779,12.93287784,14.91831674,15.65748524,0.976042153,24,4,
26.97049,13921.3779,12.93287784,14.91831674,15.65748524,0.976042153,24,4,25.86575568,37.2
3,77,1,1,1,0,0,7,16726.97049,13921.3779,12.93287784,14.91831674,15.65748524,0.976042153,24,4
20617676,3,2,203,77,1,1,1,0,0,7,16726.97049,13921.3779,12.93287784,14.91831674,15.65748524,0
6726.97049,13921.3779,12.93287784,14.91831674,15.65748524,0.976042153,24,4,25.86575568,37
77,1,1,1,0,0,7,16726.97049,13921.3779,12.93287784,14.91831674,15.65748524,0.976042153,24,4,
77,1,1,1,0,0,7,16726.97049,13921.3779,12.93287784,14.91831674,15.65748524,0.976042153,24,4,
26.97049,13921.3779,12.93287784,14.91831674,15.65748524,0.976042153,24,4,25.86575568,37.2
3,77,1,1,1,0,0,7,16726.97049,13921.3779,12.93287784,14.91831674,15.65748524,0.976042153,24,4
550772095,3,2,203,77,1,1,1,0,0,7,16726.97049,13921.3779,12.93287784,14.91831674,15.65748524
726.97049,13921.3779,12.93287784,14.91831674,15.65748524,0.976042153,24,4,25.86575568,37.

726.97049,13921.3779,12.93287784,14.91831674,15.65748524,0.976042153,24,4,25.86575568,37.
26.97049,13921.3779,12.93287784,14.91831674,15.65748524,0.976042153,24,4,25.86575568,37.2
79382324,3,2,203,77,1,1,1,0,0,7,16726.97049,13921.3779,12.93287784,14.91831674,15.65748524,0
93.12444,14125.37203,11.70147614,19.921472,14.360437,0.689113848,21,5,24.42047193,47.8115
93.12444,14125.37203,11.70147614,19.921472,14.360437,0.689113848,21,5,24.42047193,47.8115
93.12444,14125.37203,11.70147614,19.921472,14.360437,0.689113848,21,5,24.42047193,47.8115
514,19.921472,14.360437,0.689113848,21,5,24.42047193,47.81153279,110.9636583,220.2930072,
514,19.921472,14.360437,0.689113848,21,5,24.42047193,47.81153279,110.9636583,220.2930072,
514,19.921472,14.360437,0.689113848,21,5,24.42047193,47.81153279,110.9636583,220.2930072,
16793.12444,14125.37203,11.70147614,19.921472,14.360437,0.689113848,21,5,24.42047193,47.81
514,19.921472,14.360437,0.689113848,21,5,24.42047193,47.81153279,110.9636583,220.2930072,
147614,19.921472,14.360437,0.689113848,21,5,24.42047193,47.81153279,110.9636583,220.29300
70147614,19.921472,14.360437,0.689113848,21,5,24.42047193,47.81153279,110.9636583,220.293
16793.12444,14125.37203,11.70147614,19.921472,14.360437,0.689113848,21,5,24.42047193,47.81
3,78,0,1,0,1,0,7,16793.12444,14125.37203,11.70147614,19.921472,14.360437,0.689113848,21,5,24
793.12444,14125.37203,11.70147614,19.921472,14.360437,0.689113848,21,5,24.42047193,47.811
793.12444,14125.37203,11.70147614,19.921472,14.360437,0.689113848,21,5,24.42047193,47.811
93.12444,14125.37203,11.70147614,19.921472,14.360437,0.689113848,21,5,24.42047193,47.8115
147614,19.921472,14.360437,0.689113848,21,5,24.42047193,47.81153279,110.9636583,220.29300
3,78,0,1,0,1,0,7,16793.12444,14125.37203,11.70147614,19.921472,14.360437,0.689113848,21,5,24
93.12444,14125.37203,11.70147614,19.921472,14.360437,0.689113848,21,5,24.42047193,47.8115
93.12444,14125.37203,11.70147614,19.921472,14.360437,0.689113848,21,5,24.42047193,47.8115
93.12444,14125.37203,11.70147614,19.921472,14.360437,0.689113848,21,5,24.42047193,47.8115
93.12444,14125.37203,11.70147614,19.921472,14.360437,0.689113848,21,5,24.42047193,47.8115
93.12444,14125.37203,11.70147614,19.921472,14.360437,0.689113848,21,5,24.42047193,47.8115
93.12444,14125.37203,11.70147614,19.921472,14.360437,0.689113848,21,5,24.42047193,47.8115
514,19.921472,14.360437,0.689113848,21,5,24.42047193,47.81153279,110.9636583,220.2930072,
514,19.921472,14.360437,0.689113848,21,5,24.42047193,47.81153279,110.9636583,220.2930072,
147614,19.921472,14.360437,0.689113848,21,5,24.42047193,47.81153279,110.9636583,220.29300
93.12444,14125.37203,11.70147614,19.921472,14.360437,0.689113848,21,5,24.42047193,47.8115
93.12444,14125.37203,11.70147614,19.921472,14.360437,0.689113848,21,5,24.42047193,47.8115
514,19.921472,14.360437,0.689113848,21,5,24.42047193,47.81153279,110.9636583,220.2930072,
93.12444,14125.37203,11.70147614,19.921472,14.360437,0.689113848,21,5,24.42047193,47.8115
514,19.921472,14.360437,0.689113848,21,5,24.42047193,47.81153279,110.9636583,220.2930072,
514,19.921472,14.360437,0.689113848,21,5,24.42047193,47.81153279,110.9636583,220.2930072,
514,19.921472,14.360437,0.689113848,21,5,24.42047193,47.81153279,110.9636583,220.2930072,
93.12444,14125.37203,11.70147614,19.921472,14.360437,0.689113848,21,5,24.42047193,47.8115
992,31.94525969,18.89179516,1.442051805,25,9,28.91257664,28.39578639,217.5334488,227.6208
790.51585,13868.94857,14.71912992,31.94525969,18.89179516,1.442051805,25,9,28.91257664,28
69,1,1,0,1,0,8,16790.51585,13868.94857,14.71912992,31.94525969,18.89179516,1.442051805,25,9
790.51585,13868.94857,14.71912992,31.94525969,18.89179516,1.442051805,25,9,28.91257664,28
69,1,1,0,1,0,8,16790.51585,13868.94857,14.71912992,31.94525969,18.89179516,1.442051805,25,9
992,31.94525969,18.89179516,1.442051805,25,9,28.91257664,28.39578639,217.5334488,227.6208
790.51585,13868.94857,14.71912992,31.94525969,18.89179516,1.442051805,25,9,28.91257664,28
992,31.94525969,18.89179516,1.442051805,25,9,28.91257664,28.39578639,217.5334488,227.6208
790.51585,13868.94857,14.71912992,31.94525969,18.89179516,1.442051805,25,9,28.91257664,28
790.51585,13868.94857,14.71912992,31.94525969,18.89179516,1.442051805,25,9,28.91257664,28
9691162,3,2,203,69,1,1,0,1,0,8,16790.51585,13868.94857,14.71912992,31.94525969,18.89179516,1
790.51585,13868.94857,14.71912992,31.94525969,18.89179516,1.442051805,25,9,28.91257664,28
790.51585,13868.94857,14.71912992,31.94525969,18.89179516,1.442051805,25,9,28.91257664,28

790.51585,13868.94857,14.71912992,31.94525969,18.89179516,1.442051805,25,9,28.91257664,2
790.51585,13868.94857,14.71912992,31.94525969,18.89179516,1.442051805,25,9,28.91257664,2
9,1,1,0,1,0,8,16790.51585,13868.94857,14.71912992,31.94525969,18.89179516,1.442051805,25,9,
790.51585,13868.94857,14.71912992,31.94525969,18.89179516,1.442051805,25,9,28.91257664,2
790.51585,13868.94857,14.71912992,31.94525969,18.89179516,1.442051805,25,9,28.91257664,2
790.51585,13868.94857,14.71912992,31.94525969,18.89179516,1.442051805,25,9,28.91257664,2
790.51585,13868.94857,14.71912992,31.94525969,18.89179516,1.442051805,25,9,28.91257664,2
9382324,3,2,203,69,1,1,0,1,0,8,16790.51585,13868.94857,14.71912992,31.94525969,18.89179516,
790.51585,13868.94857,14.71912992,31.94525969,18.89179516,1.442051805,25,9,28.91257664,2
790.51585,13868.94857,14.71912992,31.94525969,18.89179516,1.442051805,25,9,28.91257664,2
790.51585,13868.94857,14.71912992,31.94525969,18.89179516,1.442051805,25,9,28.91257664,2
69,1,1,0,1,0,8,16790.51585,13868.94857,14.71912992,31.94525969,18.89179516,1.442051805,25,
9,1,1,0,1,0,8,16790.51585,13868.94857,14.71912992,31.94525969,18.89179516,1.442051805,25,9,
69,1,1,0,1,0,8,16790.51585,13868.94857,14.71912992,31.94525969,18.89179516,1.442051805,25,
790.51585,13868.94857,14.71912992,31.94525969,18.89179516,1.442051805,25,9,28.91257664,2
790.51585,13868.94857,14.71912992,31.94525969,18.89179516,1.442051805,25,9,28.91257664,2
69,1,1,0,1,0,8,16790.51585,13868.94857,14.71912992,31.94525969,18.89179516,1.442051805,25,
790.51585,13868.94857,14.71912992,31.94525969,18.89179516,1.442051805,25,9,28.91257664,2
790.51585,13868.94857,14.71912992,31.94525969,18.89179516,1.442051805,25,9,28.91257664,2
790.51585,13868.94857,14.71912992,31.94525969,18.89179516,1.442051805,25,9,28.91257664,2
790.51585,13868.94857,14.71912992,31.94525969,18.89179516,1.442051805,25,9,28.91257664,2
790.51585,13868.94857,14.71912992,31.94525969,18.89179516,1.442051805,25,9,28.91257664,2
2575653,33.84608018,11.03865097,0.546417561,22,9,26.92247249,33.84608018,239.2595725,243.
16933.18454,13588.13628,12.42575653,33.84608018,11.03865097,0.546417561,22,9,26.92247249,3
933.18454,13588.13628,12.42575653,33.84608018,11.03865097,0.546417561,22,9,26.92247249,33
77,1,1,0,1,0,11,16933.18454,13588.13628,12.42575653,33.84608018,11.03865097,0.546417561,22
16933.18454,13588.13628,12.42575653,33.84608018,11.03865097,0.546417561,22,9,26.92247249
6933.18454,13588.13628,12.42575653,33.84608018,11.03865097,0.546417561,22,9,26.92247249,3
6933.18454,13588.13628,12.42575653,33.84608018,11.03865097,0.546417561,22,9,26.92247249,3
6933.18454,13588.13628,12.42575653,33.84608018,11.03865097,0.546417561,22,9,26.92247249,3
553,33.84608018,11.03865097,0.546417561,22,9,26.92247249,33.84608018,239.2595725,243.3377
.42575653,33.84608018,11.03865097,0.546417561,22,9,26.92247249,33.84608018,239.2595725,24
12.42575653,33.84608018,11.03865097,0.546417561,22,9,26.92247249,33.84608018,239.2595725,
6933.18454,13588.13628,12.42575653,33.84608018,11.03865097,0.546417561,22,9,26.92247249,3
6933.18454,13588.13628,12.42575653,33.84608018,11.03865097,0.546417561,22,9,26.92247249,3
16933.18454,13588.13628,12.42575653,33.84608018,11.03865097,0.546417561,22,9,26.92247249
6933.18454,13588.13628,12.42575653,33.84608018,11.03865097,0.546417561,22,9,26.92247249,3
77,1,1,0,1,0,11,16933.18454,13588.13628,12.42575653,33.84608018,11.03865097,0.546417561,22
5653,33.84608018,11.03865097,0.546417561,22,9,26.92247249,33.84608018,239.2595725,243.337
6933.18454,13588.13628,12.42575653,33.84608018,11.03865097,0.546417561,22,9,26.92247249,3
3,77,1,1,0,1,0,11,16933.18454,13588.13628,12.42575653,33.84608018,11.03865097,0.546417561,2
2575653,33.84608018,11.03865097,0.546417561,22,9,26.92247249,33.84608018,239.2595725,243.
77,1,1,0,1,0,11,16933.18454,13588.13628,12.42575653,33.84608018,11.03865097,0.546417561,22
6933.18454,13588.13628,12.42575653,33.84608018,11.03865097,0.546417561,22,9,26.92247249,3
933.18454,13588.13628,12.42575653,33.84608018,11.03865097,0.546417561,22,9,26.92247249,33
553,33.84608018,11.03865097,0.546417561,22,9,26.92247249,33.84608018,239.2595725,243.3377
933.18454,13588.13628,12.42575653,33.84608018,11.03865097,0.546417561,22,9,26.92247249,33
77,1,1,0,1,0,11,16933.18454,13588.13628,12.42575653,33.84608018,11.03865097,0.546417561,22
6933.18454,13588.13628,12.42575653,33.84608018,11.03865097,0.546417561,22,9,26.92247249,3
933.18454,13588.13628,12.42575653,33.84608018,11.03865097,0.546417561,22,9,26.92247249,33

6933.18454,13588.13628,12.42575653,33.84608018,11.03865097,0.546417561,22,9,26.92247249,3
6933.18454,13588.13628,12.42575653,33.84608018,11.03865097,0.546417561,22,9,26.92247249,3
6933.18454,13588.13628,12.42575653,33.84608018,11.03865097,0.546417561,22,9,26.92247249,3
77,1,1,0,1,0,11,16933.18454,13588.13628,12.42575653,33.84608018,11.03865097,0.546417561,22
6933.18454,13588.13628,12.42575653,33.84608018,11.03865097,0.546417561,22,9,26.92247249,3
5653,33.84608018,11.03865097,0.546417561,22,9,26.92247249,33.84608018,239.2595725,243.337
317,7.772130509,16.05649585,0.910871208,25,2,24.97726597,34.97458729,92.3400079,204.91552
708.00427,13786.18024,13.25324317,7.772130509,16.05649585,0.910871208,25,2,24.97726597,34
708.00427,13786.18024,13.25324317,7.772130509,16.05649585,0.910871208,25,2,24.97726597,34
08.00427,13786.18024,13.25324317,7.772130509,16.05649585,0.910871208,25,2,24.97726597,34.
6708.00427,13786.18024,13.25324317,7.772130509,16.05649585,0.910871208,25,2,24.97726597,3
7,1,1,1,0,0,8,16708.00427,13786.18024,13.25324317,7.772130509,16.05649585,0.910871208,25,2,
708.00427,13786.18024,13.25324317,7.772130509,16.05649585,0.910871208,25,2,24.97726597,34
67,1,1,1,0,0,8,16708.00427,13786.18024,13.25324317,7.772130509,16.05649585,0.910871208,25,2
708.00427,13786.18024,13.25324317,7.772130509,16.05649585,0.910871208,25,2,24.97726597,34
08.00427,13786.18024,13.25324317,7.772130509,16.05649585,0.910871208,25,2,24.97726597,34.
6708.00427,13786.18024,13.25324317,7.772130509,16.05649585,0.910871208,25,2,24.97726597,3
6708.00427,13786.18024,13.25324317,7.772130509,16.05649585,0.910871208,25,2,24.97726597,3
6708.00427,13786.18024,13.25324317,7.772130509,16.05649585,0.910871208,25,2,24.97726597,3
708.00427,13786.18024,13.25324317,7.772130509,16.05649585,0.910871208,25,2,24.97726597,34
08.00427,13786.18024,13.25324317,7.772130509,16.05649585,0.910871208,25,2,24.97726597,34.
67,1,1,1,0,0,8,16708.00427,13786.18024,13.25324317,7.772130509,16.05649585,0.910871208,25,2
4317,7.772130509,16.05649585,0.910871208,25,2,24.97726597,34.97458729,92.3400079,204.9155
317,7.772130509,16.05649585,0.910871208,25,2,24.97726597,34.97458729,92.3400079,204.91552
317,7.772130509,16.05649585,0.910871208,25,2,24.97726597,34.97458729,92.3400079,204.91552
08.00427,13786.18024,13.25324317,7.772130509,16.05649585,0.910871208,25,2,24.97726597,34.
9382324,3,2,203,67,1,1,1,0,0,8,16708.00427,13786.18024,13.25324317,7.772130509,16.05649585,0
708.00427,13786.18024,13.25324317,7.772130509,16.05649585,0.910871208,25,2,24.97726597,34
08.00427,13786.18024,13.25324317,7.772130509,16.05649585,0.910871208,25,2,24.97726597,34.
317,7.772130509,16.05649585,0.910871208,25,2,24.97726597,34.97458729,92.3400079,204.91552
08.00427,13786.18024,13.25324317,7.772130509,16.05649585,0.910871208,25,2,24.97726597,34.
67,1,1,1,0,0,8,16708.00427,13786.18024,13.25324317,7.772130509,16.05649585,0.910871208,25,2
708.00427,13786.18024,13.25324317,7.772130509,16.05649585,0.910871208,25,2,24.97726597,34
708.00427,13786.18024,13.25324317,7.772130509,16.05649585,0.910871208,25,2,24.97726597,34
67,1,1,1,0,0,8,16708.00427,13786.18024,13.25324317,7.772130509,16.05649585,0.910871208,25,2
708.00427,13786.18024,13.25324317,7.772130509,16.05649585,0.910871208,25,2,24.97726597,34
67,1,1,1,0,0,8,16708.00427,13786.18024,13.25324317,7.772130509,16.05649585,0.910871208,25,2
67,1,1,1,0,0,8,16708.00427,13786.18024,13.25324317,7.772130509,16.05649585,0.910871208,25,2
708.00427,13786.18024,13.25324317,7.772130509,16.05649585,0.910871208,25,2,24.97726597,34
08.00427,13786.18024,13.25324317,7.772130509,16.05649585,0.910871208,25,2,24.97726597,34.
08.00427,13786.18024,13.25324317,7.772130509,16.05649585,0.910871208,25,2,24.97726597,34.
378,19.98209333,16.18099206,3.372279327,23,4,27.1723145,27.97493067,159.7347878,187.89366
69,0,1,0,0,0,8,17006.89949,15203.92973,14.45335878,19.98209333,16.18099206,3.372279327,23,4
006.89949,15203.92973,14.45335878,19.98209333,16.18099206,3.372279327,23,4,27.1723145,27.
006.89949,15203.92973,14.45335878,19.98209333,16.18099206,3.372279327,23,4,27.1723145,27.
378,19.98209333,16.18099206,3.372279327,23,4,27.1723145,27.97493067,159.7347878,187.89366
79382324,3,2,203,69,0,1,0,0,0,8,17006.89949,15203.92973,14.45335878,19.98209333,16.18099206
006.89949,15203.92973,14.45335878,19.98209333,16.18099206,3.372279327,23,4,27.1723145,27.
006.89949,15203.92973,14.45335878,19.98209333,16.18099206,3.372279327,23,4,27.1723145,27.
69,0,1,0,0,0,8,17006.89949,15203.92973,14.45335878,19.98209333,16.18099206,3.372279327,23,4

06.89949,15203.92973,14.45335878,19.98209333,16.18099206,3.372279327,23,4,27.1723145,27.9
7006.89949,15203.92973,14.45335878,19.98209333,16.18099206,3.372279327,23,4,27.1723145,2
006.89949,15203.92973,14.45335878,19.98209333,16.18099206,3.372279327,23,4,27.1723145,27.
006.89949,15203.92973,14.45335878,19.98209333,16.18099206,3.372279327,23,4,27.1723145,27.
006.89949,15203.92973,14.45335878,19.98209333,16.18099206,3.372279327,23,4,27.1723145,27.
378,19.98209333,16.18099206,3.372279327,23,4,27.1723145,27.97493067,159.7347878,187.89366
006.89949,15203.92973,14.45335878,19.98209333,16.18099206,3.372279327,23,4,27.1723145,27.
006.89949,15203.92973,14.45335878,19.98209333,16.18099206,3.372279327,23,4,27.1723145,27.
006.89949,15203.92973,14.45335878,19.98209333,16.18099206,3.372279327,23,4,27.1723145,27.
006.89949,15203.92973,14.45335878,19.98209333,16.18099206,3.372279327,23,4,27.1723145,27.
06.89949,15203.92973,14.45335878,19.98209333,16.18099206,3.372279327,23,4,27.1723145,27.9
006.89949,15203.92973,14.45335878,19.98209333,16.18099206,3.372279327,23,4,27.1723145,27.
006.89949,15203.92973,14.45335878,19.98209333,16.18099206,3.372279327,23,4,27.1723145,27.
006.89949,15203.92973,14.45335878,19.98209333,16.18099206,3.372279327,23,4,27.1723145,27.
006.89949,15203.92973,14.45335878,19.98209333,16.18099206,3.372279327,23,4,27.1723145,27.
378,19.98209333,16.18099206,3.372279327,23,4,27.1723145,27.97493067,159.7347878,187.89366
69,0,1,0,0,0,8,17006.89949,15203.92973,14.45335878,19.98209333,16.18099206,3.372279327,23,4
006.89949,15203.92973,14.45335878,19.98209333,16.18099206,3.372279327,23,4,27.1723145,27.
5878,19.98209333,16.18099206,3.372279327,23,4,27.1723145,27.97493067,159.7347878,187.8936
378,19.98209333,16.18099206,3.372279327,23,4,27.1723145,27.97493067,159.7347878,187.89366
3,69,0,1,0,0,0,8,17006.89949,15203.92973,14.45335878,19.98209333,16.18099206,3.372279327,23
9382324,3,2,203,69,0,1,0,0,0,8,17006.89949,15203.92973,14.45335878,19.98209333,16.18099206,3
378,19.98209333,16.18099206,3.372279327,23,4,27.1723145,27.97493067,159.7347878,187.89366
006.89949,15203.92973,14.45335878,19.98209333,16.18099206,3.372279327,23,4,27.1723145,27.
006.89949,15203.92973,14.45335878,19.98209333,16.18099206,3.372279327,23,4,27.1723145,27.
378,19.98209333,16.18099206,3.372279327,23,4,27.1723145,27.97493067,159.7347878,187.89366
338,9.160234721,15.44996245,6.859172809,22,2,25.07730704,59.54152569,170.2075381,195.8164
43338,9.160234721,15.44996245,6.859172809,22,2,25.07730704,59.54152569,170.2075381,195.81
70,0,1,1,0,0,12,16715.68645,16565.66933,12.80543338,9.160234721,15.44996245,6.859172809,22
70,0,1,1,0,0,12,16715.68645,16565.66933,12.80543338,9.160234721,15.44996245,6.859172809,22
715.68645,16565.66933,12.80543338,9.160234721,15.44996245,6.859172809,22,2,25.07730704,5
20617676,3,2,203,70,0,1,1,0,0,12,16715.68645,16565.66933,12.80543338,9.160234721,15.4499624
43338,9.160234721,15.44996245,6.859172809,22,2,25.07730704,59.54152569,170.2075381,195.81
70,0,1,1,0,0,12,16715.68645,16565.66933,12.80543338,9.160234721,15.44996245,6.859172809,22
6715.68645,16565.66933,12.80543338,9.160234721,15.44996245,6.859172809,22,2,25.07730704,
6715.68645,16565.66933,12.80543338,9.160234721,15.44996245,6.859172809,22,2,25.07730704,
70,0,1,1,0,0,12,16715.68645,16565.66933,12.80543338,9.160234721,15.44996245,6.859172809,22
6715.68645,16565.66933,12.80543338,9.160234721,15.44996245,6.859172809,22,2,25.07730704,
6715.68645,16565.66933,12.80543338,9.160234721,15.44996245,6.859172809,22,2,25.07730704,
715.68645,16565.66933,12.80543338,9.160234721,15.44996245,6.859172809,22,2,25.07730704,5
6715.68645,16565.66933,12.80543338,9.160234721,15.44996245,6.859172809,22,2,25.07730704,
0,0,1,1,0,0,12,16715.68645,16565.66933,12.80543338,9.160234721,15.44996245,6.859172809,22,
3338,9.160234721,15.44996245,6.859172809,22,2,25.07730704,59.54152569,170.2075381,195.816
6715.68645,16565.66933,12.80543338,9.160234721,15.44996245,6.859172809,22,2,25.07730704,
715.68645,16565.66933,12.80543338,9.160234721,15.44996245,6.859172809,22,2,25.07730704,5
6715.68645,16565.66933,12.80543338,9.160234721,15.44996245,6.859172809,22,2,25.07730704,
70,0,1,1,0,0,12,16715.68645,16565.66933,12.80543338,9.160234721,15.44996245,6.859172809,22
6715.68645,16565.66933,12.80543338,9.160234721,15.44996245,6.859172809,22,2,25.07730704,
715.68645,16565.66933,12.80543338,9.160234721,15.44996245,6.859172809,22,2,25.07730704,5
6715.68645,16565.66933,12.80543338,9.160234721,15.44996245,6.859172809,22,2,25.07730704,

3338,9.160234721,15.44996245,6.859172809,22,2,25.07730704,59.54152569,170.2075381,195.816
6715.68645,16565.66933,12.80543338,9.160234721,15.44996245,6.859172809,22,2,25.07730704,5
6715.68645,16565.66933,12.80543338,9.160234721,15.44996245,6.859172809,22,2,25.07730704,5
6715.68645,16565.66933,12.80543338,9.160234721,15.44996245,6.859172809,22,2,25.07730704,5
3338,9.160234721,15.44996245,6.859172809,22,2,25.07730704,59.54152569,170.2075381,195.816
6715.68645,16565.66933,12.80543338,9.160234721,15.44996245,6.859172809,22,2,25.07730704,5
6715.68645,16565.66933,12.80543338,9.160234721,15.44996245,6.859172809,22,2,25.07730704,5
6715.68645,16565.66933,12.80543338,9.160234721,15.44996245,6.859172809,22,2,25.07730704,5
3338,9.160234721,15.44996245,6.859172809,22,2,25.07730704,59.54152569,170.2075381,195.816
3338,9.160234721,15.44996245,6.859172809,22,2,25.07730704,59.54152569,170.2075381,195.816
6715.68645,16565.66933,12.80543338,9.160234721,15.44996245,6.859172809,22,2,25.07730704,5
9391,16.00796568,17.71686072,1.81462833,27,3,30.60360258,8.003982841,198.6636123,213.6477
6714.78116,12797.5841,17.32279391,16.00796568,17.71686072,1.81462833,27,3,30.60360258,8.0
391,16.00796568,17.71686072,1.81462833,27,3,30.60360258,8.003982841,198.6636123,213.64779
714.78116,12797.5841,17.32279391,16.00796568,17.71686072,1.81462833,27,3,30.60360258,8.00
7.32279391,16.00796568,17.71686072,1.81462833,27,3,30.60360258,8.003982841,198.6636123,21
91,16.00796568,17.71686072,1.81462833,27,3,30.60360258,8.003982841,198.6636123,213.647791
6714.78116,12797.5841,17.32279391,16.00796568,17.71686072,1.81462833,27,3,30.60360258,8.0
714.78116,12797.5841,17.32279391,16.00796568,17.71686072,1.81462833,27,3,30.60360258,8.00
16714.78116,12797.5841,17.32279391,16.00796568,17.71686072,1.81462833,27,3,30.60360258,8.
7.32279391,16.00796568,17.71686072,1.81462833,27,3,30.60360258,8.003982841,198.6636123,21
17.32279391,16.00796568,17.71686072,1.81462833,27,3,30.60360258,8.003982841,198.6636123,2
74,1,1,0,1,0,10,16714.78116,12797.5841,17.32279391,16.00796568,17.71686072,1.81462833,27,3,
32279391,16.00796568,17.71686072,1.81462833,27,3,30.60360258,8.003982841,198.6636123,213.
391,16.00796568,17.71686072,1.81462833,27,3,30.60360258,8.003982841,198.6636123,213.64779
7.32279391,16.00796568,17.71686072,1.81462833,27,3,30.60360258,8.003982841,198.6636123,21
391,16.00796568,17.71686072,1.81462833,27,3,30.60360258,8.003982841,198.6636123,213.64779
714.78116,12797.5841,17.32279391,16.00796568,17.71686072,1.81462833,27,3,30.60360258,8.00
1,17.32279391,16.00796568,17.71686072,1.81462833,27,3,30.60360258,8.003982841,198.663612
16714.78116,12797.5841,17.32279391,16.00796568,17.71686072,1.81462833,27,3,30.60360258,8.
714.78116,12797.5841,17.32279391,16.00796568,17.71686072,1.81462833,27,3,30.60360258,8.00
391,16.00796568,17.71686072,1.81462833,27,3,30.60360258,8.003982841,198.6636123,213.64779
6714.78116,12797.5841,17.32279391,16.00796568,17.71686072,1.81462833,27,3,30.60360258,8.0
6714.78116,12797.5841,17.32279391,16.00796568,17.71686072,1.81462833,27,3,30.60360258,8.0
74,1,1,0,1,0,10,16714.78116,12797.5841,17.32279391,16.00796568,17.71686072,1.81462833,27,3,
391,16.00796568,17.71686072,1.81462833,27,3,30.60360258,8.003982841,198.6636123,213.64779
46,22.50939518,15.53201678,3.372279327,22,4,24.22808661,31.51315326,126.3531783,192.64283
27.90661,14989.50623,14.1822946,22.50939518,15.53201678,3.372279327,22,4,24.22808661,31.5
6,22.50939518,15.53201678,3.372279327,22,4,24.22808661,31.51315326,126.3531783,192.642835
46,22.50939518,15.53201678,3.372279327,22,4,24.22808661,31.51315326,126.3531783,192.64283
3,67,1,1,0,1,0,8,16727.90661,14989.50623,14.1822946,22.50939518,15.53201678,3.372279327,22,
6727.90661,14989.50623,14.1822946,22.50939518,15.53201678,3.372279327,22,4,24.22808661,3
6727.90661,14989.50623,14.1822946,22.50939518,15.53201678,3.372279327,22,4,24.22808661,3
3,67,1,1,0,1,0,8,16727.90661,14989.50623,14.1822946,22.50939518,15.53201678,3.372279327,22,
4.1822946,22.50939518,15.53201678,3.372279327,22,4,24.22808661,31.51315326,126.3531783,19
,16727.90661,14989.50623,14.1822946,22.50939518,15.53201678,3.372279327,22,4,24.22808661,
6727.90661,14989.50623,14.1822946,22.50939518,15.53201678,3.372279327,22,4,24.22808661,3
6,22.50939518,15.53201678,3.372279327,22,4,24.22808661,31.51315326,126.3531783,192.642835
6727.90661,14989.50623,14.1822946,22.50939518,15.53201678,3.372279327,22,4,24.22808661,3
46,22.50939518,15.53201678,3.372279327,22,4,24.22808661,31.51315326,126.3531783,192.64283

27.90661,14989.50623,14.1822946,22.50939518,15.53201678,3.372279327,22,4,24.22808661,31.5
6727.90661,14989.50623,14.1822946,22.50939518,15.53201678,3.372279327,22,4,24.22808661,31
46,22.50939518,15.53201678,3.372279327,22,4,24.22808661,31.51315326,126.3531783,192.64283
46,22.50939518,15.53201678,3.372279327,22,4,24.22808661,31.51315326,126.3531783,192.64283
67,1,1,0,1,0,8,16727.90661,14989.50623,14.1822946,22.50939518,15.53201678,3.372279327,22,4,
6727.90661,14989.50623,14.1822946,22.50939518,15.53201678,3.372279327,22,4,24.22808661,31
67,1,1,0,1,0,8,16727.90661,14989.50623,14.1822946,22.50939518,15.53201678,3.372279327,22,4,
46,22.50939518,15.53201678,3.372279327,22,4,24.22808661,31.51315326,126.3531783,192.64283
46,22.50939518,15.53201678,3.372279327,22,4,24.22808661,31.51315326,126.3531783,192.64283
46,22.50939518,15.53201678,3.372279327,22,4,24.22808661,31.51315326,126.3531783,192.64283
67,1,1,0,1,0,8,16727.90661,14989.50623,14.1822946,22.50939518,15.53201678,3.372279327,22,4,
727.90661,14989.50623,14.1822946,22.50939518,15.53201678,3.372279327,22,4,24.22808661,31.
22946,22.50939518,15.53201678,3.372279327,22,4,24.22808661,31.51315326,126.3531783,192.64
27.90661,14989.50623,14.1822946,22.50939518,15.53201678,3.372279327,22,4,24.22808661,31.5
727.90661,14989.50623,14.1822946,22.50939518,15.53201678,3.372279327,22,4,24.22808661,31.
569845581,3,2,203,71,0,1,0,1,0,12,16932.5167,13307.38451,13.12429808,31.45455263,12.5963019
6932.5167,13307.38451,13.12429808,31.45455263,12.59630197,0.475248564,23,8,26.77356809,39
16932.5167,13307.38451,13.12429808,31.45455263,12.59630197,0.475248564,23,8,26.77356809,3
6932.5167,13307.38451,13.12429808,31.45455263,12.59630197,0.475248564,23,8,26.77356809,39
932.5167,13307.38451,13.12429808,31.45455263,12.59630197,0.475248564,23,8,26.77356809,39.
6932.5167,13307.38451,13.12429808,31.45455263,12.59630197,0.475248564,23,8,26.77356809,39
16932.5167,13307.38451,13.12429808,31.45455263,12.59630197,0.475248564,23,8,26.77356809,3
16932.5167,13307.38451,13.12429808,31.45455263,12.59630197,0.475248564,23,8,26.77356809,3
932.5167,13307.38451,13.12429808,31.45455263,12.59630197,0.475248564,23,8,26.77356809,39.
6932.5167,13307.38451,13.12429808,31.45455263,12.59630197,0.475248564,23,8,26.77356809,39
6932.5167,13307.38451,13.12429808,31.45455263,12.59630197,0.475248564,23,8,26.77356809,39
3,71,0,1,0,1,0,12,16932.5167,13307.38451,13.12429808,31.45455263,12.59630197,0.475248564,23
932.5167,13307.38451,13.12429808,31.45455263,12.59630197,0.475248564,23,8,26.77356809,39.
932.5167,13307.38451,13.12429808,31.45455263,12.59630197,0.475248564,23,8,26.77356809,39.
71,0,1,0,1,0,12,16932.5167,13307.38451,13.12429808,31.45455263,12.59630197,0.475248564,23,8
6932.5167,13307.38451,13.12429808,31.45455263,12.59630197,0.475248564,23,8,26.77356809,39
71,0,1,0,1,0,12,16932.5167,13307.38451,13.12429808,31.45455263,12.59630197,0.475248564,23,8
308,31.45455263,12.59630197,0.475248564,23,8,26.77356809,39.31819079,232.8458435,245.1618
6932.5167,13307.38451,13.12429808,31.45455263,12.59630197,0.475248564,23,8,26.77356809,39
308,31.45455263,12.59630197,0.475248564,23,8,26.77356809,39.31819079,232.8458435,245.1618
16932.5167,13307.38451,13.12429808,31.45455263,12.59630197,0.475248564,23,8,26.77356809,3
3,71,0,1,0,1,0,12,16932.5167,13307.38451,13.12429808,31.45455263,12.59630197,0.475248564,23
71,0,1,0,1,0,12,16932.5167,13307.38451,13.12429808,31.45455263,12.59630197,0.475248564,23,8
6932.5167,13307.38451,13.12429808,31.45455263,12.59630197,0.475248564,23,8,26.77356809,39
16932.5167,13307.38451,13.12429808,31.45455263,12.59630197,0.475248564,23,8,26.77356809,3
6932.5167,13307.38451,13.12429808,31.45455263,12.59630197,0.475248564,23,8,26.77356809,39
6932.5167,13307.38451,13.12429808,31.45455263,12.59630197,0.475248564,23,8,26.77356809,39
6932.5167,13307.38451,13.12429808,31.45455263,12.59630197,0.475248564,23,8,26.77356809,39
932.5167,13307.38451,13.12429808,31.45455263,12.59630197,0.475248564,23,8,26.77356809,39.
20617676,3,2,203,71,0,1,0,1,0,12,16932.5167,13307.38451,13.12429808,31.45455263,12.59630197
6932.5167,13307.38451,13.12429808,31.45455263,12.59630197,0.475248564,23,8,26.77356809,39
16932.5167,13307.38451,13.12429808,31.45455263,12.59630197,0.475248564,23,8,26.77356809,3
3,71,0,1,0,1,0,12,16932.5167,13307.38451,13.12429808,31.45455263,12.59630197,0.475248564,23
308,31.45455263,12.59630197,0.475248564,23,8,26.77356809,39.31819079,232.8458435,245.1618
3,71,0,1,0,1,0,12,16932.5167,13307.38451,13.12429808,31.45455263,12.59630197,0.475248564,23

08,31.45455263,12.59630197,0.475248564,23,8,26.77356809,39.31819079,232.8458435,245.1618
16932.5167,13307.38451,13.12429808,31.45455263,12.59630197,0.475248564,23,8,26.77356809,3
71,0,1,0,1,0,12,16932.5167,13307.38451,13.12429808,31.45455263,12.59630197,0.475248564,23,
932.5167,13307.38451,13.12429808,31.45455263,12.59630197,0.475248564,23,8,26.77356809,39.
,71,0,1,0,1,0,12,16932.5167,13307.38451,13.12429808,31.45455263,12.59630197,0.475248564,23
932.5167,13307.38451,13.12429808,31.45455263,12.59630197,0.475248564,23,8,26.77356809,39.
08,31.45455263,12.59630197,0.475248564,23,8,26.77356809,39.31819079,232.8458435,245.1618
16932.5167,13307.38451,13.12429808,31.45455263,12.59630197,0.475248564,23,8,26.77356809,
932.5167,13307.38451,13.12429808,31.45455263,12.59630197,0.475248564,23,8,26.77356809,39.
01,23.91361167,15.23532334,0.409341408,21,5,27.78402085,33.47905634,287.9697292,216.6019
01,23.91361167,15.23532334,0.409341408,21,5,27.78402085,33.47905634,287.9697292,216.6019
6389.04523,13864.28693,13.60850001,23.91361167,15.23532334,0.409341408,21,5,27.78402085,
389.04523,13864.28693,13.60850001,23.91361167,15.23532334,0.409341408,21,5,27.78402085,3
389.04523,13864.28693,13.60850001,23.91361167,15.23532334,0.409341408,21,5,27.78402085,3
6389.04523,13864.28693,13.60850001,23.91361167,15.23532334,0.409341408,21,5,27.78402085,
65,0,1,1,0,0,11,16389.04523,13864.28693,13.60850001,23.91361167,15.23532334,0.409341408,21
,65,0,1,1,0,0,11,16389.04523,13864.28693,13.60850001,23.91361167,15.23532334,0.409341408,2
01,23.91361167,15.23532334,0.409341408,21,5,27.78402085,33.47905634,287.9697292,216.6019
16389.04523,13864.28693,13.60850001,23.91361167,15.23532334,0.409341408,21,5,27.78402085
6389.04523,13864.28693,13.60850001,23.91361167,15.23532334,0.409341408,21,5,27.78402085,
389.04523,13864.28693,13.60850001,23.91361167,15.23532334,0.409341408,21,5,27.78402085,3
16389.04523,13864.28693,13.60850001,23.91361167,15.23532334,0.409341408,21,5,27.78402085
01,23.91361167,15.23532334,0.409341408,21,5,27.78402085,33.47905634,287.9697292,216.6019
01,23.91361167,15.23532334,0.409341408,21,5,27.78402085,33.47905634,287.9697292,216.6019
20617676,3,2,203,65,0,1,1,0,0,11,16389.04523,13864.28693,13.60850001,23.91361167,15.2353233
89.04523,13864.28693,13.60850001,23.91361167,15.23532334,0.409341408,21,5,27.78402085,33.
01,23.91361167,15.23532334,0.409341408,21,5,27.78402085,33.47905634,287.9697292,216.6019
389.04523,13864.28693,13.60850001,23.91361167,15.23532334,0.409341408,21,5,27.78402085,3
,65,0,1,1,0,0,11,16389.04523,13864.28693,13.60850001,23.91361167,15.23532334,0.409341408,2
389.04523,13864.28693,13.60850001,23.91361167,15.23532334,0.409341408,21,5,27.78402085,3
389.04523,13864.28693,13.60850001,23.91361167,15.23532334,0.409341408,21,5,27.78402085,3
01,23.91361167,15.23532334,0.409341408,21,5,27.78402085,33.47905634,287.9697292,216.6019
6389.04523,13864.28693,13.60850001,23.91361167,15.23532334,0.409341408,21,5,27.78402085,
389.04523,13864.28693,13.60850001,23.91361167,15.23532334,0.409341408,21,5,27.78402085,3
6389.04523,13864.28693,13.60850001,23.91361167,15.23532334,0.409341408,21,5,27.78402085,
01,23.91361167,15.23532334,0.409341408,21,5,27.78402085,33.47905634,287.9697292,216.6019
389.04523,13864.28693,13.60850001,23.91361167,15.23532334,0.409341408,21,5,27.78402085,3
389.04523,13864.28693,13.60850001,23.91361167,15.23532334,0.409341408,21,5,27.78402085,3
389.04523,13864.28693,13.60850001,23.91361167,15.23532334,0.409341408,21,5,27.78402085,3
389.04523,13864.28693,13.60850001,23.91361167,15.23532334,0.409341408,21,5,27.78402085,3
389.04523,13864.28693,13.60850001,23.91361167,15.23532334,0.409341408,21,5,27.78402085,3
389.04523,13864.28693,13.60850001,23.91361167,15.23532334,0.409341408,21,5,27.78402085,3
,16.00796568,17.71686072,1.81462833,27,3,30.60360258,8.003982841,198.6636123,213.6477916
84,0,1,0,1,0,9,16714.78116,12797.5841,17.32279391,16.00796568,17.71686072,1.81462833,27,3,
,84,0,1,0,1,0,9,16714.78116,12797.5841,17.32279391,16.00796568,17.71686072,1.81462833,27,3,
,84,0,1,0,1,0,9,16714.78116,12797.5841,17.32279391,16.00796568,17.71686072,1.81462833,27,3,
14.78116,12797.5841,17.32279391,16.00796568,17.71686072,1.81462833,27,3,30.60360258,8.003
6.00796568,17.71686072,1.81462833,27,3,30.60360258,8.003982841,198.6636123,213.6477916,
6714.78116,12797.5841,17.32279391,16.00796568,17.71686072,1.81462833,27,3,30.60360258,8.0
6714.78116,12797.5841,17.32279391,16.00796568,17.71686072,1.81462833,27,3,30.60360258,8.0

6714.78116,12797.5841,17.32279391,16.00796568,17.71686072,1.81462833,27,3,30.60360258,8.0
14.78116,12797.5841,17.32279391,16.00796568,17.71686072,1.81462833,27,3,30.60360258,8.003
3,84,0,1,0,1,0,9,16714.78116,12797.5841,17.32279391,16.00796568,17.71686072,1.81462833,27,3,
84,0,1,0,1,0,9,16714.78116,12797.5841,17.32279391,16.00796568,17.71686072,1.81462833,27,3,3
714.78116,12797.5841,17.32279391,16.00796568,17.71686072,1.81462833,27,3,30.60360258,8.00
91,16.00796568,17.71686072,1.81462833,27,3,30.60360258,8.003982841,198.6636123,213.647791
14.78116,12797.5841,17.32279391,16.00796568,17.71686072,1.81462833,27,3,30.60360258,8.003
714.78116,12797.5841,17.32279391,16.00796568,17.71686072,1.81462833,27,3,30.60360258,8.00
6714.78116,12797.5841,17.32279391,16.00796568,17.71686072,1.81462833,27,3,30.60360258,8.0
6714.78116,12797.5841,17.32279391,16.00796568,17.71686072,1.81462833,27,3,30.60360258,8.0
14.78116,12797.5841,17.32279391,16.00796568,17.71686072,1.81462833,27,3,30.60360258,8.003
4.78116,12797.5841,17.32279391,16.00796568,17.71686072,1.81462833,27,3,30.60360258,8.0039
84,0,1,0,1,0,9,16714.78116,12797.5841,17.32279391,16.00796568,17.71686072,1.81462833,27,3,3
91,16.00796568,17.71686072,1.81462833,27,3,30.60360258,8.003982841,198.6636123,213.647791
3,84,0,1,0,1,0,9,16714.78116,12797.5841,17.32279391,16.00796568,17.71686072,1.81462833,27,3,
91,16.00796568,17.71686072,1.81462833,27,3,30.60360258,8.003982841,198.6636123,213.647791
39691162,3,2,203,84,0,1,0,1,0,9,16714.78116,12797.5841,17.32279391,16.00796568,17.71686072,1
714.78116,12797.5841,17.32279391,16.00796568,17.71686072,1.81462833,27,3,30.60360258,8.00
714.78116,12797.5841,17.32279391,16.00796568,17.71686072,1.81462833,27,3,30.60360258,8.00
84,0,1,0,1,0,9,16714.78116,12797.5841,17.32279391,16.00796568,17.71686072,1.81462833,27,3,3
3,84,0,1,0,1,0,9,16714.78116,12797.5841,17.32279391,16.00796568,17.71686072,1.81462833,27,3,
3,84,0,1,0,1,0,9,16714.78116,12797.5841,17.32279391,16.00796568,17.71686072,1.81462833,27,3,
6714.78116,12797.5841,17.32279391,16.00796568,17.71686072,1.81462833,27,3,30.60360258,8.0
14.78116,12797.5841,17.32279391,16.00796568,17.71686072,1.81462833,27,3,30.60360258,8.003
91,16.00796568,17.71686072,1.81462833,27,3,30.60360258,8.003982841,198.6636123,213.647791
3,84,0,1,0,1,0,9,16714.78116,12797.5841,17.32279391,16.00796568,17.71686072,1.81462833,27,3,
342,21.4448468,16.09081317,1.284477451,24,4,30.86193616,25.73381615,242.5805243,220.10965
575.50861,12563.64133,15.72211842,21.4448468,16.09081317,1.284477451,24,4,30.86193616,25.
575.50861,12563.64133,15.72211842,21.4448468,16.09081317,1.284477451,24,4,30.86193616,25.
342,21.4448468,16.09081317,1.284477451,24,4,30.86193616,25.73381615,242.5805243,220.10965
575.50861,12563.64133,15.72211842,21.4448468,16.09081317,1.284477451,24,4,30.86193616,25.
7,0,1,0,1,0,9,16575.50861,12563.64133,15.72211842,21.4448468,16.09081317,1.284477451,24,4,3
75.50861,12563.64133,15.72211842,21.4448468,16.09081317,1.284477451,24,4,30.86193616,25.7
67,0,1,0,1,0,9,16575.50861,12563.64133,15.72211842,21.4448468,16.09081317,1.284477451,24,4,
575.50861,12563.64133,15.72211842,21.4448468,16.09081317,1.284477451,24,4,30.86193616,25.
342,21.4448468,16.09081317,1.284477451,24,4,30.86193616,25.73381615,242.5805243,220.10965
39691162,3,2,203,67,0,1,0,1,0,9,16575.50861,12563.64133,15.72211842,21.4448468,16.09081317,1
67,0,1,0,1,0,9,16575.50861,12563.64133,15.72211842,21.4448468,16.09081317,1.284477451,24,4,
575.50861,12563.64133,15.72211842,21.4448468,16.09081317,1.284477451,24,4,30.86193616,25.
67,0,1,0,1,0,9,16575.50861,12563.64133,15.72211842,21.4448468,16.09081317,1.284477451,24,4,
67,0,1,0,1,0,9,16575.50861,12563.64133,15.72211842,21.4448468,16.09081317,1.284477451,24,4,
5.50861,12563.64133,15.72211842,21.4448468,16.09081317,1.284477451,24,4,30.86193616,25.73
575.50861,12563.64133,15.72211842,21.4448468,16.09081317,1.284477451,24,4,30.86193616,25.
575.50861,12563.64133,15.72211842,21.4448468,16.09081317,1.284477451,24,4,30.86193616,25.
67,0,1,0,1,0,9,16575.50861,12563.64133,15.72211842,21.4448468,16.09081317,1.284477451,24,4,
75.50861,12563.64133,15.72211842,21.4448468,16.09081317,1.284477451,24,4,30.86193616,25.7
75.50861,12563.64133,15.72211842,21.4448468,16.09081317,1.284477451,24,4,30.86193616,25.7
575.50861,12563.64133,15.72211842,21.4448468,16.09081317,1.284477451,24,4,30.86193616,25.
67,0,1,0,1,0,9,16575.50861,12563.64133,15.72211842,21.4448468,16.09081317,1.284477451,24,4,
342,21.4448468,16.09081317,1.284477451,24,4,30.86193616,25.73381615,242.5805243,220.10965

6575.50861,12563.64133,15.72211842,21.4448468,16.09081317,1.284477451,24,4,30.86193616,
75.50861,12563.64133,15.72211842,21.4448468,16.09081317,1.284477451,24,4,30.86193616,25.7
575.50861,12563.64133,15.72211842,21.4448468,16.09081317,1.284477451,24,4,30.86193616,25.
575.50861,12563.64133,15.72211842,21.4448468,16.09081317,1.284477451,24,4,30.86193616,25.
842,21.4448468,16.09081317,1.284477451,24,4,30.86193616,25.73381615,242.5805243,220.10965
842,21.4448468,16.09081317,1.284477451,24,4,30.86193616,25.73381615,242.5805243,220.10965
75.50861,12563.64133,15.72211842,21.4448468,16.09081317,1.284477451,24,4,30.86193616,25.7
67,0,1,0,1,0,9,16575.50861,12563.64133,15.72211842,21.4448468,16.09081317,1.284477451,24,4,
75.50861,12563.64133,15.72211842,21.4448468,16.09081317,1.284477451,24,4,30.86193616,25.7
842,21.4448468,16.09081317,1.284477451,24,4,30.86193616,25.73381615,242.5805243,220.10965
1842,21.4448468,16.09081317,1.284477451,24,4,30.86193616,25.73381615,242.5805243,220.1096
0196,15.74026833,17.48809858,1.81462833,26,3,28.98523793,7.870134164,162.8251134,207.4433
73,1,1,0,1,0,11,16707.62546,13160.44526,16.48180196,15.74026833,17.48809858,1.81462833,26,
73,1,1,0,1,0,11,16707.62546,13160.44526,16.48180196,15.74026833,17.48809858,1.81462833,26,
6707.62546,13160.44526,16.48180196,15.74026833,17.48809858,1.81462833,26,3,28.98523793,7.
6707.62546,13160.44526,16.48180196,15.74026833,17.48809858,1.81462833,26,3,28.98523793,7.
196,15.74026833,17.48809858,1.81462833,26,3,28.98523793,7.870134164,162.8251134,207.44336
73,1,1,0,1,0,11,16707.62546,13160.44526,16.48180196,15.74026833,17.48809858,1.81462833,26,
0196,15.74026833,17.48809858,1.81462833,26,3,28.98523793,7.870134164,162.8251134,207.4433
0196,15.74026833,17.48809858,1.81462833,26,3,28.98523793,7.870134164,162.8251134,207.4433
73,1,1,0,1,0,11,16707.62546,13160.44526,16.48180196,15.74026833,17.48809858,1.81462833,26,
73,1,1,0,1,0,11,16707.62546,13160.44526,16.48180196,15.74026833,17.48809858,1.81462833,26,
73,1,1,0,1,0,11,16707.62546,13160.44526,16.48180196,15.74026833,17.48809858,1.81462833,26,
6707.62546,13160.44526,16.48180196,15.74026833,17.48809858,1.81462833,26,3,28.98523793,7.
73,1,1,0,1,0,11,16707.62546,13160.44526,16.48180196,15.74026833,17.48809858,1.81462833,26,
73,1,1,0,1,0,11,16707.62546,13160.44526,16.48180196,15.74026833,17.48809858,1.81462833,26,
3,1,1,0,1,0,11,16707.62546,13160.44526,16.48180196,15.74026833,17.48809858,1.81462833,26,3,
0196,15.74026833,17.48809858,1.81462833,26,3,28.98523793,7.870134164,162.8251134,207.4433
6707.62546,13160.44526,16.48180196,15.74026833,17.48809858,1.81462833,26,3,28.98523793,7.
6707.62546,13160.44526,16.48180196,15.74026833,17.48809858,1.81462833,26,3,28.98523793,7.
707.62546,13160.44526,16.48180196,15.74026833,17.48809858,1.81462833,26,3,28.98523793,7.8
07.62546,13160.44526,16.48180196,15.74026833,17.48809858,1.81462833,26,3,28.98523793,7.87
0196,15.74026833,17.48809858,1.81462833,26,3,28.98523793,7.870134164,162.8251134,207.4433
73,1,1,0,1,0,11,16707.62546,13160.44526,16.48180196,15.74026833,17.48809858,1.81462833,26,
6707.62546,13160.44526,16.48180196,15.74026833,17.48809858,1.81462833,26,3,28.98523793,7.
6707.62546,13160.44526,16.48180196,15.74026833,17.48809858,1.81462833,26,3,28.98523793,7.
0617676,3,2,203,73,1,1,0,1,0,11,16707.62546,13160.44526,16.48180196,15.74026833,17.48809858
707.62546,13160.44526,16.48180196,15.74026833,17.48809858,1.81462833,26,3,28.98523793,7.8
707.62546,13160.44526,16.48180196,15.74026833,17.48809858,1.81462833,26,3,28.98523793,7.8
6707.62546,13160.44526,16.48180196,15.74026833,17.48809858,1.81462833,26,3,28.98523793,7.
6707.62546,13160.44526,16.48180196,15.74026833,17.48809858,1.81462833,26,3,28.98523793,7.
707.62546,13160.44526,16.48180196,15.74026833,17.48809858,1.81462833,26,3,28.98523793,7.8
0196,15.74026833,17.48809858,1.81462833,26,3,28.98523793,7.870134164,162.8251134,207.4433
0196,15.74026833,17.48809858,1.81462833,26,3,28.98523793,7.870134164,162.8251134,207.4433
73,1,1,0,1,0,11,16707.62546,13160.44526,16.48180196,15.74026833,17.48809858,1.81462833,26,
73,1,1,0,1,0,11,16707.62546,13160.44526,16.48180196,15.74026833,17.48809858,1.81462833,26,
46,22.50939518,15.53201678,3.372279327,22,4,24.22808661,31.51315326,126.3531783,192.64283
727.90661,14989.50623,14.1822946,22.50939518,15.53201678,3.372279327,22,4,24.22808661,31.
79,0,1,0,1,0,9,16727.90661,14989.50623,14.1822946,22.50939518,15.53201678,3.372279327,22,4,
79,0,1,0,1,0,9,16727.90661,14989.50623,14.1822946,22.50939518,15.53201678,3.372279327,22,4,

46,22.50939518,15.53201678,3.372279327,22,4,24.22808661,31.51315326,126.3531783,192.64283
79,0,1,0,1,0,9,16727.90661,14989.50623,14.1822946,22.50939518,15.53201678,3.372279327,22,4,
79,0,1,0,1,0,9,16727.90661,14989.50623,14.1822946,22.50939518,15.53201678,3.372279327,22,4,
727.90661,14989.50623,14.1822946,22.50939518,15.53201678,3.372279327,22,4,24.22808661,31.
727.90661,14989.50623,14.1822946,22.50939518,15.53201678,3.372279327,22,4,24.22808661,31.
727.90661,14989.50623,14.1822946,22.50939518,15.53201678,3.372279327,22,4,24.22808661,31.
727.90661,14989.50623,14.1822946,22.50939518,15.53201678,3.372279327,22,4,24.22808661,31.
727.90661,14989.50623,14.1822946,22.50939518,15.53201678,3.372279327,22,4,24.22808661,31.
946,22.50939518,15.53201678,3.372279327,22,4,24.22808661,31.51315326,126.3531783,192.6428
46,22.50939518,15.53201678,3.372279327,22,4,24.22808661,31.51315326,126.3531783,192.64283
727.90661,14989.50623,14.1822946,22.50939518,15.53201678,3.372279327,22,4,24.22808661,31.
9691162,3,2,203,79,0,1,0,1,0,9,16727.90661,14989.50623,14.1822946,22.50939518,15.53201678,3.
727.90661,14989.50623,14.1822946,22.50939518,15.53201678,3.372279327,22,4,24.22808661,31.
27.90661,14989.50623,14.1822946,22.50939518,15.53201678,3.372279327,22,4,24.22808661,31.5
79,0,1,0,1,0,9,16727.90661,14989.50623,14.1822946,22.50939518,15.53201678,3.372279327,22,4,
79,0,1,0,1,0,9,16727.90661,14989.50623,14.1822946,22.50939518,15.53201678,3.372279327,22,4,
9,0,1,0,1,0,9,16727.90661,14989.50623,14.1822946,22.50939518,15.53201678,3.372279327,22,4,2
79,0,1,0,1,0,9,16727.90661,14989.50623,14.1822946,22.50939518,15.53201678,3.372279327,22,4,
79,0,1,0,1,0,9,16727.90661,14989.50623,14.1822946,22.50939518,15.53201678,3.372279327,22,4,
727.90661,14989.50623,14.1822946,22.50939518,15.53201678,3.372279327,22,4,24.22808661,31.
79,0,1,0,1,0,9,16727.90661,14989.50623,14.1822946,22.50939518,15.53201678,3.372279327,22,4,
7.90661,14989.50623,14.1822946,22.50939518,15.53201678,3.372279327,22,4,24.22808661,31.51
46,22.50939518,15.53201678,3.372279327,22,4,24.22808661,31.51315326,126.3531783,192.64283
727.90661,14989.50623,14.1822946,22.50939518,15.53201678,3.372279327,22,4,24.22808661,31.
727.90661,14989.50623,14.1822946,22.50939518,15.53201678,3.372279327,22,4,24.22808661,31.
727.90661,14989.50623,14.1822946,22.50939518,15.53201678,3.372279327,22,4,24.22808661,31.
727.90661,14989.50623,14.1822946,22.50939518,15.53201678,3.372279327,22,4,24.22808661,31.
79,0,1,0,1,0,9,16727.90661,14989.50623,14.1822946,22.50939518,15.53201678,3.372279327,22,4,
27.90661,14989.50623,14.1822946,22.50939518,15.53201678,3.372279327,22,4,24.22808661,31.5
727.90661,14989.50623,14.1822946,22.50939518,15.53201678,3.372279327,22,4,24.22808661,31.
79,0,1,0,1,0,9,16727.90661,14989.50623,14.1822946,22.50939518,15.53201678,3.372279327,22,4,
01,22.1288926,16.17549357,3.372279327,22,4,26.76260836,30.98044964,146.5262651,188.501850
832.62164,15040.94259,13.96310001,22.1288926,16.17549357,3.372279327,22,4,26.76260836,30.
832.62164,15040.94259,13.96310001,22.1288926,16.17549357,3.372279327,22,4,26.76260836,30.
01,22.1288926,16.17549357,3.372279327,22,4,26.76260836,30.98044964,146.5262651,188.501850
001,22.1288926,16.17549357,3.372279327,22,4,26.76260836,30.98044964,146.5262651,188.50185
32.62164,15040.94259,13.96310001,22.1288926,16.17549357,3.372279327,22,4,26.76260836,30.9
6832.62164,15040.94259,13.96310001,22.1288926,16.17549357,3.372279327,22,4,26.76260836,3
832.62164,15040.94259,13.96310001,22.1288926,16.17549357,3.372279327,22,4,26.76260836,30.
96310001,22.1288926,16.17549357,3.372279327,22,4,26.76260836,30.98044964,146.5262651,188.
001,22.1288926,16.17549357,3.372279327,22,4,26.76260836,30.98044964,146.5262651,188.50185
832.62164,15040.94259,13.96310001,22.1288926,16.17549357,3.372279327,22,4,26.76260836,30.
32.62164,15040.94259,13.96310001,22.1288926,16.17549357,3.372279327,22,4,26.76260836,30.9
32.62164,15040.94259,13.96310001,22.1288926,16.17549357,3.372279327,22,4,26.76260836,30.9
32.62164,15040.94259,13.96310001,22.1288926,16.17549357,3.372279327,22,4,26.76260836,30.9
3.96310001,22.1288926,16.17549357,3.372279327,22,4,26.76260836,30.98044964,146.5262651,18
82,1,1,0,1,0,8,16832.62164,15040.94259,13.96310001,22.1288926,16.17549357,3.372279327,22,4,
832.62164,15040.94259,13.96310001,22.1288926,16.17549357,3.372279327,22,4,26.76260836,30.
32.62164,15040.94259,13.96310001,22.1288926,16.17549357,3.372279327,22,4,26.76260836,30.9
3,82,1,1,0,1,0,8,16832.62164,15040.94259,13.96310001,22.1288926,16.17549357,3.372279327,22,

001,22.1288926,16.17549357,3.372279327,22,4,26.76260836,30.98044964,146.5262651,188.50185
32.62164,15040.94259,13.96310001,22.1288926,16.17549357,3.372279327,22,4,26.76260836,30.9
32.62164,15040.94259,13.96310001,22.1288926,16.17549357,3.372279327,22,4,26.76260836,30.9
32.62164,15040.94259,13.96310001,22.1288926,16.17549357,3.372279327,22,4,26.76260836,30.9
82,1,1,0,1,0,8,16832.62164,15040.94259,13.96310001,22.1288926,16.17549357,3.372279327,22,4,
001,22.1288926,16.17549357,3.372279327,22,4,26.76260836,30.98044964,146.5262651,188.50185
96310001,22.1288926,16.17549357,3.372279327,22,4,26.76260836,30.98044964,146.5262651,188
832.62164,15040.94259,13.96310001,22.1288926,16.17549357,3.372279327,22,4,26.76260836,30.
001,22.1288926,16.17549357,3.372279327,22,4,26.76260836,30.98044964,146.5262651,188.50185
82,1,1,0,1,0,8,16832.62164,15040.94259,13.96310001,22.1288926,16.17549357,3.372279327,22,4,
001,22.1288926,16.17549357,3.372279327,22,4,26.76260836,30.98044964,146.5262651,188.50185
32.62164,15040.94259,13.96310001,22.1288926,16.17549357,3.372279327,22,4,26.76260836,30.9
3.96310001,22.1288926,16.17549357,3.372279327,22,4,26.76260836,30.98044964,146.5262651,18
96310001,22.1288926,16.17549357,3.372279327,22,4,26.76260836,30.98044964,146.5262651,188.
832.62164,15040.94259,13.96310001,22.1288926,16.17549357,3.372279327,22,4,26.76260836,30.
310001,22.1288926,16.17549357,3.372279327,22,4,26.76260836,30.98044964,146.5262651,188.50
32.62164,15040.94259,13.96310001,22.1288926,16.17549357,3.372279327,22,4,26.76260836,30.9
54.48221,14258.17485,13.18138852,7.379938729,16.20199814,0.910871208,26,2,23.82789463,36.
3,82,1,1,0,1,0,8,16754.48221,14258.17485,13.18138852,7.379938729,16.20199814,0.910871208,26
82,1,1,0,1,0,8,16754.48221,14258.17485,13.18138852,7.379938729,16.20199814,0.910871208,26,
754.48221,14258.17485,13.18138852,7.379938729,16.20199814,0.910871208,26,2,23.82789463,36
82,1,1,0,1,0,8,16754.48221,14258.17485,13.18138852,7.379938729,16.20199814,0.910871208,26,
54.48221,14258.17485,13.18138852,7.379938729,16.20199814,0.910871208,26,2,23.82789463,36.
54.48221,14258.17485,13.18138852,7.379938729,16.20199814,0.910871208,26,2,23.82789463,36.
754.48221,14258.17485,13.18138852,7.379938729,16.20199814,0.910871208,26,2,23.82789463,36
352,7.379938729,16.20199814,0.910871208,26,2,23.82789463,36.89969365,113.8522371,195.6929
3.18138852,7.379938729,16.20199814,0.910871208,26,2,23.82789463,36.89969365,113.8522371,1
352,7.379938729,16.20199814,0.910871208,26,2,23.82789463,36.89969365,113.8522371,195.6929
82,1,1,0,1,0,8,16754.48221,14258.17485,13.18138852,7.379938729,16.20199814,0.910871208,26,
754.48221,14258.17485,13.18138852,7.379938729,16.20199814,0.910871208,26,2,23.82789463,36
352,7.379938729,16.20199814,0.910871208,26,2,23.82789463,36.89969365,113.8522371,195.6929
754.48221,14258.17485,13.18138852,7.379938729,16.20199814,0.910871208,26,2,23.82789463,36
352,7.379938729,16.20199814,0.910871208,26,2,23.82789463,36.89969365,113.8522371,195.6929
54.48221,14258.17485,13.18138852,7.379938729,16.20199814,0.910871208,26,2,23.82789463,36.
754.48221,14258.17485,13.18138852,7.379938729,16.20199814,0.910871208,26,2,23.82789463,36
352,7.379938729,16.20199814,0.910871208,26,2,23.82789463,36.89969365,113.8522371,195.6929
52,7.379938729,16.20199814,0.910871208,26,2,23.82789463,36.89969365,113.8522371,195.69292
754.48221,14258.17485,13.18138852,7.379938729,16.20199814,0.910871208,26,2,23.82789463,36
754.48221,14258.17485,13.18138852,7.379938729,16.20199814,0.910871208,26,2,23.82789463,36
754.48221,14258.17485,13.18138852,7.379938729,16.20199814,0.910871208,26,2,23.82789463,36
54.48221,14258.17485,13.18138852,7.379938729,16.20199814,0.910871208,26,2,23.82789463,36.
54.48221,14258.17485,13.18138852,7.379938729,16.20199814,0.910871208,26,2,23.82789463,36.
82,1,1,0,1,0,8,16754.48221,14258.17485,13.18138852,7.379938729,16.20199814,0.910871208,26,
754.48221,14258.17485,13.18138852,7.379938729,16.20199814,0.910871208,26,2,23.82789463,36
754.48221,14258.17485,13.18138852,7.379938729,16.20199814,0.910871208,26,2,23.82789463,36
754.48221,14258.17485,13.18138852,7.379938729,16.20199814,0.910871208,26,2,23.82789463,36
18138852,7.379938729,16.20199814,0.910871208,26,2,23.82789463,36.89969365,113.8522371,19
2,1,1,0,1,0,8,16754.48221,14258.17485,13.18138852,7.379938729,16.20199814,0.910871208,26,2,
754.48221,14258.17485,13.18138852,7.379938729,16.20199814,0.910871208,26,2,23.82789463,36
352,7.379938729,16.20199814,0.910871208,26,2,23.82789463,36.89969365,113.8522371,195.6929

54.48221,14258.17485,13.18138852,7.379938729,16.20199814,0.910871208,26,2,23.82789463,36.
754.48221,14258.17485,13.18138852,7.379938729,16.20199814,0.910871208,26,2,23.82789463,3
574,13.86782505,15.95932509,2.468522205,21,3,26.75813985,73.96173358,221.2618975,179.3364
67,1,1,1,1,0,8,16565.44508,14213.85733,12.52508674,13.86782505,15.95932509,2.468522205,21,
65.44508,14213.85733,12.52508674,13.86782505,15.95932509,2.468522205,21,3,26.75813985,73.
67,1,1,1,1,0,8,16565.44508,14213.85733,12.52508674,13.86782505,15.95932509,2.468522205,21,
67,1,1,1,1,0,8,16565.44508,14213.85733,12.52508674,13.86782505,15.95932509,2.468522205,21,
65.44508,14213.85733,12.52508674,13.86782505,15.95932509,2.468522205,21,3,26.75813985,73.
574,13.86782505,15.95932509,2.468522205,21,3,26.75813985,73.96173358,221.2618975,179.3364
65.44508,14213.85733,12.52508674,13.86782505,15.95932509,2.468522205,21,3,26.75813985,73.
67,1,1,1,1,0,8,16565.44508,14213.85733,12.52508674,13.86782505,15.95932509,2.468522205,21,
565.44508,14213.85733,12.52508674,13.86782505,15.95932509,2.468522205,21,3,26.75813985,73
565.44508,14213.85733,12.52508674,13.86782505,15.95932509,2.468522205,21,3,26.75813985,73
565.44508,14213.85733,12.52508674,13.86782505,15.95932509,2.468522205,21,3,26.75813985,73
65.44508,14213.85733,12.52508674,13.86782505,15.95932509,2.468522205,21,3,26.75813985,73.
565.44508,14213.85733,12.52508674,13.86782505,15.95932509,2.468522205,21,3,26.75813985,73
65.44508,14213.85733,12.52508674,13.86782505,15.95932509,2.468522205,21,3,26.75813985,73.
50308838,3,2,203,67,1,1,1,1,0,8,16565.44508,14213.85733,12.52508674,13.86782505,15.95932509
574,13.86782505,15.95932509,2.468522205,21,3,26.75813985,73.96173358,221.2618975,179.3364
16565.44508,14213.85733,12.52508674,13.86782505,15.95932509,2.468522205,21,3,26.75813985,
16565.44508,14213.85733,12.52508674,13.86782505,15.95932509,2.468522205,21,3,26.75813985,
0617676,3,2,203,67,1,1,1,1,0,8,16565.44508,14213.85733,12.52508674,13.86782505,15.95932509,
67,1,1,1,1,0,8,16565.44508,14213.85733,12.52508674,13.86782505,15.95932509,2.468522205,21,
65.44508,14213.85733,12.52508674,13.86782505,15.95932509,2.468522205,21,3,26.75813985,73.
65.44508,14213.85733,12.52508674,13.86782505,15.95932509,2.468522205,21,3,26.75813985,73.
65.44508,14213.85733,12.52508674,13.86782505,15.95932509,2.468522205,21,3,26.75813985,73.
565.44508,14213.85733,12.52508674,13.86782505,15.95932509,2.468522205,21,3,26.75813985,73
65.44508,14213.85733,12.52508674,13.86782505,15.95932509,2.468522205,21,3,26.75813985,73.
565.44508,14213.85733,12.52508674,13.86782505,15.95932509,2.468522205,21,3,26.75813985,73
574,13.86782505,15.95932509,2.468522205,21,3,26.75813985,73.96173358,221.2618975,179.3364
67,1,1,1,1,0,8,16565.44508,14213.85733,12.52508674,13.86782505,15.95932509,2.468522205,21,
7,1,1,1,1,0,8,16565.44508,14213.85733,12.52508674,13.86782505,15.95932509,2.468522205,21,3,
565.44508,14213.85733,12.52508674,13.86782505,15.95932509,2.468522205,21,3,26.75813985,73
65.44508,14213.85733,12.52508674,13.86782505,15.95932509,2.468522205,21,3,26.75813985,73.
574,13.86782505,15.95932509,2.468522205,21,3,26.75813985,73.96173358,221.2618975,179.3364
565.44508,14213.85733,12.52508674,13.86782505,15.95932509,2.468522205,21,3,26.75813985,73
31.22913,14709.99232,10.41849242,10.82471144,5.07965505,0.439943831,18,3,26.56715568,25.2
242,10.82471144,5.07965505,0.439943831,18,3,26.56715568,25.25766003,158.7618437,199.51412
431.22913,14709.99232,10.41849242,10.82471144,5.07965505,0.439943831,18,3,26.56715568,25.
31.22913,14709.99232,10.41849242,10.82471144,5.07965505,0.439943831,18,3,26.56715568,25.2
1849242,10.82471144,5.07965505,0.439943831,18,3,26.56715568,25.25766003,158.7618437,199.5
431.22913,14709.99232,10.41849242,10.82471144,5.07965505,0.439943831,18,3,26.56715568,25.
31.22913,14709.99232,10.41849242,10.82471144,5.07965505,0.439943831,18,3,26.56715568,25.2
242,10.82471144,5.07965505,0.439943831,18,3,26.56715568,25.25766003,158.7618437,199.51412
6431.22913,14709.99232,10.41849242,10.82471144,5.07965505,0.439943831,18,3,26.56715568,2
42,10.82471144,5.07965505,0.439943831,18,3,26.56715568,25.25766003,158.7618437,199.514129
6431.22913,14709.99232,10.41849242,10.82471144,5.07965505,0.439943831,18,3,26.56715568,2
242,10.82471144,5.07965505,0.439943831,18,3,26.56715568,25.25766003,158.7618437,199.51412
431.22913,14709.99232,10.41849242,10.82471144,5.07965505,0.439943831,18,3,26.56715568,25.
431.22913,14709.99232,10.41849242,10.82471144,5.07965505,0.439943831,18,3,26.56715568,25.

5,76,1,1,1,1,0,8,16431.22913,14709.99232,10.41849242,10.82471144,5.07965505,0.439943831,18,3
849242,10.82471144,5.07965505,0.439943831,18,3,26.56715568,25.25766003,158.7618437,199.51
5,76,1,1,1,1,0,8,16431.22913,14709.99232,10.41849242,10.82471144,5.07965505,0.439943831,18,3
431.22913,14709.99232,10.41849242,10.82471144,5.07965505,0.439943831,18,3,26.56715568,25.
31.22913,14709.99232,10.41849242,10.82471144,5.07965505,0.439943831,18,3,26.56715568,25.2
5,76,1,1,1,1,0,8,16431.22913,14709.99232,10.41849242,10.82471144,5.07965505,0.439943831,18,3
849242,10.82471144,5.07965505,0.439943831,18,3,26.56715568,25.25766003,158.7618437,199.51
849242,10.82471144,5.07965505,0.439943831,18,3,26.56715568,25.25766003,158.7618437,199.51
5,76,1,1,1,1,0,8,16431.22913,14709.99232,10.41849242,10.82471144,5.07965505,0.439943831,18,3
76,1,1,1,1,0,8,16431.22913,14709.99232,10.41849242,10.82471144,5.07965505,0.439943831,18,3,
6431.22913,14709.99232,10.41849242,10.82471144,5.07965505,0.439943831,18,3,26.56715568,25
0.41849242,10.82471144,5.07965505,0.439943831,18,3,26.56715568,25.25766003,158.7618437,19
76,1,1,1,1,0,8,16431.22913,14709.99232,10.41849242,10.82471144,5.07965505,0.439943831,18,3,
31.22913,14709.99232,10.41849242,10.82471144,5.07965505,0.439943831,18,3,26.56715568,25.2
431.22913,14709.99232,10.41849242,10.82471144,5.07965505,0.439943831,18,3,26.56715568,25.
206438,11.15385122,5.07965505,0.439943831,18,3,26.99336739,29.74360324,176.582373,201.931
0.38206438,11.15385122,5.07965505,0.439943831,18,3,26.99336739,29.74360324,176.582373,201
80.55589,14992.63173,10.38206438,11.15385122,5.07965505,0.439943831,18,3,26.99336739,29.7
80.55589,14992.63173,10.38206438,11.15385122,5.07965505,0.439943831,18,3,26.99336739,29.7
80.55589,14992.63173,10.38206438,11.15385122,5.07965505,0.439943831,18,3,26.99336739,29.7
438,11.15385122,5.07965505,0.439943831,18,3,26.99336739,29.74360324,176.582373,201.931152
85,0,1,1,0,0,9,16480.55589,14992.63173,10.38206438,11.15385122,5.07965505,0.439943831,18,3,
85,0,1,1,0,0,9,16480.55589,14992.63173,10.38206438,11.15385122,5.07965505,0.439943831,18,3,
85,0,1,1,0,0,9,16480.55589,14992.63173,10.38206438,11.15385122,5.07965505,0.439943831,18,3,
.38206438,11.15385122,5.07965505,0.439943831,18,3,26.99336739,29.74360324,176.582373,201.
438,11.15385122,5.07965505,0.439943831,18,3,26.99336739,29.74360324,176.582373,201.931152
80.55589,14992.63173,10.38206438,11.15385122,5.07965505,0.439943831,18,3,26.99336739,29.7
80.55589,14992.63173,10.38206438,11.15385122,5.07965505,0.439943831,18,3,26.99336739,29.7
480.55589,14992.63173,10.38206438,11.15385122,5.07965505,0.439943831,18,3,26.99336739,29.
0617676,3,2,205,85,0,1,1,0,0,9,16480.55589,14992.63173,10.38206438,11.15385122,5.07965505,0.
438,11.15385122,5.07965505,0.439943831,18,3,26.99336739,29.74360324,176.582373,201.931152
438,11.15385122,5.07965505,0.439943831,18,3,26.99336739,29.74360324,176.582373,201.931152
480.55589,14992.63173,10.38206438,11.15385122,5.07965505,0.439943831,18,3,26.99336739,29.
38206438,11.15385122,5.07965505,0.439943831,18,3,26.99336739,29.74360324,176.582373,201.9
10.38206438,11.15385122,5.07965505,0.439943831,18,3,26.99336739,29.74360324,176.582373,20
206438,11.15385122,5.07965505,0.439943831,18,3,26.99336739,29.74360324,176.582373,201.931
6480.55589,14992.63173,10.38206438,11.15385122,5.07965505,0.439943831,18,3,26.99336739,29
80.55589,14992.63173,10.38206438,11.15385122,5.07965505,0.439943831,18,3,26.99336739,29.7
80.55589,14992.63173,10.38206438,11.15385122,5.07965505,0.439943831,18,3,26.99336739,29.7
5,0,1,1,0,0,9,16480.55589,14992.63173,10.38206438,11.15385122,5.07965505,0.439943831,18,3,2
01,4.914504105,11.94125243,6.263850985,21,1,25.73289589,49.14504105,179.2825401,188.13606
901,4.914504105,11.94125243,6.263850985,21,1,25.73289589,49.14504105,179.2825401,188.1360
671.47638,17680.59529,13.15236901,4.914504105,11.94125243,6.263850985,21,1,25.73289589,49
71.47638,17680.59529,13.15236901,4.914504105,11.94125243,6.263850985,21,1,25.73289589,49.
671.47638,17680.59529,13.15236901,4.914504105,11.94125243,6.263850985,21,1,25.73289589,49
71.47638,17680.59529,13.15236901,4.914504105,11.94125243,6.263850985,21,1,25.73289589,49.
9382324,3,2,205,72,1,1,1,0,0,5,16671.47638,17680.59529,13.15236901,4.914504105,11.94125243,6
3.15236901,4.914504105,11.94125243,6.263850985,21,1,25.73289589,49.14504105,179.2825401,1
71.47638,17680.59529,13.15236901,4.914504105,11.94125243,6.263850985,21,1,25.73289589,49.
71.47638,17680.59529,13.15236901,4.914504105,11.94125243,6.263850985,21,1,25.73289589,49.

4.914504105,11.94125243,6.263850985,21,1,25.73289589,49.14504105,179.2825401,188.1360611
671.47638,17680.59529,13.15236901,4.914504105,11.94125243,6.263850985,21,1,25.73289589,4
71.47638,17680.59529,13.15236901,4.914504105,11.94125243,6.263850985,21,1,25.73289589,49.
.15236901,4.914504105,11.94125243,6.263850985,21,1,25.73289589,49.14504105,179.2825401,1
71.47638,17680.59529,13.15236901,4.914504105,11.94125243,6.263850985,21,1,25.73289589,49.
01,4.914504105,11.94125243,6.263850985,21,1,25.73289589,49.14504105,179.2825401,188.1360
,4.914504105,11.94125243,6.263850985,21,1,25.73289589,49.14504105,179.2825401,188.136061
671.47638,17680.59529,13.15236901,4.914504105,11.94125243,6.263850985,21,1,25.73289589,4
71.47638,17680.59529,13.15236901,4.914504105,11.94125243,6.263850985,21,1,25.73289589,49.
71.47638,17680.59529,13.15236901,4.914504105,11.94125243,6.263850985,21,1,25.73289589,49.
671.47638,17680.59529,13.15236901,4.914504105,11.94125243,6.263850985,21,1,25.73289589,4
9,5.565192744,14.96332204,6.263850985,18,1,21.64983365,50.08673469,158.233252,177.756528
75,1,1,1,1,0,11,17224.0234,21459.55025,11.39464929,5.565192744,14.96332204,6.263850985,18,
7224.0234,21459.55025,11.39464929,5.565192744,14.96332204,6.263850985,18,1,21.64983365,5
75,1,1,1,1,0,11,17224.0234,21459.55025,11.39464929,5.565192744,14.96332204,6.263850985,18,
224.0234,21459.55025,11.39464929,5.565192744,14.96332204,6.263850985,18,1,21.64983365,50.
59845581,3,2,205,75,1,1,1,1,0,11,17224.0234,21459.55025,11.39464929,5.565192744,14.96332204
75,1,1,1,1,0,11,17224.0234,21459.55025,11.39464929,5.565192744,14.96332204,6.263850985,18,
75,1,1,1,1,0,11,17224.0234,21459.55025,11.39464929,5.565192744,14.96332204,6.263850985,18,
75,1,1,1,1,0,11,17224.0234,21459.55025,11.39464929,5.565192744,14.96332204,6.263850985,18,
224.0234,21459.55025,11.39464929,5.565192744,14.96332204,6.263850985,18,1,21.64983365,50.
,75,1,1,1,1,0,11,17224.0234,21459.55025,11.39464929,5.565192744,14.96332204,6.263850985,18
224.0234,21459.55025,11.39464929,5.565192744,14.96332204,6.263850985,18,1,21.64983365,50.
29,5.565192744,14.96332204,6.263850985,18,1,21.64983365,50.08673469,158.233252,177.75652
224.0234,21459.55025,11.39464929,5.565192744,14.96332204,6.263850985,18,1,21.64983365,50.
,75,1,1,1,1,0,11,17224.0234,21459.55025,11.39464929,5.565192744,14.96332204,6.263850985,18
,75,1,1,1,1,0,11,17224.0234,21459.55025,11.39464929,5.565192744,14.96332204,6.263850985,18
7224.0234,21459.55025,11.39464929,5.565192744,14.96332204,6.263850985,18,1,21.64983365,5
7224.0234,21459.55025,11.39464929,5.565192744,14.96332204,6.263850985,18,1,21.64983365,5
224.0234,21459.55025,11.39464929,5.565192744,14.96332204,6.263850985,18,1,21.64983365,50.
75,1,1,1,1,0,11,17224.0234,21459.55025,11.39464929,5.565192744,14.96332204,6.263850985,18,
7224.0234,21459.55025,11.39464929,5.565192744,14.96332204,6.263850985,18,1,21.64983365,5
7224.0234,21459.55025,11.39464929,5.565192744,14.96332204,6.263850985,18,1,21.64983365,5
7224.0234,21459.55025,11.39464929,5.565192744,14.96332204,6.263850985,18,1,21.64983365,5
17224.0234,21459.55025,11.39464929,5.565192744,14.96332204,6.263850985,18,1,21.64983365,
7224.0234,21459.55025,11.39464929,5.565192744,14.96332204,6.263850985,18,1,21.64983365,5
929,5.565192744,14.96332204,6.263850985,18,1,21.64983365,50.08673469,158.233252,177.7565
17224.0234,21459.55025,11.39464929,5.565192744,14.96332204,6.263850985,18,1,21.64983365,
29,5.565192744,14.96332204,6.263850985,18,1,21.64983365,50.08673469,158.233252,177.75652
224.0234,21459.55025,11.39464929,5.565192744,14.96332204,6.263850985,18,1,21.64983365,50.
9,7.024023757,16.91246128,6.859172809,23,2,24.89643165,35.12011879,160.4165362,179.15667
579.17176,18264.6455,12.69229849,7.024023757,16.91246128,6.859172809,23,2,24.89643165,35.
9,7.024023757,16.91246128,6.859172809,23,2,24.89643165,35.12011879,160.4165362,179.15667
579.17176,18264.6455,12.69229849,7.024023757,16.91246128,6.859172809,23,2,24.89643165,35.
,82,0,1,0,1,0,8,16579.17176,18264.6455,12.69229849,7.024023757,16.91246128,6.859172809,23,
9,7.024023757,16.91246128,6.859172809,23,2,24.89643165,35.12011879,160.4165362,179.15667
79.17176,18264.6455,12.69229849,7.024023757,16.91246128,6.859172809,23,2,24.89643165,35.1
79.17176,18264.6455,12.69229849,7.024023757,16.91246128,6.859172809,23,2,24.89643165,35.1
9,7.024023757,16.91246128,6.859172809,23,2,24.89643165,35.12011879,160.4165362,179.15667
79.17176,18264.6455,12.69229849,7.024023757,16.91246128,6.859172809,23,2,24.89643165,35.1

,7.024023757,16.91246128,6.859172809,23,2,24.89643165,35.12011879,160.4165362,179.156674
79.17176,18264.6455,12.69229849,7.024023757,16.91246128,6.859172809,23,2,24.89643165,35.1
79.17176,18264.6455,12.69229849,7.024023757,16.91246128,6.859172809,23,2,24.89643165,35.1
79.17176,18264.6455,12.69229849,7.024023757,16.91246128,6.859172809,23,2,24.89643165,35.1
579.17176,18264.6455,12.69229849,7.024023757,16.91246128,6.859172809,23,2,24.89643165,35.
,7.024023757,16.91246128,6.859172809,23,2,24.89643165,35.12011879,160.4165362,179.156674
79.17176,18264.6455,12.69229849,7.024023757,16.91246128,6.859172809,23,2,24.89643165,35.1
49,7.024023757,16.91246128,6.859172809,23,2,24.89643165,35.12011879,160.4165362,179.15667
79.17176,18264.6455,12.69229849,7.024023757,16.91246128,6.859172809,23,2,24.89643165,35.1
79.17176,18264.6455,12.69229849,7.024023757,16.91246128,6.859172809,23,2,24.89643165,35.1
79.17176,18264.6455,12.69229849,7.024023757,16.91246128,6.859172809,23,2,24.89643165,35.1
79.17176,18264.6455,12.69229849,7.024023757,16.91246128,6.859172809,23,2,24.89643165,35.1
79.17176,18264.6455,12.69229849,7.024023757,16.91246128,6.859172809,23,2,24.89643165,35.1
20617676,3,2,205,82,0,1,0,1,0,8,16579.17176,18264.6455,12.69229849,7.024023757,16.91246128,
49,7.024023757,16.91246128,6.859172809,23,2,24.89643165,35.12011879,160.4165362,179.15667
79.17176,18264.6455,12.69229849,7.024023757,16.91246128,6.859172809,23,2,24.89643165,35.1
79.17176,18264.6455,12.69229849,7.024023757,16.91246128,6.859172809,23,2,24.89643165,35.1
82,0,1,0,1,0,8,16579.17176,18264.6455,12.69229849,7.024023757,16.91246128,6.859172809,23,2,
79.17176,18264.6455,12.69229849,7.024023757,16.91246128,6.859172809,23,2,24.89643165,35.1
579.17176,18264.6455,12.69229849,7.024023757,16.91246128,6.859172809,23,2,24.89643165,35.
79.17176,18264.6455,12.69229849,7.024023757,16.91246128,6.859172809,23,2,24.89643165,35.1
39,4.893779046,15.47078921,6.263850985,18,1,23.60396718,48.93779046,200.6491264,177.3042
69,1,0,0,1,0,9,17007.07681,19995.97223,10.97858939,4.893779046,15.47078921,6.263850985,18,
007.07681,19995.97223,10.97858939,4.893779046,15.47078921,6.263850985,18,1,23.60396718,4
39,4.893779046,15.47078921,6.263850985,18,1,23.60396718,48.93779046,200.6491264,177.3042
39,4.893779046,15.47078921,6.263850985,18,1,23.60396718,48.93779046,200.6491264,177.3042
07.07681,19995.97223,10.97858939,4.893779046,15.47078921,6.263850985,18,1,23.60396718,48.
07.07681,19995.97223,10.97858939,4.893779046,15.47078921,6.263850985,18,1,23.60396718,48.
39,4.893779046,15.47078921,6.263850985,18,1,23.60396718,48.93779046,200.6491264,177.3042
39,4.893779046,15.47078921,6.263850985,18,1,23.60396718,48.93779046,200.6491264,177.3042
007.07681,19995.97223,10.97858939,4.893779046,15.47078921,6.263850985,18,1,23.60396718,4
1544189,3,2,205,69,1,0,0,1,0,9,17007.07681,19995.97223,10.97858939,4.893779046,15.47078921
39,4.893779046,15.47078921,6.263850985,18,1,23.60396718,48.93779046,200.6491264,177.3042
007.07681,19995.97223,10.97858939,4.893779046,15.47078921,6.263850985,18,1,23.60396718,4
,69,1,0,0,1,0,9,17007.07681,19995.97223,10.97858939,4.893779046,15.47078921,6.263850985,18
39,4.893779046,15.47078921,6.263850985,18,1,23.60396718,48.93779046,200.6491264,177.3042
07.07681,19995.97223,10.97858939,4.893779046,15.47078921,6.263850985,18,1,23.60396718,48.
07.07681,19995.97223,10.97858939,4.893779046,15.47078921,6.263850985,18,1,23.60396718,48.
007.07681,19995.97223,10.97858939,4.893779046,15.47078921,6.263850985,18,1,23.60396718,4
7007.07681,19995.97223,10.97858939,4.893779046,15.47078921,6.263850985,18,1,23.60396718,
,69,1,0,0,1,0,9,17007.07681,19995.97223,10.97858939,4.893779046,15.47078921,6.263850985,18
007.07681,19995.97223,10.97858939,4.893779046,15.47078921,6.263850985,18,1,23.60396718,4
07.07681,19995.97223,10.97858939,4.893779046,15.47078921,6.263850985,18,1,23.60396718,48.
007.07681,19995.97223,10.97858939,4.893779046,15.47078921,6.263850985,18,1,23.60396718,4
,69,1,0,0,1,0,9,17007.07681,19995.97223,10.97858939,4.893779046,15.47078921,6.263850985,18
007.07681,19995.97223,10.97858939,4.893779046,15.47078921,6.263850985,18,1,23.60396718,4
007.07681,19995.97223,10.97858939,4.893779046,15.47078921,6.263850985,18,1,23.60396718,4
07.07681,19995.97223,10.97858939,4.893779046,15.47078921,6.263850985,18,1,23.60396718,48.
39,4.893779046,15.47078921,6.263850985,18,1,23.60396718,48.93779046,200.6491264,177.3042
7007.07681,19995.97223,10.97858939,4.893779046,15.47078921,6.263850985,18,1,23.60396718,

007.07681,19995.97223,10.97858939,4.893779046,15.47078921,6.263850985,18,1,23.60396718,48
07.07681,19995.97223,10.97858939,4.893779046,15.47078921,6.263850985,18,1,23.60396718,48.
07.07681,19995.97223,10.97858939,4.893779046,15.47078921,6.263850985,18,1,23.60396718,48.
07.07681,19995.97223,10.97858939,4.893779046,15.47078921,6.263850985,18,1,23.60396718,48.
21826,8.079282874,16.40172211,6.859172809,21,2,23.93043651,40.39641437,216.3905069,179.99
68,1,1,0,1,0,10,16906.60888,18676.31681,11.96521826,8.079282874,16.40172211,6.859172809,21
6906.60888,18676.31681,11.96521826,8.079282874,16.40172211,6.859172809,21,2,23.93043651,4
68,1,1,0,1,0,10,16906.60888,18676.31681,11.96521826,8.079282874,16.40172211,6.859172809,21
1826,8.079282874,16.40172211,6.859172809,21,2,23.93043651,40.39641437,216.3905069,179.998
8,1,1,0,1,0,10,16906.60888,18676.31681,11.96521826,8.079282874,16.40172211,6.859172809,21,
6906.60888,18676.31681,11.96521826,8.079282874,16.40172211,6.859172809,21,2,23.93043651,4
68,1,1,0,1,0,10,16906.60888,18676.31681,11.96521826,8.079282874,16.40172211,6.859172809,21
6906.60888,18676.31681,11.96521826,8.079282874,16.40172211,6.859172809,21,2,23.93043651,4
1826,8.079282874,16.40172211,6.859172809,21,2,23.93043651,40.39641437,216.3905069,179.998
06.60888,18676.31681,11.96521826,8.079282874,16.40172211,6.859172809,21,2,23.93043651,40.
6906.60888,18676.31681,11.96521826,8.079282874,16.40172211,6.859172809,21,2,23.93043651,4
906.60888,18676.31681,11.96521826,8.079282874,16.40172211,6.859172809,21,2,23.93043651,40
68,1,1,0,1,0,10,16906.60888,18676.31681,11.96521826,8.079282874,16.40172211,6.859172809,21
6906.60888,18676.31681,11.96521826,8.079282874,16.40172211,6.859172809,21,2,23.93043651,4
06.60888,18676.31681,11.96521826,8.079282874,16.40172211,6.859172809,21,2,23.93043651,40.
6906.60888,18676.31681,11.96521826,8.079282874,16.40172211,6.859172809,21,2,23.93043651,4
906.60888,18676.31681,11.96521826,8.079282874,16.40172211,6.859172809,21,2,23.93043651,40
21826,8.079282874,16.40172211,6.859172809,21,2,23.93043651,40.39641437,216.3905069,179.99
6906.60888,18676.31681,11.96521826,8.079282874,16.40172211,6.859172809,21,2,23.93043651,4
06.60888,18676.31681,11.96521826,8.079282874,16.40172211,6.859172809,21,2,23.93043651,40.
906.60888,18676.31681,11.96521826,8.079282874,16.40172211,6.859172809,21,2,23.93043651,40
16906.60888,18676.31681,11.96521826,8.079282874,16.40172211,6.859172809,21,2,23.93043651
68,1,1,0,1,0,10,16906.60888,18676.31681,11.96521826,8.079282874,16.40172211,6.859172809,21
1826,8.079282874,16.40172211,6.859172809,21,2,23.93043651,40.39641437,216.3905069,179.998
906.60888,18676.31681,11.96521826,8.079282874,16.40172211,6.859172809,21,2,23.93043651,40
6906.60888,18676.31681,11.96521826,8.079282874,16.40172211,6.859172809,21,2,23.93043651,4
906.60888,18676.31681,11.96521826,8.079282874,16.40172211,6.859172809,21,2,23.93043651,40
6906.60888,18676.31681,11.96521826,8.079282874,16.40172211,6.859172809,21,2,23.93043651,4
16906.60888,18676.31681,11.96521826,8.079282874,16.40172211,6.859172809,21,2,23.93043651
906.60888,18676.31681,11.96521826,8.079282874,16.40172211,6.859172809,21,2,23.93043651,40
68,1,1,0,1,0,10,16906.60888,18676.31681,11.96521826,8.079282874,16.40172211,6.859172809,21
906.60888,18676.31681,11.96521826,8.079282874,16.40172211,6.859172809,21,2,23.93043651,40
1826,8.079282874,16.40172211,6.859172809,21,2,23.93043651,40.39641437,216.3905069,179.998
1826,8.079282874,16.40172211,6.859172809,21,2,23.93043651,40.39641437,216.3905069,179.998
8522,5.76184119,11.32358648,6.263850985,16,1,23.04799652,46.09472952,96.85805408,179.7743
789.15885,21002.09294,9.795398522,5.76184119,11.32358648,6.263850985,16,1,23.04799652,46.
789.15885,21002.09294,9.795398522,5.76184119,11.32358648,6.263850985,16,1,23.04799652,46.
6789.15885,21002.09294,9.795398522,5.76184119,11.32358648,6.263850985,16,1,23.04799652,46
789.15885,21002.09294,9.795398522,5.76184119,11.32358648,6.263850985,16,1,23.04799652,46.
789.15885,21002.09294,9.795398522,5.76184119,11.32358648,6.263850985,16,1,23.04799652,46.
70,1,1,1,0,0,12,16789.15885,21002.09294,9.795398522,5.76184119,11.32358648,6.263850985,16,
8522,5.76184119,11.32358648,6.263850985,16,1,23.04799652,46.09472952,96.85805408,179.7743
789.15885,21002.09294,9.795398522,5.76184119,11.32358648,6.263850985,16,1,23.04799652,46.
6789.15885,21002.09294,9.795398522,5.76184119,11.32358648,6.263850985,16,1,23.04799652,46
70,1,1,1,0,0,12,16789.15885,21002.09294,9.795398522,5.76184119,11.32358648,6.263850985,16,

5,70,1,1,1,0,0,12,16789.15885,21002.09294,9.795398522,5.76184119,11.32358648,6.263850985,16
522,5.76184119,11.32358648,6.263850985,16,1,23.04799652,46.09472952,96.85805408,179.7743
6789.15885,21002.09294,9.795398522,5.76184119,11.32358648,6.263850985,16,1,23.04799652,4
617676,3,2,205,70,1,1,1,0,0,12,16789.15885,21002.09294,9.795398522,5.76184119,11.32358648,
50772095,3,2,205,70,1,1,1,0,0,12,16789.15885,21002.09294,9.795398522,5.76184119,11.3235864
70,1,1,1,0,0,12,16789.15885,21002.09294,9.795398522,5.76184119,11.32358648,6.263850985,16,
789.15885,21002.09294,9.795398522,5.76184119,11.32358648,6.263850985,16,1,23.04799652,46.
6789.15885,21002.09294,9.795398522,5.76184119,11.32358648,6.263850985,16,1,23.04799652,4
789.15885,21002.09294,9.795398522,5.76184119,11.32358648,6.263850985,16,1,23.04799652,46.
6789.15885,21002.09294,9.795398522,5.76184119,11.32358648,6.263850985,16,1,23.04799652,4
70,1,1,1,0,0,12,16789.15885,21002.09294,9.795398522,5.76184119,11.32358648,6.263850985,16,
6789.15885,21002.09294,9.795398522,5.76184119,11.32358648,6.263850985,16,1,23.04799652,4
70,1,1,1,0,0,12,16789.15885,21002.09294,9.795398522,5.76184119,11.32358648,6.263850985,16,
522,5.76184119,11.32358648,6.263850985,16,1,23.04799652,46.09472952,96.85805408,179.7743
184,6.330057584,16.98654773,6.859172809,24,2,26.15409264,28.48525913,128.6028974,182.127
6570.15182,18226.32443,12.83928184,6.330057584,16.98654773,6.859172809,24,2,26.15409264,
6570.15182,18226.32443,12.83928184,6.330057584,16.98654773,6.859172809,24,2,26.15409264,
570.15182,18226.32443,12.83928184,6.330057584,16.98654773,6.859172809,24,2,26.15409264,2
70.15182,18226.32443,12.83928184,6.330057584,16.98654773,6.859172809,24,2,26.15409264,28.
570.15182,18226.32443,12.83928184,6.330057584,16.98654773,6.859172809,24,2,26.15409264,2
6570.15182,18226.32443,12.83928184,6.330057584,16.98654773,6.859172809,24,2,26.15409264,
68,1,1,1,1,0,12,16570.15182,18226.32443,12.83928184,6.330057584,16.98654773,6.859172809,24
6570.15182,18226.32443,12.83928184,6.330057584,16.98654773,6.859172809,24,2,26.15409264,
570.15182,18226.32443,12.83928184,6.330057584,16.98654773,6.859172809,24,2,26.15409264,2
570.15182,18226.32443,12.83928184,6.330057584,16.98654773,6.859172809,24,2,26.15409264,2
6570.15182,18226.32443,12.83928184,6.330057584,16.98654773,6.859172809,24,2,26.15409264,
68,1,1,1,1,0,12,16570.15182,18226.32443,12.83928184,6.330057584,16.98654773,6.859172809,24
6570.15182,18226.32443,12.83928184,6.330057584,16.98654773,6.859172809,24,2,26.15409264,
570.15182,18226.32443,12.83928184,6.330057584,16.98654773,6.859172809,24,2,26.15409264,2
570.15182,18226.32443,12.83928184,6.330057584,16.98654773,6.859172809,24,2,26.15409264,2
6570.15182,18226.32443,12.83928184,6.330057584,16.98654773,6.859172809,24,2,26.15409264,
184,6.330057584,16.98654773,6.859172809,24,2,26.15409264,28.48525913,128.6028974,182.127
570.15182,18226.32443,12.83928184,6.330057584,16.98654773,6.859172809,24,2,26.15409264,2
6570.15182,18226.32443,12.83928184,6.330057584,16.98654773,6.859172809,24,2,26.15409264,
70.15182,18226.32443,12.83928184,6.330057584,16.98654773,6.859172809,24,2,26.15409264,28.
6570.15182,18226.32443,12.83928184,6.330057584,16.98654773,6.859172809,24,2,26.15409264,
6570.15182,18226.32443,12.83928184,6.330057584,16.98654773,6.859172809,24,2,26.15409264,
6570.15182,18226.32443,12.83928184,6.330057584,16.98654773,6.859172809,24,2,26.15409264,
570.15182,18226.32443,12.83928184,6.330057584,16.98654773,6.859172809,24,2,26.15409264,2
8,1,1,1,1,0,12,16570.15182,18226.32443,12.83928184,6.330057584,16.98654773,6.859172809,24,
6570.15182,18226.32443,12.83928184,6.330057584,16.98654773,6.859172809,24,2,26.15409264,
6570.15182,18226.32443,12.83928184,6.330057584,16.98654773,6.859172809,24,2,26.15409264,
570.15182,18226.32443,12.83928184,6.330057584,16.98654773,6.859172809,24,2,26.15409264,2
826,8.079282874,16.40172211,6.859172809,21,2,23.93043651,40.39641437,216.3905069,179.998
6906.60888,18676.31681,11.96521826,8.079282874,16.40172211,6.859172809,21,2,23.93043651,
826,8.079282874,16.40172211,6.859172809,21,2,23.93043651,40.39641437,216.3905069,179.998
6906.60888,18676.31681,11.96521826,8.079282874,16.40172211,6.859172809,21,2,23.93043651,
906.60888,18676.31681,11.96521826,8.079282874,16.40172211,6.859172809,21,2,23.93043651,4
906.60888,18676.31681,11.96521826,8.079282874,16.40172211,6.859172809,21,2,23.93043651,4
6906.60888,18676.31681,11.96521826,8.079282874,16.40172211,6.859172809,21,2,23.93043651,

906.60888,18676.31681,11.96521826,8.079282874,16.40172211,6.859172809,21,2,23.93043651,40
6906.60888,18676.31681,11.96521826,8.079282874,16.40172211,6.859172809,21,2,23.93043651,4
906.60888,18676.31681,11.96521826,8.079282874,16.40172211,6.859172809,21,2,23.93043651,40
84,1,1,1,1,0,12,16906.60888,18676.31681,11.96521826,8.079282874,16.40172211,6.859172809,21
6906.60888,18676.31681,11.96521826,8.079282874,16.40172211,6.859172809,21,2,23.93043651,4
84,1,1,1,1,0,12,16906.60888,18676.31681,11.96521826,8.079282874,16.40172211,6.859172809,21
21826,8.079282874,16.40172211,6.859172809,21,2,23.93043651,40.39641437,216.3905069,179.99
906.60888,18676.31681,11.96521826,8.079282874,16.40172211,6.859172809,21,2,23.93043651,40
50308838,3,2,205,84,1,1,1,1,0,12,16906.60888,18676.31681,11.96521826,8.079282874,16.4017221
84,1,1,1,1,0,12,16906.60888,18676.31681,11.96521826,8.079282874,16.40172211,6.859172809,21
6906.60888,18676.31681,11.96521826,8.079282874,16.40172211,6.859172809,21,2,23.93043651,4
6906.60888,18676.31681,11.96521826,8.079282874,16.40172211,6.859172809,21,2,23.93043651,4
16906.60888,18676.31681,11.96521826,8.079282874,16.40172211,6.859172809,21,2,23.93043651
6906.60888,18676.31681,11.96521826,8.079282874,16.40172211,6.859172809,21,2,23.93043651,4
906.60888,18676.31681,11.96521826,8.079282874,16.40172211,6.859172809,21,2,23.93043651,40
906.60888,18676.31681,11.96521826,8.079282874,16.40172211,6.859172809,21,2,23.93043651,40
906.60888,18676.31681,11.96521826,8.079282874,16.40172211,6.859172809,21,2,23.93043651,40
906.60888,18676.31681,11.96521826,8.079282874,16.40172211,6.859172809,21,2,23.93043651,40
6906.60888,18676.31681,11.96521826,8.079282874,16.40172211,6.859172809,21,2,23.93043651,4
1826,8.079282874,16.40172211,6.859172809,21,2,23.93043651,40.39641437,216.3905069,179.998
6906.60888,18676.31681,11.96521826,8.079282874,16.40172211,6.859172809,21,2,23.93043651,4
84,1,1,1,1,0,12,16906.60888,18676.31681,11.96521826,8.079282874,16.40172211,6.859172809,21
6906.60888,18676.31681,11.96521826,8.079282874,16.40172211,6.859172809,21,2,23.93043651,4
6906.60888,18676.31681,11.96521826,8.079282874,16.40172211,6.859172809,21,2,23.93043651,4
84,1,1,1,1,0,12,16906.60888,18676.31681,11.96521826,8.079282874,16.40172211,6.859172809,21
906.60888,18676.31681,11.96521826,8.079282874,16.40172211,6.859172809,21,2,23.93043651,40
6906.60888,18676.31681,11.96521826,8.079282874,16.40172211,6.859172809,21,2,23.93043651,4
84,1,1,1,1,0,12,16906.60888,18676.31681,11.96521826,8.079282874,16.40172211,6.859172809,21
98522,5.76184119,11.32358648,6.263850985,16,1,23.04799652,46.09472952,96.85805408,179.774
6789.15885,21002.09294,9.795398522,5.76184119,11.32358648,6.263850985,16,1,23.04799652,46
85,0,1,0,1,0,10,16789.15885,21002.09294,9.795398522,5.76184119,11.32358648,6.263850985,16,1
8522,5.76184119,11.32358648,6.263850985,16,1,23.04799652,46.09472952,96.85805408,179.7743
789.15885,21002.09294,9.795398522,5.76184119,11.32358648,6.263850985,16,1,23.04799652,46.
89.15885,21002.09294,9.795398522,5.76184119,11.32358648,6.263850985,16,1,23.04799652,46.0
6789.15885,21002.09294,9.795398522,5.76184119,11.32358648,6.263850985,16,1,23.04799652,46
16789.15885,21002.09294,9.795398522,5.76184119,11.32358648,6.263850985,16,1,23.04799652,4
789.15885,21002.09294,9.795398522,5.76184119,11.32358648,6.263850985,16,1,23.04799652,46.
6789.15885,21002.09294,9.795398522,5.76184119,11.32358648,6.263850985,16,1,23.04799652,46
89.15885,21002.09294,9.795398522,5.76184119,11.32358648,6.263850985,16,1,23.04799652,46.0
85,0,1,0,1,0,10,16789.15885,21002.09294,9.795398522,5.76184119,11.32358648,6.263850985,16,1
6789.15885,21002.09294,9.795398522,5.76184119,11.32358648,6.263850985,16,1,23.04799652,46
6789.15885,21002.09294,9.795398522,5.76184119,11.32358648,6.263850985,16,1,23.04799652,46
6789.15885,21002.09294,9.795398522,5.76184119,11.32358648,6.263850985,16,1,23.04799652,46
85,0,1,0,1,0,10,16789.15885,21002.09294,9.795398522,5.76184119,11.32358648,6.263850985,16,1
85,0,1,0,1,0,10,16789.15885,21002.09294,9.795398522,5.76184119,11.32358648,6.263850985,16,1
8522,5.76184119,11.32358648,6.263850985,16,1,23.04799652,46.09472952,96.85805408,179.7743
789.15885,21002.09294,9.795398522,5.76184119,11.32358648,6.263850985,16,1,23.04799652,46.
85,0,1,0,1,0,10,16789.15885,21002.09294,9.795398522,5.76184119,11.32358648,6.263850985,16,1
8522,5.76184119,11.32358648,6.263850985,16,1,23.04799652,46.09472952,96.85805408,179.7743
6789.15885,21002.09294,9.795398522,5.76184119,11.32358648,6.263850985,16,1,23.04799652,46

522,5.76184119,11.32358648,6.263850985,16,1,23.04799652,46.09472952,96.85805408,179.7743
6789.15885,21002.09294,9.795398522,5.76184119,11.32358648,6.263850985,16,1,23.04799652,4
6789.15885,21002.09294,9.795398522,5.76184119,11.32358648,6.263850985,16,1,23.04799652,4
789.15885,21002.09294,9.795398522,5.76184119,11.32358648,6.263850985,16,1,23.04799652,46.
789.15885,21002.09294,9.795398522,5.76184119,11.32358648,6.263850985,16,1,23.04799652,46.
85,0,1,0,1,0,10,16789.15885,21002.09294,9.795398522,5.76184119,11.32358648,6.263850985,16,
789.15885,21002.09294,9.795398522,5.76184119,11.32358648,6.263850985,16,1,23.04799652,46.
522,5.76184119,11.32358648,6.263850985,16,1,23.04799652,46.09472952,96.85805408,179.7743
16789.15885,21002.09294,9.795398522,5.76184119,11.32358648,6.263850985,16,1,23.04799652,
87,5.633136017,14.96332204,6.263850985,18,1,21.70403985,50.69822415,155.0975698,178.7727
87,5.633136017,14.96332204,6.263850985,18,1,21.70403985,50.69822415,155.0975698,178.7727
7192.51095,21541.38367,11.42317887,5.633136017,14.96332204,6.263850985,18,1,21.70403985,
92.51095,21541.38367,11.42317887,5.633136017,14.96332204,6.263850985,18,1,21.70403985,50.
92.51095,21541.38367,11.42317887,5.633136017,14.96332204,6.263850985,18,1,21.70403985,50.
2.51095,21541.38367,11.42317887,5.633136017,14.96332204,6.263850985,18,1,21.70403985,50.6
92.51095,21541.38367,11.42317887,5.633136017,14.96332204,6.263850985,18,1,21.70403985,50.
92.51095,21541.38367,11.42317887,5.633136017,14.96332204,6.263850985,18,1,21.70403985,50.
87,5.633136017,14.96332204,6.263850985,18,1,21.70403985,50.69822415,155.0975698,178.7727
,80,1,1,1,1,0,4,17192.51095,21541.38367,11.42317887,5.633136017,14.96332204,6.263850985,18
,1,1,1,1,0,4,17192.51095,21541.38367,11.42317887,5.633136017,14.96332204,6.263850985,18,1,
92.51095,21541.38367,11.42317887,5.633136017,14.96332204,6.263850985,18,1,21.70403985,50.
92.51095,21541.38367,11.42317887,5.633136017,14.96332204,6.263850985,18,1,21.70403985,50.
92.51095,21541.38367,11.42317887,5.633136017,14.96332204,6.263850985,18,1,21.70403985,50.
7192.51095,21541.38367,11.42317887,5.633136017,14.96332204,6.263850985,18,1,21.70403985,
2.51095,21541.38367,11.42317887,5.633136017,14.96332204,6.263850985,18,1,21.70403985,50.6
7192.51095,21541.38367,11.42317887,5.633136017,14.96332204,6.263850985,18,1,21.70403985,
7,5.633136017,14.96332204,6.263850985,18,1,21.70403985,50.69822415,155.0975698,178.77274
87,5.633136017,14.96332204,6.263850985,18,1,21.70403985,50.69822415,155.0975698,178.7727
87,5.633136017,14.96332204,6.263850985,18,1,21.70403985,50.69822415,155.0975698,178.7727
92.51095,21541.38367,11.42317887,5.633136017,14.96332204,6.263850985,18,1,21.70403985,50.
92.51095,21541.38367,11.42317887,5.633136017,14.96332204,6.263850985,18,1,21.70403985,50.
7192.51095,21541.38367,11.42317887,5.633136017,14.96332204,6.263850985,18,1,21.70403985,
,80,1,1,1,1,0,4,17192.51095,21541.38367,11.42317887,5.633136017,14.96332204,6.263850985,18
87,5.633136017,14.96332204,6.263850985,18,1,21.70403985,50.69822415,155.0975698,178.7727
92.51095,21541.38367,11.42317887,5.633136017,14.96332204,6.263850985,18,1,21.70403985,50.
92.51095,21541.38367,11.42317887,5.633136017,14.96332204,6.263850985,18,1,21.70403985,50.
92.51095,21541.38367,11.42317887,5.633136017,14.96332204,6.263850985,18,1,21.70403985,50.
92.51095,21541.38367,11.42317887,5.633136017,14.96332204,6.263850985,18,1,21.70403985,50.
87,5.633136017,14.96332204,6.263850985,18,1,21.70403985,50.69822415,155.0975698,178.7727
,80,1,1,1,1,0,4,17192.51095,21541.38367,11.42317887,5.633136017,14.96332204,6.263850985,18
192.51095,21541.38367,11.42317887,5.633136017,14.96332204,6.263850985,18,1,21.70403985,5
7192.51095,21541.38367,11.42317887,5.633136017,14.96332204,6.263850985,18,1,21.70403985,
87,5.633136017,14.96332204,6.263850985,18,1,21.70403985,50.69822415,155.0975698,178.7727
92.51095,21541.38367,11.42317887,5.633136017,14.96332204,6.263850985,18,1,21.70403985,50.
92.51095,21541.38367,11.42317887,5.633136017,14.96332204,6.263850985,18,1,21.70403985,50.
80,1,1,1,1,0,4,17192.51095,21541.38367,11.42317887,5.633136017,14.96332204,6.263850985,18,
192.51095,21541.38367,11.42317887,5.633136017,14.96332204,6.263850985,18,1,21.70403985,5
,80,1,1,1,1,0,4,17192.51095,21541.38367,11.42317887,5.633136017,14.96332204,6.263850985,18
571628,3.617275862,11.19528697,0.595321824,21,1,26.09888615,28.93820689,119.9372739,193.
5563.65478,15242.70854,12.50571628,3.617275862,11.19528697,0.595321824,21,1,26.09888615,

5563.65478,15242.70854,12.50571628,3.617275862,11.19528697,0.595321824,21,1,26.09888615,
563.65478,15242.70854,12.50571628,3.617275862,11.19528697,0.595321824,21,1,26.09888615,2
15563.65478,15242.70854,12.50571628,3.617275862,11.19528697,0.595321824,21,1,26.09888615
571628,3.617275862,11.19528697,0.595321824,21,1,26.09888615,28.93820689,119.9372739,193.5
15563.65478,15242.70854,12.50571628,3.617275862,11.19528697,0.595321824,21,1,26.09888615,
5563.65478,15242.70854,12.50571628,3.617275862,11.19528697,0.595321824,21,1,26.09888615,
563.65478,15242.70854,12.50571628,3.617275862,11.19528697,0.595321824,21,1,26.09888615,2
563.65478,15242.70854,12.50571628,3.617275862,11.19528697,0.595321824,21,1,26.09888615,2
528,3.617275862,11.19528697,0.595321824,21,1,26.09888615,28.93820689,119.9372739,193.5667
1628,3.617275862,11.19528697,0.595321824,21,1,26.09888615,28.93820689,119.9372739,193.566
,15563.65478,15242.70854,12.50571628,3.617275862,11.19528697,0.595321824,21,1,26.0988861
67,1,1,0,1,0,12,15563.65478,15242.70854,12.50571628,3.617275862,11.19528697,0.595321824,21
563.65478,15242.70854,12.50571628,3.617275862,11.19528697,0.595321824,21,1,26.09888615,2
571628,3.617275862,11.19528697,0.595321824,21,1,26.09888615,28.93820689,119.9372739,193.5
563.65478,15242.70854,12.50571628,3.617275862,11.19528697,0.595321824,21,1,26.09888615,2
5563.65478,15242.70854,12.50571628,3.617275862,11.19528697,0.595321824,21,1,26.09888615,
.50571628,3.617275862,11.19528697,0.595321824,21,1,26.09888615,28.93820689,119.9372739,1
15563.65478,15242.70854,12.50571628,3.617275862,11.19528697,0.595321824,21,1,26.09888615,
528,3.617275862,11.19528697,0.595321824,21,1,26.09888615,28.93820689,119.9372739,193.5667
563.65478,15242.70854,12.50571628,3.617275862,11.19528697,0.595321824,21,1,26.09888615,2
5563.65478,15242.70854,12.50571628,3.617275862,11.19528697,0.595321824,21,1,26.09888615,
5,67,1,1,0,1,0,12,15563.65478,15242.70854,12.50571628,3.617275862,11.19528697,0.595321824,2
5,67,1,1,0,1,0,12,15563.65478,15242.70854,12.50571628,3.617275862,11.19528697,0.595321824,2
5563.65478,15242.70854,12.50571628,3.617275862,11.19528697,0.595321824,21,1,26.09888615,
5563.65478,15242.70854,12.50571628,3.617275862,11.19528697,0.595321824,21,1,26.09888615,
563.65478,15242.70854,12.50571628,3.617275862,11.19528697,0.595321824,21,1,26.09888615,2
5,67,1,1,0,1,0,12,15563.65478,15242.70854,12.50571628,3.617275862,11.19528697,0.595321824,2
311261,10.39243,21.39276043,0.086522391,16,2,27.3479832,13.85657334,246.0326346,166.2757
15664.41972,14514.16229,12.03311261,10.39243,21.39276043,0.086522391,16,2,27.3479832,13.8
261,10.39243,21.39276043,0.086522391,16,2,27.3479832,13.85657334,246.0326346,166.2757379,
67,0,1,1,1,0,12,15664.41972,14514.16229,12.03311261,10.39243,21.39276043,0.086522391,16,2,2
12,15664.41972,14514.16229,12.03311261,10.39243,21.39276043,0.086522391,16,2,27.3479832,13
67,0,1,1,1,0,12,15664.41972,14514.16229,12.03311261,10.39243,21.39276043,0.086522391,16,2,2
664.41972,14514.16229,12.03311261,10.39243,21.39276043,0.086522391,16,2,27.3479832,13.856
1261,10.39243,21.39276043,0.086522391,16,2,27.3479832,13.85657334,246.0326346,166.2757379
664.41972,14514.16229,12.03311261,10.39243,21.39276043,0.086522391,16,2,27.3479832,13.856
5664.41972,14514.16229,12.03311261,10.39243,21.39276043,0.086522391,16,2,27.3479832,13.85
29,12.03311261,10.39243,21.39276043,0.086522391,16,2,27.3479832,13.85657334,246.0326346,1
5664.41972,14514.16229,12.03311261,10.39243,21.39276043,0.086522391,16,2,27.3479832,13.85
311261,10.39243,21.39276043,0.086522391,16,2,27.3479832,13.85657334,246.0326346,166.27573
664.41972,14514.16229,12.03311261,10.39243,21.39276043,0.086522391,16,2,27.3479832,13.856
664.41972,14514.16229,12.03311261,10.39243,21.39276043,0.086522391,16,2,27.3479832,13.856
15664.41972,14514.16229,12.03311261,10.39243,21.39276043,0.086522391,16,2,27.3479832,13.8
1261,10.39243,21.39276043,0.086522391,16,2,27.3479832,13.85657334,246.0326346,166.2757379
261,10.39243,21.39276043,0.086522391,16,2,27.3479832,13.85657334,246.0326346,166.2757379,
5664.41972,14514.16229,12.03311261,10.39243,21.39276043,0.086522391,16,2,27.3479832,13.85
664.41972,14514.16229,12.03311261,10.39243,21.39276043,0.086522391,16,2,27.3479832,13.856
5664.41972,14514.16229,12.03311261,10.39243,21.39276043,0.086522391,16,2,27.3479832,13.85
664.41972,14514.16229,12.03311261,10.39243,21.39276043,0.086522391,16,2,27.3479832,13.856
5664.41972,14514.16229,12.03311261,10.39243,21.39276043,0.086522391,16,2,27.3479832,13.85

5,67,0,1,1,1,0,12,15664.41972,14514.16229,12.03311261,10.39243,21.39276043,0.086522391,16,2,
1261,10.39243,21.39276043,0.086522391,16,2,27.3479832,13.85657334,246.0326346,166.2757379
36,17.32475742,10.16242805,0.630570036,16,4,24.47675765,0,468.6948576,191.0381085,203.5658
6211.61748,14435.82793,10.7458936,17.32475742,10.16242805,0.630570036,16,4,24.47675765,0,
458936,17.32475742,10.16242805,0.630570036,16,4,24.47675765,0,468.6948576,191.0381085,203
9382324,3,2,205,67,1,1,0,1,0,11,16211.61748,14435.82793,10.7458936,17.32475742,10.16242805,0
458936,17.32475742,10.16242805,0.630570036,16,4,24.47675765,0,468.6948576,191.0381085,203
67,1,1,0,1,0,11,16211.61748,14435.82793,10.7458936,17.32475742,10.16242805,0.630570036,16,4
6211.61748,14435.82793,10.7458936,17.32475742,10.16242805,0.630570036,16,4,24.47675765,0,
936,17.32475742,10.16242805,0.630570036,16,4,24.47675765,0,468.6948576,191.0381085,203.565
2370605,3,2,205,67,1,1,0,1,0,11,16211.61748,14435.82793,10.7458936,17.32475742,10.16242805,0
6211.61748,14435.82793,10.7458936,17.32475742,10.16242805,0.630570036,16,4,24.47675765,0,
67,1,1,0,1,0,11,16211.61748,14435.82793,10.7458936,17.32475742,10.16242805,0.630570036,16,4
6211.61748,14435.82793,10.7458936,17.32475742,10.16242805,0.630570036,16,4,24.47675765,0,
5,67,1,1,0,1,0,11,16211.61748,14435.82793,10.7458936,17.32475742,10.16242805,0.630570036,16
458936,17.32475742,10.16242805,0.630570036,16,4,24.47675765,0,468.6948576,191.0381085,203
6211.61748,14435.82793,10.7458936,17.32475742,10.16242805,0.630570036,16,4,24.47675765,0,
6211.61748,14435.82793,10.7458936,17.32475742,10.16242805,0.630570036,16,4,24.47675765,0,
211.61748,14435.82793,10.7458936,17.32475742,10.16242805,0.630570036,16,4,24.47675765,0,4
7,1,1,0,1,0,11,16211.61748,14435.82793,10.7458936,17.32475742,10.16242805,0.630570036,16,4,
.7458936,17.32475742,10.16242805,0.630570036,16,4,24.47675765,0,468.6948576,191.0381085,2
67,1,1,0,1,0,11,16211.61748,14435.82793,10.7458936,17.32475742,10.16242805,0.630570036,16,4
211.61748,14435.82793,10.7458936,17.32475742,10.16242805,0.630570036,16,4,24.47675765,0,4
6211.61748,14435.82793,10.7458936,17.32475742,10.16242805,0.630570036,16,4,24.47675765,0,
36,17.32475742,10.16242805,0.630570036,16,4,24.47675765,0,468.6948576,191.0381085,203.5658
6211.61748,14435.82793,10.7458936,17.32475742,10.16242805,0.630570036,16,4,24.47675765,0,
936,17.32475742,10.16242805,0.630570036,16,4,24.47675765,0,468.6948576,191.0381085,203.565
6211.61748,14435.82793,10.7458936,17.32475742,10.16242805,0.630570036,16,4,24.47675765,0,
6211.61748,14435.82793,10.7458936,17.32475742,10.16242805,0.630570036,16,4,24.47675765,0,
8936,17.32475742,10.16242805,0.630570036,16,4,24.47675765,0,468.6948576,191.0381085,203.56
6211.61748,14435.82793,10.7458936,17.32475742,10.16242805,0.630570036,16,4,24.47675765,0,
67,1,1,0,1,0,11,16211.61748,14435.82793,10.7458936,17.32475742,10.16242805,0.630570036,16,4
548,15.44770948,21.57066343,0.630570036,18,4,27.35359594,0,420.9833249,187.4861055,196.958
379.73288,15318.15979,12.5370648,15.44770948,21.57066343,0.630570036,18,4,27.35359594,0,4
6379.73288,15318.15979,12.5370648,15.44770948,21.57066343,0.630570036,18,4,27.35359594,0,
.5370648,15.44770948,21.57066343,0.630570036,18,4,27.35359594,0,420.9833249,187.4861055,1
16379.73288,15318.15979,12.5370648,15.44770948,21.57066343,0.630570036,18,4,27.35359594,0
370648,15.44770948,21.57066343,0.630570036,18,4,27.35359594,0,420.9833249,187.4861055,196
16379.73288,15318.15979,12.5370648,15.44770948,21.57066343,0.630570036,18,4,27.35359594,0
6379.73288,15318.15979,12.5370648,15.44770948,21.57066343,0.630570036,18,4,27.35359594,0,
370648,15.44770948,21.57066343,0.630570036,18,4,27.35359594,0,420.9833249,187.4861055,196
5,75,0,1,1,1,0,11,16379.73288,15318.15979,12.5370648,15.44770948,21.57066343,0.630570036,18
379.73288,15318.15979,12.5370648,15.44770948,21.57066343,0.630570036,18,4,27.35359594,0,4
379.73288,15318.15979,12.5370648,15.44770948,21.57066343,0.630570036,18,4,27.35359594,0,4
16379.73288,15318.15979,12.5370648,15.44770948,21.57066343,0.630570036,18,4,27.35359594,0
379.73288,15318.15979,12.5370648,15.44770948,21.57066343,0.630570036,18,4,27.35359594,0,4
.5370648,15.44770948,21.57066343,0.630570036,18,4,27.35359594,0,420.9833249,187.4861055,1
379.73288,15318.15979,12.5370648,15.44770948,21.57066343,0.630570036,18,4,27.35359594,0,4
548,15.44770948,21.57066343,0.630570036,18,4,27.35359594,0,420.9833249,187.4861055,196.958
379.73288,15318.15979,12.5370648,15.44770948,21.57066343,0.630570036,18,4,27.35359594,0,4

6379.73288,15318.15979,12.5370648,15.44770948,21.57066343,0.630570036,18,4,27.35359594,0,
548,15.44770948,21.57066343,0.630570036,18,4,27.35359594,0,420.9833249,187.4861055,196.958
370648,15.44770948,21.57066343,0.630570036,18,4,27.35359594,0,420.9833249,187.4861055,196
379.73288,15318.15979,12.5370648,15.44770948,21.57066343,0.630570036,18,4,27.35359594,0,4
370648,15.44770948,21.57066343,0.630570036,18,4,27.35359594,0,420.9833249,187.4861055,196
379.73288,15318.15979,12.5370648,15.44770948,21.57066343,0.630570036,18,4,27.35359594,0,4
548,15.44770948,21.57066343,0.630570036,18,4,27.35359594,0,420.9833249,187.4861055,196.958
379.73288,15318.15979,12.5370648,15.44770948,21.57066343,0.630570036,18,4,27.35359594,0,4
16379.73288,15318.15979,12.5370648,15.44770948,21.57066343,0.630570036,18,4,27.35359594,0
548,15.44770948,21.57066343,0.630570036,18,4,27.35359594,0,420.9833249,187.4861055,196.958
379.73288,15318.15979,12.5370648,15.44770948,21.57066343,0.630570036,18,4,27.35359594,0,4
379.73288,15318.15979,12.5370648,15.44770948,21.57066343,0.630570036,18,4,27.35359594,0,4
0648,15.44770948,21.57066343,0.630570036,18,4,27.35359594,0,420.9833249,187.4861055,196.95
2992,24.23299292,5.167747519,0.990207423,18,6,28.65357479,16.15532861,279.0357474,185.675
42992,24.23299292,5.167747519,0.990207423,18,6,28.65357479,16.15532861,279.0357474,185.67
16072.20968,15137.33475,11.46142992,24.23299292,5.167747519,0.990207423,18,6,28.65357479
5,76,0,1,0,1,0,12,16072.20968,15137.33475,11.46142992,24.23299292,5.167747519,0.990207423,1
2992,24.23299292,5.167747519,0.990207423,18,6,28.65357479,16.15532861,279.0357474,185.675
11.46142992,24.23299292,5.167747519,0.990207423,18,6,28.65357479,16.15532861,279.0357474,
76,0,1,0,1,0,12,16072.20968,15137.33475,11.46142992,24.23299292,5.167747519,0.990207423,18
072.20968,15137.33475,11.46142992,24.23299292,5.167747519,0.990207423,18,6,28.65357479,16
76,0,1,0,1,0,12,16072.20968,15137.33475,11.46142992,24.23299292,5.167747519,0.990207423,18
.46142992,24.23299292,5.167747519,0.990207423,18,6,28.65357479,16.15532861,279.0357474,18
072.20968,15137.33475,11.46142992,24.23299292,5.167747519,0.990207423,18,6,28.65357479,16
072.20968,15137.33475,11.46142992,24.23299292,5.167747519,0.990207423,18,6,28.65357479,16
6072.20968,15137.33475,11.46142992,24.23299292,5.167747519,0.990207423,18,6,28.65357479,1
76,0,1,0,1,0,12,16072.20968,15137.33475,11.46142992,24.23299292,5.167747519,0.990207423,18
16072.20968,15137.33475,11.46142992,24.23299292,5.167747519,0.990207423,18,6,28.65357479
142992,24.23299292,5.167747519,0.990207423,18,6,28.65357479,16.15532861,279.0357474,185.6
16072.20968,15137.33475,11.46142992,24.23299292,5.167747519,0.990207423,18,6,28.65357479
76,0,1,0,1,0,12,16072.20968,15137.33475,11.46142992,24.23299292,5.167747519,0.990207423,18
76,0,1,0,1,0,12,16072.20968,15137.33475,11.46142992,24.23299292,5.167747519,0.990207423,18
072.20968,15137.33475,11.46142992,24.23299292,5.167747519,0.990207423,18,6,28.65357479,16
5142992,24.23299292,5.167747519,0.990207423,18,6,28.65357479,16.15532861,279.0357474,185.
6072.20968,15137.33475,11.46142992,24.23299292,5.167747519,0.990207423,18,6,28.65357479,1
11.46142992,24.23299292,5.167747519,0.990207423,18,6,28.65357479,16.15532861,279.0357474,
11.46142992,24.23299292,5.167747519,0.990207423,18,6,28.65357479,16.15532861,279.0357474,
16072.20968,15137.33475,11.46142992,24.23299292,5.167747519,0.990207423,18,6,28.65357479
5142992,24.23299292,5.167747519,0.990207423,18,6,28.65357479,16.15532861,279.0357474,185.
76,0,1,0,1,0,12,16072.20968,15137.33475,11.46142992,24.23299292,5.167747519,0.990207423,18
0617676,3,2,205,76,0,1,0,1,0,12,16072.20968,15137.33475,11.46142992,24.23299292,5.167747519
072.20968,15137.33475,11.46142992,24.23299292,5.167747519,0.990207423,18,6,28.65357479,16
2992,24.23299292,5.167747519,0.990207423,18,6,28.65357479,16.15532861,279.0357474,185.675
3,24.37112408,5.167747519,0.990207423,18,6,28.92076699,16.24741605,267.516328,187.4065701
33068,24.37112408,5.167747519,0.990207423,18,6,28.92076699,16.24741605,267.516328,187.406
33068,24.37112408,5.167747519,0.990207423,18,6,28.92076699,16.24741605,267.516328,187.406
1.5683068,24.37112408,5.167747519,0.990207423,18,6,28.92076699,16.24741605,267.516328,187
65,1,1,0,1,0,11,16105.48561,15129.5483,11.5683068,24.37112408,5.167747519,0.990207423,18,6,
65,1,1,0,1,0,11,16105.48561,15129.5483,11.5683068,24.37112408,5.167747519,0.990207423,18,6,
16105.48561,15129.5483,11.5683068,24.37112408,5.167747519,0.990207423,18,6,28.92076699,16

3,24.37112408,5.167747519,0.990207423,18,6,28.92076699,16.24741605,267.516328,187.4065701
5,65,1,1,0,1,0,11,16105.48561,15129.5483,11.5683068,24.37112408,5.167747519,0.990207423,18,
5,65,1,1,0,1,0,11,16105.48561,15129.5483,11.5683068,24.37112408,5.167747519,0.990207423,18,
6105.48561,15129.5483,11.5683068,24.37112408,5.167747519,0.990207423,18,6,28.92076699,16.
6105.48561,15129.5483,11.5683068,24.37112408,5.167747519,0.990207423,18,6,28.92076699,16.
4.37112408,5.167747519,0.990207423,18,6,28.92076699,16.24741605,267.516328,187.4065701,2
33068,24.37112408,5.167747519,0.990207423,18,6,28.92076699,16.24741605,267.516328,187.406
33068,24.37112408,5.167747519,0.990207423,18,6,28.92076699,16.24741605,267.516328,187.406
58,24.37112408,5.167747519,0.990207423,18,6,28.92076699,16.24741605,267.516328,187.406570
5,65,1,1,0,1,0,11,16105.48561,15129.5483,11.5683068,24.37112408,5.167747519,0.990207423,18,
33068,24.37112408,5.167747519,0.990207423,18,6,28.92076699,16.24741605,267.516328,187.406
6105.48561,15129.5483,11.5683068,24.37112408,5.167747519,0.990207423,18,6,28.92076699,16.
5,65,1,1,0,1,0,11,16105.48561,15129.5483,11.5683068,24.37112408,5.167747519,0.990207423,18,
6105.48561,15129.5483,11.5683068,24.37112408,5.167747519,0.990207423,18,6,28.92076699,16.
6105.48561,15129.5483,11.5683068,24.37112408,5.167747519,0.990207423,18,6,28.92076699,16.
33068,24.37112408,5.167747519,0.990207423,18,6,28.92076699,16.24741605,267.516328,187.406
6105.48561,15129.5483,11.5683068,24.37112408,5.167747519,0.990207423,18,6,28.92076699,16.
6105.48561,15129.5483,11.5683068,24.37112408,5.167747519,0.990207423,18,6,28.92076699,16.
65,1,1,0,1,0,11,16105.48561,15129.5483,11.5683068,24.37112408,5.167747519,0.990207423,18,6,
58,24.37112408,5.167747519,0.990207423,18,6,28.92076699,16.24741605,267.516328,187.406570
58,24.37112408,5.167747519,0.990207423,18,6,28.92076699,16.24741605,267.516328,187.406570
105.48561,15129.5483,11.5683068,24.37112408,5.167747519,0.990207423,18,6,28.92076699,16.2
6105.48561,15129.5483,11.5683068,24.37112408,5.167747519,0.990207423,18,6,28.92076699,16.
1.5683068,24.37112408,5.167747519,0.990207423,18,6,28.92076699,16.24741605,267.516328,187
105.48561,15129.5483,11.5683068,24.37112408,5.167747519,0.990207423,18,6,28.92076699,16.2
33068,24.37112408,5.167747519,0.990207423,18,6,28.92076699,16.24741605,267.516328,187.406
6105.48561,15129.5483,11.5683068,24.37112408,5.167747519,0.990207423,18,6,28.92076699,16.
8,0,1,0,1,0,11,16073.21775,15388.50828,12.21612117,20.5623614,5.218287908,0.842449737,19,5,
16073.21775,15388.50828,12.21612117,20.5623614,5.218287908,0.842449737,19,5,29.08600279,
073.21775,15388.50828,12.21612117,20.5623614,5.218287908,0.842449737,19,5,29.08600279,16.
073.21775,15388.50828,12.21612117,20.5623614,5.218287908,0.842449737,19,5,29.08600279,16.
2117,20.5623614,5.218287908,0.842449737,19,5,29.08600279,16.44988912,250.7673265,186.7321
5,68,0,1,0,1,0,11,16073.21775,15388.50828,12.21612117,20.5623614,5.218287908,0.842449737,19
6073.21775,15388.50828,12.21612117,20.5623614,5.218287908,0.842449737,19,5,29.08600279,1
6073.21775,15388.50828,12.21612117,20.5623614,5.218287908,0.842449737,19,5,29.08600279,1
073.21775,15388.50828,12.21612117,20.5623614,5.218287908,0.842449737,19,5,29.08600279,16.
073.21775,15388.50828,12.21612117,20.5623614,5.218287908,0.842449737,19,5,29.08600279,16.
073.21775,15388.50828,12.21612117,20.5623614,5.218287908,0.842449737,19,5,29.08600279,16.
16073.21775,15388.50828,12.21612117,20.5623614,5.218287908,0.842449737,19,5,29.08600279,
5,68,0,1,0,1,0,11,16073.21775,15388.50828,12.21612117,20.5623614,5.218287908,0.842449737,19
6073.21775,15388.50828,12.21612117,20.5623614,5.218287908,0.842449737,19,5,29.08600279,1
6073.21775,15388.50828,12.21612117,20.5623614,5.218287908,0.842449737,19,5,29.08600279,1
2117,20.5623614,5.218287908,0.842449737,19,5,29.08600279,16.44988912,250.7673265,186.7321
73.21775,15388.50828,12.21612117,20.5623614,5.218287908,0.842449737,19,5,29.08600279,16.4
6073.21775,15388.50828,12.21612117,20.5623614,5.218287908,0.842449737,19,5,29.08600279,1
6073.21775,15388.50828,12.21612117,20.5623614,5.218287908,0.842449737,19,5,29.08600279,1
6073.21775,15388.50828,12.21612117,20.5623614,5.218287908,0.842449737,19,5,29.08600279,1
76285,10.43569581,21.39276043,0.086522391,16,2,27.76663206,13.91426107,247.2133129,166.59
68,0,1,0,0,0,12,15783.45732,14399.01356,11.97776285,10.43569581,21.39276043,0.086522391,16
5783.45732,14399.01356,11.97776285,10.43569581,21.39276043,0.086522391,16,2,27.76663206,

68,0,1,0,0,0,12,15783.45732,14399.01356,11.97776285,10.43569581,21.39276043,0.086522391,16
68,0,1,0,0,0,12,15783.45732,14399.01356,11.97776285,10.43569581,21.39276043,0.086522391,16
8,0,1,0,0,0,12,15783.45732,14399.01356,11.97776285,10.43569581,21.39276043,0.086522391,16,
783.45732,14399.01356,11.97776285,10.43569581,21.39276043,0.086522391,16,2,27.76663206,1
76285,10.43569581,21.39276043,0.086522391,16,2,27.76663206,13.91426107,247.2133129,166.59
783.45732,14399.01356,11.97776285,10.43569581,21.39276043,0.086522391,16,2,27.76663206,1
83.45732,14399.01356,11.97776285,10.43569581,21.39276043,0.086522391,16,2,27.76663206,13.
783.45732,14399.01356,11.97776285,10.43569581,21.39276043,0.086522391,16,2,27.76663206,1
5783.45732,14399.01356,11.97776285,10.43569581,21.39276043,0.086522391,16,2,27.76663206,
5783.45732,14399.01356,11.97776285,10.43569581,21.39276043,0.086522391,16,2,27.76663206,
68,0,1,0,0,0,12,15783.45732,14399.01356,11.97776285,10.43569581,21.39276043,0.086522391,16
49227905,3,2,205,68,0,1,0,0,0,12,15783.45732,14399.01356,11.97776285,10.43569581,21.3927604
5285,10.43569581,21.39276043,0.086522391,16,2,27.76663206,13.91426107,247.2133129,166.599
5285,10.43569581,21.39276043,0.086522391,16,2,27.76663206,13.91426107,247.2133129,166.599
783.45732,14399.01356,11.97776285,10.43569581,21.39276043,0.086522391,16,2,27.76663206,1
5285,10.43569581,21.39276043,0.086522391,16,2,27.76663206,13.91426107,247.2133129,166.599
68,0,1,0,0,0,12,15783.45732,14399.01356,11.97776285,10.43569581,21.39276043,0.086522391,16
783.45732,14399.01356,11.97776285,10.43569581,21.39276043,0.086522391,16,2,27.76663206,1
68,0,1,0,0,0,12,15783.45732,14399.01356,11.97776285,10.43569581,21.39276043,0.086522391,16
68,0,1,0,0,0,12,15783.45732,14399.01356,11.97776285,10.43569581,21.39276043,0.086522391,16
68,0,1,0,0,0,12,15783.45732,14399.01356,11.97776285,10.43569581,21.39276043,0.086522391,16
5783.45732,14399.01356,11.97776285,10.43569581,21.39276043,0.086522391,16,2,27.76663206,
5783.45732,14399.01356,11.97776285,10.43569581,21.39276043,0.086522391,16,2,27.76663206,
68,0,1,0,0,0,12,15783.45732,14399.01356,11.97776285,10.43569581,21.39276043,0.086522391,16
68,0,1,0,0,0,12,15783.45732,14399.01356,11.97776285,10.43569581,21.39276043,0.086522391,16
783.45732,14399.01356,11.97776285,10.43569581,21.39276043,0.086522391,16,2,27.76663206,1
68,0,1,0,0,0,12,15783.45732,14399.01356,11.97776285,10.43569581,21.39276043,0.086522391,16
68,0,1,0,0,0,12,15783.45732,14399.01356,11.97776285,10.43569581,21.39276043,0.086522391,16
5783.45732,14399.01356,11.97776285,10.43569581,21.39276043,0.086522391,16,2,27.76663206,
68,0,1,0,0,0,12,15783.45732,14399.01356,11.97776285,10.43569581,21.39276043,0.086522391,16
5191,20.69349627,5.008486053,0.842449737,17,5,29.37190797,20.69349627,242.6318631,186.206
75,1,1,1,0,0,12,16188.31984,14448.61268,10.52955191,20.69349627,5.008486053,0.842449737,17
6188.31984,14448.61268,10.52955191,20.69349627,5.008486053,0.842449737,17,5,29.37190797,
6188.31984,14448.61268,10.52955191,20.69349627,5.008486053,0.842449737,17,5,29.37190797,
188.31984,14448.61268,10.52955191,20.69349627,5.008486053,0.842449737,17,5,29.37190797,2
6188.31984,14448.61268,10.52955191,20.69349627,5.008486053,0.842449737,17,5,29.37190797,
6188.31984,14448.61268,10.52955191,20.69349627,5.008486053,0.842449737,17,5,29.37190797,
188.31984,14448.61268,10.52955191,20.69349627,5.008486053,0.842449737,17,5,29.37190797,2
16188.31984,14448.61268,10.52955191,20.69349627,5.008486053,0.842449737,17,5,29.37190797
5,1,1,1,0,0,12,16188.31984,14448.61268,10.52955191,20.69349627,5.008486053,0.842449737,17,
6188.31984,14448.61268,10.52955191,20.69349627,5.008486053,0.842449737,17,5,29.37190797,
188.31984,14448.61268,10.52955191,20.69349627,5.008486053,0.842449737,17,5,29.37190797,2
75,1,1,1,0,0,12,16188.31984,14448.61268,10.52955191,20.69349627,5.008486053,0.842449737,17
5191,20.69349627,5.008486053,0.842449737,17,5,29.37190797,20.69349627,242.6318631,186.206
188.31984,14448.61268,10.52955191,20.69349627,5.008486053,0.842449737,17,5,29.37190797,2
191,20.69349627,5.008486053,0.842449737,17,5,29.37190797,20.69349627,242.6318631,186.2068
188.31984,14448.61268,10.52955191,20.69349627,5.008486053,0.842449737,17,5,29.37190797,2
188.31984,14448.61268,10.52955191,20.69349627,5.008486053,0.842449737,17,5,29.37190797,2
6188.31984,14448.61268,10.52955191,20.69349627,5.008486053,0.842449737,17,5,29.37190797,
188.31984,14448.61268,10.52955191,20.69349627,5.008486053,0.842449737,17,5,29.37190797,2

75,1,1,1,0,0,12,16188.31984,14448.61268,10.52955191,20.69349627,5.008486053,0.842449737,17
6188.31984,14448.61268,10.52955191,20.69349627,5.008486053,0.842449737,17,5,29.37190797,2
6188.31984,14448.61268,10.52955191,20.69349627,5.008486053,0.842449737,17,5,29.37190797,2
188.31984,14448.61268,10.52955191,20.69349627,5.008486053,0.842449737,17,5,29.37190797,20
6188.31984,14448.61268,10.52955191,20.69349627,5.008486053,0.842449737,17,5,29.37190797,2
6188.31984,14448.61268,10.52955191,20.69349627,5.008486053,0.842449737,17,5,29.37190797,2
75,1,1,1,0,0,12,16188.31984,14448.61268,10.52955191,20.69349627,5.008486053,0.842449737,17
188.31984,14448.61268,10.52955191,20.69349627,5.008486053,0.842449737,17,5,29.37190797,20
188.31984,14448.61268,10.52955191,20.69349627,5.008486053,0.842449737,17,5,29.37190797,20
49227905,3,2,205,75,1,1,1,0,0,12,16188.31984,14448.61268,10.52955191,20.69349627,5.00848605
75,1,1,1,0,0,12,16188.31984,14448.61268,10.52955191,20.69349627,5.008486053,0.842449737,17
6188.31984,14448.61268,10.52955191,20.69349627,5.008486053,0.842449737,17,5,29.37190797,2
6188.31984,14448.61268,10.52955191,20.69349627,5.008486053,0.842449737,17,5,29.37190797,2
188.31984,14448.61268,10.52955191,20.69349627,5.008486053,0.842449737,17,5,29.37190797,20
7,7.322181207,12.38386352,0.26757663,21,2,27.29679397,7.322181207,337.5424955,197.9017563
87,1,1,1,1,0,10,15990.01803,14461.79292,12.51103057,7.322181207,12.38386352,0.26757663,21,2
5990.01803,14461.79292,12.51103057,7.322181207,12.38386352,0.26757663,21,2,27.29679397,7.
990.01803,14461.79292,12.51103057,7.322181207,12.38386352,0.26757663,21,2,27.29679397,7.3
1103057,7.322181207,12.38386352,0.26757663,21,2,27.29679397,7.322181207,337.5424955,197.9
1103057,7.322181207,12.38386352,0.26757663,21,2,27.29679397,7.322181207,337.5424955,197.9
990.01803,14461.79292,12.51103057,7.322181207,12.38386352,0.26757663,21,2,27.29679397,7.3
990.01803,14461.79292,12.51103057,7.322181207,12.38386352,0.26757663,21,2,27.29679397,7.3
103057,7.322181207,12.38386352,0.26757663,21,2,27.29679397,7.322181207,337.5424955,197.90
057,7.322181207,12.38386352,0.26757663,21,2,27.29679397,7.322181207,337.5424955,197.90175
990.01803,14461.79292,12.51103057,7.322181207,12.38386352,0.26757663,21,2,27.29679397,7.3
5,87,1,1,1,1,0,10,15990.01803,14461.79292,12.51103057,7.322181207,12.38386352,0.26757663,21
057,7.322181207,12.38386352,0.26757663,21,2,27.29679397,7.322181207,337.5424955,197.90175
5990.01803,14461.79292,12.51103057,7.322181207,12.38386352,0.26757663,21,2,27.29679397,7.
5990.01803,14461.79292,12.51103057,7.322181207,12.38386352,0.26757663,21,2,27.29679397,7.
15990.01803,14461.79292,12.51103057,7.322181207,12.38386352,0.26757663,21,2,27.29679397,7
3057,7.322181207,12.38386352,0.26757663,21,2,27.29679397,7.322181207,337.5424955,197.9017
292,12.51103057,7.322181207,12.38386352,0.26757663,21,2,27.29679397,7.322181207,337.54249
057,7.322181207,12.38386352,0.26757663,21,2,27.29679397,7.322181207,337.5424955,197.90175
990.01803,14461.79292,12.51103057,7.322181207,12.38386352,0.26757663,21,2,27.29679397,7.3
51103057,7.322181207,12.38386352,0.26757663,21,2,27.29679397,7.322181207,337.5424955,197.
56097,7.653558774,12.00442647,0.26757663,20,2,26.92128885,7.653558774,351.5573886,197.613
028.75751,14564.01996,12.02866097,7.653558774,12.00442647,0.26757663,20,2,26.92128885,7.6
87,0,1,1,0,0,12,16028.75751,14564.01996,12.02866097,7.653558774,12.00442647,0.26757663,20,2
6028.75751,14564.01996,12.02866097,7.653558774,12.00442647,0.26757663,20,2,26.92128885,7.
028.75751,14564.01996,12.02866097,7.653558774,12.00442647,0.26757663,20,2,26.92128885,7.6
28.75751,14564.01996,12.02866097,7.653558774,12.00442647,0.26757663,20,2,26.92128885,7.65
028.75751,14564.01996,12.02866097,7.653558774,12.00442647,0.26757663,20,2,26.92128885,7.6
16028.75751,14564.01996,12.02866097,7.653558774,12.00442647,0.26757663,20,2,26.92128885,7
87,0,1,1,0,0,12,16028.75751,14564.01996,12.02866097,7.653558774,12.00442647,0.26757663,20,2
16028.75751,14564.01996,12.02866097,7.653558774,12.00442647,0.26757663,20,2,26.92128885,7
028.75751,14564.01996,12.02866097,7.653558774,12.00442647,0.26757663,20,2,26.92128885,7.6
6028.75751,14564.01996,12.02866097,7.653558774,12.00442647,0.26757663,20,2,26.92128885,7.
87,0,1,1,0,0,12,16028.75751,14564.01996,12.02866097,7.653558774,12.00442647,0.26757663,20,2
87,0,1,1,0,0,12,16028.75751,14564.01996,12.02866097,7.653558774,12.00442647,0.26757663,20,2
87,0,1,1,0,0,12,16028.75751,14564.01996,12.02866097,7.653558774,12.00442647,0.26757663,20,2

28.75751,14564.01996,12.02866097,7.653558774,12.00442647,0.26757663,20,2,26.92128885,7.65
6028.75751,14564.01996,12.02866097,7.653558774,12.00442647,0.26757663,20,2,26.92128885,7.
6028.75751,14564.01996,12.02866097,7.653558774,12.00442647,0.26757663,20,2,26.92128885,7.
5097,7.653558774,12.00442647,0.26757663,20,2,26.92128885,7.653558774,351.5573886,197.6137
028.75751,14564.01996,12.02866097,7.653558774,12.00442647,0.26757663,20,2,26.92128885,7.6
028.75751,14564.01996,12.02866097,7.653558774,12.00442647,0.26757663,20,2,26.92128885,7.6
6028.75751,14564.01996,12.02866097,7.653558774,12.00442647,0.26757663,20,2,26.92128885,7.
87,0,1,1,0,0,12,16028.75751,14564.01996,12.02866097,7.653558774,12.00442647,0.26757663,20,
6028.75751,14564.01996,12.02866097,7.653558774,12.00442647,0.26757663,20,2,26.92128885,7.
6028.75751,14564.01996,12.02866097,7.653558774,12.00442647,0.26757663,20,2,26.92128885,7.
87,0,1,1,0,0,12,16028.75751,14564.01996,12.02866097,7.653558774,12.00442647,0.26757663,20,
6028.75751,14564.01996,12.02866097,7.653558774,12.00442647,0.26757663,20,2,26.92128885,7.
6028.75751,14564.01996,12.02866097,7.653558774,12.00442647,0.26757663,20,2,26.92128885,7.
6028.75751,14564.01996,12.02866097,7.653558774,12.00442647,0.26757663,20,2,26.92128885,7.
6028.75751,14564.01996,12.02866097,7.653558774,12.00442647,0.26757663,20,2,26.92128885,7.
9845581,3,2,205,87,0,1,1,0,0,12,16028.75751,14564.01996,12.02866097,7.653558774,12.00442647
6028.75751,14564.01996,12.02866097,7.653558774,12.00442647,0.26757663,20,2,26.92128885,7.
87,0,1,1,0,0,12,16028.75751,14564.01996,12.02866097,7.653558774,12.00442647,0.26757663,20,
5097,7.653558774,12.00442647,0.26757663,20,2,26.92128885,7.653558774,351.5573886,197.6137
5,87,0,1,1,0,0,12,16028.75751,14564.01996,12.02866097,7.653558774,12.00442647,0.26757663,20
5978,5.639248314,16.02121995,6.263850985,18,1,23.33240256,33.83548988,74.49842839,177.553
6602.24814,20012.11008,11.38165978,5.639248314,16.02121995,6.263850985,18,1,23.33240256,
6602.24814,20012.11008,11.38165978,5.639248314,16.02121995,6.263850985,18,1,23.33240256,
70,0,0,0,1,0,11,16602.24814,20012.11008,11.38165978,5.639248314,16.02121995,6.263850985,18
70,0,0,0,1,0,11,16602.24814,20012.11008,11.38165978,5.639248314,16.02121995,6.263850985,18
6602.24814,20012.11008,11.38165978,5.639248314,16.02121995,6.263850985,18,1,23.33240256,
70,0,0,0,1,0,11,16602.24814,20012.11008,11.38165978,5.639248314,16.02121995,6.263850985,18
6602.24814,20012.11008,11.38165978,5.639248314,16.02121995,6.263850985,18,1,23.33240256,
70,0,0,0,1,0,11,16602.24814,20012.11008,11.38165978,5.639248314,16.02121995,6.263850985,18
6602.24814,20012.11008,11.38165978,5.639248314,16.02121995,6.263850985,18,1,23.33240256,
602.24814,20012.11008,11.38165978,5.639248314,16.02121995,6.263850985,18,1,23.33240256,33
6602.24814,20012.11008,11.38165978,5.639248314,16.02121995,6.263850985,18,1,23.33240256,
602.24814,20012.11008,11.38165978,5.639248314,16.02121995,6.263850985,18,1,23.33240256,33
70,0,0,0,1,0,11,16602.24814,20012.11008,11.38165978,5.639248314,16.02121995,6.263850985,18
6602.24814,20012.11008,11.38165978,5.639248314,16.02121995,6.263850985,18,1,23.33240256,
602.24814,20012.11008,11.38165978,5.639248314,16.02121995,6.263850985,18,1,23.33240256,33
602.24814,20012.11008,11.38165978,5.639248314,16.02121995,6.263850985,18,1,23.33240256,33
70,0,0,0,1,0,11,16602.24814,20012.11008,11.38165978,5.639248314,16.02121995,6.263850985,18
70,0,0,0,1,0,11,16602.24814,20012.11008,11.38165978,5.639248314,16.02121995,6.263850985,18
5,70,0,0,0,1,0,11,16602.24814,20012.11008,11.38165978,5.639248314,16.02121995,6.263850985,1
6602.24814,20012.11008,11.38165978,5.639248314,16.02121995,6.263850985,18,1,23.33240256,
6602.24814,20012.11008,11.38165978,5.639248314,16.02121995,6.263850985,18,1,23.33240256,
16602.24814,20012.11008,11.38165978,5.639248314,16.02121995,6.263850985,18,1,23.33240256
6602.24814,20012.11008,11.38165978,5.639248314,16.02121995,6.263850985,18,1,23.33240256,
55978,5.639248314,16.02121995,6.263850985,18,1,23.33240256,33.83548988,74.49842839,177.55
6602.24814,20012.11008,11.38165978,5.639248314,16.02121995,6.263850985,18,1,23.33240256,
602.24814,20012.11008,11.38165978,5.639248314,16.02121995,6.263850985,18,1,23.33240256,33
602.24814,20012.11008,11.38165978,5.639248314,16.02121995,6.263850985,18,1,23.33240256,33
20617676,3,2,205,70,0,0,0,1,0,11,16602.24814,20012.11008,11.38165978,5.639248314,16.0212199
16602.24814,20012.11008,11.38165978,5.639248314,16.02121995,6.263850985,18,1,23.33240256

6602.24814,20012.11008,11.38165978,5.639248314,16.02121995,6.263850985,18,1,23.33240256,
6602.24814,20012.11008,11.38165978,5.639248314,16.02121995,6.263850985,18,1,23.33240256,
70,0,0,0,1,0,11,16602.24814,20012.11008,11.38165978,5.639248314,16.02121995,6.263850985,18
6602.24814,20012.11008,11.38165978,5.639248314,16.02121995,6.263850985,18,1,23.33240256,
6602.24814,20012.11008,11.38165978,5.639248314,16.02121995,6.263850985,18,1,23.33240256,
6602.24814,20012.11008,11.38165978,5.639248314,16.02121995,6.263850985,18,1,23.33240256,
0151,14.51050421,21.44532368,0.123400905,17,3,24.97335091,21.76575631,159.2255883,156.747
69,0,1,0,1,0,12,16300.36756,14631.54202,12.22100151,14.51050421,21.44532368,0.123400905,17
6300.36756,14631.54202,12.22100151,14.51050421,21.44532368,0.123400905,17,3,24.97335091,
6300.36756,14631.54202,12.22100151,14.51050421,21.44532368,0.123400905,17,3,24.97335091,
69,0,1,0,1,0,12,16300.36756,14631.54202,12.22100151,14.51050421,21.44532368,0.123400905,17
69,0,1,0,1,0,12,16300.36756,14631.54202,12.22100151,14.51050421,21.44532368,0.123400905,17
300.36756,14631.54202,12.22100151,14.51050421,21.44532368,0.123400905,17,3,24.97335091,21
69,0,1,0,1,0,12,16300.36756,14631.54202,12.22100151,14.51050421,21.44532368,0.123400905,17
6300.36756,14631.54202,12.22100151,14.51050421,21.44532368,0.123400905,17,3,24.97335091,
6300.36756,14631.54202,12.22100151,14.51050421,21.44532368,0.123400905,17,3,24.97335091,
0308838,3,2,205,69,0,1,0,1,0,12,16300.36756,14631.54202,12.22100151,14.51050421,21.44532368
69,0,1,0,1,0,12,16300.36756,14631.54202,12.22100151,14.51050421,21.44532368,0.123400905,17
6300.36756,14631.54202,12.22100151,14.51050421,21.44532368,0.123400905,17,3,24.97335091,
6300.36756,14631.54202,12.22100151,14.51050421,21.44532368,0.123400905,17,3,24.97335091,
6300.36756,14631.54202,12.22100151,14.51050421,21.44532368,0.123400905,17,3,24.97335091,
00.36756,14631.54202,12.22100151,14.51050421,21.44532368,0.123400905,17,3,24.97335091,21.
6300.36756,14631.54202,12.22100151,14.51050421,21.44532368,0.123400905,17,3,24.97335091,
69,0,1,0,1,0,12,16300.36756,14631.54202,12.22100151,14.51050421,21.44532368,0.123400905,17
69,0,1,0,1,0,12,16300.36756,14631.54202,12.22100151,14.51050421,21.44532368,0.123400905,17
0151,14.51050421,21.44532368,0.123400905,17,3,24.97335091,21.76575631,159.2255883,156.747
9382324,3,2,205,69,0,1,0,1,0,12,16300.36756,14631.54202,12.22100151,14.51050421,21.44532368
0151,14.51050421,21.44532368,0.123400905,17,3,24.97335091,21.76575631,159.2255883,156.747
0151,14.51050421,21.44532368,0.123400905,17,3,24.97335091,21.76575631,159.2255883,156.747
300.36756,14631.54202,12.22100151,14.51050421,21.44532368,0.123400905,17,3,24.97335091,21
6300.36756,14631.54202,12.22100151,14.51050421,21.44532368,0.123400905,17,3,24.97335091,
6300.36756,14631.54202,12.22100151,14.51050421,21.44532368,0.123400905,17,3,24.97335091,
300.36756,14631.54202,12.22100151,14.51050421,21.44532368,0.123400905,17,3,24.97335091,21
6300.36756,14631.54202,12.22100151,14.51050421,21.44532368,0.123400905,17,3,24.97335091,
69,0,1,0,1,0,12,16300.36756,14631.54202,12.22100151,14.51050421,21.44532368,0.123400905,17
300.36756,14631.54202,12.22100151,14.51050421,21.44532368,0.123400905,17,3,24.97335091,21
69,0,1,0,1,0,12,16300.36756,14631.54202,12.22100151,14.51050421,21.44532368,0.123400905,17
6300.36756,14631.54202,12.22100151,14.51050421,21.44532368,0.123400905,17,3,24.97335091,
6300.36756,14631.54202,12.22100151,14.51050421,21.44532368,0.123400905,17,3,24.97335091,
6300.36756,14631.54202,12.22100151,14.51050421,21.44532368,0.123400905,17,3,24.97335091,
5,69,0,1,0,1,0,12,16300.36756,14631.54202,12.22100151,14.51050421,21.44532368,0.123400905,1
358,18.67378965,16.08863003,1.737970367,16,3,33.25299201,37.34757929,196.2175843,208.4250
37606858,18.67378965,16.08863003,1.737970367,16,3,33.25299201,37.34757929,196.2175843,208
24.71365,22376.08185,11.87606858,18.67378965,16.08863003,1.737970367,16,3,33.25299201,37.
24.71365,22376.08185,11.87606858,18.67378965,16.08863003,1.737970367,16,3,33.25299201,37.
24.71365,22376.08185,11.87606858,18.67378965,16.08863003,1.737970367,16,3,33.25299201,37.
24.71365,22376.08185,11.87606858,18.67378965,16.08863003,1.737970367,16,3,33.25299201,37.
824.71365,22376.08185,11.87606858,18.67378965,16.08863003,1.737970367,16,3,33.25299201,37
8824.71365,22376.08185,11.87606858,18.67378965,16.08863003,1.737970367,16,3,33.25299201,
358,18.67378965,16.08863003,1.737970367,16,3,33.25299201,37.34757929,196.2175843,208.4250

358,18.67378965,16.08863003,1.737970367,16,3,33.25299201,37.34757929,196.2175843,208.4250
358,18.67378965,16.08863003,1.737970367,16,3,33.25299201,37.34757929,196.2175843,208.4250
11.87606858,18.67378965,16.08863003,1.737970367,16,3,33.25299201,37.34757929,196.2175843,
0,72,1,1,1,1,0,9,18824.71365,22376.08185,11.87606858,18.67378965,16.08863003,1.737970367,16
24.71365,22376.08185,11.87606858,18.67378965,16.08863003,1.737970367,16,3,33.25299201,37.
24.71365,22376.08185,11.87606858,18.67378965,16.08863003,1.737970367,16,3,33.25299201,37.
58,18.67378965,16.08863003,1.737970367,16,3,33.25299201,37.34757929,196.2175843,208.42500
824.71365,22376.08185,11.87606858,18.67378965,16.08863003,1.737970367,16,3,33.25299201,37
824.71365,22376.08185,11.87606858,18.67378965,16.08863003,1.737970367,16,3,33.25299201,37
824.71365,22376.08185,11.87606858,18.67378965,16.08863003,1.737970367,16,3,33.25299201,37
824.71365,22376.08185,11.87606858,18.67378965,16.08863003,1.737970367,16,3,33.25299201,37
358,18.67378965,16.08863003,1.737970367,16,3,33.25299201,37.34757929,196.2175843,208.4250
358,18.67378965,16.08863003,1.737970367,16,3,33.25299201,37.34757929,196.2175843,208.4250
72,1,1,1,1,0,9,18824.71365,22376.08185,11.87606858,18.67378965,16.08863003,1.737970367,16,3
824.71365,22376.08185,11.87606858,18.67378965,16.08863003,1.737970367,16,3,33.25299201,37
8824.71365,22376.08185,11.87606858,18.67378965,16.08863003,1.737970367,16,3,33.25299201,3
358,18.67378965,16.08863003,1.737970367,16,3,33.25299201,37.34757929,196.2175843,208.4250
0,72,1,1,1,1,0,9,18824.71365,22376.08185,11.87606858,18.67378965,16.08863003,1.737970367,16
1.87606858,18.67378965,16.08863003,1.737970367,16,3,33.25299201,37.34757929,196.2175843,2
8824.71365,22376.08185,11.87606858,18.67378965,16.08863003,1.737970367,16,3,33.25299201,3
24.71365,22376.08185,11.87606858,18.67378965,16.08863003,1.737970367,16,3,33.25299201,37.
358,18.67378965,16.08863003,1.737970367,16,3,33.25299201,37.34757929,196.2175843,208.4250
358,18.67378965,16.08863003,1.737970367,16,3,33.25299201,37.34757929,196.2175843,208.4250
358,18.67378965,16.08863003,1.737970367,16,3,33.25299201,37.34757929,196.2175843,208.4250
358,18.67378965,16.08863003,1.737970367,16,3,33.25299201,37.34757929,196.2175843,208.4250
24.71365,22376.08185,11.87606858,18.67378965,16.08863003,1.737970367,16,3,33.25299201,37.
03955857,13.83970214,13.56071175,0.792197863,15,2,33.11867572,59.97204262,194.5775094,207
9014.77955,22101.05705,11.03955857,13.83970214,13.56071175,0.792197863,15,2,33.11867572,5
014.77955,22101.05705,11.03955857,13.83970214,13.56071175,0.792197863,15,2,33.11867572,59
5857,13.83970214,13.56071175,0.792197863,15,2,33.11867572,59.97204262,194.5775094,207.427
9014.77955,22101.05705,11.03955857,13.83970214,13.56071175,0.792197863,15,2,33.11867572,5
3955857,13.83970214,13.56071175,0.792197863,15,2,33.11867572,59.97204262,194.5775094,207.
03955857,13.83970214,13.56071175,0.792197863,15,2,33.11867572,59.97204262,194.5775094,207
014.77955,22101.05705,11.03955857,13.83970214,13.56071175,0.792197863,15,2,33.11867572,59
11.03955857,13.83970214,13.56071175,0.792197863,15,2,33.11867572,59.97204262,194.5775094,
11.03955857,13.83970214,13.56071175,0.792197863,15,2,33.11867572,59.97204262,194.5775094,
9014.77955,22101.05705,11.03955857,13.83970214,13.56071175,0.792197863,15,2,33.11867572,5
55857,13.83970214,13.56071175,0.792197863,15,2,33.11867572,59.97204262,194.5775094,207.42
9014.77955,22101.05705,11.03955857,13.83970214,13.56071175,0.792197863,15,2,33.11867572,5
0,70,0,1,0,1,0,12,19014.77955,22101.05705,11.03955857,13.83970214,13.56071175,0.792197863,1
19014.77955,22101.05705,11.03955857,13.83970214,13.56071175,0.792197863,15,2,33.11867572
9014.77955,22101.05705,11.03955857,13.83970214,13.56071175,0.792197863,15,2,33.11867572,5
5857,13.83970214,13.56071175,0.792197863,15,2,33.11867572,59.97204262,194.5775094,207.427
014.77955,22101.05705,11.03955857,13.83970214,13.56071175,0.792197863,15,2,33.11867572,59
014.77955,22101.05705,11.03955857,13.83970214,13.56071175,0.792197863,15,2,33.11867572,59
5857,13.83970214,13.56071175,0.792197863,15,2,33.11867572,59.97204262,194.5775094,207.427
70,0,1,0,1,0,12,19014.77955,22101.05705,11.03955857,13.83970214,13.56071175,0.792197863,15
3955857,13.83970214,13.56071175,0.792197863,15,2,33.11867572,59.97204262,194.5775094,207.
9014.77955,22101.05705,11.03955857,13.83970214,13.56071175,0.792197863,15,2,33.11867572,5
19014.77955,22101.05705,11.03955857,13.83970214,13.56071175,0.792197863,15,2,33.11867572

014.77955,22101.05705,11.03955857,13.83970214,13.56071175,0.792197863,15,2,33.11867572,5
,70,0,1,0,1,0,12,19014.77955,22101.05705,11.03955857,13.83970214,13.56071175,0.792197863,1
9014.77955,22101.05705,11.03955857,13.83970214,13.56071175,0.792197863,15,2,33.11867572,
014.77955,22101.05705,11.03955857,13.83970214,13.56071175,0.792197863,15,2,33.11867572,5
9014.77955,22101.05705,11.03955857,13.83970214,13.56071175,0.792197863,15,2,33.11867572,
9014.77955,22101.05705,11.03955857,13.83970214,13.56071175,0.792197863,15,2,33.11867572,
857,13.83970214,13.56071175,0.792197863,15,2,33.11867572,59.97204262,194.5775094,207.427
955857,13.83970214,13.56071175,0.792197863,15,2,33.11867572,59.97204262,194.5775094,207.
9014.77955,22101.05705,11.03955857,13.83970214,13.56071175,0.792197863,15,2,33.11867572,
857,13.83970214,13.56071175,0.792197863,15,2,33.11867572,59.97204262,194.5775094,207.427
857,13.83970214,13.56071175,0.792197863,15,2,33.11867572,59.97204262,194.5775094,207.427
79382324,3,2,220,70,0,1,0,1,0,12,19014.77955,22101.05705,11.03955857,13.83970214,13.5607117
857,13.83970214,13.56071175,0.792197863,15,2,33.11867572,59.97204262,194.5775094,207.427
9014.77955,22101.05705,11.03955857,13.83970214,13.56071175,0.792197863,15,2,33.11867572,
9014.77955,22101.05705,11.03955857,13.83970214,13.56071175,0.792197863,15,2,33.11867572,
014.77955,22101.05705,11.03955857,13.83970214,13.56071175,0.792197863,15,2,33.11867572,5
19014.77955,22101.05705,11.03955857,13.83970214,13.56071175,0.792197863,15,2,33.11867572
857,13.83970214,13.56071175,0.792197863,15,2,33.11867572,59.97204262,194.5775094,207.427
014.77955,22101.05705,11.03955857,13.83970214,13.56071175,0.792197863,15,2,33.11867572,5
955857,13.83970214,13.56071175,0.792197863,15,2,33.11867572,59.97204262,194.5775094,207.
955857,13.83970214,13.56071175,0.792197863,15,2,33.11867572,59.97204262,194.5775094,207.
65,0,1,0,1,0,12,19014.77955,22101.05705,11.03955857,13.83970214,13.56071175,0.792197863,15
1.03955857,13.83970214,13.56071175,0.792197863,15,2,33.11867572,59.97204262,194.5775094,
014.77955,22101.05705,11.03955857,13.83970214,13.56071175,0.792197863,15,2,33.11867572,5
19014.77955,22101.05705,11.03955857,13.83970214,13.56071175,0.792197863,15,2,33.11867572
1.03955857,13.83970214,13.56071175,0.792197863,15,2,33.11867572,59.97204262,194.5775094,
5857,13.83970214,13.56071175,0.792197863,15,2,33.11867572,59.97204262,194.5775094,207.42
65,0,1,0,1,0,12,19014.77955,22101.05705,11.03955857,13.83970214,13.56071175,0.792197863,15
9014.77955,22101.05705,11.03955857,13.83970214,13.56071175,0.792197863,15,2,33.11867572,
014.77955,22101.05705,11.03955857,13.83970214,13.56071175,0.792197863,15,2,33.11867572,5
3955857,13.83970214,13.56071175,0.792197863,15,2,33.11867572,59.97204262,194.5775094,20
65,0,1,0,1,0,12,19014.77955,22101.05705,11.03955857,13.83970214,13.56071175,0.792197863,15
014.77955,22101.05705,11.03955857,13.83970214,13.56071175,0.792197863,15,2,33.11867572,5
11.03955857,13.83970214,13.56071175,0.792197863,15,2,33.11867572,59.97204262,194.5775094,
1.03955857,13.83970214,13.56071175,0.792197863,15,2,33.11867572,59.97204262,194.5775094,
955857,13.83970214,13.56071175,0.792197863,15,2,33.11867572,59.97204262,194.5775094,207.
857,13.83970214,13.56071175,0.792197863,15,2,33.11867572,59.97204262,194.5775094,207.427
9014.77955,22101.05705,11.03955857,13.83970214,13.56071175,0.792197863,15,2,33.11867572,
857,13.83970214,13.56071175,0.792197863,15,2,33.11867572,59.97204262,194.5775094,207.427
,65,0,1,0,1,0,12,19014.77955,22101.05705,11.03955857,13.83970214,13.56071175,0.792197863,1
9014.77955,22101.05705,11.03955857,13.83970214,13.56071175,0.792197863,15,2,33.11867572,
65,0,1,0,1,0,12,19014.77955,22101.05705,11.03955857,13.83970214,13.56071175,0.792197863,15
014.77955,22101.05705,11.03955857,13.83970214,13.56071175,0.792197863,15,2,33.11867572,5
014.77955,22101.05705,11.03955857,13.83970214,13.56071175,0.792197863,15,2,33.11867572,5
19014.77955,22101.05705,11.03955857,13.83970214,13.56071175,0.792197863,15,2,33.11867572
,11.84266043,16.45812869,1.344554061,16,2,38.89275366,39.47553475,53.45794275,223.355528
57,11.84266043,16.45812869,1.344554061,16,2,38.89275366,39.47553475,53.45794275,223.3555
617676,3,2,220,78,0,1,0,1,0,8,18371.52759,19538.94135,10.55660457,11.84266043,16.45812869,
71.52759,19538.94135,10.55660457,11.84266043,16.45812869,1.344554061,16,2,38.89275366,39.
371.52759,19538.94135,10.55660457,11.84266043,16.45812869,1.344554061,16,2,38.89275366,3

71.52759,19538.94135,10.55660457,11.84266043,16.45812869,1.344554061,16,2,38.89275366,39.
457,11.84266043,16.45812869,1.344554061,16,2,38.89275366,39.47553475,53.45794275,223.3555
78,0,1,0,1,0,8,18371.52759,19538.94135,10.55660457,11.84266043,16.45812869,1.344554061,16,
371.52759,19538.94135,10.55660457,11.84266043,16.45812869,1.344554061,16,2,38.89275366,3
55660457,11.84266043,16.45812869,1.344554061,16,2,38.89275366,39.47553475,53.45794275,22
0,78,0,1,0,1,0,8,18371.52759,19538.94135,10.55660457,11.84266043,16.45812869,1.344554061,16
10.55660457,11.84266043,16.45812869,1.344554061,16,2,38.89275366,39.47553475,53.45794275
371.52759,19538.94135,10.55660457,11.84266043,16.45812869,1.344554061,16,2,38.89275366,3
371.52759,19538.94135,10.55660457,11.84266043,16.45812869,1.344554061,16,2,38.89275366,3
560457,11.84266043,16.45812869,1.344554061,16,2,38.89275366,39.47553475,53.45794275,223.3
0,78,0,1,0,1,0,8,18371.52759,19538.94135,10.55660457,11.84266043,16.45812869,1.344554061,16
78,0,1,0,1,0,8,18371.52759,19538.94135,10.55660457,11.84266043,16.45812869,1.344554061,16,
78,0,1,0,1,0,8,18371.52759,19538.94135,10.55660457,11.84266043,16.45812869,1.344554061,16,
71.52759,19538.94135,10.55660457,11.84266043,16.45812869,1.344554061,16,2,38.89275366,39.
78,0,1,0,1,0,8,18371.52759,19538.94135,10.55660457,11.84266043,16.45812869,1.344554061,16,
371.52759,19538.94135,10.55660457,11.84266043,16.45812869,1.344554061,16,2,38.89275366,3
560457,11.84266043,16.45812869,1.344554061,16,2,38.89275366,39.47553475,53.45794275,223.3
560457,11.84266043,16.45812869,1.344554061,16,2,38.89275366,39.47553475,53.45794275,223.3
55857,13.83970214,13.56071175,0.792197863,15,2,33.11867572,59.97204262,194.5775094,207.42
78,1,0,1,1,0,8,19014.77955,22101.05705,11.03955857,13.83970214,13.56071175,0.792197863,15,
9014.77955,22101.05705,11.03955857,13.83970214,13.56071175,0.792197863,15,2,33.11867572,
78,1,0,1,1,0,8,19014.77955,22101.05705,11.03955857,13.83970214,13.56071175,0.792197863,15,
1.03955857,13.83970214,13.56071175,0.792197863,15,2,33.11867572,59.97204262,194.5775094,2
20617676,3,2,220,78,1,0,1,1,0,8,19014.77955,22101.05705,11.03955857,13.83970214,13.56071175
014.77955,22101.05705,11.03955857,13.83970214,13.56071175,0.792197863,15,2,33.11867572,5
014.77955,22101.05705,11.03955857,13.83970214,13.56071175,0.792197863,15,2,33.11867572,5
14.77955,22101.05705,11.03955857,13.83970214,13.56071175,0.792197863,15,2,33.11867572,59.
1.03955857,13.83970214,13.56071175,0.792197863,15,2,33.11867572,59.97204262,194.5775094,2
0,78,1,0,1,1,0,8,19014.77955,22101.05705,11.03955857,13.83970214,13.56071175,0.792197863,15
857,13.83970214,13.56071175,0.792197863,15,2,33.11867572,59.97204262,194.5775094,207.4274
0,78,1,0,1,1,0,8,19014.77955,22101.05705,11.03955857,13.83970214,13.56071175,0.792197863,15
9014.77955,22101.05705,11.03955857,13.83970214,13.56071175,0.792197863,15,2,33.11867572,
13.83970214,13.56071175,0.792197863,15,2,33.11867572,59.97204262,194.5775094,207.4274953
857,13.83970214,13.56071175,0.792197863,15,2,33.11867572,59.97204262,194.5775094,207.4274
19014.77955,22101.05705,11.03955857,13.83970214,13.56071175,0.792197863,15,2,33.11867572,
014.77955,22101.05705,11.03955857,13.83970214,13.56071175,0.792197863,15,2,33.11867572,5
78,1,0,1,1,0,8,19014.77955,22101.05705,11.03955857,13.83970214,13.56071175,0.792197863,15,
9014.77955,22101.05705,11.03955857,13.83970214,13.56071175,0.792197863,15,2,33.11867572,
55857,13.83970214,13.56071175,0.792197863,15,2,33.11867572,59.97204262,194.5775094,207.42
78,1,0,1,1,0,8,19014.77955,22101.05705,11.03955857,13.83970214,13.56071175,0.792197863,15,
78,1,0,1,1,0,8,19014.77955,22101.05705,11.03955857,13.83970214,13.56071175,0.792197863,15,
14.77955,22101.05705,11.03955857,13.83970214,13.56071175,0.792197863,15,2,33.11867572,59.
014.77955,22101.05705,11.03955857,13.83970214,13.56071175,0.792197863,15,2,33.11867572,5
14.77955,22101.05705,11.03955857,13.83970214,13.56071175,0.792197863,15,2,33.11867572,59.
78,1,0,1,1,0,8,19014.77955,22101.05705,11.03955857,13.83970214,13.56071175,0.792197863,15,
9014.77955,22101.05705,11.03955857,13.83970214,13.56071175,0.792197863,15,2,33.11867572,5
1.03955857,13.83970214,13.56071175,0.792197863,15,2,33.11867572,59.97204262,194.5775094,2
858,18.67378965,16.08863003,1.737970367,16,3,33.25299201,37.34757929,196.2175843,208.4250
5858,18.67378965,16.08863003,1.737970367,16,3,33.25299201,37.34757929,196.2175843,208.425
858,18.67378965,16.08863003,1.737970367,16,3,33.25299201,37.34757929,196.2175843,208.4250

18824.71365,22376.08185,11.87606858,18.67378965,16.08863003,1.737970367,16,3,33.25299201
18824.71365,22376.08185,11.87606858,18.67378965,16.08863003,1.737970367,16,3,33.25299201,
824.71365,22376.08185,11.87606858,18.67378965,16.08863003,1.737970367,16,3,33.25299201,3
824.71365,22376.08185,11.87606858,18.67378965,16.08863003,1.737970367,16,3,33.25299201,3
7606858,18.67378965,16.08863003,1.737970367,16,3,33.25299201,37.34757929,196.2175843,208.
8824.71365,22376.08185,11.87606858,18.67378965,16.08863003,1.737970367,16,3,33.25299201,3
0,75,1,1,1,1,0,12,18824.71365,22376.08185,11.87606858,18.67378965,16.08863003,1.737970367,1
7606858,18.67378965,16.08863003,1.737970367,16,3,33.25299201,37.34757929,196.2175843,208.
6858,18.67378965,16.08863003,1.737970367,16,3,33.25299201,37.34757929,196.2175843,208.425
7606858,18.67378965,16.08863003,1.737970367,16,3,33.25299201,37.34757929,196.2175843,208.
11.87606858,18.67378965,16.08863003,1.737970367,16,3,33.25299201,37.34757929,196.2175843,
18824.71365,22376.08185,11.87606858,18.67378965,16.08863003,1.737970367,16,3,33.25299201
824.71365,22376.08185,11.87606858,18.67378965,16.08863003,1.737970367,16,3,33.25299201,3
0,75,1,1,1,1,0,12,18824.71365,22376.08185,11.87606858,18.67378965,16.08863003,1.737970367,1
7606858,18.67378965,16.08863003,1.737970367,16,3,33.25299201,37.34757929,196.2175843,208.
6858,18.67378965,16.08863003,1.737970367,16,3,33.25299201,37.34757929,196.2175843,208.425
8824.71365,22376.08185,11.87606858,18.67378965,16.08863003,1.737970367,16,3,33.25299201,3
824.71365,22376.08185,11.87606858,18.67378965,16.08863003,1.737970367,16,3,33.25299201,3
0,80,1,1,1,0,0,9,19014.77955,22101.05705,11.03955857,13.83970214,13.56071175,0.792197863,15
14.77955,22101.05705,11.03955857,13.83970214,13.56071175,0.792197863,15,2,33.11867572,59.
955857,13.83970214,13.56071175,0.792197863,15,2,33.11867572,59.97204262,194.5775094,207.4
0,80,1,1,1,0,0,9,19014.77955,22101.05705,11.03955857,13.83970214,13.56071175,0.792197863,15
014.77955,22101.05705,11.03955857,13.83970214,13.56071175,0.792197863,15,2,33.11867572,59
14.77955,22101.05705,11.03955857,13.83970214,13.56071175,0.792197863,15,2,33.11867572,59.
4741211,3,2,220,80,1,1,1,0,0,9,19014.77955,22101.05705,11.03955857,13.83970214,13.56071175,0
19014.77955,22101.05705,11.03955857,13.83970214,13.56071175,0.792197863,15,2,33.11867572,
14.77955,22101.05705,11.03955857,13.83970214,13.56071175,0.792197863,15,2,33.11867572,59.
857,13.83970214,13.56071175,0.792197863,15,2,33.11867572,59.97204262,194.5775094,207.4274
57,13.83970214,13.56071175,0.792197863,15,2,33.11867572,59.97204262,194.5775094,207.42749
9014.77955,22101.05705,11.03955857,13.83970214,13.56071175,0.792197863,15,2,33.11867572,5
857,13.83970214,13.56071175,0.792197863,15,2,33.11867572,59.97204262,194.5775094,207.4274
80,1,1,1,0,0,9,19014.77955,22101.05705,11.03955857,13.83970214,13.56071175,0.792197863,15,2
955857,13.83970214,13.56071175,0.792197863,15,2,33.11867572,59.97204262,194.5775094,207.4
857,13.83970214,13.56071175,0.792197863,15,2,33.11867572,59.97204262,194.5775094,207.4274
7629395,3,2,220,80,1,1,1,0,0,9,19014.77955,22101.05705,11.03955857,13.83970214,13.56071175,0
0,80,1,1,1,0,0,9,19014.77955,22101.05705,11.03955857,13.83970214,13.56071175,0.792197863,15
9014.77955,22101.05705,11.03955857,13.83970214,13.56071175,0.792197863,15,2,33.11867572,5
857,13.83970214,13.56071175,0.792197863,15,2,33.11867572,59.97204262,194.5775094,207.4274
14.77955,22101.05705,11.03955857,13.83970214,13.56071175,0.792197863,15,2,33.11867572,59.
014.77955,22101.05705,11.03955857,13.83970214,13.56071175,0.792197863,15,2,33.11867572,59
014.77955,22101.05705,11.03955857,13.83970214,13.56071175,0.792197863,15,2,33.11867572,59
857,13.83970214,13.56071175,0.792197863,15,2,33.11867572,59.97204262,194.5775094,207.4274
5857,13.83970214,13.56071175,0.792197863,15,2,33.11867572,59.97204262,194.5775094,207.427
9014.77955,22101.05705,11.03955857,13.83970214,13.56071175,0.792197863,15,2,33.11867572,5
5857,13.83970214,13.56071175,0.792197863,15,2,33.11867572,59.97204262,194.5775094,207.427
5857,13.83970214,13.56071175,0.792197863,15,2,33.11867572,59.97204262,194.5775094,207.427
9014.77955,22101.05705,11.03955857,13.83970214,13.56071175,0.792197863,15,2,33.11867572,5
014.77955,22101.05705,11.03955857,13.83970214,13.56071175,0.792197863,15,2,33.11867572,59
11.03955857,13.83970214,13.56071175,0.792197863,15,2,33.11867572,59.97204262,194.5775094,
5857,13.83970214,13.56071175,0.792197863,15,2,33.11867572,59.97204262,194.5775094,207.427

67,1,1,1,0,0,11,19014.77955,22101.05705,11.03955857,13.83970214,13.56071175,0.792197863,15
5857,13.83970214,13.56071175,0.792197863,15,2,33.11867572,59.97204262,194.5775094,207.427
9014.77955,22101.05705,11.03955857,13.83970214,13.56071175,0.792197863,15,2,33.11867572,
5,11.03955857,13.83970214,13.56071175,0.792197863,15,2,33.11867572,59.97204262,194.577509
857,13.83970214,13.56071175,0.792197863,15,2,33.11867572,59.97204262,194.5775094,207.4274
5857,13.83970214,13.56071175,0.792197863,15,2,33.11867572,59.97204262,194.5775094,207.427
19014.77955,22101.05705,11.03955857,13.83970214,13.56071175,0.792197863,15,2,33.11867572,
11.03955857,13.83970214,13.56071175,0.792197863,15,2,33.11867572,59.97204262,194.5775094,
9014.77955,22101.05705,11.03955857,13.83970214,13.56071175,0.792197863,15,2,33.11867572,
5857,13.83970214,13.56071175,0.792197863,15,2,33.11867572,59.97204262,194.5775094,207.427
9014.77955,22101.05705,11.03955857,13.83970214,13.56071175,0.792197863,15,2,33.11867572,
9014.77955,22101.05705,11.03955857,13.83970214,13.56071175,0.792197863,15,2,33.11867572,
9014.77955,22101.05705,11.03955857,13.83970214,13.56071175,0.792197863,15,2,33.11867572,
9014.77955,22101.05705,11.03955857,13.83970214,13.56071175,0.792197863,15,2,33.11867572,
7,13.83970214,13.56071175,0.792197863,15,2,33.11867572,59.97204262,194.5775094,207.427495
9014.77955,22101.05705,11.03955857,13.83970214,13.56071175,0.792197863,15,2,33.11867572,
9014.77955,22101.05705,11.03955857,13.83970214,13.56071175,0.792197863,15,2,33.11867572,
9014.77955,22101.05705,11.03955857,13.83970214,13.56071175,0.792197863,15,2,33.11867572,
19014.77955,22101.05705,11.03955857,13.83970214,13.56071175,0.792197863,15,2,33.11867572
5857,13.83970214,13.56071175,0.792197863,15,2,33.11867572,59.97204262,194.5775094,207.427
4799,16.8190805,13.56071175,0.792197863,15,2,33.41978818,50.4572415,65.87678328,213.45542
5.89682,10.24154799,16.8190805,13.56071175,0.792197863,15,2,33.41978818,50.4572415,65.876
472.09613,23165.89682,10.24154799,16.8190805,13.56071175,0.792197863,15,2,33.41978818,50.
1,18472.09613,23165.89682,10.24154799,16.8190805,13.56071175,0.792197863,15,2,33.4197881
1,18472.09613,23165.89682,10.24154799,16.8190805,13.56071175,0.792197863,15,2,33.4197881
799,16.8190805,13.56071175,0.792197863,15,2,33.41978818,50.4572415,65.87678328,213.455421
8472.09613,23165.89682,10.24154799,16.8190805,13.56071175,0.792197863,15,2,33.41978818,5
472.09613,23165.89682,10.24154799,16.8190805,13.56071175,0.792197863,15,2,33.41978818,50.
8472.09613,23165.89682,10.24154799,16.8190805,13.56071175,0.792197863,15,2,33.41978818,5
8472.09613,23165.89682,10.24154799,16.8190805,13.56071175,0.792197863,15,2,33.41978818,5
799,16.8190805,13.56071175,0.792197863,15,2,33.41978818,50.4572415,65.87678328,213.455421
4741211,3,2,220,88,1,1,1,0,0,11,18472.09613,23165.89682,10.24154799,16.8190805,13.56071175,
8472.09613,23165.89682,10.24154799,16.8190805,13.56071175,0.792197863,15,2,33.41978818,5
8472.09613,23165.89682,10.24154799,16.8190805,13.56071175,0.792197863,15,2,33.41978818,5
8472.09613,23165.89682,10.24154799,16.8190805,13.56071175,0.792197863,15,2,33.41978818,5
88,1,1,1,0,0,11,18472.09613,23165.89682,10.24154799,16.8190805,13.56071175,0.792197863,15,
4799,16.8190805,13.56071175,0.792197863,15,2,33.41978818,50.4572415,65.87678328,213.45542
64197,8.118074978,16.24710447,0.393416306,16,1,34.49841561,56.82652484,179.9667377,195.3
38.10389,20676.03065,11.14564197,8.118074978,16.24710447,0.393416306,16,1,34.49841561,56.
438.10389,20676.03065,11.14564197,8.118074978,16.24710447,0.393416306,16,1,34.49841561,5
438.10389,20676.03065,11.14564197,8.118074978,16.24710447,0.393416306,16,1,34.49841561,5
8438.10389,20676.03065,11.14564197,8.118074978,16.24710447,0.393416306,16,1,34.49841561,
5,11.14564197,8.118074978,16.24710447,0.393416306,16,1,34.49841561,56.82652484,179.96673
75,1,1,1,1,0,8,18438.10389,20676.03065,11.14564197,8.118074978,16.24710447,0.393416306,16,
438.10389,20676.03065,11.14564197,8.118074978,16.24710447,0.393416306,16,1,34.49841561,5
438.10389,20676.03065,11.14564197,8.118074978,16.24710447,0.393416306,16,1,34.49841561,5
0,75,1,1,1,1,0,8,18438.10389,20676.03065,11.14564197,8.118074978,16.24710447,0.393416306,16
438.10389,20676.03065,11.14564197,8.118074978,16.24710447,0.393416306,16,1,34.49841561,5
8438.10389,20676.03065,11.14564197,8.118074978,16.24710447,0.393416306,16,1,34.49841561,
0,75,1,1,1,1,0,8,18438.10389,20676.03065,11.14564197,8.118074978,16.24710447,0.393416306,16

438.10389,20676.03065,11.14564197,8.118074978,16.24710447,0.393416306,16,1,34.49841561,5
438.10389,20676.03065,11.14564197,8.118074978,16.24710447,0.393416306,16,1,34.49841561,5
38.10389,20676.03065,11.14564197,8.118074978,16.24710447,0.393416306,16,1,34.49841561,56.
438.10389,20676.03065,11.14564197,8.118074978,16.24710447,0.393416306,16,1,34.49841561,5
438.10389,20676.03065,11.14564197,8.118074978,16.24710447,0.393416306,16,1,34.49841561,5
75,1,1,1,1,0,8,18438.10389,20676.03065,11.14564197,8.118074978,16.24710447,0.393416306,16,
4564197,8.118074978,16.24710447,0.393416306,16,1,34.49841561,56.82652484,179.9667377,19
64197,8.118074978,16.24710447,0.393416306,16,1,34.49841561,56.82652484,179.9667377,195.3
97,8.118074978,16.24710447,0.393416306,16,1,34.49841561,56.82652484,179.9667377,195.3141
38.10389,20676.03065,11.14564197,8.118074978,16.24710447,0.393416306,16,1,34.49841561,56.
97,8.118074978,16.24710447,0.393416306,16,1,34.49841561,56.82652484,179.9667377,195.3141
75,1,1,1,1,0,8,18438.10389,20676.03065,11.14564197,8.118074978,16.24710447,0.393416306,16,
98,7.981410292,16.24710447,0.393416306,16,1,33.46922395,59.86057719,183.4970733,199.7528
22.04021,21196.07101,11.15640798,7.981410292,16.24710447,0.393416306,16,1,33.46922395,59.
22.04021,21196.07101,11.15640798,7.981410292,16.24710447,0.393416306,16,1,33.46922395,59.
98,7.981410292,16.24710447,0.393416306,16,1,33.46922395,59.86057719,183.4970733,199.7528
22.04021,21196.07101,11.15640798,7.981410292,16.24710447,0.393416306,16,1,33.46922395,59.
98,7.981410292,16.24710447,0.393416306,16,1,33.46922395,59.86057719,183.4970733,199.7528
22.04021,21196.07101,11.15640798,7.981410292,16.24710447,0.393416306,16,1,33.46922395,59.
8322.04021,21196.07101,11.15640798,7.981410292,16.24710447,0.393416306,16,1,33.46922395,
.15640798,7.981410292,16.24710447,0.393416306,16,1,33.46922395,59.86057719,183.4970733,1
22.04021,21196.07101,11.15640798,7.981410292,16.24710447,0.393416306,16,1,33.46922395,59.
98,7.981410292,16.24710447,0.393416306,16,1,33.46922395,59.86057719,183.4970733,199.7528
0,81,0,1,1,0,0,9,18322.04021,21196.07101,11.15640798,7.981410292,16.24710447,0.393416306,1
98,7.981410292,16.24710447,0.393416306,16,1,33.46922395,59.86057719,183.4970733,199.7528
,81,0,1,1,0,0,9,18322.04021,21196.07101,11.15640798,7.981410292,16.24710447,0.393416306,16
.15640798,7.981410292,16.24710447,0.393416306,16,1,33.46922395,59.86057719,183.4970733,1
98,7.981410292,16.24710447,0.393416306,16,1,33.46922395,59.86057719,183.4970733,199.7528
98,7.981410292,16.24710447,0.393416306,16,1,33.46922395,59.86057719,183.4970733,199.7528
8322.04021,21196.07101,11.15640798,7.981410292,16.24710447,0.393416306,16,1,33.46922395,
5640798,7.981410292,16.24710447,0.393416306,16,1,33.46922395,59.86057719,183.4970733,19
,81,0,1,1,0,0,9,18322.04021,21196.07101,11.15640798,7.981410292,16.24710447,0.393416306,16
40798,7.981410292,16.24710447,0.393416306,16,1,33.46922395,59.86057719,183.4970733,199.7
22.04021,21196.07101,11.15640798,7.981410292,16.24710447,0.393416306,16,1,33.46922395,59.
98,7.981410292,16.24710447,0.393416306,16,1,33.46922395,59.86057719,183.4970733,199.7528
,81,0,1,1,0,0,9,18322.04021,21196.07101,11.15640798,7.981410292,16.24710447,0.393416306,16
98,7.981410292,16.24710447,0.393416306,16,1,33.46922395,59.86057719,183.4970733,199.7528
98,7.981410292,16.24710447,0.393416306,16,1,33.46922395,59.86057719,183.4970733,199.7528
98,7.981410292,16.24710447,0.393416306,16,1,33.46922395,59.86057719,183.4970733,199.7528
81,0,1,1,0,0,9,18322.04021,21196.07101,11.15640798,7.981410292,16.24710447,0.393416306,16,
98,7.981410292,16.24710447,0.393416306,16,1,33.46922395,59.86057719,183.4970733,199.7528
40798,7.981410292,16.24710447,0.393416306,16,1,33.46922395,59.86057719,183.4970733,199.7
22.04021,21196.07101,11.15640798,7.981410292,16.24710447,0.393416306,16,1,33.46922395,59.
,8.118074978,16.24710447,0.393416306,16,1,34.49841561,56.82652484,179.9667377,195.314106
18438.10389,20676.03065,11.14564197,8.118074978,16.24710447,0.393416306,16,1,34.49841561
8438.10389,20676.03065,11.14564197,8.118074978,16.24710447,0.393416306,16,1,34.49841561,
71,1,1,1,0,0,10,18438.10389,20676.03065,11.14564197,8.118074978,16.24710447,0.393416306,16
438.10389,20676.03065,11.14564197,8.118074978,16.24710447,0.393416306,16,1,34.49841561,5
197,8.118074978,16.24710447,0.393416306,16,1,34.49841561,56.82652484,179.9667377,195.314
438.10389,20676.03065,11.14564197,8.118074978,16.24710447,0.393416306,16,1,34.49841561,5

438.10389,20676.03065,11.14564197,8.118074978,16.24710447,0.393416306,16,1,34.49841561,5
8438.10389,20676.03065,11.14564197,8.118074978,16.24710447,0.393416306,16,1,34.49841561,
438.10389,20676.03065,11.14564197,8.118074978,16.24710447,0.393416306,16,1,34.49841561,5
1.14564197,8.118074978,16.24710447,0.393416306,16,1,34.49841561,56.82652484,179.9667377,
4564197,8.118074978,16.24710447,0.393416306,16,1,34.49841561,56.82652484,179.9667377,195.
8438.10389,20676.03065,11.14564197,8.118074978,16.24710447,0.393416306,16,1,34.49841561,
8438.10389,20676.03065,11.14564197,8.118074978,16.24710447,0.393416306,16,1,34.49841561,
0,71,1,1,1,0,0,10,18438.10389,20676.03065,11.14564197,8.118074978,16.24710447,0.393416306,1
4197,8.118074978,16.24710447,0.393416306,16,1,34.49841561,56.82652484,179.9667377,195.314
8438.10389,20676.03065,11.14564197,8.118074978,16.24710447,0.393416306,16,1,34.49841561,
438.10389,20676.03065,11.14564197,8.118074978,16.24710447,0.393416306,16,1,34.49841561,5
8438.10389,20676.03065,11.14564197,8.118074978,16.24710447,0.393416306,16,1,34.49841561,
4564197,8.118074978,16.24710447,0.393416306,16,1,34.49841561,56.82652484,179.9667377,195.
4197,8.118074978,16.24710447,0.393416306,16,1,34.49841561,56.82652484,179.9667377,195.314
438.10389,20676.03065,11.14564197,8.118074978,16.24710447,0.393416306,16,1,34.49841561,5
4197,8.118074978,16.24710447,0.393416306,16,1,34.49841561,56.82652484,179.9667377,195.314
438.10389,20676.03065,11.14564197,8.118074978,16.24710447,0.393416306,16,1,34.49841561,5
71,1,1,1,0,0,10,18438.10389,20676.03065,11.14564197,8.118074978,16.24710447,0.393416306,16
11.14564197,8.118074978,16.24710447,0.393416306,16,1,34.49841561,56.82652484,179.9667377,
8438.10389,20676.03065,11.14564197,8.118074978,16.24710447,0.393416306,16,1,34.49841561,
71,1,1,1,0,0,10,18438.10389,20676.03065,11.14564197,8.118074978,16.24710447,0.393416306,16
8438.10389,20676.03065,11.14564197,8.118074978,16.24710447,0.393416306,16,1,34.49841561,
18438.10389,20676.03065,11.14564197,8.118074978,16.24710447,0.393416306,16,1,34.49841561
4851282,12.5485665,15.45651444,1.344554061,15,2,35.16979489,33.46284399,79.60321502,221.0
1282,12.5485665,15.45651444,1.344554061,15,2,35.16979489,33.46284399,79.60321502,221.0672
1282,12.5485665,15.45651444,1.344554061,15,2,35.16979489,33.46284399,79.60321502,221.0672
0,76,0,1,0,1,0,10,18517.61472,22288.67712,10.04851282,12.5485665,15.45651444,1.344554061,15
10.04851282,12.5485665,15.45651444,1.344554061,15,2,35.16979489,33.46284399,79.60321502,2
1282,12.5485665,15.45651444,1.344554061,15,2,35.16979489,33.46284399,79.60321502,221.0672
18517.61472,22288.67712,10.04851282,12.5485665,15.45651444,1.344554061,15,2,35.16979489,
517.61472,22288.67712,10.04851282,12.5485665,15.45651444,1.344554061,15,2,35.16979489,33.
1282,12.5485665,15.45651444,1.344554061,15,2,35.16979489,33.46284399,79.60321502,221.0672
517.61472,22288.67712,10.04851282,12.5485665,15.45651444,1.344554061,15,2,35.16979489,33.
18517.61472,22288.67712,10.04851282,12.5485665,15.45651444,1.344554061,15,2,35.16979489,
1282,12.5485665,15.45651444,1.344554061,15,2,35.16979489,33.46284399,79.60321502,221.0672
282,12.5485665,15.45651444,1.344554061,15,2,35.16979489,33.46284399,79.60321502,221.06728
76,0,1,0,1,0,10,18517.61472,22288.67712,10.04851282,12.5485665,15.45651444,1.344554061,15,
0,76,0,1,0,1,0,10,18517.61472,22288.67712,10.04851282,12.5485665,15.45651444,1.344554061,15
517.61472,22288.67712,10.04851282,12.5485665,15.45651444,1.344554061,15,2,35.16979489,33.
1282,12.5485665,15.45651444,1.344554061,15,2,35.16979489,33.46284399,79.60321502,221.0672
282,12.5485665,15.45651444,1.344554061,15,2,35.16979489,33.46284399,79.60321502,221.06728
8517.61472,22288.67712,10.04851282,12.5485665,15.45651444,1.344554061,15,2,35.16979489,3
517.61472,22288.67712,10.04851282,12.5485665,15.45651444,1.344554061,15,2,35.16979489,33.
517.61472,22288.67712,10.04851282,12.5485665,15.45651444,1.344554061,15,2,35.16979489,33.
4851282,12.5485665,15.45651444,1.344554061,15,2,35.16979489,33.46284399,79.60321502,221.0
8517.61472,22288.67712,10.04851282,12.5485665,15.45651444,1.344554061,15,2,35.16979489,3
517.61472,22288.67712,10.04851282,12.5485665,15.45651444,1.344554061,15,2,35.16979489,33.
76,0,1,0,1,0,10,18517.61472,22288.67712,10.04851282,12.5485665,15.45651444,1.344554061,15,
0,76,0,1,0,1,0,10,18517.61472,22288.67712,10.04851282,12.5485665,15.45651444,1.344554061,15
76,0,1,0,1,0,10,18517.61472,22288.67712,10.04851282,12.5485665,15.45651444,1.344554061,15,

8517.61472,22288.67712,10.04851282,12.5485665,15.45651444,1.344554061,15,2,35.16979489,3
517.61472,22288.67712,10.04851282,12.5485665,15.45651444,1.344554061,15,2,35.16979489,33.
18517.61472,22288.67712,10.04851282,12.5485665,15.45651444,1.344554061,15,2,35.16979489,3
47,11.80852266,16.45812869,1.344554061,16,2,40.25025301,39.3617422,30.45889893,217.124604
19447,11.80852266,16.45812869,1.344554061,16,2,40.25025301,39.3617422,30.45889893,217.12
47,11.80852266,16.45812869,1.344554061,16,2,40.25025301,39.3617422,30.45889893,217.124604
447,11.80852266,16.45812869,1.344554061,16,2,40.25025301,39.3617422,30.45889893,217.12460
77,1,1,0,1,0,7,18498.08569,19605.36342,10.77119447,11.80852266,16.45812869,1.344554061,16,
447,11.80852266,16.45812869,1.344554061,16,2,40.25025301,39.3617422,30.45889893,217.12460
98.08569,19605.36342,10.77119447,11.80852266,16.45812869,1.344554061,16,2,40.25025301,39.
447,11.80852266,16.45812869,1.344554061,16,2,40.25025301,39.3617422,30.45889893,217.12460
8498.08569,19605.36342,10.77119447,11.80852266,16.45812869,1.344554061,16,2,40.25025301,3
447,11.80852266,16.45812869,1.344554061,16,2,40.25025301,39.3617422,30.45889893,217.12460
447,11.80852266,16.45812869,1.344554061,16,2,40.25025301,39.3617422,30.45889893,217.12460
19447,11.80852266,16.45812869,1.344554061,16,2,40.25025301,39.3617422,30.45889893,217.12
47,11.80852266,16.45812869,1.344554061,16,2,40.25025301,39.3617422,30.45889893,217.124604
447,11.80852266,16.45812869,1.344554061,16,2,40.25025301,39.3617422,30.45889893,217.12460
498.08569,19605.36342,10.77119447,11.80852266,16.45812869,1.344554061,16,2,40.25025301,39
98.08569,19605.36342,10.77119447,11.80852266,16.45812869,1.344554061,16,2,40.25025301,39.
19447,11.80852266,16.45812869,1.344554061,16,2,40.25025301,39.3617422,30.45889893,217.12
447,11.80852266,16.45812869,1.344554061,16,2,40.25025301,39.3617422,30.45889893,217.12460
98.08569,19605.36342,10.77119447,11.80852266,16.45812869,1.344554061,16,2,40.25025301,39.
98.08569,19605.36342,10.77119447,11.80852266,16.45812869,1.344554061,16,2,40.25025301,39.
498.08569,19605.36342,10.77119447,11.80852266,16.45812869,1.344554061,16,2,40.25025301,39
19447,11.80852266,16.45812869,1.344554061,16,2,40.25025301,39.3617422,30.45889893,217.12
.77119447,11.80852266,16.45812869,1.344554061,16,2,40.25025301,39.3617422,30.45889893,21
447,11.80852266,16.45812869,1.344554061,16,2,40.25025301,39.3617422,30.45889893,217.12460
8498.08569,19605.36342,10.77119447,11.80852266,16.45812869,1.344554061,16,2,40.25025301,3
447,11.80852266,16.45812869,1.344554061,16,2,40.25025301,39.3617422,30.45889893,217.12460
98.08569,19605.36342,10.77119447,11.80852266,16.45812869,1.344554061,16,2,40.25025301,39.
447,11.80852266,16.45812869,1.344554061,16,2,40.25025301,39.3617422,30.45889893,217.12460
98.08569,19605.36342,10.77119447,11.80852266,16.45812869,1.344554061,16,2,40.25025301,39.
98.08569,19605.36342,10.77119447,11.80852266,16.45812869,1.344554061,16,2,40.25025301,39.
19447,11.80852266,16.45812869,1.344554061,16,2,40.25025301,39.3617422,30.45889893,217.12
98.08569,19605.36342,10.77119447,11.80852266,16.45812869,1.344554061,16,2,40.25025301,39.
498.08569,19605.36342,10.77119447,11.80852266,16.45812869,1.344554061,16,2,40.25025301,39
447,11.80852266,16.45812869,1.344554061,16,2,40.25025301,39.3617422,30.45889893,217.12460
498.08569,19605.36342,10.77119447,11.80852266,16.45812869,1.344554061,16,2,40.25025301,39
498.08569,19605.36342,10.77119447,11.80852266,16.45812869,1.344554061,16,2,40.25025301,39
72,0,1,0,1,0,12,18498.08569,19605.36342,10.77119447,11.80852266,16.45812869,1.344554061,16
8498.08569,19605.36342,10.77119447,11.80852266,16.45812869,1.344554061,16,2,40.25025301,3
498.08569,19605.36342,10.77119447,11.80852266,16.45812869,1.344554061,16,2,40.25025301,39
9447,11.80852266,16.45812869,1.344554061,16,2,40.25025301,39.3617422,30.45889893,217.1246
18498.08569,19605.36342,10.77119447,11.80852266,16.45812869,1.344554061,16,2,40.25025301,
498.08569,19605.36342,10.77119447,11.80852266,16.45812869,1.344554061,16,2,40.25025301,39
72,0,1,0,1,0,12,18498.08569,19605.36342,10.77119447,11.80852266,16.45812869,1.344554061,16
18498.08569,19605.36342,10.77119447,11.80852266,16.45812869,1.344554061,16,2,40.25025301
0.77119447,11.80852266,16.45812869,1.344554061,16,2,40.25025301,39.3617422,30.45889893,21
8498.08569,19605.36342,10.77119447,11.80852266,16.45812869,1.344554061,16,2,40.25025301,3
498.08569,19605.36342,10.77119447,11.80852266,16.45812869,1.344554061,16,2,40.25025301,39

498.08569,19605.36342,10.77119447,11.80852266,16.45812869,1.344554061,16,2,40.25025301,3
8498.08569,19605.36342,10.77119447,11.80852266,16.45812869,1.344554061,16,2,40.25025301,
9447,11.80852266,16.45812869,1.344554061,16,2,40.25025301,39.3617422,30.45889893,217.1246
72,0,1,0,1,0,12,18498.08569,19605.36342,10.77119447,11.80852266,16.45812869,1.344554061,16
447,11.80852266,16.45812869,1.344554061,16,2,40.25025301,39.3617422,30.45889893,217.12460
9447,11.80852266,16.45812869,1.344554061,16,2,40.25025301,39.3617422,30.45889893,217.1246
8498.08569,19605.36342,10.77119447,11.80852266,16.45812869,1.344554061,16,2,40.25025301,
7119447,11.80852266,16.45812869,1.344554061,16,2,40.25025301,39.3617422,30.45889893,217.1
.77119447,11.80852266,16.45812869,1.344554061,16,2,40.25025301,39.3617422,30.45889893,21
498.08569,19605.36342,10.77119447,11.80852266,16.45812869,1.344554061,16,2,40.25025301,3
9447,11.80852266,16.45812869,1.344554061,16,2,40.25025301,39.3617422,30.45889893,217.1246
72,0,1,0,1,0,12,18498.08569,19605.36342,10.77119447,11.80852266,16.45812869,1.344554061,16
18498.08569,19605.36342,10.77119447,11.80852266,16.45812869,1.344554061,16,2,40.25025301
119447,11.80852266,16.45812869,1.344554061,16,2,40.25025301,39.3617422,30.45889893,217.12
9447,11.80852266,16.45812869,1.344554061,16,2,40.25025301,39.3617422,30.45889893,217.1246
18498.08569,19605.36342,10.77119447,11.80852266,16.45812869,1.344554061,16,2,40.25025301
18498.08569,19605.36342,10.77119447,11.80852266,16.45812869,1.344554061,16,2,40.25025301
498.08569,19605.36342,10.77119447,11.80852266,16.45812869,1.344554061,16,2,40.25025301,3
9447,11.80852266,16.45812869,1.344554061,16,2,40.25025301,39.3617422,30.45889893,217.1246
119447,11.80852266,16.45812869,1.344554061,16,2,40.25025301,39.3617422,30.45889893,217.12
8498.08569,19605.36342,10.77119447,11.80852266,16.45812869,1.344554061,16,2,40.25025301,
18498.08569,19605.36342,10.77119447,11.80852266,16.45812869,1.344554061,16,2,40.25025301
9447,11.80852266,16.45812869,1.344554061,16,2,40.25025301,39.3617422,30.45889893,217.1246
13609,10.6005456,15.74446008,1.344554061,22,2,35.20887992,42.40218242,9.260492097,228.069
8470.14657,21687.77583,12.61213609,10.6005456,15.74446008,1.344554061,22,2,35.20887992,4
8470.14657,21687.77583,12.61213609,10.6005456,15.74446008,1.344554061,22,2,35.20887992,4
470.14657,21687.77583,12.61213609,10.6005456,15.74446008,1.344554061,22,2,35.20887992,42.
3609,10.6005456,15.74446008,1.344554061,22,2,35.20887992,42.40218242,9.260492097,228.0694
3,0,1,0,1,0,11,18470.14657,21687.77583,12.61213609,10.6005456,15.74446008,1.344554061,22,2,
73,0,1,0,1,0,11,18470.14657,21687.77583,12.61213609,10.6005456,15.74446008,1.344554061,22,
8470.14657,21687.77583,12.61213609,10.6005456,15.74446008,1.344554061,22,2,35.20887992,4
3609,10.6005456,15.74446008,1.344554061,22,2,35.20887992,42.40218242,9.260492097,228.0694
2.61213609,10.6005456,15.74446008,1.344554061,22,2,35.20887992,42.40218242,9.260492097,22
73,0,1,0,1,0,11,18470.14657,21687.77583,12.61213609,10.6005456,15.74446008,1.344554061,22,
8470.14657,21687.77583,12.61213609,10.6005456,15.74446008,1.344554061,22,2,35.20887992,4
18470.14657,21687.77583,12.61213609,10.6005456,15.74446008,1.344554061,22,2,35.20887992,
213609,10.6005456,15.74446008,1.344554061,22,2,35.20887992,42.40218242,9.260492097,228.06
8470.14657,21687.77583,12.61213609,10.6005456,15.74446008,1.344554061,22,2,35.20887992,4
1213609,10.6005456,15.74446008,1.344554061,22,2,35.20887992,42.40218242,9.260492097,228.0
470.14657,21687.77583,12.61213609,10.6005456,15.74446008,1.344554061,22,2,35.20887992,42.
470.14657,21687.77583,12.61213609,10.6005456,15.74446008,1.344554061,22,2,35.20887992,42.
2.61213609,10.6005456,15.74446008,1.344554061,22,2,35.20887992,42.40218242,9.260492097,22
470.14657,21687.77583,12.61213609,10.6005456,15.74446008,1.344554061,22,2,35.20887992,42.
12.61213609,10.6005456,15.74446008,1.344554061,22,2,35.20887992,42.40218242,9.260492097,2
.61213609,10.6005456,15.74446008,1.344554061,22,2,35.20887992,42.40218242,9.260492097,22
3609,10.6005456,15.74446008,1.344554061,22,2,35.20887992,42.40218242,9.260492097,228.0694
73,0,1,0,1,0,11,18470.14657,21687.77583,12.61213609,10.6005456,15.74446008,1.344554061,22,
8470.14657,21687.77583,12.61213609,10.6005456,15.74446008,1.344554061,22,2,35.20887992,4
0,73,0,1,0,1,0,11,18470.14657,21687.77583,12.61213609,10.6005456,15.74446008,1.344554061,22
3609,10.6005456,15.74446008,1.344554061,22,2,35.20887992,42.40218242,9.260492097,228.0694

18470.14657,21687.77583,12.61213609,10.6005456,15.74446008,1.344554061,22,2,35.20887992,4
73,0,1,0,1,0,11,18470.14657,21687.77583,12.61213609,10.6005456,15.74446008,1.344554061,22,
8470.14657,21687.77583,12.61213609,10.6005456,15.74446008,1.344554061,22,2,35.20887992,4
470.14657,21687.77583,12.61213609,10.6005456,15.74446008,1.344554061,22,2,35.20887992,42.
73,0,1,0,1,0,11,18470.14657,21687.77583,12.61213609,10.6005456,15.74446008,1.344554061,22,
3609,10.6005456,15.74446008,1.344554061,22,2,35.20887992,42.40218242,9.260492097,228.0694
509,10.6005456,15.74446008,1.344554061,22,2,35.20887992,42.40218242,9.260492097,228.06946
8.48871189,16.45812869,1.737970367,16,3,36.90135866,46.22177972,103.9832187,210.2791708,
676.8879,20130.46913,11.12898118,18.48871189,16.45812869,1.737970367,16,3,36.90135866,46.
76.8879,20130.46913,11.12898118,18.48871189,16.45812869,1.737970367,16,3,36.90135866,46.2
18,18.48871189,16.45812869,1.737970367,16,3,36.90135866,46.22177972,103.9832187,210.27917
18,18.48871189,16.45812869,1.737970367,16,3,36.90135866,46.22177972,103.9832187,210.27917
18.48871189,16.45812869,1.737970367,16,3,36.90135866,46.22177972,103.9832187,210.2791708
98118,18.48871189,16.45812869,1.737970367,16,3,36.90135866,46.22177972,103.9832187,210.27
18676.8879,20130.46913,11.12898118,18.48871189,16.45812869,1.737970367,16,3,36.90135866,4
676.8879,20130.46913,11.12898118,18.48871189,16.45812869,1.737970367,16,3,36.90135866,46.
18,18.48871189,16.45812869,1.737970367,16,3,36.90135866,46.22177972,103.9832187,210.27917
18,18.48871189,16.45812869,1.737970367,16,3,36.90135866,46.22177972,103.9832187,210.27917
676.8879,20130.46913,11.12898118,18.48871189,16.45812869,1.737970367,16,3,36.90135866,46.
76.8879,20130.46913,11.12898118,18.48871189,16.45812869,1.737970367,16,3,36.90135866,46.2
2,0,1,0,1,0,9,18676.8879,20130.46913,11.12898118,18.48871189,16.45812869,1.737970367,16,3,3
76.8879,20130.46913,11.12898118,18.48871189,16.45812869,1.737970367,16,3,36.90135866,46.2
8676.8879,20130.46913,11.12898118,18.48871189,16.45812869,1.737970367,16,3,36.90135866,4
2,0,1,0,1,0,9,18676.8879,20130.46913,11.12898118,18.48871189,16.45812869,1.737970367,16,3,3
92,0,1,0,1,0,9,18676.8879,20130.46913,11.12898118,18.48871189,16.45812869,1.737970367,16,3,
12898118,18.48871189,16.45812869,1.737970367,16,3,36.90135866,46.22177972,103.9832187,21
76.8879,20130.46913,11.12898118,18.48871189,16.45812869,1.737970367,16,3,36.90135866,46.2
3118,18.48871189,16.45812869,1.737970367,16,3,36.90135866,46.22177972,103.9832187,210.279
,11.12898118,18.48871189,16.45812869,1.737970367,16,3,36.90135866,46.22177972,103.983218
3676.8879,20130.46913,11.12898118,18.48871189,16.45812869,1.737970367,16,3,36.90135866,46
18676.8879,20130.46913,11.12898118,18.48871189,16.45812869,1.737970367,16,3,36.90135866,4
8676.8879,20130.46913,11.12898118,18.48871189,16.45812869,1.737970367,16,3,36.90135866,4
98118,18.48871189,16.45812869,1.737970367,16,3,36.90135866,46.22177972,103.9832187,210.27
76.8879,20130.46913,11.12898118,18.48871189,16.45812869,1.737970367,16,3,36.90135866,46.2
18,18.48871189,16.45812869,1.737970367,16,3,36.90135866,46.22177972,103.9832187,210.27917
76.8879,20130.46913,11.12898118,18.48871189,16.45812869,1.737970367,16,3,36.90135866,46.2
0617676,3,2,220,92,0,1,0,1,0,9,18676.8879,20130.46913,11.12898118,18.48871189,16.45812869,1.
76.8879,20130.46913,11.12898118,18.48871189,16.45812869,1.737970367,16,3,36.90135866,46.2
98118,18.48871189,16.45812869,1.737970367,16,3,36.90135866,46.22177972,103.9832187,210.27
76.8879,20130.46913,11.12898118,18.48871189,16.45812869,1.737970367,16,3,36.90135866,46.2
2898118,18.48871189,16.45812869,1.737970367,16,3,36.90135866,46.22177972,103.9832187,210.
92,0,1,0,1,0,9,18676.8879,20130.46913,11.12898118,18.48871189,16.45812869,1.737970367,16,3,
0,92,0,1,0,1,0,9,18676.8879,20130.46913,11.12898118,18.48871189,16.45812869,1.737970367,16,
92,0,1,0,1,0,9,18676.8879,20130.46913,11.12898118,18.48871189,16.45812869,1.737970367,16,3,
18,18.48871189,16.45812869,1.737970367,16,3,36.90135866,46.22177972,103.9832187,210.27917
441,20.77006018,16.45812869,1.737970367,16,3,38.62352824,46.73263541,131.5080275,209.7554
79,1,1,1,1,0,9,18831.06727,21027.23344,11.28995441,20.77006018,16.45812869,1.737970367,16,
441,20.77006018,16.45812869,1.737970367,16,3,38.62352824,46.73263541,131.5080275,209.7554
79,1,1,1,1,0,9,18831.06727,21027.23344,11.28995441,20.77006018,16.45812869,1.737970367,16,
441,20.77006018,16.45812869,1.737970367,16,3,38.62352824,46.73263541,131.5080275,209.7554

441,20.77006018,16.45812869,1.737970367,16,3,38.62352824,46.73263541,131.5080275,209.7554
31.06727,21027.23344,11.28995441,20.77006018,16.45812869,1.737970367,16,3,38.62352824,46.
831.06727,21027.23344,11.28995441,20.77006018,16.45812869,1.737970367,16,3,38.62352824,4
441,20.77006018,16.45812869,1.737970367,16,3,38.62352824,46.73263541,131.5080275,209.7554
831.06727,21027.23344,11.28995441,20.77006018,16.45812869,1.737970367,16,3,38.62352824,4
41,20.77006018,16.45812869,1.737970367,16,3,38.62352824,46.73263541,131.5080275,209.75546
1.28995441,20.77006018,16.45812869,1.737970367,16,3,38.62352824,46.73263541,131.5080275,2
28995441,20.77006018,16.45812869,1.737970367,16,3,38.62352824,46.73263541,131.5080275,20
8831.06727,21027.23344,11.28995441,20.77006018,16.45812869,1.737970367,16,3,38.62352824,
8831.06727,21027.23344,11.28995441,20.77006018,16.45812869,1.737970367,16,3,38.62352824,
995441,20.77006018,16.45812869,1.737970367,16,3,38.62352824,46.73263541,131.5080275,209.7
0,79,1,1,1,1,0,9,18831.06727,21027.23344,11.28995441,20.77006018,16.45812869,1.737970367,16
441,20.77006018,16.45812869,1.737970367,16,3,38.62352824,46.73263541,131.5080275,209.7554
441,20.77006018,16.45812869,1.737970367,16,3,38.62352824,46.73263541,131.5080275,209.7554
79,1,1,1,1,0,9,18831.06727,21027.23344,11.28995441,20.77006018,16.45812869,1.737970367,16,3
995441,20.77006018,16.45812869,1.737970367,16,3,38.62352824,46.73263541,131.5080275,209.7
831.06727,21027.23344,11.28995441,20.77006018,16.45812869,1.737970367,16,3,38.62352824,4
441,20.77006018,16.45812869,1.737970367,16,3,38.62352824,46.73263541,131.5080275,209.7554
0,79,1,1,1,1,0,9,18831.06727,21027.23344,11.28995441,20.77006018,16.45812869,1.737970367,16
441,20.77006018,16.45812869,1.737970367,16,3,38.62352824,46.73263541,131.5080275,209.7554
95441,20.77006018,16.45812869,1.737970367,16,3,38.62352824,46.73263541,131.5080275,209.75
31.06727,21027.23344,11.28995441,20.77006018,16.45812869,1.737970367,16,3,38.62352824,46.
31.06727,21027.23344,11.28995441,20.77006018,16.45812869,1.737970367,16,3,38.62352824,46.
79,1,1,1,1,0,9,18831.06727,21027.23344,11.28995441,20.77006018,16.45812869,1.737970367,16,3
8831.06727,21027.23344,11.28995441,20.77006018,16.45812869,1.737970367,16,3,38.62352824,
.81122792,7.51819084,13.4161258,0.393416306,13,1,35.24491168,75.1819084,96.55020948,223.3
8614.81459,23081.53767,8.81122792,7.51819084,13.4161258,0.393416306,13,1,35.24491168,75.1
81122792,7.51819084,13.4161258,0.393416306,13,1,35.24491168,75.1819084,96.55020948,223.39
792,7.51819084,13.4161258,0.393416306,13,1,35.24491168,75.1819084,96.55020948,223.3905432
65,1,1,0,1,0,11,18614.81459,23081.53767,8.81122792,7.51819084,13.4161258,0.393416306,13,1,3
122792,7.51819084,13.4161258,0.393416306,13,1,35.24491168,75.1819084,96.55020948,223.3905
8614.81459,23081.53767,8.81122792,7.51819084,13.4161258,0.393416306,13,1,35.24491168,75.1
8614.81459,23081.53767,8.81122792,7.51819084,13.4161258,0.393416306,13,1,35.24491168,75.1
8614.81459,23081.53767,8.81122792,7.51819084,13.4161258,0.393416306,13,1,35.24491168,75.1
614.81459,23081.53767,8.81122792,7.51819084,13.4161258,0.393416306,13,1,35.24491168,75.18
8614.81459,23081.53767,8.81122792,7.51819084,13.4161258,0.393416306,13,1,35.24491168,75.1
792,7.51819084,13.4161258,0.393416306,13,1,35.24491168,75.1819084,96.55020948,223.3905432
792,7.51819084,13.4161258,0.393416306,13,1,35.24491168,75.1819084,96.55020948,223.3905432
792,7.51819084,13.4161258,0.393416306,13,1,35.24491168,75.1819084,96.55020948,223.3905432
8614.81459,23081.53767,8.81122792,7.51819084,13.4161258,0.393416306,13,1,35.24491168,75.1
8614.81459,23081.53767,8.81122792,7.51819084,13.4161258,0.393416306,13,1,35.24491168,75.1
792,7.51819084,13.4161258,0.393416306,13,1,35.24491168,75.1819084,96.55020948,223.3905432
8614.81459,23081.53767,8.81122792,7.51819084,13.4161258,0.393416306,13,1,35.24491168,75.1
792,7.51819084,13.4161258,0.393416306,13,1,35.24491168,75.1819084,96.55020948,223.3905432
8614.81459,23081.53767,8.81122792,7.51819084,13.4161258,0.393416306,13,1,35.24491168,75.1
,18614.81459,23081.53767,8.81122792,7.51819084,13.4161258,0.393416306,13,1,35.24491168,75
92,7.51819084,13.4161258,0.393416306,13,1,35.24491168,75.1819084,96.55020948,223.3905432,2
65,1,1,0,1,0,11,18614.81459,23081.53767,8.81122792,7.51819084,13.4161258,0.393416306,13,1,3
8614.81459,23081.53767,8.81122792,7.51819084,13.4161258,0.393416306,13,1,35.24491168,75.1
0,65,1,1,0,1,0,11,18614.81459,23081.53767,8.81122792,7.51819084,13.4161258,0.393416306,13,1,

614.81459,23081.53767,8.81122792,7.51819084,13.4161258,0.393416306,13,1,35.24491168,75.18
792,7.51819084,13.4161258,0.393416306,13,1,35.24491168,75.1819084,96.55020948,223.3905432
1122792,7.51819084,13.4161258,0.393416306,13,1,35.24491168,75.1819084,96.55020948,223.39
792,7.51819084,13.4161258,0.393416306,13,1,35.24491168,75.1819084,96.55020948,223.3905432
65,1,1,0,1,0,11,18614.81459,23081.53767,8.81122792,7.51819084,13.4161258,0.393416306,13,1,3
8614.81459,23081.53767,8.81122792,7.51819084,13.4161258,0.393416306,13,1,35.24491168,75.1
54.83465,22089.8048,13.18021942,10.97299591,13.46159626,1.344554061,24,2,33.74136171,40.2
54.83465,22089.8048,13.18021942,10.97299591,13.46159626,1.344554061,24,2,33.74136171,40.2
2,10.97299591,13.46159626,1.344554061,24,2,33.74136171,40.23431832,66.8294086,235.662323
73,1,1,1,1,0,9,18554.83465,22089.8048,13.18021942,10.97299591,13.46159626,1.344554061,24,2,
8554.83465,22089.8048,13.18021942,10.97299591,13.46159626,1.344554061,24,2,33.74136171,4
554.83465,22089.8048,13.18021942,10.97299591,13.46159626,1.344554061,24,2,33.74136171,40.
2,10.97299591,13.46159626,1.344554061,24,2,33.74136171,40.23431832,66.8294086,235.662323
942,10.97299591,13.46159626,1.344554061,24,2,33.74136171,40.23431832,66.8294086,235.6623
,73,1,1,1,1,0,9,18554.83465,22089.8048,13.18021942,10.97299591,13.46159626,1.344554061,24,
73,1,1,1,1,0,9,18554.83465,22089.8048,13.18021942,10.97299591,13.46159626,1.344554061,24,2,
2,10.97299591,13.46159626,1.344554061,24,2,33.74136171,40.23431832,66.8294086,235.662323
942,10.97299591,13.46159626,1.344554061,24,2,33.74136171,40.23431832,66.8294086,235.6623
8554.83465,22089.8048,13.18021942,10.97299591,13.46159626,1.344554061,24,2,33.74136171,4
54.83465,22089.8048,13.18021942,10.97299591,13.46159626,1.344554061,24,2,33.74136171,40.2
73,1,1,1,1,0,9,18554.83465,22089.8048,13.18021942,10.97299591,13.46159626,1.344554061,24,2,
021942,10.97299591,13.46159626,1.344554061,24,2,33.74136171,40.23431832,66.8294086,235.6
942,10.97299591,13.46159626,1.344554061,24,2,33.74136171,40.23431832,66.8294086,235.6623
617676,3,2,220,73,1,1,1,1,0,9,18554.83465,22089.8048,13.18021942,10.97299591,13.46159626,1.
554.83465,22089.8048,13.18021942,10.97299591,13.46159626,1.344554061,24,2,33.74136171,40.
554.83465,22089.8048,13.18021942,10.97299591,13.46159626,1.344554061,24,2,33.74136171,40.
2,10.97299591,13.46159626,1.344554061,24,2,33.74136171,40.23431832,66.8294086,235.662323
1942,10.97299591,13.46159626,1.344554061,24,2,33.74136171,40.23431832,66.8294086,235.662
554.83465,22089.8048,13.18021942,10.97299591,13.46159626,1.344554061,24,2,33.74136171,40.
554.83465,22089.8048,13.18021942,10.97299591,13.46159626,1.344554061,24,2,33.74136171,40.
554.83465,22089.8048,13.18021942,10.97299591,13.46159626,1.344554061,24,2,33.74136171,40.
942,10.97299591,13.46159626,1.344554061,24,2,33.74136171,40.23431832,66.8294086,235.6623
554.83465,22089.8048,13.18021942,10.97299591,13.46159626,1.344554061,24,2,33.74136171,40.
54.83465,22089.8048,13.18021942,10.97299591,13.46159626,1.344554061,24,2,33.74136171,40.2
554.83465,22089.8048,13.18021942,10.97299591,13.46159626,1.344554061,24,2,33.74136171,40.
,73,1,1,1,1,0,9,18554.83465,22089.8048,13.18021942,10.97299591,13.46159626,1.344554061,24,
0.97299591,13.46159626,1.344554061,24,2,33.74136171,40.23431832,66.8294086,235.6623232,2
13.18021942,10.97299591,13.46159626,1.344554061,24,2,33.74136171,40.23431832,66.8294086,
8554.83465,22089.8048,13.18021942,10.97299591,13.46159626,1.344554061,24,2,33.74136171,4
554.83465,22089.8048,13.18021942,10.97299591,13.46159626,1.344554061,24,2,33.74136171,40.
2,10.97299591,13.46159626,1.344554061,24,2,33.74136171,40.23431832,66.8294086,235.662323
0834392,13.38553506,15.93897415,1.344554061,24,2,34.51329788,43.50298894,1.33319525,229.
8234.55723,22331.15283,13.90834392,13.38553506,15.93897415,1.344554061,24,2,34.51329788,
74,0,0,0,1,0,12,18234.55723,22331.15283,13.90834392,13.38553506,15.93897415,1.344554061,24
392,13.38553506,15.93897415,1.344554061,24,2,34.51329788,43.50298894,1.33319525,229.7452
834392,13.38553506,15.93897415,1.344554061,24,2,34.51329788,43.50298894,1.33319525,229.7
18234.55723,22331.15283,13.90834392,13.38553506,15.93897415,1.344554061,24,2,34.51329788
18234.55723,22331.15283,13.90834392,13.38553506,15.93897415,1.344554061,24,2,34.51329788
834392,13.38553506,15.93897415,1.344554061,24,2,34.51329788,43.50298894,1.33319525,229.7
234.55723,22331.15283,13.90834392,13.38553506,15.93897415,1.344554061,24,2,34.51329788,4

392,13.38553506,15.93897415,1.344554061,24,2,34.51329788,43.50298894,1.33319525,229.74523
8234.55723,22331.15283,13.90834392,13.38553506,15.93897415,1.344554061,24,2,34.51329788,
234.55723,22331.15283,13.90834392,13.38553506,15.93897415,1.344554061,24,2,34.51329788,43
.90834392,13.38553506,15.93897415,1.344554061,24,2,34.51329788,43.50298894,1.33319525,229
74,0,0,0,1,0,12,18234.55723,22331.15283,13.90834392,13.38553506,15.93897415,1.344554061,24
,13.90834392,13.38553506,15.93897415,1.344554061,24,2,34.51329788,43.50298894,1.33319525
,18234.55723,22331.15283,13.90834392,13.38553506,15.93897415,1.344554061,24,2,34.51329788
74,0,0,0,1,0,12,18234.55723,22331.15283,13.90834392,13.38553506,15.93897415,1.344554061,24
[truncated: 469,642 more chars]
